# Supplementary material for: Vulnerability to climate change of United States marine mammal stocks in the western North Atlantic, Gulf of Mexico, and Caribbean
Source: PLoS One. 2023 Sep 20;18(9):e0290643. doi: 10.1371/journal.pone.0290643 (PMC10511136; doi:10.1371/journal.pone.0290643)
Supplement: S2 File — Box and whisker plots of sensitivity attribute mean scores and exposure factor mean scores for each of the five taxonomic groups. (DOCX) [file pone.0290643.s003.docx]

**S5. Taxonomic group attribute and factor scores.**


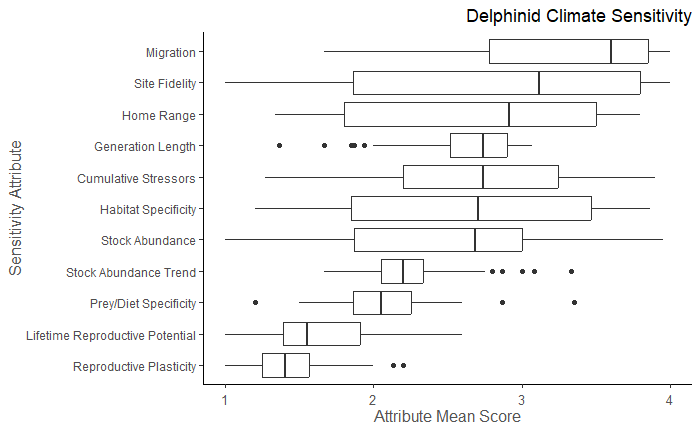


**Fig 1. Sensitivity attribute mean scores for delphinid stocks.**

Sensitivity attribute mean scores for 84 U.S. delphinid stocks in the western North Atlantic, Gulf of Mexico, and Caribbean Sea. The vertical bar represents the median; the box is bounded by the first and third quartiles; whiskers represent 1.5 times the inter-quartile range; points represent all outlying values.


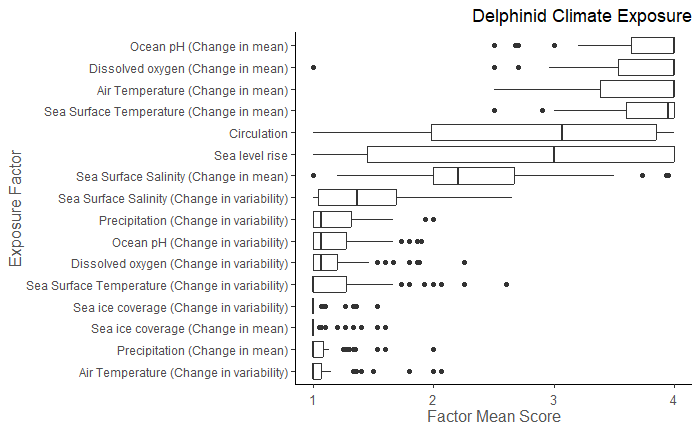


**Fig 2. Exposure factor mean scores for delphinid stocks.**

Exposure factor mean scores for 84 U.S. delphinid stocks in the western North Atlantic, Gulf of Mexico, and Caribbean Sea. The vertical bar represents the median; the box is bounded by the first and third quartiles; whiskers represent 1.5 times the inter-quartile range; points represent all outlying values.


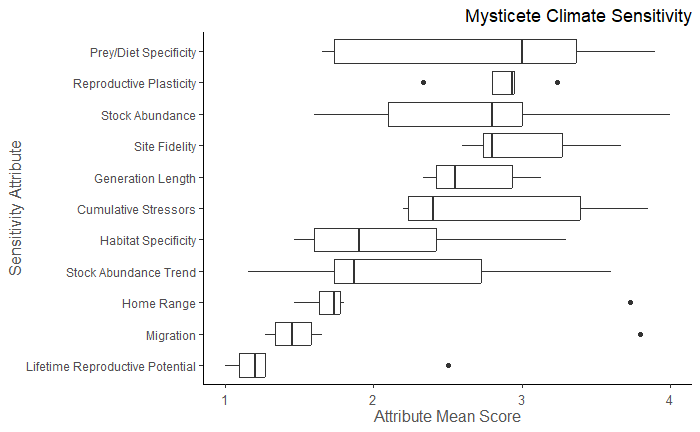


**Fig 3. Sensitivity attribute mean scores for mysticete stocks.**

Sensitivity attribute mean scores for 7 U.S. mysticete stocks in the western North Atlantic, Gulf of Mexico, and Caribbean Sea. The vertical bar represents the median; the box is bounded by the first and third quartiles; whiskers represent 1.5 times the inter-quartile range; points represent all outlying values.


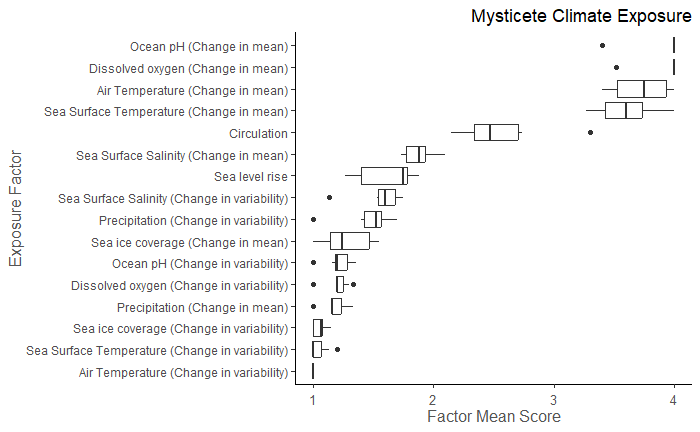


**Fig 4. Exposure factor mean scores for mysticete stocks.**

Exposure factor mean scores for 7 U.S. mysticete stocks in the western North Atlantic, Gulf of Mexico, and Caribbean Sea. The vertical bar represents the median; the box is bounded by the first and third quartiles; whiskers represent 1.5 times the inter-quartile range; points represent all outlying values.


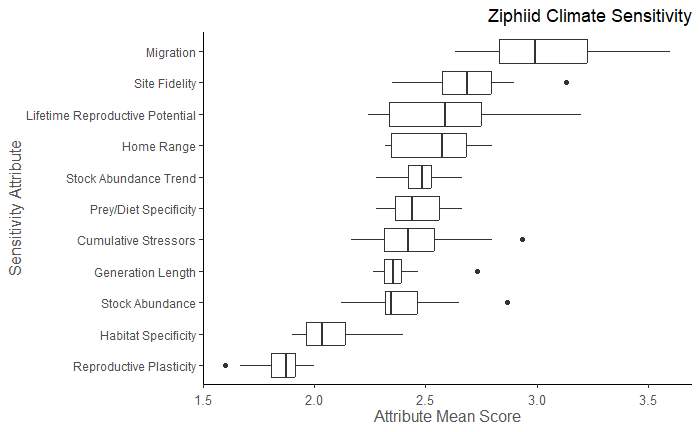


**Fig 5. Sensitivity attribute mean scores for ziphiid stocks.**

Sensitivity attribute mean scores for 8 U.S. ziphiid stocks in the western North Atlantic, Gulf of Mexico, and Caribbean Sea. The vertical bar represents the median; the box is bounded by the first and third quartiles; whiskers represent 1.5 times the inter-quartile range; points represent all outlying values.


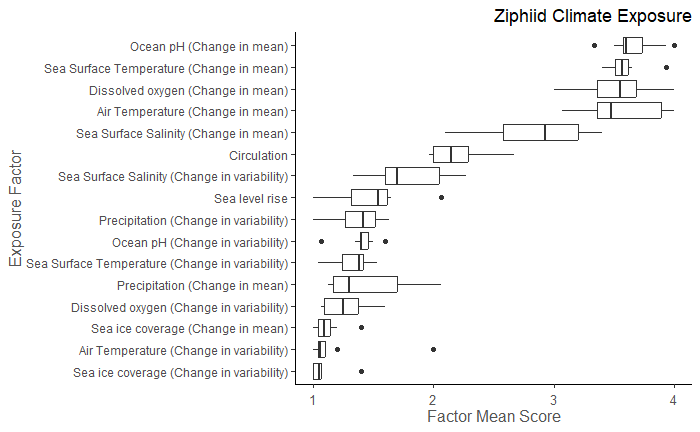


**Fig 6. Exposure factor mean scores for ziphiid stocks.**

Exposure factor mean scores for 8 U.S. ziphiid stocks in the western North Atlantic, Gulf of Mexico, and Caribbean Sea. The vertical bar represents the median; the box is bounded by the first and third quartiles; whiskers represent 1.5 times the inter-quartile range; points represent all outlying values.


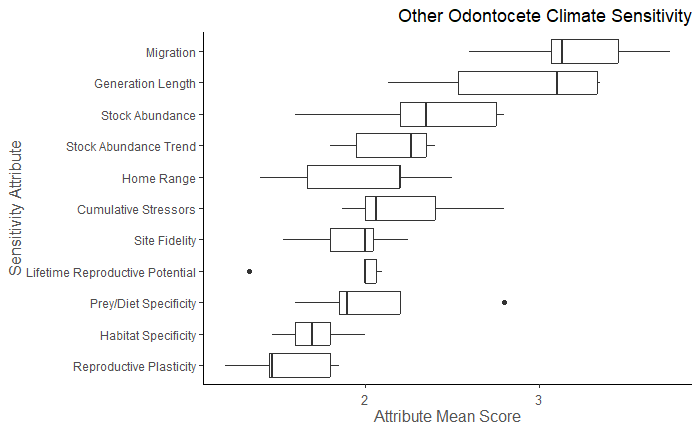


**Fig 7. Sensitivity attribute mean scores for other odontocete stocks.**

Sensitivity attribute mean scores for 5 U.S. “other odontocete” stocks in the western North Atlantic, Gulf of Mexico, and Caribbean Sea. The vertical bar represents the median; the box is bounded by the first and third quartiles; whiskers represent 1.5 times the inter-quartile range; points represent all outlying values.


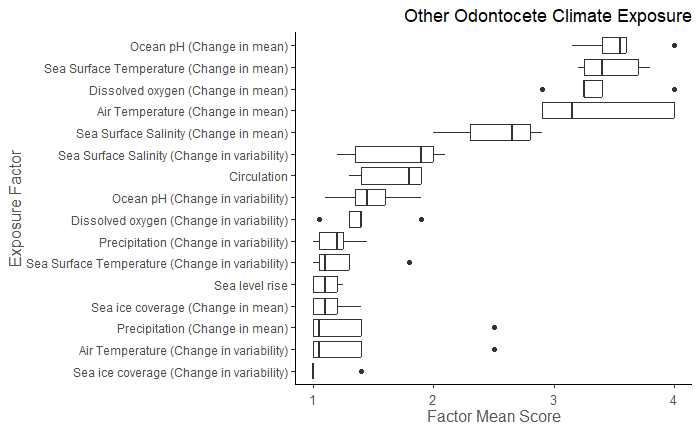


**Fig 8. Exposure factor mean scores for other odontocete stocks.**

Exposure factor mean scores for 5 U.S. “other odontocete” stocks in the western North Atlantic, Gulf of Mexico, and Caribbean Sea. The vertical bar represents the median; the box is bounded by the first and third quartiles; whiskers represent 1.5 times the inter-quartile range; points represent all outlying values.


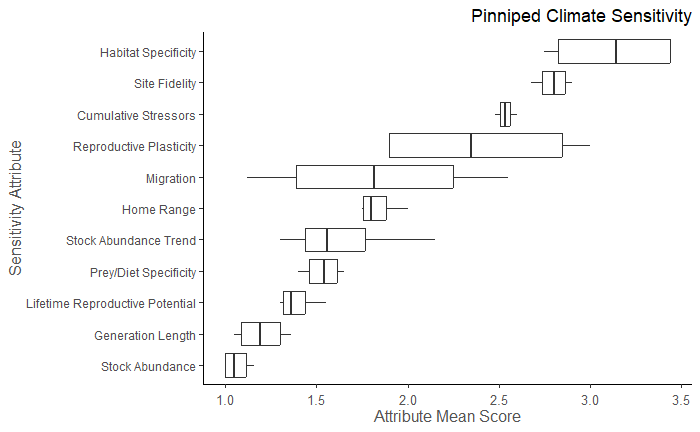


**Fig 9. Sensitivity attribute mean scores for pinniped stocks.**

Sensitivity attribute mean scores for 4 U.S. pinniped stocks in the western North Atlantic, Gulf of Mexico, and Caribbean Sea. The vertical bar represents the median; the box is bounded by the first and third quartiles; whiskers represent 1.5 times the inter-quartile range; points represent all outlying values.


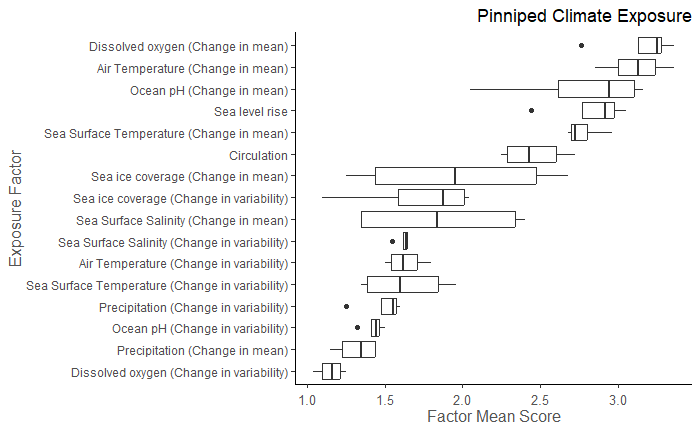


**Fig 10. Exposure factor mean scores for pinniped stocks.**

Exposure factor mean scores for 4 U.S. pinniped stocks in the western North Atlantic, Gulf of Mexico, and Caribbean Sea. The vertical bar represents the median; the box is bounded by the first and third quartiles; whiskers represent 1.5 times the inter-quartile range; points represent all outlying values.
