# Supplementary material for: Vulnerability to climate change of United States marine mammal stocks in the western North Atlantic, Gulf of Mexico, and Caribbean
Source: PLoS One. 2023 Sep 20;18(9):e0290643. doi: 10.1371/journal.pone.0290643 (PMC10511136; doi:10.1371/journal.pone.0290643)
Supplement: S3 File — Leave-one-out analysis for expert scores. (DOCX) [file pone.0290643.s004.docx]

**S6. Expert effect on vulnerability scores.**

Out of 383 expert leave-one-out scenarios, 77% (n=295) resulted in no change in score, 21% (n=80) resulted in a change in score by one category (e.g., move from low to moderate), and 2% (n=8) resulted in a change in score by two categories (e.g., move from low to high; Fig. 1).


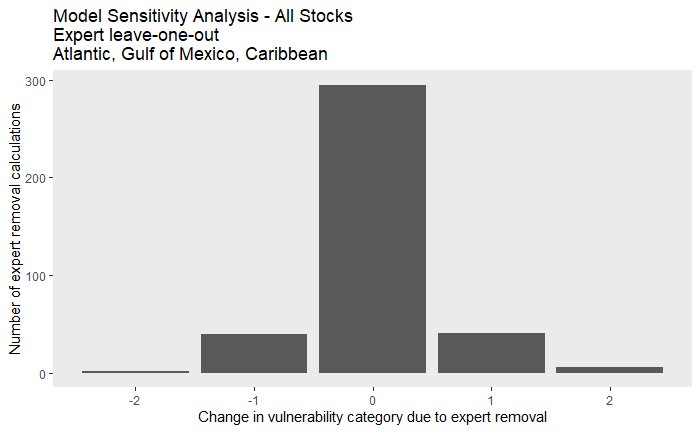


Figure 1. Change in vulnerability category resulting from expert leave-one out analysis.

The effect of an expert’s institution was more challenging to qualify, as we had some experts change affiliations over the course of the assessment and several experts held multiple affiliations. We compared scores from experts that held any NOAA affiliation (including employee, contractor, post-doc, or fellow, but excluding grantees, panelists, and advisory roles) during the course of the assessment with scores from experts that did not hold a NOAA affiliation during the course of the assessment.

Out of 237 leave-one-out scenarios that removed the scores of NOAA-affiliated experts , 76% (n=180) resulted in no change in score, 22% (n=53) resulted in a change in score by one category (e.g., move from low to moderate), and 2% (n=4) resulted in a move change in score by two categories (e.g., move from low to high; Fig. 2).

Out of 146 leave-one-out scenarios that removed the scores of non-NOAA-affiliated experts, 79% (n=115) resulted in no change in score, 18% (n=27) resulted in a change in score by one category (e.g., move from low to moderate), and 3% (n=4) resulted in a move change in score by two categories (e.g., move from low to high; Fig. 2).


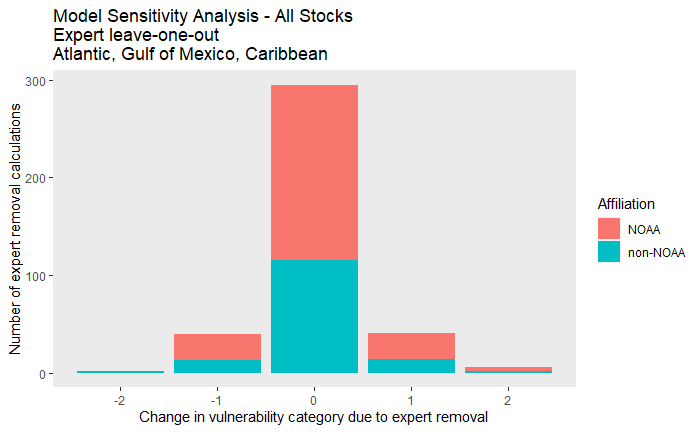


Figure 2. Change in vulnerability category resulting from expert leave-one out analysis separated by NOAA-affiliated scorers and scorers with no NOAA affiliation.

Of the seven stocks that included a shift in vulnerability score by two categories in the an expert leave-on-out analysis, five were among the stocks with the lowest data quality scores:

- Fraser's dolphin (Western North Atlantic); data quality score = 0.26
- Gervais beaked whale (Western North Atlantic); data quality score = 0.26
- Blainville's beaked whale (Western North Atlantic); data quality score = 0.37
- Sowerby's beaked whale (Western North Atlantic); data quality score = 0.37
- Rough-toothed dolphin (Western North Atlantic); data quality score = 0.52

The remaining two stocks had data quality scores of 0.85 (killer whale in the Gulf of Mexico) and 0.81 (rough-toothed dolphin in the Gulf of Mexico).
