# Supplementary material for: Vulnerability to climate change of United States marine mammal stocks in the western North Atlantic, Gulf of Mexico, and Caribbean
Source: PLoS One. 2023 Sep 20;18(9):e0290643. doi: 10.1371/journal.pone.0290643 (PMC10511136; doi:10.1371/journal.pone.0290643)
Supplement: S1 File — Detailed scoring results profile and descriptions of the climate impacts on each stock, including key drivers of vulnerability, exposure, and sensitivity; data quality and gaps; and background information on the stock. (PDF) [file pone.0290643.s002.pdf]

Atlantic spotted dolphin (Gulf of Mexico Stock) ... 5
Atlantic spotted dolphin (Puerto Rico and US Virgin Islands Stock) ... 14
Atlantic spotted dolphin (Western North Atlantic Stock) ... 24
Atlantic white-sided dolphin (Western North Atlantic Stock) ... 33
Blainville's beaked whale (Western North Atlantic Stock) ... 39
Blue whale (Western North Atlantic Stock) ... 45
Clymene dolphin (Northern Gulf of Mexico Stock) ... 51
Common bottlenose dolphin (GoMx BSE/Apalachee Bay Stock) ... 58
Common bottlenose dolphin (GoMx BSE/Barataria Bay Stock) ... 69
Common bottlenose dolphin (GoMx BSE/Calcasieu Lake Stock) ... 81
Common bottlenose dolphin (GoMx BSE/Caloosahatchee River Stock) ... 93
Common bottlenose dolphin (GoMx BSE/Choctawhatchee Bay Stock) ... 103
Common bottlenose dolphin (GoMx BSE/Chokoloskee Bay, Ten Thousand Islands, Gullivan Bay Stock) 115
Common bottlenose dolphin (GoMx BSE/Copano Bay, Aransas Bay, San Antonio Bay, Redfish Bay, Espiritu Santo Bay Stock) ... 125
Common bottlenose dolphin (GoMx BSE/Estero Bay Stock) ... 135
Common bottlenose dolphin (GoMx BSE/Florida Keys - Southwest Marathon Key to Marquesas Keys Stock) ... 145
Common bottlenose dolphin (GoMx BSE/Galveston Bay, East Bay, Trinity Bay Stock) ... 155
Common bottlenose dolphin (GoMx BSE/Laguna Madre Stock) ... 167
Common bottlenose dolphin (GoMx BSE/Matagorda Bay, Tres Palacios Bay, Lavaca Bay Stock) ... 177
Common bottlenose dolphin (GoMx BSE/Mississippi River Delta Stock) ... 187
Common bottlenose dolphin (GoMx BSE/Mississippi Sound, Lake Borgne, Bay Boudreau Stock) ... 199
Common bottlenose dolphin (GoMx BSE/Mobile Bay, Bonsecour Bay Stock) ... 212
Common bottlenose dolphin (GoMx BSE/Nueces Bay, Corpus Christi Bay Stock) ... 224
Common bottlenose dolphin (GoMx BSE/Pensacola Bay, East Bay Stock) ... 234
Common bottlenose dolphin (GoMx BSE/Perdido Bay Stock) ... 246
Common bottlenose dolphin (GoMx BSE/Pine Island Sound, Charlotte Harbor, Gasparilla Sound, Lemon Bay Stock) ... 258
Common bottlenose dolphin (GoMx BSE/Sabine Lake Stock) ... 268
Common bottlenose dolphin (GoMx BSE/Sarasota Bay, Little Sarasota Bay Stock) ... 279
Common bottlenose dolphin (GoMx BSE/St. Andrew Bay Stock) ... 291
Common bottlenose dolphin (GoMx BSE/St. Joseph Bay Stock) ... 304
Common bottlenose dolphin (GoMx BSE/St. Joseph Sound, Clearwater Harbor Stock) ... 316

Common bottlenose dolphin (GoMx BSE/St. Vincent Sound, Apalachicola Bay, St. George Sound Stock) 327
Common bottlenose dolphin (GoMx BSE/Tampa Bay Stock) 340
Common bottlenose dolphin (GoMx BSE/Terrebonne Bay, Timbalier Bay Stock) 351
Common bottlenose dolphin (GoMx BSE/Vermilion Bay, West Cote Blanche Bay, Atchafalaya Bay Stock) 363
Common bottlenose dolphin (GoMx BSE/Waccasassa Bay, Withlacoochee Bay, Crystal Bay Stock) 374
Common bottlenose dolphin (GoMx BSE/West Bay Stock) 384
Common bottlenose dolphin (GoMx BSE/Whitewater Bay Stock) 395
Common bottlenose dolphin (Gulf of Mexico, Continental Shelf Stock) 405
Common bottlenose dolphin (Gulf of Mexico, Eastern Coastal Stock) 415
Common bottlenose dolphin (Gulf of Mexico, Northern Coastal Stock) 425
Common bottlenose dolphin (Gulf of Mexico, Oceanic Stock) 435
Common bottlenose dolphin (Gulf of Mexico, Western Coastal Stock) 445
Common bottlenose dolphin (Biscayne Bay Stock) 457
Common bottlenose dolphin (Central Georgia Estuarine System Stock) 467
Common bottlenose dolphin (Charleston Estuarine System Stock) 477
Common bottlenose dolphin (Florida Bay Stock) 489
Common bottlenose dolphin (Indian River Lagoon Estuarine System Stock) 500
Common bottlenose dolphin (Jacksonville Estuarine System Stock) 513
Common bottlenose dolphin (Northern Georgia/Southern South Carolina Estuarine System Stock) 524
Common bottlenose dolphin (Northern North Carolina Estuarine System Stock) 536
Common bottlenose dolphin (Northern South Carolina Estuarine System Stock) 548
Common bottlenose dolphin (Puerto Rico and US Virgin Islands Stock) 558
Common bottlenose dolphin (Southern Georgia Estuarine System Stock) 571
Common bottlenose dolphin (Southern North Carolina Estuarine System Stock) 582
Common bottlenose dolphin (Western North Atlantic, Northern and Southern Migratory Coastal Stocks) 593
Common bottlenose dolphin (Western North Atlantic: South Carolina-Georgia, Northern Florida, and Central Florida Coastal Stocks) 603
Common bottlenose dolphin (Western North Atlantic, Offshore Stock) 613
Common dolphin, short-beaked (Western North Atlantic Stock) 622
Cuvier’s beaked whale (Gulf of Mexico Stock) 630
Cuvier’s beaked whale (Puerto Rico and US Virgin Islands Stock) 641

Cuvier’s beaked whale (Western North Atlantic Stock) 651
False killer whale (Northern Gulf of Mexico Stock) 660
False killer whale (Western North Atlantic Stock) 666
Fin whale (Western North Atlantic Stock) 671
Fraser’s dolphin (Gulf of Mexico Stock) 678
Fraser’s dolphin (Western North Atlantic Stock) 685
Gervais beaked whale (Western North Atlantic Stock) 691
Gray seal (Western North Atlantic Stock) 697
Harbor porpoise (Gulf of Maine/Bay of Fundy Stock) 704
Harbor seal (Western North Atlantic Stock) 712
Harp seal (Western North Atlantic Stock) 720
Hooded seal (Western North Atlantic Stock) 727
Humpback whale (Gulf of Maine Stock) 733
Killer whale (Gulf of Mexico Stock) 742
Killer whale (Western North Atlantic) 749
Kogia sp. (Northern Gulf of Mexico Stock Group) 755
Kogia sp. (Western North Atlantic Stock Group) 763
Melon-headed whale (Northern Gulf of Mexico Stock) 770
Melon-headed whale (Western North Atlantic Stock) 778
Mesoplodont group (Northern Gulf of Mexico Stock Group) 784
Minke whale (Canadian East Coast Stock) .... 7[illegible]2
North Atlantic right whale (Western Atlantic Stock) 799
Northern bottlenose whale (Western North Atlantic Stock) 812
Pantropical spotted dolphin (Gulf of Mexico Stock) 817
Pilot whale, long-finned (Western North Atlantic Stock) 823
Pilot whale, short-finned (Northern Gulf of Mexico Stock) 830
Pilot whale, short-finned (Puerto Rico and US Virgin Islands Stock) 837
Pilot whale, short-finned (Western North Atlantic Stock) 846
Pygmy killer whale (Northern Gulf of Mexico Stock) 853
Pygmy killer whale (Western North Atlantic Stock) 859
Rice's whale (Gulf of Mexico Stock) 864
Risso's dolphin (Northern Gulf of Mexico Stock) 870
Risso's dolphin (Western North Atlantic Stock) 878

Rough-toothed dolphin (Gulf of Mexico Stock) 886
Rough-toothed dolphin (Western North Atlantic Stock) 893
Sei whale (Nova Scotia Stock) 899
Sowerby's beaked whale (Western North Atlantic Stock) 905
Sperm whale (Northern Gulf of Mexico Stock) 912
Sperm whale (North Atlantic Stock) 920
Sperm Whale (Puerto Rico and US Virgin Islands Stock) 929
Spinner dolphin (Northern Gulf of Mexico Stock) 937
Spinner dolphin (Puerto Rico and US Virgin Islands Stock) 945
Stenella sp. (Western North Atlantic Stock Group) 952
Striped dolphin (Northern Gulf of Mexico Stock) 960
Striped dolphin (Western North Atlantic Stock) 968
True's beaked whale (Western North Atlantic Stock) 973
White-beaked dolphin (Western North Atlantic Stock) 979

Atlantic spotted dolphin − *Stenella frontalis*

Gulf of Mexico Stock

Overall Vulnerability Rank = High

Biological Sensitivity = Moderate

Climate Exposure = Very High

Data Quality = 85% of scores ≥ 2

	Stenella frontalis	Expert Scores	Data Quality	Expert Scores Plots (Portion by Category)
Sensitivity attributes	Prey/Diet Specificity	1.7	2.0	
	Habitat Specificity	2.7	2.0	
	Site Fidelity	1.9	1.3	
	Lifetime Reproductive Potential	2.5	2.0	
	Generation Length	2.3	2.0	
	Reproductive Plasticity	1.5	1.0	
	Migration	3.6	2.3	
	Home Range	2.1	1.7	
	Stock Abundance	1.3	2.3	
	Stock Abundance Trend	2.3	0.7	
	Cumulative Stressors	2.3	2.0	
	Sensitivity Score	Moderate		
Exposure variables	Sea Surface Temperature (Change in mean)	3.9	3.0	
	Sea Surface Temperature (Change in variability)	1.1	3.0	
	Air Temperature (Change in mean)	3.9	3.0	
	Air Temperature (Change in variability)	1.1	3.0	
	Precipitation (Change in mean)	1.1	3.0	
	Precipitation (Change in variability)	1.1	2.7	
	Sea Surface Salinity (Change in mean)	2.0	2.7	
	Sea Surface Salinity (Change in variability)	1.3	2.7	
	Ocean pH (Change in mean)	3.9	3.0	
	Ocean pH (Change in variability)	1.1	3.0	
	Sea ice coverage (Change in mean)	1.0	3.0	
	Sea ice coverage (Change in variability)	1.0	3.0	
	Dissolved oxygen (Change in mean)	3.9	3.0	
	Dissolved oxygen (Change in variability)	1.1	3.0	
	Circulation	2.7	2.3	
	Sea level rise	2.6	3.0	
	Exposure Score	Very High		
Overall Vulnerability Rank		High		

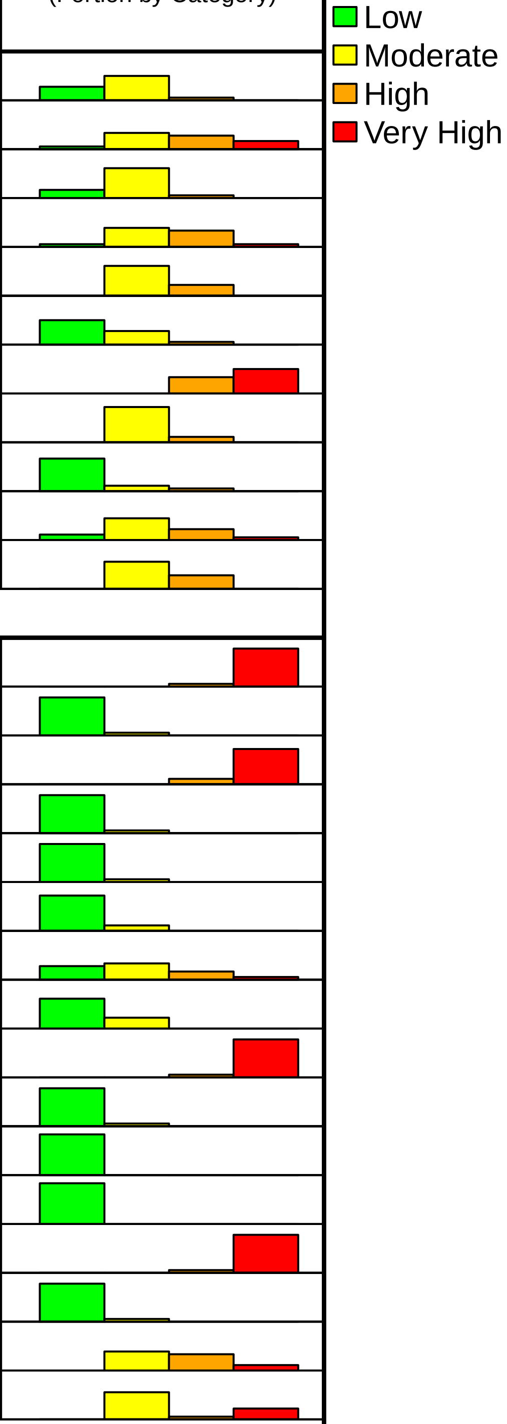

Atlantic spotted dolphin (Gulf of Mexico Stock)

Stenella frontalis

CVA Results Summary

Overall Climate Vulnerability Rank: High (80% certainty from bootstrap analysis).

Climate Exposure: Very High. Four exposure factors scored greater than 3.5: Dissolved oxygen (Standard anomaly) (3.93), Ocean pH (Standard anomaly) (3.93), Sea Surface Temperature (Standard anomaly) (3.93), and Air Temperature (Standard anomaly) (3.87).

Biological Sensitivity: Moderate. Two sensitivity attributes scored greater than 2.5: Migration (3.60) and Habitat Specificity (2.67)

Distributional Response: High

Abundance Response: Low

Phenology Response: Moderate

Data Quality: 85% of the data quality scores were 2 or greater. 64% of sensitivity attribute data quality scores were 2 or higher.

Background Information

Stock Description

The Atlantic spotted dolphin Northern Gulf of Mexico Stock includes individuals found in waters of the U.S. Exclusive Economic Zone in the Gulf of Mexico (Hayes et al. 2022).

Prey/Diet Specificity

Atlantic spotted dolphins in the Gulf of Mexico have been observed feeding on clupeid fishes (order Clupeiformes; Fertl and Würsig 1995). They have also been observed feeding in the vicinity of shrimp trawlers (Fertl and Leatherwood 1997).

Prey and diet information for Atlantic spotted dolphins in the western North Atlantic was found only in the Bahamas, where Atlantic spotted dolphins have been well-studied since the 1980’s. In the Bahamas, lizardfish (order Aulopiformes, family Synodontidae) constituted >30% of female Atlantic spotted dolphin diet during all reproductive states. Other major components of the diet included fish in the orders Beloniformes (halfbeaks [family Hemiramphidae], flyingfish [family Exocoetidae], needlefish [family Belonidae]), Anguilliformes (small eels [family Congridae]), Pleuronectiformes (flounders [family Bothidae]), Perciformes (jacks [family Carangidae], mackerel [family Scombridae], and tilefish [family Malacanthidae]), Labriformes (razorfish [family Labridae]), and Tetradontiformes (filefish [family Monacanthidae]) (Malinowski and Herzing 2015). Atlantic spotted dolphins have been observed chasing and catching flying fish (order Beloniformes) in the Bahamas (MacLeod et al. 2004).

Analysis of stomach contents of two Atlantic spotted dolphins in the Canary Islands showed the presence of cephalopods of the order Oegopsida (genera *Hisitiotheuthis* and *Enoploteuthis*) and crustaceans of the infraclass Cirripedia (Fernandez et al. 2009).

In Brazil, four studies combined for stomach content analysis of 26 individuals (Di Beneditto et al. 2001; Melo et al. 2010; Lopes et al. 2012). These studies listed a total of six cephalopod species and 18 fish species as prey items of Atlantic spotted dolphin. Melo et al. (2010) reported *Porichthys porosissimus* (order Batrachoidiformes) as the main fish species found in 10 stomachs of stranded dolphins. Di Beneditto et al. (2001) reported *Orthopristis ruber* (order Perciformes) as the main fish species found in six incidentally captured dolphins. Both studies reported the squid *Doryteuthis plei* (order Myopsida) was the most important cephalopod prey. Based on stomach content analysis of nine *S. frontalis* incidentally caught in fishing operations in southeastern Brazil, dolphins assessed preyed on at least eight different fish species of the order Perciformes (families Trichiuridae, Carangidae, Sparidae, Sciaenidae, and Scombridae), order Gadiformes (family Merluccidae), Order Clupeiformes (family Engraulidae), Order Anguilliformes (family Congridae), order Myopsida (family Loliginidae), order Sepiida (family Sepiolidae), order Octopoda (family Tremoctopodidae), and order Oegopsida (family Thysanoteuthidae), and order Decapoda (family Penaeidae; Lopes et al. 2012). Lopes et al. (2012) found *Trichiurus lepturus* was the most important fish species represented and *Doryteuthis plei* was the most represented cephalopod species.

Habitat Specificity

The larger coastal ecotype of Atlantic spotted dolphin inhabits the continental shelf and is usually found inside or near the 200m isobath, usually at least 8 to 20 km offshore (Perrin et al. 1987, 1994; Davis et al. 1998; Rice 1998; Herzing and Perrin 2018). The smaller island and offshore ecotype is known to occur in the Atlantic Ocean but not in the Gulf of Mexico (Fulling et al. 2003; Mullin and Fulling 2003; Mullin and Fulling 2004).

Atlantic spotted dolphins are often sighted beyond the shelf break in the Caribbean, Gulf of Mexico, and off the Atlantic U.S. coast (Mills and Rademacher 1996; Roden and Mullin 2000; Fulling et al. 2003; Mullin and Fulling 2003; Mullin et al. 2004).

In the Gulf of Mexico, Atlantic spotted dolphins occur primarily in continental shelf waters 10-200m deep and slope waters <500m deep, though a difference is seen between the eastern and western Gulf of Mexico (Fulling et al. 2003; Mullin and Fulling 2004; Maze-Foley and Mullin 2006). Griffin and Griffin (2003) noted a primary depth range of 20-50m in the eastern Gulf of Mexico. Mullin et al. (2004) found that Atlantic spotted dolphins were sighted in waters with a bottom depth typically <300 m. A single satellite-tagged Atlantic spotted dolphin released in the Gulf of Mexico was found to prefer shallow-water habitat (<30 m in depth; Davis et al. 1996).

Off Brazil, Atlantic spotted dolphins are seen in waters less than 1000m in depth (Moreno et al. 2005).

Site Fidelity

Information regarding Atlantic spotted dolphin site fidelity was not found in the literature.

Lifetime Reproductive Potential

Herzing (1997) reported Atlantic spotted dolphin mean calving interval of 2.96 years (SD = 1.224) with a range of 1-5 years in Lower Bahama Bank.

In the Bahamas, Herzing (1997) estimated age at sexual maturation for females at 8 to 15 years of age. Taylor et al. (2007) reported Atlantic spotted dolphin age at first reproduction of 12 years and age at last reproduction of 31 years based on values reported by Herzing (1997) and Perrin et al. (1994).

Generation Length

Taylor et al. (2007) reported Atlantic spotted dolphin generation length of 18.6 years at $r = 0.01$ and 18.3 years at $r = 0.0$ based on data from the Bahamas (Herzing 1997).

Reproductive Plasticity

Herzing (1997) reported peak calving periods in early spring and late fall for Atlantic spotted dolphin on Lower Bahama Bank.

Information regarding specific breeding locations and habitat was not found in the literature.

Malinowski and Herzing (2015) found that pregnant and lactating females had lower nutritional intake than non-reproductively active females in Lower Bahama Bank.

Migration

In the Gulf of Mexico, Griffin and Griffin (2004) reported higher densities of Atlantic spotted dolphin on the west Florida continental shelf during November through May compared to June through October. Griffin et al. (2005) suggested that Atlantic spotted dolphins more often feed over the continental shelf in winter than summer. High numbers of resighted individuals in Lower Bahama Bank in successive years suggest a stable resident population of Atlantic spotted dolphins (Herzing 1997).

Home Range

Information about Atlantic spotted dolphin home range in the Gulf of Mexico was not found in the literature.

Elsewhere, groups have shifted home ranges, such as in 2013 when half of the Little Bahama Bank Atlantic spotted dolphin community relocated 161 km south to Great Bahama Bank, which is home to a separate community of resident spotted dolphins (Herzing et al. 2017).

Stock Abundance

The abundance is estimated at 21,506 individuals (CV=0.26) based on the combined estimate of abundance from 2017 continental shelf aerial surveys and 2017-2018 vessel surveys in oceanic waters (Garrison et al. 2020; Garrison et al. 2021; Hayes et al. 2022). Previously, the stock was estimated at 37,611 individuals (CV=0.28) based on the combined estimate of abundance for both the outer continental shelf (fall surveys, 2000–2001) and oceanic waters (spring and summer surveys, 2003–2004; Waring et al. 2016).

Stock Abundance Trend

Three point estimates of Atlantic spotted dolphin abundance have been made based on data from surveys in 2009 (1,161; CV=1.021), 2017 (3,267; CV=0.52), and 2018 (8,178; CV=0.55). Pairwise comparisons of the log-transformed means were conducted between years, and found significant differences between the 2009 and 2018 estimates (see Garrison et al. 2020 and Hayes et al. 2022).

Cumulative Stressors

Atlantic spotted dolphins in the Gulf of Mexico interact with pelagic longline and shrimp trawl fisheries (Hayes et al. 2022). During the period 2015–2019, an estimated 36 Atlantic spotted dolphins (CV=0.47) were killed by interactions with the shrimp otter trawl fishery in the Gulf of Mexico (when all unidentified marine mammal interactions with the fishery were assumed to be Atlantic spotted dolphins; Soldevilla et al. 2021). Atlantic spotted dolphins are known to strand within the Gulf of Mexico (Hayes et al. 2022; also see NOAA National Marine Mammal Health and Stranding Response Database).

The Atlantic spotted dolphin stock was likely exposed to oil and dispersants resulting from the Deepwater Horizon oil spill, which may result in long term chronic health effects (NOAA 2011; Helm et al. 2015; DWH NRDA 2016; Aichinger Dias et al. 2017; Takeshita et al. 2017). An Unusual Mortality Event (UME), involving primarily bottlenose dolphins, was declared for cetaceans in the northern Gulf of Mexico from 2010 to 2014, with the Deepwater Horizon oil spill as the primary underlying cause (Litz et al. 2014; Schwacke et al. 2014; Venn-Watson et al. 2015). Atlantc spotted dolphins have also been included in UMEs in 1999–2000 and 2005–2006 due to brevetoxin (Twiner et al. 2012; Litz et al. 2014).

Distribution and Sightings

Density model results for Atlantic spotted dolphin in the Gulf of Mexico are presented by Roberts et al. (2015, 2016) and Mannocci et al. (2017).

Further Reading

Braulik, G. and T.A. Jefferson. 2018. *Stenella frontalis*. The IUCN Red List of Threatened Species 2018: e.T20732A50375312. https://dx.doi.org/10.2305/IUCN.UK.2018-2.RLTS.T20732A50375312.en

Department of the Navy. 2007. Marine Resources Assessment for the Gulf of Mexico. Department of the Navy and U.S. Fleet Forces Command, Hampton, Virginia. Contract #N62470-02-D-9997, CTO 0030.

Hayes, S.A., E. Josephson, K. Maze-Foley, P.E. Rosel, and J. Wallace. 2022. U.S. Atlantic and Gulf of Mexico marine mammal stock assessments 2021. National Oceanic and Atmospheric

Administration, National Marine Fisheries Service, Northeast Fisheries Science Center: Woods Hole, MA. NOAA Technical Memorandum NMFS-NE-288. 380 p. doi: 10.25923/6tt7-kc16

Herzing, D.L. and W.F. Perrin. 2018. Atlantic Spotted Dolphin: Stenella frontalis. In: Würsig, B., J.G.M. Thewissen, and K.M. Kovacs (Eds.), Encyclopedia of Marine Mammals (Third Edition). Academic Press. pp. 40-42. doi: 10.1016/B978-0-12-804327-1.00050-9

Literature Cited

Aichinger Dias, L., J. Litz, L. Garrison, A. Martinez, K. Barry, and T. Speakman. 2017. Exposure of cetaceans to petroleum products following the Deepwater Horizon oil spill in the Gulf of Mexico. Endangered Species Research 33:119–125. doi: 10.3354/esr00770

Davis, R.W., G.S. Fargion, N. May, T.D. Leming, M. Baumgartner, W.E. Evans, L.J. Hansen, and K. Mullin. 1998. Physical habitat of cetaceans along the continental slope in the northcentral and western Gulf of Mexico. Marine Mammal Science 14(3):490–507. doi: 10.1111/j.1748-7692.1998.tb00738.x

Davis, R.W., G.A.J. Worthy, B. Würsig, S.K. Lynn, and F.I. Townsend. 1996. Diving behavior and at-sea movements of an Atlantic spotted dolphin in the Gulf of Mexico. Marine Mammal Science 12(4):569–581. doi: 10.1111/j.1748-7692.1996.tb00069.x

Deepwater Horizon Natural Resource Damage Assessment Trustees (DWH NRDA). 2016. Deepwater Horizon oil spill: Final Programmatic Damage Assessment and Restoration Plan and Final Programmatic Environmental Impact Statement. Retrieved from: http://www.gulfspillrestoration.noaa.gov/restoration-planning/gulf-plan.1659.

Di Beneditto, A.P.M., R.M.A. Ramos, S. Siciliano, R.A. dos Santos, G. Bastos, and E. Fagundes-Neto. 2001. Stomach contents of delphinids from Rio de Janeiro, southeastern Brazil. Aquatic Mammals 27(1):24–28.

Fernández, R., M.B. Santos, M. Carrillo, M. Tejedor, and G.J. Pierce. 2009. Stomach contents of cetaceans stranded in the Canary Islands 1996–2006. Journal of the Marine Biological Association of the United Kingdom 89(5):873–883. doi: 10.1017/s0025315409000290

Fertl, D. and S. Leatherwood. 1997. Cetacean interactions with trawls: A preliminary review. Journal of Northwest Atlantic Fishery Science 22:219–248. doi: 10.2960/J.v22.a17

Fertl, D. and B. Würsig. 1995. Coordinated feeding by Atlantic spotted dolphins (*Stenella frontalis*) in the Gulf of Mexico. Aquatic Mammals 21(1):3–5.

Fulling, G.L., K.D. Mullin, and C.W. Hubard. 2003. Abundance and distribution of cetaceans in outer continental shelf waters of the U.S. Gulf of Mexico. Fishery Bulletin 101(4):923–932.

Garrison, L.P., J. Ortega-Ortiz, and G. Rappucci. 2020. Abundance of marine mammals in waters of the U.S. Gulf of Mexico during the summers of 2017 and 2018. National Oceanic and Atmospheric Administration, National Marine Fisheries Service, Southeast Fisheries Science Center: Miami, FL. Southeast Fisheries Science Center Reference Document PRBD-2020-07. 56 p. doi: 10.25923/3px6-9v48

Garrison, L.P., J. Ortega-Ortiz, and G. Rappucci. 2021. Abundance of coastal and continental shelf stocks of common bottlenose and Atlantic spotted dolphins in the northern Gulf of Mexico: 2017–2018. National Oceanic and Atmospheric Administration, National Marine Fisheries Service, Southeast Fisheries Science Center, Protected Resources and Biodiversity Division: Miami, FL. PRD-2021-01. 25 p. doi: 10.25923/vk95-t881

Griffin, B.R. and N.J. Griffin. 2004. Temporal variation in Atlantic spotted dolphin (*Stenella frontalis*) and bottlenose dolphin (*Tursiops truncatus*) densities on the West Florida Continental Shelf. Aquatic Mammals 30(3):380–390. doi: 10.1578/am.30.3.2004.380

Griffin, R.B. and N.J. Griffin. 2003. Distribution, habitat partitioning, and abundance of Atlantic spotted dolphins, bottlenose dolphins, and loggerhead sea turtles on the eastern Gulf of Mexico continental shelf. Gulf of Mexico Science 21(1):23–34. doi: 10.18785/goms.2101.03

Griffin, R.B., C. Hu, and N.J. Griffin. 2005. Movement patterns of Atlantic spotted dolphins (*Stenella frontalis*) and bottlenose dolphins (*Tursiops truncatus*) in relation to oceanographic fronts. Sixteenth Biennial Conference on the Biology of Marine Mammals, San Diego, CA, 12-16 December. p. 112.

Hayes, S.A., E. Josephson, K. Maze-Foley, P.E. Rosel, and J. Wallace. 2022. U.S. Atlantic and Gulf of Mexico marine mammal stock assessments 2021. National Oceanic and Atmospheric Administration, National Marine Fisheries Service, Northeast Fisheries Science Center: Woods Hole, MA. NOAA Technical Memorandum NMFS-NE-288. 380 p. doi: 10.25923/6tt7-kc16

Helm, R.C., D.P. Costa, T.D. DeBruyn, T.J. O'Shea, R.S. Wells, and T.M. Williams. 2015. Overview of effects of oil spills on marine mammals. In: Fingas, M. (Ed.), Handbook of oil spill science and technology, First edition. John Wiley & Sons, Inc., Hoboken, NJ. pp. 455–475.

Herzing, D.L. 1997. The life history of free-ranging atlantic spotted dolphins (*Stenella frontalis*): Age classes, color phases, and female reproduction. Marine Mammal Science 13(4):576–595. doi: 10.1111/j.1748-7692.1997.tb00085.x

Herzing, D.L., B.N. Augliere, C.R. Elliser, M.L. Green, and A.A. Pack. 2017. Exodus! Large-scale displacement and social adjustments of resident Atlantic spotted dolphins (*Stenella frontalis*) in the Bahamas. PLoS ONE 12(8):e0180304. doi: 10.1371/journal.pone.0180304

Herzing, D.L. and W.F. Perrin. 2018. Atlantic spotted dolphin: *Stenella frontalis*. In: Würsig, B., J.G.M. Thewissen, and K.M. Kovacs (Eds.), Encyclopedia of Marine Mammals (Third Edition). Academic Press. pp. 40–42. doi: 10.1016/B978-0-12-804327-1.00050-9

Litz, J.A., M.A. Baran, S.R. Bowen-Stevens, R.H. Carmichael, K.M. Colegrove, L.P. Garrison, S.E. Fire, E.M. Fougeres, R. Hardy, S. Holmes, W. Jones, B.E. Mase-Guthrie, D.K. Odell, P.E. Rosel, J.T. Saliki, D.K. Shannon, S.F. Shippee, S.M. Smith, E.M. Stratton, M.C. Tumlin, H.R. Whitehead, G.A. Worthy, and T.K. Rowles. 2014. Review of historical unusual mortality events (UMEs) in the Gulf of Mexico (1990–2009): providing context for the multi-year northern Gulf of Mexico cetacean UME declared in 2010. Diseases of Aquatic Organisms 112(2):161–175. doi: 10.3354/dao02807

Lopes, X.M., M.C.d.O. Santos, E. da Silva, M. Bassoi, and R.A. dos Santos. 2012. Feeding habits of the Atlantic spotted dolphin, *Stenella frontalis*, in southeastern Brazil. Brazilian Journal of Oceanography 60(2):189–198. doi: 10.1590/S1679-87592012000200009

MacLeod, C.D., N. Hauser, and H. Peckham. 2004. Diversity, relative density and structure of the cetacean community in summer months east of Great Abaco, Bahamas. Journal of the Marine Biological Association of the United Kingdom 84(2):469–474. doi: 10.1017/S0025315404009476h

Malinowski, C.R. and D.L. Herzing. 2015. Prey use and nutritional differences between reproductive states and age classes in Atlantic spotted dolphins (*Stenella frontalis*) in the Bahamas. Marine Mammal Science 31(4):1471–1493. doi: 10.1111/mms.12238

Mannocci, L., J.J. Roberts, D.L. Miller, and P.N. Halpin. 2017. Extrapolating cetacean densities to quantitatively assess human impacts on populations in the high seas. Conservation Biology 31(3):601–614. doi: 10.1111/cobi.12856

Maze-Foley, K. and K.D. Mullin. 2006. Cetaceans of the oceanic northern Gulf of Mexico: Distributions, group sizes and interspecific associations. Journal of Cetacean Research and Management 8(2):203–213.

Melo, C.L.C., R.A. Santos, M. Bassoi, A.C. Araújo, J. Lailson-Brito, P.R. Dorneles, and A.F. Azevedo. 2010. Feeding habits of delphinids (Mammalia: Cetacea) from Rio de Janeiro State, Brazil. Journal of

the Marine Biological Association of the United Kingdom 90(8):1509–1515. doi: 10.1017/s0025315409991639
Mills, L.R. and K.R. Rademacher. 1996. Atlantic spotted dolphins (*Stenella frontalis*) in the Gulf of Mexico. Gulf of Mexico Science 14(2):114–120. doi: 10.18785/goms.1402.08
Moreno, I.B., A.N. Zerbini, D. Danilewicz, S. de Oliveira Sa, P.C. Simões-Lopes, J. Lailson-Brito, and A.F. Azevedo. 2005. Distribution and habitat characteristics of dolphins of the genus *Stenella* (Cetacea: Delphinidae) in the Southwest Atlantic Ocean. Marine Ecology Progress Series 300:229–240. doi: 10.3354/meps300229
Mullin, K.D. and G.L. Fulling. 2003. Abundance of cetaceans in the southern U.S. North Atlantic Ocean during summer 1998. Fishery Bulletin 101:603–613.
Mullin, K.D. and G.L. Fulling. 2004. Abundance of cetaceans in the oceanic northern Gulf of Mexico, 1996–2001. Marine Mammal Science 20(4):787–807. doi: 10.1111/j.1748-7692.2004.tb01193.x
Mullin, K.D., W. Hoggard, and L.J. Hansen. 2004. Abundance and seasonal occurrence of cetaceans in outer continental shelf and slope waters of the North-central and northwestern Gulf of Mexico. Gulf of Mexico Science 22(1):62–73. doi: 10.18785/goms.2201.06
National Oceanic and Atmospheric Administration (NOAA). 2011. Public scoping for preparation of a programmatic environmental impact statement for the Deepwater Horizon BP Oil Spill. 9 p.
Perrin, W.F., D.K. Caldwell, and M.C. Caldwell. 1994. Atlantic spotted dolphin *Stenella frontalis* (G. Cuvier, 1829). In: Ridgway, S.H. and R. Harrison (Eds.), Handbook of marine mammals. Volume 5: The first book of dolphins. Academic Press, London, UK. pp. 173–190.
Perrin, W.F., E.D. Mitchell, J.G. Mead, D.K. Caldwell, M.C. Caldwell, P.J.H. Bree, and W.H. Dawbin. 1987. Revision of the spotted dolphins, *Stenella* spp. Marine Mammal Science 3(2):99–170. doi: 10.1111/j.1748-7692.1987.tb00158.x
Rice, D.W. 1998. Marine mammals of the world, systematics and distribution. Special Publication No. 4. The Society for Marine Mammalogy, Lawrence, KS. 231 p.
Roberts, J.J., B.D. Best, L. Mannocci, E. Fujioka, P.N. Halpin, D.L. Palka, L.P. Garrison, K.D. Mullin, T.V.N. Cole, C.B. Khan, W.A. McLellan, D.A. Pabst, and G.G. Lockhart. 2016. Habitat-based cetacean density models for the US Atlantic and Gulf of Mexico. Scientific Reports 6:22615. doi: 10.1038/Srep22615
Roberts, J.J., B.D. Best, L. Mannocci, E. Fujioka, P.N. Halpin, D.L. Palka, L.P. Garrison, K.D. Mullin, T.V.N. Cole, C.B. Khan, W.M. McLellan, D.A. Pabst, and G.G. Lockhart. 2015. Density model for Atlantic spotted dolphin (*Stenella frontalis*) for the U.S. Gulf of Mexico version 4.3, 2015-09-03, and supplementary report. Duke University, Marine Geospatial Ecology Lab: Durham, NC. 70 p.
Roden, C.L. and K.D. Mullin. 2000. Sightings of cetaceans in the northern Caribbean Sea and adjacent waters, winter 1995. Caribbean Journal of Science 36(3-4):280–288.
Soldevilla, M.S., L.P. Garrison, E. Scott-Denton, and J.M. Nance. 2015. Estimation of marine mammal bycatch mortality in the Gulf of Mexico shrimp otter trawl fishery. National Oceanic and Atmospheric Administration, National Marine Fisheries Service, Southeast Fisheries Science Center: Miami, FL. NOAA Technical Memorandum NMFS-SEFSC-672. 70 p. doi: 10.7289/V5SF2T46
Soldevilla, M.S., L.P. Garrison, E. Scott-Denton, and J. Primrose. 2021. Estimated bycatch mortality of marine mammals in the Gulf of Mexico shrimp otter trawl fishery during 2015 to 2019. National Oceanic and Atmospheric Administration, National Marine Fisheries Service, Southeast Fisheries Science Center: Miami, FL. NOAA Technical Memorandum NMFS-SEFSC-749. 78 p. doi: 10.25923/kftx-x751
Takeshita, R., L. Sullivan, C. Smith, T. Collier, A. Hall, T. Brosnan, T. Rowles, and L. Schwacke. 2017. The Deepwater Horizon oil spill marine mammal injury assessment. Endangered Species Research 33:95–106. doi: 10.3354/esr00808

Taylor, B.L., S.J. Chivers, J. Larese, and W.F. Perrin. 2007. Generation length and percent mature estimates for IUCN assessments of cetaceans. National Oceanic and Atmospheric Administration, National Marine Fisheries Service, Southwest Fisheries Science Center: La Jolla, CA. Administrative Report LJ-07-01. 24 p.

Twiner, M.J., L.J. Flewelling, S.E. Fire, S.R. Bowen-Stevens, J.K. Gaydos, C.K. Johnson, J.H. Landsberg, T.A. Leighfield, B. Mase-Guthrie, L. Schwacke, F.M. Van Dolah, Z. Wang, and T.K. Rowles. 2012. Comparative analysis of three brevetoxin-associated bottlenose dolphin (*Tursiops truncatus*) mortality events in the Florida Panhandle region (USA). PLoS ONE 7(8):e42974. doi: 10.1371/journal.pone.0042974

Venn-Watson, S., K.M. Colegrove, J. Litz, M. Kinsel, K. Terio, J. Saliki, S. Fire, R. Carmichael, C. Chevis, W. Hatchett, J. Pitchford, M. Tumlin, C. Field, S. Smith, R. Ewing, D. Fauquier, G. Lovewell, H. Whitehead, D. Rotstein, W. McFee, E. Fougeres, and T. Rowles. 2015. Adrenal gland and lung lesions in Gulf of Mexico common bottlenose dolphins (*Tursiops truncatus*) found dead following the Deepwater Horizon Oil Spill. PLoS ONE 10(5):e0126538. doi: 10.1371/journal.pone.0126538

Waring, G.T., E. Josephson, K. Maze-Foley, and P.E. Rosel. 2016. U.S. Atlantic and Gulf of Mexico marine mammal stock assessments - 2015. National Oceanic and Atmospheric Administration, National Marine Fisheries Service, Northeast Fisheries Science Center: Woods Hole, MA. NOAA Technical Memorandum NMFS-NE-238. 501 p. doi: 10.7289/V57S7KTN

Atlantic spotted dolphin − *Stenella frontalis*

Puerto Rico and US Virgin Islands Stock

Overall Vulnerability Rank = High

Biological Sensitivity = Moderate

Climate Exposure = Very High

Data Quality = 70% of scores ≥ 2

	Stenella frontalis	Expert Scores	Data Quality	Expert Scores Plots (Portion by Category)
Sensitivity attributes	Prey/Diet Specificity	1.9	2.0	
	Habitat Specificity	2.1	2.0	
	Site Fidelity	2.4	1.7	
	Lifetime Reproductive Potential	2.5	2.0	
	Generation Length	2.3	2.0	
	Reproductive Plasticity	1.7	1.0	
	Migration	3.4	2.0	
	Home Range	2.7	2.0	
	Stock Abundance	2.5	1.0	
	Stock Abundance Trend	2.6	0.7	
	Cumulative Stressors	2.9	1.7	
	**Sensitivity Score**	Moderate		
Exposure variables	Sea Surface Temperature (Change in mean)	3.5	2.0	
	Sea Surface Temperature (Change in variability)	1.7	2.0	
	Air Temperature (Change in mean)	3.3	2.0	
	Air Temperature (Change in variability)	1.3	2.0	
	Precipitation (Change in mean)	1.3	2.0	
	Precipitation (Change in variability)	1.5	2.0	
	Sea Surface Salinity (Change in mean)	2.6	2.0	
	Sea Surface Salinity (Change in variability)	1.7	2.0	
	Ocean pH (Change in mean)	3.6	2.0	
	Ocean pH (Change in variability)	1.7	2.0	
	Sea ice coverage (Change in mean)	1.3	1.3	
	Sea ice coverage (Change in variability)	1.3	1.3	
	Dissolved oxygen (Change in mean)	3.5	2.0	
	Dissolved oxygen (Change in variability)	1.9	2.0	
	Circulation	2.9	1.0	
	Sea level rise	1.6	2.0	
	**Exposure Score**	Very High		
**Overall Vulnerability Rank**		High		

Low
Moderate
High
Very High

Atlantic spotted dolphin (Puerto Rico and US Virgin Islands Stock)

Stenella frontalis

CVA Results Summary

Overall Climate Vulnerability Rank: High (51% certainty from bootstrap analysis).

Climate Exposure: Very High. Three exposure factors scored greater than 3.5: Ocean pH (Standard anomaly) (3.60), Dissolved oxygen (Standard anomaly) (3.53), and Sea Surface Temperature (Standard anomaly) (3.53).

Biological Sensitivity: Moderate. FIve sensitivity attributes scored greater than 2.5: Migration (3.40), Cumulative Stressors (2.93), Home Range (2.73), Species Abundance Trend (2.60), and Lifetime Reproductive Potential (2.53).

Distributional Response: Moderate

Abundance Response: Moderate

Phenology Response: Moderate

Data Quality: 70% of the data quality scores were 2 or greater. 55% of sensitivity attribute data quality scores were 2 or higher.

Background Information

Stock Description

The Puerto Rico and U.S. Virgin Islands Atlantic spotted dolphin stock includes individuals that are found in the waters of the U.S. Exclusive Economic Zone surrounding Puerto Rico and the U.S. Virgin Islands (Waring et al. 2012). It is considered a separate stock for management purposes, but no information exists to differentiate this stock from the western North Atlantic and Gulf of Mexico stocks (Waring et al. 2012).

Prey/Diet Specificity

Prey and diet information for Atlantic spotted dolphin in the western North Atlantic was found only in the Bahamas, where Atlantic spotted dolphin has been well-studied since the 1980's. In the Bahamas, lizardfish (order Aulopiformes, family Synodontidae) constituted >30% of female Atlantic spotted dolphin diet during all reproductive states. Other major components of the diet include fish in the orders Beloniformes (halfbeaks [family Hemiramphidae], flying fish [family Exocoetidae], needlefish [family Belonidae]), Anguilliformes (small eels [family Congridae]), Pleuronectiformes (flounders [family Bothidae]), Perciformes (jacks [family Carangidae], mackerel [family Scombridae], and tilefish [family Malacanthidae]), Labriformes (razorfish [family Labridae]), and Tetradontiformes (filefish [family Monacanthidae]; Malinowski and Herzing 2015). Atlantic spotted dolphins have been observed chasing and catching flying fish (order Beloniformes) in the Bahamas (MacLeod et al. 2004).

Atlantic spotted dolphins in the Gulf of Mexico have been observed feeding on clupeid fishes (order Clupeiformes; Fertl and Würsig 1995). They have also been observed feeding in the vicinity of shrimp trawlers (Fertl and Leatherwood 1997).

Stomach contents of three Atlantic spotted dolphins that stranded in Puerto Rico consisted of squid beaks from *Illex sp.* (Mignucci-Giannoni 1996). Atlantic spotted dolphins off Puerto Rico have been observed feeding on flying fish (Rodriguez-Ferrer et al. 2018).

Analysis of stomach contents of two Atlantic spotted dolphins in the Canary Islands showed the presence of cephalopods of the order Oegopsida (genera *Hisitiotheuthis* and *Enoploteuthis*) and crustaceans of the infraclass Cirripedia (Fernandez et al. 2009).

In Brazil, four studies provided stomach content analyses of 26 individuals (Di Beneditto et al. 2001; Melo et al. 2010; Lopes et al. 2012). These studies listed a total of six cephalopod species and 18 fish species as prey items of Atlantic spotted dolphins. Melo et al. (2010) reported *Porichthys porosissimus* (order Batrachoidiformes) as the main fish species found in 10 stomachs of stranded dolphins. Di Beneditto et al. (2001) reported *Orthopristis ruber* (order Perciformes) as the main fish species found in six incidentally captured dolphins. Both of these studies reported that the squid *Doryteuthis plei* (order Myopsida) was the primary cephalopod prey found. Based on stomach content analyses of nine Atlantic spotted dolphins incidentally caught in fishing operations in southeastern Brazil, the dolphins preyed on at least eight different fish species of the order Perciformes (families Trichiuridae, Carangidae, Sparidae, Sciaenidae, and Scombridae), order Gadiformes (family Merluccidae), order Clupeiformes (family Engraulidae), order Anguilliformes (family Congridae), order Myopsida (family Loliginidae), order Sepiida (family Sepiolidae), order Octopoda (family Tremoctopodidae), order Oegopsida (family Thysanoteuthidae), and order Decapoda (family Penaeidae; Lopes et al. 2012). Lopes et al. (2012) found that *Trichiurus lepturus* was the primary fish species represented, and *Doryteuthis plei* was the most represented cephalopod species.

Habitat Specificity

Atlantic spotted dolphins occupy both continental shelf and offshore habitats.

The larger coastal ecotype of Atlantic spotted dolphin inhabits the continental shelf and is usually found inside or near the 200m isobath, usually at least 8 to 20 km offshore (Perrin et al. 1987, 1994; Davis et al. 1998; Rice 1998; Herzing and Perrin 2018). The smaller island and offshore ecotype is known to occur in the Atlantic Ocean but not in the Gulf of Mexico (Perrin et al. 1987; Fulling et al. 2003; Mullin and Fulling 2003; Mullin and Fulling 2004; Adams and Rosel 2006).

In the Gulf of Mexico, Atlantic spotted dolphins occur primarily in continental shelf waters 10-200m deep and slope waters <500m deep, though a difference is seen between the eastern and western Gulf of Mexico (Fulling et al. 2003; Mullin and Fulling 2004; Maze-Foley and Mullin 2006). Griffin and Griffin (2003) noted a primary depth range of 20-50m in the eastern Gulf of Mexico. Mullin et al. (2004) found that Atlantic spotted dolphins were sighted in waters with a bottom depth typically <300 m. A single

satellite-tagged Atlantic spotted dolphin released in the Gulf of Mexico was found to prefer shallow-water habitat (<30 m in depth; Davis et al. 1996).

In the Caribbean, Atlantic spotted dolphins are mostly sighted along shelf areas of low sea floor relief or the shelf edge and rarely occur offshore (Mignucci-Giannoni 1998; Rodriguez-Ferrer et al. 2018).

Off Brazil, Atlantic spotted dolphins are seen in waters less than 1000m in depth (Moreno et al. 2005).

Site Fidelity

Information regarding Atlantic spotted dolphin site fidelity was not found in the literature.

Lifetime Reproductive Potential

Herzing (1997) reported Atlantic spotted dolphin mean calving interval of 2.96 years (SD = 1.224) with a range of 1-5 years in Lower Bahama Bank.

In the Bahamas, Herzing (1997) estimated age at sexual maturation for females at 8 to 15 years of age. Taylor et al. (2007) reported Atlantic spotted dolphin age at first reproduction of 12 years and age at last reproduction of 31 years based on values reported by Herzing (1997) and Perrin et al. (1994).

Generation Length

Taylor et al. (2007) reported Atlantic spotted dolphin generation length of 18.6 years at r = 0.01 and 18.3 years at r = 0.0 based on data from the Bahamas (Herzing 1997).

Reproductive Plasticity

Herzing (1997) reported peak calving periods in early spring and late fall for Atlantic spotted dolphins on Lower Bahama Bank.

Information regarding specific breeding locations and habitat was not found in the literature.

Malinowski and Herzing (2015) found that pregnant and lactating females had lower nutritional intake than non-reproductively active females in Lower Bahama Bank.

Migration

High numbers of resighted individuals in Lower Bahama Bank in successive years suggest a stable resident population of Atlantic spotted dolphins (Herzing 1997). In the Gulf of Mexico, Griffin and Griffin (2004) reported higher densities of Atlantic spotted dolphins on the west Florida continental shelf during November through May compared to June through October. Griffin et al. (2005) suggested that Atlantic spotted dolphins more often feed over the continental shelf in winter than summer.

Off Puerto Rico and the Virgin Islands, inshore-offshore seasonal movement is suspected. Atlantic spotted dolphin occurrence peaks in August and January and February (Mignucci-Giannoni 1998). There

may be a year-round occurrence of Atlantic spotted dolphins off Puerto Rico (Rodriguez-Ferrer et al. 2018).

Home Range

Information about Atlantic spotted dolphin home range in Puerto Rico and U.S. Virgin Islands waters was not found in the literature.

Elsewhere, groups have moved home ranges, such as in 2013 when half of the Little Bahama Bank Atlantic spotted dolphin community relocated 161 km south to Great Bahama Bank, which is home to a separate community of resident spotted dolphins (Herzing et al 2017).

Stock Abundance

The current abundance for the Puerto Rico and U.S. Virgin Islands stock of Atlantic spotted dolphins is unknown. Several line-transect surveys have been conducted in this area; however, due to few sightings and limitations of the survey vessel, it has not been possible to estimate abundance from these surveys (Waring et al. 2012).

During January-March 1995, a line-transect survey was conducted in deep waters surrounding the Virgin Islands and Puerto Rico that sighted 6 sightings of Atlantic spotted dolphins in U.S. waters (Roden and Mullin 2000; Waring et al. 2012). During February-March 2000, a line-transect survey was conducted in the eastern and southern Caribbean Sea that sighted one Atlantic spotted dolphin (Swartz and Burks 2000; Waring et al. 2012). During February-March 2001, during a line-transect survey in the eastern Bahamas, eastern Dominican Republic, Puerto Rico, and the Virgin Islands, 10 Atlantic spotted dolphins were sighted in U.S. waters (Swartz et al. 2002; Waring et al. 2012).

Stock Abundance Trend

The data are insufficient to evaluate population trends for this stock (Waring et al. 2012).

Cumulative Stressors

The Atlantic spotted dolphin has been taken and is still being taken in dolphin fisheries in the southern and eastern Caribbean (e.g., Caldwell et al. 1971; Caldwell and Caldwell 1975; Price 1985; Reeves 1988; Romero et al. 2001; Hoyt and Hvenegaard 2002; Mohammed et al. 2003; Vail 2005; World Council of Whalers 2008).

The Atlantic spotted dolphin may interact with gillnet and longline fisheries (Perrin et al. 1994). However, during the period 2001–2019, no fishing-related mortality or serious injury of Atlantic spotted dolphin was reported in Puerto Rico or the U.S. Virgin Islands waters (Garrison 2003; Garrison and Richards 2004; Garrison 2005; Fairfield Walsh and Garrison 2006; Fairfield-Walsh and Garrison 2007; Fairfield and Garrison 2008; Garrison et al. 2009; Garrison and Stokes 2010, 2012a, 2012b, 2013, 2014, 2016, 2017, 2019, 2020a, 2020b, 2021; Waring et al. 2012). This may represent an underestimation because there was no observer coverage within the Caribbean region for six of those years (Fairfield-Walsh and Garrison 2007; Garrison et al. 2009; Garrison and Stokes 2010, 2012b, 2013, 2016).

Legacy impacts on Atlantic spotted dolphins from naval operations at Roosevelt Roads in Puerto Rico that ceased in 2004 are unknown (Waring et al. 2012).

Coastal pollution may be an issue for the Atlantic spotted dolphin Puerto Rico and U.S. Virgin Islands stock. Parts of Vieques Island, Puerto Rico are listed on the U.S. Environmental Protection Agency's (EPA) Superfund National Priorities List due to unexploded ordnance and associated hazardous materials (Whitall et al. 2016; EPA 2018).

Distribution and Sightings

Density model results for Atlantic spotted dolphins in Puerto Rico and U.S. Virgin Island waters are presented by Mannocci et al. (2017).

Further Reading

Braulik, G. and T.A. Jefferson. 2018. *Stenella frontalis*. The IUCN Red List of Threatened Species 2018: e.T20732A50375312. https://dx.doi.org/10.2305/IUCN.UK.2018-2.RLTS.T20732A50375312.en

Department of the Navy. 2002. Marine Resource Assessment for the Puerto Rico/St. Croix Operating Area, Final Report. Naval Facilities Engineering Command, Norfolk, VA. Contract #N62470-95-D-1160, CTO 0030. Prepared by Geo-Marine, Inc, Plano, TX. Norfolk, VA. 273 p.

Herzing, D. L. and W. F. Perrin. 2018. Atlantic Spotted Dolphin *Stenella frontalis*. In: Würsig, B., Thewissen, J.G.M. and Kovacs, K.M. eds. Encyclopedia of marine mammals. Academic Press. p.40-42

Jefferson, T.A., M.A. Webber, and R.L. Pitman. 2015. Marine mammals of the world: A comprehensive guide to their identification. Second edition. San Diego, California: Academic Press.

Waring, G.T., E. Josephson, K. Maze-Foley, and P.E. Rosel. 2012. U.S. Atlantic and Gulf of Mexico Marine Mammal Stock Assessments - 2011. Northeast Fisheries Science Center: Woods Hole, MA. NOAA technical memorandum NMFS-NE-221. 319 p.

Literature Cited

Adams, L.D. and P.E. Rosel. 2005. Population differentiation of the Atlantic spotted dolphin (*Stenella frontalis*) in the western North Atlantic, including the Gulf of Mexico. Marine Biology 148(3):671–681. doi: 10.1007/s00227-005-0094-2

Caldwell, D.K. and M.C. Caldwell. 1975. Dolphin and small whale fisheries of the Caribbean and West Indies: Occurrence, history, and catch statistics — with special reference to the Lesser Antillean island of St. Vincent. Journal of the Fisheries Research Board of Canada 32(7):1105–1110. doi: 10.1139/f75-132

Caldwell, D.K., M.C. Caldwell, W.F. Rathjen, and J.R. Sullivan. 1971. Cetaceans from the Lesser Antillean island of St. Vincent. Fishery Bulletin 69:303–312.

Davis, R.W., G.S. Fargion, N. May, T.D. Leming, M. Baumgartner, W.E. Evans, L.J. Hansen, and K. Mullin. 1998. Physical habitat of cetaceans along the continental slope in the northcentral and western Gulf of Mexico. Marine Mammal Science 14(3):490–507. doi: 10.1111/j.1748-7692.1998.tb00738.x

Davis, R.W., G.A.J. Worthy, B. Würsig, S.K. Lynn, and F.I. Townsend. 1996. Diving behavior and at-sea movements of an Atlantic spotted dolphin in the Gulf of Mexico. Marine Mammal Science 12(4):569–581. doi: 10.1111/j.1748-7692.1996.tb00069.x

Di Beneditto, A.P.M., R.M.A. Ramos, S. Siciliano, R.A. dos Santos, G. Bastos, and E. Fagundes-Neto. 2001. Stomach contents of delphinids from Rio de Janeiro, southeastern Brazil. Aquatic Mammals 27(1):24–28.

Environmental Protection Agency (EPA). 2018. Atlantic Fleet Weapons Training Area, Vieques, PR. 28 August 2018, https://www.epa.gov/superfund/national-priorities-list-npl-sites-state#PR

Fairfield, C.P. and L.P. Garrison. 2008. Estimated bycatch of marine mammals and turtles in the U.S. Atlantic pelagic longline fleet during 2007. National Oceanic and Atmospheric Administration, National Marine Fisheries Service, Southeast Fisheries Science Center: Miami, FL. NOAA Technical Memorandum NMFS-SEFSC-572. 62 p.

Fairfield Walsh, C. and L.P. Garrison. 2006. Estimated bycatch of marine mammals and turtles in the U.S. Atlantic pelagic longline fleet during 2005. National Oceanic and Atmospheric Administration, National Marine Fisheries Service, Southeast Fisheries Science Center: Miami, FL. NOAA Technical Memorandum NMFS-SEFSC-539. 52 p.

Fairfield-Walsh, C. and L.P. Garrison. 2007. Estimated bycatch of marine mammals and turtles in the U.S. Atlantic pelagic longline fleet during 2006. National Oceanic and Atmospheric Administration, National Marine Fisheries Service, Southeast Fisheries Science Center: Miami, FL. NOAA Technical Memorandum NMFS-SEFSC-560. 54 p.

Fernández, R., M.B. Santos, M. Carrillo, M. Tejedor, and G.J. Pierce. 2009. Stomach contents of cetaceans stranded in the Canary Islands 1996–2006. Journal of the Marine Biological Association of the United Kingdom 89(5):873–883. doi: 10.1017/s0025315409000290

Fertl, D. and S. Leatherwood. 1997. Cetacean interactions with trawls: A preliminary review. Journal of Northwest Atlantic Fishery Science 22:219–248. doi: 10.2960/J.v22.a17

Fertl, D. and B. Würsig. 1995. Coordinated feeding by Atlantic spotted dolphins (*Stenella frontalis*) in the Gulf of Mexico. Aquatic Mammals 21(1):3–5.

Fulling, G.L., K.D. Mullin, and C.W. Hubard. 2003. Abundance and distribution of cetaceans in outer continental shelf waters of the U.S. Gulf of Mexico. Fishery Bulletin 101(4):923–932.

Garrison, L.P. 2003. Estimated bycatch of marine mammals and turtles in the U.S. Atlantic pelagic longline fleet during 2001-2002. National Oceanic and Atmospheric Administration, National Marine Fisheries Service, Southeast Fisheries Science Center: Miami, FL. NOAA Technical Memorandum NMFS-SEFSC-515. 52 p.

Garrison, L.P. 2005. Estimated bycatch of marine mammals and turtles in the U.S. Atlantic pelagic longline fleet during 2004. National Oceanic and Atmospheric Administration, National Marine Fisheries Service, Southeast Fisheries Science Center: Miami, FL. NOAA Technical Memorandum NMFS-SEFSC-531. 57 p.

Garrison, L.P. and P.M. Richards. 2004. Estimated bycatch of marine mammals and turtles in the U.S. Atlantic pelagic longline fleet during 2003. National Oceanic and Atmospheric Administration, National Marine Fisheries Service, Southeast Fisheries Science Center: Miami, FL. NOAA Technical Memorandum NMFS-SEFSC-527. 57 p.

Garrison, L.P. and L. Stokes. 2010. Estimated bycatch of marine mammals and turtles in the U.S. Atlantic pelagic longline fleet during 2009. National Oceanic and Atmospheric Administration, National Marine Fisheries Service, Southeast Fisheries Science Center: Miami, FL. NOAA Technical Memorandum NMFS-SEFSC-607. 64 p.

Garrison, L.P. and L. Stokes. 2012. Estimated bycatch of marine mammals and sea turtles in the U.S. Atlantic pelagic longline fleet during 2010. National Oceanic and Atmospheric Administration, National Marine Fisheries Service, Southeast Fisheries Science Center: Miami, FL. NOAA Technical Memorandum NMFS-SEFSC-624. 59 p.

Garrison, L.P. and L. Stokes. 2012. Estimated bycatch of marine mammals and sea turtles in the U.S. Atlantic pelagic longline fleet during 2011. National Oceanic and Atmospheric Administration,

National Marine Fisheries Service, Southeast Fisheries Science Center: Miami, FL. NOAA Technical Memorandum NMFS-SEFSC-632. 61 p.
Garrison, L.P. and L. Stokes. 2013. Estimated bycatch of marine mammals and sea turtles in the U.S. Atlantic pelagic longline fleet during 2012. National Oceanic and Atmospheric Administration, National Marine Fisheries Service, Southeast Fisheries Science Center: Miami, FL. NOAA Technical Memorandum NMFS-SEFSC-655. 62 p.
Garrison, L.P. and L. Stokes. 2014. Estimated bycatch of marine mammals and sea turtles in the U.S. Atlantic pelagic longline fleet during 2013. National Oceanic and Atmospheric Administration, National Marine Fisheries Service, Southeast Fisheries Science Center: Miami, FL. NOAA Technical Memorandum NMFS-SEFSC-667. 61 p.
Garrison, L.P. and L. Stokes. 2016. Estimated bycatch of marine mammals and sea turtles in the U.S. Atlantic pelagic longline fleet during 2014. National Oceanic and Atmospheric Administration, National Marine Fisheries Service, Southeast Fisheries Science Center: Miami, FL. NOAA Technical Memorandum NMFS-SEFSC-696. 62 p. doi: 10.7289/V5/TM-SEFSC-696
Garrison, L.P. and L. Stokes. 2017. Estimated bycatch of marine mammals and sea turtles in the U.S. Atlantic pelagic longline fleet during 2015. National Oceanic and Atmospheric Administration, National Marine Fisheries Service, Southeast Fisheries Science Center: Miami, FL. NOAA Technical Memorandum NMFS-SEFSC-709. 61 p. doi: 10.25923/kaf6-5c33
Garrison, L.P. and L. Stokes. 2019. Estimated bycatch of marine mammals and sea turtles in the U.S. Atlantic pelagic longline fleet during 2016. National Oceanic and Atmospheric Administration, National Marine Fisheries Service, Southeast Fisheries Science Center: Miami, FL. Southeast Fisheries Science Center Reference Document PRBD-2019-01. 43 p. doi: 10.25923/d893-d142
Garrison, L.P. and L. Stokes. 2020. Estimated bycatch of marine mammals and sea turtles in the U.S. Atlantic pelagic longline fleet during 2017. National Oceanic and Atmospheric Administration, National Marine Fisheries Service, Southeast Fisheries Science Center: Miami, FL. Southeast Fisheries Science Center Reference Document PRBD 2020-05. 58 p. doi: 10.25923/a3hs-ea60
Garrison, L.P. and L. Stokes. 2020. Estimated bycatch of marine mammals and sea turtles in the U.S. Atlantic pelagic longline fleet during 2018. National Oceanic and Atmospheric Administration, National Marine Fisheries Service, Southeast Fisheries Science Center: Miami, FL. Southeast Fisheries Science Center Reference Document PRBD 2020-08. 44 p. doi: 10.25923/777n-w316
Garrison, L.P., L. Stokes, and C. Fairfield. 2009. Estimated bycatch of marine mammals and sea turtles in the U.S. Atlantic pelagic longline fleet during 2008. National Oceanic and Atmospheric Administration, National Marine Fisheries Service, Southeast Fisheries Science Center: Miami, FL. NOAA Technical Memorandum NMFS-SEFSC-591. 63 p.
Garrison, L.P. and L.W. Stokes. 2021. Estimated bycatch of marine mammals and sea turtles in the U.S. Atlantic pelagic longline fleet during 2019. doi: 10.25923/asb3-6q44
Griffin, B.R. and N.J. Griffin. 2004. Temporal variation in Atlantic spotted dolphin (*Stenella frontalis*) and bottlenose dolphin (*Tursiops truncatus*) densities on the West Florida Continental Shelf. Aquatic Mammals 30(3):380–390. doi: 10.1578/am.30.3.2004.380
Griffin, R.B. and N.J. Griffin. 2003. Distribution, habitat partitioning, and abundance of Atlantic spotted dolphins, bottlenose dolphins, and loggerhead sea turtles on the eastern Gulf of Mexico continental shelf. Gulf of Mexico Science 21(1):23–34. doi: 10.18785/goms.2101.03
Griffin, R.B., C. Hu, and N.J. Griffin. 2005. Movement patterns of Atlantic spotted dolphins (*Stenella frontalis*) and bottlenose dolphins (*Tursiops truncatus*) in relation to oceanographic fronts. Sixteenth Biennial Conference on the Biology of Marine Mammals, San Diego, CA, 12-16 December. p. 112.

Herzing, D.L. 1997. The life history of free-ranging atlantic spotted dolphins (*Stenella frontalis*): Age classes, color phases, and female reproduction. Marine Mammal Science 13(4):576–595. doi: 10.1111/j.1748-7692.1997.tb00085.x

Herzing, D.L., B.N. Augliere, C.R. Elliser, M.L. Green, and A.A. Pack. 2017. Exodus! Large-scale displacement and social adjustments of resident Atlantic spotted dolphins (*Stenella frontalis*) in the Bahamas. PLoS ONE 12(8):e0180304. doi: 10.1371/journal.pone.0180304

Herzing, D.L. and W.F. Perrin. 2018. Atlantic spotted dolphin: *Stenella frontalis*. In: Würsig, B., J.G.M. Thewissen, and K.M. Kovacs (Eds.), Encyclopedia of Marine Mammals (Third Edition). Academic Press. pp. 40–42. doi: 10.1016/B978-0-12-804327-1.00050-9

Hoyt, E. and G.T. Hvenegaard. 2002. A review of whale-watching and whaling with applications for the Caribbean. Coastal Management 30(4):381–399. doi: 10.1080/089207502900273

Lopes, X.M., M.C.d.O. Santos, E. da Silva, M. Bassoi, and R.A. dos Santos. 2012. Feeding habits of the Atlantic spotted dolphin, *Stenella frontalis*, in southeastern Brazil. Brazilian Journal of Oceanography 60(2):189–198. doi: 10.1590/S1679-87592012000200009

MacLeod, C.D., N. Hauser, and H. Peckham. 2004. Diversity, relative density and structure of the cetacean community in summer months east of Great Abaco, Bahamas. Journal of the Marine Biological Association of the United Kingdom 84(2):469–474. doi: 10.1017/S0025315404009476h

Malinowski, C.R. and D.L. Herzing. 2015. Prey use and nutritional differences between reproductive states and age classes in Atlantic spotted dolphins (*Stenella frontalis*) in the Bahamas. Marine Mammal Science 31(4):1471–1493. doi: 10.1111/mms.12238

Mannocci, L., J.J. Roberts, D.L. Miller, and P.N. Halpin. 2017. Extrapolating cetacean densities to quantitatively assess human impacts on populations in the high seas. Conservation Biology 31(3):601–614. doi: 10.1111/cobi.12856

Maze-Foley, K. and K.D. Mullin. 2006. Cetaceans of the oceanic northern Gulf of Mexico: Distributions, group sizes and interspecific associations. Journal of Cetacean Research and Management 8(2):203–213.

Melo, C.L.C., R.A. Santos, M. Bassoi, A.C. Araújo, J. Lailson-Brito, P.R. Dorneles, and A.F. Azevedo. 2010. Feeding habits of delphinids (Mammalia: Cetacea) from Rio de Janeiro State, Brazil. Journal of the Marine Biological Association of the United Kingdom 90(8):1509–1515. doi: 10.1017/s0025315409991639

Mignucci-Giannoni, A.A. 1996. Marine mammal strandings in Puerto Rico and the United States and British Virgin Islands. University of Puerto Rico. Dissertation. 247 p.

Mignucci-Giannoni, A.A. 1998. Zoogeography of cetaceans off Puerto Rico and the Virgin Islands. Caribbean Journal of Science 34(3-4):173–190.

Mohammed, E., L.E. Straker, and C. Jardine. 2003. St. Vincent and the Grenadines: Reconstructed fisheries catches and fishing effort, 1942-2001. Fisheries Centre Research Reports 11(6):95–116.

Moreno, I.B., A.N. Zerbini, D. Danilewicz, S. de Oliveira Sa, P.C. Simões-Lopes, J. Lailson-Brito, and A.F. Azevedo. 2005. Distribution and habitat characteristics of dolphins of the genus *Stenella* (Cetacea: Delphinidae) in the Southwest Atlantic Ocean. Marine Ecology Progress Series 300:229–240. doi: 10.3354/meps300229

Mullin, K.D. and G.L. Fulling. 2003. Abundance of cetaceans in the southern U.S. North Atlantic Ocean during summer 1998. Fishery Bulletin 101:603–613.

Mullin, K.D. and G.L. Fulling. 2004. Abundance of cetaceans in the oceanic northern Gulf of Mexico, 1996–2001. Marine Mammal Science 20(4):787–807. doi: 10.1111/j.1748-7692.2004.tb01193.x

Mullin, K.D., W. Hoggard, and L.J. Hansen. 2004. Abundance and seasonal occurrence of cetaceans in outer continental shelf and slope waters of the North-central and northwestern Gulf of Mexico. Gulf of Mexico Science 22(1):62–73. doi: 10.18785/goms.2201.06

Perrin, W.F., D.K. Caldwell, and M.C. Caldwell. 1994. Atlantic spotted dolphin *Stenella frontalis* (G. Cuvier, 1829). In: Ridgway, S.H. and R. Harrison (Eds.), Handbook of marine mammals. Volume 5: The first book of dolphins. Academic Press, London, UK. pp. 173–190.

Perrin, W.F., E.D. Mitchell, J.G. Mead, D.K. Caldwell, M.C. Caldwell, P.J.H. Bree, and W.H. Dawbin. 1987. Revision of the spotted dolphins, *Stenella* spp. Marine Mammal Science 3(2):99–170. doi: 10.1111/j.1748-7692.1987.tb00158.x

Price, W.S. 1985. Whaling in the Caribbean: Historical perspective and update. Reports of the International Whaling Commission 35:413–420.

Reeves, R. 1988. Exploitation of cetaceans in St. Lucia, Lesser Antilles, January 1987. Reports of the International Whaling Commission 38(445-447).

Rice, D.W. 1998. Marine mammals of the world, systematics and distribution. Special Publication No. 4. The Society for Marine Mammalogy, Lawrence, KS. 231 p.

Roden, C.L. and K.D. Mullin. 2000. Sightings of cetaceans in the northern Caribbean Sea and adjacent waters, winter 1995. Caribbean Journal of Science 36(3-4):280–288.

Rodriguez-Ferrer, G., R. Reyes, N.M. Hammerman, and J.E. García-Hernández. 2018. Cetacean sightings in Puerto Rican waters: including the first underwater photographic documentation of a minke whale (*Balaenoptera acutorostrata*). Latin American Journal of Aquatic Mammals 13(1-2):26–36. doi: 10.5597/00246

Romero, A., A.I. Agudo, S.M. Green, and G. Notarbartolo di Sciara. 2001. Cetaceans of Venezuela: Their distribution and conservation status. National Oceanic and Atmospheric Administration, National Marine Fisheries Service: Seattle, WA. NOAA Technical Report NMFS 151. 60 p.

Swartz, S.L. and C. Burks. 2000. Cruise results, Windwards humpback (*Megaptera novaeangliae*) survey: NOAA ship Gordon Gunter cruise GU-00-01, 9 February to 3 April 2000. National Oceanic and Atmospheric Administration, National Marine Fisheries Service, Southeast Fisheries Science Center: Miami, FL. NOAA Technical Memorandum NMFS-SEFSC-438. 31 p.

Swartz, S.L., A. Martinez, J. Stamates, C. Burks, and A.A. Mignucci-Giannoni. 2002. Acoustic and visual survey of cetaceans in the waters of Puerto Rico and the Virgin Islands: February–March 2001. National Oceanic and Atmospheric Administration, National Marine Fisheries Service, Southeast Fisheries Science Center: Miami, FL. NOAA Technical Memorandum NMFS-SEFSC-463. 62 p.

Taylor, B.L., S.J. Chivers, J. Larese, and W.F. Perrin. 2007. Generation length and percent mature estimates for IUCN assessments of cetaceans. National Oceanic and Atmospheric Administration, National Marine Fisheries Service, Southwest Fisheries Science Center: La Jolla, CA. Administrative Report LJ-07-01. 24 p.

Vail, C. 2005. Socio-economic assessment of marine mammal utilization in the wider Caribbean region: Captivity, viewing and hunting. Information paper presented at the United Nations Environment Programme Regional Workshop of Experts on the Development of the Marine Mammal Action Plan for the Wider Caribbean Region, Barbados, 18-21 July 2005.

Waring, G.T., E. Josephson, K. Maze-Foley, and P.E. Rosel. 2012. U.S. Atlantic and Gulf of Mexico marine mammal stock assessments - 2011. National Oceanic and Atmospheric Administration, National Marine Fisheries Service, Northeast Fisheries Science Center: Woods Hole, MA. NOAA Technical Memorandum NMFS-NE-221. 319 p.

Whitall, D.R., A.L. Mason, M.H. Fulton, E.F. Wirth, D. Wehner, A. Ramos, A.S. Pait, B. West, E. Pisarski, B. Shaddrix, and L.A. Reed. 2016. Chemical contaminants in the marine resources of Vieques, Puerto Rico. National Oceanic Atmospheric Administration, National Ocean Service, National Centers for Coastal Ocean Science: Silver Spring, MD. NOAA Technical Memorandum NOS-NCCOS-223. 70 p. doi: 10.7289/V5/TM-NOS-NCCOS-223

World Council of Whalers. 2008. World whaling – Caribbean. http://www.worldwhalers.com/whaling_around_the_world/caribbean.htm

Atlantic spotted dolphin − *Stenella frontalis*

Western North Atlantic Stock

Overall Vulnerability Rank = Moderate

Biological Sensitivity = Low

Climate Exposure = Very High

Data Quality = 85% of scores ≥ 2

	Stenella frontalis	Expert Scores	Data Quality	Expert Scores Plots (Portion by Category)
Sensitivity attributes	Prey/Diet Specificity	1.2	2.0	
	Habitat Specificity	1.7	2.0	
	Site Fidelity	2.0	2.0	
	Lifetime Reproductive Potential	1.9	2.7	
	Generation Length	1.9	2.0	
	Reproductive Plasticity	1.0	2.0	
	Migration	3.3	1.7	
	Home Range	2.2	1.7	
	Stock Abundance	1.0	3.0	
	Stock Abundance Trend	1.8	1.3	
	Cumulative Stressors	1.3	1.7	
	Sensitivity Score	Low		
Exposure variables	Sea Surface Temperature (Change in mean)	3.5	2.3	
	Sea Surface Temperature (Change in variability)	1.0	2.0	
	Air Temperature (Change in mean)	3.0	2.7	
	Air Temperature (Change in variability)	1.0	3.0	
	Precipitation (Change in mean)	1.1	2.3	
	Precipitation (Change in variability)	1.3	2.3	
	Sea Surface Salinity (Change in mean)	3.3	2.7	
	Sea Surface Salinity (Change in variability)	1.7	2.7	
	Ocean pH (Change in mean)	3.9	3.0	
	Ocean pH (Change in variability)	1.3	2.3	
	Sea ice coverage (Change in mean)	1.0	2.7	
	Sea ice coverage (Change in variability)	1.0	2.7	
	Dissolved oxygen (Change in mean)	3.5	3.0	
	Dissolved oxygen (Change in variability)	1.2	2.3	
	Circulation	2.1	2.7	
	Sea level rise	1.3	2.7	
	Exposure Score	Very High		
	Overall Vulnerability Rank	Moderate		

Low
Moderate
High
Very High

Atlantic spotted dolphin (Western North Atlantic Stock)

Stenella frontalis

CVA Results Summary

Overall Climate Vulnerability Rank: Moderate (49% certainty from bootstrap analysis).

Climate Exposure: Very High. Three exposure factors scored greater than 3.5: Ocean pH (Standard anomaly) (3.87), Dissolved oxygen (Standard anomaly) (3.53), and Sea Surface Temperature (Standard anomaly) (3.53).

Biological Sensitivity: Low. Only one sensitivity attribute scored greater than 2.5: Migration (3.33).

Distributional Response: High

Abundance Response: Low

Phenology Response: Low

Data Quality: 85% of the data quality scores were 2 or greater. 64% of sensitivity attribute data quality scores were 2 or higher.

Background Information

Stock Description

The Atlantic spotted dolphin western North Atlantic stock includes individuals that are found in the waters of the U.S. Exclusive Economic Zone in the western North Atlantic (Hayes et al. 2020).

Prey/Diet Specificity

Prey and diet information for Atlantic spotted dolphin in the western North Atlantic was found only in the Bahamas, where Atlantic spotted dolphins have been well-studied since the 1980s. In the Bahamas, lizardfish (order Aulopiformes, family Synodontidae) constituted >30% of female Atlantic spotted dolphin diet during all reproductive states. Other major components of the diet included fish in the orders Beloniformes (halfbeaks [family Hemiramphidae], flyingfish [family Exocoetidae], needlefish [family Belonidae]), Anguilliformes (small eels [family Congridae]), Pleuronectiformes (flounders [family Bothidae]), Perciformes (jacks [family Carangidae], mackerel [family Scombridae], and tilefish [family Malacanthidae]), Labriformes (razorfish [family Labridae]), and Tetradontiformes (filefish [family Monacanthidae]; Malinowski and Herzing 2015). Atlantic spotted dolphins have been observed chasing and catching flying fish (order Beloniformes) in the Bahamas (MacLeod et al. 2004).

Atlantic spotted dolphins in the Gulf of Mexico have been observed feeding on clupeid fishes (order Clupeiformes; Fertl and Würsig 1995). They have also been observed feeding in the vicinity of shrimp trawlers (Fertl and Leatherwood 1997).

Analysis of stomach contents of two Atlantic spotted dolphins in the Canary Islands showed the presence of cephalopods of the order Oegopsida (genera *Hisitiotheuthis* and *Enoploteuthis*) and crustaceans of the infraclass Cirripedia (Fernandez et al. 2009).

In Brazil, four studies combined for stomach content analysis of 26 individuals (Di Beneditto et al. 2001; Melo et al. 2010; Lopes et al. 2012). These studies listed a total of six cephalopod species and 18 fish species as prey items of Atlantic spotted dolphins. Melo et al. (2010) reported *Porichthys porosissimus* (order Batrachoidiformes) as the main fish species found in 10 stomachs of stranded dolphins. Di Beneditto et al. (2001) reported *Orthopristis ruber* (order Perciformes) as the main fish species found in six incidentally captured dolphins. Both studies reported the squid *Doryteuthis plei* (order Myopsida) was the most important cephalopod prey. Based on stomach content analysis of nine Atlantic spotted dolphins incidentally caught in fishing operations in southeastern Brazil, dolphins assessed preyed on at least eight different fish species of the order Perciformes (families Trichiuridae, Carangidae, Sparidae, Sciaenidae, and Scombridae), order Gadiformes (family Merluccidae), Order Clupeiformes (family Engraulidae), Order Anguilliformes (family Congridae), order Myopsida (family Loliginidae), order Sepiida (family Sepiolidae), order Octopoda (family Tremoctopodidae), and order Oegopsida (family Thysanoteuthidae), and and order Decapoda (family Penaeidae; Lopes et al. 2012). Lopes et al. (2012) found *Trichiurus lepturus* was the most important fish species represented and *Doryteuthis plei* was the most represented cephalopod species.

Habitat Specificity

The larger coastal ecotype of Atlantic spotted dolphin inhabits waters over the continental shelf and is usually found inside the 200m isobath, usually at least 8 to 20 km offshore (Perrin et al. 1987, 1994; Davis et al. 1998; Rice 1998; Herzing and Perrin 2018). The smaller island and offshore ecotype is known to occur in the Atlantic Ocean but not in the Gulf of Mexico (Fulling et al. 2003; Mullin and Fulling 2003; Mullin and Fulling 2004; (Viricel and Rosel 2014). Although the two ecotypes are currently considered a single stock, genetic and morphometric evidence suggests they are on distinct evolutionary trajectories and could be considered distinct subspecies (Viricel and Rosel 2014).

Atlantic spotted dolphins are often sighted beyond the shelf break in the Caribbean, and off the Atlantic U.S. coast (Mills and Rademacher 1996; Roden and Mullin 2000; Fulling et al. 2003; Mullin and Fulling 2003; Mullin et al. 2004).

In the Gulf of Mexico, Atlantic spotted dolphin occurs primarily in continental shelf waters 10-200m deep and slope waters <500m deep, though a difference is seen between the eastern and western Gulf of Mexico (Fulling et al. 2003; Mullin and Fulling 2004; Maze-Foley and Mullin 2006). Griffin and Griffin (2003) noted a primary depth range of 20-50m in the eastern Gulf of Mexico. Mullin et al. (2004) found that Atlantic spotted dolphins were sighted in waters with a bottom depth typically <300 m. A single satellite-tagged Atlantic spotted dolphin released in the Gulf of Mexico was found to prefer shallow-water habitat (<30 m in depth; Davis et al. 1996).

Off Brazil, Atlantic spotted dolphins are seen in waters less than 1000m in depth (Moreno et al. 2005).

The Atlantic spotted dolphin, which is endemic to the warm-temperate, subtropical, and tropical Atlantic Ocean, occurs in two forms which vary in size and degree of spotting (Perrin et al. 1987; Perrin 2001; Adams and Rosel 2006). First, there is a large, heavily spotted form which inhabits continental

shelf waters generally inside or near the 200m isobath in the western North Atlantic in the Gulf of Mexico, and the Caribbean (e.g., Perrin et al. 1987; Fulling et al. 2003; Mullin and Fulling 2003; Mullin and Fulling 2004; Viricel and Rosel 2014), and second, a smaller, less spotted island and offshore form which occurs in pelagic waters of the the Atlantic Ocean and Caribbean Sea (Perrin et al. 1987; Adams and Rosel 2006). Future study may result in the different forms being made distinct subspecies (Rice 1998).

Site Fidelity

Information regarding Atlantic spotted dolphin site fidelity was not found in the literature.

Lifetime Reproductive Potential

Herzing (1997) reported Atlantic spotted dolphin mean calving interval of 2.96 years (SD = 1.224) with a range of 1-5 years in Lower Bahama Bank.

In the Bahamas, Herzing (1997) estimated age at sexual maturation for females at 8 to 15 years of age. Taylor et al. (2007) reported Atlantic spotted dolphin age at first reproduction of 12 years and age at last reproduction of 31 years based on values reported by Herzing (1997) and Perrin et al. (1994).

Generation Length

Taylor et al. (2007) reported Atlantic spotted dolphin generation length of 18.6 years at $r = 0.01$ and 18.3 years at $r = 0.0$ based on data from the Bahamas (Herzing 1997).

Reproductive Plasticity

Herzing (1997) reported peak calving periods in early spring and late fall for Atlantic spotted dolphins on Lower Bahama Bank.

Information regarding specific breeding locations and habitat was not found in the literature.

Malinowski and Herzing (2015) found that pregnant and lactating females had lower nutritional intake than non-reproductively active females in Lower Bahama Bank.

Migration

On Little Bahama Bank, sympatric communities of resident Atlantic spotted dolphins with stable size and social structure coexist with little immigration/emigration (Herzing 1997; Herzing et al. 2017). U.S. Navy assessments of Operating Areas suggested Atlantic spotted dolphins should be expected in the southern Navy Operating Areas in all seasons (DoN 2007, 2008a, 2008b, 2008c), and in the Northeast Operating Areas during the spring, summer, and fall, with greatest occurrence occurring during the summer (DoN 2005).

Home Range

In the Bahamas, groups have moved home ranges, such as in 2013 when half of the Little Bahama Bank Atlantic spotted dolphin community relocated 161 km south to Great Bahama Bank, which is home to a separate community of resident Atlantic spotted dolphins (Herzing et al. 2017).

Information about Atlantic spotted dolphin home range in other areas was not found in the literature.

Stock Abundance

Garrison (2020) and Palka (2020) estimated Atlantic spotted dolphin abundance in the western North Atlantic at 39,921 individuals (CV=0.27) based on 2016 surveys.

Stock Abundance Trend

A statistically significant linear decrease was detected in Atlantic spotted dolphin abundance using a generalized linear model and data from 2016, 2011, and 2004 (Hayes et al. 2020). Atlantic spotted dolphin abundance in the western North Atlantic was estimated at 44,715 individuals (CV=0.43), based on summer 2011 surveys covering waters from central Florida to the lower Bay of Fundy (Waring et al. 2014). The best 2004 abundance estimate for Atlantic spotted dolphins in the western North Atlantic was 50,978 individuals (CV=0.42), based on two 2004 surveys, one from the northern U.S. Atlantic and one from the southern U.S. Atlantic. (Waring et al. 2006).

Cumulative Stressors

The Atlantic spotted dolphin in the western North Atlantic may interact with gillnet and longline fisheries (Perrin et al. 1994). However, in the 2015–2019 time period, no fishery-induced mortality was reported (Garrison and Stokes 2017, 2019, 2020a, 2020b, 2021). Atlantic spotted dolphins are known to strand along the Atlantic coast, with 21 individuals reported stranded between North Carolina and Florida during the period 2013–2017 (NOAA National Marine Mammal Health and Stranding Response Database as cited by Hayes et al. 2020).

Distribution and Sightings

Density model results for Atlantic spotted dolphin in the western North Atlantic are presented by Roberts et al. (2016a, 2016b, 2017, 2018), Mannocci et al. (2017), Chavez-Rosales et al. (2019), and Palka et al. (2021a, 2021b).

Further Reading

Braulik, G. and T.A. Jefferson. 2018. *Stenella frontalis*. The IUCN Red List of Threatened Species 2018: e.T20732A50375312. https://dx.doi.org/10.2305/IUCN.UK.2018-2.RLTS.T20732A50375312.en

Department of the Navy. 2009. Marine Resources Assessment for the Chesapeake Bay. Department of the Navy, U.S. Fleet Forces Command, Norfolk, VA. Contract #N62470-D-9997, CTO 0127. Prepared by Geo-Marine, Inc., Hampton, VA. 393 p.

Hayes, S.A., E. Josephson, K. Maze-Foley, and P.E. Rosel. 2020. US Atlantic and Gulf of Mexico Marine Mammal Stock Assessments - 2019. Northeast Fisheries Science Center: Woods Hole, MA. NOAA technical memorandum NMFS-NE-264. 479 p.

Herzing, D.L. and W.F. Perrin. 2018. Atlantic Spotted Dolphin: Stenella frontalis. In: Würsig, B., J.G.M. Thewissen, and K.M. Kovacs (Eds.), Encyclopedia of Marine Mammals (Third Edition). Academic Press. pp. 40-42. doi: 10.1016/B978-0-12-804327-1.00050-9

Literature Cited

Adams, L.D. and P.E. Rosel. 2005. Population differentiation of the Atlantic spotted dolphin (*Stenella frontalis*) in the western North Atlantic, including the Gulf of Mexico. Marine Biology 148(3):671–681. doi: 10.1007/s00227-005-0094-2

Chavez-Rosales, S., D.L. Palka, L.P. Garrison, and E.A. Josephson. 2019. Environmental predictors of habitat suitability and occurrence of cetaceans in the western North Atlantic Ocean. Scientific Reports 9(1):5833. doi: 10.1038/s41598-019-42288-6

Davis, R.W., G.S. Fargion, N. May, T.D. Leming, M. Baumgartner, W.E. Evans, L.J. Hansen, and K. Mullin. 1998. Physical habitat of cetaceans along the continental slope in the northcentral and western Gulf of Mexico. Marine Mammal Science 14(3):490–507. doi: 10.1111/j.1748-7692.1998.tb00738.x

Davis, R.W., G.A.J. Worthy, B. Würsig, S.K. Lynn, and F.I. Townsend. 1996. Diving behavior and at-sea movements of an Atlantic spotted dolphin in the Gulf of Mexico. Marine Mammal Science 12(4):569–581. doi: 10.1111/j.1748-7692.1996.tb00069.x

Department of the Navy (DoN). 2005. Marine resource assessment for the Northeast Operating Areas: Atlantic City, Narragansett Bay, and Boston. Naval Facilities Engineering Command, Atlantic. Norfolk, VA. Contract #N63470-02-D-9997, Task Order 0018. Prepared by Geo-Marine Inc., Newport News, VA. 382 p.

Department of the Navy (DoN). 2007. Marine resources assessment update for the Southeastern Florida and AUTEC-Andros Operating Area, Final Report. Naval Facilities Engineering Command, Norfolk, VA. Contract #N62470-D-9997, CTO 0034. Prepared by Geo-Marine, Inc., Hampton, VA. 575 p.

Department of the Navy (DoN). 2008. Marine resources assessment update for the Charleston/Jacksonville Operating Area. Naval Facilities Engineering Command, Atlantic, Norfolk, VA. Contract #N62470-D-9997, CTO 0056. Prepared by Geo-Marine, Inc., Hampton, VA. 663 p.

Department of the Navy (DoN). 2008. Marine resources assessment update for the Cherry Point Operating Area. Department of the Navy, U.S. Fleet Forces Command, Norfolk, VA. Contract #N62470-D-9997, CTO 0056. Prepared by Geo-Marine, Inc., Hampton, VA. 643 p.

Department of the Navy (DoN). 2008. Marine resources assessment update for the Virginia Capes Operating Area. Department of the Navy, U.S. Fleet Forces Command, Norfolk, VA. Contract #N62470-D-9997, CTO 0056. Prepared by Geo-Marine, Inc., Hampton, VA. 711 p.

Di Beneditto, A.P.M., R.M.A. Ramos, S. Siciliano, R.A. dos Santos, G. Bastos, and E. Fagundes-Neto. 2001. Stomach contents of delphinids from Rio de Janeiro, southeastern Brazil. Aquatic Mammals 27(1):24–28.

Fernández, R., M.B. Santos, M. Carrillo, M. Tejedor, and G.J. Pierce. 2009. Stomach contents of cetaceans stranded in the Canary Islands 1996–2006. Journal of the Marine Biological Association of the United Kingdom 89(5):873–883. doi: 10.1017/s0025315409000290

Fertl, D. and S. Leatherwood. 1997. Cetacean interactions with trawls: A preliminary review. Journal of Northwest Atlantic Fishery Science 22:219–248. doi: 10.2960/J.v22.a17

Fertl, D. and B. Würsig. 1995. Coordinated feeding by Atlantic spotted dolphins (*Stenella frontalis*) in the Gulf of Mexico. Aquatic Mammals 21(1):3–5.

Fulling, G.L., K.D. Mullin, and C.W. Hubard. 2003. Abundance and distribution of cetaceans in outer continental shelf waters of the U.S. Gulf of Mexico. Fishery Bulletin 101(4):923–932.

Garrison, L.P. 2020. Abundance of marine mammals in waters of the U.S. southeastern Atlantic during summer 2016. National Oceanic and Atmospheric Administration, National Marine Fisheries Service, Southeast Fisheries Science Center, Protected Resources and Biodiversity Division: Miami, FL. PRBD-2020-04. 17 p.

Garrison, L.P. and L. Stokes. 2017. Estimated bycatch of marine mammals and sea turtles in the U.S. Atlantic pelagic longline fleet during 2015. National Oceanic and Atmospheric Administration, National Marine Fisheries Service, Southeast Fisheries Science Center: Miami, FL. NOAA Technical Memorandum NMFS-SEFSC-709. 61 p. doi: 10.25923/kaf6-5c33

Garrison, L.P. and L. Stokes. 2019. Estimated bycatch of marine mammals and sea turtles in the U.S. Atlantic pelagic longline fleet during 2016. National Oceanic and Atmospheric Administration, National Marine Fisheries Service, Southeast Fisheries Science Center: Miami, FL. Southeast Fisheries Science Center Reference Document PRBD-2019-01. 43 p. doi: 10.25923/d893-d142

Garrison, L.P. and L. Stokes. 2020. Estimated bycatch of marine mammals and sea turtles in the U.S. Atlantic pelagic longline fleet during 2017. National Oceanic and Atmospheric Administration, National Marine Fisheries Service, Southeast Fisheries Science Center: Miami, FL. Southeast Fisheries Science Center Reference Document PRBD 2020-05. 58 p. doi: 10.25923/a3hs-ea60

Garrison, L.P. and L. Stokes. 2020. Estimated bycatch of marine mammals and sea turtles in the U.S. Atlantic pelagic longline fleet during 2018. National Oceanic and Atmospheric Administration, National Marine Fisheries Service, Southeast Fisheries Science Center: Miami, FL. Southeast Fisheries Science Center Reference Document PRBD 2020-08. 44 p. doi: 10.25923/777n-w316

Garrison, L.P. and L.W. Stokes. 2021. Estimated bycatch of marine mammals and sea turtles in the U.S. Atlantic pelagic longline fleet during 2019. doi: 10.25923/asb3-6q44

Griffin, R.B. and N.J. Griffin. 2003. Distribution, habitat partitioning, and abundance of Atlantic spotted dolphins, bottlenose dolphins, and loggerhead sea turtles on the eastern Gulf of Mexico continental shelf. Gulf of Mexico Science 21(1):23–34. doi: 10.18785/goms.2101.03

Hayes, S.A., E. Josephson, K. Maze-Foley, and P.E. Rosel. 2020. US Atlantic and Gulf of Mexico marine mammal stock assessments - 2019. National Oceanic and Atmospheric Administration, National Marine Fisheries Service, Northeast Fisheries Science Center: Woods Hole, MA. NOAA Technical Memorandum NMFS-NE-264. 479 p.

Herzing, D.L. 1997. The life history of free-ranging atlantic spotted dolphins (*Stenella frontalis*): Age classes, color phases, and female reproduction. Marine Mammal Science 13(4):576–595. doi: 10.1111/j.1748-7692.1997.tb00085.x

Herzing, D.L., B.N. Augliere, C.R. Elliser, M.L. Green, and A.A. Pack. 2017. Exodus! Large-scale displacement and social adjustments of resident Atlantic spotted dolphins (*Stenella frontalis*) in the Bahamas. PLoS ONE 12(8):e0180304. doi: 10.1371/journal.pone.0180304

Herzing, D.L. and W.F. Perrin. 2018. Atlantic spotted dolphin: *Stenella frontalis*. In: Würsig, B., J.G.M. Thewissen, and K.M. Kovacs (Eds.), Encyclopedia of Marine Mammals (Third Edition). Academic Press. pp. 40–42. doi: 10.1016/B978-0-12-804327-1.00050-9

Lopes, X.M., M.C.d.O. Santos, E. da Silva, M. Bassoi, and R.A. dos Santos. 2012. Feeding habits of the Atlantic spotted dolphin, *Stenella frontalis*, in southeastern Brazil. Brazilian Journal of Oceanography 60(2):189–198. doi: 10.1590/S1679-87592012000200009

MacLeod, C.D., N. Hauser, and H. Peckham. 2004. Diversity, relative density and structure of the cetacean community in summer months east of Great Abaco, Bahamas. Journal of the Marine Biological Association of the United Kingdom 84(2):469–474. doi: 10.1017/S0025315404009476h

Malinowski, C.R. and D.L. Herzing. 2015. Prey use and nutritional differences between reproductive states and age classes in Atlantic spotted dolphins (*Stenella frontalis*) in the Bahamas. Marine Mammal Science 31(4):1471–1493. doi: 10.1111/mms.12238

Mannocci, L., J.J. Roberts, D.L. Miller, and P.N. Halpin. 2017. Extrapolating cetacean densities to quantitatively assess human impacts on populations in the high seas. Conservation Biology 31(3):601–614. doi: 10.1111/cobi.12856

Maze-Foley, K. and K.D. Mullin. 2006. Cetaceans of the oceanic northern Gulf of Mexico: Distributions, group sizes and interspecific associations. Journal of Cetacean Research and Management 8(2):203–213.

Melo, C.L.C., R.A. Santos, M. Bassoi, A.C. Araújo, J. Lailson-Brito, P.R. Dorneles, and A.F. Azevedo. 2010. Feeding habits of delphinids (Mammalia: Cetacea) from Rio de Janeiro State, Brazil. Journal of the Marine Biological Association of the United Kingdom 90(8):1509–1515. doi: 10.1017/s0025315409991639

Mills, L.R. and K.R. Rademacher. 1996. Atlantic spotted dolphins (*Stenella frontalis*) in the Gulf of Mexico. Gulf of Mexico Science 14(2):114–120. doi: 10.18785/goms.1402.08

Moreno, I.B., A.N. Zerbini, D. Danilewicz, S. de Oliveira Sa, P.C. Simões-Lopes, J. Lailson-Brito, and A.F. Azevedo. 2005. Distribution and habitat characteristics of dolphins of the genus *Stenella* (Cetacea: Delphinidae) in the Southwest Atlantic Ocean. Marine Ecology Progress Series 300:229–240. doi: 10.3354/meps300229

Mullin, K.D. and G.L. Fulling. 2003. Abundance of cetaceans in the southern U.S. North Atlantic Ocean during summer 1998. Fishery Bulletin 101:603–613.

Mullin, K.D. and G.L. Fulling. 2004. Abundance of cetaceans in the oceanic northern Gulf of Mexico, 1996–2001. Marine Mammal Science 20(4):787–807. doi: 10.1111/j.1748-7692.2004.tb01193.x

Mullin, K.D., W. Hoggard, and L.J. Hansen. 2004. Abundance and seasonal occurrence of cetaceans in outer continental shelf and slope waters of the North-central and northwestern Gulf of Mexico. Gulf of Mexico Science 22(1):62–73. doi: 10.18785/goms.2201.06

Palka, D. 2020. Cetacean abundance estimates in US northwestern Atlantic Ocean waters from summer 2016 line transect surveys conducted by the Northeast Fisheries Science Center. National Oceanic and Atmospheric Administration, National Marine Fisheries Service, Northeast Fisheries Science Center: Woods Hole, MA. Northeast Fisheries Science Center Reference Document 20-05. 60 p.

Palka, D., L. Aichinger Dias, E. Broughton, S. Chavez-Rosales, D. Cholewiak, G. Davis, A. DeAngelis, L. Garrison, H. Haas, J. Hatch, K. Hyde, M. Jech, E. Josephson, L. Mueller-Brennan, C. Orphanides, N. Pegg, C. Sasso, D. Sigourney, M. Soldevilla, and H. Walsh. 2021. Atlantic Marine Assessment Program for Protected Species: FY15 – FY19. US Department of the Interior, Bureau of Ocean Energy Management: Washington, DC. OCS Study BOEM 2021-051. 330 p.

Palka, D., L. Aichinger Dias, E. Broughton, S. Chavez-Rosales, D. Cholewiak, G. Davis, A. DeAngelis, L. Garrison, H. Haas, J. Hatch, K. Hyde, M. Jech, E. Josephson, L. Mueller-Brennan, C. Orphanides, N. Pegg, C. Sasso, D. Sigourney, M. Soldevilla, and H. Walsh. 2021. Supplement to final report BOEM 2021-051 Atlantic Marine Assessment Program for Protected Species: FY15 – FY19 Appendix I generalize additive density-habitat models and maps, by species. US Department of the Interior, Bureau of Ocean Energy Management: Washington, DC. OCS Study BOEM 2021-051. 330 p.

Payne, P.M., L.A. Selzer, and A.R. Knowlton. 1984. Distribution and density of cetaceans, marine turtles, and seabirds in the shelf waters of the northeastern United States, June 1980 - December 1983, based on shipboard observations. National Oceanic and Atmospheric Administration, National Marine Fisheries Service, Northeast Fisheries Science Center: Woods Hole, MA. Contract number NA-81-FA-C-00023. 246 p.

Perrin, W.F. 2001. *Stenella attenuata*. Mammalian Species 683:1–8. doi: 10.1644/1545-1410(2001)683<0001:SA>2.0.CO;2

Perrin, W.F., D.K. Caldwell, and M.C. Caldwell. 1994. Atlantic spotted dolphin *Stenella frontalis* (G. Cuvier, 1829). In: Ridgway, S.H. and R. Harrison (Eds.), Handbook of marine mammals. Volume 5: The first book of dolphins. Academic Press, London, UK. pp. 173–190.

Perrin, W.F., E.D. Mitchell, J.G. Mead, D.K. Caldwell, M.C. Caldwell, P.J.H. Bree, and W.H. Dawbin. 1987. Revision of the spotted dolphins, *Stenella* spp. Marine Mammal Science 3(2):99–170. doi: 10.1111/j.1748-7692.1987.tb00158.x

Rice, D.W. 1998. Marine mammals of the world, systematics and distribution. Special Publication No. 4. The Society for Marine Mammalogy, Lawrence, KS. 231 p.

Roberts, J.J., B.D. Best, L. Mannocci, E. Fujioka, P.N. Halpin, D.L. Palka, L.P. Garrison, K.D. Mullin, T.V.N. Cole, C.B. Khan, W.A. McLellan, D.A. Pabst, and G.G. Lockhart. 2016. Habitat-based cetacean density models for the US Atlantic and Gulf of Mexico. Scientific Reports 6:22615. doi: 10.1038/Srep22615

Roberts, J.J., L. Mannocci, and P.N. Halpin. 2016. Final project report: Marine species density data gap assessments and update for the AFTT Study Area, 2015-2016 (Base Year). Document version 1.0. Report prepared for Naval Facilities Engineering Command, Atlantic by the Duke University Marine Geospatial Ecology Lab: Durham, NC. 21 p.

Roberts, J.J., L. Mannocci, and P.N. Halpin. 2017. Final Project Report: Marine Species Density Data Gap Assessments and Update for the AFTT Study Area, 2016-2017 (Opt. Year 1). Document version 1.4. Report prepared for Naval Facilities Engineering Command, Atlantic by the Duke University Marine Geospatial Ecology Lab: Durham, NC. 76 p.

Roberts, J.J., L. Mannocci, R.S. Schick, and P.N. Halpin. 2018. Final Project Report: Marine Species Density Data Gap Assessments and Update for the AFTT Study Area, 2017-2018 (Opt. Year 2). Document version 1.2. Report prepared for Naval Facilities Engineering Command, Atlantic by the Duke University Marine Geospatial Ecology Lab: Durham, NC. 113 p.

Roden, C.L. and K.D. Mullin. 2000. Sightings of cetaceans in the northern Caribbean Sea and adjacent waters, winter 1995. Caribbean Journal of Science 36(3-4):280–288.

Taylor, B.L., S.J. Chivers, J. Larese, and W.F. Perrin. 2007. Generation length and percent mature estimates for IUCN assessments of cetaceans. National Oceanic and Atmospheric Administration, National Marine Fisheries Service, Southwest Fisheries Science Center: La Jolla, CA. Administrative Report LJ-07-01. 24 p.

Viricel, A. and P.E. Rosel. 2014. Hierarchical population structure and habitat differences in a highly mobile marine species: the Atlantic spotted dolphin. Molecular Ecology 23(20):5018–5035. doi: 10.1111/mec.12923

Waring, G., E. Josephson, K. Maze-Foley, and P. Rosel. 2014. U.S. Atlantic and Gulf of Mexico Marine Mammal Stock Assessments - 2013. National Oceanic and Atmospheric Administration, National Marine Fisheries Service, Northeast Fisheries Science Center: Woods Hole, MA. NOAA Technical Memorandum NMFS-NE-228. 464 p. doi: 10.7289/V51G0J70

Waring, G.T., E. Josephson, C.P. Fairfield, and K. Maze-Foley. 2006. U.S. Atlantic and Gulf of Mexico marine mammal stock assessments - 2005. National Oceanic and Atmospheric Administration, National Marine Fisheries Service, Northeast Fisheries Science Center: Woods Hole, MA. NOAA Technical Memorandum NMFS-NE-194. 346 p.

Atlantic white−sided dolphin − *Lagenorhynchus acutus*

Western North Atlantic Stock

Overall Vulnerability Rank = Low

Biological Sensitivity = Low

Climate Exposure = High

Data Quality = 89% of scores ≥ 2

	Lagenorhynchus acutus	Expert Scores	Data Quality	Expert Scores Plots (Portion by Category)
Sensitivity attributes	Prey/Diet Specificity	1.7	3.0	
	Habitat Specificity	2.1	3.0	
	Site Fidelity	1.7	1.7	
	Lifetime Reproductive Potential	1.9	2.7	
	Generation Length	1.7	1.7	
	Reproductive Plasticity	1.5	2.3	
	Migration	2.4	2.7	
	Home Range	1.8	3.0	
	Stock Abundance	1.2	3.0	
	Stock Abundance Trend	1.7	0.7	
	Cumulative Stressors	1.6	2.3	
	Sensitivity Score	Low		
Exposure variables	Sea Surface Temperature (Change in mean)	3.6	3.0	
	Sea Surface Temperature (Change in variability)	1.7	3.0	
	Air Temperature (Change in mean)	2.7	2.3	
	Air Temperature (Change in variability)	1.0	2.3	
	Precipitation (Change in mean)	1.1	2.3	
	Precipitation (Change in variability)	1.4	2.3	
	Sea Surface Salinity (Change in mean)	1.8	3.0	
	Sea Surface Salinity (Change in variability)	1.9	3.0	
	Ocean pH (Change in mean)	3.0	3.0	
	Ocean pH (Change in variability)	1.3	3.0	
	Sea ice coverage (Change in mean)	1.4	3.0	
	Sea ice coverage (Change in variability)	1.0	3.0	
	Dissolved oxygen (Change in mean)	3.3	3.0	
	Dissolved oxygen (Change in variability)	1.5	3.0	
	Circulation	2.1	2.3	
	Sea level rise	1.3	2.7	
	Exposure Score	High		
Overall Vulnerability Rank		Low		

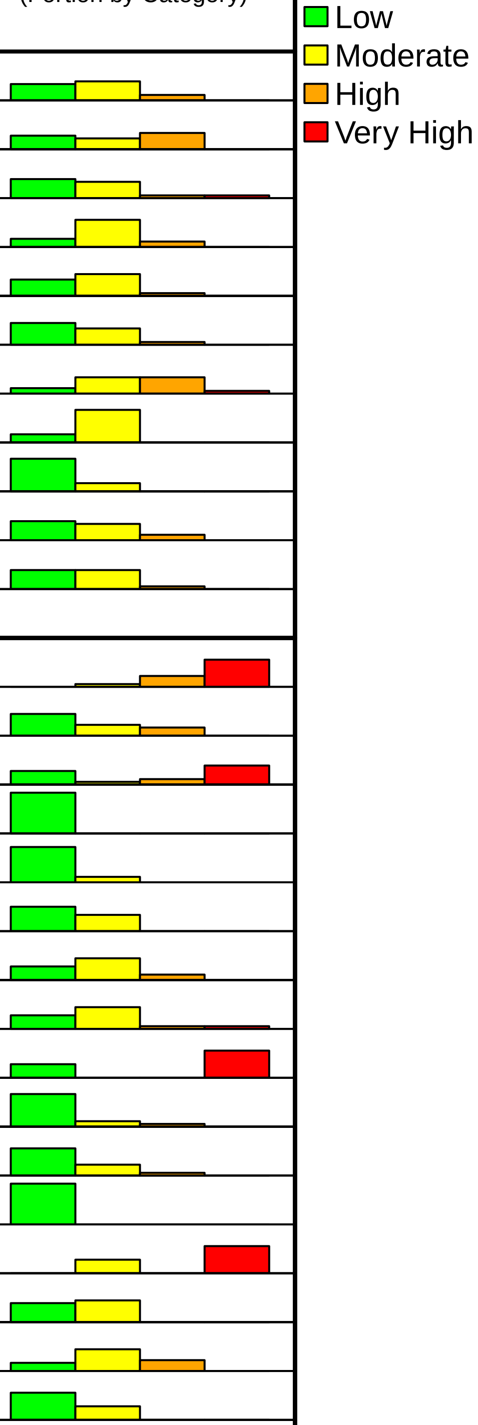

Atlantic white-sided dolphin (Western North Atlantic Stock)

Lagenorhynchus acutus

CVA Results Summary

Overall Climate Vulnerability Rank: Low (98% certainty from bootstrap analysis).

Climate Exposure: High. Three exposure factors scored 3.0 or greater: Sea Surface Temperature (Standard anomaly) (3.60), Dissolved oxygen (Standard anomaly) (3.33), and Ocean pH (Standard anomaly) (3.00).

Biological Sensitivity: Low. No sensitivity attributes scored greater than 2.5

Distributional Response: High

Abundance Response: Low

Phenology Response: Moderate

Data Quality: 89% of the data quality scores were 2 or greater. 73% of sensitivity attribute data quality scores were 2 or higher.

Background Information

Stock Description

The Atlantic white-sided dolphin western North Atlantic stock includes individuals that are found in the waters of the U.S. Exclusive Economic Zone in the western North Atlantic (Hayes et al. 2022). This stock may consist of three separate units in the Gulf of Maine, Gulf of St. Lawrence and Labrador Sea stocks (Palka et al. 1997).

Prey/Diet Specificity

Stomach-content analysis indicates white-sided dolphins in U.S. waters consume silver hake (*Merluccius bilinearis*), spoonarm octopus (*Bathypolypus bairdii*), haddock (*Melanogrammus aeglefinus*), Atlantic herring (*Clupea harengus*), gadid fishes (codfish and their relatives), sand lances, and several types of squid (Reeves et al. 1999; Craddock et al. 2009). Diet varies by season.

Habitat Specificity

White-sided dolphins are found in temperate and sub-polar waters of the North Atlantic, primarily in continental shelf waters to the 100-m depth contour (Hayes et al. 2022).

Site Fidelity

Information regarding Atlantic white-sided dolphin site fidelity was not found in the literature.

Lifetime Reproductive Potential

Taylor et al. (2007) reported Atlantic white-sided dolphin interbirth interval of 2.5 years, though some individuals may breed annually (Sergeant et al. 1980). Taylor et al. (2007) reported Atlantic white-sided dolphin age at first reproduction of 10 years and age at last reproduction of 27 and 32 (estimated) years based on information presented by Sergeant et al. (1980).

Generation Length

Taylor et al. (2007) reported generation length of 15.5 years (at r = 0.01) and 15.8 years (at r = 0.0) based on information presented by Sergeant et al. (1980).

Reproductive Plasticity

Calving occurs from May to early August, with a peak in June and July (Weinrich et al. 2001; Hayes et al. 2022).

Migration

Seasonal shifts in distribution have been suggested, with low numbers observed in the region between Georges Bank and the Gulf of Maine from January to May, high numbers from June through September, and intermediate densities from October through December (Payne and Heinemann 1990; Northridge et al. 1997).

Home Range

Information regarding Atlantic white-sided dolphin home range was not found in the literature.

Stock Abundance

Palka (2020) estimated the abundance of the western North Atlantic stock at 93,233 individuals (CV= 0.61), based on summer 2016 surveys from central Virginia to Labrador. Previously, an estimate based on June–August 2011 survey data from central Virginia to the lower Bay of Fundy suggested the population was 48,819 individuals (CV= 0.61; Hayes et al. 2022).

Stock Abundance Trend

A trend analysis has not been conducted for this stock.

Cumulative Stressors

Atlantic white-sided dolphins interact with fisheries in the western North Atlantic. During the period 2013–2017, annual bycatch in the bottom trawl fishery was estimated at 21.36 individuals (CV = 0.21) in the Northeast and 1.93 (CV = 0.94) in the Mid-Atlantic (Lyssikatos et al. 2020). Total annual estimated average fishery-related mortality or serious injury to this stock during 2013–2017 was 26 individuals (CV=0.2; Hayes et al. 2020). During 2013–2017, 123 Atlantic white-sided dolphins stranded on the U.S. and Canadian Atlantic coasts (Hayes et al. 2020). Human interaction was indicated in 5 records during this period, with one classified as a fishery interaction. Mass strandings are common for this species

(Hayes et al. 2020). Based on strandings in Massachusetts between 2000 and 2006, more than two thirds of white-sided dolphins strandings were of unknown cause and 21% were disease-related (Bogomolni et al. 2010). Strandings also occur along Canadian shorelines (Ledwell and Huntington 2010, 2011, 2012a, 2012b, 2013, 2014).

Density Models

Density model results for Atlantic white-sided dolphin in the western North Atlantic are presented by Roberts et al. (2016a, 2016b, 2017, 2018), Mannocci et al. (2017), Chavez-Rosales et al. (2019), and Palka et al. (2021a, 2021b).

Further Reading

Braulik, G. 2019. *Lagenorhynchus acutus*. The IUCN Red List of Threatened Species 2019: e.T11141A50361160. https://dx.doi.org/10.2305/IUCN.UK.2019-3.RLTS.T11141A50361160.en

Cipriano, F. 2018. Atlantic White-Sided Dolphin: *Lagenorhynchus acutus*. In: Würsig, B., J.G.M. Thewissen, and K.M. Kovacs (Eds.), Encyclopedia of Marine Mammals (Third Edition). Academic Press, London, UK. pp. 42–44. doi: 10.1016/B978-0-12-804327-1.00051-0

Department of the Navy. 2005. Marine Resource Assessment for the Northeast Operating Areas: Atlantic City, Narragansett Bay, and Boston. Naval Facilities Engineering Command, Atlantic. Norfolk, VA. Contract #N63470-02-D-9997, Task Order 0018. Prepared by Geo-Marine Inc., Newport News, VA. 382 p.

Hayes, S.A., E. Josephson, K. Maze-Foley, P.E. Rosel, and J. Wallace. 2022. U.S. Atlantic and Gulf of Mexico marine mammal stock assessments 2021. National Oceanic and Atmospheric Administration, National Marine Fisheries Service, Northeast Fisheries Science Center: Woods Hole, MA. NOAA Technical Memorandum NMFS-NE-288. 380 p. doi: 10.25923/6tt7-kc16

References

Bogomolni, A.L., K.R. Pugliares, S.M. Sharp, K. Patchett, C.T. Harry, J.M. LaRocque, K.M. Touhey, and M. Moore. 2010. Mortality trends of stranded marine mammals on Cape Cod and southeastern Massachusetts, USA, 2000 to 2006. Diseases of Aquatic Organisms 88(2):143–155. doi: 10.3354/dao02146

Chavez-Rosales, S., D.L. Palka, L.P. Garrison, and E.A. Josephson. 2019. Environmental predictors of habitat suitability and occurrence of cetaceans in the western North Atlantic Ocean. Scientific Reports 9(1):5833. doi: 10.1038/s41598-019-42288-6

Craddock, J.E., P.T. Polloni, B. Hayward, and F. Wenzel. 2009. Food habits of Atlantic white-sided dolphins (*Lagenorhynchus acutus*) off the coast of New England. Fishery Bulletin 107(3):384–394.

Hayes, S.A., E. Josephson, K. Maze-Foley, and P.E. Rosel. 2020. US Atlantic and Gulf of Mexico marine mammal stock assessments - 2019. National Oceanic and Atmospheric Administration, National Marine Fisheries Service, Northeast Fisheries Science Center: Woods Hole, MA. NOAA Technical Memorandum NMFS-NE-264. 479 p.

Hayes, S.A., E. Josephson, K. Maze-Foley, P.E. Rosel, and J. Wallace. 2022. U.S. Atlantic and Gulf of Mexico marine mammal stock assessments 2021. National Oceanic and Atmospheric Administration, National Marine Fisheries Service, Northeast Fisheries Science Center: Woods Hole, MA. NOAA Technical Memorandum NMFS-NE-288. 380 p. doi: 10.25923/6tt7-kc16

Ledwell, W. and J. Huntington. 2010. Whale, leatherback sea turtles, and basking sharks entrapped in fishing gear in Newfoundland and Labrador and a summary of the strandings, sightings and education work during 2009– 2010. A preliminary report to Fisheries and Oceans Canada: St. John's, Newfoundland, Canada. 20 p.

Ledwell, W. and J. Huntington. 2011. Whale, leatherback sea turtles, and basking sharks entrapped in fishing gear in Newfoundland and Labrador and a summary of the strandings, sightings and education work during 2010– 2011. A preliminary report to Fisheries and Oceans Canada: St. John's, Newfoundland, Canada. 25 p.

Ledwell, W. and J. Huntington. 2012. Incidental entanglements of cetacean and leatherback sea turtles in fishing gear reported during 2011-2012 and a summary of the Whale Release and Strandings Group activities. A preliminary report to Fisheries and Oceans Canada: St. John's, Newfoundland, Canada. 17 p.

Ledwell, W. and J. Huntington. 2012. Incidental entrapments in fishing gear and stranding reported to and responded to by the Whale Release and Strandings Group in Newfoundland and Labrador and a summary of the Whale Release and Strandings program during 2012. Report to Fisheries and Oceans Canada: St. John's, Newfoundland, Canada. 18 p.

Ledwell, W. and J. Huntington. 2013. Incidental entrapments in fishing gear and stranding reported to and responded to by the Whale Release and Strandings Group in Newfoundland and Labrador and a summary of the Whale Release and Strandings program during 2013. Report to Fisheries and Oceans Canada: St. John's, Newfoundland, Canada. 19 p.

Ledwell, W. and J. Huntington. 2014. Incidental entrapments and entanglements of cetaceans and leatherback sea turtles, strandings, ice entrapments reported to the Whale Release and Strandings Group in Newfoundland and Labrador and a summary of the Whale Release and Strandings program during 2014. Report to the Department of Fisheries and Oceans Canada: St. John's, Newfoundland, Canada. 23 p.

Lyssikatos, M.C., S. Chavez-Rosales, and J.M. Hatch. 2020. Estimates of cetacean and pinniped bycatch in Northeast and Mid-Atlantic bottom trawl fisheries, 2013-2017. National Oceanic and Atmospheric Administration, National Marine Fisheries Service, Northeast Fisheries Science Center: Woods Hole, MA. Northeast Fisheries Science Center Reference Document 20-04. 11 p. doi: 10.25923/5we2-g460

Mannocci, L., J.J. Roberts, D.L. Miller, and P.N. Halpin. 2017. Extrapolating cetacean densities to quantitatively assess human impacts on populations in the high seas. Conservation Biology 31(3):601–614. doi: 10.1111/cobi.12856

Northridge, S., M. Tasker, A. Webb, K. Camphuysen, and M. Leopold. 1997. White-beaked *Lagenorhynchus albirostris* and Atlantic white-sided dolphin *L. acutus* distributions in northwest European and U.S. North Atlantic waters. Reports of the International Whaling Commission 47:797–805.

Palka, D. 2020. Cetacean abundance estimates in US northwestern Atlantic Ocean waters from summer 2016 line transect surveys conducted by the Northeast Fisheries Science Center. National Oceanic and Atmospheric Administration, National Marine Fisheries Service, Northeast Fisheries Science Center: Woods Hole, MA. Northeast Fisheries Science Center Reference Document 20-05. 60 p.

Palka, D., L. Aichinger Dias, E. Broughton, S. Chavez-Rosales, D. Cholewiak, G. Davis, A. DeAngelis, L. Garrison, H. Haas, J. Hatch, K. Hyde, M. Jech, E. Josephson, L. Mueller-Brennan, C. Orphanides, N. Pegg, C. Sasso, D. Sigourney, M. Soldevilla, and H. Walsh. 2021. Atlantic Marine Assessment Program for Protected Species: FY15 – FY19. US Department of the Interior, Bureau of Ocean Energy Management: Washington, DC. OCS Study BOEM 2021-051. 330 p.

Palka, D., L. Aichinger Dias, E. Broughton, S. Chavez-Rosales, D. Cholewiak, G. Davis, A. DeAngelis, L. Garrison, H. Haas, J. Hatch, K. Hyde, M. Jech, E. Josephson, L. Mueller-Brennan, C. Orphanides, N. Pegg, C. Sasso, D. Sigourney, M. Soldevilla, and H. Walsh. 2021. Supplement to final report BOEM 2021-051 Atlantic Marine Assessment Program for Protected Species: FY15 – FY19 Appendix I generalize additive density-habitat models and maps, by species. US Department of the Interior, Bureau of Ocean Energy Management: Washington, DC. OCS Study BOEM 2021-051. 330 p.

Palka, D., A. Read, and C. Potter. 1997. Summary of knowledge of white-sided dolphins (*Lagenorhyncus acutus*) from US and Canadian Atlantic waters. Reports of the International Whaling Commission 47:729–734.

Payne, M. and D.W. Heinemann. 1990. A distributional assessment of cetaceans in the shelf and shelf edge waters of the northeastern United States based on aerial and shipboard surveys, 1978–1988. Report to NMFS. 108 p.

Reeves, R.R., C. Smeenk, R.L. Brownell, and C.C. Kinze. 1999. Atlantic white-sided dolphin *Lagenorhynchus acutus* (Gray, 1828). In: Ridgway, S.H. and R. Harrison (Eds.), Handbook of Marine Mammals. Volume 6: The Second Book of Dolphins and the Porpoises. Academic Press, London, UK. pp. 31–56.

Roberts, J.J., B.D. Best, L. Mannocci, E. Fujioka, P.N. Halpin, D.L. Palka, L.P. Garrison, K.D. Mullin, T.V.N. Cole, C.B. Khan, W.A. McLellan, D.A. Pabst, and G.G. Lockhart. 2016. Habitat-based cetacean density models for the US Atlantic and Gulf of Mexico. Scientific Reports 6:22615. doi: 10.1038/Srep22615

Roberts, J.J., L. Mannocci, and P.N. Halpin. 2016. Final project report: Marine species density data gap assessments and update for the AFTT Study Area, 2015-2016 (Base Year). Document version 1.0. Report prepared for Naval Facilities Engineering Command, Atlantic by the Duke University Marine Geospatial Ecology Lab: Durham, NC. 21 p.

Roberts, J.J., L. Mannocci, and P.N. Halpin. 2017. Final Project Report: Marine Species Density Data Gap Assessments and Update for the AFTT Study Area, 2016-2017 (Opt. Year 1). Document version 1.4. Report prepared for Naval Facilities Engineering Command, Atlantic by the Duke University Marine Geospatial Ecology Lab: Durham, NC. 76 p.

Roberts, J.J., L. Mannocci, R.S. Schick, and P.N. Halpin. 2018. Final Project Report: Marine Species Density Data Gap Assessments and Update for the AFTT Study Area, 2017-2018 (Opt. Year 2). Document version 1.2. Report prepared for Naval Facilities Engineering Command, Atlantic by the Duke University Marine Geospatial Ecology Lab: Durham, NC. 113 p.

Sergeant, D.E., D.J. St. Aubin, and J.R. Geraci. 1980. Life history and northwest Atlantic status of the Atlantic white-sided dolphin, *Lagenorhynchus acutus*. Cetology 37:1–12.

Taylor, B.L., S.J. Chivers, J. Larese, and W.F. Perrin. 2007. Generation length and percent mature estimates for IUCN assessments of cetaceans. National Oceanic and Atmospheric Administration, National Marine Fisheries Service, Southwest Fisheries Science Center: La Jolla, CA. Administrative Report LJ-07-01. 24 p.

Weinrich, M.T., C.R. Belt, and D. Morin. 2001. Behavior and ecology of the Atlantic white-sided dolphin (*Lagenorhynchus acutus*) in coastal New England waters. Marine Mammal Science 17(2):231–248. doi: 10.1111/j.1748-7692.2001.tb01268.x

Blainville's beaked whale − *Mesoplodon densirostris*

Western North Atlantic Stock

Overall Vulnerability Rank = Moderate

Biological Sensitivity = Moderate

Climate Exposure = High

Data Quality = 37% of scores ≥ 2

	Mesoplodon densirostris	Expert Scores	Data Quality	Expert Scores Plots (Portion by Category)
Sensitivity attributes	Prey/Diet Specificity	2.4	1.0	
	Habitat Specificity	2.1	1.7	
	Site Fidelity	3.1	1.3	
	Lifetime Reproductive Potential	2.7	0.7	
	Generation Length	2.3	1.0	
	Reproductive Plasticity	1.7	0.7	
	Migration	2.9	2.0	
	Home Range	2.7	2.0	
	Stock Abundance	2.3	1.0	
	Stock Abundance Trend	2.5	0.0	
	Cumulative Stressors	2.4	1.7	
	Sensitivity Score	Moderate		
Exposure variables	Sea Surface Temperature (Change in mean)	3.5	2.3	
	Sea Surface Temperature (Change in variability)	1.5	2.3	
	Air Temperature (Change in mean)	3.1	1.7	
	Air Temperature (Change in variability)	1.1	1.7	
	Precipitation (Change in mean)	1.2	2.0	
	Precipitation (Change in variability)	1.3	1.7	
	Sea Surface Salinity (Change in mean)	3.4	1.7	
	Sea Surface Salinity (Change in variability)	1.7	1.7	
	Ocean pH (Change in mean)	3.3	1.7	
	Ocean pH (Change in variability)	1.4	1.7	
	Sea ice coverage (Change in mean)	1.1	2.3	
	Sea ice coverage (Change in variability)	1.1	2.3	
	Dissolved oxygen (Change in mean)	3.0	2.0	
	Dissolved oxygen (Change in variability)	1.1	2.0	
	Circulation	2.2	1.3	
	Sea level rise	2.1	2.0	
	Exposure Score	High		
Overall Vulnerability Rank		Moderate		

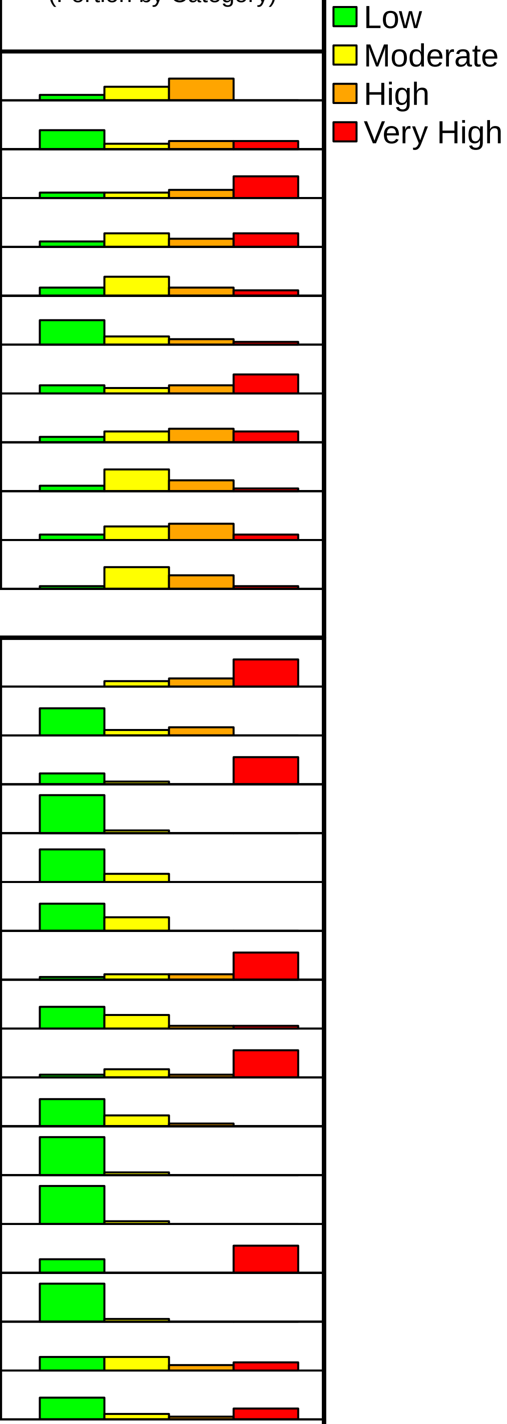

Blainville's beaked whale (Western North Atlantic Stock)

Mesoplodon densirostris

CVA Results Summary

Overall Climate Vulnerability Rank: Moderate (41% certainty from bootstrap analysis).

Climate Exposure: High. Five exposure factors scored 3.0 or greater: Sea Surface Temperature (Standard anomaly) (3.53), Sea Surface Salinity (Standard anomaly) (3.40), Ocean pH (Standard anomaly) (3.33), Air Temperature (Standard anomaly) (3.07), and Dissolved oxygen (Standard anomaly) (3.00).

Biological Sensitivity: Moderate. Five sensitivity attributes scored greater than 2.5: Site Fidelity (3.13), Migration (2.93), Home Range (2.73), Lifetime Reproductive Potential (2.73), and Species Abundance Trend (2.53).

Distributional Response: Moderate

Abundance Response: Moderate

Phenology Response: Moderate

Data Quality: 37% of the data quality scores were 2 or greater. 18% of sensitivity attribute data quality scores were 2 or higher.

Background Information

Stock Description

The Blainville's beaked whale western North Atlantic stock includes individuals that are found in the waters of the U.S. Exclusive Economic Zone in the western North Atlantic (Hayes et al. 2020).

Prey/Diet Specificity

Stomach content analysis of stranded Blainville's beaked whales from multiple geographic locations suggests that Blainville's beaked whale diet consists mainly of cephalopods and fish (Ross 1979; Aguilar et al. 1982; Santos et al. 2007).

Habitat Specificity

Little is known about beaked whale habitat preferences, but Blainville's beaked whales occur in warmer southern waters (MacLeod 2000).

Beaked whale abundance is associated with the Gulf Stream and warm-core rings (Waring et al. 1992). In summer, the continental shelf break off the northeastern U.S. is primary habitat (Waring et al. 2001). Beaked whales south of Georges Bank were found in waters with a mean SST of 20.7° to 24.9°C and a bottom depth of 500 to 2,000 m (Waring et al. 2003). In the Gulf of Mexico, beaked whales are seen in waters with a bottom depth ranging from 420 to 3,487m (Ward et al. 2005). Blainville's beaked whales in the northern Bahamas are found along shelf waters of canyon walls and in deeper offshore waters,

with most time spent along these walls where bottom depths are <800 m (Claridge 2003; MacLeod et al. 2004; MacLeod and Zuur 2005; Claridge 2006).

World-wide, beaked whales normally inhabit continental slope and deep oceanic waters (>200 m) (Waring et al. 2001; Cañadas et al. 2002; Pitman 2002; MacLeod et al. 2004; Ferguson et al. 2006; MacLeod and Mitchell 2006; Ritter and Brederlau 1999; Gannier 2000; MacLeod et al. 2004; Ferguson 2005; MacLeod and Zuur 2005; Claridge 2006) and only occasionally waters over the continental shelf (Pitman 2002). In many locations, occurrence patterns have been linked to physical features such as the continental slope, canyons, escarpments, and oceanic islands (Baird et al. 2004; MacLeod et al. 2004; MacLeod and D'Amico 2006).

Site Fidelity

Information regarding Blainville's beaked whale site fidelity was not found in the literature.

Lifetime Reproductive Potential

Taylor et al. (2007) estimated Blainville's beaked whale age at last reproduction of 40 years.

Generation Length

Information regarding Blainville's beaked whale generation length was not found in the literature.

Reproductive Plasticity

Information regarding Blainville's beaked whale mating habitat, location, and season was not found in the literature.

Migration

Information regarding Blainville's beaked whale migration was not found in the literature.

Home Range

Information regarding Blainville's beaked whale home range was not found in the literature.

Stock Abundance

The best abundance estimate for undifferentiated *Mesoplodon spp.* beaked whales in the western North Atlantic is 10,107 individuals (CV=0.27), based on 2016 surveys (Garrison 2020; Palka 2020). Previously, undifferentiated *Mesoplodon spp.* beaked whales in the western North Atlantic were estimated at 7,092 individuals (CV=0.54), based on 2011 surveys (Hayes et al. 2020).

Stock Abundance Trend

A trend analysis has not been conducted for this stock.

Cumulative Stressors

During the period 2013–2017, four Blainville's beaked whales were reported stranded along the U.S. Atlantic coast between Florida and Massachusetts (Hayes et al. 2020). Beaked whale strandings throughout their worldwide range have been associated with naval activities (Frantzis 1998; D'Amico et al. 2009; Filadelfo et al. 2009; Hayes et al. 2020).

Distribution and Sightings

Density model results for Blainville's beaked whales in the western North Atlantic are presented by Roberts et al. (2016a, 2016b, 2017) and Mannocci et al. (2017). Palka et al. (2021a, 2021b) present density model results for unidentified beaked whales.

Further Reading

Department of the Navy. 2008. Marine Resources Assessment Update for the Charleston/Jacksonville Operating Area. Naval Facilities Engineering Command, Atlantic, Norfolk, VA. Contract #N62470-D-9997, CTO 0056. Prepared by Geo-Marine, Inc., Hampton, VA. 663 p.

Hayes, S.A., E. Josephson, K. Maze-Foley, and P.E. Rosel. 2020. US Atlantic and Gulf of Mexico Marine Mammal Stock Assessments - 2019. Northeast Fisheries Science Center: Woods Hole, MA. NOAA technical memorandum NMFS-NE-264. 479 p.

MacLeod, C.D. 2018. Beaked Whales, Overview. In: Würsig, B., J.G.M. Thewissen, and K.M. Kovacs (Eds.), Encyclopedia of Marine Mammals (Third Edition). Academic Press, London, UK. pp. 80–83. doi: 10.1016/B978-0-12-804327-1.00062-5

Taylor, B.L., R. Baird, J. Barlow, S.M. Dawson, J. Ford, J.G. Mead, G. Notarbartolo di Sciara, P. Wade, and R.L. Pitman. 2008. *Mesoplodon densirostris*. The IUCN Red List of Threatened Species 2008: e.T13244A3426474. https://dx.doi.org/10.2305/IUCN.UK.2008.RLTS.T13244A3426474.en.

References

Aguilar, A., L. Jover, and J. Nadal. 1982. A note on the organochlorine contamination in a Blainville's beaked whale, *Mesoplodon densirostris* (de Blainvillle, 1817) from the Mediterranean Sea. Publicaciones Del Departmento De Zoologia, Universitat de Barcelona 7:85–90.

Baird, R.W., D.J. McSweeney, A.D. Ligon, and D.L. Webster. 2004. Tagging feasibility and diving of Cuvier's beaked whales (*Ziphius cavirostris*) and Blainville's beaked whales (*Mesoplodon densirostris*) in Hawai'i. National Oceanic and Atmospheric Administration, National Marine Fisheries Service, Southwest Fisheries Science Center: La Jolla, CA. Order No. AB133F-03-SE-0986. 15 p.

Cañadas, A., R. Sagarminaga, and S. García-Tiscar. 2002. Cetacean distribution related with depth and slope in the Mediterranean waters off southern Spain. Deep Sea Research Part I: Oceanographic Research Papers 49(11):2053–2073. doi: 10.1016/s0967-0637(02)00123-1

Claridge, D. 2003. Examining distribution and habitat preferences of deep-diving cetaceans, including beaked whales, in Northwest Providence Channel, the Bahamas using geographic information system (GIS) mapping techniques. Abstracts, Fifteenth Biennial Conference on the Biology of Marine Mammals14-19 December 2003. Greensboro, North Carolina. p. 33.

Claridge, D.E. 2006. Fine-scale distribution and habitat selection of beaked whales. Aberdeen University. Dissertation. 119 p.

D'Amico, A., R.C. Gisiner, D.R. Ketten, J.A. Hammock, C. Johnson, P.L. Tyack, and J. Mead. 2009. Beaked whale strandings and naval exercises. Aquatic Mammals 35(4):452–472. doi: 10.1578/am.35.4.2009.452

Ferguson, M.C. 2005. Cetacean population density in the eastern Pacific Ocean: Analyzing patterns with predictive spatial models. University of California, San Diego. 203 p.

Ferguson, M.C., J. Barlow, S.B. Reilly, and T. Gerrodette. 2006. Predicting Cuvier's (*Ziphius cavirostris*) and *Mesoplodon* beaked whale population density from habitat characteristics in the eastern tropical Pacific Ocean. Journal of Cetacean Research and Management 7(3):287–299.

Filadelfo, R., J. Mintz, E. Michlovich, A. D'Amico, P.L. Tyack, and D.R. Ketten. 2009. Correlating military sonar use with beaked whale mass strandings: What do the historical data show? Aquatic Mammals 35(4):435–444. doi: 10.1578/am.35.4.2009.435

Frantzis, A. 1998. Does acoustic testing strand whales? Nature 392(6671):29. doi: 10.1038/32068

Gannier, A. 2000. Distribution of cetaceans off the Society Islands (French Polynesia) as obtained from dedicated surveys. Aquatic Mammals 26(2):111–126.

Garrison, L.P. 2020. Abundance of marine mammals in waters of the U.S. southeastern Atlantic during summer 2016. National Oceanic and Atmospheric Administration, National Marine Fisheries Service, Southeast Fisheries Science Center, Protected Resources and Biodiversity Division: Miami, FL. PRBD-2020-04. 17 p.

Hayes, S.A., E. Josephson, K. Maze-Foley, and P.E. Rosel. 2020. US Atlantic and Gulf of Mexico marine mammal stock assessments - 2019. National Oceanic and Atmospheric Administration, National Marine Fisheries Service, Northeast Fisheries Science Center: Woods Hole, MA. NOAA Technical Memorandum NMFS-NE-264. 479 p.

Macleod, C.D. 2000. Review of the distribution of *Mesoplodon* species (order Cetacea, family Ziphiidae) in the North Atlantic. Mammal Review 30(1):1–8. doi: 10.1046/j.1365-2907.2000.00057.x

MacLeod, C.D. and A. D'Amico. 2006. A review of beaked whale behaviour and ecology in relation to assessing and mitigating impacts of anthropogenic noise. Journal of Cetacean Research and Management 7(3):211–221.

MacLeod, C.D., N. Hauser, and H. Peckham. 2004. Diversity, relative density and structure of the cetacean community in summer months east of Great Abaco, Bahamas. Journal of the Marine Biological Association of the United Kingdom 84(2):469–474. doi: 10.1017/S0025315404009476h

MacLeod, C.D. and G. Mitchell. 2006. Key areas for beaked whales worldwide. Journal of Cetacean Research and Management 7(3):309–322.

MacLeod, C.D. and A.F. Zuur. 2005. Habitat utilization by Blainville’s beaked whales off Great Abaco, northern Bahamas, in relation to seabed topography. Marine Biology 147(1):1–11. doi: 10.1007/s00227-004-1546-9

Mannocci, L., J.J. Roberts, D.L. Miller, and P.N. Halpin. 2017. Extrapolating cetacean densities to quantitatively assess human impacts on populations in the high seas. Conservation Biology 31(3):601–614. doi: 10.1111/cobi.12856

Palka, D. 2020. Cetacean abundance estimates in US northwestern Atlantic Ocean waters from summer 2016 line transect surveys conducted by the Northeast Fisheries Science Center. National Oceanic and Atmospheric Administration, National Marine Fisheries Service, Northeast Fisheries Science Center: Woods Hole, MA. Northeast Fisheries Science Center Reference Document 20-05. 60 p.

Palka, D., L. Aichinger Dias, E. Broughton, S. Chavez-Rosales, D. Cholewiak, G. Davis, A. DeAngelis, L. Garrison, H. Haas, J. Hatch, K. Hyde, M. Jech, E. Josephson, L. Mueller-Brennan, C. Orphanides, N. Pegg, C. Sasso, D. Sigourney, M. Soldevilla, and H. Walsh. 2021. Atlantic Marine Assessment

Program for Protected Species: FY15 – FY19. US Department of the Interior, Bureau of Ocean Energy Management: Washington, DC. OCS Study BOEM 2021-051. 330 p.

Palka, D., L. Aichinger Dias, E. Broughton, S. Chavez-Rosales, D. Cholewiak, G. Davis, A. DeAngelis, L. Garrison, H. Haas, J. Hatch, K. Hyde, M. Jech, E. Josephson, L. Mueller-Brennan, C. Orphanides, N. Pegg, C. Sasso, D. Sigourney, M. Soldevilla, and H. Walsh. 2021. Supplement to final report BOEM 2021-051 Atlantic Marine Assessment Program for Protected Species: FY15 – FY19 Appendix I generalize additive density-habitat models and maps, by species. US Department of the Interior, Bureau of Ocean Energy Management: Washington, DC. OCS Study BOEM 2021-051. 330 p.

Pitman, R.L. 2002. Mesoplodont whales *Mesoplodon* spp. In: Perrin, W.F., B. Würsig, and J.G.M. Thewissen (Eds.), Encyclopedia of Marine Mammals. Academic Press, San Diego, CA. pp. 738–742.

Ritter, F. and B. Brederlau. 1999. Behavioural observations of dense beaked whales (*Mesoplodon densirostris*) off La Gomera, Canary Islands (1995-1997). Aquatic Mammals 25(2):55–61.

Roberts, J.J., B.D. Best, L. Mannocci, E. Fujioka, P.N. Halpin, D.L. Palka, L.P. Garrison, K.D. Mullin, T.V.N. Cole, C.B. Khan, W.A. McLellan, D.A. Pabst, and G.G. Lockhart. 2016. Habitat-based cetacean density models for the US Atlantic and Gulf of Mexico. Scientific Reports 6:22615. doi: 10.1038/Srep22615

Roberts, J.J., L. Mannocci, and P.N. Halpin. 2016. Final project report: Marine species density data gap assessments and update for the AFTT Study Area, 2015-2016 (Base Year). Document version 1.0. Report prepared for Naval Facilities Engineering Command, Atlantic by the Duke University Marine Geospatial Ecology Lab: Durham, NC. 21 p.

Roberts, J.J., L. Mannocci, and P.N. Halpin. 2017. Final Project Report: Marine Species Density Data Gap Assessments and Update for the AFTT Study Area, 2016-2017 (Opt. Year 1). Document version 1.4. Report prepared for Naval Facilities Engineering Command, Atlantic by the Duke University Marine Geospatial Ecology Lab: Durham, NC. 76 p.

Ross, G.J.B. 1979. The smaller cetaceans of the south east coast of southern Africa. University of Port Elizabeth. Thesis. 415 p.

Santos, M.B., V. Martin, M. Arbelo, A. Fernandez, and G.J. Pierce. 2007. Insights into the diet of beaked whales from the atypical mass stranding in the Canary Islands in September 2002. Journal of the Marine Biological Association of the United Kingdom 87(1):243–251. doi: 10.1017/S0025315407054380

Taylor, B.L., S.J. Chivers, J. Larese, and W.F. Perrin. 2007. Generation length and percent mature estimates for IUCN assessments of cetaceans. National Oceanic and Atmospheric Administration, National Marine Fisheries Service, Southwest Fisheries Science Center: La Jolla, CA. Administrative Report LJ-07-01. 24 p.

Ward, J.A., G.H. Mitchell, A.M. Farak, and E.P. Keane. 2005. Beaked whale habitat characterization and prediction. NUWC-NPT Technical Report 11,548. Newport, RI.

Waring, G., D. Belden, M. Vecchione, and R. Gibbons. 2003. Mid-water prey in beaked whale and sperm whale deep-water habitat south of Georges Bank. Abstracts, Fifteenth Biennial Conference on the Biology of Marine Mammals. 14-19 December 2003. Greensboro, North Carolina. p. 172.

Waring, G.T., C.P. Fairfield, C.M. Ruhsam, and M. Sano. 1992. Cetaceans associated with Gulf Stream Features off the Northeastern USA Shelf. International Council for the Exploration of the Sea: Copenhagen, Denmark. CM 1992/N:12. 28 p.

Waring, G.T., T. Hamazaki, D. Sheehan, G. Wood, and S. Baker. 2001. Characterization of beaked whale (Ziphiidae) and perm whale (*Physeter macrocephalus*) summer habitat in shelf-edge and deeper waters off the Northeast U. S. Marine Mammal Science 17(4):703–717. doi: 10.1111/j.1748-7692.2001.tb01294.x

Blue whale − *Balaenoptera musculus*

Western North Atlantic Stock

Overall Vulnerability Rank = High

Biological Sensitivity = High

Climate Exposure = High

Data Quality = 81% of scores ≥ 2

	Balaenoptera musculus	Expert Scores	Data Quality	Expert Scores Plots (Portion by Category)
Sensitivity attributes	Prey/Diet Specificity	3.7	3.0	
	Habitat Specificity	1.7	2.7	
	Site Fidelity	2.6	1.8	
	Lifetime Reproductive Potential	1.3	1.7	
	Generation Length	3.1	1.7	
	Reproductive Plasticity	2.8	1.7	
	Migration	1.4	2.0	
	Home Range	1.5	2.5	
	Stock Abundance	3.0	2.0	
	Stock Abundance Trend	1.7	1.7	
	Cumulative Stressors	2.3	2.3	
	Sensitivity Score	High		
Exposure variables	Sea Surface Temperature (Change in mean)	3.3	2.5	
	Sea Surface Temperature (Change in variability)	1.1	2.5	
	Air Temperature (Change in mean)	3.4	2.5	
	Air Temperature (Change in variability)	1.0	2.5	
	Precipitation (Change in mean)	1.3	2.5	
	Precipitation (Change in variability)	1.5	2.5	
	Sea Surface Salinity (Change in mean)	1.8	2.5	
	Sea Surface Salinity (Change in variability)	1.5	2.5	
	Ocean pH (Change in mean)	4.0	2.5	
	Ocean pH (Change in variability)	1.3	2.5	
	Sea ice coverage (Change in mean)	1.4	2.5	
	Sea ice coverage (Change in variability)	1.1	2.5	
	Dissolved oxygen (Change in mean)	4.0	2.5	
	Dissolved oxygen (Change in variability)	1.3	2.5	
	Circulation	2.3	2.2	
	Sea level rise	1.3	2.5	
	Exposure Score	High		
Overall Vulnerability Rank		High		

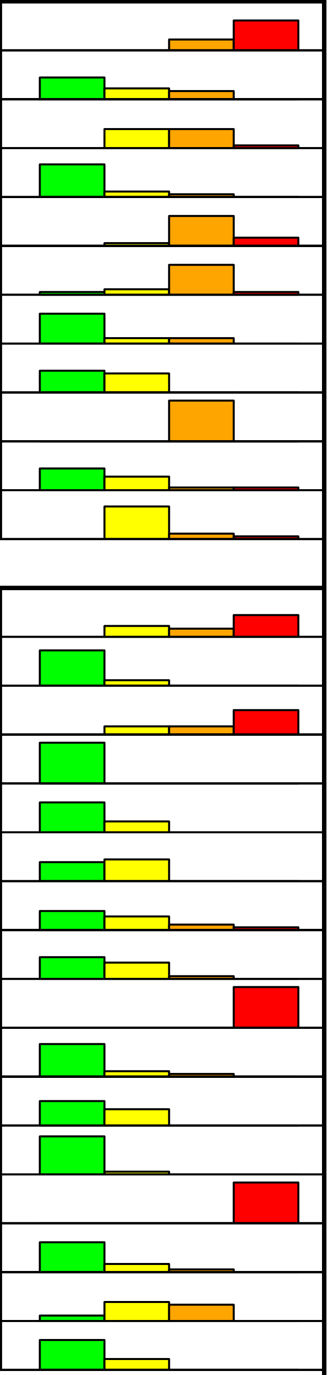

Low
Moderate
High
Very High

Blue whale (Western North Atlantic Stock)

Balaenoptera musculus

CVA Results Summary

Overall Climate Vulnerability Rank: High (57% certainty from bootstrap analysis).

Climate Exposure: High. Four exposure factors scored greater than 3.0: Dissolved oxygen (Standard anomaly) (4.00), Ocean pH (Standard anomaly) (4.00), Air Temperature (Standard anomaly) (3.40), and Sea Surface Temperature (Standard anomaly) (3.27).

Biological Sensitivity: High. Three sensitivity attributes scored greater than 3.0: Prey/Diet Specificity (3.73), Generation Time (3.13), and Species Abundance (3.00).

Distributional Response: Very High

Abundance Response: High

Phenology Response: High

Data Quality: 81 % of the data quality scores were 2 or greater 55 % of sensitivity attribute data quality scores were 2 or higher.

Background Information

Stock Description

The blue whale western North Atlantic stock includes individuals that are found in waters of the U.S. Exclusive Economic Zone in the western North Atlantic (Hayes et al. 2020). Photo-identification studies suggest that blue whales from the Gulf of St. Lawrence, Newfoundland, Nova Scotia, New England, and Greenland belong to the same stock, which is separate from blue whales sighted in the eastern North Atlantic (CETAP 1982; Wenzel et al. 1988; Sears and Calambokidis 2002; Sears and Larsen 2002; Hayes et al. 2020).

Prey/Diet Specificity

Blue whales in the North Atlantic consume almost exclusively krill (Hjort and Ruud 1929; Jonsgård 1955; Sergeant 1966; Sears et al. 1987; Christensen et al. 1992b). Blue whales may incidentally consume other prey species, such as fish and copepods (Kawamura 1980).

Habitat Specificity

Blue whales in the Atlantic are primarily found in deeper, offshore waters and are rare in shallower, shelf waters (Yochem and Leatherwood 1985; Wenzel et al. 1988). However, they associate with nearshore regions with strong tidal and current mixing in the Gulf of St. Lawrence (Sears et al. 1990). Blue whales forage along edges of continental shelves and upwelling regions (Reilly and Thayer 1990; Schoenherr 1991). In the Gulf of St. Lawrence, blue whales were found in association with high salinity, cold surface temperature waters in areas with slow currents (Doniol-Valcroze 2008).

Site Fidelity

Blue whales travel frequently and do not tend to remain in the same area for more than a week or two, although nearly half of the Gulf of St. Lawrence population was resighted in subsequent years (Sears et al. 1990).

Lifetime Reproductive Potential

Taylor et al. (2007) reported a blue whale interbirth interval of 2.5 years while Yochem and Leatherwood (1985) reported an interbirth interval of 2 years.

Female blue whales reach sexual maturity around 11 years of age (range 5 to 15 years; Mizroch et al. 1984; Yochem and Leatherwood 1985; Taylor et al. 2007). Age at last reproduction is estimated to be 65 years (Taylor et al. 2007).

Generation Length

Taylor et al. (2007) reported generation length of 21.7 at r=0.05 and 30.8 at r=0.0 based on values reported by Lockyer (1984) and Mizroch et al. (1984).

Reproductive Plasticity

Calving occurs primarily during winter months (Yochem and Leatherwood 1985).

Migration

Blue whales occur in the Gulf of St. Lawrence in spring, summer, and fall and off southern Newfoundland in winter (Sears et al. 1990; Hayes et al. 2020).

Home Range

Blue whales have been detected and tracked acoustically in much of the North Atlantic, indicating the potential for long-distance movements (Clark 1995).

Stock Abundance

The abundance of blue whales in the Northwest Atlantic has not been established (Sears et al. 1987; Hammond et al. 1990; Sears et al. 1990; Sears and Calambokidis 2002; Fisheries and Oceans Canada 2009). A total of 402 blue whales were photo-identified in the St. Lawrence estuary and northwestern Gulf of St. Lawrence from 1980 to the summer of 2008, with 20 to 105 whales identified in this region each year (Ramp and Sears 2013). Mitchell (1974) estimated that the blue whale population in the western North Atlantic may number only in the low hundreds. In waters around Iceland, Pike et al. (2019) estimated 3000 blue whales (95% CI = 1377–6534) in 2015, though whales in those waters are believed to be from a different population than those in the western North Atlantic (Wenzel et al. 1988; Sears and Calambokidis 2002; Sears and Larsen 2002; Hayes et al. 2020).

Stock Abundance Trend

A trend analysis has not been completed for this population. Elsewhere in the North Atlantic, the population off Iceland increased at 4.9% per year from 1969 to 1988 (Sigurjonsson and Gunnlaugsson 1990). Population estimates from the 1970s placed the blue whale population in the western North Atlantic in the low hundreds (Mitchell 1974). Pike et al. (2019) noted the increase in population in Icelandic waters continued through 2015.

Cumulative Stressors

Threats for North Atlantic blue whales include ship strikes, pollution, and entanglement in fishing gear although there are no recent confirmed records of mortality or serious injury to blue whales in the U.S. Atlantic EEZ (Hayes et al. 2020). Ice-related strandings and entrapments are known to occur off the coast of Newfoundland (Sears and Calambokidis 2002). Contaminants (e.g., PCBs, organochlorines) and increasing anthropogenic noise in the oceans also represent threats to blue whales in the western North Atlantic (Metcalfe et al. 2004; Sears and Perrin 2018).

Distribution and Sightings

Density model results for blue whales in the western North Atlantic are presented by Roberts et al. (2015, 2016). Palka et al. (2021a, 2021b) present blue whale sightings along survey track lines.

Further Reading

Cooke, J.G. 2018. *Balaenoptera musculus* (errata version published in 2019). The IUCN Red List of Threatened Species 2018: e.T2477A156923585. https://dx.doi.org/10.2305/IUCN.UK.2018-2.RLTS.T2477A156923585.en

Department of the Navy. 2005. Marine Resource Assessment for the Northeast Operating Areas: Atlantic City, Narragansett Bay, and Boston. Naval Facilities Engineering Command, Atlantic. Norfolk, VA. Contract #N63470-02-D-9997, Task Order 0018. Prepared by Geo-Marine Inc., Newport News, VA. 382 p.

Hayes, S.A., E. Josephson, K. Maze-Foley, and P.E. Rosel. 2020. US Atlantic and Gulf of Mexico Marine Mammal Stock Assessments - 2019. Northeast Fisheries Science Center: Woods Hole, MA. NOAA technical memorandum NMFS-NE-264. 479 p.

National Marine Fisheries Service (NMFS). 2020. Recovery Plan for the Blue Whale (*Balaenoptera musculus*): first revision. Silver Spring, MD. 118 p.

National Marine Fisheries Service (NMFS). 2020. Blue Whale (*Balaenoptera musculus*) 5-Year Review: Summary and Evaluation. Silver Spring, MD. 15 p.

Sears, R. and W.F. Perrin. 2018. Blue Whale: *Balaenoptera musculus*. In: Würsig, B., J.G.M. Thewissen, and K.M. Kovacs (Eds.), Encyclopedia of Marine Mammals (Third Edition). Academic Press, London, UK. pp. 110-114. doi: 10.1016/B978-0-12-804327-1.00070-4

References

Cetacean and Turtle Assessment Program (CETAP). 1982. A characterization of marine mammals and turtles in the Mid- and North Atlantic areas of the U.S. outer continental shelf, final report. Contract AA551-CT8-48. Bureau of Land Management: Washington, DC. 538 p.

Christensen, I., T. Haug, and N. Øien. 1992. A review of feeding and reproduction in large baleen whales (Mysticeti) and sperm whales *Physeter macrocephalus* in Norwegian and adjacent waters. Fauna Norvegica Series A 13:39–48.

Clark, C.W. 1995. Annex M. Matters arising out of the discussion of blue whales: Annex M1. Application of US Navy underwater hydrophone arrays for scientific research on whales. Reports of the International Whaling Commission 45:210–212.

Doniol-Valcroze, T. 2008. Habitat selection and niche characteristics of rorqual whales in the northern Gulf of St. Lawrence (Canada). McGill University. Dissertation. 146 p.

Fisheries and Oceans Canada (DFO). 2009. Recovery Strategy for the blue whale (*Balaenoptera musculus*), Northwest Atlantic population, in Canada [PROPOSED]. Ottawa. 62 p.

Hammond, P.S., R. Sears, and M. Bérubé. 1990. A note on problems in estimating the number of blue whales in the Gulf of St Lawrence from photo-identification data. Reports of the International Whaling Commission (Special Issue) 12:141–142.

Hayes, S.A., E. Josephson, K. Maze-Foley, and P.E. Rosel. 2020. US Atlantic and Gulf of Mexico marine mammal stock assessments - 2019. National Oceanic and Atmospheric Administration, National Marine Fisheries Service, Northeast Fisheries Science Center: Woods Hole, MA. NOAA Technical Memorandum NMFS-NE-264. 479 p.

Hjort, J. and J.T. Ruud. 1929. Whaling and fishing in the North Atlantic. Commission Internationale pour l'Exploration Scientifique de la Mer Rapports et Proces-Verbaux des Reunions 56:551.

Jonsgård, Å. 1955. The stocks of blue whales (*Balaenoptera musculus*) in the northern Atlantic Ocean and adjacent arctic waters. Norsk Hvalfangsttid 44:505–519.

Kawamura, A. 1980. A review of food of balaenopterid whales. Scientific Reports of Whales Research Institute 32:155–197.

Lockyer, C. 1984. Review of baleen whale (Mysticeti) reproduction and implications for management. Reports of the International Whaling Commission (Special Issue) 6:27–50.

Metcalfe, C., B. Koenig, T. Metcalfe, G. Paterson, and R. Sears. 2004. Intra- and inter-species differences in persistent organic contaminants in the blubber of blue whales and humpback whales from the Gulf of St. Lawrence, Canada. Marine Environmental Research 57(4):245–260. doi: 10.1016/j.marenvres.2003.08.003

Mitchell, E. 1974. Present status of Northwest Atlantic fin and other whale stocks. In: W. E. Schevill (Ed.), The whale problem: A status report. Harvard University Press, Cambridge, MA. pp. 108–169.

Mizroch, S.A., D.W. Rice, and J.M. Breiwick. 1984. The blue whale, *Balaenoptera-musculus*. Marine Fisheries Review 46(4):15–19.

Palka, D., L. Aichinger Dias, E. Broughton, S. Chavez-Rosales, D. Cholewiak, G. Davis, A. DeAngelis, L. Garrison, H. Haas, J. Hatch, K. Hyde, M. Jech, E. Josephson, L. Mueller-Brennan, C. Orphanides, N. Pegg, C. Sasso, D. Sigourney, M. Soldevilla, and H. Walsh. 2021. Atlantic Marine Assessment Program for Protected Species: FY15 – FY19. US Department of the Interior, Bureau of Ocean Energy Management: Washington, DC. OCS Study BOEM 2021-051. 330 p.

Palka, D., L. Aichinger Dias, E. Broughton, S. Chavez-Rosales, D. Cholewiak, G. Davis, A. DeAngelis, L. Garrison, H. Haas, J. Hatch, K. Hyde, M. Jech, E. Josephson, L. Mueller-Brennan, C. Orphanides, N. Pegg, C. Sasso, D. Sigourney, M. Soldevilla, and H. Walsh. 2021. Supplement to final report BOEM 2021-051 Atlantic Marine Assessment Program for Protected Species: FY15 – FY19 Appendix II species distribution maps of sightings not used in density-habitat models. US Department of the Interior, Bureau of Ocean Energy Management: Washington, DC. OCS Study BOEM 2021-051. 330 p.

Pike, D., T. Gunnlaugsson, B. Mikkelsen, S.D. Halldórsson, and G. Víkingsson. 2019. Estimates of the abundance of cetaceans in the central North Atlantic based on the NASS Icelandic and Faroese shipboard surveys conducted in 2015. NAMMCO Scientific Publications 11. doi: 10.7557/3.4941

Ramp, C., M. Berube, W. Hagen, and R. Sears. 2006. Survival of adult blue whales *Balaenoptera musculus* in the Gulf of St. Lawrence, Canada. Marine Ecology Progress Series 319:287–295. doi: 10.3354/meps319287

Reilly, S.B. and V.G. Thayer. 1990. Blue whale (*Balaenoptera-musculus*) distribution in the Eastern Tropical Pacific. Marine Mammal Science 6(4):265–277. doi: 10.1111/j.1748-7692.1990.tb00357.x

Roberts, J.J., B.D. Best, L. Mannocci, E. Fujioka, P.N. Halpin, D.L. Palka, L.P. Garrison, K.D. Mullin, T.V.N. Cole, C.B. Khan, W.A. McLellan, D.A. Pabst, and G.G. Lockhart. 2016. Habitat-based cetacean density models for the US Atlantic and Gulf of Mexico. Scientific Reports 6:22615. doi: 10.1038/Srep22615

Roberts, J.J., B.D. Best, L. Mannocci, E. Fujioka, P.N. Halpin, D.L. Palka, L.P. Garrison, K.D. Mullin, T.V.N. Cole, C.B. Khan, W.M. McLellan, D.A. Pabst, and G.G. Lockhart. 2015. Density model for blue whale (*Balaenoptera musculus*) for the U.S. East Coast version 1.3, 2015-09-26, and supplementary report. Duke University, Marine Geospatial Ecology Lab: Durham, NC. 66 p.

Schoenherr, J.R. 1991. Blue whales feeding on high-concentrations of euphausiids around Monterey Submarine-Canyon. Canadian Journal of Zoology 69(3):583–594. doi: 10.1139/z91-088

Sears, R. and J. Calambokidis. 2002. COSEWIC Assessment and update status report on the blue whale Balaenoptera musculus, Atlantic population and Pacific poulation, in Canada. Committee on the Status of Endangered Wildlife in Canada: Ottawa, Canada. 38 p.

Sears, R. and F. Larsen. 2002. Long range movements of a blue whale (*Balaenoptera musculus*) between the Gulf of St. Lawrence and West Greenland. Marine Mammal Science 18(1):281–285. doi: 10.1111/j.1748-7692.2002.tb01034.x

Sears, R. and W.F. Perrin. 2018. Blue whale: *Balaenoptera musculus*. In: Würsig, B., J.G.M. Thewissen, and K.M. Kovacs (Eds.), Encyclopedia of Marine Mammals (Third Edition). Academic Press, London, UK. pp. 110–114. doi: 10.1016/B978-0-12-804327-1.00070-4

Sears, R., F. Wenzel, and J.M. Williamson. 1987. The blue whale: a catalog of individuals from the western North Atlantic (Gulf of St. Lawrence). Mingan Island Cetacean Study: St. Lambert, Quebec, Canada. 27 p.

Sears, R., J.M. Williamson, F.W. Wenzel, M. Bérubé, D. Gendron, and P. Jones. 1990. Photographic identification of the blue whale (*Balaenoptera musculus*) in the Gulf of St. Lawrence, Canada. Reports of the International Whaling Commission (Special Issue) 12:335–342.

Sergeant, D.E. 1966. Populations of large whale species in the western North Atlantic with special reference to the fin whale. Fisheries Research Board of Canada, Arctic Biological Station. Circular No. 9. 130 p.

Sigurjonsson, J. and T. Gunnlaugsson. 1990. Recent trends in abundance of blue (*Balaenoptera musculus*) and humpback whales (*Megaptera novaeangliae*) off west and southwest Iceland, with a note on occurrence of other cetacean species. Reports of the International Whaling Commission (Special Issue) 40:537–551.

Taylor, B.L., S.J. Chivers, J. Larese, and W.F. Perrin. 2007. Generation length and percent mature estimates for IUCN assessments of cetaceans. National Oceanic and Atmospheric Administration, National Marine Fisheries Service, Southwest Fisheries Science Center: La Jolla, CA. Administrative Report LJ-07-01. 24 p.

Wenzel, F., D.K. Mattila, and P.J. Clapham. 1988. *Balaenoptera-musculus* in the Gulf of Maine. Marine Mammal Science 4(2):172–175. doi: 10.1111/j.1748-7692.1988.tb00198.x

Yochem, P.K. and S. Leatherwood. 1985. Blue whale *Balaenoptera musculus* (Linnaeus, 1758). In: Ridgway, S.H. and R. Harrison (Eds.), Handbook of marine mammals. Volume 3: The sirenians and baleen whales. Academic Press, San Diego, CA. pp. 193–240.

Clymene dolphin − *Stenella clymene*

Northern Gulf of Mexico Stock

Overall Vulnerability Rank = High

Biological Sensitivity = Moderate

Climate Exposure = Very High

Data Quality = 81% of scores ≥ 2

	Stenella clymene	Expert Scores	Data Quality	Expert Scores Plots (Portion by Category)
Sensitivity attributes	Prey/Diet Specificity	1.8	2.0	
	Habitat Specificity	1.7	2.0	
	Site Fidelity	1.7	0.7	
	Lifetime Reproductive Potential	1.5	2.0	
	Generation Length	2.1	2.0	
	Reproductive Plasticity	1.4	0.7	
	Migration	3.3	1.7	
	Home Range	1.5	2.0	
	Stock Abundance	2.5	3.0	
	Stock Abundance Trend	2.5	0.7	
	Cumulative Stressors	2.2	0.7	
	Sensitivity Score	Moderate		
Exposure variables	Sea Surface Temperature (Change in mean)	4.0	3.0	
	Sea Surface Temperature (Change in variability)	1.0	3.0	
	Air Temperature (Change in mean)	4.0	3.0	
	Air Temperature (Change in variability)	1.0	3.0	
	Precipitation (Change in mean)	1.0	3.0	
	Precipitation (Change in variability)	1.0	3.0	
	Sea Surface Salinity (Change in mean)	2.1	2.7	
	Sea Surface Salinity (Change in variability)	1.7	2.7	
	Ocean pH (Change in mean)	4.0	3.0	
	Ocean pH (Change in variability)	1.0	3.0	
	Sea ice coverage (Change in mean)	1.0	3.0	
	Sea ice coverage (Change in variability)	1.0	3.0	
	Dissolved oxygen (Change in mean)	4.0	3.0	
	Dissolved oxygen (Change in variability)	1.0	3.0	
	Circulation	1.7	2.3	
	Sea level rise	1.0	3.0	
	Exposure Score	Very High		
Overall Vulnerability Rank		High		

Low

Moderate

High

Very High

Clymene dolphin (Northern Gulf of Mexico Stock)

Stenella clymene

CVA Results Summary

Overall Climate Vulnerability Rank: High (74% certainty from bootstrap analysis).

Climate Exposure: Very High. Four exposure factors scored greater than 3.5: Air Temperature (Standard anomaly) (4.00), Dissolved oxygen (Standard anomaly) (4.00), Ocean pH (Standard anomaly) (4.00), and Sea Surface Temperature (Standard anomaly) (4.00).

Biological Sensitivity: Moderate. Two sensitivity attributes scored greater than 2.5: Migration (3.27) and Species Abundance Trend (2.53).

Distributional Response: High

Abundance Response: Low

Phenology Response: Moderate

Data Quality: 81 % of the data quality scores were 2 or greater. 55% of sensitivity attribute data quality scores were 2 or higher.

Background Information

Stock Description

The Clymene dolphin Northern Gulf of Mexico Stock includes individuals found in the waters of the U.S. Exclusive Economic Zone in the Gulf of Mexico (Hayes et al. 2021). However, this stock is likely transboundary and the stock area probably includes similar Mexican and Cuban Gulf of Mexico waters (Hayes et al. 2021).

Prey/Diet Specificity

Based on stomach content analysis of two individuals and one observation of feeding free-ranging dolphins, Clymene dolphins feed on small pelagic fish and squid (Perrin et al. 1981; Fertl et al. 1997).

Habitat Specificity

Clymene dolphins in the northern Gulf of Mexico are found in deeper waters off the continental shelf and primarily west of the Mississippi River (Mullin et al. 1994; Maze-Foley and Mullin 2006). Clymene dolphins are mostly sighted in deep waters well beyond the edge of the continental shelf (Fertl et al. 2003). They often associate with the lower slope and deep water areas in regions of cyclonic or confluence circulation (Davis et al. 2002).

Site Fidelity

Information regarding Clymene dolphin site fidelity was not found in the literature.

Lifetime Reproductive Potential

Taylor et al. (2007) reported Clymene dolphin interbirth interval of 3.0 years based on values reported by Kasuya (1985) and Calzada et al. (1996). Taylor et al. (2007) reported Clymene dolphin age at first reproduction of 7 years and estimated age at last reproduction of 29 years. Jefferson et al. (1995) noted that pregnant females aged 15 and 16 years stranded in the northern Gulf of Mexico.

Generation Length

Taylor et al. (2007) reported generation length of 14 years at r = 0.02 and 14.7 years at r = 0.0 using information from spinner dolphins (*Stenella longirostris*) as a proxy.

Reproductive Plasticity

Information regarding Clymene dolphin mating location, habitat, and season was not found in the literature.

Migration

Clymene dolphins were seen in the winter, spring and summer during GulfCet aerial surveys (Hansen et al. 1996; Mullin and Hoggard 2000) and no seasonality in occurrence is known for this species (DoN 2007).

Home Range

Although there are only a few records from Gulf of Mexico waters beyond U.S. boundaries (e.g., Jefferson and Schiro 1997), Clymene dolphins almost certainly occur throughout the oceanic Gulf of Mexico (Jefferson et al. 2008).

Stock Abundance

The abundance of northern Gulf of Mexico Clymene dolphins is estimated to be 513 individuals (CV=1.03), based on 2017 and 2018 summer surveys in which stock abundance was estimated at 1,026 (CV=1.03) for summer 2017, based on a single sighted group, and estimated at 0 for summer 2018, based on zero sightings by primary team observers (Garrison et al. 2020). A secondary team observer reported one sighting in summer 2018, but secondary team sightings are not used directly for these abundance estimates. Three groups were sighted during a winter 2018 survey (Rappucci et al. 2019), but this survey has not been utilized for abundance estimation as yet.

Previously, the population was estimated at 129 individuals (CV=1.00) from a summer 2009 oceanic survey (Waring et al. 2012) and 6,575 individuals (CV=0.36) from 2003–2004 surveys (Mullin 2007). The 2009 and 2003–2004 estimates were revised based on the 2017 survey methodology resulting in abundance estimates of 10,900 (CV=0.42) from the 2003 survey, 13,257 (CV=0.81) from the 2004 survey, and 1,319 (CV=0.78) from the 2009 survey (Garrison et al. 2020).

Mullin and Fulling (2004) estimated 17,355 individuals (CV=0.65) in April-June 1996-2001. Hansen et al. (1995) estimated 5,571 individuals (CV=0.37) in April-June 1991-1994. Roberts et al. (2015, 2016)

estimated 11,000 individuals (CV=0.16) for all months during the period 1992-2009 using a density model.

Stock Abundance Trend

Five point estimates of Clymene dolphin abundance have been made based on data from surveys covering 2003–2018, however imprecise estimates and long intervals between surveys result in poor statistical power to detect a trend (Garrison et al. 2020; Hayes et al. 2021). The DWH NRDA Trustees estimated that 2% of the Clymene dolphin stock was killed and 3% of females sustained reproductive failure following the Deepwater Horizon event (DWH NRDA 2016).

Cumulative Stressors

The Clymene dolphin Gulf of Mexico stock may interact with longline fisheries. During the period 2014–2019, no fishing-related mortality or serious injury of Clymene dolphins was reported (Garrison and Stokes 2016, 2017, 2019, 2020a, 2020b, 2021; Maze Foley and Garrison 2021). During the period 2014–2018, 16 Clymene dolphins were reported stranded along the Gulf of Mexico coast (Hayes et al. 2021).

The Clymene dolphin stock was likely exposed to oil and dispersants resulting from the Deepwater Horizon oil spill, which may result in long term chronic health effects (NOAA 2011; Helm et al. 2015; DWH NRDA 2016; Aichinger Dias et al. 2017; Takeshita et al. 2017). An Unusual Mortality Event (UME), involving primarily bottlenose dolphins, was declared for cetaceans in the northern Gulf of Mexico from 2010 to 2014, with the Deepwater Horizon oil spill as the primary underlying cause (Litz et al. 2014; Schwacke et al. 2014; Venn-Watson et al. 2015).

Distribution and Sightings

Density model results for Clymene dolphin in the Gulf of Mexico are presented by Roberts et al. (2015, 2016).

Further Reading

Hayes, S.A., E. Josephson, K. Maze-Foley, P.E. Rosel, and J. Turek. 2021. US Atlantic and Gulf of Mexico marine mammal stock assessments - 2020. National Oceanic and Atmospheric Administration, National Marine Fisheries Service, Northeast Fisheries Science Center: Woods Hole, MA. NOAA Technical Memorandum NMFS-NE-271. 394 p.

Jefferson, T.A. 2018. Clymene Dolphin: *Stenella clymene*. In: Würsig, B., Thewissen, J.G.M. and Kovacs, K.M. eds. Encyclopedia of marine mammals. Academic Press. Pages 197-200

Jefferson, T.A. and G. Braulik. 2018. *Stenella clymene*. The IUCN Red List of Threatened Species 2018: e.T20730A50373865. https://dx.doi.org/10.2305/IUCN.UK.2018-2.RLTS.T20730A50373865.en

References

Aichinger Dias, L., J. Litz, L. Garrison, A. Martinez, K. Barry, and T. Speakman. 2017. Exposure of cetaceans to petroleum products following the Deepwater Horizon oil spill in the Gulf of Mexico. Endangered Species Research 33:119–125. doi: 10.3354/esr00770

Calzada, N., A. Aguilar, T.B. Sorenson, and C. Lockyer. 1996. Reproductive biology of female striped dolphin (*Stenella coeruleoalba*) from the western Mediterranean. The Zoological Society of London 240:581–591.

Davis, R.W., J.G. Ortega-Ortiz, C.A. Ribic, W.E. Evans, D.C. Biggs, P.H. Ressler, R.B. Cady, R.R. Leben, K.D. Mullin, and B. Wursig. 2002. Cetacean habitat in the northern oceanic Gulf of Mexico. Deep-Sea Research Part I-Oceanographic Research Papers 49(1):121–142. doi: 10.1016/S0967-0637(01)00035-8

Deepwater Horizon Natural Resource Damage Assessment Trustees (DWH NRDA). 2016. Deepwater Horizon oil spill: Final Programmatic Damage Assessment and Restoration Plan and Final Programmatic Environmental Impact Statement. Retrieved from: http://www.gulfspillrestoration.noaa.gov/restoration-planning/gulf-plan.1659.

Department of the Navy (DoN). 2007. Marine resources assessment for the Gulf of Mexico. Department of the Navy, U.S. Fleet Forces Command, Norfolk, VA. Contract #N62470-02-D-9997, CTO 0030. Prepared by Geo-Marine, Inc., Hampton, VA. 445 p.

Fertl, D., T.A. Jefferson, I.B. Moreno, A.N. Zerbini, and K.D. Mullin. 2003. Distribution of the Clymene dolphin *Stenella clymene*. Mammal Review 33(3-4):253–271. doi: 10.1046/j.1365-2907.2003.00033.x

Fertl, D., A.J. Schiro, and D. Peake. 1997. Coordinated feeding by Clymene dolphins (*Stenella clymene*) in the Gulf of Mexico. Aquatic Mammals 23(2):111–112.

Garrison, L.P., J. Ortega-Ortiz, and G. Rappucci. 2020. Abundance of marine mammals in waters of the U.S. Gulf of Mexico during the summers of 2017 and 2018. National Oceanic and Atmospheric Administration, National Marine Fisheries Service, Southeast Fisheries Science Center: Miami, FL. Southeast Fisheries Science Center Reference Document PRBD-2020-07. 56 p. doi: 10.25923/3px6-9v48

Garrison, L.P. and L. Stokes. 2016. Estimated bycatch of marine mammals and sea turtles in the U.S. Atlantic pelagic longline fleet during 2014. National Oceanic and Atmospheric Administration, National Marine Fisheries Service, Southeast Fisheries Science Center: Miami, FL. NOAA Technical Memorandum NMFS-SEFSC-696. 62 p. doi: 10.7289/V5/TM-SEFSC-696

Garrison, L.P. and L. Stokes. 2017. Estimated bycatch of marine mammals and sea turtles in the U.S. Atlantic pelagic longline fleet during 2015. National Oceanic and Atmospheric Administration, National Marine Fisheries Service, Southeast Fisheries Science Center: Miami, FL. NOAA Technical Memorandum NMFS-SEFSC-709. 61 p. doi: 10.25923/kaf6-5c33

Garrison, L.P. and L. Stokes. 2019. Estimated bycatch of marine mammals and sea turtles in the U.S. Atlantic pelagic longline fleet during 2016. National Oceanic and Atmospheric Administration, National Marine Fisheries Service, Southeast Fisheries Science Center: Miami, FL. Southeast Fisheries Science Center Reference Document PRBD-2019-01. 43 p. doi: 10.25923/d893-d142

Garrison, L.P. and L. Stokes. 2020. Estimated bycatch of marine mammals and sea turtles in the U.S. Atlantic pelagic longline fleet during 2017. National Oceanic and Atmospheric Administration, National Marine Fisheries Service, Southeast Fisheries Science Center: Miami, FL. Southeast Fisheries Science Center Reference Document PRBD 2020-05. 58 p. doi: 10.25923/a3hs-ea60

Garrison, L.P. and L. Stokes. 2020. Estimated bycatch of marine mammals and sea turtles in the U.S. Atlantic pelagic longline fleet during 2018. National Oceanic and Atmospheric Administration, National Marine Fisheries Service, Southeast Fisheries Science Center: Miami, FL. Southeast Fisheries Science Center Reference Document PRBD 2020-08. 44 p. doi: 10.25923/777n-w316

Garrison, L.P. and L.W. Stokes. 2021. Estimated bycatch of marine mammals and sea turtles in the U.S. Atlantic pelagic longline fleet during 2019. doi: 10.25923/asb3-6q44

Hansen, L.J., K.D. Mullin, T.J. Jefferson, and G.P. Scott. 1996. Visual surveys aboard ships and aircraft. In: Davis, R.W. and G.S. Fargion (Eds.), Distribution and abundance of marine mammals in the

northcentral and western Gulf of Mexico: Final report. Volume II: Technical report. Minerals Management Service, Gulf of Mexico OCS Region, New Orleans, LA. pp. 55–132.

Hansen, L.J., K.D. Mullin, and C.L. Roden. 1995. Estimates of cetacean abundance in the northern Gulf of Mexico from vessel surveys. National Oceanic and Atmospheric Administration, National Marine Fisheries Service, Southeast Fisheries Science Center: Miami, FL. MIA-94/95-25. 9 p.

Hayes, S.A., E. Josephson, K. Maze-Foley, P.E. Rosel, and J. Turek. 2021. US Atlantic and Gulf of Mexico marine mammal stock assessments - 2020. National Oceanic and Atmospheric Administration, National Marine Fisheries Service, Northeast Fisheries Science Center: Woods Hole, MA. NOAA Technical Memorandum NMFS-NE-271. 394 p.

Helm, R.C., D.P. Costa, T.D. DeBruyn, T.J. O'Shea, R.S. Wells, and T.M. Williams. 2015. Overview of effects of oil spills on marine mammals. In: Fingas, M. (Ed.), Handbook of oil spill science and technology, First edition. John Wiley & Sons, Inc., Hoboken, NJ. pp. 455–475.

Jefferson, T.A., D.K. Odell, and K.T. Prunier. 1995. Notes on the biology of the Clymene dolphin (*Stenella clymene*) in the northern Gulf-of-Mexico. Marine Mammal Science 11(4):564–573. doi: 10.1111/j.1748-7692.1995.tb00679.x

Jefferson, T.A. and A.J. Schiro. 1997. Distribution of cetaceans in the offshore Gulf of Mexico. Mammal Review 27(1):27–50. doi: 10.1111/j.1365-2907.1997.tb00371.x

Jefferson, T.A., M.A. Webber, and R.L. Pitman. 2008. Marine mammals of the world. Elsevier/Academic Press, New York. 573 p.

Kasuya, T. 1985. Effect of exploitation of on reproductive parameters of the spotted and striped dolphins off the Pacific coast of Japan. Scientific Reports of the Whales Research Institute, Tokyo 36:107–138.

Litz, J.A., M.A. Baran, S.R. Bowen-Stevens, R.H. Carmichael, K.M. Colegrove, L.P. Garrison, S.E. Fire, E.M. Fougeres, R. Hardy, S. Holmes, W. Jones, B.E. Mase-Guthrie, D.K. Odell, P.E. Rosel, J.T. Saliki, D.K. Shannon, S.F. Shippee, S.M. Smith, E.M. Stratton, M.C. Tumlin, H.R. Whitehead, G.A. Worthy, and T.K. Rowles. 2014. Review of historical unusual mortality events (UMEs) in the Gulf of Mexico (1990–2009): providing context for the multi-year northern Gulf of Mexico cetacean UME declared in 2010. Diseases of Aquatic Organisms 112(2):161–175. doi: 10.3354/dao02807

Maze-Foley, K. and K.D. Mullin. 2006. Cetaceans of the oceanic northern Gulf of Mexico: Distributions, group sizes and interspecific associations. Journal of Cetacean Research and Management 8(2):203–213.

Mullin, K.D. 2007. Abundance of cetaceans in the oceanic Gulf of Mexico based on 2003-2004 ship surveys. National Oceanic and Atmospheric Administration, National Marine Fisheries Service, Southeast Fisheries Science Center: Pascagoula, MS. 26 p.

Mullin, K.D. and G.L. Fulling. 2004. Abundance of cetaceans in the oceanic northern Gulf of Mexico, 1996-2001. Marine Mammal Science 20(4):787–807. doi: 10.1111/j.1748-7692.2004.tb01193.x

Mullin, K.D. and W. Hoggard. 2000. Visual surveys of cetaceans and sea turtles from aircraft and ships. In: Davis, R.W., W.E. Evans, and B. Würsig (Eds.), Cetaceans, sea turtles and seabirds in the northern Gulf of Mexico: Distribution, abundance and habitat associations. Volume II: Technical report. U.S. Department of the Interior, Minerals Management Service, Gulf of Mexico OCS Region, New Orleans, LA. pp. 111–172.

Mullin, K.D., W. Hoggard, C.L. Roden, R.R. Lohoefener, C.M. Rogers, and B. Taggart. 1994. Cetaceans on the upper continental-slope in the North-Central Gulf-of-Mexico. Fishery Bulletin 92(4):773–786.

National Oceanic and Atmospheric Administration (NOAA). 2011. Public scoping for preparation of a programmatic environmental impact statement for the Deepwater Horizon BP Oil Spill. 9 p.

Perrin, W.F., E.D. Mitchell, J.G. Mead, D.K. Caldwell, and P.J.H. Vanbree. 1981. *Stenella-clymene*, a rediscovered tropical dolphin of the Atlantic. Journal of Mammalogy 62(3):583–598. doi: 10.2307/1380405

Rappucci, G., A. Martinez, J. Litz, L. Aichinger Dias, M. Soldevilla, K. Ternus, L.P. Garrison, and K.D. Mullin. 2019. GoMMAPPS Winter 2018 Research Cruise Report GU18-01. doi: 10.25923/8b6m-y457

Roberts, J.J., B.D. Best, L. Mannocci, E. Fujioka, P.N. Halpin, D.L. Palka, L.P. Garrison, K.D. Mullin, T.V.N. Cole, C.B. Khan, W.A. McLellan, D.A. Pabst, and G.G. Lockhart. 2016. Habitat-based cetacean density models for the US Atlantic and Gulf of Mexico. Scientific Reports 6:22615. doi: 10.1038/Srep22615

Roberts, J.J., B.D. Best, L. Mannocci, E. Fujioka, P.N. Halpin, D.L. Palka, L.P. Garrison, K.D. Mullin, T.V.N. Cole, C.B. Khan, W.M. McLellan, D.A. Pabst, and G.G. Lockhart. 2015. Density model for Clymene dolphin (*Stenella clymene*) for the U.S. Gulf of Mexico version 2.3, 2015-09-27, and supplementary report. Duke University, Marine Geospatial Ecology Lab: Durham, NC. 69 p.

Takeshita, R., L. Sullivan, C. Smith, T. Collier, A. Hall, T. Brosnan, T. Rowles, and L. Schwacke. 2017. The Deepwater Horizon oil spill marine mammal injury assessment. Endangered Species Research 33:95–106. doi: 10.3354/esr00808

Taylor, B.L., S.J. Chivers, J. Larese, and W.F. Perrin. 2007. Generation length and percent mature estimates for IUCN assessments of cetaceans. National Oceanic and Atmospheric Administration, National Marine Fisheries Service, Southwest Fisheries Science Center: La Jolla, CA. Administrative Report LJ-07-01. 24 p.

Venn-Watson, S., K.M. Colegrove, J. Litz, M. Kinsel, K. Terio, J. Saliki, S. Fire, R. Carmichael, C. Chevis, W. Hatchett, J. Pitchford, M. Tumlin, C. Field, S. Smith, R. Ewing, D. Fauquier, G. Lovewell, H. Whitehead, D. Rotstein, W. McFee, E. Fougeres, and T. Rowles. 2015. Adrenal gland and lung lesions in Gulf of Mexico common bottlenose dolphins (*Tursiops truncatus*) found dead following the Deepwater Horizon Oil Spill. PLoS ONE 10(5):e0126538. doi: 10.1371/journal.pone.0126538

Waring, G.T., E. Josephson, K. Maze-Foley, and P.E. Rosel. 2012. U.S. Atlantic and Gulf of Mexico marine mammal stock assessments - 2011. National Oceanic and Atmospheric Administration, National Marine Fisheries Service, Northeast Fisheries Science Center: Woods Hole, MA. NOAA Technical Memorandum NMFS-NE-221. 319 p.

Common bottlenose dolphin − *Tursiops truncatus*

GoMx BSE/Apalachee Bay Stock

Overall Vulnerability Rank = Very High

Biological Sensitivity = High

Climate Exposure = Very High

Data Quality = 74% of scores ≥ 2

	Tursiops truncatus	Expert Scores	Data Quality	Expert Scores Plots (Portion by Category)
Sensitivity attributes	Prey/Diet Specificity	2.0	2.0	
	Habitat Specificity	3.4	1.8	
	Site Fidelity	3.8	1.8	
	Lifetime Reproductive Potential	1.4	2.2	
	Generation Length	2.8	2.2	
	Reproductive Plasticity	1.4	2.2	
	Migration	3.8	1.8	
	Home Range	3.4	1.8	
	Stock Abundance	3.0	2.0	
	Stock Abundance Trend	2.3	1.0	
	Cumulative Stressors	3.3	1.5	
	Sensitivity Score	High		
Exposure variables	Sea Surface Temperature (Change in mean)	4.0	2.5	
	Sea Surface Temperature (Change in variability)	1.0	2.5	
	Air Temperature (Change in mean)	4.0	2.5	
	Air Temperature (Change in variability)	1.0	2.5	
	Precipitation (Change in mean)	1.0	2.5	
	Precipitation (Change in variability)	1.0	2.5	
	Sea Surface Salinity (Change in mean)	2.2	2.5	
	Sea Surface Salinity (Change in variability)	1.4	2.5	
	Ocean pH (Change in mean)	4.0	2.5	
	Ocean pH (Change in variability)	1.0	2.5	
	Sea ice coverage (Change in mean)	1.0	2.5	
	Sea ice coverage (Change in variability)	1.0	2.5	
	Dissolved oxygen (Change in mean)	4.0	2.5	
	Dissolved oxygen (Change in variability)	1.0	2.5	
	Circulation	3.8	1.2	
	Sea level rise	3.0	2.5	
	Exposure Score	Very High		
Overall Vulnerability Rank		Very High		

Low
Moderate
High
Very High

Common bottlenose dolphin (GoMx BSE/Apalachee Bay Stock)

Tursiops truncatus

CVA Results Summary

Overall Climate Vulnerability Rank: Very High (100% certainty from bootstrap analysis).

Climate Exposure: Very High. Five exposure factors scored greater than 3.5: Air Temperature (Standard anomaly) (4.00), Dissolved oxygen (Standard anomaly) (4.00), Ocean pH (Standard anomaly) (4.00), Sea Surface Temperature (Standard anomaly) (4.00), and Circulation (3.75).

Biological Sensitivity: High. Six sensitivity attributes scored greater than or equal to 3.0: Site Fidelity (3.80), Migration (3.75), Habitat Specificity (3.45), Home Range (3.45), Cumulative Stressors (3.30), and Species Abundance (3.00).

Distributional Response: High

Abundance Response: High

Phenology Response: High

Data Quality: 74% of the data quality scores were 2 or greater, 45% of sensitivity attribute data quality scores were 2 or higher.

Background Information

Stock Description

This stock includes common bottlenose dolphins found in Apalachee Bay in the northeast Gulf of Mexico along the Florida coast (Hayes et al. 2022).

Prey/Diet Specificity

Generally, common bottlenose dolphins consume a large variety of fish and/or squid (Barros and Odell 1990; Mead and Potter 1990; Wells and Scott 2018). Common bottlenose dolphins seem to show a consistent preference for sound-producing fishes (e.g., sciaenids, toadfish; Berens McCabe et al. 2010; Bowen-Stevens et al. 2021). In Sarasota Bay, Florida, Rossman et al. (2015) found some individual common bottlenose dolphins to show diet specialization despite the broader study group showing a generalist diet. Specialization can be influenced by locally abundant prey species (Shane et al. 1986; Vollmer and Rosel 2013) or can result from resource partitioning amongst sympatric populations (e.g., Torres and Read 2009; Wilson et al. 2017).

Habitat Specificity

Common bottlenose dolphins are found in a variety of marine and estuarine habitats (Wells and Scott 2018).

Site Fidelity

No specific site fidelity information was found for this stock. However, common bottlenose dolphins in other Gulf of Mexico bay, sound, and estuary (BSE) stocks show site fidelity (LaBrecque et al. 2015; Hayes et al. 2022), such as in Matagorda Bay, Espiritu Santo Bay, and San Luis Pass in Texas (Maze and Würsig 1999; Lynn and Würsig 2002; Irwin and Würsig 2004); Barataria Bay in Louisiana (Miller 2003; McDonald et al. 2017; Wells et al. 2017; Takeshita et al. 2021); Mississippi Sound in Mississippi (Hubard et al. 2004; Mackey 2010; Mullin et al. 2017); Charlotte Harbor and Pine Island Sound (Bassos-Hull et al. 2013), Sarasota Bay (Wells 1986a; Wells 1991; Wells 2014), St. Andrew Bay (Balmer et al. 2019), St. Joseph Bay (Balmer et al. 2008; B. Balmer et al. 2018), St. Vincent Sound and Apalachicola Bay (Tyson et al. 2011), and Tampa Bay in Florida (Wells et al. 1996b; Urian et al. 2009).

Lifetime Reproductive Potential

Taylor et al. (2007) reported the interbirth interval of common bottlenose dolphins as 3.80 years, though other studies have shown it to be closer to three years (Leatherwood 1977; Wells and Scott 1999; Connor et al. 2000; Wells 2003).

Generally, females reach sexual maturity before males, with females reaching sexual maturity at 5–13 years (Sergeant et al. 1973; Perrin and Reilly 1984; Wells et al. 1987; Mead and Potter 1990; Mann et al. 2000; Wells 2000; Wells and Scott 2018).

Females reproduce through most of their adult lives (Leatherwood 1977; Hohn et al. 1989; Wells and Scott 1999; Wells 2000; Wells 2003), but may produce fewer calves after they reach 30 years of age (Wells 2000). Sarasota Bay common bottlenose dolphins can give birth when they are as young as 6 years of age, and can continue until they are 48 years old (Wells and Scott 2018). Reproductive lifespan would therefore be up to 42 years based on long-term observation studies (Wells 2014; Wells and Scott 2018).

Longitudinal studies from Sarasota Bay, Florida have found that female common bottlenose dolphins can live up to 67 years (Wells and Scott 2018). Studies based on growth layer groups in teeth suggest female common bottlenose dolphins can live to 41 years along the Texas coast (Fernandez and Hohn 1998) and to 30 years along the Mississippi coast (Mattson et al. 2006).

Generation Length

Taylor et al. (2007) reported a common bottlenose dolphin generation length of 20.6–21.1 years.

Reproductive Plasticity

Common bottlenose dolphin calving is generally diffusely seasonal, with one or more peaks, although births have been reported from all seasons (Vollmer and Rosel 2013; Wells and Scott 2018). Calf sightings peak in the spring and summer months in Florida (Wells et al. 1987), Mississippi/Louisiana (Miller et al. 2010; Miller et al. 2013), and Texas (McHugh 1989; Henderson et al. 2007).

Migration

The BSE stocks in the Gulf of Mexico are thought to primarily comprise stable resident communities. However, transient or seasonal movements of some individuals into and out of a BSE stock area are recorded in some areas (LaBrecque et al. 2015; Hayes et al. 2022). Molecular genetic data support that some BSE stocks are genetically distinct from one another, and furthermore are differentiated from adjacent coastal populations (e.g., Sellas et al. 2005; Rosel et al. 2017). For example, in inshore systems along the Florida Panhandle, Toms (2019) found fine-scale genetic population structure among five stocks and variable migration rates among populations.

Year-round residency has been reported for common bottlenose dolphins in Florida (Irvine and Wells 1972; Irvine et al. 1981; Wells 1986a; Wells 1986b; Wells et al. 1987; Scott et al. 1990; Wells 1991; Wells et al. 1996a; Wells et al. 1996b; Wells et al. 1997; Wells 2003; Balmer et al. 2008; Urian et al. 2009; Tyson et al. 2011; Bassos-Hull et al. 2013; B. Balmer et al. 2018; Balmer et al. 2019; Toms 2019), Texas (Shane 1990; Bräger 1993; Bräger et al. 1994; Fertl 1994; Weller 1998; Maze and Würsig 1999; Lynn and Würsig 2002; Irwin and Würsig 2004; Shane 2004), and Louisiana/Mississippi (Hubard et al. 2004; McDonald et al. 2017; Mullin et al. 2017; Takeshita et al. 2021; Hayes et al. 2022).

Home Range

The home ranges of individuals within the Gulf of Mexico BSE stocks are considered to generally include the areas within the stock boundaries and brief excursions beyond the stock boundaries (Irvine et al. 1981; Wells 1986a; Wells 1986b; Wells 1991; Wells et al. 1996a; Wells et al. 1996b; Balmer et al. 2008; Wells 2014; Balmer et al. 2019), although some individuals show preference for small areas within the stock boundaries (Lynn and Würsig 2002; Urian et al. 2009; Mullin et al. 2017; Wells et al. 2017).

Stock Abundance

The most recent abundance estimate of 491 individuals (CV=0.39) comes from 1993 (Blaylock and Hoggard 1994; Hayes et al. 2022). More recent estimates are unavailable (Hayes et al. 2022).

Stock Abundance Trend

Data are insufficient to complete an abundance trend analysis for this stock (Hayes et al. 2022).

Cumulative Stressors

Common bottlenose dolphins face a wide range of individual and cumulative natural and anthropogenic threats (see reviews by Vollmer and Rosel 2013; Wells and Scott 2018; and Wells et al. 2019). These include such threats as shark predation, harmful algal blooms, recreational and commercial fishing gear, vessel traffic, pollution, habitat alteration, and provisioning. Dolphins along the panhandle region of Florida have been frequent targets of harassment (Vail 2016).

The nearshore and estuarine habitats along the Gulf of Mexico coast include waters adjacent to areas of high human population, some of which are highly industrialized, and areas of heavy agricultural impact. Studies have shown persistent organic pollutants (POPs) in tissues of common bottlenose dolphins from the Florida Panhandle (Wilson et al. 2012; Balmer et al. 2019) and other Florida Gulf Coast waters (Yordy et al. 2010a, 2010b, 2010c; Kucklick et al. 2011; J. Balmer et al. 2018). Similarly, mercury has been

detected in common bottlenose dolphin tissues in Florida Gulf Coast waters (Bryan et al. 2007; Woshner et al. 2008; Miller et al. 2011; Correa et al. 2013; McCormack et al. 2020a, 2020b, 2022).

An Unusual Mortality Event (UME) was declared in 1990 for the Gulf of Mexico from Florida to Texas, with more than 300 common bottlenose dolphins stranding due to an undetermined cause, although morbillivirus and cold temperatures may have contributed (Litz et al. 2014).

Further Reading

Hayes, S.A., E. Josephson, K. Maze-Foley, P.E. Rosel, and J. Wallace. 2022. U.S. Atlantic and Gulf of Mexico marine mammal stock assessments 2021. National Oceanic and Atmospheric Administration, National Marine Fisheries Service, Northeast Fisheries Science Center: Woods Hole, MA. NOAA Technical Memorandum NMFS-NE-288. 380 p. doi: 10.25923/6tt7-kc16

Wells, R.S. and M.D. Scott. 2018. Bottlenose Dolphin, *Tursiops truncatus*, Common Bottlenose Dolphin. In: Würsig, B., J.G.M. Thewissen, and K.M. Kovacs (Eds.), Encyclopedia of Marine Mammals (Third Edition). Academic Press, London, UK. pp. 118–125. doi: 10.1016/B978-0-12-804327-1.00072-8

Wells, R.S., A. Natoli, and G. Braulik. 2019. *Tursiops truncatus* (errata version published in 2019). The IUCN Red List of Threatened Species 2019: e.T22563A156932432. https://dx.doi.org/10.2305/IUCN.UK.2019-1.RLTS.T22563A156932432.en

References

Balmer, B., T. McDonald, F. Hornsby, J. Adams, J. Allen, A. Barleycorn, and L. Schwacke. 2018. Long-term trends in a northern Gulf of Mexico common bottlenose dolphin (*Tursiops truncatus*) population in the wake of the Deepwater Horizon oil spill. Journal of Cetacean Research and Management 18:1–9. doi: 10.47536/jcrm.v18i1.409

Balmer, B., S. Watwood, B. Quigley, T. Speakman, K. Barry, K. Mullin, P. Rosel, C. Sinclair, E. Zolman, and L. Schwacke. 2019. Common bottlenose dolphin (*Tursiops truncatus*) abundance and distribution patterns in St. Andrew Bay, Florida, USA. Aquatic Conservation: Marine and Freshwater Ecosystems 29(3):486–498. doi: 10.1002/aqc.3001

Balmer, B.C., R.S. Wells, S.M. Nowacek, D.P. Nowacek, L.H. Schwacke, W.A. McLellan, F.S. Scharf, T.K. Rowles, L.J. Hansen, T.R. Spradlin, and D.A. Pabst. 2008. Seasonal abundance and distribution patterns of common bottlenose dolphins (*Tursiops truncatus*) near St. Joseph Bay, Florida, USA. Journal of Cetacean Research and Management 10(2):157–167.

Balmer, J.E., G.M. Ylitalo, T.K. Rowles, K.D. Mullin, R.S. Wells, F.I. Townsend, R.W. Pearce, J.L. Bolton, E.S. Zolman, B.C. Balmer, and L.H. Schwacke. 2018. Persistent organic pollutants (POPs) in blood and blubber of common bottlenose dolphins (*Tursiops truncatus*) at three northern Gulf of Mexico sites following the Deepwater Horizon oil spill. Science of the Total Environment 621:130–137. doi: 10.1016/j.scitotenv.2017.11.209

Barros, N.B. and D.K. Odell. 1990. Food habits of bottlenose dolphins in the southeastern United States In: Leatherwood, S. and R.R. Reeves (Eds.), The Bottlenose Dolphin. Academic Press, San Diego, CA. pp. 309–328. doi: 10.1016/B978-0-12-440280-5.50020-2

Bassos-Hull, K., R.M. Perrtree, C.C. Shepard, S. Schilling, A.A. Barleycorn, J.B. Allen, B.C. Balmer, W.E. Pine, and R.S. Wells. 2013. Long-term site fidelity and seasonal abundance estimates of common bottlenose dolphins (*Tursiops truncatus*) along the southwest coast of Florida and responses to natural perturbations. Journal of Cetacean Research and Management 13(1):19–30. doi: 10.47536/jcrm.v13i1.551

Berens McCabe, E.J., D.P. Gannon, N.B. Barros, and R.S. Wells. 2010. Prey selection by resident common bottlenose dolphins (*Tursiops truncatus*) in Sarasota Bay, Florida. Marine Biology 157(5):931–942. doi: 10.1007/s00227-009-1371-2

Blaylock, R.A. and W. Hoggard. 1994. Preliminary estimates of bottlenose dolphin abundance in southern U.S. Atlantic and Gulf of Mexico continental shelf waters. National Oceanic and Atmospheric Administration, National Marine Fisheries Service, Southeast Fisheries Science Center: Miami, FL. NOAA Technical Memorandum NMFS-SEFSC-356. 10 p.

Bowen-Stevens, S.R., D.P. Gannon, R.A. Hazelkorn, G. Lovewell, K.M. Volker, S. Smith, M.C. Tumlin, and J. Litz. 2021. Diet of common bottlenose dolphins, *Tursiops truncatus*, that stranded in and near Barataria Bay, Louisiana, 2010–2012. Southeastern Naturalist 20(1). doi: 10.1656/058.020.0113

Bräger, S. 1993. Diurnal and seasonal behavior patterns of bottlenose dolphins (*Tursiops truncatus*). Marine Mammal Science 9:434–438. doi: 10.1111/j.1748-7692.1993.tb00477.x

Bräger, S., B. Würsig, A. Acevedo, and T. Henningsen. 1994. Association patterns of bottle-nosed dolphins (*Tursiops-truncatus*) in Galveston Bay, Texas. Journal of Mammalogy 75(2):431–437. doi: 10.2307/1382564

Bryan, C.E., S.J. Christopher, B.C. Balmer, and R.S. Wells. 2007. Establishing baseline levels of trace elements in blood and skin of bottlenose dolphins in Sarasota Bay, Florida: implications for non-invasive monitoring. Science of the Total Environment 388(1-3):325–342. doi: 10.1016/j.scitotenv.2007.07.046

Connor, R.C., R.S. Wells, J. Mann, and A.J. Read. 2000. The bottlenose dolphin: Social relationships in a fission-fusion society. In: Mann, J., R. Connor, P. Tyack, and H. Whitehead (Eds.), Cetacean Societies: Field Studies of Dolphins and Whales. University of Chicago Press, Chicago, IL. pp. 91–126.

Correa, L., J.M. Castellini, R.S. Wells, and T. O'Hara. 2013. Distribution of mercury and selenium in blood compartments of bottlenose dolphins (*Tursiops truncatus*) from Sarasota Bay, Florida. Environmental Toxicology and Chemistry 32(11):2441–2448. doi: 10.1002/etc.2327

Fernandez, S. and A.A. Hohn. 1998. Age, growth, and calving season of bottlenose dolphins, *Tursiops truncatus*, off coastal Texas. Fishery Bulletin 96(2):357–365.

Fertl, D. 1994. Occurrence patterns and behavior of bottlenose dolphins (*Tursiops truncatus*) in the Galveston ship channel, Texas. Texas Journal of Science 46(4):299–318.

Hayes, S.A., E. Josephson, K. Maze-Foley, P.E. Rosel, and J. Wallace. 2022. U.S. Atlantic and Gulf of Mexico marine mammal stock assessments 2021. National Oceanic and Atmospheric Administration, National Marine Fisheries Service, Northeast Fisheries Science Center: Woods Hole, MA. NOAA Technical Memorandum NMFS-NE-288. 380 p. doi: 10.25923/6tt7-kc16

Henderson, E.E. and B. Würsig. 2007. Behavior patterns of bottlenose dolphins in San Luis Pass, Texas. Gulf of Mexico Science 25(2):153–161. doi: 10.18785/goms.2502.06

Hohn, A.A., M.D. Scott, R.S. Wells, J.C. Sweeney, and A.B. Irvine. 1989. Growth layers in teeth from known-age, free-ranging bottlenose dolphins. Marine Mammal Science 5(4):315–342. doi: 10.1111/j.1748-7692.1989.tb00346.x

Hubard, C.W., K. Maze-Foley, K.D. Mullin, and W.W. Schroeder. 2004. Seasonal abundance and site fidelity of bottlenose dolphins (*Tursiops truncatus*) in Mississippi Sound. Aquatic Mammals 30(2):299–310. doi: 10.1578/am.30.2.2004.299

Irvine, A.B., M.D. Scott, R.S. Wells, and J.H. Kaufmann. 1981. Movements and activities of the Atlantic bottlenose dolphin, *Tursiops truncatus*, near Sarasota, Florida. Fishery Bulletin 79(4):671–688.

Irvine, B. and R.S. Wells. 1972. Results of attempts to tag Atlantic bottlenosed dolphins (*Tursiops truncatus*). Cetology 13:1–5.

Irwin, L.-J. and B. Würsig. 2004. A small resident community of bottlenose dolphins, *Tursiops truncatus*, in Texas: Monitoring recommendations. Gulf of Mexico Science 22(1):13–21. doi: 10.18785/goms.2201.02

Kucklick, J., L. Schwacke, R. Wells, A. Hohn, A. Guichard, J. Yordy, L. Hansen, E. Zolman, R. Wilson, J. Litz, D. Nowacek, T. Rowles, R. Pugh, B. Balmer, C. Sinclair, and P. Rosel. 2011. Bottlenose dolphins as indicators of persistent organic pollutants in the western North Atlantic Ocean and Northern Gulf of Mexico. Environmental Science & Technology 45(10):4270–4277. doi: 10.1021/es1042244

LaBrecque, E., C. Curtice, J. Harrison, S.M. Van Parijs, and P.N. Halpin. 2015. 3. Biologically Important Areas for cetaceans within U.S. Waters – Gulf of Mexico Region. Aquatic Mammals 41(1):30–38. doi: 10.1578/am.41.1.2015.30

Leatherwood, S. 1977. Some preliminary impressions on the numbers and social behavior of free-swimming bottlenosed dolphin calves (*Tursiops truncatus*) in the northern Gulf of Mexico. In: Ridgway, S.H. and K. Bernirschke (Eds.), Breeding Dolphins; Present Status, Suggestions for the Future. Final report to the US Marine Mammal Commission, Contract MM6AC009, MMC-76/07, Washington, DC. pp. 143–167.

Litz, J.A., M.A. Baran, S.R. Bowen-Stevens, R.H. Carmichael, K.M. Colegrove, L.P. Garrison, S.E. Fire, E.M. Fougeres, R. Hardy, S. Holmes, W. Jones, B.E. Mase-Guthrie, D.K. Odell, P.E. Rosel, J.T. Saliki, D.K. Shannon, S.F. Shippee, S.M. Smith, E.M. Stratton, M.C. Tumlin, H.R. Whitehead, G.A. Worthy, and T.K. Rowles. 2014. Review of historical unusual mortality events (UMEs) in the Gulf of Mexico (1990–2009): providing context for the multi-year northern Gulf of Mexico cetacean UME declared in 2010. Diseases of Aquatic Organisms 112(2):161–175. doi: 10.3354/dao02807

Lynn, S.K. and B. Würsig. 2002. Summer movement patterns of bottlenose dolphins in a Texas bay. Gulf of Mexico Science 20(1):25–37. doi: 10.18785/goms.2001.03

Mackey, A.D. 2010. Site fidelity and association patterns of bottlenose dolphins (*Tursiops truncatus*) in the Mississippi Sound. University of Southern Mississippi. Thesis. 133 p.

Mann, J., R.C. Connor, L.M. Barre, and M.R. Heithaus. 2000. Female reproductive success in bottlenose dolphins (*Tursiops* sp.): life history, habitat, provisioning, and group-size effects. Behavioral Ecology 11(2):210–219. doi: 10.1093/beheco/11.2.210

Mattson, M.C., K.D. Mullin, G.W. Ingram, and W. Hoggard. 2006. Age structure and growth of the bottlenose dolphin (*Tursiops truncatus*) from strandings in the Mississippi sound region of the north-central Gulf of Mexico from 1986 to 2003. Marine Mammal Science 22(3):654–666. doi: 10.1111/j.1748-7692.2006.00057.x

Maze, K.S. and B. Würsig. 1999. Bottlenosed dolphins of San Luis Pass, Texas: Occurrence patterns, site-fidelity, and habitat use. Aquatic Mammals 25:91–104.

McCormack, M.A., F. Battaglia, W.E. McFee, and J. Dutton. 2020. Mercury concentrations in blubber and skin from stranded bottlenose dolphins (*Tursiops truncatus*) along the Florida and Louisiana coasts (Gulf of Mexico, USA) in relation to biological variables. Environmental Research 180:108886. doi: 10.1016/j.envres.2019.108886

McCormack, M.A., B.P. Jackson, and J. Dutton. 2020. Relationship between mercury and selenium concentrations in tissues from stranded odontocetes in the northern Gulf of Mexico. Science of the Total Environment 749:141350. doi: 10.1016/j.scitotenv.2020.141350

McCormack, M.A., W.H. Nowlin, and J. Dutton. 2022. Effect of trophic position on mercury concentrations in bottlenose dolphins (*Tursiops truncatus*) from the northern Gulf of Mexico. Environmental Research 204(Pt B):112124. doi: 10.1016/j.envres.2021.112124

McDonald, T.L., F.E. Hornsby, T.R. Speakman, E.S. Zolman, K.D. Mullin, C. Sinclair, P.E. Rosel, L. Thomas, and L.H. Schwacke. 2017. Survival, density, and abundance of common bottlenose dolphins in

Barataria Bay (USA) following the Deepwater Horizon oil spill. Endangered Species Research 33:193–209. doi: 10.3354/esr00806

McHugh, M.B. 1989. Population numbers and feeding behavior of the Atlantic bottlenose dolphin (*Tursiops truncatus*) near Aransas Pass, Texas. University of Texas at Austin. Thesis. 96 p.

Mead, J.G. and C.W. Potter. 1990. Natural history of bottlenose dolphins along the central Atlantic coast of the United States. In: Leatherwood, S. and R.R. Reeves (Eds.), The Bottlenose Dolphin. Academic Press, San Diego, CA. pp. 165–195.

Miller, C.E. 2003. Abundance trends and environmental habitat usage patterns of bottlenose dolphins (*Tursiops truncatus*) in lower Barataria and Caminada bays, Louisiana. Louisiana State University and Agricultural & Mechanical College. Dissertation. 125 p.

Miller, D.L., V. Woshner, E.L. Styer, S. Ferguson, K.K. Knott, M.J. Gray, R.S. Wells, and T.M. O'Hara. 2011. Histologic findings in free-ranging Sarasota Bay bottlenose dolphin (*Tursiops truncatus*) skin: mercury, selenium, and seasonal factors. Journal of Wildlife Diseases 47(4):1012–1018. doi: 10.7589/0090-3558-47.4.1012

Miller, L.J., A.D. Mackey, T. Hoffland, M. Solangi, and S.A. Kuczaj. 2010. Potential effects of a major hurricane on Atlantic bottlenose dolphin (*Tursiops truncatus*) reproduction in the Mississippi Sound. Marine Mammal Science 26(3):707–715. doi: 10.1111/j.1748-7692.2010.00371.x

Miller, L.J., A.D. Mackey, M. Solangi, and S.A. Kuczaj. 2013. Population abundance and habitat utilization of bottlenose dolphins in the Mississippi Sound. Aquatic Conservation: Marine and Freshwater Ecosystems 23(1):145–151. doi: 10.1002/aqc.2278

Mullin, K.D., T. McDonald, R.S. Wells, B.C. Balmer, T. Speakman, C. Sinclair, E.S. Zolman, F. Hornsby, S.M. McBride, K.A. Wilkinson, and L.H. Schwacke. 2017. Density, abundance, survival, and ranging patterns of common bottlenose dolphins (*Tursiops truncatus*) in Mississippi Sound following the Deepwater Horizon oil spill. PLoS ONE 12(10):e0186265. doi: 10.1371/journal.pone.0186265

Perrin, W.F. and S.B. Reilly. 1984. Reproductive parameters of dolphins and small whales of the family Delphinidae. In: Perrin, W.F., R.L. Brownell, Jr., and D.P. DeMaster (Eds.), Reproduction in Whales, Dolphins, and Porpoises. pp. 97–134.

Rosel, P.E., L.A. Wilcox, C. Sinclair, T.R. Speakman, M.C. Tumlin, J.A. Litz, and E.S. Zolman. 2017. Genetic assignment to stock of stranded common bottlenose dolphins in southeastern Louisiana after the Deepwater Horizon oil spill. Endangered Species Research 33:221–234. doi: 10.3354/esr00780

Rossman, S., P.H. Ostrom, M. Stolen, N.B. Barros, H. Gandhi, C.A. Stricker, and R.S. Wells. 2015. Individual specialization in the foraging habits of female bottlenose dolphins living in a trophically diverse and habitat rich estuary. Oecologia 178(2):415–425. doi: 10.1007/s00442-015-3241-6

Scott, M.D. and S.J. Chivers. 1990. Distribution and herd structure of bottlenose dolphins in the eastern tropical Pacific Ocean. In: Leatherwood, S. and R.R. Reeves (Eds.), The Bottlenose Dolphin. Academic Press, San Diego, CA. pp. 387–402.

Sellas, A.B., R.S. Wells, and P.E. Rosel. 2005. Mitochondrial and nuclear DNA analyses reveal fine scale geographic structure in bottlenose dolphins (*Tursiops truncatus*) in the Gulf of Mexico. Conservation Genetics 6(5):715–728. doi: 10.1007/s10592-005-9031-7

Sergeant, D.E., D.K. Caldwell, and M.C. Caldwell. 1973. Age, growth, and maturity of bottlenosed dolphin (*Tursiops-truncatus*) from northeast Florida. Journal of the Fisheries Research Board of Canada 30(7):1009–1011. doi: 10.1139/f73-165

Shane, S.H. 1990. Behavior and ecology of the bottlenose dolphin at Sanibel Island, Florida. In: Leatherwood, S. and R.R. Reeves (Eds.), The Bottlenose Dolphin. Academic Press, San Diego, CA. pp. 245–265. doi: 10.1016/B978-0-12-440280-5.50016-0

Shane, S.H. 2004. Residence patterns, group characteristics, and association patterns of bottlenose dolphins near Sanibel Island, Florida. Gulf of Mexico Science 22(1):1–12. doi: 10.18785/goms.2201.01

Shane, S.H., R.S. Wells, and B. Würsig. 1986. Ecology, behavior and social organization of the bottlenose dolphin: A review. Marine Mammal Science 2:34–63. doi: 10.1111/j.1748-7692.1986.tb00026.x

Takeshita, R., B.C. Balmer, F. Messina, E.S. Zolman, L. Thomas, R.S. Wells, C.R. Smith, T.K. Rowles, and L.H. Schwacke. 2021. High site-fidelity in common bottlenose dolphins despite low salinity exposure and associated indicators of compromised health. PLoS ONE 16(9):e0258031. doi: 10.1371/journal.pone.0258031

Taylor, B.L., S.J. Chivers, J. Larese, and W.F. Perrin. 2007. Generation length and percent mature estimates for IUCN assessments of cetaceans. National Oceanic and Atmospheric Administration, National Marine Fisheries Service, Southwest Fisheries Science Center: La Jolla, CA. Administrative Report LJ-07-01. 24 p.

Toms, C. 2019. Filling the gaps: Common bottlenose dolphin (*Tursiops truncatus*) population dynamics, structure, and connectivity within Florida panhandle bays, sounds, and estuaries. University of Central Florida. Dissertation. 373 p.

Torres, L.G. and A.J. Read. 2009. Where to catch a fish? The influence of foraging tactics on the ecology of bottlenose dolphins (*Tursiops truncatus*) in Florida Bay, Florida. Marine Mammal Science 25(4):797–815. doi: 10.1111/j.1748-7692.2009.00297.x

Tyson, R.B., S.M. Nowacek, and D.P. Nowacek. 2011. Community structure and abundance of bottlenose dolphins *Tursiops truncatus* in coastal waters of the northeast Gulf of Mexico. Marine Ecology Progress Series 438:253–265. doi: 10.3354/meps09292

Urian, K.W., S. Hofmann, R.S. Wells, and A.J. Read. 2009. Fine-scale population structure of bottlenose dolphins (*Tursiops truncatus*) in Tampa Bay, Florida. Marine Mammal Science 25(3):619–638. doi: 10.1111/j.1748-7692.2009.00284.x

Vail, C.S. 2016. An overview of increasing incidents of bottlenose dolphin harassment in the Gulf of Mexico and possible solutions. Frontiers in Marine Science 3:110. doi: 10.3389/fmars.2016.00110

Vollmer, N.L. and P.E. Rosel. 2013. A review of common bottlenose dolphins (*Tursiops truncatus truncatus*) in the Northern Gulf of Mexico: Population biology, potential threats, and management. Southeastern Naturalist 12(m6):1–43. doi: 10.1656/058.012.m601

Weller, D.W. 1998. Global and regional variation in the biology and behavior of bottlenose dolphins. Texas A&M University. Dissertation. 142 p.

Wells, R.S. 1986a. Population structure of bottlenose dolphins: Behavioral studies along the central west coast of Florida. Contract Report to National Marine Fisheries Service, Southeast Fisheries Center. Contract No. 45-WCNF-5-003BB. 58 p.

Wells, R.S. 1986b. Structural aspects of dolphin societies. University of California, Santa Cruz. Dissertation. 234 p.

Wells, R.S. 1991. The role of long-term study in understanding the social structure of a bottlenose dolphin community. In: Pryor, K. and K. Norris (Eds.), Dolphin Societies: Discoveries and Puzzles. University of California Press, Berkeley, CA. pp. 199–226.

Wells, R.S. 2000. Reproduction in wild bottlenose dolphins: Overview of patterns observed during a long-term study. In: Duffield, D. and T. Robeck (Eds.), Bottlenose Dolphin Reproduction Workshop. AZA Marine Mammal Taxon Advisory Group, Silver Spring, MD. pp. 57–74.

Wells, R.S. 2003. Dolphin social complexity: Lessons from long-term study and life history. In: de Waal, F.B.M. and P.L. Tyack (Eds.), Animal Social Complexity: Intelligence, Culture, and Individualized Societies Harvard University Press, Cambridge, MA. pp. 32–56.

Wells, R.S. 2014. Social structure and life history of bottlenose dolphins near Sarasota Bay, Florida: Insights from four decades and five generations. Primates and cetaceans. pp. 149–172. doi: 10.1007/978-4-431-54523-1_8

Wells, R.S., M.K. Bassos, K.W. Urian, W.J. Carr, and M.D. Scott. 1996. Low-level monitoring of bottlenose dolphins, *Tursiops truncatus*, in Charlotte Harbor, Florida: 1990-1994. National Oceanic and Atmospheric Administration, National Marine Fisheries Service, Southeast Fisheries Science Center: Miami, FL. NOAA Technical Memorandum NMFS-SEFSC-384. 86 p.

Wells, R.S., M.K. Bassos, K.W. Urian, S.H. Shane, E.C.G. Owen, C.F. Weiss, W.J. Carr, and M.D. Scott. 1997. Low-level monitoring of bottlenose dolphins, *Tursiops truncatus*, in Pine Island Sound, Florida: 1996. National Oceanic and Atmospheric Administration, National Marine Fisheries Service, Southeast Fisheries Science Center: Miami, FL. Southeast Fisheries Center Contribution No. 40-WCNF601958. 86 p.

Wells, R.S., A. Natoli, and G. Braulik. 2019. *Tursiops truncatus* (errata version published in 2019). *The IUCN Red List of Threatened Species* 2019: e.T22563A156932432. https://dx.doi.org/10.2305/IUCN.UK.2019-1.RLTS.T22563A156932432.en

Wells, R.S., L.H. Schwacke, T.K. Rowles, B.C. Balmer, E. Zolman, T. Speakman, F.I. Townsend, M.C. Tumlin, A. Barleycorn, and K.A. Wilkinson. 2017. Ranging patterns of common bottlenose dolphins *Tursiops truncatus* in Barataria Bay, Louisiana, following the Deepwater Horizon oil spill. Endangered Species Research 33:159–180. doi: 10.3354/esr00732

Wells, R.S. and M.D. Scott. 1999. Bottlenose dolphin *Tursiops truncatus* (Montagu, 1821). In: Ridgway, S.H. and R. Harrison (Eds.), Handbook of marine mammals. Volume 6: The second book of dolphins and the porpoises. Academic Press, San Diego, CA. pp. 137–182.

Wells, R.S. and M.D. Scott. 2018. Bottlenose dolphin, *Tursiops truncatus*, common bottlenose dolphin. In: Würsig, B., J.G.M. Thewissen, and K.M. Kovacs (Eds.), Encyclopedia of Marine Mammals (Third Edition). Academic Press, London, UK. pp. 118–125. doi: 10.1016/B978-0-12-804327-1.00072-8

Wells, R.S., M.D. Scott, and A.B. Irvine. 1987. The social structure of free-ranging bottlenose dolphins. In: Genoways, H.H. (Ed.), Current Mammalogy. Plenum Press, New York, NY. pp. 247–305.

Wells, R.S., K.W. Urian, A.J. Read, M.K. Bassos, W.J. Carr, and M.D. Scott. 1996. Low-level monitoring of bottlenose dolphins, *Tursiops truncatus*, in Tampa Bay, Florida: 1988-1993. National Oceanic and Atmospheric Administration, National Marine Fisheries Service, Southeast Fisheries Science Center: Miami, FL. NOAA Technical Memorandum NMFS-SEFSC-385. 36 p.

Wilson, R.M., J.R. Kucklick, B.C. Balmer, R.S. Wells, J.P. Chanton, and D.P. Nowacek. 2012. Spatial distribution of bottlenose dolphins (*Tursiops truncatus*) inferred from stable isotopes and priority organic pollutants. Science of the Total Environment 425:223–230. doi: 10.1016/j.scitotenv.2012.02.030

Wilson, R.M., R.B. Tyson, J.A. Nelson, B.C. Balmer, J.P. Chanton, and D.P. Nowacek. 2017. Niche differentiation and prey selectivity among common bottlenose dolphins (*Tursiops truncatus*) sighted in St. George Sound, Gulf of Mexico. Frontiers in Marine Science 4:235. doi: 10.3389/fmars.2017.00235

Woshner, V., K. Knott, R. Wells, C. Willetto, R. Swor, and T. O'Hara. 2008. Mercury and selenium in blood and epidermis of bottlenose dolphins (*Tursiops truncatus*) from Sarasota Bay, FL: interaction and relevance to life history and hematologic parameters. Ecohealth 5(3):360–370. doi: 10.1007/s10393-008-0164-2

Yordy, J.E., M.A. Mollenhauer, R.M. Wilson, R.S. Wells, A. Hohn, J. Sweeney, L.H. Schwacke, T.K. Rowles, J.R. Kucklick, and M.M. Peden-Adams. 2010a. Complex contaminant exposure in cetaceans: a comparative E-Screen analysis of bottlenose dolphin blubber and mixtures of four persistent

organic pollutants. Environmental Toxicology and Chemistry 29(10):2143–2153. doi: 10.1002/etc.279

Yordy, J.E., D.A. Pabst, W.A. McLellan, R.S. Wells, T.K. Rowles, and J.R. Kucklick. 2010b. Tissue-specific distribution and whole-body burden estimates of persistent organic pollutants in the bottlenose dolphin (*Tursiops truncatus*). Environmental Toxicology and Chemistry 29(6):1263–1273. doi: 10.1002/etc.152

Yordy, J.E., R.S. Wells, B.C. Balmer, L.H. Schwacke, T.K. Rowles, and J.R. Kucklick. 2010c. Life history as a source of variation for persistent organic pollutant (POP) patterns in a community of common bottlenose dolphins (*Tursiops truncatus*) resident to Sarasota Bay, FL. Science of the Total Environment 408(9):2163–2172. doi: 10.1016/j.scitotenv.2010.01.032

Common bottlenose dolphin − *Tursiops truncatus*

GoMx BSE/Barataria Bay Stock

Overall Vulnerability Rank = Very High

Biological Sensitivity = Very High

Climate Exposure = Very High

Data Quality = 96% of scores ≥ 2

	Tursiops truncatus	Expert Scores	Data Quality	Expert Scores Plots (Portion by Category)
Sensitivity attributes	Prey/Diet Specificity	1.8	2.7	
	Habitat Specificity	3.8	3.0	
	Site Fidelity	3.8	3.0	
	Lifetime Reproductive Potential	1.7	2.0	
	Generation Length	2.9	2.3	
	Reproductive Plasticity	1.2	2.0	
	Migration	3.9	3.0	
	Home Range	3.5	3.0	
	Stock Abundance	2.3	3.0	
	Stock Abundance Trend	3.3	1.7	
	Cumulative Stressors	3.5	3.0	
	Sensitivity Score	Very High		
Exposure variables	Sea Surface Temperature (Change in mean)	4.0	3.0	
	Sea Surface Temperature (Change in variability)	1.0	3.0	
	Air Temperature (Change in mean)	4.0	3.0	
	Air Temperature (Change in variability)	1.0	3.0	
	Precipitation (Change in mean)	1.0	3.0	
	Precipitation (Change in variability)	1.0	3.0	
	Sea Surface Salinity (Change in mean)	1.3	3.0	
	Sea Surface Salinity (Change in variability)	1.0	3.0	
	Ocean pH (Change in mean)	4.0	3.0	
	Ocean pH (Change in variability)	1.0	3.0	
	Sea ice coverage (Change in mean)	1.0	3.0	
	Sea ice coverage (Change in variability)	1.0	3.0	
	Dissolved oxygen (Change in mean)	4.0	3.0	
	Dissolved oxygen (Change in variability)	1.0	3.0	
	Circulation	4.0	2.3	
	Sea level rise	4.0	3.0	
	Exposure Score	Very High		
Overall Vulnerability Rank		Very High		

Low
Moderate
High
Very High

Common bottlenose dolphin (GoMx BSE/Barataria Bay Stock)

Tursiops truncatus

CVA Results Summary

Overall Climate Vulnerability Rank: Very High (100% certainty from bootstrap analysis).

Climate Exposure: Very High. Six exposure factors scored greater than 3.0:Air Temperature (Standard anomaly) (4.00), Circulation (4.00), Dissolved oxygen (Standard anomaly) (4.00), Ocean pH (Standard anomaly) (4.00), Sea level rise (4.00), and Sea Surface Temperature (Standard anomaly) (4.00).

Biological Sensitivity: Very High. Six sensitivity attributes scored greater than 3.0: Migration (3.93), Habitat Specificity (3.80), Site Fidelity (3.80), Cumulative Stressors (3.53), Home Range (3.47), and Species Abundance Trend (3.33)

Distributional Response: Very High

Abundance Response: Very High

Phenology Response: Very High

Data Quality: 96% of the data quality scores were 2 or greater, 91% of sensitivity attribute data quality scores were 2 or higher.

Background Information

Stock Description

This stock includes common bottlenose dolphins in Caminada Bay, Barataria Bay east to Bastian Bay, Bay Coquette, and Gulf coastal waters extending 1 km from the shoreline (Blaylock et al. 1994; Hayes et al. 2022).

Prey/Diet Specificity

Generally, common bottlenose dolphins consume a large variety of fish and/or squid (Barros and Odell 1990; Mead and Potter 1990; Wells and Scott 2018). Common bottlenose dolphins seem to show a consistent preference for sound-producing fishes (e.g., sciaenids, toadfish; Berens McCabe et al. 2010; Bowen-Stevens et al. 2021). In Sarasota Bay, FL, Rossman et al. (2015) found some individual common bottlenose dolphins to show diet specialization despite the broader study group showing a generalist diet. Specialization can be influenced by locally abundant prey species (Shane et al. 1986; Vollmer and Rosel 2013) or can result from resource partitioning amongst sympatric populations (e.g., Torres and Read 2009; Wilson et al. 2017).

Habitat Specificity

Common bottlenose dolphins are found in a variety of marine and estuarine habitats (Wells and Scott 2018). Within Barataria Bay, common bottlenose dolphins are frequently found in areas of higher

salinity (>~11ppt) and tend to avoid areas of lower salinity (<~5ppt; Hornsby et al. 2017) and foraging habitat has been characterized by depth, distance from shore, and salinity (Miller and Baltz 2010).

Site Fidelity

Strong multi-year, year-round site fidelity has been demonstrated for this stock (Wells et al. 2017).

Other Gulf of Mexico bay, sound, and estuary (BSE) stocks show site fidelity (LaBrecque et al. 2015; Hayes et al. 2022), such as in Matagorda Bay, Espiritu Santo Bay, and San Luis Pass in Texas (Maze and Würsig 1999; Lynn and Würsig 2002; Irwin and Würsig 2004); Barataria Bay in Louisiana (Miller 2003; Wells et al. 2017; Takeshita et al. 2021); Mississippi Sound in Mississippi (Hubard et al. 2004; Mackey 2010; Mullin et al. 2017); Charlotte Harbor and Pine Island Sound (Bassos-Hull et al. 2013), Sarasota Bay (Wells 1986a; Wells 1991; Wells 2014), St. Andrew Bay (Balmer et al. 2019), St. Joseph Bay (Balmer et al. 2008; B. Balmer et al. 2018), St. Vincent Sound and Apalachicola Bay (Tyson et al. 2011), and Tampa Bay in Florida (Wells et al. 1996b; Urian et al. 2009).

Lifetime Reproductive Potential

Taylor et al. (2007) reported the interbirth interval of common bottlenose dolphins as 3.80 years, though other studies have shown it to be closer to three years (Leatherwood 1977; Wells and Scott 1999; Connor et al. 2000; Wells 2003).

Generally, females reach sexual maturity before males, with females reaching sexual maturity at 5–13 years (Sergeant et al. 1973; Perrin and Reilly 1984; Wells et al. 1987; Mead and Potter 1990; Mann et al. 2000; Wells 2000; Wells and Scott 2018).

Females reproduce through most of their adult lives (Leatherwood 1977; Hohn et al. 1989; Wells and Scott 1999; Wells 2000; Wells 2003), but may produce fewer calves after they reach 30 years of age (Wells 2000). Sarasota Bay common bottlenose dolphins can give birth when they are as young as 6 years of age, and continue until they are 48 years old (Wells and Scott 2018). Reproductive lifespan would therefore be up to 42 years based on long-term observation studies (Wells 2014; Wells and Scott 2018).

Longitudinal studies from Sarasota Bay, Florida have found that female common bottlenose dolphins can live up to 67 years (Wells and Scott 2018). Studies based on growth layer groups in teeth suggest female common bottlenose dolphins can live to 41 years along the Texas coast (Fernandez and Hohn 1998) and to 30 years along the Mississippi coast (Mattson et al. 2006).

Generation Length

Taylor et al. (2007) reported common bottlenose dolphin generation length of 20.6-21.1 years.

Reproductive Plasticity

Common bottlenose dolphin calving is generally diffusely seasonal, with one or more peaks, although births have been reported from all seasons (Vollmer and Rosel 2013; Wells and Scott 2018). Calf sightings peak in the spring and summer months in Florida (Wells et al. 1987), Mississippi/Louisiana (Miller et al. 2010, 2013), and Texas (McHugh 1989; Henderson et al. 2007).

Migration

The bay, sound, and estuary stocks in the Gulf of Mexico include transient, seasonally migratory, and stable resident communities (LaBrecque et al. 2015; Hayes et al. 2022). Molecular genetic data support that some BSE stocks are genetically distinct from one another, and furthermore are differentiated from adjacent coastal populations (e.g., Sellas et al. 2005; Rosel et al. 2017). For example, Toms (2019) found fine-scale genetic population structure among five inshore systems in the Florida Panhandle and variable migration rates among populations.

Year-round residency has been reported for common bottlenose dolphins (Hayes et al. 2022), such as in Louisiana/Mississippi (Hubard et al. 2004; Wells et al. 2017; Takeshita et al. 2021), Texas (Bräger 1993; Bräger et al. 1994; Fertl 1994; Irwin and Würsig 2004; Lynn and Würsig 2002; Maze and Würsig 1999; Shane 1990; Shane 2004; Weller 1998), and Florida (Balmer et al. 2008; Bassos-Hull et al. 2013; Irvine and Wells 1972; Irvine et al. 1981; Scott et al. 1990; Tyson et al. 2011; Urian et al. 2009; Wells 1986a; Wells 1991; Wells 2003; Wells et al. 1987; Wells et al. 1996a; Wells et al. 1996b; Wells et al. 1997). Stable isotope data suggest little movement of this population between the estuary and the nearshore environment (Cloyed et al. 2021c). Satellite tag data suggest few long-range movements outside of the bay (Lane et al. 2015).

Home Range

The home ranges of individuals within the Gulf of Mexico BSE stocks are considered to generally include the areas within the stock boundaries and brief excursions beyond the stock boundaries (Irvine et al. 1981; Wells 1986a; Wells 1986b; Wells 1991; Wells et al. 1996a; Wells et al. 1996b; Balmer et al. 2008; Wells 2014), although some individuals show preference for small areas within the stock boundaries (Lynn and Würsig 2002; Cloyed et al. 2021a, 2021b).

Stock Abundance

The best available abundance estimate for this stock is 2,071 individuals (95% CI: 1,832–2,309; CV=0.06) based on vessel-based capture-recapture photo-ID surveys conducted from March and April 2019 (Garrison et al. 2020; Hayes et al. 2022). Previously, this stock abundance was estimated at 2,306 individuals (95% CI: 2,014–2,603; CV=0.09) based on vessel-based capture-recapture photo-ID surveys conducted from June 2010 to May 2014 (McDonald et al. 2017).

Stock Abundance Trend

The data are insufficient to determine population trends for most of the Gulf of Mexico bay, sound, and estuary common bottlenose dolphin stocks (Hayes et al. 2022).

Cumulative Stressors

Common bottlenose dolphins face a wide range of individual and cumulative natural and anthropogenic threats (see reviews by Vollmer and Rosel 2013; Wells and Scott 2018; and Wells et al. 2019). These include such threats as shark predation, harmful algal blooms, recreational and commercial fishing gear, vessel traffic, pollution, habitat alteration, and provisioning.

Common bottlenose dolphins in Barataria Bay were exposed to some of the highest levels of oil and dispersants following the Deepwater Horizon oil spill, which may result in long term reproductive and chronic health effects (NOAA 2011; Michel et al. 2013; Helm et al. 2015; DWH NRDA 2016; Lane et al. 2015; De Guise et al. 2017, 2021; Kellar et al. 2017; Smith et al. 2017; Schwacke et al. 2017). An Unusual Mortality Event (UME), involving primarily bottlenose dolphins, was declared for cetaceans in the northern Gulf of Mexico from 2010 to 2014, with the Deepwater Horizon oil spill as the primary underlying cause (Litz et al. 2014; Schwacke et al. 2014; Venn-Watson et al. 2015a, 2015b).

The nearshore and estuarine habitats along the Gulf of Mexico coast include waters adjacent to areas of high human population, some of which are highly industrialized, and areas of heavy agricultural impact. Studies of bottlenose dolphin tissues from Barataria Bay have shown persistent organic pollutant (POP) in blubber and blood samples, though at concentrations lower than other southeastern U.S. sites (Kucklick et al. 2011; Balmer et al. 2015; J. Balmer et al. 2018). Similarly, mercury has been detected in common bottlenose dolphin tissues in Louisiana waters, but in lower concentrations than common bottlenose dolphins found in Florida waters (McCormack et al. 2020a, 2020b, 2022).

Dolphin morbillivirus has been detected in populations of the northern Gulf of Mexico, but has not been attributed to large scale die-offs since 1993−1994 (Lipscomb et al. 1996; Litz et al. 2014; Van Bressem et al. 2014; Fauquier et al. 2017). A UME was also declared in 1990 for the Gulf of Mexico from Florida to Texas, with more than 300 common bottlenose dolphins stranding due to an undetermined cause, although morbillivirus and cold temperatures may have contributed (Litz et al. 2014).

Distribution and Sightings

Sighting maps for this stock are presented by Mullin et al. (2018).

Further Reading

Hayes, S.A., E. Josephson, K. Maze-Foley, P.E. Rosel, and J. Wallace. 2022. U.S. Atlantic and Gulf of Mexico marine mammal stock assessments 2021. National Oceanic and Atmospheric Administration, National Marine Fisheries Service, Northeast Fisheries Science Center: Woods Hole, MA. NOAA Technical Memorandum NMFS-NE-288. 380 p. doi: 10.25923/6tt7-kc16

Wells, R.S. and M.D. Scott. 2018. Bottlenose Dolphin, *Tursiops truncatus*, Common Bottlenose Dolphin. In: Würsig, B., J.G.M. Thewissen, and K.M. Kovacs (Eds.), Encyclopedia of Marine Mammals (Third Edition). Academic Press, London, UK. pp. 118–125. doi: 10.1016/B978-0-12-804327-1.00072-8

Wells, R.S., A. Natoli, and G. Braulik. 2019. *Tursiops truncatus* (errata version published in 2019). The IUCN Red List of Threatened Species 2019: e.T22563A156932432. https://dx.doi.org/10.2305/IUCN.UK.2019-1.RLTS.T22563A156932432.en

References

Balmer, B., T. McDonald, F. Hornsby, J. Adams, J. Allen, A. Barleycorn, and L. Schwacke. 2018. Long-term trends in a northern Gulf of Mexico common bottlenose dolphin (*Tursiops truncatus*) population in the wake of the Deepwater Horizon oil spill. Journal of Cetacean Research and Management 18:1–9. doi: 10.47536/jcrm.v18i1.409

Balmer, B., S. Watwood, B. Quigley, T. Speakman, K. Barry, K. Mullin, P. Rosel, C. Sinclair, E. Zolman, and L. Schwacke. 2019. Common bottlenose dolphin (*Tursiops truncatus*) abundance and distribution

patterns in St. Andrew Bay, Florida, USA. Aquatic Conservation: Marine and Freshwater Ecosystems 29(3):486–498. doi: 10.1002/aqc.3001

Balmer, B.C., R.S. Wells, S.M. Nowacek, D.P. Nowacek, L.H. Schwacke, W.A. McLellan, F.S. Scharf, T.K. Rowles, L.J. Hansen, T.R. Spradlin, and D.A. Pabst. 2008. Seasonal abundance and distribution patterns of common bottlenose dolphins (*Tursiops truncatus*) near St. Joseph Bay, Florida, USA. Journal of Cetacean Research and Management 10(2):157–167.

Balmer, B.C., G.M. Ylitalo, L.E. McGeorge, K.A. Baugh, D. Boyd, K.D. Mullin, P.E. Rosel, C. Sinclair, R.S. Wells, E.S. Zolman, and L.H. Schwacke. 2015. Persistent organic pollutants (POPs) in blubber of common bottlenose dolphins (*Tursiops truncatus*) along the northern Gulf of Mexico coast, USA. Science of the Total Environment 527-528:306–312. doi: 10.1016/j.scitotenv.2015.05.016

Balmer, J.E., G.M. Ylitalo, T.K. Rowles, K.D. Mullin, R.S. Wells, F.I. Townsend, R.W. Pearce, J.L. Bolton, E.S. Zolman, B.C. Balmer, and L.H. Schwacke. 2018. Persistent organic pollutants (POPs) in blood and blubber of common bottlenose dolphins (*Tursiops truncatus*) at three northern Gulf of Mexico sites following the Deepwater Horizon oil spill. Science of the Total Environment 621:130–137. doi: 10.1016/j.scitotenv.2017.11.209

Barros, N.B. and D.K. Odell. 1990. Food habits of bottlenose dolphins in the southeastern United States In: Leatherwood, S. and R.R. Reeves (Eds.), The Bottlenose Dolphin. Academic Press, San Diego, CA. pp. 309–328. doi: 10.1016/B978-0-12-440280-5.50020-2

Bassos-Hull, K., R.M. Perrtree, C.C. Shepard, S. Schilling, A.A. Barleycorn, J.B. Allen, B.C. Balmer, W.E. Pine, and R.S. Wells. 2013. Long-term site fidelity and seasonal abundance estimates of common bottlenose dolphins (*Tursiops truncatus*) along the southwest coast of Florida and responses to natural perturbations. Journal of Cetacean Research and Management 13(1):19–30. doi: 10.47536/jcrm.v13i1.551

Berens McCabe, E.J., D.P. Gannon, N.B. Barros, and R.S. Wells. 2010. Prey selection by resident common bottlenose dolphins (*Tursiops truncatus*) in Sarasota Bay, Florida. Marine Biology 157(5):931–942. doi: 10.1007/s00227-009-1371-2

Blaylock, R.A. and W. Hoggard. 1994. Preliminary estimates of bottlenose dolphin abundance in southern U.S. Atlantic and Gulf of Mexico continental shelf waters. National Oceanic and Atmospheric Administration, National Marine Fisheries Service, Southeast Fisheries Science Center: Miami, FL. NOAA Technical Memorandum NMFS-SEFSC-356. 10 p.

Bowen-Stevens, S.R., D.P. Gannon, R.A. Hazelkorn, G. Lovewell, K.M. Volker, S. Smith, M.C. Tumlin, and J. Litz. 2021. Diet of common bottlenose dolphins, *Tursiops truncatus*, that stranded in and near Barataria Bay, Louisiana, 2010–2012. Southeastern Naturalist 20(1). doi: 10.1656/058.020.0113

Bräger, S. 1993. Diurnal and seasonal behavior patterns of bottlenose dolphins (*Tursiops truncatus*). Marine Mammal Science 9:434–438. doi: 10.1111/j.1748-7692.1993.tb00477.x

Bräger, S., B. Würsig, A. Acevedo, and T. Henningsen. 1994. Association patterns of bottle-nosed dolphins (*Tursiops-truncatus*) in Galveston Bay, Texas. Journal of Mammalogy 75(2):431–437. doi: 10.2307/1382564

Cloyed, C.S., B.C. Balmer, L.H. Schwacke, R. Takeshita, A. Hohn, R.S. Wells, T.K. Rowles, J.T. Saliki, C.R. Smith, M.C. Tumlin, E.S. Zolman, D.A. Fauquier, and R.H. Carmichael. 2021a. Linking morbillivirus exposure to individual habitat use of common bottlenose dolphins (*Tursiops truncatus*) between geographically different sites. Journal of Animal Ecology 90(5):1191–1204. doi: 10.1111/1365-2656.13446

Cloyed, C.S., B.C. Balmer, L.H. Schwacke, R.S. Wells, E.J. Berens McCabe, A.A. Barleycorn, J.B. Allen, T.K. Rowles, C.R. Smith, R. Takeshita, F.I. Townsend, M.C. Tumlin, E.S. Zolman, and R.H. Carmichael. 2021b. Interaction between dietary and habitat niche breadth influences cetacean vulnerability to environmental disturbance. Ecosphere 12(9):e03759. doi: 10.1002/ecs2.3759

Cloyed, C.S., R.M. Wilson, B.C. Balmer, A.A. Hohn, L.H. Schwacke, E.S. Zolman, M.C. Tumlin, R.S. Wells, A.A. Barleycorn, J.B. Allen, and R.H. Carmichael. 2021c. Specialization of a mobile, apex predator affects trophic coupling among adjacent habitats. Scientific Reports 11(1):19611. doi: 10.1038/s41598-021-99017-1

Connor, R.C., R.S. Wells, J. Mann, and A.J. Read. 2000. The bottlenose dolphin: Social relationships in a fission-fusion society. In: Mann, J., R. Connor, P. Tyack, and H. Whitehead (Eds.), Cetacean Societies: Field Studies of Dolphins and Whales. University of Chicago Press, Chicago, IL. pp. 91–126.

De Guise, S., M. Levin, E. Gebhard, L. Jasperse, L. Burdett Hart, C.R. Smith, S. Venn-Watson, F. Townsend, R. Wells, B. Balmer, E. Zolman, T. Rowles, and L. Schwacke. 2017. Changes in immune functions in bottlenose dolphins in the northern Gulf of Mexico associated with the Deepwater Horizon oil spill. Endangered Species Research 33:291–303. doi: 10.3354/esr00814

De Guise, S., M. Levin, L. Jasperse, J. Herrman, R.S. Wells, T. Rowles, and L. Schwacke. 2021. Long-term immunological alterations in bottlenose dolphin a decade after the Deepwater Horizon oil spill in the northern Gulf of Mexico: Potential for multigenerational effects. Environmental Toxicology and Chemistry 40(5):1308–1321. doi: 10.1002/etc.4980

Deepwater Horizon Natural Resource Damage Assessment Trustees (DWH NRDA). 2016. Deepwater Horizon oil spill: Final Programmatic Damage Assessment and Restoration Plan and Final Programmatic Environmental Impact Statement. Retrieved from: http://www.gulfspillrestoration.noaa.gov/restoration-planning/gulf-plan.1659.

Fauquier, D.A., J. Litz, S. Sanchez, K. Colegrove, L.H. Schwacke, L. Hart, J. Saliki, C. Smith, T. Goldstein, S. Bowen-Stevens, W. McFee, E. Fougeres, B. Mase-Guthrie, E. Stratton, R. Ewing, S. Venn-Watson, R.H. Carmichael, C. Clemons-Chevis, W. Hatchett, D. Shannon, S. Shippee, S. Smith, L. Staggs, M.C. Tumlin, N.L. Wingers, and T.K. Rowles. 2017. Evaluation of morbillivirus exposure in cetaceans from the northern Gulf of Mexico 2010-2014. Endangered Species Research 33:211–220. doi: 10.3354/esr00772

Fernandez, S. and A.A. Hohn. 1998. Age, growth, and calving season of bottlenose dolphins, *Tursiops truncatus*, off coastal Texas. Fishery Bulletin 96(2):357–365.

Fertl, D. 1994. Occurrence patterns and behavior of bottlenose dolphins (*Tursiops truncatus*) in the Galveston ship channel, Texas. Texas Journal of Science 46(4):299–318.

Garrison, L.P. and L. Stokes. 2020. Estimated bycatch of marine mammals and sea turtles in the U.S. Atlantic pelagic longline fleet during 2017. National Oceanic and Atmospheric Administration, National Marine Fisheries Service, Southeast Fisheries Science Center: Miami, FL. Southeast Fisheries Science Center Reference Document PRBD 2020-05. 58 p. doi: 10.25923/a3hs-ea60

Hayes, S.A., E. Josephson, K. Maze-Foley, P.E. Rosel, and J. Wallace. 2022. U.S. Atlantic and Gulf of Mexico marine mammal stock assessments 2021. National Oceanic and Atmospheric Administration, National Marine Fisheries Service, Northeast Fisheries Science Center: Woods Hole, MA. NOAA Technical Memorandum NMFS-NE-288. 380 p. doi: 10.25923/6tt7-kc16

Helm, R.C., D.P. Costa, T.D. DeBruyn, T.J. O'Shea, R.S. Wells, and T.M. Williams. 2015. Overview of effects of oil spills on marine mammals. In: Fingas, M. (Ed.), Handbook of oil spill science and technology, First edition. John Wiley & Sons, Inc., Hoboken, NJ. pp. 455–475.

Henderson, E.E. and B. Würsig. 2007. Behavior patterns of bottlenose dolphins in San Luis Pass, Texas. Gulf of Mexico Science 25(2):153–161. doi: 10.18785/goms.2502.06

Hohn, A.A., M.D. Scott, R.S. Wells, J.C. Sweeney, and A.B. Irvine. 1989. Growth layers in teeth from known-age, free-ranging bottlenose dolphins. Marine Mammal Science 5(4):315–342. doi: 10.1111/j.1748-7692.1989.tb00346.x

Hornsby, F.E., T.L. McDonald, B.C. Balmer, T.R. Speakman, K.D. Mullin, P.E. Rosel, R.S. Wells, A.C. Telander, P.W. Marcy, and L.H. Schwacke. 2017. Using salinity to identify common bottlenose

dolphin habitat in Barataria Bay, Louisiana, USA. Endangered Species Research 33:181–192. doi: 10.3354/esr00807

Hubard, C.W., K. Maze-Foley, K.D. Mullin, and W.W. Schroeder. 2004. Seasonal abundance and site fidelity of bottlenose dolphins (*Tursiops truncatus*) in Mississippi Sound. Aquatic Mammals 30(2):299–310. doi: 10.1578/am.30.2.2004.299

Irvine, A.B., M.D. Scott, R.S. Wells, and J.H. Kaufmann. 1981. Movements and activities of the Atlantic bottlenose dolphin, *Tursiops truncatus*, near Sarasota, Florida. Fishery Bulletin 79(4):671–688.

Irvine, B. and R.S. Wells. 1972. Results of attempts to tag Atlantic bottlenosed dolphins (*Tursiops truncatus*). Cetology 13:1–5.

Irwin, L.-J. and B. Würsig. 2004. A small resident community of bottlenose dolphins, *Tursiops truncatus*, in Texas: Monitoring recommendations. Gulf of Mexico Science 22(1):13–21. doi: 10.18785/goms.2201.02

Kellar, N.M., T.R. Speakman, C.R. Smith, S.M. Lane, B.C. Balmer, M.L. Trego, K.N. Catelani, M.N. Robbins, C.D. Allen, R.S. Wells, E.S. Zolman, T.K. Rowles, and L.H. Schwacke. 2017. Low reproductive success rates of common bottlenose dolphins *Tursiops truncatus* in the northern Gulf of Mexico following the Deepwater Horizon disaster (2010-2015). Endangered Species Research 33:143–158. doi: 10.3354/esr00775

Kucklick, J., L. Schwacke, R. Wells, A. Hohn, A. Guichard, J. Yordy, L. Hansen, E. Zolman, R. Wilson, J. Litz, D. Nowacek, T. Rowles, R. Pugh, B. Balmer, C. Sinclair, and P. Rosel. 2011. Bottlenose dolphins as indicators of persistent organic pollutants in the western North Atlantic Ocean and Northern Gulf of Mexico. Environmental Science & Technology 45(10):4270–4277. doi: 10.1021/es1042244

LaBrecque, E., C. Curtice, J. Harrison, S.M. Van Parijs, and P.N. Halpin. 2015. 3. Biologically Important Areas for cetaceans within U.S. Waters – Gulf of Mexico Region. Aquatic Mammals 41(1):30–38. doi: 10.1578/am.41.1.2015.30

Lane, S.M., C.R. Smith, J. Mitchell, B.C. Balmer, K.P. Barry, T. McDonald, C.S. Mori, P.E. Rosel, T.K. Rowles, T.R. Speakman, F.I. Townsend, M.C. Tumlin, R.S. Wells, E.S. Zolman, and L.H. Schwacke. 2015. Reproductive outcome and survival of common bottlenose dolphins sampled in Barataria Bay, Louisiana, USA, following the Deepwater Horizon oil spill. Proceedings of the Royal Society B: Biological Sciences 282(1818):20151944. doi: 10.1098/rspb.2015.1944

Leatherwood, S. 1977. Some preliminary impressions on the numbers and social behavior of free-swimming bottlenosed dolphin calves (*Tursiops truncatus*) in the northern Gulf of Mexico. In: Ridgway, S.H. and K. Bernirschke (Eds.), Breeding Dolphins; Present Status, Suggestions for the Future. Final report to the US Marine Mammal Commission, Contract MM6AC009, MMC-76/07, Washington, DC. pp. 143–167.

Lipscomb, T.P., S. Kennedy, D. Moffett, A. Krafft, B.A. Klaunberg, J.H. Lichy, G.T. Regan, G.A.J. Worthy, and J.K. Taubenberger. 1996. Morbilliviral epizootic in bottlenose dolphins of the Gulf of Mexico. Journal of Veterinary Diagnostic Investigation 8(3):283–290. doi: 10.1177/104063879600800302

Litz, J.A., M.A. Baran, S.R. Bowen-Stevens, R.H. Carmichael, K.M. Colegrove, L.P. Garrison, S.E. Fire, E.M. Fougeres, R. Hardy, S. Holmes, W. Jones, B.E. Mase-Guthrie, D.K. Odell, P.E. Rosel, J.T. Saliki, D.K. Shannon, S.F. Shippee, S.M. Smith, E.M. Stratton, M.C. Tumlin, H.R. Whitehead, G.A. Worthy, and T.K. Rowles. 2014. Review of historical unusual mortality events (UMEs) in the Gulf of Mexico (1990–2009): providing context for the multi-year northern Gulf of Mexico cetacean UME declared in 2010. Diseases of Aquatic Organisms 112(2):161–175. doi: 10.3354/dao02807

Lynn, S.K. and B. Würsig. 2002. Summer movement patterns of bottlenose dolphins in a Texas bay. Gulf of Mexico Science 20(1):25–37. doi: 10.18785/goms.2001.03

Mackey, A.D. 2010. Site fidelity and association patterns of bottlenose dolphins (*Tursiops truncatus*) in the Mississippi Sound. University of Southern Mississippi. Thesis. 133 p.

Mann, J., R.C. Connor, L.M. Barre, and M.R. Heithaus. 2000. Female reproductive success in bottlenose dolphins (*Tursiops* sp.): life history, habitat, provisioning, and group-size effects. Behavioral Ecology 11(2):210–219. doi: 10.1093/beheco/11.2.210

Mattson, M.C., K.D. Mullin, G.W. Ingram, and W. Hoggard. 2006. Age structure and growth of the bottlenose dolphin (*Tursiops truncatus*) from strandings in the Mississippi sound region of the north-central Gulf of Mexico from 1986 to 2003. Marine Mammal Science 22(3):654–666. doi: 10.1111/j.1748-7692.2006.00057.x

Maze, K.S. and B. Würsig. 1999. Bottlenosed dolphins of San Luis Pass, Texas: Occurrence patterns, site-fidelity, and habitat use. Aquatic Mammals 25:91–104.

McCormack, M.A., F. Battaglia, W.E. McFee, and J. Dutton. 2020. Mercury concentrations in blubber and skin from stranded bottlenose dolphins (*Tursiops truncatus*) along the Florida and Louisiana coasts (Gulf of Mexico, USA) in relation to biological variables. Environmental Research 180:108886. doi: 10.1016/j.envres.2019.108886

McCormack, M.A., B.P. Jackson, and J. Dutton. 2020. Relationship between mercury and selenium concentrations in tissues from stranded odontocetes in the northern Gulf of Mexico. Science of the Total Environment 749:141350. doi: 10.1016/j.scitotenv.2020.141350

McCormack, M.A., W.H. Nowlin, and J. Dutton. 2022. Effect of trophic position on mercury concentrations in bottlenose dolphins (*Tursiops truncatus*) from the northern Gulf of Mexico. Environmental Research 204(Pt B):112124. doi: 10.1016/j.envres.2021.112124

McDonald, T.L., F.E. Hornsby, T.R. Speakman, E.S. Zolman, K.D. Mullin, C. Sinclair, P.E. Rosel, L. Thomas, and L.H. Schwacke. 2017. Survival, density, and abundance of common bottlenose dolphins in Barataria Bay (USA) following the Deepwater Horizon oil spill. Endangered Species Research 33:193–209. doi: 10.3354/esr00806

McHugh, M.B. 1989. Population numbers and feeding behavior of the Atlantic bottlenose dolphin (*Tursiops truncatus*) near Aransas Pass, Texas. University of Texas at Austin. Thesis. 96 p.

Mead, J.G. and C.W. Potter. 1990. Natural history of bottlenose dolphins along the central Atlantic coast of the United States. In: Leatherwood, S. and R.R. Reeves (Eds.), The Bottlenose Dolphin. Academic Press, San Diego, CA. pp. 165–195.

Michel, J., E.H. Owens, S. Zengel, A. Graham, Z. Nixon, T. Allard, W. Holton, P.D. Reimer, A. Lamarche, M. White, N. Rutherford, C. Childs, G. Mauseth, G. Challenger, and E. Taylor. 2013. Extent and degree of shoreline oiling: Deepwater Horizon oil spill, Gulf of Mexico, USA. PLoS ONE 8(6):e65087. doi: 10.1371/journal.pone.0065087

Miller, C.E. 2003. Abundance trends and environmental habitat usage patterns of bottlenose dolphins (*Tursiops truncatus*) in lower Barataria and Caminada bays, Louisiana. Louisiana State University and Agricultural & Mechanical College. Dissertation. 125 p.

Miller, C.E. and D.M. Baltz. 2010. Environmental characterization of seasonal trends and foraging habitat of bottlenose dolphins (*Tursiops truncatus*) in northern Gulf of Mexico bays. Fishery Bulletin 108(1):79–86.

Miller, L.J., A.D. Mackey, T. Hoffland, M. Solangi, and S.A. Kuczaj. 2010. Potential effects of a major hurricane on Atlantic bottlenose dolphin (*Tursiops truncatus*) reproduction in the Mississippi Sound. Marine Mammal Science 26(3):707–715. doi: 10.1111/j.1748-7692.2010.00371.x

Miller, L.J., A.D. Mackey, M. Solangi, and S.A. Kuczaj. 2013. Population abundance and habitat utilization of bottlenose dolphins in the Mississippi Sound. Aquatic Conservation: Marine and Freshwater Ecosystems 23(1):145–151. doi: 10.1002/aqc.2278

Mullin, K.D., K. Barry, T. McDonald, J. Morey, B. Quigley, E. Ronje, L. Schwacke, S. Carrie, T. Speakman, and J. Thompson. 2018. Assessment of the overlap of Terrebonne–Timbalier Bay and Bartaria Bay common bottlenose dolphin (*Tursiops truncatus*) stocks based on photo-identification of individual dolphins. National Oceanic and Atmospheric Administration, National Marine

Fisheries Service, Southeast Fisheries Science Center: Pascagoula, MS. NOAA Technical Memorandum NMFS-SEFSC-729. 29 p. doi: 10.25923/8g4y-dg29

Mullin, K.D., T. McDonald, R.S. Wells, B.C. Balmer, T. Speakman, C. Sinclair, E.S. Zolman, F. Hornsby, S.M. McBride, K.A. Wilkinson, and L.H. Schwacke. 2017. Density, abundance, survival, and ranging patterns of common bottlenose dolphins (*Tursiops truncatus*) in Mississippi Sound following the Deepwater Horizon oil spill. PLoS ONE 12(10):e0186265. doi: 10.1371/journal.pone.0186265

National Oceanic and Atmospheric Administration (NOAA). 2011. Public scoping for preparation of a programmatic environmental impact statement for the Deepwater Horizon BP Oil Spill. 9 p.

Perrin, W.F. and S.B. Reilly. 1984. Reproductive parameters of dolphins and small whales of the family Delphinidae. In: Perrin, W.F., R.L. Brownell, Jr., and D.P. DeMaster (Eds.), Reproduction in Whales, Dolphins, and Porpoises. pp. 97–134.

Rosel, P.E., L.A. Wilcox, C. Sinclair, T.R. Speakman, M.C. Tumlin, J.A. Litz, and E.S. Zolman. 2017. Genetic assignment to stock of stranded common bottlenose dolphins in southeastern Louisiana after the Deepwater Horizon oil spill. Endangered Species Research 33:221–234. doi: 10.3354/esr00780

Rossman, S., P.H. Ostrom, M. Stolen, N.B. Barros, H. Gandhi, C.A. Stricker, and R.S. Wells. 2015. Individual specialization in the foraging habits of female bottlenose dolphins living in a trophically diverse and habitat rich estuary. Oecologia 178(2):415–425. doi: 10.1007/s00442-015-3241-6

Schwacke, L.H., C.R. Smith, F.I. Townsend, R.S. Wells, L.B. Hart, B.C. Balmer, T.K. Collier, S. De Guise, M.M. Fry, L.J. Guillette, Jr., S.V. Lamb, S.M. Lane, W.E. McFee, N.J. Place, M.C. Tumlin, G.M. Ylitalo, E.S. Zolman, and T.K. Rowles. 2014. Health of common bottlenose dolphins (*Tursiops truncatus*) in Barataria Bay, Louisiana, following the deepwater horizon oil spill. Environmental Science & Technology 48(1):93–103. doi: 10.1021/es403610f

Schwacke, L.H., L. Thomas, R.S. Wells, W.E. McFee, A.A. Hohn, K.D. Mullin, E.S. Zolman, B.M. Quigley, T.K. Rowles, and J.H. Schwacke. 2017. Quantifying injury to common bottlenose dolphins from the Deepwater Horizon oil spill using an age-, sex- and class-structured population model. Endangered Species Research 33:265–279. doi: 10.3354/esr00777

Scott, M.D. and S.J. Chivers. 1990. Distribution and herd structure of bottlenose dolphins in the eastern tropical Pacific Ocean. In: Leatherwood, S. and R.R. Reeves (Eds.), The Bottlenose Dolphin. Academic Press, San Diego, CA. pp. 387–402.

Sellas, A.B., R.S. Wells, and P.E. Rosel. 2005. Mitochondrial and nuclear DNA analyses reveal fine scale geographic structure in bottlenose dolphins (*Tursiops truncatus*) in the Gulf of Mexico. Conservation Genetics 6(5):715–728. doi: 10.1007/s10592-005-9031-7

Sergeant, D.E., D.K. Caldwell, and M.C. Caldwell. 1973. Age, growth, and maturity of bottlenosed dolphin (*Tursiops-truncatus*) from northeast Florida. Journal of the Fisheries Research Board of Canada 30(7):1009–1011. doi: 10.1139/f73-165

Shane, S.H. 1990. Behavior and ecology of the bottlenose dolphin at Sanibel Island, Florida. In: Leatherwood, S. and R.R. Reeves (Eds.), The Bottlenose Dolphin. Academic Press, San Diego, CA. pp. 245–265. doi: 10.1016/B978-0-12-440280-5.50016-0

Shane, S.H. 2004. Residence patterns, group characteristics, and association patterns of bottlenose dolphins near Sanibel Island, Florida. Gulf of Mexico Science 22(1):1–12. doi: 10.18785/goms.2201.01

Shane, S.H., R.S. Wells, and B. Würsig. 1986. Ecology, behavior and social organization of the bottlenose dolphin: A review. Marine Mammal Science 2:34–63. doi: 10.1111/j.1748-7692.1986.tb00026.x

Smith, C.R., T.K. Rowles, L.B. Hart, F.I. Townsend, R.S. Wells, E.S. Zolman, B.C. Balmer, B. Quigley, M. Ivančić, W. McKercher, M.C. Tumlin, K.D. Mullin, J.D. Adams, Q. Wu, W. McFee, T.K. Collier, and L.H. Schwacke. 2017. Slow recovery of Barataria Bay dolphin health following the Deepwater

Horizon oil spill (2013-2014), with evidence of persistent lung disease and impaired stress response. Endangered Species Research 33:127–142. doi: 10.3354/esr00778

Takeshita, R., B.C. Balmer, F. Messina, E.S. Zolman, L. Thomas, R.S. Wells, C.R. Smith, T.K. Rowles, and L.H. Schwacke. 2021. High site-fidelity in common bottlenose dolphins despite low salinity exposure and associated indicators of compromised health. PLoS ONE 16(9):e0258031. doi: 10.1371/journal.pone.0258031

Taylor, B.L., S.J. Chivers, J. Larese, and W.F. Perrin. 2007. Generation length and percent mature estimates for IUCN assessments of cetaceans. National Oceanic and Atmospheric Administration, National Marine Fisheries Service, Southwest Fisheries Science Center: La Jolla, CA. Administrative Report LJ-07-01. 24 p.

Toms, C. 2019. Filling the gaps: Common bottlenose dolphin (*Tursiops truncatus*) population dynamics, structure, and connectivity within Florida panhandle bays, sounds, and estuaries. University of Central Florida. Dissertation. 373 p.

Torres, L.G. and A.J. Read. 2009. Where to catch a fish? The influence of foraging tactics on the ecology of bottlenose dolphins (*Tursiops truncatus*) in Florida Bay, Florida. Marine Mammal Science 25(4):797–815. doi: 10.1111/j.1748-7692.2009.00297.x

Tyson, R.B., S.M. Nowacek, and D.P. Nowacek. 2011. Community structure and abundance of bottlenose dolphins *Tursiops truncatus* in coastal waters of the northeast Gulf of Mexico. Marine Ecology Progress Series 438:253–265. doi: 10.3354/meps09292

Urian, K.W., S. Hofmann, R.S. Wells, and A.J. Read. 2009. Fine-scale population structure of bottlenose dolphins (*Tursiops truncatus*) in Tampa Bay, Florida. Marine Mammal Science 25(3):619–638. doi: 10.1111/j.1748-7692.2009.00284.x

Van Bressem, M.F., P.J. Duignan, A. Banyard, M. Barbieri, K.M. Colegrove, S. De Guise, G. Di Guardo, A. Dobson, M. Domingo, D. Fauquier, A. Fernandez, T. Goldstein, B. Grenfell, K.R. Groch, F. Gulland, B.A. Jensen, P.D. Jepson, A. Hall, T. Kuiken, S. Mazzariol, S.E. Morris, O. Nielsen, J.A. Raga, T.K. Rowles, J. Saliki, E. Sierra, N. Stephens, B. Stone, I. Tomo, J. Wang, T. Waltzek, and J.F. Wellehan. 2014. Cetacean morbillivirus: current knowledge and future directions. Viruses 6(12):5145–5181. doi: 10.3390/v6125145

Venn-Watson, S., L. Garrison, J. Litz, E. Fougeres, B. Mase, G. Rappucci, E. Stratton, R. Carmichael, D. Odell, D. Shannon, S. Shippee, S. Smith, L. Staggs, M. Tumlin, H. Whitehead, and T. Rowles. 2015. Demographic clusters identified within the northern Gulf of Mexico common bottlenose dolphin (*Tursiops truncates*) unusual mortality event: January 2010-June 2013. PLoS ONE 10(2):e0117248. doi: 10.1371/journal.pone.0117248

Vollmer, N.L. and P.E. Rosel. 2013. A review of common bottlenose dolphins (*Tursiops truncatus truncatus*) in the Northern Gulf of Mexico: Population biology, potential threats, and management. Southeastern Naturalist 12(m6):1–43. doi: 10.1656/058.012.m601

Weller, D.W. 1998. Global and regional variation in the biology and behavior of bottlenose dolphins. Texas A&M University. Dissertation. 142 p.

Wells, R.S. 1986a. Population structure of bottlenose dolphins: Behavioral studies along the central west coast of Florida. Contract Report to National Marine Fisheries Service, Southeast Fisheries Center. Contract No. 45-WCNF-5-003BB. 58 p.

Wells, R.S. 1986b. Structural aspects of dolphin societies. University of California, Santa Cruz. Dissertation. 234 p.

Wells, R.S. 1991. The role of long-term study in understanding the social structure of a bottlenose dolphin community. In: Pryor, K. and K. Norris (Eds.), Dolphin Societies: Discoveries and Puzzles. University of California Press, Berkeley, CA. pp. 199–226.

Wells, R.S. 2000. Reproduction in wild bottlenose dolphins: Overview of patterns observed during a long-term study. In: Duffield, D. and T. Robeck (Eds.), Bottlenose Dolphin Reproduction Workshop. AZA Marine Mammal Taxon Advisory Group, Silver Spring, MD. pp. 57–74.

Wells, R.S. 2003. Dolphin social complexity: Lessons from long-term study and life history. In: de Waal, F.B.M. and P.L. Tyack (Eds.), Animal Social Complexity: Intelligence, Culture, and Individualized Societies Harvard University Press, Cambridge, MA. pp. 32–56.

Wells, R.S. 2014. Social structure and life history of bottlenose dolphins near Sarasota Bay, Florida: Insights from four decades and five generations. Primates and cetaceans. pp. 149–172. doi: 10.1007/978-4-431-54523-1_8

Wells, R.S., M.K. Bassos, K.W. Urian, W.J. Carr, and M.D. Scott. 1996. Low-level monitoring of bottlenose dolphins, *Tursiops truncatus*, in Charlotte Harbor, Florida: 1990-1994. National Oceanic and Atmospheric Administration, National Marine Fisheries Service, Southeast Fisheries Science Center: Miami, FL. NOAA Technical Memorandum NMFS-SEFSC-384. 86 p.

Wells, R.S., M.K. Bassos, K.W. Urian, S.H. Shane, E.C.G. Owen, C.F. Weiss, W.J. Carr, and M.D. Scott. 1997. Low-level monitoring of bottlenose dolphins, *Tursiops truncatus*, in Pine Island Sound, Florida: 1996. National Oceanic and Atmospheric Administration, National Marine Fisheries Service, Southeast Fisheries Science Center: Miami, FL. Southeast Fisheries Center Contribution No. 40-WCNF601958. 86 p.

Wells, R.S., A. Natoli, and G. Braulik. 2019. *Tursiops truncatus* (errata version published in 2019). *The IUCN Red List of Threatened Species* 2019: e.T22563A156932432. https://dx.doi.org/10.2305/IUCN.UK.2019-1.RLTS.T22563A156932432.en

Wells, R.S., L.H. Schwacke, T.K. Rowles, B.C. Balmer, E. Zolman, T. Speakman, F.I. Townsend, M.C. Tumlin, A. Barleycorn, and K.A. Wilkinson. 2017. Ranging patterns of common bottlenose dolphins *Tursiops truncatus* in Barataria Bay, Louisiana, following the Deepwater Horizon oil spill. Endangered Species Research 33:159–180. doi: 10.3354/esr00732

Wells, R.S. and M.D. Scott. 1999. Bottlenose dolphin *Tursiops truncatus* (Montagu, 1821). In: Ridgway, S.H. and R. Harrison (Eds.), Handbook of marine mammals. Volume 6: The second book of dolphins and the porpoises. Academic Press, San Diego, CA. pp. 137–182.

Wells, R.S. and M.D. Scott. 2018. Bottlenose dolphin, *Tursiops truncatus*, common bottlenose dolphin. In: Würsig, B., J.G.M. Thewissen, and K.M. Kovacs (Eds.), Encyclopedia of Marine Mammals (Third Edition). Academic Press, London, UK. pp. 118–125. doi: 10.1016/B978-0-12-804327-1.00072-8

Wells, R.S., M.D. Scott, and A.B. Irvine. 1987. The social structure of free-ranging bottlenose dolphins. In: Genoways, H.H. (Ed.), Current Mammalogy. Plenum Press, New York, NY. pp. 247–305.

Wells, R.S., K.W. Urian, A.J. Read, M.K. Bassos, W.J. Carr, and M.D. Scott. 1996. Low-level monitoring of bottlenose dolphins, *Tursiops truncatus*, in Tampa Bay, Florida: 1988-1993. National Oceanic and Atmospheric Administration, National Marine Fisheries Service, Southeast Fisheries Science Center: Miami, FL. NOAA Technical Memorandum NMFS-SEFSC-385. 36 p.

Wilson, R.M., R.B. Tyson, J.A. Nelson, B.C. Balmer, J.P. Chanton, and D.P. Nowacek. 2017. Niche differentiation and prey selectivity among common bottlenose dolphins (*Tursiops truncatus*) sighted in St. George Sound, Gulf of Mexico. Frontiers in Marine Science 4:235. doi: 10.3389/fmars.2017.00235

Common bottlenose dolphin − *Tursiops truncatus*

GoMx BSE/Calcasieu Lake Stock

Overall Vulnerability Rank = Very High

Biological Sensitivity = High

Climate Exposure = Very High

Data Quality = 81% of scores ≥ 2

	Tursiops truncatus	Expert Scores	Data Quality	Expert Scores Plots (Portion by Category)
Sensitivity attributes	Prey/Diet Specificity	2.1	2.0	
	Habitat Specificity	2.6	1.7	
	Site Fidelity	3.5	2.0	
	Lifetime Reproductive Potential	1.7	2.3	
	Generation Length	2.8	2.3	
	Reproductive Plasticity	1.3	2.0	
	Migration	3.9	1.7	
	Home Range	3.4	1.3	
	Stock Abundance	3.7	1.7	
	Stock Abundance Trend	2.3	1.3	
	Cumulative Stressors	3.2	2.3	
	Sensitivity Score	High		
Exposure variables	Sea Surface Temperature (Change in mean)	4.0	2.7	
	Sea Surface Temperature (Change in variability)	1.0	2.7	
	Air Temperature (Change in mean)	4.0	2.7	
	Air Temperature (Change in variability)	1.0	2.7	
	Precipitation (Change in mean)	1.0	2.7	
	Precipitation (Change in variability)	1.0	2.7	
	Sea Surface Salinity (Change in mean)	2.0	2.7	
	Sea Surface Salinity (Change in variability)	1.0	2.7	
	Ocean pH (Change in mean)	4.0	2.7	
	Ocean pH (Change in variability)	1.0	2.7	
	Sea ice coverage (Change in mean)	1.0	2.7	
	Sea ice coverage (Change in variability)	1.0	2.7	
	Dissolved oxygen (Change in mean)	4.0	2.7	
	Dissolved oxygen (Change in variability)	1.0	2.7	
	Circulation	3.8	2.3	
	Sea level rise	4.0	2.7	
	Exposure Score	Very High		
Overall Vulnerability Rank		Very High		

Low
Moderate
High
Very High

Common bottlenose dolphin (GoMx BSE/Calcasieu Lake Stock)

Tursiops truncatus

CVA Results Summary

Overall Climate Vulnerability Rank: Very High (100% certainty from bootstrap analysis).

Climate Exposure: Very High. Six exposure factors scored greater than 3.5:

Air Temperature (Standard anomaly) (4.0), Dissolved oxygen (Standard anomaly) (4.0), Ocean pH (Standard anomaly) (4.0), Sea level rise (4.0), Sea Surface Temperature (Standard anomaly) (4.0), and Circulation (3.8).

Biological Sensitivity: High. Five sensitivity attributes scored greater than 3.0: Migration (3.87), Species Abundance (3.67), Site Fidelity (3.47), Home Range (3.40), and Cumulative Stressors (3.20).

Distributional Response: High

Abundance Response: High

Phenology Response: High

Data Quality: 81 % of the data quality scores were 2 or greater, 55 % of sensitivity attribute data quality scores were 2 or higher.

Background Information

Stock Description

This stock includes common bottlenose dolphins in Calcasieu Lake, in Louisiana (Hayes et al. 2022).

Prey/Diet Specificity

Generally, common bottlenose dolphins consume a large variety of fish and/or squid (Barros and Odell 1990; Mead and Potter 1990; Wells and Scott 2018; Bowen-Stevens et al. 2021). Common bottlenose dolphins seem to show a consistent preference for noise-producing fishes (e.g., sciaenids, toadfish; Berens McCabe et al. 2010; Bowen-Stevens et al. 2021). In Sarasota Bay, FL, Rossman et al. (2015) found some individual common bottlenose dolphins to show diet specialization despite the broader study group showing a generalist diet. Specialization can be influenced by locally abundant prey species (Shane et al. 1986; Vollmer and Rosel 2013) or can result from resource partitioning amongst sympatric populations (e.g., Torres and Read 2009; Wilson et al. 2017).

Habitat Specificity

Common bottlenose dolphins are found in a variety of marine and estuarine habitats (Wells and Scott 2018). Similar to Sabine Lake, the Calcasieu Lake estuary has been extensively modified with deep engineered channels to accommodate large vessels, which likely resulted in highly stratified salinity in the channels (Ward 1980).

Site Fidelity

No specific site fidelity information was found for this stock. However, other Gulf of Mexico bay, sound, and estuary (BSE) stocks show site fidelity (LaBrecque et al. 2015; Hayes et al. 2022), such as in Matagorda Bay, Espiritu Santo Bay, and San Luis Pass in Texas (Maze and Würsig 1999; Lynn and Würsig 2002; Irwin and Würsig 2004); Barataria Bay in Louisiana (Miller 2003; Wells et al. 2017; Takeshita et al. 2021); Mississippi Sound in Mississippi (Hubard et al. 2004; Mackey 2010; Mullin et al. 2017); Charlotte Harbor and Pine Island Sound (Bassos-Hull et al. 2013), Sarasota Bay (Wells 1986a; Wells 1991; Wells 2014), St. Andrew Bay (Balmer et al. 2019), St. Joseph Bay (Balmer et al. 2008; B. Balmer et al. 2018), St. Vincent Sound and Apalachicola Bay (Tyson et al. 2011), and Tampa Bay in Florida (Wells et al. 1996b; Urian et al. 2009).

Lifetime Reproductive Potential

Taylor et al. (2007) reported the interbirth interval of common bottlenose dolphins as 3.80 years, though other studies have shown it to be closer to three years (Connor et al. 2000; Leatherwood 1977; Wells 2003; Wells and Scott 1999).

Generally, females reach sexual maturity before males, with females reaching sexual maturity at 5–13 years (Sergeant et al. 1973; Perrin and Reilly 1984; Wells et al. 1987; Mead and Potter 1990; Mann et al. 2000; Wells 2000; Wells and Scott 2018).

Females reproduce through most of their adult lives (Leatherwood 1977; Hohn et al. 1989; Wells and Scott 1999; Wells 2000; Wells 2003), but may produce fewer calves after they reach 30 years of age (Wells 2000). Sarasota Bay common bottlenose dolphins can give birth when they are as young as 6 years of age, and can continue until they are 48 years old (Wells and Scott 2018). Reproductive lifespan would therefore be up to 42 years based on long-term observation studies (Wells 2014; Wells and Scott 2018).

Longitudinal studies from Sarasota Bay, Florida have found that female common bottlenose dolphins can live up to 67 years (Wells and Scott 2018). Studies based on growth layer groups in teeth suggest female common bottlenose dolphins can live to 41 years along the Texas coast (Fernandez and Hohn 1998) and to 30 years along the Mississippi coast (Mattson et al. 2006).

Generation Length

Taylor et al. (2007) reported common bottlenose dolphin generation length of 20.6-21.1 years.

Reproductive Plasticity

Common bottlenose dolphin calving is generally diffusely seasonal, with one or more peaks, although births have been reported from all seasons (Urian et al. 1996; Vollmer and Rosel 2013; Wells and Scott 2018). Calf sightings peak in the spring and summer months in Florida (Wells et al. 1987), Mississippi/Louisiana (Miller et al. 2010; Miller et al. 2013), and Texas (McHugh 1989; Henderson et al. 2007).

Migration

The BSE stocks in the Gulf of Mexico are thought to primarily comprise stable resident communities. However, transient or seasonal movements of some individuals into and out of a BSE stock area are recorded in some areas , and (LaBrecque et al. 2015; Hayes et al. 2022). Molecular genetic data support that some BSE stocks are genetically distinct from one another, and furthermore are differentiated from adjacent coastal populations (e.g., Sellas et al. 2005; Rosel et al. 2017). For example, Toms (2019) found fine-scale genetic population structure among five inshore systems in the Florida Panhandle and variable migration rates among populations.

Year-round residency has been reported for common bottlenose dolphins (Hayes et al. 2022), such as in Florida (Irvine and Wells 1972; Irvine et al. 1981; Wells 1986a; Wells 1986b; Wells et al. 1987; Scott et al. 1990; Wells 1991; Wells et al. 1996a; Wells et al. 1996b; Wells et al. 1997; Wells 2003; Balmer et al. 2008; Urian et al. 2009; Tyson et al. 2011; Bassos-Hull et al. 2013; B. Balmer et al. 2018; Balmer et al. 2019; Toms 2019), Texas (Shane 1990; Bräger 1993; Bräger et al. 1994; Fertl 1994; Weller 1998; Maze and Würsig 1999; Lynn and Würsig 2002; Irwin and Würsig 2004; Shane 2004), and Louisiana/Mississippi (Hubard et al. 2004; Mullin et al. 2017; Wells et al. 2017; Takeshita et al. 2021).

Home Range

The home ranges of individuals within the Gulf of Mexico BSE stocks are considered to generally include the areas within the stock boundaries and brief excursions beyond the stock boundaries (Irvine et al. 1981; Wells 1986a; Wells 1986b; Wells 1991; Wells et al. 1996a; Wells et al. 1996b; Balmer et al. 2008; Wells et al. 2017), although some individuals show preference for small areas within the stock boundaries (Lynn and Würsig 2002).

Stock Abundance

Aerial surveys by Scott et al. (1989) estimated 0-6 individuals (CV=0.34) for this stock. These estimates likely represent an underestimation due to reduced detection probability compared to other platforms (e.g., see comparison of abundance estimates from aerial and small boat surveys for Sabine Lake, Ronje et al. 2020).

Stock Abundance Trend

Data are insufficient to complete an abundance trend analysis for this stock (Hayes et al. 2022).

Cumulative Stressors

Common bottlenose dolphins face a wide range of individual and cumulative natural and anthropogenic threats (see reviews by Vollmer and Rosel 2013; Wells and Scott 2018; and Wells et al. 2019). These include such threats as shark predation, harmful algal blooms, recreational and commercial fishing gear, vessel traffic, pollution, habitat alteration, and provisioning.

Common bottlenose dolphins in Calcasieu Lake may have been exposed to oil and dispersants following the Deepwater Horizon oil spill, which may result in long term reproductive and chronic health effects (NOAA 2011; Michel et al. 2013; Helm et al. 2015; DWH NRDA 2016; Lane et al. 2015; De Guise et al. 2017, 2021; Kellar et al. 2017; Smith et al. 2017; Schwacke et al. 2017). An Unusual Mortality Event

(UME), involving primarily bottlenose dolphins, was declared for cetaceans in the northern Gulf of Mexico from 2010 to 2014, with the Deepwater Horizon oil spill as the primary underlying cause (Litz et al. 2014; Schwacke et al. 2014; Venn-Watson et al. 2015a, 2015b).

The nearshore and estuarine habitats along the Gulf of Mexico coast include waters adjacent to areas of high human population, some of which are highly industrialized, and areas of heavy agricultural impact. Studies of bottlenose dolphin tissues from nearby Barataria Bay have shown persistent organic pollutant (POP) in blubber and blood samples, though at concentrations lower than other southeastern U.S. sites (Balmer et al. 2015; J. Balmer et al. 2018). Similarly, mercury has been detected in common bottlenose dolphin tissues in Louisiana waters, but in lower concentrations than common bottlenose dolphins found in Florida waters (McCormack et al. 2020a, 2020b, 2022).

Dolphin morbillivirus has been detected in populations of the northern Gulf of Mexico, but has not been attributed to large scale die-offs since 1993−1994 (Lipscomb et al. 1996; Litz et al. 2014; Van Bressem et al. 2014; Fauquier et al. 2017). A UME was declared in 1990 for the Gulf of Mexico from Florida to Texas, with more than 300 common bottlenose dolphins stranding due to an undetermined cause, although morbillivirus and cold temperatures may have contributed (Litz et al. 2014). This stock was also affected by a 2007 UME in which 66 common bottlenose dolphins stranded in northeast Texas and west Louisiana due to an undetermined cause (Litz et al. 2014).

Further Reading

Hayes, S.A., E. Josephson, K. Maze-Foley, P.E. Rosel, and J. Wallace. 2022. U.S. Atlantic and Gulf of Mexico marine mammal stock assessments 2021. National Oceanic and Atmospheric Administration, National Marine Fisheries Service, Northeast Fisheries Science Center: Woods Hole, MA. NOAA Technical Memorandum NMFS-NE-288. 380 p. doi: 10.25923/6tt7-kc16

Wells, R.S. and M.D. Scott. 2018. Bottlenose Dolphin, *Tursiops truncatus*, Common Bottlenose Dolphin. In: Würsig, B., J.G.M. Thewissen, and K.M. Kovacs (Eds.), Encyclopedia of Marine Mammals (Third Edition). Academic Press, London, UK. pp. 118–125. doi: 10.1016/B978-0-12-804327-1.00072-8

Wells, R.S., A Natoli, and G. Braulik. 2019. *Tursiops truncatus* (errata version published in 2019). The IUCN Red List of Threatened Species 2019: e.T22563A156932432. https://dx.doi.org/10.2305/IUCN.UK.2019-1.RLTS.T22563A156932432.en

References

Balmer, B., T. McDonald, F. Hornsby, J. Adams, J. Allen, A. Barleycorn, and L. Schwacke. 2018. Long-term trends in a northern Gulf of Mexico common bottlenose dolphin (*Tursiops truncatus*) population in the wake of the Deepwater Horizon oil spill. Journal of Cetacean Research and Management 18:1–9. doi: 10.47536/jcrm.v18i1.409

Balmer, B., S. Watwood, B. Quigley, T. Speakman, K. Barry, K. Mullin, P. Rosel, C. Sinclair, E. Zolman, and L. Schwacke. 2019. Common bottlenose dolphin (*Tursiops truncatus*) abundance and distribution patterns in St. Andrew Bay, Florida, USA. Aquatic Conservation: Marine and Freshwater Ecosystems 29(3):486–498. doi: 10.1002/aqc.3001

Balmer, B.C., R.S. Wells, S.M. Nowacek, D.P. Nowacek, L.H. Schwacke, W.A. McLellan, F.S. Scharf, T.K. Rowles, L.J. Hansen, T.R. Spradlin, and D.A. Pabst. 2008. Seasonal abundance and distribution patterns of common bottlenose dolphins (*Tursiops truncatus*) near St. Joseph Bay, Florida, USA. Journal of Cetacean Research and Management 10(2):157–167.

Balmer, B.C., G.M. Ylitalo, L.E. McGeorge, K.A. Baugh, D. Boyd, K.D. Mullin, P.E. Rosel, C. Sinclair, R.S. Wells, E.S. Zolman, and L.H. Schwacke. 2015. Persistent organic pollutants (POPs) in blubber of common bottlenose dolphins (*Tursiops truncatus*) along the northern Gulf of Mexico coast, USA. Science of the Total Environment 527-528:306–312. doi: 10.1016/j.scitotenv.2015.05.016

Balmer, J.E., G.M. Ylitalo, T.K. Rowles, K.D. Mullin, R.S. Wells, F.I. Townsend, R.W. Pearce, J.L. Bolton, E.S. Zolman, B.C. Balmer, and L.H. Schwacke. 2018. Persistent organic pollutants (POPs) in blood and blubber of common bottlenose dolphins (*Tursiops truncatus*) at three northern Gulf of Mexico sites following the Deepwater Horizon oil spill. Science of the Total Environment 621:130–137. doi: 10.1016/j.scitotenv.2017.11.209

Barros, N.B. and D.K. Odell. 1990. Food habits of bottlenose dolphins in the southeastern United States In: Leatherwood, S. and R.R. Reeves (Eds.), The Bottlenose Dolphin. Academic Press, San Diego, CA. pp. 309–328. doi: 10.1016/B978-0-12-440280-5.50020-2

Bassos-Hull, K., R.M. Perrtree, C.C. Shepard, S. Schilling, A.A. Barleycorn, J.B. Allen, B.C. Balmer, W.E. Pine, and R.S. Wells. 2013. Long-term site fidelity and seasonal abundance estimates of common bottlenose dolphins (*Tursiops truncatus*) along the southwest coast of Florida and responses to natural perturbations. Journal of Cetacean Research and Management 13(1):19–30. doi: 10.47536/jcrm.v13i1.551

Berens McCabe, E.J., D.P. Gannon, N.B. Barros, and R.S. Wells. 2010. Prey selection by resident common bottlenose dolphins (*Tursiops truncatus*) in Sarasota Bay, Florida. Marine Biology 157(5):931–942. doi: 10.1007/s00227-009-1371-2

Bowen-Stevens, S.R., D.P. Gannon, R.A. Hazelkorn, G. Lovewell, K.M. Volker, S. Smith, M.C. Tumlin, and J. Litz. 2021. Diet of common bottlenose dolphins, *Tursiops truncatus*, that stranded in and near Barataria Bay, Louisiana, 2010–2012. Southeastern Naturalist 20(1). doi: 10.1656/058.020.0113

Bräger, S. 1993. Diurnal and seasonal behavior patterns of bottlenose dolphins (*Tursiops truncatus*). Marine Mammal Science 9:434–438. doi: 10.1111/j.1748-7692.1993.tb00477.x

Bräger, S., B. Würsig, A. Acevedo, and T. Henningsen. 1994. Association patterns of bottle-nosed dolphins (*Tursiops-truncatus*) in Galveston Bay, Texas. Journal of Mammalogy 75(2):431–437. doi: 10.2307/1382564

Connor, R.C., R.S. Wells, J. Mann, and A.J. Read. 2000. The bottlenose dolphin: Social relationships in a fission-fusion society. In: Mann, J., R. Connor, P. Tyack, and H. Whitehead (Eds.), Cetacean Societies: Field Studies of Dolphins and Whales. University of Chicago Press, Chicago, IL. pp. 91–126.

De Guise, S., M. Levin, E. Gebhard, L. Jasperse, L. Burdett Hart, C.R. Smith, S. Venn-Watson, F. Townsend, R. Wells, B. Balmer, E. Zolman, T. Rowles, and L. Schwacke. 2017. Changes in immune functions in bottlenose dolphins in the northern Gulf of Mexico associated with the Deepwater Horizon oil spill. Endangered Species Research 33:291–303. doi: 10.3354/esr00814

De Guise, S., M. Levin, L. Jasperse, J. Herrman, R.S. Wells, T. Rowles, and L. Schwacke. 2021. Long-term immunological alterations in bottlenose dolphin a decade after the Deepwater Horizon oil spill in the northern Gulf of Mexico: Potential for multigenerational effects. Environmental Toxicology and Chemistry 40(5):1308–1321. doi: 10.1002/etc.4980

Deepwater Horizon Natural Resource Damage Assessment Trustees (DWH NRDA). 2016. Deepwater Horizon oil spill: Final Programmatic Damage Assessment and Restoration Plan and Final Programmatic Environmental Impact Statement. Retrieved from: http://www.gulfspillrestoration.noaa.gov/restoration-planning/gulf-plan.1659.

Fauquier, D.A., J. Litz, S. Sanchez, K. Colegrove, L.H. Schwacke, L. Hart, J. Saliki, C. Smith, T. Goldstein, S. Bowen-Stevens, W. McFee, E. Fougeres, B. Mase-Guthrie, E. Stratton, R. Ewing, S. Venn-Watson, R.H. Carmichael, C. Clemons-Chevis, W. Hatchett, D. Shannon, S. Shippee, S. Smith, L. Staggs, M.C. Tumlin, N.L. Wingers, and T.K. Rowles. 2017. Evaluation of morbillivirus exposure in

cetaceans from the northern Gulf of Mexico 2010-2014. Endangered Species Research 33:211–220. doi: 10.3354/esr00772

Fernandez, S. and A.A. Hohn. 1998. Age, growth, and calving season of bottlenose dolphins, *Tursiops truncatus*, off coastal Texas. Fishery Bulletin 96(2):357–365.

Fertl, D. 1994. Occurrence patterns and behavior of bottlenose dolphins (*Tursiops truncatus*) in the Galveston ship channel, Texas. Texas Journal of Science 46(4):299–318.

Hayes, S.A., E. Josephson, K. Maze-Foley, P.E. Rosel, and J. Wallace. 2022. U.S. Atlantic and Gulf of Mexico marine mammal stock assessments 2021. National Oceanic and Atmospheric Administration, National Marine Fisheries Service, Northeast Fisheries Science Center: Woods Hole, MA. NOAA Technical Memorandum NMFS-NE-288. 380 p. doi: 10.25923/6tt7-kc16

Helm, R.C., D.P. Costa, T.D. DeBruyn, T.J. O'Shea, R.S. Wells, and T.M. Williams. 2015. Overview of effects of oil spills on marine mammals. In: Fingas, M. (Ed.), Handbook of oil spill science and technology, First edition. John Wiley & Sons, Inc., Hoboken, NJ. pp. 455–475.

Henderson, E.E. and B. Würsig. 2007. Behavior patterns of bottlenose dolphins in San Luis Pass, Texas. Gulf of Mexico Science 25(2):153–161. doi: 10.18785/goms.2502.06

Hohn, A.A., M.D. Scott, R.S. Wells, J.C. Sweeney, and A.B. Irvine. 1989. Growth layers in teeth from known-age, free-ranging bottlenose dolphins. Marine Mammal Science 5(4):315–342. doi: 10.1111/j.1748-7692.1989.tb00346.x

Hubard, C.W., K. Maze-Foley, K.D. Mullin, and W.W. Schroeder. 2004. Seasonal abundance and site fidelity of bottlenose dolphins (*Tursiops truncatus*) in Mississippi Sound. Aquatic Mammals 30(2):299–310. doi: 10.1578/am.30.2.2004.299

Irvine, A.B., M.D. Scott, R.S. Wells, and J.H. Kaufmann. 1981. Movements and activities of the Atlantic bottlenose dolphin, *Tursiops truncatus*, near Sarasota, Florida. Fishery Bulletin 79(4):671–688.

Irvine, B. and R.S. Wells. 1972. Results of attempts to tag Atlantic bottlenosed dolphins (*Tursiops truncatus*). Cetology 13:1–5.

Irwin, L.-J. and B. Würsig. 2004. A small resident community of bottlenose dolphins, *Tursiops truncatus*, in Texas: Monitoring recommendations. Gulf of Mexico Science 22(1):13–21. doi: 10.18785/goms.2201.02

Kellar, N.M., T.R. Speakman, C.R. Smith, S.M. Lane, B.C. Balmer, M.L. Trego, K.N. Catelani, M.N. Robbins, C.D. Allen, R.S. Wells, E.S. Zolman, T.K. Rowles, and L.H. Schwacke. 2017. Low reproductive success rates of common bottlenose dolphins *Tursiops truncatus* in the northern Gulf of Mexico following the Deepwater Horizon disaster (2010-2015). Endangered Species Research 33:143–158. doi: 10.3354/esr00775

LaBrecque, E., C. Curtice, J. Harrison, S.M. Van Parijs, and P.N. Halpin. 2015. 3. Biologically Important Areas for cetaceans within U.S. Waters – Gulf of Mexico Region. Aquatic Mammals 41(1):30–38. doi: 10.1578/am.41.1.2015.30

Lane, S.M., C.R. Smith, J. Mitchell, B.C. Balmer, K.P. Barry, T. McDonald, C.S. Mori, P.E. Rosel, T.K. Rowles, T.R. Speakman, F.I. Townsend, M.C. Tumlin, R.S. Wells, E.S. Zolman, and L.H. Schwacke. 2015. Reproductive outcome and survival of common bottlenose dolphins sampled in Barataria Bay, Louisiana, USA, following the Deepwater Horizon oil spill. Proceedings of the Royal Society B: Biological Sciences 282(1818):20151944. doi: 10.1098/rspb.2015.1944

Leatherwood, S. 1977. Some preliminary impressions on the numbers and social behavior of free-swimming bottlenosed dolphin calves (*Tursiops truncatus*) in the northern Gulf of Mexico. In: Ridgway, S.H. and K. Bernirschke (Eds.), Breeding Dolphins; Present Status, Suggestions for the Future. Final report to the US Marine Mammal Commission, Contract MM6AC009, MMC-76/07, Washington, DC. pp. 143–167.

Lipscomb, T.P., S. Kennedy, D. Moffett, A. Krafft, B.A. Klaunberg, J.H. Lichy, G.T. Regan, G.A.J. Worthy, and J.K. Taubenberger. 1996. Morbilliviral epizootic in bottlenose dolphins of the Gulf of Mexico. Journal of Veterinary Diagnostic Investigation 8(3):283–290. doi: 10.1177/104063879600800302

Litz, J.A., M.A. Baran, S.R. Bowen-Stevens, R.H. Carmichael, K.M. Colegrove, L.P. Garrison, S.E. Fire, E.M. Fougeres, R. Hardy, S. Holmes, W. Jones, B.E. Mase-Guthrie, D.K. Odell, P.E. Rosel, J.T. Saliki, D.K. Shannon, S.F. Shippee, S.M. Smith, E.M. Stratton, M.C. Tumlin, H.R. Whitehead, G.A. Worthy, and T.K. Rowles. 2014. Review of historical unusual mortality events (UMEs) in the Gulf of Mexico (1990–2009): providing context for the multi-year northern Gulf of Mexico cetacean UME declared in 2010. Diseases of Aquatic Organisms 112(2):161–175. doi: 10.3354/dao02807

Lynn, S.K. and B. Würsig. 2002. Summer movement patterns of bottlenose dolphins in a Texas bay. Gulf of Mexico Science 20(1):25–37. doi: 10.18785/goms.2001.03

Mackey, A.D. 2010. Site fidelity and association patterns of bottlenose dolphins (*Tursiops truncatus*) in the Mississippi Sound. University of Southern Mississippi. Thesis. 133 p.

Mann, J., R.C. Connor, L.M. Barre, and M.R. Heithaus. 2000. Female reproductive success in bottlenose dolphins (*Tursiops* sp.): life history, habitat, provisioning, and group-size effects. Behavioral Ecology 11(2):210–219. doi: 10.1093/beheco/11.2.210

Mattson, M.C., K.D. Mullin, G.W. Ingram, and W. Hoggard. 2006. Age structure and growth of the bottlenose dolphin (*Tursiops truncatus*) from strandings in the Mississippi sound region of the north-central Gulf of Mexico from 1986 to 2003. Marine Mammal Science 22(3):654–666. doi: 10.1111/j.1748-7692.2006.00057.x

Maze, K.S. and B. Würsig. 1999. Bottlenosed dolphins of San Luis Pass, Texas: Occurrence patterns, site-fidelity, and habitat use. Aquatic Mammals 25:91–104.

McCormack, M.A., F. Battaglia, W.E. McFee, and J. Dutton. 2020a. Mercury concentrations in blubber and skin from stranded bottlenose dolphins (*Tursiops truncatus*) along the Florida and Louisiana coasts (Gulf of Mexico, USA) in relation to biological variables. Environmental Research 180:108886. doi: 10.1016/j.envres.2019.108886

McCormack, M.A., B.P. Jackson, and J. Dutton. 2020b. Relationship between mercury and selenium concentrations in tissues from stranded odontocetes in the northern Gulf of Mexico. Science of the Total Environment 749:141350. doi: 10.1016/j.scitotenv.2020.141350

McCormack, M.A., W.H. Nowlin, and J. Dutton. 2022. Effect of trophic position on mercury concentrations in bottlenose dolphins (*Tursiops truncatus*) from the northern Gulf of Mexico. Environmental Research 204(Pt B):112124. doi: 10.1016/j.envres.2021.112124

McHugh, M.B. 1989. Population numbers and feeding behavior of the Atlantic bottlenose dolphin (*Tursiops truncatus*) near Aransas Pass, Texas. University of Texas at Austin. Thesis. 96 p.

Mead, J.G. and C.W. Potter. 1990. Natural history of bottlenose dolphins along the central Atlantic coast of the United States. In: Leatherwood, S. and R.R. Reeves (Eds.), The Bottlenose Dolphin. Academic Press, San Diego, CA. pp. 165–195.

Michel, J., E.H. Owens, S. Zengel, A. Graham, Z. Nixon, T. Allard, W. Holton, P.D. Reimer, A. Lamarche, M. White, N. Rutherford, C. Childs, G. Mauseth, G. Challenger, and E. Taylor. 2013. Extent and degree of shoreline oiling: Deepwater Horizon oil spill, Gulf of Mexico, USA. PLoS ONE 8(6):e65087. doi: 10.1371/journal.pone.0065087

Miller, C.E. 2003. Abundance trends and environmental habitat usage patterns of bottlenose dolphins (*Tursiops truncatus*) in lower Barataria and Caminada bays, Louisiana. Louisiana State University and Agricultural & Mechanical College. Dissertation. 125 p.

Miller, L.J., A.D. Mackey, T. Hoffland, M. Solangi, and S.A. Kuczaj. 2010. Potential effects of a major hurricane on Atlantic bottlenose dolphin (*Tursiops truncatus*) reproduction in the Mississippi Sound. Marine Mammal Science 26(3):707–715. doi: 10.1111/j.1748-7692.2010.00371.x

Miller, L.J., A.D. Mackey, M. Solangi, and S.A. Kuczaj. 2013. Population abundance and habitat utilization of bottlenose dolphins in the Mississippi Sound. Aquatic Conservation: Marine and Freshwater Ecosystems 23(1):145–151. doi: 10.1002/aqc.2278

Mullin, K.D., T. McDonald, R.S. Wells, B.C. Balmer, T. Speakman, C. Sinclair, E.S. Zolman, F. Hornsby, S.M. McBride, K.A. Wilkinson, and L.H. Schwacke. 2017. Density, abundance, survival, and ranging patterns of common bottlenose dolphins (*Tursiops truncatus*) in Mississippi Sound following the Deepwater Horizon oil spill. PLoS ONE 12(10):e0186265. doi: 10.1371/journal.pone.0186265

National Oceanic and Atmospheric Administration (NOAA). 2011. Public scoping for preparation of a programmatic environmental impact statement for the Deepwater Horizon BP Oil Spill. 9 p.

Perrin, W.F. and S.B. Reilly. 1984. Reproductive parameters of dolphins and small whales of the family Delphinidae. In: Perrin, W.F., R.L. Brownell, Jr., and D.P. DeMaster (Eds.), Reproduction in Whales, Dolphins, and Porpoises. pp. 97–134.

Ronje, E., H. Whitehead, K. Barry, S. Piwetz, J. Struve, V. Lecours, L. Garrison, R.S. Wells, and K.D. Mullin. 2020. Abundance and occurrence of common bottlenose dolphins (*Tursiops truncatus*) in three estuaries of the northwestern Gulf of Mexico. Gulf and Caribbean Research 31:18–34. doi: 10.18785/gcr.3101.09

Rosel, P.E., L.A. Wilcox, C. Sinclair, T.R. Speakman, M.C. Tumlin, J.A. Litz, and E.S. Zolman. 2017. Genetic assignment to stock of stranded common bottlenose dolphins in southeastern Louisiana after the Deepwater Horizon oil spill. Endangered Species Research 33:221–234. doi: 10.3354/esr00780

Rossman, S., P.H. Ostrom, M. Stolen, N.B. Barros, H. Gandhi, C.A. Stricker, and R.S. Wells. 2015. Individual specialization in the foraging habits of female bottlenose dolphins living in a trophically diverse and habitat rich estuary. Oecologia 178(2):415–425. doi: 10.1007/s00442-015-3241-6

Schwacke, L.H., C.R. Smith, F.I. Townsend, R.S. Wells, L.B. Hart, B.C. Balmer, T.K. Collier, S. De Guise, M.M. Fry, L.J. Guillette, Jr., S.V. Lamb, S.M. Lane, W.E. McFee, N.J. Place, M.C. Tumlin, G.M. Ylitalo, E.S. Zolman, and T.K. Rowles. 2014. Health of common bottlenose dolphins (*Tursiops truncatus*) in Barataria Bay, Louisiana, following the deepwater horizon oil spill. Environmental Science & Technology 48(1):93–103. doi: 10.1021/es403610f

Schwacke, L.H., L. Thomas, R.S. Wells, W.E. McFee, A.A. Hohn, K.D. Mullin, E.S. Zolman, B.M. Quigley, T.K. Rowles, and J.H. Schwacke. 2017. Quantifying injury to common bottlenose dolphins from the Deepwater Horizon oil spill using an age-, sex- and class-structured population model. Endangered Species Research 33:265–279. doi: 10.3354/esr00777

Scott, G.P., D.M. Burn, L.J. Hansen, and R.E. Owen. 1989. Estimates of bottlenose dolphin abundance in the Gulf of Mexico from regional aerial surveys. National Oceanic and Atmospheric Administration, National Marine Fisheries Service, Southeast Fisheries Center: Miami, FL. Southeast Fisheries Center Contribution CRD-88/89-07. 77 p.

Scott, M.D. and S.J. Chivers. 1990. Distribution and herd structure of bottlenose dolphins in the eastern tropical Pacific Ocean. In: Leatherwood, S. and R.R. Reeves (Eds.), The Bottlenose Dolphin. Academic Press, San Diego, CA. pp. 387–402.

Sellas, A.B., R.S. Wells, and P.E. Rosel. 2005. Mitochondrial and nuclear DNA analyses reveal fine scale geographic structure in bottlenose dolphins (*Tursiops truncatus*) in the Gulf of Mexico. Conservation Genetics 6(5):715–728. doi: 10.1007/s10592-005-9031-7

Sergeant, D.E., D.K. Caldwell, and M.C. Caldwell. 1973. Age, growth, and maturity of bottlenosed dolphin (*Tursiops-truncatus*) from northeast Florida. Journal of the Fisheries Research Board of Canada 30(7):1009–1011. doi: 10.1139/f73-165

Shane, S.H. 1990. Behavior and ecology of the bottlenose dolphin at Sanibel Island, Florida. In: Leatherwood, S. and R.R. Reeves (Eds.), The Bottlenose Dolphin. Academic Press, San Diego, CA. pp. 245–265. doi: 10.1016/B978-0-12-440280-5.50016-0

Shane, S.H. 2004. Residence patterns, group characteristics, and association patterns of bottlenose dolphins near Sanibel Island, Florida. Gulf of Mexico Science 22(1):1–12. doi: 10.18785/goms.2201.01

Shane, S.H., R.S. Wells, and B. Würsig. 1986. Ecology, behavior and social organization of the bottlenose dolphin: A review. Marine Mammal Science 2:34–63. doi: 10.1111/j.1748-7692.1986.tb00026.x

Smith, C.R., T.K. Rowles, L.B. Hart, F.I. Townsend, R.S. Wells, E.S. Zolman, B.C. Balmer, B. Quigley, M. Ivančić, W. McKercher, M.C. Tumlin, K.D. Mullin, J.D. Adams, Q. Wu, W. McFee, T.K. Collier, and L.H. Schwacke. 2017. Slow recovery of Barataria Bay dolphin health following the Deepwater Horizon oil spill (2013-2014), with evidence of persistent lung disease and impaired stress response. Endangered Species Research 33:127–142. doi: 10.3354/esr00778

Takeshita, R., B.C. Balmer, F. Messina, E.S. Zolman, L. Thomas, R.S. Wells, C.R. Smith, T.K. Rowles, and L.H. Schwacke. 2021. High site-fidelity in common bottlenose dolphins despite low salinity exposure and associated indicators of compromised health. PLoS ONE 16(9):e0258031. doi: 10.1371/journal.pone.0258031

Taylor, B.L., S.J. Chivers, J. Larese, and W.F. Perrin. 2007. Generation length and percent mature estimates for IUCN assessments of cetaceans. National Oceanic and Atmospheric Administration, National Marine Fisheries Service, Southwest Fisheries Science Center: La Jolla, CA. Administrative Report LJ-07-01. 24 p.

Toms, C. 2019. Filling the gaps: Common bottlenose dolphin (*Tursiops truncatus*) population dynamics, structure, and connectivity within Florida panhandle bays, sounds, and estuaries. University of Central Florida. Dissertation. 373 p.

Torres, L.G. and A.J. Read. 2009. Where to catch a fish? The influence of foraging tactics on the ecology of bottlenose dolphins (*Tursiops truncatus*) in Florida Bay, Florida. Marine Mammal Science 25(4):797–815. doi: 10.1111/j.1748-7692.2009.00297.x

Tyson, R.B., S.M. Nowacek, and D.P. Nowacek. 2011. Community structure and abundance of bottlenose dolphins *Tursiops truncatus* in coastal waters of the northeast Gulf of Mexico. Marine Ecology Progress Series 438:253–265. doi: 10.3354/meps09292

Urian, K.W., D.A. Duffield, A.J. Read, R.S. Wells, and E.D. Shell. 1996. Seasonality of reproduction in bottlenose dolphins, *Tursiops truncatus*. Journal of Mammalogy 77(2):394–403. doi: 10.2307/1382814

Urian, K.W., S. Hofmann, R.S. Wells, and A.J. Read. 2009. Fine-scale population structure of bottlenose dolphins (*Tursiops truncatus*) in Tampa Bay, Florida. Marine Mammal Science 25(3):619–638. doi: 10.1111/j.1748-7692.2009.00284.x

Van Bressem, M.F., P.J. Duignan, A. Banyard, M. Barbieri, K.M. Colegrove, S. De Guise, G. Di Guardo, A. Dobson, M. Domingo, D. Fauquier, A. Fernandez, T. Goldstein, B. Grenfell, K.R. Groch, F. Gulland, B.A. Jensen, P.D. Jepson, A. Hall, T. Kuiken, S. Mazzariol, S.E. Morris, O. Nielsen, J.A. Raga, T.K. Rowles, J. Saliki, E. Sierra, N. Stephens, B. Stone, I. Tomo, J. Wang, T. Waltzek, and J.F. Wellehan. 2014. Cetacean morbillivirus: current knowledge and future directions. Viruses 6(12):5145–5181. doi: 10.3390/v6125145

Venn-Watson, S., L. Garrison, J. Litz, E. Fougeres, B. Mase, G. Rappucci, E. Stratton, R. Carmichael, D. Odell, D. Shannon, S. Shippee, S. Smith, L. Staggs, M. Tumlin, H. Whitehead, and T. Rowles. 2015. Demographic clusters identified within the northern Gulf of Mexico common bottlenose dolphin (*Tursiops truncates*) unusual mortality event: January 2010-June 2013. PLoS ONE 10(2):e0117248. doi: 10.1371/journal.pone.0117248

Vollmer, N.L. and P.E. Rosel. 2013. A review of common bottlenose dolphins (*Tursiops truncatus truncatus*) in the Northern Gulf of Mexico: Population biology, potential threats, and management. Southeastern Naturalist 12(m6):1–43. doi: 10.1656/058.012.m601

Ward, G.H. 1980. Hydrography and circulation processes of Gulf estuaries. In: Hamilton, P. and K.B. Macdonald (Eds.), Estuarine and Wetland Processes, Marine Science Book Series. Vol. 11. Springer Science+Business Media, New York, NY. p. 183—215. doi: 10.1007/978—1—4757—5177—2

Weller, D.W. 1998. Global and regional variation in the biology and behavior of bottlenose dolphins. Texas A&M University. Dissertation. 142 p.

Wells, R.S. 1986a. Population structure of bottlenose dolphins: Behavioral studies along the central west coast of Florida. Contract Report to National Marine Fisheries Service, Southeast Fisheries Center. Contract No. 45-WCNF-5-003BB. 58 p.

Wells, R.S. 1986b. Structural aspects of dolphin societies. University of California, Santa Cruz. Dissertation. 234 p.

Wells, R.S. 1991. The role of long-term study in understanding the social structure of a bottlenose dolphin community. In: Pryor, K. and K. Norris (Eds.), Dolphin Societies: Discoveries and Puzzles. University of California Press, Berkeley, CA. pp. 199–226.

Wells, R.S. 2000. Reproduction in wild bottlenose dolphins: Overview of patterns observed during a long-term study. In: Duffield, D. and T. Robeck (Eds.), Bottlenose Dolphin Reproduction Workshop. AZA Marine Mammal Taxon Advisory Group, Silver Spring, MD. pp. 57–74.

Wells, R.S. 2003. Dolphin social complexity: Lessons from long-term study and life history. In: de Waal, F.B.M. and P.L. Tyack (Eds.), Animal Social Complexity: Intelligence, Culture, and Individualized Societies Harvard University Press, Cambridge, MA. pp. 32–56.

Wells, R.S. 2014. Social structure and life history of bottlenose dolphins near Sarasota Bay, Florida: Insights from four decades and five generations. Primates and cetaceans. pp. 149–172. doi: 10.1007/978-4-431-54523-1_8

Wells, R.S., M.K. Bassos, K.W. Urian, W.J. Carr, and M.D. Scott. 1996. Low-level monitoring of bottlenose dolphins, *Tursiops truncatus*, in Charlotte Harbor, Florida: 1990-1994. National Oceanic and Atmospheric Administration, National Marine Fisheries Service, Southeast Fisheries Science Center: Miami, FL. NOAA Technical Memorandum NMFS-SEFSC-384. 86 p.

Wells, R.S., M.K. Bassos, K.W. Urian, S.H. Shane, E.C.G. Owen, C.F. Weiss, W.J. Carr, and M.D. Scott. 1997. Low-level monitoring of bottlenose dolphins, *Tursiops truncatus*, in Pine Island Sound, Florida: 1996. National Oceanic and Atmospheric Administration, National Marine Fisheries Service, Southeast Fisheries Science Center: Miami, FL. Southeast Fisheries Center Contribution No. 40-WCNF601958. 86 p.

Wells, R.S., A. Natoli, and G. Braulik. 2019. *Tursiops truncatus* (errata version published in 2019). *The IUCN Red List of Threatened Species* 2019: e.T22563A156932432. https://dx.doi.org/10.2305/IUCN.UK.2019-1.RLTS.T22563A156932432.en

Wells, R.S., L.H. Schwacke, T.K. Rowles, B.C. Balmer, E. Zolman, T. Speakman, F.I. Townsend, M.C. Tumlin, A. Barleycorn, and K.A. Wilkinson. 2017. Ranging patterns of common bottlenose dolphins *Tursiops truncatus* in Barataria Bay, Louisiana, following the Deepwater Horizon oil spill. Endangered Species Research 33:159–180. doi: 10.3354/esr00732

Wells, R.S. and M.D. Scott. 1999. Bottlenose dolphin *Tursiops truncatus* (Montagu, 1821). In: Ridgway, S.H. and R. Harrison (Eds.), Handbook of marine mammals. Volume 6: The second book of dolphins and the porpoises. Academic Press, San Diego, CA. pp. 137–182.

Wells, R.S. and M.D. Scott. 2018. Bottlenose dolphin, *Tursiops truncatus*, common bottlenose dolphin. In: Würsig, B., J.G.M. Thewissen, and K.M. Kovacs (Eds.), Encyclopedia of Marine Mammals

(Third Edition). Academic Press, London, UK. pp. 118–125. doi: 10.1016/B978-0-12-804327-1.00072-8

Wells, R.S., M.D. Scott, and A.B. Irvine. 1987. The social structure of free-ranging bottlenose dolphins. In: Genoways, H.H. (Ed.), Current Mammalogy. Plenum Press, New York, NY. pp. 247–305.

Wells, R.S., K.W. Urian, A.J. Read, M.K. Bassos, W.J. Carr, and M.D. Scott. 1996. Low-level monitoring of bottlenose dolphins, *Tursiops truncatus*, in Tampa Bay, Florida: 1988-1993. National Oceanic and Atmospheric Administration, National Marine Fisheries Service, Southeast Fisheries Science Center: Miami, FL. NOAA Technical Memorandum NMFS-SEFSC-385. 36 p.

Wilson, R.M., R.B. Tyson, J.A. Nelson, B.C. Balmer, J.P. Chanton, and D.P. Nowacek. 2017. Niche differentiation and prey selectivity among common bottlenose dolphins (*Tursiops truncatus*) sighted in St. George Sound, Gulf of Mexico. Frontiers in Marine Science 4:235. doi: 10.3389/fmars.2017.00235

Common bottlenose dolphin − *Tursiops truncatus*

GoMx BSE/Caloosahatchee River Stock

Overall Vulnerability Rank = Very High

Biological Sensitivity = Very High

Climate Exposure = Very High

Data Quality = 85% of scores ≥ 2

	Tursiops truncatus	Expert Scores	Data Quality	Expert Scores Plots (Portion by Category)
Sensitivity attributes	Prey/Diet Specificity	2.0	2.0	
	Habitat Specificity	3.5	1.7	
	Site Fidelity	3.9	2.3	
	Lifetime Reproductive Potential	1.9	2.0	
	Generation Length	3.1	2.0	
	Reproductive Plasticity	1.1	2.0	
	Migration	3.9	2.0	
	Home Range	3.7	2.3	
	Stock Abundance	3.4	1.3	
	Stock Abundance Trend	2.1	1.0	
	Cumulative Stressors	3.2	1.3	
	Sensitivity Score	Very High		
Exposure variables	Sea Surface Temperature (Change in mean)	3.9	2.7	
	Sea Surface Temperature (Change in variability)	1.1	2.7	
	Air Temperature (Change in mean)	3.9	2.7	
	Air Temperature (Change in variability)	1.1	2.7	
	Precipitation (Change in mean)	1.1	2.7	
	Precipitation (Change in variability)	1.1	2.7	
	Sea Surface Salinity (Change in mean)	2.1	2.7	
	Sea Surface Salinity (Change in variability)	1.2	2.7	
	Ocean pH (Change in mean)	3.9	2.7	
	Ocean pH (Change in variability)	1.1	2.7	
	Sea ice coverage (Change in mean)	1.0	3.0	
	Sea ice coverage (Change in variability)	1.0	3.0	
	Dissolved oxygen (Change in mean)	3.9	2.7	
	Dissolved oxygen (Change in variability)	1.1	2.7	
	Circulation	3.9	2.0	
	Sea level rise	3.9	3.0	
	Exposure Score	Very High		
	Overall Vulnerability Rank	Very High		

Low
Moderate
High
Very High

Common bottlenose dolphin (GoMx BSE/Caloosahatchee River Stock)

Tursiops truncatus

CVA Results Summary

Overall Climate Vulnerability Rank: Very High (100% certainty from bootstrap analysis).

Climate Exposure: Very High. Six exposure factors scored greater than 3.0: Circulation (3.93), Dissolved oxygen (Standard anomaly) (3.93), Sea level rise (3.93), Sea Surface Temperature (Standard anomaly) (3.93), Air Temperature (Standard anomaly) (3.87), and Ocean pH (Standard anomaly) (3.87)

Biological Sensitivity: Very High. Three sensitivity attributes scored greater than 3.5: Migration (3.93), Site Fidelity (3.93), and Home Range (3.73).

Distributional Response: High

Abundance Response: High

Phenology Response: High

Data Quality: 85% of the data quality scores were 2 or greater, 64% of sensitivity attribute data quality scores were 2 or higher.

Background Information

Stock Description

This stock includes common bottlenose dolphins in the Caloosahatchee River along the Gulf of Mexico coast of Florida (Hayes et al. 2019).

Prey/Diet Specificity

Generally, common bottlenose dolphins consume a large variety of fish and/or squid (Wells and Scott 2018). Common bottlenose dolphins seem to show a consistent preference for noise-producing fishes (e.g., sciaenids, toadfish; Berens McCabe et al. 2010; Bowen-Stevens et al. 2021). In Sarasota Bay, FL, Rossman et al. (2015) found some individual common bottlenose dolphins to show diet specialization despite the broader study group showing a generalist diet. Specialization can be influenced by locally abundant prey species (Shane et al. 1986; Vollmer and Rosel 2013) or can result from resource partitioning amongst sympatric populations (e.g., Torres and Read 2009; Wilson et al. 2017).

Habitat Specificity

Common bottlenose dolphins are found in a variety of marine and estuarine habitats (Wells and Scott 2018).

Site Fidelity

Multi-year site fidelity in at least the mouth of the Caloosahatchee River has been demonstrated from photographic identification surveys of the region (Wells et al. 1997; Bassos-Hull et al. 2013). Other Gulf of Mexico bay, sound, and estuary (BSE) stocks show site fidelity (LaBrecque et al. 2015; Hayes et al. 2022), such as in Matagorda Bay, Espiritu Santo Bay, and San Luis Pass in Texas (Maze and Würsig 1999; Lynn and Würsig 2002; Irwin and Würsig 2004); Barataria Bay in Louisiana (Miller 2003; Wells et al. 2017; Takeshita et al. 2021); Mississippi Sound in Mississippi (Hubard et al. 2004; Mackey 2010; Mullin et al. 2017); Charlotte Harbor and Pine Island Sound (Bassos-Hull et al. 2013), Sarasota Bay (Wells 1986a; Wells 1991; Wells 2014), St. Andrew Bay (Balmer et al. 2019), St. Joseph Bay (Balmer et al. 2008; B. Balmer et al. 2018), St. Vincent Sound and Apalachicola Bay (Tyson et al. 2011), and Tampa Bay in Florida (Wells et al. 1996b; Urian et al. 2009).

Lifetime Reproductive Potential

Taylor et al. (2007) reported the interbirth interval of common bottlenose dolphins as 3.80 years, though other studies have shown it to be closer to three years (Leatherwood 1977; Wells and Scott 1999; Connor et al. 2000; Wells 2003).

Generally, females reach sexual maturity before males, with females reaching sexual maturity at 5–13 years (Sergeant et al. 1973; Perrin and Reilly 1984; Wells et al. 1987; Mead and Potter 1990; Mann et al. 2000; Wells 2000; Wells and Scott 2018).

Females reproduce through most of their adult lives (Leatherwood 1977; Hohn et al. 1989; Wells and Scott 1999; Wells 2000; Wells 2003), but may produce fewer calves after they reach 30 years of age (Wells 2000). Sarasota Bay common bottlenose dolphins can give birth when they are as young as 6 years of age, and can continue until they are 48 years old (Wells and Scott 2018). Reproductive lifespan would therefore be up to 42 years based on long-term observation studies (Wells 2014; Wells and Scott 2018).

Studies from Sarasota Bay, Florida have found that female common bottlenose dolphins can live up to 67 years (Wells and Scott 2018). Studies from the Texas coast suggest female common bottlenose dolphins can live up to 41 years (Fernandez and Hohn 1998). Studies from the Mississippi coast suggest female common bottlenose dolphins can live up to 30 years (Mattson et al. 2006).

Generation Length

Taylor et al. (2007) reported common bottlenose dolphin generation length of 20.6-21.1 years.

Reproductive Plasticity

Common bottlenose dolphin calving is generally diffusely seasonal, with one or more peaks, although births have been reported from all seasons (Vollmer and Rosel 2013; Wells and Scott 2018). Calf sightings peak in the spring and summer months in Florida (Wells et al. 1987), Mississippi/Louisiana (Miller et al. 2010; Miller et al. 2013), and Texas (McHugh 1989; Henderson et al. 2007).

Migration

The bay, sound, and estuary stocks in the Gulf of Mexico are thought to primarily comprise stable resident communities. However, transient or seasonal movements of some individuals into and out of a BSE stock area are recorded in some areas (LaBrecque et al. 2015; Hayes et al. 2022). Molecular genetic data support that some BSE stocks are genetically distinct from one another, and furthermore are differentiated from adjacent coastal populations (e.g., Sellas et al. 2005; Rosel et al. 2017). For example, Toms (2019) found fine-scale genetic population structure among five inshore systems in the Florida Panhandle and variable migration rates among populations.

Year-round residency has been reported for common bottlenose dolphins (Hayes et al. 2022), such as in Florida (Irvine and Wells 1972; Irvine et al. 1981; Wells 1986a; Wells 1986b; Wells et al. 1987; Scott et al. 1990; Wells 1991; Wells et al. 1996a; Wells et al. 1996b; Wells et al. 1997; Wells 2003; Balmer et al. 2008; Urian et al. 2009; Tyson et al. 2011; Bassos-Hull et al. 2013; B. Balmer et al. 2018; Balmer et al. 2019; Toms 2019), Texas (Shane 1990; Bräger 1993; Bräger et al. 1994; Fertl 1994; Weller 1998; Maze and Würsig 1999; Lynn and Würsig 2002; Irwin and Würsig 2004; Shane 2004), and Louisiana/Mississippi (Hubard et al. 2004; Mullin et al. 2017; Takeshita et al. 2021).

Home Range

The home ranges of individuals within the Gulf of Mexico BSE stocks are considered to generally include the areas within the stock boundaries and brief excursions beyond the stock boundaries (Irvine et al. 1981; Wells 1986a; Wells 1986b; Wells 1991; Wells et al. 1996a; Wells et al. 1996b; Balmer et al. 2008; Wells 2014), although some individuals show preference for small areas within the stock boundaries (Lynn and Würsig 2002).

Stock Abundance

A best estimate of the abundance for this stock is unavailable (Hayes et al. 2019). The most recent survey, conducted by Scott et al. (1989) between 1983 and 1986, sighted 0 dolphins within the Caloosahatchee River.

Stock Abundance Trend

Data are insufficient to complete an abundance trend analysis for this stock (Hayes et al. 2019).

Cumulative Stressors

Common bottlenose dolphins face a wide range of individual and cumulative natural and anthropogenic threats (see reviews by Vollmer and Rosel 2013; Wells and Scott 2018; and Wells et al. 2019). These include such threats as shark predation, harmful algal blooms, recreational and commercial fishing gear (Adimey et al. 2014; McHugh et al. 2021), vessel traffic, pollution, habitat alteration, and provisioning.

The nearshore and estuarine habitats along the Gulf of Mexico coast include waters adjacent to areas of high human population, some of which are highly industrialized, and areas of heavy agricultural impact. Studies of bottlenose dolphin tissues from southwest Florida have shown persistent organic pollutant (POP) in blubber and blood samples (Kucklick et al. 2011; Balmer et al. 2015; J. Balmer et al. 2018).

Similarly, mercury has been detected in common bottlenose dolphin tissues in Florida Gulf Coast waters (McCormack et al. 2020a, 2020b, 2022).

An Unusual Mortality Event (UME) was declared in 1990 for the Gulf of Mexico from Florida to Texas, with more than 300 common bottlenose dolphins stranding due to an undetermined cause, although morbillivirus and cold temperatures may have contributed (Litz et al. 2014). This stock was also within the range of the 2005–2006 UME in central west Florida caused by brevetoxin (Litz et al. 2014).

Further Reading

Hayes, S.A., E. Josephson, K. Maze-Foley, P.E. Rosel, and J. Wallace. 2022. U.S. Atlantic and Gulf of Mexico marine mammal stock assessments 2021. National Oceanic and Atmospheric Administration, National Marine Fisheries Service, Northeast Fisheries Science Center: Woods Hole, MA. NOAA Technical Memorandum NMFS-NE-288. 380 p. doi: 10.25923/6tt7-kc16

Wells, R.S. and M.D. Scott. 2018. Bottlenose Dolphin, *Tursiops truncatus*, Common Bottlenose Dolphin. In: Würsig, B., J.G.M. Thewissen, and K.M. Kovacs (Eds.), Encyclopedia of Marine Mammals (Third Edition). Academic Press, London, UK. pp. 118–125. doi: 10.1016/B978-0-12-804327-1.00072-8

Wells, R.S., A. Natoli, and G. Braulik. 2019. *Tursiops truncatus* (errata version published in 2019). The IUCN Red List of Threatened Species 2019: e.T22563A156932432. https://dx.doi.org/10.2305/IUCN.UK.2019-1.RLTS.T22563A156932432.en

References

Adimey, N.M., C.A. Hudak, J.R. Powell, K. Bassos-Hull, A. Foley, N.A. Farmer, L. White, and K. Minch. 2014. Fishery gear interactions from stranded bottlenose dolphins, Florida manatees and sea turtles in Florida, U.S.A. Marine Pollution Bulletin 81(1):103–115. doi: 10.1016/j.marpolbul.2014.02.008

Balmer, B., T. McDonald, F. Hornsby, J. Adams, J. Allen, A. Barleycorn, and L. Schwacke. 2018. Long-term trends in a northern Gulf of Mexico common bottlenose dolphin (*Tursiops truncatus*) population in the wake of the Deepwater Horizon oil spill. Journal of Cetacean Research and Management 18:1–9. doi: 10.47536/jcrm.v18i1.409

Balmer, B., S. Watwood, B. Quigley, T. Speakman, K. Barry, K. Mullin, P. Rosel, C. Sinclair, E. Zolman, and L. Schwacke. 2019. Common bottlenose dolphin (*Tursiops truncatus*) abundance and distribution patterns in St. Andrew Bay, Florida, USA. Aquatic Conservation: Marine and Freshwater Ecosystems 29(3):486–498. doi: 10.1002/aqc.3001

Balmer, B.C., R.S. Wells, S.M. Nowacek, D.P. Nowacek, L.H. Schwacke, W.A. McLellan, F.S. Scharf, T.K. Rowles, L.J. Hansen, T.R. Spradlin, and D.A. Pabst. 2008. Seasonal abundance and distribution patterns of common bottlenose dolphins (*Tursiops truncatus*) near St. Joseph Bay, Florida, USA. Journal of Cetacean Research and Management 10(2):157–167.

Balmer, B.C., G.M. Ylitalo, L.E. McGeorge, K.A. Baugh, D. Boyd, K.D. Mullin, P.E. Rosel, C. Sinclair, R.S. Wells, E.S. Zolman, and L.H. Schwacke. 2015. Persistent organic pollutants (POPs) in blubber of common bottlenose dolphins (*Tursiops truncatus*) along the northern Gulf of Mexico coast, USA. Science of the Total Environment 527-528:306–312. doi: 10.1016/j.scitotenv.2015.05.016

Balmer, J.E., G.M. Ylitalo, T.K. Rowles, K.D. Mullin, R.S. Wells, F.I. Townsend, R.W. Pearce, J.L. Bolton, E.S. Zolman, B.C. Balmer, and L.H. Schwacke. 2018. Persistent organic pollutants (POPs) in blood and blubber of common bottlenose dolphins (*Tursiops truncatus*) at three northern Gulf of Mexico

sites following the Deepwater Horizon oil spill. Science of the Total Environment 621:130–137. doi: 10.1016/j.scitotenv.2017.11.209

Bassos-Hull, K., R.M. Perrtree, C.C. Shepard, S. Schilling, A.A. Barleycorn, J.B. Allen, B.C. Balmer, W.E. Pine, and R.S. Wells. 2013. Long-term site fidelity and seasonal abundance estimates of common bottlenose dolphins (*Tursiops truncatus*) along the southwest coast of Florida and responses to natural perturbations. Journal of Cetacean Research and Management 13(1):19–30. doi: 10.47536/jcrm.v13i1.551

Berens McCabe, E.J., D.P. Gannon, N.B. Barros, and R.S. Wells. 2010. Prey selection by resident common bottlenose dolphins (*Tursiops truncatus*) in Sarasota Bay, Florida. Marine Biology 157(5):931–942. doi: 10.1007/s00227-009-1371-2

Bowen-Stevens, S.R., D.P. Gannon, R.A. Hazelkorn, G. Lovewell, K.M. Volker, S. Smith, M.C. Tumlin, and J. Litz. 2021. Diet of common bottlenose dolphins, *Tursiops truncatus*, that stranded in and near Barataria Bay, Louisiana, 2010–2012. Southeastern Naturalist 20(1). doi: 10.1656/058.020.0113

Bräger, S. 1993. Diurnal and seasonal behavior patterns of bottlenose dolphins (*Tursiops truncatus*). Marine Mammal Science 9:434–438. doi: 10.1111/j.1748-7692.1993.tb00477.x

Bräger, S., B. Würsig, A. Acevedo, and T. Henningsen. 1994. Association patterns of bottle-nosed dolphins (*Tursiops-truncatus*) in Galveston Bay, Texas. Journal of Mammalogy 75(2):431–437. doi: 10.2307/1382564

Connor, R.C., R.S. Wells, J. Mann, and A.J. Read. 2000. The bottlenose dolphin: Social relationships in a fission-fusion society. In: Mann, J., R. Connor, P. Tyack, and H. Whitehead (Eds.), Cetacean Societies: Field Studies of Dolphins and Whales. University of Chicago Press, Chicago, IL. pp. 91–126.

Fernandez, S. and A.A. Hohn. 1998. Age, growth, and calving season of bottlenose dolphins, *Tursiops truncatus*, off coastal Texas. Fishery Bulletin 96(2):357–365.

Fertl, D. 1994. Occurrence patterns and behavior of bottlenose dolphins (*Tursiops truncatus*) in the Galveston ship channel, Texas. Texas Journal of Science 46(4):299–318.

Hayes, S.A., E. Josephson, K. Maze-Foley, and P.E. Rosel. 2019. US Atlantic and Gulf of Mexico marine mammal stock assessments - 2018. National Oceanic and Atmospheric Administration, National Marine Fisheries Service, Northeast Fisheries Science Center: Woods Hole, MA. NOAA Technical Memorandum NMFS-NE-258. 298 p. doi: 10.25923/9rrd-tx13

Hayes, S.A., E. Josephson, K. Maze-Foley, P.E. Rosel, and J. Wallace. 2022. U.S. Atlantic and Gulf of Mexico marine mammal stock assessments 2021. National Oceanic and Atmospheric Administration, National Marine Fisheries Service, Northeast Fisheries Science Center: Woods Hole, MA. NOAA Technical Memorandum NMFS-NE-288. 380 p. doi: 10.25923/6tt7-kc16

Henderson, E.E. and B. Würsig. 2007. Behavior patterns of bottlenose dolphins in San Luis Pass, Texas. Gulf of Mexico Science 25(2):153–161. doi: 10.18785/goms.2502.06

Hohn, A.A., M.D. Scott, R.S. Wells, J.C. Sweeney, and A.B. Irvine. 1989. Growth layers in teeth from known-age, free-ranging bottlenose dolphins. Marine Mammal Science 5(4):315–342. doi: 10.1111/j.1748-7692.1989.tb00346.x

Hubard, C.W., K. Maze-Foley, K.D. Mullin, and W.W. Schroeder. 2004. Seasonal abundance and site fidelity of bottlenose dolphins (*Tursiops truncatus*) in Mississippi Sound. Aquatic Mammals 30(2):299–310. doi: 10.1578/am.30.2.2004.299

Irvine, A.B., M.D. Scott, R.S. Wells, and J.H. Kaufmann. 1981. Movements and activities of the Atlantic bottlenose dolphin, *Tursiops truncatus*, near Sarasota, Florida. Fishery Bulletin 79(4):671–688.

Irvine, B. and R.S. Wells. 1972. Results of attempts to tag Atlantic bottlenosed dolphins (*Tursiops truncatus*). Cetology 13:1–5.

Irwin, L.-J. and B. Würsig. 2004. A small resident community of bottlenose dolphins, *Tursiops truncatus*, in Texas: Monitoring recommendations. Gulf of Mexico Science 22(1):13–21. doi: 10.18785/goms.2201.02

Kucklick, J., L. Schwacke, R. Wells, A. Hohn, A. Guichard, J. Yordy, L. Hansen, E. Zolman, R. Wilson, J. Litz, D. Nowacek, T. Rowles, R. Pugh, B. Balmer, C. Sinclair, and P. Rosel. 2011. Bottlenose dolphins as indicators of persistent organic pollutants in the western North Atlantic Ocean and Northern Gulf of Mexico. Environmental Science & Technology 45(10):4270–4277. doi: 10.1021/es1042244

LaBrecque, E., C. Curtice, J. Harrison, S.M. Van Parijs, and P.N. Halpin. 2015. 3. Biologically Important Areas for cetaceans within U.S. Waters – Gulf of Mexico Region. Aquatic Mammals 41(1):30–38. doi: 10.1578/am.41.1.2015.30

Leatherwood, S. 1977. Some preliminary impressions on the numbers and social behavior of free-swimming bottlenosed dolphin calves (*Tursiops truncatus*) in the northern Gulf of Mexico. In: Ridgway, S.H. and K. Bernirschke (Eds.), Breeding Dolphins; Present Status, Suggestions for the Future. Final report to the US Marine Mammal Commission, Contract MM6AC009, MMC-76/07, Washington, DC. pp. 143–167.

Litz, J.A., M.A. Baran, S.R. Bowen-Stevens, R.H. Carmichael, K.M. Colegrove, L.P. Garrison, S.E. Fire, E.M. Fougeres, R. Hardy, S. Holmes, W. Jones, B.E. Mase-Guthrie, D.K. Odell, P.E. Rosel, J.T. Saliki, D.K. Shannon, S.F. Shippee, S.M. Smith, E.M. Stratton, M.C. Tumlin, H.R. Whitehead, G.A. Worthy, and T.K. Rowles. 2014. Review of historical unusual mortality events (UMEs) in the Gulf of Mexico (1990–2009): providing context for the multi-year northern Gulf of Mexico cetacean UME declared in 2010. Diseases of Aquatic Organisms 112(2):161–175. doi: 10.3354/dao02807

Lynn, S.K. and B. Würsig. 2002. Summer movement patterns of bottlenose dolphins in a Texas bay. Gulf of Mexico Science 20(1):25–37. doi: 10.18785/goms.2001.03

Mackey, A.D. 2010. Site fidelity and association patterns of bottlenose dolphins (*Tursiops truncatus*) in the Mississippi Sound. University of Southern Mississippi. Thesis. 133 p.

Mann, J., R.C. Connor, L.M. Barre, and M.R. Heithaus. 2000. Female reproductive success in bottlenose dolphins (*Tursiops* sp.): life history, habitat, provisioning, and group-size effects. Behavioral Ecology 11(2):210–219. doi: 10.1093/beheco/11.2.210

Mattson, M.C., K.D. Mullin, G.W. Ingram, and W. Hoggard. 2006. Age structure and growth of the bottlenose dolphin (*Tursiops truncatus*) from strandings in the Mississippi sound region of the north-central Gulf of Mexico from 1986 to 2003. Marine Mammal Science 22(3):654–666. doi: 10.1111/j.1748-7692.2006.00057.x

Maze, K.S. and B. Würsig. 1999. Bottlenosed dolphins of San Luis Pass, Texas: Occurrence patterns, site-fidelity, and habitat use. Aquatic Mammals 25:91–104.

McCormack, M.A., F. Battaglia, W.E. McFee, and J. Dutton. 2020a. Mercury concentrations in blubber and skin from stranded bottlenose dolphins (*Tursiops truncatus*) along the Florida and Louisiana coasts (Gulf of Mexico, USA) in relation to biological variables. Environmental Research 180:108886. doi: 10.1016/j.envres.2019.108886

McCormack, M.A., B.P. Jackson, and J. Dutton. 2020b. Relationship between mercury and selenium concentrations in tissues from stranded odontocetes in the northern Gulf of Mexico. Science of the Total Environment 749:141350. doi: 10.1016/j.scitotenv.2020.141350

McCormack, M.A., W.H. Nowlin, and J. Dutton. 2022. Effect of trophic position on mercury concentrations in bottlenose dolphins (*Tursiops truncatus*) from the northern Gulf of Mexico. Environmental Research 204(Pt B):112124. doi: 10.1016/j.envres.2021.112124

McHugh, K.A., A.A. Barleycorn, J.B. Allen, K. Bassos-Hull, G. Lovewell, D. Boyd, A. Panike, C. Cush, D. Fauquier, B. Mase, R.C. Lacy, M.R. Greenfield, D.I. Rubenstein, A. Weaver, A. Stone, L. Oliver, K. Morse, and R.S. Wells. 2021. Staying alive: Long-term success of bottlenose dolphin

interventions in southwest Florida. Frontiers in Marine Science 7:624729. doi: 10.3389/fmars.2020.624729

McHugh, M.B. 1989. Population numbers and feeding behavior of the Atlantic bottlenose dolphin (*Tursiops truncatus*) near Aransas Pass, Texas. University of Texas at Austin. Thesis. 96 p.

Mead, J.G. and C.W. Potter. 1990. Natural history of bottlenose dolphins along the central Atlantic coast of the United States. In: Leatherwood, S. and R.R. Reeves (Eds.), The Bottlenose Dolphin. Academic Press, San Diego, CA. pp. 165–195.

Miller, C.E. 2003. Abundance trends and environmental habitat usage patterns of bottlenose dolphins (*Tursiops truncatus*) in lower Barataria and Caminada bays, Louisiana. Louisiana State University and Agricultural & Mechanical College. Dissertation. 125 p.

Miller, L.J., A.D. Mackey, T. Hoffland, M. Solangi, and S.A. Kuczaj. 2010. Potential effects of a major hurricane on Atlantic bottlenose dolphin (*Tursiops truncatus*) reproduction in the Mississippi Sound. Marine Mammal Science 26(3):707–715. doi: 10.1111/j.1748-7692.2010.00371.x

Miller, L.J., A.D. Mackey, M. Solangi, and S.A. Kuczaj. 2013. Population abundance and habitat utilization of bottlenose dolphins in the Mississippi Sound. Aquatic Conservation: Marine and Freshwater Ecosystems 23(1):145–151. doi: 10.1002/aqc.2278

Mullin, K.D., T. McDonald, R.S. Wells, B.C. Balmer, T. Speakman, C. Sinclair, E.S. Zolman, F. Hornsby, S.M. McBride, K.A. Wilkinson, and L.H. Schwacke. 2017. Density, abundance, survival, and ranging patterns of common bottlenose dolphins (*Tursiops truncatus*) in Mississippi Sound following the Deepwater Horizon oil spill. PLoS ONE 12(10):e0186265. doi: 10.1371/journal.pone.0186265

Perrin, W.F. and S.B. Reilly. 1984. Reproductive parameters of dolphins and small whales of the family Delphinidae. In: Perrin, W.F., R.L. Brownell, Jr., and D.P. DeMaster (Eds.), Reproduction in Whales, Dolphins, and Porpoises. pp. 97–134.

Rosel, P.E., L.A. Wilcox, C. Sinclair, T.R. Speakman, M.C. Tumlin, J.A. Litz, and E.S. Zolman. 2017. Genetic assignment to stock of stranded common bottlenose dolphins in southeastern Louisiana after the Deepwater Horizon oil spill. Endangered Species Research 33:221–234. doi: 10.3354/esr00780

Rossman, S., P.H. Ostrom, M. Stolen, N.B. Barros, H. Gandhi, C.A. Stricker, and R.S. Wells. 2015. Individual specialization in the foraging habits of female bottlenose dolphins living in a trophically diverse and habitat rich estuary. Oecologia 178(2):415–425. doi: 10.1007/s00442-015-3241-6

Scott, G.P., D.M. Burn, L.J. Hansen, and R.E. Owen. 1989. Estimates of bottlenose dolphin abundance in the Gulf of Mexico from regional aerial surveys. National Oceanic and Atmospheric Administration, National Marine Fisheries Service, Southeast Fisheries Center: Miami, FL. Southeast Fisheries Center Contribution CRD-88/89-07. 77 p.

Scott, M.D. and S.J. Chivers. 1990. Distribution and herd structure of bottlenose dolphins in the eastern tropical Pacific Ocean. In: Leatherwood, S. and R.R. Reeves (Eds.), The Bottlenose Dolphin. Academic Press, San Diego, CA. pp. 387–402.

Sellas, A.B., R.S. Wells, and P.E. Rosel. 2005. Mitochondrial and nuclear DNA analyses reveal fine scale geographic structure in bottlenose dolphins (*Tursiops truncatus*) in the Gulf of Mexico. Conservation Genetics 6(5):715–728. doi: 10.1007/s10592-005-9031-7

Sergeant, D.E., D.K. Caldwell, and M.C. Caldwell. 1973. Age, growth, and maturity of bottlenosed dolphin (*Tursiops-truncatus*) from northeast Florida. Journal of the Fisheries Research Board of Canada 30(7):1009–1011. doi: 10.1139/f73-165

Shane, S.H. 1990. Behavior and ecology of the bottlenose dolphin at Sanibel Island, Florida. In: Leatherwood, S. and R.R. Reeves (Eds.), The Bottlenose Dolphin. Academic Press, San Diego, CA. pp. 245–265. doi: 10.1016/B978-0-12-440280-5.50016-0

Shane, S.H. 2004. Residence patterns, group characteristics, and association patterns of bottlenose dolphins near Sanibel Island, Florida. Gulf of Mexico Science 22(1):1–12. doi: 10.18785/goms.2201.01

Shane, S.H., R.S. Wells, and B. Würsig. 1986. Ecology, behavior and social organization of the bottlenose dolphin: A review. Marine Mammal Science 2:34–63. doi: 10.1111/j.1748-7692.1986.tb00026.x

Takeshita, R., B.C. Balmer, F. Messina, E.S. Zolman, L. Thomas, R.S. Wells, C.R. Smith, T.K. Rowles, and L.H. Schwacke. 2021. High site-fidelity in common bottlenose dolphins despite low salinity exposure and associated indicators of compromised health. PLoS ONE 16(9):e0258031. doi: 10.1371/journal.pone.0258031

Taylor, B.L., S.J. Chivers, J. Larese, and W.F. Perrin. 2007. Generation length and percent mature estimates for IUCN assessments of cetaceans. National Oceanic and Atmospheric Administration, National Marine Fisheries Service, Southwest Fisheries Science Center: La Jolla, CA. Administrative Report LJ-07-01. 24 p.

Toms, C. 2019. Filling the gaps: Common bottlenose dolphin (*Tursiops truncatus*) population dynamics, structure, and connectivity within Florida panhandle bays, sounds, and estuaries. University of Central Florida. Dissertation. 373 p.

Torres, L.G. and A.J. Read. 2009. Where to catch a fish? The influence of foraging tactics on the ecology of bottlenose dolphins (*Tursiops truncatus*) in Florida Bay, Florida. Marine Mammal Science 25(4):797–815. doi: 10.1111/j.1748-7692.2009.00297.x

Tyson, R.B., S.M. Nowacek, and D.P. Nowacek. 2011. Community structure and abundance of bottlenose dolphins *Tursiops truncatus* in coastal waters of the northeast Gulf of Mexico. Marine Ecology Progress Series 438:253–265. doi: 10.3354/meps09292

Urian, K.W., S. Hofmann, R.S. Wells, and A.J. Read. 2009. Fine-scale population structure of bottlenose dolphins (*Tursiops truncatus*) in Tampa Bay, Florida. Marine Mammal Science 25(3):619–638. doi: 10.1111/j.1748-7692.2009.00284.x

Vollmer, N.L. and P.E. Rosel. 2013. A review of common bottlenose dolphins (*Tursiops truncatus truncatus*) in the Northern Gulf of Mexico: Population biology, potential threats, and management. Southeastern Naturalist 12(m6):1–43. doi: 10.1656/058.012.m601

Weller, D.W. 1998. Global and regional variation in the biology and behavior of bottlenose dolphins. Texas A&M University. Dissertation. 142 p.

Wells, R.S. 1986a. Population structure of bottlenose dolphins: Behavioral studies along the central west coast of Florida. Contract Report to National Marine Fisheries Service, Southeast Fisheries Center. Contract No. 45-WCNF-5-003BB. 58 p.

Wells, R.S. 1986b. Structural aspects of dolphin societies. University of California, Santa Cruz. Dissertation. 234 p.

Wells, R.S. 1991. The role of long-term study in understanding the social structure of a bottlenose dolphin community. In: Pryor, K. and K. Norris (Eds.), Dolphin Societies: Discoveries and Puzzles. University of California Press, Berkeley, CA. pp. 199–226.

Wells, R.S. 2000. Reproduction in wild bottlenose dolphins: Overview of patterns observed during a long-term study. In: Duffield, D. and T. Robeck (Eds.), Bottlenose Dolphin Reproduction Workshop. AZA Marine Mammal Taxon Advisory Group, Silver Spring, MD. pp. 57–74.

Wells, R.S. 2003. Dolphin social complexity: Lessons from long-term study and life history. In: de Waal, F.B.M. and P.L. Tyack (Eds.), Animal Social Complexity: Intelligence, Culture, and Individualized Societies Harvard University Press, Cambridge, MA. pp. 32–56.

Wells, R.S. 2014. Social structure and life history of bottlenose dolphins near Sarasota Bay, Florida: Insights from four decades and five generations. Primates and cetaceans. pp. 149–172. doi: 10.1007/978-4-431-54523-1_8

Wells, R.S., M.K. Bassos, K.W. Urian, W.J. Carr, and M.D. Scott. 1996. Low-level monitoring of bottlenose dolphins, *Tursiops truncatus*, in Charlotte Harbor, Florida: 1990-1994. National Oceanic and Atmospheric Administration, National Marine Fisheries Service, Southeast Fisheries Science Center: Miami, FL. NOAA Technical Memorandum NMFS-SEFSC-384. 86 p.

Wells, R.S., M.K. Bassos, K.W. Urian, S.H. Shane, E.C.G. Owen, C.F. Weiss, W.J. Carr, and M.D. Scott. 1997. Low-level monitoring of bottlenose dolphins, *Tursiops truncatus*, in Pine Island Sound, Florida: 1996. National Oceanic and Atmospheric Administration, National Marine Fisheries Service, Southeast Fisheries Science Center: Miami, FL. Southeast Fisheries Center Contribution No. 40-WCNF601958. 86 p.

Wells, R.S., A. Natoli, and G. Braulik. 2019. *Tursiops truncatus* (errata version published in 2019). *The IUCN Red List of Threatened Species* 2019: e.T22563A156932432. https://dx.doi.org/10.2305/IUCN.UK.2019-1.RLTS.T22563A156932432.en

Wells, R.S., L.H. Schwacke, T.K. Rowles, B.C. Balmer, E. Zolman, T. Speakman, F.I. Townsend, M.C. Tumlin, A. Barleycorn, and K.A. Wilkinson. 2017. Ranging patterns of common bottlenose dolphins *Tursiops truncatus* in Barataria Bay, Louisiana, following the Deepwater Horizon oil spill. Endangered Species Research 33:159–180. doi: 10.3354/esr00732

Wells, R.S. and M.D. Scott. 1999. Bottlenose dolphin *Tursiops truncatus* (Montagu, 1821). In: Ridgway, S.H. and R. Harrison (Eds.), Handbook of marine mammals. Volume 6: The second book of dolphins and the porpoises. Academic Press, San Diego, CA. pp. 137–182.

Wells, R.S. and M.D. Scott. 2018. Bottlenose dolphin, *Tursiops truncatus*, common bottlenose dolphin. In: Würsig, B., J.G.M. Thewissen, and K.M. Kovacs (Eds.), Encyclopedia of Marine Mammals (Third Edition). Academic Press, London, UK. pp. 118–125. doi: 10.1016/B978-0-12-804327-1.00072-8

Wells, R.S., M.D. Scott, and A.B. Irvine. 1987. The social structure of free-ranging bottlenose dolphins. In: Genoways, H.H. (Ed.), Current Mammalogy. Plenum Press, New York, NY. pp. 247–305.

Wells, R.S., K.W. Urian, A.J. Read, M.K. Bassos, W.J. Carr, and M.D. Scott. 1996. Low-level monitoring of bottlenose dolphins, *Tursiops truncatus*, in Tampa Bay, Florida: 1988-1993. National Oceanic and Atmospheric Administration, National Marine Fisheries Service, Southeast Fisheries Science Center: Miami, FL. NOAA Technical Memorandum NMFS-SEFSC-385. 36 p.

Wilson, R.M., R.B. Tyson, J.A. Nelson, B.C. Balmer, J.P. Chanton, and D.P. Nowacek. 2017. Niche differentiation and prey selectivity among common bottlenose dolphins (*Tursiops truncatus*) sighted in St. George Sound, Gulf of Mexico. Frontiers in Marine Science 4:235. doi: 10.3389/fmars.2017.00235

Common bottlenose dolphin − *Tursiops truncatus*

GoMx BSE/Choctawhatchee Bay Stock

Overall Vulnerability Rank = Very High

Biological Sensitivity = Very High

Climate Exposure = Very High

Data Quality = 93% of scores ≥ 2

	Tursiops truncatus	Expert Scores	Data Quality	Expert Scores Plots (Portion by Category)
Sensitivity attributes	Prey/Diet Specificity	1.8	2.0	
	Habitat Specificity	3.5	2.0	
	Site Fidelity	4.0	2.5	
	Lifetime Reproductive Potential	1.3	2.0	
	Generation Length	2.8	2.0	
	Reproductive Plasticity	1.4	2.0	
	Migration	3.9	2.2	
	Home Range	3.5	2.2	
	Stock Abundance	3.0	2.5	
	Stock Abundance Trend	2.1	1.2	
	Cumulative Stressors	3.2	2.0	
	Sensitivity Score	Very High		
Exposure variables	Sea Surface Temperature (Change in mean)	4.0	3.0	
	Sea Surface Temperature (Change in variability)	1.0	3.0	
	Air Temperature (Change in mean)	4.0	3.0	
	Air Temperature (Change in variability)	1.0	3.0	
	Precipitation (Change in mean)	1.0	3.0	
	Precipitation (Change in variability)	1.0	3.0	
	Sea Surface Salinity (Change in mean)	2.0	3.0	
	Sea Surface Salinity (Change in variability)	2.0	3.0	
	Ocean pH (Change in mean)	4.0	3.0	
	Ocean pH (Change in variability)	1.0	3.0	
	Sea ice coverage (Change in mean)	1.0	3.0	
	Sea ice coverage (Change in variability)	1.0	3.0	
	Dissolved oxygen (Change in mean)	4.0	3.0	
	Dissolved oxygen (Change in variability)	1.0	3.0	
	Circulation	4.0	1.2	
	Sea level rise	3.6	3.0	
	Exposure Score	Very High		
Overall Vulnerability Rank		Very High		

Low
Moderate
High
Very High

Common bottlenose dolphin (GoMx BSE/Choctawhatchee Bay Stock)

Tursiops truncatus

CVA Results Summary

Overall Climate Vulnerability Rank: Very High (100% certainty from bootstrap analysis).

Climate Exposure: Very High. Six exposure factors scored greater than 3.5: Air Temperature (Standard anomaly) (4.00), Dissolved oxygen (Standard anomaly) (4.00), Ocean pH (Standard anomaly) (4.00), Sea Surface Temperature (Standard anomaly) (4.00), Circulation (3.95), and Sea level rise (3.65)

Biological Sensitivity: Very High. Four sensitivity attributes scored greater than 3.5: Site Fidelity (3.95), Migration (3.90), Habitat Specificity (3.55), and Home Range (3.55)

Distributional Response: High

Abundance Response: Very High

Phenology Response: High

Data Quality: 93% of the data quality scores were 2 or greater, 91% of sensitivity attribute data quality scores were 2 or higher.

Background Information

Stock Description

This stock includes common bottlenose dolphins in Choctawhatchee Bay in the northeast Gulf of Mexico along the Florida coast. This stock is bounded by Point Washington and Jolly Bay in the east and Fort Walton Beach in the west (Waring et al. 2016).

Prey/Diet Specificity

Generally, common bottlenose dolphins consume a large variety of fish and/or squid (Barros and Odell 1990; Mead and Potter 1990; Wells and Scott 2018). Common bottlenose dolphins seem to show a consistent preference for sound-producing fishes (e.g., sciaenids, toadfish; Berens McCabe et al. 2010; Bowen-Stevens et al. 2021). In Sarasota Bay, FL, Rossman et al. (2015) found some individual common bottlenose dolphins to show diet specialization despite the broader study group showing a generalist diet. Specialization can be influenced by locally abundant prey species (Shane et al. 1986; Vollmer and Rosel 2013) or can result from resource partitioning amongst sympatric populations (e.g., Torres and Read 2009; Wilson et al. 2017).

Habitat Specificity

Common bottlenose dolphins are found in a variety of marine and estuarine habitats (Wells and Scott 2018).

Site Fidelity

No specific site fidelity information was found for this stock. However, common bottlenose dolphins in other Gulf of Mexico bay, sound, and estuary (BSE) stocks show site fidelity (LaBrecque et al. 2015; Hayes et al. 2022), such as in Matagorda Bay, Espiritu Santo Bay, and San Luis Pass in Texas (Maze and Würsig 1999; Lynn and Würsig 2002; Irwin and Würsig 2004); Barataria Bay in Louisiana (Miller 2003; Wells et al. 2017; Takeshita et al. 2021); Mississippi Sound in Mississippi (Hubard et al. 2004; Mackey 2010; Mullin et al. 2017); Charlotte Harbor and Pine Island Sound (Bassos-Hull et al. 2013), Sarasota Bay (Wells 1986a; Wells 1991; Wells 2014), St. Andrew Bay (Balmer et al. 2019), St. Joseph Bay (Balmer et al. 2008; B. Balmer et al. 2018), St. Vincent Sound and Apalachicola Bay (Tyson et al. 2011), and Tampa Bay in Florida (Wells et al. 1996b; Urian et al. 2009)

Lifetime Reproductive Potential

Taylor et al. (2007) reported the interbirth interval of common bottlenose dolphins as 3.80 years, though other studies have shown it to be closer to three years (Leatherwood 1977; Wells and Scott 1999; Connor et al. 2000; Wells 2003).

Generally, females reach sexual maturity before males, with females reaching sexual maturity at 5–13 years (Sergeant et al. 1973; Perrin and Reilly 1984; Wells et al. 1987; Mead and Potter 1990; Mann et al. 2000; Wells 2000; Wells and Scott 2018).

Females reproduce through most of their adult lives (Leatherwood 1977; Hohn et al. 1989; Wells and Scott 1999; Wells 2000; Wells 2003), but may produce fewer calves after they reach 30 years of age (Wells 2000). Sarasota Bay common bottlenose dolphins can give birth when they are as young as 6 years of age, and continue until they are 48 years old (Wells and Scott 2018). Reproductive lifespan would therefore be up to 42 years based on long-term observation studies (Wells 2014; Wells and Scott 2018).

Longitudinal studies from Sarasota Bay, Florida have found that female common bottlenose dolphins can live up to 67 years (Wells and Scott 2018). Studies based on growth layer groups in teeth suggest female common bottlenose dolphins can live to 41 years along the Texas coast (Fernandez and Hohn 1998) and to 30 years along the Mississippi coast (Mattson et al. 2006).

Generation Length

Taylor et al. (2007) reported common bottlenose dolphin generation length of 20.6-21.1 years.

Reproductive Plasticity

Common bottlenose dolphin calving is generally diffusely seasonal, with one or more peaks, although births have been reported from all seasons (Vollmer and Rosel 2013; Wells and Scott 2018). Calf sightings peak in the spring and summer months in Florida (Wells et al. 1987), Mississippi/Louisiana (Miller et al. 2010; Miller et al. 2013), and Texas (McHugh 1989; Henderson et al. 2007).

Migration

The bay, sound, and estuary stocks in the Gulf of Mexico are thought to primarily comprise stable resident communities. However, transient or seasonal movements of some individuals into and out of a BSE stock area are recorded in some areas (LaBrecque et al. 2015; Hayes et al. 2022). Molecular genetic data support that some BSE stocks are genetically distinct from one another, and furthermore are differentiated from adjacent coastal populations (e.g., Sellas et al. 2005; Rosel et al. 2017). For example, Toms (2019) found fine-scale genetic population structure among five inshore systems in the Florida Panhandle and variable migration rates among populations.

Year-round residency has been reported for common bottlenose dolphins (Hayes et al. 2022), such as in Florida (Irvine and Wells 1972; Irvine et al. 1981; Wells 1986a; Wells 1986b; Wells et al. 1987; Scott et al. 1990; Wells 1991; Wells et al. 1996a; Wells et al. 1996b; Wells et al. 1997; Wells 2003; Balmer et al. 2008; Urian et al. 2009; Tyson et al. 2011; Bassos-Hull et al. 2013; B. Balmer et al. 2018; Balmer et al. 2019; Toms 2019), Texas (Shane 1990; Bräger 1993; Bräger et al. 1994; Fertl 1994; Weller 1998; Maze and Würsig 1999; Lynn and Würsig 2002; Irwin and Würsig 2004; Shane 2004), and Louisiana/Mississippi (Hubard et al. 2004; Mullin et al. 2017; Takeshita et al. 2021).

Home Range

The home ranges of individuals within the Gulf of Mexico BSE stocks are considered to generally include the areas within the stock boundaries and brief excursions beyond the stock boundaries (Irvine et al. 1981; Wells 1986a; Wells 1986b; Wells 1991; Wells et al. 1996a; Wells et al. 1996b; Balmer et al. 2008; Wells 2014; Balmer et al. 2019), although some individuals show preference for small areas within the stock boundaries (Lynn and Würsig 2002; Wells et al. 2017).

Stock Abundance

Based on July-August 2007 surveys, the best available abundance estimate of the resident Choctawhatchee Bay Stock is 179 (CV=0.04) (Conn et al. 2011; Waring et al. 2016).

Stock Abundance Trend

Data are insufficient to complete an abundance trend analysis for this stock (Waring et al. 2016).

Cumulative Stressors

Common bottlenose dolphins face a wide range of individual and cumulative natural and anthropogenic threats (see reviews by Vollmer and Rosel 2013; Wells and Scott 2018; and Wells et al. 2019). These include such threats as shark predation, harmful algal blooms, recreational and commercial fishing gear, vessel traffic, pollution, habitat alteration, and provisioning.

Common bottlenose dolphins in Choctawhatchee Bay were possibly exposed to oil and dispersants following the Deepwater Horizon oil spill, which may result in long term reproductive and chronic health effects (NOAA 2011; Michel et al. 2013; Helm et al. 2015; DWH NRDA 2016; Lane et al. 2015; De Guise et al. 2017, 2021; Kellar et al. 2017; Smith et al. 2017; Schwacke et al. 2017). An Unusual Mortality Event (UME), involving primarily bottlenose dolphins, was declared for cetaceans in the northern Gulf of

Mexico from 2010 to 2014, with the Deepwater Horizon oil spill as the primary underlying cause (Litz et al. 2014; Schwacke et al. 2014; Venn-Watson et al. 2015a, 2015b).

The nearshore and estuarine habitats along the Gulf of Mexico coast include waters adjacent to areas of high human population, some of which are highly industrialized, and areas of heavy agricultural impact. Studies of common bottlenose dolphin tissues from the waters along the Florida panhandle have shown persistent organic pollutant (POP) in blubber and blood samples (Kucklick et al. 2011; Balmer et al. 2015; J. Balmer et al. 2018). Similarly, mercury has been detected in common bottlenose dolphin tissues in waters along the Florida panhandle (McCormack et al. 2020a, 2020b, 2022).

UMEs have been declared for common bottlenose dolphins in waters of the Florida panhandle with biotoxins (e.g., brevetoxin from *Karenia brevis*) the suspected or confirmed cause (Twiner et al. 2012; Litz et al. 2014). Dolphin morbillivirus has been detected in populations of the northern Gulf of Mexico, but has not been attributed to large scale die-offs since 1993−1994 (Lipscomb et al. 1996; Litz et al. 2014; Van Bressem et al. 2014; Fauquier et al. 2017). A UME was declared in 1990 for the Gulf of Mexico from Florida to Texas, with more than 300 common bottlenose dolphins stranding due to an undetermined cause, although morbillivirus and cold temperatures may have contributed (Litz et al. 2014).

Further Reading

Waring, G.T., E. Josephson, K. Maze-Foley, and P.E. Rosel. 2016. U.S. Atlantic and Gulf of Mexico Marine Mammal Stock Assessments - 2015. National Oceanic and Atmospheric Administration, National Marine Fisheries Service, Northeast Fisheries Science Center,: Woods Hole, MA. NOAA Technical Memorandum NMFS-NE-238. 501 p. doi: 10.7289/V57S7KTN

Wells, R.S. and M.D. Scott. 2018. Bottlenose Dolphin, *Tursiops truncatus*, Common Bottlenose Dolphin. In: Würsig, B., J.G.M. Thewissen, and K.M. Kovacs (Eds.), Encyclopedia of Marine Mammals (Third Edition). Academic Press, London, UK. pp. 118–125. doi: 10.1016/B978-0-12-804327-1.00072-8

Wells, R.S., A. Natoli, and G. Braulik. 2019. *Tursiops truncatus* (errata version published in 2019). The IUCN Red List of Threatened Species 2019: e.T22563A156932432. https://dx.doi.org/10.2305/IUCN.UK.2019-1.RLTS.T22563A156932432.en

References

Balmer, B., T. McDonald, F. Hornsby, J. Adams, J. Allen, A. Barleycorn, and L. Schwacke. 2018. Long-term trends in a northern Gulf of Mexico common bottlenose dolphin (*Tursiops truncatus*) population in the wake of the Deepwater Horizon oil spill. Journal of Cetacean Research and Management 18:1–9. doi: 10.47536/jcrm.v18i1.409

Balmer, B., S. Watwood, B. Quigley, T. Speakman, K. Barry, K. Mullin, P. Rosel, C. Sinclair, E. Zolman, and L. Schwacke. 2019. Common bottlenose dolphin (*Tursiops truncatus*) abundance and distribution patterns in St. Andrew Bay, Florida, USA. Aquatic Conservation: Marine and Freshwater Ecosystems 29(3):486–498. doi: 10.1002/aqc.3001

Balmer, B.C., R.S. Wells, S.M. Nowacek, D.P. Nowacek, L.H. Schwacke, W.A. McLellan, F.S. Scharf, T.K. Rowles, L.J. Hansen, T.R. Spradlin, and D.A. Pabst. 2008. Seasonal abundance and distribution patterns of common bottlenose dolphins (*Tursiops truncatus*) near St. Joseph Bay, Florida, USA. Journal of Cetacean Research and Management 10(2):157–167.

Balmer, B.C., G.M. Ylitalo, L.E. McGeorge, K.A. Baugh, D. Boyd, K.D. Mullin, P.E. Rosel, C. Sinclair, R.S. Wells, E.S. Zolman, and L.H. Schwacke. 2015. Persistent organic pollutants (POPs) in blubber of common bottlenose dolphins (*Tursiops truncatus*) along the northern Gulf of Mexico coast, USA. Science of the Total Environment 527-528:306–312. doi: 10.1016/j.scitotenv.2015.05.016

Balmer, J.E., G.M. Ylitalo, T.K. Rowles, K.D. Mullin, R.S. Wells, F.I. Townsend, R.W. Pearce, J.L. Bolton, E.S. Zolman, B.C. Balmer, and L.H. Schwacke. 2018. Persistent organic pollutants (POPs) in blood and blubber of common bottlenose dolphins (*Tursiops truncatus*) at three northern Gulf of Mexico sites following the Deepwater Horizon oil spill. Science of the Total Environment 621:130–137. doi: 10.1016/j.scitotenv.2017.11.209

Barros, N.B. and D.K. Odell. 1990. Food habits of bottlenose dolphins in the southeastern United States In: Leatherwood, S. and R.R. Reeves (Eds.), The Bottlenose Dolphin. Academic Press, San Diego, CA. pp. 309–328. doi: 10.1016/B978-0-12-440280-5.50020-2

Bassos-Hull, K., R.M. Perrtree, C.C. Shepard, S. Schilling, A.A. Barleycorn, J.B. Allen, B.C. Balmer, W.E. Pine, and R.S. Wells. 2013. Long-term site fidelity and seasonal abundance estimates of common bottlenose dolphins (*Tursiops truncatus*) along the southwest coast of Florida and responses to natural perturbations. Journal of Cetacean Research and Management 13(1):19–30. doi: 10.47536/jcrm.v13i1.551

Berens McCabe, E.J., D.P. Gannon, N.B. Barros, and R.S. Wells. 2010. Prey selection by resident common bottlenose dolphins (*Tursiops truncatus*) in Sarasota Bay, Florida. Marine Biology 157(5):931–942. doi: 10.1007/s00227-009-1371-2

Bowen-Stevens, S.R., D.P. Gannon, R.A. Hazelkorn, G. Lovewell, K.M. Volker, S. Smith, M.C. Tumlin, and J. Litz. 2021. Diet of common bottlenose dolphins, *Tursiops truncatus*, that stranded in and near Barataria Bay, Louisiana, 2010–2012. Southeastern Naturalist 20(1). doi: 10.1656/058.020.0113

Bräger, S. 1993. Diurnal and seasonal behavior patterns of bottlenose dolphins (*Tursiops truncatus*). Marine Mammal Science 9:434–438. doi: 10.1111/j.1748-7692.1993.tb00477.x

Bräger, S., B. Würsig, A. Acevedo, and T. Henningsen. 1994. Association patterns of bottle-nosed dolphins (*Tursiops-truncatus*) in Galveston Bay, Texas. Journal of Mammalogy 75(2):431–437. doi: 10.2307/1382564

Conn, P.B., A.M. Gorgone, A.R. Jugovich, B.L. Byrd, and L.J. Hansen. 2011. Accounting for transients when estimating abundance of bottlenose dolphins in Choctawhatchee Bay, Florida. Journal of Wildlife Management 75(3):569–579. doi: 10.1002/jwmg.94

Connor, R.C., R.S. Wells, J. Mann, and A.J. Read. 2000. The bottlenose dolphin: Social relationships in a fission-fusion society. In: Mann, J., R. Connor, P. Tyack, and H. Whitehead (Eds.), Cetacean Societies: Field Studies of Dolphins and Whales. University of Chicago Press, Chicago, IL. pp. 91–126.

De Guise, S., M. Levin, E. Gebhard, L. Jasperse, L. Burdett Hart, C.R. Smith, S. Venn-Watson, F. Townsend, R. Wells, B. Balmer, E. Zolman, T. Rowles, and L. Schwacke. 2017. Changes in immune functions in bottlenose dolphins in the northern Gulf of Mexico associated with the Deepwater Horizon oil spill. Endangered Species Research 33:291–303. doi: 10.3354/esr00814

De Guise, S., M. Levin, L. Jasperse, J. Herrman, R.S. Wells, T. Rowles, and L. Schwacke. 2021. Long-term immunological alterations in bottlenose dolphin a decade after the Deepwater Horizon oil spill in the northern Gulf of Mexico: Potential for multigenerational effects. Environmental Toxicology and Chemistry 40(5):1308–1321. doi: 10.1002/etc.4980

Deepwater Horizon Natural Resource Damage Assessment Trustees (DWH NRDA). 2016. Deepwater Horizon oil spill: Final Programmatic Damage Assessment and Restoration Plan and Final Programmatic Environmental Impact Statement. Retrieved from: http://www.gulfspillrestoration.noaa.gov/restoration-planning/gulf-plan.1659.

Fauquier, D.A., J. Litz, S. Sanchez, K. Colegrove, L.H. Schwacke, L. Hart, J. Saliki, C. Smith, T. Goldstein, S. Bowen-Stevens, W. McFee, E. Fougeres, B. Mase-Guthrie, E. Stratton, R. Ewing, S. Venn-Watson, R.H. Carmichael, C. Clemons-Chevis, W. Hatchett, D. Shannon, S. Shippee, S. Smith, L. Staggs, M.C. Tumlin, N.L. Wingers, and T.K. Rowles. 2017. Evaluation of morbillivirus exposure in cetaceans from the northern Gulf of Mexico 2010-2014. Endangered Species Research 33:211–220. doi: 10.3354/esr00772

Fernandez, S. and A.A. Hohn. 1998. Age, growth, and calving season of bottlenose dolphins, *Tursiops truncatus*, off coastal Texas. Fishery Bulletin 96(2):357–365.

Fertl, D. 1994. Occurrence patterns and behavior of bottlenose dolphins (*Tursiops truncatus*) in the Galveston ship channel, Texas. Texas Journal of Science 46(4):299–318.

Hayes, S.A., E. Josephson, K. Maze-Foley, P.E. Rosel, and J. Wallace. 2022. U.S. Atlantic and Gulf of Mexico marine mammal stock assessments 2021. National Oceanic and Atmospheric Administration, National Marine Fisheries Service, Northeast Fisheries Science Center: Woods Hole, MA. NOAA Technical Memorandum NMFS-NE-288. 380 p. doi: 10.25923/6tt7-kc16

Helm, R.C., D.P. Costa, T.D. DeBruyn, T.J. O'Shea, R.S. Wells, and T.M. Williams. 2015. Overview of effects of oil spills on marine mammals. In: Fingas, M. (Ed.), Handbook of oil spill science and technology, First edition. John Wiley & Sons, Inc., Hoboken, NJ. pp. 455–475.

Henderson, E.E. and B. Würsig. 2007. Behavior patterns of bottlenose dolphins in San Luis Pass, Texas. Gulf of Mexico Science 25(2):153–161. doi: 10.18785/goms.2502.06

Hohn, A.A., M.D. Scott, R.S. Wells, J.C. Sweeney, and A.B. Irvine. 1989. Growth layers in teeth from known-age, free-ranging bottlenose dolphins. Marine Mammal Science 5(4):315–342. doi: 10.1111/j.1748-7692.1989.tb00346.x

Hubard, C.W., K. Maze-Foley, K.D. Mullin, and W.W. Schroeder. 2004. Seasonal abundance and site fidelity of bottlenose dolphins (*Tursiops truncatus*) in Mississippi Sound. Aquatic Mammals 30(2):299–310. doi: 10.1578/am.30.2.2004.299

Irvine, A.B., M.D. Scott, R.S. Wells, and J.H. Kaufmann. 1981. Movements and activities of the Atlantic bottlenose dolphin, *Tursiops truncatus*, near Sarasota, Florida. Fishery Bulletin 79(4):671–688.

Irvine, B. and R.S. Wells. 1972. Results of attempts to tag Atlantic bottlenosed dolphins (*Tursiops truncatus*). Cetology 13:1–5.

Irwin, L.-J. and B. Würsig. 2004. A small resident community of bottlenose dolphins, *Tursiops truncatus*, in Texas: Monitoring recommendations. Gulf of Mexico Science 22(1):13–21. doi: 10.18785/goms.2201.02

Kellar, N.M., T.R. Speakman, C.R. Smith, S.M. Lane, B.C. Balmer, M.L. Trego, K.N. Catelani, M.N. Robbins, C.D. Allen, R.S. Wells, E.S. Zolman, T.K. Rowles, and L.H. Schwacke. 2017. Low reproductive success rates of common bottlenose dolphins *Tursiops truncatus* in the northern Gulf of Mexico following the Deepwater Horizon disaster (2010-2015). Endangered Species Research 33:143–158. doi: 10.3354/esr00775

Kucklick, J., L. Schwacke, R. Wells, A. Hohn, A. Guichard, J. Yordy, L. Hansen, E. Zolman, R. Wilson, J. Litz, D. Nowacek, T. Rowles, R. Pugh, B. Balmer, C. Sinclair, and P. Rosel. 2011. Bottlenose dolphins as indicators of persistent organic pollutants in the western North Atlantic Ocean and Northern Gulf of Mexico. Environmental Science & Technology 45(10):4270–4277. doi: 10.1021/es1042244

LaBrecque, E., C. Curtice, J. Harrison, S.M. Van Parijs, and P.N. Halpin. 2015. 3. Biologically Important Areas for cetaceans within U.S. Waters – Gulf of Mexico Region. Aquatic Mammals 41(1):30–38. doi: 10.1578/am.41.1.2015.30

Lane, S.M., C.R. Smith, J. Mitchell, B.C. Balmer, K.P. Barry, T. McDonald, C.S. Mori, P.E. Rosel, T.K. Rowles, T.R. Speakman, F.I. Townsend, M.C. Tumlin, R.S. Wells, E.S. Zolman, and L.H. Schwacke. 2015. Reproductive outcome and survival of common bottlenose dolphins sampled in Barataria

Bay, Louisiana, USA, following the Deepwater Horizon oil spill. Proceedings of the Royal Society B: Biological Sciences 282(1818):20151944. doi: 10.1098/rspb.2015.1944

Leatherwood, S. 1977. Some preliminary impressions on the numbers and social behavior of free-swimming bottlenosed dolphin calves (*Tursiops truncatus*) in the northern Gulf of Mexico. In: Ridgway, S.H. and K. Bernirschke (Eds.), Breeding Dolphins; Present Status, Suggestions for the Future. Final report to the US Marine Mammal Commission, Contract MM6AC009, MMC-76/07, Washington, DC. pp. 143–167.

Lipscomb, T.P., S. Kennedy, D. Moffett, A. Krafft, B.A. Klaunberg, J.H. Lichy, G.T. Regan, G.A.J. Worthy, and J.K. Taubenberger. 1996. Morbilliviral epizootic in bottlenose dolphins of the Gulf of Mexico. Journal of Veterinary Diagnostic Investigation 8(3):283–290. doi: 10.1177/104063879600800302

Litz, J.A., M.A. Baran, S.R. Bowen-Stevens, R.H. Carmichael, K.M. Colegrove, L.P. Garrison, S.E. Fire, E.M. Fougeres, R. Hardy, S. Holmes, W. Jones, B.E. Mase-Guthrie, D.K. Odell, P.E. Rosel, J.T. Saliki, D.K. Shannon, S.F. Shippee, S.M. Smith, E.M. Stratton, M.C. Tumlin, H.R. Whitehead, G.A. Worthy, and T.K. Rowles. 2014. Review of historical unusual mortality events (UMEs) in the Gulf of Mexico (1990–2009): providing context for the multi-year northern Gulf of Mexico cetacean UME declared in 2010. Diseases of Aquatic Organisms 112(2):161–175. doi: 10.3354/dao02807

Lynn, S.K. and B. Würsig. 2002. Summer movement patterns of bottlenose dolphins in a Texas bay. Gulf of Mexico Science 20(1):25–37. doi: 10.18785/goms.2001.03

Mackey, A.D. 2010. Site fidelity and association patterns of bottlenose dolphins (*Tursiops truncatus*) in the Mississippi Sound. University of Southern Mississippi. Thesis. 133 p.

Mann, J., R.C. Connor, L.M. Barre, and M.R. Heithaus. 2000. Female reproductive success in bottlenose dolphins (*Tursiops* sp.): life history, habitat, provisioning, and group-size effects. Behavioral Ecology 11(2):210–219. doi: 10.1093/beheco/11.2.210

Mattson, M.C., K.D. Mullin, G.W. Ingram, and W. Hoggard. 2006. Age structure and growth of the bottlenose dolphin (*Tursiops truncatus*) from strandings in the Mississippi sound region of the north-central Gulf of Mexico from 1986 to 2003. Marine Mammal Science 22(3):654–666. doi: 10.1111/j.1748-7692.2006.00057.x

Maze, K.S. and B. Würsig. 1999. Bottlenosed dolphins of San Luis Pass, Texas: Occurrence patterns, site-fidelity, and habitat use. Aquatic Mammals 25:91–104.

McCormack, M.A., F. Battaglia, W.E. McFee, and J. Dutton. 2020a. Mercury concentrations in blubber and skin from stranded bottlenose dolphins (*Tursiops truncatus*) along the Florida and Louisiana coasts (Gulf of Mexico, USA) in relation to biological variables. Environmental Research 180:108886. doi: 10.1016/j.envres.2019.108886

McCormack, M.A., B.P. Jackson, and J. Dutton. 2020b. Relationship between mercury and selenium concentrations in tissues from stranded odontocetes in the northern Gulf of Mexico. Science of the Total Environment 749:141350. doi: 10.1016/j.scitotenv.2020.141350

McCormack, M.A., W.H. Nowlin, and J. Dutton. 2022. Effect of trophic position on mercury concentrations in bottlenose dolphins (*Tursiops truncatus*) from the northern Gulf of Mexico. Environmental Research 204(Pt B):112124. doi: 10.1016/j.envres.2021.112124

McHugh, M.B. 1989. Population numbers and feeding behavior of the Atlantic bottlenose dolphin (*Tursiops truncatus*) near Aransas Pass, Texas. University of Texas at Austin. Thesis. 96 p.

Mead, J.G. and C.W. Potter. 1990. Natural history of bottlenose dolphins along the central Atlantic coast of the United States. In: Leatherwood, S. and R.R. Reeves (Eds.), The Bottlenose Dolphin. Academic Press, San Diego, CA. pp. 165–195.

Michel, J., E.H. Owens, S. Zengel, A. Graham, Z. Nixon, T. Allard, W. Holton, P.D. Reimer, A. Lamarche, M. White, N. Rutherford, C. Childs, G. Mauseth, G. Challenger, and E. Taylor. 2013. Extent and degree of shoreline oiling: Deepwater Horizon oil spill, Gulf of Mexico, USA. PLoS ONE 8(6):e65087. doi: 10.1371/journal.pone.0065087

Miller, C.E. 2003. Abundance trends and environmental habitat usage patterns of bottlenose dolphins (*Tursiops truncatus*) in lower Barataria and Caminada bays, Louisiana. Louisiana State University and Agricultural & Mechanical College. Dissertation. 125 p.

Miller, L.J., A.D. Mackey, T. Hoffland, M. Solangi, and S.A. Kuczaj. 2010. Potential effects of a major hurricane on Atlantic bottlenose dolphin (*Tursiops truncatus*) reproduction in the Mississippi Sound. Marine Mammal Science 26(3):707–715. doi: 10.1111/j.1748-7692.2010.00371.x

Miller, L.J., A.D. Mackey, M. Solangi, and S.A. Kuczaj. 2013. Population abundance and habitat utilization of bottlenose dolphins in the Mississippi Sound. Aquatic Conservation: Marine and Freshwater Ecosystems 23(1):145–151. doi: 10.1002/aqc.2278

Mullin, K.D., T. McDonald, R.S. Wells, B.C. Balmer, T. Speakman, C. Sinclair, E.S. Zolman, F. Hornsby, S.M. McBride, K.A. Wilkinson, and L.H. Schwacke. 2017. Density, abundance, survival, and ranging patterns of common bottlenose dolphins (*Tursiops truncatus*) in Mississippi Sound following the Deepwater Horizon oil spill. PLoS ONE 12(10):e0186265. doi: 10.1371/journal.pone.0186265

National Oceanic and Atmospheric Administration (NOAA). 2011. Public scoping for preparation of a programmatic environmental impact statement for the Deepwater Horizon BP Oil Spill. 9 p.

Perrin, W.F. and S.B. Reilly. 1984. Reproductive parameters of dolphins and small whales of the family Delphinidae. In: Perrin, W.F., R.L. Brownell, Jr., and D.P. DeMaster (Eds.), Reproduction in Whales, Dolphins, and Porpoises. pp. 97–134.

Rosel, P.E., L.A. Wilcox, C. Sinclair, T.R. Speakman, M.C. Tumlin, J.A. Litz, and E.S. Zolman. 2017. Genetic assignment to stock of stranded common bottlenose dolphins in southeastern Louisiana after the Deepwater Horizon oil spill. Endangered Species Research 33:221–234. doi: 10.3354/esr00780

Rossman, S., P.H. Ostrom, M. Stolen, N.B. Barros, H. Gandhi, C.A. Stricker, and R.S. Wells. 2015. Individual specialization in the foraging habits of female bottlenose dolphins living in a trophically diverse and habitat rich estuary. Oecologia 178(2):415–425. doi: 10.1007/s00442-015-3241-6

Schwacke, L.H., C.R. Smith, F.I. Townsend, R.S. Wells, L.B. Hart, B.C. Balmer, T.K. Collier, S. De Guise, M.M. Fry, L.J. Guillette, Jr., S.V. Lamb, S.M. Lane, W.E. McFee, N.J. Place, M.C. Tumlin, G.M. Ylitalo, E.S. Zolman, and T.K. Rowles. 2014. Health of common bottlenose dolphins (*Tursiops truncatus*) in Barataria Bay, Louisiana, following the deepwater horizon oil spill. Environmental Science & Technology 48(1):93–103. doi: 10.1021/es403610f

Schwacke, L.H., L. Thomas, R.S. Wells, W.E. McFee, A.A. Hohn, K.D. Mullin, E.S. Zolman, B.M. Quigley, T.K. Rowles, and J.H. Schwacke. 2017. Quantifying injury to common bottlenose dolphins from the Deepwater Horizon oil spill using an age-, sex- and class-structured population model. Endangered Species Research 33:265–279. doi: 10.3354/esr00777

Scott, M.D. and S.J. Chivers. 1990. Distribution and herd structure of bottlenose dolphins in the eastern tropical Pacific Ocean. In: Leatherwood, S. and R.R. Reeves (Eds.), The Bottlenose Dolphin. Academic Press, San Diego, CA. pp. 387–402.

Sellas, A.B., R.S. Wells, and P.E. Rosel. 2005. Mitochondrial and nuclear DNA analyses reveal fine scale geographic structure in bottlenose dolphins (*Tursiops truncatus*) in the Gulf of Mexico. Conservation Genetics 6(5):715–728. doi: 10.1007/s10592-005-9031-7

Sergeant, D.E., D.K. Caldwell, and M.C. Caldwell. 1973. Age, growth, and maturity of bottlenosed dolphin (*Tursiops-truncatus*) from northeast Florida. Journal of the Fisheries Research Board of Canada 30(7):1009–1011. doi: 10.1139/f73-165

Shane, S.H. 1990. Behavior and ecology of the bottlenose dolphin at Sanibel Island, Florida. In: Leatherwood, S. and R.R. Reeves (Eds.), The Bottlenose Dolphin. Academic Press, San Diego, CA. pp. 245–265. doi: 10.1016/B978-0-12-440280-5.50016-0

Shane, S.H. 2004. Residence patterns, group characteristics, and association patterns of bottlenose dolphins near Sanibel Island, Florida. Gulf of Mexico Science 22(1):1–12. doi: 10.18785/goms.2201.01

Shane, S.H., R.S. Wells, and B. Würsig. 1986. Ecology, behavior and social organization of the bottlenose dolphin: A review. Marine Mammal Science 2:34–63. doi: 10.1111/j.1748-7692.1986.tb00026.x

Smith, C.R., T.K. Rowles, L.B. Hart, F.I. Townsend, R.S. Wells, E.S. Zolman, B.C. Balmer, B. Quigley, M. Ivančić, W. McKercher, M.C. Tumlin, K.D. Mullin, J.D. Adams, Q. Wu, W. McFee, T.K. Collier, and L.H. Schwacke. 2017. Slow recovery of Barataria Bay dolphin health following the Deepwater Horizon oil spill (2013-2014), with evidence of persistent lung disease and impaired stress response. Endangered Species Research 33:127–142. doi: 10.3354/esr00778

Takeshita, R., B.C. Balmer, F. Messina, E.S. Zolman, L. Thomas, R.S. Wells, C.R. Smith, T.K. Rowles, and L.H. Schwacke. 2021. High site-fidelity in common bottlenose dolphins despite low salinity exposure and associated indicators of compromised health. PLoS ONE 16(9):e0258031. doi: 10.1371/journal.pone.0258031

Taylor, B.L., S.J. Chivers, J. Larese, and W.F. Perrin. 2007. Generation length and percent mature estimates for IUCN assessments of cetaceans. National Oceanic and Atmospheric Administration, National Marine Fisheries Service, Southwest Fisheries Science Center: La Jolla, CA. Administrative Report LJ-07-01. 24 p.

Toms, C. 2019. Filling the gaps: Common bottlenose dolphin (*Tursiops truncatus*) population dynamics, structure, and connectivity within Florida panhandle bays, sounds, and estuaries. University of Central Florida. Dissertation. 373 p.

Torres, L.G. and A.J. Read. 2009. Where to catch a fish? The influence of foraging tactics on the ecology of bottlenose dolphins (*Tursiops truncatus*) in Florida Bay, Florida. Marine Mammal Science 25(4):797–815. doi: 10.1111/j.1748-7692.2009.00297.x

Twiner, M.J., L.J. Flewelling, S.E. Fire, S.R. Bowen-Stevens, J.K. Gaydos, C.K. Johnson, J.H. Landsberg, T.A. Leighfield, B. Mase-Guthrie, L. Schwacke, F.M. Van Dolah, Z. Wang, and T.K. Rowles. 2012. Comparative analysis of three brevetoxin-associated bottlenose dolphin (*Tursiops truncatus*) mortality events in the Florida Panhandle region (USA). PLoS ONE 7(8):e42974. doi: 10.1371/journal.pone.0042974

Tyson, R.B., S.M. Nowacek, and D.P. Nowacek. 2011. Community structure and abundance of bottlenose dolphins *Tursiops truncatus* in coastal waters of the northeast Gulf of Mexico. Marine Ecology Progress Series 438:253–265. doi: 10.3354/meps09292

Urian, K.W., S. Hofmann, R.S. Wells, and A.J. Read. 2009. Fine-scale population structure of bottlenose dolphins (*Tursiops truncatus*) in Tampa Bay, Florida. Marine Mammal Science 25(3):619–638. doi: 10.1111/j.1748-7692.2009.00284.x

Van Bressem, M.F., P.J. Duignan, A. Banyard, M. Barbieri, K.M. Colegrove, S. De Guise, G. Di Guardo, A. Dobson, M. Domingo, D. Fauquier, A. Fernandez, T. Goldstein, B. Grenfell, K.R. Groch, F. Gulland, B.A. Jensen, P.D. Jepson, A. Hall, T. Kuiken, S. Mazzariol, S.E. Morris, O. Nielsen, J.A. Raga, T.K. Rowles, J. Saliki, E. Sierra, N. Stephens, B. Stone, I. Tomo, J. Wang, T. Waltzek, and J.F. Wellehan. 2014. Cetacean morbillivirus: current knowledge and future directions. Viruses 6(12):5145–5181. doi: 10.3390/v6125145

Venn-Watson, S., L. Garrison, J. Litz, E. Fougeres, B. Mase, G. Rappucci, E. Stratton, R. Carmichael, D. Odell, D. Shannon, S. Shippee, S. Smith, L. Staggs, M. Tumlin, H. Whitehead, and T. Rowles. 2015. Demographic clusters identified within the northern Gulf of Mexico common bottlenose dolphin (*Tursiops truncates*) unusual mortality event: January 2010-June 2013. PLoS ONE 10(2):e0117248. doi: 10.1371/journal.pone.0117248

Vollmer, N.L. and P.E. Rosel. 2013. A review of common bottlenose dolphins (*Tursiops truncatus truncatus*) in the Northern Gulf of Mexico: Population biology, potential threats, and management. Southeastern Naturalist 12(m6):1–43. doi: 10.1656/058.012.m601

Waring, G.T., E. Josephson, K. Maze-Foley, and P.E. Rosel. 2016. U.S. Atlantic and Gulf of Mexico marine mammal stock assessments - 2015. National Oceanic and Atmospheric Administration, National Marine Fisheries Service, Northeast Fisheries Science Center: Woods Hole, MA. NOAA Technical Memorandum NMFS-NE-238. 501 p. doi: 10.7289/V57S7KTN

Weller, D.W. 1998. Global and regional variation in the biology and behavior of bottlenose dolphins. Texas A&M University. Dissertation. 142 p.

Wells, R.S. 1986a. Population structure of bottlenose dolphins: Behavioral studies along the central west coast of Florida. Contract Report to National Marine Fisheries Service, Southeast Fisheries Center. Contract No. 45-WCNF-5-003BB. 58 p.

Wells, R.S. 1986b. Structural aspects of dolphin societies. University of California, Santa Cruz. Dissertation. 234 p.

Wells, R.S. 1991. The role of long-term study in understanding the social structure of a bottlenose dolphin community. In: Pryor, K. and K. Norris (Eds.), Dolphin Societies: Discoveries and Puzzles. University of California Press, Berkeley, CA. pp. 199–226.

Wells, R.S. 2000. Reproduction in wild bottlenose dolphins: Overview of patterns observed during a long-term study. In: Duffield, D. and T. Robeck (Eds.), Bottlenose Dolphin Reproduction Workshop. AZA Marine Mammal Taxon Advisory Group, Silver Spring, MD. pp. 57–74.

Wells, R.S. 2003. Dolphin social complexity: Lessons from long-term study and life history. In: de Waal, F.B.M. and P.L. Tyack (Eds.), Animal Social Complexity: Intelligence, Culture, and Individualized Societies Harvard University Press, Cambridge, MA. pp. 32–56.

Wells, R.S. 2014. Social structure and life history of bottlenose dolphins near Sarasota Bay, Florida: Insights from four decades and five generations. Primates and cetaceans. pp. 149–172. doi: 10.1007/978-4-431-54523-1_8

Wells, R.S., M.K. Bassos, K.W. Urian, W.J. Carr, and M.D. Scott. 1996. Low-level monitoring of bottlenose dolphins, *Tursiops truncatus*, in Charlotte Harbor, Florida: 1990-1994. National Oceanic and Atmospheric Administration, National Marine Fisheries Service, Southeast Fisheries Science Center: Miami, FL. NOAA Technical Memorandum NMFS-SEFSC-384. 86 p.

Wells, R.S., M.K. Bassos, K.W. Urian, S.H. Shane, E.C.G. Owen, C.F. Weiss, W.J. Carr, and M.D. Scott. 1997. Low-level monitoring of bottlenose dolphins, *Tursiops truncatus*, in Pine Island Sound, Florida: 1996. National Oceanic and Atmospheric Administration, National Marine Fisheries Service, Southeast Fisheries Science Center: Miami, FL. Southeast Fisheries Center Contribution No. 40-WCNF601958. 86 p.

Wells, R.S., A. Natoli, and G. Braulik. 2019. *Tursiops truncatus* (errata version published in 2019). *The IUCN Red List of Threatened Species* 2019: e.T22563A156932432. https://dx.doi.org/10.2305/IUCN.UK.2019-1.RLTS.T22563A156932432.en

Wells, R.S., L.H. Schwacke, T.K. Rowles, B.C. Balmer, E. Zolman, T. Speakman, F.I. Townsend, M.C. Tumlin, A. Barleycorn, and K.A. Wilkinson. 2017. Ranging patterns of common bottlenose dolphins *Tursiops truncatus* in Barataria Bay, Louisiana, following the Deepwater Horizon oil spill. Endangered Species Research 33:159–180. doi: 10.3354/esr00732

Wells, R.S. and M.D. Scott. 1999. Bottlenose dolphin *Tursiops truncatus* (Montagu, 1821). In: Ridgway, S.H. and R. Harrison (Eds.), Handbook of marine mammals. Volume 6: The second book of dolphins and the porpoises. Academic Press, San Diego, CA. pp. 137–182.

Wells, R.S. and M.D. Scott. 2018. Bottlenose dolphin, *Tursiops truncatus*, common bottlenose dolphin. In: Würsig, B., J.G.M. Thewissen, and K.M. Kovacs (Eds.), Encyclopedia of Marine Mammals

(Third Edition). Academic Press, London, UK. pp. 118–125. doi: 10.1016/B978-0-12-804327-1.00072-8

Wells, R.S., M.D. Scott, and A.B. Irvine. 1987. The social structure of free-ranging bottlenose dolphins. In: Genoways, H.H. (Ed.), Current Mammalogy. Plenum Press, New York, NY. pp. 247–305.

Wells, R.S., K.W. Urian, A.J. Read, M.K. Bassos, W.J. Carr, and M.D. Scott. 1996. Low-level monitoring of bottlenose dolphins, *Tursiops truncatus*, in Tampa Bay, Florida: 1988-1993. National Oceanic and Atmospheric Administration, National Marine Fisheries Service, Southeast Fisheries Science Center: Miami, FL. NOAA Technical Memorandum NMFS-SEFSC-385. 36 p.

Wilson, R.M., R.B. Tyson, J.A. Nelson, B.C. Balmer, J.P. Chanton, and D.P. Nowacek. 2017. Niche differentiation and prey selectivity among common bottlenose dolphins (*Tursiops truncatus*) sighted in St. George Sound, Gulf of Mexico. Frontiers in Marine Science 4:235. doi: 10.3389/fmars.2017.00235

Common bottlenose dolphin − *Tursiops truncatus*

GoMx BSE/Chokoloskee Bay, Ten Thousand Islands, Gullivan Bay Stock

Overall Vulnerability Rank = Very High

Biological Sensitivity = Very High

Climate Exposure = Very High

Data Quality = 81% of scores ≥ 2

	Tursiops truncatus	Expert Scores	Data Quality	Expert Scores Plots (Portion by Category)
Sensitivity attributes	Prey/Diet Specificity	2.5	1.7	
	Habitat Specificity	3.5	1.3	
	Site Fidelity	3.3	1.3	
	Lifetime Reproductive Potential	2.1	2.0	
	Generation Length	2.7	2.0	
	Reproductive Plasticity	1.3	2.0	
	Migration	3.6	2.0	
	Home Range	3.5	2.0	
	Stock Abundance	2.5	1.3	
	Stock Abundance Trend	1.9	0.7	
	Cumulative Stressors	2.9	2.0	
	Sensitivity Score	Very High		
Exposure variables	Sea Surface Temperature (Change in mean)	3.9	2.0	
	Sea Surface Temperature (Change in variability)	1.1	2.0	
	Air Temperature (Change in mean)	3.9	2.0	
	Air Temperature (Change in variability)	1.1	2.0	
	Precipitation (Change in mean)	1.1	2.0	
	Precipitation (Change in variability)	1.1	2.0	
	Sea Surface Salinity (Change in mean)	2.7	2.0	
	Sea Surface Salinity (Change in variability)	1.3	2.0	
	Ocean pH (Change in mean)	3.9	2.0	
	Ocean pH (Change in variability)	1.1	2.0	
	Sea ice coverage (Change in mean)	1.0	2.7	
	Sea ice coverage (Change in variability)	1.0	2.7	
	Dissolved oxygen (Change in mean)	3.9	2.0	
	Dissolved oxygen (Change in variability)	1.1	2.0	
	Circulation	1.2	2.0	
	Sea level rise	3.9	2.0	
	Exposure Score	Very High		
	Overall Vulnerability Rank	Very High		

Low
Moderate
High
Very High

Common bottlenose dolphin (GoMx BSE/Chokoloskee Bay, Ten Thousand Islands, Gullivan Bay Stock)

Tursiops truncatus

CVA Results Summary

Overall Climate Vulnerability Rank: Very High (100% certainty from bootstrap analysis).

Climate Exposure: Very High. Five exposure factors scored greater than 3.5: Sea level rise (3.93), Air Temperature (Standard anomaly) (3.87), Dissolved oxygen (Standard anomaly) (3.87), Ocean pH (Standard anomaly) (3.87), and Sea Surface Temperature (Standard anomaly) (3.87)

Biological Sensitivity: Very High. Three sensitivity attributes scored greater than 3.5: Migration (3.60), Habitat Specificity (3.53), and Home Range (3.53)

Distributional Response: High

Abundance Response: High

Phenology Response: High

Data Quality: 81% of the data quality scores were 2 or greater. 55% of sensitivity attribute data quality scores were 2 or higher.

Background Information

Stock Description

This stock includes common bottlenose dolphins in Chokoloskee Bay, Ten Thousand Islands, and Gullivan Bay in the eastern Gulf of Mexico along the Florida coast (Hayes et al. 2022).

Prey/Diet Specificity

Generally, common bottlenose dolphins consume a large variety of fish and/or squid (Barros and Odell 1990; Mead and Potter 1990; Wells and Scott 2018). Common bottlenose dolphins seem to show a consistent preference for sound-producing fishes (e.g., sciaenids, toadfish; Berens McCabe et al. 2010; Bowen-Stevens et al. 2021). In Sarasota Bay, FL, Rossman et al. (2015) found some individual common bottlenose dolphins to show diet specialization despite the broader study group showing a generalist diet. Specialization can be influenced by locally abundant prey species (Shane et al. 1986; Vollmer and Rosel 2013) or can result from resource partitioning amongst sympatric populations (e.g., Torres and Read 2009; Wilson et al. 2017).

Habitat Specificity

Common bottlenose dolphins are found in a variety of marine and estuarine habitats (Wells and Scott 2018).

Site Fidelity

No specific site fidelity information was found for this stock. However, common bottlenose dolphins in other Gulf of Mexico bay, sound, and estuary (BSE) stocks show site fidelity (LaBrecque et al. 2015; Hayes et al. 2022), such as in Matagorda Bay, Espiritu Santo Bay, and San Luis Pass in Texas (Maze and Würsig 1999; Lynn and Würsig 2002; Irwin and Würsig 2004); Barataria Bay in Louisiana (Miller 2003; Wells et al. 2017; Takeshita et al. 2021); Mississippi Sound in Mississippi (Hubard et al. 2004; Mackey 2010; Mullin et al. 2017); Charlotte Harbor and Pine Island Sound (Bassos-Hull et al. 2013), Sarasota Bay (Wells 1986a; Wells 1991; Wells 2014), St. Andrew Bay (Balmer et al. 2019), St. Joseph Bay (Balmer et al. 2008; B. Balmer et al. 2018), St. Vincent Sound and Apalachicola Bay (Tyson et al. 2011), and Tampa Bay in Florida (Wells et al. 1996b; Urian et al. 2009).

Lifetime Reproductive Potential

Taylor et al. (2007) reported the interbirth interval of common bottlenose dolphins as 3.80 years, though other studies have shown it to be closer to three years (Leatherwood 1977; Wells and Scott 1999; Connor et al. 2000; Wells 2003).

Generally, females reach sexual maturity before males, with females reaching sexual maturity at 5–13 years (Sergeant et al. 1973; Perrin and Reilly 1984; Wells et al. 1987; Mead and Potter 1990; Mann et al. 2000; Wells 2000; Wells and Scott 2018).

Females reproduce through most of their adult lives (Leatherwood 1977; Hohn et al. 1989; Wells and Scott 1999; Wells 2000; Wells 2003), but may produce fewer calves after they reach 30 years of age (Wells 2000). Sarasota Bay common bottlenose dolphins can give birth when they are as young as 6 years of age, and continue until they are 48 years old (Wells and Scott 2018). Reproductive lifespan would therefore be up to 42 years based on long-term observation studies (Wells 2014; Wells and Scott 2018).

Longitudinal studies from Sarasota Bay, Florida have found that female common bottlenose dolphins can live up to 67 years (Wells and Scott 2018). Studies based on growth layer groups in teeth suggest female common bottlenose dolphins can live to 41 years along the Texas coast (Fernandez and Hohn 1998) and to 30 years along the Mississippi coast (Mattson et al. 2006).

Generation Length

Taylor et al. (2007) reported common bottlenose dolphin generation length of 20.6-21.1 years.

Reproductive Plasticity

Common bottlenose dolphin calving is generally diffusely seasonal, with one or more peaks, although births have been reported from all seasons (Vollmer and Rosel 2013; Wells and Scott 2018). Calf sightings peak in the spring and summer months in Florida (Wells et al. 1987), Mississippi/Louisiana (Miller et al. 2010; Miller et al. 2013), and Texas (McHugh 1989; Henderson et al. 2007).

Migration

The BSE stocks in the Gulf of Mexico are thought to primarily comprise stable resident communities. However, transient or seasonal movements of some individuals into and out of a BSE stock area are recorded in some areas (LaBrecque et al. 2015; Hayes et al. 2022). Molecular genetic data support that some BSE stocks are genetically distinct from one another, and furthermore are differentiated from adjacent coastal populations (e.g., Sellas et al. 2005; Rosel et al. 2017). For example, Toms (2019) found fine-scale genetic population structure among five inshore systems in the Florida Panhandle and variable migration rates among populations.

Year-round residency has been reported for common bottlenose dolphins (Hayes et al. 2022), such as in Florida (Irvine and Wells 1972; Irvine et al. 1981; Wells 1986a; Wells 1986b; Wells et al. 1987; Scott et al. 1990; Wells 1991; Wells et al. 1996a; Wells et al. 1996b; Wells et al. 1997; Wells 2003; Balmer et al. 2008; Urian et al. 2009; Tyson et al. 2011; Bassos-Hull et al. 2013; B. Balmer et al. 2018; Balmer et al. 2019; Toms 2019), Texas (Shane 1990; Bräger 1993; Bräger et al. 1994; Fertl 1994; Weller 1998; Maze and Würsig 1999; Lynn and Würsig 2002; Irwin and Würsig 2004; Shane 2004), and Louisiana/Mississippi (Hubard et al. 2004; Mullin et al. 2017; Takeshita et al. 2021).

Home Range

The home ranges of individuals within the Gulf of Mexico BSE stocks are considered to generally include the areas within the stock boundaries and brief excursions beyond the stock boundaries (Irvine et al. 1981; Wells 1986a; Wells 1986b; Wells 1991; Wells et al. 1996a; Wells et al. 1996b; Balmer et al. 2008; Wells 2014; Balmer et al. 2019), although some individuals show preference for small areas within the stock boundaries (Lynn and Würsig 2002; Wells et al. 2017).

Stock Abundance

An abundance estimate is not available for this stock (Hayes et al. 2022).

Stock Abundance Trend

Data are insufficient to complete an abundance trend analysis for this stock (Hayes et al. 2022).

Cumulative Stressors

Common bottlenose dolphins face a wide range of individual and cumulative natural and anthropogenic threats (see reviews by Vollmer and Rosel 2013; Wells and Scott 2018; and Wells et al. 2019). These include such threats as shark predation, harmful algal blooms, recreational and commercial fishing gear, vessel traffic, pollution, habitat alteration, and provisioning.

The nearshore and estuarine habitats along the Gulf of Mexico coast include waters adjacent to areas of high human population, some of which are highly industrialized, and areas of heavy agricultural impact. Studies of bottlenose dolphin tissues from southwest Florida have shown persistent organic pollutant (POP) in blubber and blood samples (Kucklick et al. 2011; Balmer et al. 2015; Damseaux et al. 2017; J. Balmer et al. 2018). Similarly, mercury has been detected in common bottlenose dolphin tissues in this stock (Damseaux et al. 2017) and elsewhere in Florida Gulf Coast waters (McCormack et al. 2020a, 2020b, 2022).

An Unusual Mortality Event (UME) was declared in 1990 for the Gulf of Mexico from Florida to Texas, with more than 300 common bottlenose dolphins stranding due to an undetermined cause, although morbillivirus and cold temperatures may have contributed (Litz et al. 2014). This stock was at the southern end of the range of the 2005–2006 UME in central west Florida caused by brevetoxin (Litz et al. 2014).

Further Reading

Hayes, S.A., E. Josephson, K. Maze-Foley, P.E. Rosel, and J. Wallace. 2022. U.S. Atlantic and Gulf of Mexico marine mammal stock assessments 2021. National Oceanic and Atmospheric Administration, National Marine Fisheries Service, Northeast Fisheries Science Center: Woods Hole, MA. NOAA Technical Memorandum NMFS-NE-288. 380 p. doi: 10.25923/6tt7-kc16

Wells, R.S. and M.D. Scott. 2018. Bottlenose Dolphin, *Tursiops truncatus*, Common Bottlenose Dolphin. In: Würsig, B., J.G.M. Thewissen, and K.M. Kovacs (Eds.), Encyclopedia of Marine Mammals (Third Edition). Academic Press, London, UK. pp. 118–125. doi: 10.1016/B978-0-12-804327-1.00072-8

Wells, R.S., A. Natoli, and G. Braulik. 2019. *Tursiops truncatus* (errata version published in 2019). The IUCN Red List of Threatened Species 2019: e.T22563A156932432. https://dx.doi.org/10.2305/IUCN.UK.2019-1.RLTS.T22563A156932432.en

References

Balmer, B., T. McDonald, F. Hornsby, J. Adams, J. Allen, A. Barleycorn, and L. Schwacke. 2018. Long-term trends in a northern Gulf of Mexico common bottlenose dolphin (*Tursiops truncatus*) population in the wake of the Deepwater Horizon oil spill. Journal of Cetacean Research and Management 18:1–9. doi: 10.47536/jcrm.v18i1.409

Balmer, B., S. Watwood, B. Quigley, T. Speakman, K. Barry, K. Mullin, P. Rosel, C. Sinclair, E. Zolman, and L. Schwacke. 2019. Common bottlenose dolphin (*Tursiops truncatus*) abundance and distribution patterns in St. Andrew Bay, Florida, USA. Aquatic Conservation: Marine and Freshwater Ecosystems 29(3):486–498. doi: 10.1002/aqc.3001

Balmer, B.C., R.S. Wells, S.M. Nowacek, D.P. Nowacek, L.H. Schwacke, W.A. McLellan, F.S. Scharf, T.K. Rowles, L.J. Hansen, T.R. Spradlin, and D.A. Pabst. 2008. Seasonal abundance and distribution patterns of common bottlenose dolphins (*Tursiops truncatus*) near St. Joseph Bay, Florida, USA. Journal of Cetacean Research and Management 10(2):157–167.

Balmer, B.C., G.M. Ylitalo, L.E. McGeorge, K.A. Baugh, D. Boyd, K.D. Mullin, P.E. Rosel, C. Sinclair, R.S. Wells, E.S. Zolman, and L.H. Schwacke. 2015. Persistent organic pollutants (POPs) in blubber of common bottlenose dolphins (*Tursiops truncatus*) along the northern Gulf of Mexico coast, USA. Science of the Total Environment 527-528:306–312. doi: 10.1016/j.scitotenv.2015.05.016

Balmer, J.E., G.M. Ylitalo, T.K. Rowles, K.D. Mullin, R.S. Wells, F.I. Townsend, R.W. Pearce, J.L. Bolton, E.S. Zolman, B.C. Balmer, and L.H. Schwacke. 2018. Persistent organic pollutants (POPs) in blood and blubber of common bottlenose dolphins (*Tursiops truncatus*) at three northern Gulf of Mexico sites following the Deepwater Horizon oil spill. Science of the Total Environment 621:130–137. doi: 10.1016/j.scitotenv.2017.11.209

Barros, N.B. and D.K. Odell. 1990. Food habits of bottlenose dolphins in the southeastern United States In: Leatherwood, S. and R.R. Reeves (Eds.), The Bottlenose Dolphin. Academic Press, San Diego, CA. pp. 309–328. doi: 10.1016/B978-0-12-440280-5.50020-2

Bassos-Hull, K., R.M. Perrtree, C.C. Shepard, S. Schilling, A.A. Barleycorn, J.B. Allen, B.C. Balmer, W.E. Pine, and R.S. Wells. 2013. Long-term site fidelity and seasonal abundance estimates of common

bottlenose dolphins (*Tursiops truncatus*) along the southwest coast of Florida and responses to natural perturbations. Journal of Cetacean Research and Management 13(1):19–30. doi: 10.47536/jcrm.v13i1.551

Berens McCabe, E.J., D.P. Gannon, N.B. Barros, and R.S. Wells. 2010. Prey selection by resident common bottlenose dolphins (*Tursiops truncatus*) in Sarasota Bay, Florida. Marine Biology 157(5):931–942. doi: 10.1007/s00227-009-1371-2

Bowen-Stevens, S.R., D.P. Gannon, R.A. Hazelkorn, G. Lovewell, K.M. Volker, S. Smith, M.C. Tumlin, and J. Litz. 2021. Diet of common bottlenose dolphins, *Tursiops truncatus*, that stranded in and near Barataria Bay, Louisiana, 2010–2012. Southeastern Naturalist 20(1). doi: 10.1656/058.020.0113

Bräger, S. 1993. Diurnal and seasonal behavior patterns of bottlenose dolphins (*Tursiops truncatus*). Marine Mammal Science 9:434–438. doi: 10.1111/j.1748-7692.1993.tb00477.x

Bräger, S., B. Würsig, A. Acevedo, and T. Henningsen. 1994. Association patterns of bottle-nosed dolphins (*Tursiops-truncatus*) in Galveston Bay, Texas. Journal of Mammalogy 75(2):431–437. doi: 10.2307/1382564

Connor, R.C., R.S. Wells, J. Mann, and A.J. Read. 2000. The bottlenose dolphin: Social relationships in a fission-fusion society. In: Mann, J., R. Connor, P. Tyack, and H. Whitehead (Eds.), Cetacean Societies: Field Studies of Dolphins and Whales. University of Chicago Press, Chicago, IL. pp. 91–126.

Damseaux, F., J.J. Kiszka, M.R. Heithaus, G. Scholl, G. Eppe, J.P. Thome, J. Lewis, W. Hao, M.C. Fontaine, and K. Das. 2017. Spatial variation in the accumulation of POPs and mercury in bottlenose dolphins of the Lower Florida Keys and the coastal Everglades (South Florida). Environmental Pollution 220(Pt A):577–587. doi: 10.1016/j.envpol.2016.10.005

Fernandez, S. and A.A. Hohn. 1998. Age, growth, and calving season of bottlenose dolphins, *Tursiops truncatus*, off coastal Texas. Fishery Bulletin 96(2):357–365.

Fertl, D. 1994. Occurrence patterns and behavior of bottlenose dolphins (*Tursiops truncatus*) in the Galveston ship channel, Texas. Texas Journal of Science 46(4):299–318.

Hayes, S.A., E. Josephson, K. Maze-Foley, P.E. Rosel, and J. Wallace. 2022. U.S. Atlantic and Gulf of Mexico marine mammal stock assessments 2021. National Oceanic and Atmospheric Administration, National Marine Fisheries Service, Northeast Fisheries Science Center: Woods Hole, MA. NOAA Technical Memorandum NMFS-NE-288. 380 p. doi: 10.25923/6tt7-kc16

Henderson, E.E. and B. Würsig. 2007. Behavior patterns of bottlenose dolphins in San Luis Pass, Texas. Gulf of Mexico Science 25(2):153–161. doi: 10.18785/goms.2502.06

Hohn, A.A., M.D. Scott, R.S. Wells, J.C. Sweeney, and A.B. Irvine. 1989. Growth layers in teeth from known-age, free-ranging bottlenose dolphins. Marine Mammal Science 5(4):315–342. doi: 10.1111/j.1748-7692.1989.tb00346.x

Hubard, C.W., K. Maze-Foley, K.D. Mullin, and W.W. Schroeder. 2004. Seasonal abundance and site fidelity of bottlenose dolphins (*Tursiops truncatus*) in Mississippi Sound. Aquatic Mammals 30(2):299–310. doi: 10.1578/am.30.2.2004.299

Irvine, A.B., M.D. Scott, R.S. Wells, and J.H. Kaufmann. 1981. Movements and activities of the Atlantic bottlenose dolphin, *Tursiops truncatus*, near Sarasota, Florida. Fishery Bulletin 79(4):671–688.

Irvine, B. and R.S. Wells. 1972. Results of attempts to tag Atlantic bottlenosed dolphins (*Tursiops truncatus*). Cetology 13:1–5.

Irwin, L.-J. and B. Würsig. 2004. A small resident community of bottlenose dolphins, *Tursiops truncatus*, in Texas: Monitoring recommendations. Gulf of Mexico Science 22(1):13–21. doi: 10.18785/goms.2201.02

Kucklick, J., L. Schwacke, R. Wells, A. Hohn, A. Guichard, J. Yordy, L. Hansen, E. Zolman, R. Wilson, J. Litz, D. Nowacek, T. Rowles, R. Pugh, B. Balmer, C. Sinclair, and P. Rosel. 2011. Bottlenose dolphins as indicators of persistent organic pollutants in the western North Atlantic Ocean and Northern

Gulf of Mexico. Environmental Science & Technology 45(10):4270–4277. doi: 10.1021/es1042244

LaBrecque, E., C. Curtice, J. Harrison, S.M. Van Parijs, and P.N. Halpin. 2015. 3. Biologically Important Areas for cetaceans within U.S. Waters – Gulf of Mexico Region. Aquatic Mammals 41(1):30–38. doi: 10.1578/am.41.1.2015.30

Leatherwood, S. 1977. Some preliminary impressions on the numbers and social behavior of free-swimming bottlenosed dolphin calves (*Tursiops truncatus*) in the northern Gulf of Mexico. In: Ridgway, S.H. and K. Bernirschke (Eds.), Breeding Dolphins; Present Status, Suggestions for the Future. Final report to the US Marine Mammal Commission, Contract MM6AC009, MMC-76/07, Washington, DC. pp. 143–167.

Litz, J.A., M.A. Baran, S.R. Bowen-Stevens, R.H. Carmichael, K.M. Colegrove, L.P. Garrison, S.E. Fire, E.M. Fougeres, R. Hardy, S. Holmes, W. Jones, B.E. Mase-Guthrie, D.K. Odell, P.E. Rosel, J.T. Saliki, D.K. Shannon, S.F. Shippee, S.M. Smith, E.M. Stratton, M.C. Tumlin, H.R. Whitehead, G.A. Worthy, and T.K. Rowles. 2014. Review of historical unusual mortality events (UMEs) in the Gulf of Mexico (1990–2009): providing context for the multi-year northern Gulf of Mexico cetacean UME declared in 2010. Diseases of Aquatic Organisms 112(2):161–175. doi: 10.3354/dao02807

Lynn, S.K. and B. Würsig. 2002. Summer movement patterns of bottlenose dolphins in a Texas bay. Gulf of Mexico Science 20(1):25–37. doi: 10.18785/goms.2001.03

Mackey, A.D. 2010. Site fidelity and association patterns of bottlenose dolphins (*Tursiops truncatus*) in the Mississippi Sound. University of Southern Mississippi. Thesis. 133 p.

Mann, J., R.C. Connor, L.M. Barre, and M.R. Heithaus. 2000. Female reproductive success in bottlenose dolphins (*Tursiops* sp.): life history, habitat, provisioning, and group-size effects. Behavioral Ecology 11(2):210–219. doi: 10.1093/beheco/11.2.210

Mattson, M.C., K.D. Mullin, G.W. Ingram, and W. Hoggard. 2006. Age structure and growth of the bottlenose dolphin (*Tursiops truncatus*) from strandings in the Mississippi sound region of the north-central Gulf of Mexico from 1986 to 2003. Marine Mammal Science 22(3):654–666. doi: 10.1111/j.1748-7692.2006.00057.x

Maze, K.S. and B. Würsig. 1999. Bottlenosed dolphins of San Luis Pass, Texas: Occurrence patterns, site-fidelity, and habitat use. Aquatic Mammals 25:91–104.

McCormack, M.A., F. Battaglia, W.E. McFee, and J. Dutton. 2020a. Mercury concentrations in blubber and skin from stranded bottlenose dolphins (*Tursiops truncatus*) along the Florida and Louisiana coasts (Gulf of Mexico, USA) in relation to biological variables. Environmental Research 180:108886. doi: 10.1016/j.envres.2019.108886

McCormack, M.A., B.P. Jackson, and J. Dutton. 2020b. Relationship between mercury and selenium concentrations in tissues from stranded odontocetes in the northern Gulf of Mexico. Science of the Total Environment 749:141350. doi: 10.1016/j.scitotenv.2020.141350

McCormack, M.A., W.H. Nowlin, and J. Dutton. 2022. Effect of trophic position on mercury concentrations in bottlenose dolphins (*Tursiops truncatus*) from the northern Gulf of Mexico. Environmental Research 204(Pt B):112124. doi: 10.1016/j.envres.2021.112124

McHugh, M.B. 1989. Population numbers and feeding behavior of the Atlantic bottlenose dolphin (*Tursiops truncatus*) near Aransas Pass, Texas. University of Texas at Austin. Thesis. 96 p.

Mead, J.G. and C.W. Potter. 1990. Natural history of bottlenose dolphins along the central Atlantic coast of the United States. In: Leatherwood, S. and R.R. Reeves (Eds.), The Bottlenose Dolphin. Academic Press, San Diego, CA. pp. 165–195.

Miller, C.E. 2003. Abundance trends and environmental habitat usage patterns of bottlenose dolphins (*Tursiops truncatus*) in lower Barataria and Caminada bays, Louisiana. Louisiana State University and Agricultural & Mechanical College. Dissertation. 125 p.

Miller, L.J., A.D. Mackey, T. Hoffland, M. Solangi, and S.A. Kuczaj. 2010. Potential effects of a major hurricane on Atlantic bottlenose dolphin (*Tursiops truncatus*) reproduction in the Mississippi Sound. Marine Mammal Science 26(3):707–715. doi: 10.1111/j.1748-7692.2010.00371.x

Miller, L.J., A.D. Mackey, M. Solangi, and S.A. Kuczaj. 2013. Population abundance and habitat utilization of bottlenose dolphins in the Mississippi Sound. Aquatic Conservation: Marine and Freshwater Ecosystems 23(1):145–151. doi: 10.1002/aqc.2278

Mullin, K.D., T. McDonald, R.S. Wells, B.C. Balmer, T. Speakman, C. Sinclair, E.S. Zolman, F. Hornsby, S.M. McBride, K.A. Wilkinson, and L.H. Schwacke. 2017. Density, abundance, survival, and ranging patterns of common bottlenose dolphins (*Tursiops truncatus*) in Mississippi Sound following the Deepwater Horizon oil spill. PLoS ONE 12(10):e0186265. doi: 10.1371/journal.pone.0186265

Perrin, W.F. and S.B. Reilly. 1984. Reproductive parameters of dolphins and small whales of the family Delphinidae. In: Perrin, W.F., R.L. Brownell, Jr., and D.P. DeMaster (Eds.), Reproduction in Whales, Dolphins, and Porpoises. pp. 97–134.

Rosel, P.E., L.A. Wilcox, C. Sinclair, T.R. Speakman, M.C. Tumlin, J.A. Litz, and E.S. Zolman. 2017. Genetic assignment to stock of stranded common bottlenose dolphins in southeastern Louisiana after the Deepwater Horizon oil spill. Endangered Species Research 33:221–234. doi: 10.3354/esr00780

Rossman, S., P.H. Ostrom, M. Stolen, N.B. Barros, H. Gandhi, C.A. Stricker, and R.S. Wells. 2015. Individual specialization in the foraging habits of female bottlenose dolphins living in a trophically diverse and habitat rich estuary. Oecologia 178(2):415–425. doi: 10.1007/s00442-015-3241-6

Scott, M.D. and S.J. Chivers. 1990. Distribution and herd structure of bottlenose dolphins in the eastern tropical Pacific Ocean. In: Leatherwood, S. and R.R. Reeves (Eds.), The Bottlenose Dolphin. Academic Press, San Diego, CA. pp. 387–402.

Sellas, A.B., R.S. Wells, and P.E. Rosel. 2005. Mitochondrial and nuclear DNA analyses reveal fine scale geographic structure in bottlenose dolphins (*Tursiops truncatus*) in the Gulf of Mexico. Conservation Genetics 6(5):715–728. doi: 10.1007/s10592-005-9031-7

Sergeant, D.E., D.K. Caldwell, and M.C. Caldwell. 1973. Age, growth, and maturity of bottlenosed dolphin (*Tursiops-truncatus*) from northeast Florida. Journal of the Fisheries Research Board of Canada 30(7):1009–1011. doi: 10.1139/f73-165

Shane, S.H. 1990. Behavior and ecology of the bottlenose dolphin at Sanibel Island, Florida. In: Leatherwood, S. and R.R. Reeves (Eds.), The Bottlenose Dolphin. Academic Press, San Diego, CA. pp. 245–265. doi: 10.1016/B978-0-12-440280-5.50016-0

Shane, S.H. 2004. Residence patterns, group characteristics, and association patterns of bottlenose dolphins near Sanibel Island, Florida. Gulf of Mexico Science 22(1):1–12. doi: 10.18785/goms.2201.01

Shane, S.H., R.S. Wells, and B. Würsig. 1986. Ecology, behavior and social organization of the bottlenose dolphin: A review. Marine Mammal Science 2:34–63. doi: 10.1111/j.1748-7692.1986.tb00026.x

Takeshita, R., B.C. Balmer, F. Messina, E.S. Zolman, L. Thomas, R.S. Wells, C.R. Smith, T.K. Rowles, and L.H. Schwacke. 2021. High site-fidelity in common bottlenose dolphins despite low salinity exposure and associated indicators of compromised health. PLoS ONE 16(9):e0258031. doi: 10.1371/journal.pone.0258031

Taylor, B.L., S.J. Chivers, J. Larese, and W.F. Perrin. 2007. Generation length and percent mature estimates for IUCN assessments of cetaceans. National Oceanic and Atmospheric Administration, National Marine Fisheries Service, Southwest Fisheries Science Center: La Jolla, CA. Administrative Report LJ-07-01. 24 p.

Toms, C. 2019. Filling the gaps: Common bottlenose dolphin (*Tursiops truncatus*) population dynamics, structure, and connectivity within Florida panhandle bays, sounds, and estuaries. University of Central Florida. Dissertation. 373 p.

Torres, L.G. and A.J. Read. 2009. Where to catch a fish? The influence of foraging tactics on the ecology of bottlenose dolphins (*Tursiops truncatus*) in Florida Bay, Florida. Marine Mammal Science 25(4):797–815. doi: 10.1111/j.1748-7692.2009.00297.x

Tyson, R.B., S.M. Nowacek, and D.P. Nowacek. 2011. Community structure and abundance of bottlenose dolphins *Tursiops truncatus* in coastal waters of the northeast Gulf of Mexico. Marine Ecology Progress Series 438:253–265. doi: 10.3354/meps09292

Urian, K.W., S. Hofmann, R.S. Wells, and A.J. Read. 2009. Fine-scale population structure of bottlenose dolphins (*Tursiops truncatus*) in Tampa Bay, Florida. Marine Mammal Science 25(3):619–638. doi: 10.1111/j.1748-7692.2009.00284.x

Vollmer, N.L. and P.E. Rosel. 2013. A review of common bottlenose dolphins (*Tursiops truncatus truncatus*) in the Northern Gulf of Mexico: Population biology, potential threats, and management. Southeastern Naturalist 12(m6):1–43. doi: 10.1656/058.012.m601

Weller, D.W. 1998. Global and regional variation in the biology and behavior of bottlenose dolphins. Texas A&M University. Dissertation. 142 p.

Wells, R.S. 1986a. Population structure of bottlenose dolphins: Behavioral studies along the central west coast of Florida. Contract Report to National Marine Fisheries Service, Southeast Fisheries Center. Contract No. 45-WCNF-5-003BB. 58 p.

Wells, R.S. 1986b. Structural aspects of dolphin societies. University of California, Santa Cruz. Dissertation. 234 p.

Wells, R.S. 1991. The role of long-term study in understanding the social structure of a bottlenose dolphin community. In: Pryor, K. and K. Norris (Eds.), Dolphin Societies: Discoveries and Puzzles. University of California Press, Berkeley, CA. pp. 199–226.

Wells, R.S. 2000. Reproduction in wild bottlenose dolphins: Overview of patterns observed during a long-term study. In: Duffield, D. and T. Robeck (Eds.), Bottlenose Dolphin Reproduction Workshop. AZA Marine Mammal Taxon Advisory Group, Silver Spring, MD. pp. 57–74.

Wells, R.S. 2003. Dolphin social complexity: Lessons from long-term study and life history. In: de Waal, F.B.M. and P.L. Tyack (Eds.), Animal Social Complexity: Intelligence, Culture, and Individualized Societies Harvard University Press, Cambridge, MA. pp. 32–56.

Wells, R.S. 2014. Social structure and life history of bottlenose dolphins near Sarasota Bay, Florida: Insights from four decades and five generations. Primates and cetaceans. pp. 149–172. doi: 10.1007/978-4-431-54523-1_8

Wells, R.S., M.K. Bassos, K.W. Urian, W.J. Carr, and M.D. Scott. 1996. Low-level monitoring of bottlenose dolphins, *Tursiops truncatus*, in Charlotte Harbor, Florida: 1990-1994. National Oceanic and Atmospheric Administration, National Marine Fisheries Service, Southeast Fisheries Science Center: Miami, FL. NOAA Technical Memorandum NMFS-SEFSC-384. 86 p.

Wells, R.S., M.K. Bassos, K.W. Urian, S.H. Shane, E.C.G. Owen, C.F. Weiss, W.J. Carr, and M.D. Scott. 1997. Low-level monitoring of bottlenose dolphins, *Tursiops truncatus*, in Pine Island Sound, Florida: 1996. National Oceanic and Atmospheric Administration, National Marine Fisheries Service, Southeast Fisheries Science Center: Miami, FL. Southeast Fisheries Center Contribution No. 40-WCNF601958. 86 p.

Wells, R.S., A. Natoli, and G. Braulik. 2019. *Tursiops truncatus* (errata version published in 2019). *The IUCN Red List of Threatened Species* 2019: e.T22563A156932432. https://dx.doi.org/10.2305/IUCN.UK.2019-1.RLTS.T22563A156932432.en

Wells, R.S., L.H. Schwacke, T.K. Rowles, B.C. Balmer, E. Zolman, T. Speakman, F.I. Townsend, M.C. Tumlin, A. Barleycorn, and K.A. Wilkinson. 2017. Ranging patterns of common bottlenose

dolphins *Tursiops truncatus* in Barataria Bay, Louisiana, following the Deepwater Horizon oil spill. Endangered Species Research 33:159–180. doi: 10.3354/esr00732

Wells, R.S. and M.D. Scott. 1999. Bottlenose dolphin *Tursiops truncatus* (Montagu, 1821). In: Ridgway, S.H. and R. Harrison (Eds.), Handbook of marine mammals. Volume 6: The second book of dolphins and the porpoises. Academic Press, San Diego, CA. pp. 137–182.

Wells, R.S. and M.D. Scott. 2018. Bottlenose dolphin, *Tursiops truncatus*, common bottlenose dolphin. In: Würsig, B., J.G.M. Thewissen, and K.M. Kovacs (Eds.), Encyclopedia of Marine Mammals (Third Edition). Academic Press, London, UK. pp. 118–125. doi: 10.1016/B978-0-12-804327-1.00072-8

Wells, R.S., M.D. Scott, and A.B. Irvine. 1987. The social structure of free-ranging bottlenose dolphins. In: Genoways, H.H. (Ed.), Current Mammalogy. Plenum Press, New York, NY. pp. 247–305.

Wells, R.S., K.W. Urian, A.J. Read, M.K. Bassos, W.J. Carr, and M.D. Scott. 1996. Low-level monitoring of bottlenose dolphins, *Tursiops truncatus*, in Tampa Bay, Florida: 1988-1993. National Oceanic and Atmospheric Administration, National Marine Fisheries Service, Southeast Fisheries Science Center: Miami, FL. NOAA Technical Memorandum NMFS-SEFSC-385. 36 p.

Wilson, R.M., R.B. Tyson, J.A. Nelson, B.C. Balmer, J.P. Chanton, and D.P. Nowacek. 2017. Niche differentiation and prey selectivity among common bottlenose dolphins (*Tursiops truncatus*) sighted in St. George Sound, Gulf of Mexico. Frontiers in Marine Science 4:235. doi: 10.3389/fmars.2017.00235

Common bottlenose dolphin − *Tursiops truncatus*

GoMx BSE/Copano Bay, Aransas Bay, San Antonio Bay, Redfish Bay, Espiritu Santo Bay Stock

Overall Vulnerability Rank = Very High

Biological Sensitivity = Very High

Climate Exposure = Very High

Data Quality = 96% of scores ≥ 2

	Tursiops truncatus	Expert Scores	Data Quality	Expert Scores Plots (Portion by Category)
Sensitivity attributes	Prey/Diet Specificity	2.4	2.2	
	Habitat Specificity	2.8	2.0	
	Site Fidelity	3.4	2.5	
	Lifetime Reproductive Potential	1.5	2.2	
	Generation Length	2.9	2.2	
	Reproductive Plasticity	1.2	2.0	
	Migration	3.8	2.2	
	Home Range	3.5	2.5	
	Stock Abundance	2.8	2.0	
	Stock Abundance Trend	1.9	1.5	
	Cumulative Stressors	3.8	2.8	
	Sensitivity Score	Very High		
Exposure variables	Sea Surface Temperature (Change in mean)	4.0	2.8	
	Sea Surface Temperature (Change in variability)	1.0	2.8	
	Air Temperature (Change in mean)	4.0	2.8	
	Air Temperature (Change in variability)	1.0	2.8	
	Precipitation (Change in mean)	1.0	2.8	
	Precipitation (Change in variability)	1.0	2.8	
	Sea Surface Salinity (Change in mean)	2.0	2.8	
	Sea Surface Salinity (Change in variability)	1.0	2.8	
	Ocean pH (Change in mean)	4.0	2.8	
	Ocean pH (Change in variability)	1.0	2.8	
	Sea ice coverage (Change in mean)	1.0	2.8	
	Sea ice coverage (Change in variability)	1.0	2.8	
	Dissolved oxygen (Change in mean)	4.0	2.8	
	Dissolved oxygen (Change in variability)	1.0	2.8	
	Circulation	3.8	2.5	
	Sea level rise	4.0	2.8	
	Exposure Score	Very High		
Overall Vulnerability Rank		Very High		

Low
Moderate
High
Very High

Common bottlenose dolphin (GoMx BSE/Copano Bay, Aransas Bay, San Antonio Bay, Redfish Bay, Espiritu Santo Bay Stock)

Tursiops truncatus

CVA Results Summary

Overall Climate Vulnerability Rank: Very High (100% certainty from bootstrap analysis).

Climate Exposure: Very High. Six exposure factors scored greater than 3.5: Air Temperature (Standard anomaly) (4.00), Dissolved oxygen (Standard anomaly) (4.00), Ocean pH (Standard anomaly) (4.00), Sea level rise (4.00), Sea Surface Temperature (Standard anomaly) (4.00), and Circulation (3.85).

Biological Sensitivity: Very High. Three sensitivity attributes scored greater than 3.5: Migration (3.85), Cumulative Stressors (3.75), and Home Range (3.55).

Distributional Response: High

Abundance Response: High

Phenology Response: High

Data Quality: 96%of the data quality scores were 2 or greater, 91% of sensitivity attribute data quality scores were 2 or higher.

Background Information

Stock Description

This stock includes common bottlenose dolphins in Copano Bay, Aransas Bay, San Antonio Bay, Redfish Bay, Espiritu Santo Bay in the western Gulf of Mexico along the Texas coast (Hayes et al. 2022).

Prey/Diet Specificity

Generally, common bottlenose dolphins consume a large variety of fish and/or squid (Barros and Odell 1990; Mead and Potter 1990; Wells and Scott 2018). Common bottlenose dolphins seem to show a consistent preference for sound-producing fishes (e.g., sciaenids, toadfish; Berens McCabe et al. 2010; Bowen-Stevens et al. 2021). In Sarasota Bay, FL, Rossman et al. (2015) found some individual common bottlenose dolphins to show diet specialization despite the broader study group showing a generalist diet. Specialization can be influenced by locally abundant prey species (Shane et al. 1986; Vollmer and Rosel 2013) or can result from resource partitioning amongst sympatric populations (e.g., Torres and Read 2009; Wilson et al. 2017).

Habitat Specificity

Common bottlenose dolphins are found in a variety of marine and estuarine habitats (Wells and Scott 2018).

Site Fidelity

Common bottlenose dolphins in Matagorda Bay, Espiritu Santo Bay, and San Luis Pass in Texas have shown site fidelity (Maze and Würsig 1999; Lynn and Würsig 2002; Irwin and Würsig 2004). Common bottlenose dolphins in other Gulf of Mexico bay, sound, and estuary (BSE) stocks show site fidelity (LaBrecque et al. 2015; Hayes et al. 2022), such as in Barataria Bay in Louisiana (Miller 2003; Wells et al. 2017; Takeshita et al. 2021); Mississippi Sound in Mississippi (Hubard et al. 2004; Mackey 2010; Mullin et al. 2017); Charlotte Harbor and Pine Island Sound (Bassos-Hull et al. 2013), Sarasota Bay in Florida (Wells 1986; Wells 1991; Wells 2014), St. Andrew Bay (Balmer et al. 2019), St. Joseph Bay (Balmer et al. 2008; Balmer et al. 2018), St. Vincent Sound and Apalachicola Bay (Tyson et al. 2011), and Tampa Bay in Florida (Wells et al. 1996b; Urian et al. 2009).

Lifetime Reproductive Potential

Taylor et al. (2007) reported the interbirth interval of common bottlenose dolphins as 3.80 years, though other studies have shown it to be closer to three years (Leatherwood 1977; Wells and Scott 1999; Connor et al. 2000; Wells 2003).

Generally, females reach sexual maturity before males, with females reaching sexual maturity at 5–13 years (Sergeant et al. 1973; Perrin and Reilly 1984; Wells et al. 1987; Mead and Potter 1990; Mann et al. 2000; Wells 2000; Wells and Scott 2018).

Females reproduce throughout their adult lives (Leatherwood 1977; Hohn et al. 1989; Wells and Scott 1999; Wells 2000; Wells 2003), but may produce fewer calves after they reach 30 years of age (Wells 2000). Sarasota Bay common bottlenose dolphins can give birth when they are as young as 6 years of age, and can continue until they are 48 years old (Wells and Scott 2018). Reproductive lifespan would therefore be up to 42 years based on long-term observation studies (Wells 2014; Wells and Scott 2018).

Longitudinal studies from Sarasota Bay, Florida have found that female common bottlenose dolphins can live up to 67 years (Wells and Scott 2018). Studies based on growth layer groups in teeth suggest female common bottlenose dolphins can live to 41 years along the Texas coast (Fernandez and Hohn 1998) and to 30 years along the Mississippi coast (Mattson et al. 2006).

Generation Length

Taylor et al. (2007) reported common bottlenose dolphin generation length of 20.6-21.1 years.

Reproductive Plasticity

Common bottlenose dolphin calving is generally diffusely seasonal, with one or more peaks, although births have been reported from all seasons (Vollmer and Rosel 2013; Wells and Scott 2018). Calf sightings peak in the spring and summer months in Florida (Wells et al. 1987), Mississippi/Louisiana (Miller et al. 2010; Miller et al. 2013), and Texas (McHugh 1989; Henderson et al. 2007).

Migration

The BSE stocks in the Gulf of Mexico are thought to primarily comprise stable resident communities. However, transient or seasonal movements of some individuals into and out of a BSE stock area are

recorded in some areas(LaBrecque et al. 2015; Hayes et al. 2022). Molecular genetic data support that some BSE stocks are genetically distinct from one another, and furthermore are differentiated from adjacent coastal populations (e.g., Sellas et al. 2005; Rosel et al. 2017). For example, Toms (2019) found fine-scale genetic population structure among five inshore systems in the Florida Panhandle and variable migration rates among populations.

Year-round residency has been reported for common bottlenose dolphins (Hayes et al. 2022), such as in Florida (Irvine and Wells 1972; Irvine et al. 1981; Wells 1986a; Wells 1986b; Wells et al. 1987; Scott et al. 1990; Wells 1991; Wells et al. 1996a; Wells et al. 1996b; Wells et al. 1997; Wells 2003; Balmer et al. 2008; Urian et al. 2009; Tyson et al. 2011; Bassos-Hull et al. 2013; Balmer et al. 2018; Balmer et al. 2019; Toms 2019), Texas (Shane 1990; Bräger 1993; Bräger et al. 1994; Fertl 1994; Weller 1998; Maze and Würsig 1999; Lynn and Würsig 2002; Irwin and Würsig 2004; Shane 2004), and Louisiana/Mississippi (Hubard et al. 2004; Mullin et al. 2017; Takeshita et al. 2021).

Home Range

The home ranges of individuals within the Gulf of Mexico BSE stocks are considered to generally include the areas within the stock boundaries and brief excursions beyond the stock boundaries (Irvine et al. 1981; Wells 1986a; Wells 1986b; Wells 1991; Wells et al. 1996a; Wells et al. 1996b; Balmer et al. 2008; Wells 2014; Balmer et al. 2019), although some individuals show preference for small areas within the stock boundaries (Lynn and Würsig 2002; Wells et al. 2017).

Stock Abundance

Blaylock and Hoggard (1994) reported a best estimate of 55 individuals (CV = 0.82) for this stock (Hayes et al. 2022). More recent estimates are unavailable (Hayes et al. 2022).

Stock Abundance Trend

Data are insufficient to complete an abundance trend analysis for this stock (Hayes et al. 2022).

Cumulative Stressors

Common bottlenose dolphins face a wide range of individual and cumulative natural and anthropogenic threats (see reviews by Vollmer and Rosel 2013; Phillips and Rosel 2014; Wells and Scott 2018; and Wells et al. 2019). These include such threats as shark predation, harmful algal blooms (Fire et al. 2011, 2020), recreational and commercial fishing gear, vessel traffic, pollution, habitat alteration, and provisioning.

Unusual Mortality Events (UMEs) have been declared for common bottlenose dolphins in waters of Texas with morbillivirus the confirmed cause in 1994, a suspected cause in 1992 along with environmental conditions, and an undetermined cause in 2008 (Litz et al. 2014). A UME was declared in 1990 for the Gulf of Mexico from Florida to Texas, with more than 300 common bottlenose dolphins stranding due to an undetermined cause, although morbillivirus and cold temperatures may have contributed (Litz et al. 2014).

Further Reading

Hayes, S.A., E. Josephson, K. Maze-Foley, P.E. Rosel, and J. Wallace. 2022. U.S. Atlantic and Gulf of Mexico marine mammal stock assessments 2021. National Oceanic and Atmospheric Administration, National Marine Fisheries Service, Northeast Fisheries Science Center: Woods Hole, MA. NOAA Technical Memorandum NMFS-NE-288. 380 p. doi: 10.25923/6tt7-kc16

Wells, R.S. and M.D. Scott. 2018. Bottlenose Dolphin, *Tursiops truncatus*, Common Bottlenose Dolphin. In: Würsig, B., J.G.M. Thewissen, and K.M. Kovacs (Eds.), Encyclopedia of Marine Mammals (Third Edition). Academic Press, London, UK. pp. 118–125. doi: 10.1016/B978-0-12-804327-1.00072-8

Wells, R.S., A. Natoli, and G. Braulik. 2019. *Tursiops truncatus* (errata version published in 2019). The IUCN Red List of Threatened Species 2019: e.T22563A156932432. https://dx.doi.org/10.2305/IUCN.UK.2019-1.RLTS.T22563A156932432.en

References

Balmer, B., T. McDonald, F. Hornsby, J. Adams, J. Allen, A. Barleycorn, and L. Schwacke. 2018. Long-term trends in a northern Gulf of Mexico common bottlenose dolphin (*Tursiops truncatus*) population in the wake of the Deepwater Horizon oil spill. Journal of Cetacean Research and Management 18:1–9. doi: 10.47536/jcrm.v18i1.409

Balmer, B., S. Watwood, B. Quigley, T. Speakman, K. Barry, K. Mullin, P. Rosel, C. Sinclair, E. Zolman, and L. Schwacke. 2019. Common bottlenose dolphin (*Tursiops truncatus*) abundance and distribution patterns in St. Andrew Bay, Florida, USA. Aquatic Conservation: Marine and Freshwater Ecosystems 29(3):486–498. doi: 10.1002/aqc.3001

Balmer, B.C., R.S. Wells, S.M. Nowacek, D.P. Nowacek, L.H. Schwacke, W.A. McLellan, F.S. Scharf, T.K. Rowles, L.J. Hansen, T.R. Spradlin, and D.A. Pabst. 2008. Seasonal abundance and distribution patterns of common bottlenose dolphins (*Tursiops truncatus*) near St. Joseph Bay, Florida, USA. Journal of Cetacean Research and Management 10(2):157–167.

Barros, N.B. and D.K. Odell. 1990. Food habits of bottlenose dolphins in the southeastern United States In: Leatherwood, S. and R.R. Reeves (Eds.), The Bottlenose Dolphin. Academic Press, San Diego, CA. pp. 309–328. doi: 10.1016/B978-0-12-440280-5.50020-2

Bassos-Hull, K., R.M. Perrtree, C.C. Shepard, S. Schilling, A.A. Barleycorn, J.B. Allen, B.C. Balmer, W.E. Pine, and R.S. Wells. 2013. Long-term site fidelity and seasonal abundance estimates of common bottlenose dolphins (*Tursiops truncatus*) along the southwest coast of Florida and responses to natural perturbations. Journal of Cetacean Research and Management 13(1):19–30. doi: 10.47536/jcrm.v13i1.551

Berens McCabe, E.J., D.P. Gannon, N.B. Barros, and R.S. Wells. 2010. Prey selection by resident common bottlenose dolphins (*Tursiops truncatus*) in Sarasota Bay, Florida. Marine Biology 157(5):931–942. doi: 10.1007/s00227-009-1371-2

Blaylock, R.A. and W. Hoggard. 1994. Preliminary estimates of bottlenose dolphin abundance in southern U.S. Atlantic and Gulf of Mexico continental shelf waters. National Oceanic and Atmospheric Administration, National Marine Fisheries Service, Southeast Fisheries Science Center: Miami, FL. NOAA Technical Memorandum NMFS-SEFSC-356. 10 p.

Bowen-Stevens, S.R., D.P. Gannon, R.A. Hazelkorn, G. Lovewell, K.M. Volker, S. Smith, M.C. Tumlin, and J. Litz. 2021. Diet of common bottlenose dolphins, *Tursiops truncatus*, that stranded in and near Barataria Bay, Louisiana, 2010–2012. Southeastern Naturalist 20(1). doi: 10.1656/058.020.0113

Bräger, S. 1993. Diurnal and seasonal behavior patterns of bottlenose dolphins (*Tursiops truncatus*). Marine Mammal Science 9:434–438. doi: 10.1111/j.1748-7692.1993.tb00477.x

Bräger, S., B. Würsig, A. Acevedo, and T. Henningsen. 1994. Association patterns of bottle-nosed dolphins (*Tursiops-truncatus*) in Galveston Bay, Texas. Journal of Mammalogy 75(2):431–437. doi: 10.2307/1382564

Connor, R.C., R.S. Wells, J. Mann, and A.J. Read. 2000. The bottlenose dolphin: Social relationships in a fission-fusion society. In: Mann, J., R. Connor, P. Tyack, and H. Whitehead (Eds.), Cetacean Societies: Field Studies of Dolphins and Whales. University of Chicago Press, Chicago, IL. pp. 91–126.

Fernandez, S. and A.A. Hohn. 1998. Age, growth, and calving season of bottlenose dolphins, *Tursiops truncatus*, off coastal Texas. Fishery Bulletin 96(2):357–365.

Fertl, D. 1994. Occurrence patterns and behavior of bottlenose dolphins (*Tursiops truncatus*) in the Galveston ship channel, Texas. Texas Journal of Science 46(4):299–318.

Fire, S.E., T.A. Leighfield, G.A. Miller, S. Piwetz, E.R. Sabater, and H. Whitehead. 2020. Association between red tide exposure and detection of corresponding neurotoxins in bottlenose dolphins from Texas waters during 2007-2017. Marine Environmental Research 162:105191. doi: 10.1016/j.marenvres.2020.105191

Fire, S.E., Z. Wang, M. Byrd, H.R. Whitehead, J. Paternoster, and S.L. Morton. 2011. Co-occurrence of multiple classes of harmful algal toxins in bottlenose dolphins (*Tursiops truncatus*) stranding during an unusual mortality event in Texas, USA. Harmful Algae 10(3):330–336. doi: 10.1016/j.hal.2010.12.001

Hayes, S.A., E. Josephson, K. Maze-Foley, P.E. Rosel, and J. Wallace. 2022. U.S. Atlantic and Gulf of Mexico marine mammal stock assessments 2021. National Oceanic and Atmospheric Administration, National Marine Fisheries Service, Northeast Fisheries Science Center: Woods Hole, MA. NOAA Technical Memorandum NMFS-NE-288. 380 p. doi: 10.25923/6tt7-kc16

Henderson, E.E. and B. Würsig. 2007. Behavior patterns of bottlenose dolphins in San Luis Pass, Texas. Gulf of Mexico Science 25(2):153–161. doi: 10.18785/goms.2502.06

Hohn, A.A., M.D. Scott, R.S. Wells, J.C. Sweeney, and A.B. Irvine. 1989. Growth layers in teeth from known-age, free-ranging bottlenose dolphins. Marine Mammal Science 5(4):315–342. doi: 10.1111/j.1748-7692.1989.tb00346.x

Hubard, C.W., K. Maze-Foley, K.D. Mullin, and W.W. Schroeder. 2004. Seasonal abundance and site fidelity of bottlenose dolphins (*Tursiops truncatus*) in Mississippi Sound. Aquatic Mammals 30(2):299–310. doi: 10.1578/am.30.2.2004.299

Irvine, A.B., M.D. Scott, R.S. Wells, and J.H. Kaufmann. 1981. Movements and activities of the Atlantic bottlenose dolphin, *Tursiops truncatus*, near Sarasota, Florida. Fishery Bulletin 79(4):671–688.

Irvine, B. and R.S. Wells. 1972. Results of attempts to tag Atlantic bottlenosed dolphins (*Tursiops truncatus*). Cetology 13:1–5.

Irwin, L.-J. and B. Würsig. 2004. A small resident community of bottlenose dolphins, *Tursiops truncatus*, in Texas: Monitoring recommendations. Gulf of Mexico Science 22(1):13–21. doi: 10.18785/goms.2201.02

LaBrecque, E., C. Curtice, J. Harrison, S.M. Van Parijs, and P.N. Halpin. 2015. 3. Biologically Important Areas for cetaceans within U.S. Waters – Gulf of Mexico Region. Aquatic Mammals 41(1):30–38. doi: 10.1578/am.41.1.2015.30

Leatherwood, S. 1977. Some preliminary impressions on the numbers and social behavior of free-swimming bottlenosed dolphin calves (*Tursiops truncatus*) in the northern Gulf of Mexico. In: Ridgway, S.H. and K. Bernirschke (Eds.), Breeding Dolphins; Present Status, Suggestions for the Future. Final report to the US Marine Mammal Commission, Contract MM6AC009, MMC-76/07, Washington, DC. pp. 143–167.

Litz, J.A., M.A. Baran, S.R. Bowen-Stevens, R.H. Carmichael, K.M. Colegrove, L.P. Garrison, S.E. Fire, E.M. Fougeres, R. Hardy, S. Holmes, W. Jones, B.E. Mase-Guthrie, D.K. Odell, P.E. Rosel, J.T. Saliki, D.K.

Shannon, S.F. Shippee, S.M. Smith, E.M. Stratton, M.C. Tumlin, H.R. Whitehead, G.A. Worthy, and T.K. Rowles. 2014. Review of historical unusual mortality events (UMEs) in the Gulf of Mexico (1990–2009): providing context for the multi-year northern Gulf of Mexico cetacean UME declared in 2010. Diseases of Aquatic Organisms 112(2):161–175. doi: 10.3354/dao02807

Lynn, S.K. and B. Würsig. 2002. Summer movement patterns of bottlenose dolphins in a Texas bay. Gulf of Mexico Science 20(1):25–37. doi: 10.18785/goms.2001.03

Mackey, A.D. 2010. Site fidelity and association patterns of bottlenose dolphins (*Tursiops truncatus*) in the Mississippi Sound. University of Southern Mississippi. Thesis. 133 p.

Mann, J., R.C. Connor, L.M. Barre, and M.R. Heithaus. 2000. Female reproductive success in bottlenose dolphins (*Tursiops* sp.): life history, habitat, provisioning, and group-size effects. Behavioral Ecology 11(2):210–219. doi: 10.1093/beheco/11.2.210

Mattson, M.C., K.D. Mullin, G.W. Ingram, and W. Hoggard. 2006. Age structure and growth of the bottlenose dolphin (*Tursiops truncatus*) from strandings in the Mississippi sound region of the north-central Gulf of Mexico from 1986 to 2003. Marine Mammal Science 22(3):654–666. doi: 10.1111/j.1748-7692.2006.00057.x

Maze, K.S. and B. Würsig. 1999. Bottlenosed dolphins of San Luis Pass, Texas: Occurrence patterns, site-fidelity, and habitat use. Aquatic Mammals 25:91–104.

McHugh, M.B. 1989. Population numbers and feeding behavior of the Atlantic bottlenose dolphin (*Tursiops truncatus*) near Aransas Pass, Texas. University of Texas at Austin. Thesis. 96 p.

Mead, J.G. and C.W. Potter. 1990. Natural history of bottlenose dolphins along the central Atlantic coast of the United States. In: Leatherwood, S. and R.R. Reeves (Eds.), The Bottlenose Dolphin. Academic Press, San Diego, CA. pp. 165–195.

Miller, C.E. 2003. Abundance trends and environmental habitat usage patterns of bottlenose dolphins (*Tursiops truncatus*) in lower Barataria and Caminada bays, Louisiana. Louisiana State University and Agricultural & Mechanical College. Dissertation. 125 p.

Miller, L.J., A.D. Mackey, T. Hoffland, M. Solangi, and S.A. Kuczaj. 2010. Potential effects of a major hurricane on Atlantic bottlenose dolphin (*Tursiops truncatus*) reproduction in the Mississippi Sound. Marine Mammal Science 26(3):707–715. doi: 10.1111/j.1748-7692.2010.00371.x

Miller, L.J., A.D. Mackey, M. Solangi, and S.A. Kuczaj. 2013. Population abundance and habitat utilization of bottlenose dolphins in the Mississippi Sound. Aquatic Conservation: Marine and Freshwater Ecosystems 23(1):145–151. doi: 10.1002/aqc.2278

Mullin, K.D., T. McDonald, R.S. Wells, B.C. Balmer, T. Speakman, C. Sinclair, E.S. Zolman, F. Hornsby, S.M. McBride, K.A. Wilkinson, and L.H. Schwacke. 2017. Density, abundance, survival, and ranging patterns of common bottlenose dolphins (*Tursiops truncatus*) in Mississippi Sound following the Deepwater Horizon oil spill. PLoS ONE 12(10):e0186265. doi: 10.1371/journal.pone.0186265

Perrin, W.F. and S.B. Reilly. 1984. Reproductive parameters of dolphins and small whales of the family Delphinidae. In: Perrin, W.F., R.L. Brownell, Jr., and D.P. DeMaster (Eds.), Reproduction in Whales, Dolphins, and Porpoises. pp. 97–134.

Phillips, N.M. and P.E. Rosel. 2014. A method for prioritizing research on common bottlenose dolphin stocks through evaluating threats and data availability: Development and application to bay, sound and estuary stocks in Texas. National Oceanic and Atmospheric Administration, National Marine Fisheries Service, Southeast Fisheries Science Center: Lafayette, LA. NOAA Technical Memorandum NMFS-SEFSC-665. 146 p. doi: 10.7289/V5F769H8

Rosel, P.E., L.A. Wilcox, C. Sinclair, T.R. Speakman, M.C. Tumlin, J.A. Litz, and E.S. Zolman. 2017. Genetic assignment to stock of stranded common bottlenose dolphins in southeastern Louisiana after the Deepwater Horizon oil spill. Endangered Species Research 33:221–234. doi: 10.3354/esr00780

Rossman, S., P.H. Ostrom, M. Stolen, N.B. Barros, H. Gandhi, C.A. Stricker, and R.S. Wells. 2015. Individual specialization in the foraging habits of female bottlenose dolphins living in a trophically diverse and habitat rich estuary. Oecologia 178(2):415–425. doi: 10.1007/s00442-015-3241-6

Scott, M.D. and S.J. Chivers. 1990. Distribution and herd structure of bottlenose dolphins in the eastern tropical Pacific Ocean. In: Leatherwood, S. and R.R. Reeves (Eds.), The Bottlenose Dolphin. Academic Press, San Diego, CA. pp. 387–402.

Sellas, A.B., R.S. Wells, and P.E. Rosel. 2005. Mitochondrial and nuclear DNA analyses reveal fine scale geographic structure in bottlenose dolphins (*Tursiops truncatus*) in the Gulf of Mexico. Conservation Genetics 6(5):715–728. doi: 10.1007/s10592-005-9031-7

Sergeant, D.E., D.K. Caldwell, and M.C. Caldwell. 1973. Age, growth, and maturity of bottlenosed dolphin (*Tursiops-truncatus*) from northeast Florida. Journal of the Fisheries Research Board of Canada 30(7):1009–1011. doi: 10.1139/f73-165

Shane, S.H. 1990. Behavior and ecology of the bottlenose dolphin at Sanibel Island, Florida. In: Leatherwood, S. and R.R. Reeves (Eds.), The Bottlenose Dolphin. Academic Press, San Diego, CA. pp. 245–265. doi: 10.1016/B978-0-12-440280-5.50016-0

Shane, S.H. 2004. Residence patterns, group characteristics, and association patterns of bottlenose dolphins near Sanibel Island, Florida. Gulf of Mexico Science 22(1):1–12. doi: 10.18785/goms.2201.01

Shane, S.H., R.S. Wells, and B. Würsig. 1986. Ecology, behavior and social organization of the bottlenose dolphin: A review. Marine Mammal Science 2:34–63. doi: 10.1111/j.1748-7692.1986.tb00026.x

Takeshita, R., B.C. Balmer, F. Messina, E.S. Zolman, L. Thomas, R.S. Wells, C.R. Smith, T.K. Rowles, and L.H. Schwacke. 2021. High site-fidelity in common bottlenose dolphins despite low salinity exposure and associated indicators of compromised health. PLoS ONE 16(9):e0258031. doi: 10.1371/journal.pone.0258031

Taylor, B.L., S.J. Chivers, J. Larese, and W.F. Perrin. 2007. Generation length and percent mature estimates for IUCN assessments of cetaceans. National Oceanic and Atmospheric Administration, National Marine Fisheries Service, Southwest Fisheries Science Center: La Jolla, CA. Administrative Report LJ-07-01. 24 p.

Toms, C. 2019. Filling the gaps: Common bottlenose dolphin (*Tursiops truncatus*) population dynamics, structure, and connectivity within Florida panhandle bays, sounds, and estuaries. University of Central Florida. Dissertation. 373 p.

Torres, L.G. and A.J. Read. 2009. Where to catch a fish? The influence of foraging tactics on the ecology of bottlenose dolphins (*Tursiops truncatus*) in Florida Bay, Florida. Marine Mammal Science 25(4):797–815. doi: 10.1111/j.1748-7692.2009.00297.x

Tyson, R.B., S.M. Nowacek, and D.P. Nowacek. 2011. Community structure and abundance of bottlenose dolphins *Tursiops truncatus* in coastal waters of the northeast Gulf of Mexico. Marine Ecology Progress Series 438:253–265. doi: 10.3354/meps09292

Urian, K.W., S. Hofmann, R.S. Wells, and A.J. Read. 2009. Fine-scale population structure of bottlenose dolphins (*Tursiops truncatus*) in Tampa Bay, Florida. Marine Mammal Science 25(3):619–638. doi: 10.1111/j.1748-7692.2009.00284.x

Vollmer, N.L. and P.E. Rosel. 2013. A review of common bottlenose dolphins (*Tursiops truncatus truncatus*) in the Northern Gulf of Mexico: Population biology, potential threats, and management. Southeastern Naturalist 12(m6):1–43. doi: 10.1656/058.012.m601

Weller, D.W. 1998. Global and regional variation in the biology and behavior of bottlenose dolphins. Texas A&M University. Dissertation. 142 p.

Wells, R.S. 1986a. Population structure of bottlenose dolphins: Behavioral studies along the central west coast of Florida. Contract Report to National Marine Fisheries Service, Southeast Fisheries Center. Contract No. 45-WCNF-5-003BB. 58 p.

Wells, R.S. 1986b. Structural aspects of dolphin societies. University of California, Santa Cruz. Dissertation. 234 p.

Wells, R.S. 1991. The role of long-term study in understanding the social structure of a bottlenose dolphin community. In: Pryor, K. and K. Norris (Eds.), Dolphin Societies: Discoveries and Puzzles. University of California Press, Berkeley, CA. pp. 199–226.

Wells, R.S. 2000. Reproduction in wild bottlenose dolphins: Overview of patterns observed during a long-term study. In: Duffield, D. and T. Robeck (Eds.), Bottlenose Dolphin Reproduction Workshop. AZA Marine Mammal Taxon Advisory Group, Silver Spring, MD. pp. 57–74.

Wells, R.S. 2003. Dolphin social complexity: Lessons from long-term study and life history. In: de Waal, F.B.M. and P.L. Tyack (Eds.), Animal Social Complexity: Intelligence, Culture, and Individualized Societies Harvard University Press, Cambridge, MA. pp. 32–56.

Wells, R.S. 2014. Social structure and life history of bottlenose dolphins near Sarasota Bay, Florida: Insights from four decades and five generations. Primates and cetaceans. pp. 149–172. doi: 10.1007/978-4-431-54523-1_8

Wells, R.S., M.K. Bassos, K.W. Urian, W.J. Carr, and M.D. Scott. 1996. Low-level monitoring of bottlenose dolphins, *Tursiops truncatus*, in Charlotte Harbor, Florida: 1990-1994. National Oceanic and Atmospheric Administration, National Marine Fisheries Service, Southeast Fisheries Science Center: Miami, FL. NOAA Technical Memorandum NMFS-SEFSC-384. 86 p.

Wells, R.S., M.K. Bassos, K.W. Urian, S.H. Shane, E.C.G. Owen, C.F. Weiss, W.J. Carr, and M.D. Scott. 1997. Low-level monitoring of bottlenose dolphins, *Tursiops truncatus*, in Pine Island Sound, Florida: 1996. National Oceanic and Atmospheric Administration, National Marine Fisheries Service, Southeast Fisheries Science Center: Miami, FL. Southeast Fisheries Center Contribution No. 40-WCNF601958. 86 p.

Wells, R.S., A. Natoli, and G. Braulik. 2019. *Tursiops truncatus* (errata version published in 2019). *The IUCN Red List of Threatened Species* 2019: e.T22563A156932432. https://dx.doi.org/10.2305/IUCN.UK.2019-1.RLTS.T22563A156932432.en

Wells, R.S., L.H. Schwacke, T.K. Rowles, B.C. Balmer, E. Zolman, T. Speakman, F.I. Townsend, M.C. Tumlin, A. Barleycorn, and K.A. Wilkinson. 2017. Ranging patterns of common bottlenose dolphins *Tursiops truncatus* in Barataria Bay, Louisiana, following the Deepwater Horizon oil spill. Endangered Species Research 33:159–180. doi: 10.3354/esr00732

Wells, R.S. and M.D. Scott. 1999. Bottlenose dolphin *Tursiops truncatus* (Montagu, 1821). In: Ridgway, S.H. and R. Harrison (Eds.), Handbook of marine mammals. Volume 6: The second book of dolphins and the porpoises. Academic Press, San Diego, CA. pp. 137–182.

Wells, R.S. and M.D. Scott. 2018. Bottlenose dolphin, *Tursiops truncatus*, common bottlenose dolphin. In: Würsig, B., J.G.M. Thewissen, and K.M. Kovacs (Eds.), Encyclopedia of Marine Mammals (Third Edition). Academic Press, London, UK. pp. 118–125. doi: 10.1016/B978-0-12-804327-1.00072-8

Wells, R.S., M.D. Scott, and A.B. Irvine. 1987. The social structure of free-ranging bottlenose dolphins. In: Genoways, H.H. (Ed.), Current Mammalogy. Plenum Press, New York, NY. pp. 247–305.

Wells, R.S., K.W. Urian, A.J. Read, M.K. Bassos, W.J. Carr, and M.D. Scott. 1996. Low-level monitoring of bottlenose dolphins, *Tursiops truncatus*, in Tampa Bay, Florida: 1988-1993. National Oceanic and Atmospheric Administration, National Marine Fisheries Service, Southeast Fisheries Science Center: Miami, FL. NOAA Technical Memorandum NMFS-SEFSC-385. 36 p.

Wilson, R.M., R.B. Tyson, J.A. Nelson, B.C. Balmer, J.P. Chanton, and D.P. Nowacek. 2017. Niche differentiation and prey selectivity among common bottlenose dolphins (*Tursiops truncatus*)

sighted in St. George Sound, Gulf of Mexico. Frontiers in Marine Science 4:235. doi: 10.3389/fmars.2017.00235

Common bottlenose dolphin − *Tursiops truncatus*

GoMx BSE/Estero Bay Stock

Overall Vulnerability Rank = Very High

Biological Sensitivity = Very High

Climate Exposure = Very High

Data Quality = 89% of scores ≥ 2

	Tursiops truncatus	Expert Scores	Data Quality	Expert Scores Plots (Portion by Category)
Sensitivity attributes	Prey/Diet Specificity	2.2	2.0	
	Habitat Specificity	3.5	2.0	
	Site Fidelity	3.9	2.3	
	Lifetime Reproductive Potential	2.2	2.0	
	Generation Length	2.7	2.0	
	Reproductive Plasticity	1.1	2.0	
	Migration	3.9	2.0	
	Home Range	3.8	2.3	
	Stock Abundance	2.9	1.7	
	Stock Abundance Trend	2.1	1.3	
	Cumulative Stressors	3.5	1.7	
	Sensitivity Score	Very High		
Exposure variables	Sea Surface Temperature (Change in mean)	3.9	2.3	
	Sea Surface Temperature (Change in variability)	1.1	2.3	
	Air Temperature (Change in mean)	2.9	2.3	
	Air Temperature (Change in variability)	1.1	2.3	
	Precipitation (Change in mean)	1.1	2.3	
	Precipitation (Change in variability)	1.1	2.3	
	Sea Surface Salinity (Change in mean)	2.1	2.3	
	Sea Surface Salinity (Change in variability)	1.1	2.3	
	Ocean pH (Change in mean)	3.9	2.3	
	Ocean pH (Change in variability)	1.1	2.3	
	Sea ice coverage (Change in mean)	1.0	2.5	
	Sea ice coverage (Change in variability)	1.0	2.5	
	Dissolved oxygen (Change in mean)	3.2	2.3	
	Dissolved oxygen (Change in variability)	1.1	2.3	
	Circulation	3.5	2.7	
	Sea level rise	3.0	2.3	
	Exposure Score	Very High		
Overall Vulnerability Rank		Very High		

Low
Moderate
High
Very High

Common bottlenose dolphin (GoMx BSE/Estero Bay Stock)

Tursiops truncatus

CVA Results Summary

Overall Climate Vulnerability Rank: Very High (100% certainty from bootstrap analysis).

Climate Exposure: Very High. Three exposure factors scored greater than 3.5: Ocean pH (Standard anomaly) (3.93), Sea Surface Temperature (Standard anomaly) (3.87), and Circulation (3.53).

Biological Sensitivity: Very High. Three sensitivity attributes scored greater than 3.5: Migration (3.87), Site Fidelity (3.87), and Home Range (3.80).

Distributional Response: High

Abundance Response: High

Phenology Response: High

Data Quality: 89% of the data quality scores were 2 or greater, 73% of sensitivity attribute data quality scores were 2 or higher.

Background Information

Stock Description

This stock includes common bottlenose dolphins in Estero Bay in the eastern Gulf of Mexico along the Florida coast (Hayes et al. 2022).

Prey/Diet Specificity

Generally, common bottlenose dolphins consume a large variety of fish and/or squid (Barros and Odell 1990; Mead and Potter 1990; Wells and Scott 2018). Common bottlenose dolphins seem to show a consistent preference for sound-producing fishes (e.g. sciaenids, toadfish; Berens McCabe et al. 2010; Bowen-Stevens et al. 2021). In Sarasota Bay, FL, Rossman et al. (2015) found some individual common bottlenose dolphins to show diet specialization despite the broader study group showing a generalist diet. Specialization can be influenced by locally abundant prey species (Shane et al. 1986; Vollmer and Rosel 2013) or can result from resource partitioning amongst sympatric populations (e.g., Torres and Read 2009; Wilson et al. 2017).

Habitat Specificity

Common bottlenose dolphins are found in a variety of marine and estuarine habitats (Wells and Scott 2018).

Site Fidelity

No specific site fidelity information was found for this stock. However, common bottlenose dolphins in other Gulf of Mexico bay, sound, and estuary (BSE) stocks show site fidelity (LaBrecque et al. 2015; Hayes et al. 2022), such as in Matagorda Bay, Espiritu Santo Bay, and San Luis Pass in Texas (Maze and Würsig 1999; Lynn and Würsig 2002; Irwin and Würsig 2004); Barataria Bay in Louisiana (Miller 2003; Wells et al. 2017; Takeshita et al. 2021); Mississippi Sound in Mississippi (Hubard et al. 2004; Mackey 2010; Mullin et al. 2017); Charlotte Harbor and Pine Island Sound (Bassos-Hull et al. 2013), Sarasota Bay in Florida (Wells 1986a; Wells 1991; Wells 2014), St. Andrew Bay (Balmer et al. 2019), St. Joseph Bay (Balmer et al. 2008; B. Balmer et al. 2018), St. Vincent Sound and Apalachicola Bay (Tyson et al. 2011), and Tampa Bay in Florida (Wells et al. 1996b; Urian et al. 2009).

Lifetime Reproductive Potential

Taylor et al. (2007) reported the interbirth interval of common bottlenose dolphins as 3.80 years, though other studies have shown it to be closer to three years (Leatherwood 1977; Wells and Scott 1999; Connor et al. 2000; Wells 2003).

Generally, females reach sexual maturity before males, with females reaching sexual maturity at 5–13 years (Sergeant et al. 1973; Perrin and Reilly 1984; Wells et al. 1987; Mead and Potter 1990; Mann et al. 2000; Wells 2000; Wells and Scott 2018).

Females reproduce through most of their adult lives (Leatherwood 1977; Hohn et al. 1989; Wells and Scott 1999; Wells 2000; Wells 2003), but may produce fewer calves after they reach 30 years of age (Wells 2000). Sarasota Bay common bottlenose dolphins can give birth when they are as young as 6 years of age, and can continue until they are 48 years old (Wells and Scott 2018). Reproductive lifespan would therefore be up to 42 years based on long-term observation studies (Wells 2014; Wells and Scott 2018).

Longitudinal studies from Sarasota Bay, Florida have found that female common bottlenose dolphins can live up to 67 years (Wells and Scott 2018). Studies based on growth layer groups in teeth suggest female common bottlenose dolphins can live to 41 years along the Texas coast (Fernandez and Hohn 1998) and to 30 years along the Mississippi coast (Mattson et al. 2006).

Generation Length

Taylor et al. (2007) reported common bottlenose dolphin generation length of 20.6-21.1 years.

Reproductive Plasticity

Common bottlenose dolphin calving is generally diffusely seasonal, with one or more peaks, although births have been reported from all seasons (Vollmer and Rosel 2013; Wells and Scott 2018). Calf sightings peak in the spring and summer months in Florida (Wells et al. 1987), Mississippi/Louisiana (Miller et al. 2010; Miller et al. 2013), and Texas (McHugh 1989; Henderson et al. 2007).

Migration

The BSE stocks in the Gulf of Mexico are thought to primarily comprise stable resident communities. However, transient or seasonal movements of some individuals into and out of a BSE stock area are recorded in some areas (LaBrecque et al. 2015; Hayes et al. 2022). Molecular genetic data support that some BSE stocks are genetically distinct from one another, and furthermore are differentiated from adjacent coastal populations (e.g., Sellas et al. 2005; Rosel et al. 2017). For example, Toms (2019) found fine-scale genetic population structure among five inshore systems in the Florida Panhandle and variable migration rates among populations.

Year-round residency has been reported for common bottlenose dolphins (Hayes et al. 2022), such as in Florida (Irvine and Wells 1972; Irvine et al. 1981; Wells 1986a; Wells 1986b; Wells et al. 1987; Scott et al. 1990; Wells 1991; Wells et al. 1996a; Wells et al. 1996b; Wells et al. 1997; Wells 2003; Balmer et al. 2008; Urian et al. 2009; Tyson et al. 2011; Bassos-Hull et al. 2013; B. Balmer et al. 2018; Balmer et al. 2019; Toms 2019), Texas (Shane 1990; Bräger 1993; Bräger et al. 1994; Fertl 1994; Weller 1998; Maze and Würsig 1999; Lynn and Würsig 2002; Irwin and Würsig 2004; Shane 2004), and Louisiana/Mississippi (Hubard et al. 2004; Mullin et al. 2017; Takeshita et al. 2021).

Home Range

The home ranges of individuals within the Gulf of Mexico BSE stocks are considered to generally include the areas within the stock boundaries and brief excursions beyond the stock boundaries (Irvine et al. 1981; Wells 1986a; Wells 1986b; Wells 1991; Wells et al. 1996a; Wells et al. 1996b; Balmer et al. 2008; Wells 2014; Balmer et al. 2019), although some individuals show preference for small areas within the stock boundaries (Lynn and Würsig 2002; Wells et al. 2017).

Stock Abundance

An abundance estimate for this stock is unavailable (Hayes et al. 2022).

Stock Abundance Trend

Data are insufficient to complete an abundance trend analysis for this stock (Hayes et al. 2022).

Cumulative Stressors

Common bottlenose dolphins face a wide range of individual and cumulative natural and anthropogenic threats (see reviews by Vollmer and Rosel 2013; Wells and Scott 2018; and Wells et al. 2019). These include such threats as shark predation, harmful algal blooms, recreational and commercial fishing gear (Adimey et al. 2014; McHugh et al. 2021), vessel traffic, pollution, habitat alteration, and provisioning.

The nearshore and estuarine habitats along the Gulf of Mexico coast include waters adjacent to areas of high human population, some of which are highly industrialized, and areas of heavy agricultural impact. Studies of bottlenose dolphin tissues from southwest Florida have shown persistent organic pollutant (POP) in blubber and blood samples (Kucklick et al. 2011; Balmer et al. 2015; J. Balmer et al. 2018). Similarly, mercury has been detected in common bottlenose dolphin tissues in Florida Gulf Coast waters (McCormack et al. 2020a, 2020b, 2022).

An Unusual Mortality Event (UME) was declared in 1990 for the Gulf of Mexico from Florida to Texas, with more than 300 common bottlenose dolphins stranding due to an undetermined cause, although morbillivirus and cold temperatures may have contributed (Litz et al. 2014). This stock was within the range of the 2005–2006 UME in central west Florida caused by brevetoxin (Litz et al. 2014).

Further Reading

Hayes, S.A., E. Josephson, K. Maze-Foley, P.E. Rosel, and J. Wallace. 2022. U.S. Atlantic and Gulf of Mexico marine mammal stock assessments 2021. National Oceanic and Atmospheric Administration, National Marine Fisheries Service, Northeast Fisheries Science Center: Woods Hole, MA. NOAA Technical Memorandum NMFS-NE-288. 380 p. doi: 10.25923/6tt7-kc16

Wells, R.S. and M.D. Scott. 2018. Bottlenose Dolphin, *Tursiops truncatus*, Common Bottlenose Dolphin. In: Würsig, B., J.G.M. Thewissen, and K.M. Kovacs (Eds.), Encyclopedia of Marine Mammals (Third Edition). Academic Press, London, UK. pp. 118–125. doi: 10.1016/B978-0-12-804327-1.00072-8

Wells, R.S., A. Natoli, G. Braulik. 2019. *Tursiops truncatus* (errata version published in 2019). The IUCN Red List of Threatened Species 2019: e.T22563A156932432. https://dx.doi.org/10.2305/IUCN.UK.2019-1.RLTS.T22563A156932432.en

References

Adimey, N.M., C.A. Hudak, J.R. Powell, K. Bassos-Hull, A. Foley, N.A. Farmer, L. White, and K. Minch. 2014. Fishery gear interactions from stranded bottlenose dolphins, Florida manatees and sea turtles in Florida, U.S.A. Marine Pollution Bulletin 81(1):103–115. doi: 10.1016/j.marpolbul.2014.02.008

Balmer, B., T. McDonald, F. Hornsby, J. Adams, J. Allen, A. Barleycorn, and L. Schwacke. 2018. Long-term trends in a northern Gulf of Mexico common bottlenose dolphin (*Tursiops truncatus*) population in the wake of the Deepwater Horizon oil spill. Journal of Cetacean Research and Management 18:1–9. doi: 10.47536/jcrm.v18i1.409

Balmer, B., S. Watwood, B. Quigley, T. Speakman, K. Barry, K. Mullin, P. Rosel, C. Sinclair, E. Zolman, and L. Schwacke. 2019. Common bottlenose dolphin (*Tursiops truncatus*) abundance and distribution patterns in St. Andrew Bay, Florida, USA. Aquatic Conservation: Marine and Freshwater Ecosystems 29(3):486–498. doi: 10.1002/aqc.3001

Balmer, B.C., R.S. Wells, S.M. Nowacek, D.P. Nowacek, L.H. Schwacke, W.A. McLellan, F.S. Scharf, T.K. Rowles, L.J. Hansen, T.R. Spradlin, and D.A. Pabst. 2008. Seasonal abundance and distribution patterns of common bottlenose dolphins (*Tursiops truncatus*) near St. Joseph Bay, Florida, USA. Journal of Cetacean Research and Management 10(2):157–167.

Balmer, B.C., G.M. Ylitalo, L.E. McGeorge, K.A. Baugh, D. Boyd, K.D. Mullin, P.E. Rosel, C. Sinclair, R.S. Wells, E.S. Zolman, and L.H. Schwacke. 2015. Persistent organic pollutants (POPs) in blubber of common bottlenose dolphins (*Tursiops truncatus*) along the northern Gulf of Mexico coast, USA. Science of the Total Environment 527-528:306–312. doi: 10.1016/j.scitotenv.2015.05.016

Balmer, J.E., G.M. Ylitalo, T.K. Rowles, K.D. Mullin, R.S. Wells, F.I. Townsend, R.W. Pearce, J.L. Bolton, E.S. Zolman, B.C. Balmer, and L.H. Schwacke. 2018. Persistent organic pollutants (POPs) in blood and blubber of common bottlenose dolphins (*Tursiops truncatus*) at three northern Gulf of Mexico sites following the Deepwater Horizon oil spill. Science of the Total Environment 621:130–137. doi: 10.1016/j.scitotenv.2017.11.209

Barros, N.B. and D.K. Odell. 1990. Food habits of bottlenose dolphins in the southeastern United States In: Leatherwood, S. and R.R. Reeves (Eds.), The Bottlenose Dolphin. Academic Press, San Diego, CA. pp. 309–328. doi: 10.1016/B978-0-12-440280-5.50020-2

Bassos-Hull, K., R.M. Perrtree, C.C. Shepard, S. Schilling, A.A. Barleycorn, J.B. Allen, B.C. Balmer, W.E. Pine, and R.S. Wells. 2013. Long-term site fidelity and seasonal abundance estimates of common bottlenose dolphins (*Tursiops truncatus*) along the southwest coast of Florida and responses to natural perturbations. Journal of Cetacean Research and Management 13(1):19–30. doi: 10.47536/jcrm.v13i1.551

Berens McCabe, E.J., D.P. Gannon, N.B. Barros, and R.S. Wells. 2010. Prey selection by resident common bottlenose dolphins (*Tursiops truncatus*) in Sarasota Bay, Florida. Marine Biology 157(5):931–942. doi: 10.1007/s00227-009-1371-2

Bowen-Stevens, S.R., D.P. Gannon, R.A. Hazelkorn, G. Lovewell, K.M. Volker, S. Smith, M.C. Tumlin, and J. Litz. 2021. Diet of common bottlenose dolphins, *Tursiops truncatus*, that stranded in and near Barataria Bay, Louisiana, 2010–2012. Southeastern Naturalist 20(1). doi: 10.1656/058.020.0113

Bräger, S. 1993. Diurnal and seasonal behavior patterns of bottlenose dolphins (*Tursiops truncatus*). Marine Mammal Science 9:434–438. doi: 10.1111/j.1748-7692.1993.tb00477.x

Bräger, S., B. Würsig, A. Acevedo, and T. Henningsen. 1994. Association patterns of bottle-nosed dolphins (*Tursiops-truncatus*) in Galveston Bay, Texas. Journal of Mammalogy 75(2):431–437. doi: 10.2307/1382564

Connor, R.C., R.S. Wells, J. Mann, and A.J. Read. 2000. The bottlenose dolphin: Social relationships in a fission-fusion society. In: Mann, J., R. Connor, P. Tyack, and H. Whitehead (Eds.), Cetacean Societies: Field Studies of Dolphins and Whales. University of Chicago Press, Chicago, IL. pp. 91–126.

Fernandez, S. and A.A. Hohn. 1998. Age, growth, and calving season of bottlenose dolphins, *Tursiops truncatus*, off coastal Texas. Fishery Bulletin 96(2):357–365.

Fertl, D. 1994. Occurrence patterns and behavior of bottlenose dolphins (*Tursiops truncatus*) in the Galveston ship channel, Texas. Texas Journal of Science 46(4):299–318.

Hayes, S.A., E. Josephson, K. Maze-Foley, P.E. Rosel, and J. Wallace. 2022. U.S. Atlantic and Gulf of Mexico marine mammal stock assessments 2021. National Oceanic and Atmospheric Administration, National Marine Fisheries Service, Northeast Fisheries Science Center: Woods Hole, MA. NOAA Technical Memorandum NMFS-NE-288. 380 p. doi: 10.25923/6tt7-kc16

Henderson, E.E. and B. Würsig. 2007. Behavior patterns of bottlenose dolphins in San Luis Pass, Texas. Gulf of Mexico Science 25(2):153–161. doi: 10.18785/goms.2502.06

Hohn, A.A., M.D. Scott, R.S. Wells, J.C. Sweeney, and A.B. Irvine. 1989. Growth layers in teeth from known-age, free-ranging bottlenose dolphins. Marine Mammal Science 5(4):315–342. doi: 10.1111/j.1748-7692.1989.tb00346.x

Hubard, C.W., K. Maze-Foley, K.D. Mullin, and W.W. Schroeder. 2004. Seasonal abundance and site fidelity of bottlenose dolphins (*Tursiops truncatus*) in Mississippi Sound. Aquatic Mammals 30(2):299–310. doi: 10.1578/am.30.2.2004.299

Irvine, A.B., M.D. Scott, R.S. Wells, and J.H. Kaufmann. 1981. Movements and activities of the Atlantic bottlenose dolphin, *Tursiops truncatus*, near Sarasota, Florida. Fishery Bulletin 79(4):671–688.

Irvine, B. and R.S. Wells. 1972. Results of attempts to tag Atlantic bottlenosed dolphins (*Tursiops truncatus*). Cetology 13:1–5.

Irwin, L.-J. and B. Würsig. 2004. A small resident community of bottlenose dolphins, *Tursiops truncatus*, in Texas: Monitoring recommendations. Gulf of Mexico Science 22(1):13–21. doi: 10.18785/goms.2201.02

Kucklick, J., L. Schwacke, R. Wells, A. Hohn, A. Guichard, J. Yordy, L. Hansen, E. Zolman, R. Wilson, J. Litz, D. Nowacek, T. Rowles, R. Pugh, B. Balmer, C. Sinclair, and P. Rosel. 2011. Bottlenose dolphins as

indicators of persistent organic pollutants in the western North Atlantic Ocean and Northern Gulf of Mexico. Environmental Science & Technology 45(10):4270–4277. doi: 10.1021/es1042244

LaBrecque, E., C. Curtice, J. Harrison, S.M. Van Parijs, and P.N. Halpin. 2015. 3. Biologically Important Areas for cetaceans within U.S. Waters – Gulf of Mexico Region. Aquatic Mammals 41(1):30–38. doi: 10.1578/am.41.1.2015.30

Leatherwood, S. 1977. Some preliminary impressions on the numbers and social behavior of free-swimming bottlenosed dolphin calves (*Tursiops truncatus*) in the northern Gulf of Mexico. In: Ridgway, S.H. and K. Bernirschke (Eds.), Breeding Dolphins; Present Status, Suggestions for the Future. Final report to the US Marine Mammal Commission, Contract MM6AC009, MMC-76/07, Washington, DC. pp. 143–167.

Litz, J.A., M.A. Baran, S.R. Bowen-Stevens, R.H. Carmichael, K.M. Colegrove, L.P. Garrison, S.E. Fire, E.M. Fougeres, R. Hardy, S. Holmes, W. Jones, B.E. Mase-Guthrie, D.K. Odell, P.E. Rosel, J.T. Saliki, D.K. Shannon, S.F. Shippee, S.M. Smith, E.M. Stratton, M.C. Tumlin, H.R. Whitehead, G.A. Worthy, and T.K. Rowles. 2014. Review of historical unusual mortality events (UMEs) in the Gulf of Mexico (1990–2009): providing context for the multi-year northern Gulf of Mexico cetacean UME declared in 2010. Diseases of Aquatic Organisms 112(2):161–175. doi: 10.3354/dao02807

Lynn, S.K. and B. Würsig. 2002. Summer movement patterns of bottlenose dolphins in a Texas bay. Gulf of Mexico Science 20(1):25–37. doi: 10.18785/goms.2001.03

Mackey, A.D. 2010. Site fidelity and association patterns of bottlenose dolphins (*Tursiops truncatus*) in the Mississippi Sound. University of Southern Mississippi. Thesis. 133 p.

Mann, J., R.C. Connor, L.M. Barre, and M.R. Heithaus. 2000. Female reproductive success in bottlenose dolphins (*Tursiops* sp.): life history, habitat, provisioning, and group-size effects. Behavioral Ecology 11(2):210–219. doi: 10.1093/beheco/11.2.210

Mattson, M.C., K.D. Mullin, G.W. Ingram, and W. Hoggard. 2006. Age structure and growth of the bottlenose dolphin (*Tursiops truncatus*) from strandings in the Mississippi sound region of the north-central Gulf of Mexico from 1986 to 2003. Marine Mammal Science 22(3):654–666. doi: 10.1111/j.1748-7692.2006.00057.x

Maze, K.S. and B. Würsig. 1999. Bottlenosed dolphins of San Luis Pass, Texas: Occurrence patterns, site-fidelity, and habitat use. Aquatic Mammals 25:91–104.

McCormack, M.A., F. Battaglia, W.E. McFee, and J. Dutton. 2020a. Mercury concentrations in blubber and skin from stranded bottlenose dolphins (*Tursiops truncatus*) along the Florida and Louisiana coasts (Gulf of Mexico, USA) in relation to biological variables. Environmental Research 180:108886. doi: 10.1016/j.envres.2019.108886

McCormack, M.A., B.P. Jackson, and J. Dutton. 2020b. Relationship between mercury and selenium concentrations in tissues from stranded odontocetes in the northern Gulf of Mexico. Science of the Total Environment 749:141350. doi: 10.1016/j.scitotenv.2020.141350

McCormack, M.A., W.H. Nowlin, and J. Dutton. 2022. Effect of trophic position on mercury concentrations in bottlenose dolphins (*Tursiops truncatus*) from the northern Gulf of Mexico. Environmental Research 204(Pt B):112124. doi: 10.1016/j.envres.2021.112124

McHugh, K.A., A.A. Barleycorn, J.B. Allen, K. Bassos-Hull, G. Lovewell, D. Boyd, A. Panike, C. Cush, D. Fauquier, B. Mase, R.C. Lacy, M.R. Greenfield, D.I. Rubenstein, A. Weaver, A. Stone, L. Oliver, K. Morse, and R.S. Wells. 2021. Staying alive: Long-term success of bottlenose dolphin interventions in southwest Florida. Frontiers in Marine Science 7:624729. doi: 10.3389/fmars.2020.624729

McHugh, M.B. 1989. Population numbers and feeding behavior of the Atlantic bottlenose dolphin (*Tursiops truncatus*) near Aransas Pass, Texas. University of Texas at Austin. Thesis. 96 p.

Mead, J.G. and C.W. Potter. 1990. Natural history of bottlenose dolphins along the central Atlantic coast of the United States. In: Leatherwood, S. and R.R. Reeves (Eds.), The Bottlenose Dolphin. Academic Press, San Diego, CA. pp. 165–195.

Miller, C.E. 2003. Abundance trends and environmental habitat usage patterns of bottlenose dolphins (*Tursiops truncatus*) in lower Barataria and Caminada bays, Louisiana. Louisiana State University and Agricultural & Mechanical College. Dissertation. 125 p.

Miller, L.J., A.D. Mackey, T. Hoffland, M. Solangi, and S.A. Kuczaj. 2010. Potential effects of a major hurricane on Atlantic bottlenose dolphin (*Tursiops truncatus*) reproduction in the Mississippi Sound. Marine Mammal Science 26(3):707–715. doi: 10.1111/j.1748-7692.2010.00371.x

Miller, L.J., A.D. Mackey, M. Solangi, and S.A. Kuczaj. 2013. Population abundance and habitat utilization of bottlenose dolphins in the Mississippi Sound. Aquatic Conservation: Marine and Freshwater Ecosystems 23(1):145–151. doi: 10.1002/aqc.2278

Mullin, K.D., T. McDonald, R.S. Wells, B.C. Balmer, T. Speakman, C. Sinclair, E.S. Zolman, F. Hornsby, S.M. McBride, K.A. Wilkinson, and L.H. Schwacke. 2017. Density, abundance, survival, and ranging patterns of common bottlenose dolphins (*Tursiops truncatus*) in Mississippi Sound following the Deepwater Horizon oil spill. PLoS ONE 12(10):e0186265. doi: 10.1371/journal.pone.0186265

Perrin, W.F. and S.B. Reilly. 1984. Reproductive parameters of dolphins and small whales of the family Delphinidae. In: Perrin, W.F., R.L. Brownell, Jr., and D.P. DeMaster (Eds.), Reproduction in Whales, Dolphins, and Porpoises. pp. 97–134.

Rosel, P.E., L.A. Wilcox, C. Sinclair, T.R. Speakman, M.C. Tumlin, J.A. Litz, and E.S. Zolman. 2017. Genetic assignment to stock of stranded common bottlenose dolphins in southeastern Louisiana after the Deepwater Horizon oil spill. Endangered Species Research 33:221–234. doi: 10.3354/esr00780

Rossman, S., P.H. Ostrom, M. Stolen, N.B. Barros, H. Gandhi, C.A. Stricker, and R.S. Wells. 2015. Individual specialization in the foraging habits of female bottlenose dolphins living in a trophically diverse and habitat rich estuary. Oecologia 178(2):415–425. doi: 10.1007/s00442-015-3241-6

Scott, M.D. and S.J. Chivers. 1990. Distribution and herd structure of bottlenose dolphins in the eastern tropical Pacific Ocean. In: Leatherwood, S. and R.R. Reeves (Eds.), The Bottlenose Dolphin. Academic Press, San Diego, CA. pp. 387–402.

Sellas, A.B., R.S. Wells, and P.E. Rosel. 2005. Mitochondrial and nuclear DNA analyses reveal fine scale geographic structure in bottlenose dolphins (*Tursiops truncatus*) in the Gulf of Mexico. Conservation Genetics 6(5):715–728. doi: 10.1007/s10592-005-9031-7

Sergeant, D.E., D.K. Caldwell, and M.C. Caldwell. 1973. Age, growth, and maturity of bottlenosed dolphin (*Tursiops-truncatus*) from northeast Florida. Journal of the Fisheries Research Board of Canada 30(7):1009–1011. doi: 10.1139/f73-165

Shane, S.H. 1990. Behavior and ecology of the bottlenose dolphin at Sanibel Island, Florida. In: Leatherwood, S. and R.R. Reeves (Eds.), The Bottlenose Dolphin. Academic Press, San Diego, CA. pp. 245–265. doi: 10.1016/B978-0-12-440280-5.50016-0

Shane, S.H. 2004. Residence patterns, group characteristics, and association patterns of bottlenose dolphins near Sanibel Island, Florida. Gulf of Mexico Science 22(1):1–12. doi: 10.18785/goms.2201.01

Shane, S.H., R.S. Wells, and B. Würsig. 1986. Ecology, behavior and social organization of the bottlenose dolphin: A review. Marine Mammal Science 2:34–63. doi: 10.1111/j.1748-7692.1986.tb00026.x

Takeshita, R., B.C. Balmer, F. Messina, E.S. Zolman, L. Thomas, R.S. Wells, C.R. Smith, T.K. Rowles, and L.H. Schwacke. 2021. High site-fidelity in common bottlenose dolphins despite low salinity exposure and associated indicators of compromised health. PLoS ONE 16(9):e0258031. doi: 10.1371/journal.pone.0258031

Taylor, B.L., S.J. Chivers, J. Larese, and W.F. Perrin. 2007. Generation length and percent mature estimates for IUCN assessments of cetaceans. National Oceanic and Atmospheric Administration, National Marine Fisheries Service, Southwest Fisheries Science Center: La Jolla, CA. Administrative Report LJ-07-01. 24 p.

Toms, C. 2019. Filling the gaps: Common bottlenose dolphin (*Tursiops truncatus*) population dynamics, structure, and connectivity within Florida panhandle bays, sounds, and estuaries. University of Central Florida. Dissertation. 373 p.

Torres, L.G. and A.J. Read. 2009. Where to catch a fish? The influence of foraging tactics on the ecology of bottlenose dolphins (*Tursiops truncatus*) in Florida Bay, Florida. Marine Mammal Science 25(4):797–815. doi: 10.1111/j.1748-7692.2009.00297.x

Tyson, R.B., S.M. Nowacek, and D.P. Nowacek. 2011. Community structure and abundance of bottlenose dolphins *Tursiops truncatus* in coastal waters of the northeast Gulf of Mexico. Marine Ecology Progress Series 438:253–265. doi: 10.3354/meps09292

Urian, K.W., S. Hofmann, R.S. Wells, and A.J. Read. 2009. Fine-scale population structure of bottlenose dolphins (*Tursiops truncatus*) in Tampa Bay, Florida. Marine Mammal Science 25(3):619–638. doi: 10.1111/j.1748-7692.2009.00284.x

Vollmer, N.L. and P.E. Rosel. 2013. A review of common bottlenose dolphins (*Tursiops truncatus truncatus*) in the Northern Gulf of Mexico: Population biology, potential threats, and management. Southeastern Naturalist 12(m6):1–43. doi: 10.1656/058.012.m601

Weller, D.W. 1998. Global and regional variation in the biology and behavior of bottlenose dolphins. Texas A&M University. Dissertation. 142 p.

Wells, R.S. 1986a. Population structure of bottlenose dolphins: Behavioral studies along the central west coast of Florida. Contract Report to National Marine Fisheries Service, Southeast Fisheries Center. Contract No. 45-WCNF-5-003BB. 58 p.

Wells, R.S. 1986b. Structural aspects of dolphin societies. University of California, Santa Cruz. Dissertation. 234 p.

Wells, R.S. 1991. The role of long-term study in understanding the social structure of a bottlenose dolphin community. In: Pryor, K. and K. Norris (Eds.), Dolphin Societies: Discoveries and Puzzles. University of California Press, Berkeley, CA. pp. 199–226.

Wells, R.S. 2000. Reproduction in wild bottlenose dolphins: Overview of patterns observed during a long-term study. In: Duffield, D. and T. Robeck (Eds.), Bottlenose Dolphin Reproduction Workshop. AZA Marine Mammal Taxon Advisory Group, Silver Spring, MD. pp. 57–74.

Wells, R.S. 2003. Dolphin social complexity: Lessons from long-term study and life history. In: de Waal, F.B.M. and P.L. Tyack (Eds.), Animal Social Complexity: Intelligence, Culture, and Individualized Societies Harvard University Press, Cambridge, MA. pp. 32–56.

Wells, R.S. 2014. Social structure and life history of bottlenose dolphins near Sarasota Bay, Florida: Insights from four decades and five generations. Primates and cetaceans. pp. 149–172. doi: 10.1007/978-4-431-54523-1_8

Wells, R.S., M.K. Bassos, K.W. Urian, W.J. Carr, and M.D. Scott. 1996. Low-level monitoring of bottlenose dolphins, *Tursiops truncatus*, in Charlotte Harbor, Florida: 1990-1994. National Oceanic and Atmospheric Administration, National Marine Fisheries Service, Southeast Fisheries Science Center: Miami, FL. NOAA Technical Memorandum NMFS-SEFSC-384. 86 p.

Wells, R.S., M.K. Bassos, K.W. Urian, S.H. Shane, E.C.G. Owen, C.F. Weiss, W.J. Carr, and M.D. Scott. 1997. Low-level monitoring of bottlenose dolphins, *Tursiops truncatus*, in Pine Island Sound, Florida: 1996. National Oceanic and Atmospheric Administration, National Marine Fisheries Service, Southeast Fisheries Science Center: Miami, FL. Southeast Fisheries Center Contribution No. 40-WCNF601958. 86 p.

Wells, R.S., A. Natoli, and G. Braulik. 2019. *Tursiops truncatus* (errata version published in 2019). *The IUCN Red List of Threatened Species* 2019: e.T22563A156932432. https://dx.doi.org/10.2305/IUCN.UK.2019-1.RLTS.T22563A156932432.en

Wells, R.S., L.H. Schwacke, T.K. Rowles, B.C. Balmer, E. Zolman, T. Speakman, F.I. Townsend, M.C. Tumlin, A. Barleycorn, and K.A. Wilkinson. 2017. Ranging patterns of common bottlenose dolphins *Tursiops truncatus* in Barataria Bay, Louisiana, following the Deepwater Horizon oil spill. Endangered Species Research 33:159–180. doi: 10.3354/esr00732

Wells, R.S. and M.D. Scott. 1999. Bottlenose dolphin *Tursiops truncatus* (Montagu, 1821). In: Ridgway, S.H. and R. Harrison (Eds.), Handbook of marine mammals. Volume 6: The second book of dolphins and the porpoises. Academic Press, San Diego, CA. pp. 137–182.

Wells, R.S. and M.D. Scott. 2018. Bottlenose dolphin, *Tursiops truncatus*, common bottlenose dolphin. In: Würsig, B., J.G.M. Thewissen, and K.M. Kovacs (Eds.), Encyclopedia of Marine Mammals (Third Edition). Academic Press, London, UK. pp. 118–125. doi: 10.1016/B978-0-12-804327-1.00072-8

Wells, R.S., M.D. Scott, and A.B. Irvine. 1987. The social structure of free-ranging bottlenose dolphins. In: Genoways, H.H. (Ed.), Current Mammalogy. Plenum Press, New York, NY. pp. 247–305.

Wells, R.S., K.W. Urian, A.J. Read, M.K. Bassos, W.J. Carr, and M.D. Scott. 1996. Low-level monitoring of bottlenose dolphins, *Tursiops truncatus*, in Tampa Bay, Florida: 1988-1993. National Oceanic and Atmospheric Administration, National Marine Fisheries Service, Southeast Fisheries Science Center: Miami, FL. NOAA Technical Memorandum NMFS-SEFSC-385. 36 p.

Wilson, R.M., R.B. Tyson, J.A. Nelson, B.C. Balmer, J.P. Chanton, and D.P. Nowacek. 2017. Niche differentiation and prey selectivity among common bottlenose dolphins (*Tursiops truncatus*) sighted in St. George Sound, Gulf of Mexico. Frontiers in Marine Science 4:235. doi: 10.3389/fmars.2017.00235

Common bottlenose dolphin − *Tursiops truncatus* GoMx

BSE - Southwest Marathon Key to Marquesas Keys

Overall Vulnerability Rank = Very High

Biological Sensitivity = High

Climate Exposure = Very High

Data Quality = 85% of scores ≥ 2

	Tursiops truncatus	Expert Scores	Data Quality	Expert Scores Plots (Portion by Category)
Sensitivity attributes	Prey/Diet Specificity	2.2	2.2	
	Habitat Specificity	3.2	1.8	
	Site Fidelity	3.5	2.2	
	Lifetime Reproductive Potential	1.6	2.0	
	Generation Length	2.8	2.0	
	Reproductive Plasticity	1.2	2.0	
	Migration	3.8	2.0	
	Home Range	3.3	2.0	
	Stock Abundance	3.4	1.5	
	Stock Abundance Trend	2.4	0.8	
	Cumulative Stressors	2.8	1.8	
	Sensitivity Score	High		
Exposure variables	Sea Surface Temperature (Change in mean)	4.0	2.5	
	Sea Surface Temperature (Change in variability)	1.0	2.5	
	Air Temperature (Change in mean)	3.2	2.5	
	Air Temperature (Change in variability)	1.0	2.5	
	Precipitation (Change in mean)	1.0	2.5	
	Precipitation (Change in variability)	1.0	2.5	
	Sea Surface Salinity (Change in mean)	4.0	2.5	
	Sea Surface Salinity (Change in variability)	1.0	2.5	
	Ocean pH (Change in mean)	3.8	2.5	
	Ocean pH (Change in variability)	1.0	2.5	
	Sea ice coverage (Change in mean)	1.0	2.8	
	Sea ice coverage (Change in variability)	1.0	2.8	
	Dissolved oxygen (Change in mean)	3.8	2.5	
	Dissolved oxygen (Change in variability)	1.0	2.5	
	Circulation	2.8	2.0	
	Sea level rise	3.4	2.5	
	Exposure Score	Very High		
Overall Vulnerability Rank		Very High		

Low
Moderate
High
Very High

Common bottlenose dolphin (GoMx BSE/Florida Keys - Southwest Marathon Key to Marquesas Keys Stock)

Tursiops truncatus

CVA Results Summary

Overall Climate Vulnerability Rank: Very High (100% certainty from bootstrap analysis).

Climate Exposure: Very High. Four exposure factors scored greater than 3.5: Sea Surface Temperature (Standard anomaly) (4.00), Sea Surface Salinity (Standard anomaly) (3.95), Dissolved oxygen (Standard anomaly) (3.80), and Ocean pH (Standard anomaly) (3.80).

Biological Sensitivity: High. Five sensitivity attributes scored greater than 3.0: Migration (3.85), Site Fidelity (3.50), Species Abundance (3.35), Home Range (3.30), and Habitat Specificity (3.25).

Distributional Response: High

Abundance Response: High

Phenology Response: High

Data Quality: 85% of the data quality scores were 2 or greater, 64% of sensitivity attribute data quality scores were 2 or higher.

Background Information

Stock Description

This stock includes common bottlenose dolphins in the Florida Keys from southwest Marathon Key to Marquesas Keys in the eastern Gulf of Mexico (Hayes et al. 2022).

Prey/Diet Specificity

Generally, common bottlenose dolphins consume a large variety of fish and/or squid (Barros and Odell 1990; Mead and Potter 1990; Wells and Scott 2018). Common bottlenose dolphins seem to show a consistent preference for sound-producing fishes (e.g., sciaenids, toadfish; Berens McCabe et al. 2010; Bowen-Stevens et al. 2021). In Sarasota Bay, FL, Rossman et al. (2015) found some individual common bottlenose dolphins to show diet specialization despite the broader study group showing a generalist diet. Specialization can be influenced by locally abundant prey species (Shane et al. 1986; Vollmer and Rosel 2013) or can result from resource partitioning amongst sympatric populations (e.g., Torres and Read 2009; Wilson et al. 2017). On seagrass meadows off Key West, bottlenose dolphins create mud plumes to forage on fish, such as mullets (Lewis and Schroeder 2003).

Habitat Specificity

Common bottlenose dolphins are found in a variety of marine and estuarine habitats (Wells and Scott 2018).

Site Fidelity

No specific site fidelity information was found for this stock. However, common bottlenose dolphins in other Gulf of Mexico bay, sound, and estuary (BSE) stocks show site fidelity (LaBrecque et al. 2015; Hayes et al. 2022), such as in Matagorda Bay, Espiritu Santo Bay, and San Luis Pass in Texas (Maze and Würsig 1999; Lynn and Würsig 2002; Irwin and Würsig 2004); Barataria Bay in Louisiana (Miller 2003; Takeshita et al. 2021; Wells et al. 2017); Mississippi Sound in Mississippi (Hubard et al. 2004; Mackey 2010; Mullin et al. 2017); Charlotte Harbor and Pine Island Sound (Bassos-Hull et al. 2013), Sarasota Bay in Florida (Wells 1986a; Wells 1991; Wells 2014), St. Andrew Bay (Balmer et al. 2019), St. Joseph Bay (Balmer et al. 2008; B. Balmer et al. 2018), St. Vincent Sound and Apalachicola Bay (Tyson et al. 2011), and Tampa Bay in Florida (Wells et al. 1996b; Urian et al. 2009)

Lifetime Reproductive Potential

Taylor et al. (2007) reported the interbirth interval of common bottlenose dolphins as 3.80 years, though other studies have shown it to be closer to three years (Leatherwood 1977; Wells and Scott 1999; Connor et al. 2000; Wells 2003).

Generally, females reach sexual maturity before males, with females reaching sexual maturity at 5–13 years (Sergeant et al. 1973; Perrin and Reilly 1984; Wells et al. 1987; Mead and Potter 1990; Mann et al. 2000; Wells 2000; Wells and Scott 2018).

Females reproduce through most of their adult lives (Leatherwood 1977; Hohn et al. 1989; Wells and Scott 1999; Wells 2000; Wells 2003), but may produce fewer calves after they reach 30 years of age (Wells 2000). Sarasota Bay common bottlenose dolphins can give birth when they are as young as 6 years of age, and can continue until they are 48 years old (Wells and Scott 2018). Reproductive lifespan would therefore be up to 42 years based on long-term observation studies (Wells 2014; Wells and Scott 2018).

Longitudinal studies from Sarasota Bay, Florida have found that female common bottlenose dolphins can live up to 67 years (Wells and Scott 2018). Studies based on growth layer groups in teeth suggest female common bottlenose dolphins can live to 41 years along the Texas coast (Fernandez and Hohn 1998) and to 30 years along the Mississippi coast (Mattson et al. 2006).

Generation Length

Taylor et al. (2007) reported common bottlenose dolphin generation length of 20.6–21.1 years.

Reproductive Plasticity

Common bottlenose dolphin calving is generally diffusely seasonal, with one or more peaks, although births have been reported from all seasons (Vollmer and Rosel 2013; Wells and Scott 2018). Calf sightings peak in the spring and summer months in Florida (Wells et al. 1987), Mississippi/Louisiana (Miller et al. 2010; Miller et al. 2013), and Texas (McHugh 1989; Henderson et al. 2007).

Migration

The BSE stocks in the Gulf of Mexico are thought to primarily comprise stable resident communities. However, transient or seasonal movements of some individuals into and out of a BSE stock area are recorded in some areas (LaBrecque et al. 2015; Hayes et al. 2022). Molecular genetic data support that some BSE stocks are genetically distinct from one another, and furthermore are differentiated from adjacent coastal populations (e.g., Sellas et al. 2005; Rosel et al. 2017). For example, Toms (2019) found fine-scale genetic population structure among five inshore systems in the Florida Panhandle and variable migration rates among populations.

Year-round residency has been reported for common bottlenose dolphins (Hayes et al. 2022), such as in Florida (Irvine and Wells 1972; Irvine et al. 1981; Wells 1986a; Wells 1986b; Wells et al. 1987; Scott et al. 1990; Wells 1991; Wells et al. 1996a; Wells et al. 1996b; Wells et al. 1997; Wells 2003; Balmer et al. 2008; Urian et al. 2009; Tyson et al. 2011; Bassos-Hull et al. 2013; B. Balmer et al. 2018; Balmer et al. 2019; Toms 2019), Texas (Shane 1990; Bräger 1993; Bräger et al. 1994; Fertl 1994; Weller 1998; Maze and Würsig 1999; Lynn and Würsig 2002; Irwin and Würsig 2004; Shane 2004), and Louisiana/Mississippi (Hubard et al. 2004; Mullin et al. 2017; Takeshita et al. 2021).

Home Range

The home ranges of individuals within the Gulf of Mexico BSE stocks are considered to generally include the areas within the stock boundaries and brief excursions beyond the stock boundaries (Irvine et al. 1981; Wells 1986a; Wells 1986b; Wells 1991; Wells et al. 1996a; Wells et al. 1996b; Balmer et al. 2008; Wells 2014; Balmer et al. 2019), although some individuals show preference for small areas within the stock boundaries (Lynn and Würsig 2002; Urian et al. 2009; Mullin et al. 2017; Wells et al. 2017).

Stock Abundance

An abundance estimate for this stock is unavailable (Hayes et al. 2022).

Stock Abundance Trend

Data are insufficient to complete an abundance trend analysis for this stock (Hayes et al. 2022).

Cumulative Stressors

Common bottlenose dolphins face a wide range of individual and cumulative natural and anthropogenic threats (see reviews by Vollmer and Rosel 2013; Wells and Scott 2018; and Wells et al. 2019). These include such threats as shark predation, harmful algal blooms, recreational and commercial fishing gear, vessel traffic, pollution, habitat alteration, and provisioning.

The nearshore and estuarine habitats along the Gulf of Mexico coast include waters adjacent to areas of high human population, some of which are highly industrialized, and areas of heavy agricultural impact. Studies of bottlenose dolphin tissues from southwest Florida have shown persistent organic pollutant (POP) in blubber and blood samples (Kucklick et al. 2011; Balmer et al. 2015; Damseaux et al. 2017; J. Balmer et al. 2018). Similarly, mercury has been detected in common bottlenose dolphin tissues in this stock (Damseaux et al. 2017) and elsewhere in Florida Gulf Coast waters (McCormack et al. 2020a, 2020b, 2022).

An Unusual Mortality Event (UME) was declared in 1990 for the Gulf of Mexico from Florida to Texas, with more than 300 common bottlenose dolphins stranding due to an undetermined cause, although morbillivirus and cold temperatures may have contributed (Litz et al. 2014).

Further Reading

Hayes, S.A., E. Josephson, K. Maze-Foley, P.E. Rosel, and J. Wallace. 2022. U.S. Atlantic and Gulf of Mexico marine mammal stock assessments 2021. National Oceanic and Atmospheric Administration, National Marine Fisheries Service, Northeast Fisheries Science Center: Woods Hole, MA. NOAA Technical Memorandum NMFS-NE-288. 380 p. doi: 10.25923/6tt7-kc16

Wells, R.S. and M.D. Scott. 2018. Bottlenose Dolphin, *Tursiops truncatus*, Common Bottlenose Dolphin. In: Würsig, B., J.G.M. Thewissen, and K.M. Kovacs (Eds.), Encyclopedia of Marine Mammals (Third Edition). Academic Press, London, UK. pp. 118–125. doi: 10.1016/B978-0-12-804327-1.00072-8

Wells, R.S., A. Natoli and G. Braulik. 2019. *Tursiops truncatus* (errata version published in 2019). The IUCN Red List of Threatened Species 2019: e.T22563A156932432. https://dx.doi.org/10.2305/IUCN.UK.2019-1.RLTS.T22563A156932432.en

References

Balmer, B., T. McDonald, F. Hornsby, J. Adams, J. Allen, A. Barleycorn, and L. Schwacke. 2018. Long-term trends in a northern Gulf of Mexico common bottlenose dolphin (*Tursiops truncatus*) population in the wake of the Deepwater Horizon oil spill. Journal of Cetacean Research and Management 18:1–9. doi: 10.47536/jcrm.v18i1.409

Balmer, B., S. Watwood, B. Quigley, T. Speakman, K. Barry, K. Mullin, P. Rosel, C. Sinclair, E. Zolman, and L. Schwacke. 2019. Common bottlenose dolphin (*Tursiops truncatus*) abundance and distribution patterns in St. Andrew Bay, Florida, USA. Aquatic Conservation: Marine and Freshwater Ecosystems 29(3):486–498. doi: 10.1002/aqc.3001

Balmer, B.C., R.S. Wells, S.M. Nowacek, D.P. Nowacek, L.H. Schwacke, W.A. McLellan, F.S. Scharf, T.K. Rowles, L.J. Hansen, T.R. Spradlin, and D.A. Pabst. 2008. Seasonal abundance and distribution patterns of common bottlenose dolphins (*Tursiops truncatus*) near St. Joseph Bay, Florida, USA. Journal of Cetacean Research and Management 10(2):157–167.

Balmer, B.C., G.M. Ylitalo, L.E. McGeorge, K.A. Baugh, D. Boyd, K.D. Mullin, P.E. Rosel, C. Sinclair, R.S. Wells, E.S. Zolman, and L.H. Schwacke. 2015. Persistent organic pollutants (POPs) in blubber of common bottlenose dolphins (*Tursiops truncatus*) along the northern Gulf of Mexico coast, USA. Science of the Total Environment 527-528:306–312. doi: 10.1016/j.scitotenv.2015.05.016

Balmer, J.E., G.M. Ylitalo, T.K. Rowles, K.D. Mullin, R.S. Wells, F.I. Townsend, R.W. Pearce, J.L. Bolton, E.S. Zolman, B.C. Balmer, and L.H. Schwacke. 2018. Persistent organic pollutants (POPs) in blood and blubber of common bottlenose dolphins (*Tursiops truncatus*) at three northern Gulf of Mexico sites following the Deepwater Horizon oil spill. Science of the Total Environment 621:130–137. doi: 10.1016/j.scitotenv.2017.11.209

Barros, N.B. and D.K. Odell. 1990. Food habits of bottlenose dolphins in the southeastern United States In: Leatherwood, S. and R.R. Reeves (Eds.), The Bottlenose Dolphin. Academic Press, San Diego, CA. pp. 309–328. doi: 10.1016/B978-0-12-440280-5.50020-2

Bassos-Hull, K., R.M. Perrtree, C.C. Shepard, S. Schilling, A.A. Barleycorn, J.B. Allen, B.C. Balmer, W.E. Pine, and R.S. Wells. 2013. Long-term site fidelity and seasonal abundance estimates of common bottlenose dolphins (*Tursiops truncatus*) along the southwest coast of Florida and responses to

natural perturbations. Journal of Cetacean Research and Management 13(1):19–30. doi: 10.47536/jcrm.v13i1.551

Berens McCabe, E.J., D.P. Gannon, N.B. Barros, and R.S. Wells. 2010. Prey selection by resident common bottlenose dolphins (*Tursiops truncatus*) in Sarasota Bay, Florida. Marine Biology 157(5):931–942. doi: 10.1007/s00227-009-1371-2

Bowen-Stevens, S.R., D.P. Gannon, R.A. Hazelkorn, G. Lovewell, K.M. Volker, S. Smith, M.C. Tumlin, and J. Litz. 2021. Diet of common bottlenose dolphins, *Tursiops truncatus*, that stranded in and near Barataria Bay, Louisiana, 2010–2012. Southeastern Naturalist 20(1). doi: 10.1656/058.020.0113

Bräger, S. 1993. Diurnal and seasonal behavior patterns of bottlenose dolphins (*Tursiops truncatus*). Marine Mammal Science 9:434–438. doi: 10.1111/j.1748-7692.1993.tb00477.x

Bräger, S., B. Würsig, A. Acevedo, and T. Henningsen. 1994. Association patterns of bottle-nosed dolphins (*Tursiops-truncatus*) in Galveston Bay, Texas. Journal of Mammalogy 75(2):431–437. doi: 10.2307/1382564

Connor, R.C., R.S. Wells, J. Mann, and A.J. Read. 2000. The bottlenose dolphin: Social relationships in a fission-fusion society. In: Mann, J., R. Connor, P. Tyack, and H. Whitehead (Eds.), Cetacean Societies: Field Studies of Dolphins and Whales. University of Chicago Press, Chicago, IL. pp. 91–126.

Damseaux, F., J.J. Kiszka, M.R. Heithaus, G. Scholl, G. Eppe, J.P. Thome, J. Lewis, W. Hao, M.C. Fontaine, and K. Das. 2017. Spatial variation in the accumulation of POPs and mercury in bottlenose dolphins of the Lower Florida Keys and the coastal Everglades (South Florida). Environmental Pollution 220(Pt A):577–587. doi: 10.1016/j.envpol.2016.10.005

Fernandez, S. and A.A. Hohn. 1998. Age, growth, and calving season of bottlenose dolphins, *Tursiops truncatus*, off coastal Texas. Fishery Bulletin 96(2):357–365.

Fertl, D. 1994. Occurrence patterns and behavior of bottlenose dolphins (*Tursiops truncatus*) in the Galveston ship channel, Texas. Texas Journal of Science 46(4):299–318.

Hayes, S.A., E. Josephson, K. Maze-Foley, P.E. Rosel, and J. Wallace. 2022. U.S. Atlantic and Gulf of Mexico marine mammal stock assessments 2021. National Oceanic and Atmospheric Administration, National Marine Fisheries Service, Northeast Fisheries Science Center: Woods Hole, MA. NOAA Technical Memorandum NMFS-NE-288. 380 p. doi: 10.25923/6tt7-kc16

Henderson, E.E. and B. Würsig. 2007. Behavior patterns of bottlenose dolphins in San Luis Pass, Texas. Gulf of Mexico Science 25(2):153–161. doi: 10.18785/goms.2502.06

Hohn, A.A., M.D. Scott, R.S. Wells, J.C. Sweeney, and A.B. Irvine. 1989. Growth layers in teeth from known-age, free-ranging bottlenose dolphins. Marine Mammal Science 5(4):315–342. doi: 10.1111/j.1748-7692.1989.tb00346.x

Hubard, C.W., K. Maze-Foley, K.D. Mullin, and W.W. Schroeder. 2004. Seasonal abundance and site fidelity of bottlenose dolphins (*Tursiops truncatus*) in Mississippi Sound. Aquatic Mammals 30(2):299–310. doi: 10.1578/am.30.2.2004.299

Irvine, A.B., M.D. Scott, R.S. Wells, and J.H. Kaufmann. 1981. Movements and activities of the Atlantic bottlenose dolphin, *Tursiops truncatus*, near Sarasota, Florida. Fishery Bulletin 79(4):671–688.

Irvine, B. and R.S. Wells. 1972. Results of attempts to tag Atlantic bottlenosed dolphins (*Tursiops truncatus*). Cetology 13:1–5.

Irwin, L.-J. and B. Würsig. 2004. A small resident community of bottlenose dolphins, *Tursiops truncatus*, in Texas: Monitoring recommendations. Gulf of Mexico Science 22(1):13–21. doi: 10.18785/goms.2201.02

Kucklick, J., L. Schwacke, R. Wells, A. Hohn, A. Guichard, J. Yordy, L. Hansen, E. Zolman, R. Wilson, J. Litz, D. Nowacek, T. Rowles, R. Pugh, B. Balmer, C. Sinclair, and P. Rosel. 2011. Bottlenose dolphins as indicators of persistent organic pollutants in the western North Atlantic Ocean and Northern

Gulf of Mexico. Environmental Science & Technology 45(10):4270–4277. doi: 10.1021/es1042244
LaBrecque, E., C. Curtice, J. Harrison, S.M. Van Parijs, and P.N. Halpin. 2015. 3. Biologically Important Areas for cetaceans within U.S. Waters – Gulf of Mexico Region. Aquatic Mammals 41(1):30–38. doi: 10.1578/am.41.1.2015.30
Leatherwood, S. 1977. Some preliminary impressions on the numbers and social behavior of free-swimming bottlenosed dolphin calves (*Tursiops truncatus*) in the northern Gulf of Mexico. In: Ridgway, S.H. and K. Bernirschke (Eds.), Breeding Dolphins; Present Status, Suggestions for the Future. Final report to the US Marine Mammal Commission, Contract MM6AC009, MMC-76/07, Washington, DC. pp. 143–167.
Lewis, J.S. and W.W. Schroeder. 2003. Mud plume feeding, a unique foraging behavior of the bottlenose dolphin in the Florida Keys. Gulf of Mexico Science 21(1):92–97. doi: 10.18785/goms.2101.09
Litz, J.A., M.A. Baran, S.R. Bowen-Stevens, R.H. Carmichael, K.M. Colegrove, L.P. Garrison, S.E. Fire, E.M. Fougeres, R. Hardy, S. Holmes, W. Jones, B.E. Mase-Guthrie, D.K. Odell, P.E. Rosel, J.T. Saliki, D.K. Shannon, S.F. Shippee, S.M. Smith, E.M. Stratton, M.C. Tumlin, H.R. Whitehead, G.A. Worthy, and T.K. Rowles. 2014. Review of historical unusual mortality events (UMEs) in the Gulf of Mexico (1990–2009): providing context for the multi-year northern Gulf of Mexico cetacean UME declared in 2010. Diseases of Aquatic Organisms 112(2):161–175. doi: 10.3354/dao02807
Lynn, S.K. and B. Würsig. 2002. Summer movement patterns of bottlenose dolphins in a Texas bay. Gulf of Mexico Science 20(1):25–37. doi: 10.18785/goms.2001.03
Mackey, A.D. 2010. Site fidelity and association patterns of bottlenose dolphins (*Tursiops truncatus*) in the Mississippi Sound. University of Southern Mississippi. Thesis. 133 p.
Mann, J., R.C. Connor, L.M. Barre, and M.R. Heithaus. 2000. Female reproductive success in bottlenose dolphins (*Tursiops* sp.): life history, habitat, provisioning, and group-size effects. Behavioral Ecology 11(2):210–219. doi: 10.1093/beheco/11.2.210
Mattson, M.C., K.D. Mullin, G.W. Ingram, and W. Hoggard. 2006. Age structure and growth of the bottlenose dolphin (*Tursiops truncatus*) from strandings in the Mississippi sound region of the north-central Gulf of Mexico from 1986 to 2003. Marine Mammal Science 22(3):654–666. doi: 10.1111/j.1748-7692.2006.00057.x
Maze, K.S. and B. Würsig. 1999. Bottlenosed dolphins of San Luis Pass, Texas: Occurrence patterns, site-fidelity, and habitat use. Aquatic Mammals 25:91–104.
McCormack, M.A., F. Battaglia, W.E. McFee, and J. Dutton. 2020. Mercury concentrations in blubber and skin from stranded bottlenose dolphins (*Tursiops truncatus*) along the Florida and Louisiana coasts (Gulf of Mexico, USA) in relation to biological variables. Environmental Research 180:108886. doi: 10.1016/j.envres.2019.108886
McCormack, M.A., B.P. Jackson, and J. Dutton. 2020. Relationship between mercury and selenium concentrations in tissues from stranded odontocetes in the northern Gulf of Mexico. Science of the Total Environment 749:141350. doi: 10.1016/j.scitotenv.2020.141350
McCormack, M.A., W.H. Nowlin, and J. Dutton. 2022. Effect of trophic position on mercury concentrations in bottlenose dolphins (*Tursiops truncatus*) from the northern Gulf of Mexico. Environmental Research 204(Pt B):112124. doi: 10.1016/j.envres.2021.112124
McHugh, M.B. 1989. Population numbers and feeding behavior of the Atlantic bottlenose dolphin (*Tursiops truncatus*) near Aransas Pass, Texas. University of Texas at Austin. Thesis. 96 p.
Mead, J.G. and C.W. Potter. 1990. Natural history of bottlenose dolphins along the central Atlantic coast of the United States. In: Leatherwood, S. and R.R. Reeves (Eds.), The Bottlenose Dolphin. Academic Press, San Diego, CA. pp. 165–195.

Miller, C.E. 2003. Abundance trends and environmental habitat usage patterns of bottlenose dolphins (*Tursiops truncatus*) in lower Barataria and Caminada bays, Louisiana. Louisiana State University and Agricultural & Mechanical College. Dissertation. 125 p.

Miller, L.J., A.D. Mackey, T. Hoffland, M. Solangi, and S.A. Kuczaj. 2010. Potential effects of a major hurricane on Atlantic bottlenose dolphin (*Tursiops truncatus*) reproduction in the Mississippi Sound. Marine Mammal Science 26(3):707–715. doi: 10.1111/j.1748-7692.2010.00371.x

Miller, L.J., A.D. Mackey, M. Solangi, and S.A. Kuczaj. 2013. Population abundance and habitat utilization of bottlenose dolphins in the Mississippi Sound. Aquatic Conservation: Marine and Freshwater Ecosystems 23(1):145–151. doi: 10.1002/aqc.2278

Mullin, K.D., T. McDonald, R.S. Wells, B.C. Balmer, T. Speakman, C. Sinclair, E.S. Zolman, F. Hornsby, S.M. McBride, K.A. Wilkinson, and L.H. Schwacke. 2017. Density, abundance, survival, and ranging patterns of common bottlenose dolphins (*Tursiops truncatus*) in Mississippi Sound following the Deepwater Horizon oil spill. PLoS ONE 12(10):e0186265. doi: 10.1371/journal.pone.0186265

Perrin, W.F. and S.B. Reilly. 1984. Reproductive parameters of dolphins and small whales of the family Delphinidae. In: Perrin, W.F., R.L. Brownell, Jr., and D.P. DeMaster (Eds.), Reproduction in Whales, Dolphins, and Porpoises. pp. 97–134.

Rosel, P.E., L.A. Wilcox, C. Sinclair, T.R. Speakman, M.C. Tumlin, J.A. Litz, and E.S. Zolman. 2017. Genetic assignment to stock of stranded common bottlenose dolphins in southeastern Louisiana after the Deepwater Horizon oil spill. Endangered Species Research 33:221–234. doi: 10.3354/esr00780

Rossman, S., P.H. Ostrom, M. Stolen, N.B. Barros, H. Gandhi, C.A. Stricker, and R.S. Wells. 2015. Individual specialization in the foraging habits of female bottlenose dolphins living in a trophically diverse and habitat rich estuary. Oecologia 178(2):415–425. doi: 10.1007/s00442-015-3241-6

Scott, M.D. and S.J. Chivers. 1990. Distribution and herd structure of bottlenose dolphins in the eastern tropical Pacific Ocean. In: Leatherwood, S. and R.R. Reeves (Eds.), The Bottlenose Dolphin. Academic Press, San Diego, CA. pp. 387–402.

Sellas, A.B., R.S. Wells, and P.E. Rosel. 2005. Mitochondrial and nuclear DNA analyses reveal fine scale geographic structure in bottlenose dolphins (*Tursiops truncatus*) in the Gulf of Mexico. Conservation Genetics 6(5):715–728. doi: 10.1007/s10592-005-9031-7

Sergeant, D.E., D.K. Caldwell, and M.C. Caldwell. 1973. Age, growth, and maturity of bottlenosed dolphin (*Tursiops-truncatus*) from northeast Florida. Journal of the Fisheries Research Board of Canada 30(7):1009–1011. doi: 10.1139/f73-165

Shane, S.H. 1990. Behavior and ecology of the bottlenose dolphin at Sanibel Island, Florida. In: Leatherwood, S. and R.R. Reeves (Eds.), The Bottlenose Dolphin. Academic Press, San Diego, CA. pp. 245–265. doi: 10.1016/B978-0-12-440280-5.50016-0

Shane, S.H. 2004. Residence patterns, group characteristics, and association patterns of bottlenose dolphins near Sanibel Island, Florida. Gulf of Mexico Science 22(1):1–12. doi: 10.18785/goms.2201.01

Shane, S.H., R.S. Wells, and B. Würsig. 1986. Ecology, behavior and social organization of the bottlenose dolphin: A review. Marine Mammal Science 2:34–63. doi: 10.1111/j.1748-7692.1986.tb00026.x

Takeshita, R., B.C. Balmer, F. Messina, E.S. Zolman, L. Thomas, R.S. Wells, C.R. Smith, T.K. Rowles, and L.H. Schwacke. 2021. High site-fidelity in common bottlenose dolphins despite low salinity exposure and associated indicators of compromised health. PLoS ONE 16(9):e0258031. doi: 10.1371/journal.pone.0258031

Taylor, B.L., S.J. Chivers, J. Larese, and W.F. Perrin. 2007. Generation length and percent mature estimates for IUCN assessments of cetaceans. National Oceanic and Atmospheric

Administration, National Marine Fisheries Service, Southwest Fisheries Science Center: La Jolla, CA. Administrative Report LJ-07-01. 24 p.

Toms, C. 2019. Filling the gaps: Common bottlenose dolphin (*Tursiops truncatus*) population dynamics, structure, and connectivity within Florida panhandle bays, sounds, and estuaries. University of Central Florida. Dissertation. 373 p.

Torres, L.G. and A.J. Read. 2009. Where to catch a fish? The influence of foraging tactics on the ecology of bottlenose dolphins (*Tursiops truncatus*) in Florida Bay, Florida. Marine Mammal Science 25(4):797–815. doi: 10.1111/j.1748-7692.2009.00297.x

Tyson, R.B., S.M. Nowacek, and D.P. Nowacek. 2011. Community structure and abundance of bottlenose dolphins *Tursiops truncatus* in coastal waters of the northeast Gulf of Mexico. Marine Ecology Progress Series 438:253–265. doi: 10.3354/meps09292

Urian, K.W., S. Hofmann, R.S. Wells, and A.J. Read. 2009. Fine-scale population structure of bottlenose dolphins (*Tursiops truncatus*) in Tampa Bay, Florida. Marine Mammal Science 25(3):619–638. doi: 10.1111/j.1748-7692.2009.00284.x

Vollmer, N.L. and P.E. Rosel. 2013. A review of common bottlenose dolphins (*Tursiops truncatus truncatus*) in the Northern Gulf of Mexico: Population biology, potential threats, and management. Southeastern Naturalist 12(m6):1–43. doi: 10.1656/058.012.m601

Weller, D.W. 1998. Global and regional variation in the biology and behavior of bottlenose dolphins. Texas A&M University. Dissertation. 142 p.

Wells, R.S. 1986a. Population structure of bottlenose dolphins: Behavioral studies along the central west coast of Florida. Contract Report to National Marine Fisheries Service, Southeast Fisheries Center. Contract No. 45-WCNF-5-003BB. 58 p.

Wells, R.S. 1986b. Structural aspects of dolphin societies. University of California, Santa Cruz. Dissertation. 234 p.

Wells, R.S. 1991. The role of long-term study in understanding the social structure of a bottlenose dolphin community. In: Pryor, K. and K. Norris (Eds.), Dolphin Societies: Discoveries and Puzzles. University of California Press, Berkeley, CA. pp. 199–226.

Wells, R.S. 2000. Reproduction in wild bottlenose dolphins: Overview of patterns observed during a long-term study. In: Duffield, D. and T. Robeck (Eds.), Bottlenose Dolphin Reproduction Workshop. AZA Marine Mammal Taxon Advisory Group, Silver Spring, MD. pp. 57–74.

Wells, R.S. 2003. Dolphin social complexity: Lessons from long-term study and life history. In: de Waal, F.B.M. and P.L. Tyack (Eds.), Animal Social Complexity: Intelligence, Culture, and Individualized Societies Harvard University Press, Cambridge, MA. pp. 32–56.

Wells, R.S. 2014. Social structure and life history of bottlenose dolphins near Sarasota Bay, Florida: Insights from four decades and five generations. Primates and cetaceans. pp. 149–172. doi: 10.1007/978-4-431-54523-1_8

Wells, R.S., M.K. Bassos, K.W. Urian, W.J. Carr, and M.D. Scott. 1996. Low-level monitoring of bottlenose dolphins, *Tursiops truncatus*, in Charlotte Harbor, Florida: 1990-1994. National Oceanic and Atmospheric Administration, National Marine Fisheries Service, Southeast Fisheries Science Center: Miami, FL. NOAA Technical Memorandum NMFS-SEFSC-384. 86 p.

Wells, R.S., M.K. Bassos, K.W. Urian, S.H. Shane, E.C.G. Owen, C.F. Weiss, W.J. Carr, and M.D. Scott. 1997. Low-level monitoring of bottlenose dolphins, *Tursiops truncatus*, in Pine Island Sound, Florida: 1996. National Oceanic and Atmospheric Administration, National Marine Fisheries Service, Southeast Fisheries Science Center: Miami, FL. Southeast Fisheries Center Contribution No. 40-WCNF601958. 86 p.

Wells, R.S., A. Natoli, and G. Braulik. 2019. *Tursiops truncatus* (errata version published in 2019). *The IUCN Red List of Threatened Species* 2019: e.T22563A156932432. https://dx.doi.org/10.2305/IUCN.UK.2019-1.RLTS.T22563A156932432.en

Wells, R.S., L.H. Schwacke, T.K. Rowles, B.C. Balmer, E. Zolman, T. Speakman, F.I. Townsend, M.C. Tumlin, A. Barleycorn, and K.A. Wilkinson. 2017. Ranging patterns of common bottlenose dolphins *Tursiops truncatus* in Barataria Bay, Louisiana, following the Deepwater Horizon oil spill. Endangered Species Research 33:159–180. doi: 10.3354/esr00732

Wells, R.S. and M.D. Scott. 1999. Bottlenose dolphin *Tursiops truncatus* (Montagu, 1821). In: Ridgway, S.H. and R. Harrison (Eds.), Handbook of marine mammals. Volume 6: The second book of dolphins and the porpoises. Academic Press, San Diego, CA. pp. 137–182.

Wells, R.S. and M.D. Scott. 2018. Bottlenose dolphin, *Tursiops truncatus*, common bottlenose dolphin. In: Würsig, B., J.G.M. Thewissen, and K.M. Kovacs (Eds.), Encyclopedia of Marine Mammals (Third Edition). Academic Press, London, UK. pp. 118–125. doi: 10.1016/B978-0-12-804327-1.00072-8

Wells, R.S., M.D. Scott, and A.B. Irvine. 1987. The social structure of free-ranging bottlenose dolphins. In: Genoways, H.H. (Ed.), Current Mammalogy. Plenum Press, New York, NY. pp. 247–305.

Wells, R.S., K.W. Urian, A.J. Read, M.K. Bassos, W.J. Carr, and M.D. Scott. 1996. Low-level monitoring of bottlenose dolphins, *Tursiops truncatus*, in Tampa Bay, Florida: 1988-1993. National Oceanic and Atmospheric Administration, National Marine Fisheries Service, Southeast Fisheries Science Center: Miami, FL. NOAA Technical Memorandum NMFS-SEFSC-385. 36 p.

Wilson, R.M., R.B. Tyson, J.A. Nelson, B.C. Balmer, J.P. Chanton, and D.P. Nowacek. 2017. Niche differentiation and prey selectivity among common bottlenose dolphins (*Tursiops truncatus*) sighted in St. George Sound, Gulf of Mexico. Frontiers in Marine Science 4:235. doi: 10.3389/fmars.2017.00235

Common bottlenose dolphin − *Tursiops truncatus*

GoMx BSE/Galveston Bay, East Bay, Trinity Bay

Overall Vulnerability Rank = Very High

Biological Sensitivity = Very High

Climate Exposure = Very High

Data Quality = 96% of scores ≥ 2

	Tursiops truncatus	Expert Scores	Data Quality	Expert Scores Plots (Portion by Category)
Sensitivity attributes	Prey/Diet Specificity	2.4	2.2	
	Habitat Specificity	2.8	2.2	
	Site Fidelity	3.4	2.8	
	Lifetime Reproductive Potential	1.6	2.2	
	Generation Length	2.9	2.2	
	Reproductive Plasticity	1.2	2.0	
	Migration	3.8	2.8	
	Home Range	3.5	2.8	
	Stock Abundance	2.8	2.2	
	Stock Abundance Trend	1.8	1.8	
	Cumulative Stressors	3.9	2.8	
	Sensitivity Score	Very High		
Exposure variables	Sea Surface Temperature (Change in mean)	4.0	2.8	
	Sea Surface Temperature (Change in variability)	1.0	2.8	
	Air Temperature (Change in mean)	4.0	2.8	
	Air Temperature (Change in variability)	1.0	2.8	
	Precipitation (Change in mean)	1.0	2.8	
	Precipitation (Change in variability)	1.0	2.8	
	Sea Surface Salinity (Change in mean)	2.0	2.8	
	Sea Surface Salinity (Change in variability)	1.0	2.8	
	Ocean pH (Change in mean)	4.0	2.8	
	Ocean pH (Change in variability)	1.0	2.8	
	Sea ice coverage (Change in mean)	1.0	2.8	
	Sea ice coverage (Change in variability)	1.0	2.8	
	Dissolved oxygen (Change in mean)	4.0	2.8	
	Dissolved oxygen (Change in variability)	1.0	2.8	
	Circulation	3.8	2.5	
	Sea level rise	4.0	2.8	
	Exposure Score	Very High		
	Overall Vulnerability Rank	Very High		

Low
Moderate
High
Very High

Common bottlenose dolphin (GoMx BSE/Galveston Bay, East Bay, Trinity Bay Stock)

Tursiops truncatus

CVA Results Summary

Overall Climate Vulnerability Rank: Very High (100% certainty from bootstrap analysis).

Climate Exposure: Very High. Six exposure factors scored greater than 3.5: Air Temperature (Standard anomaly) (4.00), Dissolved oxygen (Standard anomaly) (4.00), Ocean pH (Standard anomaly) (4.00), Sea level rise (4.00), Sea Surface Temperature (Standard anomaly) (4.00), and Circulation (3.85).

Biological Sensitivity: Very High. Three sensitivity attributes scored greater than or equal to 3.5: Cumulative Stressors (3.90), Migration (3.85), and Home Range (3.50).

Distributional Response: High

Abundance Response: High

Phenology Response: High

Data Quality: 96% of the data quality scores were 2 or greater, 91% of sensitivity attribute data quality scores were 2 or higher.

Background Information

Stock Description

This stock includes common bottlenose dolphins in Galveston Bay, East Bay, and Trinity Bay in the western Gulf of Mexico along the Texas coast (Hayes et al. 2022).

Prey/Diet Specificity

Generally, common bottlenose dolphins consume a large variety of fish and/or squid (Barros and Odell 1990; Mead and Potter 1990; Wells and Scott 2018). Common bottlenose dolphins seem to show a consistent preference for noise-producing fishes (e.g., sciaenids, toadfish; Berens McCabe et al. 2010; Bowen-Stevens et al. 2021). In Sarasota Bay, FL, Rossman et al. (2015) found some individual common bottlenose dolphins to show diet specialization despite the broader study group showing a generalist diet. Specialization can be influenced by locally abundant prey species (Shane et al. 1986; Vollmer and Rosel 2013) or can result from resource partitioning amongst sympatric populations (e.g., Torres and Read 2009; Wilson et al. 2017).

Preliminary estimates of prey consumption proportions for common bottlenose dolphins in the Galveston Bay estuary were determined through stable isotope analysis and Bayesian mixing models (McDaniel and Guillen 2020). In this study, examination of 36 remote biopsy samples obtained from free ranging dolphins in Galveston Bay between 2015-2017 indicated striped mullet (*Mugil cephalus*) and spotted seatrout (*Cynoscion nebulosus*) as primary prey sources. Additional contributions came from

white shrimp (*Litopenaeus setiferus*), hardhead catfish (*Ariopsis felis*) and likely fish with similar δ13C and δ15N values not included in the study. The authors suggested prey abundance may contribute to disparities in estimated contributions of prey sources between years (McDaniel and Guillen 2020). Barros and Odell (1990) examined the stomach contents of 76 stranded common bottlenose dolphins from Gulf of Mexico shores, 22 of which were from the shoreline of northeast Texas adjacent to the Galveston Bay systems. Those 22 samples were combined with 3 additional stomach content analyses from common bottlenose dolphin strandings recovered in the states of Louisiana, Mississippi, and Alabama (1 sample each), and designated those states as Area 1 of their study. Using a combination of visual taxonomic identification (for undigested species) and species identification keys for fish otolith and cephalopod beaks, Barros and Odell (1990) reported the proportion of prey in Area 1 as fish (94.3%), cephalopods (5.2%), crustaceans (0.5%), and a single horseshoe crab. Only 32% of the stomach contents from Area 1 contained fish only, while fish/cephalopod and fish/cephalopod/shrimp were present in 36% and 32% respectively, of Area 1 stomach contents (Barros and Odell 1990). Overall, sciaenid fishes were the primary fish prey. However, the great diversity of species (over 300 different fish species found in eight stomachs from Area 1) and similarity of species to commercial fisheries bycatch suggested common bottlenose dolphins off Texas were supplementing their diet by depredating shrimp trawler bycatch or by taking fish directly out of the nets (presumably) during trawling operations (Barros and Odell 1990). Observations of common bottlenose dolphins feeding using both methods from Texas and Louisiana have been documented (Gunter 1938; Shane 1977; Gruber 1981; Henningsen and Würsig 1991; Fertl 1994a; Moreno 2005; Ronje et al. 2018).

While much research indicates sciaenid fishes are the primary prey fishes for common bottlenose dolphins in the Gulf of Mexico (Berens McCabe et al. 2010; Dunshea et al. 2013; Bowen-Stevens et al. 2021), other researchers (Gunter 1942; Shane 1990; Miller 1992) have suggested striped mullet (*Mugil cephalus*) may be a significant part of the bottlenose dolphin diet in Texas waters. Barros and Wells (1998) reviewed the importance of mullet in the diet of common bottlenose dolphins in Florida waters and suggested the species is important, yet ranks under soniferous fishes in their dietary profile.

Habitat Specificity

Common bottlenose dolphins in the Galveston Bay system often concentrate in Bolivar Roads, an energetic tidal pass between Galveston Island and the Bolivar Peninsula (Moreno and Mathews 2018; Bräger et al. 1994; Jones III 1988; Maze and Würsig 1999; Ronje et al. 2020). Relatively high common bottlenose dolphin group density has also been observed in the Galveston Ship Channel (Fertl 1994a; Moreno and Mathews 2018; Piwetz 2019; Ronje et al. 2020). These bay sub-areas are likely mixing zones for transient dolphins from the Western Coastal stock (Hayes et al. 2022), as well as resident Galveston Bay dolphins, all utilizing the deep, prey-rich engineered channels where estuary and Gulf waters meet (Maze and Würsig 1999; Ronje et al. 2020). Seasonal distribution patterns are evident throughout portions of Texas in bays, inlets and nearshore waters. Seasonal changes in Galveston Bay population density may reflect a combination of intra-bay, inter-bay, or coastal movements. Studies of lower Galveston Bay near Bolivar Roads have documented seasonal increases in dolphin activity in spring and late summer through fall, with decreases in winter months (Fertl 1994b; Jones III 1988). Ronje et al. (2020) conducted spatially comprehensive winter and summer photo-ID mark-recapture surveys of the Galveston Bay system and estimated a greater abundance of dolphins were present in summer. Similar seasonal relative abundance increases in upper Galveston Bay have been documented during “warm”

months (May - October, water temperature typically > 23°C) with fewer dolphins sighted in the upper Galveston Bay during "cold" months (November - April, water temperature typically < 23°C; Fazioli et al. 2015; Mintzer and Fazioli 2021). Previous studies have not identified strong seasonal fluctuations in abundance in the adjacent West Bay stock (Maze and Würsig 1999); but a higher density of West Bay resident dolphins was observed in the Gulf waters near San Luis Pass during cold months, and in the bay during warm months (Irwin and Würsig 2004; Henderson and Würsig 2007). More recent studies estimated slightly higher abundance in the West Bay stock area delineated by the NMFS during winter (Litz et al. 2019; Ronje et al. 2020). Similarly, in the coastal bend region of Texas near the passes, an increase in abundance during winter months and corresponding decrease during the summer has been noted in several studies (Shane 1980; McHugh 1989; Gruber 1981). Seasonal changes in density throughout Texas estuaries have been attributed to a combination of north-south migration along the coast influenced by water temperature and more localized shifts in distribution influenced by prey movements in and out of the estuary during different times of the year (Weller 1998).

Systematic and spatially comprehensive surveys have been conducted in Trinity Bay and East Bay as well as opportunistic surveys; however, dolphin groups were generally observed only on the western margins of those areas (GDRP 2021 unpublished data; Ronje et al. 2020). The overall distribution of bottlenose dolphins in the Galveston Bay system appears to be positively correlated on a north-south axis, consistent with the general gradient of salinity and distance to coast (Moreno and Mathews 2018). Mintzer and Fazioli (2021) found that both salinity and temperature predicted dolphin movements in and out of upper Galveston Bay, with an exodus of dolphins occurring with low salinity levels, regardless of the time of year and water temperature.

Site Fidelity

Photographic identification studies of areas in lower Galveston Bay and nearshore Gulf waters surrounding and including the Bolivar Roads inlet and the Galveston ship channel, indicate long-term site fidelity for a core group of bottlenose dolphins in lower Galveston Bay and the Galveston Ship Channel, yet provide evidence this region of Galveston Bay may predominantly consist of an open bottlenose dolphin population due to the high number of identified individuals (>1,000) but relatively low number of presumed year-round residents (approximately 200; Henningsen and Würsig 1991; Bräger et al. 1994; Fertl 1994b). Multi-year studies in Galveston Bay, excluding Bolivar Roads and the Galveston Ship Channel, indicate that a likely resident Galveston Bay population occurs throughout the upper and lower bay, utilizing deep channel habitats (primarily the Houston Ship Channel) in addition to open bay and nearshore habitats, with seasonal fluctuations in distribution (Fazioli et al. 2017; Fazioli and Mintzer 2020; Mintzer and Fazioli 2021; Mintzer et al. 2022). Photo-identification analyses were utilized to calculate site fidelity metrics for 442 distinct dolphins identified in upper Galveston Bay. These results were incorporated into a cluster analysis, indicating that 192 individuals (43%), demonstrated high site fidelity to upper Galveston Bay, either year-round or with a recurring seasonal pattern of residency (Mintzer et al. 2022); Remaining individuals were classified as transients (n=141, 32%) with only one sighting, or short-term users (n=109, 25%) likely to demonstrate site fidelity to Galveston Bay but lacking a specific preference for the upper portion, however, additional research is underway to evaluate how these individuals utilize other parts of the Bay (Fazioli et al. 2017; Mintzer and Fazioli 2021; Mintzer et al. 2022). Few spatially comprehensive studies have been conducted for the

Galveston Bay system and more research needs to be conducted to better determine the proportion and spatial range of enumerated individuals exhibiting site fidelity for this stock (Ronje et al. 2020).

Other Gulf of Mexico bay, sound, and estuary stocks show site fidelity (LaBrecque et al. 2015; Hayes et al. 2022), such as in Matagorda Bay and Espiritu Santo Bay in Texas (Lynn and Würsig 2002), San Luis Pass in Texas (Maze and Würsig 1999; Irwin and Würsig 2004), Barataria Bay in Louisiana (Miller 2003; Takeshita et al. 2021; Wells et al. 2017), Mississippi Sound (Hubard et al. 2004; Mackey 2010), St. Vincent Sound and Apalachicola Bay (Tyson et al. 2011), and Sarasota Bay (Wells 1986a; Wells 1986b; Wells 1991; Wells et al. 1996a; Wells et al. 1996b).

Lifetime Reproductive Potential

Taylor et al. (2007) reported the interbirth interval of common bottlenose dolphins as 3.80 years, though other studies have shown it to be closer to three years (Leatherwood 1977; Wells and Scott 1999; Connor et al. 2000; Wells 2003).

Generally, females reach sexual maturity before males, with females reaching sexual maturity at 5–13 years (Sergeant et al. 1973; Perrin and Reilly 1984; Wells et al. 1987; Mead and Potter 1990; Mann et al. 2000; Wells 2000; Wells and Scott 2018).

Females reproduce through most of their adult lives (Leatherwood 1977; Hohn et al. 1989; Wells and Scott 1999; Wells 2000; Wells 2003), but may produce fewer calves after they reach 30 years of age (Wells 2000). Sarasota Bay common bottlenose dolphins can give birth when they are as young as 6 years of age, and can continue until they are 48 years old (Wells and Scott 2018). Reproductive lifespan would therefore be up to 42 years based on long-term observation studies (Wells 2014; Wells and Scott 2018).

Longitudinal studies from Sarasota Bay, Florida have found that female common bottlenose dolphins can live up to 67 years (Wells and Scott 2018). Studies based on growth layer groups in teeth suggest female common bottlenose dolphins can live to 41 years along the Texas coast (Fernandez and Hohn 1998) and to 30 years along the Mississippi coast (Mattson et al. 2006).

Generation Length

Taylor et al. (2007) report generation length of 20.6-21.1 years.

Reproductive Plasticity

Calving may occur year round, but strong seasonal peaks are evident. Neonatal stranding rates indicate the calving peak for Northern Texas is in March (Urian et al. 1996; Fernandez and Hohn 1998; Piwetz et al. 2022).

Migration

Photo-ID studies indicate some coastal and inter-bay dolphin movements occur among adjacent stock areas (Henningsen and Würsig 1991; Maze and Würsig 1999; Ronje et al. 2020). Maze and Würsig (1999) identified three individuals that were observed in both the Galveston Bay and adjacent West Bay stock areas, with one of those individuals remaining in West Bay for up to 9 months after its initial sighting in

Galveston Bay. Ronje et al. (2020) photo-identified 40 individuals in the Galveston Bay stock area that were also observed in adjacent stock and nearshore coastal waters (West Bay n=15, Sabine Lake n=25). Given the high proportion of distinct individuals not re-sighted in Galveston Bay during previous studies (Henningsen and Würsig 1991; Bräger 1993; Ronje et al. 2020), rates of emigration and immigration may be significant.

Home Range

Previous studies of Galveston Bay common bottlenose dolphins found low recapture rates of photo-identified individuals in Gulf of Mexico waters, and distinct dorsal fin recapture rates were higher inside the bay than in the Gulf (e.g., Henningsen and Würsig 1991; Beier 2001). However, non-consecutive seasonal site fidelity has been documented for certain individuals outside of the bay boundaries for Galveston Bay (Ronje et al. 2020). This may suggest the bay boundaries and potentially nearshore coastal waters are included in a home range consistent with topographic features; however, more information needs to be collected to delineate the home range of Galveston Bay resident dolphins.

Stock Abundance

Ronje et al. (2020) estimated abundance in Galveston Bay at 841.9 individuals (95% CI = 693.5–990.4) in the winter and 1,131.5 individuals (95% CI = 846.3–1,416.7) in the summer based on 2016 surveys. Previously, Blaylock and Hoggard (1994) estimated abundance of this stock at 152 individuals (CV = 0.43).

Stock Abundance Trend

The data are insufficient to determine population trends for most of the Gulf of Mexico bay, sound, and estuary common bottlenose dolphin stocks (Hayes et al. 2022).

Cumulative Stressors

The Galveston Bay system presents a number of potential threats to bay inhabitants. Phillips and Rosel (2014) reviewed the cumulative stressors influencing the Galveston Bay system. Potential health threats range from contagious disease (e.g., *Brucella*), harmful algal blooms (HABs; Fire et al. 2011, 2020), and anthropogenic activities: mining, crude oil exploration, transport, and refinement, commercial fisheries, agricultural runoff in the form of pesticide and fertilizer, heavy metals and other chemical contaminants, marine debris, shipping and dredging, noise, tourism and boat traffic. Natural events may also pose threats: hypoxia resulting from HABs, adverse weather (e.g., hurricane storm surge), unusually high freshwater inflow (Fazioli and Mintzer 2020), habitat loss, and climate change (Phillips and Rosel 2014).

Unusual Mortality Events (UMEs) have been declared for common bottlenose dolphins in waters of Texas with morbillivirus the confirmed cause in 1994, a suspected cause in 1992 along with environmental conditions, and an undetermined cause in 2008 (Litz et al. 2014). This stock was also within the range of the 2007 UME in which 66 common bottlenose dolphins stranded in northeast Texas and west Louisiana due to an undetermined cause (Litz et al. 2014). A UME was declared in 1990 for the Gulf of Mexico from Florida to Texas, with more than 300 common bottlenose dolphins stranding due to an undetermined cause, although morbillivirus and cold temperatures may have contributed (Litz et al. 2014).

Distribution and Sightings

Sighting maps for this stock for January and July 2016 are presented in Ronje et al. (2020). Additional sighting data for August 2013 – November 2022 are available from the Galveston Bay Dolphin Research Program (GDRP) and sighting maps focused on western upper Galveston Bay, 2016-2019, are presented in Mintzer and Fazioli (2021) and Mintzer et al. (2022).

Further Reading

Hayes, S.A., E. Josephson, K. Maze-Foley, P.E. Rosel, and J. Wallace. 2022. U.S. Atlantic and Gulf of Mexico marine mammal stock assessments 2021. National Oceanic and Atmospheric Administration, National Marine Fisheries Service, Northeast Fisheries Science Center: Woods Hole, MA. NOAA Technical Memorandum NMFS-NE-288. 380 p. doi: 10.25923/6tt7-kc16

Wells, R.S. and M.D. Scott. 2018. Bottlenose Dolphin, *Tursiops truncatus*, Common Bottlenose Dolphin. In: Würsig, B., J.G.M. Thewissen, and K.M. Kovacs (Eds.), Encyclopedia of Marine Mammals (Third Edition). Academic Press, London, UK. pp. 118–125. doi: 10.1016/B978-0-12-804327-1.00072-8

Wells, R.S., A. Natoli, and G. Braulik. 2019. *Tursiops truncatus* (errata version published in 2019). The IUCN Red List of Threatened Species 2019: e.T22563A156932432. https://dx.doi.org/10.2305/IUCN.UK.2019-1.RLTS.T22563A156932432.en

References

Barros, N.B. and D.K. Odell. 1990. Food habits of bottlenose dolphins in the southeastern United States In: Leatherwood, S. and R.R. Reeves (Eds.), The Bottlenose Dolphin. Academic Press, San Diego, CA. pp. 309–328. doi: 10.1016/B978-0-12-440280-5.50020-2

Barros, N.B. and R.S. Wells. 1998. Prey and feeding patterns of resident bottlenose dolphins (*Tursiops truncatus*) in Sarasota Bay, Florida. Journal of Mammalogy 79(3):1045–1059. doi: 10.2307/1383114

Beier, A.G. 2001. Occurrence, distribution, and movement patterns of outer coastline bottlenose dolphins off Galveston Island, Texas. Texas A&M University. Thesis. 108 p.

Berens McCabe, E.J., D.P. Gannon, N.B. Barros, and R.S. Wells. 2010. Prey selection by resident common bottlenose dolphins (*Tursiops truncatus*) in Sarasota Bay, Florida. Marine Biology 157(5):931–942. doi: 10.1007/s00227-009-1371-2

Blaylock, R.A. and W. Hoggard. 1994. Preliminary estimates of bottlenose dolphin abundance in southern U.S. Atlantic and Gulf of Mexico continental shelf waters. National Oceanic and Atmospheric Administration, National Marine Fisheries Service, Southeast Fisheries Science Center: Miami, FL. NOAA Technical Memorandum NMFS-SEFSC-356. 10 p.

Bowen-Stevens, S.R., D.P. Gannon, R.A. Hazelkorn, G. Lovewell, K.M. Volker, S. Smith, M.C. Tumlin, and J. Litz. 2021. Diet of common bottlenose dolphins, *Tursiops truncatus*, that stranded in and near Barataria Bay, Louisiana, 2010–2012. Southeastern Naturalist 20(1). doi: 10.1656/058.020.0113

Bräger, S. 1993. Diurnal and seasonal behavior patterns of bottlenose dolphins (*Tursiops truncatus*). Marine Mammal Science 9:434–438. doi: 10.1111/j.1748-7692.1993.tb00477.x

Bräger, S., B. Würsig, A. Acevedo, and T. Henningsen. 1994. Association patterns of bottle-nosed dolphins (*Tursiops-truncatus*) in Galveston Bay, Texas. Journal of Mammalogy 75(2):431–437. doi: 10.2307/1382564

Connor, R.C., R.S. Wells, J. Mann, and A.J. Read. 2000. The bottlenose dolphin: Social relationships in a fission-fusion society. In: Mann, J., R. Connor, P. Tyack, and H. Whitehead (Eds.), Cetacean

Societies: Field Studies of Dolphins and Whales. University of Chicago Press, Chicago, IL. pp. 91–126.

Dunshea, G., N.B. Barros, E.J. Berens McCabe, N.J. Gales, M.A. Hindell, S.N. Jarman, and R.S. Wells. 2013. Stranded dolphin stomach contents represent the free-ranging population's diet. Biology Letters 9(3):20121036–20121036. doi: 10.1098/rsbl.2012.1036

Fazioli, K. and V. Mintzer. 2020. Short-term effects of Hurricane Harvey on bottlenose dolphins (*Tursiops truncatus*) in upper Galveston Bay, TX. Estuaries and Coasts 43(5):1013–1031. doi: 10.1007/s12237-020-00751-y

Fazioli, K., V. Mintzer, and G. Guillen. 2015. Bottlenose dolphin activity in a highly industrialized region of Galveston Bay, Texas. Society for Marine Mammalogy, 21st Biennial Conference on the Biology of Marine Mammals, San Francisco, CA,

Fazioli, K.L., V. Mintzer, and G. Guillen. 2017. Site fidelity of bottlenose dolphins (*Tursiops truncatus*) in a highly-industrialized estuary. 22nd Biennial Conference on the Biology of Marine Mammals, Halifax, Nova Scotia, Canada,

Fernandez, S. and A.A. Hohn. 1998. Age, growth, and calving season of bottlenose dolphins, *Tursiops truncatus*, off coastal Texas. Fishery Bulletin 96(2):357–365.

Fertl, D. 1994a. Occurrence patterns and behavior of bottlenose dolphins (*Tursiops truncatus*) in the Galveston ship channel, Texas. Texas Journal of Science 46(4):299–318.

Fertl, D. 1994b. Occurrence, movements, and behavior of bottlenose dolphins (*Tursiops truncatus*) in association with the shrimp fishery in Galveston Bay, Texas. Texas A&M University at Galveston. Thesis. 116 p.

Fire, S.E., T.A. Leighfield, G.A. Miller, S. Piwetz, E.R. Sabater, and H. Whitehead. 2020. Association between red tide exposure and detection of corresponding neurotoxins in bottlenose dolphins from Texas waters during 2007-2017. Marine Environmental Research 162:105191. doi: 10.1016/j.marenvres.2020.105191

Fire, S.E., Z. Wang, M. Byrd, H.R. Whitehead, J. Paternoster, and S.L. Morton. 2011. Co-occurrence of multiple classes of harmful algal toxins in bottlenose dolphins (*Tursiops truncatus*) stranding during an unusual mortality event in Texas, USA. Harmful Algae 10(3):330–336. doi: 10.1016/j.hal.2010.12.001

Gruber, J.A. 1981. Ecology of the Atlantic bottlenosed dolphin (*Tursiops truncatus*) in the Pass Cavallo area of Matagorda Bay, Texas. Texas A&M University. Thesis. 182 p.

Gunter, G. 1938. Seasonal variations in abundance of certain estuarine and marine fishes in Louisiana, with particular reference to life histories. Ecological Monographs 8:314–346.

Gunter, G. 1942. Contributions to the natural history of the bottle-nose dolphin *Tursiops truncatus* (Montague) on the Texas coast. Journal of Mammalogy 23:267–276.

Hayes, S.A., E. Josephson, K. Maze-Foley, P.E. Rosel, and J. Wallace. 2022. U.S. Atlantic and Gulf of Mexico marine mammal stock assessments 2021. National Oceanic and Atmospheric Administration, National Marine Fisheries Service, Northeast Fisheries Science Center: Woods Hole, MA. NOAA Technical Memorandum NMFS-NE-288. 380 p. doi: 10.25923/6tt7-kc16

Henderson, E.E. and B. Würsig. 2007. Behavior patterns of bottlenose dolphins in San Luis Pass, Texas. Gulf of Mexico Science 25(2):153–161. doi: 10.18785/goms.2502.06

Henningsen, T. and B. Würsig. 1991. Bottle-nosed dolphins in Galveston Bay, Texas: Numbers and activities. Proceedings of the Fifth Annual Conference of the European Cetacean Society, Sandefjord, Norway, 21–23 February 1991. pp. 36–38.

Hohn, A.A., M.D. Scott, R.S. Wells, J.C. Sweeney, and A.B. Irvine. 1989. Growth layers in teeth from known-age, free-ranging bottlenose dolphins. Marine Mammal Science 5(4):315–342. doi: 10.1111/j.1748-7692.1989.tb00346.x

Hubard, C.W., K. Maze-Foley, K.D. Mullin, and W.W. Schroeder. 2004. Seasonal abundance and site fidelity of bottlenose dolphins (*Tursiops truncatus*) in Mississippi Sound. Aquatic Mammals 30(2):299–310. doi: 10.1578/am.30.2.2004.299

Irwin, L.-J. and B. Würsig. 2004. A small resident community of bottlenose dolphins, *Tursiops truncatus*, in Texas: Monitoring recommendations. Gulf of Mexico Science 22(1):13–21. doi: 10.18785/goms.2201.02

Jones, S.C., III. 1988. Survey of the Atlantic bottlenose dolphin (*Tursiops truncatus*) population near Galveston, Texas. Texas A&M University. Thesis. 57 p.

LaBrecque, E., C. Curtice, J. Harrison, S.M. Van Parijs, and P.N. Halpin. 2015. 3. Biologically Important Areas for cetaceans within U.S. Waters – Gulf of Mexico Region. Aquatic Mammals 41(1):30–38. doi: 10.1578/am.41.1.2015.30

Leatherwood, S. 1977. Some preliminary impressions on the numbers and social behavior of free-swimming bottlenosed dolphin calves (*Tursiops truncatus*) in the northern Gulf of Mexico. In: Ridgway, S.H. and K. Bernirschke (Eds.), Breeding Dolphins; Present Status, Suggestions for the Future. Final report to the US Marine Mammal Commission, Contract MM6AC009, MMC-76/07, Washington, DC. pp. 143–167.

Litz, J.A., M.A. Baran, S.R. Bowen-Stevens, R.H. Carmichael, K.M. Colegrove, L.P. Garrison, S.E. Fire, E.M. Fougeres, R. Hardy, S. Holmes, W. Jones, B.E. Mase-Guthrie, D.K. Odell, P.E. Rosel, J.T. Saliki, D.K. Shannon, S.F. Shippee, S.M. Smith, E.M. Stratton, M.C. Tumlin, H.R. Whitehead, G.A. Worthy, and T.K. Rowles. 2014. Review of historical unusual mortality events (UMEs) in the Gulf of Mexico (1990–2009): providing context for the multi-year northern Gulf of Mexico cetacean UME declared in 2010. Diseases of Aquatic Organisms 112(2):161–175. doi: 10.3354/dao02807

Litz, J.A., E.I. Ronje, H.R. Whitehead, and L.P. Garrison. 2019. Updated abundance estimates for common bottlenose dolphins (*Tursiops truncatus*) inhabiting West Bay, Texas. Aquatic Conservation: Marine and Freshwater Ecosystems 29(11):1959–1970. doi: 10.1002/aqc.3195

Lynn, S.K. and B. Würsig. 2002. Summer movement patterns of bottlenose dolphins in a Texas bay. Gulf of Mexico Science 20(1):25–37. doi: 10.18785/goms.2001.03

Mackey, A.D. 2010. Site fidelity and association patterns of bottlenose dolphins (*Tursiops truncatus*) in the Mississippi Sound. University of Southern Mississippi. Thesis. 133 p.

Mann, J., R.C. Connor, L.M. Barre, and M.R. Heithaus. 2000. Female reproductive success in bottlenose dolphins (*Tursiops* sp.): life history, habitat, provisioning, and group-size effects. Behavioral Ecology 11(2):210–219. doi: 10.1093/beheco/11.2.210

Mattson, M.C., K.D. Mullin, G.W. Ingram, and W. Hoggard. 2006. Age structure and growth of the bottlenose dolphin (*Tursiops truncatus*) from strandings in the Mississippi sound region of the north-central Gulf of Mexico from 1986 to 2003. Marine Mammal Science 22(3):654–666. doi: 10.1111/j.1748-7692.2006.00057.x

Maze, K.S. and B. Würsig. 1999. Bottlenosed dolphins of San Luis Pass, Texas: Occurrence patterns, site-fidelity, and habitat use. Aquatic Mammals 25:91–104.

McDaniel, S. and G.J. Guillen. 2020. Foraging ecology of bottlenose dolphins (*Tursiops truncatus*) in Galveston Bay [Poster]. Galveston Bay Estuary Program, State of the Bay Symposium, Galveston, TX,

McHugh, M.B. 1989. Population numbers and feeding behavior of the Atlantic bottlenose dolphin (*Tursiops truncatus*) near Aransas Pass, Texas. University of Texas at Austin. Thesis. 96 p.

Mead, J.G. and C.W. Potter. 1990. Natural history of bottlenose dolphins along the central Atlantic coast of the United States. In: Leatherwood, S. and R.R. Reeves (Eds.), The Bottlenose Dolphin. Academic Press, San Diego, CA. pp. 165–195.

Miller, C.E. 2003. Abundance trends and environmental habitat usage patterns of bottlenose dolphins (*Tursiops truncatus*) in lower Barataria and Caminada bays, Louisiana. Louisiana State University and Agricultural & Mechanical College. Dissertation. 125 p.

Miller, W.G. 1992. An investigation of bottlenose dolphin *Tursiops truncatus* deaths in East Matagorda Bay, Texas, January 1990. Fishery Bulletin 90:791–797.

Mintzer, V.J. and K.L. Fazioli. 2021. Salinity and water temperature as predictors of bottlenose dolphin (*Tursiops truncatus*) encounter rates in upper Galveston Bay, Texas. Frontiers in Marine Science 8:754686. doi: 10.3389/fmars.2021.754686

Mintzer, V.J., A. Quackenbush, and K.L. Fazioli. 2022. Site fidelity of common bottlenose dolphins (*Tursiops truncatus*) in a highly industrialized area of the upper Galveston Bay estuary. Marine Mammal Science. doi: 10.1111/mms.12984

Moreno, M.P.T. 2005. Environmental predictors of bottlenose dolphin distribution and core feeding densities in Galveston Bay, Texas. Texas A&M University at Galveston. Dissertation. 88 p.

Moreno, P. and M. Mathews. 2018. Identifying foraging hotspots of bottlenose dolphins in a highly dynamic system: A method to enhance conservation in estuaries. Aquatic Mammals 44(6):694–710. doi: 10.1578/am.44.6.2018.694

Perrin, W.F. and S.B. Reilly. 1984. Reproductive parameters of dolphins and small whales of the family Delphinidae. In: Perrin, W.F., R.L. Brownell, Jr., and D.P. DeMaster (Eds.), Reproduction in Whales, Dolphins, and Porpoises. pp. 97–134.

Phillips, N.M. and P.E. Rosel. 2014. A method for prioritizing research on common bottlenose dolphin stocks through evaluating threats and data availability: Development and application to bay, sound and estuary stocks in Texas. National Oceanic and Atmospheric Administration, National Marine Fisheries Service, Southeast Fisheries Science Center: Lafayette, LA. NOAA Technical Memorandum NMFS-SEFSC-665. 146 p. doi: 10.7289/V5F769H8

Piwetz, S. 2019. Common bottlenose dolphin (*Tursiops truncatus*) behavior in an active narrow seaport. PLoS ONE 14(2):e0211971. doi: 10.1371/journal.pone.0211971

Piwetz, S., R. E.I., and H.R. Whitehead. 2022. Forty-year historical analysis of marine mammal strandings in Texas, from 1980 – 2019. Journal of Cetacean Research and Management 23(1):27–47. doi: 10.47536/jcrm.v23i1.345

Ronje, E., H. Whitehead, K. Barry, S. Piwetz, J. Struve, V. Lecours, L. Garrison, R.S. Wells, and K.D. Mullin. 2020. Abundance and occurrence of common bottlenose dolphins (*Tursiops truncatus*) in three estuaries of the northwestern Gulf of Mexico. Gulf and Caribbean Research 31:18–34. doi: 10.18785/gcr.3101.09

Ronje, E.I., H.R. Whitehead, S. Piwetz, and K.D. Mullin. 2018. Field summary for common bottlenose dolphin surveys on the Texas, Gulf of Mexico Coast, 2014–2016. National Oceanic and Atmospheric Administration, National Marine Fisheries Service, Southeast Fisheries Science Center, Protected Resources and Biodiversity Division: Miami, FL. NOAA Southeast Fisheries Science Center PRBD-2018-02. 31 p.

Rossman, S., P.H. Ostrom, M. Stolen, N.B. Barros, H. Gandhi, C.A. Stricker, and R.S. Wells. 2015. Individual specialization in the foraging habits of female bottlenose dolphins living in a trophically diverse and habitat rich estuary. Oecologia 178(2):415–425. doi: 10.1007/s00442-015-3241-6

Sergeant, D.E., D.K. Caldwell, and M.C. Caldwell. 1973. Age, growth, and maturity of bottlenosed dolphin (*Tursiops-truncatus*) from northeast Florida. Journal of the Fisheries Research Board of Canada 30(7):1009–1011. doi: 10.1139/f73-165

Shane, S. 1977. The population biology of the Atlantic bottlenose dolphin, *Tursiops truncatus*, in the Aransas Pass area of Texas. Texas A&M University. Thesis.

Shane, S.H. 1980. Occurrence, movements, and distribution of bottlenose dolphin, *Tursiops truncatus*, in southern Texas. Fishery Bulletin 78(3):593–601.

Shane, S.H. 1990. Behavior and ecology of the bottlenose dolphin at Sanibel Island, Florida. In: Leatherwood, S. and R.R. Reeves (Eds.), The Bottlenose Dolphin. Academic Press, San Diego, CA. pp. 245–265. doi: 10.1016/B978-0-12-440280-5.50016-0

Shane, S.H., R.S. Wells, and B. Würsig. 1986. Ecology, behavior and social organization of the bottlenose dolphin: A review. Marine Mammal Science 2:34–63. doi: 10.1111/j.1748-7692.1986.tb00026.x

Takeshita, R., B.C. Balmer, F. Messina, E.S. Zolman, L. Thomas, R.S. Wells, C.R. Smith, T.K. Rowles, and L.H. Schwacke. 2021. High site-fidelity in common bottlenose dolphins despite low salinity exposure and associated indicators of compromised health. PLoS ONE 16(9):e0258031. doi: 10.1371/journal.pone.0258031

Taylor, B.L., S.J. Chivers, J. Larese, and W.F. Perrin. 2007. Generation length and percent mature estimates for IUCN assessments of cetaceans. National Oceanic and Atmospheric Administration, National Marine Fisheries Service, Southwest Fisheries Science Center: La Jolla, CA. Administrative Report LJ-07-01. 24 p.

Torres, L.G. and A.J. Read. 2009. Where to catch a fish? The influence of foraging tactics on the ecology of bottlenose dolphins (*Tursiops truncatus*) in Florida Bay, Florida. Marine Mammal Science 25(4):797–815. doi: 10.1111/j.1748-7692.2009.00297.x

Tyson, R.B., S.M. Nowacek, and D.P. Nowacek. 2011. Community structure and abundance of bottlenose dolphins *Tursiops truncatus* in coastal waters of the northeast Gulf of Mexico. Marine Ecology Progress Series 438:253–265. doi: 10.3354/meps09292

Urian, K.W., D.A. Duffield, A.J. Read, R.S. Wells, and E.D. Shell. 1996. Seasonality of reproduction in bottlenose dolphins, *Tursiops truncatus*. Journal of Mammalogy 77(2):394–403. doi: 10.2307/1382814

Vollmer, N.L. and P.E. Rosel. 2013. A review of common bottlenose dolphins (*Tursiops truncatus truncatus*) in the Northern Gulf of Mexico: Population biology, potential threats, and management. Southeastern Naturalist 12(m6):1–43. doi: 10.1656/058.012.m601

Weller, D.W. 1998. Global and regional variation in the biology and behavior of bottlenose dolphins. Texas A&M University. Dissertation. 142 p.

Wells, R.S. 1986a. Population structure of bottlenose dolphins: Behavioral studies along the central west coast of Florida. Contract Report to National Marine Fisheries Service, Southeast Fisheries Center. Contract No. 45-WCNF-5-003BB. 58 p.

Wells, R.S. 1986b. Structural aspects of dolphin societies. University of California, Santa Cruz. Dissertation. 234 p.

Wells, R.S. 1991. The role of long-term study in understanding the social structure of a bottlenose dolphin community. In: Pryor, K. and K. Norris (Eds.), Dolphin Societies: Discoveries and Puzzles. University of California Press, Berkeley, CA. pp. 199–226.

Wells, R.S. 2000. Reproduction in wild bottlenose dolphins: Overview of patterns observed during a long-term study. In: Duffield, D. and T. Robeck (Eds.), Bottlenose Dolphin Reproduction Workshop. AZA Marine Mammal Taxon Advisory Group, Silver Spring, MD. pp. 57–74.

Wells, R.S. 2003. Dolphin social complexity: Lessons from long-term study and life history. In: de Waal, F.B.M. and P.L. Tyack (Eds.), Animal Social Complexity: Intelligence, Culture, and Individualized Societies Harvard University Press, Cambridge, MA. pp. 32–56.

Wells, R.S. 2014. Social structure and life history of bottlenose dolphins near Sarasota Bay, Florida: Insights from four decades and five generations. Primates and cetaceans. pp. 149–172. doi: 10.1007/978-4-431-54523-1_8

Wells, R.S., M.K. Bassos, K.W. Urian, W.J. Carr, and M.D. Scott. 1996. Low-level monitoring of bottlenose dolphins, *Tursiops truncatus*, in Charlotte Harbor, Florida: 1990-1994. National Oceanic and

Atmospheric Administration, National Marine Fisheries Service, Southeast Fisheries Science Center: Miami, FL. NOAA Technical Memorandum NMFS-SEFSC-384. 86 p.
Wells, R.S., L.H. Schwacke, T.K. Rowles, B.C. Balmer, E. Zolman, T. Speakman, F.I. Townsend, M.C. Tumlin, A. Barleycorn, and K.A. Wilkinson. 2017. Ranging patterns of common bottlenose dolphins *Tursiops truncatus* in Barataria Bay, Louisiana, following the Deepwater Horizon oil spill. Endangered Species Research 33:159–180. doi: 10.3354/esr00732
Wells, R.S. and M.D. Scott. 1999. Bottlenose dolphin *Tursiops truncatus* (Montagu, 1821). In: Ridgway, S.H. and R. Harrison (Eds.), Handbook of marine mammals. Volume 6: The second book of dolphins and the porpoises. Academic Press, San Diego, CA. pp. 137–182.
Wells, R.S. and M.D. Scott. 2018. Bottlenose dolphin, *Tursiops truncatus*, common bottlenose dolphin. In: Würsig, B., J.G.M. Thewissen, and K.M. Kovacs (Eds.), Encyclopedia of Marine Mammals (Third Edition). Academic Press, London, UK. pp. 118–125. doi: 10.1016/B978-0-12-804327-1.00072-8
Wells, R.S., M.D. Scott, and A.B. Irvine. 1987. The social structure of free-ranging bottlenose dolphins. In: Genoways, H.H. (Ed.), Current Mammalogy. Plenum Press, New York, NY. pp. 247–305.
Wells, R.S., K.W. Urian, A.J. Read, M.K. Bassos, W.J. Carr, and M.D. Scott. 1996. Low-level monitoring of bottlenose dolphins, *Tursiops truncatus*, in Tampa Bay, Florida: 1988-1993. National Oceanic and Atmospheric Administration, National Marine Fisheries Service, Southeast Fisheries Science Center: Miami, FL. NOAA Technical Memorandum NMFS-SEFSC-385. 36 p.
Wilson, R.M., R.B. Tyson, J.A. Nelson, B.C. Balmer, J.P. Chanton, and D.P. Nowacek. 2017. Niche differentiation and prey selectivity among common bottlenose dolphins (*Tursiops truncatus*) sighted in St. George Sound, Gulf of Mexico. Frontiers in Marine Science 4:235. doi: 10.3389/fmars.2017.00235

Common bottlenose dolphin − *Tursiops truncatus*

GoMx BSE/Laguna Madre Stock

Overall Vulnerability Rank = Very High

Biological Sensitivity = Very High

Climate Exposure = Very High

Data Quality = 93% of scores ≥ 2

Tursiops truncatus		Expert Scores	Data Quality	Expert Scores Plots (Portion by Category)
Sensitivity attributes	Prey/Diet Specificity	2.4	2.0	
	Habitat Specificity	3.0	2.0	
	Site Fidelity	3.6	2.0	
	Lifetime Reproductive Potential	1.5	2.2	
	Generation Length	2.9	2.2	
	Reproductive Plasticity	1.2	2.0	
	Migration	3.9	2.0	
	Home Range	3.6	1.8	
	Stock Abundance	3.2	2.0	
	Stock Abundance Trend	2.0	1.2	
	Cumulative Stressors	3.1	2.5	
	Sensitivity Score	Very High		
Exposure variables	Sea Surface Temperature (Change in mean)	4.0	2.5	
	Sea Surface Temperature (Change in variability)	1.0	2.5	
	Air Temperature (Change in mean)	4.0	2.5	
	Air Temperature (Change in variability)	1.0	2.5	
	Precipitation (Change in mean)	1.0	2.5	
	Precipitation (Change in variability)	1.0	2.5	
	Sea Surface Salinity (Change in mean)	2.1	2.5	
	Sea Surface Salinity (Change in variability)	1.0	2.5	
	Ocean pH (Change in mean)	4.0	2.5	
	Ocean pH (Change in variability)	1.0	2.5	
	Sea ice coverage (Change in mean)	1.0	2.5	
	Sea ice coverage (Change in variability)	1.0	2.5	
	Dissolved oxygen (Change in mean)	3.2	2.5	
	Dissolved oxygen (Change in variability)	1.0	2.5	
	Circulation	3.1	2.2	
	Sea level rise	4.0	2.5	
	Exposure Score	Very High		
Overall Vulnerability Rank		Very High		

- Low
- Moderate
- High
- Very High

Common bottlenose dolphin (GoMx BSE/Laguna Madre Stock)

Tursiops truncatus

CVA Results Summary

Overall Climate Vulnerability Rank: Very High (100% certainty from bootstrap analysis).

Climate Exposure: Very High. Four exposure factors scored greater than 3.5: Air Temperature (Standard anomaly) (4.00), Ocean pH (Standard anomaly) (4.00), Sea level rise (4.00), and Sea Surface Temperature (Standard anomaly) (4.00).

Biological Sensitivity: Very High. Three sensitivity attributes scored greater than 3.5: Migration (3.90), Site Fidelity (3.65), and Home Range (3.60).

Distributional Response: High

Abundance Response: High

Phenology Response: High

Data Quality: 93% of the data quality scores were 2 or greater, 82% of sensitivity attribute data quality scores were 2 or higher.

Background Information

Stock Description

This stock includes common bottlenose dolphins in the Laguna Madre in the western Gulf of Mexico along the Texas coast (Hayes et al. 2022).

Prey/Diet Specificity

Generally, common bottlenose dolphins consume a large variety of fish and/or squid (Barros and Odell 1990; Mead and Potter 1990; Wells and Scott 2018). Common bottlenose dolphins seem to show a consistent preference for sound-producing fishes (e.g., sciaenids, toadfish; Berens McCabe et al. 2010; Bowen-Stevens et al. 2021). In Sarasota Bay, FL, Rossman et al. (2015) found some individual common bottlenose dolphins to show diet specialization despite the broader study group showing a generalist diet. Specialization can be influenced by locally abundant prey species (Shane et al. 1986; Vollmer and Rosel 2013) or can result from resource partitioning amongst sympatric populations (e.g., Torres and Read 2009; Wilson et al. 2017).

Habitat Specificity

Common bottlenose dolphins are found in a variety of marine and estuarine habitats (Wells and Scott 2018).

Site Fidelity

Individual bottlenose dolphins identified in Laguna Madre photo-identification surveys show some degree of site fidelity, however, effort is low and spatially concentrated and further research is needed in order to determine the number of individuals exhibiting year-round site fidelity for this stock. One case study (Texas Marine Mammal Stranding Network 2019, *unpublished data*) reported a dolphin calf that became injured in 2008, disentangled in 2009, and is regularly sighted in the lower Laguna Madre as recently as 2019, now with her own calf. Similar case studies exist for individual dolphins that have become stranded, released, and tagged in the Laguna Madre between 2003 and 2019. Other Gulf of Mexico bay, sound, and estuary stocks show site fidelity (LaBrecque et al. 2015; Hayes et al. 2022), such as in Matagorda Bay and Espiritu Santo Bay in Texas (Lynn and Würsig 2002), San Luis Pass in Texas (Maze and Würsig 1999; Irwin and Würsig 2004), Barataria Bay in Louisiana (Miller 2003; Wells et al. 2017; Takeshita et al. 2021), Mississippi Sound (Hubard et al. 2004; Mackey 2010), St. Vincent Sound and Apalachicola Bay (Tyson et al. 2011), and Sarasota Bay (Wells 1986a; Wells 1986b; Wells 1991; Wells et al. 1996a; Wells et al. 1996b).

Lifetime Reproductive Potential

Taylor et al. (2007) reported the interbirth interval of common bottlenose dolphins as 3.80 years, though other studies have shown it to be closer to three years (Leatherwood 1977; Wells and Scott 1999; Connor et al. 2000; Wells 2003).

Generally, females reach sexual maturity before males, with females reaching sexual maturity at 5–13 years (Sergeant et al. 1973; Perrin and Reilly 1984; Wells et al. 1987; Mead and Potter 1990; Mann et al. 2000; Wells 2000; Wells and Scott 2018).

Females reproduce through most of their adult lives (Leatherwood 1977; Hohn et al. 1989; Wells and Scott 1999; Wells 2000; Wells 2003), but may produce fewer calves after they reach 30 years of age (Wells 2000). Sarasota Bay common bottlenose dolphins can give birth when they are as young as 6 years of age, and can continue until they are 48 years old (Wells and Scott 2018). Reproductive lifespan would therefore be up to 42 years based on long-term observation studies (Wells 2014; Wells and Scott 2018).

Longitudinal studies from Sarasota Bay, Florida have found that female common bottlenose dolphins can live up to 67 years (Wells and Scott 2018). Studies based on growth layer groups in teeth suggest female common bottlenose dolphins can live to 41 years along the Texas coast (Fernandez and Hohn 1998) and to 30 years along the Mississippi coast (Mattson et al. 2006).

Generation Length

Taylor et al. (2007) reported common bottlenose dolphin generation length of 20.6–21.1 years.

Reproductive Plasticity

Common bottlenose dolphin calving is generally diffusely seasonal, with one or more peaks, although births have been reported from all seasons (Vollmer and Rosel 2013; Wells and Scott 2018). Calf sightings peak in the spring and summer months in Florida (Wells et al. 1987), Mississippi/Louisiana (Miller et al. 2010; Miller et al. 2013), and Texas (McHugh 1989; Henderson et al. 2007).

Migration

The BSE stocks in the Gulf of Mexico are thought to primarily comprise stable resident communities. However, transient or seasonal movements of some individuals into and out of a BSE stock area are recorded in some areas (LaBrecque et al. 2015; Hayes et al. 2022). Molecular genetic data support that some BSE stocks are genetically distinct from one another, and furthermore are differentiated from adjacent coastal populations (e.g., Sellas et al. 2005; Rosel et al. 2017). For example, Toms (2019) found fine-scale genetic population structure among five inshore systems in the Florida Panhandle and variable migration rates among populations.

Year-round residency has been reported for common bottlenose dolphins (Hayes et al. 2022), such as in Florida (Irvine and Wells 1972; Irvine et al. 1981; Wells 1986a; Wells 1986b; Wells et al. 1987; Scott et al. 1990; Wells 1991; Wells et al. 1996a; Wells et al. 1996b; Wells et al. 1997; Wells 2003; Balmer et al. 2008; Urian et al. 2009; Tyson et al. 2011; Bassos-Hull et al. 2013; Balmer et al. 2018; Balmer et al. 2019; Toms 2019), Texas (Shane 1990; Bräger 1993; Bräger et al. 1994; Fertl 1994; Weller 1998; Maze and Würsig 1999; Lynn and Würsig 2002; Irwin and Würsig 2004; Shane 2004), and Louisiana/Mississippi (Hubard et al. 2004; Mullin et al. 2017; Takeshita et al. 2021).

Home Range

The home ranges of individuals within the Gulf of Mexico BSE stocks generally include the areas within the stock boundaries and brief excursions beyond the stock boundaries (Irvine et al. 1981; Wells 1986a; Wells 1986b; Wells 1991; Wells et al. 1996a; Wells et al. 1996b; Balmer et al. 2008; Wells 2014; Balmer et al. 2019), although some individuals show preference for small areas within the stock boundaries (Lynn and Würsig 2002; Urian et al. 2009; Mullin et al. 2017; Wells et al. 2017). Few studies have focused on the Laguna Madre and little is known about the site-fidelity of dolphins using the Laguna Madre. The results of several structured seasonal photo-ID surveys (Texas Marine Mammal Stranding Network 2018-19, *unpublished data*) and opportunistic observations during a dolphin disentanglement effort (Ronje et al. 2018) in the southern Laguna Madre indicate dolphin groups use the lower Laguna Madre area, primarily deeper channels and passes including the Brownsville Ship Channel, the Gulf Intracoastal Waterway, and the Brazos Santiago Pass. Dolphins travel through the Gulf inlet (Brazos-Santiago Pass) during flood tides and follow shrimp trawlers into waters well into the Brownsville Ship Channel to the Brownsville Fishing Harbor. Anecdotal reports suggest common bottlenose dolphins may travel between the Brazos-Santiago Pass and Port Mansfield, approximately 60 km north (Ronje et al. 2018). Additional photo-ID surveys reaching farther north into the Laguna Madre are needed to better understand dolphin movements in this area.

Stock Abundance

Blaylock and Boggard (1994) estimated abundance of this stock at 80 individuals (CV = 1.57). More recent estimates are unavailable (Hayes et al. 2022).

Stock Abundance Trend

Data are insufficient to complete an abundance trend analysis for this stock (Hayes et al. 2022).

Cumulative Stressors

Common bottlenose dolphins face a wide range of individual and cumulative natural and anthropogenic threats (see reviews by Vollmer and Rosel 2013; Wells and Scott 2018; and Wells et al. 2019). These include such threats as shark predation, harmful algal blooms, recreational and commercial fishing gear, vessel traffic, pollution, habitat alteration, and provisioning.

Philips and Rosel (2014) reviewed the cumulative stressors influencing the Laguna Madre system. Potential health threats range from contagious disease (e.g., *Brucella*), harmful algal blooms (HABs; Fire et al. 2011, 2020), and anthropogenic activities: mining, crude oil exploration, transport, and refinement, commercial fisheries, agricultural runoff in the form of pesticide and fertilizer, heavy metals and other chemical contaminants, marine debris, shipping and dredging, noise, tourism and boat traffic. Natural events may also pose threats: hypoxia resulting from HABs, adverse weather (e.g., hurricane storm surge), habitat loss, and climate change (Philips and Rosel 2014).

Unusual Mortality Events (UMEs) have been declared for common bottlenose dolphins in waters of Texas with morbillivirus the confirmed cause in 1994, a suspected cause in 1992 along with environmental conditions, and an undetermined cause in 2008 (Litz et al. 2014). A UME was declared in 1990 for the Gulf of Mexico from Florida to Texas, with more than 300 common bottlenose dolphins stranding due to an undetermined cause, although morbillivirus and cold temperatures may have contributed (Litz et al. 2014).

Distribution and Sightings

Sighting maps for this stock from July 2016 are presented in Ronje et al. (2018). Additional sighting data from December 2018 and August 2019 is available from the Texas Marine Mammal Stranding Network (TMMSN).

Further Reading

Hayes, S.A., E. Josephson, K. Maze-Foley, P.E. Rosel, and J. Wallace. 2022. U.S. Atlantic and Gulf of Mexico marine mammal stock assessments 2021. National Oceanic and Atmospheric Administration, National Marine Fisheries Service, Northeast Fisheries Science Center: Woods Hole, MA. NOAA Technical Memorandum NMFS-NE-288. 380 p. doi: 10.25923/6tt7-kc16

Wells, R.S. and M.D. Scott. 2018. Bottlenose Dolphin, *Tursiops truncatus*, Common Bottlenose Dolphin. In: Würsig, B., J.G.M. Thewissen, and K.M. Kovacs (Eds.), Encyclopedia of Marine Mammals (Third Edition). Academic Press, London, UK. pp. 118–125. doi: 10.1016/B978-0-12-804327-1.00072-8

Wells, R.S., A. Natoli, and G. Braulik. 2019. *Tursiops truncatus* (errata version published in 2019). The IUCN Red List of Threatened Species 2019: e.T22563A156932432. https://dx.doi.org/10.2305/IUCN.UK.2019-1.RLTS.T22563A156932432.en

References

Balmer, B., T. McDonald, F. Hornsby, J. Adams, J. Allen, A. Barleycorn, and L. Schwacke. 2018. Long-term trends in a northern Gulf of Mexico common bottlenose dolphin (*Tursiops truncatus*) population in the wake of the Deepwater Horizon oil spill. Journal of Cetacean Research and Management 18:1–9. doi: 10.47536/jcrm.v18i1.409

Balmer, B., S. Watwood, B. Quigley, T. Speakman, K. Barry, K. Mullin, P. Rosel, C. Sinclair, E. Zolman, and L. Schwacke. 2019. Common bottlenose dolphin (*Tursiops truncatus*) abundance and distribution patterns in St. Andrew Bay, Florida, USA. Aquatic Conservation: Marine and Freshwater Ecosystems 29(3):486–498. doi: 10.1002/aqc.3001

Balmer, B.C., R.S. Wells, S.M. Nowacek, D.P. Nowacek, L.H. Schwacke, W.A. McLellan, F.S. Scharf, T.K. Rowles, L.J. Hansen, T.R. Spradlin, and D.A. Pabst. 2008. Seasonal abundance and distribution patterns of common bottlenose dolphins (*Tursiops truncatus*) near St. Joseph Bay, Florida, USA. Journal of Cetacean Research and Management 10(2):157–167.

Barros, N.B. and D.K. Odell. 1990. Food habits of bottlenose dolphins in the southeastern United States In: Leatherwood, S. and R.R. Reeves (Eds.), The Bottlenose Dolphin. Academic Press, San Diego, CA. pp. 309–328. doi: 10.1016/B978-0-12-440280-5.50020-2

Bassos-Hull, K., R.M. Perrtree, C.C. Shepard, S. Schilling, A.A. Barleycorn, J.B. Allen, B.C. Balmer, W.E. Pine, and R.S. Wells. 2013. Long-term site fidelity and seasonal abundance estimates of common bottlenose dolphins (*Tursiops truncatus*) along the southwest coast of Florida and responses to natural perturbations. Journal of Cetacean Research and Management 13(1):19–30. doi: 10.47536/jcrm.v13i1.551

Berens McCabe, E.J., D.P. Gannon, N.B. Barros, and R.S. Wells. 2010. Prey selection by resident common bottlenose dolphins (*Tursiops truncatus*) in Sarasota Bay, Florida. Marine Biology 157(5):931–942. doi: 10.1007/s00227-009-1371-2

Blaylock, R.A. and W. Hoggard. 1994. Preliminary estimates of bottlenose dolphin abundance in southern U.S. Atlantic and Gulf of Mexico continental shelf waters. National Oceanic and Atmospheric Administration, National Marine Fisheries Service, Southeast Fisheries Science Center: Miami, FL. NOAA Technical Memorandum NMFS-SEFSC-356. 10 p.

Bowen-Stevens, S.R., D.P. Gannon, R.A. Hazelkorn, G. Lovewell, K.M. Volker, S. Smith, M.C. Tumlin, and J. Litz. 2021. Diet of common bottlenose dolphins, *Tursiops truncatus*, that stranded in and near Barataria Bay, Louisiana, 2010–2012. Southeastern Naturalist 20(1). doi: 10.1656/058.020.0113

Bräger, S. 1993. Diurnal and seasonal behavior patterns of bottlenose dolphins (*Tursiops truncatus*). Marine Mammal Science 9:434–438. doi: 10.1111/j.1748-7692.1993.tb00477.x

Bräger, S., B. Würsig, A. Acevedo, and T. Henningsen. 1994. Association patterns of bottle-nosed dolphins (*Tursiops-truncatus*) in Galveston Bay, Texas. Journal of Mammalogy 75(2):431–437. doi: 10.2307/1382564

Connor, R.C., R.S. Wells, J. Mann, and A.J. Read. 2000. The bottlenose dolphin: Social relationships in a fission-fusion society. In: Mann, J., R. Connor, P. Tyack, and H. Whitehead (Eds.), Cetacean Societies: Field Studies of Dolphins and Whales. University of Chicago Press, Chicago, IL. pp. 91–126.

Fernandez, S. and A.A. Hohn. 1998. Age, growth, and calving season of bottlenose dolphins, *Tursiops truncatus*, off coastal Texas. Fishery Bulletin 96(2):357–365.

Fertl, D. 1994. Occurrence patterns and behavior of bottlenose dolphins (*Tursiops truncatus*) in the Galveston ship channel, Texas. Texas Journal of Science 46(4):299–318.

Fire, S.E., T.A. Leighfield, G.A. Miller, S. Piwetz, E.R. Sabater, and H. Whitehead. 2020. Association between red tide exposure and detection of corresponding neurotoxins in bottlenose dolphins from Texas waters during 2007-2017. Marine Environmental Research 162:105191. doi: 10.1016/j.marenvres.2020.105191

Fire, S.E., Z. Wang, M. Byrd, H.R. Whitehead, J. Paternoster, and S.L. Morton. 2011. Co-occurrence of multiple classes of harmful algal toxins in bottlenose dolphins (*Tursiops truncatus*) stranding during an unusual mortality event in Texas, USA. Harmful Algae 10(3):330–336. doi: 10.1016/j.hal.2010.12.001

Hayes, S.A., E. Josephson, K. Maze-Foley, P.E. Rosel, and J. Wallace. 2022. U.S. Atlantic and Gulf of Mexico marine mammal stock assessments 2021. National Oceanic and Atmospheric Administration, National Marine Fisheries Service, Northeast Fisheries Science Center: Woods Hole, MA. NOAA Technical Memorandum NMFS-NE-288. 380 p. doi: 10.25923/6tt7-kc16

Henderson, E.E. and B. Würsig. 2007. Behavior patterns of bottlenose dolphins in San Luis Pass, Texas. Gulf of Mexico Science 25(2):153–161. doi: 10.18785/goms.2502.06

Hohn, A.A., M.D. Scott, R.S. Wells, J.C. Sweeney, and A.B. Irvine. 1989. Growth layers in teeth from known-age, free-ranging bottlenose dolphins. Marine Mammal Science 5(4):315–342. doi: 10.1111/j.1748-7692.1989.tb00346.x

Hubard, C.W., K. Maze-Foley, K.D. Mullin, and W.W. Schroeder. 2004. Seasonal abundance and site fidelity of bottlenose dolphins (*Tursiops truncatus*) in Mississippi Sound. Aquatic Mammals 30(2):299–310. doi: 10.1578/am.30.2.2004.299

Irvine, A.B., M.D. Scott, R.S. Wells, and J.H. Kaufmann. 1981. Movements and activities of the Atlantic bottlenose dolphin, *Tursiops truncatus*, near Sarasota, Florida. Fishery Bulletin 79(4):671–688.

Irvine, B. and R.S. Wells. 1972. Results of attempts to tag Atlantic bottlenosed dolphins (*Tursiops truncatus*). Cetology 13:1–5.

Irwin, L.-J. and B. Würsig. 2004. A small resident community of bottlenose dolphins, *Tursiops truncatus*, in Texas: Monitoring recommendations. Gulf of Mexico Science 22(1):13–21. doi: 10.18785/goms.2201.02

LaBrecque, E., C. Curtice, J. Harrison, S.M. Van Parijs, and P.N. Halpin. 2015. 3. Biologically Important Areas for cetaceans within U.S. Waters – Gulf of Mexico Region. Aquatic Mammals 41(1):30–38. doi: 10.1578/am.41.1.2015.30

Leatherwood, S. 1977. Some preliminary impressions on the numbers and social behavior of free-swimming bottlenosed dolphin calves (*Tursiops truncatus*) in the northern Gulf of Mexico. In: Ridgway, S.H. and K. Bernirschke (Eds.), Breeding Dolphins; Present Status, Suggestions for the Future. Final report to the US Marine Mammal Commission, Contract MM6AC009, MMC-76/07, Washington, DC. pp. 143–167.

Litz, J.A., M.A. Baran, S.R. Bowen-Stevens, R.H. Carmichael, K.M. Colegrove, L.P. Garrison, S.E. Fire, E.M. Fougeres, R. Hardy, S. Holmes, W. Jones, B.E. Mase-Guthrie, D.K. Odell, P.E. Rosel, J.T. Saliki, D.K. Shannon, S.F. Shippee, S.M. Smith, E.M. Stratton, M.C. Tumlin, H.R. Whitehead, G.A. Worthy, and T.K. Rowles. 2014. Review of historical unusual mortality events (UMEs) in the Gulf of Mexico (1990–2009): providing context for the multi-year northern Gulf of Mexico cetacean UME declared in 2010. Diseases of Aquatic Organisms 112(2):161–175. doi: 10.3354/dao02807

Lynn, S.K. and B. Würsig. 2002. Summer movement patterns of bottlenose dolphins in a Texas bay. Gulf of Mexico Science 20(1):25–37. doi: 10.18785/goms.2001.03

Mackey, A.D. 2010. Site fidelity and association patterns of bottlenose dolphins (*Tursiops truncatus*) in the Mississippi Sound. University of Southern Mississippi. Thesis. 133 p.

Mann, J., R.C. Connor, L.M. Barre, and M.R. Heithaus. 2000. Female reproductive success in bottlenose dolphins (*Tursiops* sp.): life history, habitat, provisioning, and group-size effects. Behavioral Ecology 11(2):210–219. doi: 10.1093/beheco/11.2.210

Mattson, M.C., K.D. Mullin, G.W. Ingram, and W. Hoggard. 2006. Age structure and growth of the bottlenose dolphin (*Tursiops truncatus*) from strandings in the Mississippi sound region of the north-central Gulf of Mexico from 1986 to 2003. Marine Mammal Science 22(3):654–666. doi: 10.1111/j.1748-7692.2006.00057.x

Maze, K.S. and B. Würsig. 1999. Bottlenosed dolphins of San Luis Pass, Texas: Occurrence patterns, site-fidelity, and habitat use. Aquatic Mammals 25:91–104.

McHugh, M.B. 1989. Population numbers and feeding behavior of the Atlantic bottlenose dolphin (*Tursiops truncatus*) near Aransas Pass, Texas. University of Texas at Austin. Thesis. 96 p.

Mead, J.G. and C.W. Potter. 1990. Natural history of bottlenose dolphins along the central Atlantic coast of the United States. In: Leatherwood, S. and R.R. Reeves (Eds.), The Bottlenose Dolphin. Academic Press, San Diego, CA. pp. 165–195.

Miller, C.E. 2003. Abundance trends and environmental habitat usage patterns of bottlenose dolphins (*Tursiops truncatus*) in lower Barataria and Caminada bays, Louisiana. Louisiana State University and Agricultural & Mechanical College. Dissertation. 125 p.

Miller, L.J., A.D. Mackey, T. Hoffland, M. Solangi, and S.A. Kuczaj. 2010. Potential effects of a major hurricane on Atlantic bottlenose dolphin (*Tursiops truncatus*) reproduction in the Mississippi Sound. Marine Mammal Science 26(3):707–715. doi: 10.1111/j.1748-7692.2010.00371.x

Miller, L.J., A.D. Mackey, M. Solangi, and S.A. Kuczaj. 2013. Population abundance and habitat utilization of bottlenose dolphins in the Mississippi Sound. Aquatic Conservation: Marine and Freshwater Ecosystems 23(1):145–151. doi: 10.1002/aqc.2278

Mullin, K.D., T. McDonald, R.S. Wells, B.C. Balmer, T. Speakman, C. Sinclair, E.S. Zolman, F. Hornsby, S.M. McBride, K.A. Wilkinson, and L.H. Schwacke. 2017. Density, abundance, survival, and ranging patterns of common bottlenose dolphins (*Tursiops truncatus*) in Mississippi Sound following the Deepwater Horizon oil spill. PLoS ONE 12(10):e0186265. doi: 10.1371/journal.pone.0186265

Perrin, W.F. and S.B. Reilly. 1984. Reproductive parameters of dolphins and small whales of the family Delphinidae. In: Perrin, W.F., R.L. Brownell, Jr., and D.P. DeMaster (Eds.), Reproduction in Whales, Dolphins, and Porpoises. pp. 97–134.

Phillips, N.M. and P.E. Rosel. 2014. A method for prioritizing research on common bottlenose dolphin stocks through evaluating threats and data availability: Development and application to bay, sound and estuary stocks in Texas. National Oceanic and Atmospheric Administration, National Marine Fisheries Service, Southeast Fisheries Science Center: Lafayette, LA. NOAA Technical Memorandum NMFS-SEFSC-665. 146 p. doi: 10.7289/V5F769H8

Ronje, E.I., H.R. Whitehead, S. Piwetz, and K.D. Mullin. 2018. Field summary for common bottlenose dolphin surveys on the Texas, Gulf of Mexico Coast, 2014–2016. National Oceanic and Atmospheric Administration, National Marine Fisheries Service, Southeast Fisheries Science Center, Protected Resources and Biodiversity Division: Miami, FL. NOAA Southeast Fisheries Science Center PRBD-2018-02. 31 p.

Rosel, P.E., L.A. Wilcox, C. Sinclair, T.R. Speakman, M.C. Tumlin, J.A. Litz, and E.S. Zolman. 2017. Genetic assignment to stock of stranded common bottlenose dolphins in southeastern Louisiana after the Deepwater Horizon oil spill. Endangered Species Research 33:221–234. doi: 10.3354/esr00780

Rossman, S., P.H. Ostrom, M. Stolen, N.B. Barros, H. Gandhi, C.A. Stricker, and R.S. Wells. 2015. Individual specialization in the foraging habits of female bottlenose dolphins living in a trophically diverse and habitat rich estuary. Oecologia 178(2):415–425. doi: 10.1007/s00442-015-3241-6

Scott, M.D. and S.J. Chivers. 1990. Distribution and herd structure of bottlenose dolphins in the eastern tropical Pacific Ocean. In: Leatherwood, S. and R.R. Reeves (Eds.), The Bottlenose Dolphin. Academic Press, San Diego, CA. pp. 387–402.

Sellas, A.B., R.S. Wells, and P.E. Rosel. 2005. Mitochondrial and nuclear DNA analyses reveal fine scale geographic structure in bottlenose dolphins (*Tursiops truncatus*) in the Gulf of Mexico. Conservation Genetics 6(5):715–728. doi: 10.1007/s10592-005-9031-7

Sergeant, D.E., D.K. Caldwell, and M.C. Caldwell. 1973. Age, growth, and maturity of bottlenosed dolphin (*Tursiops-truncatus*) from northeast Florida. Journal of the Fisheries Research Board of Canada 30(7):1009–1011. doi: 10.1139/f73-165

Shane, S.H. 1990. Behavior and ecology of the bottlenose dolphin at Sanibel Island, Florida. In: Leatherwood, S. and R.R. Reeves (Eds.), The Bottlenose Dolphin. Academic Press, San Diego, CA. pp. 245–265. doi: 10.1016/B978-0-12-440280-5.50016-0

Shane, S.H. 2004. Residence patterns, group characteristics, and association patterns of bottlenose dolphins near Sanibel Island, Florida. Gulf of Mexico Science 22(1):1–12. doi: 10.18785/goms.2201.01

Shane, S.H., R.S. Wells, and B. Würsig. 1986. Ecology, behavior and social organization of the bottlenose dolphin: A review. Marine Mammal Science 2:34–63. doi: 10.1111/j.1748-7692.1986.tb00026.x

Takeshita, R., B.C. Balmer, F. Messina, E.S. Zolman, L. Thomas, R.S. Wells, C.R. Smith, T.K. Rowles, and L.H. Schwacke. 2021. High site-fidelity in common bottlenose dolphins despite low salinity exposure and associated indicators of compromised health. PLoS ONE 16(9):e0258031. doi: 10.1371/journal.pone.0258031

Taylor, B.L., S.J. Chivers, J. Larese, and W.F. Perrin. 2007. Generation length and percent mature estimates for IUCN assessments of cetaceans. National Oceanic and Atmospheric Administration, National Marine Fisheries Service, Southwest Fisheries Science Center: La Jolla, CA. Administrative Report LJ-07-01. 24 p.

Toms, C. 2019. Filling the gaps: Common bottlenose dolphin (*Tursiops truncatus*) population dynamics, structure, and connectivity within Florida panhandle bays, sounds, and estuaries. University of Central Florida. Dissertation. 373 p.

Torres, L.G. and A.J. Read. 2009. Where to catch a fish? The influence of foraging tactics on the ecology of bottlenose dolphins (*Tursiops truncatus*) in Florida Bay, Florida. Marine Mammal Science 25(4):797–815. doi: 10.1111/j.1748-7692.2009.00297.x

Tyson, R.B., S.M. Nowacek, and D.P. Nowacek. 2011. Community structure and abundance of bottlenose dolphins *Tursiops truncatus* in coastal waters of the northeast Gulf of Mexico. Marine Ecology Progress Series 438:253–265. doi: 10.3354/meps09292

Urian, K.W., S. Hofmann, R.S. Wells, and A.J. Read. 2009. Fine-scale population structure of bottlenose dolphins (*Tursiops truncatus*) in Tampa Bay, Florida. Marine Mammal Science 25(3):619–638. doi: 10.1111/j.1748-7692.2009.00284.x

Vollmer, N.L. and P.E. Rosel. 2013. A review of common bottlenose dolphins (*Tursiops truncatus truncatus*) in the Northern Gulf of Mexico: Population biology, potential threats, and management. Southeastern Naturalist 12(m6):1–43. doi: 10.1656/058.012.m601

Weller, D.W. 1998. Global and regional variation in the biology and behavior of bottlenose dolphins. Texas A&M University. Dissertation. 142 p.

Wells, R.S. 1986a. Population structure of bottlenose dolphins: Behavioral studies along the central west coast of Florida. Contract Report to National Marine Fisheries Service, Southeast Fisheries Center. Contract No. 45-WCNF-5-003BB. 58 p.

Wells, R.S. 1986b. Structural aspects of dolphin societies. University of California, Santa Cruz. Dissertation. 234 p.

Wells, R.S. 1991. The role of long-term study in understanding the social structure of a bottlenose dolphin community. In: Pryor, K. and K. Norris (Eds.), Dolphin Societies: Discoveries and Puzzles. University of California Press, Berkeley, CA. pp. 199–226.

Wells, R.S. 2000. Reproduction in wild bottlenose dolphins: Overview of patterns observed during a long-term study. In: Duffield, D. and T. Robeck (Eds.), Bottlenose Dolphin Reproduction Workshop. AZA Marine Mammal Taxon Advisory Group, Silver Spring, MD. pp. 57–74.

Wells, R.S. 2003. Dolphin social complexity: Lessons from long-term study and life history. In: de Waal, F.B.M. and P.L. Tyack (Eds.), Animal Social Complexity: Intelligence, Culture, and Individualized Societies Harvard University Press, Cambridge, MA. pp. 32–56.

Wells, R.S. 2014. Social structure and life history of bottlenose dolphins near Sarasota Bay, Florida: Insights from four decades and five generations. Primates and cetaceans. pp. 149–172. doi: 10.1007/978-4-431-54523-1_8

Wells, R.S., M.K. Bassos, K.W. Urian, W.J. Carr, and M.D. Scott. 1996. Low-level monitoring of bottlenose dolphins, *Tursiops truncatus*, in Charlotte Harbor, Florida: 1990-1994. National Oceanic and Atmospheric Administration, National Marine Fisheries Service, Southeast Fisheries Science Center: Miami, FL. NOAA Technical Memorandum NMFS-SEFSC-384. 86 p.

Wells, R.S., M.K. Bassos, K.W. Urian, S.H. Shane, E.C.G. Owen, C.F. Weiss, W.J. Carr, and M.D. Scott. 1997. Low-level monitoring of bottlenose dolphins, *Tursiops truncatus*, in Pine Island Sound, Florida: 1996. National Oceanic and Atmospheric Administration, National Marine Fisheries Service, Southeast Fisheries Science Center: Miami, FL. Southeast Fisheries Center Contribution No. 40-WCNF601958. 86 p.

Wells, R.S., A. Natoli, and G. Braulik. 2019. *Tursiops truncatus* (errata version published in 2019). *The IUCN Red List of Threatened Species* 2019: e.T22563A156932432. https://dx.doi.org/10.2305/IUCN.UK.2019-1.RLTS.T22563A156932432.en

Wells, R.S., L.H. Schwacke, T.K. Rowles, B.C. Balmer, E. Zolman, T. Speakman, F.I. Townsend, M.C. Tumlin, A. Barleycorn, and K.A. Wilkinson. 2017. Ranging patterns of common bottlenose dolphins *Tursiops truncatus* in Barataria Bay, Louisiana, following the Deepwater Horizon oil spill. Endangered Species Research 33:159–180. doi: 10.3354/esr00732

Wells, R.S. and M.D. Scott. 1999. Bottlenose dolphin *Tursiops truncatus* (Montagu, 1821). In: Ridgway, S.H. and R. Harrison (Eds.), Handbook of marine mammals. Volume 6: The second book of dolphins and the porpoises. Academic Press, San Diego, CA. pp. 137–182.

Wells, R.S. and M.D. Scott. 2018. Bottlenose dolphin, *Tursiops truncatus*, common bottlenose dolphin. In: Würsig, B., J.G.M. Thewissen, and K.M. Kovacs (Eds.), Encyclopedia of Marine Mammals (Third Edition). Academic Press, London, UK. pp. 118–125. doi: 10.1016/B978-0-12-804327-1.00072-8

Wells, R.S., M.D. Scott, and A.B. Irvine. 1987. The social structure of free-ranging bottlenose dolphins. In: Genoways, H.H. (Ed.), Current Mammalogy. Plenum Press, New York, NY. pp. 247–305.

Wells, R.S., K.W. Urian, A.J. Read, M.K. Bassos, W.J. Carr, and M.D. Scott. 1996. Low-level monitoring of bottlenose dolphins, *Tursiops truncatus*, in Tampa Bay, Florida: 1988-1993. National Oceanic and Atmospheric Administration, National Marine Fisheries Service, Southeast Fisheries Science Center: Miami, FL. NOAA Technical Memorandum NMFS-SEFSC-385. 36 p.

Wilson, R.M., R.B. Tyson, J.A. Nelson, B.C. Balmer, J.P. Chanton, and D.P. Nowacek. 2017. Niche differentiation and prey selectivity among common bottlenose dolphins (*Tursiops truncatus*) sighted in St. George Sound, Gulf of Mexico. Frontiers in Marine Science 4:235. doi: 10.3389/fmars.2017.00235

Common bottlenose dolphin − *Tursiops truncatus*

GoMx BSE/Matagorda Bay, Tres Palacios Bay, Lavaca Bay Stock

Overall Vulnerability Rank = Very High

Biological Sensitivity = High

Climate Exposure = Very High

Data Quality = 96% of scores ≥ 2

	Tursiops truncatus	Expert Scores	Data Quality	Expert Scores Plots (Portion by Category)
Sensitivity attributes	Prey/Diet Specificity	2.4	2.2	
	Habitat Specificity	2.8	2.2	
	Site Fidelity	3.4	2.5	
	Lifetime Reproductive Potential	1.5	2.2	
	Generation Length	2.9	2.2	
	Reproductive Plasticity	1.2	2.0	
	Migration	3.8	2.2	
	Home Range	3.5	2.5	
	Stock Abundance	3.1	2.0	
	Stock Abundance Trend	2.0	1.8	
	Cumulative Stressors	2.8	2.8	
	Sensitivity Score	High		
Exposure variables	Sea Surface Temperature (Change in mean)	4.0	2.8	
	Sea Surface Temperature (Change in variability)	1.0	2.8	
	Air Temperature (Change in mean)	4.0	2.8	
	Air Temperature (Change in variability)	1.0	2.8	
	Precipitation (Change in mean)	1.0	2.8	
	Precipitation (Change in variability)	1.0	2.8	
	Sea Surface Salinity (Change in mean)	2.0	2.8	
	Sea Surface Salinity (Change in variability)	1.0	2.8	
	Ocean pH (Change in mean)	4.0	2.8	
	Ocean pH (Change in variability)	1.0	2.8	
	Sea ice coverage (Change in mean)	1.0	2.8	
	Sea ice coverage (Change in variability)	1.0	2.8	
	Dissolved oxygen (Change in mean)	4.0	2.8	
	Dissolved oxygen (Change in variability)	1.0	2.8	
	Circulation	3.8	2.5	
	Sea level rise	4.0	2.8	
	Exposure Score	Very High		
Overall Vulnerability Rank		Very High		

Low
Moderate
High
Very High

Common bottlenose dolphin (GoMx BSE/Matagorda Bay, Tres Palacios Bay, Lavaca Bay Stock)

Tursiops truncatus

CVA Results Summary

Overall Climate Vulnerability Rank: Very High (100% certainty from bootstrap analysis).

Climate Exposure: Very High. Six exposure factors scored greater than 3.5: Air Temperature (Standard anomaly) (4.00), Dissolved oxygen (Standard anomaly) (4.00), Ocean pH (Standard anomaly) (4.00), Sea level rise (4.00), Sea Surface Temperature (Standard anomaly) (4.00), and Circulation (3.85).

Biological Sensitivity: High. Four sensitivity attributes scored greater than 3.0: Migration (3.85), Home Range (3.55), Site Fidelity (3.45), and Species Abundance (3.10).

Distributional Response: High

Abundance Response: High

Phenology Response: High

Data Quality: 96% of the data quality scores were 2 or greater, 91% of sensitivity attribute data quality scores were 2 or higher.

Background Information

Stock Description

This stock includes common bottlenose dolphins in Matagorda Bay, Tres Palacios Bay, Lavaca Bay, and smaller bays associated with Matagorda Bay in the western Gulf of Mexico along the Texas coast (Hayes et al. 2022).

Prey/Diet Specificity

Generally, common bottlenose dolphins consume a large variety of fish and/or squid (Wells and Scott 2018). Common bottlenose dolphins seem to show a consistent preference for noise-producing fishes (e.g., sciaenids, toadfish; Berens McCabe et al. 2010; Bowen-Stevens et al. 2021). In Sarasota Bay, FL, Rossman et al. (2015) found some individual common bottlenose dolphins to show diet specialization despite the broader study group showing a generalist diet. Specialization can be influenced by locally abundant prey species (Shane et al. 1986; Vollmer and Rosel 2013) or can result from resource partitioning amongst sympatric populations (e.g., Torres and Read 2009; Wilson et al. 2017).

Habitat Specificity

Common bottlenose dolphins are found in a variety of marine and estuarine habitats (Wells and Scott 2018).

Site Fidelity

Lynn and Würsig (2002) attached radio transmitters to dolphins near Port O'Connor, TX and recorded telemetry data that indicated dolphins have strong site fidelity to Matagorda Bay and Espiritu Santo Bay. Other Gulf of Mexico bay, sound, and estuary (BSE) stocks also show site fidelity (LaBrecque et al. 2015; Hayes et al. 2022), such as in San Luis Pass in Texas (Maze and Würsig 1999; Lynn and Würsig 2002; Irwin and Würsig 2004); Barataria Bay in Louisiana (Miller 2003; Wells et al. 2017; Takeshita et al. 2021); Mississippi Sound in Mississippi (Hubard et al. 2004; Mackey 2010; Mullin et al. 2017); Charlotte Harbor and Pine Island Sound (Bassos-Hull et al. 2013), Sarasota Bay in Florida (Wells 1986a; Wells 1991; Wells 2014), St. Andrew Bay (Balmer et al. 2019), St. Joseph Bay (Balmer et al. 2008; Balmer et al. 2018), St. Vincent Sound and Apalachicola Bay (Tyson et al. 2011), and Tampa Bay in Florida (Wells et al. 1996b; Urian et al. 2009).

Lifetime Reproductive Potential

Taylor et al. (2007) reported the interbirth interval of common bottlenose dolphins as 3.80 years, though other studies have shown it to be closer to three years (Connor et al. 2000; Leatherwood 1977; Wells 2003; Wells and Scott 1999).

Generally, females reach sexual maturity before males, with females reaching sexual maturity at 5–13 years (Sergeant et al. 1973; Perrin and Reilly 1984; Wells et al. 1987; Mead and Potter 1990; Mann et al. 2000; Wells 2000; Wells and Scott 2018).

Females reproduce through most of their adult lives (Leatherwood 1977; Hohn et al. 1989; Wells and Scott 1999; Wells 2000; Wells 2003), but may produce fewer calves after they reach 30 years of age (Wells 2000). Sarasota Bay common bottlenose dolphins can give birth when they are as young as 6 years of age, and can continue until they are 48 years old (Wells and Scott 2018). Reproductive lifespan would therefore be up to 42 years based on long-term observation studies (Wells 2014; Wells and Scott 2018).

Longitudinal studies from Sarasota Bay, Florida have found that female common bottlenose dolphins can live up to 67 years (Wells and Scott 2018). Studies based on growth layer groups in teeth suggest female common bottlenose dolphins can live to 41 years along the Texas coast (Fernandez and Hohn 1998) and to 30 years along the Mississippi coast (Mattson et al. 2006).

Generation Length

Taylor et al. (2007) reported common bottlenose dolphin generation length of 20.6-21.1 years.

Reproductive Plasticity

Common bottlenose dolphin calving is generally diffusely seasonal, with one or more peaks, although births have been reported from all seasons (Urian et al. 1996; Vollmer and Rosel 2013; Wells and Scott 2018). Calf sightings peak in the spring and summer months in Florida (Wells et al. 1987), Mississippi/Louisiana (Miller et al. 2010, 2013), and Texas (McHugh 1989; Henderson et al. 2007).

Migration

The BSE stocks in the Gulf of Mexico are thought to primarily comprise stable resident communities. However, transient or seasonal movements of some individuals into and out of a BSE stock area are recorded in some areas (LaBrecque et al. 2015; Hayes et al. 2022). Molecular genetic data support that some BSE stocks are genetically distinct from one another, and furthermore are differentiated from adjacent coastal populations (e.g., Sellas et al. 2005; Rosel et al. 2017). For example, Toms (2019) found fine-scale genetic population structure among five inshore systems in the Florida Panhandle and variable migration rates among populations.

Year-round residency has been reported for common bottlenose dolphins (Hayes et al. 2022), such as in Florida (Irvine and Wells 1972; Irvine et al. 1981; Wells 1986a; Wells 1986b; Wells et al. 1987; Scott et al. 1990; Wells 1991; Wells et al. 1996a; Wells et al. 1996b; Wells et al. 1997; Wells 2003; Balmer et al. 2008; Urian et al. 2009; Tyson et al. 2011; Bassos-Hull et al. 2013; Balmer et al. 2018; Balmer et al. 2019; Toms 2019), Texas (Shane 1990; Bräger 1993; Bräger et al. 1994; Fertl 1994; Weller 1998; Maze and Würsig 1999; Lynn and Würsig 2002; Irwin and Würsig 2004; Shane 2004), and Louisiana/Mississippi (Hubard et al. 2004; Mullin et al. 2017).

Home Range

The home ranges of individuals within the Gulf of Mexico BSE stocks considered to generally include the areas within the stock boundaries and brief excursions beyond the stock boundaries (Irvine et al. 1981; Wells 1986a; Wells 1986b; Wells 1991; Wells et al. 1996a; Wells et al. 1996b; Balmer et al. 2008; Wells 2014; Balmer et al. 2019), although some individuals show preference for small areas within the stock boundaries (Lynn and Würsig 2002; Urian et al. 2009; Mullin et al. 2017; Wells et al. 2017; Takeshita et al. 2021).

Stock Abundance

Blaylock and Hoggard (1994) estimated this stock's abundance at 61 individuals (CV=0.45). More recent estimates are unavailable (Hayes et al. 2022).

Stock Abundance Trend

Data are insufficient to complete an abundance trend analysis for this stock (Hayes et al. 2022).

Cumulative Stressors

Common bottlenose dolphins face a wide range of individual and cumulative natural and anthropogenic threats (see reviews by Vollmer and Rosel 2013; Phillips and Rosel 2014; Wells and Scott 2018; and Wells et al. 2019). These include such threats as shark predation, harmful algal blooms, recreational and commercial fishing gear, vessel traffic, pollution, habitat alteration, and provisioning.

Phillips and Rosel (2014) reviewed the cumulative stressors influencing the Matagorda Bay system. Potential health threats range from contagious disease (e.g., *Brucella*), harmful algal blooms (HABs; Fire et al. 2011, 2020), and anthropogenic activities: mining, crude oil exploration, transport, and refinement, commercial fisheries, agricultural runoff in the form of pesticide and fertilizer, heavy metals and other chemical contaminants, marine debris, shipping and dredging, noise, tourism and boat traffic.

Natural events may also pose threats: hypoxia resulting from HABs, adverse weather (e.g., hurricane storm surge), habitat loss, and climate change (Phillips and Rosel 2014).

Unusual Mortality Events (UMEs) have been declared for common bottlenose dolphins in waters of Texas with morbillivirus the confirmed cause in 1994, a suspected cause in 1992 along with environmental conditions, and an undetermined cause in 2008 (Litz et al. 2014). A UME was declared in 1990 for the Gulf of Mexico from Florida to Texas, with more than 300 common bottlenose dolphins stranding due to an undetermined cause, although morbillivirus and cold temperatures may have contributed (Litz et al. 2014).

Further Reading

Hayes, S.A., E. Josephson, K. Maze-Foley, P.E. Rosel, and J. Wallace. 2022. U.S. Atlantic and Gulf of Mexico marine mammal stock assessments 2021. National Oceanic and Atmospheric Administration, National Marine Fisheries Service, Northeast Fisheries Science Center: Woods Hole, MA. NOAA Technical Memorandum NMFS-NE-288. 380 p. doi: 10.25923/6tt7-kc16

Wells, R.S. and M.D. Scott. 2018. Bottlenose Dolphin, *Tursiops truncatus*, Common Bottlenose Dolphin. In: Würsig, B., J.G.M. Thewissen, and K.M. Kovacs (Eds.), Encyclopedia of Marine Mammals (Third Edition). Academic Press, London, UK. pp. 118–125. doi: 10.1016/B978-0-12-804327-1.00072-8

Wells, R.S., A. Natoli, and G. Braulik. 2019. *Tursiops truncatus* (errata version published in 2019). The IUCN Red List of Threatened Species 2019: e.T22563A156932432. https://dx.doi.org/10.2305/IUCN.UK.2019-1.RLTS.T22563A156932432.en

References

Balmer, B., T. McDonald, F. Hornsby, J. Adams, J. Allen, A. Barleycorn, and L. Schwacke. 2018. Long-term trends in a northern Gulf of Mexico common bottlenose dolphin (*Tursiops truncatus*) population in the wake of the Deepwater Horizon oil spill. Journal of Cetacean Research and Management 18:1–9. doi: 10.47536/jcrm.v18i1.409

Balmer, B., S. Watwood, B. Quigley, T. Speakman, K. Barry, K. Mullin, P. Rosel, C. Sinclair, E. Zolman, and L. Schwacke. 2019. Common bottlenose dolphin (*Tursiops truncatus*) abundance and distribution patterns in St. Andrew Bay, Florida, USA. Aquatic Conservation: Marine and Freshwater Ecosystems 29(3):486–498. doi: 10.1002/aqc.3001

Balmer, B.C., R.S. Wells, S.M. Nowacek, D.P. Nowacek, L.H. Schwacke, W.A. McLellan, F.S. Scharf, T.K. Rowles, L.J. Hansen, T.R. Spradlin, and D.A. Pabst. 2008. Seasonal abundance and distribution patterns of common bottlenose dolphins (*Tursiops truncatus*) near St. Joseph Bay, Florida, USA. Journal of Cetacean Research and Management 10(2):157–167.

Bassos-Hull, K., R.M. Perrtree, C.C. Shepard, S. Schilling, A.A. Barleycorn, J.B. Allen, B.C. Balmer, W.E. Pine, and R.S. Wells. 2013. Long-term site fidelity and seasonal abundance estimates of common bottlenose dolphins (*Tursiops truncatus*) along the southwest coast of Florida and responses to natural perturbations. Journal of Cetacean Research and Management 13(1):19–30. doi: 10.47536/jcrm.v13i1.551

Berens McCabe, E.J., D.P. Gannon, N.B. Barros, and R.S. Wells. 2010. Prey selection by resident common bottlenose dolphins (*Tursiops truncatus*) in Sarasota Bay, Florida. Marine Biology 157(5):931–942. doi: 10.1007/s00227-009-1371-2

Blaylock, R.A. and W. Hoggard. 1994. Preliminary estimates of bottlenose dolphin abundance in southern U.S. Atlantic and Gulf of Mexico continental shelf waters. National Oceanic and

Atmospheric Administration, National Marine Fisheries Service, Southeast Fisheries Science Center: Miami, FL. NOAA Technical Memorandum NMFS-SEFSC-356. 10 p.

Bowen-Stevens, S.R., D.P. Gannon, R.A. Hazelkorn, G. Lovewell, K.M. Volker, S. Smith, M.C. Tumlin, and J. Litz. 2021. Diet of common bottlenose dolphins, *Tursiops truncatus*, that stranded in and near Barataria Bay, Louisiana, 2010–2012. Southeastern Naturalist 20(1). doi: 10.1656/058.020.0113

Bräger, S. 1993. Diurnal and seasonal behavior patterns of bottlenose dolphins (*Tursiops truncatus*). Marine Mammal Science 9:434–438. doi: 10.1111/j.1748-7692.1993.tb00477.x

Bräger, S., B. Würsig, A. Acevedo, and T. Henningsen. 1994. Association patterns of bottle-nosed dolphins (*Tursiops-truncatus*) in Galveston Bay, Texas. Journal of Mammalogy 75(2):431–437. doi: 10.2307/1382564

Connor, R.C., R.S. Wells, J. Mann, and A.J. Read. 2000. The bottlenose dolphin: Social relationships in a fission-fusion society. In: Mann, J., R. Connor, P. Tyack, and H. Whitehead (Eds.), Cetacean Societies: Field Studies of Dolphins and Whales. University of Chicago Press, Chicago, IL. pp. 91–126.

Fernandez, S. and A.A. Hohn. 1998. Age, growth, and calving season of bottlenose dolphins, *Tursiops truncatus*, off coastal Texas. Fishery Bulletin 96(2):357–365.

Fertl, D. 1994. Occurrence patterns and behavior of bottlenose dolphins (*Tursiops truncatus*) in the Galveston ship channel, Texas. Texas Journal of Science 46(4):299–318.

Fire, S.E., T.A. Leighfield, G.A. Miller, S. Piwetz, E.R. Sabater, and H. Whitehead. 2020. Association between red tide exposure and detection of corresponding neurotoxins in bottlenose dolphins from Texas waters during 2007-2017. Marine Environmental Research 162:105191. doi: 10.1016/j.marenvres.2020.105191

Fire, S.E., Z. Wang, M. Byrd, H.R. Whitehead, J. Paternoster, and S.L. Morton. 2011. Co-occurrence of multiple classes of harmful algal toxins in bottlenose dolphins (*Tursiops truncatus*) stranding during an unusual mortality event in Texas, USA. Harmful Algae 10(3):330–336. doi: 10.1016/j.hal.2010.12.001

Hayes, S.A., E. Josephson, K. Maze-Foley, P.E. Rosel, and J. Wallace. 2022. U.S. Atlantic and Gulf of Mexico marine mammal stock assessments 2021. National Oceanic and Atmospheric Administration, National Marine Fisheries Service, Northeast Fisheries Science Center: Woods Hole, MA. NOAA Technical Memorandum NMFS-NE-288. 380 p. doi: 10.25923/6tt7-kc16

Henderson, E.E. and B. Würsig. 2007. Behavior patterns of bottlenose dolphins in San Luis Pass, Texas. Gulf of Mexico Science 25(2):153–161. doi: 10.18785/goms.2502.06

Hohn, A.A., M.D. Scott, R.S. Wells, J.C. Sweeney, and A.B. Irvine. 1989. Growth layers in teeth from known-age, free-ranging bottlenose dolphins. Marine Mammal Science 5(4):315–342. doi: 10.1111/j.1748-7692.1989.tb00346.x

Hubard, C.W., K. Maze-Foley, K.D. Mullin, and W.W. Schroeder. 2004. Seasonal abundance and site fidelity of bottlenose dolphins (*Tursiops truncatus*) in Mississippi Sound. Aquatic Mammals 30(2):299–310. doi: 10.1578/am.30.2.2004.299

Irvine, A.B., M.D. Scott, R.S. Wells, and J.H. Kaufmann. 1981. Movements and activities of the Atlantic bottlenose dolphin, *Tursiops truncatus*, near Sarasota, Florida. Fishery Bulletin 79(4):671–688.

Irvine, B. and R.S. Wells. 1972. Results of attempts to tag Atlantic bottlenosed dolphins (*Tursiops truncatus*). Cetology 13:1–5.

Irwin, L.-J. and B. Würsig. 2004. A small resident community of bottlenose dolphins, *Tursiops truncatus*, in Texas: Monitoring recommendations. Gulf of Mexico Science 22(1):13–21. doi: 10.18785/goms.2201.02

LaBrecque, E., C. Curtice, J. Harrison, S.M. Van Parijs, and P.N. Halpin. 2015. 3. Biologically Important Areas for cetaceans within U.S. Waters – Gulf of Mexico Region. Aquatic Mammals 41(1):30–38. doi: 10.1578/am.41.1.2015.30

Leatherwood, S. 1977. Some preliminary impressions on the numbers and social behavior of free-swimming bottlenosed dolphin calves (*Tursiops truncatus*) in the northern Gulf of Mexico. In: Ridgway, S.H. and K. Bernirschke (Eds.), Breeding Dolphins; Present Status, Suggestions for the Future. Final report to the US Marine Mammal Commission, Contract MM6AC009, MMC-76/07, Washington, DC. pp. 143–167.

Litz, J.A., M.A. Baran, S.R. Bowen-Stevens, R.H. Carmichael, K.M. Colegrove, L.P. Garrison, S.E. Fire, E.M. Fougeres, R. Hardy, S. Holmes, W. Jones, B.E. Mase-Guthrie, D.K. Odell, P.E. Rosel, J.T. Saliki, D.K. Shannon, S.F. Shippee, S.M. Smith, E.M. Stratton, M.C. Tumlin, H.R. Whitehead, G.A. Worthy, and T.K. Rowles. 2014. Review of historical unusual mortality events (UMEs) in the Gulf of Mexico (1990–2009): providing context for the multi-year northern Gulf of Mexico cetacean UME declared in 2010. Diseases of Aquatic Organisms 112(2):161–175. doi: 10.3354/dao02807

Lynn, S.K. and B. Würsig. 2002. Summer movement patterns of bottlenose dolphins in a Texas bay. Gulf of Mexico Science 20(1):25–37. doi: 10.18785/goms.2001.03

Mackey, A.D. 2010. Site fidelity and association patterns of bottlenose dolphins (*Tursiops truncatus*) in the Mississippi Sound. University of Southern Mississippi. Thesis. 133 p.

Mann, J., R.C. Connor, L.M. Barre, and M.R. Heithaus. 2000. Female reproductive success in bottlenose dolphins (*Tursiops* sp.): life history, habitat, provisioning, and group-size effects. Behavioral Ecology 11(2):210–219. doi: 10.1093/beheco/11.2.210

Mattson, M.C., K.D. Mullin, G.W. Ingram, and W. Hoggard. 2006. Age structure and growth of the bottlenose dolphin (*Tursiops truncatus*) from strandings in the Mississippi sound region of the north-central Gulf of Mexico from 1986 to 2003. Marine Mammal Science 22(3):654–666. doi: 10.1111/j.1748-7692.2006.00057.x

Maze, K.S. and B. Würsig. 1999. Bottlenosed dolphins of San Luis Pass, Texas: Occurrence patterns, site-fidelity, and habitat use. Aquatic Mammals 25:91–104.

McHugh, M.B. 1989. Population numbers and feeding behavior of the Atlantic bottlenose dolphin (*Tursiops truncatus*) near Aransas Pass, Texas. University of Texas at Austin. Thesis. 96 p.

Mead, J.G. and C.W. Potter. 1990. Natural history of bottlenose dolphins along the central Atlantic coast of the United States. In: Leatherwood, S. and R.R. Reeves (Eds.), The Bottlenose Dolphin. Academic Press, San Diego, CA. pp. 165–195.

Miller, C.E. 2003. Abundance trends and environmental habitat usage patterns of bottlenose dolphins (*Tursiops truncatus*) in lower Barataria and Caminada bays, Louisiana. Louisiana State University and Agricultural & Mechanical College. Dissertation. 125 p.

Miller, L.J., A.D. Mackey, T. Hoffland, M. Solangi, and S.A. Kuczaj. 2010. Potential effects of a major hurricane on Atlantic bottlenose dolphin (*Tursiops truncatus*) reproduction in the Mississippi Sound. Marine Mammal Science 26(3):707–715. doi: 10.1111/j.1748-7692.2010.00371.x

Miller, L.J., A.D. Mackey, M. Solangi, and S.A. Kuczaj. 2013. Population abundance and habitat utilization of bottlenose dolphins in the Mississippi Sound. Aquatic Conservation: Marine and Freshwater Ecosystems 23(1):145–151. doi: 10.1002/aqc.2278

Mullin, K.D., T. McDonald, R.S. Wells, B.C. Balmer, T. Speakman, C. Sinclair, E.S. Zolman, F. Hornsby, S.M. McBride, K.A. Wilkinson, and L.H. Schwacke. 2017. Density, abundance, survival, and ranging patterns of common bottlenose dolphins (*Tursiops truncatus*) in Mississippi Sound following the Deepwater Horizon oil spill. PLoS ONE 12(10):e0186265. doi: 10.1371/journal.pone.0186265

Perrin, W.F. and S.B. Reilly. 1984. Reproductive parameters of dolphins and small whales of the family Delphinidae. In: Perrin, W.F., R.L. Brownell, Jr., and D.P. DeMaster (Eds.), Reproduction in Whales, Dolphins, and Porpoises. pp. 97–134.

Phillips, N.M. and P.E. Rosel. 2014. A method for prioritizing research on common bottlenose dolphin stocks through evaluating threats and data availability: Development and application to bay, sound and estuary stocks in Texas. National Oceanic and Atmospheric Administration, National

Marine Fisheries Service, Southeast Fisheries Science Center: Lafayette, LA. NOAA Technical Memorandum NMFS-SEFSC-665. 146 p. doi: 10.7289/V5F769H8

Rosel, P.E., L.A. Wilcox, C. Sinclair, T.R. Speakman, M.C. Tumlin, J.A. Litz, and E.S. Zolman. 2017. Genetic assignment to stock of stranded common bottlenose dolphins in southeastern Louisiana after the Deepwater Horizon oil spill. Endangered Species Research 33:221–234. doi: 10.3354/esr00780

Rossman, S., P.H. Ostrom, M. Stolen, N.B. Barros, H. Gandhi, C.A. Stricker, and R.S. Wells. 2015. Individual specialization in the foraging habits of female bottlenose dolphins living in a trophically diverse and habitat rich estuary. Oecologia 178(2):415–425. doi: 10.1007/s00442-015-3241-6

Scott, M.D. and S.J. Chivers. 1990. Distribution and herd structure of bottlenose dolphins in the eastern tropical Pacific Ocean. In: Leatherwood, S. and R.R. Reeves (Eds.), The Bottlenose Dolphin. Academic Press, San Diego, CA. pp. 387–402.

Sellas, A.B., R.S. Wells, and P.E. Rosel. 2005. Mitochondrial and nuclear DNA analyses reveal fine scale geographic structure in bottlenose dolphins (*Tursiops truncatus*) in the Gulf of Mexico. Conservation Genetics 6(5):715–728. doi: 10.1007/s10592-005-9031-7

Sergeant, D.E., D.K. Caldwell, and M.C. Caldwell. 1973. Age, growth, and maturity of bottlenosed dolphin (*Tursiops-truncatus*) from northeast Florida. Journal of the Fisheries Research Board of Canada 30(7):1009–1011. doi: 10.1139/f73-165

Shane, S.H. 1990. Behavior and ecology of the bottlenose dolphin at Sanibel Island, Florida. In: Leatherwood, S. and R.R. Reeves (Eds.), The Bottlenose Dolphin. Academic Press, San Diego, CA. pp. 245–265. doi: 10.1016/B978-0-12-440280-5.50016-0

Shane, S.H. 2004. Residence patterns, group characteristics, and association patterns of bottlenose dolphins near Sanibel Island, Florida. Gulf of Mexico Science 22(1):1–12. doi: 10.18785/goms.2201.01

Shane, S.H., R.S. Wells, and B. Würsig. 1986. Ecology, behavior and social organization of the bottlenose dolphin: A review. Marine Mammal Science 2:34–63. doi: 10.1111/j.1748-7692.1986.tb00026.x

Takeshita, R., B.C. Balmer, F. Messina, E.S. Zolman, L. Thomas, R.S. Wells, C.R. Smith, T.K. Rowles, and L.H. Schwacke. 2021. High site-fidelity in common bottlenose dolphins despite low salinity exposure and associated indicators of compromised health. PLoS ONE 16(9):e0258031. doi: 10.1371/journal.pone.0258031

Taylor, B.L., S.J. Chivers, J. Larese, and W.F. Perrin. 2007. Generation length and percent mature estimates for IUCN assessments of cetaceans. National Oceanic and Atmospheric Administration, National Marine Fisheries Service, Southwest Fisheries Science Center: La Jolla, CA. Administrative Report LJ-07-01. 24 p.

Toms, C. 2019. Filling the gaps: Common bottlenose dolphin (*Tursiops truncatus*) population dynamics, structure, and connectivity within Florida panhandle bays, sounds, and estuaries. University of Central Florida. Dissertation. 373 p.

Torres, L.G. and A.J. Read. 2009. Where to catch a fish? The influence of foraging tactics on the ecology of bottlenose dolphins (*Tursiops truncatus*) in Florida Bay, Florida. Marine Mammal Science 25(4):797–815. doi: 10.1111/j.1748-7692.2009.00297.x

Tyson, R.B., S.M. Nowacek, and D.P. Nowacek. 2011. Community structure and abundance of bottlenose dolphins *Tursiops truncatus* in coastal waters of the northeast Gulf of Mexico. Marine Ecology Progress Series 438:253–265. doi: 10.3354/meps09292

Urian, K.W., D.A. Duffield, A.J. Read, R.S. Wells, and E.D. Shell. 1996. Seasonality of reproduction in bottlenose dolphins, *Tursiops truncatus*. Journal of Mammalogy 77(2):394–403. doi: 10.2307/1382814

Urian, K.W., S. Hofmann, R.S. Wells, and A.J. Read. 2009. Fine-scale population structure of bottlenose dolphins (*Tursiops truncatus*) in Tampa Bay, Florida. Marine Mammal Science 25(3):619–638. doi: 10.1111/j.1748-7692.2009.00284.x

Vollmer, N.L. and P.E. Rosel. 2013. A review of common bottlenose dolphins (*Tursiops truncatus truncatus*) in the Northern Gulf of Mexico: Population biology, potential threats, and management. Southeastern Naturalist 12(m6):1–43. doi: 10.1656/058.012.m601

Weller, D.W. 1998. Global and regional variation in the biology and behavior of bottlenose dolphins. Texas A&M University. Dissertation. 142 p.

Wells, R.S. 1986a. Population structure of bottlenose dolphins: Behavioral studies along the central west coast of Florida. Contract Report to National Marine Fisheries Service, Southeast Fisheries Center. Contract No. 45-WCNF-5-003BB. 58 p.

Wells, R.S. 1986b. Structural aspects of dolphin societies. University of California, Santa Cruz. Dissertation. 234 p.

Wells, R.S. 1991. The role of long-term study in understanding the social structure of a bottlenose dolphin community. In: Pryor, K. and K. Norris (Eds.), Dolphin Societies: Discoveries and Puzzles. University of California Press, Berkeley, CA. pp. 199–226.

Wells, R.S. 2000. Reproduction in wild bottlenose dolphins: Overview of patterns observed during a long-term study. In: Duffield, D. and T. Robeck (Eds.), Bottlenose Dolphin Reproduction Workshop. AZA Marine Mammal Taxon Advisory Group, Silver Spring, MD. pp. 57–74.

Wells, R.S. 2003. Dolphin social complexity: Lessons from long-term study and life history. In: de Waal, F.B.M. and P.L. Tyack (Eds.), Animal Social Complexity: Intelligence, Culture, and Individualized Societies Harvard University Press, Cambridge, MA. pp. 32–56.

Wells, R.S. 2014. Social structure and life history of bottlenose dolphins near Sarasota Bay, Florida: Insights from four decades and five generations. Primates and cetaceans. pp. 149–172. doi: 10.1007/978-4-431-54523-1_8

Wells, R.S., M.K. Bassos, K.W. Urian, W.J. Carr, and M.D. Scott. 1996. Low-level monitoring of bottlenose dolphins, *Tursiops truncatus*, in Charlotte Harbor, Florida: 1990-1994. National Oceanic and Atmospheric Administration, National Marine Fisheries Service, Southeast Fisheries Science Center: Miami, FL. NOAA Technical Memorandum NMFS-SEFSC-384. 86 p.

Wells, R.S., M.K. Bassos, K.W. Urian, S.H. Shane, E.C.G. Owen, C.F. Weiss, W.J. Carr, and M.D. Scott. 1997. Low-level monitoring of bottlenose dolphins, *Tursiops truncatus*, in Pine Island Sound, Florida: 1996. National Oceanic and Atmospheric Administration, National Marine Fisheries Service, Southeast Fisheries Science Center: Miami, FL. Southeast Fisheries Center Contribution No. 40-WCNF601958. 86 p.

Wells, R.S., A. Natoli, and G. Braulik. 2019. *Tursiops truncatus* (errata version published in 2019). *The IUCN Red List of Threatened Species* 2019: e.T22563A156932432. https://dx.doi.org/10.2305/IUCN.UK.2019-1.RLTS.T22563A156932432.en

Wells, R.S., L.H. Schwacke, T.K. Rowles, B.C. Balmer, E. Zolman, T. Speakman, F.I. Townsend, M.C. Tumlin, A. Barleycorn, and K.A. Wilkinson. 2017. Ranging patterns of common bottlenose dolphins *Tursiops truncatus* in Barataria Bay, Louisiana, following the Deepwater Horizon oil spill. Endangered Species Research 33:159–180. doi: 10.3354/esr00732

Wells, R.S. and M.D. Scott. 1999. Bottlenose dolphin *Tursiops truncatus* (Montagu, 1821). In: Ridgway, S.H. and R. Harrison (Eds.), Handbook of marine mammals. Volume 6: The second book of dolphins and the porpoises. Academic Press, San Diego, CA. pp. 137–182.

Wells, R.S. and M.D. Scott. 2018. Bottlenose dolphin, *Tursiops truncatus*, common bottlenose dolphin. In: Würsig, B., J.G.M. Thewissen, and K.M. Kovacs (Eds.), Encyclopedia of Marine Mammals (Third Edition). Academic Press, London, UK. pp. 118–125. doi: 10.1016/B978-0-12-804327-1.00072-8

Wells, R.S., M.D. Scott, and A.B. Irvine. 1987. The social structure of free-ranging bottlenose dolphins. In: Genoways, H.H. (Ed.), Current Mammalogy. Plenum Press, New York, NY. pp. 247–305.

Wells, R.S., K.W. Urian, A.J. Read, M.K. Bassos, W.J. Carr, and M.D. Scott. 1996. Low-level monitoring of bottlenose dolphins, *Tursiops truncatus*, in Tampa Bay, Florida: 1988-1993. National Oceanic and Atmospheric Administration, National Marine Fisheries Service, Southeast Fisheries Science Center: Miami, FL. NOAA Technical Memorandum NMFS-SEFSC-385. 36 p.

Wilson, R.M., R.B. Tyson, J.A. Nelson, B.C. Balmer, J.P. Chanton, and D.P. Nowacek. 2017. Niche differentiation and prey selectivity among common bottlenose dolphins (*Tursiops truncatus*) sighted in St. George Sound, Gulf of Mexico. Frontiers in Marine Science 4:235. doi: 10.3389/fmars.2017.00235

Common bottlenose dolphin − *Tursiops truncatus*

GoMx BSE/Mississippi River Delta Stock

Overall Vulnerability Rank = Very High

Biological Sensitivity = Very High

Climate Exposure = Very High

Data Quality = 96% of scores ≥ 2

	Tursiops truncatus	Expert Scores	Data Quality	Expert Scores Plots (Portion by Category)
Sensitivity attributes	Prey/Diet Specificity	2.1	2.7	
	Habitat Specificity	3.7	2.0	
	Site Fidelity	3.7	2.0	
	Lifetime Reproductive Potential	1.3	2.0	
	Generation Length	2.5	2.3	
	Reproductive Plasticity	2.2	2.3	
	Migration	3.9	2.0	
	Home Range	3.4	2.0	
	Stock Abundance	3.0	2.7	
	Stock Abundance Trend	2.4	1.0	
	Cumulative Stressors	2.9	2.3	
	Sensitivity Score	Very High		
Exposure variables	Sea Surface Temperature (Change in mean)	4.0	2.7	
	Sea Surface Temperature (Change in variability)	1.0	2.7	
	Air Temperature (Change in mean)	4.0	2.7	
	Air Temperature (Change in variability)	1.0	2.7	
	Precipitation (Change in mean)	1.0	2.7	
	Precipitation (Change in variability)	1.0	2.7	
	Sea Surface Salinity (Change in mean)	1.0	2.7	
	Sea Surface Salinity (Change in variability)	1.0	2.7	
	Ocean pH (Change in mean)	4.0	2.7	
	Ocean pH (Change in variability)	1.0	2.7	
	Sea ice coverage (Change in mean)	1.0	3.0	
	Sea ice coverage (Change in variability)	1.0	3.0	
	Dissolved oxygen (Change in mean)	4.0	2.7	
	Dissolved oxygen (Change in variability)	1.0	2.7	
	Circulation	3.7	2.7	
	Sea level rise	4.0	2.7	
	Exposure Score	Very High		
Overall Vulnerability Rank		Very High		

Low
Moderate
High
Very High

Common bottlenose dolphin (GoMx BSE/Mississippi River Delta Stock)

Tursiops truncatus

CVA Results Summary

Overall Climate Vulnerability Rank: Very High (100% certainty from bootstrap analysis).

Climate Exposure: Very High. Six exposure factors scored greater than 3.5: Air Temperature (Standard anomaly) (4.00), Dissolved oxygen (Standard anomaly) (4.00), Ocean pH (Standard anomaly) (4.00), Sea level rise (4.00), Sea Surface Temperature (Standard anomaly) (4.00), and Circulation (3.67).

Biological Sensitivity: Very High. Three sensitivity attributes scored greater than 3.5: Migration (3.87), Habitat Specificity (3.67), and Site Fidelity (3.67).

Distributional Response: High

Abundance Response: High

Phenology Response: High

Data Quality: 96% of the data quality scores were 2 or greater, 91% of sensitivity attribute data quality scores were 2 or higher.

Background Information

Stock Description

This stock includes common bottlenose dolphins in the Mississippi River Delta in the northern Gulf of Mexico along the Louisiana coast (Hayes et al. 2022).

Prey/Diet Specificity

Generally, common bottlenose dolphins consume a large variety of fish and/or squid (Barros and Odell 1990; Mead and Potter 1990; Wells and Scott 2018). Common bottlenose dolphins seem to show a consistent preference for sound-producing fishes (e.g., sciaenids, toadfish; Berens McCabe et al. 2010; Bowen-Stevens et al. 2021). In Sarasota Bay, Florida, Rossman et al. (2015) found some individual common bottlenose dolphins to show diet specialization despite the broader study group showing a generalist diet. Specialization can be influenced by locally abundant prey species (Shane et al. 1986; Vollmer and Rosel 2013) or can result from resource partitioning amongst sympatric populations (e.g., Torres and Read 2009; Wilson et al. 2017).

Habitat Specificity

Common bottlenose dolphins are found in a variety of marine and estuarine habitats (Wells and Scott 2018).

Site Fidelity

No specific site fidelity information was found for this stock. However, common bottlenose dolphins in other Gulf of Mexico bay, sound, and estuary (BSE) stocks show site fidelity (LaBrecque et al. 2015; Hayes et al. 2022), such as in Matagorda Bay, Espiritu Santo Bay, and San Luis Pass in Texas (Maze and Würsig 1999; Lynn and Würsig 2002; Irwin and Würsig 2004); Barataria Bay in Louisiana (Miller 2003; McDonald et al. 2017; Wells et al. 2017; Takeshita et al. 2021); Mississippi Sound in Mississippi (Hubard et al. 2004; Mackey 2010; Mullin et al. 2017); Charlotte Harbor and Pine Island Sound (Bassos-Hull et al. 2013), Sarasota Bay in Florida (Wells 1986a; Wells 1991; Wells 2014), St. Andrew Bay (Balmer et al. 2019), St. Joseph Bay (Balmer et al. 2008; B. Balmer et al. 2018), St. Vincent Sound and Apalachicola Bay (Tyson et al. 2011), and Tampa Bay in Florida (Wells et al. 1996b; Urian et al. 2009).

Lifetime Reproductive Potential

Taylor et al. (2007) reported the interbirth interval of common bottlenose dolphins as 3.80 years, though other studies have shown it to be closer to three years (Leatherwood 1977; Wells and Scott 1999; Connor et al. 2000; Wells 2003).

Generally, females reach sexual maturity before males, with females reaching sexual maturity at 5–13 years (Sergeant et al. 1973; Perrin and Reilly 1984; Wells et al. 1987; Mead and Potter 1990; Mann et al. 2000; Wells 2000; Wells and Scott 2018).

Females reproduce through most of their adult lives (Leatherwood 1977; Hohn et al. 1989; Wells and Scott 1999; Wells 2000; Wells 2003), but may produce fewer calves after they reach 30 years of age (Wells 2000). Sarasota Bay common bottlenose dolphins can give birth when they are as young as 6 years of age, and can continue until they are 48 years old (Wells and Scott 2018). Reproductive lifespan would therefore be up to 42 years based on long-term observation studies (Wells 2014; Wells and Scott 2018).

Longitudinal studies from Sarasota Bay, Florida have found that female common bottlenose dolphins can live up to 67 years (Wells and Scott 2018). Studies based on growth layer groups in teeth suggest female common bottlenose dolphins can live to 41 years along the Texas coast (Fernandez and Hohn 1998) and to 30 years along the Mississippi coast (Mattson et al. 2006).

Generation Length

Taylor et al. (2007) reported common bottlenose dolphin generation length of 20.6–21.1 years.

Reproductive Plasticity

Common bottlenose dolphin calving is generally diffusely seasonal, with one or more peaks, although births have been reported from all seasons (Vollmer and Rosel 2013; Wells and Scott 2018). Calf sightings peak in the spring and summer months in Florida (Wells et al. 1987), Mississippi/Louisiana (Miller et al. 2010; Miller et al. 2013), and Texas (McHugh 1989; Henderson et al. 2007).

Migration

The BSE stocks in the Gulf of Mexico are thought to primarily comprise stable resident communities. However, transient or seasonal movements of some individuals into and out of a BSE stock area are recorded in some areas (LaBrecque et al. 2015; Hayes et al. 2022). Molecular genetic data support that some BSE stocks are genetically distinct from one another, and furthermore are differentiated from adjacent coastal populations (e.g., Sellas et al. 2005; Rosel et al. 2017). For example, Toms (2019) found fine-scale genetic population structure among five inshore systems in the Florida Panhandle and variable migration rates among populations.

Year-round residency has been reported for common bottlenose dolphins (Hayes et al. 2022), such as in Florida (Irvine and Wells 1972; Irvine et al. 1981; Wells 1986a; Wells 1986b; Wells et al. 1987; Scott et al. 1990; Wells 1991; Wells et al. 1996a; Wells et al. 1996b; Wells et al. 1997; Wells 2003; Balmer et al. 2008; Urian et al. 2009; Tyson et al. 2011; Bassos-Hull et al. 2013; B. Balmer et al. 2018; Balmer et al. 2019; Toms 2019), Texas (Shane 1990; Bräger 1993; Bräger et al. 1994; Fertl 1994; Weller 1998; Maze and Würsig 1999; Lynn and Würsig 2002; Irwin and Würsig 2004; Shane 2004), and Louisiana/Mississippi (Hubard et al. 2004; McDonald et al. 2017; Mullin et al. 2017; Takeshita et al. 2021).

Home Range

The home ranges of individuals within the Gulf of Mexico BSE stocks are considered to generally include the areas within the stock boundaries and brief excursions beyond the stock boundaries (Irvine et al. 1981; Wells 1986a; Wells 1986b; Wells 1991; Wells et al. 1996a; Wells et al. 1996b; Balmer et al. 2008; Wells 2014; Balmer et al. 2019), although some individuals show preference for small areas within the stock boundaries (Lynn and Würsig 2002; Urian et al. 2009; Mullin et al. 2017; Wells et al. 2017).

Stock Abundance

Hayes et al. (2022) reported this stock's abundance estimate at 1,446 individuals (CV = 0.19) based on 2017 and 2018 surveys (Garrison et al. 2021). Hayes et al. (2019) reported this stock's abundance estimate at 332 individuals (CV = 0.93).

Stock Abundance Trend

Data are insufficient to complete an abundance trend analysis for this stock (Hayes et al. 2022).

Cumulative Stressors

Common bottlenose dolphins face a wide range of individual and cumulative natural and anthropogenic threats (see reviews by Vollmer and Rosel 2013; Wells and Scott 2018; and Wells et al. 2019). These include such threats as shark predation, harmful algal blooms, recreational and commercial fishing gear, vessel traffic, pollution, habitat alteration, and provisioning.

Common bottlenose dolphins in the Mississippi River Delta were likely exposed to high levels of oil and dispersants following the Deepwater Horizon oil spill, which may result in long term reproductive and chronic health effects (NOAA 2011; Michel et al. 2013; Helm et al. 2015; DWH NRDA 2016; Lane et al. 2015; De Guise et al. 2017, 2021; Kellar et al. 2017; Smith et al. 2017; Schwacke et al. 2017). An Unusual Mortality Event (UME), involving primarily bottlenose dolphins, was declared for cetaceans in the

northern Gulf of Mexico from 2010 to 2014, with the Deepwater Horizon oil spill as the primary underlying cause (Litz et al. 2014; Schwacke et al. 2014; Venn-Watson et al. 2015a, 2015b).

The nearshore and estuarine habitats along the Gulf of Mexico coast include waters adjacent to areas of high human population, some of which are highly industrialized, and areas of heavy agricultural impact. Studies of bottlenose dolphin tissues from nearby Barataria Bay have shown persistent organic pollutant (POP) in blubber and blood samples, though at concentrations lower than other southeastern U.S. sites (Kucklick et al. 2011; Balmer et al. 2015; J. Balmer et al. 2018). Similarly, mercury has been detected in common bottlenose dolphin tissues in Louisiana waters, but in lower concentrations than common bottlenose dolphins found in Florida waters (McCormack et al. 2020a, 2020b, 2022).

Dolphin morbillivirus has been detected in populations of the northern Gulf of Mexico, but has not been attributed to large scale die-offs since 1993−1994 (Lipscomb et al. 1996; Litz et al. 2014; Van Bressem et al. 2014; Fauquier et al. 2017). A UME was declared in 1990 for the Gulf of Mexico from Florida to Texas, with more than 300 common bottlenose dolphins stranding due to an undetermined cause, although morbillivirus and cold temperatures may have contributed (Litz et al. 2014).

Further Reading

Hayes, S.A., E. Josephson, K. Maze-Foley, P.E. Rosel, and J. Wallace. 2022. U.S. Atlantic and Gulf of Mexico marine mammal stock assessments 2021. National Oceanic and Atmospheric Administration, National Marine Fisheries Service, Northeast Fisheries Science Center: Woods Hole, MA. NOAA Technical Memorandum NMFS-NE-288. 380 p. doi: 10.25923/6tt7-kc16

Wells, R.S. and M.D. Scott. 2018. Bottlenose Dolphin, *Tursiops truncatus*, Common Bottlenose Dolphin. In: Würsig, B., J.G.M. Thewissen, and K.M. Kovacs (Eds.), Encyclopedia of Marine Mammals (Third Edition). Academic Press, London, UK. pp. 118–125. doi: 10.1016/B978-0-12-804327-1.00072-8

Wells, R.S., A. Natoli, and G. Braulik. 2019. *Tursiops truncatus* (errata version published in 2019). The IUCN Red List of Threatened Species 2019: e.T22563A156932432. https://dx.doi.org/10.2305/IUCN.UK.2019-1.RLTS.T22563A156932432.en

References

Balmer, B., T. McDonald, F. Hornsby, J. Adams, J. Allen, A. Barleycorn, and L. Schwacke. 2018. Long-term trends in a northern Gulf of Mexico common bottlenose dolphin (*Tursiops truncatus*) population in the wake of the Deepwater Horizon oil spill. Journal of Cetacean Research and Management 18:1–9. doi: 10.47536/jcrm.v18i1.409

Balmer, B., S. Watwood, B. Quigley, T. Speakman, K. Barry, K. Mullin, P. Rosel, C. Sinclair, E. Zolman, and L. Schwacke. 2019. Common bottlenose dolphin (*Tursiops truncatus*) abundance and distribution patterns in St. Andrew Bay, Florida, USA. Aquatic Conservation: Marine and Freshwater Ecosystems 29(3):486–498. doi: 10.1002/aqc.3001

Balmer, B.C., R.S. Wells, S.M. Nowacek, D.P. Nowacek, L.H. Schwacke, W.A. McLellan, F.S. Scharf, T.K. Rowles, L.J. Hansen, T.R. Spradlin, and D.A. Pabst. 2008. Seasonal abundance and distribution patterns of common bottlenose dolphins (*Tursiops truncatus*) near St. Joseph Bay, Florida, USA. Journal of Cetacean Research and Management 10(2):157–167.

Balmer, B.C., G.M. Ylitalo, L.E. McGeorge, K.A. Baugh, D. Boyd, K.D. Mullin, P.E. Rosel, C. Sinclair, R.S. Wells, E.S. Zolman, and L.H. Schwacke. 2015. Persistent organic pollutants (POPs) in blubber of

common bottlenose dolphins (*Tursiops truncatus*) along the northern Gulf of Mexico coast, USA. Science of the Total Environment 527-528:306–312. doi: 10.1016/j.scitotenv.2015.05.016

Balmer, J.E., G.M. Ylitalo, T.K. Rowles, K.D. Mullin, R.S. Wells, F.I. Townsend, R.W. Pearce, J.L. Bolton, E.S. Zolman, B.C. Balmer, and L.H. Schwacke. 2018. Persistent organic pollutants (POPs) in blood and blubber of common bottlenose dolphins (*Tursiops truncatus*) at three northern Gulf of Mexico sites following the Deepwater Horizon oil spill. Science of the Total Environment 621:130–137. doi: 10.1016/j.scitotenv.2017.11.209

Barros, N.B. and D.K. Odell. 1990. Food habits of bottlenose dolphins in the southeastern United States In: Leatherwood, S. and R.R. Reeves (Eds.), The Bottlenose Dolphin. Academic Press, San Diego, CA. pp. 309–328. doi: 10.1016/B978-0-12-440280-5.50020-2

Bassos-Hull, K., R.M. Perrtree, C.C. Shepard, S. Schilling, A.A. Barleycorn, J.B. Allen, B.C. Balmer, W.E. Pine, and R.S. Wells. 2013. Long-term site fidelity and seasonal abundance estimates of common bottlenose dolphins (*Tursiops truncatus*) along the southwest coast of Florida and responses to natural perturbations. Journal of Cetacean Research and Management 13(1):19–30. doi: 10.47536/jcrm.v13i1.551

Berens McCabe, E.J., D.P. Gannon, N.B. Barros, and R.S. Wells. 2010. Prey selection by resident common bottlenose dolphins (*Tursiops truncatus*) in Sarasota Bay, Florida. Marine Biology 157(5):931–942. doi: 10.1007/s00227-009-1371-2

Bowen-Stevens, S.R., D.P. Gannon, R.A. Hazelkorn, G. Lovewell, K.M. Volker, S. Smith, M.C. Tumlin, and J. Litz. 2021. Diet of common bottlenose dolphins, *Tursiops truncatus*, that stranded in and near Barataria Bay, Louisiana, 2010–2012. Southeastern Naturalist 20(1). doi: 10.1656/058.020.0113

Bräger, S. 1993. Diurnal and seasonal behavior patterns of bottlenose dolphins (*Tursiops truncatus*). Marine Mammal Science 9:434–438. doi: 10.1111/j.1748-7692.1993.tb00477.x

Bräger, S., B. Würsig, A. Acevedo, and T. Henningsen. 1994. Association patterns of bottle-nosed dolphins (*Tursiops-truncatus*) in Galveston Bay, Texas. Journal of Mammalogy 75(2):431–437. doi: 10.2307/1382564

Connor, R.C., R.S. Wells, J. Mann, and A.J. Read. 2000. The bottlenose dolphin: Social relationships in a fission-fusion society. In: Mann, J., R. Connor, P. Tyack, and H. Whitehead (Eds.), Cetacean Societies: Field Studies of Dolphins and Whales. University of Chicago Press, Chicago, IL. pp. 91–126.

De Guise, S., M. Levin, E. Gebhard, L. Jasperse, L. Burdett Hart, C.R. Smith, S. Venn-Watson, F. Townsend, R. Wells, B. Balmer, E. Zolman, T. Rowles, and L. Schwacke. 2017. Changes in immune functions in bottlenose dolphins in the northern Gulf of Mexico associated with the Deepwater Horizon oil spill. Endangered Species Research 33:291–303. doi: 10.3354/esr00814

De Guise, S., M. Levin, L. Jasperse, J. Herrman, R.S. Wells, T. Rowles, and L. Schwacke. 2021. Long-term immunological alterations in bottlenose dolphin a decade after the Deepwater Horizon oil spill in the northern Gulf of Mexico: Potential for multigenerational effects. Environmental Toxicology and Chemistry 40(5):1308–1321. doi: 10.1002/etc.4980

Deepwater Horizon Natural Resource Damage Assessment Trustees (DWH NRDA). 2016. Deepwater Horizon oil spill: Final Programmatic Damage Assessment and Restoration Plan and Final Programmatic Environmental Impact Statement. Retrieved from: http://www.gulfspillrestoration.noaa.gov/restoration-planning/gulf-plan.1659.

Fauquier, D.A., J. Litz, S. Sanchez, K. Colegrove, L.H. Schwacke, L. Hart, J. Saliki, C. Smith, T. Goldstein, S. Bowen-Stevens, W. McFee, E. Fougeres, B. Mase-Guthrie, E. Stratton, R. Ewing, S. Venn-Watson, R.H. Carmichael, C. Clemons-Chevis, W. Hatchett, D. Shannon, S. Shippee, S. Smith, L. Staggs, M.C. Tumlin, N.L. Wingers, and T.K. Rowles. 2017. Evaluation of morbillivirus exposure in cetaceans from the northern Gulf of Mexico 2010-2014. Endangered Species Research 33:211–220. doi: 10.3354/esr00772

Fernandez, S. and A.A. Hohn. 1998. Age, growth, and calving season of bottlenose dolphins, *Tursiops truncatus*, off coastal Texas. Fishery Bulletin 96(2):357–365.

Fertl, D. 1994. Occurrence patterns and behavior of bottlenose dolphins (*Tursiops truncatus*) in the Galveston ship channel, Texas. Texas Journal of Science 46(4):299–318.

Garrison, L.P., J. Ortega-Ortiz, and G. Rappucci. 2021. Abundance of coastal and continental shelf stocks of common bottlenose and Atlantic spotted dolphins in the northern Gulf of Mexico: 2017–2018. National Oceanic and Atmospheric Administration, National Marine Fisheries Service, Southeast Fisheries Science Center, Protected Resources and Biodiversity Division: Miami, FL. PRD-2021-01. 25 p. doi: 10.25923/vk95-t881

Hayes, S.A., E. Josephson, K. Maze-Foley, and P.E. Rosel. 2019. US Atlantic and Gulf of Mexico marine mammal stock assessments - 2018. National Oceanic and Atmospheric Administration, National Marine Fisheries Service, Northeast Fisheries Science Center: Woods Hole, MA. NOAA Technical Memorandum NMFS-NE-258. 298 p. doi: 10.25923/9rrd-tx13

Hayes, S.A., E. Josephson, K. Maze-Foley, P.E. Rosel, and J. Wallace. 2022. U.S. Atlantic and Gulf of Mexico marine mammal stock assessments 2021. National Oceanic and Atmospheric Administration, National Marine Fisheries Service, Northeast Fisheries Science Center: Woods Hole, MA. NOAA Technical Memorandum NMFS-NE-288. 380 p. doi: 10.25923/6tt7-kc16

Helm, R.C., D.P. Costa, T.D. DeBruyn, T.J. O'Shea, R.S. Wells, and T.M. Williams. 2015. Overview of effects of oil spills on marine mammals. In: Fingas, M. (Ed.), Handbook of oil spill science and technology, First edition. John Wiley & Sons, Inc., Hoboken, NJ. pp. 455–475.

Henderson, E.E. and B. Würsig. 2007. Behavior patterns of bottlenose dolphins in San Luis Pass, Texas. Gulf of Mexico Science 25(2):153–161. doi: 10.18785/goms.2502.06

Hohn, A.A., M.D. Scott, R.S. Wells, J.C. Sweeney, and A.B. Irvine. 1989. Growth layers in teeth from known-age, free-ranging bottlenose dolphins. Marine Mammal Science 5(4):315–342. doi: 10.1111/j.1748-7692.1989.tb00346.x

Hubard, C.W., K. Maze-Foley, K.D. Mullin, and W.W. Schroeder. 2004. Seasonal abundance and site fidelity of bottlenose dolphins (*Tursiops truncatus*) in Mississippi Sound. Aquatic Mammals 30(2):299–310. doi: 10.1578/am.30.2.2004.299

Irvine, A.B., M.D. Scott, R.S. Wells, and J.H. Kaufmann. 1981. Movements and activities of the Atlantic bottlenose dolphin, *Tursiops truncatus*, near Sarasota, Florida. Fishery Bulletin 79(4):671–688.

Irvine, B. and R.S. Wells. 1972. Results of attempts to tag Atlantic bottlenosed dolphins (*Tursiops truncatus*). Cetology 13:1–5.

Irwin, L.-J. and B. Würsig. 2004. A small resident community of bottlenose dolphins, *Tursiops truncatus*, in Texas: Monitoring recommendations. Gulf of Mexico Science 22(1):13–21. doi: 10.18785/goms.2201.02

Kellar, N.M., T.R. Speakman, C.R. Smith, S.M. Lane, B.C. Balmer, M.L. Trego, K.N. Catelani, M.N. Robbins, C.D. Allen, R.S. Wells, E.S. Zolman, T.K. Rowles, and L.H. Schwacke. 2017. Low reproductive success rates of common bottlenose dolphins *Tursiops truncatus* in the northern Gulf of Mexico following the Deepwater Horizon disaster (2010-2015). Endangered Species Research 33:143–158. doi: 10.3354/esr00775

Kucklick, J., L. Schwacke, R. Wells, A. Hohn, A. Guichard, J. Yordy, L. Hansen, E. Zolman, R. Wilson, J. Litz, D. Nowacek, T. Rowles, R. Pugh, B. Balmer, C. Sinclair, and P. Rosel. 2011. Bottlenose dolphins as indicators of persistent organic pollutants in the western North Atlantic Ocean and Northern Gulf of Mexico. Environmental Science & Technology 45(10):4270–4277. doi: 10.1021/es1042244

LaBrecque, E., C. Curtice, J. Harrison, S.M. Van Parijs, and P.N. Halpin. 2015. 3. Biologically Important Areas for cetaceans within U.S. Waters – Gulf of Mexico Region. Aquatic Mammals 41(1):30–38. doi: 10.1578/am.41.1.2015.30

Lane, S.M., C.R. Smith, J. Mitchell, B.C. Balmer, K.P. Barry, T. McDonald, C.S. Mori, P.E. Rosel, T.K. Rowles, T.R. Speakman, F.I. Townsend, M.C. Tumlin, R.S. Wells, E.S. Zolman, and L.H. Schwacke. 2015. Reproductive outcome and survival of common bottlenose dolphins sampled in Barataria Bay, Louisiana, USA, following the Deepwater Horizon oil spill. Proceedings of the Royal Society B: Biological Sciences 282(1818):20151944. doi: 10.1098/rspb.2015.1944

Leatherwood, S. 1977. Some preliminary impressions on the numbers and social behavior of free-swimming bottlenosed dolphin calves (*Tursiops truncatus*) in the northern Gulf of Mexico. In: Ridgway, S.H. and K. Bernirschke (Eds.), Breeding Dolphins; Present Status, Suggestions for the Future. Final report to the US Marine Mammal Commission, Contract MM6AC009, MMC-76/07, Washington, DC. pp. 143–167.

Lipscomb, T.P., S. Kennedy, D. Moffett, A. Krafft, B.A. Klaunberg, J.H. Lichy, G.T. Regan, G.A.J. Worthy, and J.K. Taubenberger. 1996. Morbilliviral epizootic in bottlenose dolphins of the Gulf of Mexico. Journal of Veterinary Diagnostic Investigation 8(3):283–290. doi: 10.1177/104063879600800302

Litz, J.A., M.A. Baran, S.R. Bowen-Stevens, R.H. Carmichael, K.M. Colegrove, L.P. Garrison, S.E. Fire, E.M. Fougeres, R. Hardy, S. Holmes, W. Jones, B.E. Mase-Guthrie, D.K. Odell, P.E. Rosel, J.T. Saliki, D.K. Shannon, S.F. Shippee, S.M. Smith, E.M. Stratton, M.C. Tumlin, H.R. Whitehead, G.A. Worthy, and T.K. Rowles. 2014. Review of historical unusual mortality events (UMEs) in the Gulf of Mexico (1990–2009): providing context for the multi-year northern Gulf of Mexico cetacean UME declared in 2010. Diseases of Aquatic Organisms 112(2):161–175. doi: 10.3354/dao02807

Lynn, S.K. and B. Würsig. 2002. Summer movement patterns of bottlenose dolphins in a Texas bay. Gulf of Mexico Science 20(1):25–37. doi: 10.18785/goms.2001.03

Mackey, A.D. 2010. Site fidelity and association patterns of bottlenose dolphins (*Tursiops truncatus*) in the Mississippi Sound. University of Southern Mississippi. Thesis. 133 p.

Mann, J., R.C. Connor, L.M. Barre, and M.R. Heithaus. 2000. Female reproductive success in bottlenose dolphins (*Tursiops* sp.): life history, habitat, provisioning, and group-size effects. Behavioral Ecology 11(2):210–219. doi: 10.1093/beheco/11.2.210

Mattson, M.C., K.D. Mullin, G.W. Ingram, and W. Hoggard. 2006. Age structure and growth of the bottlenose dolphin (*Tursiops truncatus*) from strandings in the Mississippi sound region of the north-central Gulf of Mexico from 1986 to 2003. Marine Mammal Science 22(3):654–666. doi: 10.1111/j.1748-7692.2006.00057.x

Maze, K.S. and B. Würsig. 1999. Bottlenosed dolphins of San Luis Pass, Texas: Occurrence patterns, site-fidelity, and habitat use. Aquatic Mammals 25:91–104.

McCormack, M.A., F. Battaglia, W.E. McFee, and J. Dutton. 2020a. Mercury concentrations in blubber and skin from stranded bottlenose dolphins (*Tursiops truncatus*) along the Florida and Louisiana coasts (Gulf of Mexico, USA) in relation to biological variables. Environmental Research 180:108886. doi: 10.1016/j.envres.2019.108886

McCormack, M.A., B.P. Jackson, and J. Dutton. 2020b. Relationship between mercury and selenium concentrations in tissues from stranded odontocetes in the northern Gulf of Mexico. Science of the Total Environment 749:141350. doi: 10.1016/j.scitotenv.2020.141350

McCormack, M.A., W.H. Nowlin, and J. Dutton. 2022. Effect of trophic position on mercury concentrations in bottlenose dolphins (*Tursiops truncatus*) from the northern Gulf of Mexico. Environmental Research 204(Pt B):112124. doi: 10.1016/j.envres.2021.112124

McDonald, T.L., F.E. Hornsby, T.R. Speakman, E.S. Zolman, K.D. Mullin, C. Sinclair, P.E. Rosel, L. Thomas, and L.H. Schwacke. 2017. Survival, density, and abundance of common bottlenose dolphins in Barataria Bay (USA) following the Deepwater Horizon oil spill. Endangered Species Research 33:193–209. doi: 10.3354/esr00806

McHugh, M.B. 1989. Population numbers and feeding behavior of the Atlantic bottlenose dolphin (*Tursiops truncatus*) near Aransas Pass, Texas. University of Texas at Austin. Thesis. 96 p.

Mead, J.G. and C.W. Potter. 1990. Natural history of bottlenose dolphins along the central Atlantic coast of the United States. In: Leatherwood, S. and R.R. Reeves (Eds.), The Bottlenose Dolphin. Academic Press, San Diego, CA. pp. 165–195.

Michel, J., E.H. Owens, S. Zengel, A. Graham, Z. Nixon, T. Allard, W. Holton, P.D. Reimer, A. Lamarche, M. White, N. Rutherford, C. Childs, G. Mauseth, G. Challenger, and E. Taylor. 2013. Extent and degree of shoreline oiling: Deepwater Horizon oil spill, Gulf of Mexico, USA. PLoS ONE 8(6):e65087. doi: 10.1371/journal.pone.0065087

Miller, C.E. 2003. Abundance trends and environmental habitat usage patterns of bottlenose dolphins (*Tursiops truncatus*) in lower Barataria and Caminada bays, Louisiana. Louisiana State University and Agricultural & Mechanical College. Dissertation. 125 p.

Miller, L.J., A.D. Mackey, T. Hoffland, M. Solangi, and S.A. Kuczaj. 2010. Potential effects of a major hurricane on Atlantic bottlenose dolphin (*Tursiops truncatus*) reproduction in the Mississippi Sound. Marine Mammal Science 26(3):707–715. doi: 10.1111/j.1748-7692.2010.00371.x

Miller, L.J., A.D. Mackey, M. Solangi, and S.A. Kuczaj. 2013. Population abundance and habitat utilization of bottlenose dolphins in the Mississippi Sound. Aquatic Conservation: Marine and Freshwater Ecosystems 23(1):145–151. doi: 10.1002/aqc.2278

Mullin, K.D., T. McDonald, R.S. Wells, B.C. Balmer, T. Speakman, C. Sinclair, E.S. Zolman, F. Hornsby, S.M. McBride, K.A. Wilkinson, and L.H. Schwacke. 2017. Density, abundance, survival, and ranging patterns of common bottlenose dolphins (*Tursiops truncatus*) in Mississippi Sound following the Deepwater Horizon oil spill. PLoS ONE 12(10):e0186265. doi: 10.1371/journal.pone.0186265

National Oceanic and Atmospheric Administration (NOAA). 2011. Public scoping for preparation of a programmatic environmental impact statement for the Deepwater Horizon BP Oil Spill. 9 p.

Perrin, W.F. and S.B. Reilly. 1984. Reproductive parameters of dolphins and small whales of the family Delphinidae. In: Perrin, W.F., R.L. Brownell, Jr., and D.P. DeMaster (Eds.), Reproduction in Whales, Dolphins, and Porpoises. pp. 97–134.

Rosel, P.E., L.A. Wilcox, C. Sinclair, T.R. Speakman, M.C. Tumlin, J.A. Litz, and E.S. Zolman. 2017. Genetic assignment to stock of stranded common bottlenose dolphins in southeastern Louisiana after the Deepwater Horizon oil spill. Endangered Species Research 33:221–234. doi: 10.3354/esr00780

Rossman, S., P.H. Ostrom, M. Stolen, N.B. Barros, H. Gandhi, C.A. Stricker, and R.S. Wells. 2015. Individual specialization in the foraging habits of female bottlenose dolphins living in a trophically diverse and habitat rich estuary. Oecologia 178(2):415–425. doi: 10.1007/s00442-015-3241-6

Schwacke, L.H., C.R. Smith, F.I. Townsend, R.S. Wells, L.B. Hart, B.C. Balmer, T.K. Collier, S. De Guise, M.M. Fry, L.J. Guillette, Jr., S.V. Lamb, S.M. Lane, W.E. McFee, N.J. Place, M.C. Tumlin, G.M. Ylitalo, E.S. Zolman, and T.K. Rowles. 2014. Health of common bottlenose dolphins (*Tursiops truncatus*) in Barataria Bay, Louisiana, following the deepwater horizon oil spill. Environmental Science & Technology 48(1):93–103. doi: 10.1021/es403610f

Schwacke, L.H., L. Thomas, R.S. Wells, W.E. McFee, A.A. Hohn, K.D. Mullin, E.S. Zolman, B.M. Quigley, T.K. Rowles, and J.H. Schwacke. 2017. Quantifying injury to common bottlenose dolphins from the Deepwater Horizon oil spill using an age-, sex- and class-structured population model. Endangered Species Research 33:265–279. doi: 10.3354/esr00777

Scott, M.D. and S.J. Chivers. 1990. Distribution and herd structure of bottlenose dolphins in the eastern tropical Pacific Ocean. In: Leatherwood, S. and R.R. Reeves (Eds.), The Bottlenose Dolphin. Academic Press, San Diego, CA. pp. 387–402.

Sellas, A.B., R.S. Wells, and P.E. Rosel. 2005. Mitochondrial and nuclear DNA analyses reveal fine scale geographic structure in bottlenose dolphins (*Tursiops truncatus*) in the Gulf of Mexico. Conservation Genetics 6(5):715–728. doi: 10.1007/s10592-005-9031-7

Sergeant, D.E., D.K. Caldwell, and M.C. Caldwell. 1973. Age, growth, and maturity of bottlenosed dolphin (*Tursiops-truncatus*) from northeast Florida. Journal of the Fisheries Research Board of Canada 30(7):1009–1011. doi: 10.1139/f73-165

Shane, S.H. 1990. Behavior and ecology of the bottlenose dolphin at Sanibel Island, Florida. In: Leatherwood, S. and R.R. Reeves (Eds.), The Bottlenose Dolphin. Academic Press, San Diego, CA. pp. 245–265. doi: 10.1016/B978-0-12-440280-5.50016-0

Shane, S.H. 2004. Residence patterns, group characteristics, and association patterns of bottlenose dolphins near Sanibel Island, Florida. Gulf of Mexico Science 22(1):1–12. doi: 10.18785/goms.2201.01

Shane, S.H., R.S. Wells, and B. Würsig. 1986. Ecology, behavior and social organization of the bottlenose dolphin: A review. Marine Mammal Science 2:34–63. doi: 10.1111/j.1748-7692.1986.tb00026.x

Smith, C.R., T.K. Rowles, L.B. Hart, F.I. Townsend, R.S. Wells, E.S. Zolman, B.C. Balmer, B. Quigley, M. Ivančić, W. McKercher, M.C. Tumlin, K.D. Mullin, J.D. Adams, Q. Wu, W. McFee, T.K. Collier, and L.H. Schwacke. 2017. Slow recovery of Barataria Bay dolphin health following the Deepwater Horizon oil spill (2013-2014), with evidence of persistent lung disease and impaired stress response. Endangered Species Research 33:127–142. doi: 10.3354/esr00778

Takeshita, R., B.C. Balmer, F. Messina, E.S. Zolman, L. Thomas, R.S. Wells, C.R. Smith, T.K. Rowles, and L.H. Schwacke. 2021. High site-fidelity in common bottlenose dolphins despite low salinity exposure and associated indicators of compromised health. PLoS ONE 16(9):e0258031. doi: 10.1371/journal.pone.0258031

Taylor, B.L., S.J. Chivers, J. Larese, and W.F. Perrin. 2007. Generation length and percent mature estimates for IUCN assessments of cetaceans. National Oceanic and Atmospheric Administration, National Marine Fisheries Service, Southwest Fisheries Science Center: La Jolla, CA. Administrative Report LJ-07-01. 24 p.

Toms, C. 2019. Filling the gaps: Common bottlenose dolphin (*Tursiops truncatus*) population dynamics, structure, and connectivity within Florida panhandle bays, sounds, and estuaries. University of Central Florida. Dissertation. 373 p.

Torres, L.G. and A.J. Read. 2009. Where to catch a fish? The influence of foraging tactics on the ecology of bottlenose dolphins (*Tursiops truncatus*) in Florida Bay, Florida. Marine Mammal Science 25(4):797–815. doi: 10.1111/j.1748-7692.2009.00297.x

Tyson, R.B., S.M. Nowacek, and D.P. Nowacek. 2011. Community structure and abundance of bottlenose dolphins *Tursiops truncatus* in coastal waters of the northeast Gulf of Mexico. Marine Ecology Progress Series 438:253–265. doi: 10.3354/meps09292

Urian, K.W., S. Hofmann, R.S. Wells, and A.J. Read. 2009. Fine-scale population structure of bottlenose dolphins (*Tursiops truncatus*) in Tampa Bay, Florida. Marine Mammal Science 25(3):619–638. doi: 10.1111/j.1748-7692.2009.00284.x

Van Bressem, M.F., P.J. Duignan, A. Banyard, M. Barbieri, K.M. Colegrove, S. De Guise, G. Di Guardo, A. Dobson, M. Domingo, D. Fauquier, A. Fernandez, T. Goldstein, B. Grenfell, K.R. Groch, F. Gulland, B.A. Jensen, P.D. Jepson, A. Hall, T. Kuiken, S. Mazzariol, S.E. Morris, O. Nielsen, J.A. Raga, T.K. Rowles, J. Saliki, E. Sierra, N. Stephens, B. Stone, I. Tomo, J. Wang, T. Waltzek, and J.F. Wellehan. 2014. Cetacean morbillivirus: current knowledge and future directions. Viruses 6(12):5145–5181. doi: 10.3390/v6125145

Venn-Watson, S., L. Garrison, J. Litz, E. Fougeres, B. Mase, G. Rappucci, E. Stratton, R. Carmichael, D. Odell, D. Shannon, S. Shippee, S. Smith, L. Staggs, M. Tumlin, H. Whitehead, and T. Rowles. 2015. Demographic clusters identified within the northern Gulf of Mexico common bottlenose dolphin (*Tursiops truncates*) unusual mortality event: January 2010-June 2013. PLoS ONE 10(2):e0117248. doi: 10.1371/journal.pone.0117248

Vollmer, N.L. and P.E. Rosel. 2013. A review of common bottlenose dolphins (*Tursiops truncatus truncatus*) in the Northern Gulf of Mexico: Population biology, potential threats, and management. Southeastern Naturalist 12(m6):1–43. doi: 10.1656/058.012.m601

Weller, D.W. 1998. Global and regional variation in the biology and behavior of bottlenose dolphins. Texas A&M University. Dissertation. 142 p.

Wells, R.S. 1986a. Population structure of bottlenose dolphins: Behavioral studies along the central west coast of Florida. Contract Report to National Marine Fisheries Service, Southeast Fisheries Center. Contract No. 45-WCNF-5-003BB. 58 p.

Wells, R.S. 1986b. Structural aspects of dolphin societies. University of California, Santa Cruz. Dissertation. 234 p.

Wells, R.S. 1991. The role of long-term study in understanding the social structure of a bottlenose dolphin community. In: Pryor, K. and K. Norris (Eds.), Dolphin Societies: Discoveries and Puzzles. University of California Press, Berkeley, CA. pp. 199–226.

Wells, R.S. 2000. Reproduction in wild bottlenose dolphins: Overview of patterns observed during a long-term study. In: Duffield, D. and T. Robeck (Eds.), Bottlenose Dolphin Reproduction Workshop. AZA Marine Mammal Taxon Advisory Group, Silver Spring, MD. pp. 57–74.

Wells, R.S. 2003. Dolphin social complexity: Lessons from long-term study and life history. In: de Waal, F.B.M. and P.L. Tyack (Eds.), Animal Social Complexity: Intelligence, Culture, and Individualized Societies Harvard University Press, Cambridge, MA. pp. 32–56.

Wells, R.S. 2014. Social structure and life history of bottlenose dolphins near Sarasota Bay, Florida: Insights from four decades and five generations. Primates and cetaceans. pp. 149–172. doi: 10.1007/978-4-431-54523-1_8

Wells, R.S., M.K. Bassos, K.W. Urian, W.J. Carr, and M.D. Scott. 1996. Low-level monitoring of bottlenose dolphins, *Tursiops truncatus*, in Charlotte Harbor, Florida: 1990-1994. National Oceanic and Atmospheric Administration, National Marine Fisheries Service, Southeast Fisheries Science Center: Miami, FL. NOAA Technical Memorandum NMFS-SEFSC-384. 86 p.

Wells, R.S., M.K. Bassos, K.W. Urian, S.H. Shane, E.C.G. Owen, C.F. Weiss, W.J. Carr, and M.D. Scott. 1997. Low-level monitoring of bottlenose dolphins, *Tursiops truncatus*, in Pine Island Sound, Florida: 1996. National Oceanic and Atmospheric Administration, National Marine Fisheries Service, Southeast Fisheries Science Center: Miami, FL. Southeast Fisheries Center Contribution No. 40-WCNF601958. 86 p.

Wells, R.S., A. Natoli, and G. Braulik. 2019. *Tursiops truncatus* (errata version published in 2019). *The IUCN Red List of Threatened Species* 2019: e.T22563A156932432. https://dx.doi.org/10.2305/IUCN.UK.2019-1.RLTS.T22563A156932432.en

Wells, R.S., L.H. Schwacke, T.K. Rowles, B.C. Balmer, E. Zolman, T. Speakman, F.I. Townsend, M.C. Tumlin, A. Barleycorn, and K.A. Wilkinson. 2017. Ranging patterns of common bottlenose dolphins *Tursiops truncatus* in Barataria Bay, Louisiana, following the Deepwater Horizon oil spill. Endangered Species Research 33:159–180. doi: 10.3354/esr00732

Wells, R.S. and M.D. Scott. 1999. Bottlenose dolphin *Tursiops truncatus* (Montagu, 1821). In: Ridgway, S.H. and R. Harrison (Eds.), Handbook of marine mammals. Volume 6: The second book of dolphins and the porpoises. Academic Press, San Diego, CA. pp. 137–182.

Wells, R.S. and M.D. Scott. 2018. Bottlenose dolphin, *Tursiops truncatus*, common bottlenose dolphin. In: Würsig, B., J.G.M. Thewissen, and K.M. Kovacs (Eds.), Encyclopedia of Marine Mammals (Third Edition). Academic Press, London, UK. pp. 118–125. doi: 10.1016/B978-0-12-804327-1.00072-8

Wells, R.S., M.D. Scott, and A.B. Irvine. 1987. The social structure of free-ranging bottlenose dolphins. In: Genoways, H.H. (Ed.), Current Mammalogy. Plenum Press, New York, NY. pp. 247–305.

Wells, R.S., K.W. Urian, A.J. Read, M.K. Bassos, W.J. Carr, and M.D. Scott. 1996. Low-level monitoring of bottlenose dolphins, *Tursiops truncatus*, in Tampa Bay, Florida: 1988-1993. National Oceanic and Atmospheric Administration, National Marine Fisheries Service, Southeast Fisheries Science Center: Miami, FL. NOAA Technical Memorandum NMFS-SEFSC-385. 36 p.

Wilson, R.M., R.B. Tyson, J.A. Nelson, B.C. Balmer, J.P. Chanton, and D.P. Nowacek. 2017. Niche differentiation and prey selectivity among common bottlenose dolphins (*Tursiops truncatus*) sighted in St. George Sound, Gulf of Mexico. Frontiers in Marine Science 4:235. doi: 10.3389/fmars.2017.00235

Common bottlenose dolphin − *Tursiops truncatus*

GoMx BSE/Mississippi Sound, Lake Borgne, Bay Boudreau Stock

Overall Vulnerability Rank = Very High

Biological Sensitivity = High

Climate Exposure = Very High

Data Quality = 96% of scores ≥ 2

	Tursiops truncatus	Expert Scores	Data Quality	Expert Scores Plots (Portion by Category)
Sensitivity attributes	Prey/Diet Specificity	1.8	2.2	
	Habitat Specificity	3.4	2.5	
	Site Fidelity	3.8	2.8	
	Lifetime Reproductive Potential	1.2	2.0	
	Generation Length	2.8	2.2	
	Reproductive Plasticity	1.9	2.2	
	Migration	3.8	3.0	
	Home Range	3.2	3.0	
	Stock Abundance	2.2	3.0	
	Stock Abundance Trend	2.8	1.8	
	Cumulative Stressors	3.2	2.8	
	Sensitivity Score	High		
Exposure variables	Sea Surface Temperature (Change in mean)	4.0	3.0	
	Sea Surface Temperature (Change in variability)	1.0	3.0	
	Air Temperature (Change in mean)	4.0	3.0	
	Air Temperature (Change in variability)	1.0	3.0	
	Precipitation (Change in mean)	1.0	3.0	
	Precipitation (Change in variability)	1.0	3.0	
	Sea Surface Salinity (Change in mean)	1.8	3.0	
	Sea Surface Salinity (Change in variability)	1.6	3.0	
	Ocean pH (Change in mean)	4.0	3.0	
	Ocean pH (Change in variability)	1.0	3.0	
	Sea ice coverage (Change in mean)	1.0	3.0	
	Sea ice coverage (Change in variability)	1.0	3.0	
	Dissolved oxygen (Change in mean)	4.0	3.0	
	Dissolved oxygen (Change in variability)	1.0	3.0	
	Circulation	3.8	2.5	
	Sea level rise	3.5	3.0	
	Exposure Score	Very High		
Overall Vulnerability Rank		Very High		

Low
Moderate
High
Very High

Common bottlenose dolphin (GoMx BSE/Mississippi Sound, Lake Borgne, Bay Boudreau Stock)

Tursiops truncatus

CVA Results Summary

Overall Climate Vulnerability Rank: Very High (100% certainty from bootstrap analysis).

Climate Exposure: Very High. Six exposure factors scored greater than or equal to 3.5: Air Temperature (Standard anomaly) (4.00), Dissolved oxygen (Standard anomaly) (4.00), Ocean pH (Standard anomaly) (4.00), Sea Surface Temperature (Standard anomaly) (4.00), Circulation (3.80), and Sea level rise (3.50).

Biological Sensitivity: High. Five sensitivity attributes scored greater than 3.0: Migration (3.80), Site Fidelity (3.75), Habitat Specificity (3.35), Cumulative Stressors (3.20), and Home Range (3.20).

Distributional Response: High

Abundance Response: High

Phenology Response: High

Data Quality: 96% of the data quality scores were 2 or greater, 91% of sensitivity attribute data quality scores were 2 or higher.

Background Information

Stock Description

This stock includes common bottlenose dolphins in Mississippi Sound, Lake Borgne, and Bay Boudreau in the northern Gulf of Mexico along the Louisiana, Mississippi, and Alabama coasts. It includes the adjacent Gulf coastal waters extending 1 km from the barrier islands and passes that bound the bays (Hayes et al. 2022).

Prey/Diet Specificity

Generally, common bottlenose dolphins consume a large variety of fish and/or squid (Barros and Odell 1990; Mead and Potter 1990; Wells and Scott 2018). Common bottlenose dolphins seem to show a consistent preference for sound-producing fishes (e.g., sciaenids, toadfish; Berens McCabe et al. 2010; Bowen-Stevens et al. 2021). In Sarasota Bay, Florida, Rossman et al. (2015) found some individual common bottlenose dolphins to show diet specialization despite the broader study group showing a generalist diet. Specialization can be influenced by locally abundant prey species (Shane et al. 1986; Vollmer and Rosel 2013) or can result from resource partitioning amongst sympatric populations (e.g., Torres and Read 2009; Wilson et al. 2017).

Habitat Specificity

Studies in Mississippi Sound have suggested dolphins show a preference for specific habitats, such as mainland coasts, channels, or barrier islands, and environmental conditions, such as temperature and salinity (Hubard et al. 2004; Pitchford et al. 2016a, 2016b; Mullin et al. 2017). A study using satellite-linked telemetry suggested two different patterns of ranging (one around the barrier islands, another in inshore waters with seasonal use of the mid-portion of Mississippi Sound), representing different habitat use (Mullin et al. 2017). Mississippi Sound is an open embayment with large passes between the barrier islands, including two dredged shipping channels (Hayes et al. 2022).

Site Fidelity

In general, photo-ID studies within Mississippi Sound have resulted in low re-sighting rates of dolphins, and many animals sighted only one time (Hubard et al. 2004; Mackey 2010). However, long-term site fidelity has been shown by resightings of animals originally freeze-branded in 1982–1983 that were still present during studies in the 1990s and early 2000s (Hubard et al. 2004; Mackey 2010). Hubard et al. (2004) suggested some animals displayed spatial and/or temporal site fidelity patterns. Year-round residents, seasonal residents, and transients were all reported by Mackey (2010).

Other common bottlenose dolphin Gulf of Mexico bay, sound, and estuary (BSE) stocks show site fidelity (LaBrecque et al. 2015; Hayes et al. 2022), such as in Matagorda Bay, Espiritu Santo Bay, and San Luis Pass in Texas (Maze and Würsig 1999; Lynn and Würsig 2002; Irwin and Würsig 2004); Barataria Bay in Louisiana (Miller 2003; McDonald et al. 2017; Wells et al. 2017; Takeshita et al. 2021); Charlotte Harbor and Pine Island Sound (Bassos-Hull et al. 2013), Sarasota Bay in Florida (Wells 1986a; Wells 1991; Wells 2014), St. Andrew Bay (Balmer et al. 2019), St. Joseph Bay (Balmer et al. 2008; B. Balmer et al. 2018), St. Vincent Sound and Apalachicola Bay (Tyson et al. 2011), and Tampa Bay in Florida (Wells et al. 1996b; Urian et al. 2009).

Lifetime Reproductive Potential

Taylor et al. (2007) reported the interbirth interval of common bottlenose dolphins as 3.80 years, though other studies have shown it to be closer to three years (Leatherwood 1977; Wells and Scott 1999; Connor et al. 2000; Wells 2003).

Generally, females reach sexual maturity before males, with females reaching sexual maturity at 5–13 years (Sergeant et al. 1973; Perrin and Reilly 1984; Wells et al. 1987; Mead and Potter 1990; Mann et al. 2000; Wells 2000; Wells and Scott 2018).

Females reproduce through most of their adult lives (Leatherwood 1977; Hohn et al. 1989; Wells and Scott 1999; Wells 2000; Wells 2003), but may produce fewer calves after they reach 30 years of age (Wells 2000). Sarasota Bay common bottlenose dolphins can give birth when they are as young as 6 years of age, and can continue until they are 48 years old (Wells and Scott 2018). Reproductive lifespan would therefore be up to 42 years based on long-term observation studies (Wells 2014; Wells and Scott 2018).

Longitudinal studies from Sarasota Bay, Florida have found that female common bottlenose dolphins can live up to 67 years (Wells and Scott 2018). Studies based on growth layer groups in teeth suggest

female common bottlenose dolphins can live to 41 years along the Texas coast (Fernandez and Hohn 1998) and to 30 years along the Mississippi coast (Mattson et al. 2006).

Generation Length

Taylor et al. (2007) reported common bottlenose dolphin generation length of 20.6–21.1 years.

Reproductive Plasticity

Common bottlenose dolphin calving is generally diffusely seasonal, with one or more peaks, although births have been reported from all seasons (Vollmer and Rosel 2013; Wells and Scott 2018). Calf sightings peak in the spring and summer months in Florida (Wells et al. 1987), Mississippi/Louisiana (Miller et al. 2010; Miller et al. 2013), and Texas (McHugh 1989; Henderson et al. 2007).

Migration

The BSE stocks in the Gulf of Mexico are thought to primarily comprise stable resident communities. However, transient or seasonal movements of some individuals into and out of a BSE stock area are recorded in some area (LaBrecque et al. 2015; Hayes et al. 2022). Molecular genetic data support that some BSE stocks are genetically distinct from one another, and furthermore are differentiated from adjacent coastal populations (e.g., Sellas et al. 2005; Rosel et al. 2017). For example, Toms (2019) found fine-scale genetic population structure among five inshore systems in the Florida Panhandle and variable migration rates among populations.

During photo-ID studies conducted during 2004–2007 within Mississippi Sound, Mackey (2010) identified 687 dolphins, of which the majority, 498 (73.5%) were classified as transients. A smaller number, 70 (10%), were classified as year-round residents, and 109 (16%) were classified as seasonal residents. Bay, sound, and estuary stocks appear to be genetically distinct from other BSE populations adjacent coastal populations, continental shelf, and oceanic populations (Sellas et al. 2005; Vollmer 2011).

Year-round residency has been reported for common bottlenose dolphins (Hayes et al. 2022), such as in Florida (Irvine and Wells 1972; Irvine et al. 1981; Wells 1986a; Wells 1986b; Wells et al. 1987; Scott et al. 1990; Wells 1991; Wells et al. 1996a; Wells et al. 1996b; Wells et al. 1997; Wells 2003; Balmer et al. 2008; Urian et al. 2009; Tyson et al. 2011; Bassos-Hull et al. 2013; B. Balmer et al. 2018; Balmer et al. 2019; Toms 2019) and Texas (Shane 1990; Bräger 1993; Bräger et al. 1994; Fertl 1994; Weller 1998; Maze and Würsig 1999; Lynn and Würsig 2002; Irwin and Würsig 2004; Shane 2004), in addition to Louisiana/Mississippi (Hubard et al. 2004; Mackey 2010; McDonald et al. 2017; Mullin et al. 2017; Takeshita et al. 2021).

Home Range

The home ranges of individuals within the Gulf of Mexico BSE stocks are considered to generally include the areas within the stock boundaries and brief excursions beyond the stock boundaries (Irvine et al. 1981; Wells 1986a; Wells 1986b; Wells 1991; Wells et al. 1996a; Wells et al. 1996b; Balmer et al. 2008; Wells 2014; Balmer et al. 2019), although some individuals show preference for small areas within the stock boundaries (Lynn and Würsig 2002; Urian et al. 2009; Mullin et al. 2017; Wells et al. 2017; Cloyed

et al. 2021a, 2021b). A study using satellite-linked telemetry suggested two different patterns of ranging within Mississippi Sound: animals that ranged exclusively around the barrier islands, and those that ranged in inshore waters with seasonal use of the mid-portion of the sound (Mullin et al. 2017).

Stock Abundance

Garrison et al. (2021) estimated the abundance of this stock at 1,265 (CV=0.35) from winter 2018 aerial survey data. Previously, Mullin et al. (2017) estimated the abundance of this stock at 3,046 (95% CI: 2,702–3,293; CV=0.06) from January 2012 vessel-based capture-recapture photo-ID surveys.

Stock Abundance Trend

This stock was estimated to experience a 62% decline in population size due to the Deepwater Horizon oil spill (DWH MMIQT 2015; Schwacke et al. 2017). Using photo-identification data from 2011–June 2015, Samuelson et al. (2021) estimated the monthly population growth rate at 1.005 (95% CI: 0.998–1.013).

Cumulative Stressors

Common bottlenose dolphins face a wide range of individual and cumulative natural and anthropogenic threats (see reviews by Vollmer and Rosel 2013; Wells and Scott 2018; and Wells et al. 2019). These include such threats as shark predation, harmful algal blooms, recreational and commercial fishing gear, vessel traffic, pollution, habitat alteration, and provisioning.

Common bottlenose dolphins in Mississippi Sound were exposed to high levels of oil and dispersants following the Deepwater Horizon oil spill, which may result in long term reproductive and chronic health effects (NOAA 2011; Michel et al. 2013; Helm et al. 2015; DWH NRDA 2016; Lane et al. 2015; De Guise et al. 2017, 2021; Kellar et al. 2017; Smith et al. 2017; Schwacke et al. 2017). An Unusual Mortality Event (UME), involving primarily bottlenose dolphins, was declared for cetaceans in the northern Gulf of Mexico from 2010 to 2014, with the Deepwater Horizon oil spill as the primary underlying cause (Litz et al. 2014; Schwacke et al. 2014; Venn-Watson et al. 2015a, 2015b).

The nearshore and estuarine habitats along the Gulf of Mexico coast include waters adjacent to areas of high human population, some of which are highly industrialized, and areas of heavy agricultural impact. Studies of bottlenose dolphin tissues from Mississippi Sound have shown persistent organic pollutant (POP) in blubber and blood samples, though at concentrations lower than other southeastern U.S. sites (Kucklick et al. 2011; Balmer et al. 2015; J. Balmer et al. 2018). Similarly, mercury has been detected in common bottlenose dolphin tissues in Louisiana waters, but in lower concentrations than common bottlenose dolphins found in Florida waters (McCormack et al. 2020a, 2020b, 2022).

Dolphin morbillivirus has been detected in populations of the northern Gulf of Mexico, but has not been attributed to large scale die-offs since 1993−1994 (Lipscomb et al. 1996; Litz et al. 2014; Van Bressem et al. 2014; Fauquier et al. 2017). A UME was declared in 1990 for the Gulf of Mexico from Florida to Texas, with more than 300 common bottlenose dolphins stranding due to an undetermined cause, although morbillivirus and cold temperatures may have contributed (Litz et al. 2014). This stock was also within the range of the 1996 Mississippi UME that was suspected to have been caused by brevetoxin (Litz et al. 2014).

Distribution and Sightings

Sighting maps and spatial density models for this stock are presented by Pitchford et al. (2016a, 2016b).

Further Reading

Hayes, S.A., E. Josephson, K. Maze-Foley, P.E. Rosel, and J. Wallace. 2022. U.S. Atlantic and Gulf of Mexico marine mammal stock assessments 2021. National Oceanic and Atmospheric Administration, National Marine Fisheries Service, Northeast Fisheries Science Center: Woods Hole, MA. NOAA Technical Memorandum NMFS-NE-288. 380 p. doi: 10.25923/6tt7-kc16

Wells, R.S. and M.D. Scott. 2018. Bottlenose Dolphin, *Tursiops truncatus*, Common Bottlenose Dolphin. In: Würsig, B., J.G.M. Thewissen, and K.M. Kovacs (Eds.), Encyclopedia of Marine Mammals (Third Edition). Academic Press, London, UK. pp. 118–125. doi: 10.1016/B978-0-12-804327-1.00072-8

Wells, R.S., A. Natoli, and G. Braulik. 2019. *Tursiops truncatus* (errata version published in 2019). The IUCN Red List of Threatened Species 2019: e.T22563A156932432. https://dx.doi.org/10.2305/IUCN.UK.2019-1.RLTS.T22563A156932432.en

References

Balmer, B., T. McDonald, F. Hornsby, J. Adams, J. Allen, A. Barleycorn, and L. Schwacke. 2018. Long-term trends in a northern Gulf of Mexico common bottlenose dolphin (*Tursiops truncatus*) population in the wake of the Deepwater Horizon oil spill. Journal of Cetacean Research and Management 18:1–9. doi: 10.47536/jcrm.v18i1.409

Balmer, B., S. Watwood, B. Quigley, T. Speakman, K. Barry, K. Mullin, P. Rosel, C. Sinclair, E. Zolman, and L. Schwacke. 2019. Common bottlenose dolphin (*Tursiops truncatus*) abundance and distribution patterns in St. Andrew Bay, Florida, USA. Aquatic Conservation: Marine and Freshwater Ecosystems 29(3):486–498. doi: 10.1002/aqc.3001

Balmer, B.C., R.S. Wells, S.M. Nowacek, D.P. Nowacek, L.H. Schwacke, W.A. McLellan, F.S. Scharf, T.K. Rowles, L.J. Hansen, T.R. Spradlin, and D.A. Pabst. 2008. Seasonal abundance and distribution patterns of common bottlenose dolphins (*Tursiops truncatus*) near St. Joseph Bay, Florida, USA. Journal of Cetacean Research and Management 10(2):157–167.

Balmer, B.C., G.M. Ylitalo, L.E. McGeorge, K.A. Baugh, D. Boyd, K.D. Mullin, P.E. Rosel, C. Sinclair, R.S. Wells, E.S. Zolman, and L.H. Schwacke. 2015. Persistent organic pollutants (POPs) in blubber of common bottlenose dolphins (*Tursiops truncatus*) along the northern Gulf of Mexico coast, USA. Science of the Total Environment 527-528:306–312. doi: 10.1016/j.scitotenv.2015.05.016

Barros, N.B. and D.K. Odell. 1990. Food habits of bottlenose dolphins in the southeastern United States In: Leatherwood, S. and R.R. Reeves (Eds.), The Bottlenose Dolphin. Academic Press, San Diego, CA. pp. 309–328. doi: 10.1016/B978-0-12-440280-5.50020-2

Bassos-Hull, K., R.M. Perrtree, C.C. Shepard, S. Schilling, A.A. Barleycorn, J.B. Allen, B.C. Balmer, W.E. Pine, and R.S. Wells. 2013. Long-term site fidelity and seasonal abundance estimates of common bottlenose dolphins (*Tursiops truncatus*) along the southwest coast of Florida and responses to natural perturbations. Journal of Cetacean Research and Management 13(1):19–30. doi: 10.47536/jcrm.v13i1.551

Berens McCabe, E.J., D.P. Gannon, N.B. Barros, and R.S. Wells. 2010. Prey selection by resident common bottlenose dolphins (*Tursiops truncatus*) in Sarasota Bay, Florida. Marine Biology 157(5):931–942. doi: 10.1007/s00227-009-1371-2

Bowen-Stevens, S.R., D.P. Gannon, R.A. Hazelkorn, G. Lovewell, K.M. Volker, S. Smith, M.C. Tumlin, and J. Litz. 2021. Diet of common bottlenose dolphins, *Tursiops truncatus*, that stranded in and near Barataria Bay, Louisiana, 2010–2012. Southeastern Naturalist 20(1). doi: 10.1656/058.020.0113

Bräger, S. 1993. Diurnal and seasonal behavior patterns of bottlenose dolphins (*Tursiops truncatus*). Marine Mammal Science 9:434–438. doi: 10.1111/j.1748-7692.1993.tb00477.x

Bräger, S., B. Würsig, A. Acevedo, and T. Henningsen. 1994. Association patterns of bottle-nosed dolphins (*Tursiops-truncatus*) in Galveston Bay, Texas. Journal of Mammalogy 75(2):431–437. doi: 10.2307/1382564

Cloyed, C.S., B.C. Balmer, L.H. Schwacke, R.S. Wells, E.J. Berens McCabe, A.A. Barleycorn, J.B. Allen, T.K. Rowles, C.R. Smith, R. Takeshita, F.I. Townsend, M.C. Tumlin, E.S. Zolman, and R.H. Carmichael. 2021a. Interaction between dietary and habitat niche breadth influences cetacean vulnerability to environmental disturbance. Ecosphere 12(9):e03759. doi: 10.1002/ecs2.3759

Cloyed, C.S., R.M. Wilson, B.C. Balmer, A.A. Hohn, L.H. Schwacke, E.S. Zolman, M.C. Tumlin, R.S. Wells, A.A. Barleycorn, J.B. Allen, and R.H. Carmichael. 2021b. Specialization of a mobile, apex predator affects trophic coupling among adjacent habitats. Scientific Reports 11(1):19611. doi: 10.1038/s41598-021-99017-1

Connor, R.C., R.S. Wells, J. Mann, and A.J. Read. 2000. The bottlenose dolphin: Social relationships in a fission-fusion society. In: Mann, J., R. Connor, P. Tyack, and H. Whitehead (Eds.), Cetacean Societies: Field Studies of Dolphins and Whales. University of Chicago Press, Chicago, IL. pp. 91–126.

De Guise, S., M. Levin, E. Gebhard, L. Jasperse, L. Burdett Hart, C.R. Smith, S. Venn-Watson, F. Townsend, R. Wells, B. Balmer, E. Zolman, T. Rowles, and L. Schwacke. 2017. Changes in immune functions in bottlenose dolphins in the northern Gulf of Mexico associated with the Deepwater Horizon oil spill. Endangered Species Research 33:291–303. doi: 10.3354/esr00814

De Guise, S., M. Levin, L. Jasperse, J. Herrman, R.S. Wells, T. Rowles, and L. Schwacke. 2021. Long-term immunological alterations in bottlenose dolphin a decade after the Deepwater Horizon oil spill in the northern Gulf of Mexico: Potential for multigenerational effects. Environmental Toxicology and Chemistry 40(5):1308–1321. doi: 10.1002/etc.4980

Deepwater Horizon Marine Mammai Injury Quantification Team (DWH MMIQT). 2015. Models and analyses for the quantification of injury to Gulf of Mexico cetaceans from the Deepwater Horizon oil spill. 64 p.

Deepwater Horizon Natural Resource Damage Assessment Trustees (DWH NRDA). 2016. Deepwater Horizon oil spill: Final Programmatic Damage Assessment and Restoration Plan and Final Programmatic Environmental Impact Statement. Retrieved from: http://www.gulfspillrestoration.noaa.gov/restoration-planning/gulf-plan.1659.

Fauquier, D.A., J. Litz, S. Sanchez, K. Colegrove, L.H. Schwacke, L. Hart, J. Saliki, C. Smith, T. Goldstein, S. Bowen-Stevens, W. McFee, E. Fougeres, B. Mase-Guthrie, E. Stratton, R. Ewing, S. Venn-Watson, R.H. Carmichael, C. Clemons-Chevis, W. Hatchett, D. Shannon, S. Shippee, S. Smith, L. Staggs, M.C. Tumlin, N.L. Wingers, and T.K. Rowles. 2017. Evaluation of morbillivirus exposure in cetaceans from the northern Gulf of Mexico 2010-2014. Endangered Species Research 33:211–220. doi: 10.3354/esr00772

Fernandez, S. and A.A. Hohn. 1998. Age, growth, and calving season of bottlenose dolphins, *Tursiops truncatus*, off coastal Texas. Fishery Bulletin 96(2):357–365.

Fertl, D. 1994. Occurrence patterns and behavior of bottlenose dolphins (*Tursiops truncatus*) in the Galveston ship channel, Texas. Texas Journal of Science 46(4):299–318.

Garrison, L.P., J. Ortega-Ortiz, and G. Rappucci. 2021. Abundance of coastal and continental shelf stocks of common bottlenose and Atlantic spotted dolphins in the northern Gulf of Mexico: 2017–2018. National Oceanic and Atmospheric Administration, National Marine Fisheries Service,

Southeast Fisheries Science Center, Protected Resources and Biodiversity Division: Miami, FL. PRD-2021-01. 25 p. doi: 10.25923/vk95-t881

Hayes, S.A., E. Josephson, K. Maze-Foley, P.E. Rosel, and J. Wallace. 2022. U.S. Atlantic and Gulf of Mexico marine mammal stock assessments 2021. National Oceanic and Atmospheric Administration, National Marine Fisheries Service, Northeast Fisheries Science Center: Woods Hole, MA. NOAA Technical Memorandum NMFS-NE-288. 380 p. doi: 10.25923/6tt7-kc16

Helm, R.C., D.P. Costa, T.D. DeBruyn, T.J. O'Shea, R.S. Wells, and T.M. Williams. 2015. Overview of effects of oil spills on marine mammals. In: Fingas, M. (Ed.), Handbook of oil spill science and technology, First edition. John Wiley & Sons, Inc., Hoboken, NJ. pp. 455–475.

Henderson, E.E. and B. Würsig. 2007. Behavior patterns of bottlenose dolphins in San Luis Pass, Texas. Gulf of Mexico Science 25(2):153–161. doi: 10.18785/goms.2502.06

Hohn, A.A., M.D. Scott, R.S. Wells, J.C. Sweeney, and A.B. Irvine. 1989. Growth layers in teeth from known-age, free-ranging bottlenose dolphins. Marine Mammal Science 5(4):315–342. doi: 10.1111/j.1748-7692.1989.tb00346.x

Hubard, C.W., K. Maze-Foley, K.D. Mullin, and W.W. Schroeder. 2004. Seasonal abundance and site fidelity of bottlenose dolphins (*Tursiops truncatus*) in Mississippi Sound. Aquatic Mammals 30(2):299–310. doi: 10.1578/am.30.2.2004.299

Irvine, A.B., M.D. Scott, R.S. Wells, and J.H. Kaufmann. 1981. Movements and activities of the Atlantic bottlenose dolphin, *Tursiops truncatus*, near Sarasota, Florida. Fishery Bulletin 79(4):671–688.

Irvine, B. and R.S. Wells. 1972. Results of attempts to tag Atlantic bottlenosed dolphins (*Tursiops truncatus*). Cetology 13:1–5.

Irwin, L.-J. and B. Würsig. 2004. A small resident community of bottlenose dolphins, *Tursiops truncatus*, in Texas: Monitoring recommendations. Gulf of Mexico Science 22(1):13–21. doi: 10.18785/goms.2201.02

Kellar, N.M., T.R. Speakman, C.R. Smith, S.M. Lane, B.C. Balmer, M.L. Trego, K.N. Catelani, M.N. Robbins, C.D. Allen, R.S. Wells, E.S. Zolman, T.K. Rowles, and L.H. Schwacke. 2017. Low reproductive success rates of common bottlenose dolphins *Tursiops truncatus* in the northern Gulf of Mexico following the Deepwater Horizon disaster (2010-2015). Endangered Species Research 33:143–158. doi: 10.3354/esr00775

Kucklick, J., L. Schwacke, R. Wells, A. Hohn, A. Guichard, J. Yordy, L. Hansen, E. Zolman, R. Wilson, J. Litz, D. Nowacek, T. Rowles, R. Pugh, B. Balmer, C. Sinclair, and P. Rosel. 2011. Bottlenose dolphins as indicators of persistent organic pollutants in the western North Atlantic Ocean and Northern Gulf of Mexico. Environmental Science & Technology 45(10):4270–4277. doi: 10.1021/es1042244

LaBrecque, E., C. Curtice, J. Harrison, S.M. Van Parijs, and P.N. Halpin. 2015. 3. Biologically Important Areas for cetaceans within U.S. Waters – Gulf of Mexico Region. Aquatic Mammals 41(1):30–38. doi: 10.1578/am.41.1.2015.30

Lane, S.M., C.R. Smith, J. Mitchell, B.C. Balmer, K.P. Barry, T. McDonald, C.S. Mori, P.E. Rosel, T.K. Rowles, T.R. Speakman, F.I. Townsend, M.C. Tumlin, R.S. Wells, E.S. Zolman, and L.H. Schwacke. 2015. Reproductive outcome and survival of common bottlenose dolphins sampled in Barataria Bay, Louisiana, USA, following the Deepwater Horizon oil spill. Proceedings of the Royal Society B: Biological Sciences 282(1818):20151944. doi: 10.1098/rspb.2015.1944

Leatherwood, S. 1977. Some preliminary impressions on the numbers and social behavior of free-swimming bottlenosed dolphin calves (*Tursiops truncatus*) in the northern Gulf of Mexico. In: Ridgway, S.H. and K. Bernirschke (Eds.), Breeding Dolphins; Present Status, Suggestions for the Future. Final report to the US Marine Mammal Commission, Contract MM6AC009, MMC-76/07, Washington, DC. pp. 143–167.

Lipscomb, T.P., S. Kennedy, D. Moffett, A. Krafft, B.A. Klaunberg, J.H. Lichy, G.T. Regan, G.A.J. Worthy, and J.K. Taubenberger. 1996. Morbilliviral epizootic in bottlenose dolphins of the Gulf of Mexico. Journal of Veterinary Diagnostic Investigation 8(3):283–290. doi: 10.1177/104063879600800302

Litz, J.A., M.A. Baran, S.R. Bowen-Stevens, R.H. Carmichael, K.M. Colegrove, L.P. Garrison, S.E. Fire, E.M. Fougeres, R. Hardy, S. Holmes, W. Jones, B.E. Mase-Guthrie, D.K. Odell, P.E. Rosel, J.T. Saliki, D.K. Shannon, S.F. Shippee, S.M. Smith, E.M. Stratton, M.C. Tumlin, H.R. Whitehead, G.A. Worthy, and T.K. Rowles. 2014. Review of historical unusual mortality events (UMEs) in the Gulf of Mexico (1990–2009): providing context for the multi-year northern Gulf of Mexico cetacean UME declared in 2010. Diseases of Aquatic Organisms 112(2):161–175. doi: 10.3354/dao02807

Lynn, S.K. and B. Würsig. 2002. Summer movement patterns of bottlenose dolphins in a Texas bay. Gulf of Mexico Science 20(1):25–37. doi: 10.18785/goms.2001.03

Mackey, A.D. 2010. Site fidelity and association patterns of bottlenose dolphins (*Tursiops truncatus*) in the Mississippi Sound. University of Southern Mississippi. Thesis. 133 p.

Mann, J., R.C. Connor, L.M. Barre, and M.R. Heithaus. 2000. Female reproductive success in bottlenose dolphins (*Tursiops* sp.): life history, habitat, provisioning, and group-size effects. Behavioral Ecology 11(2):210–219. doi: 10.1093/beheco/11.2.210

Mattson, M.C., K.D. Mullin, G.W. Ingram, and W. Hoggard. 2006. Age structure and growth of the bottlenose dolphin (*Tursiops truncatus*) from strandings in the Mississippi sound region of the north-central Gulf of Mexico from 1986 to 2003. Marine Mammal Science 22(3):654–666. doi: 10.1111/j.1748-7692.2006.00057.x

Maze, K.S. and B. Würsig. 1999. Bottlenosed dolphins of San Luis Pass, Texas: Occurrence patterns, site-fidelity, and habitat use. Aquatic Mammals 25:91–104.

McCormack, M.A., F. Battaglia, W.E. McFee, and J. Dutton. 2020a. Mercury concentrations in blubber and skin from stranded bottlenose dolphins (*Tursiops truncatus*) along the Florida and Louisiana coasts (Gulf of Mexico, USA) in relation to biological variables. Environmental Research 180:108886. doi: 10.1016/j.envres.2019.108886

McCormack, M.A., B.P. Jackson, and J. Dutton. 2020b. Relationship between mercury and selenium concentrations in tissues from stranded odontocetes in the northern Gulf of Mexico. Science of the Total Environment 749:141350. doi: 10.1016/j.scitotenv.2020.141350

McCormack, M.A., W.H. Nowlin, and J. Dutton. 2022. Effect of trophic position on mercury concentrations in bottlenose dolphins (*Tursiops truncatus*) from the northern Gulf of Mexico. Environmental Research 204(Pt B):112124. doi: 10.1016/j.envres.2021.112124

McDonald, T.L., F.E. Hornsby, T.R. Speakman, E.S. Zolman, K.D. Mullin, C. Sinclair, P.E. Rosel, L. Thomas, and L.H. Schwacke. 2017. Survival, density, and abundance of common bottlenose dolphins in Barataria Bay (USA) following the Deepwater Horizon oil spill. Endangered Species Research 33:193–209. doi: 10.3354/esr00806

McHugh, M.B. 1989. Population numbers and feeding behavior of the Atlantic bottlenose dolphin (*Tursiops truncatus*) near Aransas Pass, Texas. University of Texas at Austin. Thesis. 96 p.

Mead, J.G. and C.W. Potter. 1990. Natural history of bottlenose dolphins along the central Atlantic coast of the United States. In: Leatherwood, S. and R.R. Reeves (Eds.), The Bottlenose Dolphin. Academic Press, San Diego, CA. pp. 165–195.

Michel, J., E.H. Owens, S. Zengel, A. Graham, Z. Nixon, T. Allard, W. Holton, P.D. Reimer, A. Lamarche, M. White, N. Rutherford, C. Childs, G. Mauseth, G. Challenger, and E. Taylor. 2013. Extent and degree of shoreline oiling: Deepwater Horizon oil spill, Gulf of Mexico, USA. PLoS ONE 8(6):e65087. doi: 10.1371/journal.pone.0065087

Miller, C.E. 2003. Abundance trends and environmental habitat usage patterns of bottlenose dolphins (*Tursiops truncatus*) in lower Barataria and Caminada bays, Louisiana. Louisiana State University and Agricultural & Mechanical College. Dissertation. 125 p.

Miller, L.J., A.D. Mackey, T. Hoffland, M. Solangi, and S.A. Kuczaj. 2010. Potential effects of a major hurricane on Atlantic bottlenose dolphin (*Tursiops truncatus*) reproduction in the Mississippi Sound. Marine Mammal Science 26(3):707–715. doi: 10.1111/j.1748-7692.2010.00371.x

Miller, L.J., A.D. Mackey, M. Solangi, and S.A. Kuczaj. 2013. Population abundance and habitat utilization of bottlenose dolphins in the Mississippi Sound. Aquatic Conservation: Marine and Freshwater Ecosystems 23(1):145–151. doi: 10.1002/aqc.2278

Mullin, K.D., T. McDonald, R.S. Wells, B.C. Balmer, T. Speakman, C. Sinclair, E.S. Zolman, F. Hornsby, S.M. McBride, K.A. Wilkinson, and L.H. Schwacke. 2017. Density, abundance, survival, and ranging patterns of common bottlenose dolphins (*Tursiops truncatus*) in Mississippi Sound following the Deepwater Horizon oil spill. PLoS ONE 12(10):e0186265. doi: 10.1371/journal.pone.0186265

National Oceanic and Atmospheric Administration (NOAA). 2011. Public scoping for preparation of a programmatic environmental impact statement for the Deepwater Horizon BP Oil Spill. 9 p.

Perrin, W.F. and S.B. Reilly. 1984. Reproductive parameters of dolphins and small whales of the family Delphinidae. In: Perrin, W.F., R.L. Brownell, Jr., and D.P. DeMaster (Eds.), Reproduction in Whales, Dolphins, and Porpoises. pp. 97–134.

Pitchford, J.L., V.A. Howard, J.K. Shelley, B.J.S. Serafin, A.T. Coleman, and M. Solangi. 2016. Predictive spatial modelling of seasonal bottlenose dolphin (*Tursiops truncatus*) distributions in the Mississippi Sound. Aquatic Conservation: Marine and Freshwater Ecosystems 26(2):289–306. doi: 10.1002/aqc.2547

Pitchford, J.L., E.E. Pulis, K. Evans, J.K. Shelley, B.J.S. Serafin, and M. Solangi. 2016. Seasonal density estimates of *Tursiops truncatus* (bottlenose dolphin) in the Mississippi Sound from 2011 to 2013. Southeastern Naturalist 15(2):188–206. doi: 10.1656/058.015.0201

Rosel, P.E., L.A. Wilcox, C. Sinclair, T.R. Speakman, M.C. Tumlin, J.A. Litz, and E.S. Zolman. 2017. Genetic assignment to stock of stranded common bottlenose dolphins in southeastern Louisiana after the Deepwater Horizon oil spill. Endangered Species Research 33:221–234. doi: 10.3354/esr00780

Rossman, S., P.H. Ostrom, M. Stolen, N.B. Barros, H. Gandhi, C.A. Stricker, and R.S. Wells. 2015. Individual specialization in the foraging habits of female bottlenose dolphins living in a trophically diverse and habitat rich estuary. Oecologia 178(2):415–425. doi: 10.1007/s00442-015-3241-6

Samuelson, M.M., M. Fujiwara, E.E. Pulis, J. Pitchford, V.A. Howard, and M. Solangi. 2021. Comprehensive evaluation of survival and population growth for common bottlenose dolphins (*Tursiops truncatus*) in the Mississippi Sound, USA, following the Deepwater Horizon oil spill. Southeastern Naturalist 20(1):178–191. doi: 10.1656/058.020.0121

Schwacke, L.H., C.R. Smith, F.I. Townsend, R.S. Wells, L.B. Hart, B.C. Balmer, T.K. Collier, S. De Guise, M.M. Fry, L.J. Guillette, Jr., S.V. Lamb, S.M. Lane, W.E. McFee, N.J. Place, M.C. Tumlin, G.M. Ylitalo, E.S. Zolman, and T.K. Rowles. 2014. Health of common bottlenose dolphins (*Tursiops truncatus*) in Barataria Bay, Louisiana, following the deepwater horizon oil spill. Environmental Science & Technology 48(1):93–103. doi: 10.1021/es403610f

Schwacke, L.H., L. Thomas, R.S. Wells, W.E. McFee, A.A. Hohn, K.D. Mullin, E.S. Zolman, B.M. Quigley, T.K. Rowles, and J.H. Schwacke. 2017. Quantifying injury to common bottlenose dolphins from the Deepwater Horizon oil spill using an age-, sex- and class-structured population model. Endangered Species Research 33:265–279. doi: 10.3354/esr00777

Scott, M.D. and S.J. Chivers. 1990. Distribution and herd structure of bottlenose dolphins in the eastern tropical Pacific Ocean. In: Leatherwood, S. and R.R. Reeves (Eds.), The Bottlenose Dolphin. Academic Press, San Diego, CA. pp. 387–402.

Sellas, A.B., R.S. Wells, and P.E. Rosel. 2005. Mitochondrial and nuclear DNA analyses reveal fine scale geographic structure in bottlenose dolphins (*Tursiops truncatus*) in the Gulf of Mexico. Conservation Genetics 6(5):715–728. doi: 10.1007/s10592-005-9031-7

Sergeant, D.E., D.K. Caldwell, and M.C. Caldwell. 1973. Age, growth, and maturity of bottlenosed dolphin (*Tursiops-truncatus*) from northeast Florida. Journal of the Fisheries Research Board of Canada 30(7):1009–1011. doi: 10.1139/f73-165

Shane, S.H. 1990. Behavior and ecology of the bottlenose dolphin at Sanibel Island, Florida. In: Leatherwood, S. and R.R. Reeves (Eds.), The Bottlenose Dolphin. Academic Press, San Diego, CA. pp. 245–265. doi: 10.1016/B978-0-12-440280-5.50016-0

Shane, S.H. 2004. Residence patterns, group characteristics, and association patterns of bottlenose dolphins near Sanibel Island, Florida. Gulf of Mexico Science 22(1):1–12. doi: 10.18785/goms.2201.01

Shane, S.H., R.S. Wells, and B. Würsig. 1986. Ecology, behavior and social organization of the bottlenose dolphin: A review. Marine Mammal Science 2:34–63. doi: 10.1111/j.1748-7692.1986.tb00026.x

Smith, C.R., T.K. Rowles, L.B. Hart, F.I. Townsend, R.S. Wells, E.S. Zolman, B.C. Balmer, B. Quigley, M. Ivančić, W. McKercher, M.C. Tumlin, K.D. Mullin, J.D. Adams, Q. Wu, W. McFee, T.K. Collier, and L.H. Schwacke. 2017. Slow recovery of Barataria Bay dolphin health following the Deepwater Horizon oil spill (2013-2014), with evidence of persistent lung disease and impaired stress response. Endangered Species Research 33:127–142. doi: 10.3354/esr00778

Takeshita, R., B.C. Balmer, F. Messina, E.S. Zolman, L. Thomas, R.S. Wells, C.R. Smith, T.K. Rowles, and L.H. Schwacke. 2021. High site-fidelity in common bottlenose dolphins despite low salinity exposure and associated indicators of compromised health. PLoS ONE 16(9):e0258031. doi: 10.1371/journal.pone.0258031

Taylor, B.L., S.J. Chivers, J. Larese, and W.F. Perrin. 2007. Generation length and percent mature estimates for IUCN assessments of cetaceans. National Oceanic and Atmospheric Administration, National Marine Fisheries Service, Southwest Fisheries Science Center: La Jolla, CA. Administrative Report LJ-07-01. 24 p.

Toms, C. 2019. Filling the gaps: Common bottlenose dolphin (*Tursiops truncatus*) population dynamics, structure, and connectivity within Florida panhandle bays, sounds, and estuaries. University of Central Florida. Dissertation. 373 p.

Torres, L.G. and A.J. Read. 2009. Where to catch a fish? The influence of foraging tactics on the ecology of bottlenose dolphins (*Tursiops truncatus*) in Florida Bay, Florida. Marine Mammal Science 25(4):797–815. doi: 10.1111/j.1748-7692.2009.00297.x

Tyson, R.B., S.M. Nowacek, and D.P. Nowacek. 2011. Community structure and abundance of bottlenose dolphins *Tursiops truncatus* in coastal waters of the northeast Gulf of Mexico. Marine Ecology Progress Series 438:253–265. doi: 10.3354/meps09292

Urian, K.W., S. Hofmann, R.S. Wells, and A.J. Read. 2009. Fine-scale population structure of bottlenose dolphins (*Tursiops truncatus*) in Tampa Bay, Florida. Marine Mammal Science 25(3):619–638. doi: 10.1111/j.1748-7692.2009.00284.x

Van Bressem, M.F., P.J. Duignan, A. Banyard, M. Barbieri, K.M. Colegrove, S. De Guise, G. Di Guardo, A. Dobson, M. Domingo, D. Fauquier, A. Fernandez, T. Goldstein, B. Grenfell, K.R. Groch, F. Gulland, B.A. Jensen, P.D. Jepson, A. Hall, T. Kuiken, S. Mazzariol, S.E. Morris, O. Nielsen, J.A. Raga, T.K. Rowles, J. Saliki, E. Sierra, N. Stephens, B. Stone, I. Tomo, J. Wang, T. Waltzek, and J.F. Wellehan. 2014. Cetacean morbillivirus: current knowledge and future directions. Viruses 6(12):5145–5181. doi: 10.3390/v6125145

Venn-Watson, S., L. Garrison, J. Litz, E. Fougeres, B. Mase, G. Rappucci, E. Stratton, R. Carmichael, D. Odell, D. Shannon, S. Shippee, S. Smith, L. Staggs, M. Tumlin, H. Whitehead, and T. Rowles. 2015. Demographic clusters identified within the northern Gulf of Mexico common bottlenose dolphin

(*Tursiops truncates*) unusual mortality event: January 2010-June 2013. PLoS ONE 10(2):e0117248. doi: 10.1371/journal.pone.0117248

Vollmer, N.L. 2011. Population structure of common bottlenose dolphins in coastal and offshore waters of the Gulf of Mexico revealed by genetic and environmental analysis. University of Louisiana at Lafayette. Dissertation. 409 p.

Vollmer, N.L. and P.E. Rosel. 2013. A review of common bottlenose dolphins (*Tursiops truncatus truncatus*) in the Northern Gulf of Mexico: Population biology, potential threats, and management. Southeastern Naturalist 12(m6):1–43. doi: 10.1656/058.012.m601

Weller, D.W. 1998. Global and regional variation in the biology and behavior of bottlenose dolphins. Texas A&M University. Dissertation. 142 p.

Wells, R.S. 1986a. Population structure of bottlenose dolphins: Behavioral studies along the central west coast of Florida. Contract Report to National Marine Fisheries Service, Southeast Fisheries Center. Contract No. 45-WCNF-5-003BB. 58 p.

Wells, R.S. 1986b. Structural aspects of dolphin societies. University of California, Santa Cruz. Dissertation. 234 p.

Wells, R.S. 1991. The role of long-term study in understanding the social structure of a bottlenose dolphin community. In: Pryor, K. and K. Norris (Eds.), Dolphin Societies: Discoveries and Puzzles. University of California Press, Berkeley, CA. pp. 199–226.

Wells, R.S. 2000. Reproduction in wild bottlenose dolphins: Overview of patterns observed during a long-term study. In: Duffield, D. and T. Robeck (Eds.), Bottlenose Dolphin Reproduction Workshop. AZA Marine Mammal Taxon Advisory Group, Silver Spring, MD. pp. 57–74.

Wells, R.S. 2003. Dolphin social complexity: Lessons from long-term study and life history. In: de Waal, F.B.M. and P.L. Tyack (Eds.), Animal Social Complexity: Intelligence, Culture, and Individualized Societies Harvard University Press, Cambridge, MA. pp. 32–56.

Wells, R.S. 2014. Social structure and life history of bottlenose dolphins near Sarasota Bay, Florida: Insights from four decades and five generations. Primates and cetaceans. pp. 149–172. doi: 10.1007/978-4-431-54523-1_8

Wells, R.S., M.K. Bassos, K.W. Urian, W.J. Carr, and M.D. Scott. 1996. Low-level monitoring of bottlenose dolphins, *Tursiops truncatus*, in Charlotte Harbor, Florida: 1990-1994. National Oceanic and Atmospheric Administration, National Marine Fisheries Service, Southeast Fisheries Science Center: Miami, FL. NOAA Technical Memorandum NMFS-SEFSC-384. 86 p.

Wells, R.S., M.K. Bassos, K.W. Urian, S.H. Shane, E.C.G. Owen, C.F. Weiss, W.J. Carr, and M.D. Scott. 1997. Low-level monitoring of bottlenose dolphins, *Tursiops truncatus*, in Pine Island Sound, Florida: 1996. National Oceanic and Atmospheric Administration, National Marine Fisheries Service, Southeast Fisheries Science Center: Miami, FL. Southeast Fisheries Center Contribution No. 40-WCNF601958. 86 p.

Wells, R.S., A. Natoli, and G. Braulik. 2019. *Tursiops truncatus* (errata version published in 2019). *The IUCN Red List of Threatened Species* 2019: e.T22563A156932432. https://dx.doi.org/10.2305/IUCN.UK.2019-1.RLTS.T22563A156932432.en

Wells, R.S., L.H. Schwacke, T.K. Rowles, B.C. Balmer, E. Zolman, T. Speakman, F.I. Townsend, M.C. Tumlin, A. Barleycorn, and K.A. Wilkinson. 2017. Ranging patterns of common bottlenose dolphins *Tursiops truncatus* in Barataria Bay, Louisiana, following the Deepwater Horizon oil spill. Endangered Species Research 33:159–180. doi: 10.3354/esr00732

Wells, R.S. and M.D. Scott. 1999. Bottlenose dolphin *Tursiops truncatus* (Montagu, 1821). In: Ridgway, S.H. and R. Harrison (Eds.), Handbook of marine mammals. Volume 6: The second book of dolphins and the porpoises. Academic Press, San Diego, CA. pp. 137–182.

Wells, R.S. and M.D. Scott. 2018. Bottlenose dolphin, *Tursiops truncatus*, common bottlenose dolphin. In: Würsig, B., J.G.M. Thewissen, and K.M. Kovacs (Eds.), Encyclopedia of Marine Mammals

(Third Edition). Academic Press, London, UK. pp. 118–125. doi: 10.1016/B978-0-12-804327-1.00072-8

Wells, R.S., M.D. Scott, and A.B. Irvine. 1987. The social structure of free-ranging bottlenose dolphins. In: Genoways, H.H. (Ed.), Current Mammalogy. Plenum Press, New York, NY. pp. 247–305.

Wells, R.S., K.W. Urian, A.J. Read, M.K. Bassos, W.J. Carr, and M.D. Scott. 1996. Low-level monitoring of bottlenose dolphins, *Tursiops truncatus*, in Tampa Bay, Florida: 1988-1993. National Oceanic and Atmospheric Administration, National Marine Fisheries Service, Southeast Fisheries Science Center: Miami, FL. NOAA Technical Memorandum NMFS-SEFSC-385. 36 p.

Wilson, R.M., R.B. Tyson, J.A. Nelson, B.C. Balmer, J.P. Chanton, and D.P. Nowacek. 2017. Niche differentiation and prey selectivity among common bottlenose dolphins (*Tursiops truncatus*) sighted in St. George Sound, Gulf of Mexico. Frontiers in Marine Science 4:235. doi: 10.3389/fmars.2017.00235

Common bottlenose dolphin − *Tursiops truncatus*

GoMx BSE/Mobile Bay, Bonsecour Bay Stock

Overall Vulnerability Rank = Very High

Biological Sensitivity = High

Climate Exposure = Very High

Data Quality = 93% of scores ≥ 2

	Tursiops truncatus	Expert Scores	Data Quality	Expert Scores Plots (Portion by Category)
Sensitivity attributes	Prey/Diet Specificity	1.9	2.2	
	Habitat Specificity	3.4	2.0	
	Site Fidelity	3.8	2.0	
	Lifetime Reproductive Potential	1.1	2.2	
	Generation Length	2.6	2.2	
	Reproductive Plasticity	1.7	1.8	
	Migration	3.8	2.2	
	Home Range	3.4	2.2	
	Stock Abundance	3.1	2.2	
	Stock Abundance Trend	2.2	1.2	
	Cumulative Stressors	2.8	2.0	
	Sensitivity Score	High		
Exposure variables	Sea Surface Temperature (Change in mean)	4.0	2.8	
	Sea Surface Temperature (Change in variability)	1.0	2.8	
	Air Temperature (Change in mean)	4.0	2.8	
	Air Temperature (Change in variability)	1.0	2.8	
	Precipitation (Change in mean)	1.0	2.8	
	Precipitation (Change in variability)	1.0	2.8	
	Sea Surface Salinity (Change in mean)	2.0	2.8	
	Sea Surface Salinity (Change in variability)	1.0	2.8	
	Ocean pH (Change in mean)	4.0	2.8	
	Ocean pH (Change in variability)	1.0	2.8	
	Sea ice coverage (Change in mean)	1.0	3.0	
	Sea ice coverage (Change in variability)	1.0	3.0	
	Dissolved oxygen (Change in mean)	4.0	2.8	
	Dissolved oxygen (Change in variability)	1.0	2.8	
	Circulation	3.7	2.2	
	Sea level rise	4.0	2.8	
	Exposure Score	Very High		
Overall Vulnerability Rank		Very High		

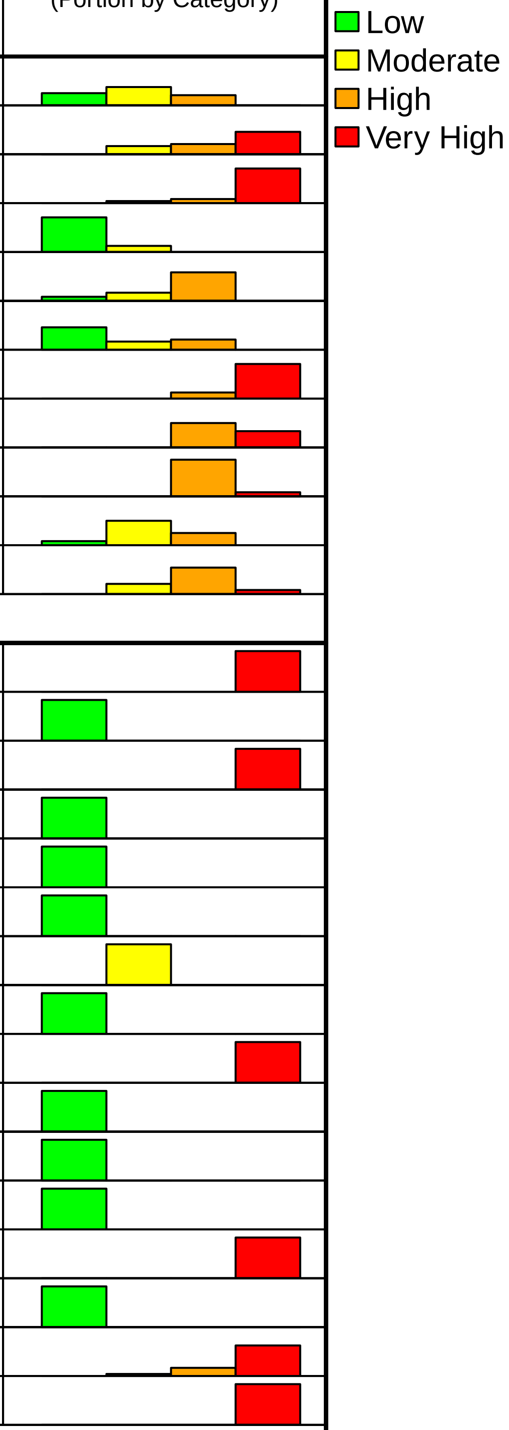

Common bottlenose dolphin (GoMx BSE/Mobile Bay, Bonsecour Bay Stock)

Tursiops truncatus

CVA Results Summary

Overall Climate Vulnerability Rank: Very High (100% certainty from bootstrap analysis).

Climate Exposure: Very High. Four exposure factors scored greater than 3.5: Air Temperature (Standard anomaly) (4.0), Dissolved oxygen (Standard anomaly) (4.0), Ocean pH (Standard anomaly) (4.0), Sea level rise (4.0), Sea Surface Temperature (Standard anomaly) (4.0), and Circulation (3.7).

Biological Sensitivity: High. Five sensitivity attributes scored greater than 3.0: Migration (3.85), Site Fidelity (3.80), Home Range (3.40), Habitat Specificity (3.35), and Species Abundance (3.10).

Distributional Response: High

Abundance Response: High

Phenology Response: High

Data Quality: 93% of the data quality scores were 2 or greater, 82% of sensitivity attribute data quality scores were 2 or higher.

Background Information

Stock Description

This stock includes common bottlenose dolphins in Mobile Bay and Bonsecour Bay in the northern Gulf of Mexico along the Alabama coast (Hayes et al. 2022).

Prey/Diet Specificity

Generally, common bottlenose dolphins consume a large variety of fish and/or squid (Barros and Odell 1990; Mead and Potter 1990; Wells and Scott 2018). Common bottlenose dolphins seem to show a consistent preference for sound-producing fishes (e.g., sciaenids, toadfish; Berens McCabe et al. 2010; Bowen-Stevens et al. 2021). In Sarasota Bay, FL, Rossman et al. (2015) found some individual common bottlenose dolphins to show diet specialization despite the broader study group showing a generalist diet. Specialization can be influenced by locally abundant prey species (Shane et al. 1986; Vollmer and Rosel 2013) or can result from resource partitioning amongst sympatric populations (e.g., Torres and Read 2009; Wilson et al. 2017).

Habitat Specificity

Common bottlenose dolphins are found in a variety of marine and estuarine habitats (Wells and Scott 2018).

Site Fidelity

No specific site fidelity information was found for this stock. However, common bottlenose dolphins in other Gulf of Mexico bay, sound, and estuary (BSE) stocks show site fidelity (LaBrecque et al. 2015; Hayes et al. 2022), such as in Matagorda Bay, Espiritu Santo Bay, and San Luis Pass in Texas (Maze and Würsig 1999; Lynn and Würsig 2002; Irwin and Würsig 2004); Barataria Bay in Louisiana (Miller 2003; McDonald et al. 2017; Wells et al. 2017; Takeshita et al. 2021); Mississippi Sound in Mississippi (Hubard et al. 2004; Mackey 2010; Mullin et al. 2017); Charlotte Harbor and Pine Island Sound (Bassos-Hull et al. 2013), Sarasota Bay in Florida (Wells 1986a; Wells 1991; Wells 2014), St. Andrew Bay (Balmer et al. 2019), St. Joseph Bay (Balmer et al. 2008; B. Balmer et al. 2018), St. Vincent Sound and Apalachicola Bay (Tyson et al. 2011), and Tampa Bay in Florida (Wells et al. 1996b; Urian et al. 2009).

Lifetime Reproductive Potential

Taylor et al. (2007) reported the interbirth interval of common bottlenose dolphins as 3.80 years, though other studies have shown it to be closer to three years (Leatherwood 1977; Wells and Scott 1999; Connor et al. 2000; Wells 2003).

Generally, females reach sexual maturity before males, with females reaching sexual maturity at 5–13 years (Sergeant et al. 1973; Perrin and Reilly 1984; Wells et al. 1987; Mead and Potter 1990; Mann et al. 2000; Wells 2000; Wells and Scott 2018).

Females reproduce through most of their adult lives (Leatherwood 1977; Hohn et al. 1989; Wells and Scott 1999; Wells 2000; Wells 2003), but may produce fewer calves after they reach 30 years of age (Wells 2000). Sarasota Bay common bottlenose dolphins can give birth when they are as young as 6 years of age, and can continue until they are 48 years old (Wells and Scott 2018). Reproductive lifespan would therefore be up to 42 years based on long-term observation studies (Wells 2014; Wells and Scott 2018).

Longitudinal studies from Sarasota Bay, Florida have found that female common bottlenose dolphins can live up to 67 years (Wells and Scott 2018). Studies based on growth layer groups in teeth suggest female common bottlenose dolphins can live to 41 years along the Texas coast (Fernandez and Hohn 1998) and to 30 years along the Mississippi coast (Mattson et al. 2006).

Generation Length

Taylor et al. (2007) reported common bottlenose dolphin generation length of 20.6–21.1 years.

Reproductive Plasticity

Common bottlenose dolphin calving is generally diffusely seasonal, with one or more peaks, although births have been reported from all seasons (Vollmer and Rosel 2013; Wells and Scott 2018). Calf sightings peak in the spring and summer months in Florida (Wells et al. 1987), Mississippi/Louisiana (Miller et al. 2010; Miller et al. 2013), and Texas (McHugh 1989; Henderson et al. 2007).

Migration

The BSE stocks in the Gulf of Mexico are thought to primarily comprise stable resident communities. However, transient or seasonal movements of some individuals into and out of a BSE stock area are recorded in some areas (LaBrecque et al. 2015; Hayes et al. 2022). Molecular genetic data support that some BSE stocks are genetically distinct from one another, and furthermore are differentiated from adjacent coastal populations (e.g., Sellas et al. 2005; Rosel et al. 2017). For example, Toms (2019) found fine-scale genetic population structure among five inshore systems in the Florida Panhandle and variable migration rates among populations.

Year-round residency has been reported for common bottlenose dolphins (Hayes et al. 2022), such as in Florida (Irvine and Wells 1972; Irvine et al. 1981; Wells 1986a; Wells 1986b; Wells et al. 1987; Scott et al. 1990; Wells 1991; Wells et al. 1996a; Wells et al. 1996b; Wells et al. 1997; Wells 2003; Balmer et al. 2008; Urian et al. 2009; Tyson et al. 2011; Bassos-Hull et al. 2013; B. Balmer et al. 2018; Balmer et al. 2019; Toms 2019), Texas (Shane 1990; Bräger 1993; Bräger et al. 1994; Fertl 1994; Weller 1998; Maze and Würsig 1999; Lynn and Würsig 2002; Irwin and Würsig 2004; Shane 2004), and Louisiana/Mississippi (Hubard et al. 2004; McDonald et al. 2017; Mullin et al. 2017; Takeshita et al. 2021).

Home Range

The home ranges of individuals within the Gulf of Mexico BSE stocks are considered to generally include the areas within the stock boundaries and brief excursions beyond the stock boundaries (Irvine et al. 1981; Wells 1986a; Wells 1986b; Wells 1991; Wells et al. 1996a; Wells et al. 1996b; Balmer et al. 2008; Wells 2014; Balmer et al. 2019), although some individuals show preference for small areas within the stock boundaries (Lynn and Würsig 2002; Urian et al. 2009; Mullin et al. 2017; Wells et al. 2017; Cloyed et al. 2021a, 2021b).

Stock Abundance

Blaylock and Hoggard (1994) estimated this stock's abundance at 122 individuals (CV= 0.34). More recent estimates are unavailable (Hayes et al. 2022).

Stock Abundance Trend

Data are insufficient to complete an abundance trend analysis for this stock (Hayes et al. 2022).

Cumulative Stressors

Common bottlenose dolphins face a wide range of individual and cumulative natural and anthropogenic threats (see reviews by Vollmer and Rosel 2013; Wells and Scott 2018; and Wells et al. 2019). These include such threats as shark predation, harmful algal blooms, recreational and commercial fishing gear, vessel traffic, pollution, habitat alteration, and provisioning.

Common bottlenose dolphins in Mobile Bay were possibly exposed to oil and dispersants following the Deepwater Horizon oil spill, which may result in long term reproductive and chronic health effects (NOAA 2011; Michel et al. 2013; Helm et al. 2015; DWH NRDA 2016; Lane et al. 2015; De Guise et al. 2017, 2021; Kellar et al. 2017; Smith et al. 2017; Schwacke et al. 2017). An Unusual Mortality Event (UME), involving primarily bottlenose dolphins, was declared for cetaceans in the northern Gulf of

Mexico from 2010 to 2014, with the Deepwater Horizon oil spill as the primary underlying cause (Litz et al. 2014; Schwacke et al. 2014; Venn-Watson et al. 2015a, b).

The nearshore and estuarine habitats along the Gulf of Mexico coast include waters adjacent to areas of high human population, some of which are highly industrialized, and areas of heavy agricultural impact. Studies of bottlenose dolphin tissues from the northern Gulf of Mexico have shown persistent organic pollutant (POP) in blubber and blood samples, though at concentrations lower than other southeastern U.S. sites (Kucklick et al. 2011; Balmer et al. 2015; J. Balmer et al. 2018).

Dolphin morbillivirus has been detected in populations of the northern Gulf of Mexico, but has not been attributed to large scale die-offs since 1993−1994 (Lipscomb et al. 1996; Litz et al. 2014; Van Bressem et al. 2014; Fauquier et al. 2017). A UME was declared in 1990 for the Gulf of Mexico from Florida to Texas, with more than 300 common bottlenose dolphins stranding due to an undetermined cause, although morbillivirus and cold temperatures may have contributed (Litz et al. 2014).

Further Reading

Hayes, S.A., E. Josephson, K. Maze-Foley, P.E. Rosel, and J. Wallace. 2022. U.S. Atlantic and Gulf of Mexico marine mammal stock assessments 2021. National Oceanic and Atmospheric Administration, National Marine Fisheries Service, Northeast Fisheries Science Center: Woods Hole, MA. NOAA Technical Memorandum NMFS-NE-288. 380 p. doi: 10.25923/6tt7-kc16

Wells, R.S. and M.D. Scott. 2018. Bottlenose Dolphin, *Tursiops truncatus*, Common Bottlenose Dolphin. In: Würsig, B., J.G.M. Thewissen, and K.M. Kovacs (Eds.), Encyclopedia of Marine Mammals (Third Edition). Academic Press, London, UK. pp. 118–125. doi: 10.1016/B978-0-12-804327-1.00072-8

Wells, R.S., A. Natoli, and G. Braulik. 2019. *Tursiops truncatus* (errata version published in 2019). The IUCN Red List of Threatened Species 2019: e.T22563A156932432. https://dx.doi.org/10.2305/IUCN.UK.2019-1.RLTS.T22563A156932432.en

References

Balmer, B., T. McDonald, F. Hornsby, J. Adams, J. Allen, A. Barleycorn, and L. Schwacke. 2018. Long-term trends in a northern Gulf of Mexico common bottlenose dolphin (*Tursiops truncatus*) population in the wake of the Deepwater Horizon oil spill. Journal of Cetacean Research and Management 18:1–9. doi: 10.47536/jcrm.v18i1.409

Balmer, B., S. Watwood, B. Quigley, T. Speakman, K. Barry, K. Mullin, P. Rosel, C. Sinclair, E. Zolman, and L. Schwacke. 2019. Common bottlenose dolphin (*Tursiops truncatus*) abundance and distribution patterns in St. Andrew Bay, Florida, USA. Aquatic Conservation: Marine and Freshwater Ecosystems 29(3):486–498. doi: 10.1002/aqc.3001

Balmer, B.C., R.S. Wells, S.M. Nowacek, D.P. Nowacek, L.H. Schwacke, W.A. McLellan, F.S. Scharf, T.K. Rowles, L.J. Hansen, T.R. Spradlin, and D.A. Pabst. 2008. Seasonal abundance and distribution patterns of common bottlenose dolphins (*Tursiops truncatus*) near St. Joseph Bay, Florida, USA. Journal of Cetacean Research and Management 10(2):157–167.

Balmer, B.C., G.M. Ylitalo, L.E. McGeorge, K.A. Baugh, D. Boyd, K.D. Mullin, P.E. Rosel, C. Sinclair, R.S. Wells, E.S. Zolman, and L.H. Schwacke. 2015. Persistent organic pollutants (POPs) in blubber of common bottlenose dolphins (*Tursiops truncatus*) along the northern Gulf of Mexico coast, USA. Science of the Total Environment 527-528:306–312. doi: 10.1016/j.scitotenv.2015.05.016

Balmer, J.E., G.M. Ylitalo, T.K. Rowles, K.D. Mullin, R.S. Wells, F.I. Townsend, R.W. Pearce, J.L. Bolton, E.S. Zolman, B.C. Balmer, and L.H. Schwacke. 2018. Persistent organic pollutants (POPs) in blood and blubber of common bottlenose dolphins (*Tursiops truncatus*) at three northern Gulf of Mexico sites following the Deepwater Horizon oil spill. Science of the Total Environment 621:130–137. doi: 10.1016/j.scitotenv.2017.11.209

Barros, N.B. and D.K. Odell. 1990. Food habits of bottlenose dolphins in the southeastern United States In: Leatherwood, S. and R.R. Reeves (Eds.), The Bottlenose Dolphin. Academic Press, San Diego, CA. pp. 309–328. doi: 10.1016/B978-0-12-440280-5.50020-2

Bassos-Hull, K., R.M. Perrtree, C.C. Shepard, S. Schilling, A.A. Barleycorn, J.B. Allen, B.C. Balmer, W.E. Pine, and R.S. Wells. 2013. Long-term site fidelity and seasonal abundance estimates of common bottlenose dolphins (*Tursiops truncatus*) along the southwest coast of Florida and responses to natural perturbations. Journal of Cetacean Research and Management 13(1):19–30. doi: 10.47536/jcrm.v13i1.551

Berens McCabe, E.J., D.P. Gannon, N.B. Barros, and R.S. Wells. 2010. Prey selection by resident common bottlenose dolphins (*Tursiops truncatus*) in Sarasota Bay, Florida. Marine Biology 157(5):931–942. doi: 10.1007/s00227-009-1371-2

Blaylock, R.A. and W. Hoggard. 1994. Preliminary estimates of bottlenose dolphin abundance in southern U.S. Atlantic and Gulf of Mexico continental shelf waters. National Oceanic and Atmospheric Administration, National Marine Fisheries Service, Southeast Fisheries Science Center: Miami, FL. NOAA Technical Memorandum NMFS-SEFSC-356. 10 p.

Bowen-Stevens, S.R., D.P. Gannon, R.A. Hazelkorn, G. Lovewell, K.M. Volker, S. Smith, M.C. Tumlin, and J. Litz. 2021. Diet of common bottlenose dolphins, *Tursiops truncatus*, that stranded in and near Barataria Bay, Louisiana, 2010–2012. Southeastern Naturalist 20(1). doi: 10.1656/058.020.0113

Bräger, S. 1993. Diurnal and seasonal behavior patterns of bottlenose dolphins (*Tursiops truncatus*). Marine Mammal Science 9:434–438. doi: 10.1111/j.1748-7692.1993.tb00477.x

Bräger, S., B. Würsig, A. Acevedo, and T. Henningsen. 1994. Association patterns of bottle-nosed dolphins (*Tursiops-truncatus*) in Galveston Bay, Texas. Journal of Mammalogy 75(2):431–437. doi: 10.2307/1382564

Cloyed, C.S., B.C. Balmer, L.H. Schwacke, R.S. Wells, E.J. Berens McCabe, A.A. Barleycorn, J.B. Allen, T.K. Rowles, C.R. Smith, R. Takeshita, F.I. Townsend, M.C. Tumlin, E.S. Zolman, and R.H. Carmichael. 2021a. Interaction between dietary and habitat niche breadth influences cetacean vulnerability to environmental disturbance. Ecosphere 12(9):e03759. doi: 10.1002/ecs2.3759

Cloyed, C.S., R.M. Wilson, B.C. Balmer, A.A. Hohn, L.H. Schwacke, E.S. Zolman, M.C. Tumlin, R.S. Wells, A.A. Barleycorn, J.B. Allen, and R.H. Carmichael. 2021b. Specialization of a mobile, apex predator affects trophic coupling among adjacent habitats. Scientific Reports 11(1):19611. doi: 10.1038/s41598-021-99017-1

Connor, R.C., R.S. Wells, J. Mann, and A.J. Read. 2000. The bottlenose dolphin: Social relationships in a fission-fusion society. In: Mann, J., R. Connor, P. Tyack, and H. Whitehead (Eds.), Cetacean Societies: Field Studies of Dolphins and Whales. University of Chicago Press, Chicago, IL. pp. 91–126.

De Guise, S., M. Levin, E. Gebhard, L. Jasperse, L. Burdett Hart, C.R. Smith, S. Venn-Watson, F. Townsend, R. Wells, B. Balmer, E. Zolman, T. Rowles, and L. Schwacke. 2017. Changes in immune functions in bottlenose dolphins in the northern Gulf of Mexico associated with the Deepwater Horizon oil spill. Endangered Species Research 33:291–303. doi: 10.3354/esr00814

De Guise, S., M. Levin, L. Jasperse, J. Herrman, R.S. Wells, T. Rowles, and L. Schwacke. 2021. Long-term immunological alterations in bottlenose dolphin a decade after the Deepwater Horizon oil spill in the northern Gulf of Mexico: Potential for multigenerational effects. Environmental Toxicology and Chemistry 40(5):1308–1321. doi: 10.1002/etc.4980

Deepwater Horizon Natural Resource Damage Assessment Trustees (DWH NRDA). 2016. Deepwater Horizon oil spill: Final Programmatic Damage Assessment and Restoration Plan and Final Programmatic Environmental Impact Statement. Retrieved from: http://www.gulfspillrestoration.noaa.gov/restoration-planning/gulf-plan.1659.

Fauquier, D.A., J. Litz, S. Sanchez, K. Colegrove, L.H. Schwacke, L. Hart, J. Saliki, C. Smith, T. Goldstein, S. Bowen-Stevens, W. McFee, E. Fougeres, B. Mase-Guthrie, E. Stratton, R. Ewing, S. Venn-Watson, R.H. Carmichael, C. Clemons-Chevis, W. Hatchett, D. Shannon, S. Shippee, S. Smith, L. Staggs, M.C. Tumlin, N.L. Wingers, and T.K. Rowles. 2017. Evaluation of morbillivirus exposure in cetaceans from the northern Gulf of Mexico 2010-2014. Endangered Species Research 33:211–220. doi: 10.3354/esr00772

Fernandez, S. and A.A. Hohn. 1998. Age, growth, and calving season of bottlenose dolphins, *Tursiops truncatus*, off coastal Texas. Fishery Bulletin 96(2):357–365.

Fertl, D. 1994. Occurrence patterns and behavior of bottlenose dolphins (*Tursiops truncatus*) in the Galveston ship channel, Texas. Texas Journal of Science 46(4):299–318.

Hayes, S.A., E. Josephson, K. Maze-Foley, P.E. Rosel, and J. Wallace. 2022. U.S. Atlantic and Gulf of Mexico marine mammal stock assessments 2021. National Oceanic and Atmospheric Administration, National Marine Fisheries Service, Northeast Fisheries Science Center: Woods Hole, MA. NOAA Technical Memorandum NMFS-NE-288. 380 p. doi: 10.25923/6tt7-kc16

Helm, R.C., D.P. Costa, T.D. DeBruyn, T.J. O'Shea, R.S. Wells, and T.M. Williams. 2015. Overview of effects of oil spills on marine mammals. In: Fingas, M. (Ed.), Handbook of oil spill science and technology, First edition. John Wiley & Sons, Inc., Hoboken, NJ. pp. 455–475.

Henderson, E.E. and B. Würsig. 2007. Behavior patterns of bottlenose dolphins in San Luis Pass, Texas. Gulf of Mexico Science 25(2):153–161. doi: 10.18785/goms.2502.06

Hohn, A.A., M.D. Scott, R.S. Wells, J.C. Sweeney, and A.B. Irvine. 1989. Growth layers in teeth from known-age, free-ranging bottlenose dolphins. Marine Mammal Science 5(4):315–342. doi: 10.1111/j.1748-7692.1989.tb00346.x

Hubard, C.W., K. Maze-Foley, K.D. Mullin, and W.W. Schroeder. 2004. Seasonal abundance and site fidelity of bottlenose dolphins (*Tursiops truncatus*) in Mississippi Sound. Aquatic Mammals 30(2):299–310. doi: 10.1578/am.30.2.2004.299

Irvine, A.B., M.D. Scott, R.S. Wells, and J.H. Kaufmann. 1981. Movements and activities of the Atlantic bottlenose dolphin, *Tursiops truncatus*, near Sarasota, Florida. Fishery Bulletin 79(4):671–688.

Irvine, B. and R.S. Wells. 1972. Results of attempts to tag Atlantic bottlenosed dolphins (*Tursiops truncatus*). Cetology 13:1–5.

Irwin, L.-J. and B. Würsig. 2004. A small resident community of bottlenose dolphins, *Tursiops truncatus*, in Texas: Monitoring recommendations. Gulf of Mexico Science 22(1):13–21. doi: 10.18785/goms.2201.02

Kellar, N.M., T.R. Speakman, C.R. Smith, S.M. Lane, B.C. Balmer, M.L. Trego, K.N. Catelani, M.N. Robbins, C.D. Allen, R.S. Wells, E.S. Zolman, T.K. Rowles, and L.H. Schwacke. 2017. Low reproductive success rates of common bottlenose dolphins *Tursiops truncatus* in the northern Gulf of Mexico following the Deepwater Horizon disaster (2010-2015). Endangered Species Research 33:143–158. doi: 10.3354/esr00775

Kucklick, J., L. Schwacke, R. Wells, A. Hohn, A. Guichard, J. Yordy, L. Hansen, E. Zolman, R. Wilson, J. Litz, D. Nowacek, T. Rowles, R. Pugh, B. Balmer, C. Sinclair, and P. Rosel. 2011. Bottlenose dolphins as indicators of persistent organic pollutants in the western North Atlantic Ocean and Northern Gulf of Mexico. Environmental Science & Technology 45(10):4270–4277. doi: 10.1021/es1042244

LaBrecque, E., C. Curtice, J. Harrison, S.M. Van Parijs, and P.N. Halpin. 2015. 3. Biologically Important Areas for cetaceans within U.S. Waters – Gulf of Mexico Region. Aquatic Mammals 41(1):30–38. doi: 10.1578/am.41.1.2015.30

Lane, S.M., C.R. Smith, J. Mitchell, B.C. Balmer, K.P. Barry, T. McDonald, C.S. Mori, P.E. Rosel, T.K. Rowles, T.R. Speakman, F.I. Townsend, M.C. Tumlin, R.S. Wells, E.S. Zolman, and L.H. Schwacke. 2015. Reproductive outcome and survival of common bottlenose dolphins sampled in Barataria Bay, Louisiana, USA, following the Deepwater Horizon oil spill. Proceedings of the Royal Society B: Biological Sciences 282(1818):20151944. doi: 10.1098/rspb.2015.1944

Leatherwood, S. 1977. Some preliminary impressions on the numbers and social behavior of free-swimming bottlenosed dolphin calves (*Tursiops truncatus*) in the northern Gulf of Mexico. In: Ridgway, S.H. and K. Bernirschke (Eds.), Breeding Dolphins; Present Status, Suggestions for the Future. Final report to the US Marine Mammal Commission, Contract MM6AC009, MMC-76/07, Washington, DC. pp. 143–167.

Lipscomb, T.P., S. Kennedy, D. Moffett, A. Krafft, B.A. Klaunberg, J.H. Lichy, G.T. Regan, G.A.J. Worthy, and J.K. Taubenberger. 1996. Morbilliviral epizootic in bottlenose dolphins of the Gulf of Mexico. Journal of Veterinary Diagnostic Investigation 8(3):283–290. doi: 10.1177/104063879600800302

Litz, J.A., M.A. Baran, S.R. Bowen-Stevens, R.H. Carmichael, K.M. Colegrove, L.P. Garrison, S.E. Fire, E.M. Fougeres, R. Hardy, S. Holmes, W. Jones, B.E. Mase-Guthrie, D.K. Odell, P.E. Rosel, J.T. Saliki, D.K. Shannon, S.F. Shippee, S.M. Smith, E.M. Stratton, M.C. Tumlin, H.R. Whitehead, G.A. Worthy, and T.K. Rowles. 2014. Review of historical unusual mortality events (UMEs) in the Gulf of Mexico (1990–2009): providing context for the multi-year northern Gulf of Mexico cetacean UME declared in 2010. Diseases of Aquatic Organisms 112(2):161–175. doi: 10.3354/dao02807

Lynn, S.K. and B. Würsig. 2002. Summer movement patterns of bottlenose dolphins in a Texas bay. Gulf of Mexico Science 20(1):25–37. doi: 10.18785/goms.2001.03

Mackey, A.D. 2010. Site fidelity and association patterns of bottlenose dolphins (*Tursiops truncatus*) in the Mississippi Sound. University of Southern Mississippi. Thesis. 133 p.

Mann, J., R.C. Connor, L.M. Barre, and M.R. Heithaus. 2000. Female reproductive success in bottlenose dolphins (*Tursiops* sp.): life history, habitat, provisioning, and group-size effects. Behavioral Ecology 11(2):210–219. doi: 10.1093/beheco/11.2.210

Mattson, M.C., K.D. Mullin, G.W. Ingram, and W. Hoggard. 2006. Age structure and growth of the bottlenose dolphin (*Tursiops truncatus*) from strandings in the Mississippi sound region of the north-central Gulf of Mexico from 1986 to 2003. Marine Mammal Science 22(3):654–666. doi: 10.1111/j.1748-7692.2006.00057.x

Maze, K.S. and B. Würsig. 1999. Bottlenosed dolphins of San Luis Pass, Texas: Occurrence patterns, site-fidelity, and habitat use. Aquatic Mammals 25:91–104.

McDonald, T.L., F.E. Hornsby, T.R. Speakman, E.S. Zolman, K.D. Mullin, C. Sinclair, P.E. Rosel, L. Thomas, and L.H. Schwacke. 2017. Survival, density, and abundance of common bottlenose dolphins in Barataria Bay (USA) following the Deepwater Horizon oil spill. Endangered Species Research 33:193–209. doi: 10.3354/esr00806

McHugh, M.B. 1989. Population numbers and feeding behavior of the Atlantic bottlenose dolphin (*Tursiops truncatus*) near Aransas Pass, Texas. University of Texas at Austin. Thesis. 96 p.

Mead, J.G. and C.W. Potter. 1990. Natural history of bottlenose dolphins along the central Atlantic coast of the United States. In: Leatherwood, S. and R.R. Reeves (Eds.), The Bottlenose Dolphin. Academic Press, San Diego, CA. pp. 165–195.

Michel, J., E.H. Owens, S. Zengel, A. Graham, Z. Nixon, T. Allard, W. Holton, P.D. Reimer, A. Lamarche, M. White, N. Rutherford, C. Childs, G. Mauseth, G. Challenger, and E. Taylor. 2013. Extent and degree of shoreline oiling: Deepwater Horizon oil spill, Gulf of Mexico, USA. PLoS ONE 8(6):e65087. doi: 10.1371/journal.pone.0065087

Miller, C.E. 2003. Abundance trends and environmental habitat usage patterns of bottlenose dolphins (*Tursiops truncatus*) in lower Barataria and Caminada bays, Louisiana. Louisiana State University and Agricultural & Mechanical College. Dissertation. 125 p.

Miller, L.J., A.D. Mackey, T. Hoffland, M. Solangi, and S.A. Kuczaj. 2010. Potential effects of a major hurricane on Atlantic bottlenose dolphin (*Tursiops truncatus*) reproduction in the Mississippi Sound. Marine Mammal Science 26(3):707–715. doi: 10.1111/j.1748-7692.2010.00371.x

Miller, L.J., A.D. Mackey, M. Solangi, and S.A. Kuczaj. 2013. Population abundance and habitat utilization of bottlenose dolphins in the Mississippi Sound. Aquatic Conservation: Marine and Freshwater Ecosystems 23(1):145–151. doi: 10.1002/aqc.2278

Mullin, K.D., T. McDonald, R.S. Wells, B.C. Balmer, T. Speakman, C. Sinclair, E.S. Zolman, F. Hornsby, S.M. McBride, K.A. Wilkinson, and L.H. Schwacke. 2017. Density, abundance, survival, and ranging patterns of common bottlenose dolphins (*Tursiops truncatus*) in Mississippi Sound following the Deepwater Horizon oil spill. PLoS ONE 12(10):e0186265. doi: 10.1371/journal.pone.0186265

National Oceanic and Atmospheric Administration (NOAA). 2011. Public scoping for preparation of a programmatic environmental impact statement for the Deepwater Horizon BP Oil Spill. 9 p.

Perrin, W.F. and S.B. Reilly. 1984. Reproductive parameters of dolphins and small whales of the family Delphinidae. In: Perrin, W.F., R.L. Brownell, Jr., and D.P. DeMaster (Eds.), Reproduction in Whales, Dolphins, and Porpoises. pp. 97–134.

Rosel, P.E., L.A. Wilcox, C. Sinclair, T.R. Speakman, M.C. Tumlin, J.A. Litz, and E.S. Zolman. 2017. Genetic assignment to stock of stranded common bottlenose dolphins in southeastern Louisiana after the Deepwater Horizon oil spill. Endangered Species Research 33:221–234. doi: 10.3354/esr00780

Rossman, S., P.H. Ostrom, M. Stolen, N.B. Barros, H. Gandhi, C.A. Stricker, and R.S. Wells. 2015. Individual specialization in the foraging habits of female bottlenose dolphins living in a trophically diverse and habitat rich estuary. Oecologia 178(2):415–425. doi: 10.1007/s00442-015-3241-6

Schwacke, L.H., C.R. Smith, F.I. Townsend, R.S. Wells, L.B. Hart, B.C. Balmer, T.K. Collier, S. De Guise, M.M. Fry, L.J. Guillette, Jr., S.V. Lamb, S.M. Lane, W.E. McFee, N.J. Place, M.C. Tumlin, G.M. Ylitalo, E.S. Zolman, and T.K. Rowles. 2014. Health of common bottlenose dolphins (*Tursiops truncatus*) in Barataria Bay, Louisiana, following the deepwater horizon oil spill. Environmental Science & Technology 48(1):93–103. doi: 10.1021/es403610f

Schwacke, L.H., L. Thomas, R.S. Wells, W.E. McFee, A.A. Hohn, K.D. Mullin, E.S. Zolman, B.M. Quigley, T.K. Rowles, and J.H. Schwacke. 2017. Quantifying injury to common bottlenose dolphins from the Deepwater Horizon oil spill using an age-, sex- and class-structured population model. Endangered Species Research 33:265–279. doi: 10.3354/esr00777

Scott, M.D. and S.J. Chivers. 1990. Distribution and herd structure of bottlenose dolphins in the eastern tropical Pacific Ocean. In: Leatherwood, S. and R.R. Reeves (Eds.), The Bottlenose Dolphin. Academic Press, San Diego, CA. pp. 387–402.

Sellas, A.B., R.S. Wells, and P.E. Rosel. 2005. Mitochondrial and nuclear DNA analyses reveal fine scale geographic structure in bottlenose dolphins (*Tursiops truncatus*) in the Gulf of Mexico. Conservation Genetics 6(5):715–728. doi: 10.1007/s10592-005-9031-7

Sergeant, D.E., D.K. Caldwell, and M.C. Caldwell. 1973. Age, growth, and maturity of bottlenosed dolphin (*Tursiops-truncatus*) from northeast Florida. Journal of the Fisheries Research Board of Canada 30(7):1009–1011. doi: 10.1139/f73-165

Shane, S.H. 1990. Behavior and ecology of the bottlenose dolphin at Sanibel Island, Florida. In: Leatherwood, S. and R.R. Reeves (Eds.), The Bottlenose Dolphin. Academic Press, San Diego, CA. pp. 245–265. doi: 10.1016/B978-0-12-440280-5.50016-0

Shane, S.H. 2004. Residence patterns, group characteristics, and association patterns of bottlenose dolphins near Sanibel Island, Florida. Gulf of Mexico Science 22(1):1–12. doi: 10.18785/goms.2201.01

Shane, S.H., R.S. Wells, and B. Würsig. 1986. Ecology, behavior and social organization of the bottlenose dolphin: A review. Marine Mammal Science 2:34–63. doi: 10.1111/j.1748-7692.1986.tb00026.x

Smith, C.R., T.K. Rowles, L.B. Hart, F.I. Townsend, R.S. Wells, E.S. Zolman, B.C. Balmer, B. Quigley, M. Ivančić, W. McKercher, M.C. Tumlin, K.D. Mullin, J.D. Adams, Q. Wu, W. McFee, T.K. Collier, and L.H. Schwacke. 2017. Slow recovery of Barataria Bay dolphin health following the Deepwater Horizon oil spill (2013-2014), with evidence of persistent lung disease and impaired stress response. Endangered Species Research 33:127–142. doi: 10.3354/esr00778

Takeshita, R., B.C. Balmer, F. Messina, E.S. Zolman, L. Thomas, R.S. Wells, C.R. Smith, T.K. Rowles, and L.H. Schwacke. 2021. High site-fidelity in common bottlenose dolphins despite low salinity exposure and associated indicators of compromised health. PLoS ONE 16(9):e0258031. doi: 10.1371/journal.pone.0258031

Taylor, B.L., S.J. Chivers, J. Larese, and W.F. Perrin. 2007. Generation length and percent mature estimates for IUCN assessments of cetaceans. National Oceanic and Atmospheric Administration, National Marine Fisheries Service, Southwest Fisheries Science Center: La Jolla, CA. Administrative Report LJ-07-01. 24 p.

Toms, C. 2019. Filling the gaps: Common bottlenose dolphin (*Tursiops truncatus*) population dynamics, structure, and connectivity within Florida panhandle bays, sounds, and estuaries. University of Central Florida. Dissertation. 373 p.

Torres, L.G. and A.J. Read. 2009. Where to catch a fish? The influence of foraging tactics on the ecology of bottlenose dolphins (*Tursiops truncatus*) in Florida Bay, Florida. Marine Mammal Science 25(4):797–815. doi: 10.1111/j.1748-7692.2009.00297.x

Tyson, R.B., S.M. Nowacek, and D.P. Nowacek. 2011. Community structure and abundance of bottlenose dolphins *Tursiops truncatus* in coastal waters of the northeast Gulf of Mexico. Marine Ecology Progress Series 438:253–265. doi: 10.3354/meps09292

Urian, K.W., S. Hofmann, R.S. Wells, and A.J. Read. 2009. Fine-scale population structure of bottlenose dolphins (*Tursiops truncatus*) in Tampa Bay, Florida. Marine Mammal Science 25(3):619–638. doi: 10.1111/j.1748-7692.2009.00284.x

Van Bressem, M.F., P.J. Duignan, A. Banyard, M. Barbieri, K.M. Colegrove, S. De Guise, G. Di Guardo, A. Dobson, M. Domingo, D. Fauquier, A. Fernandez, T. Goldstein, B. Grenfell, K.R. Groch, F. Gulland, B.A. Jensen, P.D. Jepson, A. Hall, T. Kuiken, S. Mazzariol, S.E. Morris, O. Nielsen, J.A. Raga, T.K. Rowles, J. Saliki, E. Sierra, N. Stephens, B. Stone, I. Tomo, J. Wang, T. Waltzek, and J.F. Wellehan. 2014. Cetacean morbillivirus: current knowledge and future directions. Viruses 6(12):5145–5181. doi: 10.3390/v6125145

Venn-Watson, S., L. Garrison, J. Litz, E. Fougeres, B. Mase, G. Rappucci, E. Stratton, R. Carmichael, D. Odell, D. Shannon, S. Shippee, S. Smith, L. Staggs, M. Tumlin, H. Whitehead, and T. Rowles. 2015. Demographic clusters identified within the northern Gulf of Mexico common bottlenose dolphin (*Tursiops truncates*) unusual mortality event: January 2010-June 2013. PLoS ONE 10(2):e0117248. doi: 10.1371/journal.pone.0117248

Vollmer, N.L. and P.E. Rosel. 2013. A review of common bottlenose dolphins (*Tursiops truncatus truncatus*) in the Northern Gulf of Mexico: Population biology, potential threats, and management. Southeastern Naturalist 12(m6):1–43. doi: 10.1656/058.012.m601

Weller, D.W. 1998. Global and regional variation in the biology and behavior of bottlenose dolphins. Texas A&M University. Dissertation. 142 p.

Wells, R.S. 1986a. Population structure of bottlenose dolphins: Behavioral studies along the central west coast of Florida. Contract Report to National Marine Fisheries Service, Southeast Fisheries Center. Contract No. 45-WCNF-5-003BB. 58 p.

Wells, R.S. 1986b. Structural aspects of dolphin societies. University of California, Santa Cruz. Dissertation. 234 p.

Wells, R.S. 1991. The role of long-term study in understanding the social structure of a bottlenose dolphin community. In: Pryor, K. and K. Norris (Eds.), Dolphin Societies: Discoveries and Puzzles. University of California Press, Berkeley, CA. pp. 199–226.

Wells, R.S. 2000. Reproduction in wild bottlenose dolphins: Overview of patterns observed during a long-term study. In: Duffield, D. and T. Robeck (Eds.), Bottlenose Dolphin Reproduction Workshop. AZA Marine Mammal Taxon Advisory Group, Silver Spring, MD. pp. 57–74.

Wells, R.S. 2003. Dolphin social complexity: Lessons from long-term study and life history. In: de Waal, F.B.M. and P.L. Tyack (Eds.), Animal Social Complexity: Intelligence, Culture, and Individualized Societies Harvard University Press, Cambridge, MA. pp. 32–56.

Wells, R.S. 2014. Social structure and life history of bottlenose dolphins near Sarasota Bay, Florida: Insights from four decades and five generations. Primates and cetaceans. pp. 149–172. doi: 10.1007/978-4-431-54523-1_8

Wells, R.S., M.K. Bassos, K.W. Urian, W.J. Carr, and M.D. Scott. 1996. Low-level monitoring of bottlenose dolphins, *Tursiops truncatus*, in Charlotte Harbor, Florida: 1990-1994. National Oceanic and Atmospheric Administration, National Marine Fisheries Service, Southeast Fisheries Science Center: Miami, FL. NOAA Technical Memorandum NMFS-SEFSC-384. 86 p.

Wells, R.S., M.K. Bassos, K.W. Urian, S.H. Shane, E.C.G. Owen, C.F. Weiss, W.J. Carr, and M.D. Scott. 1997. Low-level monitoring of bottlenose dolphins, *Tursiops truncatus*, in Pine Island Sound, Florida: 1996. National Oceanic and Atmospheric Administration, National Marine Fisheries Service, Southeast Fisheries Science Center: Miami, FL. Southeast Fisheries Center Contribution No. 40-WCNF601958. 86 p.

Wells, R.S., A. Natoli, and G. Braulik. 2019. *Tursiops truncatus* (errata version published in 2019). *The IUCN Red List of Threatened Species* 2019: e.T22563A156932432. https://dx.doi.org/10.2305/IUCN.UK.2019-1.RLTS.T22563A156932432.en

Wells, R.S., L.H. Schwacke, T.K. Rowles, B.C. Balmer, E. Zolman, T. Speakman, F.I. Townsend, M.C. Tumlin, A. Barleycorn, and K.A. Wilkinson. 2017. Ranging patterns of common bottlenose dolphins *Tursiops truncatus* in Barataria Bay, Louisiana, following the Deepwater Horizon oil spill. Endangered Species Research 33:159–180. doi: 10.3354/esr00732

Wells, R.S. and M.D. Scott. 1999. Bottlenose dolphin *Tursiops truncatus* (Montagu, 1821). In: Ridgway, S.H. and R. Harrison (Eds.), Handbook of marine mammals. Volume 6: The second book of dolphins and the porpoises. Academic Press, San Diego, CA. pp. 137–182.

Wells, R.S. and M.D. Scott. 2018. Bottlenose dolphin, *Tursiops truncatus*, common bottlenose dolphin. In: Würsig, B., J.G.M. Thewissen, and K.M. Kovacs (Eds.), Encyclopedia of Marine Mammals (Third Edition). Academic Press, London, UK. pp. 118–125. doi: 10.1016/B978-0-12-804327-1.00072-8

Wells, R.S., M.D. Scott, and A.B. Irvine. 1987. The social structure of free-ranging bottlenose dolphins. In: Genoways, H.H. (Ed.), Current Mammalogy. Plenum Press, New York, NY. pp. 247–305.

Wells, R.S., K.W. Urian, A.J. Read, M.K. Bassos, W.J. Carr, and M.D. Scott. 1996. Low-level monitoring of bottlenose dolphins, *Tursiops truncatus*, in Tampa Bay, Florida: 1988-1993. National Oceanic and Atmospheric Administration, National Marine Fisheries Service, Southeast Fisheries Science Center: Miami, FL. NOAA Technical Memorandum NMFS-SEFSC-385. 36 p.

Wilson, R.M., R.B. Tyson, J.A. Nelson, B.C. Balmer, J.P. Chanton, and D.P. Nowacek. 2017. Niche differentiation and prey selectivity among common bottlenose dolphins (*Tursiops truncatus*)

sighted in St. George Sound, Gulf of Mexico. Frontiers in Marine Science 4:235. doi: 10.3389/fmars.2017.00235

Common bottlenose dolphin − *Tursiops truncatus*

GoMx BSE/Nueces Bay, Corpus Christi Bay Stock

Overall Vulnerability Rank = Very High

Biological Sensitivity = Very High

Climate Exposure = Very High

Data Quality = 93% of scores ≥ 2

Tursiops truncatus		Expert Scores	Data Quality	Expert Scores Plots (Portion by Category)
Sensitivity attributes	Prey/Diet Specificity	2.4	2.2	
	Habitat Specificity	2.8	2.0	
	Site Fidelity	3.4	2.2	
	Lifetime Reproductive Potential	1.5	2.2	
	Generation Length	2.9	2.2	
	Reproductive Plasticity	1.2	2.0	
	Migration	3.8	2.2	
	Home Range	3.5	2.5	
	Stock Abundance	3.0	1.8	
	Stock Abundance Trend	2.0	1.5	
	Cumulative Stressors	3.5	2.8	
	Sensitivity Score	Very High		
Exposure variables	Sea Surface Temperature (Change in mean)	3.8	2.8	
	Sea Surface Temperature (Change in variability)	1.0	2.8	
	Air Temperature (Change in mean)	4.0	2.8	
	Air Temperature (Change in variability)	1.0	2.8	
	Precipitation (Change in mean)	1.0	2.8	
	Precipitation (Change in variability)	1.0	2.8	
	Sea Surface Salinity (Change in mean)	2.0	2.8	
	Sea Surface Salinity (Change in variability)	1.0	2.8	
	Ocean pH (Change in mean)	4.0	2.8	
	Ocean pH (Change in variability)	1.0	2.8	
	Sea ice coverage (Change in mean)	1.0	2.8	
	Sea ice coverage (Change in variability)	1.0	2.8	
	Dissolved oxygen (Change in mean)	3.2	2.8	
	Dissolved oxygen (Change in variability)	1.0	2.8	
	Circulation	3.2	2.5	
	Sea level rise	4.0	2.8	
	Exposure Score	Very High		
Overall Vulnerability Rank		Very High		

Low
Moderate
High
Very High

Common bottlenose dolphin (GoMx BSE/Nueces Bay, Corpus Christi Bay Stock)

Tursiops truncatus

CVA Results Summary

Overall Climate Vulnerability Rank: Very High (100% certainty from bootstrap analysis).

Climate Exposure: Very High. Four exposure factors scored greater than 3.5: Air Temperature (Standard anomaly) (4.00), Ocean pH (Standard anomaly) (4.00), Sea level rise (4.00), and Sea Surface Temperature (Standard anomaly) (3.85).

Biological Sensitivity: Very High. Three sensitivity attributes scored greater than 3.5: Migration (3.85), Cumulative Stressors (3.55), and Home Range (3.50).

Distributional Response: High

Abundance Response: High

Phenology Response: High

Data Quality: 93% of the data quality scores were 2 or greater, 82% of sensitivity attribute data quality scores were 2 or higher.

Background Information

Stock Description

This stock includes common bottlenose dolphins in Nueces Bay and Corpus Christi Bay in the western Gulf of Mexico along the Texas coast (Hayes et al. 2022).

Prey/Diet Specificity

Generally, common bottlenose dolphins consume a large variety of fish and/or squid (Barros and Odell 1990; Mead and Potter 1990; Wells and Scott 2018). Common bottlenose dolphins seem to show a consistent preference for noise-producing fishes (e.g., sciaenids, toadfish; Berens McCabe et al. 2010; Bowen-Stevens et al. 2021). In Sarasota Bay, FL, Rossman et al. (2015) found some individual common bottlenose dolphins to show diet specialization despite the broader study group showing a generalist diet. Specialization can be influenced by locally abundant prey species (Shane et al. 1986; Vollmer and Rosel 2013) or can result from resource partitioning amongst sympatric populations (e.g., Torres and Read 2009; Wilson et al. 2017).

Habitat Specificity

Common bottlenose dolphins are found in a variety of marine and estuarine habitats (Wells and Scott 2018).

Site Fidelity

No specific site fidelity information was found for this stock. However, other Gulf of Mexico bay, sound, and estuary (BSE) stocks show site fidelity (LaBrecque et al. 2015; Hayes et al. 2022), such as in Matagorda Bay and Espiritu Santo Bay in Texas (Lynn and Würsig 2002), San Luis Pass in Texas (Maze and Würsig 1999; Irwin and Würsig 2004), Barataria Bay in Louisiana (Miller 2003; Wells et al. 2017; Takeshita et al. 2021), Mississippi Sound (Hubard et al. 2004; Mackey 2010), St. Vincent Sound and Apalachicola Bay (Tyson et al. 2011), Tampa Bay (Wells et al. 1996b; Urian et al. 2009), and Charlotte Harbor and Pine Island Sound (Wells et al. 1996a; Wells et al. 1997; Bassos-Hull et al. 2013).

Lifetime Reproductive Potential

Taylor et al. (2007) reported the interbirth interval of common bottlenose dolphins as 3.80 years, though other studies have shown it to be closer to three years (Connor et al. 2000; Leatherwood 1977; Wells 2003; Wells and Scott 1999).

Generally, females reach sexual maturity before males, with females reaching sexual maturity at 5–13 years (Sergeant et al. 1973; Perrin and Reilly 1984; Wells et al. 1987; Mead and Potter 1990; Mann et al. 2000; Wells 2000; Wells and Scott 2018).

Females reproduce through most of their adult lives (Leatherwood 1977; Hohn et al. 1989; Wells and Scott 1999; Wells 2000; Wells 2003), but may produce fewer calves after they reach 30 years of age (Wells 2000). Sarasota Bay common bottlenose dolphins can give birth when they are as young as 6 years of age, and can continue until they are 48 years old (Wells and Scott 2018). Reproductive lifespan would therefore be up to 42 years based on long-term observation studies (Wells 2014; Wells and Scott 2018).

Longitudinal studies from Sarasota Bay, Florida have found that female common bottlenose dolphins can live up to 67 years (Wells and Scott 2018). Studies based on growth layer groups in teeth suggest female common bottlenose dolphins can live to 41 years along the Texas coast (Fernandez and Hohn 1998) and to 30 years along the Mississippi coast (Mattson et al. 2006).

Generation Length

Taylor et al. (2007) reported common bottlenose dolphin generation length of 20.6-21.1 years.

Reproductive Plasticity

Common bottlenose dolphin calving is generally diffusely seasonal, with one or more peaks, although births have been reported from all seasons (Urian et al. 1996; Vollmer and Rosel 2013; Wells and Scott 2018). Calf sightings peak in the spring and summer months in Florida (Wells et al. 1987), Mississippi/Louisiana (Miller et al. 2010; Miller et al. 2013), and Texas (McHugh 1989; Henderson et al. 2007).

Migration

The BSE stocks in the Gulf of Mexico are thought to primarily comprise stable resident communities. However, transient or seasonal movements of some individuals into and out of a BSE stock area are

recorded in some areas (LaBrecque et al. 2015; Hayes et al. 2022). Molecular genetic data support that some BSE stocks are genetically distinct from one another, and furthermore are differentiated from adjacent coastal populations (e.g., Sellas et al. 2005; Rosel et al. 2017). For example, Toms (2019) found fine-scale genetic population structure among five inshore systems in the Florida Panhandle and variable migration rates among populations.

Year-round residency has been reported for common bottlenose dolphins (Hayes et al. 2022), such as in Florida (Irvine and Wells 1972; Irvine et al. 1981; Wells 1986a; Wells 1986b; Wells et al. 1987; Scott et al. 1990; Wells 1991; Wells et al. 1996a; Wells et al. 1996b; Wells et al. 1997; Wells 2003; Balmer et al. 2008; Urian et al. 2009; Tyson et al. 2011; Bassos-Hull et al. 2013; Balmer et al. 2018; Balmer et al. 2019; Toms 2019), Texas (Shane 1990; Bräger 1993; Bräger et al. 1994; Fertl 1994; Weller 1998; Maze and Würsig 1999; Lynn and Würsig 2002; Irwin and Würsig 2004; Shane 2004), and Louisiana/Mississippi (Hubard et al. 2004; Mullin et al. 2017; Wells et al. 2017; Takeshita et al. 2021).

Home Range

The home ranges of individuals within the Gulf of Mexico BSE stocks are considered to generally include the areas within the stock boundaries and brief excursions beyond the stock boundaries (Irvine et al. 1981; Wells 1986a; Wells 1986b; Wells 1991; Wells et al. 1996a; Wells et al. 1996b; Balmer et al. 2008; Wells et al. 2017), although some individuals show preference for small areas within the stock boundaries (Lynn and Würsig 2002).

Stock Abundance

Blaylock and Hoggard (1994) estimated this stock's abundance at 58 individuals (CV = 0.61). More recent estimates are unavailable (Hayes et al. 2022).

Stock Abundance Trend

Data are insufficient to complete an abundance trend analysis for this stock (Hayes et al. 2022).

Cumulative Stressors

Common bottlenose dolphins face a wide range of individual and cumulative natural and anthropogenic threats (see reviews by Vollmer and Rosel 2013; Phillips and Rosel 2014; Wells and Scott 2018; and Wells et al. 2019). These include such threats as shark predation, harmful algal blooms, recreational and commercial fishing gear, vessel traffic, pollution, habitat alteration, and provisioning.

Phillips and Rosel (2014) reviewed the cumulative stressors influencing the Nueces Bay and Corpus Christi Bay system. Potential health threats range from contagious disease (e.g., *Brucella*), harmful algal blooms (HABs; Fire et al. 2011, 2020), and anthropogenic activities: mining, crude oil exploration, transport, and refinement, commercial fisheries, agricultural runoff in the form of pesticide and fertilizer, heavy metals and other chemical contaminants, marine debris, shipping and dredging, noise, tourism and boat traffic. Natural events may also pose threats: hypoxia resulting from HABs, adverse weather (e.g., hurricane storm surge), habitat loss, and climate change (Phillips and Rosel 2014).

Unusual Mortality Events (UMEs) have been declared for common bottlenose dolphins in waters of Texas with morbillivirus the confirmed cause in 1994, a suspected cause in 1992 along with

environmental conditions, and an undetermined cause in 2008 (Litz et al. 2014). A UME was declared in 1990 for the Gulf of Mexico from Florida to Texas, with more than 300 common bottlenose dolphins stranding due to an undetermined cause, although morbillivirus and cold temperatures may have contributed (Litz et al. 2014).

Further Reading

Hayes, S.A., E. Josephson, K. Maze-Foley, P.E. Rosel, and J. Wallace. 2022. U.S. Atlantic and Gulf of Mexico marine mammal stock assessments 2021. National Oceanic and Atmospheric Administration, National Marine Fisheries Service, Northeast Fisheries Science Center: Woods Hole, MA. NOAA Technical Memorandum NMFS-NE-288. 380 p. doi: 10.25923/6tt7-kc16

Wells, R.S. and M.D. Scott. 2018. Bottlenose Dolphin, *Tursiops truncatus*, Common Bottlenose Dolphin. In: Würsig, B., J.G.M. Thewissen, and K.M. Kovacs (Eds.), Encyclopedia of Marine Mammals (Third Edition). Academic Press, London, UK. pp. 118–125. doi: 10.1016/B978-0-12-804327-1.00072-8

Wells, R.S., A. Natoli, and G. Braulik. 2019. *Tursiops truncatus* (errata version published in 2019). The IUCN Red List of Threatened Species 2019: e.T22563A156932432. https://dx.doi.org/10.2305/IUCN.UK.2019-1.RLTS.T22563A156932432.en

References

Balmer, B., T. McDonald, F. Hornsby, J. Adams, J. Allen, A. Barleycorn, and L. Schwacke. 2018. Long-term trends in a northern Gulf of Mexico common bottlenose dolphin (*Tursiops truncatus*) population in the wake of the Deepwater Horizon oil spill. Journal of Cetacean Research and Management 18:1–9. doi: 10.47536/jcrm.v18i1.409

Balmer, B., S. Watwood, B. Quigley, T. Speakman, K. Barry, K. Mullin, P. Rosel, C. Sinclair, E. Zolman, and L. Schwacke. 2019. Common bottlenose dolphin (*Tursiops truncatus*) abundance and distribution patterns in St. Andrew Bay, Florida, USA. Aquatic Conservation: Marine and Freshwater Ecosystems 29(3):486–498. doi: 10.1002/aqc.3001

Balmer, B.C., R.S. Wells, S.M. Nowacek, D.P. Nowacek, L.H. Schwacke, W.A. McLellan, F.S. Scharf, T.K. Rowles, L.J. Hansen, T.R. Spradlin, and D.A. Pabst. 2008. Seasonal abundance and distribution patterns of common bottlenose dolphins (*Tursiops truncatus*) near St. Joseph Bay, Florida, USA. Journal of Cetacean Research and Management 10(2):157–167.

Barros, N.B. and D.K. Odell. 1990. Food habits of bottlenose dolphins in the southeastern United States In: Leatherwood, S. and R.R. Reeves (Eds.), The Bottlenose Dolphin. Academic Press, San Diego, CA. pp. 309–328. doi: 10.1016/B978-0-12-440280-5.50020-2

Bassos-Hull, K., R.M. Perrtree, C.C. Shepard, S. Schilling, A.A. Barleycorn, J.B. Allen, B.C. Balmer, W.E. Pine, and R.S. Wells. 2013. Long-term site fidelity and seasonal abundance estimates of common bottlenose dolphins (*Tursiops truncatus*) along the southwest coast of Florida and responses to natural perturbations. Journal of Cetacean Research and Management 13(1):19–30. doi: 10.47536/jcrm.v13i1.551

Berens McCabe, E.J., D.P. Gannon, N.B. Barros, and R.S. Wells. 2010. Prey selection by resident common bottlenose dolphins (*Tursiops truncatus*) in Sarasota Bay, Florida. Marine Biology 157(5):931–942. doi: 10.1007/s00227-009-1371-2

Blaylock, R.A. and W. Hoggard. 1994. Preliminary estimates of bottlenose dolphin abundance in southern U.S. Atlantic and Gulf of Mexico continental shelf waters. National Oceanic and Atmospheric Administration, National Marine Fisheries Service, Southeast Fisheries Science Center: Miami, FL. NOAA Technical Memorandum NMFS-SEFSC-356. 10 p.

Bowen-Stevens, S.R., D.P. Gannon, R.A. Hazelkorn, G. Lovewell, K.M. Volker, S. Smith, M.C. Tumlin, and J. Litz. 2021. Diet of common bottlenose dolphins, *Tursiops truncatus*, that stranded in and near Barataria Bay, Louisiana, 2010–2012. Southeastern Naturalist 20(1). doi: 10.1656/058.020.0113

Bräger, S. 1993. Diurnal and seasonal behavior patterns of bottlenose dolphins (*Tursiops truncatus*). Marine Mammal Science 9:434–438. doi: 10.1111/j.1748-7692.1993.tb00477.x

Bräger, S., B. Würsig, A. Acevedo, and T. Henningsen. 1994. Association patterns of bottle-nosed dolphins (*Tursiops-truncatus*) in Galveston Bay, Texas. Journal of Mammalogy 75(2):431–437. doi: 10.2307/1382564

Connor, R.C., R.S. Wells, J. Mann, and A.J. Read. 2000. The bottlenose dolphin: Social relationships in a fission-fusion society. In: Mann, J., R. Connor, P. Tyack, and H. Whitehead (Eds.), Cetacean Societies: Field Studies of Dolphins and Whales. University of Chicago Press, Chicago, IL. pp. 91–126.

Fernandez, S. and A.A. Hohn. 1998. Age, growth, and calving season of bottlenose dolphins, *Tursiops truncatus*, off coastal Texas. Fishery Bulletin 96(2):357–365.

Fertl, D. 1994. Occurrence patterns and behavior of bottlenose dolphins (*Tursiops truncatus*) in the Galveston ship channel, Texas. Texas Journal of Science 46(4):299–318.

Fire, S.E., T.A. Leighfield, G.A. Miller, S. Piwetz, E.R. Sabater, and H. Whitehead. 2020. Association between red tide exposure and detection of corresponding neurotoxins in bottlenose dolphins from Texas waters during 2007-2017. Marine Environmental Research 162:105191. doi: 10.1016/j.marenvres.2020.105191

Fire, S.E., Z. Wang, M. Byrd, H.R. Whitehead, J. Paternoster, and S.L. Morton. 2011. Co-occurrence of multiple classes of harmful algal toxins in bottlenose dolphins (*Tursiops truncatus*) stranding during an unusual mortality event in Texas, USA. Harmful Algae 10(3):330–336. doi: 10.1016/j.hal.2010.12.001

Hayes, S.A., E. Josephson, K. Maze-Foley, P.E. Rosel, and J. Wallace. 2022. U.S. Atlantic and Gulf of Mexico marine mammal stock assessments 2021. National Oceanic and Atmospheric Administration, National Marine Fisheries Service, Northeast Fisheries Science Center: Woods Hole, MA. NOAA Technical Memorandum NMFS-NE-288. 380 p. doi: 10.25923/6tt7-kc16

Henderson, E.E. and B. Würsig. 2007. Behavior patterns of bottlenose dolphins in San Luis Pass, Texas. Gulf of Mexico Science 25(2):153–161. doi: 10.18785/goms.2502.06

Hohn, A.A., M.D. Scott, R.S. Wells, J.C. Sweeney, and A.B. Irvine. 1989. Growth layers in teeth from known-age, free-ranging bottlenose dolphins. Marine Mammal Science 5(4):315–342. doi: 10.1111/j.1748-7692.1989.tb00346.x

Hubard, C.W., K. Maze-Foley, K.D. Mullin, and W.W. Schroeder. 2004. Seasonal abundance and site fidelity of bottlenose dolphins (*Tursiops truncatus*) in Mississippi Sound. Aquatic Mammals 30(2):299–310. doi: 10.1578/am.30.2.2004.299

Irvine, A.B., M.D. Scott, R.S. Wells, and J.H. Kaufmann. 1981. Movements and activities of the Atlantic bottlenose dolphin, *Tursiops truncatus*, near Sarasota, Florida. Fishery Bulletin 79(4):671–688.

Irvine, B. and R.S. Wells. 1972. Results of attempts to tag Atlantic bottlenosed dolphins (*Tursiops truncatus*). Cetology 13:1–5.

Irwin, L.-J. and B. Würsig. 2004. A small resident community of bottlenose dolphins, *Tursiops truncatus*, in Texas: Monitoring recommendations. Gulf of Mexico Science 22(1):13–21. doi: 10.18785/goms.2201.02

LaBrecque, E., C. Curtice, J. Harrison, S.M. Van Parijs, and P.N. Halpin. 2015. 3. Biologically Important Areas for cetaceans within U.S. Waters – Gulf of Mexico Region. Aquatic Mammals 41(1):30–38. doi: 10.1578/am.41.1.2015.30

Leatherwood, S. 1977. Some preliminary impressions on the numbers and social behavior of free-swimming bottlenosed dolphin calves (*Tursiops truncatus*) in the northern Gulf of Mexico. In:

Ridgway, S.H. and K. Bernirschke (Eds.), Breeding Dolphins; Present Status, Suggestions for the Future. Final report to the US Marine Mammal Commission, Contract MM6AC009, MMC-76/07, Washington, DC. pp. 143–167.

Litz, J.A., M.A. Baran, S.R. Bowen-Stevens, R.H. Carmichael, K.M. Colegrove, L.P. Garrison, S.E. Fire, E.M. Fougeres, R. Hardy, S. Holmes, W. Jones, B.E. Mase-Guthrie, D.K. Odell, P.E. Rosel, J.T. Saliki, D.K. Shannon, S.F. Shippee, S.M. Smith, E.M. Stratton, M.C. Tumlin, H.R. Whitehead, G.A. Worthy, and T.K. Rowles. 2014. Review of historical unusual mortality events (UMEs) in the Gulf of Mexico (1990–2009): providing context for the multi-year northern Gulf of Mexico cetacean UME declared in 2010. Diseases of Aquatic Organisms 112(2):161–175. doi: 10.3354/dao02807

Lynn, S.K. and B. Würsig. 2002. Summer movement patterns of bottlenose dolphins in a Texas bay. Gulf of Mexico Science 20(1):25–37. doi: 10.18785/goms.2001.03

Mackey, A.D. 2010. Site fidelity and association patterns of bottlenose dolphins (*Tursiops truncatus*) in the Mississippi Sound. University of Southern Mississippi. Thesis. 133 p.

Mann, J., R.C. Connor, L.M. Barre, and M.R. Heithaus. 2000. Female reproductive success in bottlenose dolphins (*Tursiops* sp.): life history, habitat, provisioning, and group-size effects. Behavioral Ecology 11(2):210–219. doi: 10.1093/beheco/11.2.210

Mattson, M.C., K.D. Mullin, G.W. Ingram, and W. Hoggard. 2006. Age structure and growth of the bottlenose dolphin (*Tursiops truncatus*) from strandings in the Mississippi sound region of the north-central Gulf of Mexico from 1986 to 2003. Marine Mammal Science 22(3):654–666. doi: 10.1111/j.1748-7692.2006.00057.x

Maze, K.S. and B. Würsig. 1999. Bottlenosed dolphins of San Luis Pass, Texas: Occurrence patterns, site-fidelity, and habitat use. Aquatic Mammals 25:91–104.

McHugh, M.B. 1989. Population numbers and feeding behavior of the Atlantic bottlenose dolphin (*Tursiops truncatus*) near Aransas Pass, Texas. University of Texas at Austin. Thesis. 96 p.

Mead, J.G. and C.W. Potter. 1990. Natural history of bottlenose dolphins along the central Atlantic coast of the United States. In: Leatherwood, S. and R.R. Reeves (Eds.), The Bottlenose Dolphin. Academic Press, San Diego, CA. pp. 165–195.

Miller, C.E. 2003. Abundance trends and environmental habitat usage patterns of bottlenose dolphins (*Tursiops truncatus*) in lower Barataria and Caminada bays, Louisiana. Louisiana State University and Agricultural & Mechanical College. Dissertation. 125 p.

Miller, L.J., A.D. Mackey, T. Hoffland, M. Solangi, and S.A. Kuczaj. 2010. Potential effects of a major hurricane on Atlantic bottlenose dolphin (*Tursiops truncatus*) reproduction in the Mississippi Sound. Marine Mammal Science 26(3):707–715. doi: 10.1111/j.1748-7692.2010.00371.x

Miller, L.J., A.D. Mackey, M. Solangi, and S.A. Kuczaj. 2013. Population abundance and habitat utilization of bottlenose dolphins in the Mississippi Sound. Aquatic Conservation: Marine and Freshwater Ecosystems 23(1):145–151. doi: 10.1002/aqc.2278

Mullin, K.D., T. McDonald, R.S. Wells, B.C. Balmer, T. Speakman, C. Sinclair, E.S. Zolman, F. Hornsby, S.M. McBride, K.A. Wilkinson, and L.H. Schwacke. 2017. Density, abundance, survival, and ranging patterns of common bottlenose dolphins (*Tursiops truncatus*) in Mississippi Sound following the Deepwater Horizon oil spill. PLoS ONE 12(10):e0186265. doi: 10.1371/journal.pone.0186265

Perrin, W.F. and S.B. Reilly. 1984. Reproductive parameters of dolphins and small whales of the family Delphinidae. In: Perrin, W.F., R.L. Brownell, Jr., and D.P. DeMaster (Eds.), Reproduction in Whales, Dolphins, and Porpoises. pp. 97–134.

Phillips, N.M. and P.E. Rosel. 2014. A method for prioritizing research on common bottlenose dolphin stocks through evaluating threats and data availability: Development and application to bay, sound and estuary stocks in Texas. National Oceanic and Atmospheric Administration, National Marine Fisheries Service, Southeast Fisheries Science Center: Lafayette, LA. NOAA Technical Memorandum NMFS-SEFSC-665. 146 p. doi: 10.7289/V5F769H8

Rosel, P.E., L.A. Wilcox, C. Sinclair, T.R. Speakman, M.C. Tumlin, J.A. Litz, and E.S. Zolman. 2017. Genetic assignment to stock of stranded common bottlenose dolphins in southeastern Louisiana after the Deepwater Horizon oil spill. Endangered Species Research 33:221–234. doi: 10.3354/esr00780

Rossman, S., P.H. Ostrom, M. Stolen, N.B. Barros, H. Gandhi, C.A. Stricker, and R.S. Wells. 2015. Individual specialization in the foraging habits of female bottlenose dolphins living in a trophically diverse and habitat rich estuary. Oecologia 178(2):415–425. doi: 10.1007/s00442-015-3241-6

Scott, M.D. and S.J. Chivers. 1990. Distribution and herd structure of bottlenose dolphins in the eastern tropical Pacific Ocean. In: Leatherwood, S. and R.R. Reeves (Eds.), The Bottlenose Dolphin. Academic Press, San Diego, CA. pp. 387–402.

Sellas, A.B., R.S. Wells, and P.E. Rosel. 2005. Mitochondrial and nuclear DNA analyses reveal fine scale geographic structure in bottlenose dolphins (*Tursiops truncatus*) in the Gulf of Mexico. Conservation Genetics 6(5):715–728. doi: 10.1007/s10592-005-9031-7

Sergeant, D.E., D.K. Caldwell, and M.C. Caldwell. 1973. Age, growth, and maturity of bottlenosed dolphin (*Tursiops-truncatus*) from northeast Florida. Journal of the Fisheries Research Board of Canada 30(7):1009–1011. doi: 10.1139/f73-165

Shane, S.H. 1990. Behavior and ecology of the bottlenose dolphin at Sanibel Island, Florida. In: Leatherwood, S. and R.R. Reeves (Eds.), The Bottlenose Dolphin. Academic Press, San Diego, CA. pp. 245–265. doi: 10.1016/B978-0-12-440280-5.50016-0

Shane, S.H. 2004. Residence patterns, group characteristics, and association patterns of bottlenose dolphins near Sanibel Island, Florida. Gulf of Mexico Science 22(1):1–12. doi: 10.18785/goms.2201.01

Shane, S.H., R.S. Wells, and B. Würsig. 1986. Ecology, behavior and social organization of the bottlenose dolphin: A review. Marine Mammal Science 2:34–63. doi: 10.1111/j.1748-7692.1986.tb00026.x

Takeshita, R., B.C. Balmer, F. Messina, E.S. Zolman, L. Thomas, R.S. Wells, C.R. Smith, T.K. Rowles, and L.H. Schwacke. 2021. High site-fidelity in common bottlenose dolphins despite low salinity exposure and associated indicators of compromised health. PLoS ONE 16(9):e0258031. doi: 10.1371/journal.pone.0258031

Taylor, B.L., S.J. Chivers, J. Larese, and W.F. Perrin. 2007. Generation length and percent mature estimates for IUCN assessments of cetaceans. National Oceanic and Atmospheric Administration, National Marine Fisheries Service, Southwest Fisheries Science Center: La Jolla, CA. Administrative Report LJ-07-01. 24 p.

Toms, C. 2019. Filling the gaps: Common bottlenose dolphin (*Tursiops truncatus*) population dynamics, structure, and connectivity within Florida panhandle bays, sounds, and estuaries. University of Central Florida. Dissertation. 373 p.

Torres, L.G. and A.J. Read. 2009. Where to catch a fish? The influence of foraging tactics on the ecology of bottlenose dolphins (*Tursiops truncatus*) in Florida Bay, Florida. Marine Mammal Science 25(4):797–815. doi: 10.1111/j.1748-7692.2009.00297.x

Tyson, R.B., S.M. Nowacek, and D.P. Nowacek. 2011. Community structure and abundance of bottlenose dolphins *Tursiops truncatus* in coastal waters of the northeast Gulf of Mexico. Marine Ecology Progress Series 438:253–265. doi: 10.3354/meps09292

Urian, K.W., D.A. Duffield, A.J. Read, R.S. Wells, and E.D. Shell. 1996. Seasonality of reproduction in bottlenose dolphins, *Tursiops truncatus*. Journal of Mammalogy 77(2):394–403. doi: 10.2307/1382814

Urian, K.W., S. Hofmann, R.S. Wells, and A.J. Read. 2009. Fine-scale population structure of bottlenose dolphins (*Tursiops truncatus*) in Tampa Bay, Florida. Marine Mammal Science 25(3):619–638. doi: 10.1111/j.1748-7692.2009.00284.x

Vollmer, N.L. and P.E. Rosel. 2013. A review of common bottlenose dolphins (*Tursiops truncatus truncatus*) in the Northern Gulf of Mexico: Population biology, potential threats, and management. Southeastern Naturalist 12(m6):1–43. doi: 10.1656/058.012.m601

Weller, D.W. 1998. Global and regional variation in the biology and behavior of bottlenose dolphins. Texas A&M University. Dissertation. 142 p.

Wells, R.S. 1986a. Population structure of bottlenose dolphins: Behavioral studies along the central west coast of Florida. Contract Report to National Marine Fisheries Service, Southeast Fisheries Center. Contract No. 45-WCNF-5-003BB. 58 p.

Wells, R.S. 1986b. Structural aspects of dolphin societies. University of California, Santa Cruz. Dissertation. 234 p.

Wells, R.S. 1991. The role of long-term study in understanding the social structure of a bottlenose dolphin community. In: Pryor, K. and K. Norris (Eds.), Dolphin Societies: Discoveries and Puzzles. University of California Press, Berkeley, CA. pp. 199–226.

Wells, R.S. 2000. Reproduction in wild bottlenose dolphins: Overview of patterns observed during a long-term study. In: Duffield, D. and T. Robeck (Eds.), Bottlenose Dolphin Reproduction Workshop. AZA Marine Mammal Taxon Advisory Group, Silver Spring, MD. pp. 57–74.

Wells, R.S. 2003. Dolphin social complexity: Lessons from long-term study and life history. In: de Waal, F.B.M. and P.L. Tyack (Eds.), Animal Social Complexity: Intelligence, Culture, and Individualized Societies Harvard University Press, Cambridge, MA. pp. 32–56.

Wells, R.S. 2014. Social structure and life history of bottlenose dolphins near Sarasota Bay, Florida: Insights from four decades and five generations. Primates and cetaceans. pp. 149–172. doi: 10.1007/978-4-431-54523-1_8

Wells, R.S., M.K. Bassos, K.W. Urian, W.J. Carr, and M.D. Scott. 1996. Low-level monitoring of bottlenose dolphins, *Tursiops truncatus*, in Charlotte Harbor, Florida: 1990-1994. National Oceanic and Atmospheric Administration, National Marine Fisheries Service, Southeast Fisheries Science Center: Miami, FL. NOAA Technical Memorandum NMFS-SEFSC-384. 86 p.

Wells, R.S., M.K. Bassos, K.W. Urian, S.H. Shane, E.C.G. Owen, C.F. Weiss, W.J. Carr, and M.D. Scott. 1997. Low-level monitoring of bottlenose dolphins, *Tursiops truncatus*, in Pine Island Sound, Florida: 1996. National Oceanic and Atmospheric Administration, National Marine Fisheries Service, Southeast Fisheries Science Center: Miami, FL. Southeast Fisheries Center Contribution No. 40-WCNF601958. 86 p.

Wells, R.S., A. Natoli, and G. Braulik. 2019. *Tursiops truncatus* (errata version published in 2019). *The IUCN Red List of Threatened Species* 2019: e.T22563A156932432. https://dx.doi.org/10.2305/IUCN.UK.2019-1.RLTS.T22563A156932432.en

Wells, R.S., L.H. Schwacke, T.K. Rowles, B.C. Balmer, E. Zolman, T. Speakman, F.I. Townsend, M.C. Tumlin, A. Barleycorn, and K.A. Wilkinson. 2017. Ranging patterns of common bottlenose dolphins *Tursiops truncatus* in Barataria Bay, Louisiana, following the Deepwater Horizon oil spill. Endangered Species Research 33:159–180. doi: 10.3354/esr00732

Wells, R.S. and M.D. Scott. 1999. Bottlenose dolphin *Tursiops truncatus* (Montagu, 1821). In: Ridgway, S.H. and R. Harrison (Eds.), Handbook of marine mammals. Volume 6: The second book of dolphins and the porpoises. Academic Press, San Diego, CA. pp. 137–182.

Wells, R.S. and M.D. Scott. 2018. Bottlenose dolphin, *Tursiops truncatus*, common bottlenose dolphin. In: Würsig, B., J.G.M. Thewissen, and K.M. Kovacs (Eds.), Encyclopedia of Marine Mammals (Third Edition). Academic Press, London, UK. pp. 118–125. doi: 10.1016/B978-0-12-804327-1.00072-8

Wells, R.S., M.D. Scott, and A.B. Irvine. 1987. The social structure of free-ranging bottlenose dolphins. In: Genoways, H.H. (Ed.), Current Mammalogy. Plenum Press, New York, NY. pp. 247–305.

Wells, R.S., K.W. Urian, A.J. Read, M.K. Bassos, W.J. Carr, and M.D. Scott. 1996. Low-level monitoring of bottlenose dolphins, *Tursiops truncatus*, in Tampa Bay, Florida: 1988-1993. National Oceanic and Atmospheric Administration, National Marine Fisheries Service, Southeast Fisheries Science Center: Miami, FL. NOAA Technical Memorandum NMFS-SEFSC-385. 36 p.

Wilson, R.M., R.B. Tyson, J.A. Nelson, B.C. Balmer, J.P. Chanton, and D.P. Nowacek. 2017. Niche differentiation and prey selectivity among common bottlenose dolphins (*Tursiops truncatus*) sighted in St. George Sound, Gulf of Mexico. Frontiers in Marine Science 4:235. doi: 10.3389/fmars.2017.00235

Common bottlenose dolphin − *Tursiops truncatus*

GoMx BSE/Pensacola Bay, East Bay Stock

Overall Vulnerability Rank = Very High

Biological Sensitivity = Very High

Climate Exposure = Very High

Data Quality = 93% of scores ≥ 2

	Tursiops truncatus	Expert Scores	Data Quality	Expert Scores Plots (Portion by Category)
Sensitivity attributes	Prey/Diet Specificity	2.1	2.0	
	Habitat Specificity	3.7	2.3	
	Site Fidelity	3.9	2.3	
	Lifetime Reproductive Potential	1.2	2.0	
	Generation Length	2.9	2.0	
	Reproductive Plasticity	1.4	2.0	
	Migration	3.7	2.3	
	Home Range	3.5	2.3	
	Stock Abundance	3.1	2.7	
	Stock Abundance Trend	2.2	1.7	
	Cumulative Stressors	3.6	2.3	
	Sensitivity Score	Very High		
Exposure variables	Sea Surface Temperature (Change in mean)	4.0	2.7	
	Sea Surface Temperature (Change in variability)	1.0	2.7	
	Air Temperature (Change in mean)	4.0	2.7	
	Air Temperature (Change in variability)	1.0	2.7	
	Precipitation (Change in mean)	1.0	2.7	
	Precipitation (Change in variability)	1.0	2.7	
	Sea Surface Salinity (Change in mean)	2.0	2.7	
	Sea Surface Salinity (Change in variability)	1.3	2.7	
	Ocean pH (Change in mean)	4.0	2.7	
	Ocean pH (Change in variability)	1.0	2.7	
	Sea ice coverage (Change in mean)	1.0	2.7	
	Sea ice coverage (Change in variability)	1.0	2.7	
	Dissolved oxygen (Change in mean)	4.0	2.7	
	Dissolved oxygen (Change in variability)	1.0	2.7	
	Circulation	3.9	1.7	
	Sea level rise	4.0	2.7	
	Exposure Score	Very High		
Overall Vulnerability Rank		Very High		

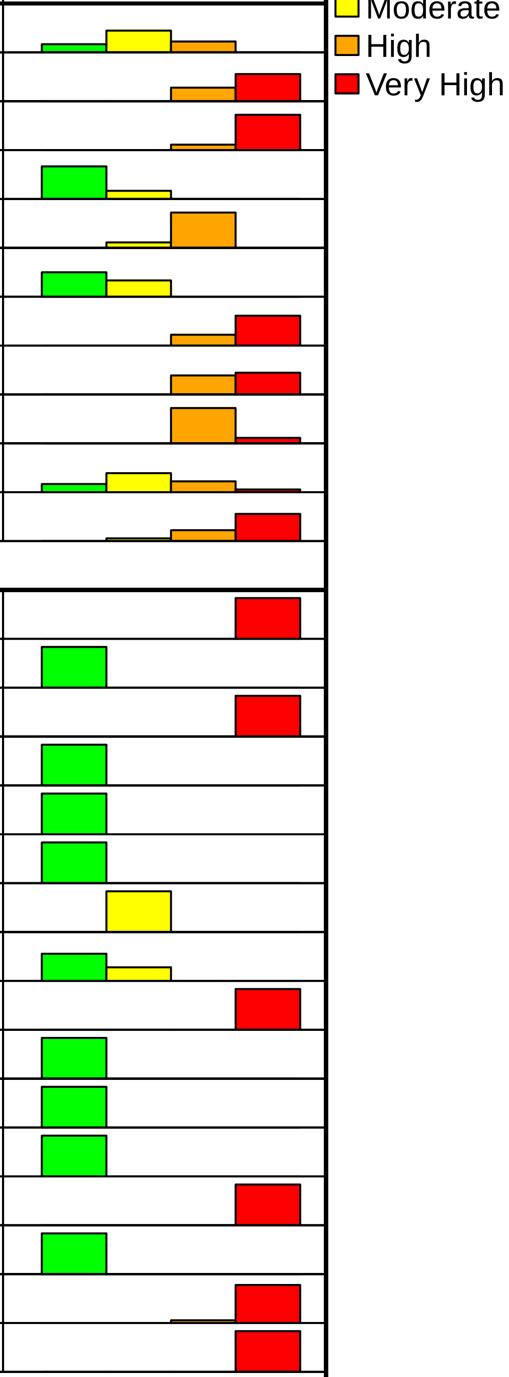

Common bottlenose dolphin (GoMx BSE/Pensacola Bay, East Bay Stock)

Tursiops truncatus

CVA Results Summary

Overall Climate Vulnerability Rank: Very High (100% certainty from bootstrap analysis).

Climate Exposure: Very High. Six exposure factors scored greater than 3.5: Air Temperature (Standard anomaly) (4.00), Dissolved oxygen (Standard anomaly) (4.00), Ocean pH (Standard anomaly) (4.00), Sea level rise (4.00), Sea Surface Temperature (Standard anomaly) (4.00), and Circulation (3.93).

Biological Sensitivity: Very High. Five sensitivity attributes scored greater than 3.5: Site Fidelity (3.87), Migration (3.73), Habitat Specificity (3.67), Cumulative Stressors (3.60), and Home Range (3.53).

Distributional Response: Very High

Abundance Response: Very High

Phenology Response: Very High

Data Quality: 93% of the data quality scores were 2 or greater, 91% of sensitivity attribute data quality scores were 2 or higher.

Background Information

Stock Description

This stock includes common bottlenose dolphins in Pensacola Bay and East Bay in the northern Gulf of Mexico along the Florida coast (Hayes et al. 2022).

Prey/Diet Specificity

Generally, common bottlenose dolphins consume a large variety of fish and/or squid (Barros and Odell 1990; Mead and Potter 1990; Wells and Scott 2018). Common bottlenose dolphins seem to show a consistent preference for sound-producing fishes (e.g., sciaenids, toadfish; Berens McCabe et al. 2010; Bowen-Stevens et al. 2021). In Sarasota Bay, Florida, Rossman et al. (2015) found some individual common bottlenose dolphins to show diet specialization despite the broader study group showing a generalist diet. Specialization can be influenced by locally abundant prey species (Shane et al. 1986; Vollmer and Rosel 2013) or can result from resource partitioning amongst sympatric populations (e.g., Torres and Read 2009; Wilson et al. 2017).

Habitat Specificity

Common bottlenose dolphins are found in a variety of marine and estuarine habitats (Wells and Scott 2018).

Site Fidelity

Toms (2019) reported that 70% of dolphins sighted during a capture-mark-recapture photo-ID study carried out over a 2.5 year period (June 2013 - January 2016) were seen more than once in the study area. In that study, 43% of all dolphins identified were seen in just one season and 30% of these were seen only once. 26% of dolphins sighted were seen across two seasons, 20% across three seasons, and 12% were seen in all four seasons.

Common bottlenose dolphins in other Gulf of Mexico bay, sound, and estuary (BSE) stocks show site fidelity (LaBrecque et al. 2015; Hayes et al. 2022), such as in Matagorda Bay, Espiritu Santo Bay, and San Luis Pass in Texas (Maze and Würsig 1999; Lynn and Würsig 2002; Irwin and Würsig 2004); Barataria Bay in Louisiana (Miller 2003; McDonald et al. 2017; Wells et al. 2017; Takeshita et al. 2021); Mississippi Sound in Mississippi (Hubard et al. 2004; Mackey 2010; Mullin et al. 2017); Charlotte Harbor and Pine Island Sound (Bassos-Hull et al. 2013), Sarasota Bay (Wells 1986a; Wells 1991; Wells 2014), St. Andrew Bay (Balmer et al. 2019), St. Joseph Bay (Balmer et al. 2008; B. Balmer et al. 2018), St. Vincent Sound and Apalachicola Bay (Tyson et al. 2011), and Tampa Bay in Florida (Wells et al. 1996b; Urian et al. 2009).

Lifetime Reproductive Potential

Taylor et al. (2007) reported the interbirth interval of common bottlenose dolphins as 3.80 years, though other studies have shown it to be closer to three years (Leatherwood 1977; Wells and Scott 1999; Connor et al. 2000; Wells 2003).

Generally, females reach sexual maturity before males, with females reaching sexual maturity at 5–13 years (Sergeant et al. 1973; Perrin and Reilly 1984; Wells et al. 1987; Mead and Potter 1990; Mann et al. 2000; Wells 2000; Wells and Scott 2018).

Females reproduce through most of their adult lives (Leatherwood 1977; Hohn et al. 1989; Wells and Scott 1999; Wells 2000; Wells 2003), but may produce fewer calves after they reach 30 years of age (Wells 2000). Sarasota Bay common bottlenose dolphins can give birth when they are as young as 6 years of age, and can continue until they are 48 years old (Wells and Scott 2018). Reproductive lifespan would therefore be up to 42 years based on long-term observation studies (Wells 2014; Wells and Scott 2018).

Longitudinal studies from Sarasota Bay, Florida have found that female common bottlenose dolphins can live up to 67 years (Wells and Scott 2018). Studies based on growth layer groups in teeth suggest female common bottlenose dolphins can live to 41 years along the Texas coast (Fernandez and Hohn 1998) and to 30 years along the Mississippi coast (Mattson et al. 2006).

Generation Length

Taylor et al. (2007) reported a common bottlenose dolphin generation length of 20.6–21.1 years.

Reproductive Plasticity

Common bottlenose dolphin calving is generally diffusely seasonal, with one or more peaks, although births have been reported from all seasons (Vollmer and Rosel 2013; Wells and Scott 2018). Calf

sightings peak in the spring and summer months in Florida (Wells et al. 1987), Mississippi/Louisiana (Miller et al. 2010; Miller et al. 2013), and Texas (McHugh 1989; Henderson et al. 2007).

Migration

The bay, sound, and estuary stocks in the Gulf of Mexico are thought to primarily comprise stable resident communities. However, transient or seasonal movements of some individuals into and out of a BSE stock area are recorded in some areas (LaBrecque et al. 2015; Hayes et al. 2022). Molecular genetic data support that some BSE stocks are genetically distinct from one another, and furthermore are differentiated from adjacent coastal populations (e.g., Sellas et al. 2005; Rosel et al. 2017). For example, Toms (2019) found fine-scale genetic population structure among five inshore systems in the Florida Panhandle and variable migration rates among populations.

Year-round residency has been reported for common bottlenose dolphins (Hayes et al. 2022), such as in Florida (Irvine and Wells 1972; Irvine et al. 1981; Wells 1986a; Wells 1986b; Wells et al. 1987; Scott et al. 1990; Wells 1991; Wells et al. 1996a; Wells et al. 1996b; Wells et al. 1997; Wells 2003; Balmer et al. 2008; Urian et al. 2009; Tyson et al. 2011; Bassos-Hull et al. 2013; B. Balmer et al. 2018; Balmer et al. 2019; Toms 2019), Texas (Shane 1990; Bräger 1993; Bräger et al. 1994; Fertl 1994; Weller 1998; Maze and Würsig 1999; Lynn and Würsig 2002; Irwin and Würsig 2004; Shane 2004), and Louisiana/Mississippi (Hubard et al. 2004; McDonald et al. 2017; Mullin et al. 2017; Takeshita et al. 2021).

Home Range

The home ranges of individuals within the Gulf of Mexico BSE stocks are considered to generally include the areas within the stock boundaries and brief excursions beyond the stock boundaries (Irvine et al. 1981; Wells 1986a; Wells 1986b; Wells 1991; Wells et al. 1996a; Wells et al. 1996b; Balmer et al. 2008; Wells 2014; Balmer et al. 2019), although some individuals show preference for small areas within the stock boundaries (Lynn and Würsig 2002; Urian et al. 2009; Mullin et al. 2017; Wells et al. 2017).

Stock Abundance

Blaylock and Hoggard (1994) estimated this stock’s abundance at 33 individuals (CV = 0.80). More recently, Toms (2019) completed a photo-ID mark-recapture analysis and estimated this stock’s abundance to be between 222 (95% CI: 173–286; Spring 2015) and 309 (95% CI: 211–453; Winter 2013/2014) individuals; 43% of individuals were considered to be transients.

Stock Abundance Trend

Data are insufficient to complete an abundance trend analysis for this stock (Hayes et al. 2022).

Cumulative Stressors

Common bottlenose dolphins face a wide range of individual and cumulative natural and anthropogenic threats (see reviews by Vollmer and Rosel 2013; Wells and Scott 2018; and Wells et al. 2019). These include such threats as shark predation, harmful algal blooms, recreational and commercial fishing gear, vessel traffic, pollution, habitat alteration, and provisioning.

Common bottlenose dolphins in Pensacola Bay and East Bay were possibly exposed to oil and dispersants following the Deepwater Horizon oil spill, which may result in long term reproductive and chronic health effects (NOAA 2011; Michel et al. 2013; Helm et al. 2015; DWH NRDA 2016; Lane et al. 2015; De Guise et al. 2017, 2021; Kellar et al. 2017; Smith et al. 2017; Schwacke et al. 2017). An Unusual Mortality Event (UME), involving primarily bottlenose dolphins, was declared for cetaceans in the northern Gulf of Mexico from 2010 to 2014, with the Deepwater Horizon oil spill as the primary underlying cause (Litz et al. 2014; Schwacke et al. 2014; Venn-Watson et al. 2015a, 2015b).

The nearshore and estuarine habitats along the Gulf of Mexico coast include waters adjacent to areas of high human population, some of which are highly industrialized, and areas of heavy agricultural impact. Studies of bottlenose dolphin tissues from the northern Gulf of Mexico have shown persistent organic pollutants (POPs) in blubber and blood samples, though at concentrations lower than other southeastern U.S. sites (Kucklick et al. 2011; Balmer et al. 2015; J. Balmer et al. 2018). Similarly, mercury has been detected in common bottlenose dolphin tissues in Florida waters (McCormack et al. 2020a, 2020b, 2022).

UMEs have been declared for common bottlenose dolphins in waters of the Florida panhandle with biotoxins (e.g., brevetoxin from *Karenia brevis*) the suspected or confirmed cause (Twiner et al. 2012; Litz et al. 2014). Dolphin morbillivirus has been detected in populations of the northern Gulf of Mexico, but has not been attributed to large scale die-offs since 1993−1994 (Lipscomb et al. 1996; Litz et al. 2014; Van Bressem et al. 2014; Fauquier et al. 2017). A UME was declared in 1990 for the Gulf of Mexico from Florida to Texas, with more than 300 common bottlenose dolphins stranding due to an undetermined cause, although morbillivirus and cold temperatures may have contributed (Litz et al. 2014).

Further Reading

Hayes, S.A., E. Josephson, K. Maze-Foley, P.E. Rosel, and J. Wallace. 2022. U.S. Atlantic and Gulf of Mexico marine mammal stock assessments 2021. National Oceanic and Atmospheric Administration, National Marine Fisheries Service, Northeast Fisheries Science Center: Woods Hole, MA. NOAA Technical Memorandum NMFS-NE-288. 380 p. doi: 10.25923/6tt7-kc16

Wells, R.S. and M.D. Scott. 2018. Bottlenose Dolphin, *Tursiops truncatus*, Common Bottlenose Dolphin. In: Würsig, B., J.G.M. Thewissen, and K.M. Kovacs (Eds.), Encyclopedia of Marine Mammals (Third Edition). Academic Press, London, UK. pp. 118–125. doi: 10.1016/B978-0-12-804327-1.00072-8

Wells, R.S., A. Natoli, and G. Braulik. 2019. *Tursiops truncatus* (errata version published in 2019). The IUCN Red List of Threatened Species 2019: e.T22563A156932432. https://dx.doi.org/10.2305/IUCN.UK.2019-1.RLTS.T22563A156932432.en

References

Balmer, B., T. McDonald, F. Hornsby, J. Adams, J. Allen, A. Barleycorn, and L. Schwacke. 2018. Long-term trends in a northern Gulf of Mexico common bottlenose dolphin (*Tursiops truncatus*) population in the wake of the Deepwater Horizon oil spill. Journal of Cetacean Research and Management 18:1–9. doi: 10.47536/jcrm.v18i1.409

Balmer, B., S. Watwood, B. Quigley, T. Speakman, K. Barry, K. Mullin, P. Rosel, C. Sinclair, E. Zolman, and L. Schwacke. 2019. Common bottlenose dolphin (*Tursiops truncatus*) abundance and distribution

patterns in St. Andrew Bay, Florida, USA. Aquatic Conservation: Marine and Freshwater Ecosystems 29(3):486–498. doi: 10.1002/aqc.3001

Balmer, B.C., R.S. Wells, S.M. Nowacek, D.P. Nowacek, L.H. Schwacke, W.A. McLellan, F.S. Scharf, T.K. Rowles, L.J. Hansen, T.R. Spradlin, and D.A. Pabst. 2008. Seasonal abundance and distribution patterns of common bottlenose dolphins (*Tursiops truncatus*) near St. Joseph Bay, Florida, USA. Journal of Cetacean Research and Management 10(2):157–167.

Balmer, B.C., G.M. Ylitalo, L.E. McGeorge, K.A. Baugh, D. Boyd, K.D. Mullin, P.E. Rosel, C. Sinclair, R.S. Wells, E.S. Zolman, and L.H. Schwacke. 2015. Persistent organic pollutants (POPs) in blubber of common bottlenose dolphins (*Tursiops truncatus*) along the northern Gulf of Mexico coast, USA. Science of the Total Environment 527-528:306–312. doi: 10.1016/j.scitotenv.2015.05.016

Balmer, J.E., G.M. Ylitalo, T.K. Rowles, K.D. Mullin, R.S. Wells, F.I. Townsend, R.W. Pearce, J.L. Bolton, E.S. Zolman, B.C. Balmer, and L.H. Schwacke. 2018. Persistent organic pollutants (POPs) in blood and blubber of common bottlenose dolphins (*Tursiops truncatus*) at three northern Gulf of Mexico sites following the Deepwater Horizon oil spill. Science of the Total Environment 621:130–137. doi: 10.1016/j.scitotenv.2017.11.209

Barros, N.B. and D.K. Odell. 1990. Food habits of bottlenose dolphins in the southeastern United States In: Leatherwood, S. and R.R. Reeves (Eds.), The Bottlenose Dolphin. Academic Press, San Diego, CA. pp. 309–328. doi: 10.1016/B978-0-12-440280-5.50020-2

Bassos-Hull, K., R.M. Perrtree, C.C. Shepard, S. Schilling, A.A. Barleycorn, J.B. Allen, B.C. Balmer, W.E. Pine, and R.S. Wells. 2013. Long-term site fidelity and seasonal abundance estimates of common bottlenose dolphins (*Tursiops truncatus*) along the southwest coast of Florida and responses to natural perturbations. Journal of Cetacean Research and Management 13(1):19–30. doi: 10.47536/jcrm.v13i1.551

Berens McCabe, E.J., D.P. Gannon, N.B. Barros, and R.S. Wells. 2010. Prey selection by resident common bottlenose dolphins (*Tursiops truncatus*) in Sarasota Bay, Florida. Marine Biology 157(5):931–942. doi: 10.1007/s00227-009-1371-2

Blaylock, R.A. and W. Hoggard. 1994. Preliminary estimates of bottlenose dolphin abundance in southern U.S. Atlantic and Gulf of Mexico continental shelf waters. National Oceanic and Atmospheric Administration, National Marine Fisheries Service, Southeast Fisheries Science Center: Miami, FL. NOAA Technical Memorandum NMFS-SEFSC-356. 10 p.

Bowen-Stevens, S.R., D.P. Gannon, R.A. Hazelkorn, G. Lovewell, K.M. Volker, S. Smith, M.C. Tumlin, and J. Litz. 2021. Diet of common bottlenose dolphins, *Tursiops truncatus*, that stranded in and near Barataria Bay, Louisiana, 2010–2012. Southeastern Naturalist 20(1). doi: 10.1656/058.020.0113

Bräger, S. 1993. Diurnal and seasonal behavior patterns of bottlenose dolphins (*Tursiops truncatus*). Marine Mammal Science 9:434–438. doi: 10.1111/j.1748-7692.1993.tb00477.x

Bräger, S., B. Würsig, A. Acevedo, and T. Henningsen. 1994. Association patterns of bottle-nosed dolphins (*Tursiops-truncatus*) in Galveston Bay, Texas. Journal of Mammalogy 75(2):431–437. doi: 10.2307/1382564

Connor, R.C., R.S. Wells, J. Mann, and A.J. Read. 2000. The bottlenose dolphin: Social relationships in a fission-fusion society. In: Mann, J., R. Connor, P. Tyack, and H. Whitehead (Eds.), Cetacean Societies: Field Studies of Dolphins and Whales. University of Chicago Press, Chicago, IL. pp. 91–126.

De Guise, S., M. Levin, E. Gebhard, L. Jasperse, L. Burdett Hart, C.R. Smith, S. Venn-Watson, F. Townsend, R. Wells, B. Balmer, E. Zolman, T. Rowles, and L. Schwacke. 2017. Changes in immune functions in bottlenose dolphins in the northern Gulf of Mexico associated with the Deepwater Horizon oil spill. Endangered Species Research 33:291–303. doi: 10.3354/esr00814

De Guise, S., M. Levin, L. Jasperse, J. Herrman, R.S. Wells, T. Rowles, and L. Schwacke. 2021. Long-term immunological alterations in bottlenose dolphin a decade after the Deepwater Horizon oil spill

in the northern Gulf of Mexico: Potential for multigenerational effects. Environmental Toxicology and Chemistry 40(5):1308–1321. doi: 10.1002/etc.4980

Deepwater Horizon Natural Resource Damage Assessment Trustees (DWH NRDA). 2016. Deepwater Horizon oil spill: Final Programmatic Damage Assessment and Restoration Plan and Final Programmatic Environmental Impact Statement. Retrieved from: http://www.gulfspillrestoration.noaa.gov/restoration-planning/gulf-plan.1659.

Fauquier, D.A., J. Litz, S. Sanchez, K. Colegrove, L.H. Schwacke, L. Hart, J. Saliki, C. Smith, T. Goldstein, S. Bowen-Stevens, W. McFee, E. Fougeres, B. Mase-Guthrie, E. Stratton, R. Ewing, S. Venn-Watson, R.H. Carmichael, C. Clemons-Chevis, W. Hatchett, D. Shannon, S. Shippee, S. Smith, L. Staggs, M.C. Tumlin, N.L. Wingers, and T.K. Rowles. 2017. Evaluation of morbillivirus exposure in cetaceans from the northern Gulf of Mexico 2010-2014. Endangered Species Research 33:211–220. doi: 10.3354/esr00772

Fernandez, S. and A.A. Hohn. 1998. Age, growth, and calving season of bottlenose dolphins, *Tursiops truncatus*, off coastal Texas. Fishery Bulletin 96(2):357–365.

Fertl, D. 1994. Occurrence patterns and behavior of bottlenose dolphins (*Tursiops truncatus*) in the Galveston ship channel, Texas. Texas Journal of Science 46(4):299–318.

Hayes, S.A., E. Josephson, K. Maze-Foley, P.E. Rosel, and J. Wallace. 2022. U.S. Atlantic and Gulf of Mexico marine mammal stock assessments 2021. National Oceanic and Atmospheric Administration, National Marine Fisheries Service, Northeast Fisheries Science Center: Woods Hole, MA. NOAA Technical Memorandum NMFS-NE-288. 380 p. doi: 10.25923/6tt7-kc16

Helm, R.C., D.P. Costa, T.D. DeBruyn, T.J. O'Shea, R.S. Wells, and T.M. Williams. 2015. Overview of effects of oil spills on marine mammals. In: Fingas, M. (Ed.), Handbook of oil spill science and technology, First edition. John Wiley & Sons, Inc., Hoboken, NJ. pp. 455–475.

Henderson, E.E. and B. Würsig. 2007. Behavior patterns of bottlenose dolphins in San Luis Pass, Texas. Gulf of Mexico Science 25(2):153–161. doi: 10.18785/goms.2502.06

Hohn, A.A., M.D. Scott, R.S. Wells, J.C. Sweeney, and A.B. Irvine. 1989. Growth layers in teeth from known-age, free-ranging bottlenose dolphins. Marine Mammal Science 5(4):315–342. doi: 10.1111/j.1748-7692.1989.tb00346.x

Hubard, C.W., K. Maze-Foley, K.D. Mullin, and W.W. Schroeder. 2004. Seasonal abundance and site fidelity of bottlenose dolphins (*Tursiops truncatus*) in Mississippi Sound. Aquatic Mammals 30(2):299–310. doi: 10.1578/am.30.2.2004.299

Irvine, A.B., M.D. Scott, R.S. Wells, and J.H. Kaufmann. 1981. Movements and activities of the Atlantic bottlenose dolphin, *Tursiops truncatus*, near Sarasota, Florida. Fishery Bulletin 79(4):671–688.

Irvine, B. and R.S. Wells. 1972. Results of attempts to tag Atlantic bottlenosed dolphins (*Tursiops truncatus*). Cetology 13:1–5.

Irwin, L.-J. and B. Würsig. 2004. A small resident community of bottlenose dolphins, *Tursiops truncatus*, in Texas: Monitoring recommendations. Gulf of Mexico Science 22(1):13–21. doi: 10.18785/goms.2201.02

Kellar, N.M., T.R. Speakman, C.R. Smith, S.M. Lane, B.C. Balmer, M.L. Trego, K.N. Catelani, M.N. Robbins, C.D. Allen, R.S. Wells, E.S. Zolman, T.K. Rowles, and L.H. Schwacke. 2017. Low reproductive success rates of common bottlenose dolphins *Tursiops truncatus* in the northern Gulf of Mexico following the Deepwater Horizon disaster (2010-2015). Endangered Species Research 33:143–158. doi: 10.3354/esr00775

Kucklick, J., L. Schwacke, R. Wells, A. Hohn, A. Guichard, J. Yordy, L. Hansen, E. Zolman, R. Wilson, J. Litz, D. Nowacek, T. Rowles, R. Pugh, B. Balmer, C. Sinclair, and P. Rosel. 2011. Bottlenose dolphins as indicators of persistent organic pollutants in the western North Atlantic Ocean and Northern Gulf of Mexico. Environmental Science & Technology 45(10):4270–4277. doi: 10.1021/es1042244

LaBrecque, E., C. Curtice, J. Harrison, S.M. Van Parijs, and P.N. Halpin. 2015. 3. Biologically Important Areas for cetaceans within U.S. Waters – Gulf of Mexico Region. Aquatic Mammals 41(1):30–38. doi: 10.1578/am.41.1.2015.30

Lane, S.M., C.R. Smith, J. Mitchell, B.C. Balmer, K.P. Barry, T. McDonald, C.S. Mori, P.E. Rosel, T.K. Rowles, T.R. Speakman, F.I. Townsend, M.C. Tumlin, R.S. Wells, E.S. Zolman, and L.H. Schwacke. 2015. Reproductive outcome and survival of common bottlenose dolphins sampled in Barataria Bay, Louisiana, USA, following the Deepwater Horizon oil spill. Proceedings of the Royal Society B: Biological Sciences 282(1818):20151944. doi: 10.1098/rspb.2015.1944

Leatherwood, S. 1977. Some preliminary impressions on the numbers and social behavior of free-swimming bottlenosed dolphin calves (*Tursiops truncatus*) in the northern Gulf of Mexico. In: Ridgway, S.H. and K. Bernirschke (Eds.), Breeding Dolphins; Present Status, Suggestions for the Future. Final report to the US Marine Mammal Commission, Contract MM6AC009, MMC-76/07, Washington, DC. pp. 143–167.

Lipscomb, T.P., S. Kennedy, D. Moffett, A. Krafft, B.A. Klaunberg, J.H. Lichy, G.T. Regan, G.A.J. Worthy, and J.K. Taubenberger. 1996. Morbilliviral epizootic in bottlenose dolphins of the Gulf of Mexico. Journal of Veterinary Diagnostic Investigation 8(3):283–290. doi: 10.1177/104063879600800302

Litz, J.A., M.A. Baran, S.R. Bowen-Stevens, R.H. Carmichael, K.M. Colegrove, L.P. Garrison, S.E. Fire, E.M. Fougeres, R. Hardy, S. Holmes, W. Jones, B.E. Mase-Guthrie, D.K. Odell, P.E. Rosel, J.T. Saliki, D.K. Shannon, S.F. Shippee, S.M. Smith, E.M. Stratton, M.C. Tumlin, H.R. Whitehead, G.A. Worthy, and T.K. Rowles. 2014. Review of historical unusual mortality events (UMEs) in the Gulf of Mexico (1990–2009): providing context for the multi-year northern Gulf of Mexico cetacean UME declared in 2010. Diseases of Aquatic Organisms 112(2):161–175. doi: 10.3354/dao02807

Lynn, S.K. and B. Würsig. 2002. Summer movement patterns of bottlenose dolphins in a Texas bay. Gulf of Mexico Science 20(1):25–37. doi: 10.18785/goms.2001.03

Mackey, A.D. 2010. Site fidelity and association patterns of bottlenose dolphins (*Tursiops truncatus*) in the Mississippi Sound. University of Southern Mississippi. Thesis. 133 p.

Mann, J., R.C. Connor, L.M. Barre, and M.R. Heithaus. 2000. Female reproductive success in bottlenose dolphins (*Tursiops* sp.): life history, habitat, provisioning, and group-size effects. Behavioral Ecology 11(2):210–219. doi: 10.1093/beheco/11.2.210

Mattson, M.C., K.D. Mullin, G.W. Ingram, and W. Hoggard. 2006. Age structure and growth of the bottlenose dolphin (*Tursiops truncatus*) from strandings in the Mississippi sound region of the north-central Gulf of Mexico from 1986 to 2003. Marine Mammal Science 22(3):654–666. doi: 10.1111/j.1748-7692.2006.00057.x

Maze, K.S. and B. Würsig. 1999. Bottlenosed dolphins of San Luis Pass, Texas: Occurrence patterns, site-fidelity, and habitat use. Aquatic Mammals 25:91–104.

McCormack, M.A., F. Battaglia, W.E. McFee, and J. Dutton. 2020a. Mercury concentrations in blubber and skin from stranded bottlenose dolphins (*Tursiops truncatus*) along the Florida and Louisiana coasts (Gulf of Mexico, USA) in relation to biological variables. Environmental Research 180:108886. doi: 10.1016/j.envres.2019.108886

McCormack, M.A., B.P. Jackson, and J. Dutton. 2020b. Relationship between mercury and selenium concentrations in tissues from stranded odontocetes in the northern Gulf of Mexico. Science of the Total Environment 749:141350. doi: 10.1016/j.scitotenv.2020.141350

McCormack, M.A., W.H. Nowlin, and J. Dutton. 2022. Effect of trophic position on mercury concentrations in bottlenose dolphins (*Tursiops truncatus*) from the northern Gulf of Mexico. Environmental Research 204(Pt B):112124. doi: 10.1016/j.envres.2021.112124

McDonald, T.L., F.E. Hornsby, T.R. Speakman, E.S. Zolman, K.D. Mullin, C. Sinclair, P.E. Rosel, L. Thomas, and L.H. Schwacke. 2017. Survival, density, and abundance of common bottlenose dolphins in

Barataria Bay (USA) following the Deepwater Horizon oil spill. Endangered Species Research 33:193–209. doi: 10.3354/esr00806

McHugh, M.B. 1989. Population numbers and feeding behavior of the Atlantic bottlenose dolphin (*Tursiops truncatus*) near Aransas Pass, Texas. University of Texas at Austin. Thesis. 96 p.

Mead, J.G. and C.W. Potter. 1990. Natural history of bottlenose dolphins along the central Atlantic coast of the United States. In: Leatherwood, S. and R.R. Reeves (Eds.), The Bottlenose Dolphin. Academic Press, San Diego, CA. pp. 165–195.

Michel, J., E.H. Owens, S. Zengel, A. Graham, Z. Nixon, T. Allard, W. Holton, P.D. Reimer, A. Lamarche, M. White, N. Rutherford, C. Childs, G. Mauseth, G. Challenger, and E. Taylor. 2013. Extent and degree of shoreline oiling: Deepwater Horizon oil spill, Gulf of Mexico, USA. PLoS ONE 8(6):e65087. doi: 10.1371/journal.pone.0065087

Miller, C.E. 2003. Abundance trends and environmental habitat usage patterns of bottlenose dolphins (*Tursiops truncatus*) in lower Barataria and Caminada bays, Louisiana. Louisiana State University and Agricultural & Mechanical College. Dissertation. 125 p.

Miller, L.J., A.D. Mackey, T. Hoffland, M. Solangi, and S.A. Kuczaj. 2010. Potential effects of a major hurricane on Atlantic bottlenose dolphin (*Tursiops truncatus*) reproduction in the Mississippi Sound. Marine Mammal Science 26(3):707–715. doi: 10.1111/j.1748-7692.2010.00371.x

Miller, L.J., A.D. Mackey, M. Solangi, and S.A. Kuczaj. 2013. Population abundance and habitat utilization of bottlenose dolphins in the Mississippi Sound. Aquatic Conservation: Marine and Freshwater Ecosystems 23(1):145–151. doi: 10.1002/aqc.2278

Mullin, K.D., T. McDonald, R.S. Wells, B.C. Balmer, T. Speakman, C. Sinclair, E.S. Zolman, F. Hornsby, S.M. McBride, K.A. Wilkinson, and L.H. Schwacke. 2017. Density, abundance, survival, and ranging patterns of common bottlenose dolphins (*Tursiops truncatus*) in Mississippi Sound following the Deepwater Horizon oil spill. PLoS ONE 12(10):e0186265. doi: 10.1371/journal.pone.0186265

National Oceanic and Atmospheric Administration (NOAA). 2011. Public scoping for preparation of a programmatic environmental impact statement for the Deepwater Horizon BP Oil Spill. 9 p.

Perrin, W.F. and S.B. Reilly. 1984. Reproductive parameters of dolphins and small whales of the family Delphinidae. In: Perrin, W.F., R.L. Brownell, Jr., and D.P. DeMaster (Eds.), Reproduction in Whales, Dolphins, and Porpoises. pp. 97–134.

Rosel, P.E., L.A. Wilcox, C. Sinclair, T.R. Speakman, M.C. Tumlin, J.A. Litz, and E.S. Zolman. 2017. Genetic assignment to stock of stranded common bottlenose dolphins in southeastern Louisiana after the Deepwater Horizon oil spill. Endangered Species Research 33:221–234. doi: 10.3354/esr00780

Rossman, S., P.H. Ostrom, M. Stolen, N.B. Barros, H. Gandhi, C.A. Stricker, and R.S. Wells. 2015. Individual specialization in the foraging habits of female bottlenose dolphins living in a trophically diverse and habitat rich estuary. Oecologia 178(2):415–425. doi: 10.1007/s00442-015-3241-6

Schwacke, L.H., C.R. Smith, F.I. Townsend, R.S. Wells, L.B. Hart, B.C. Balmer, T.K. Collier, S. De Guise, M.M. Fry, L.J. Guillette, Jr., S.V. Lamb, S.M. Lane, W.E. McFee, N.J. Place, M.C. Tumlin, G.M. Ylitalo, E.S. Zolman, and T.K. Rowles. 2014. Health of common bottlenose dolphins (*Tursiops truncatus*) in Barataria Bay, Louisiana, following the deepwater horizon oil spill. Environmental Science & Technology 48(1):93–103. doi: 10.1021/es403610f

Schwacke, L.H., L. Thomas, R.S. Wells, W.E. McFee, A.A. Hohn, K.D. Mullin, E.S. Zolman, B.M. Quigley, T.K. Rowles, and J.H. Schwacke. 2017. Quantifying injury to common bottlenose dolphins from the Deepwater Horizon oil spill using an age-, sex- and class-structured population model. Endangered Species Research 33:265–279. doi: 10.3354/esr00777

Scott, M.D. and S.J. Chivers. 1990. Distribution and herd structure of bottlenose dolphins in the eastern tropical Pacific Ocean. In: Leatherwood, S. and R.R. Reeves (Eds.), The Bottlenose Dolphin. Academic Press, San Diego, CA. pp. 387–402.

Sellas, A.B., R.S. Wells, and P.E. Rosel. 2005. Mitochondrial and nuclear DNA analyses reveal fine scale geographic structure in bottlenose dolphins (*Tursiops truncatus*) in the Gulf of Mexico. Conservation Genetics 6(5):715–728. doi: 10.1007/s10592-005-9031-7

Sergeant, D.E., D.K. Caldwell, and M.C. Caldwell. 1973. Age, growth, and maturity of bottlenosed dolphin (*Tursiops-truncatus*) from northeast Florida. Journal of the Fisheries Research Board of Canada 30(7):1009–1011. doi: 10.1139/f73-165

Shane, S.H. 1990. Behavior and ecology of the bottlenose dolphin at Sanibel Island, Florida. In: Leatherwood, S. and R.R. Reeves (Eds.), The Bottlenose Dolphin. Academic Press, San Diego, CA. pp. 245–265. doi: 10.1016/B978-0-12-440280-5.50016-0

Shane, S.H. 2004. Residence patterns, group characteristics, and association patterns of bottlenose dolphins near Sanibel Island, Florida. Gulf of Mexico Science 22(1):1–12. doi: 10.18785/goms.2201.01

Shane, S.H., R.S. Wells, and B. Würsig. 1986. Ecology, behavior and social organization of the bottlenose dolphin: A review. Marine Mammal Science 2:34–63. doi: 10.1111/j.1748-7692.1986.tb00026.x

Smith, C.R., T.K. Rowles, L.B. Hart, F.I. Townsend, R.S. Wells, E.S. Zolman, B.C. Balmer, B. Quigley, M. Ivančić, W. McKercher, M.C. Tumlin, K.D. Mullin, J.D. Adams, Q. Wu, W. McFee, T.K. Collier, and L.H. Schwacke. 2017. Slow recovery of Barataria Bay dolphin health following the Deepwater Horizon oil spill (2013-2014), with evidence of persistent lung disease and impaired stress response. Endangered Species Research 33:127–142. doi: 10.3354/esr00778

Takeshita, R., B.C. Balmer, F. Messina, E.S. Zolman, L. Thomas, R.S. Wells, C.R. Smith, T.K. Rowles, and L.H. Schwacke. 2021. High site-fidelity in common bottlenose dolphins despite low salinity exposure and associated indicators of compromised health. PLoS ONE 16(9):e0258031. doi: 10.1371/journal.pone.0258031

Taylor, B.L., S.J. Chivers, J. Larese, and W.F. Perrin. 2007. Generation length and percent mature estimates for IUCN assessments of cetaceans. National Oceanic and Atmospheric Administration, National Marine Fisheries Service, Southwest Fisheries Science Center: La Jolla, CA. Administrative Report LJ-07-01. 24 p.

Toms, C. 2019. Filling the gaps: Common bottlenose dolphin (*Tursiops truncatus*) population dynamics, structure, and connectivity within Florida panhandle bays, sounds, and estuaries. University of Central Florida. Dissertation. 373 p.

Torres, L.G. and A.J. Read. 2009. Where to catch a fish? The influence of foraging tactics on the ecology of bottlenose dolphins (*Tursiops truncatus*) in Florida Bay, Florida. Marine Mammal Science 25(4):797–815. doi: 10.1111/j.1748-7692.2009.00297.x

Twiner, M.J., L.J. Flewelling, S.E. Fire, S.R. Bowen-Stevens, J.K. Gaydos, C.K. Johnson, J.H. Landsberg, T.A. Leighfield, B. Mase-Guthrie, L. Schwacke, F.M. Van Dolah, Z. Wang, and T.K. Rowles. 2012. Comparative analysis of three brevetoxin-associated bottlenose dolphin (*Tursiops truncatus*) mortality events in the Florida Panhandle region (USA). PLoS ONE 7(8):e42974. doi: 10.1371/journal.pone.0042974

Tyson, R.B., S.M. Nowacek, and D.P. Nowacek. 2011. Community structure and abundance of bottlenose dolphins *Tursiops truncatus* in coastal waters of the northeast Gulf of Mexico. Marine Ecology Progress Series 438:253–265. doi: 10.3354/meps09292

Urian, K.W., S. Hofmann, R.S. Wells, and A.J. Read. 2009. Fine-scale population structure of bottlenose dolphins (*Tursiops truncatus*) in Tampa Bay, Florida. Marine Mammal Science 25(3):619–638. doi: 10.1111/j.1748-7692.2009.00284.x

Van Bressem, M.F., P.J. Duignan, A. Banyard, M. Barbieri, K.M. Colegrove, S. De Guise, G. Di Guardo, A. Dobson, M. Domingo, D. Fauquier, A. Fernandez, T. Goldstein, B. Grenfell, K.R. Groch, F. Gulland, B.A. Jensen, P.D. Jepson, A. Hall, T. Kuiken, S. Mazzariol, S.E. Morris, O. Nielsen, J.A. Raga, T.K. Rowles, J. Saliki, E. Sierra, N. Stephens, B. Stone, I. Tomo, J. Wang, T. Waltzek, and J.F. Wellehan. 2014. Cetacean morbillivirus: current knowledge and future directions. Viruses 6(12):5145–5181. doi: 10.3390/v6125145

Venn-Watson, S., L. Garrison, J. Litz, E. Fougeres, B. Mase, G. Rappucci, E. Stratton, R. Carmichael, D. Odell, D. Shannon, S. Shippee, S. Smith, L. Staggs, M. Tumlin, H. Whitehead, and T. Rowles. 2015. Demographic clusters identified within the northern Gulf of Mexico common bottlenose dolphin (*Tursiops truncates*) unusual mortality event: January 2010-June 2013. PLoS ONE 10(2):e0117248. doi: 10.1371/journal.pone.0117248

Vollmer, N.L. and P.E. Rosel. 2013. A review of common bottlenose dolphins (*Tursiops truncatus truncatus*) in the Northern Gulf of Mexico: Population biology, potential threats, and management. Southeastern Naturalist 12(m6):1–43. doi: 10.1656/058.012.m601

Weller, D.W. 1998. Global and regional variation in the biology and behavior of bottlenose dolphins. Texas A&M University. Dissertation. 142 p.

Wells, R.S. 1986a. Population structure of bottlenose dolphins: Behavioral studies along the central west coast of Florida. Contract Report to National Marine Fisheries Service, Southeast Fisheries Center. Contract No. 45-WCNF-5-003BB. 58 p.

Wells, R.S. 1986b. Structural aspects of dolphin societies. University of California, Santa Cruz. Dissertation. 234 p.

Wells, R.S. 1991. The role of long-term study in understanding the social structure of a bottlenose dolphin community. In: Pryor, K. and K. Norris (Eds.), Dolphin Societies: Discoveries and Puzzles. University of California Press, Berkeley, CA. pp. 199–226.

Wells, R.S. 2000. Reproduction in wild bottlenose dolphins: Overview of patterns observed during a long-term study. In: Duffield, D. and T. Robeck (Eds.), Bottlenose Dolphin Reproduction Workshop. AZA Marine Mammal Taxon Advisory Group, Silver Spring, MD. pp. 57–74.

Wells, R.S. 2003. Dolphin social complexity: Lessons from long-term study and life history. In: de Waal, F.B.M. and P.L. Tyack (Eds.), Animal Social Complexity: Intelligence, Culture, and Individualized Societies Harvard University Press, Cambridge, MA. pp. 32–56.

Wells, R.S. 2014. Social structure and life history of bottlenose dolphins near Sarasota Bay, Florida: Insights from four decades and five generations. Primates and cetaceans. pp. 149–172. doi: 10.1007/978-4-431-54523-1_8

Wells, R.S., M.K. Bassos, K.W. Urian, W.J. Carr, and M.D. Scott. 1996. Low-level monitoring of bottlenose dolphins, *Tursiops truncatus*, in Charlotte Harbor, Florida: 1990-1994. National Oceanic and Atmospheric Administration, National Marine Fisheries Service, Southeast Fisheries Science Center: Miami, FL. NOAA Technical Memorandum NMFS-SEFSC-384. 86 p.

Wells, R.S., M.K. Bassos, K.W. Urian, S.H. Shane, E.C.G. Owen, C.F. Weiss, W.J. Carr, and M.D. Scott. 1997. Low-level monitoring of bottlenose dolphins, *Tursiops truncatus*, in Pine Island Sound, Florida: 1996. National Oceanic and Atmospheric Administration, National Marine Fisheries Service, Southeast Fisheries Science Center: Miami, FL. Southeast Fisheries Center Contribution No. 40-WCNF601958. 86 p.

Wells, R.S., A. Natoli, and G. Braulik. 2019. *Tursiops truncatus* (errata version published in 2019). *The IUCN Red List of Threatened Species* 2019: e.T22563A156932432. https://dx.doi.org/10.2305/IUCN.UK.2019-1.RLTS.T22563A156932432.en

Wells, R.S., L.H. Schwacke, T.K. Rowles, B.C. Balmer, E. Zolman, T. Speakman, F.I. Townsend, M.C. Tumlin, A. Barleycorn, and K.A. Wilkinson. 2017. Ranging patterns of common bottlenose

dolphins *Tursiops truncatus* in Barataria Bay, Louisiana, following the Deepwater Horizon oil spill. Endangered Species Research 33:159–180. doi: 10.3354/esr00732

Wells, R.S. and M.D. Scott. 1999. Bottlenose dolphin *Tursiops truncatus* (Montagu, 1821). In: Ridgway, S.H. and R. Harrison (Eds.), Handbook of marine mammals. Volume 6: The second book of dolphins and the porpoises. Academic Press, San Diego, CA. pp. 137–182.

Wells, R.S. and M.D. Scott. 2018. Bottlenose dolphin, *Tursiops truncatus*, common bottlenose dolphin. In: Würsig, B., J.G.M. Thewissen, and K.M. Kovacs (Eds.), Encyclopedia of Marine Mammals (Third Edition). Academic Press, London, UK. pp. 118–125. doi: 10.1016/B978-0-12-804327-1.00072-8

Wells, R.S., M.D. Scott, and A.B. Irvine. 1987. The social structure of free-ranging bottlenose dolphins. In: Genoways, H.H. (Ed.), Current Mammalogy. Plenum Press, New York, NY. pp. 247–305.

Wells, R.S., K.W. Urian, A.J. Read, M.K. Bassos, W.J. Carr, and M.D. Scott. 1996. Low-level monitoring of bottlenose dolphins, *Tursiops truncatus*, in Tampa Bay, Florida: 1988-1993. National Oceanic and Atmospheric Administration, National Marine Fisheries Service, Southeast Fisheries Science Center: Miami, FL. NOAA Technical Memorandum NMFS-SEFSC-385. 36 p.

Wilson, R.M., R.B. Tyson, J.A. Nelson, B.C. Balmer, J.P. Chanton, and D.P. Nowacek. 2017. Niche differentiation and prey selectivity among common bottlenose dolphins (*Tursiops truncatus*) sighted in St. George Sound, Gulf of Mexico. Frontiers in Marine Science 4:235. doi: 10.3389/fmars.2017.00235

Common bottlenose dolphin − *Tursiops truncatus*

GoMx BSE/Perdido Bay Stock

Overall Vulnerability Rank = Very High

Biological Sensitivity = Very High

Climate Exposure = Very High

Data Quality = 93% of scores ≥ 2

Tursiops truncatus		Expert Scores	Data Quality	Expert Scores Plots (Portion by Category)
Sensitivity attributes	Prey/Diet Specificity	1.9	2.0	
	Habitat Specificity	3.7	2.2	
	Site Fidelity	3.9	2.2	
	Lifetime Reproductive Potential	1.2	2.0	
	Generation Length	2.8	2.0	
	Reproductive Plasticity	1.4	2.0	
	Migration	3.8	2.2	
	Home Range	3.5	2.0	
	Stock Abundance	3.1	2.0	
	Stock Abundance Trend	2.2	1.5	
	Cumulative Stressors	3.5	2.2	
	Sensitivity Score	Very High		
Exposure variables	Sea Surface Temperature (Change in mean)	4.0	2.8	
	Sea Surface Temperature (Change in variability)	1.0	2.8	
	Air Temperature (Change in mean)	4.0	2.8	
	Air Temperature (Change in variability)	1.0	2.8	
	Precipitation (Change in mean)	1.0	2.8	
	Precipitation (Change in variability)	1.0	2.8	
	Sea Surface Salinity (Change in mean)	2.0	2.8	
	Sea Surface Salinity (Change in variability)	1.0	2.8	
	Ocean pH (Change in mean)	4.0	2.8	
	Ocean pH (Change in variability)	1.0	2.8	
	Sea ice coverage (Change in mean)	1.0	2.8	
	Sea ice coverage (Change in variability)	1.0	2.8	
	Dissolved oxygen (Change in mean)	4.0	2.8	
	Dissolved oxygen (Change in variability)	1.0	2.8	
	Circulation	4.0	1.5	
	Sea level rise	4.0	2.8	
	Exposure Score	Very High		
Overall Vulnerability Rank		Very High		

Low
Moderate
High
Very High

Common bottlenose dolphin (GoMx BSE/Perdido Bay Stock)

Tursiops truncatus

CVA Results Summary

Overall Climate Vulnerability Rank: Very High (100% certainty from bootstrap analysis).

Climate Exposure: Very High. Six exposure factors scored greater than 3.5: Air Temperature (Standard anomaly) (4.00), Dissolved oxygen (Standard anomaly) (4.00), Ocean pH (Standard anomaly) (4.00), Sea level rise (4.00), Sea Surface Temperature (Standard anomaly) (4.00), and Circulation (3.95).

Biological Sensitivity: Very High. Five sensitivity attributes scored greater than or equal to 3.5: Site Fidelity (3.90), Migration (3.80), Habitat Specificity (3.70), Cumulative Stressors (3.55), and Home Range (3.50).

Distributional Response: Very High

Abundance Response: Very High

Phenology Response: Very High

Data Quality: 93% of the data quality scores were 2 or greater, 91% of sensitivity attribute data quality scores were 2 or higher.

Background Information

Stock Description

This stock includes common bottlenose dolphins in Perdido Bay in the northern Gulf of Mexico along the Alabama and Florida coasts (Hayes et al. 2022).

Prey/Diet Specificity

Generally, common bottlenose dolphins consume a large variety of fish and/or squid (Barros and Odell 1990; Mead and Potter 1990; Wells and Scott 2018). Common bottlenose dolphins seem to show a consistent preference for sound-producing fishes (e.g., sciaenids, toadfish; Berens McCabe et al. 2010; Bowen-Stevens et al. 2021). In Sarasota Bay, Florida, Rossman et al. (2015) found some individual common bottlenose dolphins to show diet specialization despite the broader study group showing a generalist diet. Specialization can be influenced by locally abundant prey species (Shane et al. 1986; Vollmer and Rosel 2013) or can result from resource partitioning amongst sympatric populations (e.g., Torres and Read 2009; Wilson et al. 2017).

Habitat Specificity

Common bottlenose dolphins are found in a variety of marine and estuarine habitats (Wells and Scott 2018).

Site Fidelity

No specific site fidelity information was found for this stock. However, common bottlenose dolphins in other Gulf of Mexico bay, sound, and estuary (BSE) stocks show site fidelity (LaBrecque et al. 2015; Hayes et al. 2022), such as in Matagorda Bay, Espiritu Santo Bay, and San Luis Pass in Texas (Maze and Würsig 1999; Lynn and Würsig 2002; Irwin and Würsig 2004); Barataria Bay in Louisiana (Miller 2003; McDonald et al. 2017; Wells et al. 2017; Takeshita et al. 2021); Mississippi Sound in Mississippi (Hubard et al. 2004; Mackey 2010; Mullin et al. 2017); Charlotte Harbor and Pine Island Sound (Bassos-Hull et al. 2013), Sarasota Bay (Wells 1986a; Wells 1991; Wells 2014), St. Andrew Bay (Balmer et al. 2019), St. Joseph Bay (Balmer et al. 2008; B. Balmer et al. 2018), St. Vincent Sound and Apalachicola Bay (Tyson et al. 2011), and Tampa Bay in Florida (Wells et al. 1996b; Urian et al. 2009).

Lifetime Reproductive Potential

Taylor et al. (2007) reported the interbirth interval of common bottlenose dolphins as 3.80 years, though other studies have shown it to be closer to three years (Leatherwood 1977; Wells and Scott 1999; Connor et al. 2000; Wells 2003).

Generally, females reach sexual maturity before males, with females reaching sexual maturity at 5–13 years (Sergeant et al. 1973; Perrin and Reilly 1984; Wells et al. 1987; Mead and Potter 1990; Mann et al. 2000; Wells 2000; Wells and Scott 2018).

Females reproduce through most of their adult lives (Leatherwood 1977; Hohn et al. 1989; Wells and Scott 1999; Wells 2000; Wells 2003), but may produce fewer calves after they reach 30 years of age (Wells 2000). Sarasota Bay common bottlenose dolphins can give birth when they are as young as 6 years of age, and can continue until they are 48 years old (Wells and Scott 2018). Reproductive lifespan would therefore be up to 42 years based on long-term observation studies (Wells 2014; Wells and Scott 2018).

Longitudinal studies from Sarasota Bay, Florida have found that female common bottlenose dolphins can live up to 67 years (Wells and Scott 2018). Studies based on growth layer groups in teeth suggest female common bottlenose dolphins can live to 41 years along the Texas coast (Fernandez and Hohn 1998) and to 30 years along the Mississippi coast (Mattson et al. 2006).

Generation Length

Taylor et al. (2007) reported a common bottlenose dolphin generation length of 20.6–21.1 years.

Reproductive Plasticity

Common bottlenose dolphin calving is generally diffusely seasonal, with one or more peaks, although births have been reported from all seasons (Vollmer and Rosel 2013; Wells and Scott 2018). Calf sightings peak in the spring and summer months in Florida (Wells et al. 1987), Mississippi/Louisiana (Miller et al. 2010; Miller et al. 2013), and Texas (McHugh 1989; Henderson 2004).

Migration

The bay, sound, and estuary stocks in the Gulf of Mexico are thought to primarily comprise stable resident communities. However, transient or seasonal movements of some individuals into and out of a BSE stock area are recorded in some areas (LaBrecque et al. 2015; Hayes et al. 2022). Molecular genetic data support that some BSE stocks are genetically distinct from one another, and furthermore are differentiated from adjacent coastal populations (e.g., Sellas et al. 2005; Rosel et al. 2017). For example, Toms (2019) found fine-scale genetic population structure among five inshore systems in the Florida Panhandle and variable migration rates among populations.

Year-round residency has been reported for common bottlenose dolphins (Hayes et al. 2022), such as in Florida (Irvine and Wells 1972; Irvine et al. 1981; Wells 1986a; Wells 1986b; Wells et al. 1987; Scott et al. 1990; Wells 1991; Wells et al. 1996a; Wells et al. 1996b; Wells et al. 1997; Wells 2003; Balmer et al. 2008; Urian et al. 2009; Tyson et al. 2011; Bassos-Hull et al. 2013; B. Balmer et al. 2018; Balmer et al. 2019; Toms 2019), Texas (Shane 1990; Bräger 1993; Bräger et al. 1994; Fertl 1994; Weller 1998; Maze and Würsig 1999; Lynn and Würsig 2002; Irwin and Würsig 2004; Shane 2004), and Louisiana/Mississippi (Hubard et al. 2004; McDonald et al. 2017; Mullin et al. 2017; Takeshita et al. 2021).

Home Range

The home ranges of individuals within the Gulf of Mexico BSE stocks are considered to generally include the areas within the stock boundaries and brief excursions beyond the stock boundaries (Irvine et al. 1981; Wells 1986a; Wells 1986b; Wells 1991; Wells et al. 1996a; Wells et al. 1996b; Balmer et al. 2008; Wells 2014; Balmer et al. 2019), although some individuals show preference for small areas within the stock boundaries (Lynn and Würsig 2002; Urian et al. 2009; Mullin et al. 2017; Wells et al. 2017).

Stock Abundance

An abundance estimate for this stock is unavailable (Hayes et al. 2022).

Stock Abundance Trend

Data are insufficient to complete an abundance trend analysis for this stock (Hayes et al. 2022).

Cumulative Stressors

Common bottlenose dolphins face a wide range of individual and cumulative natural and anthropogenic threats (see reviews by Vollmer and Rosel 2013; Wells and Scott 2018; and Wells et al. 2019). These include such threats as shark predation, harmful algal blooms, recreational and commercial fishing gear, vessel traffic, pollution, habitat alteration, and provisioning.

Common bottlenose dolphins in Perdido Bay were possibly exposed to oil and dispersants following the Deepwater Horizon oil spill, which may result in long term reproductive and chronic health effects (NOAA 2011; Michel et al. 2013; Helm et al. 2015; DWH NRDA 2016; Lane et al. 2015; De Guise et al. 2017, 2021; Kellar et al. 2017; Smith et al. 2017; Schwacke et al. 2017). An Unusual Mortality Event (UME), involving primarily bottlenose dolphins, was declared for cetaceans in the northern Gulf of Mexico from 2010 to 2014, with the Deepwater Horizon oil spill as the primary underlying cause (Litz et al. 2014; Schwacke et al. 2014; Venn-Watson et al. 2015a, 2015b).

The nearshore and estuarine habitats along the Gulf of Mexico coast include waters adjacent to areas of high human population, some of which are highly industrialized, and areas of heavy agricultural impact. Studies of common bottlenose dolphin tissues from the northern Gulf of Mexico have shown persistent organic pollutants (POPs) in blubber and blood samples (Kucklick et al. 2011; Balmer et al. 2015; J. Balmer et al. 2018). Similarly, mercury has been detected in common bottlenose dolphin tissues in waters along the Florida panhandle (McCormack et al. 2020a, 2020b, 2022).

UMEs have been declared for common bottlenose dolphins in waters of the Florida panhandle with biotoxins (e.g., brevetoxin from *Karenia brevis*) the suspected or confirmed cause (Twiner et al. 2012; Litz et al. 2014). Dolphin morbillivirus has been detected in populations of the northern Gulf of Mexico, but has not been attributed to large scale die-offs since 1993−1994 (Lipscomb et al. 1996; Litz et al. 2014; Van Bressem et al. 2014; Fauquier et al. 2017). A UME was declared in 1990 for the Gulf of Mexico from Florida to Texas, with more than 300 common bottlenose dolphins stranding due to an undetermined cause, although morbillivirus and cold temperatures may have contributed (Litz et al. 2014).

Further Reading

Hayes, S.A., E. Josephson, K. Maze-Foley, P.E. Rosel, and J. Wallace. 2022. U.S. Atlantic and Gulf of Mexico marine mammal stock assessments 2021. National Oceanic and Atmospheric Administration, National Marine Fisheries Service, Northeast Fisheries Science Center: Woods Hole, MA. NOAA Technical Memorandum NMFS-NE-288. 380 p. doi: 10.25923/6tt7-kc16

Wells, R.S. and M.D. Scott. 2018. Bottlenose Dolphin, *Tursiops truncatus*, Common Bottlenose Dolphin. In: Würsig, B., J.G.M. Thewissen, and K.M. Kovacs (Eds.), Encyclopedia of Marine Mammals (Third Edition). Academic Press, London, UK. pp. 118–125. doi: 10.1016/B978-0-12-804327-1.00072-8

Wells, R.S., A. Natoli, and G. Braulik. 2019. *Tursiops truncatus* (errata version published in 2019). The IUCN Red List of Threatened Species 2019: e.T22563A156932432. https://dx.doi.org/10.2305/IUCN.UK.2019-1.RLTS.T22563A156932432.en

References

Balmer, B., T. McDonald, F. Hornsby, J. Adams, J. Allen, A. Barleycorn, and L. Schwacke. 2018. Long-term trends in a northern Gulf of Mexico common bottlenose dolphin (*Tursiops truncatus*) population in the wake of the Deepwater Horizon oil spill. Journal of Cetacean Research and Management 18:1–9. doi: 10.47536/jcrm.v18i1.409

Balmer, B., S. Watwood, B. Quigley, T. Speakman, K. Barry, K. Mullin, P. Rosel, C. Sinclair, E. Zolman, and L. Schwacke. 2019. Common bottlenose dolphin (*Tursiops truncatus*) abundance and distribution patterns in St. Andrew Bay, Florida, USA. Aquatic Conservation: Marine and Freshwater Ecosystems 29(3):486–498. doi: 10.1002/aqc.3001

Balmer, B.C., R.S. Wells, S.M. Nowacek, D.P. Nowacek, L.H. Schwacke, W.A. McLellan, F.S. Scharf, T.K. Rowles, L.J. Hansen, T.R. Spradlin, and D.A. Pabst. 2008. Seasonal abundance and distribution patterns of common bottlenose dolphins (*Tursiops truncatus*) near St. Joseph Bay, Florida, USA. Journal of Cetacean Research and Management 10(2):157–167.

Balmer, B.C., G.M. Ylitalo, L.E. McGeorge, K.A. Baugh, D. Boyd, K.D. Mullin, P.E. Rosel, C. Sinclair, R.S. Wells, E.S. Zolman, and L.H. Schwacke. 2015. Persistent organic pollutants (POPs) in blubber of common bottlenose dolphins (*Tursiops truncatus*) along the northern Gulf of Mexico coast, USA. Science of the Total Environment 527-528:306–312. doi: 10.1016/j.scitotenv.2015.05.016

Balmer, J.E., G.M. Ylitalo, T.K. Rowles, K.D. Mullin, R.S. Wells, F.I. Townsend, R.W. Pearce, J.L. Bolton, E.S. Zolman, B.C. Balmer, and L.H. Schwacke. 2018. Persistent organic pollutants (POPs) in blood and blubber of common bottlenose dolphins (*Tursiops truncatus*) at three northern Gulf of Mexico sites following the Deepwater Horizon oil spill. Science of the Total Environment 621:130–137. doi: 10.1016/j.scitotenv.2017.11.209

Barros, N.B. and D.K. Odell. 1990. Food habits of bottlenose dolphins in the southeastern United States In: Leatherwood, S. and R.R. Reeves (Eds.), The Bottlenose Dolphin. Academic Press, San Diego, CA. pp. 309–328. doi: 10.1016/B978-0-12-440280-5.50020-2

Bassos-Hull, K., R.M. Perrtree, C.C. Shepard, S. Schilling, A.A. Barleycorn, J.B. Allen, B.C. Balmer, W.E. Pine, and R.S. Wells. 2013. Long-term site fidelity and seasonal abundance estimates of common bottlenose dolphins (*Tursiops truncatus*) along the southwest coast of Florida and responses to natural perturbations. Journal of Cetacean Research and Management 13(1):19–30. doi: 10.47536/jcrm.v13i1.551

Berens McCabe, E.J., D.P. Gannon, N.B. Barros, and R.S. Wells. 2010. Prey selection by resident common bottlenose dolphins (*Tursiops truncatus*) in Sarasota Bay, Florida. Marine Biology 157(5):931–942. doi: 10.1007/s00227-009-1371-2

Bowen-Stevens, S.R., D.P. Gannon, R.A. Hazelkorn, G. Lovewell, K.M. Volker, S. Smith, M.C. Tumlin, and J. Litz. 2021. Diet of common bottlenose dolphins, *Tursiops truncatus*, that stranded in and near Barataria Bay, Louisiana, 2010–2012. Southeastern Naturalist 20(1). doi: 10.1656/058.020.0113

Bräger, S. 1993. Diurnal and seasonal behavior patterns of bottlenose dolphins (*Tursiops truncatus*). Marine Mammal Science 9:434–438. doi: 10.1111/j.1748-7692.1993.tb00477.x

Bräger, S., B. Würsig, A. Acevedo, and T. Henningsen. 1994. Association patterns of bottle-nosed dolphins (*Tursiops-truncatus*) in Galveston Bay, Texas. Journal of Mammalogy 75(2):431–437. doi: 10.2307/1382564

Connor, R.C., R.S. Wells, J. Mann, and A.J. Read. 2000. The bottlenose dolphin: Social relationships in a fission-fusion society. In: Mann, J., R. Connor, P. Tyack, and H. Whitehead (Eds.), Cetacean Societies: Field Studies of Dolphins and Whales. University of Chicago Press, Chicago, IL. pp. 91–126.

De Guise, S., M. Levin, E. Gebhard, L. Jasperse, L. Burdett Hart, C.R. Smith, S. Venn-Watson, F. Townsend, R. Wells, B. Balmer, E. Zolman, T. Rowles, and L. Schwacke. 2017. Changes in immune functions in bottlenose dolphins in the northern Gulf of Mexico associated with the Deepwater Horizon oil spill. Endangered Species Research 33:291–303. doi: 10.3354/esr00814

De Guise, S., M. Levin, L. Jasperse, J. Herrman, R.S. Wells, T. Rowles, and L. Schwacke. 2021. Long-term immunological alterations in bottlenose dolphin a decade after the Deepwater Horizon oil spill in the northern Gulf of Mexico: Potential for multigenerational effects. Environmental Toxicology and Chemistry 40(5):1308–1321. doi: 10.1002/etc.4980

Deepwater Horizon Natural Resource Damage Assessment Trustees (DWH NRDA). 2016. Deepwater Horizon oil spill: Final Programmatic Damage Assessment and Restoration Plan and Final Programmatic Environmental Impact Statement. Retrieved from: http://www.gulfspillrestoration.noaa.gov/restoration-planning/gulf-plan.1659.

Fauquier, D.A., J. Litz, S. Sanchez, K. Colegrove, L.H. Schwacke, L. Hart, J. Saliki, C. Smith, T. Goldstein, S. Bowen-Stevens, W. McFee, E. Fougeres, B. Mase-Guthrie, E. Stratton, R. Ewing, S. Venn-Watson, R.H. Carmichael, C. Clemons-Chevis, W. Hatchett, D. Shannon, S. Shippee, S. Smith, L. Staggs, M.C. Tumlin, N.L. Wingers, and T.K. Rowles. 2017. Evaluation of morbillivirus exposure in cetaceans from the northern Gulf of Mexico 2010-2014. Endangered Species Research 33:211–220. doi: 10.3354/esr00772

Fernandez, S. and A.A. Hohn. 1998. Age, growth, and calving season of bottlenose dolphins, *Tursiops truncatus*, off coastal Texas. Fishery Bulletin 96(2):357–365.

Fertl, D. 1994. Occurrence patterns and behavior of bottlenose dolphins (*Tursiops truncatus*) in the Galveston ship channel, Texas. Texas Journal of Science 46(4):299–318.

Hayes, S.A., E. Josephson, K. Maze-Foley, P.E. Rosel, and J. Wallace. 2022. U.S. Atlantic and Gulf of Mexico marine mammal stock assessments 2021. National Oceanic and Atmospheric Administration, National Marine Fisheries Service, Northeast Fisheries Science Center: Woods Hole, MA. NOAA Technical Memorandum NMFS-NE-288. 380 p. doi: 10.25923/6tt7-kc16

Helm, R.C., D.P. Costa, T.D. DeBruyn, T.J. O'Shea, R.S. Wells, and T.M. Williams. 2015. Overview of effects of oil spills on marine mammals. In: Fingas, M. (Ed.), Handbook of oil spill science and technology, First edition. John Wiley & Sons, Inc., Hoboken, NJ. pp. 455–475.

Henderson, E.E. and B. Würsig. 2007. Behavior patterns of bottlenose dolphins in San Luis Pass, Texas. Gulf of Mexico Science 25(2):153–161. doi: 10.18785/goms.2502.06

Hohn, A.A., M.D. Scott, R.S. Wells, J.C. Sweeney, and A.B. Irvine. 1989. Growth layers in teeth from known-age, free-ranging bottlenose dolphins. Marine Mammal Science 5(4):315–342. doi: 10.1111/j.1748-7692.1989.tb00346.x

Hubard, C.W., K. Maze-Foley, K.D. Mullin, and W.W. Schroeder. 2004. Seasonal abundance and site fidelity of bottlenose dolphins (*Tursiops truncatus*) in Mississippi Sound. Aquatic Mammals 30(2):299–310. doi: 10.1578/am.30.2.2004.299

Irvine, A.B., M.D. Scott, R.S. Wells, and J.H. Kaufmann. 1981. Movements and activities of the Atlantic bottlenose dolphin, *Tursiops truncatus*, near Sarasota, Florida. Fishery Bulletin 79(4):671–688.

Irvine, B. and R.S. Wells. 1972. Results of attempts to tag Atlantic bottlenosed dolphins (*Tursiops truncatus*). Cetology 13:1–5.

Irwin, L.-J. and B. Würsig. 2004. A small resident community of bottlenose dolphins, *Tursiops truncatus*, in Texas: Monitoring recommendations. Gulf of Mexico Science 22(1):13–21. doi: 10.18785/goms.2201.02

Kellar, N.M., T.R. Speakman, C.R. Smith, S.M. Lane, B.C. Balmer, M.L. Trego, K.N. Catelani, M.N. Robbins, C.D. Allen, R.S. Wells, E.S. Zolman, T.K. Rowles, and L.H. Schwacke. 2017. Low reproductive success rates of common bottlenose dolphins *Tursiops truncatus* in the northern Gulf of Mexico following the Deepwater Horizon disaster (2010-2015). Endangered Species Research 33:143–158. doi: 10.3354/esr00775

Kucklick, J., L. Schwacke, R. Wells, A. Hohn, A. Guichard, J. Yordy, L. Hansen, E. Zolman, R. Wilson, J. Litz, D. Nowacek, T. Rowles, R. Pugh, B. Balmer, C. Sinclair, and P. Rosel. 2011. Bottlenose dolphins as indicators of persistent organic pollutants in the western North Atlantic Ocean and Northern Gulf of Mexico. Environmental Science & Technology 45(10):4270–4277. doi: 10.1021/es1042244

LaBrecque, E., C. Curtice, J. Harrison, S.M. Van Parijs, and P.N. Halpin. 2015. 3. Biologically Important Areas for cetaceans within U.S. Waters – Gulf of Mexico Region. Aquatic Mammals 41(1):30–38. doi: 10.1578/am.41.1.2015.30

Lane, S.M., C.R. Smith, J. Mitchell, B.C. Balmer, K.P. Barry, T. McDonald, C.S. Mori, P.E. Rosel, T.K. Rowles, T.R. Speakman, F.I. Townsend, M.C. Tumlin, R.S. Wells, E.S. Zolman, and L.H. Schwacke. 2015. Reproductive outcome and survival of common bottlenose dolphins sampled in Barataria Bay, Louisiana, USA, following the Deepwater Horizon oil spill. Proceedings of the Royal Society B: Biological Sciences 282(1818):20151944. doi: 10.1098/rspb.2015.1944

Leatherwood, S. 1977. Some preliminary impressions on the numbers and social behavior of free-swimming bottlenosed dolphin calves (*Tursiops truncatus*) in the northern Gulf of Mexico. In: Ridgway, S.H. and K. Bernirschke (Eds.), Breeding Dolphins; Present Status, Suggestions for the Future. Final report to the US Marine Mammal Commission, Contract MM6AC009, MMC-76/07, Washington, DC. pp. 143–167.

Lipscomb, T.P., S. Kennedy, D. Moffett, A. Krafft, B.A. Klaunberg, J.H. Lichy, G.T. Regan, G.A.J. Worthy, and J.K. Taubenberger. 1996. Morbilliviral epizootic in bottlenose dolphins of the Gulf of Mexico. Journal of Veterinary Diagnostic Investigation 8(3):283–290. doi: 10.1177/104063879600800302

Litz, J.A., M.A. Baran, S.R. Bowen-Stevens, R.H. Carmichael, K.M. Colegrove, L.P. Garrison, S.E. Fire, E.M. Fougeres, R. Hardy, S. Holmes, W. Jones, B.E. Mase-Guthrie, D.K. Odell, P.E. Rosel, J.T. Saliki, D.K. Shannon, S.F. Shippee, S.M. Smith, E.M. Stratton, M.C. Tumlin, H.R. Whitehead, G.A. Worthy, and T.K. Rowles. 2014. Review of historical unusual mortality events (UMEs) in the Gulf of Mexico (1990–2009): providing context for the multi-year northern Gulf of Mexico cetacean UME declared in 2010. Diseases of Aquatic Organisms 112(2):161–175. doi: 10.3354/dao02807

Lynn, S.K. and B. Würsig. 2002. Summer movement patterns of bottlenose dolphins in a Texas bay. Gulf of Mexico Science 20(1):25–37. doi: 10.18785/goms.2001.03

Mackey, A.D. 2010. Site fidelity and association patterns of bottlenose dolphins (*Tursiops truncatus*) in the Mississippi Sound. University of Southern Mississippi. Thesis. 133 p.

Mann, J., R.C. Connor, L.M. Barre, and M.R. Heithaus. 2000. Female reproductive success in bottlenose dolphins (*Tursiops* sp.): life history, habitat, provisioning, and group-size effects. Behavioral Ecology 11(2):210–219. doi: 10.1093/beheco/11.2.210

Mattson, M.C., K.D. Mullin, G.W. Ingram, and W. Hoggard. 2006. Age structure and growth of the bottlenose dolphin (*Tursiops truncatus*) from strandings in the Mississippi sound region of the north-central Gulf of Mexico from 1986 to 2003. Marine Mammal Science 22(3):654–666. doi: 10.1111/j.1748-7692.2006.00057.x

Maze, K.S. and B. Würsig. 1999. Bottlenosed dolphins of San Luis Pass, Texas: Occurrence patterns, site-fidelity, and habitat use. Aquatic Mammals 25:91–104.

McCormack, M.A., F. Battaglia, W.E. McFee, and J. Dutton. 2020a. Mercury concentrations in blubber and skin from stranded bottlenose dolphins (*Tursiops truncatus*) along the Florida and Louisiana coasts (Gulf of Mexico, USA) in relation to biological variables. Environmental Research 180:108886. doi: 10.1016/j.envres.2019.108886

McCormack, M.A., B.P. Jackson, and J. Dutton. 2020b. Relationship between mercury and selenium concentrations in tissues from stranded odontocetes in the northern Gulf of Mexico. Science of the Total Environment 749:141350. doi: 10.1016/j.scitotenv.2020.141350

McCormack, M.A., W.H. Nowlin, and J. Dutton. 2022. Effect of trophic position on mercury concentrations in bottlenose dolphins (*Tursiops truncatus*) from the northern Gulf of Mexico. Environmental Research 204(Pt B):112124. doi: 10.1016/j.envres.2021.112124

McDonald, T.L., F.E. Hornsby, T.R. Speakman, E.S. Zolman, K.D. Mullin, C. Sinclair, P.E. Rosel, L. Thomas, and L.H. Schwacke. 2017. Survival, density, and abundance of common bottlenose dolphins in Barataria Bay (USA) following the Deepwater Horizon oil spill. Endangered Species Research 33:193–209. doi: 10.3354/esr00806

McHugh, M.B. 1989. Population numbers and feeding behavior of the Atlantic bottlenose dolphin (*Tursiops truncatus*) near Aransas Pass, Texas. University of Texas at Austin. Thesis. 96 p.

Mead, J.G. and C.W. Potter. 1990. Natural history of bottlenose dolphins along the central Atlantic coast of the United States. In: Leatherwood, S. and R.R. Reeves (Eds.), The Bottlenose Dolphin. Academic Press, San Diego, CA. pp. 165–195.

Michel, J., E.H. Owens, S. Zengel, A. Graham, Z. Nixon, T. Allard, W. Holton, P.D. Reimer, A. Lamarche, M. White, N. Rutherford, C. Childs, G. Mauseth, G. Challenger, and E. Taylor. 2013. Extent and degree of shoreline oiling: Deepwater Horizon oil spill, Gulf of Mexico, USA. PLoS ONE 8(6):e65087. doi: 10.1371/journal.pone.0065087

Miller, C.E. 2003. Abundance trends and environmental habitat usage patterns of bottlenose dolphins (*Tursiops truncatus*) in lower Barataria and Caminada bays, Louisiana. Louisiana State University and Agricultural & Mechanical College. Dissertation. 125 p.

Miller, L.J., A.D. Mackey, T. Hoffland, M. Solangi, and S.A. Kuczaj. 2010. Potential effects of a major hurricane on Atlantic bottlenose dolphin (*Tursiops truncatus*) reproduction in the Mississippi Sound. Marine Mammal Science 26(3):707–715. doi: 10.1111/j.1748-7692.2010.00371.x

Miller, L.J., A.D. Mackey, M. Solangi, and S.A. Kuczaj. 2013. Population abundance and habitat utilization of bottlenose dolphins in the Mississippi Sound. Aquatic Conservation: Marine and Freshwater Ecosystems 23(1):145–151. doi: 10.1002/aqc.2278

Mullin, K.D., T. McDonald, R.S. Wells, B.C. Balmer, T. Speakman, C. Sinclair, E.S. Zolman, F. Hornsby, S.M. McBride, K.A. Wilkinson, and L.H. Schwacke. 2017. Density, abundance, survival, and ranging patterns of common bottlenose dolphins (*Tursiops truncatus*) in Mississippi Sound following the Deepwater Horizon oil spill. PLoS ONE 12(10):e0186265. doi: 10.1371/journal.pone.0186265

National Oceanic and Atmospheric Administration (NOAA). 2011. Public scoping for preparation of a programmatic environmental impact statement for the Deepwater Horizon BP Oil Spill. 9 p.

Perrin, W.F. and S.B. Reilly. 1984. Reproductive parameters of dolphins and small whales of the family Delphinidae. In: Perrin, W.F., R.L. Brownell, Jr., and D.P. DeMaster (Eds.), Reproduction in Whales, Dolphins, and Porpoises. pp. 97–134.

Rosel, P.E., L.A. Wilcox, C. Sinclair, T.R. Speakman, M.C. Tumlin, J.A. Litz, and E.S. Zolman. 2017. Genetic assignment to stock of stranded common bottlenose dolphins in southeastern Louisiana after the Deepwater Horizon oil spill. Endangered Species Research 33:221–234. doi: 10.3354/esr00780

Rossman, S., P.H. Ostrom, M. Stolen, N.B. Barros, H. Gandhi, C.A. Stricker, and R.S. Wells. 2015. Individual specialization in the foraging habits of female bottlenose dolphins living in a trophically diverse and habitat rich estuary. Oecologia 178(2):415–425. doi: 10.1007/s00442-015-3241-6

Schwacke, L.H., C.R. Smith, F.I. Townsend, R.S. Wells, L.B. Hart, B.C. Balmer, T.K. Collier, S. De Guise, M.M. Fry, L.J. Guillette, Jr., S.V. Lamb, S.M. Lane, W.E. McFee, N.J. Place, M.C. Tumlin, G.M. Ylitalo, E.S. Zolman, and T.K. Rowles. 2014. Health of common bottlenose dolphins (*Tursiops truncatus*) in Barataria Bay, Louisiana, following the deepwater horizon oil spill. Environmental Science & Technology 48(1):93–103. doi: 10.1021/es403610f

Schwacke, L.H., L. Thomas, R.S. Wells, W.E. McFee, A.A. Hohn, K.D. Mullin, E.S. Zolman, B.M. Quigley, T.K. Rowles, and J.H. Schwacke. 2017. Quantifying injury to common bottlenose dolphins from the Deepwater Horizon oil spill using an age-, sex- and class-structured population model. Endangered Species Research 33:265–279. doi: 10.3354/esr00777

Scott, M.D. and S.J. Chivers. 1990. Distribution and herd structure of bottlenose dolphins in the eastern tropical Pacific Ocean. In: Leatherwood, S. and R.R. Reeves (Eds.), The Bottlenose Dolphin. Academic Press, San Diego, CA. pp. 387–402.

Sellas, A.B., R.S. Wells, and P.E. Rosel. 2005. Mitochondrial and nuclear DNA analyses reveal fine scale geographic structure in bottlenose dolphins (*Tursiops truncatus*) in the Gulf of Mexico. Conservation Genetics 6(5):715–728. doi: 10.1007/s10592-005-9031-7

Sergeant, D.E., D.K. Caldwell, and M.C. Caldwell. 1973. Age, growth, and maturity of bottlenosed dolphin (*Tursiops-truncatus*) from northeast Florida. Journal of the Fisheries Research Board of Canada 30(7):1009–1011. doi: 10.1139/f73-165

Shane, S.H. 1990. Behavior and ecology of the bottlenose dolphin at Sanibel Island, Florida. In: Leatherwood, S. and R.R. Reeves (Eds.), The Bottlenose Dolphin. Academic Press, San Diego, CA. pp. 245–265. doi: 10.1016/B978-0-12-440280-5.50016-0

Shane, S.H. 2004. Residence patterns, group characteristics, and association patterns of bottlenose dolphins near Sanibel Island, Florida. Gulf of Mexico Science 22(1):1–12. doi: 10.18785/goms.2201.01

Shane, S.H., R.S. Wells, and B. Würsig. 1986. Ecology, behavior and social organization of the bottlenose dolphin: A review. Marine Mammal Science 2:34–63. doi: 10.1111/j.1748-7692.1986.tb00026.x

Smith, C.R., T.K. Rowles, L.B. Hart, F.I. Townsend, R.S. Wells, E.S. Zolman, B.C. Balmer, B. Quigley, M. Ivančić, W. McKercher, M.C. Tumlin, K.D. Mullin, J.D. Adams, Q. Wu, W. McFee, T.K. Collier, and L.H. Schwacke. 2017. Slow recovery of Barataria Bay dolphin health following the Deepwater Horizon oil spill (2013-2014), with evidence of persistent lung disease and impaired stress response. Endangered Species Research 33:127–142. doi: 10.3354/esr00778

Takeshita, R., B.C. Balmer, F. Messina, E.S. Zolman, L. Thomas, R.S. Wells, C.R. Smith, T.K. Rowles, and L.H. Schwacke. 2021. High site-fidelity in common bottlenose dolphins despite low salinity exposure and associated indicators of compromised health. PLoS ONE 16(9):e0258031. doi: 10.1371/journal.pone.0258031

Taylor, B.L., S.J. Chivers, J. Larese, and W.F. Perrin. 2007. Generation length and percent mature estimates for IUCN assessments of cetaceans. National Oceanic and Atmospheric Administration, National Marine Fisheries Service, Southwest Fisheries Science Center: La Jolla, CA. Administrative Report LJ-07-01. 24 p.

Toms, C. 2019. Filling the gaps: Common bottlenose dolphin (*Tursiops truncatus*) population dynamics, structure, and connectivity within Florida panhandle bays, sounds, and estuaries. University of Central Florida. Dissertation. 373 p.

Torres, L.G. and A.J. Read. 2009. Where to catch a fish? The influence of foraging tactics on the ecology of bottlenose dolphins (*Tursiops truncatus*) in Florida Bay, Florida. Marine Mammal Science 25(4):797–815. doi: 10.1111/j.1748-7692.2009.00297.x

Twiner, M.J., L.J. Flewelling, S.E. Fire, S.R. Bowen-Stevens, J.K. Gaydos, C.K. Johnson, J.H. Landsberg, T.A. Leighfield, B. Mase-Guthrie, L. Schwacke, F.M. Van Dolah, Z. Wang, and T.K. Rowles. 2012. Comparative analysis of three brevetoxin-associated bottlenose dolphin (*Tursiops truncatus*) mortality events in the Florida Panhandle region (USA). PLoS ONE 7(8):e42974. doi: 10.1371/journal.pone.0042974

Tyson, R.B., S.M. Nowacek, and D.P. Nowacek. 2011. Community structure and abundance of bottlenose dolphins *Tursiops truncatus* in coastal waters of the northeast Gulf of Mexico. Marine Ecology Progress Series 438:253–265. doi: 10.3354/meps09292

Urian, K.W., S. Hofmann, R.S. Wells, and A.J. Read. 2009. Fine-scale population structure of bottlenose dolphins (*Tursiops truncatus*) in Tampa Bay, Florida. Marine Mammal Science 25(3):619–638. doi: 10.1111/j.1748-7692.2009.00284.x

Van Bressem, M.F., P.J. Duignan, A. Banyard, M. Barbieri, K.M. Colegrove, S. De Guise, G. Di Guardo, A. Dobson, M. Domingo, D. Fauquier, A. Fernandez, T. Goldstein, B. Grenfell, K.R. Groch, F. Gulland, B.A. Jensen, P.D. Jepson, A. Hall, T. Kuiken, S. Mazzariol, S.E. Morris, O. Nielsen, J.A. Raga, T.K. Rowles, J. Saliki, E. Sierra, N. Stephens, B. Stone, I. Tomo, J. Wang, T. Waltzek, and J.F. Wellehan. 2014. Cetacean morbillivirus: current knowledge and future directions. Viruses 6(12):5145–5181. doi: 10.3390/v6125145

Venn-Watson, S., L. Garrison, J. Litz, E. Fougeres, B. Mase, G. Rappucci, E. Stratton, R. Carmichael, D. Odell, D. Shannon, S. Shippee, S. Smith, L. Staggs, M. Tumlin, H. Whitehead, and T. Rowles. 2015. Demographic clusters identified within the northern Gulf of Mexico common bottlenose dolphin (*Tursiops truncates*) unusual mortality event: January 2010-June 2013. PLoS ONE 10(2):e0117248. doi: 10.1371/journal.pone.0117248

Vollmer, N.L. and P.E. Rosel. 2013. A review of common bottlenose dolphins (*Tursiops truncatus truncatus*) in the Northern Gulf of Mexico: Population biology, potential threats, and management. Southeastern Naturalist 12(m6):1–43. doi: 10.1656/058.012.m601

Weller, D.W. 1998. Global and regional variation in the biology and behavior of bottlenose dolphins. Texas A&M University. Dissertation. 142 p.

Wells, R.S. 1986a. Population structure of bottlenose dolphins: Behavioral studies along the central west coast of Florida. Contract Report to National Marine Fisheries Service, Southeast Fisheries Center. Contract No. 45-WCNF-5-003BB. 58 p.

Wells, R.S. 1986b. Structural aspects of dolphin societies. University of California, Santa Cruz. Dissertation. 234 p.

Wells, R.S. 1991. The role of long-term study in understanding the social structure of a bottlenose dolphin community. In: Pryor, K. and K. Norris (Eds.), Dolphin Societies: Discoveries and Puzzles. University of California Press, Berkeley, CA. pp. 199–226.

Wells, R.S. 2000. Reproduction in wild bottlenose dolphins: Overview of patterns observed during a long-term study. In: Duffield, D. and T. Robeck (Eds.), Bottlenose Dolphin Reproduction Workshop. AZA Marine Mammal Taxon Advisory Group, Silver Spring, MD. pp. 57–74.

Wells, R.S. 2003. Dolphin social complexity: Lessons from long-term study and life history. In: de Waal, F.B.M. and P.L. Tyack (Eds.), Animal Social Complexity: Intelligence, Culture, and Individualized Societies Harvard University Press, Cambridge, MA. pp. 32–56.

Wells, R.S. 2014. Social structure and life history of bottlenose dolphins near Sarasota Bay, Florida: Insights from four decades and five generations. Primates and cetaceans. pp. 149–172. doi: 10.1007/978-4-431-54523-1_8

Wells, R.S., M.K. Bassos, K.W. Urian, W.J. Carr, and M.D. Scott. 1996. Low-level monitoring of bottlenose dolphins, *Tursiops truncatus*, in Charlotte Harbor, Florida: 1990-1994. National Oceanic and Atmospheric Administration, National Marine Fisheries Service, Southeast Fisheries Science Center: Miami, FL. NOAA Technical Memorandum NMFS-SEFSC-384. 86 p.

Wells, R.S., M.K. Bassos, K.W. Urian, S.H. Shane, E.C.G. Owen, C.F. Weiss, W.J. Carr, and M.D. Scott. 1997. Low-level monitoring of bottlenose dolphins, *Tursiops truncatus*, in Pine Island Sound, Florida: 1996. National Oceanic and Atmospheric Administration, National Marine Fisheries Service, Southeast Fisheries Science Center: Miami, FL. Southeast Fisheries Center Contribution No. 40-WCNF601958. 86 p.

Wells, R.S., A. Natoli, and G. Braulik. 2019. *Tursiops truncatus* (errata version published in 2019). *The IUCN Red List of Threatened Species* 2019: e.T22563A156932432. https://dx.doi.org/10.2305/IUCN.UK.2019-1.RLTS.T22563A156932432.en

Wells, R.S., L.H. Schwacke, T.K. Rowles, B.C. Balmer, E. Zolman, T. Speakman, F.I. Townsend, M.C. Tumlin, A. Barleycorn, and K.A. Wilkinson. 2017. Ranging patterns of common bottlenose dolphins *Tursiops truncatus* in Barataria Bay, Louisiana, following the Deepwater Horizon oil spill. Endangered Species Research 33:159–180. doi: 10.3354/esr00732

Wells, R.S. and M.D. Scott. 1999. Bottlenose dolphin *Tursiops truncatus* (Montagu, 1821). In: Ridgway, S.H. and R. Harrison (Eds.), Handbook of marine mammals. Volume 6: The second book of dolphins and the porpoises. Academic Press, San Diego, CA. pp. 137–182.

Wells, R.S. and M.D. Scott. 2018. Bottlenose dolphin, *Tursiops truncatus*, common bottlenose dolphin. In: Würsig, B., J.G.M. Thewissen, and K.M. Kovacs (Eds.), Encyclopedia of Marine Mammals (Third Edition). Academic Press, London, UK. pp. 118–125. doi: 10.1016/B978-0-12-804327-1.00072-8

Wells, R.S., M.D. Scott, and A.B. Irvine. 1987. The social structure of free-ranging bottlenose dolphins. In: Genoways, H.H. (Ed.), Current Mammalogy. Plenum Press, New York, NY. pp. 247–305.

Wells, R.S., K.W. Urian, A.J. Read, M.K. Bassos, W.J. Carr, and M.D. Scott. 1996. Low-level monitoring of bottlenose dolphins, *Tursiops truncatus*, in Tampa Bay, Florida: 1988-1993. National Oceanic and Atmospheric Administration, National Marine Fisheries Service, Southeast Fisheries Science Center: Miami, FL. NOAA Technical Memorandum NMFS-SEFSC-385. 36 p.

Wilson, R.M., R.B. Tyson, J.A. Nelson, B.C. Balmer, J.P. Chanton, and D.P. Nowacek. 2017. Niche differentiation and prey selectivity among common bottlenose dolphins (*Tursiops truncatus*)

sighted in St. George Sound, Gulf of Mexico. Frontiers in Marine Science 4:235. doi: 10.3389/fmars.2017.00235

Common bottlenose dolphin − *Tursiops truncatus*

GoMx BSE/Pine Island Sound, Charlotte Harbor, Gasparilla Sound, Lemon Bay Stock

Overall Vulnerability Rank = Very High

Biological Sensitivity = Very High

Climate Exposure = Very High

Data Quality = 96% of scores ≥ 2

	Tursiops truncatus	Expert Scores	Data Quality	Expert Scores Plots (Portion by Category)
Sensitivity attributes	Prey/Diet Specificity	2.1	2.3	
	Habitat Specificity	3.7	2.3	
	Site Fidelity	3.9	3.0	
	Lifetime Reproductive Potential	1.9	2.3	
	Generation Length	2.8	2.3	
	Reproductive Plasticity	1.4	2.3	
	Migration	3.9	2.7	
	Home Range	3.8	3.0	
	Stock Abundance	2.9	2.7	
	Stock Abundance Trend	2.1	1.3	
	Cumulative Stressors	3.7	2.3	
	Sensitivity Score	Very High		
Exposure variables	Sea Surface Temperature (Change in mean)	3.9	2.7	
	Sea Surface Temperature (Change in variability)	1.1	2.7	
	Air Temperature (Change in mean)	3.9	2.7	
	Air Temperature (Change in variability)	1.1	2.7	
	Precipitation (Change in mean)	1.1	2.7	
	Precipitation (Change in variability)	1.1	2.7	
	Sea Surface Salinity (Change in mean)	1.7	2.7	
	Sea Surface Salinity (Change in variability)	1.5	2.7	
	Ocean pH (Change in mean)	3.9	2.7	
	Ocean pH (Change in variability)	1.1	2.7	
	Sea ice coverage (Change in mean)	1.0	3.0	
	Sea ice coverage (Change in variability)	1.0	3.0	
	Dissolved oxygen (Change in mean)	3.9	2.7	
	Dissolved oxygen (Change in variability)	1.1	2.7	
	Circulation	3.9	3.0	
	Sea level rise	3.9	2.7	
	Exposure Score	Very High		
Overall Vulnerability Rank		Very High		

Low
Moderate
High
Very High

Common bottlenose dolphin (GoMx BSE/Pine Island Sound, Charlotte Harbor, Gasparilla Sound, Lemon Bay Stock)

Tursiops truncatus

CVA Results Summary

Overall Climate Vulnerability Rank: Very High (100% certainty from bootstrap analysis).

Climate Exposure: Very High. Six exposure factors scored greater than 3.5: Air Temperature (Standard anomaly) (3.93), Dissolved oxygen (Standard anomaly) (3.93), Ocean pH (Standard anomaly) (3.93), Sea level rise (3.93), Sea Surface Temperature (Standard anomaly) (3.93), and Circulation (3.87).

Biological Sensitivity: Very High. Five sensitivity attributes scored greater than 3.5: Migration (3.93), Site Fidelity (3.93), Home Range (3.80), Cumulative Stressors (3.67), and Habitat Specificity (3.67).

Distributional Response: Very High

Abundance Response: Very High

Phenology Response: Very High

Data Quality: 96% of the data quality scores were 2 or greater, 91% of sensitivity attribute data quality scores were 2 or higher.

Background Information

Stock Description

This stock includes common bottlenose dolphins in Pine Island Sound, Charlotte Harbor, Gasparilla Sound, and Lemon Bay in the eastern Gulf of Mexico along the Florida coast (Hayes et al. 2022).

Prey/Diet Specificity

Generally, common bottlenose dolphins consume a large variety of fish and/or squid (Barros and Odell 1990; Mead and Potter 1990; Wells and Scott 2018). Common bottlenose dolphins seem to show a consistent preference for sound-producing fishes (e.g., sciaenids, toadfish; Berens McCabe et al. 2010; Bowen-Stevens et al. 2021). In Sarasota Bay, Florida, Rossman et al. (2015) found some individual common bottlenose dolphins to show diet specialization despite the broader study group showing a generalist diet. Specialization can be influenced by locally abundant prey species (Shane et al. 1986; Vollmer and Rosel 2013) or can result from resource partitioning amongst sympatric populations (e.g., Torres and Read 2009; Wilson et al. 2017).

Habitat Specificity

Common bottlenose dolphins are found in a variety of marine and estuarine habitats (Wells and Scott 2018).

Site Fidelity

Strong multi-year, year-round site fidelity has been demonstrated for bottlenose dolphins in this region (Wells et al. 1996a; Wells et al. 1997; Bassos-Hull et al. 2013). Other Gulf of Mexico bay, sound, and estuary (BSE) stocks show site fidelity (LaBrecque et al. 2015; Hayes et al. 2022), such as in Matagorda Bay, Espiritu Santo Bay, and San Luis Pass in Texas (Maze and Würsig 1999; Lynn and Würsig 2002; Irwin and Würsig 2004); Barataria Bay in Louisiana (Miller 2003; McDonald et al. 2017; Wells et al. 2017; Takeshita et al. 2021); Mississippi Sound in Mississippi (Hubard et al. 2004; Mackey 2010; Mullin et al. 2017); St. Andrew Bay (Balmer et al. 2019), St. Joseph Bay (Balmer et al. 2008; B. Balmer et al. 2018), St. Vincent Sound and Apalachicola Bay (Tyson et al. 2011), and Tampa Bay in Florida (Wells et al. 1996b; Urian et al. 2009).

Lifetime Reproductive Potential

Taylor et al. (2007) reported the interbirth interval of common bottlenose dolphins as 3.80 years, though other studies have shown it to be closer to three years (Leatherwood 1977; Wells and Scott 1999; Connor et al. 2000; Wells 2003).

Generally, females reach sexual maturity before males, with females reaching sexual maturity at 5–13 years (Sergeant et al. 1973; Perrin and Reilly 1984; Wells et al. 1987; Mead and Potter 1990; Mann et al. 2000; Wells 2000; Wells and Scott 2018).

Females reproduce through most of their adult lives (Leatherwood 1977; Hohn et al. 1989; Wells and Scott 1999; Wells 2000; Wells 2003), but may produce fewer calves after they reach 30 years of age (Wells 2000). Sarasota Bay common bottlenose dolphins can give birth when they are as young as 6 years of age, and can continue until they are 48 years old (Wells and Scott 2018). Reproductive lifespan would therefore be up to 42 years based on long-term observation studies (Wells 2014; Wells and Scott 2018).

Longitudinal studies from Sarasota Bay, Florida have found that female common bottlenose dolphins can live up to 67 years (Wells and Scott 2018). Studies based on growth layer groups in teeth suggest female common bottlenose dolphins can live to 41 years along the Texas coast (Fernandez and Hohn 1998) and to 30 years along the Mississippi coast (Mattson et al. 2006).

Generation Length

Taylor et al. (2007) reported common bottlenose dolphin generation length of 20.6–21.1 years.

Reproductive Plasticity

Common bottlenose dolphin calving is generally diffusely seasonal, with one or more peaks, although births have been reported from all seasons (Vollmer and Rosel 2013; Wells and Scott 2018). Calf sightings peak in the spring and summer months in Florida (Wells et al. 1987), Mississippi/Louisiana (Miller et al. 2010; Miller et al. 2013), and Texas (McHugh 1989; Henderson et al. 2007).

Migration

The BSE stocks in the Gulf of Mexico are thought to primarily comprise stable resident communities. However, transient or seasonal movements of some individuals into and out of a BSE stock area are recorded in some areas (LaBrecque et al. 2015; Hayes et al. 2022). Molecular genetic data support that some BSE stocks are genetically distinct from one another, and furthermore are differentiated from adjacent coastal populations (e.g., Sellas et al. 2005; Rosel et al. 2017). For example, Toms (2019) found fine-scale genetic population structure among five inshore systems in the Florida Panhandle and variable migration rates among populations.

Year-round residency has been reported for common bottlenose dolphins (Hayes et al. 2022), such as in Florida (Irvine and Wells 1972; Irvine et al. 1981; Wells 1986a; Wells 1986b; Wells et al. 1987; Scott et al. 1990; Wells 1991; Wells et al. 1996a; Wells et al. 1996b; Wells et al. 1997; Wells 2003; Balmer et al. 2008; Urian et al. 2009; Tyson et al. 2011; Bassos-Hull et al. 2013; B. Balmer et al. 2018; Balmer et al. 2019; Toms 2019), Texas (Shane 1990; Bräger 1993; Bräger et al. 1994; Fertl 1994; Weller 1998; Maze and Würsig 1999; Lynn and Würsig 2002; Irwin and Würsig 2004; Shane 2004), and Louisiana/Mississippi (Hubard et al. 2004; McDonald et al. 2017; Mullin et al. 2017; Takeshita et al. 2021).

Home Range

The home ranges of individuals within the Gulf of Mexico BSE stocks are considered to generally include the areas within the stock boundaries and brief excursions beyond the stock boundaries (Irvine et al. 1981; Wells 1986a; Wells 1986b; Wells 1991; Wells et al. 1996a; Wells et al. 1996b; Balmer et al. 2008; Wells 2014; Balmer et al. 2019), although some individuals show preference for small areas within the stock boundaries (Lynn and Würsig 2002; Urian et al. 2009; Mullin et al. 2017; Wells et al. 2017; Cloyed et al. 2021).

Stock Abundance

Bassos-Hull et al. (2013) estimated this stock's abundance at 826 individuals (CV = 0.09).

Stock Abundance Trend

Data are insufficient to complete an abundance trend analysis for this stock (Hayes et al. 2022).

Cumulative Stressors

Common bottlenose dolphins face a wide range of individual and cumulative natural and anthropogenic threats (see reviews by Vollmer and Rosel 2013; Wells and Scott 2018; and Wells et al. 2019). These include such threats as shark predation, harmful algal blooms, recreational and commercial fishing gear (Adimey et al. 2014; McHugh et al. 2021), vessel traffic, pollution, habitat alteration, and provisioning.

The nearshore and estuarine habitats along the Gulf of Mexico coast include waters adjacent to areas of high human population, some of which are highly industrialized, and areas of heavy agricultural impact. Studies of bottlenose dolphin tissues from southwest Florida have shown persistent organic pollutants (POPs) in blubber and blood samples (Kucklick et al. 2011; Balmer et al. 2015; J. Balmer et al. 2018). Similarly, mercury has been detected in common bottlenose dolphin tissues in Florida Gulf Coast waters (McCormack et al. 2020a, 2020b, 2022).

An Unusual Mortality Event (UME) was declared in 1990 for the Gulf of Mexico from Florida to Texas, with more than 300 common bottlenose dolphins stranding due to an undetermined cause, although morbillivirus and cold temperatures may have contributed (Litz et al. 2014). This stock was also within the range of the 2005–2006 UME in central west Florida caused by brevetoxin (Litz et al. 2014).

Further Reading

Hayes, S.A., E. Josephson, K. Maze-Foley, P.E. Rosel, and J. Wallace. 2022. U.S. Atlantic and Gulf of Mexico marine mammal stock assessments 2021. National Oceanic and Atmospheric Administration, National Marine Fisheries Service, Northeast Fisheries Science Center: Woods Hole, MA. NOAA Technical Memorandum NMFS-NE-288. 380 p. doi: 10.25923/6tt7-kc16

Wells, R.S. and M.D. Scott. 2018. Bottlenose Dolphin, *Tursiops truncatus*, Common Bottlenose Dolphin. In: Würsig, B., J.G.M. Thewissen, and K.M. Kovacs (Eds.), Encyclopedia of Marine Mammals (Third Edition). Academic Press, London, UK. pp. 118–125. doi: 10.1016/B978-0-12-804327-1.00072-8

Wells, R.S., A. Natoli, and G. Braulik. 2019. *Tursiops truncatus* (errata version published in 2019). The IUCN Red List of Threatened Species 2019: e.T22563A156932432. https://dx.doi.org/10.2305/IUCN.UK.2019-1.RLTS.T22563A156932432.en

References

Adimey, N.M., C.A. Hudak, J.R. Powell, K. Bassos-Hull, A. Foley, N.A. Farmer, L. White, and K. Minch. 2014. Fishery gear interactions from stranded bottlenose dolphins, Florida manatees and sea turtles in Florida, U.S.A. Marine Pollution Bulletin 81(1):103–115. doi: 10.1016/j.marpolbul.2014.02.008

Balmer, B., T. McDonald, F. Hornsby, J. Adams, J. Allen, A. Barleycorn, and L. Schwacke. 2018. Long-term trends in a northern Gulf of Mexico common bottlenose dolphin (*Tursiops truncatus*) population in the wake of the Deepwater Horizon oil spill. Journal of Cetacean Research and Management 18:1–9. doi: 10.47536/jcrm.v18i1.409

Balmer, B., S. Watwood, B. Quigley, T. Speakman, K. Barry, K. Mullin, P. Rosel, C. Sinclair, E. Zolman, and L. Schwacke. 2019. Common bottlenose dolphin (*Tursiops truncatus*) abundance and distribution patterns in St. Andrew Bay, Florida, USA. Aquatic Conservation: Marine and Freshwater Ecosystems 29(3):486–498. doi: 10.1002/aqc.3001

Balmer, B.C., R.S. Wells, S.M. Nowacek, D.P. Nowacek, L.H. Schwacke, W.A. McLellan, F.S. Scharf, T.K. Rowles, L.J. Hansen, T.R. Spradlin, and D.A. Pabst. 2008. Seasonal abundance and distribution patterns of common bottlenose dolphins (*Tursiops truncatus*) near St. Joseph Bay, Florida, USA. Journal of Cetacean Research and Management 10(2):157–167.

Balmer, B.C., G.M. Ylitalo, L.E. McGeorge, K.A. Baugh, D. Boyd, K.D. Mullin, P.E. Rosel, C. Sinclair, R.S. Wells, E.S. Zolman, and L.H. Schwacke. 2015. Persistent organic pollutants (POPs) in blubber of common bottlenose dolphins (*Tursiops truncatus*) along the northern Gulf of Mexico coast, USA. Science of the Total Environment 527-528:306–312. doi: 10.1016/j.scitotenv.2015.05.016

Balmer, J.E., G.M. Ylitalo, T.K. Rowles, K.D. Mullin, R.S. Wells, F.I. Townsend, R.W. Pearce, J.L. Bolton, E.S. Zolman, B.C. Balmer, and L.H. Schwacke. 2018. Persistent organic pollutants (POPs) in blood and blubber of common bottlenose dolphins (*Tursiops truncatus*) at three northern Gulf of Mexico sites following the Deepwater Horizon oil spill. Science of the Total Environment 621:130–137. doi: 10.1016/j.scitotenv.2017.11.209

Barros, N.B. and D.K. Odell. 1990. Food habits of bottlenose dolphins in the southeastern United States In: Leatherwood, S. and R.R. Reeves (Eds.), The Bottlenose Dolphin. Academic Press, San Diego, CA. pp. 309–328. doi: 10.1016/B978-0-12-440280-5.50020-2

Bassos-Hull, K., R.M. Perrtree, C.C. Shepard, S. Schilling, A.A. Barleycorn, J.B. Allen, B.C. Balmer, W.E. Pine, and R.S. Wells. 2013. Long-term site fidelity and seasonal abundance estimates of common bottlenose dolphins (*Tursiops truncatus*) along the southwest coast of Florida and responses to natural perturbations. Journal of Cetacean Research and Management 13(1):19–30. doi: 10.47536/jcrm.v13i1.551

Berens McCabe, E.J., D.P. Gannon, N.B. Barros, and R.S. Wells. 2010. Prey selection by resident common bottlenose dolphins (*Tursiops truncatus*) in Sarasota Bay, Florida. Marine Biology 157(5):931–942. doi: 10.1007/s00227-009-1371-2

Bowen-Stevens, S.R., D.P. Gannon, R.A. Hazelkorn, G. Lovewell, K.M. Volker, S. Smith, M.C. Tumlin, and J. Litz. 2021. Diet of common bottlenose dolphins, *Tursiops truncatus*, that stranded in and near Barataria Bay, Louisiana, 2010–2012. Southeastern Naturalist 20(1). doi: 10.1656/058.020.0113

Bräger, S. 1993. Diurnal and seasonal behavior patterns of bottlenose dolphins (*Tursiops truncatus*). Marine Mammal Science 9:434–438. doi: 10.1111/j.1748-7692.1993.tb00477.x

Bräger, S., B. Würsig, A. Acevedo, and T. Henningsen. 1994. Association patterns of bottle-nosed dolphins (*Tursiops-truncatus*) in Galveston Bay, Texas. Journal of Mammalogy 75(2):431–437. doi: 10.2307/1382564

Cloyed, C.S., R.M. Wilson, B.C. Balmer, A.A. Hohn, L.H. Schwacke, E.S. Zolman, M.C. Tumlin, R.S. Wells, A.A. Barleycorn, J.B. Allen, and R.H. Carmichael. 2021. Specialization of a mobile, apex predator affects trophic coupling among adjacent habitats. Scientific Reports 11(1):19611. doi: 10.1038/s41598-021-99017-1

Connor, R.C., R.S. Wells, J. Mann, and A.J. Read. 2000. The bottlenose dolphin: Social relationships in a fission-fusion society. In: Mann, J., R. Connor, P. Tyack, and H. Whitehead (Eds.), Cetacean Societies: Field Studies of Dolphins and Whales. University of Chicago Press, Chicago, IL. pp. 91–126.

Fernandez, S. and A.A. Hohn. 1998. Age, growth, and calving season of bottlenose dolphins, *Tursiops truncatus*, off coastal Texas. Fishery Bulletin 96(2):357–365.

Fertl, D. 1994. Occurrence patterns and behavior of bottlenose dolphins (*Tursiops truncatus*) in the Galveston ship channel, Texas. Texas Journal of Science 46(4):299–318.

Hayes, S.A., E. Josephson, K. Maze-Foley, P.E. Rosel, and J. Wallace. 2022. U.S. Atlantic and Gulf of Mexico marine mammal stock assessments 2021. National Oceanic and Atmospheric Administration, National Marine Fisheries Service, Northeast Fisheries Science Center: Woods Hole, MA. NOAA Technical Memorandum NMFS-NE-288. 380 p. doi: 10.25923/6tt7-kc16

Henderson, E.E. and B. Würsig. 2007. Behavior patterns of bottlenose dolphins in San Luis Pass, Texas. Gulf of Mexico Science 25(2):153–161. doi: 10.18785/goms.2502.06

Hohn, A.A., M.D. Scott, R.S. Wells, J.C. Sweeney, and A.B. Irvine. 1989. Growth layers in teeth from known-age, free-ranging bottlenose dolphins. Marine Mammal Science 5(4):315–342. doi: 10.1111/j.1748-7692.1989.tb00346.x

Hubard, C.W., K. Maze-Foley, K.D. Mullin, and W.W. Schroeder. 2004. Seasonal abundance and site fidelity of bottlenose dolphins (*Tursiops truncatus*) in Mississippi Sound. Aquatic Mammals 30(2):299–310. doi: 10.1578/am.30.2.2004.299

Irvine, A.B., M.D. Scott, R.S. Wells, and J.H. Kaufmann. 1981. Movements and activities of the Atlantic bottlenose dolphin, *Tursiops truncatus*, near Sarasota, Florida. Fishery Bulletin 79(4):671–688.

Irvine, B. and R.S. Wells. 1972. Results of attempts to tag Atlantic bottlenosed dolphins (*Tursiops truncatus*). Cetology 13:1–5.

Irwin, L.-J. and B. Würsig. 2004. A small resident community of bottlenose dolphins, *Tursiops truncatus*, in Texas: Monitoring recommendations. Gulf of Mexico Science 22(1):13–21. doi: 10.18785/goms.2201.02

Kucklick, J., L. Schwacke, R. Wells, A. Hohn, A. Guichard, J. Yordy, L. Hansen, E. Zolman, R. Wilson, J. Litz, D. Nowacek, T. Rowles, R. Pugh, B. Balmer, C. Sinclair, and P. Rosel. 2011. Bottlenose dolphins as indicators of persistent organic pollutants in the western North Atlantic Ocean and Northern Gulf of Mexico. Environmental Science & Technology 45(10):4270–4277. doi: 10.1021/es1042244

LaBrecque, E., C. Curtice, J. Harrison, S.M. Van Parijs, and P.N. Halpin. 2015. 3. Biologically Important Areas for cetaceans within U.S. Waters – Gulf of Mexico Region. Aquatic Mammals 41(1):30–38. doi: 10.1578/am.41.1.2015.30

Leatherwood, S. 1977. Some preliminary impressions on the numbers and social behavior of free-swimming bottlenosed dolphin calves (*Tursiops truncatus*) in the northern Gulf of Mexico. In: Ridgway, S.H. and K. Bernirschke (Eds.), Breeding Dolphins; Present Status, Suggestions for the Future. Final report to the US Marine Mammal Commission, Contract MM6AC009, MMC-76/07, Washington, DC. pp. 143–167.

Litz, J.A., M.A. Baran, S.R. Bowen-Stevens, R.H. Carmichael, K.M. Colegrove, L.P. Garrison, S.E. Fire, E.M. Fougeres, R. Hardy, S. Holmes, W. Jones, B.E. Mase-Guthrie, D.K. Odell, P.E. Rosel, J.T. Saliki, D.K. Shannon, S.F. Shippee, S.M. Smith, E.M. Stratton, M.C. Tumlin, H.R. Whitehead, G.A. Worthy, and T.K. Rowles. 2014. Review of historical unusual mortality events (UMEs) in the Gulf of Mexico (1990–2009): providing context for the multi-year northern Gulf of Mexico cetacean UME declared in 2010. Diseases of Aquatic Organisms 112(2):161–175. doi: 10.3354/dao02807

Lynn, S.K. and B. Würsig. 2002. Summer movement patterns of bottlenose dolphins in a Texas bay. Gulf of Mexico Science 20(1):25–37. doi: 10.18785/goms.2001.03

Mackey, A.D. 2010. Site fidelity and association patterns of bottlenose dolphins (*Tursiops truncatus*) in the Mississippi Sound. University of Southern Mississippi. Thesis. 133 p.

Mann, J., R.C. Connor, L.M. Barre, and M.R. Heithaus. 2000. Female reproductive success in bottlenose dolphins (*Tursiops* sp.): life history, habitat, provisioning, and group-size effects. Behavioral Ecology 11(2):210–219. doi: 10.1093/beheco/11.2.210

Mattson, M.C., K.D. Mullin, G.W. Ingram, and W. Hoggard. 2006. Age structure and growth of the bottlenose dolphin (*Tursiops truncatus*) from strandings in the Mississippi sound region of the north-central Gulf of Mexico from 1986 to 2003. Marine Mammal Science 22(3):654–666. doi: 10.1111/j.1748-7692.2006.00057.x

Maze, K.S. and B. Würsig. 1999. Bottlenosed dolphins of San Luis Pass, Texas: Occurrence patterns, site-fidelity, and habitat use. Aquatic Mammals 25:91–104.

McCormack, M.A., F. Battaglia, W.E. McFee, and J. Dutton. 2020a. Mercury concentrations in blubber and skin from stranded bottlenose dolphins (*Tursiops truncatus*) along the Florida and Louisiana coasts (Gulf of Mexico, USA) in relation to biological variables. Environmental Research 180:108886. doi: 10.1016/j.envres.2019.108886

McCormack, M.A., B.P. Jackson, and J. Dutton. 2020b. Relationship between mercury and selenium concentrations in tissues from stranded odontocetes in the northern Gulf of Mexico. Science of the Total Environment 749:141350. doi: 10.1016/j.scitotenv.2020.141350

McCormack, M.A., W.H. Nowlin, and J. Dutton. 2022. Effect of trophic position on mercury concentrations in bottlenose dolphins (*Tursiops truncatus*) from the northern Gulf of Mexico. Environmental Research 204(Pt B):112124. doi: 10.1016/j.envres.2021.112124

McDonald, T.L., F.E. Hornsby, T.R. Speakman, E.S. Zolman, K.D. Mullin, C. Sinclair, P.E. Rosel, L. Thomas, and L.H. Schwacke. 2017. Survival, density, and abundance of common bottlenose dolphins in

Barataria Bay (USA) following the Deepwater Horizon oil spill. Endangered Species Research 33:193–209. doi: 10.3354/esr00806

McHugh, K.A., A.A. Barleycorn, J.B. Allen, K. Bassos-Hull, G. Lovewell, D. Boyd, A. Panike, C. Cush, D. Fauquier, B. Mase, R.C. Lacy, M.R. Greenfield, D.I. Rubenstein, A. Weaver, A. Stone, L. Oliver, K. Morse, and R.S. Wells. 2021. Staying alive: Long-term success of bottlenose dolphin interventions in southwest Florida. Frontiers in Marine Science 7:624729. doi: 10.3389/fmars.2020.624729

McHugh, M.B. 1989. Population numbers and feeding behavior of the Atlantic bottlenose dolphin (*Tursiops truncatus*) near Aransas Pass, Texas. University of Texas at Austin. Thesis. 96 p.

Mead, J.G. and C.W. Potter. 1990. Natural history of bottlenose dolphins along the central Atlantic coast of the United States. In: Leatherwood, S. and R.R. Reeves (Eds.), The Bottlenose Dolphin. Academic Press, San Diego, CA. pp. 165–195.

Miller, C.E. 2003. Abundance trends and environmental habitat usage patterns of bottlenose dolphins (*Tursiops truncatus*) in lower Barataria and Caminada bays, Louisiana. Louisiana State University and Agricultural & Mechanical College. Dissertation. 125 p.

Miller, L.J., A.D. Mackey, T. Hoffland, M. Solangi, and S.A. Kuczaj. 2010. Potential effects of a major hurricane on Atlantic bottlenose dolphin (*Tursiops truncatus*) reproduction in the Mississippi Sound. Marine Mammal Science 26(3):707–715. doi: 10.1111/j.1748-7692.2010.00371.x

Miller, L.J., A.D. Mackey, M. Solangi, and S.A. Kuczaj. 2013. Population abundance and habitat utilization of bottlenose dolphins in the Mississippi Sound. Aquatic Conservation: Marine and Freshwater Ecosystems 23(1):145–151. doi: 10.1002/aqc.2278

Mullin, K.D., T. McDonald, R.S. Wells, B.C. Balmer, T. Speakman, C. Sinclair, E.S. Zolman, F. Hornsby, S.M. McBride, K.A. Wilkinson, and L.H. Schwacke. 2017. Density, abundance, survival, and ranging patterns of common bottlenose dolphins (*Tursiops truncatus*) in Mississippi Sound following the Deepwater Horizon oil spill. PLoS ONE 12(10):e0186265. doi: 10.1371/journal.pone.0186265

Perrin, W.F. and S.B. Reilly. 1984. Reproductive parameters of dolphins and small whales of the family Delphinidae. In: Perrin, W.F., R.L. Brownell, Jr., and D.P. DeMaster (Eds.), Reproduction in Whales, Dolphins, and Porpoises. pp. 97–134.

Rosel, P.E., L.A. Wilcox, C. Sinclair, T.R. Speakman, M.C. Tumlin, J.A. Litz, and E.S. Zolman. 2017. Genetic assignment to stock of stranded common bottlenose dolphins in southeastern Louisiana after the Deepwater Horizon oil spill. Endangered Species Research 33:221–234. doi: 10.3354/esr00780

Rossman, S., P.H. Ostrom, M. Stolen, N.B. Barros, H. Gandhi, C.A. Stricker, and R.S. Wells. 2015. Individual specialization in the foraging habits of female bottlenose dolphins living in a trophically diverse and habitat rich estuary. Oecologia 178(2):415–425. doi: 10.1007/s00442-015-3241-6

Scott, M.D. and S.J. Chivers. 1990. Distribution and herd structure of bottlenose dolphins in the eastern tropical Pacific Ocean. In: Leatherwood, S. and R.R. Reeves (Eds.), The Bottlenose Dolphin. Academic Press, San Diego, CA. pp. 387–402.

Sellas, A.B., R.S. Wells, and P.E. Rosel. 2005. Mitochondrial and nuclear DNA analyses reveal fine scale geographic structure in bottlenose dolphins (*Tursiops truncatus*) in the Gulf of Mexico. Conservation Genetics 6(5):715–728. doi: 10.1007/s10592-005-9031-7

Sergeant, D.E., D.K. Caldwell, and M.C. Caldwell. 1973. Age, growth, and maturity of bottlenosed dolphin (*Tursiops-truncatus*) from northeast Florida. Journal of the Fisheries Research Board of Canada 30(7):1009–1011. doi: 10.1139/f73-165

Shane, S.H. 1990. Behavior and ecology of the bottlenose dolphin at Sanibel Island, Florida. In: Leatherwood, S. and R.R. Reeves (Eds.), The Bottlenose Dolphin. Academic Press, San Diego, CA. pp. 245–265. doi: 10.1016/B978-0-12-440280-5.50016-0

Shane, S.H. 2004. Residence patterns, group characteristics, and association patterns of bottlenose dolphins near Sanibel Island, Florida. Gulf of Mexico Science 22(1):1–12. doi: 10.18785/goms.2201.01

Shane, S.H., R.S. Wells, and B. Würsig. 1986. Ecology, behavior and social organization of the bottlenose dolphin: A review. Marine Mammal Science 2:34–63. doi: 10.1111/j.1748-7692.1986.tb00026.x

Takeshita, R., B.C. Balmer, F. Messina, E.S. Zolman, L. Thomas, R.S. Wells, C.R. Smith, T.K. Rowles, and L.H. Schwacke. 2021. High site-fidelity in common bottlenose dolphins despite low salinity exposure and associated indicators of compromised health. PLoS ONE 16(9):e0258031. doi: 10.1371/journal.pone.0258031

Taylor, B.L., S.J. Chivers, J. Larese, and W.F. Perrin. 2007. Generation length and percent mature estimates for IUCN assessments of cetaceans. National Oceanic and Atmospheric Administration, National Marine Fisheries Service, Southwest Fisheries Science Center: La Jolla, CA. Administrative Report LJ-07-01. 24 p.

Toms, C. 2019. Filling the gaps: Common bottlenose dolphin (*Tursiops truncatus*) population dynamics, structure, and connectivity within Florida panhandle bays, sounds, and estuaries. University of Central Florida. Dissertation. 373 p.

Torres, L.G. and A.J. Read. 2009. Where to catch a fish? The influence of foraging tactics on the ecology of bottlenose dolphins (*Tursiops truncatus*) in Florida Bay, Florida. Marine Mammal Science 25(4):797–815. doi: 10.1111/j.1748-7692.2009.00297.x

Tyson, R.B., S.M. Nowacek, and D.P. Nowacek. 2011. Community structure and abundance of bottlenose dolphins *Tursiops truncatus* in coastal waters of the northeast Gulf of Mexico. Marine Ecology Progress Series 438:253–265. doi: 10.3354/meps09292

Urian, K.W., S. Hofmann, R.S. Wells, and A.J. Read. 2009. Fine-scale population structure of bottlenose dolphins (*Tursiops truncatus*) in Tampa Bay, Florida. Marine Mammal Science 25(3):619–638. doi: 10.1111/j.1748-7692.2009.00284.x

Vollmer, N.L. and P.E. Rosel. 2013. A review of common bottlenose dolphins (*Tursiops truncatus truncatus*) in the Northern Gulf of Mexico: Population biology, potential threats, and management. Southeastern Naturalist 12(m6):1–43. doi: 10.1656/058.012.m601

Weller, D.W. 1998. Global and regional variation in the biology and behavior of bottlenose dolphins. Texas A&M University. Dissertation. 142 p.

Wells, R.S. 1986a. Population structure of bottlenose dolphins: Behavioral studies along the central west coast of Florida. Contract Report to National Marine Fisheries Service, Southeast Fisheries Center. Contract No. 45-WCNF-5-003BB. 58 p.

Wells, R.S. 1986b. Structural aspects of dolphin societies. University of California, Santa Cruz. Dissertation. 234 p.

Wells, R.S. 1991. The role of long-term study in understanding the social structure of a bottlenose dolphin community. In: Pryor, K. and K. Norris (Eds.), Dolphin Societies: Discoveries and Puzzles. University of California Press, Berkeley, CA. pp. 199–226.

Wells, R.S. 2000. Reproduction in wild bottlenose dolphins: Overview of patterns observed during a long-term study. In: Duffield, D. and T. Robeck (Eds.), Bottlenose Dolphin Reproduction Workshop. AZA Marine Mammal Taxon Advisory Group, Silver Spring, MD. pp. 57–74.

Wells, R.S. 2003. Dolphin social complexity: Lessons from long-term study and life history. In: de Waal, F.B.M. and P.L. Tyack (Eds.), Animal Social Complexity: Intelligence, Culture, and Individualized Societies Harvard University Press, Cambridge, MA. pp. 32–56.

Wells, R.S. 2014. Social structure and life history of bottlenose dolphins near Sarasota Bay, Florida: Insights from four decades and five generations. Primates and cetaceans. pp. 149–172. doi: 10.1007/978-4-431-54523-1_8

Wells, R.S., M.K. Bassos, K.W. Urian, W.J. Carr, and M.D. Scott. 1996. Low-level monitoring of bottlenose dolphins, *Tursiops truncatus*, in Charlotte Harbor, Florida: 1990-1994. National Oceanic and Atmospheric Administration, National Marine Fisheries Service, Southeast Fisheries Science Center: Miami, FL. NOAA Technical Memorandum NMFS-SEFSC-384. 86 p.

Wells, R.S., M.K. Bassos, K.W. Urian, S.H. Shane, E.C.G. Owen, C.F. Weiss, W.J. Carr, and M.D. Scott. 1997. Low-level monitoring of bottlenose dolphins, *Tursiops truncatus*, in Pine Island Sound, Florida: 1996. National Oceanic and Atmospheric Administration, National Marine Fisheries Service, Southeast Fisheries Science Center: Miami, FL. Southeast Fisheries Center Contribution No. 40-WCNF601958. 86 p.

Wells, R.S., A. Natoli, and G. Braulik. 2019. *Tursiops truncatus* (errata version published in 2019). *The IUCN Red List of Threatened Species* 2019: e.T22563A156932432. https://dx.doi.org/10.2305/IUCN.UK.2019-1.RLTS.T22563A156932432.en

Wells, R.S., L.H. Schwacke, T.K. Rowles, B.C. Balmer, E. Zolman, T. Speakman, F.I. Townsend, M.C. Tumlin, A. Barleycorn, and K.A. Wilkinson. 2017. Ranging patterns of common bottlenose dolphins *Tursiops truncatus* in Barataria Bay, Louisiana, following the Deepwater Horizon oil spill. Endangered Species Research 33:159–180. doi: 10.3354/esr00732

Wells, R.S. and M.D. Scott. 1999. Bottlenose dolphin *Tursiops truncatus* (Montagu, 1821). In: Ridgway, S.H. and R. Harrison (Eds.), Handbook of marine mammals. Volume 6: The second book of dolphins and the porpoises. Academic Press, San Diego, CA. pp. 137–182.

Wells, R.S. and M.D. Scott. 2018. Bottlenose dolphin, *Tursiops truncatus*, common bottlenose dolphin. In: Würsig, B., J.G.M. Thewissen, and K.M. Kovacs (Eds.), Encyclopedia of Marine Mammals (Third Edition). Academic Press, London, UK. pp. 118–125. doi: 10.1016/B978-0-12-804327-1.00072-8

Wells, R.S., M.D. Scott, and A.B. Irvine. 1987. The social structure of free-ranging bottlenose dolphins. In: Genoways, H.H. (Ed.), Current Mammalogy. Plenum Press, New York, NY. pp. 247–305.

Wells, R.S., K.W. Urian, A.J. Read, M.K. Bassos, W.J. Carr, and M.D. Scott. 1996. Low-level monitoring of bottlenose dolphins, *Tursiops truncatus*, in Tampa Bay, Florida: 1988-1993. National Oceanic and Atmospheric Administration, National Marine Fisheries Service, Southeast Fisheries Science Center: Miami, FL. NOAA Technical Memorandum NMFS-SEFSC-385. 36 p.

Wilson, R.M., R.B. Tyson, J.A. Nelson, B.C. Balmer, J.P. Chanton, and D.P. Nowacek. 2017. Niche differentiation and prey selectivity among common bottlenose dolphins (*Tursiops truncatus*) sighted in St. George Sound, Gulf of Mexico. Frontiers in Marine Science 4:235. doi: 10.3389/fmars.2017.00235

Common bottlenose dolphin − *Tursiops truncatus*

GoMx BSE/Sabine Lake Stock

Overall Vulnerability Rank = Very High

Biological Sensitivity = Very High

Climate Exposure = Very High

Data Quality = 96% of scores ≥ 2

	Tursiops truncatus	Expert Scores	Data Quality	Expert Scores Plots (Portion by Category)
Sensitivity attributes	Prey/Diet Specificity	2.4	2.2	
	Habitat Specificity	2.5	2.0	
	Site Fidelity	3.4	2.5	
	Lifetime Reproductive Potential	1.5	2.2	
	Generation Length	2.9	2.2	
	Reproductive Plasticity	1.2	2.0	
	Migration	3.8	2.5	
	Home Range	3.5	2.5	
	Stock Abundance	3.8	2.0	
	Stock Abundance Trend	2.0	1.5	
	Cumulative Stressors	3.2	2.5	
	Sensitivity Score	Very High		
Exposure variables	Sea Surface Temperature (Change in mean)	4.0	2.8	
	Sea Surface Temperature (Change in variability)	1.0	2.8	
	Air Temperature (Change in mean)	4.0	2.8	
	Air Temperature (Change in variability)	1.0	2.8	
	Precipitation (Change in mean)	1.0	2.8	
	Precipitation (Change in variability)	1.0	2.8	
	Sea Surface Salinity (Change in mean)	2.0	2.8	
	Sea Surface Salinity (Change in variability)	1.0	2.8	
	Ocean pH (Change in mean)	4.0	2.8	
	Ocean pH (Change in variability)	1.0	2.8	
	Sea ice coverage (Change in mean)	1.0	2.8	
	Sea ice coverage (Change in variability)	1.0	2.8	
	Dissolved oxygen (Change in mean)	4.0	2.8	
	Dissolved oxygen (Change in variability)	1.0	2.8	
	Circulation	3.8	2.5	
	Sea level rise	4.0	2.8	
	Exposure Score	Very High		
	Overall Vulnerability Rank	Very High		

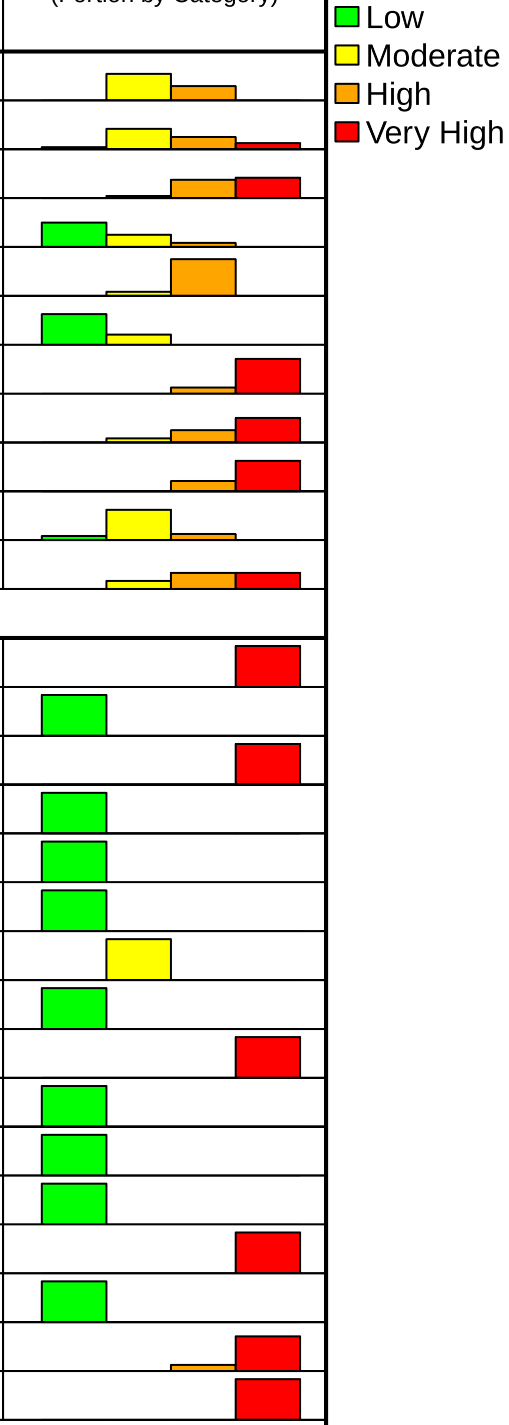

Common bottlenose dolphin (GoMx BSE/Sabine Lake Stock)

Tursiops truncatus

CVA Results Summary

Overall Climate Vulnerability Rank: Very High (100% certainty from bootstrap analysis).

Climate Exposure: Very High. Six exposure factors scored greater than 3.5: Air Temperature (Standard anomaly) (4.00), Dissolved oxygen (Standard anomaly) (4.00), Ocean pH (Standard anomaly) (4.00), Sea level rise (4.00), Sea Surface Temperature (Standard anomaly) (4.00), and Circulation (3.85).

Biological Sensitivity: Very High. Three sensitivity attributes scored greater than or equal to 3.5: Migration (3.85), Species Abundance (3.75), and Home Range (3.50).

Distributional Response: High

Abundance Response: High

Phenology Response: High

Data Quality: 96% of the data quality scores were 2 or greater, 91% of sensitivity attribute data quality scores were 2 or higher.

Background Information

Stock Description

This stock includes common bottlenose dolphins in Sabine Lake in the northwest Gulf of Mexico along the Texas and Louisiana coasts (Hayes et al. 2022).

Prey/Diet Specificity

Generally, common bottlenose dolphins consume a large variety of fish and/or squid (Barros and Odell 1990; Mead and Potter 1990; Wells and Scott 2018). Common bottlenose dolphins seem to show a consistent preference for sound-producing fishes (e.g., sciaenids, toadfish; Berens McCabe et al. 2010; Bowen-Stevens et al. 2021). In Sarasota Bay, Florida, Rossman et al. (2015) found some individual common bottlenose dolphins to show diet specialization despite the broader study group showing a generalist diet. Specialization can be influenced by locally abundant prey species (Shane et al. 1986; Vollmer and Rosel 2013) or can result from resource partitioning amongst sympatric populations (e.g., Torres and Read 2009; Wilson et al. 2017).

Habitat Specificity

Common bottlenose dolphins are found in a variety of marine and estuarine habitats (Wells and Scott 2018). The primary source of freshwater into Sabine Lake is the Neches and Sabine rivers, and their water flow is regulated by upstream hydroelectric dams that contribute to seasonal fluctuations in the surface salinity of the Sabine Lake area. The Sabine Lake estuary has been extensively modified with deep engineered channels to accommodate large vessels en route to Port Arthur and Beaumont, TX.

The engineered channels removed previous restrictions to Gulf tidal influx, and as a result the salinity in the channels is highly stratified (Ward 1980).

Site Fidelity

Preliminary data from photo-ID mark-recapture surveys suggests there may be common bottlenose dolphins with site-fidelity to Sabine Lake (Ronje et al. 2020a). Dolphins were also found to use the Gulf waters near the end of and adjacent to the Sabine Pass Channel, particularly during the winter season (Ronje et al. 2020b), possibly because Sabine Lake salinity is typically lowest in winter (Orlando et al. 1993). Few surveys have been conducted in Sabine Lake and more research needs to be conducted to better describe the site fidelity of common bottlenose dolphins to the Sabine Lake area. However, common bottlenose dolphins in other Gulf of Mexico bay, sound, and estuary (BSE) stocks show site fidelity (LaBrecque et al. 2015; Hayes et al. 2022), such as in Matagorda Bay, Espiritu Santo Bay, and San Luis Pass in Texas (Maze and Würsig 1999; Lynn and Würsig 2002; Irwin and Würsig 2004); Barataria Bay in Louisiana (Miller 2003; McDonald et al. 2017; Wells et al. 2017; Takeshita et al. 2021); Mississippi Sound in Mississippi (Hubard et al. 2004; Mackey 2010; Mullin et al. 2017); Charlotte Harbor and Pine Island Sound (Bassos-Hull et al. 2013), Sarasota Bay in Florida (Wells 1986a; Wells 1991; Wells 2014), St. Andrew Bay (Balmer et al. 2019), St. Joseph Bay (Balmer et al. 2008; Balmer et al. 2018), St. Vincent Sound and Apalachicola Bay (Tyson et al. 2011), and Tampa Bay in Florida (Wells et al. 1996b; Urian et al. 2009).

Lifetime Reproductive Potential

Taylor et al. (2007) reported the interbirth interval of common bottlenose dolphins as 3.80 years, though other studies have shown it to be closer to three years (Leatherwood 1977; Wells and Scott 1999; Connor et al. 2000; Wells 2003).

Generally, females reach sexual maturity before males, with females reaching sexual maturity at 5–13 years (Sergeant et al. 1973; Perrin and Reilly 1984; Wells et al. 1987; Mead and Potter 1990; Mann et al. 2000; Wells 2000; Wells and Scott 2018).

Females reproduce through most of their adult lives (Leatherwood 1977; Hohn et al. 1989; Wells and Scott 1999; Wells 2000; Wells 2003), but may produce fewer calves after they reach 30 years of age (Wells 2000). Sarasota Bay common bottlenose dolphins can give birth when they are as young as 6 years of age, and can continue until they are 48 years old (Wells and Scott 2018). Reproductive lifespan would therefore be up to 42 years based on long-term observation studies (Wells 2014; Wells and Scott 2018).

Longitudinal studies from Sarasota Bay, Florida have found that female common bottlenose dolphins can live up to 67 years (Wells and Scott 2018). Studies based on growth layer groups in teeth suggest female common bottlenose dolphins can live to 41 years along the Texas coast (Fernandez and Hohn 1998) and to 30 years along the Mississippi coast (Mattson et al. 2006).

Generation Length

Taylor et al. (2007) reported common bottlenose dolphin generation length of 20.6–21.1 years.

Reproductive Plasticity

Common bottlenose dolphin calving is generally diffusely seasonal, with one or more peaks, although births have been reported from all seasons (Vollmer and Rosel 2013; Wells and Scott 2018). Calf sightings peak in the spring and summer months in Florida (Wells et al. 1987), Mississippi/Louisiana (Miller et al. 2010; Miller et al. 2013), and Texas (McHugh 1989; Henderson et al. 2007).

Migration

The BSE stocks in the Gulf of Mexico are thought to primarily comprise stable resident communities. However, transient or seasonal movements of some individuals into and out of a BSE stock area are recorded in some areas (LaBrecque et al. 2015; Hayes et al. 2022). Molecular genetic data support that some BSE stocks are genetically distinct from one another, and furthermore are differentiated from adjacent coastal populations (e.g., Sellas et al. 2005; Rosel et al. 2017). For example, Toms (2019) found fine-scale genetic population structure among five inshore systems in the Florida Panhandle and variable migration rates among populations.

Year-round residency has been reported for common bottlenose dolphins (Hayes et al. 2022), such as in Florida (Irvine and Wells 1972; Irvine et al. 1981; Wells 1986a; Wells 1986b; Wells et al. 1987; Scott et al. 1990; Wells 1991; Wells et al. 1996a; Wells et al. 1996b; Wells et al. 1997; Wells 2003; Balmer et al. 2008; Urian et al. 2009; Tyson et al. 2011; Bassos-Hull et al. 2013; Balmer et al. 2018; Balmer et al. 2019; Toms 2019), Texas (Shane 1990; Bräger 1993; Bräger et al. 1994; Fertl 1994; Weller 1998; Maze and Würsig 1999; Lynn and Würsig 2002; Irwin and Würsig 2004; Shane 2004), and Louisiana/Mississippi (Hubard et al. 2004; McDonald et al. 2017; Mullin et al. 2017; Takeshita et al. 2021).

Ronje et al. (2020b) photo-identified 25 apparently transient individuals that were observed in both the Sabine Lake and Galveston Bay stock areas, a distance of approximately 90 km to the Gulf pass of each bay. Some individuals observed in Sabine Lake were also observed well into the Galveston Bay estuary, including upper Bay and the western side of Trinity Bay (Ronje et al. 2020b). It is not known if those individuals are transients or were seasonally migrating.

Home Range

The home ranges of individuals within the Gulf of Mexico BSE stocks are considered to generally include the areas within the stock boundaries and brief excursions beyond the stock boundaries (Irvine et al. 1981; Wells 1986a; Wells 1986b; Wells 1991; Wells et al. 1996a; Wells et al. 1996b; Balmer et al. 2008; Wells 2014; Balmer et al. 2019), although some individuals show preference for small areas within the stock boundaries (Lynn and Würsig 2002; Urian et al. 2009; Mullin et al. 2017; Wells et al. 2017). Photo-identified individuals observed in both winter and summer seasons in Sabine Lake were found to range at the extreme north and south margins of the Sabine Lake estuary (including the Neches and Sabine Rivers at the north end), and anecdotal reports suggest dolphins range as far west as Beaumont, TX (Ronje et al. 2020b) but more data on individual site-fidelity needs to be collected to better assess the home range of resident individuals.

Stock Abundance

Ronje et al. (2020b) used photo-ID mark-recapture encounter histories to estimate abundance of this stock. By including all distinctive individuals observed in the bay or Gulf pass to the bay in ≥ 1 season plus individuals observed in coastal waters during both summer and winter seasons (but not observed in other survey areas), Ronje et al. (2020b) estimated the common bottlenose dolphin population of Sabine Lake to be 121.6 individuals (95% CI=73.0–170.3) in the winter and 162.2 individuals (95% CI=114.3–210.2) in the summer.

Stock Abundance Trend

Data are insufficient to complete a trend analysis for this stock (Hayes et al. 2022).

Cumulative Stressors

Common bottlenose dolphins face a wide range of individual and cumulative natural and anthropogenic threats (see reviews by Vollmer and Rosel 2013; Phillips and Rosel 2014; Wells and Scott 2018; and Wells et al. 2019). These include such threats as shark predation, harmful algal blooms, recreational and commercial fishing gear, vessel traffic, pollution, habitat alteration, and provisioning.

Phillips and Rosel (2014) reviewed the cumulative stressors influencing the Sabine Lake system. Potential health threats range from contagious disease (e.g., *Brucella*), harmful algal blooms (HABs; Fire et al. 2011, 2020), and anthropogenic activities: mining, crude oil exploration, transport, and refinement, commercial fisheries, agricultural runoff in the form of pesticide and fertilizer, heavy metals and other chemical contaminants, marine debris, shipping and dredging, noise, tourism and boat traffic. Natural events may also pose threats: hypoxia resulting from HABs, adverse weather (e.g., hurricane storm surge), habitat loss, and climate change (Phillips and Rosel 2014).

Unusual Mortality Events (UMEs) have been declared for common bottlenose dolphins in waters of Texas with morbillivirus the confirmed cause in 1994, a suspected cause in 1992 along with environmental conditions, and an undetermined cause in 2008 (Litz et al. 2014). This stock was also within the range of the 2007 UME in which 66 common bottlenose dolphins stranded in northeast Texas and west Louisiana due to an undetermined cause (Litz et al. 2014). A UME was declared in 1990 for the Gulf of Mexico from Florida to Texas, with more than 300 common bottlenose dolphins stranding due to an undetermined cause, although morbillivirus and cold temperatures may have contributed (Litz et al. 2014).

Distribution and Sightings

Sighting maps for this stock are presented in Ronje et al. (2020b).

Further Reading

Hayes, S.A., E. Josephson, K. Maze-Foley, P.E. Rosel, and J. Wallace. 2022. U.S. Atlantic and Gulf of Mexico marine mammal stock assessments 2021. National Oceanic and Atmospheric Administration, National Marine Fisheries Service, Northeast Fisheries Science Center: Woods Hole, MA. NOAA Technical Memorandum NMFS-NE-288. 380 p. doi: 10.25923/6tt7-kc16

Wells, R.S. and M.D. Scott. 2018. Bottlenose Dolphin, *Tursiops truncatus*, Common Bottlenose Dolphin. In: Würsig, B., J.G.M. Thewissen, and K.M. Kovacs (Eds.), Encyclopedia of Marine Mammals (Third Edition). Academic Press, London, UK. pp. 118–125. doi: 10.1016/B978-0-12-804327-1.00072-8

Wells, R.S., A. Natoli, and G. Braulik. 2019. *Tursiops truncatus* (errata version published in 2019). The IUCN Red List of Threatened Species 2019: e.T22563A156932432. doi: 10.2305/IUCN.UK.2019-1.RLTS.T22563A156932432.en

References

Balmer, B., T. McDonald, F. Hornsby, J. Adams, J. Allen, A. Barleycorn, and L. Schwacke. 2018. Long-term trends in a northern Gulf of Mexico common bottlenose dolphin (*Tursiops truncatus*) population in the wake of the Deepwater Horizon oil spill. Journal of Cetacean Research and Management 18:1–9. doi: 10.47536/jcrm.v18i1.409

Balmer, B., S. Watwood, B. Quigley, T. Speakman, K. Barry, K. Mullin, P. Rosel, C. Sinclair, E. Zolman, and L. Schwacke. 2019. Common bottlenose dolphin (*Tursiops truncatus*) abundance and distribution patterns in St. Andrew Bay, Florida, USA. Aquatic Conservation: Marine and Freshwater Ecosystems 29(3):486–498. doi: 10.1002/aqc.3001

Balmer, B.C., R.S. Wells, S.M. Nowacek, D.P. Nowacek, L.H. Schwacke, W.A. McLellan, F.S. Scharf, T.K. Rowles, L.J. Hansen, T.R. Spradlin, and D.A. Pabst. 2008. Seasonal abundance and distribution patterns of common bottlenose dolphins (*Tursiops truncatus*) near St. Joseph Bay, Florida, USA. Journal of Cetacean Research and Management 10(2):157–167.

Barros, N.B. and D.K. Odell. 1990. Food habits of bottlenose dolphins in the southeastern United States In: Leatherwood, S. and R.R. Reeves (Eds.), The Bottlenose Dolphin. Academic Press, San Diego, CA. pp. 309–328. doi: 10.1016/B978-0-12-440280-5.50020-2

Bassos-Hull, K., R.M. Perrtree, C.C. Shepard, S. Schilling, A.A. Barleycorn, J.B. Allen, B.C. Balmer, W.E. Pine, and R.S. Wells. 2013. Long-term site fidelity and seasonal abundance estimates of common bottlenose dolphins (*Tursiops truncatus*) along the southwest coast of Florida and responses to natural perturbations. Journal of Cetacean Research and Management 13(1):19–30. doi: 10.47536/jcrm.v13i1.551

Berens McCabe, E.J., D.P. Gannon, N.B. Barros, and R.S. Wells. 2010. Prey selection by resident common bottlenose dolphins (*Tursiops truncatus*) in Sarasota Bay, Florida. Marine Biology 157(5):931–942. doi: 10.1007/s00227-009-1371-2

Bowen-Stevens, S.R., D.P. Gannon, R.A. Hazelkorn, G. Lovewell, K.M. Volker, S. Smith, M.C. Tumlin, and J. Litz. 2021. Diet of common bottlenose dolphins, *Tursiops truncatus*, that stranded in and near Barataria Bay, Louisiana, 2010–2012. Southeastern Naturalist 20(1). doi: 10.1656/058.020.0113

Bräger, S. 1993. Diurnal and seasonal behavior patterns of bottlenose dolphins (*Tursiops truncatus*). Marine Mammal Science 9:434–438. doi: 10.1111/j.1748-7692.1993.tb00477.x

Bräger, S., B. Würsig, A. Acevedo, and T. Henningsen. 1994. Association patterns of bottle-nosed dolphins (*Tursiops-truncatus*) in Galveston Bay, Texas. Journal of Mammalogy 75(2):431–437. doi: 10.2307/1382564

Connor, R.C., R.S. Wells, J. Mann, and A.J. Read. 2000. The bottlenose dolphin: Social relationships in a fission-fusion society. In: Mann, J., R. Connor, P. Tyack, and H. Whitehead (Eds.), Cetacean Societies: Field Studies of Dolphins and Whales. University of Chicago Press, Chicago, IL. pp. 91–126.

Fernandez, S. and A.A. Hohn. 1998. Age, growth, and calving season of bottlenose dolphins, *Tursiops truncatus*, off coastal Texas. Fishery Bulletin 96(2):357–365.

Fertl, D. 1994. Occurrence patterns and behavior of bottlenose dolphins (*Tursiops truncatus*) in the Galveston ship channel, Texas. Texas Journal of Science 46(4):299–318.

Fire, S.E., T.A. Leighfield, G.A. Miller, S. Piwetz, E.R. Sabater, and H. Whitehead. 2020. Association between red tide exposure and detection of corresponding neurotoxins in bottlenose dolphins from Texas waters during 2007-2017. Marine Environmental Research 162:105191. doi: 10.1016/j.marenvres.2020.105191

Fire, S.E., Z. Wang, M. Byrd, H.R. Whitehead, J. Paternoster, and S.L. Morton. 2011. Co-occurrence of multiple classes of harmful algal toxins in bottlenose dolphins (*Tursiops truncatus*) stranding during an unusual mortality event in Texas, USA. Harmful Algae 10(3):330–336. doi: 10.1016/j.hal.2010.12.001

Hayes, S.A., E. Josephson, K. Maze-Foley, P.E. Rosel, and J. Wallace. 2022. U.S. Atlantic and Gulf of Mexico marine mammal stock assessments 2021. National Oceanic and Atmospheric Administration, National Marine Fisheries Service, Northeast Fisheries Science Center: Woods Hole, MA. NOAA Technical Memorandum NMFS-NE-288. 380 p. doi: 10.25923/6tt7-kc16

Henderson, E.E. and B. Würsig. 2007. Behavior patterns of bottlenose dolphins in San Luis Pass, Texas. Gulf of Mexico Science 25(2):153–161. doi: 10.18785/goms.2502.06

Hohn, A.A., M.D. Scott, R.S. Wells, J.C. Sweeney, and A.B. Irvine. 1989. Growth layers in teeth from known-age, free-ranging bottlenose dolphins. Marine Mammal Science 5(4):315–342. doi: 10.1111/j.1748-7692.1989.tb00346.x

Hubard, C.W., K. Maze-Foley, K.D. Mullin, and W.W. Schroeder. 2004. Seasonal abundance and site fidelity of bottlenose dolphins (*Tursiops truncatus*) in Mississippi Sound. Aquatic Mammals 30(2):299–310. doi: 10.1578/am.30.2.2004.299

Irvine, A.B., M.D. Scott, R.S. Wells, and J.H. Kaufmann. 1981. Movements and activities of the Atlantic bottlenose dolphin, *Tursiops truncatus*, near Sarasota, Florida. Fishery Bulletin 79(4):671–688.

Irvine, B. and R.S. Wells. 1972. Results of attempts to tag Atlantic bottlenosed dolphins (*Tursiops truncatus*). Cetology 13:1–5.

Irwin, L.-J. and B. Würsig. 2004. A small resident community of bottlenose dolphins, *Tursiops truncatus*, in Texas: Monitoring recommendations. Gulf of Mexico Science 22(1):13–21. doi: 10.18785/goms.2201.02

LaBrecque, E., C. Curtice, J. Harrison, S.M. Van Parijs, and P.N. Halpin. 2015. 3. Biologically Important Areas for cetaceans within U.S. Waters – Gulf of Mexico Region. Aquatic Mammals 41(1):30–38. doi: 10.1578/am.41.1.2015.30

Leatherwood, S. 1977. Some preliminary impressions on the numbers and social behavior of free-swimming bottlenosed dolphin calves (*Tursiops truncatus*) in the northern Gulf of Mexico. In: Ridgway, S.H. and K. Bernirschke (Eds.), Breeding Dolphins; Present Status, Suggestions for the Future. Final report to the US Marine Mammal Commission, Contract MM6AC009, MMC-76/07, Washington, DC. pp. 143–167.

Litz, J.A., M.A. Baran, S.R. Bowen-Stevens, R.H. Carmichael, K.M. Colegrove, L.P. Garrison, S.E. Fire, E.M. Fougeres, R. Hardy, S. Holmes, W. Jones, B.E. Mase-Guthrie, D.K. Odell, P.E. Rosel, J.T. Saliki, D.K. Shannon, S.F. Shippee, S.M. Smith, E.M. Stratton, M.C. Tumlin, H.R. Whitehead, G.A. Worthy, and T.K. Rowles. 2014. Review of historical unusual mortality events (UMEs) in the Gulf of Mexico (1990–2009): providing context for the multi-year northern Gulf of Mexico cetacean UME declared in 2010. Diseases of Aquatic Organisms 112(2):161–175. doi: 10.3354/dao02807

Lynn, S.K. and B. Würsig. 2002. Summer movement patterns of bottlenose dolphins in a Texas bay. Gulf of Mexico Science 20(1):25–37. doi: 10.18785/goms.2001.03

Mackey, A.D. 2010. Site fidelity and association patterns of bottlenose dolphins (*Tursiops truncatus*) in the Mississippi Sound. University of Southern Mississippi. Thesis. 133 p.

Mann, J., R.C. Connor, L.M. Barre, and M.R. Heithaus. 2000. Female reproductive success in bottlenose dolphins (*Tursiops* sp.): life history, habitat, provisioning, and group-size effects. Behavioral Ecology 11(2):210–219. doi: 10.1093/beheco/11.2.210

Mattson, M.C., K.D. Mullin, G.W. Ingram, and W. Hoggard. 2006. Age structure and growth of the bottlenose dolphin (*Tursiops truncatus*) from strandings in the Mississippi sound region of the north-central Gulf of Mexico from 1986 to 2003. Marine Mammal Science 22(3):654–666. doi: 10.1111/j.1748-7692.2006.00057.x

Maze, K.S. and B. Würsig. 1999. Bottlenosed dolphins of San Luis Pass, Texas: Occurrence patterns, site-fidelity, and habitat use. Aquatic Mammals 25:91–104.

McDonald, T.L., F.E. Hornsby, T.R. Speakman, E.S. Zolman, K.D. Mullin, C. Sinclair, P.E. Rosel, L. Thomas, and L.H. Schwacke. 2017. Survival, density, and abundance of common bottlenose dolphins in Barataria Bay (USA) following the Deepwater Horizon oil spill. Endangered Species Research 33:193–209. doi: 10.3354/esr00806

McHugh, M.B. 1989. Population numbers and feeding behavior of the Atlantic bottlenose dolphin (*Tursiops truncatus*) near Aransas Pass, Texas. University of Texas at Austin. Thesis. 96 p.

Mead, J.G. and C.W. Potter. 1990. Natural history of bottlenose dolphins along the central Atlantic coast of the United States. In: Leatherwood, S. and R.R. Reeves (Eds.), The Bottlenose Dolphin. Academic Press, San Diego, CA. pp. 165–195.

Miller, C.E. 2003. Abundance trends and environmental habitat usage patterns of bottlenose dolphins (*Tursiops truncatus*) in lower Barataria and Caminada bays, Louisiana. Louisiana State University and Agricultural & Mechanical College. Dissertation. 125 p.

Miller, L.J., A.D. Mackey, T. Hoffland, M. Solangi, and S.A. Kuczaj. 2010. Potential effects of a major hurricane on Atlantic bottlenose dolphin (*Tursiops truncatus*) reproduction in the Mississippi Sound. Marine Mammal Science 26(3):707–715. doi: 10.1111/j.1748-7692.2010.00371.x

Miller, L.J., A.D. Mackey, M. Solangi, and S.A. Kuczaj. 2013. Population abundance and habitat utilization of bottlenose dolphins in the Mississippi Sound. Aquatic Conservation: Marine and Freshwater Ecosystems 23(1):145–151. doi: 10.1002/aqc.2278

Mullin, K.D., T. McDonald, R.S. Wells, B.C. Balmer, T. Speakman, C. Sinclair, E.S. Zolman, F. Hornsby, S.M. McBride, K.A. Wilkinson, and L.H. Schwacke. 2017. Density, abundance, survival, and ranging patterns of common bottlenose dolphins (*Tursiops truncatus*) in Mississippi Sound following the Deepwater Horizon oil spill. PLoS ONE 12(10):e0186265. doi: 10.1371/journal.pone.0186265

Orlando, S.P.J., L.P. Rozas, G.H. Ward, and C.J. Klein. 1993. Salinity characteristics of Gulf of Mexico estuaries. NOAA's National Estuarine Inventory Series. National Oceanic and Atmospheric Administration, Office of Ocean Resources Conservation and Assessment: Silver Spring, MD. 209 p.

Perrin, W.F. and S.B. Reilly. 1984. Reproductive parameters of dolphins and small whales of the family Delphinidae. In: Perrin, W.F., R.L. Brownell, Jr., and D.P. DeMaster (Eds.), Reproduction in Whales, Dolphins, and Porpoises. pp. 97–134.

Phillips, N.M. and P.E. Rosel. 2014. A method for prioritizing research on common bottlenose dolphin stocks through evaluating threats and data availability: Development and application to bay, sound and estuary stocks in Texas. National Oceanic and Atmospheric Administration, National Marine Fisheries Service, Southeast Fisheries Science Center: Lafayette, LA. NOAA Technical Memorandum NMFS-SEFSC-665. 146 p. doi: 10.7289/V5F769H8

Ronje, E., S. Piwetz, H. Whitehead, and K.D. Mullin. 2020. Intraspecific aggression towards common bottlenose dolphin calves, Northern Gulf of Mexico. Gulf and Caribbean Research 31:SC6–SC12. doi: 10.18785/gcr.3101.07

Ronje, E., H. Whitehead, K. Barry, S. Piwetz, J. Struve, V. Lecours, L. Garrison, R.S. Wells, and K.D. Mullin. 2020. Abundance and occurrence of common bottlenose dolphins (*Tursiops truncatus*) in three

estuaries of the northwestern Gulf of Mexico. Gulf and Caribbean Research 31:18–34. doi: 10.18785/gcr.3101.09

Rosel, P.E., L.A. Wilcox, C. Sinclair, T.R. Speakman, M.C. Tumlin, J.A. Litz, and E.S. Zolman. 2017. Genetic assignment to stock of stranded common bottlenose dolphins in southeastern Louisiana after the Deepwater Horizon oil spill. Endangered Species Research 33:221–234. doi: 10.3354/esr00780

Rossman, S., P.H. Ostrom, M. Stolen, N.B. Barros, H. Gandhi, C.A. Stricker, and R.S. Wells. 2015. Individual specialization in the foraging habits of female bottlenose dolphins living in a trophically diverse and habitat rich estuary. Oecologia 178(2):415–425. doi: 10.1007/s00442-015-3241-6

Scott, M.D. and S.J. Chivers. 1990. Distribution and herd structure of bottlenose dolphins in the eastern tropical Pacific Ocean. In: Leatherwood, S. and R.R. Reeves (Eds.), The Bottlenose Dolphin. Academic Press, San Diego, CA. pp. 387–402.

Sellas, A.B., R.S. Wells, and P.E. Rosel. 2005. Mitochondrial and nuclear DNA analyses reveal fine scale geographic structure in bottlenose dolphins (*Tursiops truncatus*) in the Gulf of Mexico. Conservation Genetics 6(5):715–728. doi: 10.1007/s10592-005-9031-7

Sergeant, D.E., D.K. Caldwell, and M.C. Caldwell. 1973. Age, growth, and maturity of bottlenosed dolphin (*Tursiops-truncatus*) from northeast Florida. Journal of the Fisheries Research Board of Canada 30(7):1009–1011. doi: 10.1139/f73-165

Shane, S.H. 1990. Behavior and ecology of the bottlenose dolphin at Sanibel Island, Florida. In: Leatherwood, S. and R.R. Reeves (Eds.), The Bottlenose Dolphin. Academic Press, San Diego, CA. pp. 245–265. doi: 10.1016/B978-0-12-440280-5.50016-0

Shane, S.H. 2004. Residence patterns, group characteristics, and association patterns of bottlenose dolphins near Sanibel Island, Florida. Gulf of Mexico Science 22(1):1–12. doi: 10.18785/goms.2201.01

Shane, S.H., R.S. Wells, and B. Würsig. 1986. Ecology, behavior and social organization of the bottlenose dolphin: A review. Marine Mammal Science 2:34–63. doi: 10.1111/j.1748-7692.1986.tb00026.x

Takeshita, R., B.C. Balmer, F. Messina, E.S. Zolman, L. Thomas, R.S. Wells, C.R. Smith, T.K. Rowles, and L.H. Schwacke. 2021. High site-fidelity in common bottlenose dolphins despite low salinity exposure and associated indicators of compromised health. PLoS ONE 16(9):e0258031. doi: 10.1371/journal.pone.0258031

Taylor, B.L., S.J. Chivers, J. Larese, and W.F. Perrin. 2007. Generation length and percent mature estimates for IUCN assessments of cetaceans. National Oceanic and Atmospheric Administration, National Marine Fisheries Service, Southwest Fisheries Science Center: La Jolla, CA. Administrative Report LJ-07-01. 24 p.

Toms, C. 2019. Filling the gaps: Common bottlenose dolphin (*Tursiops truncatus*) population dynamics, structure, and connectivity within Florida panhandle bays, sounds, and estuaries. University of Central Florida. Dissertation. 373 p.

Torres, L.G. and A.J. Read. 2009. Where to catch a fish? The influence of foraging tactics on the ecology of bottlenose dolphins (*Tursiops truncatus*) in Florida Bay, Florida. Marine Mammal Science 25(4):797–815. doi: 10.1111/j.1748-7692.2009.00297.x

Tyson, R.B., S.M. Nowacek, and D.P. Nowacek. 2011. Community structure and abundance of bottlenose dolphins *Tursiops truncatus* in coastal waters of the northeast Gulf of Mexico. Marine Ecology Progress Series 438:253–265. doi: 10.3354/meps09292

Urian, K.W., S. Hofmann, R.S. Wells, and A.J. Read. 2009. Fine-scale population structure of bottlenose dolphins (*Tursiops truncatus*) in Tampa Bay, Florida. Marine Mammal Science 25(3):619–638. doi: 10.1111/j.1748-7692.2009.00284.x

Vollmer, N.L. and P.E. Rosel. 2013. A review of common bottlenose dolphins (*Tursiops truncatus truncatus*) in the Northern Gulf of Mexico: Population biology, potential threats, and management. Southeastern Naturalist 12(m6):1–43. doi: 10.1656/058.012.m601

Ward, G.H. 1980. Hydrography and circulation processes of Gulf estuaries. In: Hamilton, P. and K.B. Macdonald (Eds.), Estuarine and Wetland Processes, Marine Science Book Series. Vol. 11. Springer Science+Business Media, New York, NY. p. 183—215. doi: 10.1007/978—1—4757—5177—2

Weller, D.W. 1998. Global and regional variation in the biology and behavior of bottlenose dolphins. Texas A&M University. Dissertation. 142 p.

Wells, R.S. 1986a. Population structure of bottlenose dolphins: Behavioral studies along the central west coast of Florida. Contract Report to National Marine Fisheries Service, Southeast Fisheries Center. Contract No. 45-WCNF-5-003BB. 58 p.

Wells, R.S. 1986b. Structural aspects of dolphin societies. University of California, Santa Cruz. Dissertation. 234 p.

Wells, R.S. 1991. The role of long-term study in understanding the social structure of a bottlenose dolphin community. In: Pryor, K. and K. Norris (Eds.), Dolphin Societies: Discoveries and Puzzles. University of California Press, Berkeley, CA. pp. 199–226.

Wells, R.S. 2000. Reproduction in wild bottlenose dolphins: Overview of patterns observed during a long-term study. In: Duffield, D. and T. Robeck (Eds.), Bottlenose Dolphin Reproduction Workshop. AZA Marine Mammal Taxon Advisory Group, Silver Spring, MD. pp. 57–74.

Wells, R.S. 2003. Dolphin social complexity: Lessons from long-term study and life history. In: de Waal, F.B.M. and P.L. Tyack (Eds.), Animal Social Complexity: Intelligence, Culture, and Individualized Societies Harvard University Press, Cambridge, MA. pp. 32–56.

Wells, R.S. 2014. Social structure and life history of bottlenose dolphins near Sarasota Bay, Florida: Insights from four decades and five generations. Primates and cetaceans. pp. 149–172. doi: 10.1007/978-4-431-54523-1_8

Wells, R.S., M.K. Bassos, K.W. Urian, W.J. Carr, and M.D. Scott. 1996. Low-level monitoring of bottlenose dolphins, *Tursiops truncatus*, in Charlotte Harbor, Florida: 1990-1994. National Oceanic and Atmospheric Administration, National Marine Fisheries Service, Southeast Fisheries Science Center: Miami, FL. NOAA Technical Memorandum NMFS-SEFSC-384. 86 p.

Wells, R.S., M.K. Bassos, K.W. Urian, S.H. Shane, E.C.G. Owen, C.F. Weiss, W.J. Carr, and M.D. Scott. 1997. Low-level monitoring of bottlenose dolphins, *Tursiops truncatus*, in Pine Island Sound, Florida: 1996. National Oceanic and Atmospheric Administration, National Marine Fisheries Service, Southeast Fisheries Science Center: Miami, FL. Southeast Fisheries Center Contribution No. 40-WCNF601958. 86 p.

Wells, R.S., A. Natoli, and G. Braulik. 2019. *Tursiops truncatus* (errata version published in 2019). *The IUCN Red List of Threatened Species* 2019: e.T22563A156932432. https://dx.doi.org/10.2305/IUCN.UK.2019-1.RLTS.T22563A156932432.en

Wells, R.S., L.H. Schwacke, T.K. Rowles, B.C. Balmer, E. Zolman, T. Speakman, F.I. Townsend, M.C. Tumlin, A. Barleycorn, and K.A. Wilkinson. 2017. Ranging patterns of common bottlenose dolphins *Tursiops truncatus* in Barataria Bay, Louisiana, following the Deepwater Horizon oil spill. Endangered Species Research 33:159–180. doi: 10.3354/esr00732

Wells, R.S. and M.D. Scott. 1999. Bottlenose dolphin *Tursiops truncatus* (Montagu, 1821). In: Ridgway, S.H. and R. Harrison (Eds.), Handbook of marine mammals. Volume 6: The second book of dolphins and the porpoises. Academic Press, San Diego, CA. pp. 137–182.

Wells, R.S. and M.D. Scott. 2018. Bottlenose dolphin, *Tursiops truncatus*, common bottlenose dolphin. In: Würsig, B., J.G.M. Thewissen, and K.M. Kovacs (Eds.), Encyclopedia of Marine Mammals

(Third Edition). Academic Press, London, UK. pp. 118–125. doi: 10.1016/B978-0-12-804327-1.00072-8

Wells, R.S., M.D. Scott, and A.B. Irvine. 1987. The social structure of free-ranging bottlenose dolphins. In: Genoways, H.H. (Ed.), Current Mammalogy. Plenum Press, New York, NY. pp. 247–305.

Wells, R.S., K.W. Urian, A.J. Read, M.K. Bassos, W.J. Carr, and M.D. Scott. 1996. Low-level monitoring of bottlenose dolphins, *Tursiops truncatus*, in Tampa Bay, Florida: 1988-1993. National Oceanic and Atmospheric Administration, National Marine Fisheries Service, Southeast Fisheries Science Center: Miami, FL. NOAA Technical Memorandum NMFS-SEFSC-385. 36 p.

Wilson, R.M., R.B. Tyson, J.A. Nelson, B.C. Balmer, J.P. Chanton, and D.P. Nowacek. 2017. Niche differentiation and prey selectivity among common bottlenose dolphins (*Tursiops truncatus*) sighted in St. George Sound, Gulf of Mexico. Frontiers in Marine Science 4:235. doi: 10.3389/fmars.2017.00235

Common bottlenose dolphin − *Tursiops truncatus* GoMx

BSE/Sarasota Bay, Little Sarasota Bay Stock

Overall Vulnerability Rank = Very High

Biological Sensitivity = Very High

Climate Exposure = Very High

Data Quality = 100% of scores≥ 2

	Tursiops truncatus	Expert Scores	Data Quality	Expert Scores Plots (Portion by Category)
Sensitivity attributes	Prey/Diet Specificity	1.6	3.0	
	Habitat Specificity	3.9	3.0	
	Site Fidelity	4.0	3.0	
	Lifetime Reproductive Potential	1.5	3.0	
	Generation Length	3.0	3.0	
	Reproductive Plasticity	1.3	3.0	
	Migration	4.0	3.0	
	Home Range	3.5	3.0	
	Stock Abundance	3.0	3.0	
	Stock Abundance Trend	2.0	2.7	
	Cumulative Stressors	3.7	3.0	
	Sensitivity Score	Very High		
Exposure variables	Sea Surface Temperature (Change in mean)	4.0	3.0	
	Sea Surface Temperature (Change in variability)	1.0	3.0	
	Air Temperature (Change in mean)	4.0	3.0	
	Air Temperature (Change in variability)	1.0	3.0	
	Precipitation (Change in mean)	1.0	3.0	
	Precipitation (Change in variability)	1.0	3.0	
	Sea Surface Salinity (Change in mean)	1.4	3.0	
	Sea Surface Salinity (Change in variability)	2.0	3.0	
	Ocean pH (Change in mean)	4.0	3.0	
	Ocean pH (Change in variability)	1.0	3.0	
	Sea ice coverage (Change in mean)	1.0	3.0	
	Sea ice coverage (Change in variability)	1.0	3.0	
	Dissolved oxygen (Change in mean)	4.0	3.0	
	Dissolved oxygen (Change in variability)	1.0	3.0	
	Circulation	3.9	2.3	
	Sea level rise	4.0	3.0	
	Exposure Score	Very High		
Overall Vulnerability Rank		Very High		

Low
Moderate
High
Very High

Common bottlenose dolphin (GoMx BSE/Sarasota Bay, Little Sarasota Bay Stock)

Tursiops truncatus

CVA Results Summary

Overall Climate Vulnerability Rank: Very High (100% certainty from bootstrap analysis).

Climate Exposure: Very High. Six exposure factors scored greater than 3.5: Air Temperature (Standard anomaly) (4.00), Dissolved oxygen (Standard anomaly) (4.00), Ocean pH (Standard anomaly) (4.00), Sea level rise (4.00), Sea Surface Temperature (Standard anomaly) (4.00), and Circulation (3.93).

Biological Sensitivity: Very High. Four sensitivity attributes scored greater than 3.5: Migration (4.00), Site Fidelity (4.00), Habitat Specificity (3.87), and Cumulative Stressors (3.67).

Distributional Response: Very High

Abundance Response: Very High

Phenology Response: Very High

Data Quality: 100% of the data quality scores were 2 or greater, 100 % of sensitivity attribute data quality scores were 2 or higher.

Background Information

Stock Description

This stock includes common bottlenose dolphins in Sarasota Bay and Little Sarasota Bay along the Gulf of Mexico coast of Florida (Hayes et al. 2022).

Prey/Diet Specificity

Generally, common bottlenose dolphins consume a large variety of fish and/or squid (Wells and Scott 2018). Common bottlenose dolphins seem to show a consistent preference for noise-producing fishes (e.g., sciaenids, toadfish; Berens McCabe et al. 2010; Bowen-Stevens et al. 2021) and individuals of this stock have been found to have a flexible diet consisting primarily of fish (Cloyed et al. 2021a). In Sarasota Bay, FL, Rossman et al. (2015a, 2015b) found some individual common bottlenose dolphins to show diet specialization despite the broader study group showing a generalist diet. Specialization can be influenced by locally abundant prey species (Shane et al. 1986; Vollmer and Rosel 2013) or can result from resource partitioning amongst sympatric populations (e.g., Torres and Read 2009; Wilson et al. 2017).

Habitat Specificity

Common bottlenose dolphins are found in a variety of marine and estuarine habitats (Wells and Scott 2018).

Site Fidelity

Strong site fidelity was first defined for common bottlenose dolphins based on research initiated on this stock in 1970 (Irvine and Wells 1972; Irvine et al. 1981; Wells 2014). The dolphins of this stock are year-round, multi-decadal, multi-generational residents of the inshore waters from southern Tampa Bay to Venice Inlet (Wells 2014).

Other Gulf of Mexico bay, sound, and estuary (BSE) stocks also show site fidelity (LaBrecque et al. 2015; Hayes et al. 2022), such as in Matagorda Bay and Espiritu Santo Bay in Texas (Lynn and Würsig 2002), San Luis Pass in Texas (Maze and Würsig 1999; Irwin and Würsig 2004), Barataria Bay in Louisiana (Miller 2003; Wells et al. 2017; Takeshita et al. 2021), Mississippi Sound (Hubard et al. 2004; Mackey 2010), St. Vincent Sound and Apalachicola Bay (Tyson et al. 2011), Tampa Bay (Wells et al. 1996b; Urian et al. 2009), and Charlotte Harbor and Pine Island Sound (Wells et al. 1996a; Wells et al. 1997; Bassos-Hull et al. 2013).

Lifetime Reproductive Potential

Taylor et al. (2007) reported the interbirth interval of common bottlenose dolphins as 3.80 years, though other studies have shown it to be closer to three years (Leatherwood 1977; Wells and Scott 1999; Connor et al. 2000; Wells 2003).

Generally, females reach sexual maturity before males, with females reaching sexual maturity at 5–13 years (Sergeant et al. 1973; Perrin and Reilly 1984; Wells et al. 1987; Mead and Potter 1990; Mann et al. 2000; Wells 2000; Wells and Scott 2018).

Females reproduce through most of their adult lives (Leatherwood 1977; Hohn et al. 1989; Wells and Scott 1999; Wells 2000; Wells 2003), but may produce fewer calves after they reach 30 years of age (Wells 2000). Sarasota Bay common bottlenose dolphins can give birth when they are as young as 6 years of age, and can continue until they are 48 years old (Wells and Scott 2018). Reproductive lifespan would therefore be up to 42 years based on long-term observation studies (Wells 2014; Wells and Scott 2018).

Longitudinal studies from Sarasota Bay, Florida have found that female common bottlenose dolphins can live up to 67 years (Wells and Scott 2018). Studies based on growth layer groups in teeth suggest female common bottlenose dolphins can live to 41 years along the Texas coast (Fernandez and Hohn 1998) and to 30 years along the Mississippi coast (Mattson et al. 2006).

Generation Length

Taylor et al. (2007) reported common bottlenose dolphin generation length of 20.6-21.1 years.

Reproductive Plasticity

Common bottlenose dolphin calving is generally diffusely seasonal, with one or more peaks, although births have been reported from all seasons (Urian et al. 1996; Vollmer and Rosel 2013; Wells and Scott 2018). Calf sightings peak in the spring and summer months in Florida (Wells et al. 1987), Mississippi/Louisiana (Miller et al. 2010; Miller et al. 2013), and Texas (McHugh 1989; Henderson et al. 2007).

Migration

The bay, sound, and estuary (BSE) stocks in the Gulf of Mexico are thought to primarily comprise stable resident communities. However, transient or seasonal movements of some individuals into and out of a BSE stock area are recorded in some areas (LaBrecque et al. 2015; Hayes et al. 2022). Molecular genetic data support that some BSE stocks are genetically distinct from one another, and furthermore are differentiated from adjacent coastal populations (e.g., Sellas et al. 2005; Rosel et al. 2017). For example, Toms (2019) found fine-scale genetic population structure among five inshore systems in the Florida Panhandle and variable migration rates among populations.

Year-round residency has been reported for common bottlenose dolphins (Hayes et al. 2022), such as in Florida (Balmer et al. 2008; Bassos-Hull et al. 2013; Irvine and Wells 1972; Irvine et al. 1981; Scott et al. 1990; Tyson et al. 2011; Urian et al. 2009; Wells 1986; Wells 1991; Wells 2003; Wells et al. 1987; Wells et al. 1996a; Wells et al. 1996b; Wells et al. 1997), Texas (Bräger 1993; Bräger et al. 1994; Fertl 1994; Irwin and Würsig 2004; Lynn and Würsig 2002; Maze and Würsig 1999; Shane 1990; Shane 2004; Weller 1998), and Louisiana/Mississippi (Hubard et al. 2004; Wells et al. 2017; Takeshita et al. 2021).

Home Range

The home ranges of individuals within the Gulf of Mexico BSE stocks are considered to generally include the areas within the stock boundaries and brief excursions beyond the stock boundaries (Irvine et al. 1981; Wells 1986; Wells 1991; Wells et al. 1996a; Wells et al. 1996b; Balmer et al. 2008; Wells 2014), although some individuals show preference for small areas within the stock boundaries (Lynn and Würsig 2002; Cloyed et al. 2021b).

Stock Abundance

Tyson and Wells (2016) estimated this stock's abundance at 158 individuals (CV = 0.27).

Stock Abundance Trend

The bottlenose dolphins of Sarasota Bay have increased in abundance since an initial estimate of 102 dolphins (95% CI = 90–117) in 1976 (Irvine et al. 1981), and a subsequent estimate of 98 (95% CI = 89–108) in 1983 (Wells 1986), to the 2015 estimate of 158 dolphins (Tyson and Wells 2016).

Cumulative Stressors

Common bottlenose dolphins face a wide range of individual and cumulative natural and anthropogenic threats (see reviews by Vollmer and Rosel 2013; Wells and Scott 2018; and Wells et al. 2019). These include such threats as shark predation (Wilkinson et al. 2017), harmful algal blooms (Fire et al. 2007, 2008a, 2008b, 2021; McHugh et al. 2011; Twiner et al. 2011), recreational and commercial fishing gear (Powell and Wells 2011), vessel traffic, pollution, habitat alteration, and provisioning (Christiansen et al. 2016). Common bottlenose dolphins in the Sarasota Bay, Little Sarasota Bay Stock interact with the stone crab trap/pot fisheries (86 FR 3028, January 14, 2021).

The nearshore and estuarine habitats along the Gulf of Mexico coast include waters adjacent to areas of high human population, some of which are highly industrialized, and areas of heavy agricultural impact. Studies of common bottlenose dolphin tissues from Sarasota Bay have shown persistent organic

pollutants (POPs) in blubber and blood samples (Yordy et al. 2010a, 2010b, 2010c; Kucklick et al. 2011; Balmer et al. 2018). Similarly, mercury has been detected in common bottlenose dolphin tissues in Sarasota Bay and Florida Gulf Coast waters (Bryan et al. 2007; Woshner et al. 2008; Miller et al. 2011; Correa et al. 2013; McCormack et al. 2020a, 2020b, 2022). Other compounds such as perfluoroalkyl phosphinic acids (PFPIAs), perfluoroalkyl carboxylates (PFCAs), perfluoroalkanesulfonates (PFSAs), and phthalates have been found in individuals of this stock (Houde et al. 2005, 2006; De Silva et al. 2016; Hart et al. 2018; Dziobak et al. 2021).

An Unusual Mortality Event (UME) was declared in 1990 for the Gulf of Mexico from Florida to Texas, with more than 300 common bottlenose dolphins stranding due to an undetermined cause, although morbillivirus and cold temperatures may have contributed (Litz et al. 2014). This stock was also within the range of the 2005–2006 UME in central west Florida caused by brevetoxin and the 1991 Sarasota UME suspected to have been caused by biotoxin (Litz et al. 2014).

Further Reading

Hayes, S.A., E. Josephson, K. Maze-Foley, P.E. Rosel, and J. Wallace. 2022. U.S. Atlantic and Gulf of Mexico marine mammal stock assessments 2021. National Oceanic and Atmospheric Administration, National Marine Fisheries Service, Northeast Fisheries Science Center: Woods Hole, MA. NOAA Technical Memorandum NMFS-NE-288. 380 p. doi: 10.25923/6tt7-kc16

Wells, R.S. and M.D. Scott. 2018. Bottlenose Dolphin, *Tursiops truncatus*, Common Bottlenose Dolphin. In: Würsig, B., J.G.M. Thewissen, and K.M. Kovacs (Eds.), Encyclopedia of Marine Mammals (Third Edition). Academic Press, London, UK. pp. 118–125. doi: 10.1016/B978-0-12-804327-1.00072-8

Wells, R.S., A. Natoli, and G. Braulik. 2019. *Tursiops truncatus* (errata version published in 2019). The IUCN Red List of Threatened Species 2019: e.T22563A156932432. https://dx.doi.org/10.2305/IUCN.UK.2019-1.RLTS.T22563A156932432.en

References

Balmer, B.C., R.S. Wells, S.M. Nowacek, D.P. Nowacek, L.H. Schwacke, W.A. McLellan, F.S. Scharf, T.K. Rowles, L.J. Hansen, T.R. Spradlin, and D.A. Pabst. 2008. Seasonal abundance and distribution patterns of common bottlenose dolphins (*Tursiops truncatus*) near St. Joseph Bay, Florida, USA. Journal of Cetacean Research and Management 10(2):157–167.

Balmer, J.E., G.M. Ylitalo, T.K. Rowles, K.D. Mullin, R.S. Wells, F.I. Townsend, R.W. Pearce, J.L. Bolton, E.S. Zolman, B.C. Balmer, and L.H. Schwacke. 2018. Persistent organic pollutants (POPs) in blood and blubber of common bottlenose dolphins (*Tursiops truncatus*) at three northern Gulf of Mexico sites following the Deepwater Horizon oil spill. Science of the Total Environment 621:130–137. doi: 10.1016/j.scitotenv.2017.11.209

Bassos-Hull, K., R.M. Perrtree, C.C. Shepard, S. Schilling, A.A. Barleycorn, J.B. Allen, B.C. Balmer, W.E. Pine, and R.S. Wells. 2013. Long-term site fidelity and seasonal abundance estimates of common bottlenose dolphins (*Tursiops truncatus*) along the southwest coast of Florida and responses to natural perturbations. Journal of Cetacean Research and Management 13(1):19–30. doi: 10.47536/jcrm.v13i1.551

Berens McCabe, E.J., D.P. Gannon, N.B. Barros, and R.S. Wells. 2010. Prey selection by resident common bottlenose dolphins (*Tursiops truncatus*) in Sarasota Bay, Florida. Marine Biology 157(5):931–942. doi: 10.1007/s00227-009-1371-2

Bowen-Stevens, S.R., D.P. Gannon, R.A. Hazelkorn, G. Lovewell, K.M. Volker, S. Smith, M.C. Tumlin, and J. Litz. 2021. Diet of common bottlenose dolphins, *Tursiops truncatus*, that stranded in and near Barataria Bay, Louisiana, 2010–2012. Southeastern Naturalist 20(1). doi: 10.1656/058.020.0113

Bräger, S. 1993. Diurnal and seasonal behavior patterns of bottlenose dolphins (*Tursiops truncatus*). Marine Mammal Science 9:434–438. doi: 10.1111/j.1748-7692.1993.tb00477.x

Bräger, S., B. Würsig, A. Acevedo, and T. Henningsen. 1994. Association patterns of bottle-nosed dolphins (*Tursiops-truncatus*) in Galveston Bay, Texas. Journal of Mammalogy 75(2):431–437. doi: 10.2307/1382564

Bryan, C.E., S.J. Christopher, B.C. Balmer, and R.S. Wells. 2007. Establishing baseline levels of trace elements in blood and skin of bottlenose dolphins in Sarasota Bay, Florida: implications for non-invasive monitoring. Science of the Total Environment 388(1-3):325–342. doi: 10.1016/j.scitotenv.2007.07.046

Christiansen, F., K.A. McHugh, L. Bejder, E.M. Siegal, D. Lusseau, E.B. McCabe, G. Lovewell, and R.S. Wells. 2016. Food provisioning increases the risk of injury in a long-lived marine top predator. Royal Society Open Science 3(12):160560. doi: 10.1098/rsos.160560

Cloyed, C.S., B.C. Balmer, L.H. Schwacke, R.S. Wells, E.J. Berens McCabe, A.A. Barleycorn, J.B. Allen, T.K. Rowles, C.R. Smith, R. Takeshita, F.I. Townsend, M.C. Tumlin, E.S. Zolman, and R.H. Carmichael. 2021a. Interaction between dietary and habitat niche breadth influences cetacean vulnerability to environmental disturbance. Ecosphere 12(9):e03759. doi: 10.1002/ecs2.3759

Cloyed, C.S., R.M. Wilson, B.C. Balmer, A.A. Hohn, L.H. Schwacke, E.S. Zolman, M.C. Tumlin, R.S. Wells, A.A. Barleycorn, J.B. Allen, and R.H. Carmichael. 2021b. Specialization of a mobile, apex predator affects trophic coupling among adjacent habitats. Scientific Reports 11(1):19611. doi: 10.1038/s41598-021-99017-1

Connor, R.C., R.S. Wells, J. Mann, and A.J. Read. 2000. The bottlenose dolphin: Social relationships in a fission-fusion society. In: Mann, J., R. Connor, P. Tyack, and H. Whitehead (Eds.), Cetacean Societies: Field Studies of Dolphins and Whales. University of Chicago Press, Chicago, IL. pp. 91–126.

Correa, L., J.M. Castellini, R.S. Wells, and T. O'Hara. 2013. Distribution of mercury and selenium in blood compartments of bottlenose dolphins (*Tursiops truncatus*) from Sarasota Bay, Florida. Environmental Toxicology and Chemistry 32(11):2441–2448. doi: 10.1002/etc.2327

De Silva, A.O., C. Spencer, K.C. Ho, M. Al Tarhuni, C. Go, M. Houde, S.R. de Solla, R.A. Lavoie, L.E. King, D.C. Muir, P.A. Fair, R.S. Wells, and G.D. Bossart. 2016. Perfluoroalkylphosphinic acids in northern pike (*Esox lucius*), double-crested cormorants (*Phalacrocorax auritus*), and bottlenose dolphins (*Tursiops truncatus*) in relation to other perfluoroalkyl acids. Environmental Science & Technology 50(20):10903–10913. doi: 10.1021/acs.est.6b03515

Dziobak, M.K., R.S. Wells, E.C. Pisarski, E.F. Wirth, and L.B. Hart. 2021. Demographic Assessment of Mono(2-ethylhexyl) Phthalate (MEHP) and Monoethyl Phthalate (MEP) Concentrations in Common Bottlenose Dolphins (*Tursiops truncatus*) From Sarasota Bay, FL, USA. Geohealth 5(5):e2020GH000348. doi: 10.1029/2020GH000348

Fernandez, S. and A.A. Hohn. 1998. Age, growth, and calving season of bottlenose dolphins, *Tursiops truncatus*, off coastal Texas. Fishery Bulletin 96(2):357–365.

Fertl, D. 1994. Occurrence patterns and behavior of bottlenose dolphins (*Tursiops truncatus*) in the Galveston ship channel, Texas. Texas Journal of Science 46(4):299–318.

Fire, S.E., D. Fauquier, L.J. Flewelling, M. Henry, J. Naar, R. Pierce, and R.S. Wells. 2007. Brevetoxin exposure in bottlenose dolphins (*Tursiops truncatus*) associated with *Karenia brevis* blooms in Sarasota Bay, Florida. Marine Biology 152(4):827–834. doi: 10.1007/s00227-007-0733-x

Fire, S.E., L.J. Flewelling, J. Naar, M.J. Twiner, M.S. Henry, R.H. Pierce, D.P. Gannon, Z.H. Wang, L. Davidson, and R.S. Wells. 2008a. Prevalence of brevetoxins in prey fish of bottlenose dolphins in Sarasota Bay, Florida. Marine Ecology Progress Series 368:283–294. doi: 10.3354/meps07643

Fire, S.E., L.J. Flewelling, Z.H. Wang, J. Naar, M.S. Henry, R.H. Pierce, and R.S. Wells. 2008b. Florida red tide and brevetoxins: Association and exposure in live resident bottlenose dolphins (*Tursiops truncatus*) in the eastern Gulf of Mexico, USA. Marine Mammal Science 24(4):831–844. doi: 10.1111/j.1748-7692.2008.00221.x

Fire, S.E., G.A. Miller, E.R. Sabater, and R.S. Wells. 2021. Utility of red tide (*Karenia brevis*) monitoring data as a predictive tool to estimate brevetoxin accumulation in live, free-ranging marine mammals. Frontiers in Marine Science 8:611310. doi: 10.3389/fmars.2021.611310

Hart, L.B., B. Beckingham, R.S. Wells, M. Alten Flagg, K. Wischusen, A. Moors, J. Kucklick, E. Pisarski, and E. Wirth. 2018. Urinary phthalate metabolites in common bottlenose dolphins (*Tursiops truncatus*) from Sarasota Bay, FL, USA. Geohealth 2(10):313–326. doi: 10.1029/2018GH000146

Hayes, S.A., E. Josephson, K. Maze-Foley, P.E. Rosel, and J. Wallace. 2022. U.S. Atlantic and Gulf of Mexico marine mammal stock assessments 2021. National Oceanic and Atmospheric Administration, National Marine Fisheries Service, Northeast Fisheries Science Center: Woods Hole, MA. NOAA Technical Memorandum NMFS-NE-288. 380 p. doi: 10.25923/6tt7-kc16

Henderson, E.E. and B. Würsig. 2007. Behavior patterns of bottlenose dolphins in San Luis Pass, Texas. Gulf of Mexico Science 25(2):153–161. doi: 10.18785/goms.2502.06

Hohn, A.A., M.D. Scott, R.S. Wells, J.C. Sweeney, and A.B. Irvine. 1989. Growth layers in teeth from known-age, free-ranging bottlenose dolphins. Marine Mammal Science 5(4):315–342. doi: 10.1111/j.1748-7692.1989.tb00346.x

Houde, M., T.A. Bujas, J. Small, R.S. Wells, P.A. Fair, G.D. Bossart, K.R. Solomon, and D.C. Muir. 2006. Biomagnification of perfluoroalkyl compounds in the bottlenose dolphin (*Tursiops truncatus*) food web. Environmental Science & Technology 40(13):4138–4144. doi: 10.1021/es060233b

Houde, M., R.S. Wells, P.A. Fair, G.D. Bossart, A.A. Hohn, T.K. Rowles, J.C. Sweeney, K.R. Solomon, and D.C.G. Muir. 2005. Polyfluoroalkyl compounds in free-ranging bottlenose dolphins (*Tursiops truncatus*) from the Gulf of Mexico and the Atlantic Ocean. Environmental Science & Technology 39(17):6591–6598. doi: 10.1021/es0506556

Hubard, C.W., K. Maze-Foley, K.D. Mullin, and W.W. Schroeder. 2004. Seasonal abundance and site fidelity of bottlenose dolphins (*Tursiops truncatus*) in Mississippi Sound. Aquatic Mammals 30(2):299–310. doi: 10.1578/am.30.2.2004.299

Irvine, A.B., M.D. Scott, R.S. Wells, and J.H. Kaufmann. 1981. Movements and activities of the Atlantic bottlenose dolphin, *Tursiops truncatus*, near Sarasota, Florida. Fishery Bulletin 79(4):671–688.

Irvine, B. and R.S. Wells. 1972. Results of attempts to tag Atlantic bottlenosed dolphins (*Tursiops truncatus*). Cetology 13:1–5.

Irwin, L.-J. and B. Würsig. 2004. A small resident community of bottlenose dolphins, *Tursiops truncatus*, in Texas: Monitoring recommendations. Gulf of Mexico Science 22(1):13–21. doi: 10.18785/goms.2201.02

Kucklick, J., L. Schwacke, R. Wells, A. Hohn, A. Guichard, J. Yordy, L. Hansen, E. Zolman, R. Wilson, J. Litz, D. Nowacek, T. Rowles, R. Pugh, B. Balmer, C. Sinclair, and P. Rosel. 2011. Bottlenose dolphins as indicators of persistent organic pollutants in the western North Atlantic Ocean and Northern Gulf of Mexico. Environmental Science & Technology 45(10):4270–4277. doi: 10.1021/es1042244

LaBrecque, E., C. Curtice, J. Harrison, S.M. Van Parijs, and P.N. Halpin. 2015. 3. Biologically Important Areas for cetaceans within U.S. Waters – Gulf of Mexico Region. Aquatic Mammals 41(1):30–38. doi: 10.1578/am.41.1.2015.30

Leatherwood, S. 1977. Some preliminary impressions on the numbers and social behavior of free-swimming bottlenosed dolphin calves (*Tursiops truncatus*) in the northern Gulf of Mexico. In: Ridgway, S.H. and K. Bernirschke (Eds.), Breeding Dolphins; Present Status, Suggestions for the Future. Final report to the US Marine Mammal Commission, Contract MM6AC009, MMC-76/07, Washington, DC. pp. 143–167.

Litz, J.A., M.A. Baran, S.R. Bowen-Stevens, R.H. Carmichael, K.M. Colegrove, L.P. Garrison, S.E. Fire, E.M. Fougeres, R. Hardy, S. Holmes, W. Jones, B.E. Mase-Guthrie, D.K. Odell, P.E. Rosel, J.T. Saliki, D.K. Shannon, S.F. Shippee, S.M. Smith, E.M. Stratton, M.C. Tumlin, H.R. Whitehead, G.A. Worthy, and T.K. Rowles. 2014. Review of historical unusual mortality events (UMEs) in the Gulf of Mexico (1990–2009): providing context for the multi-year northern Gulf of Mexico cetacean UME declared in 2010. Diseases of Aquatic Organisms 112(2):161–175. doi: 10.3354/dao02807

Lynn, S.K. and B. Würsig. 2002. Summer movement patterns of bottlenose dolphins in a Texas bay. Gulf of Mexico Science 20(1):25–37. doi: 10.18785/goms.2001.03

Mackey, A.D. 2010. Site fidelity and association patterns of bottlenose dolphins (*Tursiops truncatus*) in the Mississippi Sound. University of Southern Mississippi. Thesis. 133 p.

Mann, J., R.C. Connor, L.M. Barre, and M.R. Heithaus. 2000. Female reproductive success in bottlenose dolphins (*Tursiops* sp.): life history, habitat, provisioning, and group-size effects. Behavioral Ecology 11(2):210–219. doi: 10.1093/beheco/11.2.210

Mattson, M.C., K.D. Mullin, G.W. Ingram, and W. Hoggard. 2006. Age structure and growth of the bottlenose dolphin (*Tursiops truncatus*) from strandings in the Mississippi sound region of the north-central Gulf of Mexico from 1986 to 2003. Marine Mammal Science 22(3):654–666. doi: 10.1111/j.1748-7692.2006.00057.x

Maze, K.S. and B. Würsig. 1999. Bottlenosed dolphins of San Luis Pass, Texas: Occurrence patterns, site-fidelity, and habitat use. Aquatic Mammals 25:91–104.

McCormack, M.A., F. Battaglia, W.E. McFee, and J. Dutton. 2020a. Mercury concentrations in blubber and skin from stranded bottlenose dolphins (*Tursiops truncatus*) along the Florida and Louisiana coasts (Gulf of Mexico, USA) in relation to biological variables. Environmental Research 180:108886. doi: 10.1016/j.envres.2019.108886

McCormack, M.A., B.P. Jackson, and J. Dutton. 2020b. Relationship between mercury and selenium concentrations in tissues from stranded odontocetes in the northern Gulf of Mexico. Science of the Total Environment 749:141350. doi: 10.1016/j.scitotenv.2020.141350

McCormack, M.A., W.H. Nowlin, and J. Dutton. 2022. Effect of trophic position on mercury concentrations in bottlenose dolphins (*Tursiops truncatus*) from the northern Gulf of Mexico. Environmental Research 204(Pt B):112124. doi: 10.1016/j.envres.2021.112124

McHugh, K.A., J.B. Allen, A.A. Barleycorn, and R.S. Wells. 2011. Severe *Karenia brevis* red tides influence juvenile bottlenose dolphin (*Tursiops truncatus*) behavior in Sarasota Bay, Florida. Marine Mammal Science 27(3):622–643. doi: 10.1111/j.1748-7692.2010.00428.x

McHugh, M.B. 1989. Population numbers and feeding behavior of the Atlantic bottlenose dolphin (*Tursiops truncatus*) near Aransas Pass, Texas. University of Texas at Austin. Thesis. 96 p.

Mead, J.G. and C.W. Potter. 1990. Natural history of bottlenose dolphins along the central Atlantic coast of the United States. In: Leatherwood, S. and R.R. Reeves (Eds.), The Bottlenose Dolphin. Academic Press, San Diego, CA. pp. 165–195.

Miller, C.E. 2003. Abundance trends and environmental habitat usage patterns of bottlenose dolphins (*Tursiops truncatus*) in lower Barataria and Caminada bays, Louisiana. Louisiana State University and Agricultural & Mechanical College. Dissertation. 125 p.

Miller, D.L., V. Woshner, E.L. Styer, S. Ferguson, K.K. Knott, M.J. Gray, R.S. Wells, and T.M. O'Hara. 2011. Histologic findings in free-ranging Sarasota Bay bottlenose dolphin (*Tursiops truncatus*) skin: mercury, selenium, and seasonal factors. Journal of Wildlife Diseases 47(4):1012–1018. doi: 10.7589/0090-3558-47.4.1012

Miller, L.J., A.D. Mackey, T. Hoffland, M. Solangi, and S.A. Kuczaj. 2010. Potential effects of a major hurricane on Atlantic bottlenose dolphin (*Tursiops truncatus*) reproduction in the Mississippi Sound. Marine Mammal Science 26(3):707–715. doi: 10.1111/j.1748-7692.2010.00371.x

Miller, L.J., A.D. Mackey, M. Solangi, and S.A. Kuczaj. 2013. Population abundance and habitat utilization of bottlenose dolphins in the Mississippi Sound. Aquatic Conservation: Marine and Freshwater Ecosystems 23(1):145–151. doi: 10.1002/aqc.2278

Perrin, W.F. and S.B. Reilly. 1984. Reproductive parameters of dolphins and small whales of the family Delphinidae. In: Perrin, W.F., R.L. Brownell, Jr., and D.P. DeMaster (Eds.), Reproduction in Whales, Dolphins, and Porpoises. pp. 97–134.

Powell, J.R. and R.S. Wells. 2011. Recreational fishing depredation and associated behaviors involving common bottlenose dolphins (*Tursiops truncatus*) in Sarasota Bay, Florida. Marine Mammal Science 27(1):111–129. doi: 10.1111/j.1748-7692.2010.00401.x

Rosel, P.E., L.A. Wilcox, C. Sinclair, T.R. Speakman, M.C. Tumlin, J.A. Litz, and E.S. Zolman. 2017. Genetic assignment to stock of stranded common bottlenose dolphins in southeastern Louisiana after the Deepwater Horizon oil spill. Endangered Species Research 33:221–234. doi: 10.3354/esr00780

Rossman, S., E. Berens McCabe, N.B. Barros, H. Gandhi, P.H. Ostrom, C.A. Stricker, and R.S. Wells. 2015. Foraging habits in a generalist predator: Sex and age influence habitat selection and resource use among bottlenose dolphins (*Tursiops truncatus*). Marine Mammal Science 31(1):155–168. doi: 10.1111/mms.12143

Rossman, S., P.H. Ostrom, M. Stolen, N.B. Barros, H. Gandhi, C.A. Stricker, and R.S. Wells. 2015. Individual specialization in the foraging habits of female bottlenose dolphins living in a trophically diverse and habitat rich estuary. Oecologia 178(2):415–425. doi: 10.1007/s00442-015-3241-6

Scott, M.D. and S.J. Chivers. 1990. Distribution and herd structure of bottlenose dolphins in the eastern tropical Pacific Ocean. In: Leatherwood, S. and R.R. Reeves (Eds.), The Bottlenose Dolphin. Academic Press, San Diego, CA. pp. 387–402.

Sellas, A.B., R.S. Wells, and P.E. Rosel. 2005. Mitochondrial and nuclear DNA analyses reveal fine scale geographic structure in bottlenose dolphins (*Tursiops truncatus*) in the Gulf of Mexico. Conservation Genetics 6(5):715–728. doi: 10.1007/s10592-005-9031-7

Sergeant, D.E., D.K. Caldwell, and M.C. Caldwell. 1973. Age, growth, and maturity of bottlenosed dolphin (*Tursiops-truncatus*) from northeast Florida. Journal of the Fisheries Research Board of Canada 30(7):1009–1011. doi: 10.1139/f73-165

Shane, S.H. 1990. Behavior and ecology of the bottlenose dolphin at Sanibel Island, Florida. In: Leatherwood, S. and R.R. Reeves (Eds.), The Bottlenose Dolphin. Academic Press, San Diego, CA. pp. 245–265. doi: 10.1016/B978-0-12-440280-5.50016-0

Shane, S.H. 2004. Residence patterns, group characteristics, and association patterns of bottlenose dolphins near Sanibel Island, Florida. Gulf of Mexico Science 22(1):1–12. doi: 10.18785/goms.2201.01

Shane, S.H., R.S. Wells, and B. Würsig. 1986. Ecology, behavior and social organization of the bottlenose dolphin: A review. Marine Mammal Science 2:34–63. doi: 10.1111/j.1748-7692.1986.tb00026.x

Takeshita, R., B.C. Balmer, F. Messina, E.S. Zolman, L. Thomas, R.S. Wells, C.R. Smith, T.K. Rowles, and L.H. Schwacke. 2021. High site-fidelity in common bottlenose dolphins despite low salinity

exposure and associated indicators of compromised health. PLoS ONE 16(9):e0258031. doi: 10.1371/journal.pone.0258031

Taylor, B.L., S.J. Chivers, J. Larese, and W.F. Perrin. 2007. Generation length and percent mature estimates for IUCN assessments of cetaceans. National Oceanic and Atmospheric Administration, National Marine Fisheries Service, Southwest Fisheries Science Center: La Jolla, CA. Administrative Report LJ-07-01. 24 p.

Toms, C. 2019. Filling the gaps: Common bottlenose dolphin (*Tursiops truncatus*) population dynamics, structure, and connectivity within Florida panhandle bays, sounds, and estuaries. University of Central Florida. Dissertation. 373 p.

Torres, L.G. and A.J. Read. 2009. Where to catch a fish? The influence of foraging tactics on the ecology of bottlenose dolphins (*Tursiops truncatus*) in Florida Bay, Florida. Marine Mammal Science 25(4):797–815. doi: 10.1111/j.1748-7692.2009.00297.x

Twiner, M.J., S. Fire, L. Schwacke, L. Davidson, Z. Wang, S. Morton, S. Roth, B. Balmer, T.K. Rowles, and R.S. Wells. 2011. Concurrent exposure of bottlenose dolphins (*Tursiops truncatus*) to multiple algal toxins in Sarasota Bay, Florida, USA. PLoS ONE 6(3):e17394. doi: 10.1371/journal.pone.0017394

Tyson, R.B., S.M. Nowacek, and D.P. Nowacek. 2011. Community structure and abundance of bottlenose dolphins *Tursiops truncatus* in coastal waters of the northeast Gulf of Mexico. Marine Ecology Progress Series 438:253–265. doi: 10.3354/meps09292

Tyson, R.B. and R.S. Wells. 2016. Sarasota Bay/Little Sarasota Bay bottlenose dolphin abundance estimates: 2015. Prepared for National Marine Fisheries Service northern Gulf of Mexico bay, sound and estuary bottlenose dolphin stock Blocks B20 and B35, combined. Southeast Fisheries Science Center Reference Document PRBD-2016-02. 22 p. doi: 10.7289/V5/RD-PRBD-2016-02

Urian, K.W., D.A. Duffield, A.J. Read, R.S. Wells, and E.D. Shell. 1996. Seasonality of reproduction in bottlenose dolphins, *Tursiops truncatus*. Journal of Mammalogy 77(2):394–403. doi: 10.2307/1382814

Urian, K.W., S. Hofmann, R.S. Wells, and A.J. Read. 2009. Fine-scale population structure of bottlenose dolphins (*Tursiops truncatus*) in Tampa Bay, Florida. Marine Mammal Science 25(3):619–638. doi: 10.1111/j.1748-7692.2009.00284.x

Vollmer, N.L. and P.E. Rosel. 2013. A review of common bottlenose dolphins (*Tursiops truncatus truncatus*) in the Northern Gulf of Mexico: Population biology, potential threats, and management. Southeastern Naturalist 12(m6):1–43. doi: 10.1656/058.012.m601

Weller, D.W. 1998. Global and regional variation in the biology and behavior of bottlenose dolphins. Texas A&M University. Dissertation. 142 p.

Wells, R.S. 1986. Structural aspects of dolphin societies. University of California, Santa Cruz. Dissertation. 234 p.

Wells, R.S. 1991. The role of long-term study in understanding the social structure of a bottlenose dolphin community. In: Pryor, K. and K. Norris (Eds.), Dolphin Societies: Discoveries and Puzzles. University of California Press, Berkeley, CA. pp. 199–226.

Wells, R.S. 2000. Reproduction in wild bottlenose dolphins: Overview of patterns observed during a long-term study. In: Duffield, D. and T. Robeck (Eds.), Bottlenose Dolphin Reproduction Workshop. AZA Marine Mammal Taxon Advisory Group, Silver Spring, MD. pp. 57–74.

Wells, R.S. 2003. Dolphin social complexity: Lessons from long-term study and life history. In: de Waal, F.B.M. and P.L. Tyack (Eds.), Animal Social Complexity: Intelligence, Culture, and Individualized Societies Harvard University Press, Cambridge, MA. pp. 32–56.

Wells, R.S. 2014. Social structure and life history of bottlenose dolphins near Sarasota Bay, Florida: Insights from four decades and five generations. Primates and cetaceans. pp. 149–172. doi: 10.1007/978-4-431-54523-1_8

Wells, R.S., M.K. Bassos, K.W. Urian, W.J. Carr, and M.D. Scott. 1996. Low-level monitoring of bottlenose dolphins, *Tursiops truncatus*, in Charlotte Harbor, Florida: 1990-1994. National Oceanic and Atmospheric Administration, National Marine Fisheries Service, Southeast Fisheries Science Center: Miami, FL. NOAA Technical Memorandum NMFS-SEFSC-384. 86 p.

Wells, R.S., M.K. Bassos, K.W. Urian, S.H. Shane, E.C.G. Owen, C.F. Weiss, W.J. Carr, and M.D. Scott. 1997. Low-level monitoring of bottlenose dolphins, *Tursiops truncatus*, in Pine Island Sound, Florida: 1996. National Oceanic and Atmospheric Administration, National Marine Fisheries Service, Southeast Fisheries Science Center: Miami, FL. Southeast Fisheries Center Contribution No. 40-WCNF601958. 86 p.

Wells, R.S., A. Natoli, and G. Braulik. 2019. *Tursiops truncatus* (errata version published in 2019). *The IUCN Red List of Threatened Species* 2019: e.T22563A156932432. https://dx.doi.org/10.2305/IUCN.UK.2019-1.RLTS.T22563A156932432.en

Wells, R.S., L.H. Schwacke, T.K. Rowles, B.C. Balmer, E. Zolman, T. Speakman, F.I. Townsend, M.C. Tumlin, A. Barleycorn, and K.A. Wilkinson. 2017. Ranging patterns of common bottlenose dolphins *Tursiops truncatus* in Barataria Bay, Louisiana, following the Deepwater Horizon oil spill. Endangered Species Research 33:159–180. doi: 10.3354/esr00732

Wells, R.S. and M.D. Scott. 1999. Bottlenose dolphin *Tursiops truncatus* (Montagu, 1821). In: Ridgway, S.H. and R. Harrison (Eds.), Handbook of marine mammals. Volume 6: The second book of dolphins and the porpoises. Academic Press, San Diego, CA. pp. 137–182.

Wells, R.S. and M.D. Scott. 2018. Bottlenose dolphin, *Tursiops truncatus*, common bottlenose dolphin. In: Würsig, B., J.G.M. Thewissen, and K.M. Kovacs (Eds.), Encyclopedia of Marine Mammals (Third Edition). Academic Press, London, UK. pp. 118–125. doi: 10.1016/B978-0-12-804327-1.00072-8

Wells, R.S. and M.D. Scott. 2018. Bottlenose dolphin, *Tursiops aduncus*. Indo-Pacific bottlenose. In: Perrin, W.F., B. Würsig, and J.G.M. Thewissen (Eds.), Encyclopedia of Marine Mammals, Third Edition. Academic Press, London, UK. pp. 125–130.

Wells, R.S., M.D. Scott, and A.B. Irvine. 1987. The social structure of free-ranging bottlenose dolphins. In: Genoways, H.H. (Ed.), Current Mammalogy. Plenum Press, New York, NY. pp. 247–305.

Wells, R.S., K.W. Urian, A.J. Read, M.K. Bassos, W.J. Carr, and M.D. Scott. 1996. Low-level monitoring of bottlenose dolphins, *Tursiops truncatus*, in Tampa Bay, Florida: 1988-1993. National Oceanic and Atmospheric Administration, National Marine Fisheries Service, Southeast Fisheries Science Center: Miami, FL. NOAA Technical Memorandum NMFS-SEFSC-385. 36 p.

Wilkinson, K.A., R.S. Wells, W.E. Pine, and R.R. Borkhataria. 2017. Shark bite scar frequency in resident common bottlenose dolphins (*Tursiops truncatus*) in Sarasota Bay, Florida. Marine Mammal Science 33(2):678–686. doi: 10.1111/mms.12385

Wilson, R.M., R.B. Tyson, J.A. Nelson, B.C. Balmer, J.P. Chanton, and D.P. Nowacek. 2017. Niche differentiation and prey selectivity among common bottlenose dolphins (*Tursiops truncatus*) sighted in St. George Sound, Gulf of Mexico. Frontiers in Marine Science 4:235. doi: 10.3389/fmars.2017.00235

Woshner, V., K. Knott, R. Wells, C. Willetto, R. Swor, and T. O'Hara. 2008. Mercury and selenium in blood and epidermis of bottlenose dolphins (*Tursiops truncatus*) from Sarasota Bay, FL: interaction and relevance to life history and hematologic parameters. Ecohealth 5(3):360–370. doi: 10.1007/s10393-008-0164-2

Yordy, J.E., M.A. Mollenhauer, R.M. Wilson, R.S. Wells, A. Hohn, J. Sweeney, L.H. Schwacke, T.K. Rowles, J.R. Kucklick, and M.M. Peden-Adams. 2010a. Complex contaminant exposure in cetaceans: a comparative E-Screen analysis of bottlenose dolphin blubber and mixtures of four persistent organic pollutants. Environmental Toxicology and Chemistry 29(10):2143–2153. doi: 10.1002/etc.279

Yordy, J.E., D.A. Pabst, W.A. McLellan, R.S. Wells, T.K. Rowles, and J.R. Kucklick. 2010b. Tissue-specific distribution and whole-body burden estimates of persistent organic pollutants in the bottlenose dolphin (*Tursiops truncatus*). Environmental Toxicology and Chemistry 29(6):1263–1273. doi: 10.1002/etc.152

Yordy, J.E., R.S. Wells, B.C. Balmer, L.H. Schwacke, T.K. Rowles, and J.R. Kucklick. 2010c. Life history as a source of variation for persistent organic pollutant (POP) patterns in a community of common bottlenose dolphins (*Tursiops truncatus*) resident to Sarasota Bay, FL. Science of the Total Environment 408(9):2163–2172. doi: 10.1016/j.scitotenv.2010.01.032

Common bottlenose dolphin − *Tursiops truncatus*

GoMx BSE/St. Andrew Bay Stock

Overall Vulnerability Rank = Very High

Biological Sensitivity = Very High

Climate Exposure = Very High

Data Quality = 93% of scores ≥ 2

	Tursiops truncatus	Expert Scores	Data Quality	Expert Scores Plots (Portion by Category)
Sensitivity attributes	Prey/Diet Specificity	2.1	2.0	
	Habitat Specificity	3.7	2.0	
	Site Fidelity	3.8	2.3	
	Lifetime Reproductive Potential	1.2	2.0	
	Generation Length	2.9	2.0	
	Reproductive Plasticity	1.4	2.0	
	Migration	3.7	2.3	
	Home Range	3.4	2.0	
	Stock Abundance	3.1	2.3	
	Stock Abundance Trend	2.2	1.7	
	Cumulative Stressors	3.6	2.3	
	Sensitivity Score	Very High		
Exposure variables	Sea Surface Temperature (Change in mean)	4.0	3.0	
	Sea Surface Temperature (Change in variability)	1.0	3.0	
	Air Temperature (Change in mean)	4.0	3.0	
	Air Temperature (Change in variability)	1.0	3.0	
	Precipitation (Change in mean)	1.0	3.0	
	Precipitation (Change in variability)	1.0	3.0	
	Sea Surface Salinity (Change in mean)	2.0	3.0	
	Sea Surface Salinity (Change in variability)	1.8	3.0	
	Ocean pH (Change in mean)	4.0	3.0	
	Ocean pH (Change in variability)	1.0	3.0	
	Sea ice coverage (Change in mean)	1.0	3.0	
	Sea ice coverage (Change in variability)	1.0	3.0	
	Dissolved oxygen (Change in mean)	4.0	3.0	
	Dissolved oxygen (Change in variability)	1.0	3.0	
	Circulation	3.9	1.7	
	Sea level rise	3.0	3.0	
	Exposure Score	Very High		
Overall Vulnerability Rank		Very High		

Low
Moderate
High
Very High

Common bottlenose dolphin (GoMx BSE/St. Andrew Bay Stock)

Tursiops truncatus

CVA Results Summary

Overall Climate Vulnerability Rank: Very High (100% certainty from bootstrap analysis).

Climate Exposure: Very High. Five exposure factors scored greater than 3.5: Air Temperature (Standard anomaly) (4.00), Dissolved oxygen (Standard anomaly) (4.00), Ocean pH (Standard anomaly) (4.00), Sea Surface Temperature (Standard anomaly) (4.00), and Circulation (3.93).

Biological Sensitivity: Very High. Four sensitivity attributes scored greater than 3.5: Site Fidelity (3.80), Migration (3.73), Habitat Specificity (3.67), and Cumulative Stressors (3.60).

Distributional Response: Very High

Abundance Response: Very High

Phenology Response: Very High

Data Quality: 93% of the data quality scores were 2 or greater, 91% of sensitivity attribute data quality scores were 2 or higher.

Background Information

Stock Description

This stock includes common bottlenose dolphins in St. Andrew Bay in the northeast Gulf of Mexico along the Florida coast (Hayes et al. 2020).

Prey/Diet Specificity

Generally, common bottlenose dolphins consume a large variety of fish and/or squid (Barros and Odell 1990; Mead and Potter 1990; Wells and Scott 2018). Common bottlenose dolphins seem to show a consistent preference for sound-producing fishes (e.g., sciaenids, toadfish; Berens McCabe et al. 2010; Bowen-Stevens et al. 2021). In Sarasota Bay, Florida, Rossman et al. (2015) found some individual common bottlenose dolphins to show diet specialization despite the broader study group showing a generalist diet. Specialization can be influenced by locally abundant prey species (Shane et al. 1986; Vollmer and Rosel 2013) or can result from resource partitioning amongst sympatric populations (e.g., Torres and Read 2009; Wilson et al. 2017).

Habitat Specificity

Common bottlenose dolphins are found in a variety of marine and estuarine habitats (Wells and Scott 2018).

Site Fidelity

Studies from St. Andrew Bay suggest common bottlenose dolphins in this stock show site fidelity (Bouveroux et al. 2014; Balmer et al. 2019a).

Common bottlenose dolphins in other Gulf of Mexico bay, sound, and estuary (BSE) stocks also show site fidelity (LaBrecque et al. 2015; Hayes et al. 2022), such as in Matagorda Bay, Espiritu Santo Bay, and San Luis Pass in Texas (Maze and Würsig 1999; Lynn and Würsig 2002; Irwin and Würsig 2004); Barataria Bay in Louisiana (Miller 2003; McDonald et al. 2017; Wells et al. 2017; Takeshita et al. 2021); Mississippi Sound in Mississippi (Hubard et al. 2004; Mackey 2010; Mullin et al. 2017); Charlotte Harbor and Pine Island Sound (Bassos-Hull et al. 2013), Sarasota Bay (Wells 1986a; Wells 1991; Wells 2014), St. Joseph Bay (Balmer et al. 2008; B. Balmer et al. 2018), St. Vincent Sound and Apalachicola Bay (Tyson et al. 2011), and Tampa Bay in Florida (Wells et al. 1996b; Urian et al. 2009).

Lifetime Reproductive Potential

Taylor et al. (2007) reported the interbirth interval of common bottlenose dolphins as 3.80 years, though other studies have shown it to be closer to three years (Leatherwood 1977; Wells and Scott 1999; Connor et al. 2000; Wells 2003).

Generally, females reach sexual maturity before males, with females reaching sexual maturity at 5–13 years (Sergeant et al. 1973; Perrin and Reilly 1984; Wells et al. 1987; Mead and Potter 1990; Mann et al. 2000; Wells 2000; Wells and Scott 2018).

Females reproduce through most of their adult lives (Leatherwood 1977; Hohn et al. 1989; Wells and Scott 1999; Wells 2000; Wells 2003), but may produce fewer calves after they reach 30 years of age (Wells 2000). Sarasota Bay common bottlenose dolphins can give birth when they are as young as 6 years of age, and can continue until they are 48 years old (Wells and Scott 2018). Reproductive lifespan would therefore be up to 42 years based on long-term observation studies (Wells 2014; Wells and Scott 2018).

Longitudinal studies from Sarasota Bay, Florida have found that female common bottlenose dolphins can live up to 67 years (Wells and Scott 2018). Studies based on growth layer groups in teeth suggest female common bottlenose dolphins can live to 41 years along the Texas coast (Fernandez and Hohn 1998) and to 30 years along the Mississippi coast (Mattson et al. 2006).

Generation Length

Taylor et al. (2007) reported a common bottlenose dolphin generation length of 20.6–21.1 years.

Reproductive Plasticity

Common bottlenose dolphin calving is generally diffusely seasonal, with one or more peaks, although births have been reported from all seasons (Vollmer and Rosel 2013; Wells and Scott 2018). Calf sightings peak in the spring and summer months in Florida (Wells et al. 1987), Mississippi/Louisiana (Miller et al. 2010; Miller et al. 2013), and Texas (McHugh 1989; Henderson et al. 2007).

Migration

The bay, sound, and estuary stocks in the Gulf of Mexico are thought to primarily comprise stable resident communities. However, transient or seasonal movements of some individuals into and out of a BSE stock area are recorded in some areas (LaBrecque et al. 2015; Hayes et al. 2022). Molecular genetic data support that some BSE stocks are genetically distinct from one another, and furthermore are differentiated from adjacent coastal populations (e.g., Sellas et al. 2005; Rosel et al. 2017). For example, Toms (2019) found fine-scale genetic population structure among five inshore systems in the Florida Panhandle and variable migration rates among populations.

Year-round residency has been reported for common bottlenose dolphins (Hayes et al. 2022), such as in Florida (Irvine and Wells 1972; Irvine et al. 1981; Wells 1986a; Wells 1986b; Wells et al. 1987; Scott et al. 1990; Wells 1991; Wells et al. 1996a; Wells et al. 1996b; Wells et al. 1997; Wells 2003; Balmer et al. 2008; Urian et al. 2009; Tyson et al. 2011; Bassos-Hull et al. 2013; B. Balmer et al. 2018; Balmer et al. 2019a; Toms 2019), Texas (Shane 1990; Bräger 1993; Bräger et al. 1994; Fertl 1994; Weller 1998; Maze and Würsig 1999; Lynn and Würsig 2002; Irwin and Würsig 2004; Shane 2004), and Louisiana/Mississippi (Hubard et al. 2004; McDonald et al. 2017; Mullin et al. 2017; Takeshita et al. 2021).

Home Range

The home ranges of individuals within the Gulf of Mexico BSE stocks are considered to generally include the areas within the stock boundaries and brief excursions beyond the stock boundaries (Irvine et al. 1981; Wells 1986a; Wells 1986b; Wells 1991; Wells et al. 1996a; Wells et al. 1996b; Balmer et al. 2008; Wells 2014; Balmer et al. 2019a), although some individuals show preference for small areas within the stock boundaries (Lynn and Würsig 2002; Urian et al. 2009; Mullin et al. 2017; Wells et al. 2017; Cloyed et al. 2021). Balmer et al. (2019a) found few dolphins sighted in both St. Andrew Bay and along coastal waters, suggesting there may be minimal overlap between the St. Andrew Bay stock and the Northern Coastal stock.

Stock Abundance

Balmer et al. (2019) completed a photo-ID mark-recapture analysis between 2015 and 2016 and estimated this stock's abundance to be lowest in April 2016 (199 individuals; 95% CI 173–246), and highest in October 2016 (315 individuals; 95% CI 274–378). Previously, Blaylock and Hoggard (1994) estimated this stock's abundance at 124 individuals (CV = 0.57). Bouveroux et al. (2014) used robust design photo-ID mark-recapture models between March 2004 - July 2007 and found that seasonal abundance estimates ranged from 89 (CI 95%= 71–161) to 183 (CI 95%= 169–208) dolphins, even though 263 distinctive dolphins were identified during the study.

Stock Abundance Trend

Data are insufficient to complete an abundance trend analysis for this stock (Hayes et al. 2020).

Cumulative Stressors

Common bottlenose dolphins face a wide range of individual and cumulative natural and anthropogenic threats (see reviews by Vollmer and Rosel 2013; Wells and Scott 2018; and Wells et al. 2019). These

include such threats as shark predation, harmful algal blooms (Twiner et al. 2012), recreational and commercial fishing gear, vessel traffic, pollution, habitat alteration, and provisioning.

The nearshore and estuarine habitats along the Gulf of Mexico coast include waters adjacent to areas of high human population, some of which are highly industrialized, and areas of heavy agricultural impact. Studies have shown persistent organic pollutants (POPs) in tissues of common bottlenose dolphins from St. Andrew Bay (Wilson et al. 2012; Balmer et al. 2019b) and other Florida Gulf Coast waters (Yordy et al. 2010a, 2010b, 2010c; Kucklick et al. 2011; J. Balmer et al. 2018). Similarly, mercury has been detected in common bottlenose dolphin tissues in Florida Gulf Coast waters (Bryan et al. 2007; Woshner et al. 2008; Miller et al. 2011; Correa et al. 2013; McCormack et al. 2020a, 2020b, 2022).

Common bottlenose dolphins in St. Andrew Bay were possibly exposed to oil and dispersants following the Deepwater Horizon oil spill, which may result in long term reproductive and chronic health effects (NOAA 2011; Michel et al. 2013; Helm et al. 2015; DWH NRDA 2016; Lane et al. 2015; De Guise et al. 2017, 2021; Kellar et al. 2017; Smith et al. 2017; Schwacke et al. 2017). An Unusual Mortality Event (UME), involving primarily bottlenose dolphins, was declared for cetaceans in the northern Gulf of Mexico from 2010 to 2014, with the Deepwater Horizon oil spill as the primary underlying cause (Litz et al. 2014; Schwacke et al. 2014; Venn-Watson et al. 2015a, 2015b).

UMEs have been declared for common bottlenose dolphins in waters of the Florida panhandle with biotoxins (e.g., brevetoxin from *Karenia brevis*) the suspected or confirmed cause (Twiner et al. 2012; Litz et al. 2014). Dolphin morbillivirus has been detected in populations of the northern Gulf of Mexico, but has not been attributed to large scale die-offs since 1993−1994 (Lipscomb et al. 1996; Litz et al. 2014; Van Bressem et al. 2014; Fauquier et al. 2017). A UME was declared in 1990 for the Gulf of Mexico from Florida to Texas, with more than 300 common bottlenose dolphins stranding due to an undetermined cause, although morbillivirus and cold temperatures may have contributed (Litz et al. 2014).

Further Reading

Hayes, S.A., E. Josephson, K. Maze-Foley, and P.E. Rosel. 2020. US Atlantic and Gulf of Mexico Marine Mammal Stock Assessments - 2019. National Oceanic and Atmospheric Administration, National Marine Fisheries Service, Northeast Fisheries Science Center: Woods Hole, MA. NOAA Technical Memorandum NMFS-NE-264. 479 p.

Wells, R.S. and M.D. Scott. 2018. Bottlenose Dolphin, *Tursiops truncatus*, Common Bottlenose Dolphin. In: Würsig, B., J.G.M. Thewissen, and K.M. Kovacs (Eds.), Encyclopedia of Marine Mammals (Third Edition). Academic Press, London, UK. pp. 118–125. doi: 10.1016/B978-0-12-804327-1.00072-8

Wells, R.S., A. Natoli, and G. Braulik. 2019. *Tursiops truncatus* (errata version published in 2019). The IUCN Red List of Threatened Species 2019: e.T22563A156932432. https://dx.doi.org/10.2305/IUCN.UK.2019-1.RLTS.T22563A156932432.en

References

Balmer, B., T. McDonald, F. Hornsby, J. Adams, J. Allen, A. Barleycorn, and L. Schwacke. 2018. Long-term trends in a northern Gulf of Mexico common bottlenose dolphin (*Tursiops truncatus*) population in the wake of the Deepwater Horizon oil spill. Journal of Cetacean Research and Management 18:1–9. doi: 10.47536/jcrm.v18i1.409

Balmer, B., S. Watwood, B. Quigley, T. Speakman, K. Barry, K. Mullin, P. Rosel, C. Sinclair, E. Zolman, and L. Schwacke. 2019. Common bottlenose dolphin (*Tursiops truncatus*) abundance and distribution patterns in St. Andrew Bay, Florida, USA. Aquatic Conservation: Marine and Freshwater Ecosystems 29(3):486–498. doi: 10.1002/aqc.3001

Balmer, B., G. Ylitalo, S. Watwood, B. Quigley, J. Bolton, K. Mullin, P. Rosel, T. Rowles, T. Speakman, L. Wilcox, E. Zolman, and L. Schwacke. 2019. Comparison of persistent organic pollutants (POPs) between small cetaceans in coastal and estuarine waters of the northern Gulf of Mexico. Marine Pollution Bulletin 145:239–247. doi: 10.1016/j.marpolbul.2019.05.017

Balmer, B.C., R.S. Wells, S.M. Nowacek, D.P. Nowacek, L.H. Schwacke, W.A. McLellan, F.S. Scharf, T.K. Rowles, L.J. Hansen, T.R. Spradlin, and D.A. Pabst. 2008. Seasonal abundance and distribution patterns of common bottlenose dolphins (*Tursiops truncatus*) near St. Joseph Bay, Florida, USA. Journal of Cetacean Research and Management 10(2):157–167.

Balmer, J.E., G.M. Ylitalo, T.K. Rowles, K.D. Mullin, R.S. Wells, F.I. Townsend, R.W. Pearce, J.L. Bolton, E.S. Zolman, B.C. Balmer, and L.H. Schwacke. 2018. Persistent organic pollutants (POPs) in blood and blubber of common bottlenose dolphins (*Tursiops truncatus*) at three northern Gulf of Mexico sites following the Deepwater Horizon oil spill. Science of the Total Environment 621:130–137. doi: 10.1016/j.scitotenv.2017.11.209

Barros, N.B. and D.K. Odell. 1990. Food habits of bottlenose dolphins in the southeastern United States In: Leatherwood, S. and R.R. Reeves (Eds.), The Bottlenose Dolphin. Academic Press, San Diego, CA. pp. 309–328. doi: 10.1016/B978-0-12-440280-5.50020-2

Bassos-Hull, K., R.M. Perrtree, C.C. Shepard, S. Schilling, A.A. Barleycorn, J.B. Allen, B.C. Balmer, W.E. Pine, and R.S. Wells. 2013. Long-term site fidelity and seasonal abundance estimates of common bottlenose dolphins (*Tursiops truncatus*) along the southwest coast of Florida and responses to natural perturbations. Journal of Cetacean Research and Management 13(1):19–30. doi: 10.47536/jcrm.v13i1.551

Berens McCabe, E.J., D.P. Gannon, N.B. Barros, and R.S. Wells. 2010. Prey selection by resident common bottlenose dolphins (*Tursiops truncatus*) in Sarasota Bay, Florida. Marine Biology 157(5):931–942. doi: 10.1007/s00227-009-1371-2

Blaylock, R.A. and W. Hoggard. 1994. Preliminary estimates of bottlenose dolphin abundance in southern U.S. Atlantic and Gulf of Mexico continental shelf waters. National Oceanic and Atmospheric Administration, National Marine Fisheries Service, Southeast Fisheries Science Center: Miami, FL. NOAA Technical Memorandum NMFS-SEFSC-356. 10 p.

Bouveroux, T., R.B. Tyson, and D.P. Nowacek. 2014. Abundance and site fidelity of bottlenose dolphins in coastal waters near Panama City, Florida. Journal of Cetacean Research and Management 14:37–42. doi: 10.47536/jcrm.v14i1.521

Bowen-Stevens, S.R., D.P. Gannon, R.A. Hazelkorn, G. Lovewell, K.M. Volker, S. Smith, M.C. Tumlin, and J. Litz. 2021. Diet of common bottlenose dolphins, *Tursiops truncatus*, that stranded in and near Barataria Bay, Louisiana, 2010–2012. Southeastern Naturalist 20(1). doi: 10.1656/058.020.0113

Bräger, S. 1993. Diurnal and seasonal behavior patterns of bottlenose dolphins (*Tursiops truncatus*). Marine Mammal Science 9:434–438. doi: 10.1111/j.1748-7692.1993.tb00477.x

Bräger, S., B. Würsig, A. Acevedo, and T. Henningsen. 1994. Association patterns of bottle-nosed dolphins (*Tursiops-truncatus*) in Galveston Bay, Texas. Journal of Mammalogy 75(2):431–437. doi: 10.2307/1382564

Bryan, C.E., S.J. Christopher, B.C. Balmer, and R.S. Wells. 2007. Establishing baseline levels of trace elements in blood and skin of bottlenose dolphins in Sarasota Bay, Florida: implications for non-invasive monitoring. Science of the Total Environment 388(1-3):325–342. doi: 10.1016/j.scitotenv.2007.07.046

Cloyed, C.S., R.M. Wilson, B.C. Balmer, A.A. Hohn, L.H. Schwacke, E.S. Zolman, M.C. Tumlin, R.S. Wells, A.A. Barleycorn, J.B. Allen, and R.H. Carmichael. 2021. Specialization of a mobile, apex predator affects trophic coupling among adjacent habitats. Scientific Reports 11(1):19611. doi: 10.1038/s41598-021-99017-1

Connor, R.C., R.S. Wells, J. Mann, and A.J. Read. 2000. The bottlenose dolphin: Social relationships in a fission-fusion society. In: Mann, J., R. Connor, P. Tyack, and H. Whitehead (Eds.), Cetacean Societies: Field Studies of Dolphins and Whales. University of Chicago Press, Chicago, IL. pp. 91–126.

Correa, L., J.M. Castellini, R.S. Wells, and T. O'Hara. 2013. Distribution of mercury and selenium in blood compartments of bottlenose dolphins (*Tursiops truncatus*) from Sarasota Bay, Florida. Environmental Toxicology and Chemistry 32(11):2441–2448. doi: 10.1002/etc.2327

De Guise, S., M. Levin, E. Gebhard, L. Jasperse, L. Burdett Hart, C.R. Smith, S. Venn-Watson, F. Townsend, R. Wells, B. Balmer, E. Zolman, T. Rowles, and L. Schwacke. 2017. Changes in immune functions in bottlenose dolphins in the northern Gulf of Mexico associated with the Deepwater Horizon oil spill. Endangered Species Research 33:291–303. doi: 10.3354/esr00814

De Guise, S., M. Levin, L. Jasperse, J. Herrman, R.S. Wells, T. Rowles, and L. Schwacke. 2021. Long-term immunological alterations in bottlenose dolphin a decade after the Deepwater Horizon oil spill in the northern Gulf of Mexico: Potential for multigenerational effects. Environmental Toxicology and Chemistry 40(5):1308–1321. doi: 10.1002/etc.4980

Deepwater Horizon Natural Resource Damage Assessment Trustees (DWH NRDA). 2016. Deepwater Horizon oil spill: Final Programmatic Damage Assessment and Restoration Plan and Final Programmatic Environmental Impact Statement. Retrieved from: http://www.gulfspillrestoration.noaa.gov/restoration-planning/gulf-plan.1659.

Fauquier, D.A., J. Litz, S. Sanchez, K. Colegrove, L.H. Schwacke, L. Hart, J. Saliki, C. Smith, T. Goldstein, S. Bowen-Stevens, W. McFee, E. Fougeres, B. Mase-Guthrie, E. Stratton, R. Ewing, S. Venn-Watson, R.H. Carmichael, C. Clemons-Chevis, W. Hatchett, D. Shannon, S. Shippee, S. Smith, L. Staggs, M.C. Tumlin, N.L. Wingers, and T.K. Rowles. 2017. Evaluation of morbillivirus exposure in cetaceans from the northern Gulf of Mexico 2010-2014. Endangered Species Research 33:211–220. doi: 10.3354/esr00772

Fernandez, S. and A.A. Hohn. 1998. Age, growth, and calving season of bottlenose dolphins, *Tursiops truncatus*, off coastal Texas. Fishery Bulletin 96(2):357–365.

Fertl, D. 1994. Occurrence patterns and behavior of bottlenose dolphins (*Tursiops truncatus*) in the Galveston ship channel, Texas. Texas Journal of Science 46(4):299–318.

Hayes, S.A., E. Josephson, K. Maze-Foley, and P.E. Rosel. 2020. US Atlantic and Gulf of Mexico marine mammal stock assessments - 2019. National Oceanic and Atmospheric Administration, National Marine Fisheries Service, Northeast Fisheries Science Center: Woods Hole, MA. NOAA Technical Memorandum NMFS-NE-264. 479 p.

Hayes, S.A., E. Josephson, K. Maze-Foley, P.E. Rosel, and J. Wallace. 2022. U.S. Atlantic and Gulf of Mexico marine mammal stock assessments 2021. National Oceanic and Atmospheric Administration, National Marine Fisheries Service, Northeast Fisheries Science Center: Woods Hole, MA. NOAA Technical Memorandum NMFS-NE-288. 380 p. doi: 10.25923/6tt7-kc16

Helm, R.C., D.P. Costa, T.D. DeBruyn, T.J. O'Shea, R.S. Wells, and T.M. Williams. 2015. Overview of effects of oil spills on marine mammals. In: Fingas, M. (Ed.), Handbook of oil spill science and technology, First edition. John Wiley & Sons, Inc., Hoboken, NJ. pp. 455–475.

Henderson, E.E. and B. Würsig. 2007. Behavior patterns of bottlenose dolphins in San Luis Pass, Texas. Gulf of Mexico Science 25(2):153–161. doi: 10.18785/goms.2502.06

Hohn, A.A., M.D. Scott, R.S. Wells, J.C. Sweeney, and A.B. Irvine. 1989. Growth layers in teeth from known-age, free-ranging bottlenose dolphins. Marine Mammal Science 5(4):315–342. doi: 10.1111/j.1748-7692.1989.tb00346.x

Hubard, C.W., K. Maze-Foley, K.D. Mullin, and W.W. Schroeder. 2004. Seasonal abundance and site fidelity of bottlenose dolphins (*Tursiops truncatus*) in Mississippi Sound. Aquatic Mammals 30(2):299–310. doi: 10.1578/am.30.2.2004.299

Irvine, A.B., M.D. Scott, R.S. Wells, and J.H. Kaufmann. 1981. Movements and activities of the Atlantic bottlenose dolphin, *Tursiops truncatus*, near Sarasota, Florida. Fishery Bulletin 79(4):671–688.

Irvine, B. and R.S. Wells. 1972. Results of attempts to tag Atlantic bottlenosed dolphins (*Tursiops truncatus*). Cetology 13:1–5.

Irwin, L.-J. and B. Würsig. 2004. A small resident community of bottlenose dolphins, *Tursiops truncatus*, in Texas: Monitoring recommendations. Gulf of Mexico Science 22(1):13–21. doi: 10.18785/goms.2201.02

Kellar, N.M., T.R. Speakman, C.R. Smith, S.M. Lane, B.C. Balmer, M.L. Trego, K.N. Catelani, M.N. Robbins, C.D. Allen, R.S. Wells, E.S. Zolman, T.K. Rowles, and L.H. Schwacke. 2017. Low reproductive success rates of common bottlenose dolphins *Tursiops truncatus* in the northern Gulf of Mexico following the Deepwater Horizon disaster (2010-2015). Endangered Species Research 33:143–158. doi: 10.3354/esr00775

Kucklick, J., L. Schwacke, R. Wells, A. Hohn, A. Guichard, J. Yordy, L. Hansen, E. Zolman, R. Wilson, J. Litz, D. Nowacek, T. Rowles, R. Pugh, B. Balmer, C. Sinclair, and P. Rosel. 2011. Bottlenose dolphins as indicators of persistent organic pollutants in the western North Atlantic Ocean and Northern Gulf of Mexico. Environmental Science & Technology 45(10):4270–4277. doi: 10.1021/es1042244

LaBrecque, E., C. Curtice, J. Harrison, S.M. Van Parijs, and P.N. Halpin. 2015. 3. Biologically Important Areas for cetaceans within U.S. Waters – Gulf of Mexico Region. Aquatic Mammals 41(1):30–38. doi: 10.1578/am.41.1.2015.30

Lane, S.M., C.R. Smith, J. Mitchell, B.C. Balmer, K.P. Barry, T. McDonald, C.S. Mori, P.E. Rosel, T.K. Rowles, T.R. Speakman, F.I. Townsend, M.C. Tumlin, R.S. Wells, E.S. Zolman, and L.H. Schwacke. 2015. Reproductive outcome and survival of common bottlenose dolphins sampled in Barataria Bay, Louisiana, USA, following the Deepwater Horizon oil spill. Proceedings of the Royal Society B: Biological Sciences 282(1818):20151944. doi: 10.1098/rspb.2015.1944

Leatherwood, S. 1977. Some preliminary impressions on the numbers and social behavior of free-swimming bottlenosed dolphin calves (*Tursiops truncatus*) in the northern Gulf of Mexico. In: Ridgway, S.H. and K. Bernirschke (Eds.), Breeding Dolphins; Present Status, Suggestions for the Future. Final report to the US Marine Mammal Commission, Contract MM6AC009, MMC-76/07, Washington, DC. pp. 143–167.

Lipscomb, T.P., S. Kennedy, D. Moffett, A. Krafft, B.A. Klaunberg, J.H. Lichy, G.T. Regan, G.A.J. Worthy, and J.K. Taubenberger. 1996. Morbilliviral epizootic in bottlenose dolphins of the Gulf of Mexico. Journal of Veterinary Diagnostic Investigation 8(3):283–290. doi: 10.1177/104063879600800302

Litz, J.A., M.A. Baran, S.R. Bowen-Stevens, R.H. Carmichael, K.M. Colegrove, L.P. Garrison, S.E. Fire, E.M. Fougeres, R. Hardy, S. Holmes, W. Jones, B.E. Mase-Guthrie, D.K. Odell, P.E. Rosel, J.T. Saliki, D.K. Shannon, S.F. Shippee, S.M. Smith, E.M. Stratton, M.C. Tumlin, H.R. Whitehead, G.A. Worthy, and T.K. Rowles. 2014. Review of historical unusual mortality events (UMEs) in the Gulf of Mexico (1990–2009): providing context for the multi-year northern Gulf of Mexico cetacean UME declared in 2010. Diseases of Aquatic Organisms 112(2):161–175. doi: 10.3354/dao02807

Lynn, S.K. and B. Würsig. 2002. Summer movement patterns of bottlenose dolphins in a Texas bay. Gulf of Mexico Science 20(1):25–37. doi: 10.18785/goms.2001.03

Mackey, A.D. 2010. Site fidelity and association patterns of bottlenose dolphins (*Tursiops truncatus*) in the Mississippi Sound. University of Southern Mississippi. Thesis. 133 p.

Mann, J., R.C. Connor, L.M. Barre, and M.R. Heithaus. 2000. Female reproductive success in bottlenose dolphins (*Tursiops* sp.): life history, habitat, provisioning, and group-size effects. Behavioral Ecology 11(2):210–219. doi: 10.1093/beheco/11.2.210

Mattson, M.C., K.D. Mullin, G.W. Ingram, and W. Hoggard. 2006. Age structure and growth of the bottlenose dolphin (*Tursiops truncatus*) from strandings in the Mississippi sound region of the north-central Gulf of Mexico from 1986 to 2003. Marine Mammal Science 22(3):654–666. doi: 10.1111/j.1748-7692.2006.00057.x

Maze, K.S. and B. Würsig. 1999. Bottlenosed dolphins of San Luis Pass, Texas: Occurrence patterns, site-fidelity, and habitat use. Aquatic Mammals 25:91–104.

McCormack, M.A., F. Battaglia, W.E. McFee, and J. Dutton. 2020a. Mercury concentrations in blubber and skin from stranded bottlenose dolphins (*Tursiops truncatus*) along the Florida and Louisiana coasts (Gulf of Mexico, USA) in relation to biological variables. Environmental Research 180:108886. doi: 10.1016/j.envres.2019.108886

McCormack, M.A., B.P. Jackson, and J. Dutton. 2020b. Relationship between mercury and selenium concentrations in tissues from stranded odontocetes in the northern Gulf of Mexico. Science of the Total Environment 749:141350. doi: 10.1016/j.scitotenv.2020.141350

McCormack, M.A., W.H. Nowlin, and J. Dutton. 2022. Effect of trophic position on mercury concentrations in bottlenose dolphins (*Tursiops truncatus*) from the northern Gulf of Mexico. Environmental Research 204(Pt B):112124. doi: 10.1016/j.envres.2021.112124

McDonald, T.L., F.E. Hornsby, T.R. Speakman, E.S. Zolman, K.D. Mullin, C. Sinclair, P.E. Rosel, L. Thomas, and L.H. Schwacke. 2017. Survival, density, and abundance of common bottlenose dolphins in Barataria Bay (USA) following the Deepwater Horizon oil spill. Endangered Species Research 33:193–209. doi: 10.3354/esr00806

McHugh, M.B. 1989. Population numbers and feeding behavior of the Atlantic bottlenose dolphin (*Tursiops truncatus*) near Aransas Pass, Texas. University of Texas at Austin. Thesis. 96 p.

Mead, J.G. and C.W. Potter. 1990. Natural history of bottlenose dolphins along the central Atlantic coast of the United States. In: Leatherwood, S. and R.R. Reeves (Eds.), The Bottlenose Dolphin. Academic Press, San Diego, CA. pp. 165–195.

Michel, J., E.H. Owens, S. Zengel, A. Graham, Z. Nixon, T. Allard, W. Holton, P.D. Reimer, A. Lamarche, M. White, N. Rutherford, C. Childs, G. Mauseth, G. Challenger, and E. Taylor. 2013. Extent and degree of shoreline oiling: Deepwater Horizon oil spill, Gulf of Mexico, USA. PLoS ONE 8(6):e65087. doi: 10.1371/journal.pone.0065087

Miller, C.E. 2003. Abundance trends and environmental habitat usage patterns of bottlenose dolphins (*Tursiops truncatus*) in lower Barataria and Caminada bays, Louisiana. Louisiana State University and Agricultural & Mechanical College. Dissertation. 125 p.

Miller, D.L., V. Woshner, E.L. Styer, S. Ferguson, K.K. Knott, M.J. Gray, R.S. Wells, and T.M. O'Hara. 2011. Histologic findings in free-ranging Sarasota Bay bottlenose dolphin (*Tursiops truncatus*) skin: mercury, selenium, and seasonal factors. Journal of Wildlife Diseases 47(4):1012–1018. doi: 10.7589/0090-3558-47.4.1012

Miller, L.J., A.D. Mackey, T. Hoffland, M. Solangi, and S.A. Kuczaj. 2010. Potential effects of a major hurricane on Atlantic bottlenose dolphin (*Tursiops truncatus*) reproduction in the Mississippi Sound. Marine Mammal Science 26(3):707–715. doi: 10.1111/j.1748-7692.2010.00371.x

Miller, L.J., A.D. Mackey, M. Solangi, and S.A. Kuczaj. 2013. Population abundance and habitat utilization of bottlenose dolphins in the Mississippi Sound. Aquatic Conservation: Marine and Freshwater Ecosystems 23(1):145–151. doi: 10.1002/aqc.2278

Mullin, K.D., T. McDonald, R.S. Wells, B.C. Balmer, T. Speakman, C. Sinclair, E.S. Zolman, F. Hornsby, S.M. McBride, K.A. Wilkinson, and L.H. Schwacke. 2017. Density, abundance, survival, and ranging patterns of common bottlenose dolphins (*Tursiops truncatus*) in Mississippi Sound following the Deepwater Horizon oil spill. PLoS ONE 12(10):e0186265. doi: 10.1371/journal.pone.0186265

National Oceanic and Atmospheric Administration (NOAA). 2011. Public scoping for preparation of a programmatic environmental impact statement for the Deepwater Horizon BP Oil Spill. 9 p.

Perrin, W.F. and S.B. Reilly. 1984. Reproductive parameters of dolphins and small whales of the family Delphinidae. In: Perrin, W.F., R.L. Brownell, Jr., and D.P. DeMaster (Eds.), Reproduction in Whales, Dolphins, and Porpoises. pp. 97–134.

Rosel, P.E., L.A. Wilcox, C. Sinclair, T.R. Speakman, M.C. Tumlin, J.A. Litz, and E.S. Zolman. 2017. Genetic assignment to stock of stranded common bottlenose dolphins in southeastern Louisiana after the Deepwater Horizon oil spill. Endangered Species Research 33:221–234. doi: 10.3354/esr00780

Rossman, S., P.H. Ostrom, M. Stolen, N.B. Barros, H. Gandhi, C.A. Stricker, and R.S. Wells. 2015. Individual specialization in the foraging habits of female bottlenose dolphins living in a trophically diverse and habitat rich estuary. Oecologia 178(2):415–425. doi: 10.1007/s00442-015-3241-6

Schwacke, L.H., C.R. Smith, F.I. Townsend, R.S. Wells, L.B. Hart, B.C. Balmer, T.K. Collier, S. De Guise, M.M. Fry, L.J. Guillette, Jr., S.V. Lamb, S.M. Lane, W.E. McFee, N.J. Place, M.C. Tumlin, G.M. Ylitalo, E.S. Zolman, and T.K. Rowles. 2014. Health of common bottlenose dolphins (*Tursiops truncatus*) in Barataria Bay, Louisiana, following the deepwater horizon oil spill. Environmental Science & Technology 48(1):93–103. doi: 10.1021/es403610f

Schwacke, L.H., L. Thomas, R.S. Wells, W.E. McFee, A.A. Hohn, K.D. Mullin, E.S. Zolman, B.M. Quigley, T.K. Rowles, and J.H. Schwacke. 2017. Quantifying injury to common bottlenose dolphins from the Deepwater Horizon oil spill using an age-, sex- and class-structured population model. Endangered Species Research 33:265–279. doi: 10.3354/esr00777

Scott, M.D. and S.J. Chivers. 1990. Distribution and herd structure of bottlenose dolphins in the eastern tropical Pacific Ocean. In: Leatherwood, S. and R.R. Reeves (Eds.), The Bottlenose Dolphin. Academic Press, San Diego, CA. pp. 387–402.

Sellas, A.B., R.S. Wells, and P.E. Rosel. 2005. Mitochondrial and nuclear DNA analyses reveal fine scale geographic structure in bottlenose dolphins (*Tursiops truncatus*) in the Gulf of Mexico. Conservation Genetics 6(5):715–728. doi: 10.1007/s10592-005-9031-7

Sergeant, D.E., D.K. Caldwell, and M.C. Caldwell. 1973. Age, growth, and maturity of bottlenosed dolphin (*Tursiops-truncatus*) from northeast Florida. Journal of the Fisheries Research Board of Canada 30(7):1009–1011. doi: 10.1139/f73-165

Shane, S.H. 1990. Behavior and ecology of the bottlenose dolphin at Sanibel Island, Florida. In: Leatherwood, S. and R.R. Reeves (Eds.), The Bottlenose Dolphin. Academic Press, San Diego, CA. pp. 245–265. doi: 10.1016/B978-0-12-440280-5.50016-0

Shane, S.H. 2004. Residence patterns, group characteristics, and association patterns of bottlenose dolphins near Sanibel Island, Florida. Gulf of Mexico Science 22(1):1–12. doi: 10.18785/goms.2201.01

Shane, S.H., R.S. Wells, and B. Würsig. 1986. Ecology, behavior and social organization of the bottlenose dolphin: A review. Marine Mammal Science 2:34–63. doi: 10.1111/j.1748-7692.1986.tb00026.x

Smith, C.R., T.K. Rowles, L.B. Hart, F.I. Townsend, R.S. Wells, E.S. Zolman, B.C. Balmer, B. Quigley, M. Ivančić, W. McKercher, M.C. Tumlin, K.D. Mullin, J.D. Adams, Q. Wu, W. McFee, T.K. Collier, and L.H. Schwacke. 2017. Slow recovery of Barataria Bay dolphin health following the Deepwater Horizon oil spill (2013-2014), with evidence of persistent lung disease and impaired stress response. Endangered Species Research 33:127–142. doi: 10.3354/esr00778

Takeshita, R., B.C. Balmer, F. Messina, E.S. Zolman, L. Thomas, R.S. Wells, C.R. Smith, T.K. Rowles, and L.H. Schwacke. 2021. High site-fidelity in common bottlenose dolphins despite low salinity exposure and associated indicators of compromised health. PLoS ONE 16(9):e0258031. doi: 10.1371/journal.pone.0258031

Taylor, B.L., S.J. Chivers, J. Larese, and W.F. Perrin. 2007. Generation length and percent mature estimates for IUCN assessments of cetaceans. National Oceanic and Atmospheric Administration, National Marine Fisheries Service, Southwest Fisheries Science Center: La Jolla, CA. Administrative Report LJ-07-01. 24 p.

Toms, C. 2019. Filling the gaps: Common bottlenose dolphin (*Tursiops truncatus*) population dynamics, structure, and connectivity within Florida panhandle bays, sounds, and estuaries. University of Central Florida. Dissertation. 373 p.

Torres, L.G. and A.J. Read. 2009. Where to catch a fish? The influence of foraging tactics on the ecology of bottlenose dolphins (*Tursiops truncatus*) in Florida Bay, Florida. Marine Mammal Science 25(4):797–815. doi: 10.1111/j.1748-7692.2009.00297.x

Twiner, M.J., L.J. Flewelling, S.E. Fire, S.R. Bowen-Stevens, J.K. Gaydos, C.K. Johnson, J.H. Landsberg, T.A. Leighfield, B. Mase-Guthrie, L. Schwacke, F.M. Van Dolah, Z. Wang, and T.K. Rowles. 2012. Comparative analysis of three brevetoxin-associated bottlenose dolphin (*Tursiops truncatus*) mortality events in the Florida Panhandle region (USA). PLoS ONE 7(8):e42974. doi: 10.1371/journal.pone.0042974

Tyson, R.B., S.M. Nowacek, and D.P. Nowacek. 2011. Community structure and abundance of bottlenose dolphins *Tursiops truncatus* in coastal waters of the northeast Gulf of Mexico. Marine Ecology Progress Series 438:253–265. doi: 10.3354/meps09292

Urian, K.W., S. Hofmann, R.S. Wells, and A.J. Read. 2009. Fine-scale population structure of bottlenose dolphins (*Tursiops truncatus*) in Tampa Bay, Florida. Marine Mammal Science 25(3):619–638. doi: 10.1111/j.1748-7692.2009.00284.x

Van Bressem, M.F., P.J. Duignan, A. Banyard, M. Barbieri, K.M. Colegrove, S. De Guise, G. Di Guardo, A. Dobson, M. Domingo, D. Fauquier, A. Fernandez, T. Goldstein, B. Grenfell, K.R. Groch, F. Gulland, B.A. Jensen, P.D. Jepson, A. Hall, T. Kuiken, S. Mazzariol, S.E. Morris, O. Nielsen, J.A. Raga, T.K. Rowles, J. Saliki, E. Sierra, N. Stephens, B. Stone, I. Tomo, J. Wang, T. Waltzek, and J.F. Wellehan. 2014. Cetacean morbillivirus: current knowledge and future directions. Viruses 6(12):5145–5181. doi: 10.3390/v6125145

Venn-Watson, S., L. Garrison, J. Litz, E. Fougeres, B. Mase, G. Rappucci, E. Stratton, R. Carmichael, D. Odell, D. Shannon, S. Shippee, S. Smith, L. Staggs, M. Tumlin, H. Whitehead, and T. Rowles. 2015. Demographic clusters identified within the northern Gulf of Mexico common bottlenose dolphin (*Tursiops truncates*) unusual mortality event: January 2010-June 2013. PLoS ONE 10(2):e0117248. doi: 10.1371/journal.pone.0117248

Vollmer, N.L. and P.E. Rosel. 2013. A review of common bottlenose dolphins (*Tursiops truncatus truncatus*) in the Northern Gulf of Mexico: Population biology, potential threats, and management. Southeastern Naturalist 12(m6):1–43. doi: 10.1656/058.012.m601

Weller, D.W. 1998. Global and regional variation in the biology and behavior of bottlenose dolphins. Texas A&M University. Dissertation. 142 p.

Wells, R.S. 1986a. Population structure of bottlenose dolphins: Behavioral studies along the central west coast of Florida. Contract Report to National Marine Fisheries Service, Southeast Fisheries Center. Contract No. 45-WCNF-5-003BB. 58 p.

Wells, R.S. 1986b. Structural aspects of dolphin societies. University of California, Santa Cruz. Dissertation. 234 p.

Wells, R.S. 1991. The role of long-term study in understanding the social structure of a bottlenose dolphin community. In: Pryor, K. and K. Norris (Eds.), Dolphin Societies: Discoveries and Puzzles. University of California Press, Berkeley, CA. pp. 199–226.
Wells, R.S. 2000. Reproduction in wild bottlenose dolphins: Overview of patterns observed during a long-term study. In: Duffield, D. and T. Robeck (Eds.), Bottlenose Dolphin Reproduction Workshop. AZA Marine Mammal Taxon Advisory Group, Silver Spring, MD. pp. 57–74.
Wells, R.S. 2003. Dolphin social complexity: Lessons from long-term study and life history. In: de Waal, F.B.M. and P.L. Tyack (Eds.), Animal Social Complexity: Intelligence, Culture, and Individualized Societies Harvard University Press, Cambridge, MA. pp. 32–56.
Wells, R.S. 2014. Social structure and life history of bottlenose dolphins near Sarasota Bay, Florida: Insights from four decades and five generations. Primates and cetaceans. pp. 149–172. doi: 10.1007/978-4-431-54523-1_8
Wells, R.S., M.K. Bassos, K.W. Urian, W.J. Carr, and M.D. Scott. 1996. Low-level monitoring of bottlenose dolphins, *Tursiops truncatus*, in Charlotte Harbor, Florida: 1990-1994. National Oceanic and Atmospheric Administration, National Marine Fisheries Service, Southeast Fisheries Science Center: Miami, FL. NOAA Technical Memorandum NMFS-SEFSC-384. 86 p.
Wells, R.S., M.K. Bassos, K.W. Urian, S.H. Shane, E.C.G. Owen, C.F. Weiss, W.J. Carr, and M.D. Scott. 1997. Low-level monitoring of bottlenose dolphins, *Tursiops truncatus*, in Pine Island Sound, Florida: 1996. National Oceanic and Atmospheric Administration, National Marine Fisheries Service, Southeast Fisheries Science Center: Miami, FL. Southeast Fisheries Center Contribution No. 40-WCNF601958. 86 p.
Wells, R.S., A. Natoli, and G. Braulik. 2019. *Tursiops truncatus* (errata version published in 2019). *The IUCN Red List of Threatened Species* 2019: e.T22563A156932432. https://dx.doi.org/10.2305/IUCN.UK.2019-1.RLTS.T22563A156932432.en
Wells, R.S., L.H. Schwacke, T.K. Rowles, B.C. Balmer, E. Zolman, T. Speakman, F.I. Townsend, M.C. Tumlin, A. Barleycorn, and K.A. Wilkinson. 2017. Ranging patterns of common bottlenose dolphins *Tursiops truncatus* in Barataria Bay, Louisiana, following the Deepwater Horizon oil spill. Endangered Species Research 33:159–180. doi: 10.3354/esr00732
Wells, R.S. and M.D. Scott. 1999. Bottlenose dolphin *Tursiops truncatus* (Montagu, 1821). In: Ridgway, S.H. and R. Harrison (Eds.), Handbook of marine mammals. Volume 6: The second book of dolphins and the porpoises. Academic Press, San Diego, CA. pp. 137–182.
Wells, R.S. and M.D. Scott. 2018. Bottlenose dolphin, *Tursiops truncatus*, common bottlenose dolphin. In: Würsig, B., J.G.M. Thewissen, and K.M. Kovacs (Eds.), Encyclopedia of Marine Mammals (Third Edition). Academic Press, London, UK. pp. 118–125. doi: 10.1016/B978-0-12-804327-1.00072-8
Wells, R.S., M.D. Scott, and A.B. Irvine. 1987. The social structure of free-ranging bottlenose dolphins. In: Genoways, H.H. (Ed.), Current Mammalogy. Plenum Press, New York, NY. pp. 247–305.
Wells, R.S., K.W. Urian, A.J. Read, M.K. Bassos, W.J. Carr, and M.D. Scott. 1996. Low-level monitoring of bottlenose dolphins, *Tursiops truncatus*, in Tampa Bay, Florida: 1988-1993. National Oceanic and Atmospheric Administration, National Marine Fisheries Service, Southeast Fisheries Science Center: Miami, FL. NOAA Technical Memorandum NMFS-SEFSC-385. 36 p.
Wilson, R.M., R.B. Tyson, J.A. Nelson, B.C. Balmer, J.P. Chanton, and D.P. Nowacek. 2017. Niche differentiation and prey selectivity among common bottlenose dolphins (*Tursiops truncatus*) sighted in St. George Sound, Gulf of Mexico. Frontiers in Marine Science 4:235. doi: 10.3389/fmars.2017.00235
Woshner, V., K. Knott, R. Wells, C. Willetto, R. Swor, and T. O'Hara. 2008. Mercury and selenium in blood and epidermis of bottlenose dolphins (*Tursiops truncatus*) from Sarasota Bay, FL: interaction and

relevance to life history and hematologic parameters. Ecohealth 5(3):360–370. doi: 10.1007/s10393-008-0164-2

Yordy, J.E., M.A. Mollenhauer, R.M. Wilson, R.S. Wells, A. Hohn, J. Sweeney, L.H. Schwacke, T.K. Rowles, J.R. Kucklick, and M.M. Peden-Adams. 2010a. Complex contaminant exposure in cetaceans: a comparative E-Screen analysis of bottlenose dolphin blubber and mixtures of four persistent organic pollutants. Environmental Toxicology and Chemistry 29(10):2143–2153. doi: 10.1002/etc.279

Yordy, J.E., D.A. Pabst, W.A. McLellan, R.S. Wells, T.K. Rowles, and J.R. Kucklick. 2010b. Tissue-specific distribution and whole-body burden estimates of persistent organic pollutants in the bottlenose dolphin (*Tursiops truncatus*). Environmental Toxicology and Chemistry 29(6):1263–1273. doi: 10.1002/etc.152

Yordy, J.E., R.S. Wells, B.C. Balmer, L.H. Schwacke, T.K. Rowles, and J.R. Kucklick. 2010c. Life history as a source of variation for persistent organic pollutant (POP) patterns in a community of common bottlenose dolphins (*Tursiops truncatus*) resident to Sarasota Bay, FL. Science of the Total Environment 408(9):2163–2172. doi: 10.1016/j.scitotenv.2010.01.032

Common bottlenose dolphin − *Tursiops truncatus*

GoMx BSE/St. Joseph Bay Stock

Overall Vulnerability Rank = Very High

Biological Sensitivity = Very High

Climate Exposure = Very High

Data Quality = 93% of scores ≥ 2

	Tursiops truncatus	Expert Scores	Data Quality
Sensitivity attributes	Prey/Diet Specificity	1.9	2.0
	Habitat Specificity	3.5	2.0
	Site Fidelity	3.9	3.0
	Lifetime Reproductive Potential	1.3	2.0
	Generation Length	2.9	2.0
	Reproductive Plasticity	1.3	2.0
	Migration	3.8	3.0
	Home Range	3.6	2.7
	Stock Abundance	3.0	3.0
	Stock Abundance Trend	2.1	1.7
	Cumulative Stressors	3.3	2.0
	Sensitivity Score	Very High	
Exposure variables	Sea Surface Temperature (Change in mean)	4.0	3.0
	Sea Surface Temperature (Change in variability)	1.0	3.0
	Air Temperature (Change in mean)	4.0	3.0
	Air Temperature (Change in variability)	1.0	3.0
	Precipitation (Change in mean)	1.0	3.0
	Precipitation (Change in variability)	1.0	3.0
	Sea Surface Salinity (Change in mean)	2.0	3.0
	Sea Surface Salinity (Change in variability)	1.0	3.0
	Ocean pH (Change in mean)	4.0	3.0
	Ocean pH (Change in variability)	1.0	3.0
	Sea ice coverage (Change in mean)	1.0	3.0
	Sea ice coverage (Change in variability)	1.0	3.0
	Dissolved oxygen (Change in mean)	4.0	3.0
	Dissolved oxygen (Change in variability)	1.0	3.0
	Circulation	3.9	1.3
	Sea level rise	3.0	3.0
	Exposure Score	Very High	
Overall Vulnerability Rank		Very High	

Expert Scores Plots (Portion by Category)

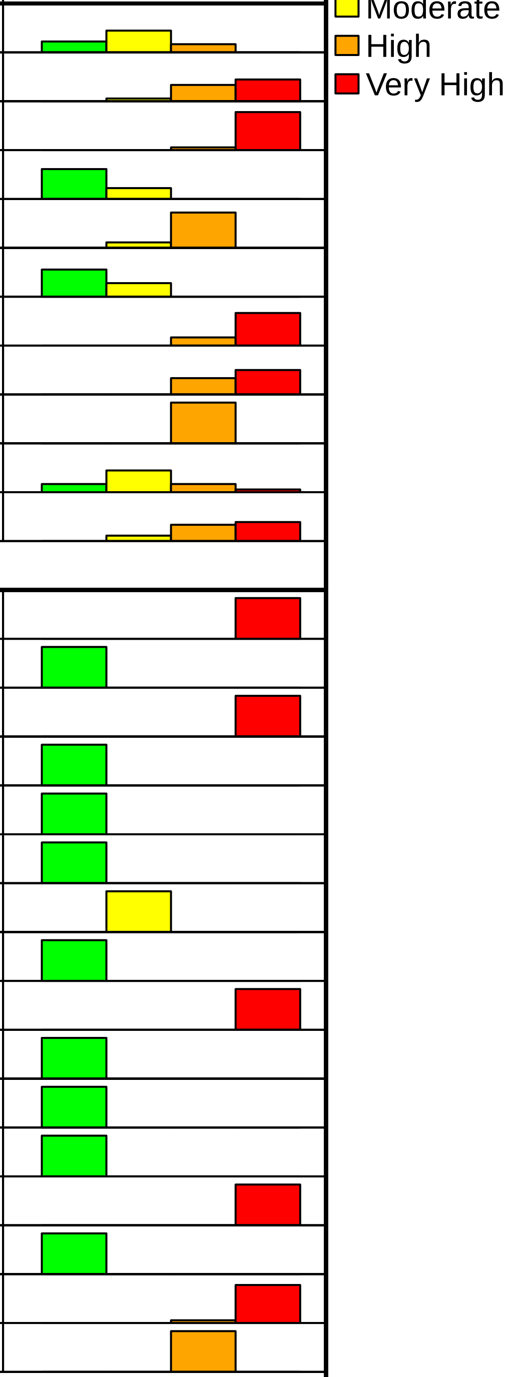

Common bottlenose dolphin (GoMx BSE/St. Joseph Bay Stock)

Tursiops truncatus

CVA Results Summary

Overall Climate Vulnerability Rank: Very High (100% certainty from bootstrap analysis).

Climate Exposure: Very High. Five exposure factors scored greater than 3.5: Air Temperature (Standard anomaly) (4.00), Dissolved oxygen (Standard anomaly) (4.00), Ocean pH (Standard anomaly) (4.00), Sea Surface Temperature (Standard anomaly) (4.00), and Circulation (3.93).

Biological Sensitivity: Very High. Three sensitivity attributes scored greater than 3.5: Site Fidelity (3.93), Migration (3.80), and Home Range (3.60).

Distributional Response: High

Abundance Response: High

Phenology Response: High

Data Quality: 93% of the data quality scores were 2 or greater, 91% of sensitivity attribute data quality scores were 2 or higher.

Background Information

Stock Description

This stock includes common bottlenose dolphins in St. Joseph Bay, Crooked Island Sound and coastal waters out to 2 km from shore along St. Joseph Peninsula and Crooked Island in the northeast Gulf of Mexico along the Florida coast (Hayes et al. 2020).

Prey/Diet Specificity

Generally, common bottlenose dolphins consume a large variety of fish and/or squid (Barros and Odell 1990; Mead and Potter 1990; Wells and Scott 2018). Common bottlenose dolphins seem to show a consistent preference for sound-producing fishes (e.g., sciaenids, toadfish; Berens McCabe et al. 2010; Bowen-Stevens et al. 2021). In Sarasota Bay, Florida, Rossman et al. (2015) found some individual common bottlenose dolphins to show diet specialization despite the broader study group showing a generalist diet. Specialization can be influenced by locally abundant prey species (Shane et al. 1986; Vollmer and Rosel 2013) or can result from resource partitioning amongst sympatric populations (e.g., Torres and Read 2009; Wilson et al. 2017).

Habitat Specificity

Common bottlenose dolphins are found in a variety of marine and estuarine habitats (Wells and Scott 2018).

Site Fidelity

Studies from St. Joseph Bay suggest common bottlenose dolphins in this stock show site fidelity (Balmer et al. 2008; Balmer et al. 2018).

Common bottlenose dolphins in other Gulf of Mexico bay, sound, and estuary (BSE) stocks also show site fidelity (LaBrecque et al. 2015; Hayes et al. 2022), such as in Charlotte Harbor and Pine Island Sound (Bassos-Hull et al. 2013), Sarasota Bay (Wells 1986a; Wells 1991; Wells 2014), St. Andrew Bay (Balmer et al. 2019), St. Vincent Sound and Apalachicola Bay (Tyson et al. 2011), and Tampa Bay in Florida (Wells et al. 1996b; Urian et al. 2009); Matagorda Bay, Espiritu Santo Bay, and San Luis Pass in Texas (Maze and Würsig 1999; Lynn and Würsig 2002; Irwin and Würsig 2004); Barataria Bay in Louisiana (Miller 2003; McDonald et al. 2017; Wells et al. 2017; Takeshita et al. 2021); and Mississippi Sound in Mississippi (Hubard et al. 2004; Mackey 2010; Mullin et al. 2017).

Lifetime Reproductive Potential

Taylor et al. (2007) reported the interbirth interval of common bottlenose dolphins as 3.80 years, though other studies have shown it to be closer to three years (Leatherwood 1977; Wells and Scott 1999; Connor et al. 2000; Wells 2003).

Generally, females reach sexual maturity before males, with females reaching sexual maturity at 5–13 years (Sergeant et al. 1973; Perrin and Reilly 1984; Wells et al. 1987; Mead and Potter 1990; Mann et al. 2000; Wells 2000; Wells and Scott 2018).

Females reproduce through most of their adult lives (Leatherwood 1977; Hohn et al. 1989; Wells and Scott 1999; Wells 2000; Wells 2003), but may produce fewer calves after they reach 30 years of age (Wells 2000). Sarasota Bay common bottlenose dolphins can give birth when they are as young as 6 years of age, and can continue until they are 48 years old (Wells and Scott 2018). Reproductive lifespan would therefore be up to 42 years based on long-term observation studies (Wells 2014; Wells and Scott 2018).

Longitudinal studies from Sarasota Bay, Florida have found that female common bottlenose dolphins can live up to 67 years (Wells and Scott 2018). Studies based on growth layer groups in teeth suggest female common bottlenose dolphins can live to 41 years along the Texas coast (Fernandez and Hohn 1998) and to 30 years along the Mississippi coast (Mattson et al. 2006).

Generation Length

Taylor et al. (2007) reported common bottlenose dolphin generation length of 20.6–21.1 years.

Reproductive Plasticity

Common bottlenose dolphin calving is generally diffusely seasonal, with one or more peaks, although births have been reported from all seasons (Vollmer and Rosel 2013; Wells and Scott 2018). Calf sightings peak in the spring and summer months in Florida (Wells et al. 1987), Mississippi/Louisiana (Miller et al. 2010; Miller et al. 2013), and Texas (McHugh 1989; Henderson et al. 2007).

Migration

The BSE stocks in the Gulf of Mexico are thought to primarily comprise stable resident communities. However, transient or seasonal movements of some individuals into and out of a BSE stock area are recorded in some areas (LaBrecque et al. 2015; Hayes et al. 2022). Molecular genetic data support that some BSE stocks are genetically distinct from one another, and furthermore are differentiated from adjacent coastal populations (e.g., Sellas et al. 2005; Rosel et al. 2017). For example, Toms (2019) found fine-scale genetic population structure among five inshore systems in the Florida Panhandle and variable migration rates among populations.

Year-round residency has been reported for common bottlenose dolphins (Hayes et al. 2022), such as in Florida (Irvine and Wells 1972; Irvine et al. 1981; Wells 1986a; Wells 1986b; Wells et al. 1987; Scott et al. 1990; Wells 1991; Wells et al. 1996a; Wells et al. 1996b; Wells et al. 1997; Wells 2003; Balmer et al. 2008; Urian et al. 2009; Tyson et al. 2011; Bassos-Hull et al. 2013; Balmer et al. 2018; Balmer et al. 2019; Toms 2019), Texas (Shane 1990; Bräger 1993; Bräger et al. 1994; Fertl 1994; Weller 1998; Maze and Würsig 1999; Lynn and Würsig 2002; Irwin and Würsig 2004; Shane 2004), and Louisiana/Mississippi (Hubard et al. 2004; McDonald et al. 2017; Mullin et al. 2017; Takeshita et al. 2021).

Home Range

The home ranges of individuals within the Gulf of Mexico BSE stocks are considered to generally include the areas within the stock boundaries and brief excursions beyond the stock boundaries (Irvine et al. 1981; Wells 1986a; Wells 1986b; Wells 1991; Wells et al. 1996a; Wells et al. 1996b; Balmer et al. 2008; Wells 2014; Balmer et al. 2019), although some individuals show preference for small areas within the stock boundaries (Lynn and Würsig 2002; Urian et al. 2009; Mullin et al. 2017; Wells et al. 2017; Cloyed et al. 2021).

Stock Abundance

Balmer et al. (2018) estimated the abundance of this stock at 142 individuals (95% CI: 92–190; CV=0.17), based on a February 2011 vessel-based capture-recapture photo-ID survey.

Stock Abundance Trend

There is no evidence of a trend in this stock's abundance based on estimates from February/March 2005, February 2006, and February 2011 (Balmer et al. 2018; Hayes et al. 2020).

Cumulative Stressors

Common bottlenose dolphins face a wide range of individual and cumulative natural and anthropogenic threats (see reviews by Vollmer and Rosel 2013; Wells and Scott 2018; and Wells et al. 2019). These include such threats as shark predation, harmful algal blooms (Schwacke et al. 2010; Twiner et al. 2012), recreational and commercial fishing gear, vessel traffic, pollution, habitat alteration, and provisioning.

The nearshore and estuarine habitats along the Gulf of Mexico coast include waters adjacent to areas of high human population, some of which are highly industrialized, and areas of heavy agricultural impact. Studies have shown persistent organic pollutants (POPs) in tissues of common bottlenose dolphins from St. Joseph Bay (Yordy et al. 2010a; Wilson et al. 2012; Balmer et al. 2015) and other Florida Gulf Coast

waters (Yordy et al. 2010b, 2010c; Kucklick et al. 2011; Balmer et al. 2018). Similarly, mercury has been detected in common bottlenose dolphin tissues in Florida Gulf Coast waters (Bryan et al. 2007; Woshner et al. 2008; Miller et al. 2011; Correa et al. 2013; McCormack et al. 2020a, 2020b, 2022).

Common bottlenose dolphins in St. Joseph Bay were possibly exposed to oil and dispersants following the Deepwater Horizon oil spill, which may result in long term reproductive and chronic health effects (NOAA 2011; Michel et al. 2013; Helm et al. 2015; DWH NRDA 2016; Lane et al. 2015; De Guise et al. 2017, 2021; Kellar et al. 2017; Smith et al. 2017; Schwacke et al. 2017). An Unusual Mortality Event (UME), involving primarily bottlenose dolphins, was declared for cetaceans in the northern Gulf of Mexico from 2010 to 2014, with the Deepwater Horizon oil spill as the primary underlying cause (Litz et al. 2014; Schwacke et al. 2014; Venn-Watson et al. 2015a, 2015b).

UMEs have been declared for common bottlenose dolphins in waters of the Florida panhandle with biotoxins (e.g., brevetoxin from *Karenia brevis*) the suspected or confirmed cause (Twiner et al. 2012; Litz et al. 2014). Dolphin morbillivirus has been detected in populations of the northern Gulf of Mexico, but has not been attributed to large scale die-offs since 1993−1994 (Lipscomb et al. 1996; Litz et al. 2014; Van Bressem et al. 2014; Fauquier et al. 2017). A UME was declared in 1990 for the Gulf of Mexico from Florida to Texas, with more than 300 common bottlenose dolphins stranding due to an undetermined cause, although morbillivirus and cold temperatures may have contributed (Litz et al. 2014).

Further Reading

Hayes, S.A., E. Josephson, K. Maze-Foley, and P.E. Rosel. 2020. US Atlantic and Gulf of Mexico Marine Mammal Stock Assessments - 2019. National Oceanic and Atmospheric Administration, National Marine Fisheries Service, Northeast Fisheries Science Center: Woods Hole, MA. NOAA Technical Memorandum NMFS-NE-264. 479 p.

Wells, R.S. and M.D. Scott. 2018. Bottlenose Dolphin, *Tursiops truncatus*, Common Bottlenose Dolphin. In: Würsig, B., J.G.M. Thewissen, and K.M. Kovacs (Eds.), Encyclopedia of Marine Mammals (Third Edition). Academic Press, London, UK. pp. 118–125. doi: 10.1016/B978-0-12-804327-1.00072-8

Wells, R.S., A. Natoli, and G. Braulik. 2019. *Tursiops truncatus* (errata version published in 2019). The IUCN Red List of Threatened Species 2019: e.T22563A156932432. https://dx.doi.org/10.2305/IUCN.UK.2019-1.RLTS.T22563A156932432.en

References

Balmer, B., T. McDonald, F. Hornsby, J. Adams, J. Allen, A. Barleycorn, and L. Schwacke. 2018. Long-term trends in a northern Gulf of Mexico common bottlenose dolphin (*Tursiops truncatus*) population in the wake of the Deepwater Horizon oil spill. Journal of Cetacean Research and Management 18:1–9. doi: 10.47536/jcrm.v18i1.409

Balmer, B., S. Watwood, B. Quigley, T. Speakman, K. Barry, K. Mullin, P. Rosel, C. Sinclair, E. Zolman, and L. Schwacke. 2019. Common bottlenose dolphin (*Tursiops truncatus*) abundance and distribution patterns in St. Andrew Bay, Florida, USA. Aquatic Conservation: Marine and Freshwater Ecosystems 29(3):486–498. doi: 10.1002/aqc.3001

Balmer, B.C., R.S. Wells, S.M. Nowacek, D.P. Nowacek, L.H. Schwacke, W.A. McLellan, F.S. Scharf, T.K. Rowles, L.J. Hansen, T.R. Spradlin, and D.A. Pabst. 2008. Seasonal abundance and distribution

patterns of common bottlenose dolphins (*Tursiops truncatus*) near St. Joseph Bay, Florida, USA. Journal of Cetacean Research and Management 10(2):157–167.

Balmer, B.C., G.M. Ylitalo, L.E. McGeorge, K.A. Baugh, D. Boyd, K.D. Mullin, P.E. Rosel, C. Sinclair, R.S. Wells, E.S. Zolman, and L.H. Schwacke. 2015. Persistent organic pollutants (POPs) in blubber of common bottlenose dolphins (*Tursiops truncatus*) along the northern Gulf of Mexico coast, USA. Science of the Total Environment 527-528:306–312. doi: 10.1016/j.scitotenv.2015.05.016

Barros, N.B. and D.K. Odell. 1990. Food habits of bottlenose dolphins in the southeastern United States In: Leatherwood, S. and R.R. Reeves (Eds.), The Bottlenose Dolphin. Academic Press, San Diego, CA. pp. 309–328. doi: 10.1016/B978-0-12-440280-5.50020-2

Bassos-Hull, K., R.M. Perrtree, C.C. Shepard, S. Schilling, A.A. Barleycorn, J.B. Allen, B.C. Balmer, W.E. Pine, and R.S. Wells. 2013. Long-term site fidelity and seasonal abundance estimates of common bottlenose dolphins (*Tursiops truncatus*) along the southwest coast of Florida and responses to natural perturbations. Journal of Cetacean Research and Management 13(1):19–30. doi: 10.47536/jcrm.v13i1.551

Berens McCabe, E.J., D.P. Gannon, N.B. Barros, and R.S. Wells. 2010. Prey selection by resident common bottlenose dolphins (*Tursiops truncatus*) in Sarasota Bay, Florida. Marine Biology 157(5):931–942. doi: 10.1007/s00227-009-1371-2

Bowen-Stevens, S.R., D.P. Gannon, R.A. Hazelkorn, G. Lovewell, K.M. Volker, S. Smith, M.C. Tumlin, and J. Litz. 2021. Diet of common bottlenose dolphins, *Tursiops truncatus*, that stranded in and near Barataria Bay, Louisiana, 2010–2012. Southeastern Naturalist 20(1). doi: 10.1656/058.020.0113

Bräger, S. 1993. Diurnal and seasonal behavior patterns of bottlenose dolphins (*Tursiops truncatus*). Marine Mammal Science 9:434–438. doi: 10.1111/j.1748-7692.1993.tb00477.x

Bräger, S., B. Würsig, A. Acevedo, and T. Henningsen. 1994. Association patterns of bottle-nosed dolphins (*Tursiops-truncatus*) in Galveston Bay, Texas. Journal of Mammalogy 75(2):431–437. doi: 10.2307/1382564

Bryan, C.E., S.J. Christopher, B.C. Balmer, and R.S. Wells. 2007. Establishing baseline levels of trace elements in blood and skin of bottlenose dolphins in Sarasota Bay, Florida: implications for non-invasive monitoring. Science of the Total Environment 388(1-3):325–342. doi: 10.1016/j.scitotenv.2007.07.046

Cloyed, C.S., R.M. Wilson, B.C. Balmer, A.A. Hohn, L.H. Schwacke, E.S. Zolman, M.C. Tumlin, R.S. Wells, A.A. Barleycorn, J.B. Allen, and R.H. Carmichael. 2021. Specialization of a mobile, apex predator affects trophic coupling among adjacent habitats. Scientific Reports 11(1):19611. doi: 10.1038/s41598-021-99017-1

Connor, R.C., R.S. Wells, J. Mann, and A.J. Read. 2000. The bottlenose dolphin: Social relationships in a fission-fusion society. In: Mann, J., R. Connor, P. Tyack, and H. Whitehead (Eds.), Cetacean Societies: Field Studies of Dolphins and Whales. University of Chicago Press, Chicago, IL. pp. 91–126.

Correa, L., J.M. Castellini, R.S. Wells, and T. O'Hara. 2013. Distribution of mercury and selenium in blood compartments of bottlenose dolphins (*Tursiops truncatus*) from Sarasota Bay, Florida. Environmental Toxicology and Chemistry 32(11):2441–2448. doi: 10.1002/etc.2327

De Guise, S., M. Levin, E. Gebhard, L. Jasperse, L. Burdett Hart, C.R. Smith, S. Venn-Watson, F. Townsend, R. Wells, B. Balmer, E. Zolman, T. Rowles, and L. Schwacke. 2017. Changes in immune functions in bottlenose dolphins in the northern Gulf of Mexico associated with the Deepwater Horizon oil spill. Endangered Species Research 33:291–303. doi: 10.3354/esr00814

De Guise, S., M. Levin, L. Jasperse, J. Herrman, R.S. Wells, T. Rowles, and L. Schwacke. 2021. Long-term immunological alterations in bottlenose dolphin a decade after the Deepwater Horizon oil spill in the northern Gulf of Mexico: Potential for multigenerational effects. Environmental Toxicology and Chemistry 40(5):1308–1321. doi: 10.1002/etc.4980

Deepwater Horizon Natural Resource Damage Assessment Trustees (DWH NRDA). 2016. Deepwater Horizon oil spill: Final Programmatic Damage Assessment and Restoration Plan and Final Programmatic Environmental Impact Statement. Retrieved from: http://www.gulfspillrestoration.noaa.gov/restoration-planning/gulf-plan.1659.

Fauquier, D.A., J. Litz, S. Sanchez, K. Colegrove, L.H. Schwacke, L. Hart, J. Saliki, C. Smith, T. Goldstein, S. Bowen-Stevens, W. McFee, E. Fougeres, B. Mase-Guthrie, E. Stratton, R. Ewing, S. Venn-Watson, R.H. Carmichael, C. Clemons-Chevis, W. Hatchett, D. Shannon, S. Shippee, S. Smith, L. Staggs, M.C. Tumlin, N.L. Wingers, and T.K. Rowles. 2017. Evaluation of morbillivirus exposure in cetaceans from the northern Gulf of Mexico 2010-2014. Endangered Species Research 33:211–220. doi: 10.3354/esr00772

Fernandez, S. and A.A. Hohn. 1998. Age, growth, and calving season of bottlenose dolphins, *Tursiops truncatus*, off coastal Texas. Fishery Bulletin 96(2):357–365.

Fertl, D. 1994. Occurrence patterns and behavior of bottlenose dolphins (*Tursiops truncatus*) in the Galveston ship channel, Texas. Texas Journal of Science 46(4):299–318.

Hayes, S.A., E. Josephson, K. Maze-Foley, and P.E. Rosel. 2020. US Atlantic and Gulf of Mexico marine mammal stock assessments - 2019. National Oceanic and Atmospheric Administration, National Marine Fisheries Service, Northeast Fisheries Science Center: Woods Hole, MA. NOAA Technical Memorandum NMFS-NE-264. 479 p.

Hayes, S.A., E. Josephson, K. Maze-Foley, P.E. Rosel, and J. Wallace. 2022. U.S. Atlantic and Gulf of Mexico marine mammal stock assessments 2021. National Oceanic and Atmospheric Administration, National Marine Fisheries Service, Northeast Fisheries Science Center: Woods Hole, MA. NOAA Technical Memorandum NMFS-NE-288. 380 p. doi: 10.25923/6tt7-kc16

Helm, R.C., D.P. Costa, T.D. DeBruyn, T.J. O'Shea, R.S. Wells, and T.M. Williams. 2015. Overview of effects of oil spills on marine mammals. In: Fingas, M. (Ed.), Handbook of oil spill science and technology, First edition. John Wiley & Sons, Inc., Hoboken, NJ. pp. 455–475.

Henderson, E.E. and B. Würsig. 2007. Behavior patterns of bottlenose dolphins in San Luis Pass, Texas. Gulf of Mexico Science 25(2):153–161. doi: 10.18785/goms.2502.06

Hohn, A.A., M.D. Scott, R.S. Wells, J.C. Sweeney, and A.B. Irvine. 1989. Growth layers in teeth from known-age, free-ranging bottlenose dolphins. Marine Mammal Science 5(4):315–342. doi: 10.1111/j.1748-7692.1989.tb00346.x

Hubard, C.W., K. Maze-Foley, K.D. Mullin, and W.W. Schroeder. 2004. Seasonal abundance and site fidelity of bottlenose dolphins (*Tursiops truncatus*) in Mississippi Sound. Aquatic Mammals 30(2):299–310. doi: 10.1578/am.30.2.2004.299

Irvine, A.B., M.D. Scott, R.S. Wells, and J.H. Kaufmann. 1981. Movements and activities of the Atlantic bottlenose dolphin, *Tursiops truncatus*, near Sarasota, Florida. Fishery Bulletin 79(4):671–688.

Irvine, B. and R.S. Wells. 1972. Results of attempts to tag Atlantic bottlenosed dolphins (*Tursiops truncatus*). Cetology 13:1–5.

Irwin, L.-J. and B. Würsig. 2004. A small resident community of bottlenose dolphins, *Tursiops truncatus*, in Texas: Monitoring recommendations. Gulf of Mexico Science 22(1):13–21. doi: 10.18785/goms.2201.02

Kellar, N.M., T.R. Speakman, C.R. Smith, S.M. Lane, B.C. Balmer, M.L. Trego, K.N. Catelani, M.N. Robbins, C.D. Allen, R.S. Wells, E.S. Zolman, T.K. Rowles, and L.H. Schwacke. 2017. Low reproductive success rates of common bottlenose dolphins *Tursiops truncatus* in the northern Gulf of Mexico following the Deepwater Horizon disaster (2010-2015). Endangered Species Research 33:143–158. doi: 10.3354/esr00775

Kucklick, J., L. Schwacke, R. Wells, A. Hohn, A. Guichard, J. Yordy, L. Hansen, E. Zolman, R. Wilson, J. Litz, D. Nowacek, T. Rowles, R. Pugh, B. Balmer, C. Sinclair, and P. Rosel. 2011. Bottlenose dolphins as indicators of persistent organic pollutants in the western North Atlantic Ocean and Northern

Gulf of Mexico. Environmental Science & Technology 45(10):4270–4277. doi: 10.1021/es1042244

LaBrecque, E., C. Curtice, J. Harrison, S.M. Van Parijs, and P.N. Halpin. 2015. 3. Biologically Important Areas for cetaceans within U.S. Waters – Gulf of Mexico Region. Aquatic Mammals 41(1):30–38. doi: 10.1578/am.41.1.2015.30

Lane, S.M., C.R. Smith, J. Mitchell, B.C. Balmer, K.P. Barry, T. McDonald, C.S. Mori, P.E. Rosel, T.K. Rowles, T.R. Speakman, F.I. Townsend, M.C. Tumlin, R.S. Wells, E.S. Zolman, and L.H. Schwacke. 2015. Reproductive outcome and survival of common bottlenose dolphins sampled in Barataria Bay, Louisiana, USA, following the Deepwater Horizon oil spill. Proceedings of the Royal Society B: Biological Sciences 282(1818):20151944. doi: 10.1098/rspb.2015.1944

Leatherwood, S. 1977. Some preliminary impressions on the numbers and social behavior of free-swimming bottlenosed dolphin calves (*Tursiops truncatus*) in the northern Gulf of Mexico. In: Ridgway, S.H. and K. Bernirschke (Eds.), Breeding Dolphins; Present Status, Suggestions for the Future. Final report to the US Marine Mammal Commission, Contract MM6AC009, MMC-76/07, Washington, DC. pp. 143–167.

Lipscomb, T.P., S. Kennedy, D. Moffett, A. Krafft, B.A. Klaunberg, J.H. Lichy, G.T. Regan, G.A.J. Worthy, and J.K. Taubenberger. 1996. Morbilliviral epizootic in bottlenose dolphins of the Gulf of Mexico. Journal of Veterinary Diagnostic Investigation 8(3):283–290. doi: 10.1177/104063879600800302

Litz, J.A., M.A. Baran, S.R. Bowen-Stevens, R.H. Carmichael, K.M. Colegrove, L.P. Garrison, S.E. Fire, E.M. Fougeres, R. Hardy, S. Holmes, W. Jones, B.E. Mase-Guthrie, D.K. Odell, P.E. Rosel, J.T. Saliki, D.K. Shannon, S.F. Shippee, S.M. Smith, E.M. Stratton, M.C. Tumlin, H.R. Whitehead, G.A. Worthy, and T.K. Rowles. 2014. Review of historical unusual mortality events (UMEs) in the Gulf of Mexico (1990–2009): providing context for the multi-year northern Gulf of Mexico cetacean UME declared in 2010. Diseases of Aquatic Organisms 112(2):161–175. doi: 10.3354/dao02807

Lynn, S.K. and B. Würsig. 2002. Summer movement patterns of bottlenose dolphins in a Texas bay. Gulf of Mexico Science 20(1):25–37. doi: 10.18785/goms.2001.03

Mackey, A.D. 2010. Site fidelity and association patterns of bottlenose dolphins (*Tursiops truncatus*) in the Mississippi Sound. University of Southern Mississippi. Thesis. 133 p.

Mann, J., R.C. Connor, L.M. Barre, and M.R. Heithaus. 2000. Female reproductive success in bottlenose dolphins (*Tursiops* sp.): life history, habitat, provisioning, and group-size effects. Behavioral Ecology 11(2):210–219. doi: 10.1093/beheco/11.2.210

Mattson, M.C., K.D. Mullin, G.W. Ingram, and W. Hoggard. 2006. Age structure and growth of the bottlenose dolphin (*Tursiops truncatus*) from strandings in the Mississippi sound region of the north-central Gulf of Mexico from 1986 to 2003. Marine Mammal Science 22(3):654–666. doi: 10.1111/j.1748-7692.2006.00057.x

Maze, K.S. and B. Würsig. 1999. Bottlenosed dolphins of San Luis Pass, Texas: Occurrence patterns, site-fidelity, and habitat use. Aquatic Mammals 25:91–104.

McCormack, M.A., F. Battaglia, W.E. McFee, and J. Dutton. 2020a. Mercury concentrations in blubber and skin from stranded bottlenose dolphins (*Tursiops truncatus*) along the Florida and Louisiana coasts (Gulf of Mexico, USA) in relation to biological variables. Environmental Research 180:108886. doi: 10.1016/j.envres.2019.108886

McCormack, M.A., B.P. Jackson, and J. Dutton. 2020b. Relationship between mercury and selenium concentrations in tissues from stranded odontocetes in the northern Gulf of Mexico. Science of the Total Environment 749:141350. doi: 10.1016/j.scitotenv.2020.141350

McCormack, M.A., W.H. Nowlin, and J. Dutton. 2022. Effect of trophic position on mercury concentrations in bottlenose dolphins (*Tursiops truncatus*) from the northern Gulf of Mexico. Environmental Research 204(Pt B):112124. doi: 10.1016/j.envres.2021.112124

McDonald, T.L., F.E. Hornsby, T.R. Speakman, E.S. Zolman, K.D. Mullin, C. Sinclair, P.E. Rosel, L. Thomas, and L.H. Schwacke. 2017. Survival, density, and abundance of common bottlenose dolphins in Barataria Bay (USA) following the Deepwater Horizon oil spill. Endangered Species Research 33:193–209. doi: 10.3354/esr00806

McHugh, M.B. 1989. Population numbers and feeding behavior of the Atlantic bottlenose dolphin (*Tursiops truncatus*) near Aransas Pass, Texas. University of Texas at Austin. Thesis. 96 p.

Mead, J.G. and C.W. Potter. 1990. Natural history of bottlenose dolphins along the central Atlantic coast of the United States. In: Leatherwood, S. and R.R. Reeves (Eds.), The Bottlenose Dolphin. Academic Press, San Diego, CA. pp. 165–195.

Michel, J., E.H. Owens, S. Zengel, A. Graham, Z. Nixon, T. Allard, W. Holton, P.D. Reimer, A. Lamarche, M. White, N. Rutherford, C. Childs, G. Mauseth, G. Challenger, and E. Taylor. 2013. Extent and degree of shoreline oiling: Deepwater Horizon oil spill, Gulf of Mexico, USA. PLoS ONE 8(6):e65087. doi: 10.1371/journal.pone.0065087

Miller, C.E. 2003. Abundance trends and environmental habitat usage patterns of bottlenose dolphins (*Tursiops truncatus*) in lower Barataria and Caminada bays, Louisiana. Louisiana State University and Agricultural & Mechanical College. Dissertation. 125 p.

Miller, D.L., V. Woshner, E.L. Styer, S. Ferguson, K.K. Knott, M.J. Gray, R.S. Wells, and T.M. O'Hara. 2011. Histologic findings in free-ranging Sarasota Bay bottlenose dolphin (*Tursiops truncatus*) skin: mercury, selenium, and seasonal factors. Journal of Wildlife Diseases 47(4):1012–1018. doi: 10.7589/0090-3558-47.4.1012

Miller, L.J., A.D. Mackey, T. Hoffland, M. Solangi, and S.A. Kuczaj. 2010. Potential effects of a major hurricane on Atlantic bottlenose dolphin (*Tursiops truncatus*) reproduction in the Mississippi Sound. Marine Mammal Science 26(3):707–715. doi: 10.1111/j.1748-7692.2010.00371.x

Miller, L.J., A.D. Mackey, M. Solangi, and S.A. Kuczaj. 2013. Population abundance and habitat utilization of bottlenose dolphins in the Mississippi Sound. Aquatic Conservation: Marine and Freshwater Ecosystems 23(1):145–151. doi: 10.1002/aqc.2278

Mullin, K.D., T. McDonald, R.S. Wells, B.C. Balmer, T. Speakman, C. Sinclair, E.S. Zolman, F. Hornsby, S.M. McBride, K.A. Wilkinson, and L.H. Schwacke. 2017. Density, abundance, survival, and ranging patterns of common bottlenose dolphins (*Tursiops truncatus*) in Mississippi Sound following the Deepwater Horizon oil spill. PLoS ONE 12(10):e0186265. doi: 10.1371/journal.pone.0186265

National Oceanic and Atmospheric Administration (NOAA). 2011. Public scoping for preparation of a programmatic environmental impact statement for the Deepwater Horizon BP Oil Spill. 9 p.

Perrin, W.F. and S.B. Reilly. 1984. Reproductive parameters of dolphins and small whales of the family Delphinidae. In: Perrin, W.F., R.L. Brownell, Jr., and D.P. DeMaster (Eds.), Reproduction in Whales, Dolphins, and Porpoises. pp. 97–134.

Rosel, P.E., L.A. Wilcox, C. Sinclair, T.R. Speakman, M.C. Tumlin, J.A. Litz, and E.S. Zolman. 2017. Genetic assignment to stock of stranded common bottlenose dolphins in southeastern Louisiana after the Deepwater Horizon oil spill. Endangered Species Research 33:221–234. doi: 10.3354/esr00780

Rossman, S., P.H. Ostrom, M. Stolen, N.B. Barros, H. Gandhi, C.A. Stricker, and R.S. Wells. 2015. Individual specialization in the foraging habits of female bottlenose dolphins living in a trophically diverse and habitat rich estuary. Oecologia 178(2):415–425. doi: 10.1007/s00442-015-3241-6

Schwacke, L.H., C.R. Smith, F.I. Townsend, R.S. Wells, L.B. Hart, B.C. Balmer, T.K. Collier, S. De Guise, M.M. Fry, L.J. Guillette, Jr., S.V. Lamb, S.M. Lane, W.E. McFee, N.J. Place, M.C. Tumlin, G.M. Ylitalo, E.S. Zolman, and T.K. Rowles. 2014. Health of common bottlenose dolphins (*Tursiops truncatus*) in Barataria Bay, Louisiana, following the deepwater horizon oil spill. Environmental Science & Technology 48(1):93–103. doi: 10.1021/es403610f

Schwacke, L.H., L. Thomas, R.S. Wells, W.E. McFee, A.A. Hohn, K.D. Mullin, E.S. Zolman, B.M. Quigley, T.K. Rowles, and J.H. Schwacke. 2017. Quantifying injury to common bottlenose dolphins from the Deepwater Horizon oil spill using an age-, sex- and class-structured population model. Endangered Species Research 33:265–279. doi: 10.3354/esr00777

Schwacke, L.H., M.J. Twiner, S. De Guise, B.C. Balmer, R.S. Wells, F.I. Townsend, D.C. Rotstein, R.A. Varela, L.J. Hansen, E.S. Zolman, T.R. Spradlin, M. Levin, H. Leibrecht, Z. Wang, and T.K. Rowles. 2010. Eosinophilia and biotoxin exposure in bottlenose dolphins (*Tursiops truncatus*) from a coastal area impacted by repeated mortality events. Environmental Research 110(6):548–555. doi: 10.1016/j.envres.2010.05.003

Scott, M.D. and S.J. Chivers. 1990. Distribution and herd structure of bottlenose dolphins in the eastern tropical Pacific Ocean. In: Leatherwood, S. and R.R. Reeves (Eds.), The Bottlenose Dolphin. Academic Press, San Diego, CA. pp. 387–402.

Sellas, A.B., R.S. Wells, and P.E. Rosel. 2005. Mitochondrial and nuclear DNA analyses reveal fine scale geographic structure in bottlenose dolphins (*Tursiops truncatus*) in the Gulf of Mexico. Conservation Genetics 6(5):715–728. doi: 10.1007/s10592-005-9031-7

Sergeant, D.E., D.K. Caldwell, and M.C. Caldwell. 1973. Age, growth, and maturity of bottlenosed dolphin (*Tursiops-truncatus*) from northeast Florida. Journal of the Fisheries Research Board of Canada 30(7):1009–1011. doi: 10.1139/f73-165

Shane, S.H. 1990. Behavior and ecology of the bottlenose dolphin at Sanibel Island, Florida. In: Leatherwood, S. and R.R. Reeves (Eds.), The Bottlenose Dolphin. Academic Press, San Diego, CA. pp. 245–265. doi: 10.1016/B978-0-12-440280-5.50016-0

Shane, S.H. 2004. Residence patterns, group characteristics, and association patterns of bottlenose dolphins near Sanibel Island, Florida. Gulf of Mexico Science 22(1):1–12. doi: 10.18785/goms.2201.01

Shane, S.H., R.S. Wells, and B. Würsig. 1986. Ecology, behavior and social organization of the bottlenose dolphin: A review. Marine Mammal Science 2:34–63. doi: 10.1111/j.1748-7692.1986.tb00026.x

Smith, C.R., T.K. Rowles, L.B. Hart, F.I. Townsend, R.S. Wells, E.S. Zolman, B.C. Balmer, B. Quigley, M. Ivančić, W. McKercher, M.C. Tumlin, K.D. Mullin, J.D. Adams, Q. Wu, W. McFee, T.K. Collier, and L.H. Schwacke. 2017. Slow recovery of Barataria Bay dolphin health following the Deepwater Horizon oil spill (2013-2014), with evidence of persistent lung disease and impaired stress response. Endangered Species Research 33:127–142. doi: 10.3354/esr00778

Takeshita, R., B.C. Balmer, F. Messina, E.S. Zolman, L. Thomas, R.S. Wells, C.R. Smith, T.K. Rowles, and L.H. Schwacke. 2021. High site-fidelity in common bottlenose dolphins despite low salinity exposure and associated indicators of compromised health. PLoS ONE 16(9):e0258031. doi: 10.1371/journal.pone.0258031

Taylor, B.L., S.J. Chivers, J. Larese, and W.F. Perrin. 2007. Generation length and percent mature estimates for IUCN assessments of cetaceans. National Oceanic and Atmospheric Administration, National Marine Fisheries Service, Southwest Fisheries Science Center: La Jolla, CA. Administrative Report LJ-07-01. 24 p.

Toms, C. 2019. Filling the gaps: Common bottlenose dolphin (*Tursiops truncatus*) population dynamics, structure, and connectivity within Florida panhandle bays, sounds, and estuaries. University of Central Florida. Dissertation. 373 p.

Torres, L.G. and A.J. Read. 2009. Where to catch a fish? The influence of foraging tactics on the ecology of bottlenose dolphins (*Tursiops truncatus*) in Florida Bay, Florida. Marine Mammal Science 25(4):797–815. doi: 10.1111/j.1748-7692.2009.00297.x

Twiner, M.J., L.J. Flewelling, S.E. Fire, S.R. Bowen-Stevens, J.K. Gaydos, C.K. Johnson, J.H. Landsberg, T.A. Leighfield, B. Mase-Guthrie, L. Schwacke, F.M. Van Dolah, Z. Wang, and T.K. Rowles. 2012. Comparative analysis of three brevetoxin-associated bottlenose dolphin (*Tursiops truncatus*)

mortality events in the Florida Panhandle region (USA). PLoS ONE 7(8):e42974. doi: 10.1371/journal.pone.0042974
Tyson, R.B., S.M. Nowacek, and D.P. Nowacek. 2011. Community structure and abundance of bottlenose dolphins *Tursiops truncatus* in coastal waters of the northeast Gulf of Mexico. Marine Ecology Progress Series 438:253–265. doi: 10.3354/meps09292
Urian, K.W., S. Hofmann, R.S. Wells, and A.J. Read. 2009. Fine-scale population structure of bottlenose dolphins (*Tursiops truncatus*) in Tampa Bay, Florida. Marine Mammal Science 25(3):619–638. doi: 10.1111/j.1748-7692.2009.00284.x
Van Bressem, M.F., P.J. Duignan, A. Banyard, M. Barbieri, K.M. Colegrove, S. De Guise, G. Di Guardo, A. Dobson, M. Domingo, D. Fauquier, A. Fernandez, T. Goldstein, B. Grenfell, K.R. Groch, F. Gulland, B.A. Jensen, P.D. Jepson, A. Hall, T. Kuiken, S. Mazzariol, S.E. Morris, O. Nielsen, J.A. Raga, T.K. Rowles, J. Saliki, E. Sierra, N. Stephens, B. Stone, I. Tomo, J. Wang, T. Waltzek, and J.F. Wellehan. 2014. Cetacean morbillivirus: current knowledge and future directions. Viruses 6(12):5145–5181. doi: 10.3390/v6125145
Venn-Watson, S., L. Garrison, J. Litz, E. Fougeres, B. Mase, G. Rappucci, E. Stratton, R. Carmichael, D. Odell, D. Shannon, S. Shippee, S. Smith, L. Staggs, M. Tumlin, H. Whitehead, and T. Rowles. 2015. Demographic clusters identified within the northern Gulf of Mexico common bottlenose dolphin (*Tursiops truncates*) unusual mortality event: January 2010-June 2013. PLoS ONE 10(2):e0117248. doi: 10.1371/journal.pone.0117248
Vollmer, N.L. and P.E. Rosel. 2013. A review of common bottlenose dolphins (*Tursiops truncatus truncatus*) in the Northern Gulf of Mexico: Population biology, potential threats, and management. Southeastern Naturalist 12(m6):1–43. doi: 10.1656/058.012.m601
Weller, D.W. 1998. Global and regional variation in the biology and behavior of bottlenose dolphins. Texas A&M University. Dissertation. 142 p.
Wells, R.S. 1986a. Population structure of bottlenose dolphins: Behavioral studies along the central west coast of Florida. Contract Report to National Marine Fisheries Service, Southeast Fisheries Center. Contract No. 45-WCNF-5-003BB. 58 p.
Wells, R.S. 1986b. Structural aspects of dolphin societies. University of California, Santa Cruz. Dissertation. 234 p.
Wells, R.S. 1991. The role of long-term study in understanding the social structure of a bottlenose dolphin community. In: Pryor, K. and K. Norris (Eds.), Dolphin Societies: Discoveries and Puzzles. University of California Press, Berkeley, CA. pp. 199–226.
Wells, R.S. 2000. Reproduction in wild bottlenose dolphins: Overview of patterns observed during a long-term study. In: Duffield, D. and T. Robeck (Eds.), Bottlenose Dolphin Reproduction Workshop. AZA Marine Mammal Taxon Advisory Group, Silver Spring, MD. pp. 57–74.
Wells, R.S. 2003. Dolphin social complexity: Lessons from long-term study and life history. In: de Waal, F.B.M. and P.L. Tyack (Eds.), Animal Social Complexity: Intelligence, Culture, and Individualized Societies Harvard University Press, Cambridge, MA. pp. 32–56.
Wells, R.S. 2014. Social structure and life history of bottlenose dolphins near Sarasota Bay, Florida: Insights from four decades and five generations. Primates and cetaceans. pp. 149–172. doi: 10.1007/978-4-431-54523-1_8
Wells, R.S., M.K. Bassos, K.W. Urian, W.J. Carr, and M.D. Scott. 1996. Low-level monitoring of bottlenose dolphins, *Tursiops truncatus*, in Charlotte Harbor, Florida: 1990-1994. National Oceanic and Atmospheric Administration, National Marine Fisheries Service, Southeast Fisheries Science Center: Miami, FL. NOAA Technical Memorandum NMFS-SEFSC-384. 86 p.
Wells, R.S., M.K. Bassos, K.W. Urian, S.H. Shane, E.C.G. Owen, C.F. Weiss, W.J. Carr, and M.D. Scott. 1997. Low-level monitoring of bottlenose dolphins, *Tursiops truncatus*, in Pine Island Sound, Florida: 1996. National Oceanic and Atmospheric Administration, National Marine Fisheries

Service, Southeast Fisheries Science Center: Miami, FL. Southeast Fisheries Center Contribution No. 40-WCNF601958. 86 p.
Wells, R.S., A. Natoli, and G. Braulik. 2019. *Tursiops truncatus* (errata version published in 2019). *The IUCN Red List of Threatened Species* 2019: e.T22563A156932432. https://dx.doi.org/10.2305/IUCN.UK.2019-1.RLTS.T22563A156932432.en
Wells, R.S., L.H. Schwacke, T.K. Rowles, B.C. Balmer, E. Zolman, T. Speakman, F.I. Townsend, M.C. Tumlin, A. Barleycorn, and K.A. Wilkinson. 2017. Ranging patterns of common bottlenose dolphins *Tursiops truncatus* in Barataria Bay, Louisiana, following the Deepwater Horizon oil spill. Endangered Species Research 33:159–180. doi: 10.3354/esr00732
Wells, R.S. and M.D. Scott. 1999. Bottlenose dolphin *Tursiops truncatus* (Montagu, 1821). In: Ridgway, S.H. and R. Harrison (Eds.), Handbook of marine mammals. Volume 6: The second book of dolphins and the porpoises. Academic Press, San Diego, CA. pp. 137–182.
Wells, R.S. and M.D. Scott. 2018. Bottlenose dolphin, *Tursiops truncatus*, common bottlenose dolphin. In: Würsig, B., J.G.M. Thewissen, and K.M. Kovacs (Eds.), Encyclopedia of Marine Mammals (Third Edition). Academic Press, London, UK. pp. 118–125. doi: 10.1016/B978-0-12-804327-1.00072-8
Wells, R.S., M.D. Scott, and A.B. Irvine. 1987. The social structure of free-ranging bottlenose dolphins. In: Genoways, H.H. (Ed.), Current Mammalogy. Plenum Press, New York, NY. pp. 247–305.
Wells, R.S., K.W. Urian, A.J. Read, M.K. Bassos, W.J. Carr, and M.D. Scott. 1996. Low-level monitoring of bottlenose dolphins, *Tursiops truncatus*, in Tampa Bay, Florida: 1988-1993. National Oceanic and Atmospheric Administration, National Marine Fisheries Service, Southeast Fisheries Science Center: Miami, FL. NOAA Technical Memorandum NMFS-SEFSC-385. 36 p.
Wilson, R.M., R.B. Tyson, J.A. Nelson, B.C. Balmer, J.P. Chanton, and D.P. Nowacek. 2017. Niche differentiation and prey selectivity among common bottlenose dolphins (*Tursiops truncatus*) sighted in St. George Sound, Gulf of Mexico. Frontiers in Marine Science 4:235. doi: 10.3389/fmars.2017.00235
Woshner, V., K. Knott, R. Wells, C. Willetto, R. Swor, and T. O'Hara. 2008. Mercury and selenium in blood and epidermis of bottlenose dolphins (*Tursiops truncatus*) from Sarasota Bay, FL: interaction and relevance to life history and hematologic parameters. Ecohealth 5(3):360–370. doi: 10.1007/s10393-008-0164-2
Yordy, J.E., M.A. Mollenhauer, R.M. Wilson, R.S. Wells, A. Hohn, J. Sweeney, L.H. Schwacke, T.K. Rowles, J.R. Kucklick, and M.M. Peden-Adams. 2010a. Complex contaminant exposure in cetaceans: a comparative E-Screen analysis of bottlenose dolphin blubber and mixtures of four persistent organic pollutants. Environmental Toxicology and Chemistry 29(10):2143–2153. doi: 10.1002/etc.279
Yordy, J.E., D.A. Pabst, W.A. McLellan, R.S. Wells, T.K. Rowles, and J.R. Kucklick. 2010b. Tissue-specific distribution and whole-body burden estimates of persistent organic pollutants in the bottlenose dolphin (*Tursiops truncatus*). Environmental Toxicology and Chemistry 29(6):1263–1273. doi: 10.1002/etc.152
Yordy, J.E., R.S. Wells, B.C. Balmer, L.H. Schwacke, T.K. Rowles, and J.R. Kucklick. 2010c. Life history as a source of variation for persistent organic pollutant (POP) patterns in a community of common bottlenose dolphins (*Tursiops truncatus*) resident to Sarasota Bay, FL. Science of the Total Environment 408(9):2163–2172. doi: 10.1016/j.scitotenv.2010.01.032

Common bottlenose dolphin − *Tursiops truncatus*

GoMx BSE/St. Joseph Sound, Clearwater Harbor Stock

Overall Vulnerability Rank = Very High

Biological Sensitivity = Very High

Climate Exposure = Very High

Data Quality = 93% of scores ≥ 2

	Tursiops truncatus	Expert Scores	Data Quality	Expert Scores Plots (Portion by Category)
Sensitivity attributes	Prey/Diet Specificity	1.9	2.2	
	Habitat Specificity	3.7	2.2	
	Site Fidelity	3.8	2.0	
	Lifetime Reproductive Potential	1.5	2.2	
	Generation Length	2.9	2.2	
	Reproductive Plasticity	1.4	2.2	
	Migration	3.8	2.2	
	Home Range	3.5	2.2	
	Stock Abundance	3.1	1.5	
	Stock Abundance Trend	2.2	1.5	
	Cumulative Stressors	3.5	2.2	
	Sensitivity Score	Very High		
Exposure variables	Sea Surface Temperature (Change in mean)	4.0	2.5	
	Sea Surface Temperature (Change in variability)	1.0	2.5	
	Air Temperature (Change in mean)	4.0	2.5	
	Air Temperature (Change in variability)	1.0	2.5	
	Precipitation (Change in mean)	1.0	2.5	
	Precipitation (Change in variability)	1.0	2.5	
	Sea Surface Salinity (Change in mean)	1.0	2.5	
	Sea Surface Salinity (Change in variability)	2.0	2.5	
	Ocean pH (Change in mean)	4.0	2.5	
	Ocean pH (Change in variability)	1.0	2.5	
	Sea ice coverage (Change in mean)	1.0	2.3	
	Sea ice coverage (Change in variability)	1.0	2.3	
	Dissolved oxygen (Change in mean)	4.0	2.5	
	Dissolved oxygen (Change in variability)	1.0	2.5	
	Circulation	3.9	2.2	
	Sea level rise	4.0	2.5	
	Exposure Score	Very High		
	Overall Vulnerability Rank	Very High		

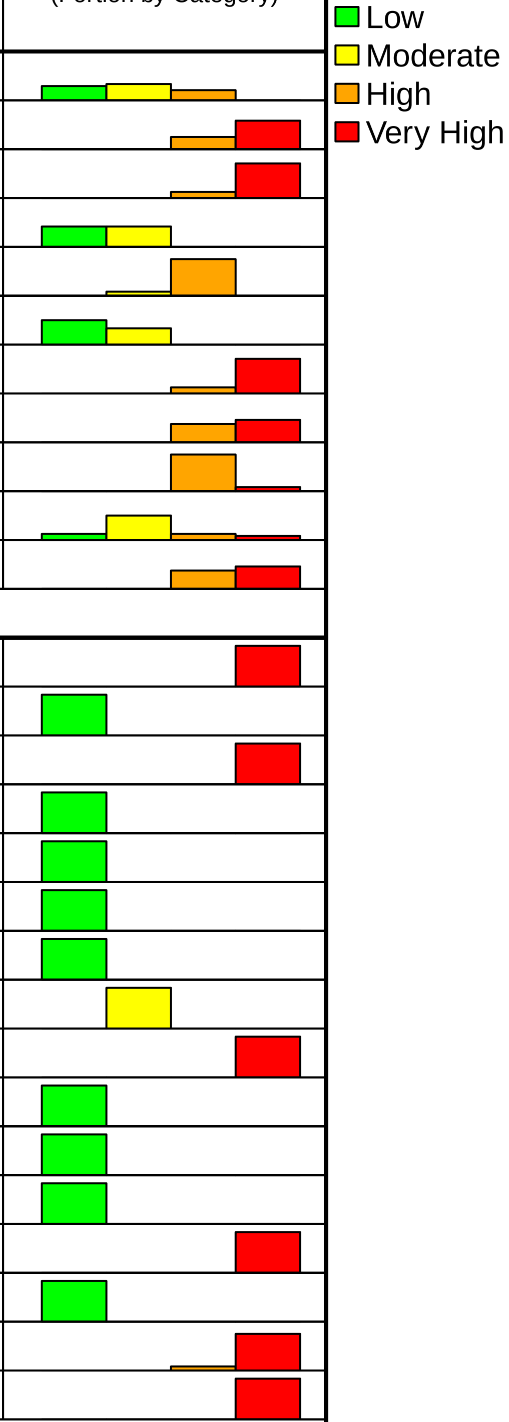

Common bottlenose dolphin (GoMx BSE/St. Joseph Sound, Clearwater Harbor Stock)

Tursiops truncatus

CVA Results Summary

Overall Climate Vulnerability Rank: Very High (100% certainty from bootstrap analysis).

Climate Exposure: Very High. Six exposure factors scored greater than 3.5: Air Temperature (Standard anomaly) (4.0), Dissolved oxygen (Standard anomaly) (4.0), Ocean pH (Standard anomaly) (4.0), Sea level rise (4.0), Sea Surface Temperature (Standard anomaly) (4.0), and Circulation (3.9).

Biological Sensitivity: Very High. Five sensitivity attributes scored greater than 3.5: Migration (3.85), Site Fidelity (3.85), Habitat Specificity (3.70), Cumulative Stressors (3.55), and Home Range (3.55).

Distributional Response: Very High

Abundance Response: Very High

Phenology Response: Very High

Data Quality: 93% of the data quality scores were 2 or greater, 82% of sensitivity attribute data quality scores were 2 or higher.

Background Information

Stock Description

This stock includes common bottlenose dolphins in St. Joseph Sound and Clearwater Harbor in the eastern Gulf of Mexico along the Florida coast (Hayes et al. 2019).

Prey/Diet Specificity

Generally, common bottlenose dolphins consume a large variety of fish and/or squid (Barros and Odell 1990; Mead and Potter 1990; Wells and Scott 2018). Common bottlenose dolphins seem to show a consistent preference for sound-producing fishes (e.g., sciaenids, toadfish; Berens McCabe et al. 2010; Bowen-Stevens et al. 2021). In Sarasota Bay, Florida, Rossman et al. (2015) found some individual common bottlenose dolphins to show diet specialization despite the broader study group showing a generalist diet. Specialization can be influenced by locally abundant prey species (Shane et al. 1986; Vollmer and Rosel 2013) or can result from resource partitioning amongst sympatric populations (e.g., Torres and Read 2009; Wilson et al. 2017).

Habitat Specificity

Common bottlenose dolphins are found in a variety of marine and estuarine habitats (Wells and Scott 2018).

Site Fidelity

No specific site fidelity information was found for this stock. However, common bottlenose dolphins in other Gulf of Mexico bay, sound, and estuary (BSE) stocks show site fidelity (LaBrecque et al. 2015; Hayes et al. 2022), such as in Charlotte Harbor and Pine Island Sound (Bassos-Hull et al. 2013), Sarasota Bay (Wells 1986a; Wells 1991; Wells 2014), St. Andrew Bay (Balmer et al. 2019), St. Joseph Bay (Balmer et al. 2008; B. Balmer et al. 2018), St. Vincent Sound and Apalachicola Bay (Tyson et al. 2011), and Tampa Bay in Florida (Wells et al. 1996b; Urian et al. 2009); Matagorda Bay, Espiritu Santo Bay, and San Luis Pass in Texas (Maze and Würsig 1999; Lynn and Würsig 2002; Irwin and Würsig 2004); Barataria Bay in Louisiana (Miller 2003; McDonald et al. 2017; Wells et al. 2017; Takeshita et al. 2021); and Mississippi Sound in Mississippi/Louisiana (Hubard et al. 2004; Mackey 2010; Mullin et al. 2017).

Lifetime Reproductive Potential

Taylor et al. (2007) reported the interbirth interval of common bottlenose dolphins as 3.80 years, though other studies have shown it to be closer to three years (Leatherwood 1977; Wells and Scott 1999; Connor et al. 2000; Wells 2003).

Generally, females reach sexual maturity before males, with females reaching sexual maturity at 5–13 years (Sergeant et al. 1973; Perrin and Reilly 1984; Wells et al. 1987; Mead and Potter 1990; Mann et al. 2000; Wells 2000; Wells and Scott 2018).

Females reproduce through most of their adult lives (Leatherwood 1977; Hohn et al. 1989; Wells and Scott 1999; Wells 2000; Wells 2003), but may produce fewer calves after they reach 30 years of age (Wells 2000). Sarasota Bay common bottlenose dolphins can give birth when they are as young as 6 years of age, and can continue until they are 48 years old (Wells and Scott 2018). Reproductive lifespan would therefore be up to 42 years based on long-term observation studies (Wells 2014; Wells and Scott 2018).

Longitudinal studies from Sarasota Bay, Florida have found that female common bottlenose dolphins can live up to 67 years (Wells and Scott 2018). Studies based on growth layer groups in teeth suggest female common bottlenose dolphins can live to 41 years along the Texas coast (Fernandez and Hohn 1998) and to 30 years along the Mississippi coast (Mattson et al. 2006).

Generation Length

Taylor et al. (2007) reported a common bottlenose dolphin generation length of 20.6–21.1 years.

Reproductive Plasticity

Common bottlenose dolphin calving is generally diffusely seasonal, with one or more peaks, although births have been reported from all seasons (Vollmer and Rosel 2013; Wells and Scott 2018). Calf sightings peak in the spring and summer months in Florida (Wells et al. 1987), Mississippi/Louisiana (Miller et al. 2010; Miller et al. 2013), and Texas (McHugh 1989; Henderson et al. 2007).

Migration

The bay, sound, and estuary stocks in the Gulf of Mexico are thought to primarily comprise stable resident communities. However, transient or seasonal movements of some individuals into and out of a BSE stock area are recorded in some areas (LaBrecque et al. 2015; Hayes et al. 2022). Molecular genetic data support that some BSE stocks are genetically distinct from one another, and furthermore are differentiated from adjacent coastal populations (e.g., Sellas et al. 2005; Rosel et al. 2017). For example, Toms (2019) found fine-scale genetic population structure among five inshore systems in the Florida Panhandle and variable migration rates among populations.

Year-round residency has been reported for common bottlenose dolphins (Hayes et al. 2022), such as in Florida (Irvine and Wells 1972; Irvine et al. 1981; Wells 1986a; Wells 1986b; Wells et al. 1987; Scott et al. 1990; Wells 1991; Wells et al. 1996a; Wells et al. 1996b; Wells et al. 1997; Wells 2003; Balmer et al. 2008; Urian et al. 2009; Tyson et al. 2011; Bassos-Hull et al. 2013; B. Balmer et al. 2018; Balmer et al. 2019; Toms 2019), Texas (Shane 1990; Bräger 1993; Bräger et al. 1994; Fertl 1994; Weller 1998; Maze and Würsig 1999; Lynn and Würsig 2002; Irwin and Würsig 2004; Shane 2004), and Louisiana/Mississippi (Hubard et al. 2004; McDonald et al. 2017; Mullin et al. 2017; Takeshita et al. 2021).

Home Range

The home ranges of individuals within the Gulf of Mexico BSE stocks are considered to generally include the areas within the stock boundaries and brief excursions beyond the stock boundaries (Irvine et al. 1981; Wells 1986a; Wells 1986b; Wells 1991; Wells et al. 1996a; Wells et al. 1996b; Balmer et al. 2008; Wells 2014; Balmer et al. 2019), although some individuals show preference for small areas within the stock boundaries (Lynn and Würsig 2002; Urian et al. 2009; Mullin et al. 2017; Wells et al. 2017).

Stock Abundance

An abundance estimate for this stock is unavailable (Hayes et al. 2019).

Stock Abundance Trend

Data are insufficient to complete an abundance trend analysis for this stock (Hayes et al. 2019).

Cumulative Stressors

Common bottlenose dolphins face a wide range of individual and cumulative natural and anthropogenic threats (see reviews by Vollmer and Rosel 2013; Wells and Scott 2018; and Wells et al. 2019). These include such threats as shark predation, harmful algal blooms, recreational and commercial fishing gear (McHugh et al. 2021), vessel traffic, pollution, habitat alteration, and provisioning.

The nearshore and estuarine habitats along the Gulf of Mexico coast include waters adjacent to areas of high human population, some of which are highly industrialized, and areas of heavy agricultural impact. Studies of common bottlenose dolphin tissues from Florida Gulf Coast waters have shown persistent organic pollutants (POPs) in blubber and blood samples (Yordy et al. 2010a, 2010b, 2010c; Kucklick et al. 2011; J. Balmer et al. 2018). Similarly, mercury has been detected in common bottlenose dolphin tissues in Florida Gulf Coast waters (Bryan et al. 2007; Woshner et al. 2008; Miller et al. 2011; Correa et al. 2013; McCormack et al. 2020a, 2020b, 2022).

An Unusual Mortality Event (UME) was declared in 1990 for the Gulf of Mexico from Florida to Texas, with more than 300 common bottlenose dolphins stranding due to an undetermined cause, although morbillivirus and cold temperatures may have contributed (Litz et al. 2014). This stock was also within the range of the 2005–2006 UME in central west Florida caused by brevetoxin (Litz et al. 2014).

Further Reading

Hayes, S.A., E. Josephson, K. Maze-Foley, P.E. Rosel, and J. Wallace. 2022. U.S. Atlantic and Gulf of Mexico marine mammal stock assessments 2021. National Oceanic and Atmospheric Administration, National Marine Fisheries Service, Northeast Fisheries Science Center: Woods Hole, MA. NOAA Technical Memorandum NMFS-NE-288. 380 p. doi: 10.25923/6tt7-kc16

Wells, R.S. and M.D. Scott. 2018. Bottlenose Dolphin, *Tursiops truncatus*, Common Bottlenose Dolphin. In: Würsig, B., J.G.M. Thewissen, and K.M. Kovacs (Eds.), Encyclopedia of Marine Mammals (Third Edition). Academic Press, London, UK. pp. 118–125. doi: 10.1016/B978-0-12-804327-1.00072-8

Wells, R.S., A. Natoli, and G. Braulik. 2019. *Tursiops truncatus* (errata version published in 2019). The IUCN Red List of Threatened Species 2019: e.T22563A156932432. doi: doi.org/10.2305/IUCN.UK.2019-1.RLTS.T22563A156932432.en

References

Balmer, B., T. McDonald, F. Hornsby, J. Adams, J. Allen, A. Barleycorn, and L. Schwacke. 2018. Long-term trends in a northern Gulf of Mexico common bottlenose dolphin (*Tursiops truncatus*) population in the wake of the Deepwater Horizon oil spill. Journal of Cetacean Research and Management 18:1–9. doi: 10.47536/jcrm.v18i1.409

Balmer, B., S. Watwood, B. Quigley, T. Speakman, K. Barry, K. Mullin, P. Rosel, C. Sinclair, E. Zolman, and L. Schwacke. 2019. Common bottlenose dolphin (*Tursiops truncatus*) abundance and distribution patterns in St. Andrew Bay, Florida, USA. Aquatic Conservation: Marine and Freshwater Ecosystems 29(3):486–498. doi: 10.1002/aqc.3001

Balmer, B.C., R.S. Wells, S.M. Nowacek, D.P. Nowacek, L.H. Schwacke, W.A. McLellan, F.S. Scharf, T.K. Rowles, L.J. Hansen, T.R. Spradlin, and D.A. Pabst. 2008. Seasonal abundance and distribution patterns of common bottlenose dolphins (*Tursiops truncatus*) near St. Joseph Bay, Florida, USA. Journal of Cetacean Research and Management 10(2):157–167.

Balmer, J.E., G.M. Ylitalo, T.K. Rowles, K.D. Mullin, R.S. Wells, F.I. Townsend, R.W. Pearce, J.L. Bolton, E.S. Zolman, B.C. Balmer, and L.H. Schwacke. 2018. Persistent organic pollutants (POPs) in blood and blubber of common bottlenose dolphins (*Tursiops truncatus*) at three northern Gulf of Mexico sites following the Deepwater Horizon oil spill. Science of the Total Environment 621:130–137. doi: 10.1016/j.scitotenv.2017.11.209

Barros, N.B. and D.K. Odell. 1990. Food habits of bottlenose dolphins in the southeastern United States In: Leatherwood, S. and R.R. Reeves (Eds.), The Bottlenose Dolphin. Academic Press, San Diego, CA. pp. 309–328. doi: 10.1016/B978-0-12-440280-5.50020-2

Bassos-Hull, K., R.M. Perrtree, C.C. Shepard, S. Schilling, A.A. Barleycorn, J.B. Allen, B.C. Balmer, W.E. Pine, and R.S. Wells. 2013. Long-term site fidelity and seasonal abundance estimates of common bottlenose dolphins (*Tursiops truncatus*) along the southwest coast of Florida and responses to natural perturbations. Journal of Cetacean Research and Management 13(1):19–30. doi: 10.47536/jcrm.v13i1.551

Berens McCabe, E.J., D.P. Gannon, N.B. Barros, and R.S. Wells. 2010. Prey selection by resident common bottlenose dolphins (*Tursiops truncatus*) in Sarasota Bay, Florida. Marine Biology 157(5):931–942. doi: 10.1007/s00227-009-1371-2

Bowen-Stevens, S.R., D.P. Gannon, R.A. Hazelkorn, G. Lovewell, K.M. Volker, S. Smith, M.C. Tumlin, and J. Litz. 2021. Diet of common bottlenose dolphins, *Tursiops truncatus*, that stranded in and near Barataria Bay, Louisiana, 2010–2012. Southeastern Naturalist 20(1). doi: 10.1656/058.020.0113

Bräger, S. 1993. Diurnal and seasonal behavior patterns of bottlenose dolphins (*Tursiops truncatus*). Marine Mammal Science 9:434–438. doi: 10.1111/j.1748-7692.1993.tb00477.x

Bräger, S., B. Würsig, A. Acevedo, and T. Henningsen. 1994. Association patterns of bottle-nosed dolphins (*Tursiops-truncatus*) in Galveston Bay, Texas. Journal of Mammalogy 75(2):431–437. doi: 10.2307/1382564

Bryan, C.E., S.J. Christopher, B.C. Balmer, and R.S. Wells. 2007. Establishing baseline levels of trace elements in blood and skin of bottlenose dolphins in Sarasota Bay, Florida: implications for non-invasive monitoring. Science of the Total Environment 388(1-3):325–342. doi: 10.1016/j.scitotenv.2007.07.046

Connor, R.C., R.S. Wells, J. Mann, and A.J. Read. 2000. The bottlenose dolphin: Social relationships in a fission-fusion society. In: Mann, J., R. Connor, P. Tyack, and H. Whitehead (Eds.), Cetacean Societies: Field Studies of Dolphins and Whales. University of Chicago Press, Chicago, IL. pp. 91–126.

Correa, L., J.M. Castellini, R.S. Wells, and T. O'Hara. 2013. Distribution of mercury and selenium in blood compartments of bottlenose dolphins (*Tursiops truncatus*) from Sarasota Bay, Florida. Environmental Toxicology and Chemistry 32(11):2441–2448. doi: 10.1002/etc.2327

Fernandez, S. and A.A. Hohn. 1998. Age, growth, and calving season of bottlenose dolphins, *Tursiops truncatus*, off coastal Texas. Fishery Bulletin 96(2):357–365.

Fertl, D. 1994. Occurrence patterns and behavior of bottlenose dolphins (*Tursiops truncatus*) in the Galveston ship channel, Texas. Texas Journal of Science 46(4):299–318.

Hayes, S.A., E. Josephson, K. Maze-Foley, and P.E. Rosel. 2019. US Atlantic and Gulf of Mexico marine mammal stock assessments - 2018. National Oceanic and Atmospheric Administration, National Marine Fisheries Service, Northeast Fisheries Science Center: Woods Hole, MA. NOAA Technical Memorandum NMFS-NE-258. 298 p. doi: 10.25923/9rrd-tx13

Hayes, S.A., E. Josephson, K. Maze-Foley, P.E. Rosel, and J. Wallace. 2022. U.S. Atlantic and Gulf of Mexico marine mammal stock assessments 2021. National Oceanic and Atmospheric Administration, National Marine Fisheries Service, Northeast Fisheries Science Center: Woods Hole, MA. NOAA Technical Memorandum NMFS-NE-288. 380 p. doi: 10.25923/6tt7-kc16

Henderson, E.E. and B. Würsig. 2007. Behavior patterns of bottlenose dolphins in San Luis Pass, Texas. Gulf of Mexico Science 25(2):153–161. doi: 10.18785/goms.2502.06

Hohn, A.A., M.D. Scott, R.S. Wells, J.C. Sweeney, and A.B. Irvine. 1989. Growth layers in teeth from known-age, free-ranging bottlenose dolphins. Marine Mammal Science 5(4):315–342. doi: 10.1111/j.1748-7692.1989.tb00346.x

Hubard, C.W., K. Maze-Foley, K.D. Mullin, and W.W. Schroeder. 2004. Seasonal abundance and site fidelity of bottlenose dolphins (*Tursiops truncatus*) in Mississippi Sound. Aquatic Mammals 30(2):299–310. doi: 10.1578/am.30.2.2004.299

Irvine, A.B., M.D. Scott, R.S. Wells, and J.H. Kaufmann. 1981. Movements and activities of the Atlantic bottlenose dolphin, *Tursiops truncatus*, near Sarasota, Florida. Fishery Bulletin 79(4):671–688.

Irvine, B. and R.S. Wells. 1972. Results of attempts to tag Atlantic bottlenosed dolphins (*Tursiops truncatus*). Cetology 13:1–5.

Irwin, L.-J. and B. Würsig. 2004. A small resident community of bottlenose dolphins, *Tursiops truncatus*, in Texas: Monitoring recommendations. Gulf of Mexico Science 22(1):13–21. doi: 10.18785/goms.2201.02

Kucklick, J., L. Schwacke, R. Wells, A. Hohn, A. Guichard, J. Yordy, L. Hansen, E. Zolman, R. Wilson, J. Litz, D. Nowacek, T. Rowles, R. Pugh, B. Balmer, C. Sinclair, and P. Rosel. 2011. Bottlenose dolphins as indicators of persistent organic pollutants in the western North Atlantic Ocean and Northern Gulf of Mexico. Environmental Science & Technology 45(10):4270–4277. doi: 10.1021/es1042244

LaBrecque, E., C. Curtice, J. Harrison, S.M. Van Parijs, and P.N. Halpin. 2015. 3. Biologically Important Areas for cetaceans within U.S. Waters – Gulf of Mexico Region. Aquatic Mammals 41(1):30–38. doi: 10.1578/am.41.1.2015.30

Leatherwood, S. 1977. Some preliminary impressions on the numbers and social behavior of free-swimming bottlenosed dolphin calves (*Tursiops truncatus*) in the northern Gulf of Mexico. In: Ridgway, S.H. and K. Bernirschke (Eds.), Breeding Dolphins; Present Status, Suggestions for the Future. Final report to the US Marine Mammal Commission, Contract MM6AC009, MMC-76/07, Washington, DC. pp. 143–167.

Litz, J.A., M.A. Baran, S.R. Bowen-Stevens, R.H. Carmichael, K.M. Colegrove, L.P. Garrison, S.E. Fire, E.M. Fougeres, R. Hardy, S. Holmes, W. Jones, B.E. Mase-Guthrie, D.K. Odell, P.E. Rosel, J.T. Saliki, D.K. Shannon, S.F. Shippee, S.M. Smith, E.M. Stratton, M.C. Tumlin, H.R. Whitehead, G.A. Worthy, and T.K. Rowles. 2014. Review of historical unusual mortality events (UMEs) in the Gulf of Mexico (1990–2009): providing context for the multi-year northern Gulf of Mexico cetacean UME declared in 2010. Diseases of Aquatic Organisms 112(2):161–175. doi: 10.3354/dao02807

Lynn, S.K. and B. Würsig. 2002. Summer movement patterns of bottlenose dolphins in a Texas bay. Gulf of Mexico Science 20(1):25–37. doi: 10.18785/goms.2001.03

Mackey, A.D. 2010. Site fidelity and association patterns of bottlenose dolphins (*Tursiops truncatus*) in the Mississippi Sound. University of Southern Mississippi. Thesis. 133 p.

Mann, J., R.C. Connor, L.M. Barre, and M.R. Heithaus. 2000. Female reproductive success in bottlenose dolphins (*Tursiops* sp.): life history, habitat, provisioning, and group-size effects. Behavioral Ecology 11(2):210–219. doi: 10.1093/beheco/11.2.210

Mattson, M.C., K.D. Mullin, G.W. Ingram, and W. Hoggard. 2006. Age structure and growth of the bottlenose dolphin (*Tursiops truncatus*) from strandings in the Mississippi sound region of the north-central Gulf of Mexico from 1986 to 2003. Marine Mammal Science 22(3):654–666. doi: 10.1111/j.1748-7692.2006.00057.x

Maze, K.S. and B. Würsig. 1999. Bottlenosed dolphins of San Luis Pass, Texas: Occurrence patterns, site-fidelity, and habitat use. Aquatic Mammals 25:91–104.

McCormack, M.A., F. Battaglia, W.E. McFee, and J. Dutton. 2020a. Mercury concentrations in blubber and skin from stranded bottlenose dolphins (*Tursiops truncatus*) along the Florida and Louisiana coasts (Gulf of Mexico, USA) in relation to biological variables. Environmental Research 180:108886. doi: 10.1016/j.envres.2019.108886

McCormack, M.A., B.P. Jackson, and J. Dutton. 2020b. Relationship between mercury and selenium concentrations in tissues from stranded odontocetes in the northern Gulf of Mexico. Science of the Total Environment 749:141350. doi: 10.1016/j.scitotenv.2020.141350

McCormack, M.A., W.H. Nowlin, and J. Dutton. 2022. Effect of trophic position on mercury concentrations in bottlenose dolphins (*Tursiops truncatus*) from the northern Gulf of Mexico. Environmental Research 204(Pt B):112124. doi: 10.1016/j.envres.2021.112124

McDonald, T.L., F.E. Hornsby, T.R. Speakman, E.S. Zolman, K.D. Mullin, C. Sinclair, P.E. Rosel, L. Thomas, and L.H. Schwacke. 2017. Survival, density, and abundance of common bottlenose dolphins in

Barataria Bay (USA) following the Deepwater Horizon oil spill. Endangered Species Research 33:193–209. doi: 10.3354/esr00806

McHugh, K.A., A.A. Barleycorn, J.B. Allen, K. Bassos-Hull, G. Lovewell, D. Boyd, A. Panike, C. Cush, D. Fauquier, B. Mase, R.C. Lacy, M.R. Greenfield, D.I. Rubenstein, A. Weaver, A. Stone, L. Oliver, K. Morse, and R.S. Wells. 2021. Staying alive: Long-term success of bottlenose dolphin interventions in southwest Florida. Frontiers in Marine Science 7:624729. doi: 10.3389/fmars.2020.624729

McHugh, M.B. 1989. Population numbers and feeding behavior of the Atlantic bottlenose dolphin (*Tursiops truncatus*) near Aransas Pass, Texas. University of Texas at Austin. Thesis. 96 p.

Mead, J.G. and C.W. Potter. 1990. Natural history of bottlenose dolphins along the central Atlantic coast of the United States. In: Leatherwood, S. and R.R. Reeves (Eds.), The Bottlenose Dolphin. Academic Press, San Diego, CA. pp. 165–195.

Miller, C.E. 2003. Abundance trends and environmental habitat usage patterns of bottlenose dolphins (*Tursiops truncatus*) in lower Barataria and Caminada bays, Louisiana. Louisiana State University and Agricultural & Mechanical College. Dissertation. 125 p.

Miller, D.L., V. Woshner, E.L. Styer, S. Ferguson, K.K. Knott, M.J. Gray, R.S. Wells, and T.M. O'Hara. 2011. Histologic findings in free-ranging Sarasota Bay bottlenose dolphin (*Tursiops truncatus*) skin: mercury, selenium, and seasonal factors. Journal of Wildlife Diseases 47(4):1012–1018. doi: 10.7589/0090-3558-47.4.1012

Miller, L.J., A.D. Mackey, T. Hoffland, M. Solangi, and S.A. Kuczaj. 2010. Potential effects of a major hurricane on Atlantic bottlenose dolphin (*Tursiops truncatus*) reproduction in the Mississippi Sound. Marine Mammal Science 26(3):707–715. doi: 10.1111/j.1748-7692.2010.00371.x

Miller, L.J., A.D. Mackey, M. Solangi, and S.A. Kuczaj. 2013. Population abundance and habitat utilization of bottlenose dolphins in the Mississippi Sound. Aquatic Conservation: Marine and Freshwater Ecosystems 23(1):145–151. doi: 10.1002/aqc.2278

Mullin, K.D., T. McDonald, R.S. Wells, B.C. Balmer, T. Speakman, C. Sinclair, E.S. Zolman, F. Hornsby, S.M. McBride, K.A. Wilkinson, and L.H. Schwacke. 2017. Density, abundance, survival, and ranging patterns of common bottlenose dolphins (*Tursiops truncatus*) in Mississippi Sound following the Deepwater Horizon oil spill. PLoS ONE 12(10):e0186265. doi: 10.1371/journal.pone.0186265

Perrin, W.F. and S.B. Reilly. 1984. Reproductive parameters of dolphins and small whales of the family Delphinidae. In: Perrin, W.F., R.L. Brownell, Jr., and D.P. DeMaster (Eds.), Reproduction in Whales, Dolphins, and Porpoises. pp. 97–134.

Rosel, P.E., L.A. Wilcox, C. Sinclair, T.R. Speakman, M.C. Tumlin, J.A. Litz, and E.S. Zolman. 2017. Genetic assignment to stock of stranded common bottlenose dolphins in southeastern Louisiana after the Deepwater Horizon oil spill. Endangered Species Research 33:221–234. doi: 10.3354/esr00780

Rossman, S., P.H. Ostrom, M. Stolen, N.B. Barros, H. Gandhi, C.A. Stricker, and R.S. Wells. 2015. Individual specialization in the foraging habits of female bottlenose dolphins living in a trophically diverse and habitat rich estuary. Oecologia 178(2):415–425. doi: 10.1007/s00442-015-3241-6

Scott, M.D. and S.J. Chivers. 1990. Distribution and herd structure of bottlenose dolphins in the eastern tropical Pacific Ocean. In: Leatherwood, S. and R.R. Reeves (Eds.), The Bottlenose Dolphin. Academic Press, San Diego, CA. pp. 387–402.

Sellas, A.B., R.S. Wells, and P.E. Rosel. 2005. Mitochondrial and nuclear DNA analyses reveal fine scale geographic structure in bottlenose dolphins (*Tursiops truncatus*) in the Gulf of Mexico. Conservation Genetics 6(5):715–728. doi: 10.1007/s10592-005-9031-7

Sergeant, D.E., D.K. Caldwell, and M.C. Caldwell. 1973. Age, growth, and maturity of bottlenosed dolphin (*Tursiops-truncatus*) from northeast Florida. Journal of the Fisheries Research Board of Canada 30(7):1009–1011. doi: 10.1139/f73-165

Shane, S.H. 1990. Behavior and ecology of the bottlenose dolphin at Sanibel Island, Florida. In: Leatherwood, S. and R.R. Reeves (Eds.), The Bottlenose Dolphin. Academic Press, San Diego, CA. pp. 245–265. doi: 10.1016/B978-0-12-440280-5.50016-0

Shane, S.H. 2004. Residence patterns, group characteristics, and association patterns of bottlenose dolphins near Sanibel Island, Florida. Gulf of Mexico Science 22(1):1–12. doi: 10.18785/goms.2201.01

Shane, S.H., R.S. Wells, and B. Würsig. 1986. Ecology, behavior and social organization of the bottlenose dolphin: A review. Marine Mammal Science 2:34–63. doi: 10.1111/j.1748-7692.1986.tb00026.x

Takeshita, R., B.C. Balmer, F. Messina, E.S. Zolman, L. Thomas, R.S. Wells, C.R. Smith, T.K. Rowles, and L.H. Schwacke. 2021. High site-fidelity in common bottlenose dolphins despite low salinity exposure and associated indicators of compromised health. PLoS ONE 16(9):e0258031. doi: 10.1371/journal.pone.0258031

Taylor, B.L., S.J. Chivers, J. Larese, and W.F. Perrin. 2007. Generation length and percent mature estimates for IUCN assessments of cetaceans. National Oceanic and Atmospheric Administration, National Marine Fisheries Service, Southwest Fisheries Science Center: La Jolla, CA. Administrative Report LJ-07-01. 24 p.

Toms, C. 2019. Filling the gaps: Common bottlenose dolphin (*Tursiops truncatus*) population dynamics, structure, and connectivity within Florida panhandle bays, sounds, and estuaries. University of Central Florida. Dissertation. 373 p.

Torres, L.G. and A.J. Read. 2009. Where to catch a fish? The influence of foraging tactics on the ecology of bottlenose dolphins (*Tursiops truncatus*) in Florida Bay, Florida. Marine Mammal Science 25(4):797–815. doi: 10.1111/j.1748-7692.2009.00297.x

Tyson, R.B., S.M. Nowacek, and D.P. Nowacek. 2011. Community structure and abundance of bottlenose dolphins *Tursiops truncatus* in coastal waters of the northeast Gulf of Mexico. Marine Ecology Progress Series 438:253–265. doi: 10.3354/meps09292

Urian, K.W., S. Hofmann, R.S. Wells, and A.J. Read. 2009. Fine-scale population structure of bottlenose dolphins (*Tursiops truncatus*) in Tampa Bay, Florida. Marine Mammal Science 25(3):619–638. doi: 10.1111/j.1748-7692.2009.00284.x

Vollmer, N.L. and P.E. Rosel. 2013. A review of common bottlenose dolphins (*Tursiops truncatus truncatus*) in the Northern Gulf of Mexico: Population biology, potential threats, and management. Southeastern Naturalist 12(m6):1–43. doi: 10.1656/058.012.m601

Weller, D.W. 1998. Global and regional variation in the biology and behavior of bottlenose dolphins. Texas A&M University. Dissertation. 142 p.

Wells, R.S. 1986a. Population structure of bottlenose dolphins: Behavioral studies along the central west coast of Florida. Contract Report to National Marine Fisheries Service, Southeast Fisheries Center. Contract No. 45-WCNF-5-003BB. 58 p.

Wells, R.S. 1986b. Structural aspects of dolphin societies. University of California, Santa Cruz. Dissertation. 234 p.

Wells, R.S. 1991. The role of long-term study in understanding the social structure of a bottlenose dolphin community. In: Pryor, K. and K. Norris (Eds.), Dolphin Societies: Discoveries and Puzzles. University of California Press, Berkeley, CA. pp. 199–226.

Wells, R.S. 2000. Reproduction in wild bottlenose dolphins: Overview of patterns observed during a long-term study. In: Duffield, D. and T. Robeck (Eds.), Bottlenose Dolphin Reproduction Workshop. AZA Marine Mammal Taxon Advisory Group, Silver Spring, MD. pp. 57–74.

Wells, R.S. 2003. Dolphin social complexity: Lessons from long-term study and life history. In: de Waal, F.B.M. and P.L. Tyack (Eds.), Animal Social Complexity: Intelligence, Culture, and Individualized Societies Harvard University Press, Cambridge, MA. pp. 32–56.

Wells, R.S. 2014. Social structure and life history of bottlenose dolphins near Sarasota Bay, Florida: Insights from four decades and five generations. Primates and cetaceans. pp. 149–172. doi: 10.1007/978-4-431-54523-1_8

Wells, R.S., M.K. Bassos, K.W. Urian, W.J. Carr, and M.D. Scott. 1996. Low-level monitoring of bottlenose dolphins, *Tursiops truncatus*, in Charlotte Harbor, Florida: 1990-1994. National Oceanic and Atmospheric Administration, National Marine Fisheries Service, Southeast Fisheries Science Center: Miami, FL. NOAA Technical Memorandum NMFS-SEFSC-384. 86 p.

Wells, R.S., M.K. Bassos, K.W. Urian, S.H. Shane, E.C.G. Owen, C.F. Weiss, W.J. Carr, and M.D. Scott. 1997. Low-level monitoring of bottlenose dolphins, *Tursiops truncatus*, in Pine Island Sound, Florida: 1996. National Oceanic and Atmospheric Administration, National Marine Fisheries Service, Southeast Fisheries Science Center: Miami, FL. Southeast Fisheries Center Contribution No. 40-WCNF601958. 86 p.

Wells, R.S., A. Natoli, and G. Braulik. 2019. *Tursiops truncatus* (errata version published in 2019). *The IUCN Red List of Threatened Species* 2019: e.T22563A156932432. https://dx.doi.org/10.2305/IUCN.UK.2019-1.RLTS.T22563A156932432.en

Wells, R.S., L.H. Schwacke, T.K. Rowles, B.C. Balmer, E. Zolman, T. Speakman, F.I. Townsend, M.C. Tumlin, A. Barleycorn, and K.A. Wilkinson. 2017. Ranging patterns of common bottlenose dolphins *Tursiops truncatus* in Barataria Bay, Louisiana, following the Deepwater Horizon oil spill. Endangered Species Research 33:159–180. doi: 10.3354/esr00732

Wells, R.S. and M.D. Scott. 1999. Bottlenose dolphin *Tursiops truncatus* (Montagu, 1821). In: Ridgway, S.H. and R. Harrison (Eds.), Handbook of marine mammals. Volume 6: The second book of dolphins and the porpoises. Academic Press, San Diego, CA. pp. 137–182.

Wells, R.S. and M.D. Scott. 2018. Bottlenose dolphin, *Tursiops truncatus*, common bottlenose dolphin. In: Würsig, B., J.G.M. Thewissen, and K.M. Kovacs (Eds.), Encyclopedia of Marine Mammals (Third Edition). Academic Press, London, UK. pp. 118–125. doi: 10.1016/B978-0-12-804327-1.00072-8

Wells, R.S., M.D. Scott, and A.B. Irvine. 1987. The social structure of free-ranging bottlenose dolphins. In: Genoways, H.H. (Ed.), Current Mammalogy. Plenum Press, New York, NY. pp. 247–305.

Wells, R.S., K.W. Urian, A.J. Read, M.K. Bassos, W.J. Carr, and M.D. Scott. 1996. Low-level monitoring of bottlenose dolphins, *Tursiops truncatus*, in Tampa Bay, Florida: 1988-1993. National Oceanic and Atmospheric Administration, National Marine Fisheries Service, Southeast Fisheries Science Center: Miami, FL. NOAA Technical Memorandum NMFS-SEFSC-385. 36 p.

Wilson, R.M., R.B. Tyson, J.A. Nelson, B.C. Balmer, J.P. Chanton, and D.P. Nowacek. 2017. Niche differentiation and prey selectivity among common bottlenose dolphins (*Tursiops truncatus*) sighted in St. George Sound, Gulf of Mexico. Frontiers in Marine Science 4:235. doi: 10.3389/fmars.2017.00235

Woshner, V., K. Knott, R. Wells, C. Willetto, R. Swor, and T. O'Hara. 2008. Mercury and selenium in blood and epidermis of bottlenose dolphins (*Tursiops truncatus*) from Sarasota Bay, FL: interaction and relevance to life history and hematologic parameters. Ecohealth 5(3):360–370. doi: 10.1007/s10393-008-0164-2

Yordy, J.E., M.A. Mollenhauer, R.M. Wilson, R.S. Wells, A. Hohn, J. Sweeney, L.H. Schwacke, T.K. Rowles, J.R. Kucklick, and M.M. Peden-Adams. 2010a. Complex contaminant exposure in cetaceans: a comparative E-Screen analysis of bottlenose dolphin blubber and mixtures of four persistent organic pollutants. Environmental Toxicology and Chemistry 29(10):2143–2153. doi: 10.1002/etc.279

Yordy, J.E., D.A. Pabst, W.A. McLellan, R.S. Wells, T.K. Rowles, and J.R. Kucklick. 2010b. Tissue-specific distribution and whole-body burden estimates of persistent organic pollutants in the bottlenose dolphin (*Tursiops truncatus*). Environmental Toxicology and Chemistry 29(6):1263–1273. doi: 10.1002/etc.152

Yordy, J.E., R.S. Wells, B.C. Balmer, L.H. Schwacke, T.K. Rowles, and J.R. Kucklick. 2010c. Life history as a source of variation for persistent organic pollutant (POP) patterns in a community of common bottlenose dolphins (*Tursiops truncatus*) resident to Sarasota Bay, FL. Science of the Total Environment 408(9):2163–2172. doi: 10.1016/j.scitotenv.2010.01.032

Common bottlenose dolphin − *Tursiops truncatus*

GoMx BSE/St. Vincent Sound, Apalachicola Bay, St. George Sound Stock

Overall Vulnerability Rank = Very High

Biological Sensitivity = Very High

Climate Exposure = Very High

Data Quality = 93% of scores ≥ 2

	Tursiops truncatus	Expert Scores	Data Quality	Expert Scores Plots (Portion by Category)
Sensitivity attributes	Prey/Diet Specificity	2.1	2.0	
	Habitat Specificity	3.6	2.2	
	Site Fidelity	3.8	2.5	
	Lifetime Reproductive Potential	1.2	2.2	
	Generation Length	2.9	2.2	
	Reproductive Plasticity	1.6	2.2	
	Migration	3.6	2.2	
	Home Range	3.4	2.2	
	Stock Abundance	3.0	2.2	
	Stock Abundance Trend	2.2	1.2	
	Cumulative Stressors	3.6	2.2	
	Sensitivity Score	Very High		
Exposure variables	Sea Surface Temperature (Change in mean)	4.0	2.8	
	Sea Surface Temperature (Change in variability)	1.0	2.8	
	Air Temperature (Change in mean)	4.0	2.8	
	Air Temperature (Change in variability)	1.0	2.8	
	Precipitation (Change in mean)	1.2	2.8	
	Precipitation (Change in variability)	1.0	2.8	
	Sea Surface Salinity (Change in mean)	2.0	2.8	
	Sea Surface Salinity (Change in variability)	1.0	2.8	
	Ocean pH (Change in mean)	4.0	2.8	
	Ocean pH (Change in variability)	1.0	2.8	
	Sea ice coverage (Change in mean)	1.0	2.8	
	Sea ice coverage (Change in variability)	1.0	2.8	
	Dissolved oxygen (Change in mean)	4.0	2.8	
	Dissolved oxygen (Change in variability)	1.0	2.8	
	Circulation	3.8	1.8	
	Sea level rise	3.0	2.8	
	Exposure Score	Very High		
Overall Vulnerability Rank		Very High		

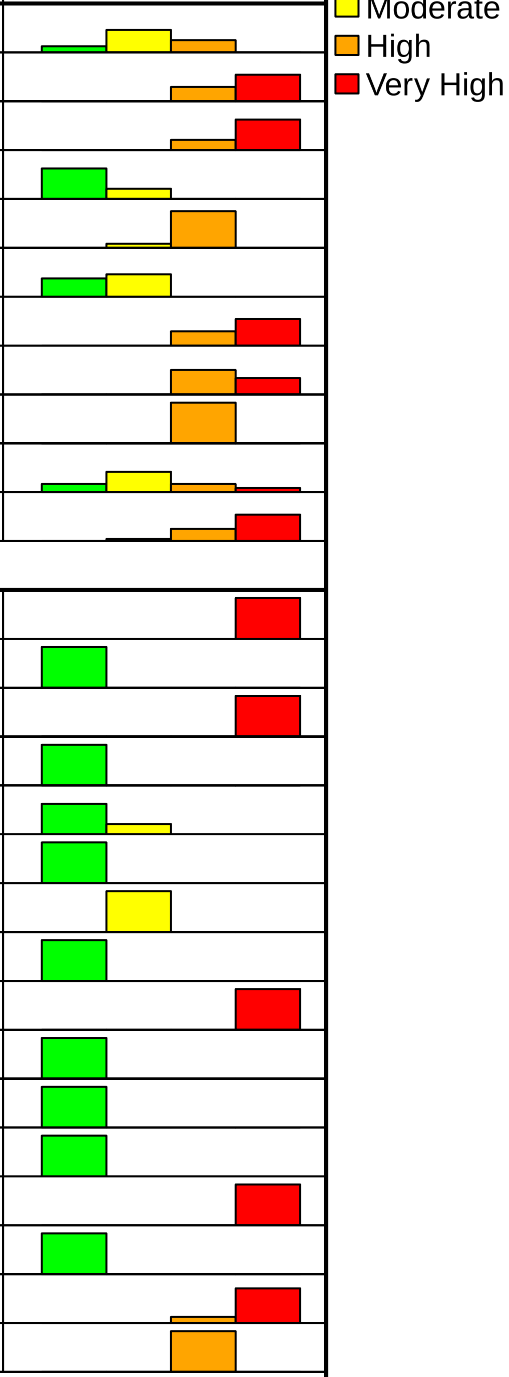

Common bottlenose dolphin (GoMx BSE/St. Vincent Sound, Apalachicola Bay, St. George Sound Stock)

Tursiops truncatus

CVA Results Summary

Overall Climate Vulnerability Rank: Very High (100% certainty from bootstrap analysis).

Climate Exposure: Very High. Five exposure factors scored greater than 3.5: Air Temperature (Standard anomaly) (4.00), Dissolved oxygen (Standard anomaly) (4.00), Ocean pH (Standard anomaly) (4.00), Sea Surface Temperature (Standard anomaly) (4.00), and Circulation (3.85).

Biological Sensitivity: Very High. Four sensitivity attributes scored greater than 3.5: Site Fidelity (3.75), Habitat Specificity (3.65), Migration (3.65), and Cumulative Stressors (3.60).

Distributional Response: High

Abundance Response: Very High

Phenology Response: High

Data Quality: 93% of the data quality scores were 2 or greater, 91% of sensitivity attribute data quality scores were 2 or higher.

Background Information

Stock Description

This stock includes common bottlenose dolphins in St. Vincent Sound, Apalachicola Bay, and St. George Sound in the northeast Gulf of Mexico along the Florida coast (Hayes et al. 2022).

Prey/Diet Specificity

Generally, common bottlenose dolphins consume a large variety of fish and/or squid (Barros and Odell 1990; Mead and Potter 1990; Wells and Scott 2018). Common bottlenose dolphins seem to show a consistent preference for sound-producing fishes (e.g., sciaenids, toadfish; Berens McCabe et al. 2010; Bowen-Stevens et al. 2021). In Sarasota Bay, Florida, Rossman et al. (2015) found some individual common bottlenose dolphins to show diet specialization despite the broader study group showing a generalist diet. Specialization can be influenced by locally abundant prey species (Shane et al. 1986; Vollmer and Rosel 2013) or can result from resource partitioning amongst sympatric populations (e.g., Torres and Read 2009; Wilson et al. 2017).

Habitat Specificity

Common bottlenose dolphins are found in a variety of marine and estuarine habitats (Wells and Scott 2018).

Site Fidelity

Studies from St. Vincent Sound and Apalachicola Bay suggest common bottlenose dolphins in this stock show site fidelity (Tyson et al. 2011).

Common bottlenose dolphins in other Gulf of Mexico bay, sound, and estuary (BSE) stocks also show site fidelity (LaBrecque et al. 2015; Hayes et al. 2022), such as in Charlotte Harbor and Pine Island Sound (Bassos-Hull et al. 2013), Sarasota Bay (Wells 1986a; Wells 1991; Wells 2014), St. Andrew Bay (Balmer et al. 2019a), St. Joseph Bay (Balmer et al. 2008; B. Balmer et al. 2018), St. Vincent Sound and Apalachicola Bay (Tyson et al. 2011), and Tampa Bay in Florida (Wells et al. 1996b; Urian et al. 2009); Matagorda Bay, Espiritu Santo Bay, and San Luis Pass in Texas (Maze and Würsig 1999; Lynn and Würsig 2002; Irwin and Würsig 2004); Barataria Bay in Louisiana (Miller 2003; McDonald et al. 2017; Wells et al. 2017); and Mississippi Sound in Mississippi (Hubard et al. 2004; Mackey 2010; Mullin et al. 2017; Takeshita et al. 2021).

Lifetime Reproductive Potential

Taylor et al. (2007) reported the interbirth interval of common bottlenose dolphins as 3.80 years, though other studies have shown it to be closer to three years (Leatherwood 1977; Wells and Scott 1999; Connor et al. 2000; Wells 2003).

Generally, females reach sexual maturity before males, with females reaching sexual maturity at 5–13 years (Sergeant et al. 1973; Perrin and Reilly 1984; Wells et al. 1987; Mead and Potter 1990; Mann et al. 2000; Wells 2000; Wells and Scott 2018).

Females reproduce through most of their adult lives (Leatherwood 1977; Hohn et al. 1989; Wells and Scott 1999; Wells 2000; Wells 2003), but may produce fewer calves after they reach 30 years of age (Wells 2000). Sarasota Bay common bottlenose dolphins can give birth when they are as young as 6 years of age, and can continue until they are 48 years old (Wells and Scott 2018). Reproductive lifespan would therefore be up to 42 years based on long-term observation studies (Wells 2014; Wells and Scott 2018).

Longitudinal studies from Sarasota Bay, Florida have found that female common bottlenose dolphins can live up to 67 years (Wells and Scott 2018). Studies based on growth layer groups in teeth suggest female common bottlenose dolphins can live to 41 years along the Texas coast (Fernandez and Hohn 1998) and to 30 years along the Mississippi coast (Mattson et al. 2006).

Generation Length

Taylor et al. (2007) reported a common bottlenose dolphin generation length of 20.6–21.1 years.

Reproductive Plasticity

Common bottlenose dolphin calving is generally diffusely seasonal, with one or more peaks, although births have been reported from all seasons (Vollmer and Rosel 2013; Wells and Scott 2018). Calf sightings peak in the spring and summer months in Florida (Wells et al. 1987), Mississippi/Louisiana (Miller et al. 2010; Miller et al. 2013), and Texas (McHugh 1989; Henderson 2004).

Migration

The bay, sound, and estuary stocks in the Gulf of Mexico are thought to primarily comprise stable resident communities. However, transient or seasonal movements of some individuals into and out of a BSE stock area are recorded in some areas (LaBrecque et al. 2015; Hayes et al. 2022). Molecular genetic data support that some BSE stocks are genetically distinct from one another, and furthermore are differentiated from adjacent coastal populations (e.g., Sellas et al. 2005; Rosel et al. 2017). For example, Toms (2019) found fine-scale genetic population structure among five inshore systems in the Florida Panhandle and variable migration rates among populations.

Year-round residency has been reported for common bottlenose dolphins (Hayes et al. 2022), such as in Florida (Irvine and Wells 1972; Irvine et al. 1981; Wells 1986a; Wells 1986b; Wells et al. 1987; Scott et al. 1990; Wells 1991; Wells et al. 1996a; Wells et al. 1996b; Wells et al. 1997; Wells 2003; Balmer et al. 2008; Urian et al. 2009; Tyson et al. 2011; Bassos-Hull et al. 2013; B. Balmer et al. 2018; Balmer et al. 2019a; Toms 2019), Texas (Shane 1990; Bräger 1993; Bräger et al. 1994; Fertl 1994; Weller 1998; Maze and Würsig 1999; Lynn and Würsig 2002; Irwin and Würsig 2004; Shane 2004), and Louisiana/Mississippi (Hubard et al. 2004; McDonald et al. 2017; Mullin et al. 2017; Takeshita et al. 2021).

Home Range

The home ranges of individuals within the Gulf of Mexico BSE stocks are considered to generally include the areas within the stock boundaries and brief excursions beyond the stock boundaries (Irvine et al. 1981; Wells 1986a; Wells 1986b; Wells 1991; Wells et al. 1996a; Wells et al. 1996b; Balmer et al. 2008; Wells 2014; Balmer et al. 2019a), although some individuals show preference for small areas within the stock boundaries (Lynn and Würsig 2002; Urian et al. 2009; Mullin et al. 2017; Wells et al. 2017). Stable isotope data suggests individuals in St. George Sound maintain a restricted home range (Wilson et al. 2013).

Stock Abundance

Tyson et al. (2011) found that two parapatric dolphin communities exist in the waters stretching from St. Vincent Sound to Alligator Harbor, with the eastern community (inhabiting St. George Sound and Alligator Harbor) supporting a more transient population than the western community (inhabiting St. Vincent Sound and Apalachicola Bay). Independent estimates of abundance were calculated using the Chapman modification of the Lincoln-Petersen method for June 2007 and for January and February 2008 for the eastern area (242 individuals [141−343], 395 individuals [273−516]) and for the western survey area (197 individuals [130−264], 111 individuals [71−150]), respectively. However, given these communities are still considered a single stock, their abundance estimate is considered the sum of the June 2007 estimates (439 individuals; CV=0.14; Tyson et al. 2011; Hayes et al. 2022).

Stock Abundance Trend

Data are insufficient to complete an abundance trend analysis for this stock (Hayes et al. 2022).

Cumulative Stressors

Common bottlenose dolphins face a wide range of individual and cumulative natural and anthropogenic threats (see reviews by Vollmer and Rosel 2013; Wells and Scott 2018; and Wells et al. 2019). These include such threats as shark predation, harmful algal blooms (Twiner et al. 2012), recreational and commercial fishing gear, vessel traffic, pollution, habitat alteration, and provisioning.

The nearshore and estuarine habitats along the Gulf of Mexico coast include waters adjacent to areas of high human population, some of which are highly industrialized, and areas of heavy agricultural impact. Studies have shown persistent organic pollutants (POPs) in tissues of common bottlenose dolphins from the Florida Panhandle (Wilson et al. 2012; Balmer et al. 2019b) and other Florida Gulf Coast waters (Yordy et al. 2010a, 2010b, 2010c; Kucklick et al. 2011; J. Balmer et al. 2018). Similarly, mercury has been detected in common bottlenose dolphin tissues in Florida Gulf Coast waters (Bryan et al. 2007; Woshner et al. 2008; Miller et al. 2011; Correa et al. 2013; McCormack et al. 2020a, 2020b, 2022).

Common bottlenose dolphins in this stock were possibly exposed to oil and dispersants following the Deepwater Horizon oil spill, which may result in long term reproductive and chronic health effects (NOAA 2011; Michel et al. 2013; Helm et al. 2015; DWH NRDA 2016; Lane et al. 2015; De Guise et al. 2017, 2021; Kellar et al. 2017; Smith et al. 2017; Schwacke et al. 2017). An Unusual Mortality Event (UME), involving primarily bottlenose dolphins, was declared for cetaceans in the northern Gulf of Mexico from 2010 to 2014, with the Deepwater Horizon oil spill as the primary underlying cause (Litz et al. 2014; Schwacke et al. 2014; Venn-Watson et al. 2015a, 2015b).

UMEs have been declared for common bottlenose dolphins in waters of the Florida panhandle with biotoxins (e.g., brevetoxin from *Karenia brevis*) the suspected or confirmed cause (Twiner et al. 2012; Litz et al. 2014). Dolphin morbillivirus has been detected in populations of the northern Gulf of Mexico, but has not been attributed to large scale die-offs since 1993−1994 (Lipscomb et al. 1996; Litz et al. 2014; Van Bressem et al. 2014; Fauquier et al. 2017). A UME was declared in 1990 for the Gulf of Mexico from Florida to Texas, with more than 300 common bottlenose dolphins stranding due to an undetermined cause, although morbillivirus and cold temperatures may have contributed (Litz et al. 2014).

Further Reading

Hayes, S.A., E. Josephson, K. Maze-Foley, P.E. Rosel, and J. Wallace. 2022. U.S. Atlantic and Gulf of Mexico marine mammal stock assessments 2021. National Oceanic and Atmospheric Administration, National Marine Fisheries Service, Northeast Fisheries Science Center: Woods Hole, MA. NOAA Technical Memorandum NMFS-NE-288. 380 p. doi: 10.25923/6tt7-kc16

Wells, R.S. and M.D. Scott. 2018. Bottlenose Dolphin, *Tursiops truncatus*, Common Bottlenose Dolphin. In: Würsig, B., J.G.M. Thewissen, and K.M. Kovacs (Eds.), Encyclopedia of Marine Mammals (Third Edition). Academic Press, London, UK. pp. 118–125. doi: 10.1016/B978-0-12-804327-1.00072-8

Wells, R.S., A. Natoli, and G. Braulik. 2019. *Tursiops truncatus* (errata version published in 2019). The IUCN Red List of Threatened Species 2019: e.T22563A156932432. https://dx.doi.org/10.2305/IUCN.UK.2019-1.RLTS.T22563A156932432.en

References

Balmer, B., T. McDonald, F. Hornsby, J. Adams, J. Allen, A. Barleycorn, and L. Schwacke. 2018. Long-term trends in a northern Gulf of Mexico common bottlenose dolphin (*Tursiops truncatus*) population in the wake of the Deepwater Horizon oil spill. Journal of Cetacean Research and Management 18:1–9. doi: 10.47536/jcrm.v18i1.409

Balmer, B., S. Watwood, B. Quigley, T. Speakman, K. Barry, K. Mullin, P. Rosel, C. Sinclair, E. Zolman, and L. Schwacke. 2019. Common bottlenose dolphin (*Tursiops truncatus*) abundance and distribution patterns in St. Andrew Bay, Florida, USA. Aquatic Conservation: Marine and Freshwater Ecosystems 29(3):486–498. doi: 10.1002/aqc.3001

Balmer, B., G. Ylitalo, S. Watwood, B. Quigley, J. Bolton, K. Mullin, P. Rosel, T. Rowles, T. Speakman, L. Wilcox, E. Zolman, and L. Schwacke. 2019. Comparison of persistent organic pollutants (POPs) between small cetaceans in coastal and estuarine waters of the northern Gulf of Mexico. Marine Pollution Bulletin 145:239–247. doi: 10.1016/j.marpolbul.2019.05.017

Balmer, B.C., R.S. Wells, S.M. Nowacek, D.P. Nowacek, L.H. Schwacke, W.A. McLellan, F.S. Scharf, T.K. Rowles, L.J. Hansen, T.R. Spradlin, and D.A. Pabst. 2008. Seasonal abundance and distribution patterns of common bottlenose dolphins (*Tursiops truncatus*) near St. Joseph Bay, Florida, USA. Journal of Cetacean Research and Management 10(2):157–167.

Balmer, J.E., G.M. Ylitalo, T.K. Rowles, K.D. Mullin, R.S. Wells, F.I. Townsend, R.W. Pearce, J.L. Bolton, E.S. Zolman, B.C. Balmer, and L.H. Schwacke. 2018. Persistent organic pollutants (POPs) in blood and blubber of common bottlenose dolphins (*Tursiops truncatus*) at three northern Gulf of Mexico sites following the Deepwater Horizon oil spill. Science of the Total Environment 621:130–137. doi: 10.1016/j.scitotenv.2017.11.209

Barros, N.B. and D.K. Odell. 1990. Food habits of bottlenose dolphins in the southeastern United States In: Leatherwood, S. and R.R. Reeves (Eds.), The Bottlenose Dolphin. Academic Press, San Diego, CA. pp. 309–328. doi: 10.1016/B978-0-12-440280-5.50020-2

Bassos-Hull, K., R.M. Perrtree, C.C. Shepard, S. Schilling, A.A. Barleycorn, J.B. Allen, B.C. Balmer, W.E. Pine, and R.S. Wells. 2013. Long-term site fidelity and seasonal abundance estimates of common bottlenose dolphins (*Tursiops truncatus*) along the southwest coast of Florida and responses to natural perturbations. Journal of Cetacean Research and Management 13(1):19–30. doi: 10.47536/jcrm.v13i1.551

Berens McCabe, E.J., D.P. Gannon, N.B. Barros, and R.S. Wells. 2010. Prey selection by resident common bottlenose dolphins (*Tursiops truncatus*) in Sarasota Bay, Florida. Marine Biology 157(5):931–942. doi: 10.1007/s00227-009-1371-2

Bowen-Stevens, S.R., D.P. Gannon, R.A. Hazelkorn, G. Lovewell, K.M. Volker, S. Smith, M.C. Tumlin, and J. Litz. 2021. Diet of common bottlenose dolphins, *Tursiops truncatus*, that stranded in and near Barataria Bay, Louisiana, 2010–2012. Southeastern Naturalist 20(1). doi: 10.1656/058.020.0113

Bräger, S. 1993. Diurnal and seasonal behavior patterns of bottlenose dolphins (*Tursiops truncatus*). Marine Mammal Science 9:434–438. doi: 10.1111/j.1748-7692.1993.tb00477.x

Bräger, S., B. Würsig, A. Acevedo, and T. Henningsen. 1994. Association patterns of bottle-nosed dolphins (*Tursiops-truncatus*) in Galveston Bay, Texas. Journal of Mammalogy 75(2):431–437. doi: 10.2307/1382564

Bryan, C.E., S.J. Christopher, B.C. Balmer, and R.S. Wells. 2007. Establishing baseline levels of trace elements in blood and skin of bottlenose dolphins in Sarasota Bay, Florida: implications for non-invasive monitoring. Science of the Total Environment 388(1-3):325–342. doi: 10.1016/j.scitotenv.2007.07.046

Connor, R.C., R.S. Wells, J. Mann, and A.J. Read. 2000. The bottlenose dolphin: Social relationships in a fission-fusion society. In: Mann, J., R. Connor, P. Tyack, and H. Whitehead (Eds.), Cetacean

Societies: Field Studies of Dolphins and Whales. University of Chicago Press, Chicago, IL. pp. 91–126.

Correa, L., J.M. Castellini, R.S. Wells, and T. O'Hara. 2013. Distribution of mercury and selenium in blood compartments of bottlenose dolphins (*Tursiops truncatus*) from Sarasota Bay, Florida. Environmental Toxicology and Chemistry 32(11):2441–2448. doi: 10.1002/etc.2327

De Guise, S., M. Levin, E. Gebhard, L. Jasperse, L. Burdett Hart, C.R. Smith, S. Venn-Watson, F. Townsend, R. Wells, B. Balmer, E. Zolman, T. Rowles, and L. Schwacke. 2017. Changes in immune functions in bottlenose dolphins in the northern Gulf of Mexico associated with the Deepwater Horizon oil spill. Endangered Species Research 33:291–303. doi: 10.3354/esr00814

De Guise, S., M. Levin, L. Jasperse, J. Herrman, R.S. Wells, T. Rowles, and L. Schwacke. 2021. Long-term immunological alterations in bottlenose dolphin a decade after the Deepwater Horizon oil spill in the northern Gulf of Mexico: Potential for multigenerational effects. Environmental Toxicology and Chemistry 40(5):1308–1321. doi: 10.1002/etc.4980

Deepwater Horizon Natural Resource Damage Assessment Trustees (DWH NRDA). 2016. Deepwater Horizon oil spill: Final Programmatic Damage Assessment and Restoration Plan and Final Programmatic Environmental Impact Statement. Retrieved from: http://www.gulfspillrestoration.noaa.gov/restoration-planning/gulf-plan.1659.

Fauquier, D.A., J. Litz, S. Sanchez, K. Colegrove, L.H. Schwacke, L. Hart, J. Saliki, C. Smith, T. Goldstein, S. Bowen-Stevens, W. McFee, E. Fougeres, B. Mase-Guthrie, E. Stratton, R. Ewing, S. Venn-Watson, R.H. Carmichael, C. Clemons-Chevis, W. Hatchett, D. Shannon, S. Shippee, S. Smith, L. Staggs, M.C. Tumlin, N.L. Wingers, and T.K. Rowles. 2017. Evaluation of morbillivirus exposure in cetaceans from the northern Gulf of Mexico 2010-2014. Endangered Species Research 33:211–220. doi: 10.3354/esr00772

Fernandez, S. and A.A. Hohn. 1998. Age, growth, and calving season of bottlenose dolphins, *Tursiops truncatus*, off coastal Texas. Fishery Bulletin 96(2):357–365.

Fertl, D. 1994. Occurrence patterns and behavior of bottlenose dolphins (*Tursiops truncatus*) in the Galveston ship channel, Texas. Texas Journal of Science 46(4):299–318.

Hayes, S.A., E. Josephson, K. Maze-Foley, P.E. Rosel, and J. Wallace. 2022. U.S. Atlantic and Gulf of Mexico marine mammal stock assessments 2021. National Oceanic and Atmospheric Administration, National Marine Fisheries Service, Northeast Fisheries Science Center: Woods Hole, MA. NOAA Technical Memorandum NMFS-NE-288. 380 p. doi: 10.25923/6tt7-kc16

Helm, R.C., D.P. Costa, T.D. DeBruyn, T.J. O'Shea, R.S. Wells, and T.M. Williams. 2015. Overview of effects of oil spills on marine mammals. In: Fingas, M. (Ed.), Handbook of oil spill science and technology, First edition. John Wiley & Sons, Inc., Hoboken, NJ. pp. 455–475.

Henderson, E.E. and B. Würsig. 2007. Behavior patterns of bottlenose dolphins in San Luis Pass, Texas. Gulf of Mexico Science 25(2):153–161. doi: 10.18785/goms.2502.06

Hohn, A.A., M.D. Scott, R.S. Wells, J.C. Sweeney, and A.B. Irvine. 1989. Growth layers in teeth from known-age, free-ranging bottlenose dolphins. Marine Mammal Science 5(4):315–342. doi: 10.1111/j.1748-7692.1989.tb00346.x

Hubard, C.W., K. Maze-Foley, K.D. Mullin, and W.W. Schroeder. 2004. Seasonal abundance and site fidelity of bottlenose dolphins (*Tursiops truncatus*) in Mississippi Sound. Aquatic Mammals 30(2):299–310. doi: 10.1578/am.30.2.2004.299

Irvine, A.B., M.D. Scott, R.S. Wells, and J.H. Kaufmann. 1981. Movements and activities of the Atlantic bottlenose dolphin, *Tursiops truncatus*, near Sarasota, Florida. Fishery Bulletin 79(4):671–688.

Irvine, B. and R.S. Wells. 1972. Results of attempts to tag Atlantic bottlenosed dolphins (*Tursiops truncatus*). Cetology 13:1–5.

Irwin, L.-J. and B. Würsig. 2004. A small resident community of bottlenose dolphins, *Tursiops truncatus*, in Texas: Monitoring recommendations. Gulf of Mexico Science 22(1):13–21. doi: 10.18785/goms.2201.02

Kellar, N.M., T.R. Speakman, C.R. Smith, S.M. Lane, B.C. Balmer, M.L. Trego, K.N. Catelani, M.N. Robbins, C.D. Allen, R.S. Wells, E.S. Zolman, T.K. Rowles, and L.H. Schwacke. 2017. Low reproductive success rates of common bottlenose dolphins *Tursiops truncatus* in the northern Gulf of Mexico following the Deepwater Horizon disaster (2010-2015). Endangered Species Research 33:143–158. doi: 10.3354/esr00775

Kucklick, J., L. Schwacke, R. Wells, A. Hohn, A. Guichard, J. Yordy, L. Hansen, E. Zolman, R. Wilson, J. Litz, D. Nowacek, T. Rowles, R. Pugh, B. Balmer, C. Sinclair, and P. Rosel. 2011. Bottlenose dolphins as indicators of persistent organic pollutants in the western North Atlantic Ocean and Northern Gulf of Mexico. Environmental Science & Technology 45(10):4270–4277. doi: 10.1021/es1042244

LaBrecque, E., C. Curtice, J. Harrison, S.M. Van Parijs, and P.N. Halpin. 2015. 3. Biologically Important Areas for cetaceans within U.S. Waters – Gulf of Mexico Region. Aquatic Mammals 41(1):30–38. doi: 10.1578/am.41.1.2015.30

Lane, S.M., C.R. Smith, J. Mitchell, B.C. Balmer, K.P. Barry, T. McDonald, C.S. Mori, P.E. Rosel, T.K. Rowles, T.R. Speakman, F.I. Townsend, M.C. Tumlin, R.S. Wells, E.S. Zolman, and L.H. Schwacke. 2015. Reproductive outcome and survival of common bottlenose dolphins sampled in Barataria Bay, Louisiana, USA, following the Deepwater Horizon oil spill. Proceedings of the Royal Society B: Biological Sciences 282(1818):20151944. doi: 10.1098/rspb.2015.1944

Leatherwood, S. 1977. Some preliminary impressions on the numbers and social behavior of free-swimming bottlenosed dolphin calves (*Tursiops truncatus*) in the northern Gulf of Mexico. In: Ridgway, S.H. and K. Bernirschke (Eds.), Breeding Dolphins; Present Status, Suggestions for the Future. Final report to the US Marine Mammal Commission, Contract MM6AC009, MMC-76/07, Washington, DC. pp. 143–167.

Lipscomb, T.P., S. Kennedy, D. Moffett, A. Krafft, B.A. Klaunberg, J.H. Lichy, G.T. Regan, G.A.J. Worthy, and J.K. Taubenberger. 1996. Morbilliviral epizootic in bottlenose dolphins of the Gulf of Mexico. Journal of Veterinary Diagnostic Investigation 8(3):283–290. doi: 10.1177/104063879600800302

Litz, J.A., M.A. Baran, S.R. Bowen-Stevens, R.H. Carmichael, K.M. Colegrove, L.P. Garrison, S.E. Fire, E.M. Fougeres, R. Hardy, S. Holmes, W. Jones, B.E. Mase-Guthrie, D.K. Odell, P.E. Rosel, J.T. Saliki, D.K. Shannon, S.F. Shippee, S.M. Smith, E.M. Stratton, M.C. Tumlin, H.R. Whitehead, G.A. Worthy, and T.K. Rowles. 2014. Review of historical unusual mortality events (UMEs) in the Gulf of Mexico (1990–2009): providing context for the multi-year northern Gulf of Mexico cetacean UME declared in 2010. Diseases of Aquatic Organisms 112(2):161–175. doi: 10.3354/dao02807

Lynn, S.K. and B. Würsig. 2002. Summer movement patterns of bottlenose dolphins in a Texas bay. Gulf of Mexico Science 20(1):25–37. doi: 10.18785/goms.2001.03

Mackey, A.D. 2010. Site fidelity and association patterns of bottlenose dolphins (*Tursiops truncatus*) in the Mississippi Sound. University of Southern Mississippi. Thesis. 133 p.

Mann, J., R.C. Connor, L.M. Barre, and M.R. Heithaus. 2000. Female reproductive success in bottlenose dolphins (*Tursiops* sp.): life history, habitat, provisioning, and group-size effects. Behavioral Ecology 11(2):210–219. doi: 10.1093/beheco/11.2.210

Mattson, M.C., K.D. Mullin, G.W. Ingram, and W. Hoggard. 2006. Age structure and growth of the bottlenose dolphin (*Tursiops truncatus*) from strandings in the Mississippi sound region of the north-central Gulf of Mexico from 1986 to 2003. Marine Mammal Science 22(3):654–666. doi: 10.1111/j.1748-7692.2006.00057.x

Maze, K.S. and B. Würsig. 1999. Bottlenosed dolphins of San Luis Pass, Texas: Occurrence patterns, site-fidelity, and habitat use. Aquatic Mammals 25:91–104.

McCormack, M.A., F. Battaglia, W.E. McFee, and J. Dutton. 2020a. Mercury concentrations in blubber and skin from stranded bottlenose dolphins (*Tursiops truncatus*) along the Florida and Louisiana coasts (Gulf of Mexico, USA) in relation to biological variables. Environmental Research 180:108886. doi: 10.1016/j.envres.2019.108886

McCormack, M.A., B.P. Jackson, and J. Dutton. 2020b. Relationship between mercury and selenium concentrations in tissues from stranded odontocetes in the northern Gulf of Mexico. Science of the Total Environment 749:141350. doi: 10.1016/j.scitotenv.2020.141350

McCormack, M.A., W.H. Nowlin, and J. Dutton. 2022. Effect of trophic position on mercury concentrations in bottlenose dolphins (*Tursiops truncatus*) from the northern Gulf of Mexico. Environmental Research 204(Pt B):112124. doi: 10.1016/j.envres.2021.112124

McDonald, T.L., F.E. Hornsby, T.R. Speakman, E.S. Zolman, K.D. Mullin, C. Sinclair, P.E. Rosel, L. Thomas, and L.H. Schwacke. 2017. Survival, density, and abundance of common bottlenose dolphins in Barataria Bay (USA) following the Deepwater Horizon oil spill. Endangered Species Research 33:193–209. doi: 10.3354/esr00806

McHugh, M.B. 1989. Population numbers and feeding behavior of the Atlantic bottlenose dolphin (*Tursiops truncatus*) near Aransas Pass, Texas. University of Texas at Austin. Thesis. 96 p.

Mead, J.G. and C.W. Potter. 1990. Natural history of bottlenose dolphins along the central Atlantic coast of the United States. In: Leatherwood, S. and R.R. Reeves (Eds.), The Bottlenose Dolphin. Academic Press, San Diego, CA. pp. 165–195.

Michel, J., E.H. Owens, S. Zengel, A. Graham, Z. Nixon, T. Allard, W. Holton, P.D. Reimer, A. Lamarche, M. White, N. Rutherford, C. Childs, G. Mauseth, G. Challenger, and E. Taylor. 2013. Extent and degree of shoreline oiling: Deepwater Horizon oil spill, Gulf of Mexico, USA. PLoS ONE 8(6):e65087. doi: 10.1371/journal.pone.0065087

Miller, C.E. 2003. Abundance trends and environmental habitat usage patterns of bottlenose dolphins (*Tursiops truncatus*) in lower Barataria and Caminada bays, Louisiana. Louisiana State University and Agricultural & Mechanical College. Dissertation. 125 p.

Miller, D.L., V. Woshner, E.L. Styer, S. Ferguson, K.K. Knott, M.J. Gray, R.S. Wells, and T.M. O'Hara. 2011. Histologic findings in free-ranging Sarasota Bay bottlenose dolphin (*Tursiops truncatus*) skin: mercury, selenium, and seasonal factors. Journal of Wildlife Diseases 47(4):1012–1018. doi: 10.7589/0090-3558-47.4.1012

Miller, L.J., A.D. Mackey, T. Hoffland, M. Solangi, and S.A. Kuczaj. 2010. Potential effects of a major hurricane on Atlantic bottlenose dolphin (*Tursiops truncatus*) reproduction in the Mississippi Sound. Marine Mammal Science 26(3):707–715. doi: 10.1111/j.1748-7692.2010.00371.x

Miller, L.J., A.D. Mackey, M. Solangi, and S.A. Kuczaj. 2013. Population abundance and habitat utilization of bottlenose dolphins in the Mississippi Sound. Aquatic Conservation: Marine and Freshwater Ecosystems 23(1):145–151. doi: 10.1002/aqc.2278

Mullin, K.D., T. McDonald, R.S. Wells, B.C. Balmer, T. Speakman, C. Sinclair, E.S. Zolman, F. Hornsby, S.M. McBride, K.A. Wilkinson, and L.H. Schwacke. 2017. Density, abundance, survival, and ranging patterns of common bottlenose dolphins (*Tursiops truncatus*) in Mississippi Sound following the Deepwater Horizon oil spill. PLoS ONE 12(10):e0186265. doi: 10.1371/journal.pone.0186265

National Oceanic and Atmospheric Administration (NOAA). 2011. Public scoping for preparation of a programmatic environmental impact statement for the Deepwater Horizon BP Oil Spill. 9 p.

Perrin, W.F. and S.B. Reilly. 1984. Reproductive parameters of dolphins and small whales of the family Delphinidae. In: Perrin, W.F., R.L. Brownell, Jr., and D.P. DeMaster (Eds.), Reproduction in Whales, Dolphins, and Porpoises. pp. 97–134.

Rosel, P.E., L.A. Wilcox, C. Sinclair, T.R. Speakman, M.C. Tumlin, J.A. Litz, and E.S. Zolman. 2017. Genetic assignment to stock of stranded common bottlenose dolphins in southeastern Louisiana after

the Deepwater Horizon oil spill. Endangered Species Research 33:221–234. doi: 10.3354/esr00780
Rossman, S., P.H. Ostrom, M. Stolen, N.B. Barros, H. Gandhi, C.A. Stricker, and R.S. Wells. 2015. Individual specialization in the foraging habits of female bottlenose dolphins living in a trophically diverse and habitat rich estuary. Oecologia 178(2):415–425. doi: 10.1007/s00442-015-3241-6
Schwacke, L.H., C.R. Smith, F.I. Townsend, R.S. Wells, L.B. Hart, B.C. Balmer, T.K. Collier, S. De Guise, M.M. Fry, L.J. Guillette, Jr., S.V. Lamb, S.M. Lane, W.E. McFee, N.J. Place, M.C. Tumlin, G.M. Ylitalo, E.S. Zolman, and T.K. Rowles. 2014. Health of common bottlenose dolphins (*Tursiops truncatus*) in Barataria Bay, Louisiana, following the deepwater horizon oil spill. Environmental Science & Technology 48(1):93–103. doi: 10.1021/es403610f
Schwacke, L.H., L. Thomas, R.S. Wells, W.E. McFee, A.A. Hohn, K.D. Mullin, E.S. Zolman, B.M. Quigley, T.K. Rowles, and J.H. Schwacke. 2017. Quantifying injury to common bottlenose dolphins from the Deepwater Horizon oil spill using an age-, sex- and class-structured population model. Endangered Species Research 33:265–279. doi: 10.3354/esr00777
Scott, M.D. and S.J. Chivers. 1990. Distribution and herd structure of bottlenose dolphins in the eastern tropical Pacific Ocean. In: Leatherwood, S. and R.R. Reeves (Eds.), The Bottlenose Dolphin. Academic Press, San Diego, CA. pp. 387–402.
Sellas, A.B., R.S. Wells, and P.E. Rosel. 2005. Mitochondrial and nuclear DNA analyses reveal fine scale geographic structure in bottlenose dolphins (*Tursiops truncatus*) in the Gulf of Mexico. Conservation Genetics 6(5):715–728. doi: 10.1007/s10592-005-9031-7
Sergeant, D.E., D.K. Caldwell, and M.C. Caldwell. 1973. Age, growth, and maturity of bottlenosed dolphin (*Tursiops-truncatus*) from northeast Florida. Journal of the Fisheries Research Board of Canada 30(7):1009–1011. doi: 10.1139/f73-165
Shane, S.H. 1990. Behavior and ecology of the bottlenose dolphin at Sanibel Island, Florida. In: Leatherwood, S. and R.R. Reeves (Eds.), The Bottlenose Dolphin. Academic Press, San Diego, CA. pp. 245–265. doi: 10.1016/B978-0-12-440280-5.50016-0
Shane, S.H. 2004. Residence patterns, group characteristics, and association patterns of bottlenose dolphins near Sanibel Island, Florida. Gulf of Mexico Science 22(1):1–12. doi: 10.18785/goms.2201.01
Shane, S.H., R.S. Wells, and B. Würsig. 1986. Ecology, behavior and social organization of the bottlenose dolphin: A review. Marine Mammal Science 2:34–63. doi: 10.1111/j.1748-7692.1986.tb00026.x
Smith, C.R., T.K. Rowles, L.B. Hart, F.I. Townsend, R.S. Wells, E.S. Zolman, B.C. Balmer, B. Quigley, M. Ivančić, W. McKercher, M.C. Tumlin, K.D. Mullin, J.D. Adams, Q. Wu, W. McFee, T.K. Collier, and L.H. Schwacke. 2017. Slow recovery of Barataria Bay dolphin health following the Deepwater Horizon oil spill (2013-2014), with evidence of persistent lung disease and impaired stress response. Endangered Species Research 33:127–142. doi: 10.3354/esr00778
Takeshita, R., B.C. Balmer, F. Messina, E.S. Zolman, L. Thomas, R.S. Wells, C.R. Smith, T.K. Rowles, and L.H. Schwacke. 2021. High site-fidelity in common bottlenose dolphins despite low salinity exposure and associated indicators of compromised health. PLoS ONE 16(9):e0258031. doi: 10.1371/journal.pone.0258031
Taylor, B.L., S.J. Chivers, J. Larese, and W.F. Perrin. 2007. Generation length and percent mature estimates for IUCN assessments of cetaceans. National Oceanic and Atmospheric Administration, National Marine Fisheries Service, Southwest Fisheries Science Center: La Jolla, CA. Administrative Report LJ-07-01. 24 p.
Toms, C. 2019. Filling the gaps: Common bottlenose dolphin (*Tursiops truncatus*) population dynamics, structure, and connectivity within Florida panhandle bays, sounds, and estuaries. University of Central Florida. Dissertation. 373 p.

Torres, L.G. and A.J. Read. 2009. Where to catch a fish? The influence of foraging tactics on the ecology of bottlenose dolphins (*Tursiops truncatus*) in Florida Bay, Florida. Marine Mammal Science 25(4):797–815. doi: 10.1111/j.1748-7692.2009.00297.x

Twiner, M.J., L.J. Flewelling, S.E. Fire, S.R. Bowen-Stevens, J.K. Gaydos, C.K. Johnson, J.H. Landsberg, T.A. Leighfield, B. Mase-Guthrie, L. Schwacke, F.M. Van Dolah, Z. Wang, and T.K. Rowles. 2012. Comparative analysis of three brevetoxin-associated bottlenose dolphin (*Tursiops truncatus*) mortality events in the Florida Panhandle region (USA). PLoS ONE 7(8):e42974. doi: 10.1371/journal.pone.0042974

Tyson, R.B., S.M. Nowacek, and D.P. Nowacek. 2011. Community structure and abundance of bottlenose dolphins *Tursiops truncatus* in coastal waters of the northeast Gulf of Mexico. Marine Ecology Progress Series 438:253–265. doi: 10.3354/meps09292

Urian, K.W., S. Hofmann, R.S. Wells, and A.J. Read. 2009. Fine-scale population structure of bottlenose dolphins (*Tursiops truncatus*) in Tampa Bay, Florida. Marine Mammal Science 25(3):619–638. doi: 10.1111/j.1748-7692.2009.00284.x

Van Bressem, M.F., P.J. Duignan, A. Banyard, M. Barbieri, K.M. Colegrove, S. De Guise, G. Di Guardo, A. Dobson, M. Domingo, D. Fauquier, A. Fernandez, T. Goldstein, B. Grenfell, K.R. Groch, F. Gulland, B.A. Jensen, P.D. Jepson, A. Hall, T. Kuiken, S. Mazzariol, S.E. Morris, O. Nielsen, J.A. Raga, T.K. Rowles, J. Saliki, E. Sierra, N. Stephens, B. Stone, I. Tomo, J. Wang, T. Waltzek, and J.F. Wellehan. 2014. Cetacean morbillivirus: current knowledge and future directions. Viruses 6(12):5145–5181. doi: 10.3390/v6125145

Venn-Watson, S., L. Garrison, J. Litz, E. Fougeres, B. Mase, G. Rappucci, E. Stratton, R. Carmichael, D. Odell, D. Shannon, S. Shippee, S. Smith, L. Staggs, M. Tumlin, H. Whitehead, and T. Rowles. 2015. Demographic clusters identified within the northern Gulf of Mexico common bottlenose dolphin (*Tursiops truncates*) unusual mortality event: January 2010-June 2013. PLoS ONE 10(2):e0117248. doi: 10.1371/journal.pone.0117248

Vollmer, N.L. and P.E. Rosel. 2013. A review of common bottlenose dolphins (*Tursiops truncatus truncatus*) in the Northern Gulf of Mexico: Population biology, potential threats, and management. Southeastern Naturalist 12(m6):1–43. doi: 10.1656/058.012.m601

Weller, D.W. 1998. Global and regional variation in the biology and behavior of bottlenose dolphins. Texas A&M University. Dissertation. 142 p.

Wells, R.S. 1986a. Population structure of bottlenose dolphins: Behavioral studies along the central west coast of Florida. Contract Report to National Marine Fisheries Service, Southeast Fisheries Center. Contract No. 45-WCNF-5-003BB. 58 p.

Wells, R.S. 1986b. Structural aspects of dolphin societies. University of California, Santa Cruz. Dissertation. 234 p.

Wells, R.S. 1991. The role of long-term study in understanding the social structure of a bottlenose dolphin community. In: Pryor, K. and K. Norris (Eds.), Dolphin Societies: Discoveries and Puzzles. University of California Press, Berkeley, CA. pp. 199–226.

Wells, R.S. 2000. Reproduction in wild bottlenose dolphins: Overview of patterns observed during a long-term study. In: Duffield, D. and T. Robeck (Eds.), Bottlenose Dolphin Reproduction Workshop. AZA Marine Mammal Taxon Advisory Group, Silver Spring, MD. pp. 57–74.

Wells, R.S. 2003. Dolphin social complexity: Lessons from long-term study and life history. In: de Waal, F.B.M. and P.L. Tyack (Eds.), Animal Social Complexity: Intelligence, Culture, and Individualized Societies Harvard University Press, Cambridge, MA. pp. 32–56.

Wells, R.S. 2014. Social structure and life history of bottlenose dolphins near Sarasota Bay, Florida: Insights from four decades and five generations. Primates and cetaceans. pp. 149–172. doi: 10.1007/978-4-431-54523-1_8

Wells, R.S., M.K. Bassos, K.W. Urian, W.J. Carr, and M.D. Scott. 1996. Low-level monitoring of bottlenose dolphins, *Tursiops truncatus*, in Charlotte Harbor, Florida: 1990-1994. National Oceanic and Atmospheric Administration, National Marine Fisheries Service, Southeast Fisheries Science Center: Miami, FL. NOAA Technical Memorandum NMFS-SEFSC-384. 86 p.

Wells, R.S., M.K. Bassos, K.W. Urian, S.H. Shane, E.C.G. Owen, C.F. Weiss, W.J. Carr, and M.D. Scott. 1997. Low-level monitoring of bottlenose dolphins, *Tursiops truncatus*, in Pine Island Sound, Florida: 1996. National Oceanic and Atmospheric Administration, National Marine Fisheries Service, Southeast Fisheries Science Center: Miami, FL. Southeast Fisheries Center Contribution No. 40-WCNF601958. 86 p.

Wells, R.S., A. Natoli, and G. Braulik. 2019. *Tursiops truncatus* (errata version published in 2019). *The IUCN Red List of Threatened Species* 2019: e.T22563A156932432. https://dx.doi.org/10.2305/IUCN.UK.2019-1.RLTS.T22563A156932432.en

Wells, R.S., L.H. Schwacke, T.K. Rowles, B.C. Balmer, E. Zolman, T. Speakman, F.I. Townsend, M.C. Tumlin, A. Barleycorn, and K.A. Wilkinson. 2017. Ranging patterns of common bottlenose dolphins *Tursiops truncatus* in Barataria Bay, Louisiana, following the Deepwater Horizon oil spill. Endangered Species Research 33:159–180. doi: 10.3354/esr00732

Wells, R.S. and M.D. Scott. 1999. Bottlenose dolphin *Tursiops truncatus* (Montagu, 1821). In: Ridgway, S.H. and R. Harrison (Eds.), Handbook of marine mammals. Volume 6: The second book of dolphins and the porpoises. Academic Press, San Diego, CA. pp. 137–182.

Wells, R.S. and M.D. Scott. 2018. Bottlenose dolphin, *Tursiops truncatus*, common bottlenose dolphin. In: Würsig, B., J.G.M. Thewissen, and K.M. Kovacs (Eds.), Encyclopedia of Marine Mammals (Third Edition). Academic Press, London, UK. pp. 118–125. doi: 10.1016/B978-0-12-804327-1.00072-8

Wells, R.S., M.D. Scott, and A.B. Irvine. 1987. The social structure of free-ranging bottlenose dolphins. In: Genoways, H.H. (Ed.), Current Mammalogy. Plenum Press, New York, NY. pp. 247–305.

Wells, R.S., K.W. Urian, A.J. Read, M.K. Bassos, W.J. Carr, and M.D. Scott. 1996. Low-level monitoring of bottlenose dolphins, *Tursiops truncatus*, in Tampa Bay, Florida: 1988-1993. National Oceanic and Atmospheric Administration, National Marine Fisheries Service, Southeast Fisheries Science Center: Miami, FL. NOAA Technical Memorandum NMFS-SEFSC-385. 36 p.

Wilson, R.M., J.A. Nelson, B.C. Balmer, D.P. Nowacek, and J.P. Chanton. 2013. Stable isotope variation in the northern Gulf of Mexico constrains bottlenose dolphin (*Tursiops truncatus*) foraging ranges. Marine Biology 160(11):2967–2980. doi: 10.1007/s00227-013-2287-4

Wilson, R.M., R.B. Tyson, J.A. Nelson, B.C. Balmer, J.P. Chanton, and D.P. Nowacek. 2017. Niche differentiation and prey selectivity among common bottlenose dolphins (*Tursiops truncatus*) sighted in St. George Sound, Gulf of Mexico. Frontiers in Marine Science 4:235. doi: 10.3389/fmars.2017.00235

Woshner, V., K. Knott, R. Wells, C. Willetto, R. Swor, and T. O'Hara. 2008. Mercury and selenium in blood and epidermis of bottlenose dolphins (*Tursiops truncatus*) from Sarasota Bay, FL: interaction and relevance to life history and hematologic parameters. Ecohealth 5(3):360–370. doi: 10.1007/s10393-008-0164-2

Yordy, J.E., M.A. Mollenhauer, R.M. Wilson, R.S. Wells, A. Hohn, J. Sweeney, L.H. Schwacke, T.K. Rowles, J.R. Kucklick, and M.M. Peden-Adams. 2010a. Complex contaminant exposure in cetaceans: a comparative E-Screen analysis of bottlenose dolphin blubber and mixtures of four persistent organic pollutants. Environmental Toxicology and Chemistry 29(10):2143–2153. doi: 10.1002/etc.279

Yordy, J.E., D.A. Pabst, W.A. McLellan, R.S. Wells, T.K. Rowles, and J.R. Kucklick. 2010b. Tissue-specific distribution and whole-body burden estimates of persistent organic pollutants in the bottlenose

dolphin (*Tursiops truncatus*). Environmental Toxicology and Chemistry 29(6):1263–1273. doi: 10.1002/etc.152

Yordy, J.E., R.S. Wells, B.C. Balmer, L.H. Schwacke, T.K. Rowles, and J.R. Kucklick. 2010c. Life history as a source of variation for persistent organic pollutant (POP) patterns in a community of common bottlenose dolphins (*Tursiops truncatus*) resident to Sarasota Bay, FL. Science of the Total Environment 408(9):2163–2172. doi: 10.1016/j.scitotenv.2010.01.032

Common bottlenose dolphin − *Tursiops truncatus*

GoMx BSE/Tampa Bay Stock

Overall Vulnerability Rank = Very High

Biological Sensitivity = Very High

Climate Exposure = Very High

Data Quality = 93% of scores ≥ 2

	Tursiops truncatus	Expert Scores	Data Quality	Expert Scores Plots (Portion by Category)
Sensitivity attributes	Prey/Diet Specificity	1.7	2.3	
	Habitat Specificity	3.7	3.0	
	Site Fidelity	3.9	3.0	
	Lifetime Reproductive Potential	1.6	2.3	
	Generation Length	2.9	2.7	
	Reproductive Plasticity	1.5	2.3	
	Migration	3.9	3.0	
	Home Range	3.7	3.0	
	Stock Abundance	3.0	1.7	
	Stock Abundance Trend	2.1	1.3	
	Cumulative Stressors	3.7	2.3	
	Sensitivity Score	Very High		
Exposure variables	Sea Surface Temperature (Change in mean)	4.0	3.0	
	Sea Surface Temperature (Change in variability)	1.0	3.0	
	Air Temperature (Change in mean)	4.0	3.0	
	Air Temperature (Change in variability)	1.0	3.0	
	Precipitation (Change in mean)	1.0	3.0	
	Precipitation (Change in variability)	1.0	3.0	
	Sea Surface Salinity (Change in mean)	1.0	3.0	
	Sea Surface Salinity (Change in variability)	2.0	3.0	
	Ocean pH (Change in mean)	4.0	3.0	
	Ocean pH (Change in variability)	1.0	3.0	
	Sea ice coverage (Change in mean)	1.0	3.0	
	Sea ice coverage (Change in variability)	1.0	3.0	
	Dissolved oxygen (Change in mean)	4.0	3.0	
	Dissolved oxygen (Change in variability)	1.0	3.0	
	Circulation	3.9	2.7	
	Sea level rise	4.0	3.0	
	Exposure Score	Very High		
Overall Vulnerability Rank		Very High		

Low Moderate High Very High

Common bottlenose dolphin (GoMx BSE/Tampa Bay Stock)

Tursiops truncatus

CVA Results Summary

Overall Climate Vulnerability Rank: Very High (100% certainty from bootstrap analysis).

Climate Exposure: Very High. Six exposure factors scored greater than 3.5: Air Temperature (Standard anomaly) (4.00), Dissolved oxygen (Standard anomaly) (4.00), Ocean pH (Standard anomaly) (4.00), Sea level rise (4.00), Sea Surface Temperature (Standard anomaly) (4.00), and Circulation (3.93).

Biological Sensitivity: Very High. Five sensitivity attributes scored greater than or equal to 3.5: Migration (3.93), Site Fidelity (3.93), Habitat Specificity (3.73), Cumulative Stressors (3.67), and Home Range (3.67)

Distributional Response: Very High

Abundance Response: Very High

Phenology Response: Very High

Data Quality: 93% of the data quality scores were 2 or greater, 82% of sensitivity attribute data quality scores were 2 or higher.

Background Information

Stock Description

This stock includes common bottlenose dolphins in Tampa Bay in the eastern Gulf of Mexico along the Florida coast (Hayes et al. 2022).

Prey/Diet Specificity

Generally, common bottlenose dolphins consume a large variety of fish and/or squid (Barros and Odell 1990; Mead and Potter 1990; Wells and Scott 2018). Common bottlenose dolphins seem to show a consistent preference for sound-producing fishes (e.g., sciaenids, toadfish; Berens McCabe et al. 2010; Bowen-Stevens et al. 2021). In Sarasota Bay, Florida, Rossman et al. (2015) found some individual common bottlenose dolphins to show diet specialization despite the broader study group showing a generalist diet. Specialization can be influenced by locally abundant prey species (Shane et al. 1986; Vollmer and Rosel 2013) or can result from resource partitioning amongst sympatric populations (e.g., Torres and Read 2009; Wilson et al. 2017).

Habitat Specificity

Common bottlenose dolphins are found in a variety of marine and estuarine habitats (Wells and Scott 2018).

Site Fidelity

Bottlenose dolphins in Tampa Bay have been shown to demonstrate strong site fidelity (Wells et al. 1996; Urian et al. 2009). Common bottlenose dolphins in other Gulf of Mexico bay, sound, and estuary (BSE) stocks show site fidelity (LaBrecque et al. 2015; Hayes et al. 2022), such as in Charlotte Harbor and Pine Island Sound (Bassos-Hull et al. 2013), Sarasota Bay (Wells 1986a; Wells 1991; Wells 2014), St. Andrew Bay (Balmer et al. 2019), St. Joseph Bay (Balmer et al. 2008; B. Balmer et al. 2018), and St. Vincent Sound and Apalachicola Bay (Tyson et al. 2011); Matagorda Bay, Espiritu Santo Bay, and San Luis Pass in Texas (Maze and Würsig 1999; Lynn and Würsig 2002; Irwin and Würsig 2004); Barataria Bay in Louisiana (Miller 2003; McDonald et al. 2017; Wells et al. 2017); and Mississippi Sound in Mississippi (Hubard et al. 2004; Mackey 2010; Mullin et al. 2017; Takeshita et al. 2021).

Lifetime Reproductive Potential

Taylor et al. (2007) reported the interbirth interval of common bottlenose dolphins as 3.80 years, though other studies have shown it to be closer to three years (Leatherwood 1977; Wells and Scott 1999; Connor et al. 2000; Wells 2003).

Generally, females reach sexual maturity before males, with females reaching sexual maturity at 5–13 years (Sergeant et al. 1973; Perrin and Reilly 1984; Wells et al. 1987; Mead and Potter 1990; Mann et al. 2000; Wells 2000; Wells and Scott 2018).

Females reproduce through most of their adult lives (Leatherwood 1977; Hohn et al. 1989; Wells and Scott 1999; Wells 2000; Wells 2003), but may produce fewer calves after they reach 30 years of age (Wells 2000). Sarasota Bay common bottlenose dolphins can give birth when they are as young as 6 years of age, and can continue until they are 48 years old (Wells and Scott 2018). Reproductive lifespan would therefore be up to 42 years based on long-term observation studies (Wells 2014; Wells and Scott 2018).

Longitudinal studies from Sarasota Bay, Florida have found that female common bottlenose dolphins can live up to 67 years (Wells and Scott 2018). Studies based on growth layer groups in teeth suggest female common bottlenose dolphins can live to 41 years along the Texas coast (Fernandez and Hohn 1998) and to 30 years along the Mississippi coast (Mattson et al. 2006).

Generation Length

Taylor et al. (2007) reported common bottlenose dolphin generation length of 20.6–21.1 years.

Reproductive Plasticity

Common bottlenose dolphin calving is generally diffusely seasonal, with one or more peaks, although births have been reported from all seasons (Vollmer and Rosel 2013; Wells and Scott 2018). Calf sightings peak in the spring and summer months in Florida (Wells et al. 1987), Mississippi/Louisiana (Miller et al. 2010; Miller et al. 2013), and Texas (McHugh 1989; Henderson et al. 2007).

Migration

The BSE stocks in the Gulf of Mexico are thought to primarily comprise stable resident communities. However, transient or seasonal movements of some individuals into and out of a BSE stock area are recorded in some areas (LaBrecque et al. 2015; Hayes et al. 2022). Molecular genetic data support that some BSE stocks are genetically distinct from one another, and furthermore are differentiated from adjacent coastal populations (e.g., Sellas et al. 2005; Rosel et al. 2017). For example, Toms (2019) found fine-scale genetic population structure among five inshore systems in the Florida Panhandle and variable migration rates among populations.

Year-round residency has been reported for common bottlenose dolphins (Hayes et al. 2022), such as in Florida (Irvine and Wells 1972; Irvine et al. 1981; Wells 1986a; Wells 1986b; Wells et al. 1987; Scott et al. 1990; Wells 1991; Wells et al. 1996a; Wells et al. 1996b; Wells et al. 1997; Wells 2003; Balmer et al. 2008; Urian et al. 2009; Tyson et al. 2011; Bassos-Hull et al. 2013; B. Balmer et al. 2018; Balmer et al. 2019; Toms 2019), Texas (Shane 1990; Bräger 1993; Bräger et al. 1994; Fertl 1994; Weller 1998; Maze and Würsig 1999; Lynn and Würsig 2002; Irwin and Würsig 2004; Shane 2004), and Louisiana/Mississippi (Hubard et al. 2004; McDonald et al. 2017; Mullin et al. 2017).

Home Range

The home ranges of individuals within the Gulf of Mexico BSE stocks are considered to generally include the areas within the stock boundaries and brief excursions beyond the stock boundaries (Irvine et al. 1981; Wells 1986a; Wells 1986b; Wells 1991; Wells et al. 1996a; Wells et al. 1996b; Balmer et al. 2008; Wells 2014; Balmer et al. 2019), although some individuals show preference for small areas within the stock boundaries (Lynn and Würsig 2002; Urian et al. 2009; Mullin et al. 2017; Wells et al. 2017; Cloyed et al. 2021).

Stock Abundance

An abundance estimate for this stock is unavailable (Hayes et al. 2022).

Stock Abundance Trend

Data are insufficient to complete an abundance trend analysis for this stock (Hayes et al. 2022).

Cumulative Stressors

Common bottlenose dolphins face a wide range of individual and cumulative natural and anthropogenic threats (see reviews by Vollmer and Rosel 2013; Wells and Scott 2018; and Wells et al. 2019). These include such threats as shark predation, harmful algal blooms, recreational and commercial fishing gear (McHugh et al. 2021), vessel traffic (MacQueeney et al. 2022), pollution, habitat alteration, and provisioning.

The nearshore and estuarine habitats along the Gulf of Mexico coast include waters adjacent to areas of high human population, some of which are highly industrialized, and areas of heavy agricultural impact. Studies of common bottlenose dolphin tissues from nearby Sarasota Bay have shown persistent organic pollutants (POPs) in blubber and blood samples (Yordy et al. 2010a, 2010b, 2010c; Kucklick et al. 2011; J. Balmer et al. 2018). Similarly, mercury has been detected in common bottlenose dolphin tissues in

Florida Gulf Coast waters (Bryan et al. 2007; Woshner et al. 2008; Miller et al. 2011; Correa et al. 2013; McCormack et al. 2020a, 2020b, 2022).

An Unusual Mortality Event (UME) was declared in 1990 for the Gulf of Mexico from Florida to Texas, with more than 300 common bottlenose dolphins stranding due to an undetermined cause, although morbillivirus and cold temperatures may have contributed (Litz et al. 2014). This stock was also within the range of the 2005–2006 UME in central west Florida caused by brevetoxin (Litz et al. 2014).

Further Reading

Hayes, S.A., E. Josephson, K. Maze-Foley, P.E. Rosel, and J. Wallace. 2022. U.S. Atlantic and Gulf of Mexico marine mammal stock assessments 2021. National Oceanic and Atmospheric Administration, National Marine Fisheries Service, Northeast Fisheries Science Center: Woods Hole, MA. NOAA Technical Memorandum NMFS-NE-288. 380 p. doi: 10.25923/6tt7-kc16

Wells, R.S. and M.D. Scott. 2018. Bottlenose Dolphin, *Tursiops truncatus*, Common Bottlenose Dolphin. In: Würsig, B., J.G.M. Thewissen, and K.M. Kovacs (Eds.), Encyclopedia of Marine Mammals (Third Edition). Academic Press, London, UK. pp. 118–125. doi: 10.1016/B978-0-12-804327-1.00072-8

Wells, R.S., A. Natoli, and G. Braulik. 2019. *Tursiops truncatus* (errata version published in 2019). The IUCN Red List of Threatened Species 2019: e.T22563A156932432. https://dx.doi.org/10.2305/IUCN.UK.2019-1.RLTS.T22563A156932432.en

References

Balmer, B., T. McDonald, F. Hornsby, J. Adams, J. Allen, A. Barleycorn, and L. Schwacke. 2018. Long-term trends in a northern Gulf of Mexico common bottlenose dolphin (*Tursiops truncatus*) population in the wake of the Deepwater Horizon oil spill. Journal of Cetacean Research and Management 18:1–9. doi: 10.47536/jcrm.v18i1.409

Balmer, B., S. Watwood, B. Quigley, T. Speakman, K. Barry, K. Mullin, P. Rosel, C. Sinclair, E. Zolman, and L. Schwacke. 2019. Common bottlenose dolphin (*Tursiops truncatus*) abundance and distribution patterns in St. Andrew Bay, Florida, USA. Aquatic Conservation: Marine and Freshwater Ecosystems 29(3):486–498. doi: 10.1002/aqc.3001

Balmer, B.C., R.S. Wells, S.M. Nowacek, D.P. Nowacek, L.H. Schwacke, W.A. McLellan, F.S. Scharf, T.K. Rowles, L.J. Hansen, T.R. Spradlin, and D.A. Pabst. 2008. Seasonal abundance and distribution patterns of common bottlenose dolphins (*Tursiops truncatus*) near St. Joseph Bay, Florida, USA. Journal of Cetacean Research and Management 10(2):157–167.

Balmer, J.E., G.M. Ylitalo, T.K. Rowles, K.D. Mullin, R.S. Wells, F.I. Townsend, R.W. Pearce, J.L. Bolton, E.S. Zolman, B.C. Balmer, and L.H. Schwacke. 2018. Persistent organic pollutants (POPs) in blood and blubber of common bottlenose dolphins (*Tursiops truncatus*) at three northern Gulf of Mexico sites following the Deepwater Horizon oil spill. Science of the Total Environment 621:130–137. doi: 10.1016/j.scitotenv.2017.11.209

Barros, N.B. and D.K. Odell. 1990. Food habits of bottlenose dolphins in the southeastern United States In: Leatherwood, S. and R.R. Reeves (Eds.), The Bottlenose Dolphin. Academic Press, San Diego, CA. pp. 309–328. doi: 10.1016/B978-0-12-440280-5.50020-2

Bassos-Hull, K., R.M. Perrtree, C.C. Shepard, S. Schilling, A.A. Barleycorn, J.B. Allen, B.C. Balmer, W.E. Pine, and R.S. Wells. 2013. Long-term site fidelity and seasonal abundance estimates of common bottlenose dolphins (*Tursiops truncatus*) along the southwest coast of Florida and responses to

natural perturbations. Journal of Cetacean Research and Management 13(1):19–30. doi: 10.47536/jcrm.v13i1.551

Berens McCabe, E.J., D.P. Gannon, N.B. Barros, and R.S. Wells. 2010. Prey selection by resident common bottlenose dolphins (*Tursiops truncatus*) in Sarasota Bay, Florida. Marine Biology 157(5):931–942. doi: 10.1007/s00227-009-1371-2

Bowen-Stevens, S.R., D.P. Gannon, R.A. Hazelkorn, G. Lovewell, K.M. Volker, S. Smith, M.C. Tumlin, and J. Litz. 2021. Diet of common bottlenose dolphins, *Tursiops truncatus*, that stranded in and near Barataria Bay, Louisiana, 2010–2012. Southeastern Naturalist 20(1). doi: 10.1656/058.020.0113

Bräger, S. 1993. Diurnal and seasonal behavior patterns of bottlenose dolphins (*Tursiops truncatus*). Marine Mammal Science 9:434–438. doi: 10.1111/j.1748-7692.1993.tb00477.x

Bräger, S., B. Würsig, A. Acevedo, and T. Henningsen. 1994. Association patterns of bottle-nosed dolphins (*Tursiops-truncatus*) in Galveston Bay, Texas. Journal of Mammalogy 75(2):431–437. doi: 10.2307/1382564

Bryan, C.E., S.J. Christopher, B.C. Balmer, and R.S. Wells. 2007. Establishing baseline levels of trace elements in blood and skin of bottlenose dolphins in Sarasota Bay, Florida: implications for non-invasive monitoring. Science of the Total Environment 388(1-3):325–342. doi: 10.1016/j.scitotenv.2007.07.046

Cloyed, C.S., R.M. Wilson, B.C. Balmer, A.A. Hohn, L.H. Schwacke, E.S. Zolman, M.C. Tumlin, R.S. Wells, A.A. Barleycorn, J.B. Allen, and R.H. Carmichael. 2021. Specialization of a mobile, apex predator affects trophic coupling among adjacent habitats. Scientific Reports 11(1):19611. doi: 10.1038/s41598-021-99017-1

Connor, R.C., R.S. Wells, J. Mann, and A.J. Read. 2000. The bottlenose dolphin: Social relationships in a fission-fusion society. In: Mann, J., R. Connor, P. Tyack, and H. Whitehead (Eds.), Cetacean Societies: Field Studies of Dolphins and Whales. University of Chicago Press, Chicago, IL. pp. 91–126.

Correa, L., J.M. Castellini, R.S. Wells, and T. O'Hara. 2013. Distribution of mercury and selenium in blood compartments of bottlenose dolphins (*Tursiops truncatus*) from Sarasota Bay, Florida. Environmental Toxicology and Chemistry 32(11):2441–2448. doi: 10.1002/etc.2327

Fernandez, S. and A.A. Hohn. 1998. Age, growth, and calving season of bottlenose dolphins, *Tursiops truncatus*, off coastal Texas. Fishery Bulletin 96(2):357–365.

Fertl, D. 1994. Occurrence patterns and behavior of bottlenose dolphins (*Tursiops truncatus*) in the Galveston ship channel, Texas. Texas Journal of Science 46(4):299–318.

Hayes, S.A., E. Josephson, K. Maze-Foley, P.E. Rosel, and J. Wallace. 2022. U.S. Atlantic and Gulf of Mexico marine mammal stock assessments 2021. National Oceanic and Atmospheric Administration, National Marine Fisheries Service, Northeast Fisheries Science Center: Woods Hole, MA. NOAA Technical Memorandum NMFS-NE-288. 380 p. doi: 10.25923/6tt7-kc16

Henderson, E.E. and B. Würsig. 2007. Behavior patterns of bottlenose dolphins in San Luis Pass, Texas. Gulf of Mexico Science 25(2):153–161. doi: 10.18785/goms.2502.06

Hohn, A.A., M.D. Scott, R.S. Wells, J.C. Sweeney, and A.B. Irvine. 1989. Growth layers in teeth from known-age, free-ranging bottlenose dolphins. Marine Mammal Science 5(4):315–342. doi: 10.1111/j.1748-7692.1989.tb00346.x

Hubard, C.W., K. Maze-Foley, K.D. Mullin, and W.W. Schroeder. 2004. Seasonal abundance and site fidelity of bottlenose dolphins (*Tursiops truncatus*) in Mississippi Sound. Aquatic Mammals 30(2):299–310. doi: 10.1578/am.30.2.2004.299

Irvine, A.B., M.D. Scott, R.S. Wells, and J.H. Kaufmann. 1981. Movements and activities of the Atlantic bottlenose dolphin, *Tursiops truncatus*, near Sarasota, Florida. Fishery Bulletin 79(4):671–688.

Irvine, B. and R.S. Wells. 1972. Results of attempts to tag Atlantic bottlenosed dolphins (*Tursiops truncatus*). Cetology 13:1–5.

Irwin, L.-J. and B. Würsig. 2004. A small resident community of bottlenose dolphins, *Tursiops truncatus*, in Texas: Monitoring recommendations. Gulf of Mexico Science 22(1):13–21. doi: 10.18785/goms.2201.02

Kucklick, J., L. Schwacke, R. Wells, A. Hohn, A. Guichard, J. Yordy, L. Hansen, E. Zolman, R. Wilson, J. Litz, D. Nowacek, T. Rowles, R. Pugh, B. Balmer, C. Sinclair, and P. Rosel. 2011. Bottlenose dolphins as indicators of persistent organic pollutants in the western North Atlantic Ocean and Northern Gulf of Mexico. Environmental Science & Technology 45(10):4270–4277. doi: 10.1021/es1042244

LaBrecque, E., C. Curtice, J. Harrison, S.M. Van Parijs, and P.N. Halpin. 2015. 3. Biologically Important Areas for cetaceans within U.S. Waters – Gulf of Mexico Region. Aquatic Mammals 41(1):30–38. doi: 10.1578/am.41.1.2015.30

Leatherwood, S. 1977. Some preliminary impressions on the numbers and social behavior of free-swimming bottlenosed dolphin calves (*Tursiops truncatus*) in the northern Gulf of Mexico. In: Ridgway, S.H. and K. Bernirschke (Eds.), Breeding Dolphins; Present Status, Suggestions for the Future. Final report to the US Marine Mammal Commission, Contract MM6AC009, MMC-76/07, Washington, DC. pp. 143–167.

Litz, J.A., M.A. Baran, S.R. Bowen-Stevens, R.H. Carmichael, K.M. Colegrove, L.P. Garrison, S.E. Fire, E.M. Fougeres, R. Hardy, S. Holmes, W. Jones, B.E. Mase-Guthrie, D.K. Odell, P.E. Rosel, J.T. Saliki, D.K. Shannon, S.F. Shippee, S.M. Smith, E.M. Stratton, M.C. Tumlin, H.R. Whitehead, G.A. Worthy, and T.K. Rowles. 2014. Review of historical unusual mortality events (UMEs) in the Gulf of Mexico (1990–2009): providing context for the multi-year northern Gulf of Mexico cetacean UME declared in 2010. Diseases of Aquatic Organisms 112(2):161–175. doi: 10.3354/dao02807

Lynn, S.K. and B. Würsig. 2002. Summer movement patterns of bottlenose dolphins in a Texas bay. Gulf of Mexico Science 20(1):25–37. doi: 10.18785/goms.2001.03

Mackey, A.D. 2010. Site fidelity and association patterns of bottlenose dolphins (*Tursiops truncatus*) in the Mississippi Sound. University of Southern Mississippi. Thesis. 133 p.

MacQueeney, M., M.A.M. Collier, P. Simard, and S. Gowans. 2021. Characterizing human interactions with bottlenose dolphins (*Tursiops truncatus*) in Tampa Bay. Human Dimensions of Wildlife 27(4):397–406. doi: 10.1080/10871209.2021.1950241

Mann, J., R.C. Connor, L.M. Barre, and M.R. Heithaus. 2000. Female reproductive success in bottlenose dolphins (*Tursiops* sp.): life history, habitat, provisioning, and group-size effects. Behavioral Ecology 11(2):210–219. doi: 10.1093/beheco/11.2.210

Mattson, M.C., K.D. Mullin, G.W. Ingram, and W. Hoggard. 2006. Age structure and growth of the bottlenose dolphin (*Tursiops truncatus*) from strandings in the Mississippi sound region of the north-central Gulf of Mexico from 1986 to 2003. Marine Mammal Science 22(3):654–666. doi: 10.1111/j.1748-7692.2006.00057.x

Maze, K.S. and B. Würsig. 1999. Bottlenosed dolphins of San Luis Pass, Texas: Occurrence patterns, site-fidelity, and habitat use. Aquatic Mammals 25:91–104.

McCormack, M.A., F. Battaglia, W.E. McFee, and J. Dutton. 2020a. Mercury concentrations in blubber and skin from stranded bottlenose dolphins (*Tursiops truncatus*) along the Florida and Louisiana coasts (Gulf of Mexico, USA) in relation to biological variables. Environmental Research 180:108886. doi: 10.1016/j.envres.2019.108886

McCormack, M.A., B.P. Jackson, and J. Dutton. 2020b. Relationship between mercury and selenium concentrations in tissues from stranded odontocetes in the northern Gulf of Mexico. Science of the Total Environment 749:141350. doi: 10.1016/j.scitotenv.2020.141350

McCormack, M.A., W.H. Nowlin, and J. Dutton. 2022. Effect of trophic position on mercury concentrations in bottlenose dolphins (*Tursiops truncatus*) from the northern Gulf of Mexico. Environmental Research 204(Pt B):112124. doi: 10.1016/j.envres.2021.112124

McDonald, T.L., F.E. Hornsby, T.R. Speakman, E.S. Zolman, K.D. Mullin, C. Sinclair, P.E. Rosel, L. Thomas, and L.H. Schwacke. 2017. Survival, density, and abundance of common bottlenose dolphins in Barataria Bay (USA) following the Deepwater Horizon oil spill. Endangered Species Research 33:193–209. doi: 10.3354/esr00806

McHugh, K.A., A.A. Barleycorn, J.B. Allen, K. Bassos-Hull, G. Lovewell, D. Boyd, A. Panike, C. Cush, D. Fauquier, B. Mase, R.C. Lacy, M.R. Greenfield, D.I. Rubenstein, A. Weaver, A. Stone, L. Oliver, K. Morse, and R.S. Wells. 2021. Staying alive: Long-term success of bottlenose dolphin interventions in southwest Florida. Frontiers in Marine Science 7:624729. doi: 10.3389/fmars.2020.624729

McHugh, M.B. 1989. Population numbers and feeding behavior of the Atlantic bottlenose dolphin (*Tursiops truncatus*) near Aransas Pass, Texas. University of Texas at Austin. Thesis. 96 p.

Mead, J.G. and C.W. Potter. 1990. Natural history of bottlenose dolphins along the central Atlantic coast of the United States. In: Leatherwood, S. and R.R. Reeves (Eds.), The Bottlenose Dolphin. Academic Press, San Diego, CA. pp. 165–195.

Miller, C.E. 2003. Abundance trends and environmental habitat usage patterns of bottlenose dolphins (*Tursiops truncatus*) in lower Barataria and Caminada bays, Louisiana. Louisiana State University and Agricultural & Mechanical College. Dissertation. 125 p.

Miller, D.L., V. Woshner, E.L. Styer, S. Ferguson, K.K. Knott, M.J. Gray, R.S. Wells, and T.M. O'Hara. 2011. Histologic findings in free-ranging Sarasota Bay bottlenose dolphin (*Tursiops truncatus*) skin: mercury, selenium, and seasonal factors. Journal of Wildlife Diseases 47(4):1012–1018. doi: 10.7589/0090-3558-47.4.1012

Miller, L.J., A.D. Mackey, T. Hoffland, M. Solangi, and S.A. Kuczaj. 2010. Potential effects of a major hurricane on Atlantic bottlenose dolphin (*Tursiops truncatus*) reproduction in the Mississippi Sound. Marine Mammal Science 26(3):707–715. doi: 10.1111/j.1748-7692.2010.00371.x

Miller, L.J., A.D. Mackey, M. Solangi, and S.A. Kuczaj. 2013. Population abundance and habitat utilization of bottlenose dolphins in the Mississippi Sound. Aquatic Conservation: Marine and Freshwater Ecosystems 23(1):145–151. doi: 10.1002/aqc.2278

Mullin, K.D., T. McDonald, R.S. Wells, B.C. Balmer, T. Speakman, C. Sinclair, E.S. Zolman, F. Hornsby, S.M. McBride, K.A. Wilkinson, and L.H. Schwacke. 2017. Density, abundance, survival, and ranging patterns of common bottlenose dolphins (*Tursiops truncatus*) in Mississippi Sound following the Deepwater Horizon oil spill. PLoS ONE 12(10):e0186265. doi: 10.1371/journal.pone.0186265

Perrin, W.F. and S.B. Reilly. 1984. Reproductive parameters of dolphins and small whales of the family Delphinidae. In: Perrin, W.F., R.L. Brownell, Jr., and D.P. DeMaster (Eds.), Reproduction in Whales, Dolphins, and Porpoises. pp. 97–134.

Rosel, P.E., L.A. Wilcox, C. Sinclair, T.R. Speakman, M.C. Tumlin, J.A. Litz, and E.S. Zolman. 2017. Genetic assignment to stock of stranded common bottlenose dolphins in southeastern Louisiana after the Deepwater Horizon oil spill. Endangered Species Research 33:221–234. doi: 10.3354/esr00780

Rossman, S., P.H. Ostrom, M. Stolen, N.B. Barros, H. Gandhi, C.A. Stricker, and R.S. Wells. 2015. Individual specialization in the foraging habits of female bottlenose dolphins living in a trophically diverse and habitat rich estuary. Oecologia 178(2):415–425. doi: 10.1007/s00442-015-3241-6

Scott, M.D. and S.J. Chivers. 1990. Distribution and herd structure of bottlenose dolphins in the eastern tropical Pacific Ocean. In: Leatherwood, S. and R.R. Reeves (Eds.), The Bottlenose Dolphin. Academic Press, San Diego, CA. pp. 387–402.

Sellas, A.B., R.S. Wells, and P.E. Rosel. 2005. Mitochondrial and nuclear DNA analyses reveal fine scale geographic structure in bottlenose dolphins (*Tursiops truncatus*) in the Gulf of Mexico. Conservation Genetics 6(5):715–728. doi: 10.1007/s10592-005-9031-7

Sergeant, D.E., D.K. Caldwell, and M.C. Caldwell. 1973. Age, growth, and maturity of bottlenosed dolphin (*Tursiops-truncatus*) from northeast Florida. Journal of the Fisheries Research Board of Canada 30(7):1009–1011. doi: 10.1139/f73-165

Shane, S.H. 1990. Behavior and ecology of the bottlenose dolphin at Sanibel Island, Florida. In: Leatherwood, S. and R.R. Reeves (Eds.), The Bottlenose Dolphin. Academic Press, San Diego, CA. pp. 245–265. doi: 10.1016/B978-0-12-440280-5.50016-0

Shane, S.H. 2004. Residence patterns, group characteristics, and association patterns of bottlenose dolphins near Sanibel Island, Florida. Gulf of Mexico Science 22(1):1–12. doi: 10.18785/goms.2201.01

Shane, S.H., R.S. Wells, and B. Würsig. 1986. Ecology, behavior and social organization of the bottlenose dolphin: A review. Marine Mammal Science 2:34–63. doi: 10.1111/j.1748-7692.1986.tb00026.x

Takeshita, R., B.C. Balmer, F. Messina, E.S. Zolman, L. Thomas, R.S. Wells, C.R. Smith, T.K. Rowles, and L.H. Schwacke. 2021. High site-fidelity in common bottlenose dolphins despite low salinity exposure and associated indicators of compromised health. PLoS ONE 16(9):e0258031. doi: 10.1371/journal.pone.0258031

Taylor, B.L., S.J. Chivers, J. Larese, and W.F. Perrin. 2007. Generation length and percent mature estimates for IUCN assessments of cetaceans. National Oceanic and Atmospheric Administration, National Marine Fisheries Service, Southwest Fisheries Science Center: La Jolla, CA. Administrative Report LJ-07-01. 24 p.

Toms, C. 2019. Filling the gaps: Common bottlenose dolphin (*Tursiops truncatus*) population dynamics, structure, and connectivity within Florida panhandle bays, sounds, and estuaries. University of Central Florida. Dissertation. 373 p.

Torres, L.G. and A.J. Read. 2009. Where to catch a fish? The influence of foraging tactics on the ecology of bottlenose dolphins (*Tursiops truncatus*) in Florida Bay, Florida. Marine Mammal Science 25(4):797–815. doi: 10.1111/j.1748-7692.2009.00297.x

Tyson, R.B., S.M. Nowacek, and D.P. Nowacek. 2011. Community structure and abundance of bottlenose dolphins *Tursiops truncatus* in coastal waters of the northeast Gulf of Mexico. Marine Ecology Progress Series 438:253–265. doi: 10.3354/meps09292

Urian, K.W., S. Hofmann, R.S. Wells, and A.J. Read. 2009. Fine-scale population structure of bottlenose dolphins (*Tursiops truncatus*) in Tampa Bay, Florida. Marine Mammal Science 25(3):619–638. doi: 10.1111/j.1748-7692.2009.00284.x

Vollmer, N.L. and P.E. Rosel. 2013. A review of common bottlenose dolphins (*Tursiops truncatus truncatus*) in the Northern Gulf of Mexico: Population biology, potential threats, and management. Southeastern Naturalist 12(m6):1–43. doi: 10.1656/058.012.m601

Weller, D.W. 1998. Global and regional variation in the biology and behavior of bottlenose dolphins. Texas A&M University. Dissertation. 142 p.

Wells, R.S. 1986a. Population structure of bottlenose dolphins: Behavioral studies along the central west coast of Florida. Contract Report to National Marine Fisheries Service, Southeast Fisheries Center. Contract No. 45-WCNF-5-003BB. 58 p.

Wells, R.S. 1986b. Structural aspects of dolphin societies. University of California, Santa Cruz. Dissertation. 234 p.

Wells, R.S. 1991. The role of long-term study in understanding the social structure of a bottlenose dolphin community. In: Pryor, K. and K. Norris (Eds.), Dolphin Societies: Discoveries and Puzzles. University of California Press, Berkeley, CA. pp. 199–226.

Wells, R.S. 2000. Reproduction in wild bottlenose dolphins: Overview of patterns observed during a long-term study. In: Duffield, D. and T. Robeck (Eds.), Bottlenose Dolphin Reproduction Workshop. AZA Marine Mammal Taxon Advisory Group, Silver Spring, MD. pp. 57–74.

Wells, R.S. 2003. Dolphin social complexity: Lessons from long-term study and life history. In: de Waal, F.B.M. and P.L. Tyack (Eds.), Animal Social Complexity: Intelligence, Culture, and Individualized Societies Harvard University Press, Cambridge, MA. pp. 32–56.

Wells, R.S. 2014. Social structure and life history of bottlenose dolphins near Sarasota Bay, Florida: Insights from four decades and five generations. Primates and cetaceans. pp. 149–172. doi: 10.1007/978-4-431-54523-1_8

Wells, R.S., M.K. Bassos, K.W. Urian, W.J. Carr, and M.D. Scott. 1996. Low-level monitoring of bottlenose dolphins, *Tursiops truncatus*, in Charlotte Harbor, Florida: 1990-1994. National Oceanic and Atmospheric Administration, National Marine Fisheries Service, Southeast Fisheries Science Center: Miami, FL. NOAA Technical Memorandum NMFS-SEFSC-384. 86 p.

Wells, R.S., M.K. Bassos, K.W. Urian, S.H. Shane, E.C.G. Owen, C.F. Weiss, W.J. Carr, and M.D. Scott. 1997. Low-level monitoring of bottlenose dolphins, *Tursiops truncatus*, in Pine Island Sound, Florida: 1996. National Oceanic and Atmospheric Administration, National Marine Fisheries Service, Southeast Fisheries Science Center: Miami, FL. Southeast Fisheries Center Contribution No. 40-WCNF601958. 86 p.

Wells, R.S., A. Natoli, and G. Braulik. 2019. *Tursiops truncatus* (errata version published in 2019). *The IUCN Red List of Threatened Species* 2019: e.T22563A156932432. https://dx.doi.org/10.2305/IUCN.UK.2019-1.RLTS.T22563A156932432.en

Wells, R.S., L.H. Schwacke, T.K. Rowles, B.C. Balmer, E. Zolman, T. Speakman, F.I. Townsend, M.C. Tumlin, A. Barleycorn, and K.A. Wilkinson. 2017. Ranging patterns of common bottlenose dolphins *Tursiops truncatus* in Barataria Bay, Louisiana, following the Deepwater Horizon oil spill. Endangered Species Research 33:159–180. doi: 10.3354/esr00732

Wells, R.S. and M.D. Scott. 1999. Bottlenose dolphin *Tursiops truncatus* (Montagu, 1821). In: Ridgway, S.H. and R. Harrison (Eds.), Handbook of marine mammals. Volume 6: The second book of dolphins and the porpoises. Academic Press, San Diego, CA. pp. 137–182.

Wells, R.S. and M.D. Scott. 2018. Bottlenose dolphin, *Tursiops truncatus*, common bottlenose dolphin. In: Würsig, B., J.G.M. Thewissen, and K.M. Kovacs (Eds.), Encyclopedia of Marine Mammals (Third Edition). Academic Press, London, UK. pp. 118–125. doi: 10.1016/B978-0-12-804327-1.00072-8

Wells, R.S., M.D. Scott, and A.B. Irvine. 1987. The social structure of free-ranging bottlenose dolphins. In: Genoways, H.H. (Ed.), Current Mammalogy. Plenum Press, New York, NY. pp. 247–305.

Wells, R.S., K.W. Urian, A.J. Read, M.K. Bassos, W.J. Carr, and M.D. Scott. 1996. Low-level monitoring of bottlenose dolphins, *Tursiops truncatus*, in Tampa Bay, Florida: 1988-1993. National Oceanic and Atmospheric Administration, National Marine Fisheries Service, Southeast Fisheries Science Center: Miami, FL. NOAA Technical Memorandum NMFS-SEFSC-385. 36 p.

Wilson, R.M., R.B. Tyson, J.A. Nelson, B.C. Balmer, J.P. Chanton, and D.P. Nowacek. 2017. Niche differentiation and prey selectivity among common bottlenose dolphins (*Tursiops truncatus*) sighted in St. George Sound, Gulf of Mexico. Frontiers in Marine Science 4:235. doi: 10.3389/fmars.2017.00235

Woshner, V., K. Knott, R. Wells, C. Willetto, R. Swor, and T. O'Hara. 2008. Mercury and selenium in blood and epidermis of bottlenose dolphins (*Tursiops truncatus*) from Sarasota Bay, FL: interaction and relevance to life history and hematologic parameters. Ecohealth 5(3):360–370. doi: 10.1007/s10393-008-0164-2

Yordy, J.E., M.A. Mollenhauer, R.M. Wilson, R.S. Wells, A. Hohn, J. Sweeney, L.H. Schwacke, T.K. Rowles, J.R. Kucklick, and M.M. Peden-Adams. 2010a. Complex contaminant exposure in cetaceans: a comparative E-Screen analysis of bottlenose dolphin blubber and mixtures of four persistent organic pollutants. Environmental Toxicology and Chemistry 29(10):2143–2153. doi: 10.1002/etc.279

Yordy, J.E., D.A. Pabst, W.A. McLellan, R.S. Wells, T.K. Rowles, and J.R. Kucklick. 2010b. Tissue-specific distribution and whole-body burden estimates of persistent organic pollutants in the bottlenose dolphin (*Tursiops truncatus*). Environmental Toxicology and Chemistry 29(6):1263–1273. doi: 10.1002/etc.152

Yordy, J.E., R.S. Wells, B.C. Balmer, L.H. Schwacke, T.K. Rowles, and J.R. Kucklick. 2010c. Life history as a source of variation for persistent organic pollutant (POP) patterns in a community of common bottlenose dolphins (*Tursiops truncatus*) resident to Sarasota Bay, FL. Science of the Total Environment 408(9):2163–2172. doi: 10.1016/j.scitotenv.2010.01.032

Common bottlenose dolphin − *Tursiops truncatus*

GoMx BSE/Terrebonne Bay, Timbalier Bay Stock

Overall Vulnerability Rank = Very High

Biological Sensitivity = Very High

Climate Exposure = Very High

Data Quality = 89% of scores ≥ 2

	Tursiops truncatus	Expert Scores	Data Quality	Expert Scores Plots (Portion by Category)
Sensitivity attributes	Prey/Diet Specificity	2.4	2.3	
	Habitat Specificity	3.5	1.7	
	Site Fidelity	3.7	2.0	
	Lifetime Reproductive Potential	1.5	1.7	
	Generation Length	2.9	2.0	
	Reproductive Plasticity	1.1	2.0	
	Migration	3.9	2.0	
	Home Range	3.7	2.0	
	Stock Abundance	2.0	2.7	
	Stock Abundance Trend	2.9	1.0	
	Cumulative Stressors	3.3	2.3	
	Sensitivity Score	Very High		
Exposure variables	Sea Surface Temperature (Change in mean)	4.0	2.7	
	Sea Surface Temperature (Change in variability)	1.0	2.7	
	Air Temperature (Change in mean)	4.0	2.7	
	Air Temperature (Change in variability)	1.0	2.7	
	Precipitation (Change in mean)	1.0	2.7	
	Precipitation (Change in variability)	1.0	2.7	
	Sea Surface Salinity (Change in mean)	1.7	2.7	
	Sea Surface Salinity (Change in variability)	1.0	2.7	
	Ocean pH (Change in mean)	4.0	2.7	
	Ocean pH (Change in variability)	1.0	2.7	
	Sea ice coverage (Change in mean)	1.0	3.0	
	Sea ice coverage (Change in variability)	1.0	3.0	
	Dissolved oxygen (Change in mean)	4.0	2.7	
	Dissolved oxygen (Change in variability)	1.0	2.7	
	Circulation	3.8	2.0	
	Sea level rise	4.0	2.7	
	Exposure Score	Very High		
Overall Vulnerability Rank		Very High		

Low
Moderate
High
Very High

Common bottlenose dolphin (GoMx BSE/Terrebonne Bay, Timbalier Bay Stock)

Tursiops truncatus

CVA Results Summary

Overall Climate Vulnerability Rank: Very High (100% certainty from bootstrap analysis).

Climate Exposure: Very High. Six exposure factors scored greater than 3.5: Air Temperature (Standard anomaly) (4.00), Dissolved oxygen (Standard anomaly) (4.00), Ocean pH (Standard anomaly) (4.00), Sea level rise (4.00), Sea Surface Temperature (Standard anomaly) (4.00), and Circulation (3.80).

Biological Sensitivity: Very High. Three sensitivity attributes scored greater than 3.5: Migration (3.93), Home Range (3.67), and Site Fidelity (3.67).

Distributional Response: High

Abundance Response: High

Phenology Response: High

Data Quality: 89% of the data quality scores were 2 or greater, 73% of sensitivity attribute data quality scores were 2 or higher.

Background Information

Stock Description

This stock includes common bottlenose dolphins in estuarine waters from Bay Junop to Bayou LaFourche, including Bay Junop, Caillou Lake, Lake Pelto, Terrebonne Bay, Lake Barre, Lake Felicity, Timbalier Bay, and Lake Raccourci, and adjacent waters out to 1 km from the barrier islands in the northern Gulf of Mexico along the Louisiana coast (Hayes et al. 2019).

Prey/Diet Specificity

Generally, common bottlenose dolphins consume a large variety of fish and/or squid (Barros and Odell 1990; Mead and Potter 1990; Wells and Scott 2018). Common bottlenose dolphins seem to show a consistent preference for sound-producing fishes (e.g., sciaenids, toadfish; Berens McCabe et al. 2010; Bowen-Stevens et al. 2021). In Sarasota Bay, Florida, Rossman et al. (2015) found some individual common bottlenose dolphins to show diet specialization despite the broader study group showing a generalist diet. Specialization can be influenced by locally abundant prey species (Shane et al. 1986; Vollmer and Rosel 2013) or can result from resource partitioning amongst sympatric populations (e.g., Torres and Read 2009; Wilson et al. 2017).

Habitat Specificity

Common bottlenose dolphins are found in a variety of marine and estuarine habitats (Wells and Scott 2018).

Site Fidelity

No specific site fidelity information was found for this stock. However, common bottlenose dolphins in other Gulf of Mexico bay, sound, and estuary (BSE) stocks show site fidelity (LaBrecque et al. 2015; Hayes et al. 2022), such as in Barataria Bay in Louisiana (Miller 2003; McDonald et al. 2017; Wells et al. 2017; Takeshita et al. 2021); Matagorda Bay, Espiritu Santo Bay, and San Luis Pass in Texas (Maze and Würsig 1999; Lynn and Würsig 2002; Irwin and Würsig 2004); Mississippi Sound in Mississippi (Hubard et al. 2004; Mackey 2010; Mullin et al. 2017); Charlotte Harbor and Pine Island Sound (Bassos-Hull et al. 2013), Sarasota Bay (Wells 1986a; Wells 1991; Wells 2014), St. Andrew Bay (Balmer et al. 2019), St. Joseph Bay (Balmer et al. 2008; B. Balmer et al. 2018), St. Vincent Sound and Apalachicola Bay (Tyson et al. 2011), and Tampa Bay in Florida (Wells et al. 1996b; Urian et al. 2009).

Lifetime Reproductive Potential

Taylor et al. (2007) reported the interbirth interval of common bottlenose dolphins as 3.80 years, though other studies have shown it to be closer to three years (Leatherwood 1977; Wells and Scott 1999; Connor et al. 2000; Wells 2003).

Generally, females reach sexual maturity before males, with females reaching sexual maturity at 5–13 years (Sergeant et al. 1973; Perrin and Reilly 1984; Wells et al. 1987; Mead and Potter 1990; Mann et al. 2000; Wells 2000; Wells and Scott 2018).

Females reproduce through most of their adult lives (Leatherwood 1977; Hohn et al. 1989; Wells and Scott 1999; Wells 2000; Wells 2003), but may produce fewer calves after they reach 30 years of age (Wells 2000). Sarasota Bay common bottlenose dolphins can give birth when they are as young as 6 years of age, and can continue until they are 48 years old (Wells and Scott 2018). Reproductive lifespan would therefore be up to 42 years based on long-term observation studies (Wells 2014; Wells and Scott 2018).

Longitudinal studies from Sarasota Bay, Florida have found that female common bottlenose dolphins can live up to 67 years (Wells and Scott 2018). Studies based on growth layer groups in teeth suggest female common bottlenose dolphins can live to 41 years along the Texas coast (Fernandez and Hohn 1998) and to 30 years along the Mississippi coast (Mattson et al. 2006).

Generation Length

Taylor et al. (2007) reported common bottlenose dolphin generation length of 20.6–21.1 years.

Reproductive Plasticity

Common bottlenose dolphin calving is generally diffusely seasonal, with one or more peaks, although births have been reported from all seasons (Vollmer and Rosel 2013; Wells and Scott 2018). Calf

sightings peak in the spring and summer months in Florida (Wells et al. 1987), Mississippi/Louisiana (Miller et al. 2010; Miller et al. 2013), and Texas (McHugh 1989; Henderson et al. 2007).

Migration

The BSE stocks in the Gulf of Mexico are thought to primarily comprise stable resident communities. However, transient or seasonal movements of some individuals into and out of a BSE stock area are recorded in some areas (LaBrecque et al. 2015; Hayes et al. 2022). Molecular genetic data support that some BSE stocks are genetically distinct from one another, and furthermore are differentiated from adjacent coastal populations (e.g., Sellas et al. 2005; Rosel et al. 2017). For example, Toms (2019) found fine-scale genetic population structure among five inshore systems in the Florida Panhandle and variable migration rates among populations.

Year-round residency has been reported for common bottlenose dolphins (Hayes et al. 2022), such as in Florida (Irvine and Wells 1972; Irvine et al. 1981; Wells 1986a; Wells 1986b; Wells et al. 1987; Scott et al. 1990; Wells 1991; Wells et al. 1996a; Wells et al. 1996b; Wells et al. 1997; Wells 2003; Balmer et al. 2008; Urian et al. 2009; Tyson et al. 2011; Bassos-Hull et al. 2013; B. Balmer et al. 2018; Balmer et al. 2019; Toms 2019), Texas (Shane 1990; Bräger 1993; Bräger et al. 1994; Fertl 1994; Weller 1998; Maze and Würsig 1999; Lynn and Würsig 2002; Irwin and Würsig 2004; Shane 2004), and Louisiana/Mississippi (Hubard et al. 2004; McDonald et al. 2017; Mullin et al. 2017; Takeshita et al. 2021).

Home Range

The home ranges of individuals within the Gulf of Mexico BSE stocks are considered to generally include the areas within the stock boundaries and brief excursions beyond the stock boundaries (Irvine et al. 1981; Wells 1986a; Wells 1986b; Wells 1991; Wells et al. 1996a; Wells et al. 1996b; Balmer et al. 2008; Wells 2014; Balmer et al. 2019), although some individuals show preference for small areas within the stock boundaries (Lynn and Würsig 2002; Urian et al. 2009; Mullin et al. 2017; Wells et al. 2017).

Stock Abundance

Litz et al. (2018) estimated this stock’s abundance at 3,870 (CV=0.15; 95% CI: 2934–5210), based on June 2016 vessel-based capture-recapture photo-ID surveys.

Stock Abundance Trend

Data are insufficient to complete an abundance trend analysis for this stock (Hayes et al. 2019).

Cumulative Stressors

Common bottlenose dolphins face a wide range of individual and cumulative natural and anthropogenic threats (see reviews by Vollmer and Rosel 2013; Wells and Scott 2018; and Wells et al. 2019). These include such threats as shark predation, harmful algal blooms, recreational and commercial fishing gear, vessel traffic, pollution, habitat alteration, and provisioning.

Common bottlenose dolphins in this stock were exposed to oil and dispersants following the Deepwater Horizon oil spill, which may result in long term reproductive and chronic health effects (NOAA 2011; Michel et al. 2013; Helm et al. 2015; DWH NRDA 2016; Lane et al. 2015; De Guise et al. 2017, 2021;

Kellar et al. 2017; Smith et al. 2017; Schwacke et al. 2017). An Unusual Mortality Event (UME), involving primarily bottlenose dolphins, was declared for cetaceans in the northern Gulf of Mexico from 2010 to 2014, with the Deepwater Horizon oil spill as the primary underlying cause (Litz et al. 2014; Schwacke et al. 2014; Venn-Watson et al. 2015a, 2015b).

The nearshore and estuarine habitats along the Gulf of Mexico coast include waters adjacent to areas of high human population, some of which are highly industrialized, and areas of heavy agricultural impact. Studies of bottlenose dolphin tissues from nearby Barataria Bay have shown persistent organic pollutants (POPs) in blubber and blood samples, though at concentrations lower than other southeastern U.S. sites (Kucklick et al. 2011; Balmer et al. 2015; J. Balmer et al. 2018). Similarly, mercury has been detected in common bottlenose dolphin tissues in Louisiana waters, but in lower concentrations than common bottlenose dolphins found in Florida waters (McCormack et al. 2020a, 2020b, 2022).

Dolphin morbillivirus has been detected in populations of the northern Gulf of Mexico, but has not been attributed to large scale die-offs since 1993−1994 (Lipscomb et al. 1996; Litz et al. 2014; Van Bressem et al. 2014; Fauquier et al. 2017). A UME was declared in 1990 for the Gulf of Mexico from Florida to Texas, with more than 300 common bottlenose dolphins stranding due to an undetermined cause, although morbillivirus and cold temperatures may have contributed (Litz et al. 2014).

Distribution and Sightings

Sighting maps for this stock are presented by Mullin et al. (2018).

Further Reading

Hayes, S.A., E. Josephson, K. Maze-Foley, and P.E. Rosel. 2019. US Atlantic and Gulf of Mexico Marine Mammal Stock Assessments - 2018. National Oceanic and Atmospheric Administration, National Marine Fisheries Service, Northeast Fisheries Science Center: Woods Hole, MA. NOAA technical memorandum NMFS-NE-258. 298 p. doi: 10.25923/9rrd-tx13

Wells, R.S. and M.D. Scott. 2018. Bottlenose Dolphin, *Tursiops truncatus*, Common Bottlenose Dolphin. In: Würsig, B., J.G.M. Thewissen, and K.M. Kovacs (Eds.), Encyclopedia of Marine Mammals (Third Edition). Academic Press, London, UK. pp. 118–125. doi: 10.1016/B978-0-12-804327-1.00072-8

Wells, R.S., A. Natoli, and G. Braulik. 2019. *Tursiops truncatus* (errata version published in 2019). The IUCN Red List of Threatened Species 2019: e.T22563A156932432. https://dx.doi.org/10.2305/IUCN.UK.2019-1.RLTS.T22563A156932432.en

References

Balmer, B., T. McDonald, F. Hornsby, J. Adams, J. Allen, A. Barleycorn, and L. Schwacke. 2018. Long-term trends in a northern Gulf of Mexico common bottlenose dolphin (*Tursiops truncatus*) population in the wake of the Deepwater Horizon oil spill. Journal of Cetacean Research and Management 18:1–9. doi: 10.47536/jcrm.v18i1.409

Balmer, B., S. Watwood, B. Quigley, T. Speakman, K. Barry, K. Mullin, P. Rosel, C. Sinclair, E. Zolman, and L. Schwacke. 2019. Common bottlenose dolphin (*Tursiops truncatus*) abundance and distribution patterns in St. Andrew Bay, Florida, USA. Aquatic Conservation: Marine and Freshwater Ecosystems 29(3):486–498. doi: 10.1002/aqc.3001

Balmer, B.C., R.S. Wells, S.M. Nowacek, D.P. Nowacek, L.H. Schwacke, W.A. McLellan, F.S. Scharf, T.K. Rowles, L.J. Hansen, T.R. Spradlin, and D.A. Pabst. 2008. Seasonal abundance and distribution patterns of common bottlenose dolphins (*Tursiops truncatus*) near St. Joseph Bay, Florida, USA. Journal of Cetacean Research and Management 10(2):157–167.

Balmer, B.C., G.M. Ylitalo, L.E. McGeorge, K.A. Baugh, D. Boyd, K.D. Mullin, P.E. Rosel, C. Sinclair, R.S. Wells, E.S. Zolman, and L.H. Schwacke. 2015. Persistent organic pollutants (POPs) in blubber of common bottlenose dolphins (*Tursiops truncatus*) along the northern Gulf of Mexico coast, USA. Science of the Total Environment 527-528:306–312. doi: 10.1016/j.scitotenv.2015.05.016

Balmer, J.E., G.M. Ylitalo, T.K. Rowles, K.D. Mullin, R.S. Wells, F.I. Townsend, R.W. Pearce, J.L. Bolton, E.S. Zolman, B.C. Balmer, and L.H. Schwacke. 2018. Persistent organic pollutants (POPs) in blood and blubber of common bottlenose dolphins (*Tursiops truncatus*) at three northern Gulf of Mexico sites following the Deepwater Horizon oil spill. Science of the Total Environment 621:130–137. doi: 10.1016/j.scitotenv.2017.11.209

Barros, N.B. and D.K. Odell. 1990. Food habits of bottlenose dolphins in the southeastern United States In: Leatherwood, S. and R.R. Reeves (Eds.), The Bottlenose Dolphin. Academic Press, San Diego, CA. pp. 309–328. doi: 10.1016/B978-0-12-440280-5.50020-2

Bassos-Hull, K., R.M. Perrtree, C.C. Shepard, S. Schilling, A.A. Barleycorn, J.B. Allen, B.C. Balmer, W.E. Pine, and R.S. Wells. 2013. Long-term site fidelity and seasonal abundance estimates of common bottlenose dolphins (*Tursiops truncatus*) along the southwest coast of Florida and responses to natural perturbations. Journal of Cetacean Research and Management 13(1):19–30. doi: 10.47536/jcrm.v13i1.551

Berens McCabe, E.J., D.P. Gannon, N.B. Barros, and R.S. Wells. 2010. Prey selection by resident common bottlenose dolphins (*Tursiops truncatus*) in Sarasota Bay, Florida. Marine Biology 157(5):931–942. doi: 10.1007/s00227-009-1371-2

Bowen-Stevens, S.R., D.P. Gannon, R.A. Hazelkorn, G. Lovewell, K.M. Volker, S. Smith, M.C. Tumlin, and J. Litz. 2021. Diet of common bottlenose dolphins, *Tursiops truncatus*, that stranded in and near Barataria Bay, Louisiana, 2010–2012. Southeastern Naturalist 20(1). doi: 10.1656/058.020.0113

Bräger, S. 1993. Diurnal and seasonal behavior patterns of bottlenose dolphins (*Tursiops truncatus*). Marine Mammal Science 9:434–438. doi: 10.1111/j.1748-7692.1993.tb00477.x

Bräger, S., B. Würsig, A. Acevedo, and T. Henningsen. 1994. Association patterns of bottle-nosed dolphins (*Tursiops-truncatus*) in Galveston Bay, Texas. Journal of Mammalogy 75(2):431–437. doi: 10.2307/1382564

Connor, R.C., R.S. Wells, J. Mann, and A.J. Read. 2000. The bottlenose dolphin: Social relationships in a fission-fusion society. In: Mann, J., R. Connor, P. Tyack, and H. Whitehead (Eds.), Cetacean Societies: Field Studies of Dolphins and Whales. University of Chicago Press, Chicago, IL. pp. 91–126.

De Guise, S., M. Levin, E. Gebhard, L. Jasperse, L. Burdett Hart, C.R. Smith, S. Venn-Watson, F. Townsend, R. Wells, B. Balmer, E. Zolman, T. Rowles, and L. Schwacke. 2017. Changes in immune functions in bottlenose dolphins in the northern Gulf of Mexico associated with the Deepwater Horizon oil spill. Endangered Species Research 33:291–303. doi: 10.3354/esr00814

De Guise, S., M. Levin, L. Jasperse, J. Herrman, R.S. Wells, T. Rowles, and L. Schwacke. 2021. Long-term immunological alterations in bottlenose dolphin a decade after the Deepwater Horizon oil spill in the northern Gulf of Mexico: Potential for multigenerational effects. Environmental Toxicology and Chemistry 40(5):1308–1321. doi: 10.1002/etc.4980

Deepwater Horizon Natural Resource Damage Assessment Trustees (DWH NRDA). 2016. Deepwater Horizon oil spill: Final Programmatic Damage Assessment and Restoration Plan and Final Programmatic Environmental Impact Statement. Retrieved from: http://www.gulfspillrestoration.noaa.gov/restoration-planning/gulf-plan.1659.

Fauquier, D.A., J. Litz, S. Sanchez, K. Colegrove, L.H. Schwacke, L. Hart, J. Saliki, C. Smith, T. Goldstein, S. Bowen-Stevens, W. McFee, E. Fougeres, B. Mase-Guthrie, E. Stratton, R. Ewing, S. Venn-Watson, R.H. Carmichael, C. Clemons-Chevis, W. Hatchett, D. Shannon, S. Shippee, S. Smith, L. Staggs, M.C. Tumlin, N.L. Wingers, and T.K. Rowles. 2017. Evaluation of morbillivirus exposure in cetaceans from the northern Gulf of Mexico 2010-2014. Endangered Species Research 33:211–220. doi: 10.3354/esr00772

Fernandez, S. and A.A. Hohn. 1998. Age, growth, and calving season of bottlenose dolphins, *Tursiops truncatus*, off coastal Texas. Fishery Bulletin 96(2):357–365.

Fertl, D. 1994. Occurrence patterns and behavior of bottlenose dolphins (*Tursiops truncatus*) in the Galveston ship channel, Texas. Texas Journal of Science 46(4):299–318.

Hayes, S.A., E. Josephson, K. Maze-Foley, and P.E. Rosel. 2019. US Atlantic and Gulf of Mexico marine mammal stock assessments - 2018. National Oceanic and Atmospheric Administration, National Marine Fisheries Service, Northeast Fisheries Science Center: Woods Hole, MA. NOAA Technical Memorandum NMFS-NE-258. 298 p. doi: 10.25923/9rrd-tx13

Hayes, S.A., E. Josephson, K. Maze-Foley, P.E. Rosel, and J. Wallace. 2022. U.S. Atlantic and Gulf of Mexico marine mammal stock assessments 2021. National Oceanic and Atmospheric Administration, National Marine Fisheries Service, Northeast Fisheries Science Center: Woods Hole, MA. NOAA Technical Memorandum NMFS-NE-288. 380 p. doi: 10.25923/6tt7-kc16

Helm, R.C., D.P. Costa, T.D. DeBruyn, T.J. O'Shea, R.S. Wells, and T.M. Williams. 2015. Overview of effects of oil spills on marine mammals. In: Fingas, M. (Ed.), Handbook of oil spill science and technology, First edition. John Wiley & Sons, Inc., Hoboken, NJ. pp. 455–475.

Henderson, E.E. and B. Würsig. 2007. Behavior patterns of bottlenose dolphins in San Luis Pass, Texas. Gulf of Mexico Science 25(2):153–161. doi: 10.18785/goms.2502.06

Hohn, A.A., M.D. Scott, R.S. Wells, J.C. Sweeney, and A.B. Irvine. 1989. Growth layers in teeth from known-age, free-ranging bottlenose dolphins. Marine Mammal Science 5(4):315–342. doi: 10.1111/j.1748-7692.1989.tb00346.x

Hubard, C.W., K. Maze-Foley, K.D. Mullin, and W.W. Schroeder. 2004. Seasonal abundance and site fidelity of bottlenose dolphins (*Tursiops truncatus*) in Mississippi Sound. Aquatic Mammals 30(2):299–310. doi: 10.1578/am.30.2.2004.299

Irvine, A.B., M.D. Scott, R.S. Wells, and J.H. Kaufmann. 1981. Movements and activities of the Atlantic bottlenose dolphin, *Tursiops truncatus*, near Sarasota, Florida. Fishery Bulletin 79(4):671–688.

Irvine, B. and R.S. Wells. 1972. Results of attempts to tag Atlantic bottlenosed dolphins (*Tursiops truncatus*). Cetology 13:1–5.

Irwin, L.-J. and B. Würsig. 2004. A small resident community of bottlenose dolphins, *Tursiops truncatus*, in Texas: Monitoring recommendations. Gulf of Mexico Science 22(1):13–21. doi: 10.18785/goms.2201.02

Kellar, N.M., T.R. Speakman, C.R. Smith, S.M. Lane, B.C. Balmer, M.L. Trego, K.N. Catelani, M.N. Robbins, C.D. Allen, R.S. Wells, E.S. Zolman, T.K. Rowles, and L.H. Schwacke. 2017. Low reproductive success rates of common bottlenose dolphins *Tursiops truncatus* in the northern Gulf of Mexico following the Deepwater Horizon disaster (2010-2015). Endangered Species Research 33:143–158. doi: 10.3354/esr00775

Kucklick, J., L. Schwacke, R. Wells, A. Hohn, A. Guichard, J. Yordy, L. Hansen, E. Zolman, R. Wilson, J. Litz, D. Nowacek, T. Rowles, R. Pugh, B. Balmer, C. Sinclair, and P. Rosel. 2011. Bottlenose dolphins as indicators of persistent organic pollutants in the western North Atlantic Ocean and Northern Gulf of Mexico. Environmental Science & Technology 45(10):4270–4277. doi: 10.1021/es1042244

LaBrecque, E., C. Curtice, J. Harrison, S.M. Van Parijs, and P.N. Halpin. 2015. 3. Biologically Important Areas for cetaceans within U.S. Waters – Gulf of Mexico Region. Aquatic Mammals 41(1):30–38. doi: 10.1578/am.41.1.2015.30

Lane, S.M., C.R. Smith, J. Mitchell, B.C. Balmer, K.P. Barry, T. McDonald, C.S. Mori, P.E. Rosel, T.K. Rowles, T.R. Speakman, F.I. Townsend, M.C. Tumlin, R.S. Wells, E.S. Zolman, and L.H. Schwacke. 2015. Reproductive outcome and survival of common bottlenose dolphins sampled in Barataria Bay, Louisiana, USA, following the Deepwater Horizon oil spill. Proceedings of the Royal Society B: Biological Sciences 282(1818):20151944. doi: 10.1098/rspb.2015.1944

Leatherwood, S. 1977. Some preliminary impressions on the numbers and social behavior of free-swimming bottlenosed dolphin calves (*Tursiops truncatus*) in the northern Gulf of Mexico. In: Ridgway, S.H. and K. Bernirschke (Eds.), Breeding Dolphins; Present Status, Suggestions for the Future. Final report to the US Marine Mammal Commission, Contract MM6AC009, MMC-76/07, Washington, DC. pp. 143–167.

Lipscomb, T.P., S. Kennedy, D. Moffett, A. Krafft, B.A. Klaunberg, J.H. Lichy, G.T. Regan, G.A.J. Worthy, and J.K. Taubenberger. 1996. Morbilliviral epizootic in bottlenose dolphins of the Gulf of Mexico. Journal of Veterinary Diagnostic Investigation 8(3):283–290. doi: 10.1177/104063879600800302

Litz, J., C. Sinclair, and L.P. Garrison. 2018. Terrebonne-Timbalier Bay, Louisiana, common bottlenose dolphin abundance estimate 2016. National Oceanic and Atmospheric Administration, National Marine Fisheries Service, Southeast Fisheries Science Center: Miami, FL. SEFSC Report PRBD-2018-03. 9 p.

Litz, J.A., M.A. Baran, S.R. Bowen-Stevens, R.H. Carmichael, K.M. Colegrove, L.P. Garrison, S.E. Fire, E.M. Fougeres, R. Hardy, S. Holmes, W. Jones, B.E. Mase-Guthrie, D.K. Odell, P.E. Rosel, J.T. Saliki, D.K. Shannon, S.F. Shippee, S.M. Smith, E.M. Stratton, M.C. Tumlin, H.R. Whitehead, G.A. Worthy, and T.K. Rowles. 2014. Review of historical unusual mortality events (UMEs) in the Gulf of Mexico (1990–2009): providing context for the multi-year northern Gulf of Mexico cetacean UME declared in 2010. Diseases of Aquatic Organisms 112(2):161–175. doi: 10.3354/dao02807

Lynn, S.K. and B. Würsig. 2002. Summer movement patterns of bottlenose dolphins in a Texas bay. Gulf of Mexico Science 20(1):25–37. doi: 10.18785/goms.2001.03

Mackey, A.D. 2010. Site fidelity and association patterns of bottlenose dolphins (*Tursiops truncatus*) in the Mississippi Sound. University of Southern Mississippi. Thesis. 133 p.

Mann, J., R.C. Connor, L.M. Barre, and M.R. Heithaus. 2000. Female reproductive success in bottlenose dolphins (*Tursiops* sp.): life history, habitat, provisioning, and group-size effects. Behavioral Ecology 11(2):210–219. doi: 10.1093/beheco/11.2.210

Mattson, M.C., K.D. Mullin, G.W. Ingram, and W. Hoggard. 2006. Age structure and growth of the bottlenose dolphin (*Tursiops truncatus*) from strandings in the Mississippi sound region of the north-central Gulf of Mexico from 1986 to 2003. Marine Mammal Science 22(3):654–666. doi: 10.1111/j.1748-7692.2006.00057.x

Maze, K.S. and B. Würsig. 1999. Bottlenosed dolphins of San Luis Pass, Texas: Occurrence patterns, site-fidelity, and habitat use. Aquatic Mammals 25:91–104.

McCormack, M.A., F. Battaglia, W.E. McFee, and J. Dutton. 2020a. Mercury concentrations in blubber and skin from stranded bottlenose dolphins (*Tursiops truncatus*) along the Florida and Louisiana coasts (Gulf of Mexico, USA) in relation to biological variables. Environmental Research 180:108886. doi: 10.1016/j.envres.2019.108886

McCormack, M.A., B.P. Jackson, and J. Dutton. 2020b. Relationship between mercury and selenium concentrations in tissues from stranded odontocetes in the northern Gulf of Mexico. Science of the Total Environment 749:141350. doi: 10.1016/j.scitotenv.2020.141350

McCormack, M.A., W.H. Nowlin, and J. Dutton. 2022. Effect of trophic position on mercury concentrations in bottlenose dolphins (*Tursiops truncatus*) from the northern Gulf of Mexico. Environmental Research 204(Pt B):112124. doi: 10.1016/j.envres.2021.112124

McDonald, T.L., F.E. Hornsby, T.R. Speakman, E.S. Zolman, K.D. Mullin, C. Sinclair, P.E. Rosel, L. Thomas, and L.H. Schwacke. 2017. Survival, density, and abundance of common bottlenose dolphins in Barataria Bay (USA) following the Deepwater Horizon oil spill. Endangered Species Research 33:193–209. doi: 10.3354/esr00806

McHugh, M.B. 1989. Population numbers and feeding behavior of the Atlantic bottlenose dolphin (*Tursiops truncatus*) near Aransas Pass, Texas. University of Texas at Austin. Thesis. 96 p.

Mead, J.G. and C.W. Potter. 1990. Natural history of bottlenose dolphins along the central Atlantic coast of the United States. In: Leatherwood, S. and R.R. Reeves (Eds.), The Bottlenose Dolphin. Academic Press, San Diego, CA. pp. 165–195.

Michel, J., E.H. Owens, S. Zengel, A. Graham, Z. Nixon, T. Allard, W. Holton, P.D. Reimer, A. Lamarche, M. White, N. Rutherford, C. Childs, G. Mauseth, G. Challenger, and E. Taylor. 2013. Extent and degree of shoreline oiling: Deepwater Horizon oil spill, Gulf of Mexico, USA. PLoS ONE 8(6):e65087. doi: 10.1371/journal.pone.0065087

Miller, C.E. 2003. Abundance trends and environmental habitat usage patterns of bottlenose dolphins (*Tursiops truncatus*) in lower Barataria and Caminada bays, Louisiana. Louisiana State University and Agricultural & Mechanical College. Dissertation. 125 p.

Miller, L.J., A.D. Mackey, T. Hoffland, M. Solangi, and S.A. Kuczaj. 2010. Potential effects of a major hurricane on Atlantic bottlenose dolphin (*Tursiops truncatus*) reproduction in the Mississippi Sound. Marine Mammal Science 26(3):707–715. doi: 10.1111/j.1748-7692.2010.00371.x

Miller, L.J., A.D. Mackey, M. Solangi, and S.A. Kuczaj. 2013. Population abundance and habitat utilization of bottlenose dolphins in the Mississippi Sound. Aquatic Conservation: Marine and Freshwater Ecosystems 23(1):145–151. doi: 10.1002/aqc.2278

Mullin, K.D., K. Barry, T. McDonald, J. Morey, B. Quigley, E. Ronje, L. Schwacke, S. Carrie, T. Speakman, and J. Thompson. 2018. Assessment of the overlap of Terrebonne–Timbalier Bay and Bartaria Bay common bottlenose dolphin (*Tursiops truncatus*) stocks based on photo-identification of individual dolphins. National Oceanic and Atmospheric Administration, National Marine Fisheries Service, Southeast Fisheries Science Center: Pascagoula, MS. NOAA Technical Memorandum NMFS-SEFSC-729. 29 p. doi: 10.25923/8g4y-dg29

Mullin, K.D., T. McDonald, R.S. Wells, B.C. Balmer, T. Speakman, C. Sinclair, E.S. Zolman, F. Hornsby, S.M. McBride, K.A. Wilkinson, and L.H. Schwacke. 2017. Density, abundance, survival, and ranging patterns of common bottlenose dolphins (*Tursiops truncatus*) in Mississippi Sound following the Deepwater Horizon oil spill. PLoS ONE 12(10):e0186265. doi: 10.1371/journal.pone.0186265

National Oceanic and Atmospheric Administration (NOAA). 2011. Public scoping for preparation of a programmatic environmental impact statement for the Deepwater Horizon BP Oil Spill. 9 p.

Perrin, W.F. and S.B. Reilly. 1984. Reproductive parameters of dolphins and small whales of the family Delphinidae. In: Perrin, W.F., R.L. Brownell, Jr., and D.P. DeMaster (Eds.), Reproduction in Whales, Dolphins, and Porpoises. pp. 97–134.

Rosel, P.E., L.A. Wilcox, C. Sinclair, T.R. Speakman, M.C. Tumlin, J.A. Litz, and E.S. Zolman. 2017. Genetic assignment to stock of stranded common bottlenose dolphins in southeastern Louisiana after the Deepwater Horizon oil spill. Endangered Species Research 33:221–234. doi: 10.3354/esr00780

Rossman, S., P.H. Ostrom, M. Stolen, N.B. Barros, H. Gandhi, C.A. Stricker, and R.S. Wells. 2015. Individual specialization in the foraging habits of female bottlenose dolphins living in a trophically diverse and habitat rich estuary. Oecologia 178(2):415–425. doi: 10.1007/s00442-015-3241-6

Schwacke, L.H., C.R. Smith, F.I. Townsend, R.S. Wells, L.B. Hart, B.C. Balmer, T.K. Collier, S. De Guise, M.M. Fry, L.J. Guillette, Jr., S.V. Lamb, S.M. Lane, W.E. McFee, N.J. Place, M.C. Tumlin, G.M. Ylitalo, E.S. Zolman, and T.K. Rowles. 2014. Health of common bottlenose dolphins (*Tursiops truncatus*) in Barataria Bay, Louisiana, following the deepwater horizon oil spill. Environmental Science & Technology 48(1):93–103. doi: 10.1021/es403610f

Schwacke, L.H., L. Thomas, R.S. Wells, W.E. McFee, A.A. Hohn, K.D. Mullin, E.S. Zolman, B.M. Quigley, T.K. Rowles, and J.H. Schwacke. 2017. Quantifying injury to common bottlenose dolphins from the Deepwater Horizon oil spill using an age-, sex- and class-structured population model. Endangered Species Research 33:265–279. doi: 10.3354/esr00777

Scott, M.D. and S.J. Chivers. 1990. Distribution and herd structure of bottlenose dolphins in the eastern tropical Pacific Ocean. In: Leatherwood, S. and R.R. Reeves (Eds.), The Bottlenose Dolphin. Academic Press, San Diego, CA. pp. 387–402.

Sellas, A.B., R.S. Wells, and P.E. Rosel. 2005. Mitochondrial and nuclear DNA analyses reveal fine scale geographic structure in bottlenose dolphins (*Tursiops truncatus*) in the Gulf of Mexico. Conservation Genetics 6(5):715–728. doi: 10.1007/s10592-005-9031-7

Sergeant, D.E., D.K. Caldwell, and M.C. Caldwell. 1973. Age, growth, and maturity of bottlenosed dolphin (*Tursiops-truncatus*) from northeast Florida. Journal of the Fisheries Research Board of Canada 30(7):1009–1011. doi: 10.1139/f73-165

Shane, S.H. 1990. Behavior and ecology of the bottlenose dolphin at Sanibel Island, Florida. In: Leatherwood, S. and R.R. Reeves (Eds.), The Bottlenose Dolphin. Academic Press, San Diego, CA. pp. 245–265. doi: 10.1016/B978-0-12-440280-5.50016-0

Shane, S.H. 2004. Residence patterns, group characteristics, and association patterns of bottlenose dolphins near Sanibel Island, Florida. Gulf of Mexico Science 22(1):1–12. doi: 10.18785/goms.2201.01

Shane, S.H., R.S. Wells, and B. Würsig. 1986. Ecology, behavior and social organization of the bottlenose dolphin: A review. Marine Mammal Science 2:34–63. doi: 10.1111/j.1748-7692.1986.tb00026.x

Smith, C.R., T.K. Rowles, L.B. Hart, F.I. Townsend, R.S. Wells, E.S. Zolman, B.C. Balmer, B. Quigley, M. Ivančić, W. McKercher, M.C. Tumlin, K.D. Mullin, J.D. Adams, Q. Wu, W. McFee, T.K. Collier, and L.H. Schwacke. 2017. Slow recovery of Barataria Bay dolphin health following the Deepwater Horizon oil spill (2013-2014), with evidence of persistent lung disease and impaired stress response. Endangered Species Research 33:127–142. doi: 10.3354/esr00778

Takeshita, R., B.C. Balmer, F. Messina, E.S. Zolman, L. Thomas, R.S. Wells, C.R. Smith, T.K. Rowles, and L.H. Schwacke. 2021. High site-fidelity in common bottlenose dolphins despite low salinity exposure and associated indicators of compromised health. PLoS ONE 16(9):e0258031. doi: 10.1371/journal.pone.0258031

Taylor, B.L., S.J. Chivers, J. Larese, and W.F. Perrin. 2007. Generation length and percent mature estimates for IUCN assessments of cetaceans. National Oceanic and Atmospheric Administration, National Marine Fisheries Service, Southwest Fisheries Science Center: La Jolla, CA. Administrative Report LJ-07-01. 24 p.

Toms, C. 2019. Filling the gaps: Common bottlenose dolphin (*Tursiops truncatus*) population dynamics, structure, and connectivity within Florida panhandle bays, sounds, and estuaries. University of Central Florida. Dissertation. 373 p.

Torres, L.G. and A.J. Read. 2009. Where to catch a fish? The influence of foraging tactics on the ecology of bottlenose dolphins (*Tursiops truncatus*) in Florida Bay, Florida. Marine Mammal Science 25(4):797–815. doi: 10.1111/j.1748-7692.2009.00297.x

Tyson, R.B., S.M. Nowacek, and D.P. Nowacek. 2011. Community structure and abundance of bottlenose dolphins *Tursiops truncatus* in coastal waters of the northeast Gulf of Mexico. Marine Ecology Progress Series 438:253–265. doi: 10.3354/meps09292

Urian, K.W., S. Hofmann, R.S. Wells, and A.J. Read. 2009. Fine-scale population structure of bottlenose dolphins (*Tursiops truncatus*) in Tampa Bay, Florida. Marine Mammal Science 25(3):619–638. doi: 10.1111/j.1748-7692.2009.00284.x

Van Bressem, M.F., P.J. Duignan, A. Banyard, M. Barbieri, K.M. Colegrove, S. De Guise, G. Di Guardo, A. Dobson, M. Domingo, D. Fauquier, A. Fernandez, T. Goldstein, B. Grenfell, K.R. Groch, F. Gulland, B.A. Jensen, P.D. Jepson, A. Hall, T. Kuiken, S. Mazzariol, S.E. Morris, O. Nielsen, J.A. Raga, T.K. Rowles, J. Saliki, E. Sierra, N. Stephens, B. Stone, I. Tomo, J. Wang, T. Waltzek, and J.F. Wellehan. 2014. Cetacean morbillivirus: current knowledge and future directions. Viruses 6(12):5145–5181. doi: 10.3390/v6125145

Venn-Watson, S., L. Garrison, J. Litz, E. Fougeres, B. Mase, G. Rappucci, E. Stratton, R. Carmichael, D. Odell, D. Shannon, S. Shippee, S. Smith, L. Staggs, M. Tumlin, H. Whitehead, and T. Rowles. 2015. Demographic clusters identified within the northern Gulf of Mexico common bottlenose dolphin (*Tursiops truncates*) unusual mortality event: January 2010-June 2013. PLoS ONE 10(2):e0117248. doi: 10.1371/journal.pone.0117248

Vollmer, N.L. and P.E. Rosel. 2013. A review of common bottlenose dolphins (*Tursiops truncatus truncatus*) in the Northern Gulf of Mexico: Population biology, potential threats, and management. Southeastern Naturalist 12(m6):1–43. doi: 10.1656/058.012.m601

Weller, D.W. 1998. Global and regional variation in the biology and behavior of bottlenose dolphins. Texas A&M University. Dissertation. 142 p.

Wells, R.S. 1986a. Population structure of bottlenose dolphins: Behavioral studies along the central west coast of Florida. Contract Report to National Marine Fisheries Service, Southeast Fisheries Center. Contract No. 45-WCNF-5-003BB. 58 p.

Wells, R.S. 1986b. Structural aspects of dolphin societies. University of California, Santa Cruz. Dissertation. 234 p.

Wells, R.S. 1991. The role of long-term study in understanding the social structure of a bottlenose dolphin community. In: Pryor, K. and K. Norris (Eds.), Dolphin Societies: Discoveries and Puzzles. University of California Press, Berkeley, CA. pp. 199–226.

Wells, R.S. 2000. Reproduction in wild bottlenose dolphins: Overview of patterns observed during a long-term study. In: Duffield, D. and T. Robeck (Eds.), Bottlenose Dolphin Reproduction Workshop. AZA Marine Mammal Taxon Advisory Group, Silver Spring, MD. pp. 57–74.

Wells, R.S. 2003. Dolphin social complexity: Lessons from long-term study and life history. In: de Waal, F.B.M. and P.L. Tyack (Eds.), Animal Social Complexity: Intelligence, Culture, and Individualized Societies Harvard University Press, Cambridge, MA. pp. 32–56.

Wells, R.S. 2014. Social structure and life history of bottlenose dolphins near Sarasota Bay, Florida: Insights from four decades and five generations. Primates and cetaceans. pp. 149–172. doi: 10.1007/978-4-431-54523-1_8

Wells, R.S., M.K. Bassos, K.W. Urian, W.J. Carr, and M.D. Scott. 1996. Low-level monitoring of bottlenose dolphins, *Tursiops truncatus*, in Charlotte Harbor, Florida: 1990-1994. National Oceanic and Atmospheric Administration, National Marine Fisheries Service, Southeast Fisheries Science Center: Miami, FL. NOAA Technical Memorandum NMFS-SEFSC-384. 86 p.

Wells, R.S., M.K. Bassos, K.W. Urian, S.H. Shane, E.C.G. Owen, C.F. Weiss, W.J. Carr, and M.D. Scott. 1997. Low-level monitoring of bottlenose dolphins, *Tursiops truncatus*, in Pine Island Sound, Florida: 1996. National Oceanic and Atmospheric Administration, National Marine Fisheries Service, Southeast Fisheries Science Center: Miami, FL. Southeast Fisheries Center Contribution No. 40-WCNF601958. 86 p.

Wells, R.S., A. Natoli, and G. Braulik. 2019. *Tursiops truncatus* (errata version published in 2019). *The IUCN Red List of Threatened Species* 2019: e.T22563A156932432. https://dx.doi.org/10.2305/IUCN.UK.2019-1.RLTS.T22563A156932432.en

Wells, R.S., L.H. Schwacke, T.K. Rowles, B.C. Balmer, E. Zolman, T. Speakman, F.I. Townsend, M.C. Tumlin, A. Barleycorn, and K.A. Wilkinson. 2017. Ranging patterns of common bottlenose dolphins *Tursiops truncatus* in Barataria Bay, Louisiana, following the Deepwater Horizon oil spill. Endangered Species Research 33:159–180. doi: 10.3354/esr00732

Wells, R.S. and M.D. Scott. 1999. Bottlenose dolphin *Tursiops truncatus* (Montagu, 1821). In: Ridgway, S.H. and R. Harrison (Eds.), Handbook of marine mammals. Volume 6: The second book of dolphins and the porpoises. Academic Press, San Diego, CA. pp. 137–182.

Wells, R.S. and M.D. Scott. 2018. Bottlenose dolphin, *Tursiops truncatus*, common bottlenose dolphin. In: Würsig, B., J.G.M. Thewissen, and K.M. Kovacs (Eds.), Encyclopedia of Marine Mammals (Third Edition). Academic Press, London, UK. pp. 118–125. doi: 10.1016/B978-0-12-804327-1.00072-8

Wells, R.S., M.D. Scott, and A.B. Irvine. 1987. The social structure of free-ranging bottlenose dolphins. In: Genoways, H.H. (Ed.), Current Mammalogy. Plenum Press, New York, NY. pp. 247–305.

Wells, R.S., K.W. Urian, A.J. Read, M.K. Bassos, W.J. Carr, and M.D. Scott. 1996. Low-level monitoring of bottlenose dolphins, *Tursiops truncatus*, in Tampa Bay, Florida: 1988-1993. National Oceanic and Atmospheric Administration, National Marine Fisheries Service, Southeast Fisheries Science Center: Miami, FL. NOAA Technical Memorandum NMFS-SEFSC-385. 36 p.

Wilson, R.M., R.B. Tyson, J.A. Nelson, B.C. Balmer, J.P. Chanton, and D.P. Nowacek. 2017. Niche differentiation and prey selectivity among common bottlenose dolphins (*Tursiops truncatus*) sighted in St. George Sound, Gulf of Mexico. Frontiers in Marine Science 4:235. doi: 10.3389/fmars.2017.00235

Common bottlenose dolphin − *Tursiops truncatus*

GoMx BSE/Vermilion Bay, West Cote Blanche Bay, Atchafalaya Bay Stock

Overall Vulnerability Rank = Very High

Biological Sensitivity = Very High

Climate Exposure = Very High

Data Quality = 89% of scores ≥ 2

	Tursiops truncatus	Expert Scores	Data Quality	Expert Scores Plots (Portion by Category)
Sensitivity attributes	Prey/Diet Specificity	1.9	2.0	
	Habitat Specificity	3.8	2.0	
	Site Fidelity	3.7	2.0	
	Lifetime Reproductive Potential	1.4	2.0	
	Generation Length	2.9	2.3	
	Reproductive Plasticity	1.3	2.0	
	Migration	3.9	2.0	
	Home Range	3.5	2.0	
	Stock Abundance	3.7	1.7	
	Stock Abundance Trend	2.3	1.0	
	Cumulative Stressors	2.7	2.3	
	Sensitivity Score	Very High		
Exposure variables	Sea Surface Temperature (Change in mean)	4.0	2.7	
	Sea Surface Temperature (Change in variability)	1.0	2.7	
	Air Temperature (Change in mean)	4.0	2.7	
	Air Temperature (Change in variability)	1.0	2.7	
	Precipitation (Change in mean)	1.0	2.7	
	Precipitation (Change in variability)	1.0	2.7	
	Sea Surface Salinity (Change in mean)	1.2	2.7	
	Sea Surface Salinity (Change in variability)	1.0	2.7	
	Ocean pH (Change in mean)	4.0	2.7	
	Ocean pH (Change in variability)	1.0	2.7	
	Sea ice coverage (Change in mean)	1.0	3.0	
	Sea ice coverage (Change in variability)	1.0	3.0	
	Dissolved oxygen (Change in mean)	4.0	2.7	
	Dissolved oxygen (Change in variability)	1.0	2.7	
	Circulation	3.8	1.7	
	Sea level rise	4.0	2.7	
	Exposure Score	Very High		
Overall Vulnerability Rank		Very High		

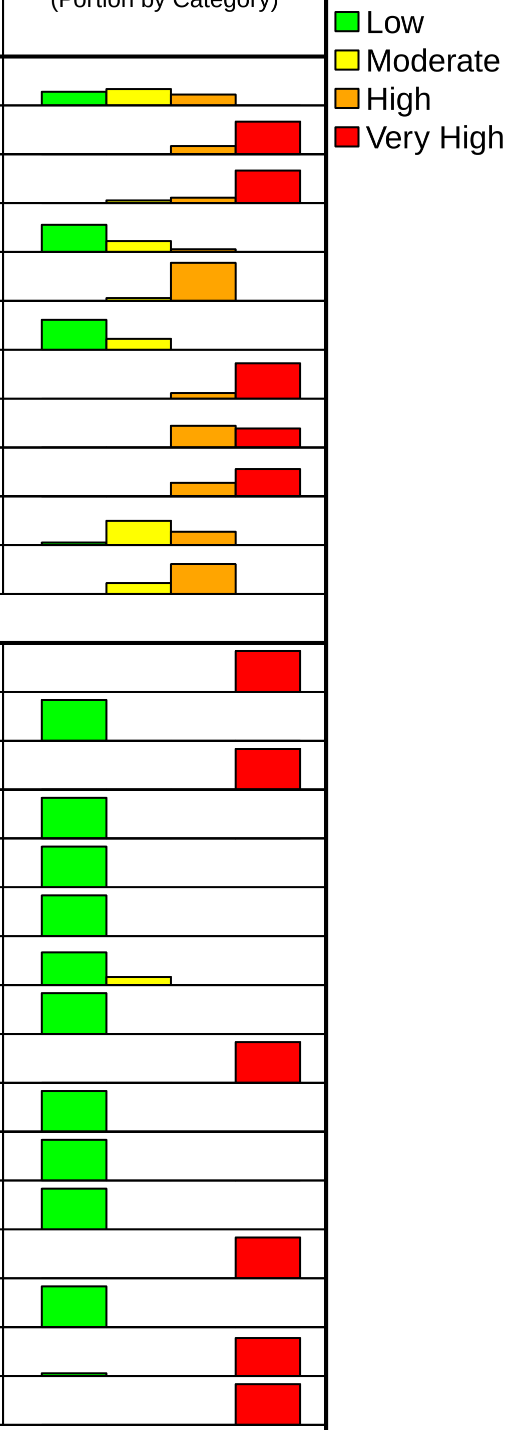

Common bottlenose dolphin (GoMx BSE/Vermilion Bay, West Cote Blanche Bay, Atchafalaya Bay Stock)

Tursiops truncatus

CVA Results Summary

Overall Climate Vulnerability Rank: Very High (100% certainty from bootstrap analysis).

Climate Exposure: Very High. Six exposure factors scored greater than 3.5: Air Temperature (Standard anomaly) (4.0), Dissolved oxygen (Standard anomaly) (4.0), Ocean pH (Standard anomaly) (4.0), Sea level rise (4.0), Sea Surface Temperature (Standard anomaly) (4.0), and Circulation (3.8).

Biological Sensitivity: Very High. Four sensitivity attributes scored greater than 3.5: Migration (3.87), Habitat Specificity (3.80), Site Fidelity (3.73), and Species Abundance (3.67).

Distributional Response: Very High

Abundance Response: Very High

Phenology Response: Very High

Data Quality: 89% of the data quality scores were 2 or greater, 82% of sensitivity attribute data quality scores were 2 or higher.

Background Information

Stock Description

This stock includes common bottlenose dolphins in Vermilion Bay, West Cote Blanche Bay, and Atchafalaya Bay in the northern Gulf of Mexico along the Louisiana coast (Hayes et al. 2022).

Prey/Diet Specificity

Generally, common bottlenose dolphins consume a large variety of fish and/or squid (Barros and Odell 1990; Mead and Potter 1990; Wells and Scott 2018). Common bottlenose dolphins seem to show a consistent preference for sound-producing fishes (e.g., sciaenids, toadfish; Berens McCabe et al. 2010; Bowen-Stevens et al. 2021). In Sarasota Bay, Florida, Rossman et al. (2015) found some individual common bottlenose dolphins to show diet specialization despite the broader study group showing a generalist diet. Specialization can be influenced by locally abundant prey species (Shane et al. 1986; Vollmer and Rosel 2013) or can result from resource partitioning amongst sympatric populations (e.g., Torres and Read 2009; Wilson et al. 2017).

Habitat Specificity

Common bottlenose dolphins are able to adapt to a variety of marine and estuarine habitats (Wells and Scott 2018).

Site Fidelity

No specific site fidelity information was found for this stock. However, common bottlenose dolphins in other Gulf of Mexico bay, sound, and estuary (BSE) stocks show site fidelity (LaBrecque et al. 2015; Hayes et al. 2022), such as in Barataria Bay in Louisiana (Miller 2003; McDonald et al. 2017; Wells et al. 2017; Takeshita et al. 2021); Matagorda Bay, Espiritu Santo Bay, and San Luis Pass in Texas (Maze and Würsig 1999; Lynn and Würsig 2002; Irwin and Würsig 2004); Mississippi Sound in Mississippi (Hubard et al. 2004; Mackey 2010; Mullin et al. 2017); Charlotte Harbor and Pine Island Sound (Bassos-Hull et al. 2013), Sarasota Bay (Wells 1986a; Wells 1991; Wells 2014), St. Andrew Bay (Balmer et al. 2019), St. Joseph Bay (Balmer et al. 2008; B. Balmer et al. 2018), St. Vincent Sound and Apalachicola Bay (Tyson et al. 2011), and Tampa Bay in Florida (Wells et al. 1996b; Urian et al. 2009).

Lifetime Reproductive Potential

Taylor et al. (2007) reported the interbirth interval of common bottlenose dolphins as 3.80 years, though other studies have shown it to be closer to three years (Leatherwood 1977; Wells and Scott 1999; Connor et al. 2000; Wells 2003).

Generally, females reach sexual maturity before males, with females reaching sexual maturity at 5–13 years (Sergeant et al. 1973; Perrin and Reilly 1984; Wells et al. 1987; Mead and Potter 1990; Mann et al. 2000; Wells 2000; Wells and Scott 2018).

Females reproduce through most of their adult lives (Leatherwood 1977; Hohn et al. 1989; Wells and Scott 1999; Wells 2000; Wells 2003), but may produce fewer calves after they reach 30 years of age (Wells 2000). Sarasota Bay common bottlenose dolphins can give birth when they are as young as 6 years of age, and can continue until they are 48 years old (Wells and Scott 2018). Reproductive lifespan would therefore be up to 42 years based on long-term observation studies (Wells 2014; Wells and Scott 2018).

Longitudinal studies from Sarasota Bay, Florida have found that female common bottlenose dolphins can live up to 67 years (Wells and Scott 2018). Studies based on growth layer groups in teeth suggest female common bottlenose dolphins can live to 41 years along the Texas coast (Fernandez and Hohn 1998) and to 30 years along the Mississippi coast (Mattson et al. 2006).

Generation Length

Taylor et al. (2007) reported common bottlenose dolphin generation length of 20.6–21.1 years.

Reproductive Plasticity

Common bottlenose dolphin calving is generally diffusely seasonal, with one or more peaks, although births have been reported from all seasons (Vollmer and Rosel 2013; Wells and Scott 2018). Calf sightings peak in the spring and summer months in Florida (Wells et al. 1987), Mississippi/Louisiana (Miller et al. 2010; Miller et al. 2013), and Texas (McHugh 1989; Henderson et al. 2007).

Migration

The BSE stocks in the Gulf of Mexico are thought to primarily comprise stable resident communities. However, transient or seasonal movements of some individuals into and out of a BSE stock area are recorded in some areas (LaBrecque et al. 2015; Hayes et al. 2022). Molecular genetic data support that some BSE stocks are genetically distinct from one another, and furthermore are differentiated from adjacent coastal populations (e.g., Sellas et al. 2005; Rosel et al. 2017). For example, Toms (2019) found fine-scale genetic population structure among five inshore systems in the Florida Panhandle and variable migration rates among populations.

Year-round residency has been reported for common bottlenose dolphins (Hayes et al. 2022), such as in Florida (Irvine and Wells 1972; Irvine et al. 1981; Wells 1986a; Wells 1986b; Wells et al. 1987; Scott et al. 1990; Wells 1991; Wells et al. 1996a; Wells et al. 1996b; Wells et al. 1997; Wells 2003; Balmer et al. 2008; Urian et al. 2009; Tyson et al. 2011; Bassos-Hull et al. 2013; B. Balmer et al. 2018; Balmer et al. 2019; Toms 2019), Texas (Shane 1990; Bräger 1993; Bräger et al. 1994; Fertl 1994; Weller 1998; Maze and Würsig 1999; Lynn and Würsig 2002; Irwin and Würsig 2004; Shane 2004), and Louisiana/Mississippi (Hubard et al. 2004; McDonald et al. 2017; Mullin et al. 2017; Takeshita et al. 2021).

Home Range

The home ranges of individuals within the Gulf of Mexico BSE stocks are considered to generally include the areas within the stock boundaries and brief excursions beyond the stock boundaries (Irvine et al. 1981; Wells 1986a; Wells 1986b; Wells 1991; Wells et al. 1996a; Wells et al. 1996b; Balmer et al. 2008; Wells 2014; Balmer et al. 2019), although some individuals show preference for small areas within the stock boundaries (Lynn and Würsig 2002; Urian et al. 2009; Mullin et al. 2017; Wells et al. 2017).

Stock Abundance

An abundance estimate for this stock is unavailable (Hayes et al. 2022).

Stock Abundance Trend

Data are insufficient to complete an abundance trend analysis for this stock (Hayes et al. 2022).

Cumulative Stressors

Common bottlenose dolphins face a wide range of individual and cumulative natural and anthropogenic threats (see reviews by Vollmer and Rosel 2013; Wells and Scott 2018; and Wells et al. 2019). These include such threats as shark predation, harmful algal blooms, recreational and commercial fishing gear, vessel traffic, pollution, habitat alteration, and provisioning.

Common bottlenose dolphins in this stock were exposed to oil and dispersants following the Deepwater Horizon oil spill, which may result in long term reproductive and chronic health effects (NOAA 2011; Michel et al. 2013; Helm et al. 2015; DWH NRDA 2016; Lane et al. 2015; De Guise et al. 2017, 2021; Kellar et al. 2017; Smith et al. 2017; Schwacke et al. 2017). An Unusual Mortality Event (UME), involving primarily bottlenose dolphins, was declared for cetaceans in the northern Gulf of Mexico from 2010 to 2014, with the Deepwater Horizon oil spill as the primary underlying cause (Litz et al. 2014; Schwacke et al. 2014; Venn-Watson et al. 2015a, 2015b).

The nearshore and estuarine habitats along the Gulf of Mexico coast include waters adjacent to areas of high human population, some of which are highly industrialized, and areas of heavy agricultural impact. Studies of bottlenose dolphin tissues from nearby Barataria Bay have shown persistent organic pollutants (POPs) in blubber and blood samples, though at concentrations lower than other southeastern U.S. sites (Kucklick et al. 2011; Balmer et al. 2015; J. Balmer et al. 2018). Similarly, mercury has been detected in common bottlenose dolphin tissues in Louisiana waters, but in lower concentrations than common bottlenose dolphins found in Florida waters (McCormack et al. 2020a, 2020b, 2022).

Dolphin morbillivirus has been detected in populations of the northern Gulf of Mexico, but has not been attributed to large scale die-offs since 1993−1994 (Lipscomb et al. 1996; Litz et al. 2014; Van Bressem et al. 2014; Fauquier et al. 2017). A UME was declared in 1990 for the Gulf of Mexico from Florida to Texas, with more than 300 common bottlenose dolphins stranding due to an undetermined cause, although morbillivirus and cold temperatures may have contributed (Litz et al. 2014).

Further Reading

Hayes, S.A., E. Josephson, K. Maze-Foley, P.E. Rosel, and J. Wallace. 2022. U.S. Atlantic and Gulf of Mexico marine mammal stock assessments 2021. National Oceanic and Atmospheric Administration, National Marine Fisheries Service, Northeast Fisheries Science Center: Woods Hole, MA. NOAA Technical Memorandum NMFS-NE-288. 380 p. doi: 10.25923/6tt7-kc16

Wells, R.S. and M.D. Scott. 2018. Bottlenose Dolphin, *Tursiops truncatus*, Common Bottlenose Dolphin. In: Würsig, B., J.G.M. Thewissen, and K.M. Kovacs (Eds.), Encyclopedia of Marine Mammals (Third Edition). Academic Press, London, UK. pp. 118–125. doi: 10.1016/B978-0-12-804327-1.00072-8

Wells, R.S., A. Natoli, and G. Braulik. 2019. *Tursiops truncatus* (errata version published in 2019). The IUCN Red List of Threatened Species 2019: e.T22563A156932432. doi: 10.2305/IUCN.UK.2019-1.RLTS.T22563A156932432.en

References

Balmer, B., T. McDonald, F. Hornsby, J. Adams, J. Allen, A. Barleycorn, and L. Schwacke. 2018. Long-term trends in a northern Gulf of Mexico common bottlenose dolphin (*Tursiops truncatus*) population in the wake of the Deepwater Horizon oil spill. Journal of Cetacean Research and Management 18:1–9. doi: 10.47536/jcrm.v18i1.409

Balmer, B., S. Watwood, B. Quigley, T. Speakman, K. Barry, K. Mullin, P. Rosel, C. Sinclair, E. Zolman, and L. Schwacke. 2019. Common bottlenose dolphin (*Tursiops truncatus*) abundance and distribution patterns in St. Andrew Bay, Florida, USA. Aquatic Conservation: Marine and Freshwater Ecosystems 29(3):486–498. doi: 10.1002/aqc.3001

Balmer, B.C., R.S. Wells, S.M. Nowacek, D.P. Nowacek, L.H. Schwacke, W.A. McLellan, F.S. Scharf, T.K. Rowles, L.J. Hansen, T.R. Spradlin, and D.A. Pabst. 2008. Seasonal abundance and distribution patterns of common bottlenose dolphins (*Tursiops truncatus*) near St. Joseph Bay, Florida, USA. Journal of Cetacean Research and Management 10(2):157–167.

Balmer, B.C., G.M. Ylitalo, L.E. McGeorge, K.A. Baugh, D. Boyd, K.D. Mullin, P.E. Rosel, C. Sinclair, R.S. Wells, E.S. Zolman, and L.H. Schwacke. 2015. Persistent organic pollutants (POPs) in blubber of common bottlenose dolphins (*Tursiops truncatus*) along the northern Gulf of Mexico coast, USA. Science of the Total Environment 527-528:306–312. doi: 10.1016/j.scitotenv.2015.05.016

Balmer, J.E., G.M. Ylitalo, T.K. Rowles, K.D. Mullin, R.S. Wells, F.I. Townsend, R.W. Pearce, J.L. Bolton, E.S. Zolman, B.C. Balmer, and L.H. Schwacke. 2018. Persistent organic pollutants (POPs) in blood and blubber of common bottlenose dolphins (*Tursiops truncatus*) at three northern Gulf of Mexico sites following the Deepwater Horizon oil spill. Science of the Total Environment 621:130–137. doi: 10.1016/j.scitotenv.2017.11.209

Barros, N.B. and D.K. Odell. 1990. Food habits of bottlenose dolphins in the southeastern United States In: Leatherwood, S. and R.R. Reeves (Eds.), The Bottlenose Dolphin. Academic Press, San Diego, CA. pp. 309–328. doi: 10.1016/B978-0-12-440280-5.50020-2

Bassos-Hull, K., R.M. Perrtree, C.C. Shepard, S. Schilling, A.A. Barleycorn, J.B. Allen, B.C. Balmer, W.E. Pine, and R.S. Wells. 2013. Long-term site fidelity and seasonal abundance estimates of common bottlenose dolphins (*Tursiops truncatus*) along the southwest coast of Florida and responses to natural perturbations. Journal of Cetacean Research and Management 13(1):19–30. doi: 10.47536/jcrm.v13i1.551

Berens McCabe, E.J., D.P. Gannon, N.B. Barros, and R.S. Wells. 2010. Prey selection by resident common bottlenose dolphins (*Tursiops truncatus*) in Sarasota Bay, Florida. Marine Biology 157(5):931–942. doi: 10.1007/s00227-009-1371-2

Bowen-Stevens, S.R., D.P. Gannon, R.A. Hazelkorn, G. Lovewell, K.M. Volker, S. Smith, M.C. Tumlin, and J. Litz. 2021. Diet of common bottlenose dolphins, *Tursiops truncatus*, that stranded in and near Barataria Bay, Louisiana, 2010–2012. Southeastern Naturalist 20(1). doi: 10.1656/058.020.0113

Bräger, S. 1993. Diurnal and seasonal behavior patterns of bottlenose dolphins (*Tursiops truncatus*). Marine Mammal Science 9:434–438. doi: 10.1111/j.1748-7692.1993.tb00477.x

Bräger, S., B. Würsig, A. Acevedo, and T. Henningsen. 1994. Association patterns of bottle-nosed dolphins (*Tursiops-truncatus*) in Galveston Bay, Texas. Journal of Mammalogy 75(2):431–437. doi: 10.2307/1382564

Connor, R.C., R.S. Wells, J. Mann, and A.J. Read. 2000. The bottlenose dolphin: Social relationships in a fission-fusion society. In: Mann, J., R. Connor, P. Tyack, and H. Whitehead (Eds.), Cetacean Societies: Field Studies of Dolphins and Whales. University of Chicago Press, Chicago, IL. pp. 91–126.

De Guise, S., M. Levin, E. Gebhard, L. Jasperse, L. Burdett Hart, C.R. Smith, S. Venn-Watson, F. Townsend, R. Wells, B. Balmer, E. Zolman, T. Rowles, and L. Schwacke. 2017. Changes in immune functions in bottlenose dolphins in the northern Gulf of Mexico associated with the Deepwater Horizon oil spill. Endangered Species Research 33:291–303. doi: 10.3354/esr00814

De Guise, S., M. Levin, L. Jasperse, J. Herrman, R.S. Wells, T. Rowles, and L. Schwacke. 2021. Long-term immunological alterations in bottlenose dolphin a decade after the Deepwater Horizon oil spill in the northern Gulf of Mexico: Potential for multigenerational effects. Environmental Toxicology and Chemistry 40(5):1308–1321. doi: 10.1002/etc.4980

Deepwater Horizon Natural Resource Damage Assessment Trustees (DWH NRDA). 2016. Deepwater Horizon oil spill: Final Programmatic Damage Assessment and Restoration Plan and Final Programmatic Environmental Impact Statement. Retrieved from: http://www.gulfspillrestoration.noaa.gov/restoration-planning/gulf-plan.1659.

Fauquier, D.A., J. Litz, S. Sanchez, K. Colegrove, L.H. Schwacke, L. Hart, J. Saliki, C. Smith, T. Goldstein, S. Bowen-Stevens, W. McFee, E. Fougeres, B. Mase-Guthrie, E. Stratton, R. Ewing, S. Venn-Watson, R.H. Carmichael, C. Clemons-Chevis, W. Hatchett, D. Shannon, S. Shippee, S. Smith, L. Staggs, M.C. Tumlin, N.L. Wingers, and T.K. Rowles. 2017. Evaluation of morbillivirus exposure in cetaceans from the northern Gulf of Mexico 2010-2014. Endangered Species Research 33:211–220. doi: 10.3354/esr00772

Fernandez, S. and A.A. Hohn. 1998. Age, growth, and calving season of bottlenose dolphins, *Tursiops truncatus*, off coastal Texas. Fishery Bulletin 96(2):357–365.

Fertl, D. 1994. Occurrence patterns and behavior of bottlenose dolphins (*Tursiops truncatus*) in the Galveston ship channel, Texas. Texas Journal of Science 46(4):299–318.

Hayes, S.A., E. Josephson, K. Maze-Foley, P.E. Rosel, and J. Wallace. 2022. U.S. Atlantic and Gulf of Mexico marine mammal stock assessments 2021. National Oceanic and Atmospheric Administration, National Marine Fisheries Service, Northeast Fisheries Science Center: Woods Hole, MA. NOAA Technical Memorandum NMFS-NE-288. 380 p. doi: 10.25923/6tt7-kc16

Helm, R.C., D.P. Costa, T.D. DeBruyn, T.J. O'Shea, R.S. Wells, and T.M. Williams. 2015. Overview of effects of oil spills on marine mammals. In: Fingas, M. (Ed.), Handbook of oil spill science and technology, First edition. John Wiley & Sons, Inc., Hoboken, NJ. pp. 455–475.

Henderson, E.E. and B. Würsig. 2007. Behavior patterns of bottlenose dolphins in San Luis Pass, Texas. Gulf of Mexico Science 25(2):153–161. doi: 10.18785/goms.2502.06

Hohn, A.A., M.D. Scott, R.S. Wells, J.C. Sweeney, and A.B. Irvine. 1989. Growth layers in teeth from known-age, free-ranging bottlenose dolphins. Marine Mammal Science 5(4):315–342. doi: 10.1111/j.1748-7692.1989.tb00346.x

Hubard, C.W., K. Maze-Foley, K.D. Mullin, and W.W. Schroeder. 2004. Seasonal abundance and site fidelity of bottlenose dolphins (*Tursiops truncatus*) in Mississippi Sound. Aquatic Mammals 30(2):299–310. doi: 10.1578/am.30.2.2004.299

Irvine, A.B., M.D. Scott, R.S. Wells, and J.H. Kaufmann. 1981. Movements and activities of the Atlantic bottlenose dolphin, *Tursiops truncatus*, near Sarasota, Florida. Fishery Bulletin 79(4):671–688.

Irvine, B. and R.S. Wells. 1972. Results of attempts to tag Atlantic bottlenosed dolphins (*Tursiops truncatus*). Cetology 13:1–5.

Irwin, L.-J. and B. Würsig. 2004. A small resident community of bottlenose dolphins, *Tursiops truncatus*, in Texas: Monitoring recommendations. Gulf of Mexico Science 22(1):13–21. doi: 10.18785/goms.2201.02

Kellar, N.M., T.R. Speakman, C.R. Smith, S.M. Lane, B.C. Balmer, M.L. Trego, K.N. Catelani, M.N. Robbins, C.D. Allen, R.S. Wells, E.S. Zolman, T.K. Rowles, and L.H. Schwacke. 2017. Low reproductive success rates of common bottlenose dolphins *Tursiops truncatus* in the northern Gulf of Mexico following the Deepwater Horizon disaster (2010-2015). Endangered Species Research 33:143–158. doi: 10.3354/esr00775

Kucklick, J., L. Schwacke, R. Wells, A. Hohn, A. Guichard, J. Yordy, L. Hansen, E. Zolman, R. Wilson, J. Litz, D. Nowacek, T. Rowles, R. Pugh, B. Balmer, C. Sinclair, and P. Rosel. 2011. Bottlenose dolphins as indicators of persistent organic pollutants in the western North Atlantic Ocean and Northern Gulf of Mexico. Environmental Science & Technology 45(10):4270–4277. doi: 10.1021/es1042244

LaBrecque, E., C. Curtice, J. Harrison, S.M. Van Parijs, and P.N. Halpin. 2015. 3. Biologically Important Areas for cetaceans within U.S. Waters – Gulf of Mexico Region. Aquatic Mammals 41(1):30–38. doi: 10.1578/am.41.1.2015.30

Lane, S.M., C.R. Smith, J. Mitchell, B.C. Balmer, K.P. Barry, T. McDonald, C.S. Mori, P.E. Rosel, T.K. Rowles, T.R. Speakman, F.I. Townsend, M.C. Tumlin, R.S. Wells, E.S. Zolman, and L.H. Schwacke. 2015. Reproductive outcome and survival of common bottlenose dolphins sampled in Barataria Bay, Louisiana, USA, following the Deepwater Horizon oil spill. Proceedings of the Royal Society B: Biological Sciences 282(1818):20151944. doi: 10.1098/rspb.2015.1944

Leatherwood, S. 1977. Some preliminary impressions on the numbers and social behavior of free-swimming bottlenosed dolphin calves (*Tursiops truncatus*) in the northern Gulf of Mexico. In: Ridgway, S.H. and K. Bernirschke (Eds.), Breeding Dolphins; Present Status, Suggestions for the Future. Final report to the US Marine Mammal Commission, Contract MM6AC009, MMC-76/07, Washington, DC. pp. 143–167.

Lipscomb, T.P., S. Kennedy, D. Moffett, A. Krafft, B.A. Klaunberg, J.H. Lichy, G.T. Regan, G.A.J. Worthy, and J.K. Taubenberger. 1996. Morbilliviral epizootic in bottlenose dolphins of the Gulf of Mexico. Journal of Veterinary Diagnostic Investigation 8(3):283–290. doi: 10.1177/104063879600800302

Litz, J.A., M.A. Baran, S.R. Bowen-Stevens, R.H. Carmichael, K.M. Colegrove, L.P. Garrison, S.E. Fire, E.M. Fougeres, R. Hardy, S. Holmes, W. Jones, B.E. Mase-Guthrie, D.K. Odell, P.E. Rosel, J.T. Saliki, D.K. Shannon, S.F. Shippee, S.M. Smith, E.M. Stratton, M.C. Tumlin, H.R. Whitehead, G.A. Worthy, and T.K. Rowles. 2014. Review of historical unusual mortality events (UMEs) in the Gulf of Mexico (1990–2009): providing context for the multi-year northern Gulf of Mexico cetacean UME declared in 2010. Diseases of Aquatic Organisms 112(2):161–175. doi: 10.3354/dao02807

Lynn, S.K. and B. Würsig. 2002. Summer movement patterns of bottlenose dolphins in a Texas bay. Gulf of Mexico Science 20(1):25–37. doi: 10.18785/goms.2001.03

Mackey, A.D. 2010. Site fidelity and association patterns of bottlenose dolphins (*Tursiops truncatus*) in the Mississippi Sound. University of Southern Mississippi. Thesis. 133 p.

Mann, J., R.C. Connor, L.M. Barre, and M.R. Heithaus. 2000. Female reproductive success in bottlenose dolphins (*Tursiops* sp.): life history, habitat, provisioning, and group-size effects. Behavioral Ecology 11(2):210–219. doi: 10.1093/beheco/11.2.210

Mattson, M.C., K.D. Mullin, G.W. Ingram, and W. Hoggard. 2006. Age structure and growth of the bottlenose dolphin (*Tursiops truncatus*) from strandings in the Mississippi sound region of the north-central Gulf of Mexico from 1986 to 2003. Marine Mammal Science 22(3):654–666. doi: 10.1111/j.1748-7692.2006.00057.x

Maze, K.S. and B. Würsig. 1999. Bottlenosed dolphins of San Luis Pass, Texas: Occurrence patterns, site-fidelity, and habitat use. Aquatic Mammals 25:91–104.

McCormack, M.A., F. Battaglia, W.E. McFee, and J. Dutton. 2020a. Mercury concentrations in blubber and skin from stranded bottlenose dolphins (*Tursiops truncatus*) along the Florida and Louisiana coasts (Gulf of Mexico, USA) in relation to biological variables. Environmental Research 180:108886. doi: 10.1016/j.envres.2019.108886

McCormack, M.A., B.P. Jackson, and J. Dutton. 2020b. Relationship between mercury and selenium concentrations in tissues from stranded odontocetes in the northern Gulf of Mexico. Science of the Total Environment 749:141350. doi: 10.1016/j.scitotenv.2020.141350

McCormack, M.A., W.H. Nowlin, and J. Dutton. 2022. Effect of trophic position on mercury concentrations in bottlenose dolphins (*Tursiops truncatus*) from the northern Gulf of Mexico. Environmental Research 204(Pt B):112124. doi: 10.1016/j.envres.2021.112124

McDonald, T.L., F.E. Hornsby, T.R. Speakman, E.S. Zolman, K.D. Mullin, C. Sinclair, P.E. Rosel, L. Thomas, and L.H. Schwacke. 2017. Survival, density, and abundance of common bottlenose dolphins in Barataria Bay (USA) following the Deepwater Horizon oil spill. Endangered Species Research 33:193–209. doi: 10.3354/esr00806

McHugh, M.B. 1989. Population numbers and feeding behavior of the Atlantic bottlenose dolphin (*Tursiops truncatus*) near Aransas Pass, Texas. University of Texas at Austin. Thesis. 96 p.

Mead, J.G. and C.W. Potter. 1990. Natural history of bottlenose dolphins along the central Atlantic coast of the United States. In: Leatherwood, S. and R.R. Reeves (Eds.), The Bottlenose Dolphin. Academic Press, San Diego, CA. pp. 165–195.

Michel, J., E.H. Owens, S. Zengel, A. Graham, Z. Nixon, T. Allard, W. Holton, P.D. Reimer, A. Lamarche, M. White, N. Rutherford, C. Childs, G. Mauseth, G. Challenger, and E. Taylor. 2013. Extent and degree of shoreline oiling: Deepwater Horizon oil spill, Gulf of Mexico, USA. PLoS ONE 8(6):e65087. doi: 10.1371/journal.pone.0065087

Miller, C.E. 2003. Abundance trends and environmental habitat usage patterns of bottlenose dolphins (*Tursiops truncatus*) in lower Barataria and Caminada bays, Louisiana. Louisiana State University and Agricultural & Mechanical College. Dissertation. 125 p.

Miller, L.J., A.D. Mackey, T. Hoffland, M. Solangi, and S.A. Kuczaj. 2010. Potential effects of a major hurricane on Atlantic bottlenose dolphin (*Tursiops truncatus*) reproduction in the Mississippi Sound. Marine Mammal Science 26(3):707–715. doi: 10.1111/j.1748-7692.2010.00371.x

Miller, L.J., A.D. Mackey, M. Solangi, and S.A. Kuczaj. 2013. Population abundance and habitat utilization of bottlenose dolphins in the Mississippi Sound. Aquatic Conservation: Marine and Freshwater Ecosystems 23(1):145–151. doi: 10.1002/aqc.2278

Mullin, K.D., T. McDonald, R.S. Wells, B.C. Balmer, T. Speakman, C. Sinclair, E.S. Zolman, F. Hornsby, S.M. McBride, K.A. Wilkinson, and L.H. Schwacke. 2017. Density, abundance, survival, and ranging patterns of common bottlenose dolphins (*Tursiops truncatus*) in Mississippi Sound following the Deepwater Horizon oil spill. PLoS ONE 12(10):e0186265. doi: 10.1371/journal.pone.0186265

National Oceanic and Atmospheric Administration (NOAA). 2011. Public scoping for preparation of a programmatic environmental impact statement for the Deepwater Horizon BP Oil Spill. 9 p.

Perrin, W.F. and S.B. Reilly. 1984. Reproductive parameters of dolphins and small whales of the family Delphinidae. In: Perrin, W.F., R.L. Brownell, Jr., and D.P. DeMaster (Eds.), Reproduction in Whales, Dolphins, and Porpoises. pp. 97–134.

Rosel, P.E., L.A. Wilcox, C. Sinclair, T.R. Speakman, M.C. Tumlin, J.A. Litz, and E.S. Zolman. 2017. Genetic assignment to stock of stranded common bottlenose dolphins in southeastern Louisiana after the Deepwater Horizon oil spill. Endangered Species Research 33:221–234. doi: 10.3354/esr00780

Rossman, S., P.H. Ostrom, M. Stolen, N.B. Barros, H. Gandhi, C.A. Stricker, and R.S. Wells. 2015. Individual specialization in the foraging habits of female bottlenose dolphins living in a trophically diverse and habitat rich estuary. Oecologia 178(2):415–425. doi: 10.1007/s00442-015-3241-6

Schwacke, L.H., C.R. Smith, F.I. Townsend, R.S. Wells, L.B. Hart, B.C. Balmer, T.K. Collier, S. De Guise, M.M. Fry, L.J. Guillette, Jr., S.V. Lamb, S.M. Lane, W.E. McFee, N.J. Place, M.C. Tumlin, G.M. Ylitalo, E.S. Zolman, and T.K. Rowles. 2014. Health of common bottlenose dolphins (*Tursiops truncatus*) in Barataria Bay, Louisiana, following the deepwater horizon oil spill. Environmental Science & Technology 48(1):93–103. doi: 10.1021/es403610f

Schwacke, L.H., L. Thomas, R.S. Wells, W.E. McFee, A.A. Hohn, K.D. Mullin, E.S. Zolman, B.M. Quigley, T.K. Rowles, and J.H. Schwacke. 2017. Quantifying injury to common bottlenose dolphins from the Deepwater Horizon oil spill using an age-, sex- and class-structured population model. Endangered Species Research 33:265–279. doi: 10.3354/esr00777

Scott, M.D. and S.J. Chivers. 1990. Distribution and herd structure of bottlenose dolphins in the eastern tropical Pacific Ocean. In: Leatherwood, S. and R.R. Reeves (Eds.), The Bottlenose Dolphin. Academic Press, San Diego, CA. pp. 387–402.

Sellas, A.B., R.S. Wells, and P.E. Rosel. 2005. Mitochondrial and nuclear DNA analyses reveal fine scale geographic structure in bottlenose dolphins (*Tursiops truncatus*) in the Gulf of Mexico. Conservation Genetics 6(5):715–728. doi: 10.1007/s10592-005-9031-7

Sergeant, D.E., D.K. Caldwell, and M.C. Caldwell. 1973. Age, growth, and maturity of bottlenosed dolphin (*Tursiops-truncatus*) from northeast Florida. Journal of the Fisheries Research Board of Canada 30(7):1009–1011. doi: 10.1139/f73-165

Shane, S.H. 1990. Behavior and ecology of the bottlenose dolphin at Sanibel Island, Florida. In: Leatherwood, S. and R.R. Reeves (Eds.), The Bottlenose Dolphin. Academic Press, San Diego, CA. pp. 245–265. doi: 10.1016/B978-0-12-440280-5.50016-0

Shane, S.H. 2004. Residence patterns, group characteristics, and association patterns of bottlenose dolphins near Sanibel Island, Florida. Gulf of Mexico Science 22(1):1–12. doi: 10.18785/goms.2201.01

Shane, S.H., R.S. Wells, and B. Würsig. 1986. Ecology, behavior and social organization of the bottlenose dolphin: A review. Marine Mammal Science 2:34–63. doi: 10.1111/j.1748-7692.1986.tb00026.x

Smith, C.R., T.K. Rowles, L.B. Hart, F.I. Townsend, R.S. Wells, E.S. Zolman, B.C. Balmer, B. Quigley, M. Ivančić, W. McKercher, M.C. Tumlin, K.D. Mullin, J.D. Adams, Q. Wu, W. McFee, T.K. Collier, and L.H. Schwacke. 2017. Slow recovery of Barataria Bay dolphin health following the Deepwater Horizon oil spill (2013-2014), with evidence of persistent lung disease and impaired stress response. Endangered Species Research 33:127–142. doi: 10.3354/esr00778

Takeshita, R., B.C. Balmer, F. Messina, E.S. Zolman, L. Thomas, R.S. Wells, C.R. Smith, T.K. Rowles, and L.H. Schwacke. 2021. High site-fidelity in common bottlenose dolphins despite low salinity exposure and associated indicators of compromised health. PLoS ONE 16(9):e0258031. doi: 10.1371/journal.pone.0258031

Taylor, B.L., S.J. Chivers, J. Larese, and W.F. Perrin. 2007. Generation length and percent mature estimates for IUCN assessments of cetaceans. National Oceanic and Atmospheric Administration, National Marine Fisheries Service, Southwest Fisheries Science Center: La Jolla, CA. Administrative Report LJ-07-01. 24 p.

Toms, C. 2019. Filling the gaps: Common bottlenose dolphin (*Tursiops truncatus*) population dynamics, structure, and connectivity within Florida panhandle bays, sounds, and estuaries. University of Central Florida. Dissertation. 373 p.

Torres, L.G. and A.J. Read. 2009. Where to catch a fish? The influence of foraging tactics on the ecology of bottlenose dolphins (*Tursiops truncatus*) in Florida Bay, Florida. Marine Mammal Science 25(4):797–815. doi: 10.1111/j.1748-7692.2009.00297.x

Tyson, R.B., S.M. Nowacek, and D.P. Nowacek. 2011. Community structure and abundance of bottlenose dolphins *Tursiops truncatus* in coastal waters of the northeast Gulf of Mexico. Marine Ecology Progress Series 438:253–265. doi: 10.3354/meps09292

Urian, K.W., S. Hofmann, R.S. Wells, and A.J. Read. 2009. Fine-scale population structure of bottlenose dolphins (*Tursiops truncatus*) in Tampa Bay, Florida. Marine Mammal Science 25(3):619–638. doi: 10.1111/j.1748-7692.2009.00284.x

Van Bressem, M.F., P.J. Duignan, A. Banyard, M. Barbieri, K.M. Colegrove, S. De Guise, G. Di Guardo, A. Dobson, M. Domingo, D. Fauquier, A. Fernandez, T. Goldstein, B. Grenfell, K.R. Groch, F. Gulland, B.A. Jensen, P.D. Jepson, A. Hall, T. Kuiken, S. Mazzariol, S.E. Morris, O. Nielsen, J.A. Raga, T.K. Rowles, J. Saliki, E. Sierra, N. Stephens, B. Stone, I. Tomo, J. Wang, T. Waltzek, and J.F. Wellehan. 2014. Cetacean morbillivirus: current knowledge and future directions. Viruses 6(12):5145–5181. doi: 10.3390/v6125145

Venn-Watson, S., L. Garrison, J. Litz, E. Fougeres, B. Mase, G. Rappucci, E. Stratton, R. Carmichael, D. Odell, D. Shannon, S. Shippee, S. Smith, L. Staggs, M. Tumlin, H. Whitehead, and T. Rowles. 2015. Demographic clusters identified within the northern Gulf of Mexico common bottlenose dolphin (*Tursiops truncates*) unusual mortality event: January 2010-June 2013. PLoS ONE 10(2):e0117248. doi: 10.1371/journal.pone.0117248

Vollmer, N.L. and P.E. Rosel. 2013. A review of common bottlenose dolphins (*Tursiops truncatus truncatus*) in the Northern Gulf of Mexico: Population biology, potential threats, and management. Southeastern Naturalist 12(m6):1–43. doi: 10.1656/058.012.m601

Weller, D.W. 1998. Global and regional variation in the biology and behavior of bottlenose dolphins. Texas A&M University. Dissertation. 142 p.

Wells, R.S. 1986a. Population structure of bottlenose dolphins: Behavioral studies along the central west coast of Florida. Contract Report to National Marine Fisheries Service, Southeast Fisheries Center. Contract No. 45-WCNF-5-003BB. 58 p.

Wells, R.S. 1986b. Structural aspects of dolphin societies. University of California, Santa Cruz. Dissertation. 234 p.

Wells, R.S. 1991. The role of long-term study in understanding the social structure of a bottlenose dolphin community. In: Pryor, K. and K. Norris (Eds.), Dolphin Societies: Discoveries and Puzzles. University of California Press, Berkeley, CA. pp. 199–226.

Wells, R.S. 2000. Reproduction in wild bottlenose dolphins: Overview of patterns observed during a long-term study. In: Duffield, D. and T. Robeck (Eds.), Bottlenose Dolphin Reproduction Workshop. AZA Marine Mammal Taxon Advisory Group, Silver Spring, MD. pp. 57–74.

Wells, R.S. 2003. Dolphin social complexity: Lessons from long-term study and life history. In: de Waal, F.B.M. and P.L. Tyack (Eds.), Animal Social Complexity: Intelligence, Culture, and Individualized Societies Harvard University Press, Cambridge, MA. pp. 32–56.

Wells, R.S. 2014. Social structure and life history of bottlenose dolphins near Sarasota Bay, Florida: Insights from four decades and five generations. Primates and cetaceans. pp. 149–172. doi: 10.1007/978-4-431-54523-1_8

Wells, R.S., M.K. Bassos, K.W. Urian, W.J. Carr, and M.D. Scott. 1996. Low-level monitoring of bottlenose dolphins, *Tursiops truncatus*, in Charlotte Harbor, Florida: 1990-1994. National Oceanic and Atmospheric Administration, National Marine Fisheries Service, Southeast Fisheries Science Center: Miami, FL. NOAA Technical Memorandum NMFS-SEFSC-384. 86 p.

Wells, R.S., M.K. Bassos, K.W. Urian, S.H. Shane, E.C.G. Owen, C.F. Weiss, W.J. Carr, and M.D. Scott. 1997. Low-level monitoring of bottlenose dolphins, *Tursiops truncatus*, in Pine Island Sound, Florida: 1996. National Oceanic and Atmospheric Administration, National Marine Fisheries Service, Southeast Fisheries Science Center: Miami, FL. Southeast Fisheries Center Contribution No. 40-WCNF601958. 86 p.

Wells, R.S., A. Natoli, and G. Braulik. 2019. *Tursiops truncatus* (errata version published in 2019). *The IUCN Red List of Threatened Species* 2019: e.T22563A156932432. https://dx.doi.org/10.2305/IUCN.UK.2019-1.RLTS.T22563A156932432.en

Wells, R.S., L.H. Schwacke, T.K. Rowles, B.C. Balmer, E. Zolman, T. Speakman, F.I. Townsend, M.C. Tumlin, A. Barleycorn, and K.A. Wilkinson. 2017. Ranging patterns of common bottlenose dolphins *Tursiops truncatus* in Barataria Bay, Louisiana, following the Deepwater Horizon oil spill. Endangered Species Research 33:159–180. doi: 10.3354/esr00732

Wells, R.S. and M.D. Scott. 1999. Bottlenose dolphin *Tursiops truncatus* (Montagu, 1821). In: Ridgway, S.H. and R. Harrison (Eds.), Handbook of marine mammals. Volume 6: The second book of dolphins and the porpoises. Academic Press, San Diego, CA. pp. 137–182.

Wells, R.S. and M.D. Scott. 2018. Bottlenose dolphin, *Tursiops truncatus*, common bottlenose dolphin. In: Würsig, B., J.G.M. Thewissen, and K.M. Kovacs (Eds.), Encyclopedia of Marine Mammals (Third Edition). Academic Press, London, UK. pp. 118–125. doi: 10.1016/B978-0-12-804327-1.00072-8

Wells, R.S., M.D. Scott, and A.B. Irvine. 1987. The social structure of free-ranging bottlenose dolphins. In: Genoways, H.H. (Ed.), Current Mammalogy. Plenum Press, New York, NY. pp. 247–305.

Wells, R.S., K.W. Urian, A.J. Read, M.K. Bassos, W.J. Carr, and M.D. Scott. 1996. Low-level monitoring of bottlenose dolphins, *Tursiops truncatus*, in Tampa Bay, Florida: 1988-1993. National Oceanic and Atmospheric Administration, National Marine Fisheries Service, Southeast Fisheries Science Center: Miami, FL. NOAA Technical Memorandum NMFS-SEFSC-385. 36 p.

Wilson, R.M., R.B. Tyson, J.A. Nelson, B.C. Balmer, J.P. Chanton, and D.P. Nowacek. 2017. Niche differentiation and prey selectivity among common bottlenose dolphins (*Tursiops truncatus*) sighted in St. George Sound, Gulf of Mexico. Frontiers in Marine Science 4:235. doi: 10.3389/fmars.2017.00235

Common bottlenose dolphin − *Tursiops truncatus*

GoMx BSE/Waccasassa Bay, Withlacoochee Bay, Crystal Bay Stock

Overall Vulnerability Rank = Very High

Biological Sensitivity = Very High

Climate Exposure = Very High

Data Quality = 78% of scores ≥ 2

	Tursiops truncatus	Expert Scores	Data Quality	Expert Scores Plots (Portion by Category)
Sensitivity attributes	Prey/Diet Specificity	2.1	2.0	
	Habitat Specificity	3.6	2.0	
	Site Fidelity	3.7	1.7	
	Lifetime Reproductive Potential	1.3	2.3	
	Generation Length	2.9	2.3	
	Reproductive Plasticity	1.5	2.3	
	Migration	3.7	1.7	
	Home Range	3.4	1.7	
	Stock Abundance	3.1	1.7	
	Stock Abundance Trend	2.3	1.3	
	Cumulative Stressors	3.8	2.0	
	Sensitivity Score	Very High		
Exposure variables	Sea Surface Temperature (Change in mean)	4.0	2.3	
	Sea Surface Temperature (Change in variability)	1.0	2.3	
	Air Temperature (Change in mean)	4.0	2.3	
	Air Temperature (Change in variability)	1.0	2.3	
	Precipitation (Change in mean)	1.0	2.3	
	Precipitation (Change in variability)	1.0	2.3	
	Sea Surface Salinity (Change in mean)	1.5	2.3	
	Sea Surface Salinity (Change in variability)	1.8	2.3	
	Ocean pH (Change in mean)	4.0	2.3	
	Ocean pH (Change in variability)	1.0	2.3	
	Sea ice coverage (Change in mean)	1.0	2.3	
	Sea ice coverage (Change in variability)	1.0	2.3	
	Dissolved oxygen (Change in mean)	4.0	2.3	
	Dissolved oxygen (Change in variability)	1.0	2.3	
	Circulation	3.7	1.7	
	Sea level rise	4.0	2.3	
	Exposure Score	Very High		
Overall Vulnerability Rank		Very High		

Low
Moderate
High
Very High

Common bottlenose dolphin (GoMx BSE/Waccasassa Bay, Withlacoochee Bay, Crystal Bay Stock)

Tursiops truncatus

CVA Results Summary

Overall Climate Vulnerability Rank: Very High (100% certainty from bootstrap analysis).

Climate Exposure: Very High. Six exposure factors scored greater than 3.5: Air Temperature (Standard anomaly) (4.00), Dissolved oxygen (Standard anomaly) (4.00), Ocean pH (Standard anomaly) (4.00), Sea level rise (4.00), Sea Surface Temperature (Standard anomaly) (4.00), and Circulation (3.73).

Biological Sensitivity: Very High. Four sensitivity attributes scored greater than 3.5: Cumulative Stressors (3.80), Site Fidelity (3.73), Migration (3.67), and Habitat Specificity (3.60).

Distributional Response: High

Abundance Response: Very High

Phenology Response: High

Data Quality: 78% of the data quality scores were 2 or greater, 55% of sensitivity attribute data quality scores were 2 or higher.

Background Information

Stock Description

This stock includes common bottlenose dolphins in Waccasassa Bay, Withlacoochee Bay, and Crystal Bay in the northeast Gulf of Mexico along the Florida coast (Hayes et al. 2022).

Prey/Diet Specificity

Generally, common bottlenose dolphins consume a large variety of fish and/or squid (Barros and Odell 1990; Mead and Potter 1990; Wells and Scott 2018). Common bottlenose dolphins seem to show a consistent preference for sound-producing fishes (e.g., sciaenids, toadfish; Berens McCabe et al. 2010; Bowen-Stevens et al. 2021). In Sarasota Bay, Florida, Rossman et al. (2015) found some individual common bottlenose dolphins to show diet specialization despite the broader study group showing a generalist diet. Specialization can be influenced by locally abundant prey species (Shane et al. 1986; Vollmer and Rosel 2013) or can result from resource partitioning amongst sympatric populations (e.g., Torres and Read 2009; Wilson et al. 2017).

Habitat Specificity

Common bottlenose dolphins are found in a variety of marine and estuarine habitats (Wells and Scott 2018).

Site Fidelity

No specific site fidelity information was found for this stock. However, common bottlenose dolphins in other Gulf of Mexico bay, sound, and estuary (BSE) stocks show site fidelity (LaBrecque et al. 2015; Hayes et al. 2022), such as in Charlotte Harbor and Pine Island Sound (Bassos-Hull et al. 2013), Sarasota Bay (Wells 1986a; Wells 1991; Wells 2014), St. Andrew Bay (Balmer et al. 2019), St. Joseph Bay (Balmer et al. 2008; B. Balmer et al. 2018), St. Vincent Sound and Apalachicola Bay (Tyson et al. 2011), and Tampa Bay in Florida (Wells et al. 1996b; Urian et al. 2009); Matagorda Bay, Espiritu Santo Bay, and San Luis Pass in Texas (Maze and Würsig 1999; Lynn and Würsig 2002; Irwin and Würsig 2004); Barataria Bay in Louisiana (Miller 2003; McDonald et al. 2017; Wells et al. 2017; Takeshita et al. 2021); and Mississippi Sound in Mississippi (Hubard et al. 2004; Mackey 2010; Mullin et al. 2017).

Lifetime Reproductive Potential

Taylor et al. (2007) reported the interbirth interval of common bottlenose dolphins as 3.80 years, though other studies have shown it to be closer to three years (Leatherwood 1977; Wells and Scott 1999; Connor et al. 2000; Wells 2003).

Generally, females reach sexual maturity before males, with females reaching sexual maturity at 5–13 years (Sergeant et al. 1973; Perrin and Reilly 1984; Wells et al. 1987; Mead and Potter 1990; Mann et al. 2000; Wells 2000; Wells and Scott 2018).

Females reproduce through most of their adult lives (Leatherwood 1977; Hohn et al. 1989; Wells and Scott 1999; Wells 2000; Wells 2003), but may produce fewer calves after they reach 30 years of age (Wells 2000). Sarasota Bay common bottlenose dolphins can give birth when they are as young as 6 years of age, and can continue until they are 48 years old (Wells and Scott 2018). Reproductive lifespan would therefore be up to 42 years based on long-term observation studies (Wells 2014; Wells and Scott 2018).

Longitudinal studies from Sarasota Bay, Florida have found that female common bottlenose dolphins can live up to 67 years (Wells and Scott 2018). Studies based on growth layer groups in teeth suggest female common bottlenose dolphins can live to 41 years along the Texas coast (Fernandez and Hohn 1998) and to 30 years along the Mississippi coast (Mattson et al. 2006).

Generation Length

Taylor et al. (2007) reported a common bottlenose dolphin generation length of 20.6–21.1 years.

Reproductive Plasticity

Common bottlenose dolphin calving is generally diffusely seasonal, with one or more peaks, although births have been reported from all seasons (Vollmer and Rosel 2013; Wells and Scott 2018). Calf sightings peak in the spring and summer months in Florida (Wells et al. 1987), Mississippi/Louisiana (Miller et al. 2010; Miller et al. 2013), and Texas (McHugh 1989; Henderson et al. 2007).

Migration

The bay, sound, and estuary stocks in the Gulf of Mexico are thought to primarily comprise stable resident communities. However, transient or seasonal movements of some individuals into and out of a BSE stock area are recorded in some areas (LaBrecque et al. 2015; Hayes et al. 2022). Molecular genetic data support that some BSE stocks are genetically distinct from one another, and furthermore are differentiated from adjacent coastal populations (e.g., Sellas et al. 2005; Rosel et al. 2017). For example, Toms (2019) found fine-scale genetic population structure among five inshore systems in the Florida Panhandle and variable migration rates among populations.

Year-round residency has been reported for common bottlenose dolphins (Hayes et al. 2022), such as in Florida (Irvine and Wells 1972; Irvine et al. 1981; Wells 1986a; Wells 1986b; Wells et al. 1987; Scott et al. 1990; Wells 1991; Wells et al. 1996a; Wells et al. 1996b; Wells et al. 1997; Wells 2003; Balmer et al. 2008; Urian et al. 2009; Tyson et al. 2011; Bassos-Hull et al. 2013; B. Balmer et al. 2018; Balmer et al. 2019; Toms 2019), Texas (Shane 1990; Bräger 1993; Bräger et al. 1994; Fertl 1994; Weller 1998; Maze and Würsig 1999; Lynn and Würsig 2002; Irwin and Würsig 2004; Shane 2004), and Louisiana/Mississippi (Hubard et al. 2004; McDonald et al. 2017; Mullin et al. 2017; Takeshita et al. 2021).

Home Range

The home ranges of individuals within the Gulf of Mexico BSE stocks are considered to generally include the areas within the stock boundaries and brief excursions beyond the stock boundaries (Irvine et al. 1981; Wells 1986a; Wells 1986b; Wells 1991; Wells et al. 1996a; Wells et al. 1996b; Balmer et al. 2008; Wells 2014; Balmer et al. 2019), although some individuals show preference for small areas within the stock boundaries (Lynn and Würsig 2002; Urian et al. 2009; Mullin et al. 2017; Wells et al. 2017).

Stock Abundance

An abundance estimate for this stock is unavailable (Hayes et al. 2022).

Stock Abundance Trend

Data are insufficient to complete an abundance trend analysis for this stock (Hayes et al. 2022).

Cumulative Stressors

Common bottlenose dolphins face a wide range of individual and cumulative natural and anthropogenic threats (see reviews by Vollmer and Rosel 2013; Wells and Scott 2018; and Wells et al. 2019). These include such threats as shark predation, harmful algal blooms, recreational and commercial fishing gear, vessel traffic, pollution, habitat alteration, and provisioning.

The nearshore and estuarine habitats along the Gulf of Mexico coast include waters adjacent to areas of high human population, some of which are highly industrialized, and areas of heavy agricultural impact. Studies of common bottlenose dolphin tissues from Florida Gulf Coast waters have shown persistent organic pollutants (POPs) in blubber and blood samples (Yordy et al. 2010a, 2010b, 2010c; Kucklick et al. 2011; J. Balmer et al. 2018). Similarly, mercury has been detected in common bottlenose dolphin tissues in Florida Gulf Coast waters (Bryan et al. 2007; Woshner et al. 2008; Miller et al. 2011; Correa et al. 2013; McCormack et al. 2020a, 2020b, 2022).

An Unusual Mortality Event (UME) was declared in 1990 for the Gulf of Mexico from Florida to Texas, with more than 300 common bottlenose dolphins stranding due to an undetermined cause, although morbillivirus and cold temperatures may have contributed (Litz et al. 2014). This stock was also within the range of the 2005–2006 UME in central west Florida caused by brevetoxin (Litz et al. 2014).

Further Reading

Hayes, S.A., E. Josephson, K. Maze-Foley, P.E. Rosel, and J. Wallace. 2022. U.S. Atlantic and Gulf of Mexico marine mammal stock assessments 2021. National Oceanic and Atmospheric Administration, National Marine Fisheries Service, Northeast Fisheries Science Center: Woods Hole, MA. NOAA Technical Memorandum NMFS-NE-288. 380 p. doi: 10.25923/6tt7-kc16

Wells, R.S. and M.D. Scott. 2018. Bottlenose Dolphin, *Tursiops truncatus*, Common Bottlenose Dolphin. In: Würsig, B., J.G.M. Thewissen, and K.M. Kovacs (Eds.), Encyclopedia of Marine Mammals (Third Edition). Academic Press, London, UK. pp. 118–125. doi: 10.1016/B978-0-12-804327-1.00072-8

Wells, R.S., A. Natoli, and G. Braulik. 2019. *Tursiops truncatus* (errata version published in 2019). The IUCN Red List of Threatened Species 2019: e.T22563A156932432. https://dx.doi.org/10.2305/IUCN.UK.2019-1.RLTS.T22563A156932432.en

References

Balmer, B., T. McDonald, F. Hornsby, J. Adams, J. Allen, A. Barleycorn, and L. Schwacke. 2018. Long-term trends in a northern Gulf of Mexico common bottlenose dolphin (*Tursiops truncatus*) population in the wake of the Deepwater Horizon oil spill. Journal of Cetacean Research and Management 18:1–9. doi: 10.47536/jcrm.v18i1.409

Balmer, B., S. Watwood, B. Quigley, T. Speakman, K. Barry, K. Mullin, P. Rosel, C. Sinclair, E. Zolman, and L. Schwacke. 2019. Common bottlenose dolphin (*Tursiops truncatus*) abundance and distribution patterns in St. Andrew Bay, Florida, USA. Aquatic Conservation: Marine and Freshwater Ecosystems 29(3):486–498. doi: 10.1002/aqc.3001

Balmer, B.C., R.S. Wells, S.M. Nowacek, D.P. Nowacek, L.H. Schwacke, W.A. McLellan, F.S. Scharf, T.K. Rowles, L.J. Hansen, T.R. Spradlin, and D.A. Pabst. 2008. Seasonal abundance and distribution patterns of common bottlenose dolphins (*Tursiops truncatus*) near St. Joseph Bay, Florida, USA. Journal of Cetacean Research and Management 10(2):157–167.

Balmer, J.E., G.M. Ylitalo, T.K. Rowles, K.D. Mullin, R.S. Wells, F.I. Townsend, R.W. Pearce, J.L. Bolton, E.S. Zolman, B.C. Balmer, and L.H. Schwacke. 2018. Persistent organic pollutants (POPs) in blood and blubber of common bottlenose dolphins (*Tursiops truncatus*) at three northern Gulf of Mexico sites following the Deepwater Horizon oil spill. Science of the Total Environment 621:130–137. doi: 10.1016/j.scitotenv.2017.11.209

Barros, N.B. and D.K. Odell. 1990. Food habits of bottlenose dolphins in the southeastern United States In: Leatherwood, S. and R.R. Reeves (Eds.), The Bottlenose Dolphin. Academic Press, San Diego, CA. pp. 309–328. doi: 10.1016/B978-0-12-440280-5.50020-2

Bassos-Hull, K., R.M. Perrtree, C.C. Shepard, S. Schilling, A.A. Barleycorn, J.B. Allen, B.C. Balmer, W.E. Pine, and R.S. Wells. 2013. Long-term site fidelity and seasonal abundance estimates of common bottlenose dolphins (*Tursiops truncatus*) along the southwest coast of Florida and responses to natural perturbations. Journal of Cetacean Research and Management 13(1):19–30. doi: 10.47536/jcrm.v13i1.551

Berens McCabe, E.J., D.P. Gannon, N.B. Barros, and R.S. Wells. 2010. Prey selection by resident common bottlenose dolphins (*Tursiops truncatus*) in Sarasota Bay, Florida. Marine Biology 157(5):931–942. doi: 10.1007/s00227-009-1371-2

Bowen-Stevens, S.R., D.P. Gannon, R.A. Hazelkorn, G. Lovewell, K.M. Volker, S. Smith, M.C. Tumlin, and J. Litz. 2021. Diet of common bottlenose dolphins, *Tursiops truncatus*, that stranded in and near Barataria Bay, Louisiana, 2010–2012. Southeastern Naturalist 20(1). doi: 10.1656/058.020.0113

Bräger, S. 1993. Diurnal and seasonal behavior patterns of bottlenose dolphins (*Tursiops truncatus*). Marine Mammal Science 9:434–438. doi: 10.1111/j.1748-7692.1993.tb00477.x

Bräger, S., B. Würsig, A. Acevedo, and T. Henningsen. 1994. Association patterns of bottle-nosed dolphins (*Tursiops-truncatus*) in Galveston Bay, Texas. Journal of Mammalogy 75(2):431–437. doi: 10.2307/1382564

Bryan, C.E., S.J. Christopher, B.C. Balmer, and R.S. Wells. 2007. Establishing baseline levels of trace elements in blood and skin of bottlenose dolphins in Sarasota Bay, Florida: implications for non-invasive monitoring. Science of the Total Environment 388(1-3):325–342. doi: 10.1016/j.scitotenv.2007.07.046

Connor, R.C., R.S. Wells, J. Mann, and A.J. Read. 2000. The bottlenose dolphin: Social relationships in a fission-fusion society. In: Mann, J., R. Connor, P. Tyack, and H. Whitehead (Eds.), Cetacean Societies: Field Studies of Dolphins and Whales. University of Chicago Press, Chicago, IL. pp. 91–126.

Correa, L., J.M. Castellini, R.S. Wells, and T. O'Hara. 2013. Distribution of mercury and selenium in blood compartments of bottlenose dolphins (*Tursiops truncatus*) from Sarasota Bay, Florida. Environmental Toxicology and Chemistry 32(11):2441–2448. doi: 10.1002/etc.2327

Fernandez, S. and A.A. Hohn. 1998. Age, growth, and calving season of bottlenose dolphins, *Tursiops truncatus*, off coastal Texas. Fishery Bulletin 96(2):357–365.

Fertl, D. 1994. Occurrence patterns and behavior of bottlenose dolphins (*Tursiops truncatus*) in the Galveston ship channel, Texas. Texas Journal of Science 46(4):299–318.

Hayes, S.A., E. Josephson, K. Maze-Foley, P.E. Rosel, and J. Wallace. 2022. U.S. Atlantic and Gulf of Mexico marine mammal stock assessments 2021. National Oceanic and Atmospheric Administration, National Marine Fisheries Service, Northeast Fisheries Science Center: Woods Hole, MA. NOAA Technical Memorandum NMFS-NE-288. 380 p. doi: 10.25923/6tt7-kc16

Henderson, E.E. and B. Würsig. 2007. Behavior patterns of bottlenose dolphins in San Luis Pass, Texas. Gulf of Mexico Science 25(2):153–161. doi: 10.18785/goms.2502.06

Hohn, A.A., M.D. Scott, R.S. Wells, J.C. Sweeney, and A.B. Irvine. 1989. Growth layers in teeth from known-age, free-ranging bottlenose dolphins. Marine Mammal Science 5(4):315–342. doi: 10.1111/j.1748-7692.1989.tb00346.x

Hubard, C.W., K. Maze-Foley, K.D. Mullin, and W.W. Schroeder. 2004. Seasonal abundance and site fidelity of bottlenose dolphins (*Tursiops truncatus*) in Mississippi Sound. Aquatic Mammals 30(2):299–310. doi: 10.1578/am.30.2.2004.299

Irvine, A.B., M.D. Scott, R.S. Wells, and J.H. Kaufmann. 1981. Movements and activities of the Atlantic bottlenose dolphin, *Tursiops truncatus*, near Sarasota, Florida. Fishery Bulletin 79(4):671–688.

Irvine, B. and R.S. Wells. 1972. Results of attempts to tag Atlantic bottlenosed dolphins (*Tursiops truncatus*). Cetology 13:1–5.

Irwin, L.-J. and B. Würsig. 2004. A small resident community of bottlenose dolphins, *Tursiops truncatus*, in Texas: Monitoring recommendations. Gulf of Mexico Science 22(1):13–21. doi: 10.18785/goms.2201.02

Kucklick, J., L. Schwacke, R. Wells, A. Hohn, A. Guichard, J. Yordy, L. Hansen, E. Zolman, R. Wilson, J. Litz, D. Nowacek, T. Rowles, R. Pugh, B. Balmer, C. Sinclair, and P. Rosel. 2011. Bottlenose dolphins as indicators of persistent organic pollutants in the western North Atlantic Ocean and Northern

Gulf of Mexico. Environmental Science & Technology 45(10):4270–4277. doi: 10.1021/es1042244

LaBrecque, E., C. Curtice, J. Harrison, S.M. Van Parijs, and P.N. Halpin. 2015. 3. Biologically Important Areas for cetaceans within U.S. Waters – Gulf of Mexico Region. Aquatic Mammals 41(1):30–38. doi: 10.1578/am.41.1.2015.30

Leatherwood, S. 1977. Some preliminary impressions on the numbers and social behavior of free-swimming bottlenosed dolphin calves (*Tursiops truncatus*) in the northern Gulf of Mexico. In: Ridgway, S.H. and K. Bernirschke (Eds.), Breeding Dolphins; Present Status, Suggestions for the Future. Final report to the US Marine Mammal Commission, Contract MM6AC009, MMC-76/07, Washington, DC. pp. 143–167.

Litz, J.A., M.A. Baran, S.R. Bowen-Stevens, R.H. Carmichael, K.M. Colegrove, L.P. Garrison, S.E. Fire, E.M. Fougeres, R. Hardy, S. Holmes, W. Jones, B.E. Mase-Guthrie, D.K. Odell, P.E. Rosel, J.T. Saliki, D.K. Shannon, S.F. Shippee, S.M. Smith, E.M. Stratton, M.C. Tumlin, H.R. Whitehead, G.A. Worthy, and T.K. Rowles. 2014. Review of historical unusual mortality events (UMEs) in the Gulf of Mexico (1990–2009): providing context for the multi-year northern Gulf of Mexico cetacean UME declared in 2010. Diseases of Aquatic Organisms 112(2):161–175. doi: 10.3354/dao02807

Lynn, S.K. and B. Würsig. 2002. Summer movement patterns of bottlenose dolphins in a Texas bay. Gulf of Mexico Science 20(1):25–37. doi: 10.18785/goms.2001.03

Mackey, A.D. 2010. Site fidelity and association patterns of bottlenose dolphins (*Tursiops truncatus*) in the Mississippi Sound. University of Southern Mississippi. Thesis. 133 p.

Mann, J., R.C. Connor, L.M. Barre, and M.R. Heithaus. 2000. Female reproductive success in bottlenose dolphins (*Tursiops* sp.): life history, habitat, provisioning, and group-size effects. Behavioral Ecology 11(2):210–219. doi: 10.1093/beheco/11.2.210

Mattson, M.C., K.D. Mullin, G.W. Ingram, and W. Hoggard. 2006. Age structure and growth of the bottlenose dolphin (*Tursiops truncatus*) from strandings in the Mississippi sound region of the north-central Gulf of Mexico from 1986 to 2003. Marine Mammal Science 22(3):654–666. doi: 10.1111/j.1748-7692.2006.00057.x

Maze, K.S. and B. Würsig. 1999. Bottlenosed dolphins of San Luis Pass, Texas: Occurrence patterns, site-fidelity, and habitat use. Aquatic Mammals 25:91–104.

McCormack, M.A., F. Battaglia, W.E. McFee, and J. Dutton. 2020a. Mercury concentrations in blubber and skin from stranded bottlenose dolphins (*Tursiops truncatus*) along the Florida and Louisiana coasts (Gulf of Mexico, USA) in relation to biological variables. Environmental Research 180:108886. doi: 10.1016/j.envres.2019.108886

McCormack, M.A., B.P. Jackson, and J. Dutton. 2020b. Relationship between mercury and selenium concentrations in tissues from stranded odontocetes in the northern Gulf of Mexico. Science of the Total Environment 749:141350. doi: 10.1016/j.scitotenv.2020.141350

McCormack, M.A., W.H. Nowlin, and J. Dutton. 2022. Effect of trophic position on mercury concentrations in bottlenose dolphins (*Tursiops truncatus*) from the northern Gulf of Mexico. Environmental Research 204(Pt B):112124. doi: 10.1016/j.envres.2021.112124

McDonald, T.L., F.E. Hornsby, T.R. Speakman, E.S. Zolman, K.D. Mullin, C. Sinclair, P.E. Rosel, L. Thomas, and L.H. Schwacke. 2017. Survival, density, and abundance of common bottlenose dolphins in Barataria Bay (USA) following the Deepwater Horizon oil spill. Endangered Species Research 33:193–209. doi: 10.3354/esr00806

McHugh, M.B. 1989. Population numbers and feeding behavior of the Atlantic bottlenose dolphin (*Tursiops truncatus*) near Aransas Pass, Texas. University of Texas at Austin. Thesis. 96 p.

Mead, J.G. and C.W. Potter. 1990. Natural history of bottlenose dolphins along the central Atlantic coast of the United States. In: Leatherwood, S. and R.R. Reeves (Eds.), The Bottlenose Dolphin. Academic Press, San Diego, CA. pp. 165–195.

Miller, C.E. 2003. Abundance trends and environmental habitat usage patterns of bottlenose dolphins (*Tursiops truncatus*) in lower Barataria and Caminada bays, Louisiana. Louisiana State University and Agricultural & Mechanical College. Dissertation. 125 p.

Miller, D.L., V. Woshner, E.L. Styer, S. Ferguson, K.K. Knott, M.J. Gray, R.S. Wells, and T.M. O'Hara. 2011. Histologic findings in free-ranging Sarasota Bay bottlenose dolphin (*Tursiops truncatus*) skin: mercury, selenium, and seasonal factors. Journal of Wildlife Diseases 47(4):1012–1018. doi: 10.7589/0090-3558-47.4.1012

Miller, L.J., A.D. Mackey, T. Hoffland, M. Solangi, and S.A. Kuczaj. 2010. Potential effects of a major hurricane on Atlantic bottlenose dolphin (*Tursiops truncatus*) reproduction in the Mississippi Sound. Marine Mammal Science 26(3):707–715. doi: 10.1111/j.1748-7692.2010.00371.x

Miller, L.J., A.D. Mackey, M. Solangi, and S.A. Kuczaj. 2013. Population abundance and habitat utilization of bottlenose dolphins in the Mississippi Sound. Aquatic Conservation: Marine and Freshwater Ecosystems 23(1):145–151. doi: 10.1002/aqc.2278

Mullin, K.D., T. McDonald, R.S. Wells, B.C. Balmer, T. Speakman, C. Sinclair, E.S. Zolman, F. Hornsby, S.M. McBride, K.A. Wilkinson, and L.H. Schwacke. 2017. Density, abundance, survival, and ranging patterns of common bottlenose dolphins (*Tursiops truncatus*) in Mississippi Sound following the Deepwater Horizon oil spill. PLoS ONE 12(10):e0186265. doi: 10.1371/journal.pone.0186265

Perrin, W.F. and S.B. Reilly. 1984. Reproductive parameters of dolphins and small whales of the family Delphinidae. In: Perrin, W.F., R.L. Brownell, Jr., and D.P. DeMaster (Eds.), Reproduction in Whales, Dolphins, and Porpoises. pp. 97–134.

Rosel, P.E., L.A. Wilcox, C. Sinclair, T.R. Speakman, M.C. Tumlin, J.A. Litz, and E.S. Zolman. 2017. Genetic assignment to stock of stranded common bottlenose dolphins in southeastern Louisiana after the Deepwater Horizon oil spill. Endangered Species Research 33:221–234. doi: 10.3354/esr00780

Rossman, S., P.H. Ostrom, M. Stolen, N.B. Barros, H. Gandhi, C.A. Stricker, and R.S. Wells. 2015. Individual specialization in the foraging habits of female bottlenose dolphins living in a trophically diverse and habitat rich estuary. Oecologia 178(2):415–425. doi: 10.1007/s00442-015-3241-6

Scott, M.D. and S.J. Chivers. 1990. Distribution and herd structure of bottlenose dolphins in the eastern tropical Pacific Ocean. In: Leatherwood, S. and R.R. Reeves (Eds.), The Bottlenose Dolphin. Academic Press, San Diego, CA. pp. 387–402.

Sellas, A.B., R.S. Wells, and P.E. Rosel. 2005. Mitochondrial and nuclear DNA analyses reveal fine scale geographic structure in bottlenose dolphins (*Tursiops truncatus*) in the Gulf of Mexico. Conservation Genetics 6(5):715–728. doi: 10.1007/s10592-005-9031-7

Sergeant, D.E., D.K. Caldwell, and M.C. Caldwell. 1973. Age, growth, and maturity of bottlenosed dolphin (*Tursiops-truncatus*) from northeast Florida. Journal of the Fisheries Research Board of Canada 30(7):1009–1011. doi: 10.1139/f73-165

Shane, S.H. 1990. Behavior and ecology of the bottlenose dolphin at Sanibel Island, Florida. In: Leatherwood, S. and R.R. Reeves (Eds.), The Bottlenose Dolphin. Academic Press, San Diego, CA. pp. 245–265. doi: 10.1016/B978-0-12-440280-5.50016-0

Shane, S.H. 2004. Residence patterns, group characteristics, and association patterns of bottlenose dolphins near Sanibel Island, Florida. Gulf of Mexico Science 22(1):1–12. doi: 10.18785/goms.2201.01

Shane, S.H., R.S. Wells, and B. Würsig. 1986. Ecology, behavior and social organization of the bottlenose dolphin: A review. Marine Mammal Science 2:34–63. doi: 10.1111/j.1748-7692.1986.tb00026.x

Takeshita, R., B.C. Balmer, F. Messina, E.S. Zolman, L. Thomas, R.S. Wells, C.R. Smith, T.K. Rowles, and L.H. Schwacke. 2021. High site-fidelity in common bottlenose dolphins despite low salinity

exposure and associated indicators of compromised health. PLoS ONE 16(9):e0258031. doi: 10.1371/journal.pone.0258031
Taylor, B.L., S.J. Chivers, J. Larese, and W.F. Perrin. 2007. Generation length and percent mature estimates for IUCN assessments of cetaceans. National Oceanic and Atmospheric Administration, National Marine Fisheries Service, Southwest Fisheries Science Center: La Jolla, CA. Administrative Report LJ-07-01. 24 p.
Toms, C. 2019. Filling the gaps: Common bottlenose dolphin (*Tursiops truncatus*) population dynamics, structure, and connectivity within Florida panhandle bays, sounds, and estuaries. University of Central Florida. Dissertation. 373 p.
Torres, L.G. and A.J. Read. 2009. Where to catch a fish? The influence of foraging tactics on the ecology of bottlenose dolphins (*Tursiops truncatus*) in Florida Bay, Florida. Marine Mammal Science 25(4):797–815. doi: 10.1111/j.1748-7692.2009.00297.x
Tyson, R.B., S.M. Nowacek, and D.P. Nowacek. 2011. Community structure and abundance of bottlenose dolphins *Tursiops truncatus* in coastal waters of the northeast Gulf of Mexico. Marine Ecology Progress Series 438:253–265. doi: 10.3354/meps09292
Urian, K.W., S. Hofmann, R.S. Wells, and A.J. Read. 2009. Fine-scale population structure of bottlenose dolphins (*Tursiops truncatus*) in Tampa Bay, Florida. Marine Mammal Science 25(3):619–638. doi: 10.1111/j.1748-7692.2009.00284.x
Vollmer, N.L. and P.E. Rosel. 2013. A review of common bottlenose dolphins (*Tursiops truncatus truncatus*) in the Northern Gulf of Mexico: Population biology, potential threats, and management. Southeastern Naturalist 12(m6):1–43. doi: 10.1656/058.012.m601
Weller, D.W. 1998. Global and regional variation in the biology and behavior of bottlenose dolphins. Texas A&M University. Dissertation. 142 p.
Wells, R.S. 1986a. Population structure of bottlenose dolphins: Behavioral studies along the central west coast of Florida. Contract Report to National Marine Fisheries Service, Southeast Fisheries Center. Contract No. 45-WCNF-5-003BB. 58 p.
Wells, R.S. 1986b. Structural aspects of dolphin societies. University of California, Santa Cruz. Dissertation. 234 p.
Wells, R.S. 1991. The role of long-term study in understanding the social structure of a bottlenose dolphin community. In: Pryor, K. and K. Norris (Eds.), Dolphin Societies: Discoveries and Puzzles. University of California Press, Berkeley, CA. pp. 199–226.
Wells, R.S. 2000. Reproduction in wild bottlenose dolphins: Overview of patterns observed during a long-term study. In: Duffield, D. and T. Robeck (Eds.), Bottlenose Dolphin Reproduction Workshop. AZA Marine Mammal Taxon Advisory Group, Silver Spring, MD. pp. 57–74.
Wells, R.S. 2003. Dolphin social complexity: Lessons from long-term study and life history. In: de Waal, F.B.M. and P.L. Tyack (Eds.), Animal Social Complexity: Intelligence, Culture, and Individualized Societies Harvard University Press, Cambridge, MA. pp. 32–56.
Wells, R.S. 2014. Social structure and life history of bottlenose dolphins near Sarasota Bay, Florida: Insights from four decades and five generations. Primates and cetaceans. pp. 149–172. doi: 10.1007/978-4-431-54523-1_8
Wells, R.S., M.K. Bassos, K.W. Urian, W.J. Carr, and M.D. Scott. 1996. Low-level monitoring of bottlenose dolphins, *Tursiops truncatus*, in Charlotte Harbor, Florida: 1990-1994. National Oceanic and Atmospheric Administration, National Marine Fisheries Service, Southeast Fisheries Science Center: Miami, FL. NOAA Technical Memorandum NMFS-SEFSC-384. 86 p.
Wells, R.S., M.K. Bassos, K.W. Urian, S.H. Shane, E.C.G. Owen, C.F. Weiss, W.J. Carr, and M.D. Scott. 1997. Low-level monitoring of bottlenose dolphins, *Tursiops truncatus*, in Pine Island Sound, Florida: 1996. National Oceanic and Atmospheric Administration, National Marine Fisheries

Service, Southeast Fisheries Science Center: Miami, FL. Southeast Fisheries Center Contribution No. 40-WCNF601958. 86 p.

Wells, R.S., A. Natoli, and G. Braulik. 2019. *Tursiops truncatus* (errata version published in 2019). *The IUCN Red List of Threatened Species* 2019: e.T22563A156932432. https://dx.doi.org/10.2305/IUCN.UK.2019-1.RLTS.T22563A156932432.en

Wells, R.S., L.H. Schwacke, T.K. Rowles, B.C. Balmer, E. Zolman, T. Speakman, F.I. Townsend, M.C. Tumlin, A. Barleycorn, and K.A. Wilkinson. 2017. Ranging patterns of common bottlenose dolphins *Tursiops truncatus* in Barataria Bay, Louisiana, following the Deepwater Horizon oil spill. Endangered Species Research 33:159–180. doi: 10.3354/esr00732

Wells, R.S. and M.D. Scott. 1999. Bottlenose dolphin *Tursiops truncatus* (Montagu, 1821). In: Ridgway, S.H. and R. Harrison (Eds.), Handbook of marine mammals. Volume 6: The second book of dolphins and the porpoises. Academic Press, San Diego, CA. pp. 137–182.

Wells, R.S. and M.D. Scott. 2018. Bottlenose dolphin, *Tursiops truncatus*, common bottlenose dolphin. In: Würsig, B., J.G.M. Thewissen, and K.M. Kovacs (Eds.), Encyclopedia of Marine Mammals (Third Edition). Academic Press, London, UK. pp. 118–125. doi: 10.1016/B978-0-12-804327-1.00072-8

Wells, R.S., M.D. Scott, and A.B. Irvine. 1987. The social structure of free-ranging bottlenose dolphins. In: Genoways, H.H. (Ed.), Current Mammalogy. Plenum Press, New York, NY. pp. 247–305.

Wells, R.S., K.W. Urian, A.J. Read, M.K. Bassos, W.J. Carr, and M.D. Scott. 1996. Low-level monitoring of bottlenose dolphins, *Tursiops truncatus*, in Tampa Bay, Florida: 1988-1993. National Oceanic and Atmospheric Administration, National Marine Fisheries Service, Southeast Fisheries Science Center: Miami, FL. NOAA Technical Memorandum NMFS-SEFSC-385. 36 p.

Wilson, R.M., R.B. Tyson, J.A. Nelson, B.C. Balmer, J.P. Chanton, and D.P. Nowacek. 2017. Niche differentiation and prey selectivity among common bottlenose dolphins (*Tursiops truncatus*) sighted in St. George Sound, Gulf of Mexico. Frontiers in Marine Science 4:235. doi: 10.3389/fmars.2017.00235

Woshner, V., K. Knott, R. Wells, C. Willetto, R. Swor, and T. O'Hara. 2008. Mercury and selenium in blood and epidermis of bottlenose dolphins (*Tursiops truncatus*) from Sarasota Bay, FL: interaction and relevance to life history and hematologic parameters. Ecohealth 5(3):360–370. doi: 10.1007/s10393-008-0164-2

Yordy, J.E., M.A. Mollenhauer, R.M. Wilson, R.S. Wells, A. Hohn, J. Sweeney, L.H. Schwacke, T.K. Rowles, J.R. Kucklick, and M.M. Peden-Adams. 2010a. Complex contaminant exposure in cetaceans: a comparative E-Screen analysis of bottlenose dolphin blubber and mixtures of four persistent organic pollutants. Environmental Toxicology and Chemistry 29(10):2143–2153. doi: 10.1002/etc.279

Yordy, J.E., D.A. Pabst, W.A. McLellan, R.S. Wells, T.K. Rowles, and J.R. Kucklick. 2010b. Tissue-specific distribution and whole-body burden estimates of persistent organic pollutants in the bottlenose dolphin (*Tursiops truncatus*). Environmental Toxicology and Chemistry 29(6):1263–1273. doi: 10.1002/etc.152

Yordy, J.E., R.S. Wells, B.C. Balmer, L.H. Schwacke, T.K. Rowles, and J.R. Kucklick. 2010c. Life history as a source of variation for persistent organic pollutant (POP) patterns in a community of common bottlenose dolphins (*Tursiops truncatus*) resident to Sarasota Bay, FL. Science of the Total Environment 408(9):2163–2172. doi: 10.1016/j.scitotenv.2010.01.032

Common bottlenose dolphin − *Tursiops truncatus*

GoMx BSE/West Bay Stock

Overall Vulnerability Rank = Very High

Biological Sensitivity = Very High

Climate Exposure = Very High

Data Quality = 96% of scores ≥ 2

	Tursiops truncatus	Expert Scores	Data Quality	Expert Scores Plots (Portion by Category)
Sensitivity attributes	Prey/Diet Specificity	2.4	2.2	
	Habitat Specificity	2.8	2.2	
	Site Fidelity	3.6	2.8	
	Lifetime Reproductive Potential	1.5	2.2	
	Generation Length	2.9	2.2	
	Reproductive Plasticity	1.2	2.2	
	Migration	3.8	2.5	
	Home Range	3.5	2.5	
	Stock Abundance	4.0	2.8	
	Stock Abundance Trend	2.0	1.8	
	Cumulative Stressors	2.5	2.8	
	Sensitivity Score	Very High		
Exposure variables	Sea Surface Temperature (Change in mean)	4.0	2.8	
	Sea Surface Temperature (Change in variability)	1.0	2.8	
	Air Temperature (Change in mean)	4.0	2.8	
	Air Temperature (Change in variability)	1.0	2.8	
	Precipitation (Change in mean)	1.0	2.8	
	Precipitation (Change in variability)	1.0	2.8	
	Sea Surface Salinity (Change in mean)	2.0	2.8	
	Sea Surface Salinity (Change in variability)	1.2	2.8	
	Ocean pH (Change in mean)	4.0	2.8	
	Ocean pH (Change in variability)	1.9	2.8	
	Sea ice coverage (Change in mean)	1.0	2.8	
	Sea ice coverage (Change in variability)	1.0	2.8	
	Dissolved oxygen (Change in mean)	4.0	2.8	
	Dissolved oxygen (Change in variability)	1.0	2.8	
	Circulation	3.8	2.5	
	Sea level rise	4.0	2.8	
	Exposure Score	Very High		
Overall Vulnerability Rank		Very High		

Low
Moderate
High
Very High

Common bottlenose dolphin (GoMx BSE/West Bay Stock)

Tursiops truncatus

CVA Results Summary

Overall Climate Vulnerability Rank: Very High (100% certainty from bootstrap analysis).

Climate Exposure: Very High. Six exposure factors scored greater than 3.5: Air Temperature (Standard anomaly) (4.00), Dissolved oxygen (Standard anomaly) (4.00), Ocean pH (Standard anomaly) (4.00), Sea level rise (4.00), Sea Surface Temperature (Standard anomaly) (4.00), and Circulation (3.85).

Biological Sensitivity: Very High. Four sensitivity attributes scored greater than 3.5: Species Abundance (3.95), Migration (3.80), Site Fidelity (3.65), and Home Range (3.55).

Distributional Response: High

Abundance Response: Very High

Phenology Response: High

Data Quality: 96% of the data quality scores were 2 or greater, 91% of sensitivity attribute data quality scores were 2 or higher.

Background Information

Stock Description

This stock includes common bottlenose dolphins in West Bay in the western Gulf of Mexico along the Texas coast (Hayes et al. 2022).

Prey/Diet Specificity

Generally, common bottlenose dolphins consume a large variety of fish and/or squid (Wells and Scott 2018). Common bottlenose dolphins seem to show a consistent preference for noise-producing fishes (e.g., sciaenids, toadfish; Berens McCabe et al. 2010; Bowen-Stevens et al. 2021). In Sarasota Bay, FL, Rossman et al. (2015) found common bottlenose dolphins to show diet specialization despite the broader group showing diet generalist.

Prey studies focusing on common bottlenose dolphins (*Tursiops truncatus*) in the West Bay estuary were not found specifically pertaining to those waters. However, Barros and Odell (1990) examined the stomach contents of 76 stranded bottlenose dolphins from Gulf of Mexico shores, 22 of which were from the shoreline of northeast Texas adjacent to the Galveston Bay system just to the north of West Bay. Those 22 samples were combined with 3 additional stomach content analyses from common bottlenose dolphin strandings recovered in the states of Louisiana, Mississippi, and Alabama (1 sample each), and designated those states as Area 1 of their study. Using a combination of visual taxonomic identification (for undigested species) and species identification keys for fish otolith and cephalopod beaks, Barros and Odell (1990) reported the proportion of prey in Area 1 as fish (94.3%), cephalopods (5.2%), crustaceans (0.5%), and a single horseshoe crab. Only 32% of the stomach contents from Area 1

contained fish only, while fish/cephalopod and fish/cephalopod/shrimp were present in 36% and 32% respectively, of Area 1 stomach contents (Barros and Odell 1990). Overall, sciaenid fishes were the primary fish prey. However, the great diversity of species (over 300 different fish species found in eight stomachs from Area 1) and similarity of species to commercial fisheries bycatch suggests bottlenose dolphins off Texas were supplementing their diet by depredating shrimp trawler bycatch or by taking fish directly out of the nets (presumably) during trawling operations (Barros and Odell 1990). Observations of common bottlenose dolphins feeding using both methods from Texas and Louisiana have been well documented (Gunter 1938; Shane 1977; Gruber 1981; Henningsen and Würsig 1991; Fertl 1994b; Moreno 2005).

While much research indicates sciaenid fishes are the primary prey fishes for bottlenose dolphins in the Gulf of Mexico (Berens McCabe et al. 2010; Dunshea et al. 2013), other researchers (Gunter 1942; Shane 1990; Miller 1992) have suggested striped mullet (*Mugil cephalus*) may be a significant part of the bottlenose dolphin diet in Texas waters. Barros and Wells (1998) reviewed the importance of mullet in the diet of common bottlenose dolphins in Florida waters and suggested the species is important, yet ranks under soniferous fishes in their dietary profile.

Habitat Specificity

Common bottlenose dolphins are found in a variety of marine and estuarine habitats (Wells and Scott 2018).

Site Fidelity

Maze and Würsig (1999) assessed photo-ID data collected from West Bay in years ranging from 1990 to 1996, and found~20% of the individuals observed in 1995–1996 were also present in 1990. Irwin and Würsig (2004) further analyzed photo-ID data collected in West Bay from 1990–2001, and found evidence for strong site fidelity for West Bay individuals. Common bottlenose dolphins in other Gulf of Mexico bay, sound, and estuary (BSE) stocks show site fidelity (LaBrecque et al. 2015; Hayes et al. 2022), such as in Matagorda Bay, Espiritu Santo Bay, and San Luis Pass in Texas (Maze and Würsig 1999; Lynn and Würsig 2002; Irwin and Würsig 2004); Barataria Bay in Louisiana (Miller 2003; McDonald et al. 2017; Wells et al. 2017; Takeshita et al. 2021); Mississippi Sound in Mississippi (Hubard et al. 2004; Mackey 2010; Mullin et al. 2017); Charlotte Harbor and Pine Island Sound (Bassos-Hull et al. 2013), Sarasota Bay (Wells 1986a; Wells 1991; Wells 2014), St. Andrew Bay (Balmer et al. 2019), St. Joseph Bay (Balmer et al. 2008; Balmer et al. 2018), St. Vincent Sound and Apalachicola Bay (Tyson et al. 2011), and Tampa Bay in Florida (Wells et al. 1996b; Urian et al. 2009).

Lifetime Reproductive Potential

Taylor et al. (2007) reported the interbirth interval of common bottlenose dolphins as 3.80 years, though other studies have shown it to be closer to three years (Leatherwood 1977; Wells and Scott 1999; Connor et al. 2000; Wells 2003).

Generally, females reach sexual maturity before males, with females reaching sexual maturity at 5–13 years (Sergeant et al. 1973; Perrin and Reilly 1984; Wells et al. 1987; Mead and Potter 1990; Mann et al. 2000; Wells 2000; Wells and Scott 2018).

Females reproduce through most of their adult lives (Leatherwood 1977; Hohn et al. 1989; Wells and Scott 1999; Wells 2000; Wells 2003), but may produce fewer calves after they reach 30 years of age (Wells 2000). Sarasota Bay common bottlenose dolphins can give birth when they are as young as 6 years of age, and can continue until they are 48 years old (Wells and Scott 2018). Reproductive lifespan would therefore be up to 42 years based on long-term observation studies (Wells 2014; Wells and Scott 2018).

Longitudinal studies from Sarasota Bay, Florida have found that female common bottlenose dolphins can live up to 67 years (Wells and Scott 2018). Studies based on growth layer groups in teeth suggest female common bottlenose dolphins can live to 41 years along the Texas coast (Fernandez and Hohn 1998) and to 30 years along the Mississippi coast (Mattson et al. 2006).

Generation Length

Taylor et al. (2007) reported common bottlenose dolphin generation length of 20.6–21.1 years.

Reproductive Plasticity

Common bottlenose dolphin calving is generally diffusely seasonal, with one or more peaks, although births have been reported from all seasons (Vollmer and Rosel 2013; Wells and Scott 2018). Calf sightings peak in the spring and summer months in Florida (Wells et al. 1987), Mississippi/Louisiana (Miller et al. 2010; Miller et al. 2013), and Texas (McHugh 1989; Henderson and Würsig 2007).

Migration

The BSE stocks in the Gulf of Mexico are thought to primarily comprise stable resident communities. However, transient or seasonal movements of some individuals into and out of a BSE stock area are recorded in some areas (LaBrecque et al. 2015; Hayes et al. 2022). Molecular genetic data support that some BSE stocks are genetically distinct from one another, and furthermore are differentiated from adjacent coastal populations (e.g., Sellas et al. 2005; Rosel et al. 2017). For example, Toms (2019) found fine-scale genetic population structure among five inshore systems in the Florida Panhandle and variable migration rates among populations.

Year-round residency has been reported for common bottlenose dolphins (Hayes et al. 2022), such as in Florida (Irvine and Wells 1972; Irvine et al. 1981; Wells 1986a; Wells 1986b; Wells et al. 1987; Scott et al. 1990; Wells 1991; Wells et al. 1996a; Wells et al. 1996b; Wells et al. 1997; Wells 2003; Balmer et al. 2008; Urian et al. 2009; Tyson et al. 2011; Bassos-Hull et al. 2013; Balmer et al. 2018; Balmer et al. 2019; Toms 2019), Texas (Shane 1990; Bräger 1993; Bräger et al. 1994; Fertl 1994a; Weller 1998; Maze and Würsig 1999; Lynn and Würsig 2002; Irwin and Würsig 2004; Shane 2004), and Louisiana/Mississippi (Hubard et al. 2004; McDonald et al. 2017; Mullin et al. 2017; Takeshita et al. 2021).

Photo-ID studies indicate coastal and inter-bay dolphin movements occur among adjacent stock areas on the north Texas coast (Henningsen and Würsig 1991; Maze and Würsig 1999; Ronje et al. 2020). Maze and Würsig (1999) identified 3 individuals that were observed in both the Galveston Bay and adjacent West Bay stock areas, with one of those individuals remaining in West Bay for up to 9 months after its initial sighting in Galveston Bay. Ronje et al. (2020) photo-identified 15 individuals in the West Bay stock area that were also observed in the adjacent Galveston Bay stock area. More information

needs to be collected to determine if any migratory patterns exist, but transient individuals are thought to frequent the San Luis Pass at the southeastern end of West Bay.

Home Range

The home ranges of individuals within the Gulf of Mexico BSE stocks are considered to generally include the areas within the stock boundaries and brief excursions beyond the stock boundaries (Irvine et al. 1981; Wells 1986a; Wells 1986b; Wells 1991; Wells et al. 1996a; Wells et al. 1996b; Balmer et al. 2008; Wells 2014; Balmer et al. 2019), although some individuals show preference for small areas within the stock boundaries (Lynn and Würsig 2002; Urian et al. 2009; Mullin et al. 2017; Wells et al. 2017). Maze and Würsig (1999), Irwin and Würsig (2004), and Henderson and Würsig (2007) noted higher resident density in the Gulf during cold months and in West Bay during warm months. San Luis Pass (a natural sandy pass connecting West Bay to the Gulf of Mexico) is a relatively small part of West Bay, yet typically contains the highest dolphin density for the West Bay stock (Maze and Würsig 1999).

Stock Abundance

Litz et al. (2019) used photo-identification mark-recapture data to estimate West Bay abundance at 50.63 [unconditional SE 2.23] in the winter and 44.36 [unconditional SE 1.18] in the summer. Ronje et al. (2020) estimated the abundance of bottlenose dolphins in West Bay at 37.9 individuals (95% CI = 28.7–47.1) in the winter and 36.5 individuals (95% CI = 32.7–40.3) in the summer.

Stock Abundance Trend

The estimate of 36.5-37.9 individuals (Ronje et al. 2020) compares similarly with the Irwin and Würsig (2004) estimated abundance of 32 individuals (CV = 0.15) using data from the time period 1997–2001, suggesting the population is relatively stable. Previously, Maze and Würsig (1999) identified 71 individuals, 37 of which used both bay and GOM coastal waters, during the time period 1995–1996.

Cumulative Stressors

Common bottlenose dolphins face a wide range of individual and cumulative natural and anthropogenic threats (see reviews by Vollmer and Rosel 2013; Phillips and Rosel 2014; Wells and Scott 2018; and Wells et al. 2019). These include such threats as shark predation, harmful algal blooms, recreational and commercial fishing gear, vessel traffic, pollution, habitat alteration, and provisioning.

Phillips and Rosel (2014) reviewed the cumulative stressors influencing the West Bay system. Potential health threats range from contagious disease (e.g., *Brucella*), harmful algal blooms (HABs; Fire et al. 2011, 2020), and anthropogenic activities: mining, crude oil exploration, transport, and refinement, commercial fisheries, agricultural runoff in the form of pesticide and fertilizer, heavy metals and other chemical contaminants, marine debris, shipping and dredging, noise, tourism and boat traffic. Natural events may also pose threats: hypoxia resulting from HABs, adverse weather (e.g., hurricane storm surge), habitat loss, and climate change (Phillips and Rosel 2014).

Unusual Mortality Events (UMEs) have been declared for common bottlenose dolphins in waters of Texas with morbillivirus the confirmed cause in 1994, a suspected cause in 1992 along with environmental conditions, and an undetermined cause in 2008 (Litz et al. 2014). This stock was also

within the range of the 2007 UME in which 66 common bottlenose dolphins stranded in northeast Texas and west Louisiana due to an undetermined cause (Litz et al. 2014). A UME was declared in 1990 for the Gulf of Mexico from Florida to Texas, with more than 300 common bottlenose dolphins stranding due to an undetermined cause, although morbillivirus and cold temperatures may have contributed (Litz et al. 2014)

Distribution and Sightings

Sighting maps for this stock from December 2014 and June 2015 are presented in Ronje et al. (2020).

Further Reading

Hayes, S.A., E. Josephson, K. Maze-Foley, P.E. Rosel, and J. Wallace. 2022. U.S. Atlantic and Gulf of Mexico marine mammal stock assessments 2021. National Oceanic and Atmospheric Administration, National Marine Fisheries Service, Northeast Fisheries Science Center: Woods Hole, MA. NOAA Technical Memorandum NMFS-NE-288. 380 p. doi: 10.25923/6tt7-kc16

Wells, R.S. and M.D. Scott. 2018. Bottlenose Dolphin, *Tursiops truncatus*, Common Bottlenose Dolphin. In: Würsig, B., J.G.M. Thewissen, and K.M. Kovacs (Eds.), Encyclopedia of Marine Mammals (Third Edition). Academic Press, London, UK. pp. 118–125. doi: 10.1016/B978-0-12-804327-1.00072-8

Wells, R.S, A. Natoli, and G. Braulik. 2019. *Tursiops truncatus* (errata version published in 2019). The IUCN Red List of Threatened Species 2019: e.T22563A156932432. https://dx.doi.org/10.2305/IUCN.UK.2019-1.RLTS.T22563A156932432.en

References

Balmer, B., T. McDonald, F. Hornsby, J. Adams, J. Allen, A. Barleycorn, and L. Schwacke. 2018. Long-term trends in a northern Gulf of Mexico common bottlenose dolphin (*Tursiops truncatus*) population in the wake of the Deepwater Horizon oil spill. Journal of Cetacean Research and Management 18:1–9. doi: 10.47536/jcrm.v18i1.409

Balmer, B., S. Watwood, B. Quigley, T. Speakman, K. Barry, K. Mullin, P. Rosel, C. Sinclair, E. Zolman, and L. Schwacke. 2019. Common bottlenose dolphin (*Tursiops truncatus*) abundance and distribution patterns in St. Andrew Bay, Florida, USA. Aquatic Conservation: Marine and Freshwater Ecosystems 29(3):486–498. doi: 10.1002/aqc.3001

Balmer, B.C., R.S. Wells, S.M. Nowacek, D.P. Nowacek, L.H. Schwacke, W.A. McLellan, F.S. Scharf, T.K. Rowles, L.J. Hansen, T.R. Spradlin, and D.A. Pabst. 2008. Seasonal abundance and distribution patterns of common bottlenose dolphins (*Tursiops truncatus*) near St. Joseph Bay, Florida, USA. Journal of Cetacean Research and Management 10(2):157–167.

Barros, N.B. and D.K. Odell. 1990. Food habits of bottlenose dolphins in the southeastern United States In: Leatherwood, S. and R.R. Reeves (Eds.), The Bottlenose Dolphin. Academic Press, San Diego, CA. pp. 309–328. doi: 10.1016/B978-0-12-440280-5.50020-2

Barros, N.B. and R.S. Wells. 1998. Prey and feeding patterns of resident bottlenose dolphins (*Tursiops truncatus*) in Sarasota Bay, Florida. Journal of Mammalogy 79(3):1045–1059. doi: 10.2307/1383114

Bassos-Hull, K., R.M. Perrtree, C.C. Shepard, S. Schilling, A.A. Barleycorn, J.B. Allen, B.C. Balmer, W.E. Pine, and R.S. Wells. 2013. Long-term site fidelity and seasonal abundance estimates of common bottlenose dolphins (*Tursiops truncatus*) along the southwest coast of Florida and responses to

natural perturbations. Journal of Cetacean Research and Management 13(1):19–30. doi: 10.47536/jcrm.v13i1.551

Berens McCabe, E.J., D.P. Gannon, N.B. Barros, and R.S. Wells. 2010. Prey selection by resident common bottlenose dolphins (*Tursiops truncatus*) in Sarasota Bay, Florida. Marine Biology 157(5):931–942. doi: 10.1007/s00227-009-1371-2

Bowen-Stevens, S.R., D.P. Gannon, R.A. Hazelkorn, G. Lovewell, K.M. Volker, S. Smith, M.C. Tumlin, and J. Litz. 2021. Diet of common bottlenose dolphins, *Tursiops truncatus*, that stranded in and near Barataria Bay, Louisiana, 2010–2012. Southeastern Naturalist 20(1). doi: 10.1656/058.020.0113

Bräger, S. 1993. Diurnal and seasonal behavior patterns of bottlenose dolphins (*Tursiops truncatus*). Marine Mammal Science 9:434–438. doi: 10.1111/j.1748-7692.1993.tb00477.x

Bräger, S., B. Würsig, A. Acevedo, and T. Henningsen. 1994. Association patterns of bottle-nosed dolphins (*Tursiops-truncatus*) in Galveston Bay, Texas. Journal of Mammalogy 75(2):431–437. doi: 10.2307/1382564

Connor, R.C., R.S. Wells, J. Mann, and A.J. Read. 2000. The bottlenose dolphin: Social relationships in a fission-fusion society. In: Mann, J., R. Connor, P. Tyack, and H. Whitehead (Eds.), Cetacean Societies: Field Studies of Dolphins and Whales. University of Chicago Press, Chicago, IL. pp. 91–126.

Dunshea, G., N.B. Barros, E.J. Berens McCabe, N.J. Gales, M.A. Hindell, S.N. Jarman, and R.S. Wells. 2013. Stranded dolphin stomach contents represent the free-ranging population's diet. Biology Letters 9(3):20121036–20121036. doi: 10.1098/rsbl.2012.1036

Fernandez, S. and A.A. Hohn. 1998. Age, growth, and calving season of bottlenose dolphins, *Tursiops truncatus*, off coastal Texas. Fishery Bulletin 96(2):357–365.

Fertl, D. 1994a. Occurrence patterns and behavior of bottlenose dolphins (*Tursiops truncatus*) in the Galveston ship channel, Texas. Texas Journal of Science 46(4):299–318.

Fertl, D. 1994b. Occurrence, movements, and behavior of bottlenose dolphins (*Tursiops truncatus*) in association with the shrimp fishery in Galveston Bay, Texas. Texas A&M University at Galveston. Thesis. 116 p.

Fire, S.E., T.A. Leighfield, G.A. Miller, S. Piwetz, E.R. Sabater, and H. Whitehead. 2020. Association between red tide exposure and detection of corresponding neurotoxins in bottlenose dolphins from Texas waters during 2007-2017. Marine Environmental Research 162:105191. doi: 10.1016/j.marenvres.2020.105191

Fire, S.E., Z. Wang, M. Byrd, H.R. Whitehead, J. Paternoster, and S.L. Morton. 2011. Co-occurrence of multiple classes of harmful algal toxins in bottlenose dolphins (*Tursiops truncatus*) stranding during an unusual mortality event in Texas, USA. Harmful Algae 10(3):330–336. doi: 10.1016/j.hal.2010.12.001

Gruber, J.A. 1981. Ecology of the Atlantic bottlenosed dolphin (*Tursiops truncatus*) in the Pass Cavallo area of Matagorda Bay, Texas. Texas A&M University. Thesis. 182 p.

Gunter, G. 1938. Seasonal variations in abundance of certain estuarine and marine fishes in Louisiana, with particular reference to life histories. Ecological Monographs 8:314–346.

Gunter, G. 1942. Contributions to the natural history of the bottle-nose dolphin *Tursiops truncatus* (Montague) on the Texas coast. Journal of Mammalogy 23:267–276.

Hayes, S.A., E. Josephson, K. Maze-Foley, P.E. Rosel, and J. Wallace. 2022. U.S. Atlantic and Gulf of Mexico marine mammal stock assessments 2021. National Oceanic and Atmospheric Administration, National Marine Fisheries Service, Northeast Fisheries Science Center: Woods Hole, MA. NOAA Technical Memorandum NMFS-NE-288. 380 p. doi: 10.25923/6tt7-kc16

Henderson, E.E. and B. Würsig. 2007. Behavior patterns of bottlenose dolphins in San Luis Pass, Texas. Gulf of Mexico Science 25(2):153–161. doi: 10.18785/goms.2502.06

Henningsen, T. and B. Würsig. 1991. Bottle-nosed dolphins in Galveston Bay, Texas: Numbers and activities. Proceedings of the Fifth Annual Conference of the European Cetacean Society, Sandefjord, Norway, 21–23 February 1991. pp. 36–38.

Hohn, A.A., M.D. Scott, R.S. Wells, J.C. Sweeney, and A.B. Irvine. 1989. Growth layers in teeth from known-age, free-ranging bottlenose dolphins. Marine Mammal Science 5(4):315–342. doi: 10.1111/j.1748-7692.1989.tb00346.x

Hubard, C.W., K. Maze-Foley, K.D. Mullin, and W.W. Schroeder. 2004. Seasonal abundance and site fidelity of bottlenose dolphins (*Tursiops truncatus*) in Mississippi Sound. Aquatic Mammals 30(2):299–310. doi: 10.1578/am.30.2.2004.299

Irvine, A.B., M.D. Scott, R.S. Wells, and J.H. Kaufmann. 1981. Movements and activities of the Atlantic bottlenose dolphin, *Tursiops truncatus*, near Sarasota, Florida. Fishery Bulletin 79(4):671–688.

Irvine, B. and R.S. Wells. 1972. Results of attempts to tag Atlantic bottlenosed dolphins (*Tursiops truncatus*). Cetology 13:1–5.

Irwin, L.-J. and B. Würsig. 2004. A small resident community of bottlenose dolphins, *Tursiops truncatus*, in Texas: Monitoring recommendations. Gulf of Mexico Science 22(1):13–21. doi: 10.18785/goms.2201.02

LaBrecque, E., C. Curtice, J. Harrison, S.M. Van Parijs, and P.N. Halpin. 2015. 3. Biologically Important Areas for cetaceans within U.S. Waters – Gulf of Mexico Region. Aquatic Mammals 41(1):30–38. doi: 10.1578/am.41.1.2015.30

Leatherwood, S. 1977. Some preliminary impressions on the numbers and social behavior of free-swimming bottlenosed dolphin calves (*Tursiops truncatus*) in the northern Gulf of Mexico. In: Ridgway, S.H. and K. Bernirschke (Eds.), Breeding Dolphins; Present Status, Suggestions for the Future. Final report to the US Marine Mammal Commission, Contract MM6AC009, MMC-76/07, Washington, DC. pp. 143–167.

Litz, J.A., M.A. Baran, S.R. Bowen-Stevens, R.H. Carmichael, K.M. Colegrove, L.P. Garrison, S.E. Fire, E.M. Fougeres, R. Hardy, S. Holmes, W. Jones, B.E. Mase-Guthrie, D.K. Odell, P.E. Rosel, J.T. Saliki, D.K. Shannon, S.F. Shippee, S.M. Smith, E.M. Stratton, M.C. Tumlin, H.R. Whitehead, G.A. Worthy, and T.K. Rowles. 2014. Review of historical unusual mortality events (UMEs) in the Gulf of Mexico (1990–2009): providing context for the multi-year northern Gulf of Mexico cetacean UME declared in 2010. Diseases of Aquatic Organisms 112(2):161–175. doi: 10.3354/dao02807

Litz, J.A., E.I. Ronje, H.R. Whitehead, and L.P. Garrison. 2019. Updated abundance estimates for common bottlenose dolphins (*Tursiops truncatus*) inhabiting West Bay, Texas. Aquatic Conservation: Marine and Freshwater Ecosystems 29(11):1959–1970. doi: 10.1002/aqc.3195

Lynn, S.K. and B. Würsig. 2002. Summer movement patterns of bottlenose dolphins in a Texas bay. Gulf of Mexico Science 20(1):25–37. doi: 10.18785/goms.2001.03

Mackey, A.D. 2010. Site fidelity and association patterns of bottlenose dolphins (*Tursiops truncatus*) in the Mississippi Sound. University of Southern Mississippi. Thesis. 133 p.

Mann, J., R.C. Connor, L.M. Barre, and M.R. Heithaus. 2000. Female reproductive success in bottlenose dolphins (*Tursiops* sp.): life history, habitat, provisioning, and group-size effects. Behavioral Ecology 11(2):210–219. doi: 10.1093/beheco/11.2.210

Mattson, M.C., K.D. Mullin, G.W. Ingram, and W. Hoggard. 2006. Age structure and growth of the bottlenose dolphin (*Tursiops truncatus*) from strandings in the Mississippi sound region of the north-central Gulf of Mexico from 1986 to 2003. Marine Mammal Science 22(3):654–666. doi: 10.1111/j.1748-7692.2006.00057.x

Maze, K.S. and B. Würsig. 1999. Bottlenosed dolphins of San Luis Pass, Texas: Occurrence patterns, site-fidelity, and habitat use. Aquatic Mammals 25:91–104.

McDonald, T.L., F.E. Hornsby, T.R. Speakman, E.S. Zolman, K.D. Mullin, C. Sinclair, P.E. Rosel, L. Thomas, and L.H. Schwacke. 2017. Survival, density, and abundance of common bottlenose dolphins in

Barataria Bay (USA) following the Deepwater Horizon oil spill. Endangered Species Research 33:193–209. doi: 10.3354/esr00806

McHugh, M.B. 1989. Population numbers and feeding behavior of the Atlantic bottlenose dolphin (*Tursiops truncatus*) near Aransas Pass, Texas. University of Texas at Austin. Thesis. 96 p.

Mead, J.G. and C.W. Potter. 1990. Natural history of bottlenose dolphins along the central Atlantic coast of the United States. In: Leatherwood, S. and R.R. Reeves (Eds.), The Bottlenose Dolphin. Academic Press, San Diego, CA. pp. 165–195.

Miller, C.E. 2003. Abundance trends and environmental habitat usage patterns of bottlenose dolphins (*Tursiops truncatus*) in lower Barataria and Caminada bays, Louisiana. Louisiana State University and Agricultural & Mechanical College. Dissertation. 125 p.

Miller, L.J., A.D. Mackey, T. Hoffland, M. Solangi, and S.A. Kuczaj. 2010. Potential effects of a major hurricane on Atlantic bottlenose dolphin (*Tursiops truncatus*) reproduction in the Mississippi Sound. Marine Mammal Science 26(3):707–715. doi: 10.1111/j.1748-7692.2010.00371.x

Miller, L.J., A.D. Mackey, M. Solangi, and S.A. Kuczaj. 2013. Population abundance and habitat utilization of bottlenose dolphins in the Mississippi Sound. Aquatic Conservation: Marine and Freshwater Ecosystems 23(1):145–151. doi: 10.1002/aqc.2278

Miller, W.G. 1992. An investigation of bottlenose dolphin *Tursiops truncatus* deaths in East Matagorda Bay, Texas, January 1990. Fishery Bulletin 90:791–797.

Moreno, M.P.T. 2005. Environmental predictors of bottlenose dolphin distribution and core feeding densities in Galveston Bay, Texas. Texas A&M University at Galveston. Dissertation. 88 p.

Mullin, K.D., T. McDonald, R.S. Wells, B.C. Balmer, T. Speakman, C. Sinclair, E.S. Zolman, F. Hornsby, S.M. McBride, K.A. Wilkinson, and L.H. Schwacke. 2017. Density, abundance, survival, and ranging patterns of common bottlenose dolphins (*Tursiops truncatus*) in Mississippi Sound following the Deepwater Horizon oil spill. PLoS ONE 12(10):e0186265. doi: 10.1371/journal.pone.0186265

Perrin, W.F. and S.B. Reilly. 1984. Reproductive parameters of dolphins and small whales of the family Delphinidae. In: Perrin, W.F., R.L. Brownell, Jr., and D.P. DeMaster (Eds.), Reproduction in Whales, Dolphins, and Porpoises. pp. 97–134.

Phillips, N.M. and P.E. Rosel. 2014. A method for prioritizing research on common bottlenose dolphin stocks through evaluating threats and data availability: Development and application to bay, sound and estuary stocks in Texas. National Oceanic and Atmospheric Administration, National Marine Fisheries Service, Southeast Fisheries Science Center: Lafayette, LA. NOAA Technical Memorandum NMFS-SEFSC-665. 146 p. doi: 10.7289/V5F769H8

Ronje, E., H. Whitehead, K. Barry, S. Piwetz, J. Struve, V. Lecours, L. Garrison, R.S. Wells, and K.D. Mullin. 2020. Abundance and occurrence of common bottlenose dolphins (*Tursiops truncatus*) in three estuaries of the northwestern Gulf of Mexico. Gulf and Caribbean Research 31:18–34. doi: 10.18785/gcr.3101.09

Rosel, P.E., L.A. Wilcox, C. Sinclair, T.R. Speakman, M.C. Tumlin, J.A. Litz, and E.S. Zolman. 2017. Genetic assignment to stock of stranded common bottlenose dolphins in southeastern Louisiana after the Deepwater Horizon oil spill. Endangered Species Research 33:221–234. doi: 10.3354/esr00780

Rossman, S., P.H. Ostrom, M. Stolen, N.B. Barros, H. Gandhi, C.A. Stricker, and R.S. Wells. 2015. Individual specialization in the foraging habits of female bottlenose dolphins living in a trophically diverse and habitat rich estuary. Oecologia 178(2):415–425. doi: 10.1007/s00442-015-3241-6

Scott, M.D. and S.J. Chivers. 1990. Distribution and herd structure of bottlenose dolphins in the eastern tropical Pacific Ocean. In: Leatherwood, S. and R.R. Reeves (Eds.), The Bottlenose Dolphin. Academic Press, San Diego, CA. pp. 387–402.

Sellas, A.B., R.S. Wells, and P.E. Rosel. 2005. Mitochondrial and nuclear DNA analyses reveal fine scale geographic structure in bottlenose dolphins (*Tursiops truncatus*) in the Gulf of Mexico. Conservation Genetics 6(5):715–728. doi: 10.1007/s10592-005-9031-7

Sergeant, D.E., D.K. Caldwell, and M.C. Caldwell. 1973. Age, growth, and maturity of bottlenosed dolphin (*Tursiops-truncatus*) from northeast Florida. Journal of the Fisheries Research Board of Canada 30(7):1009–1011. doi: 10.1139/f73-165

Shane, S. 1977. The population biology of the Atlantic bottlenose dolphin, *Tursiops truncatus*, in the Aransas Pass area of Texas. Texas A&M University. Thesis.

Shane, S.H. 1990. Behavior and ecology of the bottlenose dolphin at Sanibel Island, Florida. In: Leatherwood, S. and R.R. Reeves (Eds.), The Bottlenose Dolphin. Academic Press, San Diego, CA. pp. 245–265. doi: 10.1016/B978-0-12-440280-5.50016-0

Shane, S.H., R.S. Wells, and B. Würsig. 1986. Ecology, behavior and social organization of the bottlenose dolphin: A review. Marine Mammal Science 2:34–63. doi: 10.1111/j.1748-7692.1986.tb00026.x

Takeshita, R., B.C. Balmer, F. Messina, E.S. Zolman, L. Thomas, R.S. Wells, C.R. Smith, T.K. Rowles, and L.H. Schwacke. 2021. High site-fidelity in common bottlenose dolphins despite low salinity exposure and associated indicators of compromised health. PLoS ONE 16(9):e0258031. doi: 10.1371/journal.pone.0258031

Taylor, B.L., S.J. Chivers, J. Larese, and W.F. Perrin. 2007. Generation length and percent mature estimates for IUCN assessments of cetaceans. National Oceanic and Atmospheric Administration, National Marine Fisheries Service, Southwest Fisheries Science Center: La Jolla, CA. Administrative Report LJ-07-01. 24 p.

Toms, C. 2019. Filling the gaps: Common bottlenose dolphin (*Tursiops truncatus*) population dynamics, structure, and connectivity within Florida panhandle bays, sounds, and estuaries. University of Central Florida. Dissertation. 373 p.

Tyson, R.B., S.M. Nowacek, and D.P. Nowacek. 2011. Community structure and abundance of bottlenose dolphins *Tursiops truncatus* in coastal waters of the northeast Gulf of Mexico. Marine Ecology Progress Series 438:253–265. doi: 10.3354/meps09292

Urian, K.W., S. Hofmann, R.S. Wells, and A.J. Read. 2009. Fine-scale population structure of bottlenose dolphins (*Tursiops truncatus*) in Tampa Bay, Florida. Marine Mammal Science 25(3):619–638. doi: 10.1111/j.1748-7692.2009.00284.x

Vollmer, N.L. and P.E. Rosel. 2013. A review of common bottlenose dolphins (*Tursiops truncatus truncatus*) in the Northern Gulf of Mexico: Population biology, potential threats, and management. Southeastern Naturalist 12(m6):1–43. doi: 10.1656/058.012.m601

Weller, D.W. 1998. Global and regional variation in the biology and behavior of bottlenose dolphins. Texas A&M University. Dissertation. 142 p.

Wells, R.S. 1986a. Population structure of bottlenose dolphins: Behavioral studies along the central west coast of Florida. Contract Report to National Marine Fisheries Service, Southeast Fisheries Center. Contract No. 45-WCNF-5-003BB. 58 p.

Wells, R.S. 1986b. Structural aspects of dolphin societies. University of California, Santa Cruz. Dissertation. 234 p.

Wells, R.S. 1991. The role of long-term study in understanding the social structure of a bottlenose dolphin community. In: Pryor, K. and K. Norris (Eds.), Dolphin Societies: Discoveries and Puzzles. University of California Press, Berkeley, CA. pp. 199–226.

Wells, R.S. 2000. Reproduction in wild bottlenose dolphins: Overview of patterns observed during a long-term study. In: Duffield, D. and T. Robeck (Eds.), Bottlenose Dolphin Reproduction Workshop. AZA Marine Mammal Taxon Advisory Group, Silver Spring, MD. pp. 57–74.

Wells, R.S. 2003. Dolphin social complexity: Lessons from long-term study and life history. In: de Waal, F.B.M. and P.L. Tyack (Eds.), Animal Social Complexity: Intelligence, Culture, and Individualized Societies Harvard University Press, Cambridge, MA. pp. 32–56.

Wells, R.S. 2014. Social structure and life history of bottlenose dolphins near Sarasota Bay, Florida: Insights from four decades and five generations. Primates and cetaceans. pp. 149–172. doi: 10.1007/978-4-431-54523-1_8

Wells, R.S., M.K. Bassos, K.W. Urian, W.J. Carr, and M.D. Scott. 1996. Low-level monitoring of bottlenose dolphins, *Tursiops truncatus*, in Charlotte Harbor, Florida: 1990-1994. National Oceanic and Atmospheric Administration, National Marine Fisheries Service, Southeast Fisheries Science Center: Miami, FL. NOAA Technical Memorandum NMFS-SEFSC-384. 86 p.

Wells, R.S., L.H. Schwacke, T.K. Rowles, B.C. Balmer, E. Zolman, T. Speakman, F.I. Townsend, M.C. Tumlin, A. Barleycorn, and K.A. Wilkinson. 2017. Ranging patterns of common bottlenose dolphins *Tursiops truncatus* in Barataria Bay, Louisiana, following the Deepwater Horizon oil spill. Endangered Species Research 33:159–180. doi: 10.3354/esr00732

Wells, R.S. and M.D. Scott. 1999. Bottlenose dolphin *Tursiops truncatus* (Montagu, 1821). In: Ridgway, S.H. and R. Harrison (Eds.), Handbook of marine mammals. Volume 6: The second book of dolphins and the porpoises. Academic Press, San Diego, CA. pp. 137–182.

Wells, R.S. and M.D. Scott. 2018. Bottlenose dolphin, *Tursiops truncatus*, common bottlenose dolphin. In: Würsig, B., J.G.M. Thewissen, and K.M. Kovacs (Eds.), Encyclopedia of Marine Mammals (Third Edition). Academic Press, London, UK. pp. 118–125. doi: 10.1016/B978-0-12-804327-1.00072-8

Wells, R.S., M.D. Scott, and A.B. Irvine. 1987. The social structure of free-ranging bottlenose dolphins. In: Genoways, H.H. (Ed.), Current Mammalogy. Plenum Press, New York, NY. pp. 247–305.

Wells, R.S., K.W. Urian, A.J. Read, M.K. Bassos, W.J. Carr, and M.D. Scott. 1996. Low-level monitoring of bottlenose dolphins, *Tursiops truncatus*, in Tampa Bay, Florida: 1988-1993. National Oceanic and Atmospheric Administration, National Marine Fisheries Service, Southeast Fisheries Science Center: Miami, FL. NOAA Technical Memorandum NMFS-SEFSC-385. 36 p.

Common bottlenose dolphin − *Tursiops truncatus*

GoMx BSE/Whitewater Bay Stock

Overall Vulnerability Rank = Very High

Biological Sensitivity = High

Climate Exposure = Very High

Data Quality = 78% of scores ≥ 2

	Tursiops truncatus	Expert Scores	Data Quality	Expert Scores Plots (Portion by Category)
Sensitivity attributes	Prey/Diet Specificity	2.5	1.3	
	Habitat Specificity	3.3	1.7	
	Site Fidelity	3.1	1.7	
	Lifetime Reproductive Potential	2.1	2.0	
	Generation Length	2.7	2.0	
	Reproductive Plasticity	1.3	2.0	
	Migration	3.8	2.0	
	Home Range	3.8	2.3	
	Stock Abundance	2.6	1.3	
	Stock Abundance Trend	1.9	1.0	
	Cumulative Stressors	2.8	1.3	
	Sensitivity Score	High		
Exposure variables	Sea Surface Temperature (Change in mean)	3.9	2.3	
	Sea Surface Temperature (Change in variability)	2.1	2.3	
	Air Temperature (Change in mean)	2.9	2.3	
	Air Temperature (Change in variability)	1.1	2.3	
	Precipitation (Change in mean)	1.1	2.3	
	Precipitation (Change in variability)	1.1	2.3	
	Sea Surface Salinity (Change in mean)	3.2	2.3	
	Sea Surface Salinity (Change in variability)	1.1	2.3	
	Ocean pH (Change in mean)	3.7	2.3	
	Ocean pH (Change in variability)	1.1	2.3	
	Sea ice coverage (Change in mean)	1.0	3.0	
	Sea ice coverage (Change in variability)	1.0	3.0	
	Dissolved oxygen (Change in mean)	3.7	2.3	
	Dissolved oxygen (Change in variability)	1.1	2.3	
	Circulation	3.8	2.7	
	Sea level rise	3.9	2.3	
	Exposure Score	Very High		
Overall Vulnerability Rank		Very High		

Low
Moderate
High
Very High

Common bottlenose dolphin (GoMx BSE/Whitewater Bay Stock)

Tursiops truncatus

CVA Results Summary

Overall Climate Vulnerability Rank: Very High (100% certainty from bootstrap analysis).

Climate Exposure: Very High. Five exposure factors scored greater than 3.5: Sea Surface Temperature (Standard anomaly) (3.93), Sea level rise (3.87), Circulation (3.80), Dissolved oxygen (Standard anomaly) (3.73), and Ocean pH (Standard anomaly) (3.73).

Biological Sensitivity: High. Four sensitivity attributes scored greater than 3.0: Home Range (3.80), Migration (3.80), Habitat Specificity (3.33), and Site Fidelity (3.13).

Distributional Response: High

Abundance Response: High

Phenology Response: High

Data Quality: 78% of the data quality scores were 2 or greater, 45% of sensitivity attribute data quality scores were 2 or higher.

Background Information

Stock Description

This stock includes common bottlenose dolphins in Whitewater Bay in the eastern Gulf of Mexico along the Florida coast (Hayes et al. 2022).

Prey/Diet Specificity

Generally, common bottlenose dolphins consume a large variety of fish and/or squid (Barros and Odell 1990; Mead and Potter 1990; Wells and Scott 2018). Common bottlenose dolphins seem to show a consistent preference for sound-producing fishes (e.g., sciaenids, toadfish; Berens McCabe et al. 2010; Bowen-Stevens et al. 2021). In Sarasota Bay, Florida, Rossman et al. (2015) found some individual common bottlenose dolphins to show diet specialization despite the broader study group showing a generalist diet. Specialization can be influenced by locally abundant prey species (Shane et al. 1986; Vollmer and Rosel 2013) or can result from resource partitioning amongst sympatric populations (e.g., Torres and Read 2009; Wilson et al. 2017; Matich et al. 2017).

Habitat Specificity

Common bottlenose dolphins are found in a variety of marine and estuarine habitats (Wells and Scott 2018).

Site Fidelity

Limited information is available on site fidelity for this stock. However, from August 2010 to June 2012, a total of 92 individuals were identified in Whitewater Bay (Sarabia et al. 2018), but recapture rates were not documented. In other regions, common bottlenose dolphins in other Gulf of Mexico bay, sound, and estuary (BSE) stocks show site fidelity (LaBrecque et al. 2015; Hayes et al. 2022), such as in Charlotte Harbor and Pine Island Sound (Bassos-Hull et al. 2013), Sarasota Bay (Wells 1986a; Wells 1991; Wells 2014), St. Andrew Bay (Balmer et al. 2019), St. Joseph Bay (Balmer et al. 2008; B. Balmer et al. 2018), St. Vincent Sound and Apalachicola Bay (Tyson et al. 2011), and Tampa Bay in Florida (Wells et al. 1996b; Urian et al. 2009); Matagorda Bay, Espiritu Santo Bay, and San Luis Pass in Texas (Maze and Würsig 1999; Lynn and Würsig 2002; Irwin and Würsig 2004); Barataria Bay in Louisiana (Miller 2003; McDonald et al. 2017; Wells et al. 2017; Takeshita et al. 2021); and Mississippi Sound in Mississippi (Hubard et al. 2004; Mackey 2010; Mullin et al. 2017).

Lifetime Reproductive Potential

Taylor et al. (2007) reported the interbirth interval of common bottlenose dolphins as 3.80 years, though other studies have shown it to be closer to three years (Leatherwood 1977; Wells and Scott 1999; Connor et al. 2000; Wells 2003).

Generally, females reach sexual maturity before males, with females reaching sexual maturity at 5–13 years (Sergeant et al. 1973; Perrin and Reilly 1984; Wells et al. 1987; Mead and Potter 1990; Mann et al. 2000; Wells 2000; Wells and Scott 2018).

Females reproduce through most of their adult lives (Leatherwood 1977; Hohn et al. 1989; Wells and Scott 1999; Wells 2000; Wells 2003), but may produce fewer calves after they reach 30 years of age (Wells 2000). Sarasota common bottlenose dolphins can give birth when they are as young as 6 years of age, and can continue until they are 48 years old (Wells and Scott 2018). Reproductive lifespan would therefore be up to 42 years based on long-term observation studies (Wells 2014; Wells and Scott 2018).

Longitudinal studies from Sarasota Bay, Florida have found that female common bottlenose dolphins can live up to 67 years (Wells and Scott 2018). Studies based on growth layer groups in teeth suggest female common bottlenose dolphins can live to 41 years along the Texas coast (Fernandez and Hohn 1998) and to 30 years along the Mississippi coast (Mattson et al. 2006).

Generation Length

Taylor et al. (2007) reported common bottlenose dolphin generation length of 20.6–21.1 years.

Reproductive Plasticity

Common bottlenose dolphin calving is generally diffusely seasonal, with one or more peaks, although births have been reported from all seasons (Vollmer and Rosel 2013; Wells and Scott 2018). Calf sightings peak in the spring and summer months in Florida (Wells et al. 1987), Mississippi/Louisiana (Miller et al. 2010; Miller et al. 2013), and Texas (McHugh 1989; Henderson and Würsig 2007).

Migration

The BSE stocks in the Gulf of Mexico are thought to primarily comprise stable resident communities. However, transient or seasonal movements of some individuals into and out of a BSE stock area are recorded in some areas (LaBrecque et al. 2015; Hayes et al. 2022). Molecular genetic data support that some BSE stocks are genetically distinct from one another, and furthermore are differentiated from adjacent coastal populations (e.g., Sellas et al. 2005; Rosel et al. 2017). For example, Toms (2019) found fine-scale genetic population structure among five inshore systems in the Florida Panhandle and variable migration rates among populations.

Year-round residency has been reported for common bottlenose dolphins (Hayes et al. 2022), such as in Florida (Irvine and Wells 1972; Irvine et al. 1981; Wells 1986a; Wells 1986b; Wells et al. 1987; Scott et al. 1990; Wells 1991; Wells et al. 1996a; Wells et al. 1996b; Wells et al. 1997; Wells 2003; Balmer et al. 2008; Urian et al. 2009; Tyson et al. 2011; Bassos-Hull et al. 2013; B. Balmer et al. 2018; Balmer et al. 2019; Toms 2019), Texas (Shane 1990; Bräger 1993; Bräger et al. 1994; Fertl 1994; Weller 1998; Maze and Würsig 1999; Lynn and Würsig 2002; Irwin and Würsig 2004; Shane 2004), and Louisiana/Mississippi (Hubard et al. 2004; McDonald et al. 2017; Mullin et al. 2017; Takeshita et al. 2021).

Home Range

The home ranges of individuals within the Gulf of Mexico BSE stocks are considered to generally include the areas within the stock boundaries and brief excursions beyond the stock boundaries (Irvine et al. 1981; Wells 1986a; Wells 1986b; Wells 1991; Wells et al. 1996a; Wells et al. 1996b; Balmer et al. 2008; Wells 2014; Balmer et al. 2019), although some individuals show preference for small areas within the stock boundaries (Lynn and Würsig 2002; Urian et al. 2009; Mullin et al. 2017; Wells et al. 2017).

Stock Abundance

An abundance estimate for this stock is unavailable (Hayes et al. 2022).

Stock Abundance Trend

Data are insufficient to complete an abundance trend analysis for this stock (Hayes et al. 2022).

Cumulative Stressors

Common bottlenose dolphins face a wide range of individual and cumulative natural and anthropogenic threats (see reviews by Vollmer and Rosel 2013; Wells and Scott 2018; and Wells et al. 2019). These include such threats as shark predation, harmful algal blooms, recreational and commercial fishing gear, vessel traffic, pollution, habitat alteration, and provisioning.

The nearshore and estuarine habitats along the Gulf of Mexico coast include waters adjacent to areas of high human population, some of which are highly industrialized, and areas of heavy agricultural impact. Studies of bottlenose dolphin tissues from southwest Florida have shown persistent organic pollutants (POPs) in blubber and blood samples (Balmer et al. 2015; Damseaux et al. 2017; J. Balmer et al. 2018). Similarly, mercury has been detected in common bottlenose dolphin tissues in this stock (Damseaux et al. 2017) and elsewhere in Florida Gulf Coast waters (McCormack et al. 2020a, 2020b, 2022).

An Unusual Mortality Event (UME) was declared in 1990 for the Gulf of Mexico from Florida to Texas, with more than 300 common bottlenose dolphins stranding due to an undetermined cause, although morbillivirus and cold temperatures may have contributed (Litz et al. 2014).

Further Reading

Hayes, S.A., E. Josephson, K. Maze-Foley, P.E. Rosel, and J. Wallace. 2022. U.S. Atlantic and Gulf of Mexico marine mammal stock assessments 2021. National Oceanic and Atmospheric Administration, National Marine Fisheries Service, Northeast Fisheries Science Center: Woods Hole, MA. NOAA Technical Memorandum NMFS-NE-288. 380 p. doi: 10.25923/6tt7-kc16

Wells, R.S. and M.D. Scott. 2018. Bottlenose Dolphin, *Tursiops truncatus*, Common Bottlenose Dolphin. In: Würsig, B., J.G.M. Thewissen, and K.M. Kovacs (Eds.), Encyclopedia of Marine Mammals (Third Edition). Academic Press, London, UK. pp. 118–125. doi: 10.1016/B978-0-12-804327-1.00072-8

Wells, R.S., A. Natoli, and G. Braulik. 2019. *Tursiops truncatus* (errata version published in 2019). The IUCN Red List of Threatened Species 2019: e.T22563A156932432. https://dx.doi.org/10.2305/IUCN.UK.2019-1.RLTS.T22563A156932432.en

References

Balmer, B., T. McDonald, F. Hornsby, J. Adams, J. Allen, A. Barleycorn, and L. Schwacke. 2018. Long-term trends in a northern Gulf of Mexico common bottlenose dolphin (*Tursiops truncatus*) population in the wake of the Deepwater Horizon oil spill. Journal of Cetacean Research and Management 18:1–9. doi: 10.47536/jcrm.v18i1.409

Balmer, B., S. Watwood, B. Quigley, T. Speakman, K. Barry, K. Mullin, P. Rosel, C. Sinclair, E. Zolman, and L. Schwacke. 2019. Common bottlenose dolphin (*Tursiops truncatus*) abundance and distribution patterns in St. Andrew Bay, Florida, USA. Aquatic Conservation: Marine and Freshwater Ecosystems 29(3):486–498. doi: 10.1002/aqc.3001

Balmer, B.C., R.S. Wells, S.M. Nowacek, D.P. Nowacek, L.H. Schwacke, W.A. McLellan, F.S. Scharf, T.K. Rowles, L.J. Hansen, T.R. Spradlin, and D.A. Pabst. 2008. Seasonal abundance and distribution patterns of common bottlenose dolphins (*Tursiops truncatus*) near St. Joseph Bay, Florida, USA. Journal of Cetacean Research and Management 10(2):157–167.

Balmer, B.C., G.M. Ylitalo, L.E. McGeorge, K.A. Baugh, D. Boyd, K.D. Mullin, P.E. Rosel, C. Sinclair, R.S. Wells, E.S. Zolman, and L.H. Schwacke. 2015. Persistent organic pollutants (POPs) in blubber of common bottlenose dolphins (*Tursiops truncatus*) along the northern Gulf of Mexico coast, USA. Science of the Total Environment 527-528:306–312. doi: 10.1016/j.scitotenv.2015.05.016

Balmer, J.E., G.M. Ylitalo, T.K. Rowles, K.D. Mullin, R.S. Wells, F.I. Townsend, R.W. Pearce, J.L. Bolton, E.S. Zolman, B.C. Balmer, and L.H. Schwacke. 2018. Persistent organic pollutants (POPs) in blood and blubber of common bottlenose dolphins (*Tursiops truncatus*) at three northern Gulf of Mexico sites following the Deepwater Horizon oil spill. Science of the Total Environment 621:130–137. doi: 10.1016/j.scitotenv.2017.11.209

Barros, N.B. and D.K. Odell. 1990. Food habits of bottlenose dolphins in the southeastern United States In: Leatherwood, S. and R.R. Reeves (Eds.), The Bottlenose Dolphin. Academic Press, San Diego, CA. pp. 309–328. doi: 10.1016/B978-0-12-440280-5.50020-2

Bassos-Hull, K., R.M. Perrtree, C.C. Shepard, S. Schilling, A.A. Barleycorn, J.B. Allen, B.C. Balmer, W.E. Pine, and R.S. Wells. 2013. Long-term site fidelity and seasonal abundance estimates of common bottlenose dolphins (*Tursiops truncatus*) along the southwest coast of Florida and responses to

natural perturbations. Journal of Cetacean Research and Management 13(1):19–30. doi: 10.47536/jcrm.v13i1.551

Berens McCabe, E.J., D.P. Gannon, N.B. Barros, and R.S. Wells. 2010. Prey selection by resident common bottlenose dolphins (*Tursiops truncatus*) in Sarasota Bay, Florida. Marine Biology 157(5):931–942. doi: 10.1007/s00227-009-1371-2

Bowen-Stevens, S.R., D.P. Gannon, R.A. Hazelkorn, G. Lovewell, K.M. Volker, S. Smith, M.C. Tumlin, and J. Litz. 2021. Diet of common bottlenose dolphins, *Tursiops truncatus*, that stranded in and near Barataria Bay, Louisiana, 2010–2012. Southeastern Naturalist 20(1). doi: 10.1656/058.020.0113

Bräger, S. 1993. Diurnal and seasonal behavior patterns of bottlenose dolphins (*Tursiops truncatus*). Marine Mammal Science 9:434–438. doi: 10.1111/j.1748-7692.1993.tb00477.x

Bräger, S., B. Würsig, A. Acevedo, and T. Henningsen. 1994. Association patterns of bottle-nosed dolphins (*Tursiops-truncatus*) in Galveston Bay, Texas. Journal of Mammalogy 75(2):431–437. doi: 10.2307/1382564

Connor, R.C., R.S. Wells, J. Mann, and A.J. Read. 2000. The bottlenose dolphin: Social relationships in a fission-fusion society. In: Mann, J., R. Connor, P. Tyack, and H. Whitehead (Eds.), Cetacean Societies: Field Studies of Dolphins and Whales. University of Chicago Press, Chicago, IL. pp. 91–126.

Damseaux, F., J.J. Kiszka, M.R. Heithaus, G. Scholl, G. Eppe, J.P. Thome, J. Lewis, W. Hao, M.C. Fontaine, and K. Das. 2017. Spatial variation in the accumulation of POPs and mercury in bottlenose dolphins of the Lower Florida Keys and the coastal Everglades (South Florida). Environmental Pollution 220(Pt A):577–587. doi: 10.1016/j.envpol.2016.10.005

Fernandez, S. and A.A. Hohn. 1998. Age, growth, and calving season of bottlenose dolphins, *Tursiops truncatus*, off coastal Texas. Fishery Bulletin 96(2):357–365.

Fertl, D. 1994. Occurrence patterns and behavior of bottlenose dolphins (*Tursiops truncatus*) in the Galveston ship channel, Texas. Texas Journal of Science 46(4):299–318.

Hayes, S.A., E. Josephson, K. Maze-Foley, P.E. Rosel, and J. Wallace. 2022. U.S. Atlantic and Gulf of Mexico marine mammal stock assessments 2021. National Oceanic and Atmospheric Administration, National Marine Fisheries Service, Northeast Fisheries Science Center: Woods Hole, MA. NOAA Technical Memorandum NMFS-NE-288. 380 p. doi: 10.25923/6tt7-kc16

Henderson, E.E. and B. Würsig. 2007. Behavior patterns of bottlenose dolphins in San Luis Pass, Texas. Gulf of Mexico Science 25(2):153–161. doi: 10.18785/goms.2502.06

Hohn, A.A., M.D. Scott, R.S. Wells, J.C. Sweeney, and A.B. Irvine. 1989. Growth layers in teeth from known-age, free-ranging bottlenose dolphins. Marine Mammal Science 5(4):315–342. doi: 10.1111/j.1748-7692.1989.tb00346.x

Hubard, C.W., K. Maze-Foley, K.D. Mullin, and W.W. Schroeder. 2004. Seasonal abundance and site fidelity of bottlenose dolphins (*Tursiops truncatus*) in Mississippi Sound. Aquatic Mammals 30(2):299–310. doi: 10.1578/am.30.2.2004.299

Irvine, A.B., M.D. Scott, R.S. Wells, and J.H. Kaufmann. 1981. Movements and activities of the Atlantic bottlenose dolphin, *Tursiops truncatus*, near Sarasota, Florida. Fishery Bulletin 79(4):671–688.

Irvine, B. and R.S. Wells. 1972. Results of attempts to tag Atlantic bottlenosed dolphins (*Tursiops truncatus*). Cetology 13:1–5.

Irwin, L.-J. and B. Würsig. 2004. A small resident community of bottlenose dolphins, *Tursiops truncatus*, in Texas: Monitoring recommendations. Gulf of Mexico Science 22(1):13–21. doi: 10.18785/goms.2201.02

LaBrecque, E., C. Curtice, J. Harrison, S.M. Van Parijs, and P.N. Halpin. 2015. 3. Biologically Important Areas for cetaceans within U.S. Waters – Gulf of Mexico Region. Aquatic Mammals 41(1):30–38. doi: 10.1578/am.41.1.2015.30

Leatherwood, S. 1977. Some preliminary impressions on the numbers and social behavior of free-swimming bottlenosed dolphin calves (*Tursiops truncatus*) in the northern Gulf of Mexico. In: Ridgway, S.H. and K. Bernirschke (Eds.), Breeding Dolphins; Present Status, Suggestions for the Future. Final report to the US Marine Mammal Commission, Contract MM6AC009, MMC-76/07, Washington, DC. pp. 143–167.

Litz, J.A., M.A. Baran, S.R. Bowen-Stevens, R.H. Carmichael, K.M. Colegrove, L.P. Garrison, S.E. Fire, E.M. Fougeres, R. Hardy, S. Holmes, W. Jones, B.E. Mase-Guthrie, D.K. Odell, P.E. Rosel, J.T. Saliki, D.K. Shannon, S.F. Shippee, S.M. Smith, E.M. Stratton, M.C. Tumlin, H.R. Whitehead, G.A. Worthy, and T.K. Rowles. 2014. Review of historical unusual mortality events (UMEs) in the Gulf of Mexico (1990–2009): providing context for the multi-year northern Gulf of Mexico cetacean UME declared in 2010. Diseases of Aquatic Organisms 112(2):161–175. doi: 10.3354/dao02807

Lynn, S.K. and B. Würsig. 2002. Summer movement patterns of bottlenose dolphins in a Texas bay. Gulf of Mexico Science 20(1):25–37. doi: 10.18785/goms.2001.03

Mackey, A.D. 2010. Site fidelity and association patterns of bottlenose dolphins (*Tursiops truncatus*) in the Mississippi Sound. University of Southern Mississippi. Thesis. 133 p.

Mann, J., R.C. Connor, L.M. Barre, and M.R. Heithaus. 2000. Female reproductive success in bottlenose dolphins (*Tursiops* sp.): life history, habitat, provisioning, and group-size effects. Behavioral Ecology 11(2):210–219. doi: 10.1093/beheco/11.2.210

Matich, P., J.S. Ault, R.E. Boucek, D.R. Bryan, K.R. Gastrich, C.L. Harvey, M.R. Heithaus, J.J. Kiszka, V. Paz, J.S. Rehage, and A.E. Rosenblatt. 2017. Ecological niche partitioning within a large predator guild in a nutrient-limited estuary. Limnology and Oceanography 62(3):934–953. doi: 10.1002/lno.10477

Mattson, M.C., K.D. Mullin, G.W. Ingram, and W. Hoggard. 2006. Age structure and growth of the bottlenose dolphin (*Tursiops truncatus*) from strandings in the Mississippi sound region of the north-central Gulf of Mexico from 1986 to 2003. Marine Mammal Science 22(3):654–666. doi: 10.1111/j.1748-7692.2006.00057.x

Maze, K.S. and B. Würsig. 1999. Bottlenosed dolphins of San Luis Pass, Texas: Occurrence patterns, site-fidelity, and habitat use. Aquatic Mammals 25:91–104.

McCormack, M.A., F. Battaglia, W.E. McFee, and J. Dutton. 2020a. Mercury concentrations in blubber and skin from stranded bottlenose dolphins (*Tursiops truncatus*) along the Florida and Louisiana coasts (Gulf of Mexico, USA) in relation to biological variables. Environmental Research 180:108886. doi: 10.1016/j.envres.2019.108886

McCormack, M.A., B.P. Jackson, and J. Dutton. 2020b. Relationship between mercury and selenium concentrations in tissues from stranded odontocetes in the northern Gulf of Mexico. Science of the Total Environment 749:141350. doi: 10.1016/j.scitotenv.2020.141350

McCormack, M.A., W.H. Nowlin, and J. Dutton. 2022. Effect of trophic position on mercury concentrations in bottlenose dolphins (*Tursiops truncatus*) from the northern Gulf of Mexico. Environmental Research 204(Pt B):112124. doi: 10.1016/j.envres.2021.112124

McDonald, T.L., F.E. Hornsby, T.R. Speakman, E.S. Zolman, K.D. Mullin, C. Sinclair, P.E. Rosel, L. Thomas, and L.H. Schwacke. 2017. Survival, density, and abundance of common bottlenose dolphins in Barataria Bay (USA) following the Deepwater Horizon oil spill. Endangered Species Research 33:193–209. doi: 10.3354/esr00806

McHugh, M.B. 1989. Population numbers and feeding behavior of the Atlantic bottlenose dolphin (*Tursiops truncatus*) near Aransas Pass, Texas. University of Texas at Austin. Thesis. 96 p.

Mead, J.G. and C.W. Potter. 1990. Natural history of bottlenose dolphins along the central Atlantic coast of the United States. In: Leatherwood, S. and R.R. Reeves (Eds.), The Bottlenose Dolphin. Academic Press, San Diego, CA. pp. 165–195.

Miller, C.E. 2003. Abundance trends and environmental habitat usage patterns of bottlenose dolphins (*Tursiops truncatus*) in lower Barataria and Caminada bays, Louisiana. Louisiana State University and Agricultural & Mechanical College. Dissertation. 125 p.

Miller, L.J., A.D. Mackey, T. Hoffland, M. Solangi, and S.A. Kuczaj. 2010. Potential effects of a major hurricane on Atlantic bottlenose dolphin (*Tursiops truncatus*) reproduction in the Mississippi Sound. Marine Mammal Science 26(3):707–715. doi: 10.1111/j.1748-7692.2010.00371.x

Miller, L.J., A.D. Mackey, M. Solangi, and S.A. Kuczaj. 2013. Population abundance and habitat utilization of bottlenose dolphins in the Mississippi Sound. Aquatic Conservation: Marine and Freshwater Ecosystems 23(1):145–151. doi: 10.1002/aqc.2278

Mullin, K.D., T. McDonald, R.S. Wells, B.C. Balmer, T. Speakman, C. Sinclair, E.S. Zolman, F. Hornsby, S.M. McBride, K.A. Wilkinson, and L.H. Schwacke. 2017. Density, abundance, survival, and ranging patterns of common bottlenose dolphins (*Tursiops truncatus*) in Mississippi Sound following the Deepwater Horizon oil spill. PLoS ONE 12(10):e0186265. doi: 10.1371/journal.pone.0186265

Perrin, W.F. and S.B. Reilly. 1984. Reproductive parameters of dolphins and small whales of the family Delphinidae. In: Perrin, W.F., R.L. Brownell, Jr., and D.P. DeMaster (Eds.), Reproduction in Whales, Dolphins, and Porpoises. pp. 97–134.

Rosel, P.E., L.A. Wilcox, C. Sinclair, T.R. Speakman, M.C. Tumlin, J.A. Litz, and E.S. Zolman. 2017. Genetic assignment to stock of stranded common bottlenose dolphins in southeastern Louisiana after the Deepwater Horizon oil spill. Endangered Species Research 33:221–234. doi: 10.3354/esr00780

Rossman, S., P.H. Ostrom, M. Stolen, N.B. Barros, H. Gandhi, C.A. Stricker, and R.S. Wells. 2015. Individual specialization in the foraging habits of female bottlenose dolphins living in a trophically diverse and habitat rich estuary. Oecologia 178(2):415–425. doi: 10.1007/s00442-015-3241-6

Sarabia, R.E., M.R. Heithaus, and J.J. Kiszka. 2018. Spatial and temporal variation in abundance, group size and behaviour of bottlenose dolphins in the Florida coastal Everglades. Journal of the Marine Biological Association of the United Kingdom 98(5):1097–1107. doi: 10.1017/s002531541700090x

Scott, M.D. and S.J. Chivers. 1990. Distribution and herd structure of bottlenose dolphins in the eastern tropical Pacific Ocean. In: Leatherwood, S. and R.R. Reeves (Eds.), The Bottlenose Dolphin. Academic Press, San Diego, CA. pp. 387–402.

Sellas, A.B., R.S. Wells, and P.E. Rosel. 2005. Mitochondrial and nuclear DNA analyses reveal fine scale geographic structure in bottlenose dolphins (*Tursiops truncatus*) in the Gulf of Mexico. Conservation Genetics 6(5):715–728. doi: 10.1007/s10592-005-9031-7

Sergeant, D.E., D.K. Caldwell, and M.C. Caldwell. 1973. Age, growth, and maturity of bottlenosed dolphin (*Tursiops-truncatus*) from northeast Florida. Journal of the Fisheries Research Board of Canada 30(7):1009–1011. doi: 10.1139/f73-165

Shane, S.H. 1990. Behavior and ecology of the bottlenose dolphin at Sanibel Island, Florida. In: Leatherwood, S. and R.R. Reeves (Eds.), The Bottlenose Dolphin. Academic Press, San Diego, CA. pp. 245–265. doi: 10.1016/B978-0-12-440280-5.50016-0

Shane, S.H. 2004. Residence patterns, group characteristics, and association patterns of bottlenose dolphins near Sanibel Island, Florida. Gulf of Mexico Science 22(1):1–12. doi: 10.18785/goms.2201.01

Shane, S.H., R.S. Wells, and B. Würsig. 1986. Ecology, behavior and social organization of the bottlenose dolphin: A review. Marine Mammal Science 2:34–63. doi: 10.1111/j.1748-7692.1986.tb00026.x

Takeshita, R., B.C. Balmer, F. Messina, E.S. Zolman, L. Thomas, R.S. Wells, C.R. Smith, T.K. Rowles, and L.H. Schwacke. 2021. High site-fidelity in common bottlenose dolphins despite low salinity

exposure and associated indicators of compromised health. PLoS ONE 16(9):e0258031. doi: 10.1371/journal.pone.0258031

Taylor, B.L., S.J. Chivers, J. Larese, and W.F. Perrin. 2007. Generation length and percent mature estimates for IUCN assessments of cetaceans. National Oceanic and Atmospheric Administration, National Marine Fisheries Service, Southwest Fisheries Science Center: La Jolla, CA. Administrative Report LJ-07-01. 24 p.

Toms, C. 2019. Filling the gaps: Common bottlenose dolphin (*Tursiops truncatus*) population dynamics, structure, and connectivity within Florida panhandle bays, sounds, and estuaries. University of Central Florida. Dissertation. 373 p.

Torres, L.G. and A.J. Read. 2009. Where to catch a fish? The influence of foraging tactics on the ecology of bottlenose dolphins (*Tursiops truncatus*) in Florida Bay, Florida. Marine Mammal Science 25(4):797–815. doi: 10.1111/j.1748-7692.2009.00297.x

Tyson, R.B., S.M. Nowacek, and D.P. Nowacek. 2011. Community structure and abundance of bottlenose dolphins *Tursiops truncatus* in coastal waters of the northeast Gulf of Mexico. Marine Ecology Progress Series 438:253–265. doi: 10.3354/meps09292

Urian, K.W., S. Hofmann, R.S. Wells, and A.J. Read. 2009. Fine-scale population structure of bottlenose dolphins (*Tursiops truncatus*) in Tampa Bay, Florida. Marine Mammal Science 25(3):619–638. doi: 10.1111/j.1748-7692.2009.00284.x

Vollmer, N.L. and P.E. Rosel. 2013. A review of common bottlenose dolphins (*Tursiops truncatus truncatus*) in the Northern Gulf of Mexico: Population biology, potential threats, and management. Southeastern Naturalist 12(m6):1–43. doi: 10.1656/058.012.m601

Weller, D.W. 1998. Global and regional variation in the biology and behavior of bottlenose dolphins. Texas A&M University. Dissertation. 142 p.

Wells, R.S. 1986a. Population structure of bottlenose dolphins: Behavioral studies along the central west coast of Florida. Contract Report to National Marine Fisheries Service, Southeast Fisheries Center. Contract No. 45-WCNF-5-003BB. 58 p.

Wells, R.S. 1986b. Structural aspects of dolphin societies. University of California, Santa Cruz. Dissertation. 234 p.

Wells, R.S. 1991. The role of long-term study in understanding the social structure of a bottlenose dolphin community. In: Pryor, K. and K. Norris (Eds.), Dolphin Societies: Discoveries and Puzzles. University of California Press, Berkeley, CA. pp. 199–226.

Wells, R.S. 2000. Reproduction in wild bottlenose dolphins: Overview of patterns observed during a long-term study. In: Duffield, D. and T. Robeck (Eds.), Bottlenose Dolphin Reproduction Workshop. AZA Marine Mammal Taxon Advisory Group, Silver Spring, MD. pp. 57–74.

Wells, R.S. 2003. Dolphin social complexity: Lessons from long-term study and life history. In: de Waal, F.B.M. and P.L. Tyack (Eds.), Animal Social Complexity: Intelligence, Culture, and Individualized Societies Harvard University Press, Cambridge, MA. pp. 32–56.

Wells, R.S. 2014. Social structure and life history of bottlenose dolphins near Sarasota Bay, Florida: Insights from four decades and five generations. Primates and cetaceans. pp. 149–172. doi: 10.1007/978-4-431-54523-1_8

Wells, R.S., M.K. Bassos, K.W. Urian, W.J. Carr, and M.D. Scott. 1996. Low-level monitoring of bottlenose dolphins, *Tursiops truncatus*, in Charlotte Harbor, Florida: 1990-1994. National Oceanic and Atmospheric Administration, National Marine Fisheries Service, Southeast Fisheries Science Center: Miami, FL. NOAA Technical Memorandum NMFS-SEFSC-384. 86 p.

Wells, R.S., M.K. Bassos, K.W. Urian, S.H. Shane, E.C.G. Owen, C.F. Weiss, W.J. Carr, and M.D. Scott. 1997. Low-level monitoring of bottlenose dolphins, *Tursiops truncatus*, in Pine Island Sound, Florida: 1996. National Oceanic and Atmospheric Administration, National Marine Fisheries

Service, Southeast Fisheries Science Center: Miami, FL. Southeast Fisheries Center Contribution No. 40-WCNF601958. 86 p.

Wells, R.S., A. Natoli, and G. Braulik. 2019. *Tursiops truncatus* (errata version published in 2019). *The IUCN Red List of Threatened Species* 2019: e.T22563A156932432. https://dx.doi.org/10.2305/IUCN.UK.2019-1.RLTS.T22563A156932432.en

Wells, R.S., L.H. Schwacke, T.K. Rowles, B.C. Balmer, E. Zolman, T. Speakman, F.I. Townsend, M.C. Tumlin, A. Barleycorn, and K.A. Wilkinson. 2017. Ranging patterns of common bottlenose dolphins *Tursiops truncatus* in Barataria Bay, Louisiana, following the Deepwater Horizon oil spill. Endangered Species Research 33:159–180. doi: 10.3354/esr00732

Wells, R.S. and M.D. Scott. 1999. Bottlenose dolphin *Tursiops truncatus* (Montagu, 1821). In: Ridgway, S.H. and R. Harrison (Eds.), Handbook of marine mammals. Volume 6: The second book of dolphins and the porpoises. Academic Press, San Diego, CA. pp. 137–182.

Wells, R.S. and M.D. Scott. 2018. Bottlenose dolphin, *Tursiops truncatus*, common bottlenose dolphin. In: Würsig, B., J.G.M. Thewissen, and K.M. Kovacs (Eds.), Encyclopedia of Marine Mammals (Third Edition). Academic Press, London, UK. pp. 118–125. doi: 10.1016/B978-0-12-804327-1.00072-8

Wells, R.S., M.D. Scott, and A.B. Irvine. 1987. The social structure of free-ranging bottlenose dolphins. In: Genoways, H.H. (Ed.), Current Mammalogy. Plenum Press, New York, NY. pp. 247–305.

Wells, R.S., K.W. Urian, A.J. Read, M.K. Bassos, W.J. Carr, and M.D. Scott. 1996. Low-level monitoring of bottlenose dolphins, *Tursiops truncatus*, in Tampa Bay, Florida: 1988-1993. National Oceanic and Atmospheric Administration, National Marine Fisheries Service, Southeast Fisheries Science Center: Miami, FL. NOAA Technical Memorandum NMFS-SEFSC-385. 36 p.

Wilson, R.M., R.B. Tyson, J.A. Nelson, B.C. Balmer, J.P. Chanton, and D.P. Nowacek. 2017. Niche differentiation and prey selectivity among common bottlenose dolphins (*Tursiops truncatus*) sighted in St. George Sound, Gulf of Mexico. Frontiers in Marine Science 4:235. doi: 10.3389/fmars.2017.00235

Common bottlenose dolphin − *Tursiops truncatus*

Gulf of Mexico, Continental Shelf Stock

Overall Vulnerability Rank = High

Biological Sensitivity = Moderate

Climate Exposure = Very High

Data Quality = 89% of scores ≥ 2

	Tursiops truncatus	Expert Scores	Data Quality
Sensitivity attributes	Prey/Diet Specificity	2.1	2.0
	Habitat Specificity	2.3	2.3
	Site Fidelity	2.3	2.7
	Lifetime Reproductive Potential	1.3	2.0
	Generation Length	2.7	2.0
	Reproductive Plasticity	1.3	1.7
	Migration	2.8	2.3
	Home Range	2.3	2.3
	Stock Abundance	1.1	3.0
	Stock Abundance Trend	2.1	1.3
	Cumulative Stressors	2.7	3.0
	Sensitivity Score	Moderate	
Exposure variables	Sea Surface Temperature (Change in mean)	4.0	3.0
	Sea Surface Temperature (Change in variability)	1.0	3.0
	Air Temperature (Change in mean)	4.0	3.0
	Air Temperature (Change in variability)	1.0	3.0
	Precipitation (Change in mean)	1.0	3.0
	Precipitation (Change in variability)	1.0	3.0
	Sea Surface Salinity (Change in mean)	2.4	2.7
	Sea Surface Salinity (Change in variability)	1.2	2.7
	Ocean pH (Change in mean)	4.0	3.0
	Ocean pH (Change in variability)	1.0	3.0
	Sea ice coverage (Change in mean)	1.0	3.0
	Sea ice coverage (Change in variability)	1.0	3.0
	Dissolved oxygen (Change in mean)	4.0	3.0
	Dissolved oxygen (Change in variability)	1.0	3.0
	Circulation	2.9	1.7
	Sea level rise	2.7	3.0
	Exposure Score	Very High	
Overall Vulnerability Rank		High	

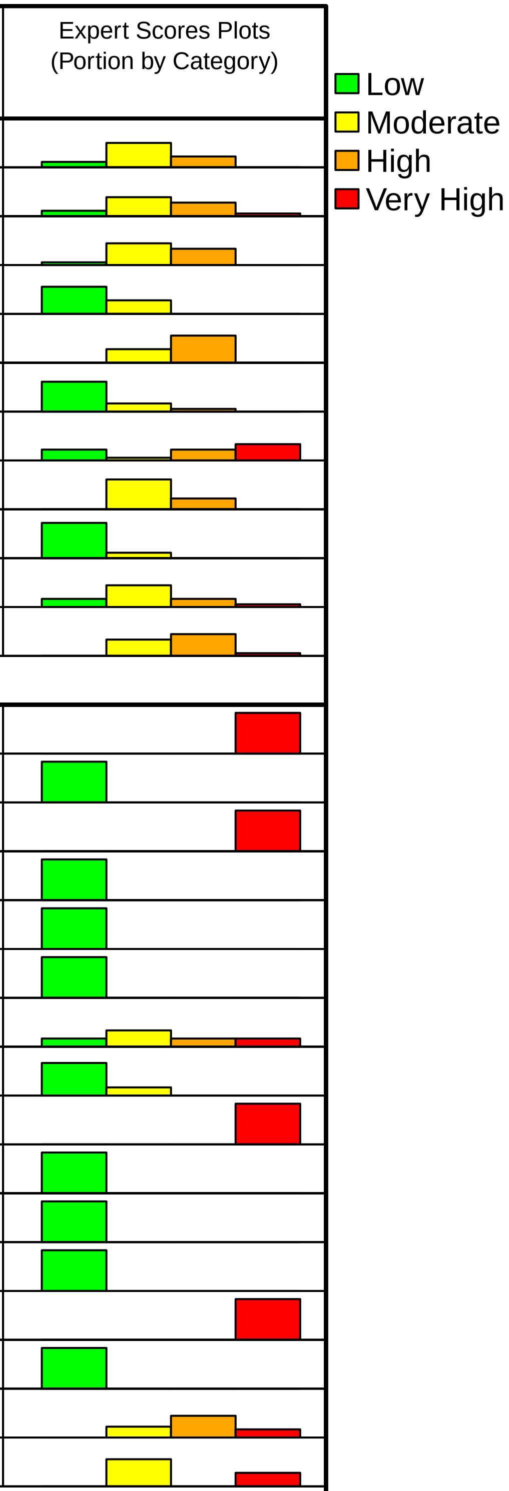

Common bottlenose dolphin (Gulf of Mexico, Continental Shelf Stock)

Tursiops truncatus

CVA Results Summary

Overall Climate Vulnerability Rank: High (96% certainty from bootstrap analysis).

Climate Exposure: Very High. Four exposure factors scored greater than 3.5: Air Temperature (Standard anomaly) (4.00), Dissolved oxygen (Standard anomaly) (4.00), Ocean pH (Standard anomaly) (4.00), and Sea Surface Temperature (Standard anomaly) (4.00).

Biological Sensitivity: Moderate. Three sensitivity attributes scored greater than 2.5: Migration (2.80), Cumulative Stressors (2.67), and Generation Time (2.67).

Distributional Response: Moderate

Abundance Response: Moderate

Phenology Response: Moderate

Data Quality: 89% of the data quality scores were 2 or greater, 82% of sensitivity attribute data quality scores were 2 or higher.

Background Information

Stock Description

This stock includes common bottlenose dolphins in waters from 20 to 200 m deep the northern Gulf of Mexico from the U.S.-Mexican border to the Florida Keys and adjacent Mexican and Cuban territorial waters (Hayes et al. 2022). The stock includes genetically distinct “coastal” and “offshore” ecotypes of bottlenose dolphins, but consists primarily of the coastal ecotype (Hoelzel et al. 1998; Vollmer and Rosel 2017).

Prey/Diet Specificity

Generally, common bottlenose dolphins consume a large variety of fish and/or squid (Barros and Odell 1990; Mead and Potter 1990; Wells and Scott 2018). Common bottlenose dolphins seem to show a consistent preference for sound-producing fishes (e.g., sciaenids, toadfish; Berens McCabe et al. 2010; Bowen-Stevens et al. 2021). In Sarasota Bay, Florida, Rossman et al. (2015) found some individual common bottlenose dolphins to show diet specialization despite the broader study group showing a generalist diet. Specialization can be influenced by locally abundant prey species (Shane et al. 1986; Vollmer and Rosel 2013) or can result from resource partitioning amongst sympatric populations (e.g., Torres and Read 2009; Wilson et al. 2017).

Habitat Specificity

Common bottlenose dolphins are found in a variety of marine and estuarine habitats (Wells and Scott 2018).

Site Fidelity

No specific site fidelity information was found for this stock. However, common bottlenose dolphins in Gulf of Mexico bay, sound, and estuary (BSE) stocks show site fidelity (LaBrecque et al. 2015; Hayes et al. 2019), such as in Matagorda Bay, Espiritu Santo Bay, and San Luis Pass in Texas (Maze and Würsig 1999; Lynn and Würsig 2002; Irwin and Würsig 2004); Barataria Bay in Louisiana (Miller 2003; Wells et al. 2017; Takeshita et al. 2021); Mississippi Sound in Mississippi (Hubard et al. 2004; Mackey 2010; Mullin et al. 2017); Charlotte Harbor and Pine Island Sound (Bassos-Hull et al. 2013), Sarasota Bay (Wells 1986; Wells 1991; Wells 2014), St. Andrew Bay (Balmer et al. 2019), St. Joseph Bay (Balmer et al. 2008; Balmer et al. 2018), St. Vincent Sound and Apalachicola Bay (Tyson et al. 2011), and Tampa Bay in Florida (Wells et al. 1996; Urian et al. 2009).

Lifetime Reproductive Potential

Taylor et al. (2007) reported the interbirth interval of common bottlenose dolphins as 3.80 years, though other studies have shown it to be closer to three years (Leatherwood 1977; Wells and Scott 1999; Connor et al. 2000; Wells 2003).

Generally, females reach sexual maturity before males, with females reaching sexual maturity at 5–13 years (Sergeant et al. 1973; Perrin and Reilly 1984; Wells et al. 1987; Mead and Potter 1990; Mann et al. 2000; Wells 2000; Wells and Scott 2018).

Females reproduce through most of their adult lives (Leatherwood 1977; Hohn et al. 1989; Wells and Scott 1999; Wells 2000; Wells 2003), but may produce fewer calves after they reach 30 years of age (Wells 2000). Sarasota Bay common bottlenose dolphins can give birth when they are as young as 6 years of age, and can continue until they are 48 years old (Wells and Scott 2018). Reproductive lifespan would therefore be up to 42 years based on long-term observation studies (Wells 2014; Wells and Scott 2018).

Longitudinal studies from Sarasota Bay, Florida have found that female common bottlenose dolphins can live up to 67 years (Wells and Scott 2018). Studies based on growth layer groups in teeth suggest female common bottlenose dolphins can live to 41 years along the Texas coast (Fernandez and Hohn 1998) and to 30 years along the Mississippi coast (Mattson et al. 2006).

Generation Length

Taylor et al. (2007) reported a common bottlenose dolphin generation length of 20.6–21.1 years.

Reproductive Plasticity

Common bottlenose dolphin calving is generally diffusely seasonal, with one or more peaks, although births have been reported from all seasons (Vollmer and Rosel 2013; Wells and Scott 2018). Calf sightings peak in the spring and summer months in Florida (Wells et al. 1987), Mississippi/Louisiana (Miller et al. 2010; Miller et al. 2013), and Texas (McHugh 1989; Henderson and Würsig 2007).

Migration

Data are insufficient to characterize migration patterns (or the lack thereof) for this stock. However, genetic data do support the presence of multiple differentiated populations of common bottlenose dolphins in coastal and offshore waters with very low levels of migration among them (Vollmer and Rosel 2017). Molecular genetic data further support that coastal populations are genetically differentiated from BSE populations (Sellas et al. 2005; Rosel et al. 2017). In waters of the West Florida Shelf, acoustic and visual sighting data suggest seasonal movement from deeper waters in the winter and spring into shallower waters during the summer and fall (Simard et al. 2015).

Home Range

Data are insufficient to characterize home range patterns (or the lack thereof) for this stock.

Stock Abundance

This stock is estimated at 63,280 individuals (CV=0.10; Garrison et al. 2021; Hayes et al. 2022) based on summer 2017 and fall 2018 aerial surveys. Previously, this stock was estimated at 51,192 individuals (CV=0.10) based on seasonal abundance estimates from spring 2011, summer 2011, fall 2011 and winter 2012 aerial surveys (Waring et al. 2016).

Stock Abundance Trend

Data are insufficient to complete an abundance trend analysis for this stock (Hayes et al. 2022).

Cumulative Stressors

Common bottlenose dolphins face a wide range of individual and cumulative natural and anthropogenic threats (see reviews by Vollmer and Rosel 2013; Wells and Scott 2018; and Wells et al. 2019). These include such threats as shark predation, harmful algal blooms, recreational and commercial fishing gear, vessel traffic, pollution, habitat alteration, and provisioning.

Common bottlenose dolphins in the northern Gulf of Mexico were exposed to oil and dispersants following the Deepwater Horizon oil spill, which may result in long term reproductive and chronic health effects (NOAA 2011; Michel et al. 2013; Helm et al. 2015; DWH NRDA 2016; Lane et al. 2015; Aichinger Dias et al. 2017; De Guise et al. 2017, 2021; Kellar et al. 2017; Smith et al. 2017; Schwacke et al. 2017). An Unusual Mortality Event (UME), involving primarily bottlenose dolphins, was declared for cetaceans in the northern Gulf of Mexico from 2010 to 2014, with the Deepwater Horizon oil spill as the primary underlying cause (Litz et al. 2014; Schwacke et al. 2014; Venn-Watson et al. 2015a, 2015b). A UME was declared in 1990 for the Gulf of Mexico from Florida to Texas, with more than 300 common bottlenose dolphins stranding due to an undetermined cause, although morbillivirus and cold temperatures may have contributed (Litz et al. 2014).

The northern Gulf of Mexico continental shelf common bottlenose dolphin stock potentially interacts with four commercial fisheries (shrimp trawl commercial fishery; shark bottom longline/hook-and-line; snapper-grouper and other reef fish; and commercial passenger fishing vessel/hook-and-line; Hayes et al. 2022).

Distribution and Sightings

Density model results for common bottlenose dolphins in the Gulf of Mexico are presented by Roberts et al. (2015, 2016) and Mannocci et al. (2017).

Further Reading

Hayes, S.A., E. Josephson, K. Maze-Foley, P.E. Rosel, and J. Wallace. 2022. U.S. Atlantic and Gulf of Mexico marine mammal stock assessments 2021. National Oceanic and Atmospheric Administration, National Marine Fisheries Service, Northeast Fisheries Science Center: Woods Hole, MA. NOAA Technical Memorandum NMFS-NE-288. 380 p. doi: 10.25923/6tt7-kc16

Wells, R. S. and M. D. Scott. 2018. Bottlenose Dolphin, *Tursiops truncatus*, Common Bottlenose Dolphin. In: Würsig, B., Thewissen, J.G.M. and Kovacs, K.M. eds. Encyclopedia of marine mammals. Academic Press. Pages 118-125

Wells, R.S., A. Natoli, and G. Braulik. 2019. *Tursiops truncatus* (errata version published in 2019). The IUCN Red List of Threatened Species 2019: e.T22563A156932432. https://dx.doi.org/10.2305/IUCN.UK.2019-1.RLTS.T22563A156932432.en

References

Aichinger Dias, L., J. Litz, L. Garrison, A. Martinez, K. Barry, and T. Speakman. 2017. Exposure of cetaceans to petroleum products following the Deepwater Horizon oil spill in the Gulf of Mexico. Endangered Species Research 33:119–125. doi: 10.3354/esr00770

Balmer, B., T. McDonald, F. Hornsby, J. Adams, J. Allen, A. Barleycorn, and L. Schwacke. 2018. Long-term trends in a northern Gulf of Mexico common bottlenose dolphin (*Tursiops truncatus*) population in the wake of the Deepwater Horizon oil spill. Journal of Cetacean Research and Management 18:1–9. doi: 10.47536/jcrm.v18i1.409

Balmer, B., S. Watwood, B. Quigley, T. Speakman, K. Barry, K. Mullin, P. Rosel, C. Sinclair, E. Zolman, and L. Schwacke. 2019. Common bottlenose dolphin (*Tursiops truncatus*) abundance and distribution patterns in St. Andrew Bay, Florida, USA. Aquatic Conservation: Marine and Freshwater Ecosystems 29(3):486–498. doi: 10.1002/aqc.3001

Balmer, B.C., R.S. Wells, S.M. Nowacek, D.P. Nowacek, L.H. Schwacke, W.A. McLellan, F.S. Scharf, T.K. Rowles, L.J. Hansen, T.R. Spradlin, and D.A. Pabst. 2008. Seasonal abundance and distribution patterns of common bottlenose dolphins (*Tursiops truncatus*) near St. Joseph Bay, Florida, USA. Journal of Cetacean Research and Management 10(2):157–167.

Barros, N.B. and D.K. Odell. 1990. Food habits of bottlenose dolphins in the southeastern United States In: Leatherwood, S. and R.R. Reeves (Eds.), The Bottlenose Dolphin. Academic Press, San Diego, CA. pp. 309–328. doi: 10.1016/B978-0-12-440280-5.50020-2

Bassos-Hull, K., R.M. Perrtree, C.C. Shepard, S. Schilling, A.A. Barleycorn, J.B. Allen, B.C. Balmer, W.E. Pine, and R.S. Wells. 2013. Long-term site fidelity and seasonal abundance estimates of common bottlenose dolphins (*Tursiops truncatus*) along the southwest coast of Florida and responses to natural perturbations. Journal of Cetacean Research and Management 13(1):19–30. doi: 10.47536/jcrm.v13i1.551

Berens McCabe, E.J., D.P. Gannon, N.B. Barros, and R.S. Wells. 2010. Prey selection by resident common bottlenose dolphins (*Tursiops truncatus*) in Sarasota Bay, Florida. Marine Biology 157(5):931–942. doi: 10.1007/s00227-009-1371-2

Bowen-Stevens, S.R., D.P. Gannon, R.A. Hazelkorn, G. Lovewell, K.M. Volker, S. Smith, M.C. Tumlin, and J. Litz. 2021. Diet of common bottlenose dolphins, *Tursiops truncatus*, that stranded in and near Barataria Bay, Louisiana, 2010–2012. Southeastern Naturalist 20(1). doi: 10.1656/058.020.0113

Connor, R.C., R.S. Wells, J. Mann, and A.J. Read. 2000. The bottlenose dolphin: Social relationships in a fission-fusion society. In: Mann, J., R. Connor, P. Tyack, and H. Whitehead (Eds.), Cetacean Societies: Field Studies of Dolphins and Whales. University of Chicago Press, Chicago, IL. pp. 91–126.

De Guise, S., M. Levin, E. Gebhard, L. Jasperse, L. Burdett Hart, C.R. Smith, S. Venn-Watson, F. Townsend, R. Wells, B. Balmer, E. Zolman, T. Rowles, and L. Schwacke. 2017. Changes in immune functions in bottlenose dolphins in the northern Gulf of Mexico associated with the Deepwater Horizon oil spill. Endangered Species Research 33:291–303. doi: 10.3354/esr00814

De Guise, S., M. Levin, L. Jasperse, J. Herrman, R.S. Wells, T. Rowles, and L. Schwacke. 2021. Long-term immunological alterations in bottlenose dolphin a decade after the Deepwater Horizon oil spill in the northern Gulf of Mexico: Potential for multigenerational effects. Environmental Toxicology and Chemistry 40(5):1308–1321. doi: 10.1002/etc.4980

Deepwater Horizon Natural Resource Damage Assessment Trustees (DWH NRDA). 2016. Deepwater Horizon oil spill: Final Programmatic Damage Assessment and Restoration Plan and Final Programmatic Environmental Impact Statement. Retrieved from: http://www.gulfspillrestoration.noaa.gov/restoration-planning/gulf-plan.1659.

Fernandez, S. and A.A. Hohn. 1998. Age, growth, and calving season of bottlenose dolphins, *Tursiops truncatus*, off coastal Texas. Fishery Bulletin 96(2):357–365.

Garrison, L.P., J. Ortega-Ortiz, and G. Rappucci. 2021. Abundance of coastal and continental shelf stocks of common bottlenose and Atlantic spotted dolphins in the northern Gulf of Mexico: 2017–2018. National Oceanic and Atmospheric Administration, National Marine Fisheries Service, Southeast Fisheries Science Center, Protected Resources and Biodiversity Division: Miami, FL. PRD-2021-01. 25 p. doi: 10.25923/vk95-t881

Hayes, S.A., E. Josephson, K. Maze-Foley, and P.E. Rosel. 2019. US Atlantic and Gulf of Mexico marine mammal stock assessments - 2018. National Oceanic and Atmospheric Administration, National Marine Fisheries Service, Northeast Fisheries Science Center: Woods Hole, MA. NOAA Technical Memorandum NMFS-NE-258. 298 p. doi: 10.25923/9rrd-tx13

Hayes, S.A., E. Josephson, K. Maze-Foley, P.E. Rosel, and J. Wallace. 2022. U.S. Atlantic and Gulf of Mexico marine mammal stock assessments 2021. National Oceanic and Atmospheric Administration, National Marine Fisheries Service, Northeast Fisheries Science Center: Woods Hole, MA. NOAA Technical Memorandum NMFS-NE-288. 380 p. doi: 10.25923/6tt7-kc16

Helm, R.C., D.P. Costa, T.D. DeBruyn, T.J. O'Shea, R.S. Wells, and T.M. Williams. 2015. Overview of effects of oil spills on marine mammals. In: Fingas, M. (Ed.), Handbook of oil spill science and technology, First edition. John Wiley & Sons, Inc., Hoboken, NJ. pp. 455–475.

Henderson, E.E. and B. Würsig. 2007. Behavior patterns of bottlenose dolphins in San Luis Pass, Texas. Gulf of Mexico Science 25(2):153–161. doi: 10.18785/goms.2502.06

Hoelzel, A.R., C.W. Potter, and P.B. Best. 1998. Genetic differentiation between parapatric 'nearshore' and 'offshore' populations of the bottlenose dolphin. Proceedings of the Royal Society of London. Series B: Biological Sciences 265(1402):1177–1183. doi: 10.1098/rspb.1998.0416

Hohn, A.A., M.D. Scott, R.S. Wells, J.C. Sweeney, and A.B. Irvine. 1989. Growth layers in teeth from known-age, free-ranging bottlenose dolphins. Marine Mammal Science 5(4):315–342. doi: 10.1111/j.1748-7692.1989.tb00346.x

Hubard, C.W., K. Maze-Foley, K.D. Mullin, and W.W. Schroeder. 2004. Seasonal abundance and site fidelity of bottlenose dolphins (*Tursiops truncatus*) in Mississippi Sound. Aquatic Mammals 30(2):299–310. doi: 10.1578/am.30.2.2004.299

Irwin, L.-J. and B. Würsig. 2004. A small resident community of bottlenose dolphins, *Tursiops truncatus*, in Texas: Monitoring recommendations. Gulf of Mexico Science 22(1):13–21. doi: 10.18785/goms.2201.02

Kellar, N.M., T.R. Speakman, C.R. Smith, S.M. Lane, B.C. Balmer, M.L. Trego, K.N. Catelani, M.N. Robbins, C.D. Allen, R.S. Wells, E.S. Zolman, T.K. Rowles, and L.H. Schwacke. 2017. Low reproductive success rates of common bottlenose dolphins *Tursiops truncatus* in the northern Gulf of Mexico following the Deepwater Horizon disaster (2010-2015). Endangered Species Research 33:143–158. doi: 10.3354/esr00775

LaBrecque, E., C. Curtice, J. Harrison, S.M. Van Parijs, and P.N. Halpin. 2015. 3. Biologically Important Areas for cetaceans within U.S. Waters – Gulf of Mexico Region. Aquatic Mammals 41(1):30–38. doi: 10.1578/am.41.1.2015.30

Lane, S.M., C.R. Smith, J. Mitchell, B.C. Balmer, K.P. Barry, T. McDonald, C.S. Mori, P.E. Rosel, T.K. Rowles, T.R. Speakman, F.I. Townsend, M.C. Tumlin, R.S. Wells, E.S. Zolman, and L.H. Schwacke. 2015. Reproductive outcome and survival of common bottlenose dolphins sampled in Barataria Bay, Louisiana, USA, following the Deepwater Horizon oil spill. Proceedings of the Royal Society B: Biological Sciences 282(1818):20151944. doi: 10.1098/rspb.2015.1944

Leatherwood, S. 1977. Some preliminary impressions on the numbers and social behavior of free-swimming bottlenosed dolphin calves (*Tursiops truncatus*) in the northern Gulf of Mexico. In: Ridgway, S.H. and K. Bernirschke (Eds.), Breeding Dolphins; Present Status, Suggestions for the Future. Final report to the US Marine Mammal Commission, Contract MM6AC009, MMC-76/07, Washington, DC. pp. 143–167.

Litz, J.A., M.A. Baran, S.R. Bowen-Stevens, R.H. Carmichael, K.M. Colegrove, L.P. Garrison, S.E. Fire, E.M. Fougeres, R. Hardy, S. Holmes, W. Jones, B.E. Mase-Guthrie, D.K. Odell, P.E. Rosel, J.T. Saliki, D.K. Shannon, S.F. Shippee, S.M. Smith, E.M. Stratton, M.C. Tumlin, H.R. Whitehead, G.A. Worthy, and T.K. Rowles. 2014. Review of historical unusual mortality events (UMEs) in the Gulf of Mexico (1990–2009): providing context for the multi-year northern Gulf of Mexico cetacean UME declared in 2010. Diseases of Aquatic Organisms 112(2):161–175. doi: 10.3354/dao02807

Lynn, S.K. and B. Würsig. 2002. Summer movement patterns of bottlenose dolphins in a Texas bay. Gulf of Mexico Science 20(1):25–37. doi: 10.18785/goms.2001.03

Mackey, A.D. 2010. Site fidelity and association patterns of bottlenose dolphins (*Tursiops truncatus*) in the Mississippi Sound. University of Southern Mississippi. Thesis. 133 p.

Mann, J., R.C. Connor, L.M. Barre, and M.R. Heithaus. 2000. Female reproductive success in bottlenose dolphins (*Tursiops* sp.): life history, habitat, provisioning, and group-size effects. Behavioral Ecology 11(2):210–219. doi: 10.1093/beheco/11.2.210

Mannocci, L., J.J. Roberts, D.L. Miller, and P.N. Halpin. 2017. Extrapolating cetacean densities to quantitatively assess human impacts on populations in the high seas. Conservation Biology 31(3):601–614. doi: 10.1111/cobi.12856

Mattson, M.C., K.D. Mullin, G.W. Ingram, and W. Hoggard. 2006. Age structure and growth of the bottlenose dolphin (*Tursiops truncatus*) from strandings in the Mississippi sound region of the north-central Gulf of Mexico from 1986 to 2003. Marine Mammal Science 22(3):654–666. doi: 10.1111/j.1748-7692.2006.00057.x

Maze, K.S. and B. Würsig. 1999. Bottlenosed dolphins of San Luis Pass, Texas: Occurrence patterns, site-fidelity, and habitat use. Aquatic Mammals 25:91–104.

McHugh, M.B. 1989. Population numbers and feeding behavior of the Atlantic bottlenose dolphin (*Tursiops truncatus*) near Aransas Pass, Texas. University of Texas at Austin. Thesis. 96 p.

Mead, J.G. and C.W. Potter. 1990. Natural history of bottlenose dolphins along the central Atlantic coast of the United States. In: Leatherwood, S. and R.R. Reeves (Eds.), The Bottlenose Dolphin. Academic Press, San Diego, CA. pp. 165–195.

Michel, J., E.H. Owens, S. Zengel, A. Graham, Z. Nixon, T. Allard, W. Holton, P.D. Reimer, A. Lamarche, M. White, N. Rutherford, C. Childs, G. Mauseth, G. Challenger, and E. Taylor. 2013. Extent and

degree of shoreline oiling: Deepwater Horizon oil spill, Gulf of Mexico, USA. PLoS ONE 8(6):e65087. doi: 10.1371/journal.pone.0065087
Miller, C.E. 2003. Abundance trends and environmental habitat usage patterns of bottlenose dolphins (*Tursiops truncatus*) in lower Barataria and Caminada bays, Louisiana. Louisiana State University and Agricultural & Mechanical College. Dissertation. 125 p.
Miller, L.J., A.D. Mackey, T. Hoffland, M. Solangi, and S.A. Kuczaj. 2010. Potential effects of a major hurricane on Atlantic bottlenose dolphin (*Tursiops truncatus*) reproduction in the Mississippi Sound. Marine Mammal Science 26(3):707–715. doi: 10.1111/j.1748-7692.2010.00371.x
Miller, L.J., A.D. Mackey, M. Solangi, and S.A. Kuczaj. 2013. Population abundance and habitat utilization of bottlenose dolphins in the Mississippi Sound. Aquatic Conservation: Marine and Freshwater Ecosystems 23(1):145–151. doi: 10.1002/aqc.2278
Mullin, K.D., T. McDonald, R.S. Wells, B.C. Balmer, T. Speakman, C. Sinclair, E.S. Zolman, F. Hornsby, S.M. McBride, K.A. Wilkinson, and L.H. Schwacke. 2017. Density, abundance, survival, and ranging patterns of common bottlenose dolphins (*Tursiops truncatus*) in Mississippi Sound following the Deepwater Horizon oil spill. PLoS ONE 12(10):e0186265. doi: 10.1371/journal.pone.0186265
National Oceanic and Atmospheric Administration (NOAA). 2011. Public scoping for preparation of a programmatic environmental impact statement for the Deepwater Horizon BP Oil Spill. 9 p.
Perrin, W.F. and S.B. Reilly. 1984. Reproductive parameters of dolphins and small whales of the family Delphinidae. In: Perrin, W.F., R.L. Brownell, Jr., and D.P. DeMaster (Eds.), Reproduction in Whales, Dolphins, and Porpoises. pp. 97–134.
Roberts, J.J., B.D. Best, L. Mannocci, E. Fujioka, P.N. Halpin, D.L. Palka, L.P. Garrison, K.D. Mullin, T.V.N. Cole, C.B. Khan, W.A. McLellan, D.A. Pabst, and G.G. Lockhart. 2016. Habitat-based cetacean density models for the US Atlantic and Gulf of Mexico. Scientific Reports 6:22615. doi: 10.1038/Srep22615
Roberts, J.J., B.D. Best, L. Mannocci, E. Fujioka, P.N. Halpin, D.L. Palka, L.P. Garrison, K.D. Mullin, T.V.N. Cole, C.B. Khan, W.M. McLellan, D.A. Pabst, and G.G. Lockhart. 2015. Density model for bottlenose dolphin (*Tursiops truncatus*) for the U.S. Gulf of Mexico version 3.3, 2015-10-07, and supplementary report. Duke University, Marine Geospatial Ecology Lab: Durham, NC. 84 p.
Rosel, P.E., L.A. Wilcox, C. Sinclair, T.R. Speakman, M.C. Tumlin, J.A. Litz, and E.S. Zolman. 2017. Genetic assignment to stock of stranded common bottlenose dolphins in southeastern Louisiana after the Deepwater Horizon oil spill. Endangered Species Research 33:221–234. doi: 10.3354/esr00780
Rossman, S., P.H. Ostrom, M. Stolen, N.B. Barros, H. Gandhi, C.A. Stricker, and R.S. Wells. 2015. Individual specialization in the foraging habits of female bottlenose dolphins living in a trophically diverse and habitat rich estuary. Oecologia 178(2):415–425. doi: 10.1007/s00442-015-3241-6
Schwacke, L.H., C.R. Smith, F.I. Townsend, R.S. Wells, L.B. Hart, B.C. Balmer, T.K. Collier, S. De Guise, M.M. Fry, L.J. Guillette, Jr., S.V. Lamb, S.M. Lane, W.E. McFee, N.J. Place, M.C. Tumlin, G.M. Ylitalo, E.S. Zolman, and T.K. Rowles. 2014. Health of common bottlenose dolphins (*Tursiops truncatus*) in Barataria Bay, Louisiana, following the deepwater horizon oil spill. Environmental Science & Technology 48(1):93–103. doi: 10.1021/es403610f
Schwacke, L.H., L. Thomas, R.S. Wells, W.E. McFee, A.A. Hohn, K.D. Mullin, E.S. Zolman, B.M. Quigley, T.K. Rowles, and J.H. Schwacke. 2017. Quantifying injury to common bottlenose dolphins from the Deepwater Horizon oil spill using an age-, sex- and class-structured population model. Endangered Species Research 33:265–279. doi: 10.3354/esr00777
Sellas, A.B., R.S. Wells, and P.E. Rosel. 2005. Mitochondrial and nuclear DNA analyses reveal fine scale geographic structure in bottlenose dolphins (*Tursiops truncatus*) in the Gulf of Mexico. Conservation Genetics 6(5):715–728. doi: 10.1007/s10592-005-9031-7

Sergeant, D.E., D.K. Caldwell, and M.C. Caldwell. 1973. Age, growth, and maturity of bottlenosed dolphin (*Tursiops-truncatus*) from northeast Florida. Journal of the Fisheries Research Board of Canada 30(7):1009–1011. doi: 10.1139/f73-165

Shane, S.H., R.S. Wells, and B. Würsig. 1986. Ecology, behavior and social organization of the bottlenose dolphin: A review. Marine Mammal Science 2:34–63. doi: 10.1111/j.1748-7692.1986.tb00026.x

Simard, P., C.C. Wall, J.B. Allen, R.S. Wells, S. Gowans, and E.A. Forys. 2015. Dolphin distribution on the west Florida Shelf using visual surveys and passive acoustic monitoring. Aquatic Mammals 41(2):167–187. doi: 10.1578/am.41.2.2015.167

Smith, C.R., T.K. Rowles, L.B. Hart, F.I. Townsend, R.S. Wells, E.S. Zolman, B.C. Balmer, B. Quigley, M. Ivančić, W. McKercher, M.C. Tumlin, K.D. Mullin, J.D. Adams, Q. Wu, W. McFee, T.K. Collier, and L.H. Schwacke. 2017. Slow recovery of Barataria Bay dolphin health following the Deepwater Horizon oil spill (2013-2014), with evidence of persistent lung disease and impaired stress response. Endangered Species Research 33:127–142. doi: 10.3354/esr00778

Takeshita, R., B.C. Balmer, F. Messina, E.S. Zolman, L. Thomas, R.S. Wells, C.R. Smith, T.K. Rowles, and L.H. Schwacke. 2021. High site-fidelity in common bottlenose dolphins despite low salinity exposure and associated indicators of compromised health. PLoS ONE 16(9):e0258031. doi: 10.1371/journal.pone.0258031

Taylor, B.L., S.J. Chivers, J. Larese, and W.F. Perrin. 2007. Generation length and percent mature estimates for IUCN assessments of cetaceans. National Oceanic and Atmospheric Administration, National Marine Fisheries Service, Southwest Fisheries Science Center: La Jolla, CA. Administrative Report LJ-07-01. 24 p.

Torres, L.G. and A.J. Read. 2009. Where to catch a fish? The influence of foraging tactics on the ecology of bottlenose dolphins (*Tursiops truncatus*) in Florida Bay, Florida. Marine Mammal Science 25(4):797–815. doi: 10.1111/j.1748-7692.2009.00297.x

Tyson, R.B., S.M. Nowacek, and D.P. Nowacek. 2011. Community structure and abundance of bottlenose dolphins *Tursiops truncatus* in coastal waters of the northeast Gulf of Mexico. Marine Ecology Progress Series 438:253–265. doi: 10.3354/meps09292

Urian, K.W., S. Hofmann, R.S. Wells, and A.J. Read. 2009. Fine-scale population structure of bottlenose dolphins (*Tursiops truncatus*) in Tampa Bay, Florida. Marine Mammal Science 25(3):619–638. doi: 10.1111/j.1748-7692.2009.00284.x

Venn-Watson, S., L. Garrison, J. Litz, E. Fougeres, B. Mase, G. Rappucci, E. Stratton, R. Carmichael, D. Odell, D. Shannon, S. Shippee, S. Smith, L. Staggs, M. Tumlin, H. Whitehead, and T. Rowles. 2015. Demographic clusters identified within the northern Gulf of Mexico common bottlenose dolphin (*Tursiops truncates*) unusual mortality event: January 2010-June 2013. PLoS ONE 10(2):e0117248. doi: 10.1371/journal.pone.0117248

Vollmer, N.L. 2011. Population structure of common bottlenose dolphins in coastal and offshore waters of the Gulf of Mexico revealed by genetic and environmental analysis. University of Louisiana at Lafayette. Dissertation. 409 p.

Vollmer, N.L. and P.E. Rosel. 2013. A review of common bottlenose dolphins (*Tursiops truncatus truncatus*) in the Northern Gulf of Mexico: Population biology, potential threats, and management. Southeastern Naturalist 12(m6):1–43. doi: 10.1656/058.012.m601

Vollmer, N.L. and P.E. Rosel. 2017. Fine-scale population structure of common bottlenose dolphins (*Tursiops truncatus*) in offshore and coastal waters of the US Gulf of Mexico. Marine Biology 164(8):160. doi: 10.1007/s00227-017-3186-x

Waring, G.T., E. Josephson, K. Maze-Foley, and P.E. Rosel. 2016. U.S. Atlantic and Gulf of Mexico marine mammal stock assessments - 2015. National Oceanic and Atmospheric Administration, National Marine Fisheries Service, Northeast Fisheries Science Center: Woods Hole, MA. NOAA Technical Memorandum NMFS-NE-238. 501 p. doi: 10.7289/V57S7KTN

Wells, R.S. 1986. Population structure of bottlenose dolphins: Behavioral studies along the central west coast of Florida. Contract Report to National Marine Fisheries Service, Southeast Fisheries Center. Contract No. 45-WCNF-5-003BB. 58 p.

Wells, R.S. 1991. The role of long-term study in understanding the social structure of a bottlenose dolphin community. In: Pryor, K. and K. Norris (Eds.), Dolphin Societies: Discoveries and Puzzles. University of California Press, Berkeley, CA. pp. 199–226.

Wells, R.S. 2000. Reproduction in wild bottlenose dolphins: Overview of patterns observed during a long-term study. In: Duffield, D. and T. Robeck (Eds.), Bottlenose Dolphin Reproduction Workshop. AZA Marine Mammal Taxon Advisory Group, Silver Spring, MD. pp. 57–74.

Wells, R.S. 2003. Dolphin social complexity: Lessons from long-term study and life history. In: de Waal, F.B.M. and P.L. Tyack (Eds.), Animal Social Complexity: Intelligence, Culture, and Individualized Societies Harvard University Press, Cambridge, MA. pp. 32–56.

Wells, R.S. 2014. Social structure and life history of bottlenose dolphins near Sarasota Bay, Florida: Insights from four decades and five generations. Primates and cetaceans. pp. 149–172. doi: 10.1007/978-4-431-54523-1_8

Wells, R.S., A. Natoli, and G. Braulik. 2019. *Tursiops truncatus* (errata version published in 2019). *The IUCN Red List of Threatened Species* 2019: e.T22563A156932432. https://dx.doi.org/10.2305/IUCN.UK.2019-1.RLTS.T22563A156932432.en

Wells, R.S., L.H. Schwacke, T.K. Rowles, B.C. Balmer, E. Zolman, T. Speakman, F.I. Townsend, M.C. Tumlin, A. Barleycorn, and K.A. Wilkinson. 2017. Ranging patterns of common bottlenose dolphins *Tursiops truncatus* in Barataria Bay, Louisiana, following the Deepwater Horizon oil spill. Endangered Species Research 33:159–180. doi: 10.3354/esr00732

Wells, R.S. and M.D. Scott. 1999. Bottlenose dolphin *Tursiops truncatus* (Montagu, 1821). In: Ridgway, S.H. and R. Harrison (Eds.), Handbook of marine mammals. Volume 6: The second book of dolphins and the porpoises. Academic Press, San Diego, CA. pp. 137–182.

Wells, R.S. and M.D. Scott. 2018. Bottlenose dolphin, *Tursiops truncatus*, common bottlenose dolphin. In: Würsig, B., J.G.M. Thewissen, and K.M. Kovacs (Eds.), Encyclopedia of Marine Mammals (Third Edition). Academic Press, London, UK. pp. 118–125. doi: 10.1016/B978-0-12-804327-1.00072-8

Wells, R.S., M.D. Scott, and A.B. Irvine. 1987. The social structure of free-ranging bottlenose dolphins. In: Genoways, H.H. (Ed.), Current Mammalogy. Plenum Press, New York, NY. pp. 247–305.

Wells, R.S., K.W. Urian, A.J. Read, M.K. Bassos, W.J. Carr, and M.D. Scott. 1996. Low-level monitoring of bottlenose dolphins, *Tursiops truncatus*, in Tampa Bay, Florida: 1988-1993. National Oceanic and Atmospheric Administration, National Marine Fisheries Service, Southeast Fisheries Science Center: Miami, FL. NOAA Technical Memorandum NMFS-SEFSC-385. 36 p.

Wilson, R.M., R.B. Tyson, J.A. Nelson, B.C. Balmer, J.P. Chanton, and D.P. Nowacek. 2017. Niche differentiation and prey selectivity among common bottlenose dolphins (*Tursiops truncatus*) sighted in St. George Sound, Gulf of Mexico. Frontiers in Marine Science 4:235. doi: 10.3389/fmars.2017.00235

Common bottlenose dolphin − *Tursiops truncatus*

Gulf of Mexico, Eastern Coastal Stock

Overall Vulnerability Rank = Very High

Biological Sensitivity = High

Climate Exposure = Very High

Data Quality = 93% of scores ≥ 2

	Tursiops truncatus	Expert Scores	Data Quality	Expert Scores Plots (Portion by Category)
Sensitivity attributes	Prey/Diet Specificity	2.0	2.2	
	Habitat Specificity	2.5	2.5	
	Site Fidelity	3.1	2.5	
	Lifetime Reproductive Potential	1.8	2.0	
	Generation Length	2.7	2.0	
	Reproductive Plasticity	1.2	1.8	
	Migration	3.5	2.5	
	Home Range	2.2	2.5	
	Stock Abundance	1.2	3.0	
	Stock Abundance Trend	2.1	1.5	
	Cumulative Stressors	2.8	2.2	
	Sensitivity Score	High		
Exposure variables	Sea Surface Temperature (Change in mean)	4.0	3.0	
	Sea Surface Temperature (Change in variability)	1.0	3.0	
	Air Temperature (Change in mean)	4.0	3.0	
	Air Temperature (Change in variability)	1.0	3.0	
	Precipitation (Change in mean)	1.0	3.0	
	Precipitation (Change in variability)	1.0	3.0	
	Sea Surface Salinity (Change in mean)	2.5	3.0	
	Sea Surface Salinity (Change in variability)	1.4	3.0	
	Ocean pH (Change in mean)	4.0	3.0	
	Ocean pH (Change in variability)	1.0	3.0	
	Sea ice coverage (Change in mean)	1.0	3.0	
	Sea ice coverage (Change in variability)	1.0	3.0	
	Dissolved oxygen (Change in mean)	4.0	3.0	
	Dissolved oxygen (Change in variability)	1.0	3.0	
	Circulation	3.0	2.8	
	Sea level rise	2.8	3.0	
	Exposure Score	Very High		
Overall Vulnerability Rank		Very High		

Low
Moderate
High
Very High

Common bottlenose dolphin (Gulf of Mexico, Eastern Coastal Stock)

Tursiops truncatus

CVA Results Summary

Overall Climate Vulnerability Rank: Very High (78% certainty from bootstrap analysis).

Climate Exposure: Very High. Four exposure factors scored greater than 3.5: Air Temperature (Standard anomaly) (3.95), Dissolved oxygen (Standard anomaly) (3.95), Ocean pH (Standard anomaly) (3.95), and Sea Surface Temperature (Standard anomaly) (3.95).

Biological Sensitivity: High. Two sensitivity attributes scored greater than 3.0: Migration (3.55) and Site Fidelity (3.10).

Distributional Response: Moderate

Abundance Response: Moderate

Phenology Response: Moderate

Data Quality: 93% of the data quality scores were 2 or greater, 82% of sensitivity attribute data quality scores were 2 or higher.

Background Information

Stock Description

This stock includes common bottlenose dolphins in coastal waters (defined as waters between the shore, barrier islands or presumed outer bay boundaries out to the 20-m isobath) in an area of the eastern Gulf of Mexico that extends from 84W longitude to Key West, Florida (Hayes et al. 2022).

Prey/Diet Specificity

Generally, common bottlenose dolphins consume a large variety of fish and/or squid (Barros and Odell 1990; Mead and Potter 1990; Wells and Scott 2018). Common bottlenose dolphins seem to show a consistent preference for sound-producing fishes (e.g., sciaenids, toadfish; Berens McCabe et al. 2010; Bowen-Stevens et al. 2021). In Sarasota Bay, Florida, Rossman et al. (2015) found some individual common bottlenose dolphins to show diet specialization despite the broader study group showing a generalist diet. Specialization can be influenced by locally abundant prey species (Shane et al. 1986; Vollmer and Rosel 2013) or can result from resource partitioning amongst sympatric populations (e.g., Torres and Read 2009; Wilson et al. 2017).

Habitat Specificity

Common bottlenose dolphins are found in a variety of marine and estuarine habitats (Wells and Scott 2018).

Site Fidelity

No specific site fidelity information was found for this stock. However, common bottlenose dolphins in Gulf of Mexico bay, sound, and estuary (BSE) stocks show site fidelity (LaBrecque et al. 2015; Hayes et al. 2022), such as in Matagorda Bay, Espiritu Santo Bay, and San Luis Pass in Texas (Maze and Würsig 1999; Lynn and Würsig 2002; Irwin and Würsig 2004); Barataria Bay in Louisiana (Miller 2003; McDonald et al. 2017; Wells et al. 2017; Takeshita et al. 2021); Mississippi Sound in Mississippi (Hubard et al. 2004; Mackey 2010; Mullin et al. 2017); Charlotte Harbor and Pine Island Sound (Bassos-Hull et al. 2013), Sarasota Bay (Wells 1986; Wells 1991; Wells 2014), St. Andrew Bay (Balmer et al. 2019), St. Joseph Bay (Balmer et al. 2008; B. Balmer et al. 2018), St. Vincent Sound and Apalachicola Bay (Tyson et al. 2011), and Tampa Bay in Florida (Wells et al. 1996; Urian et al. 2009).

Lifetime Reproductive Potential

Taylor et al. (2007) reported the interbirth interval of common bottlenose dolphins as 3.80 years, though other studies have shown it to be closer to three years (Leatherwood 1977; Wells and Scott 1999; Connor et al. 2000; Wells 2003).

Generally, females reach sexual maturity before males, with females reaching sexual maturity at 5–13 years (Sergeant et al. 1973; Perrin and Reilly 1984; Wells et al. 1987; Mead and Potter 1990; Mann et al. 2000; Wells 2000; Wells and Scott 2018).

Females reproduce through most of their adult lives (Leatherwood 1977; Hohn et al. 1989; Wells and Scott 1999; Wells 2000; Wells 2003), but may produce fewer calves after they reach 30 years of age (Wells 2000). Sarasota Bay common bottlenose dolphins can give birth when they are as young as 6 years of age, and can continue until they are 48 years old (Wells and Scott 2018). Reproductive lifespan would therefore be up to 42 years based on long-term observation studies (Wells 2014; Wells and Scott 2018).

Longitudinal studies from Sarasota Bay, Florida have found that female common bottlenose dolphins can live up to 67 years (Wells and Scott 2018). Studies based on growth layer groups in teeth suggest female common bottlenose dolphins can live to 41 years along the Texas coast (Fernandez and Hohn 1998) and to 30 years along the Mississippi coast (Mattson et al. 2006).

Generation Length

Taylor et al. (2007) reported a common bottlenose dolphin generation length of 20.6–21.1 years.

Reproductive Plasticity

Common bottlenose dolphin calving is generally diffusely seasonal, with one or more peaks, although births have been reported from all seasons (Vollmer and Rosel 2013; Wells and Scott 2018). Calf sightings peak in the spring and summer months in Florida (Wells et al. 1987), Mississippi/Louisiana (Miller et al. 2010; Miller et al. 2013), and Texas (McHugh 1989; Henderson and Würsig 2007).

Migration

Data are insufficient to characterize migration patterns (or the lack thereof) for this stock. However, genetic data do support the presence of multiple differentiated populations of common bottlenose dolphins in coastal and offshore waters with very low levels of migration among them (Vollmer and Rosel 2017). Molecular genetic data further support that coastal populations are genetically differentiated from BSE populations (Sellas et al. 2005; Rosel et al. 2017). In waters of the West Florida Shelf, acoustic and visual sighting data suggest seasonal movement from deeper waters in the winter and spring into shallower waters during the summer and fall (Simard et al. 2015).

Home Range

Data are insufficient to characterize home range patterns (or the lack thereof) for this stock.

Stock Abundance

The best abundance estimate available for this stock is 16,407 individuals (CV=0.17; Garrison et al. 2021; Hayes et al. 2022) based on summer 2017 and fall 2018 aerial surveys. Previously, this stock was estimated at 12,388 individuals (CV=0.13) based on seasonal abundance estimates from spring 2011, summer 2011, fall 2011 and winter 2012 aerial surveys (Waring et al. 2016).

Stock Abundance Trend

Data are insufficient to complete an abundance trend analysis for this stock (Hayes et al. 2022).

Cumulative Stressors

Common bottlenose dolphins face a wide range of individual and cumulative natural and anthropogenic threats (see reviews by Vollmer and Rosel 2013; Wells and Scott 2018; and Wells et al. 2019). These include such threats as shark predation, harmful algal blooms, recreational and commercial fishing gear, vessel traffic, pollution, habitat alteration, and provisioning.

The Gulf of Mexico eastern coastal common bottlenose dolphin stock potentially interacts with eight commercial fisheries (Southeastern U.S. Atlantic, Gulf of Mexico shrimp trawl; Gulf of Mexico gillnet; Southeastern U.S. Atlantic, Gulf of Mexico stone crab trap/pot; Southeastern U.S. Atlantic, Gulf of Mexico shark bottom longline/hook-and-line; Florida spiny lobster trap/pot; Gulf of Mexico blue crab trap/pot; Florida West Coast sardine purse seine; and commercial passenger fishing vessel/hook-and-line; Hayes et al. 2022).

The nearshore and estuarine habitats along the Gulf of Mexico coast include waters adjacent to areas of high human population, some of which are highly industrialized, and areas of heavy agricultural impact. Studies of common bottlenose dolphin tissues from Florida Gulf Coast waters have shown persistent organic pollutants (POPs) in blubber and blood samples (Yordy et al. 2010a, 2010b, 2010c; Kucklick et al. 2011; J. Balmer et al. 2018). Similarly, mercury has been detected in common bottlenose dolphin tissues in Florida Gulf Coast waters (Bryan et al. 2007; Woshner et al. 2008; Miller et al. 2011; Correa et al. 2013; McCormack et al. 2020a, 2020b, 2022).

Five Unusual Mortality Events (UMEs) in the Gulf of Mexico have overlapped with the eastern coastal common bottlenose dolphin stock. The causes of these UMEs have included morbillivirus and brevetoxin (Litz et al. 2014).

Distribution and Sightings

Density model results for common bottlenose dolphins in the Gulf of Mexico are presented by Roberts et al. (2015, 2016) and Mannocci et al. (2017).

Further Reading

Hayes, S.A., E. Josephson, K. Maze-Foley, P.E. Rosel, and J. Wallace. 2022. U.S. Atlantic and Gulf of Mexico marine mammal stock assessments 2021. National Oceanic and Atmospheric Administration, National Marine Fisheries Service, Northeast Fisheries Science Center: Woods Hole, MA. NOAA Technical Memorandum NMFS-NE-288. 380 p. doi: 10.25923/6tt7-kc16

Wells, R. S. and M. D. Scott. 2018. Bottlenose Dolphin, *Tursiops truncatus*, Common Bottlenose Dolphin. In: Würsig, B., Thewissen, J.G.M. and Kovacs, K.M. eds. Encyclopedia of marine mammals. Academic Press. Pages 118-125

Wells, R.S., A. Natoli, and G. Braulik. 2019. *Tursiops truncatus* (errata version published in 2019). The IUCN Red List of Threatened Species 2019: e.T22563A156932432. https://dx.doi.org/10.2305/IUCN.UK.2019-1.RLTS.T22563A156932432.en

References

Balmer, B., T. McDonald, F. Hornsby, J. Adams, J. Allen, A. Barleycorn, and L. Schwacke. 2018. Long-term trends in a northern Gulf of Mexico common bottlenose dolphin (*Tursiops truncatus*) population in the wake of the Deepwater Horizon oil spill. Journal of Cetacean Research and Management 18:1–9. doi: 10.47536/jcrm.v18i1.409

Balmer, B., S. Watwood, B. Quigley, T. Speakman, K. Barry, K. Mullin, P. Rosel, C. Sinclair, E. Zolman, and L. Schwacke. 2019. Common bottlenose dolphin (*Tursiops truncatus*) abundance and distribution patterns in St. Andrew Bay, Florida, USA. Aquatic Conservation: Marine and Freshwater Ecosystems 29(3):486–498. doi: 10.1002/aqc.3001

Balmer, B.C., R.S. Wells, S.M. Nowacek, D.P. Nowacek, L.H. Schwacke, W.A. McLellan, F.S. Scharf, T.K. Rowles, L.J. Hansen, T.R. Spradlin, and D.A. Pabst. 2008. Seasonal abundance and distribution patterns of common bottlenose dolphins (*Tursiops truncatus*) near St. Joseph Bay, Florida, USA. Journal of Cetacean Research and Management 10(2):157–167.

Balmer, J.E., G.M. Ylitalo, T.K. Rowles, K.D. Mullin, R.S. Wells, F.I. Townsend, R.W. Pearce, J.L. Bolton, E.S. Zolman, B.C. Balmer, and L.H. Schwacke. 2018. Persistent organic pollutants (POPs) in blood and blubber of common bottlenose dolphins (*Tursiops truncatus*) at three northern Gulf of Mexico sites following the Deepwater Horizon oil spill. Science of the Total Environment 621:130–137. doi: 10.1016/j.scitotenv.2017.11.209

Barros, N.B. and D.K. Odell. 1990. Food habits of bottlenose dolphins in the southeastern United States In: Leatherwood, S. and R.R. Reeves (Eds.), The Bottlenose Dolphin. Academic Press, San Diego, CA. pp. 309–328. doi: 10.1016/B978-0-12-440280-5.50020-2

Bassos-Hull, K., R.M. Perrtree, C.C. Shepard, S. Schilling, A.A. Barleycorn, J.B. Allen, B.C. Balmer, W.E. Pine, and R.S. Wells. 2013. Long-term site fidelity and seasonal abundance estimates of common bottlenose dolphins (*Tursiops truncatus*) along the southwest coast of Florida and responses to

natural perturbations. Journal of Cetacean Research and Management 13(1):19–30. doi: 10.47536/jcrm.v13i1.551

Berens McCabe, E.J., D.P. Gannon, N.B. Barros, and R.S. Wells. 2010. Prey selection by resident common bottlenose dolphins (*Tursiops truncatus*) in Sarasota Bay, Florida. Marine Biology 157(5):931–942. doi: 10.1007/s00227-009-1371-2

Bowen-Stevens, S.R., D.P. Gannon, R.A. Hazelkorn, G. Lovewell, K.M. Volker, S. Smith, M.C. Tumlin, and J. Litz. 2021. Diet of common bottlenose dolphins, *Tursiops truncatus*, that stranded in and near Barataria Bay, Louisiana, 2010–2012. Southeastern Naturalist 20(1). doi: 10.1656/058.020.0113

Bryan, C.E., S.J. Christopher, B.C. Balmer, and R.S. Wells. 2007. Establishing baseline levels of trace elements in blood and skin of bottlenose dolphins in Sarasota Bay, Florida: implications for non-invasive monitoring. Science of the Total Environment 388(1-3):325–342. doi: 10.1016/j.scitotenv.2007.07.046

Connor, R.C., R.S. Wells, J. Mann, and A.J. Read. 2000. The bottlenose dolphin: Social relationships in a fission-fusion society. In: Mann, J., R. Connor, P. Tyack, and H. Whitehead (Eds.), Cetacean Societies: Field Studies of Dolphins and Whales. University of Chicago Press, Chicago, IL. pp. 91–126.

Correa, L., J.M. Castellini, R.S. Wells, and T. O'Hara. 2013. Distribution of mercury and selenium in blood compartments of bottlenose dolphins (*Tursiops truncatus*) from Sarasota Bay, Florida. Environmental Toxicology and Chemistry 32(11):2441–2448. doi: 10.1002/etc.2327

Fernandez, S. and A.A. Hohn. 1998. Age, growth, and calving season of bottlenose dolphins, *Tursiops truncatus*, off coastal Texas. Fishery Bulletin 96(2):357–365.

Garrison, L.P., J. Ortega-Ortiz, and G. Rappucci. 2021. Abundance of coastal and continental shelf stocks of common bottlenose and Atlantic spotted dolphins in the northern Gulf of Mexico: 2017–2018. National Oceanic and Atmospheric Administration, National Marine Fisheries Service, Southeast Fisheries Science Center, Protected Resources and Biodiversity Division: Miami, FL. PRD-2021-01. 25 p. doi: 10.25923/vk95-t881

Hayes, S.A., E. Josephson, K. Maze-Foley, P.E. Rosel, and J. Wallace. 2022. U.S. Atlantic and Gulf of Mexico marine mammal stock assessments 2021. National Oceanic and Atmospheric Administration, National Marine Fisheries Service, Northeast Fisheries Science Center: Woods Hole, MA. NOAA Technical Memorandum NMFS-NE-288. 380 p. doi: 10.25923/6tt7-kc16

Henderson, E.E. and B. Würsig. 2007. Behavior patterns of bottlenose dolphins in San Luis Pass, Texas. Gulf of Mexico Science 25(2):153–161. doi: 10.18785/goms.2502.06

Hohn, A.A., M.D. Scott, R.S. Wells, J.C. Sweeney, and A.B. Irvine. 1989. Growth layers in teeth from known-age, free-ranging bottlenose dolphins. Marine Mammal Science 5(4):315–342. doi: 10.1111/j.1748-7692.1989.tb00346.x

Hubard, C.W., K. Maze-Foley, K.D. Mullin, and W.W. Schroeder. 2004. Seasonal abundance and site fidelity of bottlenose dolphins (*Tursiops truncatus*) in Mississippi Sound. Aquatic Mammals 30(2):299–310. doi: 10.1578/am.30.2.2004.299

Irwin, L.-J. and B. Würsig. 2004. A small resident community of bottlenose dolphins, *Tursiops truncatus*, in Texas: Monitoring recommendations. Gulf of Mexico Science 22(1):13–21. doi: 10.18785/goms.2201.02

Kucklick, J., L. Schwacke, R. Wells, A. Hohn, A. Guichard, J. Yordy, L. Hansen, E. Zolman, R. Wilson, J. Litz, D. Nowacek, T. Rowles, R. Pugh, B. Balmer, C. Sinclair, and P. Rosel. 2011. Bottlenose dolphins as indicators of persistent organic pollutants in the western North Atlantic Ocean and Northern Gulf of Mexico. Environmental Science & Technology 45(10):4270–4277. doi: 10.1021/es1042244

LaBrecque, E., C. Curtice, J. Harrison, S.M. Van Parijs, and P.N. Halpin. 2015. 3. Biologically Important Areas for cetaceans within U.S. Waters – Gulf of Mexico Region. Aquatic Mammals 41(1):30–38. doi: 10.1578/am.41.1.2015.30

Leatherwood, S. 1977. Some preliminary impressions on the numbers and social behavior of free-swimming bottlenosed dolphin calves (*Tursiops truncatus*) in the northern Gulf of Mexico. In: Ridgway, S.H. and K. Bernirschke (Eds.), Breeding Dolphins; Present Status, Suggestions for the Future. Final report to the US Marine Mammal Commission, Contract MM6AC009, MMC-76/07, Washington, DC. pp. 143–167.

Litz, J.A., M.A. Baran, S.R. Bowen-Stevens, R.H. Carmichael, K.M. Colegrove, L.P. Garrison, S.E. Fire, E.M. Fougeres, R. Hardy, S. Holmes, W. Jones, B.E. Mase-Guthrie, D.K. Odell, P.E. Rosel, J.T. Saliki, D.K. Shannon, S.F. Shippee, S.M. Smith, E.M. Stratton, M.C. Tumlin, H.R. Whitehead, G.A. Worthy, and T.K. Rowles. 2014. Review of historical unusual mortality events (UMEs) in the Gulf of Mexico (1990–2009): providing context for the multi-year northern Gulf of Mexico cetacean UME declared in 2010. Diseases of Aquatic Organisms 112(2):161–175. doi: 10.3354/dao02807

Lynn, S.K. and B. Würsig. 2002. Summer movement patterns of bottlenose dolphins in a Texas bay. Gulf of Mexico Science 20(1):25–37. doi: 10.18785/goms.2001.03

Mackey, A.D. 2010. Site fidelity and association patterns of bottlenose dolphins (*Tursiops truncatus*) in the Mississippi Sound. University of Southern Mississippi. Thesis. 133 p.

Mann, J., R.C. Connor, L.M. Barre, and M.R. Heithaus. 2000. Female reproductive success in bottlenose dolphins (*Tursiops* sp.): life history, habitat, provisioning, and group-size effects. Behavioral Ecology 11(2):210–219. doi: 10.1093/beheco/11.2.210

Mannocci, L., J.J. Roberts, D.L. Miller, and P.N. Halpin. 2017. Extrapolating cetacean densities to quantitatively assess human impacts on populations in the high seas. Conservation Biology 31(3):601–614. doi: 10.1111/cobi.12856

Mattson, M.C., K.D. Mullin, G.W. Ingram, and W. Hoggard. 2006. Age structure and growth of the bottlenose dolphin (*Tursiops truncatus*) from strandings in the Mississippi sound region of the north-central Gulf of Mexico from 1986 to 2003. Marine Mammal Science 22(3):654–666. doi: 10.1111/j.1748-7692.2006.00057.x

Maze, K.S. and B. Würsig. 1999. Bottlenosed dolphins of San Luis Pass, Texas: Occurrence patterns, site-fidelity, and habitat use. Aquatic Mammals 25:91–104.

McCormack, M.A., F. Battaglia, W.E. McFee, and J. Dutton. 2020a. Mercury concentrations in blubber and skin from stranded bottlenose dolphins (*Tursiops truncatus*) along the Florida and Louisiana coasts (Gulf of Mexico, USA) in relation to biological variables. Environmental Research 180:108886. doi: 10.1016/j.envres.2019.108886

McCormack, M.A., B.P. Jackson, and J. Dutton. 2020b. Relationship between mercury and selenium concentrations in tissues from stranded odontocetes in the northern Gulf of Mexico. Science of the Total Environment 749:141350. doi: 10.1016/j.scitotenv.2020.141350

McCormack, M.A., W.H. Nowlin, and J. Dutton. 2022. Effect of trophic position on mercury concentrations in bottlenose dolphins (*Tursiops truncatus*) from the northern Gulf of Mexico. Environmental Research 204(Pt B):112124. doi: 10.1016/j.envres.2021.112124

McDonald, T.L., F.E. Hornsby, T.R. Speakman, E.S. Zolman, K.D. Mullin, C. Sinclair, P.E. Rosel, L. Thomas, and L.H. Schwacke. 2017. Survival, density, and abundance of common bottlenose dolphins in Barataria Bay (USA) following the Deepwater Horizon oil spill. Endangered Species Research 33:193–209. doi: 10.3354/esr00806

McHugh, M.B. 1989. Population numbers and feeding behavior of the Atlantic bottlenose dolphin (*Tursiops truncatus*) near Aransas Pass, Texas. University of Texas at Austin. Thesis. 96 p.

Mead, J.G. and C.W. Potter. 1990. Natural history of bottlenose dolphins along the central Atlantic coast of the United States. In: Leatherwood, S. and R.R. Reeves (Eds.), The Bottlenose Dolphin. Academic Press, San Diego, CA. pp. 165–195.

Miller, C.E. 2003. Abundance trends and environmental habitat usage patterns of bottlenose dolphins (*Tursiops truncatus*) in lower Barataria and Caminada bays, Louisiana. Louisiana State University and Agricultural & Mechanical College. Dissertation. 125 p.

Miller, D.L., V. Woshner, E.L. Styer, S. Ferguson, K.K. Knott, M.J. Gray, R.S. Wells, and T.M. O'Hara. 2011. Histologic findings in free-ranging Sarasota Bay bottlenose dolphin (*Tursiops truncatus*) skin: mercury, selenium, and seasonal factors. Journal of Wildlife Diseases 47(4):1012–1018. doi: 10.7589/0090-3558-47.4.1012

Miller, L.J., A.D. Mackey, T. Hoffland, M. Solangi, and S.A. Kuczaj. 2010. Potential effects of a major hurricane on Atlantic bottlenose dolphin (*Tursiops truncatus*) reproduction in the Mississippi Sound. Marine Mammal Science 26(3):707–715. doi: 10.1111/j.1748-7692.2010.00371.x

Miller, L.J., A.D. Mackey, M. Solangi, and S.A. Kuczaj. 2013. Population abundance and habitat utilization of bottlenose dolphins in the Mississippi Sound. Aquatic Conservation: Marine and Freshwater Ecosystems 23(1):145–151. doi: 10.1002/aqc.2278

Mullin, K.D., T. McDonald, R.S. Wells, B.C. Balmer, T. Speakman, C. Sinclair, E.S. Zolman, F. Hornsby, S.M. McBride, K.A. Wilkinson, and L.H. Schwacke. 2017. Density, abundance, survival, and ranging patterns of common bottlenose dolphins (*Tursiops truncatus*) in Mississippi Sound following the Deepwater Horizon oil spill. PLoS ONE 12(10):e0186265. doi: 10.1371/journal.pone.0186265

Perrin, W.F. and S.B. Reilly. 1984. Reproductive parameters of dolphins and small whales of the family Delphinidae. In: Perrin, W.F., R.L. Brownell, Jr., and D.P. DeMaster (Eds.), Reproduction in Whales, Dolphins, and Porpoises. pp. 97–134.

Roberts, J.J., B.D. Best, L. Mannocci, E. Fujioka, P.N. Halpin, D.L. Palka, L.P. Garrison, K.D. Mullin, T.V.N. Cole, C.B. Khan, W.A. McLellan, D.A. Pabst, and G.G. Lockhart. 2016. Habitat-based cetacean density models for the US Atlantic and Gulf of Mexico. Scientific Reports 6:22615. doi: 10.1038/Srep22615

Roberts, J.J., B.D. Best, L. Mannocci, E. Fujioka, P.N. Halpin, D.L. Palka, L.P. Garrison, K.D. Mullin, T.V.N. Cole, C.B. Khan, W.M. McLellan, D.A. Pabst, and G.G. Lockhart. 2015. Density model for bottlenose dolphin (*Tursiops truncatus*) for the U.S. Gulf of Mexico version 3.3, 2015-10-07, and supplementary report. Duke University, Marine Geospatial Ecology Lab: Durham, NC. 84 p.

Rosel, P.E., L.A. Wilcox, C. Sinclair, T.R. Speakman, M.C. Tumlin, J.A. Litz, and E.S. Zolman. 2017. Genetic assignment to stock of stranded common bottlenose dolphins in southeastern Louisiana after the Deepwater Horizon oil spill. Endangered Species Research 33:221–234. doi: 10.3354/esr00780

Rossman, S., P.H. Ostrom, M. Stolen, N.B. Barros, H. Gandhi, C.A. Stricker, and R.S. Wells. 2015. Individual specialization in the foraging habits of female bottlenose dolphins living in a trophically diverse and habitat rich estuary. Oecologia 178(2):415–425. doi: 10.1007/s00442-015-3241-6

Sellas, A.B., R.S. Wells, and P.E. Rosel. 2005. Mitochondrial and nuclear DNA analyses reveal fine scale geographic structure in bottlenose dolphins (*Tursiops truncatus*) in the Gulf of Mexico. Conservation Genetics 6(5):715–728. doi: 10.1007/s10592-005-9031-7

Sergeant, D.E., D.K. Caldwell, and M.C. Caldwell. 1973. Age, growth, and maturity of bottlenosed dolphin (*Tursiops-truncatus*) from northeast Florida. Journal of the Fisheries Research Board of Canada 30(7):1009–1011. doi: 10.1139/f73-165

Shane, S.H., R.S. Wells, and B. Würsig. 1986. Ecology, behavior and social organization of the bottlenose dolphin: A review. Marine Mammal Science 2:34–63. doi: 10.1111/j.1748-7692.1986.tb00026.x

Simard, P., C.C. Wall, J.B. Allen, R.S. Wells, S. Gowans, and E.A. Forys. 2015. Dolphin distribution on the west Florida Shelf using visual surveys and passive acoustic monitoring. Aquatic Mammals 41(2):167–187. doi: 10.1578/am.41.2.2015.167

Takeshita, R., B.C. Balmer, F. Messina, E.S. Zolman, L. Thomas, R.S. Wells, C.R. Smith, T.K. Rowles, and L.H. Schwacke. 2021. High site-fidelity in common bottlenose dolphins despite low salinity exposure and associated indicators of compromised health. PLoS ONE 16(9):e0258031. doi: 10.1371/journal.pone.0258031

Taylor, B.L., S.J. Chivers, J. Larese, and W.F. Perrin. 2007. Generation length and percent mature estimates for IUCN assessments of cetaceans. National Oceanic and Atmospheric Administration, National Marine Fisheries Service, Southwest Fisheries Science Center: La Jolla, CA. Administrative Report LJ-07-01. 24 p.

Torres, L.G. and A.J. Read. 2009. Where to catch a fish? The influence of foraging tactics on the ecology of bottlenose dolphins (*Tursiops truncatus*) in Florida Bay, Florida. Marine Mammal Science 25(4):797–815. doi: 10.1111/j.1748-7692.2009.00297.x

Tyson, R.B., S.M. Nowacek, and D.P. Nowacek. 2011. Community structure and abundance of bottlenose dolphins *Tursiops truncatus* in coastal waters of the northeast Gulf of Mexico. Marine Ecology Progress Series 438:253–265. doi: 10.3354/meps09292

Urian, K.W., S. Hofmann, R.S. Wells, and A.J. Read. 2009. Fine-scale population structure of bottlenose dolphins (*Tursiops truncatus*) in Tampa Bay, Florida. Marine Mammal Science 25(3):619–638. doi: 10.1111/j.1748-7692.2009.00284.x

Vollmer, N.L. and P.E. Rosel. 2013. A review of common bottlenose dolphins (*Tursiops truncatus truncatus*) in the Northern Gulf of Mexico: Population biology, potential threats, and management. Southeastern Naturalist 12(m6):1–43. doi: 10.1656/058.012.m601

Vollmer, N.L. and P.E. Rosel. 2017. Fine-scale population structure of common bottlenose dolphins (*Tursiops truncatus*) in offshore and coastal waters of the US Gulf of Mexico. Marine Biology 164(8):160. doi: 10.1007/s00227-017-3186-x

Waring, G.T., E. Josephson, K. Maze-Foley, and P.E. Rosel. 2016. U.S. Atlantic and Gulf of Mexico marine mammal stock assessments - 2015. National Oceanic and Atmospheric Administration, National Marine Fisheries Service, Northeast Fisheries Science Center: Woods Hole, MA. NOAA Technical Memorandum NMFS-NE-238. 501 p. doi: 10.7289/V57S7KTN

Wells, R.S. 1986. Population structure of bottlenose dolphins: Behavioral studies along the central west coast of Florida. Contract Report to National Marine Fisheries Service, Southeast Fisheries Center. Contract No. 45-WCNF-5-003BB. 58 p.

Wells, R.S. 1991. The role of long-term study in understanding the social structure of a bottlenose dolphin community. In: Pryor, K. and K. Norris (Eds.), Dolphin Societies: Discoveries and Puzzles. University of California Press, Berkeley, CA. pp. 199–226.

Wells, R.S. 2000. Reproduction in wild bottlenose dolphins: Overview of patterns observed during a long-term study. In: Duffield, D. and T. Robeck (Eds.), Bottlenose Dolphin Reproduction Workshop. AZA Marine Mammal Taxon Advisory Group, Silver Spring, MD. pp. 57–74.

Wells, R.S. 2003. Dolphin social complexity: Lessons from long-term study and life history. In: de Waal, F.B.M. and P.L. Tyack (Eds.), Animal Social Complexity: Intelligence, Culture, and Individualized Societies Harvard University Press, Cambridge, MA. pp. 32–56.

Wells, R.S. 2014. Social structure and life history of bottlenose dolphins near Sarasota Bay, Florida: Insights from four decades and five generations. Primates and cetaceans. pp. 149–172. doi: 10.1007/978-4-431-54523-1_8

Wells, R.S., A. Natoli, and G. Braulik. 2019. *Tursiops truncatus* (errata version published in 2019). *The IUCN Red List of Threatened Species* 2019: e.T22563A156932432. https://dx.doi.org/10.2305/IUCN.UK.2019-1.RLTS.T22563A156932432.en

Wells, R.S., L.H. Schwacke, T.K. Rowles, B.C. Balmer, E. Zolman, T. Speakman, F.I. Townsend, M.C. Tumlin, A. Barleycorn, and K.A. Wilkinson. 2017. Ranging patterns of common bottlenose dolphins *Tursiops truncatus* in Barataria Bay, Louisiana, following the Deepwater Horizon oil spill. Endangered Species Research 33:159–180. doi: 10.3354/esr00732

Wells, R.S. and M.D. Scott. 1999. Bottlenose dolphin *Tursiops truncatus* (Montagu, 1821). In: Ridgway, S.H. and R. Harrison (Eds.), Handbook of marine mammals. Volume 6: The second book of dolphins and the porpoises. Academic Press, San Diego, CA. pp. 137–182.

Wells, R.S. and M.D. Scott. 2018. Bottlenose dolphin, *Tursiops truncatus*, common bottlenose dolphin. In: Würsig, B., J.G.M. Thewissen, and K.M. Kovacs (Eds.), Encyclopedia of Marine Mammals (Third Edition). Academic Press, London, UK. pp. 118–125. doi: 10.1016/B978-0-12-804327-1.00072-8

Wells, R.S., M.D. Scott, and A.B. Irvine. 1987. The social structure of free-ranging bottlenose dolphins. In: Genoways, H.H. (Ed.), Current Mammalogy. Plenum Press, New York, NY. pp. 247–305.

Wells, R.S., K.W. Urian, A.J. Read, M.K. Bassos, W.J. Carr, and M.D. Scott. 1996. Low-level monitoring of bottlenose dolphins, *Tursiops truncatus*, in Tampa Bay, Florida: 1988-1993. National Oceanic and Atmospheric Administration, National Marine Fisheries Service, Southeast Fisheries Science Center: Miami, FL. NOAA Technical Memorandum NMFS-SEFSC-385. 36 p.

Wilson, R.M., R.B. Tyson, J.A. Nelson, B.C. Balmer, J.P. Chanton, and D.P. Nowacek. 2017. Niche differentiation and prey selectivity among common bottlenose dolphins (*Tursiops truncatus*) sighted in St. George Sound, Gulf of Mexico. Frontiers in Marine Science 4:235. doi: 10.3389/fmars.2017.00235

Woshner, V., K. Knott, R. Wells, C. Willetto, R. Swor, and T. O'Hara. 2008. Mercury and selenium in blood and epidermis of bottlenose dolphins (*Tursiops truncatus*) from Sarasota Bay, FL: interaction and relevance to life history and hematologic parameters. Ecohealth 5(3):360–370. doi: 10.1007/s10393-008-0164-2

Yordy, J.E., M.A. Mollenhauer, R.M. Wilson, R.S. Wells, A. Hohn, J. Sweeney, L.H. Schwacke, T.K. Rowles, J.R. Kucklick, and M.M. Peden-Adams. 2010a. Complex contaminant exposure in cetaceans: a comparative E-Screen analysis of bottlenose dolphin blubber and mixtures of four persistent organic pollutants. Environmental Toxicology and Chemistry 29(10):2143–2153. doi: 10.1002/etc.279

Yordy, J.E., D.A. Pabst, W.A. McLellan, R.S. Wells, T.K. Rowles, and J.R. Kucklick. 2010b. Tissue-specific distribution and whole-body burden estimates of persistent organic pollutants in the bottlenose dolphin (*Tursiops truncatus*). Environmental Toxicology and Chemistry 29(6):1263–1273. doi: 10.1002/etc.152

Yordy, J.E., R.S. Wells, B.C. Balmer, L.H. Schwacke, T.K. Rowles, and J.R. Kucklick. 2010c. Life history as a source of variation for persistent organic pollutant (POP) patterns in a community of common bottlenose dolphins (*Tursiops truncatus*) resident to Sarasota Bay, FL. Science of the Total Environment 408(9):2163–2172. doi: 10.1016/j.scitotenv.2010.01.032

Common bottlenose dolphin − *Tursiops truncatus*

Gulf of Mexico, Northern Coastal Stock

Overall Vulnerability Rank = Very High

Biological Sensitivity = High

Climate Exposure = Very High

Data Quality = 93% of scores ≥ 2

	Tursiops truncatus	Expert Scores	Data Quality	Expert Scores Plots (Portion by Category)
Sensitivity attributes	Prey/Diet Specificity	2.0	2.4	
	Habitat Specificity	2.6	2.6	
	Site Fidelity	2.8	2.8	
	Lifetime Reproductive Potential	1.5	2.0	
	Generation Length	3.0	2.0	
	Reproductive Plasticity	1.1	1.6	
	Migration	3.4	1.8	
	Home Range	2.3	2.2	
	Stock Abundance	2.0	3.0	
	Stock Abundance Trend	3.1	2.0	
	Cumulative Stressors	3.2	3.0	
	Sensitivity Score	High		
Exposure variables	Sea Surface Temperature (Change in mean)	4.0	2.8	
	Sea Surface Temperature (Change in variability)	1.0	2.8	
	Air Temperature (Change in mean)	4.0	2.8	
	Air Temperature (Change in variability)	1.0	2.8	
	Precipitation (Change in mean)	1.0	2.8	
	Precipitation (Change in variability)	1.0	2.8	
	Sea Surface Salinity (Change in mean)	1.6	2.8	
	Sea Surface Salinity (Change in variability)	1.2	2.8	
	Ocean pH (Change in mean)	4.0	2.8	
	Ocean pH (Change in variability)	1.0	2.8	
	Sea ice coverage (Change in mean)	1.0	2.8	
	Sea ice coverage (Change in variability)	1.0	2.8	
	Dissolved oxygen (Change in mean)	4.0	2.8	
	Dissolved oxygen (Change in variability)	1.0	2.8	
	Circulation	3.4	2.6	
	Sea level rise	3.1	2.8	
	Exposure Score	Very High		
Overall Vulnerability Rank		Very High		

Low

Moderate

High

Very High

Common bottlenose dolphin (Gulf of Mexico, Northern Coastal Stock)

Tursiops truncatus

CVA Results Summary

Overall Climate Vulnerability Rank: Very High (100% certainty from bootstrap analysis).

Climate Exposure: Very High. Four exposure factors scored greater than 3.5: Air Temperature (Standard anomaly) (4.00), Dissolved oxygen (Standard anomaly) (4.00), Ocean pH (Standard anomaly) (4.00), and Sea Surface Temperature (Standard anomaly) (4.00).

Biological Sensitivity: High. Four sensitivity attributes scored greater than 3.0: Migration (3.40), Cumulative Stressors (3.24), Species Abundance Trend (3.08), and Generation Time (3.04).

Distributional Response: High

Abundance Response: High

Phenology Response: High

Data Quality: 93% of the data quality scores were 2 or greater, 82% of sensitivity attribute data quality scores were 2 or higher.

Background Information

Stock Description

This stock includes common bottlenose dolphins in coastal waters (defined as waters between the shore, barrier islands or presumed outer bay boundaries out to the 20-m isobath) in an area of the northern Gulf of Mexico that extends from 84W longitude to the Mississippi River Delta (Waring et al. 2016).

Prey/Diet Specificity

Generally, common bottlenose dolphins consume a large variety of fish and/or squid (Barros and Odell 1990; Mead and Potter 1990; Wells and Scott 2018). Common bottlenose dolphins seem to show a consistent preference for sound-producing fishes (e.g., sciaenids, toadfish; Berens McCabe et al. 2010; Bowen-Stevens et al. 2021). In Sarasota Bay, Florida, Rossman et al. (2015) found some individual common bottlenose dolphins to show diet specialization despite the broader study group showing a generalist diet. Specialization can be influenced by locally abundant prey species (Shane et al. 1986; Vollmer and Rosel 2013) or can result from resource partitioning amongst sympatric populations (e.g., Torres and Read 2009; Wilson et al. 2017).

Habitat Specificity

Common bottlenose dolphins are found in a variety of marine and estuarine habitats (Wells and Scott 2018).

Site Fidelity

No specific site fidelity information was found for this stock. However, common bottlenose dolphins in Gulf of Mexico bay, sound, and estuary (BSE) stocks show site fidelity (LaBrecque et al. 2015; Hayes et al. 2022), such as in Matagorda Bay, Espiritu Santo Bay, and San Luis Pass in Texas (Maze and Würsig 1999; Lynn and Würsig 2002; Irwin and Würsig 2004); Barataria Bay in Louisiana (Miller 2003; McDonald et al. 2017; Wells et al. 2017; Takeshita et al. 2021); Mississippi Sound in Mississippi (Hubard et al. 2004; Mackey 2010; Mullin et al. 2017); Charlotte Harbor and Pine Island Sound (Bassos-Hull et al. 2013), Sarasota Bay (Wells 1986; Wells 1991; Wells 2014), St. Andrew Bay (Balmer et al. 2019), St. Joseph Bay (Balmer et al. 2008; Balmer et al. 2018), St. Vincent Sound and Apalachicola Bay (Tyson et al. 2011), and Tampa Bay in Florida (Wells et al. 1996; Urian et al. 2009).

Lifetime Reproductive Potential

Taylor et al. (2007) reported the interbirth interval of common bottlenose dolphins as 3.80 years, though other studies have shown it to be closer to three years (Leatherwood 1977; Wells and Scott 1999; Connor et al. 2000; Wells 2003).

Generally, females reach sexual maturity before males, with females reaching sexual maturity at 5–13 years (Sergeant et al. 1973; Perrin and Reilly 1984; Wells et al. 1987; Mead and Potter 1990; Mann et al. 2000; Wells 2000; Wells and Scott 2018).

Females reproduce throughout most of their adult lives (Leatherwood 1977; Hohn et al. 1989; Wells and Scott 1999; Wells 2000; Wells 2003), but may produce fewer calves after they reach 30 years of age (Wells 2000). Sarasota Bay common bottlenose dolphins can give birth when they are as young as 6 years of age, and can continue until they are 48 years old (Wells and Scott 2018). Reproductive lifespan would therefore be up to 42 years based on long-term observation studies (Wells 2014; Wells and Scott 2018).

Longitudinal studies from Sarasota Bay, Florida have found that female common bottlenose dolphins can live up to 67 years (Wells and Scott 2018). Studies based on growth layer groups in teeth suggest female common bottlenose dolphins can live to 41 years along the Texas coast (Fernandez and Hohn 1998) and to 30 years along the Mississippi coast (Mattson et al. 2006).

Generation Length

Taylor et al. (2007) reported a common bottlenose dolphin generation length of 20.6–21.1 years.

Reproductive Plasticity

Common bottlenose dolphin calving is generally diffusely seasonal, with one or more peaks, although births have been reported from all seasons (Vollmer and Rosel 2013; Wells and Scott 2018). Calf sightings peak in the spring and summer months in Florida (Wells et al. 1987), Mississippi/Louisiana (Miller et al. 2010; Miller et al. 2013), and Texas (McHugh 1989; Henderson and Würsig 2007).

Migration

Data are insufficient to characterize migration patterns (or the lack thereof) for this stock. However, genetic data do support the presence of multiple differentiated populations of common bottlenose dolphins in coastal and offshore waters with very low levels of migration among them (Vollmer and Rosel 2017). Molecular genetic data further support that coastal populations are genetically differentiated from BSE populations (Sellas et al. 2005; Rosel et al. 2017).

Home Range

Data are insufficient to characterize home range patterns (or the lack thereof) for this stock.

Stock Abundance

The best abundance estimate available for this stock is 11,543 individuals (CV=0.19; Garrison et al. 2021; Hayes et al. 2022) based on summer 2017, winter 2018, and fall 2018 aerial surveys. Previously, this stock was estimated at 7,185 individuals (CV=0.21) based on seasonal abundance estimates from spring 2011, summer 2011, fall 2011 and winter 2012 aerial surveys (Waring et al. 2016).

Stock Abundance Trend

Data are insufficient to complete an abundance trend analysis for this stock (Hayes et al. 2022).

Cumulative Stressors

Common bottlenose dolphins face a wide range of individual and cumulative natural and anthropogenic threats (see reviews by Vollmer and Rosel 2013; Wells and Scott 2018; and Wells et al. 2019). These include such threats as shark predation, harmful algal blooms, recreational and commercial fishing gear, vessel traffic, pollution, habitat alteration, and provisioning.

Common bottlenose dolphins in the northern Gulf of Mexico were exposed to oil and dispersants following the Deepwater Horizon oil spill, which may result in long term reproductive and chronic health effects (NOAA 2011; Michel et al. 2013; Helm et al. 2015; DWH NRDA 2016; Lane et al. 2015; Aichinger Dias et al. 2017; De Guise et al. 2017, 2021; Kellar et al. 2017; Smith et al. 2017; Schwacke et al. 2017). An Unusual Mortality Event (UME), involving primarily bottlenose dolphins, was declared for cetaceans in the northern Gulf of Mexico from 2010 to 2014, with the Deepwater Horizon oil spill as the primary underlying cause (Litz et al. 2014; Schwacke et al. 2014; Venn-Watson et al. 2015a, 2015b).

The Gulf of Mexico northern coastal common bottlenose dolphin stock potentially interacts with six commercial fisheries (Southeastern U.S. Atlantic, Gulf of Mexico shrimp trawl; Gulf of Mexico menhaden purse seine; Gulf of Mexico gillnet; Southeastern U.S. Atlantic, Gulf of Mexico stone crab trap/pot; Gulf of Mexico blue crab trap/pot; and commercial passenger fishing vessel/hook-and-line; Hayes et al. 2022).

The nearshore and estuarine habitats along the Gulf of Mexico coast include waters adjacent to areas of high human population, some of which are highly industrialized, and areas of heavy agricultural impact. Studies of common bottlenose dolphin tissues from Gulf Coast waters along the Florida panhandle have shown persistent organic pollutants (POPs) in blubber and blood samples (Kucklick et al. 2011; Balmer et

al. 2018). Similarly, mercury has been detected in common bottlenose dolphin tissues in Florida Gulf Coast waters (Bryan et al. 2007; Woshner et al. 2008; McCormack et al. 2020a, 2020b, 2022).

An additional five UMEs in the Gulf of Mexico have overlapped with the northern coastal common bottlenose dolphin stock. The causes of these UMEs have included brevetoxin and dolphin morbillivirus (Litz et al. 2014). Although morbillivirus has been detected in populations of the northern Gulf of Mexico, it has not been attributed to large scale die-offs since 1993−1994 (Lipscomb et al. 1996; Litz et al. 2014; Van Bressem et al. 2014; Fauquier et al. 2017).

Distribution and Sightings

Density model results for common bottlenose dolphins in the Gulf of Mexico are presented by Roberts et al. (2015, 2016) and Mannocci et al. (2017).

Further Reading

Hayes, S.A., E. Josephson, K. Maze-Foley, P.E. Rosel, and J. Wallace. 2022. U.S. Atlantic and Gulf of Mexico marine mammal stock assessments 2021. National Oceanic and Atmospheric Administration, National Marine Fisheries Service, Northeast Fisheries Science Center: Woods Hole, MA. NOAA Technical Memorandum NMFS-NE-288. 380 p. doi: 10.25923/6tt7-kc16

Wells, R.S. and M.D. Scott. 2018. Bottlenose dolphin, *Tursiops truncatus*, common bottlenose dolphin. In: Würsig, B., J.G.M. Thewissen, and K.M. Kovacs (Eds.), Encyclopedia of Marine Mammals (Third Edition). Academic Press, London, UK. pp. 118–125. doi: 10.1016/B978-0-12-804327-1.00072-8

Wells, R.S., A. Natoli, and G. Braulik. 2019. *Tursiops truncatus* (errata version published in 2019). The IUCN Red List of Threatened Species 2019: e.T22563A156932432. https://dx.doi.org/10.2305/IUCN.UK.2019-1.RLTS.T22563A156932432.en

References

Aichinger Dias, L., J. Litz, L. Garrison, A. Martinez, K. Barry, and T. Speakman. 2017. Exposure of cetaceans to petroleum products following the Deepwater Horizon oil spill in the Gulf of Mexico. Endangered Species Research 33:119–125. doi: 10.3354/esr00770

Balmer, B., T. McDonald, F. Hornsby, J. Adams, J. Allen, A. Barleycorn, and L. Schwacke. 2018. Long-term trends in a northern Gulf of Mexico common bottlenose dolphin (*Tursiops truncatus*) population in the wake of the Deepwater Horizon oil spill. Journal of Cetacean Research and Management 18:1–9. doi: 10.47536/jcrm.v18i1.409

Balmer, B., S. Watwood, B. Quigley, T. Speakman, K. Barry, K. Mullin, P. Rosel, C. Sinclair, E. Zolman, and L. Schwacke. 2019. Common bottlenose dolphin (*Tursiops truncatus*) abundance and distribution patterns in St. Andrew Bay, Florida, USA. Aquatic Conservation: Marine and Freshwater Ecosystems 29(3):486–498. doi: 10.1002/aqc.3001

Balmer, B.C., R.S. Wells, S.M. Nowacek, D.P. Nowacek, L.H. Schwacke, W.A. McLellan, F.S. Scharf, T.K. Rowles, L.J. Hansen, T.R. Spradlin, and D.A. Pabst. 2008. Seasonal abundance and distribution patterns of common bottlenose dolphins (*Tursiops truncatus*) near St. Joseph Bay, Florida, USA. Journal of Cetacean Research and Management 10(2):157–167.

Barros, N.B. and D.K. Odell. 1990. Food habits of bottlenose dolphins in the southeastern United States In: Leatherwood, S. and R.R. Reeves (Eds.), The Bottlenose Dolphin. Academic Press, San Diego, CA. pp. 309–328. doi: 10.1016/B978-0-12-440280-5.50020-2

Bassos-Hull, K., R.M. Perrtree, C.C. Shepard, S. Schilling, A.A. Barleycorn, J.B. Allen, B.C. Balmer, W.E. Pine, and R.S. Wells. 2013. Long-term site fidelity and seasonal abundance estimates of common bottlenose dolphins (*Tursiops truncatus*) along the southwest coast of Florida and responses to natural perturbations. Journal of Cetacean Research and Management 13(1):19–30. doi: 10.47536/jcrm.v13i1.551

Berens McCabe, E.J., D.P. Gannon, N.B. Barros, and R.S. Wells. 2010. Prey selection by resident common bottlenose dolphins (*Tursiops truncatus*) in Sarasota Bay, Florida. Marine Biology 157(5):931–942. doi: 10.1007/s00227-009-1371-2

Bowen-Stevens, S.R., D.P. Gannon, R.A. Hazelkorn, G. Lovewell, K.M. Volker, S. Smith, M.C. Tumlin, and J. Litz. 2021. Diet of common bottlenose dolphins, *Tursiops truncatus*, that stranded in and near Barataria Bay, Louisiana, 2010–2012. Southeastern Naturalist 20(1). doi: 10.1656/058.020.0113

Bryan, C.E., S.J. Christopher, B.C. Balmer, and R.S. Wells. 2007. Establishing baseline levels of trace elements in blood and skin of bottlenose dolphins in Sarasota Bay, Florida: implications for non-invasive monitoring. Science of the Total Environment 388(1-3):325–342. doi: 10.1016/j.scitotenv.2007.07.046

Connor, R.C., R.S. Wells, J. Mann, and A.J. Read. 2000. The bottlenose dolphin: Social relationships in a fission-fusion society. In: Mann, J., R. Connor, P. Tyack, and H. Whitehead (Eds.), Cetacean Societies: Field Studies of Dolphins and Whales. University of Chicago Press, Chicago, IL. pp. 91–126.

De Guise, S., M. Levin, E. Gebhard, L. Jasperse, L. Burdett Hart, C.R. Smith, S. Venn-Watson, F. Townsend, R. Wells, B. Balmer, E. Zolman, T. Rowles, and L. Schwacke. 2017. Changes in immune functions in bottlenose dolphins in the northern Gulf of Mexico associated with the Deepwater Horizon oil spill. Endangered Species Research 33:291–303. doi: 10.3354/esr00814

De Guise, S., M. Levin, L. Jasperse, J. Herrman, R.S. Wells, T. Rowles, and L. Schwacke. 2021. Long-term immunological alterations in bottlenose dolphin a decade after the Deepwater Horizon oil spill in the northern Gulf of Mexico: Potential for multigenerational effects. Environmental Toxicology and Chemistry 40(5):1308–1321. doi: 10.1002/etc.4980

Deepwater Horizon Natural Resource Damage Assessment Trustees (DWH NRDA). 2016. Deepwater Horizon oil spill: Final Programmatic Damage Assessment and Restoration Plan and Final Programmatic Environmental Impact Statement. Retrieved from: http://www.gulfspillrestoration.noaa.gov/restoration-planning/gulf-plan.1659.

Fauquier, D.A., J. Litz, S. Sanchez, K. Colegrove, L.H. Schwacke, L. Hart, J. Saliki, C. Smith, T. Goldstein, S. Bowen-Stevens, W. McFee, E. Fougeres, B. Mase-Guthrie, E. Stratton, R. Ewing, S. Venn-Watson, R.H. Carmichael, C. Clemons-Chevis, W. Hatchett, D. Shannon, S. Shippee, S. Smith, L. Staggs, M.C. Tumlin, N.L. Wingers, and T.K. Rowles. 2017. Evaluation of morbillivirus exposure in cetaceans from the northern Gulf of Mexico 2010-2014. Endangered Species Research 33:211–220. doi: 10.3354/esr00772

Fernandez, S. and A.A. Hohn. 1998. Age, growth, and calving season of bottlenose dolphins, *Tursiops truncatus*, off coastal Texas. Fishery Bulletin 96(2):357–365.

Garrison, L.P., J. Ortega-Ortiz, and G. Rappucci. 2021. Abundance of coastal and continental shelf stocks of common bottlenose and Atlantic spotted dolphins in the northern Gulf of Mexico: 2017–2018. National Oceanic and Atmospheric Administration, National Marine Fisheries Service, Southeast Fisheries Science Center, Protected Resources and Biodiversity Division: Miami, FL. PRD-2021-01. 25 p. doi: 10.25923/vk95-t881

Hayes, S.A., E. Josephson, K. Maze-Foley, P.E. Rosel, and J. Wallace. 2022. U.S. Atlantic and Gulf of Mexico marine mammal stock assessments 2021. National Oceanic and Atmospheric Administration, National Marine Fisheries Service, Northeast Fisheries Science Center: Woods Hole, MA. NOAA Technical Memorandum NMFS-NE-288. 380 p. doi: 10.25923/6tt7-kc16

Helm, R.C., D.P. Costa, T.D. DeBruyn, T.J. O'Shea, R.S. Wells, and T.M. Williams. 2015. Overview of effects of oil spills on marine mammals. In: Fingas, M. (Ed.), Handbook of oil spill science and technology, First edition. John Wiley & Sons, Inc., Hoboken, NJ. pp. 455–475.

Henderson, E.E. and B. Würsig. 2007. Behavior patterns of bottlenose dolphins in San Luis Pass, Texas. Gulf of Mexico Science 25(2):153–161. doi: 10.18785/goms.2502.06

Hohn, A.A., M.D. Scott, R.S. Wells, J.C. Sweeney, and A.B. Irvine. 1989. Growth layers in teeth from known-age, free-ranging bottlenose dolphins. Marine Mammal Science 5(4):315–342. doi: 10.1111/j.1748-7692.1989.tb00346.x

Hubard, C.W., K. Maze-Foley, K.D. Mullin, and W.W. Schroeder. 2004. Seasonal abundance and site fidelity of bottlenose dolphins (*Tursiops truncatus*) in Mississippi Sound. Aquatic Mammals 30(2):299–310. doi: 10.1578/am.30.2.2004.299

Irwin, L.-J. and B. Würsig. 2004. A small resident community of bottlenose dolphins, *Tursiops truncatus*, in Texas: Monitoring recommendations. Gulf of Mexico Science 22(1):13–21. doi: 10.18785/goms.2201.02

Kellar, N.M., T.R. Speakman, C.R. Smith, S.M. Lane, B.C. Balmer, M.L. Trego, K.N. Catelani, M.N. Robbins, C.D. Allen, R.S. Wells, E.S. Zolman, T.K. Rowles, and L.H. Schwacke. 2017. Low reproductive success rates of common bottlenose dolphins *Tursiops truncatus* in the northern Gulf of Mexico following the Deepwater Horizon disaster (2010-2015). Endangered Species Research 33:143–158. doi: 10.3354/esr00775

Kucklick, J., L. Schwacke, R. Wells, A. Hohn, A. Guichard, J. Yordy, L. Hansen, E. Zolman, R. Wilson, J. Litz, D. Nowacek, T. Rowles, R. Pugh, B. Balmer, C. Sinclair, and P. Rosel. 2011. Bottlenose dolphins as indicators of persistent organic pollutants in the western North Atlantic Ocean and Northern Gulf of Mexico. Environmental Science & Technology 45(10):4270–4277. doi: 10.1021/es1042244

LaBrecque, E., C. Curtice, J. Harrison, S.M. Van Parijs, and P.N. Halpin. 2015. 3. Biologically Important Areas for cetaceans within U.S. Waters – Gulf of Mexico Region. Aquatic Mammals 41(1):30–38. doi: 10.1578/am.41.1.2015.30

Lane, S.M., C.R. Smith, J. Mitchell, B.C. Balmer, K.P. Barry, T. McDonald, C.S. Mori, P.E. Rosel, T.K. Rowles, T.R. Speakman, F.I. Townsend, M.C. Tumlin, R.S. Wells, E.S. Zolman, and L.H. Schwacke. 2015. Reproductive outcome and survival of common bottlenose dolphins sampled in Barataria Bay, Louisiana, USA, following the Deepwater Horizon oil spill. Proceedings of the Royal Society B: Biological Sciences 282(1818):20151944. doi: 10.1098/rspb.2015.1944

Leatherwood, S. 1977. Some preliminary impressions on the numbers and social behavior of free-swimming bottlenosed dolphin calves (*Tursiops truncatus*) in the northern Gulf of Mexico. In: Ridgway, S.H. and K. Bernirschke (Eds.), Breeding Dolphins; Present Status, Suggestions for the Future. Final report to the US Marine Mammal Commission, Contract MM6AC009, MMC-76/07, Washington, DC. pp. 143–167.

Lipscomb, T.P., S. Kennedy, D. Moffett, A. Krafft, B.A. Klaunberg, J.H. Lichy, G.T. Regan, G.A.J. Worthy, and J.K. Taubenberger. 1996. Morbilliviral epizootic in bottlenose dolphins of the Gulf of Mexico. Journal of Veterinary Diagnostic Investigation 8(3):283–290. doi: 10.1177/104063879600800302

Litz, J.A., M.A. Baran, S.R. Bowen-Stevens, R.H. Carmichael, K.M. Colegrove, L.P. Garrison, S.E. Fire, E.M. Fougeres, R. Hardy, S. Holmes, W. Jones, B.E. Mase-Guthrie, D.K. Odell, P.E. Rosel, J.T. Saliki, D.K. Shannon, S.F. Shippee, S.M. Smith, E.M. Stratton, M.C. Tumlin, H.R. Whitehead, G.A. Worthy, and T.K. Rowles. 2014. Review of historical unusual mortality events (UMEs) in the Gulf of Mexico (1990–2009): providing context for the multi-year northern Gulf of Mexico cetacean UME declared in 2010. Diseases of Aquatic Organisms 112(2):161–175. doi: 10.3354/dao02807

Lynn, S.K. and B. Würsig. 2002. Summer movement patterns of bottlenose dolphins in a Texas bay. Gulf of Mexico Science 20(1):25–37. doi: 10.18785/goms.2001.03

Mackey, A.D. 2010. Site fidelity and association patterns of bottlenose dolphins (*Tursiops truncatus*) in the Mississippi Sound. University of Southern Mississippi. Thesis. 133 p.

Mann, J., R.C. Connor, L.M. Barre, and M.R. Heithaus. 2000. Female reproductive success in bottlenose dolphins (*Tursiops* sp.): life history, habitat, provisioning, and group-size effects. Behavioral Ecology 11(2):210–219. doi: 10.1093/beheco/11.2.210

Mannocci, L., J.J. Roberts, D.L. Miller, and P.N. Halpin. 2017. Extrapolating cetacean densities to quantitatively assess human impacts on populations in the high seas. Conservation Biology 31(3):601–614. doi: 10.1111/cobi.12856

Mattson, M.C., K.D. Mullin, G.W. Ingram, and W. Hoggard. 2006. Age structure and growth of the bottlenose dolphin (*Tursiops truncatus*) from strandings in the Mississippi sound region of the north-central Gulf of Mexico from 1986 to 2003. Marine Mammal Science 22(3):654–666. doi: 10.1111/j.1748-7692.2006.00057.x

Maze, K.S. and B. Würsig. 1999. Bottlenosed dolphins of San Luis Pass, Texas: Occurrence patterns, site-fidelity, and habitat use. Aquatic Mammals 25:91–104.

McCormack, M.A., F. Battaglia, W.E. McFee, and J. Dutton. 2020a. Mercury concentrations in blubber and skin from stranded bottlenose dolphins (*Tursiops truncatus*) along the Florida and Louisiana coasts (Gulf of Mexico, USA) in relation to biological variables. Environmental Research 180:108886. doi: 10.1016/j.envres.2019.108886

McCormack, M.A., B.P. Jackson, and J. Dutton. 2020b. Relationship between mercury and selenium concentrations in tissues from stranded odontocetes in the northern Gulf of Mexico. Science of the Total Environment 749:141350. doi: 10.1016/j.scitotenv.2020.141350

McCormack, M.A., W.H. Nowlin, and J. Dutton. 2022. Effect of trophic position on mercury concentrations in bottlenose dolphins (*Tursiops truncatus*) from the northern Gulf of Mexico. Environmental Research 204(Pt B):112124. doi: 10.1016/j.envres.2021.112124

McDonald, T.L., F.E. Hornsby, T.R. Speakman, E.S. Zolman, K.D. Mullin, C. Sinclair, P.E. Rosel, L. Thomas, and L.H. Schwacke. 2017. Survival, density, and abundance of common bottlenose dolphins in Barataria Bay (USA) following the Deepwater Horizon oil spill. Endangered Species Research 33:193–209. doi: 10.3354/esr00806

McHugh, M.B. 1989. Population numbers and feeding behavior of the Atlantic bottlenose dolphin (*Tursiops truncatus*) near Aransas Pass, Texas. University of Texas at Austin. Thesis. 96 p.

Mead, J.G. and C.W. Potter. 1990. Natural history of bottlenose dolphins along the central Atlantic coast of the United States. In: Leatherwood, S. and R.R. Reeves (Eds.), The Bottlenose Dolphin. Academic Press, San Diego, CA. pp. 165–195.

Michel, J., E.H. Owens, S. Zengel, A. Graham, Z. Nixon, T. Allard, W. Holton, P.D. Reimer, A. Lamarche, M. White, N. Rutherford, C. Childs, G. Mauseth, G. Challenger, and E. Taylor. 2013. Extent and degree of shoreline oiling: Deepwater Horizon oil spill, Gulf of Mexico, USA. PLoS ONE 8(6):e65087. doi: 10.1371/journal.pone.0065087

Miller, C.E. 2003. Abundance trends and environmental habitat usage patterns of bottlenose dolphins (*Tursiops truncatus*) in lower Barataria and Caminada bays, Louisiana. Louisiana State University and Agricultural & Mechanical College. Dissertation. 125 p.

Miller, L.J., A.D. Mackey, T. Hoffland, M. Solangi, and S.A. Kuczaj. 2010. Potential effects of a major hurricane on Atlantic bottlenose dolphin (*Tursiops truncatus*) reproduction in the Mississippi Sound. Marine Mammal Science 26(3):707–715. doi: 10.1111/j.1748-7692.2010.00371.x

Miller, L.J., A.D. Mackey, M. Solangi, and S.A. Kuczaj. 2013. Population abundance and habitat utilization of bottlenose dolphins in the Mississippi Sound. Aquatic Conservation: Marine and Freshwater Ecosystems 23(1):145–151. doi: 10.1002/aqc.2278

Mullin, K.D., T. McDonald, R.S. Wells, B.C. Balmer, T. Speakman, C. Sinclair, E.S. Zolman, F. Hornsby, S.M. McBride, K.A. Wilkinson, and L.H. Schwacke. 2017. Density, abundance, survival, and ranging

patterns of common bottlenose dolphins (*Tursiops truncatus*) in Mississippi Sound following the Deepwater Horizon oil spill. PLoS ONE 12(10):e0186265. doi: 10.1371/journal.pone.0186265

National Oceanic and Atmospheric Administration (NOAA). 2011. Public scoping for preparation of a programmatic environmental impact statement for the Deepwater Horizon BP Oil Spill. 9 p.

Perrin, W.F. and S.B. Reilly. 1984. Reproductive parameters of dolphins and small whales of the family Delphinidae. In: Perrin, W.F., R.L. Brownell, Jr., and D.P. DeMaster (Eds.), Reproduction in Whales, Dolphins, and Porpoises. pp. 97–134.

Roberts, J.J., B.D. Best, L. Mannocci, E. Fujioka, P.N. Halpin, D.L. Palka, L.P. Garrison, K.D. Mullin, T.V.N. Cole, C.B. Khan, W.A. McLellan, D.A. Pabst, and G.G. Lockhart. 2016. Habitat-based cetacean density models for the US Atlantic and Gulf of Mexico. Scientific Reports 6:22615. doi: 10.1038/Srep22615

Roberts, J.J., B.D. Best, L. Mannocci, E. Fujioka, P.N. Halpin, D.L. Palka, L.P. Garrison, K.D. Mullin, T.V.N. Cole, C.B. Khan, W.M. McLellan, D.A. Pabst, and G.G. Lockhart. 2015. Density model for bottlenose dolphin (*Tursiops truncatus*) for the U.S. Gulf of Mexico version 3.3, 2015-10-07, and supplementary report. Duke University, Marine Geospatial Ecology Lab: Durham, NC. 84 p.

Rosel, P.E., L.A. Wilcox, C. Sinclair, T.R. Speakman, M.C. Tumlin, J.A. Litz, and E.S. Zolman. 2017. Genetic assignment to stock of stranded common bottlenose dolphins in southeastern Louisiana after the Deepwater Horizon oil spill. Endangered Species Research 33:221–234. doi: 10.3354/esr00780

Rossman, S., P.H. Ostrom, M. Stolen, N.B. Barros, H. Gandhi, C.A. Stricker, and R.S. Wells. 2015. Individual specialization in the foraging habits of female bottlenose dolphins living in a trophically diverse and habitat rich estuary. Oecologia 178(2):415–425. doi: 10.1007/s00442-015-3241-6

Schwacke, L.H., C.R. Smith, F.I. Townsend, R.S. Wells, L.B. Hart, B.C. Balmer, T.K. Collier, S. De Guise, M.M. Fry, L.J. Guillette, Jr., S.V. Lamb, S.M. Lane, W.E. McFee, N.J. Place, M.C. Tumlin, G.M. Ylitalo, E.S. Zolman, and T.K. Rowles. 2014. Health of common bottlenose dolphins (*Tursiops truncatus*) in Barataria Bay, Louisiana, following the deepwater horizon oil spill. Environmental Science & Technology 48(1):93–103. doi: 10.1021/es403610f

Schwacke, L.H., L. Thomas, R.S. Wells, W.E. McFee, A.A. Hohn, K.D. Mullin, E.S. Zolman, B.M. Quigley, T.K. Rowles, and J.H. Schwacke. 2017. Quantifying injury to common bottlenose dolphins from the Deepwater Horizon oil spill using an age-, sex- and class-structured population model. Endangered Species Research 33:265–279. doi: 10.3354/esr00777

Sellas, A.B., R.S. Wells, and P.E. Rosel. 2005. Mitochondrial and nuclear DNA analyses reveal fine scale geographic structure in bottlenose dolphins (*Tursiops truncatus*) in the Gulf of Mexico. Conservation Genetics 6(5):715–728. doi: 10.1007/s10592-005-9031-7

Sergeant, D.E., D.K. Caldwell, and M.C. Caldwell. 1973. Age, growth, and maturity of bottlenosed dolphin (*Tursiops-truncatus*) from northeast Florida. Journal of the Fisheries Research Board of Canada 30(7):1009–1011. doi: 10.1139/f73-165

Shane, S.H., R.S. Wells, and B. Würsig. 1986. Ecology, behavior and social organization of the bottlenose dolphin: A review. Marine Mammal Science 2:34–63. doi: 10.1111/j.1748-7692.1986.tb00026.x

Smith, C.R., T.K. Rowles, L.B. Hart, F.I. Townsend, R.S. Wells, E.S. Zolman, B.C. Balmer, B. Quigley, M. Ivančić, W. McKercher, M.C. Tumlin, K.D. Mullin, J.D. Adams, Q. Wu, W. McFee, T.K. Collier, and L.H. Schwacke. 2017. Slow recovery of Barataria Bay dolphin health following the Deepwater Horizon oil spill (2013-2014), with evidence of persistent lung disease and impaired stress response. Endangered Species Research 33:127–142. doi: 10.3354/esr00778

Takeshita, R., B.C. Balmer, F. Messina, E.S. Zolman, L. Thomas, R.S. Wells, C.R. Smith, T.K. Rowles, and L.H. Schwacke. 2021. High site-fidelity in common bottlenose dolphins despite low salinity

exposure and associated indicators of compromised health. PLoS ONE 16(9):e0258031. doi: 10.1371/journal.pone.0258031
Taylor, B.L., S.J. Chivers, J. Larese, and W.F. Perrin. 2007. Generation length and percent mature estimates for IUCN assessments of cetaceans. National Oceanic and Atmospheric Administration, National Marine Fisheries Service, Southwest Fisheries Science Center: La Jolla, CA. Administrative Report LJ-07-01. 24 p.
Torres, L.G. and A.J. Read. 2009. Where to catch a fish? The influence of foraging tactics on the ecology of bottlenose dolphins (*Tursiops truncatus*) in Florida Bay, Florida. Marine Mammal Science 25(4):797–815. doi: 10.1111/j.1748-7692.2009.00297.x
Tyson, R.B., S.M. Nowacek, and D.P. Nowacek. 2011. Community structure and abundance of bottlenose dolphins *Tursiops truncatus* in coastal waters of the northeast Gulf of Mexico. Marine Ecology Progress Series 438:253–265. doi: 10.3354/meps09292
Urian, K.W., S. Hofmann, R.S. Wells, and A.J. Read. 2009. Fine-scale population structure of bottlenose dolphins (*Tursiops truncatus*) in Tampa Bay, Florida. Marine Mammal Science 25(3):619–638. doi: 10.1111/j.1748-7692.2009.00284.x
Van Bressem, M.F., P.J. Duignan, A. Banyard, M. Barbieri, K.M. Colegrove, S. De Guise, G. Di Guardo, A. Dobson, M. Domingo, D. Fauquier, A. Fernandez, T. Goldstein, B. Grenfell, K.R. Groch, F. Gulland, B.A. Jensen, P.D. Jepson, A. Hall, T. Kuiken, S. Mazzariol, S.E. Morris, O. Nielsen, J.A. Raga, T.K. Rowles, J. Saliki, E. Sierra, N. Stephens, B. Stone, I. Tomo, J. Wang, T. Waltzek, and J.F. Wellehan. 2014. Cetacean morbillivirus: current knowledge and future directions. Viruses 6(12):5145–5181. doi: 10.3390/v6125145
Venn-Watson, S., L. Garrison, J. Litz, E. Fougeres, B. Mase, G. Rappucci, E. Stratton, R. Carmichael, D. Odell, D. Shannon, S. Shippee, S. Smith, L. Staggs, M. Tumlin, H. Whitehead, and T. Rowles. 2015. Demographic clusters identified within the northern Gulf of Mexico common bottlenose dolphin (*Tursiops truncates*) unusual mortality event: January 2010-June 2013. PLoS ONE 10(2):e0117248. doi: 10.1371/journal.pone.0117248
Vollmer, N.L. and P.E. Rosel. 2013. A review of common bottlenose dolphins (*Tursiops truncatus truncatus*) in the Northern Gulf of Mexico: Population biology, potential threats, and management. Southeastern Naturalist 12(m6):1–43. doi: 10.1656/058.012.m601
Vollmer, N.L. and P.E. Rosel. 2017. Fine-scale population structure of common bottlenose dolphins (*Tursiops truncatus*) in offshore and coastal waters of the US Gulf of Mexico. Marine Biology 164(8):160. doi: 10.1007/s00227-017-3186-x
Waring, G.T., E. Josephson, K. Maze-Foley, and P.E. Rosel. 2016. U.S. Atlantic and Gulf of Mexico marine mammal stock assessments - 2015. National Oceanic and Atmospheric Administration, National Marine Fisheries Service, Northeast Fisheries Science Center: Woods Hole, MA. NOAA Technical Memorandum NMFS-NE-238. 501 p. doi: 10.7289/V57S7KTN
Wells, R.S. 1986. Population structure of bottlenose dolphins: Behavioral studies along the central west coast of Florida. Contract Report to National Marine Fisheries Service, Southeast Fisheries Center. Contract No. 45-WCNF-5-003BB. 58 p.
Wells, R.S. 1991. The role of long-term study in understanding the social structure of a bottlenose dolphin community. In: Pryor, K. and K. Norris (Eds.), Dolphin Societies: Discoveries and Puzzles. University of California Press, Berkeley, CA. pp. 199–226.
Wells, R.S. 2000. Reproduction in wild bottlenose dolphins: Overview of patterns observed during a long-term study. In: Duffield, D. and T. Robeck (Eds.), Bottlenose Dolphin Reproduction Workshop. AZA Marine Mammal Taxon Advisory Group, Silver Spring, MD. pp. 57–74.
Wells, R.S. 2003. Dolphin social complexity: Lessons from long-term study and life history. In: de Waal, F.B.M. and P.L. Tyack (Eds.), Animal Social Complexity: Intelligence, Culture, and Individualized Societies Harvard University Press, Cambridge, MA. pp. 32–56.

Wells, R.S. 2014. Social structure and life history of bottlenose dolphins near Sarasota Bay, Florida: Insights from four decades and five generations. Primates and cetaceans. pp. 149–172. doi: 10.1007/978-4-431-54523-1_8

Wells, R.S., A. Natoli, and G. Braulik. 2019. *Tursiops truncatus* (errata version published in 2019). *The IUCN Red List of Threatened Species* 2019: e.T22563A156932432. https://dx.doi.org/10.2305/IUCN.UK.2019-1.RLTS.T22563A156932432.en

Wells, R.S., L.H. Schwacke, T.K. Rowles, B.C. Balmer, E. Zolman, T. Speakman, F.I. Townsend, M.C. Tumlin, A. Barleycorn, and K.A. Wilkinson. 2017. Ranging patterns of common bottlenose dolphins *Tursiops truncatus* in Barataria Bay, Louisiana, following the Deepwater Horizon oil spill. Endangered Species Research 33:159–180. doi: 10.3354/esr00732

Wells, R.S. and M.D. Scott. 1999. Bottlenose dolphin *Tursiops truncatus* (Montagu, 1821). In: Ridgway, S.H. and R. Harrison (Eds.), Handbook of marine mammals. Volume 6: The second book of dolphins and the porpoises. Academic Press, San Diego, CA. pp. 137–182.

Wells, R.S. and M.D. Scott. 2018. Bottlenose dolphin, *Tursiops truncatus*, common bottlenose dolphin. In: Würsig, B., J.G.M. Thewissen, and K.M. Kovacs (Eds.), Encyclopedia of Marine Mammals (Third Edition). Academic Press, London, UK. pp. 118–125. doi: 10.1016/B978-0-12-804327-1.00072-8

Wells, R.S., M.D. Scott, and A.B. Irvine. 1987. The social structure of free-ranging bottlenose dolphins. In: Genoways, H.H. (Ed.), Current Mammalogy. Plenum Press, New York, NY. pp. 247–305.

Wells, R.S., K.W. Urian, A.J. Read, M.K. Bassos, W.J. Carr, and M.D. Scott. 1996. Low-level monitoring of bottlenose dolphins, *Tursiops truncatus*, in Tampa Bay, Florida: 1988-1993. National Oceanic and Atmospheric Administration, National Marine Fisheries Service, Southeast Fisheries Science Center: Miami, FL. NOAA Technical Memorandum NMFS-SEFSC-385. 36 p.

Wilson, R.M., R.B. Tyson, J.A. Nelson, B.C. Balmer, J.P. Chanton, and D.P. Nowacek. 2017. Niche differentiation and prey selectivity among common bottlenose dolphins (*Tursiops truncatus*) sighted in St. George Sound, Gulf of Mexico. Frontiers in Marine Science 4:235. doi: 10.3389/fmars.2017.00235

Woshner, V., K. Knott, R. Wells, C. Willetto, R. Swor, and T. O'Hara. 2008. Mercury and selenium in blood and epidermis of bottlenose dolphins (*Tursiops truncatus*) from Sarasota Bay, FL: interaction and relevance to life history and hematologic parameters. Ecohealth 5(3):360–370. doi: 10.1007/s10393-008-0164-2

Common bottlenose dolphin − *Tursiops truncatus*

Gulf of Mexico, Oceanic Stock

Overall Vulnerability Rank = High

Biological Sensitivity = Moderate

Climate Exposure = Very High

Data Quality = 85% of scores ≥ 2

	Tursiops truncatus	Expert Scores	Data Quality	Expert Scores Plots (Portion by Category)
Sensitivity attributes	Prey/Diet Specificity	2.1	2.0	
	Habitat Specificity	1.9	2.0	
	Site Fidelity	2.1	1.7	
	Lifetime Reproductive Potential	1.3	2.3	
	Generation Length	2.8	2.3	
	Reproductive Plasticity	1.3	1.7	
	Migration	3.6	2.0	
	Home Range	2.1	1.7	
	Stock Abundance	1.9	2.7	
	Stock Abundance Trend	2.3	1.3	
	Cumulative Stressors	2.2	2.3	
	Sensitivity Score	Moderate		
Exposure variables	Sea Surface Temperature (Change in mean)	4.0	2.7	
	Sea Surface Temperature (Change in variability)	1.0	2.7	
	Air Temperature (Change in mean)	4.0	2.7	
	Air Temperature (Change in variability)	1.0	2.7	
	Precipitation (Change in mean)	1.0	2.7	
	Precipitation (Change in variability)	1.0	2.7	
	Sea Surface Salinity (Change in mean)	2.3	2.3	
	Sea Surface Salinity (Change in variability)	1.3	2.3	
	Ocean pH (Change in mean)	4.0	2.7	
	Ocean pH (Change in variability)	1.0	2.7	
	Sea ice coverage (Change in mean)	1.0	2.7	
	Sea ice coverage (Change in variability)	1.0	2.7	
	Dissolved oxygen (Change in mean)	4.0	2.7	
	Dissolved oxygen (Change in variability)	1.0	2.7	
	Circulation	1.7	2.3	
	Sea level rise	1.3	2.7	
	Exposure Score	Very High		
Overall Vulnerability Rank		High		

Low
Moderate
High
Very High

Common bottlenose dolphin (Gulf of Mexico, Oceanic Stock)

Tursiops truncatus

CVA Results Summary

Overall Climate Vulnerability Rank: High (96% certainty from bootstrap analysis).

Climate Exposure: Very High. Four exposure factors scored greater than 3.5: Air Temperature (Standard anomaly) (4.00), Dissolved oxygen (Standard anomaly) (4.00), Ocean pH (Standard anomaly) (4.00), and Sea Surface Temperature (Standard anomaly) (4.00).

Biological Sensitivity: Moderate. Two sensitivity attributes scored greater than 2.5: Migration (3.60) and Generation Time (2.80).

Distributional Response: Moderate

Abundance Response: Low

Phenology Response: Low

Data Quality: 85% of the data quality scores were 2 or greater, 64% of sensitivity attribute data quality scores were 2 or higher.

Background Information

Stock Description

This stock includes common bottlenose dolphins in oceanic waters seaward from the 200-m isobath in the U.S. Exclusive Economic Zone and adjacent offshore waters of the Gulf of Mexico (Hayes et al. 2021).

Prey/Diet Specificity

Generally, common bottlenose dolphins consume a large variety of fish and/or squid (Barros and Odell 1990; Mead and Potter 1990; Wells and Scott 2018). Common bottlenose dolphins seem to show a consistent preference for sound-producing fishes (e.g., sciaenids, toadfish; Berens McCabe et al. 2010; Bowen-Stevens et al. 2021). In Sarasota Bay, Florida, Rossman et al. (2015) found some individual common bottlenose dolphins to show diet specialization despite the broader study group showing a generalist diet. Specialization can be influenced by locally abundant prey species (Shane et al. 1986; Vollmer and Rosel 2013) or can result from resource partitioning amongst sympatric populations (e.g., Torres and Read 2009; Wilson et al. 2017).

Habitat Specificity

Common bottlenose dolphins are able to adapt to a variety of marine and estuarine habitats (Wells and Scott 2018).

Site Fidelity

No specific site fidelity information was found for this stock.

Lifetime Reproductive Potential

Taylor et al. (2007) reported the interbirth interval of common bottlenose dolphins as 3.80 years, though other studies have shown it to be closer to three years (Leatherwood 1977; Wells and Scott 1999; Connor et al. 2000; Wells 2003).

Generally, females reach sexual maturity before males, with females reaching sexual maturity at 5–13 years (Sergeant et al. 1973; Perrin and Reilly 1984; Wells et al. 1987; Mead and Potter 1990; Mann et al. 2000; Wells 2000; Wells and Scott 2018).

Females reproduce throughout their adult lives (Leatherwood 1977; Hohn et al. 1989; Wells and Scott 1999; Wells 2000; Wells 2003), but may produce fewer calves after they reach 30 years of age (Wells 2000). Sarasota common bottlenose dolphins can give birth when they are as young as 6 years of age, and can continue until they are 48 years old (Wells and Scott 2018). Reproductive lifespan would therefore be up to 42 years based on long-term observation studies (Wells 2014; Wells and Scott 2018).

Longitudinal studies from Sarasota Bay, Florida have found that female common bottlenose dolphins can live up to 67 years (Wells and Scott 2018). Studies based on growth layer groups in teeth suggest female common bottlenose dolphins can live to 41 years along the Texas coast (Fernandez and Hohn 1998) and to 30 years along the Mississippi coast (Mattson et al. 2006).

Generation Length

Taylor et al. (2007) reported common bottlenose dolphin generation length of 20.6–21.1 years.

Reproductive Plasticity

Common bottlenose dolphin calving is generally diffusely seasonal, with one or more peaks, although births have been reported from all seasons (Vollmer and Rosel 2013; Wells and Scott 2018). Calf sightings peak in the spring and summer months in Florida (Wells et al. 1987), Mississippi/Louisiana (Miller et al. 2010; Miller et al. 2013), and Texas (McHugh 1989; Henderson and Würsig 2007).

Migration

Data are insufficient to characterize migration patterns (or the lack thereof) for this stock. However, genetic data do support the presence of multiple differentiated populations of common bottlenose dolphins in coastal and offshore waters with very low levels of migration among them (Vollmer and Rosel 2017). Molecular genetic data further support that coastal populations are genetically differentiated from bay, sound, and estuary (BSE) populations (Sellas et al. 2005; Rosel et al. 2017).

Home Range

Data are insufficient to characterize home range patterns (or the lack thereof) for this stock.

Stock Abundance

The abundance of this stock is estimated to be 7,462 individuals (CV=0.31) based on 2017 and 2018 surveys (Garrison et al. 2020; Hayes et al. 2021). Previously, the stock was estimated at 5,806 individuals (CV=0.39) based on 2009 oceanic surveys (Waring et al. 2016).

Stock Abundance Trend

Five point estimates of bottlenose dolphin abundance have been made based on data from surveys in 2003 (21,350; CV=0.47), 2004 (8,864; CV=0.50), 2009 (9,640; CV=0.66), 2017 (8,756; CV=0.41), and 2018 (5,833; CV=0.46). Pairwise comparisons of the log-transformed means were conducted between years, and there were no significant differences between survey years (see Garrison et al. 2020 and Hayes et al. 2021).

Cumulative Stressors

Common bottlenose dolphins face a wide range of individual and cumulative natural and anthropogenic threats (see reviews by Vollmer and Rosel 2013; Wells and Scott 2018; and Wells et al. 2019). These include such threats as shark predation, harmful algal blooms, recreational and commercial fishing gear, vessel traffic, pollution, habitat alteration, and provisioning.

Common bottlenose dolphins in the northern Gulf of Mexico were exposed to oil and dispersants following the Deepwater Horizon oil spill, which may result in long term reproductive and chronic health effects (NOAA 2011; Michel et al. 2013; Helm et al. 2015; DWH NRDA 2016; Lane et al. 2015; Aichinger Dias et al. 2017; De Guise et al. 2017, 2021; Kellar et al. 2017; Smith et al. 2017; Schwacke et al. 2017). An Unusual Mortality Event (UME), involving primarily bottlenose dolphins, was declared for cetaceans in the northern Gulf of Mexico from 2010 to 2014, with the Deepwater Horizon oil spill as the primary underlying cause (Litz et al. 2014; Schwacke et al. 2014; Venn-Watson et al. 2015a, 2015b). An Unusual Mortality Event (UME) was declared in 1990 for the Gulf of Mexico from Florida to Texas, with more than 300 common bottlenose dolphins stranding due to an undetermined cause, although morbillivirus and cold temperatures may have contributed (Litz et al. 2014).

Distribution and Sightings

Density model results for common bottlenose dolphins in the Gulf of Mexico are presented by Roberts et al. (2015, 2016) and Mannocci et al. (2017).

Further Reading

Hayes, S.A., E. Josephson, K. Maze-Foley, P.E. Rosel, and J. Turek. 2021. US Atlantic and Gulf of Mexico Marine Mammal Stock Assessments - 2020. National Oceanic and Atmospheric Administration, National Marine Fisheries Service, Northeast Fisheries Science Center: Woods Hole, MA. NOAA Technical Memorandum NMFS-NE-271. 394 p.

Wells, R.S. and M.D. Scott. 2018. Bottlenose dolphin, *Tursiops truncatus*, common bottlenose dolphin. In: Würsig, B., J.G.M. Thewissen, and K.M. Kovacs (Eds.), Encyclopedia of Marine Mammals (Third Edition). Academic Press, London, UK. pp. 118–125. doi: 10.1016/B978-0-12-804327-1.00072-8

Wells, R.S., A. Natoli, and G. Braulik. 2019. *Tursiops truncatus* (errata version published in 2019). The IUCN Red List of Threatened Species 2019: e.T22563A156932432. https://dx.doi.org/10.2305/IUCN.UK.2019-1.RLTS.T22563A156932432.en

References

Aichinger Dias, L., J. Litz, L. Garrison, A. Martinez, K. Barry, and T. Speakman. 2017. Exposure of cetaceans to petroleum products following the Deepwater Horizon oil spill in the Gulf of Mexico. Endangered Species Research 33:119–125. doi: 10.3354/esr00770

Barros, N.B. and D.K. Odell. 1990. Food habits of bottlenose dolphins in the southeastern United States In: Leatherwood, S. and R.R. Reeves (Eds.), The Bottlenose Dolphin. Academic Press, San Diego, CA. pp. 309–328. doi: 10.1016/B978-0-12-440280-5.50020-2

Berens McCabe, E.J., D.P. Gannon, N.B. Barros, and R.S. Wells. 2010. Prey selection by resident common bottlenose dolphins (*Tursiops truncatus*) in Sarasota Bay, Florida. Marine Biology 157(5):931–942. doi: 10.1007/s00227-009-1371-2

Bowen-Stevens, S.R., D.P. Gannon, R.A. Hazelkorn, G. Lovewell, K.M. Volker, S. Smith, M.C. Tumlin, and J. Litz. 2021. Diet of common bottlenose dolphins, *Tursiops truncatus*, that stranded in and near Barataria Bay, Louisiana, 2010–2012. Southeastern Naturalist 20(1). doi: 10.1656/058.020.0113

Connor, R.C., R.S. Wells, J. Mann, and A.J. Read. 2000. The bottlenose dolphin: Social relationships in a fission-fusion society. In: Mann, J., R. Connor, P. Tyack, and H. Whitehead (Eds.), Cetacean Societies: Field Studies of Dolphins and Whales. University of Chicago Press, Chicago, IL. pp. 91–126.

De Guise, S., M. Levin, E. Gebhard, L. Jasperse, L. Burdett Hart, C.R. Smith, S. Venn-Watson, F. Townsend, R. Wells, B. Balmer, E. Zolman, T. Rowles, and L. Schwacke. 2017. Changes in immune functions in bottlenose dolphins in the northern Gulf of Mexico associated with the Deepwater Horizon oil spill. Endangered Species Research 33:291–303. doi: 10.3354/esr00814

De Guise, S., M. Levin, L. Jasperse, J. Herrman, R.S. Wells, T. Rowles, and L. Schwacke. 2021. Long-term immunological alterations in bottlenose dolphin a decade after the Deepwater Horizon oil spill in the northern Gulf of Mexico: Potential for multigenerational effects. Environmental Toxicology and Chemistry 40(5):1308–1321. doi: 10.1002/etc.4980

Deepwater Horizon Natural Resource Damage Assessment Trustees (DWH NRDA). 2016. Deepwater Horizon oil spill: Final Programmatic Damage Assessment and Restoration Plan and Final Programmatic Environmental Impact Statement. Retrieved from: http://www.gulfspillrestoration.noaa.gov/restoration-planning/gulf-plan.1659.

Fernandez, S. and A.A. Hohn. 1998. Age, growth, and calving season of bottlenose dolphins, *Tursiops truncatus*, off coastal Texas. Fishery Bulletin 96(2):357–365.

Garrison, L.P., J. Ortega-Ortiz, and G. Rappucci. 2020. Abundance of marine mammals in waters of the U.S. Gulf of Mexico during the summers of 2017 and 2018. National Oceanic and Atmospheric Administration, National Marine Fisheries Service, Southeast Fisheries Science Center: Miami, FL. Southeast Fisheries Science Center Reference Document PRBD-2020-07. 56 p. doi: 10.25923/3px6-9v48

Hayes, S.A., E. Josephson, K. Maze-Foley, P.E. Rosel, and J. Turek. 2021. US Atlantic and Gulf of Mexico marine mammal stock assessments - 2020. National Oceanic and Atmospheric Administration, National Marine Fisheries Service, Northeast Fisheries Science Center: Woods Hole, MA. NOAA Technical Memorandum NMFS-NE-271. 394 p.

Helm, R.C., D.P. Costa, T.D. DeBruyn, T.J. O'Shea, R.S. Wells, and T.M. Williams. 2015. Overview of effects of oil spills on marine mammals. In: Fingas, M. (Ed.), Handbook of oil spill science and technology, First edition. John Wiley & Sons, Inc., Hoboken, NJ. pp. 455–475.

Henderson, E.E. and B. Würsig. 2007. Behavior patterns of bottlenose dolphins in San Luis Pass, Texas. Gulf of Mexico Science 25(2):153–161. doi: 10.18785/goms.2502.06

Hohn, A.A., M.D. Scott, R.S. Wells, J.C. Sweeney, and A.B. Irvine. 1989. Growth layers in teeth from known-age, free-ranging bottlenose dolphins. Marine Mammal Science 5(4):315–342. doi: 10.1111/j.1748-7692.1989.tb00346.x

Kellar, N.M., T.R. Speakman, C.R. Smith, S.M. Lane, B.C. Balmer, M.L. Trego, K.N. Catelani, M.N. Robbins, C.D. Allen, R.S. Wells, E.S. Zolman, T.K. Rowles, and L.H. Schwacke. 2017. Low reproductive success rates of common bottlenose dolphins *Tursiops truncatus* in the northern Gulf of Mexico following the Deepwater Horizon disaster (2010-2015). Endangered Species Research 33:143–158. doi: 10.3354/esr00775

Lane, S.M., C.R. Smith, J. Mitchell, B.C. Balmer, K.P. Barry, T. McDonald, C.S. Mori, P.E. Rosel, T.K. Rowles, T.R. Speakman, F.I. Townsend, M.C. Tumlin, R.S. Wells, E.S. Zolman, and L.H. Schwacke. 2015. Reproductive outcome and survival of common bottlenose dolphins sampled in Barataria Bay, Louisiana, USA, following the Deepwater Horizon oil spill. Proceedings of the Royal Society B: Biological Sciences 282(1818):20151944. doi: 10.1098/rspb.2015.1944

Leatherwood, S. 1977. Some preliminary impressions on the numbers and social behavior of free-swimming bottlenosed dolphin calves (*Tursiops truncatus*) in the northern Gulf of Mexico. In: Ridgway, S.H. and K. Bernirschke (Eds.), Breeding Dolphins; Present Status, Suggestions for the Future. Final report to the US Marine Mammal Commission, Contract MM6AC009, MMC-76/07, Washington, DC. pp. 143–167.

Litz, J.A., M.A. Baran, S.R. Bowen-Stevens, R.H. Carmichael, K.M. Colegrove, L.P. Garrison, S.E. Fire, E.M. Fougeres, R. Hardy, S. Holmes, W. Jones, B.E. Mase-Guthrie, D.K. Odell, P.E. Rosel, J.T. Saliki, D.K. Shannon, S.F. Shippee, S.M. Smith, E.M. Stratton, M.C. Tumlin, H.R. Whitehead, G.A. Worthy, and T.K. Rowles. 2014. Review of historical unusual mortality events (UMEs) in the Gulf of Mexico (1990–2009): providing context for the multi-year northern Gulf of Mexico cetacean UME declared in 2010. Diseases of Aquatic Organisms 112(2):161–175. doi: 10.3354/dao02807

Mann, J., R.C. Connor, L.M. Barre, and M.R. Heithaus. 2000. Female reproductive success in bottlenose dolphins (*Tursiops* sp.): life history, habitat, provisioning, and group-size effects. Behavioral Ecology 11(2):210–219. doi: 10.1093/beheco/11.2.210

Mannocci, L., J.J. Roberts, D.L. Miller, and P.N. Halpin. 2017. Extrapolating cetacean densities to quantitatively assess human impacts on populations in the high seas. Conservation Biology 31(3):601–614. doi: 10.1111/cobi.12856

Mattson, M.C., K.D. Mullin, G.W. Ingram, and W. Hoggard. 2006. Age structure and growth of the bottlenose dolphin (*Tursiops truncatus*) from strandings in the Mississippi sound region of the north-central Gulf of Mexico from 1986 to 2003. Marine Mammal Science 22(3):654–666. doi: 10.1111/j.1748-7692.2006.00057.x

McHugh, M.B. 1989. Population numbers and feeding behavior of the Atlantic bottlenose dolphin (*Tursiops truncatus*) near Aransas Pass, Texas. University of Texas at Austin. Thesis. 96 p.

Mead, J.G. and C.W. Potter. 1990. Natural history of bottlenose dolphins along the central Atlantic coast of the United States. In: Leatherwood, S. and R.R. Reeves (Eds.), The Bottlenose Dolphin. Academic Press, San Diego, CA. pp. 165–195.

Michel, J., E.H. Owens, S. Zengel, A. Graham, Z. Nixon, T. Allard, W. Holton, P.D. Reimer, A. Lamarche, M. White, N. Rutherford, C. Childs, G. Mauseth, G. Challenger, and E. Taylor. 2013. Extent and degree of shoreline oiling: Deepwater Horizon oil spill, Gulf of Mexico, USA. PLoS ONE 8(6):e65087. doi: 10.1371/journal.pone.0065087

Miller, L.J., A.D. Mackey, T. Hoffland, M. Solangi, and S.A. Kuczaj. 2010. Potential effects of a major hurricane on Atlantic bottlenose dolphin (*Tursiops truncatus*) reproduction in the Mississippi Sound. Marine Mammal Science 26(3):707–715. doi: 10.1111/j.1748-7692.2010.00371.x

Miller, L.J., A.D. Mackey, M. Solangi, and S.A. Kuczaj. 2013. Population abundance and habitat utilization of bottlenose dolphins in the Mississippi Sound. Aquatic Conservation: Marine and Freshwater Ecosystems 23(1):145–151. doi: 10.1002/aqc.2278

National Oceanic and Atmospheric Administration (NOAA). 2011. Public scoping for preparation of a programmatic environmental impact statement for the Deepwater Horizon BP Oil Spill. 9 p.

Perrin, W.F. and S.B. Reilly. 1984. Reproductive parameters of dolphins and small whales of the family Delphinidae. In: Perrin, W.F., R.L. Brownell, Jr., and D.P. DeMaster (Eds.), Reproduction in Whales, Dolphins, and Porpoises. pp. 97–134.

Roberts, J.J., B.D. Best, L. Mannocci, E. Fujioka, P.N. Halpin, D.L. Palka, L.P. Garrison, K.D. Mullin, T.V.N. Cole, C.B. Khan, W.A. McLellan, D.A. Pabst, and G.G. Lockhart. 2016. Habitat-based cetacean density models for the US Atlantic and Gulf of Mexico. Scientific Reports 6:22615. doi: 10.1038/Srep22615

Roberts, J.J., B.D. Best, L. Mannocci, E. Fujioka, P.N. Halpin, D.L. Palka, L.P. Garrison, K.D. Mullin, T.V.N. Cole, C.B. Khan, W.M. McLellan, D.A. Pabst, and G.G. Lockhart. 2015. Density model for bottlenose dolphin (*Tursiops truncatus*) for the U.S. Gulf of Mexico version 3.3, 2015-10-07, and supplementary report. Duke University, Marine Geospatial Ecology Lab: Durham, NC. 84 p.

Rosel, P.E., L.A. Wilcox, C. Sinclair, T.R. Speakman, M.C. Tumlin, J.A. Litz, and E.S. Zolman. 2017. Genetic assignment to stock of stranded common bottlenose dolphins in southeastern Louisiana after the Deepwater Horizon oil spill. Endangered Species Research 33:221–234. doi: 10.3354/esr00780

Rossman, S., P.H. Ostrom, M. Stolen, N.B. Barros, H. Gandhi, C.A. Stricker, and R.S. Wells. 2015. Individual specialization in the foraging habits of female bottlenose dolphins living in a trophically diverse and habitat rich estuary. Oecologia 178(2):415–425. doi: 10.1007/s00442-015-3241-6

Schwacke, L.H., L. Thomas, R.S. Wells, W.E. McFee, A.A. Hohn, K.D. Mullin, E.S. Zolman, B.M. Quigley, T.K. Rowles, and J.H. Schwacke. 2017. Quantifying injury to common bottlenose dolphins from the Deepwater Horizon oil spill using an age-, sex- and class-structured population model. Endangered Species Research 33:265–279. doi: 10.3354/esr00777

Sellas, A.B., R.S. Wells, and P.E. Rosel. 2005. Mitochondrial and nuclear DNA analyses reveal fine scale geographic structure in bottlenose dolphins (*Tursiops truncatus*) in the Gulf of Mexico. Conservation Genetics 6(5):715–728. doi: 10.1007/s10592-005-9031-7

Sergeant, D.E., D.K. Caldwell, and M.C. Caldwell. 1973. Age, growth, and maturity of bottlenosed dolphin (*Tursiops-truncatus*) from northeast Florida. Journal of the Fisheries Research Board of Canada 30(7):1009–1011. doi: 10.1139/f73-165

Shane, S.H., R.S. Wells, and B. Würsig. 1986. Ecology, behavior and social organization of the bottlenose dolphin: A review. Marine Mammal Science 2:34–63. doi: 10.1111/j.1748-7692.1986.tb00026.x

Smith, C.R., T.K. Rowles, L.B. Hart, F.I. Townsend, R.S. Wells, E.S. Zolman, B.C. Balmer, B. Quigley, M. Ivančić, W. McKercher, M.C. Tumlin, K.D. Mullin, J.D. Adams, Q. Wu, W. McFee, T.K. Collier, and L.H. Schwacke. 2017. Slow recovery of Barataria Bay dolphin health following the Deepwater Horizon oil spill (2013-2014), with evidence of persistent lung disease and impaired stress response. Endangered Species Research 33:127–142. doi: 10.3354/esr00778

Taylor, B.L., S.J. Chivers, J. Larese, and W.F. Perrin. 2007. Generation length and percent mature estimates for IUCN assessments of cetaceans. National Oceanic and Atmospheric Administration, National Marine Fisheries Service, Southwest Fisheries Science Center: La Jolla, CA. Administrative Report LJ-07-01. 24 p.

Torres, L.G. and A.J. Read. 2009. Where to catch a fish? The influence of foraging tactics on the ecology of bottlenose dolphins (*Tursiops truncatus*) in Florida Bay, Florida. Marine Mammal Science 25(4):797–815. doi: 10.1111/j.1748-7692.2009.00297.x

Venn-Watson, S., K.M. Colegrove, J. Litz, M. Kinsel, K. Terio, J. Saliki, S. Fire, R. Carmichael, C. Chevis, W. Hatchett, J. Pitchford, M. Tumlin, C. Field, S. Smith, R. Ewing, D. Fauquier, G. Lovewell, H. Whitehead, D. Rotstein, W. McFee, E. Fougeres, and T. Rowles. 2015a. Adrenal gland and lung lesions in Gulf of Mexico common bottlenose dolphins (*Tursiops truncatus*) found dead following the Deepwater Horizon Oil Spill. PLoS ONE 10(5):e0126538. doi: 10.1371/journal.pone.0126538

Venn-Watson, S., L. Garrison, J. Litz, E. Fougeres, B. Mase, G. Rappucci, E. Stratton, R. Carmichael, D. Odell, D. Shannon, S. Shippee, S. Smith, L. Staggs, M. Tumlin, H. Whitehead, and T. Rowles. 2015b. Demographic clusters identified within the northern Gulf of Mexico common bottlenose dolphin (*Tursiops truncates*) unusual mortality event: January 2010-June 2013. PLoS ONE 10(2):e0117248. doi: 10.1371/journal.pone.0117248

Vollmer, N.L. and P.E. Rosel. 2013. A review of common bottlenose dolphins (*Tursiops truncatus truncatus*) in the Northern Gulf of Mexico: Population biology, potential threats, and management. Southeastern Naturalist 12(m6):1–43. doi: 10.1656/058.012.m601

Vollmer, N.L. and P.E. Rosel. 2017. Fine-scale population structure of common bottlenose dolphins (*Tursiops truncatus*) in offshore and coastal waters of the US Gulf of Mexico. Marine Biology 164(8):160. doi: 10.1007/s00227-017-3186-x

Waring, G.T., E. Josephson, K. Maze-Foley, and P.E. Rosel. 2016. U.S. Atlantic and Gulf of Mexico marine mammal stock assessments - 2015. National Oceanic and Atmospheric Administration, National Marine Fisheries Service, Northeast Fisheries Science Center: Woods Hole, MA. NOAA Technical Memorandum NMFS-NE-238. 501 p. doi: 10.7289/V57S7KTN

Wells, R.S. 2000. Reproduction in wild bottlenose dolphins: Overview of patterns observed during a long-term study. In: Duffield, D. and T. Robeck (Eds.), Bottlenose Dolphin Reproduction Workshop. AZA Marine Mammal Taxon Advisory Group, Silver Spring, MD. pp. 57–74.

Wells, R.S. 2003. Dolphin social complexity: Lessons from long-term study and life history. In: de Waal, F.B.M. and P.L. Tyack (Eds.), Animal Social Complexity: Intelligence, Culture, and Individualized Societies Harvard University Press, Cambridge, MA. pp. 32–56.

Wells, R.S. 2014. Social structure and life history of bottlenose dolphins near Sarasota Bay, Florida: Insights from four decades and five generations. Primates and cetaceans. pp. 149–172. doi: 10.1007/978-4-431-54523-1_8

Wells, R.S., A. Natoli, and G. Braulik. 2019. *Tursiops truncatus* (errata version published in 2019). *The IUCN Red List of Threatened Species* 2019: e.T22563A156932432. https://dx.doi.org/10.2305/IUCN.UK.2019-1.RLTS.T22563A156932432.en

Wells, R.S. and M.D. Scott. 1999. Bottlenose dolphin *Tursiops truncatus* (Montagu, 1821). In: Ridgway, S.H. and R. Harrison (Eds.), Handbook of marine mammals. Volume 6: The second book of dolphins and the porpoises. Academic Press, San Diego, CA. pp. 137–182.

Wells, R.S. and M.D. Scott. 2018. Bottlenose dolphin, *Tursiops truncatus*, common bottlenose dolphin. In: Würsig, B., J.G.M. Thewissen, and K.M. Kovacs (Eds.), Encyclopedia of Marine Mammals (Third Edition). Academic Press, London, UK. pp. 118–125. doi: 10.1016/B978-0-12-804327-1.00072-8

Wells, R.S., M.D. Scott, and A.B. Irvine. 1987. The social structure of free-ranging bottlenose dolphins. In: Genoways, H.H. (Ed.), Current Mammalogy. Plenum Press, New York, NY. pp. 247–305.

Wilson, R.M., R.B. Tyson, J.A. Nelson, B.C. Balmer, J.P. Chanton, and D.P. Nowacek. 2017. Niche differentiation and prey selectivity among common bottlenose dolphins (*Tursiops truncatus*) sighted in St. George Sound, Gulf of Mexico. Frontiers in Marine Science 4:235. doi: 10.3389/fmars.2017.00235

Common bottlenose dolphin − *Tursiops truncatus*

Gulf of Mexico, Western Coastal Stock

Overall Vulnerability Rank = High

Biological Sensitivity = Moderate

Climate Exposure = Very High

Data Quality = 93% of scores ≥ 2

	Tursiops truncatus	Expert Scores	Data Quality	Expert Scores Plots (Portion by Category)
Sensitivity attributes	Prey/Diet Specificity	2.1	2.5	
	Habitat Specificity	2.1	2.2	
	Site Fidelity	2.4	2.2	
	Lifetime Reproductive Potential	1.6	2.2	
	Generation Length	2.8	2.2	
	Reproductive Plasticity	1.4	1.8	
	Migration	3.4	2.2	
	Home Range	2.1	2.0	
	Stock Abundance	1.4	2.5	
	Stock Abundance Trend	1.9	1.5	
	Cumulative Stressors	2.5	2.5	
	Sensitivity Score	Moderate		
Exposure variables	Sea Surface Temperature (Change in mean)	4.0	2.8	
	Sea Surface Temperature (Change in variability)	1.0	2.8	
	Air Temperature (Change in mean)	4.0	2.8	
	Air Temperature (Change in variability)	1.0	2.8	
	Precipitation (Change in mean)	1.0	2.8	
	Precipitation (Change in variability)	1.0	2.8	
	Sea Surface Salinity (Change in mean)	1.8	2.8	
	Sea Surface Salinity (Change in variability)	1.0	2.8	
	Ocean pH (Change in mean)	4.0	2.8	
	Ocean pH (Change in variability)	1.0	2.8	
	Sea ice coverage (Change in mean)	1.0	2.8	
	Sea ice coverage (Change in variability)	1.0	2.8	
	Dissolved oxygen (Change in mean)	4.0	2.8	
	Dissolved oxygen (Change in variability)	1.0	2.8	
	Circulation	3.4	2.5	
	Sea level rise	3.5	2.8	
	Exposure Score	Very High		
Overall Vulnerability Rank		High		

Legend: Low, Moderate, High, Very High

Common bottlenose dolphin (Gulf of Mexico, Western Coastal Stock)

Tursiops truncatus

CVA Results Summary

Overall Climate Vulnerability Rank: High (99% certainty from bootstrap analysis).

Climate Exposure: Very High. Five exposure factors scored greater than or equal to 3.5: Air Temperature (Standard anomaly) (4.00), Dissolved oxygen (Standard anomaly) (4.00), Ocean pH (Standard anomaly) (4.00), Sea Surface Temperature (Standard anomaly) (4.00), and Sea level rise (3.50).

Biological Sensitivity: Moderate. Three sensitivity attributes scored greater than 2.5: Migration (3.45), Generation Time (2.80), and Cumulative Stressors (2.55).

Distributional Response: Moderate

Abundance Response: Moderate

Phenology Response: Moderate

Data Quality: 93% of the data quality scores were 2 or greater, 82% of sensitivity attribute data quality scores were 2 or higher.

Background Information

Stock Description

This stock includes common bottlenose dolphins in coastal waters (defined as waters between the shore, barrier islands or presumed outer bay boundaries out to the 20-m isobath) in an area of the northwestern Gulf of Mexico that extends from the Mississippi River Delta to the Texas-Mexico border (Hayes et al. 2022).

Prey/Diet Specificity

Generally, common bottlenose dolphins consume a large variety of fish and/or squid (Barros and Odell 1990; Mead and Potter 1990; Wells and Scott 2018). Common bottlenose dolphins seem to show a consistent preference for sound-producing fishes (e.g., sciaenids, toadfish; Berens McCabe et al. 2010; Bowen-Stevens et al. 2021). In Sarasota Bay, Florida, Rossman et al. (2015) found some individual common bottlenose dolphins to show diet specialization despite the broader study group showing a generalist diet. Specialization can be influenced by locally abundant prey species (Shane et al. 1986; Vollmer and Rosel 2013) or can result from resource partitioning amongst sympatric populations (e.g., Torres and Read 2009; Wilson et al. 2017).

In nearby Galveston Bay estuary, preliminary estimates of prey consumption proportions for bottlenose dolphins were determined through stable isotope analysis and Bayesian mixing models (McDaniel and Guillen 2020). In this study, examination of 36 remote biopsy samples obtained from free ranging dolphins in Galveston Bay between 2015-2017 indicate striped mullet (*Mugil cephalus*) and spotted seatrout (*Cynoscion nebulosus*) as primary prey sources. Additional contributions came from white

shrimp (*Litopenaeus setiferus*), hardhead catfish (*Ariopsis felis*) and likely fish with similar δ13C and δ15N values not included in the study. The authors suggest prey abundance may contribute to disparities in estimated contributions of prey sources between years (McDaniel and Guillen 2020). Barros and Odell (1990) examined the stomach contents of 76 stranded bottlenose dolphins from Gulf of Mexico shores, 22 of which were from the shoreline of northeast Texas adjacent to the Galveston Bay systems. Those 22 samples were combined with 3 additional stomach content analyses from bottlenose dolphin strandings recovered in the states of Louisiana, Mississippi, and Alabama (1 sample each), and designated those states as Area 1 of their study. Using a combination of visual taxonomic identification (for undigested species) and species identification keys for fish otolith and cephalopod beaks, Barros and Odell (1990) reported the proportion of prey in Area 1 as fish (94.3%), cephalopods (5.2%), crustaceans (0.5%), and a single horseshoe crab. Only 32% of the stomach contents from Area 1 contained fish only, while fish/cephalopod and fish/cephalopod/shrimp were present in 36% and 32% respectively, of Area 1 stomach contents (Barros and Odell 1990). Overall, sciaenid fishes were the primary fish prey. However, the great diversity of species (over 300 different fish species found in eight stomachs from Area 1) and similarity of species to commercial fisheries bycatch suggests bottlenose dolphins off Texas were supplementing their diet by depredating shrimp trawler bycatch or by taking fish directly out of the nets (presumably) during trawling operations (Barros and Odell 1990). Observations of bottlenose dolphins feeding using both methods from Texas and Louisiana have been well documented (Gunter 1938; Shane 1977; Gruber 1981; Henningsen and Würsig 1991; Fertl 1994; Moreno 2005).

While much research indicates sciaenid fishes are the primary prey fishes for bottlenose dolphins in the Gulf of Mexico (Berens McCabe et al. 2010; Dunshea et al. 2013), other researchers (Gunter 1942; Shane 1990; Miller 1992) have suggested striped mullet (*Mugil cephalus*) may be a significant part of the bottlenose dolphin diet in Texas waters. Barros and Wells (1998) reviewed the importance of mullet in the diet of bottlenose dolphins in Florida waters and suggested the species is important, yet ranks under soniferous fishes in their dietary profile.

Habitat Specificity

Common bottlenose dolphins are able to adapt to a variety of marine and estuarine habitats (Wells and Scott 2018).

Site Fidelity

No specific site fidelity information was found for this stock. However, common bottlenose dolphins in Gulf of Mexico bay, sound, and estuary (BSE) stocks show site fidelity (LaBrecque et al. 2015; Hayes et al. 2022), such as in Matagorda Bay, Espiritu Santo Bay, and San Luis Pass in Texas (Maze and Würsig 1999; Lynn and Würsig 2002; Irwin and Würsig 2004); Barataria Bay in Louisiana (Miller 2003; McDonald et al. 2017; Wells et al. 2017; Takeshita et al. 2021); Mississippi Sound in Mississippi (Hubard et al. 2004; Mackey 2010; Mullin et al. 2017); Charlotte Harbor and Pine Island Sound (Bassos-Hull et al. 2013), Sarasota Bay (Wells 1986; Wells 1991; Wells 2014), St. Andrew Bay (Balmer et al. 2019), St. Joseph Bay (Balmer et al. 2008; Balmer et al. 2018), St. Vincent Sound and Apalachicola Bay (Tyson et al. 2011), and Tampa Bay in Florida (Wells et al. 1996; Urian et al. 2009).

Lifetime Reproductive Potential

Taylor et al. (2007) reported the interbirth interval of common bottlenose dolphins as 3.80 years, though other studies have shown it to be closer to three years (Leatherwood 1977; Wells and Scott 1999; Connor et al. 2000; Wells 2003).

Generally, females reach sexual maturity before males, with females reaching sexual maturity at 5–13 years (Sergeant et al. 1973; Perrin and Reilly 1984; Wells et al. 1987; Mead and Potter 1990; Mann et al. 2000; Wells 2000; Wells and Scott 2018).

Females reproduce throughout their adult lives (Leatherwood 1977; Hohn et al. 1989; Wells and Scott 1999; Wells 2000; Wells 2003), but may produce fewer calves after they reach 30 years of age (Wells 2000). Sarasota common bottlenose dolphins can give birth when they are as young as 6 years of age, and continue until they are 48 years old (Wells and Scott 2018). Reproductive lifespan would therefore be up to 42 years based on long-term observation studies (Wells 2014; Wells and Scott 2018).

Longitudinal studies from Sarasota Bay, Florida have found that female common bottlenose dolphins can live up to 67 years (Wells and Scott 2018). Studies based on growth layer groups in teeth suggest female common bottlenose dolphins can live to 41 years along the Texas coast (Fernandez and Hohn 1998) and to 30 years along the Mississippi coast (Mattson et al. 2006).

Generation Length

Taylor et al. (2007) reported common bottlenose dolphin generation length of 20.6–21.1 years.

Reproductive Plasticity

Common bottlenose dolphin calving is generally diffusely seasonal, with one or more peaks, although births have been reported from all seasons (Vollmer and Rosel 2013; Wells and Scott 2018). Calf sightings peak in the spring and summer months in Florida (Wells et al. 1987), Mississippi/Louisiana (Miller et al. 2010; Miller et al. 2013), and Texas (McHugh 1989; Henderson and Würsig 2007).

Migration

Data are insufficient to characterize migration patterns (or the lack thereof) for this stock. However, genetic data do support the presence of multiple differentiated populations of common bottlenose dolphins in coastal and offshore waters with very low levels of migration among them (Vollmer and Rosel 2017). Molecular genetic data further support that coastal populations are genetically differentiated from BSE populations (Sellas et al. 2005; Rosel et al. 2017).

Inter-bay movements of individuals thought to be part of coastal stocks have been reported for the north-central Gulf of Mexico (Balmer et al. 2016; Ronje et al. 2017) and similar movements of the Western Coastal Stock may coincide with seasonal changes in water temperature and prey density. Seasonal changes in density throughout Texas have also been attributed to a combination of north - south migration along the coast influenced by water temperature and more localized shifts in distribution influenced by prey movements in and out of the estuary during different times of the year (Weller 1998). Gruber (1981) observed an individual in Matagorda Bay previously identified in the Port Aransas area, [illegible]95 km to the southwest. Jones (1991) reported 2 dolphins that ranged between Gulf

inlets 500 - 600 km apart, and found 11 others traveled a distance < 300 km. Coastal movements have also been reported in north Texas between Galveston Bay and West Bay (Maze and Würsig 1999; Ronje et al. 2020) and between Galveston Bay and Sabine Lake (Ronje et al. 2020). Lynn and Würsig (2002) reviewed Texas dolphin movement patterns and hypothesized that while small localized communities of Texas dolphins that exhibit high site fidelity to individual bays may be highly susceptible to anthropogenic threats, these populations may have the ability to recover due to the presence of transients and migratory individuals traveling between bays. The authors stressed the unknown aspect of genetic mixing in these communities and the need for additional research. Sellas et al. (2005) concluded little interbreeding occurs between coastal and estuarine populations in their study on the Florida coast; however, more research needs to be conducted to determine the migratory habits of the Western Coastal Stock.

Home Range

Data are insufficient to characterize home range patterns (or the lack thereof) for this stock.

Stock Abundance

The best abundance estimate available for this stock is 20,759 individuals (CV=0.13; Garrison et al. 2021; Hayes et al. 2022) based on summer 2017 and fall 2018 aerial surveys. Previously, this stock was estimated at 20,161 individuals (CV=0.17) based on seasonal abundance estimates from spring 2011, summer 2011, fall 2011 and winter 2012 aerial surveys (Waring et al. 2016).

Stock Abundance Trend

Data are insufficient to complete an abundance trend analysis for this stock (Hayes et al. 2022).

Cumulative Stressors

Common bottlenose dolphins face a wide range of individual and cumulative natural and anthropogenic threats (see reviews by Vollmer and Rosel 2013; Phillips and Rosel 2014; Wells and Scott 2018; and Wells et al. 2019). These include such threats as shark predation, harmful algal blooms, recreational and commercial fishing gear, vessel traffic, pollution, habitat alteration, and provisioning.

Common bottlenose dolphins in the northern Gulf of Mexico were exposed to oil and dispersants following the Deepwater Horizon oil spill, which may result in long term reproductive and chronic health effects (NOAA 2011; Michel et al. 2013; Helm et al. 2015; DWH NRDA 2016; Lane et al. 2015; Aichinger Dias et al. 2017; De Guise et al. 2017, 2021; Kellar et al. 2017; Smith et al. 2017; Schwacke et al. 2017). An Unusual Mortality Event (UME), involving primarily bottlenose dolphins, was declared for cetaceans in the northern Gulf of Mexico from 2010 to 2014, with the Deepwater Horizon oil spill as the primary underlying cause (Litz et al. 2014; Schwacke et al. 2014; Venn-Watson et al. 2015a, 2015b).

The Gulf of Mexico western coastal common bottlenose dolphin stock potentially interacts with five commercial fisheries (Southeastern U.S. Atlantic, Gulf of Mexico shrimp trawl; Gulf of Mexico menhaden purse seine; Gulf of Mexico gillnet; Gulf of Mexico blue crab trap/pot; and commercial passenger fishing vessel/hook-and-line; Hayes et al. 2022).

The nearshore and estuarine habitats along the Gulf of Mexico coast include waters adjacent to areas of high human population, some of which are highly industrialized, and areas of heavy agricultural impact. Studies of common bottlenose dolphin tissues from western Gulf Coast waters have shown persistent organic pollutants (POPs) in blubber and blood samples (Kucklick et al. 2011; Balmer et al. 2018). Similarly, mercury has been detected in common bottlenose dolphin tissues from western Gulf Coast waters (Bryan et al. 2007; Woshner et al. 2008; McCormack et al. 2020a, 2020b, 2022).

An additional five UMEs in the Gulf of Mexico have overlapped with the western coastal common bottlenose dolphin stock. The suspected causes of these UMEs have included environmental conditions, brevetoxin, and dolphin morbillivirus (Litz et al. 2014). Although morbillivirus morbillivirus has been detected in populations of the northern Gulf of Mexico, it has not been attributed to large scale die-offs since 1993−1994 (Lipscomb et al. 1996; Litz et al. 2014; Van Bressem et al. 2014; Fauquier et al. 2017).

Distribution and Sightings

Density model results for common bottlenose dolphins in the Gulf of Mexico are presented by Roberts et al. (2015, 2016) and Mannocci et al. (2017).

Further Reading

Hayes, S.A., E. Josephson, K. Maze-Foley, P.E. Rosel, and J. Wallace. 2022. U.S. Atlantic and Gulf of Mexico marine mammal stock assessments 2021. National Oceanic and Atmospheric Administration, National Marine Fisheries Service, Northeast Fisheries Science Center: Woods Hole, MA. NOAA Technical Memorandum NMFS-NE-288. 380 p. doi: 10.25923/6tt7-kc16

Wells, R.S. and M.D. Scott. 2018. Bottlenose dolphin, *Tursiops truncatus*, common bottlenose dolphin. In: Würsig, B., J.G.M. Thewissen, and K.M. Kovacs (Eds.), Encyclopedia of Marine Mammals (Third Edition). Academic Press, London, UK. pp. 118–125. doi: 10.1016/B978-0-12-804327-1.00072-8

Wells, R.S., A. Natoli, and G. Braulik. 2019. *Tursiops truncatus* (errata version published in 2019). The IUCN Red List of Threatened Species 2019: e.T22563A156932432. https://dx.doi.org/10.2305/IUCN.UK.2019-1.RLTS.T22563A156932432.en

References

Aichinger Dias, L., J. Litz, L. Garrison, A. Martinez, K. Barry, and T. Speakman. 2017. Exposure of cetaceans to petroleum products following the Deepwater Horizon oil spill in the Gulf of Mexico. Endangered Species Research 33:119–125. doi: 10.3354/esr00770

Balmer, B., T. McDonald, F. Hornsby, J. Adams, J. Allen, A. Barleycorn, and L. Schwacke. 2018. Long-term trends in a northern Gulf of Mexico common bottlenose dolphin (*Tursiops truncatus*) population in the wake of the Deepwater Horizon oil spill. Journal of Cetacean Research and Management 18:1–9. doi: 10.47536/jcrm.v18i1.409

Balmer, B., C. Sinclair, T. Speakman, B. Quigley, K. Barry, C. Cush, M. Hendon, K. Mullin, E. Ronje, P. Rosel, L. Schwacke, R. Wells, and E. Zolman. 2016. Extended movements of common bottlenose dolphins (*Tursiops truncatus*) along the northern Gulf of Mexico's central coast. Gulf of Mexico Science 33(1):93–97. doi: 10.18785/goms.3301.08

Balmer, B., S. Watwood, B. Quigley, T. Speakman, K. Barry, K. Mullin, P. Rosel, C. Sinclair, E. Zolman, and L. Schwacke. 2019. Common bottlenose dolphin (*Tursiops truncatus*) abundance and distribution

patterns in St. Andrew Bay, Florida, USA. Aquatic Conservation: Marine and Freshwater Ecosystems 29(3):486–498. doi: 10.1002/aqc.3001

Balmer, B.C., R.S. Wells, S.M. Nowacek, D.P. Nowacek, L.H. Schwacke, W.A. McLellan, F.S. Scharf, T.K. Rowles, L.J. Hansen, T.R. Spradlin, and D.A. Pabst. 2008. Seasonal abundance and distribution patterns of common bottlenose dolphins (*Tursiops truncatus*) near St. Joseph Bay, Florida, USA. Journal of Cetacean Research and Management 10(2):157–167.

Barros, N.B. and D.K. Odell. 1990. Food habits of bottlenose dolphins in the southeastern United States In: Leatherwood, S. and R.R. Reeves (Eds.), The Bottlenose Dolphin. Academic Press, San Diego, CA. pp. 309–328. doi: 10.1016/B978-0-12-440280-5.50020-2

Barros, N.B. and R.S. Wells. 1998. Prey and feeding patterns of resident bottlenose dolphins (*Tursiops truncatus*) in Sarasota Bay, Florida. Journal of Mammalogy 79(3):1045–1059. doi: 10.2307/1383114

Bassos-Hull, K., R.M. Perrtree, C.C. Shepard, S. Schilling, A.A. Barleycorn, J.B. Allen, B.C. Balmer, W.E. Pine, and R.S. Wells. 2013. Long-term site fidelity and seasonal abundance estimates of common bottlenose dolphins (*Tursiops truncatus*) along the southwest coast of Florida and responses to natural perturbations. Journal of Cetacean Research and Management 13(1):19–30. doi: 10.47536/jcrm.v13i1.551

Berens McCabe, E.J., D.P. Gannon, N.B. Barros, and R.S. Wells. 2010. Prey selection by resident common bottlenose dolphins (*Tursiops truncatus*) in Sarasota Bay, Florida. Marine Biology 157(5):931–942. doi: 10.1007/s00227-009-1371-2

Bowen-Stevens, S.R., D.P. Gannon, R.A. Hazelkorn, G. Lovewell, K.M. Volker, S. Smith, M.C. Tumlin, and J. Litz. 2021. Diet of common bottlenose dolphins, *Tursiops truncatus*, that stranded in and near Barataria Bay, Louisiana, 2010–2012. Southeastern Naturalist 20(1). doi: 10.1656/058.020.0113

Bryan, C.E., S.J. Christopher, B.C. Balmer, and R.S. Wells. 2007. Establishing baseline levels of trace elements in blood and skin of bottlenose dolphins in Sarasota Bay, Florida: implications for non-invasive monitoring. Science of the Total Environment 388(1-3):325–342. doi: 10.1016/j.scitotenv.2007.07.046

Connor, R.C., R.S. Wells, J. Mann, and A.J. Read. 2000. The bottlenose dolphin: Social relationships in a fission-fusion society. In: Mann, J., R. Connor, P. Tyack, and H. Whitehead (Eds.), Cetacean Societies: Field Studies of Dolphins and Whales. University of Chicago Press, Chicago, IL. pp. 91–126.

De Guise, S., M. Levin, E. Gebhard, L. Jasperse, L. Burdett Hart, C.R. Smith, S. Venn-Watson, F. Townsend, R. Wells, B. Balmer, E. Zolman, T. Rowles, and L. Schwacke. 2017. Changes in immune functions in bottlenose dolphins in the northern Gulf of Mexico associated with the Deepwater Horizon oil spill. Endangered Species Research 33:291–303. doi: 10.3354/esr00814

De Guise, S., M. Levin, L. Jasperse, J. Herrman, R.S. Wells, T. Rowles, and L. Schwacke. 2021. Long-term immunological alterations in bottlenose dolphin a decade after the Deepwater Horizon oil spill in the northern Gulf of Mexico: Potential for multigenerational effects. Environmental Toxicology and Chemistry 40(5):1308–1321. doi: 10.1002/etc.4980

Deepwater Horizon Natural Resource Damage Assessment Trustees (DWH NRDA). 2016. Deepwater Horizon oil spill: Final Programmatic Damage Assessment and Restoration Plan and Final Programmatic Environmental Impact Statement. Retrieved from: http://www.gulfspillrestoration.noaa.gov/restoration-planning/gulf-plan.1659.

Dunshea, G., N.B. Barros, E.J. Berens McCabe, N.J. Gales, M.A. Hindell, S.N. Jarman, and R.S. Wells. 2013. Stranded dolphin stomach contents represent the free-ranging population's diet. Biology Letters 9(3):20121036–20121036. doi: 10.1098/rsbl.2012.1036

Fauquier, D.A., J. Litz, S. Sanchez, K. Colegrove, L.H. Schwacke, L. Hart, J. Saliki, C. Smith, T. Goldstein, S. Bowen-Stevens, W. McFee, E. Fougeres, B. Mase-Guthrie, E. Stratton, R. Ewing, S. Venn-Watson,

R.H. Carmichael, C. Clemons-Chevis, W. Hatchett, D. Shannon, S. Shippee, S. Smith, L. Staggs, M.C. Tumlin, N.L. Wingers, and T.K. Rowles. 2017. Evaluation of morbillivirus exposure in cetaceans from the northern Gulf of Mexico 2010-2014. Endangered Species Research 33:211–220. doi: 10.3354/esr00772

Fernandez, S. and A.A. Hohn. 1998. Age, growth, and calving season of bottlenose dolphins, *Tursiops truncatus*, off coastal Texas. Fishery Bulletin 96(2):357–365.

Fertl, D. 1994. Occurrence patterns and behavior of bottlenose dolphins (*Tursiops truncatus*) in the Galveston ship channel, Texas. Texas Journal of Science 46(4):299–318.

Garrison, L.P., J. Ortega-Ortiz, and G. Rappucci. 2021. Abundance of coastal and continental shelf stocks of common bottlenose and Atlantic spotted dolphins in the northern Gulf of Mexico: 2017–2018. National Oceanic and Atmospheric Administration, National Marine Fisheries Service, Southeast Fisheries Science Center, Protected Resources and Biodiversity Division: Miami, FL. PRD-2021-01. 25 p. doi: 10.25923/vk95-t881

Gruber, J.A. 1981. Ecology of the Atlantic bottlenosed dolphin (*Tursiops truncatus*) in the Pass Cavallo area of Matagorda Bay, Texas. Texas A&M University. Thesis. 182 p.

Gunter, G. 1938. Seasonal variations in abundance of certain estuarine and marine fishes in Louisiana, with particular reference to life histories. Ecological Monographs 8:314–346.

Gunter, G. 1942. Contributions to the natural history of the bottle-nose dolphin *Tursiops truncatus* (Montague) on the Texas coast. Journal of Mammalogy 23:267–276.

Hayes, S.A., E. Josephson, K. Maze-Foley, P.E. Rosel, and J. Wallace. 2022. U.S. Atlantic and Gulf of Mexico marine mammal stock assessments 2021. National Oceanic and Atmospheric Administration, National Marine Fisheries Service, Northeast Fisheries Science Center: Woods Hole, MA. NOAA Technical Memorandum NMFS-NE-288. 380 p. doi: 10.25923/6tt7-kc16

Helm, R.C., D.P. Costa, T.D. DeBruyn, T.J. O'Shea, R.S. Wells, and T.M. Williams. 2015. Overview of effects of oil spills on marine mammals. In: Fingas, M. (Ed.), Handbook of oil spill science and technology, First edition. John Wiley & Sons, Inc., Hoboken, NJ. pp. 455–475.

Henderson, E.E. and B. Würsig. 2007. Behavior patterns of bottlenose dolphins in San Luis Pass, Texas. Gulf of Mexico Science 25(2):153–161. doi: 10.18785/goms.2502.06

Henningsen, T. and B. Würsig. 1991. Bottle-nosed dolphins in Galveston Bay, Texas: Numbers and activities. Proceedings of the Fifth Annual Conference of the European Cetacean Society, Sandefjord, Norway, 21–23 February 1991. pp. 36–38.

Hohn, A.A., M.D. Scott, R.S. Wells, J.C. Sweeney, and A.B. Irvine. 1989. Growth layers in teeth from known-age, free-ranging bottlenose dolphins. Marine Mammal Science 5(4):315–342. doi: 10.1111/j.1748-7692.1989.tb00346.x

Hubard, C.W., K. Maze-Foley, K.D. Mullin, and W.W. Schroeder. 2004. Seasonal abundance and site fidelity of bottlenose dolphins (*Tursiops truncatus*) in Mississippi Sound. Aquatic Mammals 30(2):299–310. doi: 10.1578/am.30.2.2004.299

Irwin, L.-J. and B. Würsig. 2004. A small resident community of bottlenose dolphins, *Tursiops truncatus*, in Texas: Monitoring recommendations. Gulf of Mexico Science 22(1):13–21. doi: 10.18785/goms.2201.02

Jones, S.C., III. 1991. Movements of bottlenose dolphins between inlets along the Texas coast. Abstracts of the 9th Biennial Conference on the Biology of Marine Mammals, Chicago, IL, 7-11 November 1991. p. 37.

Kellar, N.M., T.R. Speakman, C.R. Smith, S.M. Lane, B.C. Balmer, M.L. Trego, K.N. Catelani, M.N. Robbins, C.D. Allen, R.S. Wells, E.S. Zolman, T.K. Rowles, and L.H. Schwacke. 2017. Low reproductive success rates of common bottlenose dolphins *Tursiops truncatus* in the northern Gulf of Mexico following the Deepwater Horizon disaster (2010-2015). Endangered Species Research 33:143–158. doi: 10.3354/esr00775

Kucklick, J., L. Schwacke, R. Wells, A. Hohn, A. Guichard, J. Yordy, L. Hansen, E. Zolman, R. Wilson, J. Litz, D. Nowacek, T. Rowles, R. Pugh, B. Balmer, C. Sinclair, and P. Rosel. 2011. Bottlenose dolphins as indicators of persistent organic pollutants in the western North Atlantic Ocean and Northern Gulf of Mexico. Environmental Science & Technology 45(10):4270–4277. doi: 10.1021/es1042244

LaBrecque, E., C. Curtice, J. Harrison, S.M. Van Parijs, and P.N. Halpin. 2015. 3. Biologically Important Areas for cetaceans within U.S. Waters – Gulf of Mexico Region. Aquatic Mammals 41(1):30–38. doi: 10.1578/am.41.1.2015.30

Lane, S.M., C.R. Smith, J. Mitchell, B.C. Balmer, K.P. Barry, T. McDonald, C.S. Mori, P.E. Rosel, T.K. Rowles, T.R. Speakman, F.I. Townsend, M.C. Tumlin, R.S. Wells, E.S. Zolman, and L.H. Schwacke. 2015. Reproductive outcome and survival of common bottlenose dolphins sampled in Barataria Bay, Louisiana, USA, following the Deepwater Horizon oil spill. Proceedings of the Royal Society B: Biological Sciences 282(1818):20151944. doi: 10.1098/rspb.2015.1944

Leatherwood, S. 1977. Some preliminary impressions on the numbers and social behavior of free-swimming bottlenosed dolphin calves (*Tursiops truncatus*) in the northern Gulf of Mexico. In: Ridgway, S.H. and K. Bernirschke (Eds.), Breeding Dolphins; Present Status, Suggestions for the Future. Final report to the US Marine Mammal Commission, Contract MM6AC009, MMC-76/07, Washington, DC. pp. 143–167.

Lipscomb, T.P., S. Kennedy, D. Moffett, A. Krafft, B.A. Klaunberg, J.H. Lichy, G.T. Regan, G.A.J. Worthy, and J.K. Taubenberger. 1996. Morbilliviral epizootic in bottlenose dolphins of the Gulf of Mexico. Journal of Veterinary Diagnostic Investigation 8(3):283–290. doi: 10.1177/104063879600800302

Litz, J.A., M.A. Baran, S.R. Bowen-Stevens, R.H. Carmichael, K.M. Colegrove, L.P. Garrison, S.E. Fire, E.M. Fougeres, R. Hardy, S. Holmes, W. Jones, B.E. Mase-Guthrie, D.K. Odell, P.E. Rosel, J.T. Saliki, D.K. Shannon, S.F. Shippee, S.M. Smith, E.M. Stratton, M.C. Tumlin, H.R. Whitehead, G.A. Worthy, and T.K. Rowles. 2014. Review of historical unusual mortality events (UMEs) in the Gulf of Mexico (1990–2009): providing context for the multi-year northern Gulf of Mexico cetacean UME declared in 2010. Diseases of Aquatic Organisms 112(2):161–175. doi: 10.3354/dao02807

Lynn, S.K. and B. Würsig. 2002. Summer movement patterns of bottlenose dolphins in a Texas bay. Gulf of Mexico Science 20(1):25–37. doi: 10.18785/goms.2001.03

Mackey, A.D. 2010. Site fidelity and association patterns of bottlenose dolphins (*Tursiops truncatus*) in the Mississippi Sound. University of Southern Mississippi. Thesis. 133 p.

Mann, J., R.C. Connor, L.M. Barre, and M.R. Heithaus. 2000. Female reproductive success in bottlenose dolphins (*Tursiops* sp.): life history, habitat, provisioning, and group-size effects. Behavioral Ecology 11(2):210–219. doi: 10.1093/beheco/11.2.210

Mannocci, L., J.J. Roberts, D.L. Miller, and P.N. Halpin. 2017. Extrapolating cetacean densities to quantitatively assess human impacts on populations in the high seas. Conservation Biology 31(3):601–614. doi: 10.1111/cobi.12856

Mattson, M.C., K.D. Mullin, G.W. Ingram, and W. Hoggard. 2006. Age structure and growth of the bottlenose dolphin (*Tursiops truncatus*) from strandings in the Mississippi sound region of the north-central Gulf of Mexico from 1986 to 2003. Marine Mammal Science 22(3):654–666. doi: 10.1111/j.1748-7692.2006.00057.x

Maze, K.S. and B. Würsig. 1999. Bottlenosed dolphins of San Luis Pass, Texas: Occurrence patterns, site-fidelity, and habitat use. Aquatic Mammals 25:91–104.

McCormack, M.A., F. Battaglia, W.E. McFee, and J. Dutton. 2020a. Mercury concentrations in blubber and skin from stranded bottlenose dolphins (*Tursiops truncatus*) along the Florida and Louisiana coasts (Gulf of Mexico, USA) in relation to biological variables. Environmental Research 180:108886. doi: 10.1016/j.envres.2019.108886

McCormack, M.A., B.P. Jackson, and J. Dutton. 2020b. Relationship between mercury and selenium concentrations in tissues from stranded odontocetes in the northern Gulf of Mexico. Science of the Total Environment 749:141350. doi: 10.1016/j.scitotenv.2020.141350

McCormack, M.A., W.H. Nowlin, and J. Dutton. 2022. Effect of trophic position on mercury concentrations in bottlenose dolphins (*Tursiops truncatus*) from the northern Gulf of Mexico. Environmental Research 204(Pt B):112124. doi: 10.1016/j.envres.2021.112124

McDaniel, S. and G.J. Guillen. 2020. Foraging ecology of bottlenose dolphins (*Tursiops truncatus*) in Galveston Bay [Poster]. Galveston Bay Estuary Program, State of the Bay Symposium, Galveston, TX,

McDonald, T.L., F.E. Hornsby, T.R. Speakman, E.S. Zolman, K.D. Mullin, C. Sinclair, P.E. Rosel, L. Thomas, and L.H. Schwacke. 2017. Survival, density, and abundance of common bottlenose dolphins in Barataria Bay (USA) following the Deepwater Horizon oil spill. Endangered Species Research 33:193–209. doi: 10.3354/esr00806

McHugh, M.B. 1989. Population numbers and feeding behavior of the Atlantic bottlenose dolphin (*Tursiops truncatus*) near Aransas Pass, Texas. University of Texas at Austin. Thesis. 96 p.

Mead, J.G. and C.W. Potter. 1990. Natural history of bottlenose dolphins along the central Atlantic coast of the United States. In: Leatherwood, S. and R.R. Reeves (Eds.), The Bottlenose Dolphin. Academic Press, San Diego, CA. pp. 165–195.

Michel, J., E.H. Owens, S. Zengel, A. Graham, Z. Nixon, T. Allard, W. Holton, P.D. Reimer, A. Lamarche, M. White, N. Rutherford, C. Childs, G. Mauseth, G. Challenger, and E. Taylor. 2013. Extent and degree of shoreline oiling: Deepwater Horizon oil spill, Gulf of Mexico, USA. PLoS ONE 8(6):e65087. doi: 10.1371/journal.pone.0065087

Miller, C.E. 2003. Abundance trends and environmental habitat usage patterns of bottlenose dolphins (*Tursiops truncatus*) in lower Barataria and Caminada bays, Louisiana. Louisiana State University and Agricultural & Mechanical College. Dissertation. 125 p.

Miller, L.J., A.D. Mackey, T. Hoffland, M. Solangi, and S.A. Kuczaj. 2010. Potential effects of a major hurricane on Atlantic bottlenose dolphin (*Tursiops truncatus*) reproduction in the Mississippi Sound. Marine Mammal Science 26(3):707–715. doi: 10.1111/j.1748-7692.2010.00371.x

Miller, L.J., A.D. Mackey, M. Solangi, and S.A. Kuczaj. 2013. Population abundance and habitat utilization of bottlenose dolphins in the Mississippi Sound. Aquatic Conservation: Marine and Freshwater Ecosystems 23(1):145–151. doi: 10.1002/aqc.2278

Miller, W.G. 1992. An investigation of bottlenose dolphin *Tursiops truncatus* deaths in East Matagorda Bay, Texas, January 1990. Fishery Bulletin 90:791–797.

Moreno, M.P.T. 2005. Environmental predictors of bottlenose dolphin distribution and core feeding densities in Galveston Bay, Texas. Texas A&M University at Galveston. Dissertation. 88 p.

Mullin, K.D., T. McDonald, R.S. Wells, B.C. Balmer, T. Speakman, C. Sinclair, E.S. Zolman, F. Hornsby, S.M. McBride, K.A. Wilkinson, and L.H. Schwacke. 2017. Density, abundance, survival, and ranging patterns of common bottlenose dolphins (*Tursiops truncatus*) in Mississippi Sound following the Deepwater Horizon oil spill. PLoS ONE 12(10):e0186265. doi: 10.1371/journal.pone.0186265

National Oceanic and Atmospheric Administration (NOAA). 2011. Public scoping for preparation of a programmatic environmental impact statement for the Deepwater Horizon BP Oil Spill. 9 p.

Perrin, W.F. and S.B. Reilly. 1984. Reproductive parameters of dolphins and small whales of the family Delphinidae. In: Perrin, W.F., R.L. Brownell, Jr., and D.P. DeMaster (Eds.), Reproduction in Whales, Dolphins, and Porpoises. pp. 97–134.

Phillips, N.M. and P.E. Rosel. 2014. A method for prioritizing research on common bottlenose dolphin stocks through evaluating threats and data availability: Development and application to bay, sound and estuary stocks in Texas. National Oceanic and Atmospheric Administration, National

Marine Fisheries Service, Southeast Fisheries Science Center: Lafayette, LA. NOAA Technical Memorandum NMFS-SEFSC-665. 146 p. doi: 10.7289/V5F769H8

Roberts, J.J., B.D. Best, L. Mannocci, E. Fujioka, P.N. Halpin, D.L. Palka, L.P. Garrison, K.D. Mullin, T.V.N. Cole, C.B. Khan, W.A. McLellan, D.A. Pabst, and G.G. Lockhart. 2016. Habitat-based cetacean density models for the US Atlantic and Gulf of Mexico. Scientific Reports 6:22615. doi: 10.1038/Srep22615

Roberts, J.J., B.D. Best, L. Mannocci, E. Fujioka, P.N. Halpin, D.L. Palka, L.P. Garrison, K.D. Mullin, T.V.N. Cole, C.B. Khan, W.M. McLellan, D.A. Pabst, and G.G. Lockhart. 2015. Density model for bottlenose dolphin (*Tursiops truncatus*) for the U.S. Gulf of Mexico version 3.3, 2015-10-07, and supplementary report. Duke University, Marine Geospatial Ecology Lab: Durham, NC. 84 p.

Ronje, E., H. Whitehead, K. Barry, S. Piwetz, J. Struve, V. Lecours, L. Garrison, R.S. Wells, and K.D. Mullin. 2020. Abundance and occurrence of common bottlenose dolphins (*Tursiops truncatus*) in three estuaries of the northwestern Gulf of Mexico. Gulf and Caribbean Research 31:18–34. doi: 10.18785/gcr.3101.09

Ronje, E.I., H.R. Whitehead, S. Piwetz, K. Barry, C. Sinclair, and K.D. Mullin. 2017. Seasonal distribution and coastal movements of common bottlenose dolphins in bays and adjacent coastal waters of Texas. 22nd Biennial Conference of the Society of Marine Mammalogy, Halifax, Nova Scotia, Canada, 22–28 October 2017

Rosel, P.E., L.A. Wilcox, C. Sinclair, T.R. Speakman, M.C. Tumlin, J.A. Litz, and E.S. Zolman. 2017. Genetic assignment to stock of stranded common bottlenose dolphins in southeastern Louisiana after the Deepwater Horizon oil spill. Endangered Species Research 33:221–234. doi: 10.3354/esr00780

Rossman, S., P.H. Ostrom, M. Stolen, N.B. Barros, H. Gandhi, C.A. Stricker, and R.S. Wells. 2015. Individual specialization in the foraging habits of female bottlenose dolphins living in a trophically diverse and habitat rich estuary. Oecologia 178(2):415–425. doi: 10.1007/s00442-015-3241-6

Schwacke, L.H., C.R. Smith, F.I. Townsend, R.S. Wells, L.B. Hart, B.C. Balmer, T.K. Collier, S. De Guise, M.M. Fry, L.J. Guillette, Jr., S.V. Lamb, S.M. Lane, W.E. McFee, N.J. Place, M.C. Tumlin, G.M. Ylitalo, E.S. Zolman, and T.K. Rowles. 2014. Health of common bottlenose dolphins (*Tursiops truncatus*) in Barataria Bay, Louisiana, following the deepwater horizon oil spill. Environmental Science & Technology 48(1):93–103. doi: 10.1021/es403610f

Schwacke, L.H., L. Thomas, R.S. Wells, W.E. McFee, A.A. Hohn, K.D. Mullin, E.S. Zolman, B.M. Quigley, T.K. Rowles, and J.H. Schwacke. 2017. Quantifying injury to common bottlenose dolphins from the Deepwater Horizon oil spill using an age-, sex- and class-structured population model. Endangered Species Research 33:265–279. doi: 10.3354/esr00777

Sellas, A.B., R.S. Wells, and P.E. Rosel. 2005. Mitochondrial and nuclear DNA analyses reveal fine scale geographic structure in bottlenose dolphins (*Tursiops truncatus*) in the Gulf of Mexico. Conservation Genetics 6(5):715–728. doi: 10.1007/s10592-005-9031-7

Sergeant, D.E., D.K. Caldwell, and M.C. Caldwell. 1973. Age, growth, and maturity of bottlenosed dolphin (*Tursiops-truncatus*) from northeast Florida. Journal of the Fisheries Research Board of Canada 30(7):1009–1011. doi: 10.1139/f73-165

Shane, S. 1977. The population biology of the Atlantic bottlenose dolphin, *Tursiops truncatus*, in the Aransas Pass area of Texas. Texas A&M University. Thesis.

Shane, S.H. 1990. Behavior and ecology of the bottlenose dolphin at Sanibel Island, Florida. In: Leatherwood, S. and R.R. Reeves (Eds.), The Bottlenose Dolphin. Academic Press, San Diego, CA. pp. 245–265. doi: 10.1016/B978-0-12-440280-5.50016-0

Shane, S.H., R.S. Wells, and B. Würsig. 1986. Ecology, behavior and social organization of the bottlenose dolphin: A review. Marine Mammal Science 2:34–63. doi: 10.1111/j.1748-7692.1986.tb00026.x

Smith, C.R., T.K. Rowles, L.B. Hart, F.I. Townsend, R.S. Wells, E.S. Zolman, B.C. Balmer, B. Quigley, M. Ivančić, W. McKercher, M.C. Tumlin, K.D. Mullin, J.D. Adams, Q. Wu, W. McFee, T.K. Collier, and L.H. Schwacke. 2017. Slow recovery of Barataria Bay dolphin health following the Deepwater Horizon oil spill (2013-2014), with evidence of persistent lung disease and impaired stress response. Endangered Species Research 33:127–142. doi: 10.3354/esr00778

Takeshita, R., B.C. Balmer, F. Messina, E.S. Zolman, L. Thomas, R.S. Wells, C.R. Smith, T.K. Rowles, and L.H. Schwacke. 2021. High site-fidelity in common bottlenose dolphins despite low salinity exposure and associated indicators of compromised health. PLoS ONE 16(9):e0258031. doi: 10.1371/journal.pone.0258031

Taylor, B.L., S.J. Chivers, J. Larese, and W.F. Perrin. 2007. Generation length and percent mature estimates for IUCN assessments of cetaceans. National Oceanic and Atmospheric Administration, National Marine Fisheries Service, Southwest Fisheries Science Center: La Jolla, CA. Administrative Report LJ-07-01. 24 p.

Torres, L.G. and A.J. Read. 2009. Where to catch a fish? The influence of foraging tactics on the ecology of bottlenose dolphins (*Tursiops truncatus*) in Florida Bay, Florida. Marine Mammal Science 25(4):797–815. doi: 10.1111/j.1748-7692.2009.00297.x

Tyson, R.B., S.M. Nowacek, and D.P. Nowacek. 2011. Community structure and abundance of bottlenose dolphins *Tursiops truncatus* in coastal waters of the northeast Gulf of Mexico. Marine Ecology Progress Series 438:253–265. doi: 10.3354/meps09292

Urian, K.W., S. Hofmann, R.S. Wells, and A.J. Read. 2009. Fine-scale population structure of bottlenose dolphins (*Tursiops truncatus*) in Tampa Bay, Florida. Marine Mammal Science 25(3):619–638. doi: 10.1111/j.1748-7692.2009.00284.x

Van Bressem, M.F., P.J. Duignan, A. Banyard, M. Barbieri, K.M. Colegrove, S. De Guise, G. Di Guardo, A. Dobson, M. Domingo, D. Fauquier, A. Fernandez, T. Goldstein, B. Grenfell, K.R. Groch, F. Gulland, B.A. Jensen, P.D. Jepson, A. Hall, T. Kuiken, S. Mazzariol, S.E. Morris, O. Nielsen, J.A. Raga, T.K. Rowles, J. Saliki, E. Sierra, N. Stephens, B. Stone, I. Tomo, J. Wang, T. Waltzek, and J.F. Wellehan. 2014. Cetacean morbillivirus: current knowledge and future directions. Viruses 6(12):5145–5181. doi: 10.3390/v6125145

Venn-Watson, S., L. Garrison, J. Litz, E. Fougeres, B. Mase, G. Rappucci, E. Stratton, R. Carmichael, D. Odell, D. Shannon, S. Shippee, S. Smith, L. Staggs, M. Tumlin, H. Whitehead, and T. Rowles. 2015. Demographic clusters identified within the northern Gulf of Mexico common bottlenose dolphin (*Tursiops truncates*) unusual mortality event: January 2010-June 2013. PLoS ONE 10(2):e0117248. doi: 10.1371/journal.pone.0117248

Vollmer, N.L. and P.E. Rosel. 2013. A review of common bottlenose dolphins (*Tursiops truncatus truncatus*) in the Northern Gulf of Mexico: Population biology, potential threats, and management. Southeastern Naturalist 12(m6):1–43. doi: 10.1656/058.012.m601

Vollmer, N.L. and P.E. Rosel. 2017. Fine-scale population structure of common bottlenose dolphins (*Tursiops truncatus*) in offshore and coastal waters of the US Gulf of Mexico. Marine Biology 164(8):160. doi: 10.1007/s00227-017-3186-x

Waring, G.T., E. Josephson, K. Maze-Foley, and P.E. Rosel. 2016. U.S. Atlantic and Gulf of Mexico marine mammal stock assessments - 2015. National Oceanic and Atmospheric Administration, National Marine Fisheries Service, Northeast Fisheries Science Center: Woods Hole, MA. NOAA Technical Memorandum NMFS-NE-238. 501 p. doi: 10.7289/V57S7KTN

Weller, D.W. 1998. Global and regional variation in the biology and behavior of bottlenose dolphins. Texas A&M University. Dissertation. 142 p.

Wells, R.S. 1986. Population structure of bottlenose dolphins: Behavioral studies along the central west coast of Florida. Contract Report to National Marine Fisheries Service, Southeast Fisheries Center. Contract No. 45-WCNF-5-003BB. 58 p.

Wells, R.S. 1991. The role of long-term study in understanding the social structure of a bottlenose dolphin community. In: Pryor, K. and K. Norris (Eds.), Dolphin Societies: Discoveries and Puzzles. University of California Press, Berkeley, CA. pp. 199–226.

Wells, R.S. 2000. Reproduction in wild bottlenose dolphins: Overview of patterns observed during a long-term study. In: Duffield, D. and T. Robeck (Eds.), Bottlenose Dolphin Reproduction Workshop. AZA Marine Mammal Taxon Advisory Group, Silver Spring, MD. pp. 57–74.

Wells, R.S. 2003. Dolphin social complexity: Lessons from long-term study and life history. In: de Waal, F.B.M. and P.L. Tyack (Eds.), Animal Social Complexity: Intelligence, Culture, and Individualized Societies Harvard University Press, Cambridge, MA. pp. 32–56.

Wells, R.S. 2014. Social structure and life history of bottlenose dolphins near Sarasota Bay, Florida: Insights from four decades and five generations. Primates and cetaceans. pp. 149–172. doi: 10.1007/978-4-431-54523-1_8

Wells, R.S., M.K. Bassos, K.W. Urian, W.J. Carr, and M.D. Scott. 1996. Low-level monitoring of bottlenose dolphins, *Tursiops truncatus*, in Charlotte Harbor, Florida: 1990-1994. National Oceanic and Atmospheric Administration, National Marine Fisheries Service, Southeast Fisheries Science Center: Miami, FL. NOAA Technical Memorandum NMFS-SEFSC-384. 86 p.

Wells, R.S., A. Natoli, and G. Braulik. 2019. *Tursiops truncatus* (errata version published in 2019). *The IUCN Red List of Threatened Species* 2019: e.T22563A156932432. https://dx.doi.org/10.2305/IUCN.UK.2019-1.RLTS.T22563A156932432.en

Wells, R.S., L.H. Schwacke, T.K. Rowles, B.C. Balmer, E. Zolman, T. Speakman, F.I. Townsend, M.C. Tumlin, A. Barleycorn, and K.A. Wilkinson. 2017. Ranging patterns of common bottlenose dolphins *Tursiops truncatus* in Barataria Bay, Louisiana, following the Deepwater Horizon oil spill. Endangered Species Research 33:159–180. doi: 10.3354/esr00732

Wells, R.S. and M.D. Scott. 1999. Bottlenose dolphin *Tursiops truncatus* (Montagu, 1821). In: Ridgway, S.H. and R. Harrison (Eds.), Handbook of marine mammals. Volume 6: The second book of dolphins and the porpoises. Academic Press, San Diego, CA. pp. 137–182.

Wells, R.S. and M.D. Scott. 2018. Bottlenose dolphin, *Tursiops truncatus*, common bottlenose dolphin. In: Würsig, B., J.G.M. Thewissen, and K.M. Kovacs (Eds.), Encyclopedia of Marine Mammals (Third Edition). Academic Press, London, UK. pp. 118–125. doi: 10.1016/B978-0-12-804327-1.00072-8

Wells, R.S., M.D. Scott, and A.B. Irvine. 1987. The social structure of free-ranging bottlenose dolphins. In: Genoways, H.H. (Ed.), Current Mammalogy. Plenum Press, New York, NY. pp. 247–305.

Wilson, R.M., R.B. Tyson, J.A. Nelson, B.C. Balmer, J.P. Chanton, and D.P. Nowacek. 2017. Niche differentiation and prey selectivity among common bottlenose dolphins (*Tursiops truncatus*) sighted in St. George Sound, Gulf of Mexico. Frontiers in Marine Science 4:235. doi: 10.3389/fmars.2017.00235

Woshner, V., K. Knott, R. Wells, C. Willetto, R. Swor, and T. O'Hara. 2008. Mercury and selenium in blood and epidermis of bottlenose dolphins (*Tursiops truncatus*) from Sarasota Bay, FL: interaction and relevance to life history and hematologic parameters. Ecohealth 5(3):360–370. doi: 10.1007/s10393-008-0164-2

Common bottlenose dolphin − *Tursiops truncatus*

Biscayne Bay Stock

Overall Vulnerability Rank = Very High

Biological Sensitivity = Very High

Climate Exposure = Very High

Data Quality = 96% of scores ≥ 2

	Tursiops truncatus	Expert Scores	Data Quality	Expert Scores Plots (Portion by Category)
Sensitivity attributes	Prey/Diet Specificity	2.0	2.5	
	Habitat Specificity	3.4	2.2	
	Site Fidelity	3.6	2.2	
	Lifetime Reproductive Potential	1.9	2.2	
	Generation Length	2.7	2.2	
	Reproductive Plasticity	1.1	2.2	
	Migration	3.8	2.5	
	Home Range	3.6	3.0	
	Stock Abundance	2.7	2.8	
	Stock Abundance Trend	2.1	2.2	
	Cumulative Stressors	3.4	1.5	
	Sensitivity Score	Very High		
Exposure variables	Sea Surface Temperature (Change in mean)	4.0	3.0	
	Sea Surface Temperature (Change in variability)	1.0	3.0	
	Air Temperature (Change in mean)	3.2	3.0	
	Air Temperature (Change in variability)	1.0	3.0	
	Precipitation (Change in mean)	1.1	3.0	
	Precipitation (Change in variability)	1.1	3.0	
	Sea Surface Salinity (Change in mean)	3.2	2.8	
	Sea Surface Salinity (Change in variability)	1.1	3.0	
	Ocean pH (Change in mean)	4.0	3.0	
	Ocean pH (Change in variability)	1.0	3.0	
	Sea ice coverage (Change in mean)	1.0	3.0	
	Sea ice coverage (Change in variability)	1.0	3.0	
	Dissolved oxygen (Change in mean)	3.9	3.0	
	Dissolved oxygen (Change in variability)	1.1	3.0	
	Circulation	3.3	2.5	
	Sea level rise	3.9	3.0	
	Exposure Score	Very High		
	Overall Vulnerability Rank	Very High		

Low

Moderate

High

Very High

Common bottlenose dolphin (Biscayne Bay Stock)

Tursiops truncatus

CVA Results Summary

Overall Climate Vulnerability Rank: Very High (100% certainty from bootstrap analysis).

Climate Exposure: Very High. Four exposure factors scored greater than 3.5: Ocean pH (Standard anomaly) (3.95), Sea Surface Temperature (Standard anomaly) (3.95), Dissolved oxygen (Standard anomaly) (3.90), and Sea level rise (3.90).

Biological Sensitivity: Very High. Three sensitivity attributes scored greater than 3.5: Migration (3.85), Home Range (3.65), and Site Fidelity (3.65).

Distributional Response: High

Abundance Response: High

Phenology Response: High

Data Quality: 96% of the data quality scores were 2 or greater, 91% of sensitivity attribute data quality scores were 2 or higher.

Background Information

Stock Description

This stock includes common bottlenose dolphins in Biscayne Bay in the western North Atlantic along the coast of Miami, Florida from Haulover Inlet in the north and Card Sound bridge in the south (Waring et al. 2014).

Prey/Diet Specificity

Generally, common bottlenose dolphins consume a large variety of fish and/or squid (Barros and Odell 1990; Mead and Potter 1990; Wells and Scott 2018). Common bottlenose dolphins seem to show a consistent preference for sound-producing fishes (e.g., sciaenids, toadfish; Berens McCabe et al. 2010; Bowen-Stevens et al. 2021). Estuarine stocks along the southeast U.S. Atlantic coast have been reported to consume a variety of prey, with preference for sciaenids (Gannon and Waples 2004; Pate and McFee 2012). In Sarasota Bay, Florida, Rossman et al. (2015) found some individual common bottlenose dolphins to show diet specialization despite the broader study group showing a generalist diet. Specialization can be influenced by locally abundant prey species (Shane et al. 1986; Vollmer and Rosel 2013) or can result from resource partitioning amongst sympatric populations (e.g., Torres and Read 2009; Wilson et al. 2017).

Habitat Specificity

Common bottlenose dolphins are found in a variety of marine and estuarine habitats (Wells and Scott 2018).

Site Fidelity

The NMFS Southeast Fisheries Science Center has been conducting photo-ID surveys of the common bottlenose dolphin Biscayne Bay Stock since 1990 and suggests 80% of the individuals in the stock are long-term residents (Litz 2007). Other estuarine stocks in the western North Atlantic have shown site fidelity (Waring et al. 2014), such as in South Carolina (Sloan 2006; Waring et al. 2016), Georgia (Pulster and Maruya 2008; Balmer et al. 2011; Kucklick et al. 2011; Balmer et al. 2013), northern Florida (Caldwell 2001), central Florida (Odell and Asper 1990; Mazzoil et al. 2005, 2008a, 2008b), and southern Florida (Litz 2007; Torres 2007; McClellan et al. 2000).

Lifetime Reproductive Potential

Taylor et al. (2007) reported the interbirth interval of common bottlenose dolphins as 3.80 years, though other studies have shown it to be closer to three years (Leatherwood 1977; Wells and Scott 1999; Connor et al. 2000; Wells 2003). Thayer (2008) estimated mean interbirth interval of 2.9 years in North Carolina.

Generally, females reach sexual maturity before males, with females reaching sexual maturity at 5–13 years (Sergeant et al. 1973; Perrin and Reilly 1984; Wells et al. 1987; Mead and Potter 1990; Mann et al. 2000; Wells 2000; Wells and Scott 2018).

Females reproduce through most of their adult lives (Leatherwood 1977; Hohn et al. 1989; Wells and Scott 1999; Wells 2000; Wells 2003), but may produce fewer calves after they reach 30 years of age (Wells 2000). Sarasota Bay common bottlenose dolphins can give birth when they are as young as 6 years of age, and can continue until they are 48 years old (Wells and Scott 2018). Reproductive lifespan would therefore be up to 42 years based on long-term observation studies (Wells 2014; Wells and Scott 2018).

Longitudinal studies from Sarasota Bay, Florida have found that female bottlenose dolphins can live up to 67 years (Wells and Scott 2018). Studies based on growth layer groups in teeth suggest female bottlenose dolphins can live to 41 years along the Texas coast (Fernandez and Hohn 1998) and to 30 years along the Mississippi coast (Mattson et al. 2006).

Generation Length

Taylor et al. (2007) reported common bottlenose dolphin generation length of 20.6–21.1 years.

Reproductive Plasticity

Common bottlenose dolphin calving is generally diffusely seasonal, with one or more peaks, although births have been reported from all seasons (Vollmer and Rosel 2013; Wells and Scott 2018). Calf sightings in South Carolina peak in spring and early summer and again in early autumn (McFee et al. 2014). In North Carolina, Thayer et al. (2003) observed a strong birth peak in spring based on stranding data and a diffuse birth peak from late spring to early fall based on sightings. Calf sightings peak in the spring and summer months in Florida (Wells et al. 1987), Mississippi/Louisiana (Miller et al. 2010; Miller et al. 2013), and Texas (McHugh 1989; Henderson and Würsig 2007).

Migration

The estuarine stocks in the western North Atlantic are thought to primarily comprise stable resident communities. However, transient or seasonal movements of some individuals into and out of stock areas are recorded in some areas (Waring et al. 2014; LaBrecque et al. 2015). Other estuarine stocks in the western North Atlantic are known to spend warm months in estuarine waters then move to nearshore waters during cool months (Gubbins et al. 2003; Durden et al. 2011; Waring et al. 2014; Waring et al. 2016), though some remain entirely in estuarine waters (Mazzoil et al. 2008a).

Home Range

The home range of individuals varies within the Biscayne Bay study area, which is bounded by Haulover Inlet to the north and Barnes Sound the south (Litz 2007). Some individuals are seen throughout the bay whereas others have smaller home ranges within the study area. It is suspected that some individuals may use areas outside these boundaries (Waring et al. 2014).

The home ranges of individuals within the western North Atlantic estuarine stocks generally include the areas within the stock boundaries and brief excursions beyond the stock boundaries (Waring et al. 2014; LaBrecque et al. 2015). In Gulf of Mexico bay, sound, and estuary stocks, some individuals show preference for small areas within the stock boundaries (Lynn and Würsig 2002).

Stock Abundance

Odell (1979) assumed one group of 13 individuals occupied Biscayne Bay. Photo-id surveys have identified 229 unique individuals from 1990 through 2007, with 157 unique individuals identified during surveys conducted between 2003 and 2007, however it is not understood if these were resident individuals or transient individuals from other stocks (Waring et al. 2014).

Stock Abundance Trend

Data are insufficient to complete an abundance trend analysis for this stock (Waring et al. 2014).

Cumulative Stressors

Common bottlenose dolphins face a wide range of individual and cumulative natural and anthropogenic threats (see reviews by Vollmer and Rosel 2013; Wells and Scott 2018; and Wells et al. 2019). These include such threats as shark predation, harmful algal blooms, recreational and commercial fishing gear, vessel traffic, pollution, habitat alteration, and provisioning. In 2018, a dead dolphin stranded in Biscayne Bay with ingested monofilament line which likely contributed to its cause of death. Also in 2018, a dead dolphin stranded in Biscayne Bay with wounds consistent with a vessel strike (NOAA National Marine Mammal Health and Stranding Response Database unpublished data, accessed 18 November 2021).

The Biscayne Bay common bottlenose dolphin stock may interact with crab or lobster pots fisheries or hook and line fisheries, however observer coverage is lacking (Waring et al. 2014). More recently, 2 live dolphins were reported entangled in pot gear in 2020. One was confirmed to be entangled in a commercial blue crab pot and one in an unknown pot/trap. Both were released alive (NOAA National

Marine Mammal Health and Stranding Response Database unpublished data, accessed 18 November 2021).

The nearshore and estuarine habitats along the Atlantic seaboard are adjacent to areas of high human population and some are highly industrialized. Studies of bottlenose dolphin tissues from estuaries in the southeast U.S. have shown persistent organic pollutant (POP; Schwacke et al. 2002, 2012; Hansen et al. 2004; Litz et al. 2007; Pulster et al. 2009; Fair et al. 2010; Yordy et al. 2010; Balmer et al. 2011; Kucklick et al. 2011; ; Reif et al. 2017) and heavy metal (Durden et al. 2007; Stavros et al. 2007, 2008, 2011; Titcomb et al. 2017; Reif et al. 2017; Page-Karjian et al. 2020) concentrations that may result in adverse effects on health or reproductive rates, particularly in urbanized, developed, and agricultural areas. In Biscayne Bay, elevated POP concentrations were observed in individuals associated with the northern, more polluted areas compared to individuals associated with the less polluted areas of Biscayne Bay, suggesting that exposure to pollutants may be highly variable based on geography (Litz et al. 2007; Kucklick et al. 2011).

Individuals of this stock have been reported stranded, with some strandings showing evidence of human interactions (Waring et al. 2014; also see NOAA National Marine Mammal Health and Stranding Response Database).

Further Reading

Waring, G.T., E. Josephson, K. Maze-Foley, P.E. Rosel, T.V.N. Cole, L. Engleby, L.P. Garrison, A. Henry, K. Mullin, C. Orphanides, R.M. Pace, D.L. Palka, M. Lyssikatos, and F.W. Wenzel. 2014. U.S. Atlantic and Gulf of Mexico Marine Mammal Stock Assessments - 2013. National Oceanic and Atmospheric Administration, National Marine Fisheries Service, Northeast Fisheries Science Center: Woods Hole, MA. NOAA Technical Memorandum NMFS-NE-228. 464 p. doi: 10.7289/V51G0J70

Wells, R.S. and M.D. Scott. 2018. Bottlenose Dolphin, *Tursiops truncatus*, Common Bottlenose Dolphin. In: Würsig, B., J.G.M. Thewissen, and K.M. Kovacs (Eds.), Encyclopedia of Marine Mammals (Third Edition). Academic Press, London, UK. pp. 118–125. doi: 10.1016/B978-0-12-804327-1.00072-8

Wells, R.S., A. Natoli, and G. Braulik. 2019. *Tursiops truncatus* (errata version published in 2019). The IUCN Red List of Threatened Species 2019: e.T22563A156932432. https://dx.doi.org/10.2305/IUCN.UK.2019-1.RLTS.T22563A156932432.en

References

Balmer, B.C., L.H. Schwacke, R.S. Wells, J.D. Adams, R. Clay George, S.M. Lane, W.A. McLellan, P.E. Rosel, K. Sparks, T. Speakman, E.S. Zolman, and D. Ann Pabst. 2013. Comparison of abundance and habitat usage for common bottlenose dolphins between sites exposed to differential anthropogenic stressors within the estuaries of southern Georgia, U.S.A. Marine Mammal Science 29(2):E114–E135. doi: 10.1111/j.1748-7692.2012.00598.x

Balmer, B.C., L.H. Schwacke, R.S. Wells, R.C. George, J. Hoguet, J.R. Kucklick, S.M. Lane, A. Martinez, W.A. McLellan, P.E. Rosel, T.K. Rowles, K. Sparks, T. Speakman, E.S. Zolman, and D.A. Pabst. 2011. Relationship between persistent organic pollutants (POPs) and ranging patterns in

common bottlenose dolphins (*Tursiops truncatus*) from coastal Georgia, USA. Science of the Total Environment 409(11):2094–2101. doi: 10.1016/j.scitotenv.2011.01.052

Barros, N.B. and D.K. Odell. 1990. Food habits of bottlenose dolphins in the southeastern United States In: Leatherwood, S. and R.R. Reeves (Eds.), The Bottlenose Dolphin. Academic Press, San Diego, CA. pp. 309–328. doi: 10.1016/B978-0-12-440280-5.50020-2

Berens McCabe, E.J., D.P. Gannon, N.B. Barros, and R.S. Wells. 2010. Prey selection by resident common bottlenose dolphins (*Tursiops truncatus*) in Sarasota Bay, Florida. Marine Biology 157(5):931–942. doi: 10.1007/s00227-009-1371-2

Bowen-Stevens, S.R., D.P. Gannon, R.A. Hazelkorn, G. Lovewell, K.M. Volker, S. Smith, M.C. Tumlin, and J. Litz. 2021. Diet of common bottlenose dolphins, *Tursiops truncatus*, that stranded in and near Barataria Bay, Louisiana, 2010–2012. Southeastern Naturalist 20(1). doi: 10.1656/058.020.0113

Caldwell, M. 2001. Social and genetic structure of bottlenose dolphin (*Tursiops truncatus*) in Jacksonville, Florida. University of Miami. 143 p.

Connor, R.C., R.S. Wells, J. Mann, and A.J. Read. 2000. The bottlenose dolphin: Social relationships in a fission-fusion society. In: Mann, J., R. Connor, P. Tyack, and H. Whitehead (Eds.), Cetacean Societies: Field Studies of Dolphins and Whales. University of Chicago Press, Chicago, IL. pp. 91–126.

Durden, W.N., E.D. Stolen, and M.K. Stolen. 2011. Abundance, distribution, and group composition of Indian River Lagoon bottlenose dolphins (*Tursiops truncatus*). Aquatic Mammals 37(2):175–186. doi: 10.1578/Am.37.2.2011.175

Durden, W.N., M.K. Stolen, D.H. Adams, and E.D. Stolen. 2007. Mercury and selenium concentrations in stranded bottlenose dolphins from the Indian River Lagoon system, Florida. Bulletin of Marine Science 81(1):37–54.

Fair, P.A., J. Adams, G. Mitchum, T.C. Hulsey, J.S. Reif, M. Houde, D. Muir, E. Wirth, D. Wetzel, E. Zolman, W. McFee, and G.D. Bossart. 2010. Contaminant blubber burdens in Atlantic bottlenose dolphins (*Tursiops truncatus*) from two southeastern US estuarine areas: Concentrations and patterns of PCBs, pesticides, PBDEs, PFCs, and PAHs. Science of the Total Environment 408(7):1577–1597. doi: 10.1016/j.scitotenv.2009.12.021

Fernandez, S. and A.A. Hohn. 1998. Age, growth, and calving season of bottlenose dolphins, *Tursiops truncatus*, off coastal Texas. Fishery Bulletin 96(2):357–365.

Gannon, D.P. and D.M. Waples. 2004. Diets of coastal bottlenose dolphins from the U. S. Mid-Atlantic coast differ by habitat. Marine Mammal Science 20(3):527–545. doi: 10.1111/j.1748-7692.2004.tb01177.x

Gubbins, C.M., M. Caldwell, S.G. Barco, K.A. Rittmaster, N. Bowles, and V.G. Thayer. 2003. Abundance and sighting patterns of bottlenose dolphins (*Tursiops truncatus*) at four northwest Atlantic coastal sites. Journal of Cetacean Research and Management 5(2):141–148.

Hansen, L.J., L.H. Schwacke, G.B. Mitchum, A.A. Hohn, R.S. Wells, E.S. Zolman, and P.A. Fair. 2004. Geographic variation in polychorinated biphenyl and organochlorine pesticide concentrations in the blubber of bottlenose dolphins from the US Atlantic coast. Science of the Total Environment 319(1-3):147–172. doi: 10.1016/S0048-9697(03)00371-1

Henderson, E.E. and B. Würsig. 2007. Behavior patterns of bottlenose dolphins in San Luis Pass, Texas. Gulf of Mexico Science 25(2):153–161. doi: 10.18785/goms.2502.06

Hohn, A.A., M.D. Scott, R.S. Wells, J.C. Sweeney, and A.B. Irvine. 1989. Growth layers in teeth from known-age, free-ranging bottlenose dolphins. Marine Mammal Science 5(4):315–342. doi: 10.1111/j.1748-7692.1989.tb00346.x

Kucklick, J., L. Schwacke, R. Wells, A. Hohn, A. Guichard, J. Yordy, L. Hansen, E. Zolman, R. Wilson, J. Litz, D. Nowacek, T. Rowles, R. Pugh, B. Balmer, C. Sinclair, and P. Rosel. 2011. Bottlenose dolphins as indicators of persistent organic pollutants in the western North Atlantic Ocean and Northern

Gulf of Mexico. Environmental Science & Technology 45(10):4270–4277. doi: 10.1021/es1042244
LaBrecque, E., C. Curtice, J. Harrison, S.M. Van Parijs, and P.N. Halpin. 2015. 2. Biologically Important Areas for Cetaceans Within U.S. Waters – East Coast Region. Aquatic Mammals 41(1):17–29. doi: 10.1578/am.41.1.2015.17
Leatherwood, S. 1977. Some preliminary impressions on the numbers and social behavior of free-swimming bottlenosed dolphin calves (*Tursiops truncatus*) in the northern Gulf of Mexico. In: Ridgway, S.H. and K. Bernirschke (Eds.), Breeding Dolphins; Present Status, Suggestions for the Future. Final report to the US Marine Mammal Commission, Contract MM6AC009, MMC-76/07, Washington, DC. pp. 143–167.
Litz, J.A. 2007. Social structure, genetic structure, and persistent organohalogen pollutants in bottlenose dolphins (*Tursiops truncatus*) in Biscayne Bay, Florida. University of Miami. 140 p.
Litz, J.A., L.P. Garrison, L.A. Fieber, A. Martinez, J.P. Contillo, and J.R. Kucklick. 2007. Fine-scale spatial variation of persistent organic pollutants in bottlenose dolphins (*Tursiops truncatus*) in Biscayne Bay, Florida. Environmental Science & Technology 41(21):7222–7228. doi: 10.1021/es070440r
Lynn, S.K. and B. Würsig. 2002. Summer movement patterns of bottlenose dolphins in a Texas bay. Gulf of Mexico Science 20(1):25–37. doi: 10.18785/goms.2001.03
Mann, J., R.C. Connor, L.M. Barre, and M.R. Heithaus. 2000. Female reproductive success in bottlenose dolphins (*Tursiops* sp.): life history, habitat, provisioning, and group-size effects. Behavioral Ecology 11(2):210–219. doi: 10.1093/beheco/11.2.210
Mattson, M.C., K.D. Mullin, G.W. Ingram, and W. Hoggard. 2006. Age structure and growth of the bottlenose dolphin (*Tursiops truncatus*) from strandings in the Mississippi sound region of the north-central Gulf of Mexico from 1986 to 2003. Marine Mammal Science 22(3):654–666. doi: 10.1111/j.1748-7692.2006.00057.x
Mazzoil, M., S.D. McCulloch, and R.H. Defran. 2005. Observations on the site fidelity of bottlenose dolphins (*Tursiops truncatus*) in the Indian River Lagoon, Florida. Florida Scientist 68(4):217–226.
Mazzoil, M., J.S. Reif, M. Youngbluth, M.E. Murdoch, S.E. Bechdel, E. Howells, S.D. McCulloch, L.J. Hansen, and G.D. Bossart. 2008a. Home ranges of bottlenose dolphins (*Tursiops truncatus*) in the Indian River Lagoon, Florida: Environmental correlates and implications for management strategies. Ecohealth 5(3):278–288. doi: 10.1007/s10393-008-0194-9
Mazzoil, M.S., S.D. McCulloch, M.J. Youngbluth, D.S. Kilpatrick, M.E. Murdoch, B. Mase-Guthrie, D.K. Odell, and G.D. Bossart. 2008b. Radio-tracking and survivorship of two rehabilitated bottlenose dolphins (*Tursiops truncatus*) in the Indian River Lagoon, Florida. Aquatic Mammals 34:54–64. doi: 10.1578/AM.34.1.2008.54
McClellan, D.B., J. Browder, J.L. Tobias, G.J. Konoval, M.D. Heron, O.L. Bass, and J. Osborne. 2000. Opportunistic sightings of bottlenose dolphin, *Tursiops truncatus*, along the southeast Florida coast and Florida Bay, 1992-1997. National Oceanic and Atmospheric Administration, National Marine Fisheries Service, Southeast Fisheries Science Center: Miami, FL. NOAA Technical Memorandum NMFS-SEFSC-435. 18 p.
McFee, W.E., T.R. Speakman, L. Balthis, J.D. Adams, and E.S. Zolman. 2014. Reproductive seasonality of a recently designated bottlenose dolphin stock near Charleston, South Carolina, U.S.A. Marine Mammal Science 30(2):528–543. doi: 10.1111/mms.12055
McHugh, M.B. 1989. Population numbers and feeding behavior of the Atlantic bottlenose dolphin (*Tursiops truncatus*) near Aransas Pass, Texas. University of Texas at Austin. Thesis. 96 p.
Mead, J.G. and C.W. Potter. 1990. Natural history of bottlenose dolphins along the central Atlantic coast of the United States. In: Leatherwood, S. and R.R. Reeves (Eds.), The Bottlenose Dolphin. Academic Press, San Diego, CA. pp. 165–195.

Miller, L.J., A.D. Mackey, T. Hoffland, M. Solangi, and S.A. Kuczaj. 2010. Potential effects of a major hurricane on Atlantic bottlenose dolphin (*Tursiops truncatus*) reproduction in the Mississippi Sound. Marine Mammal Science 26(3):707–715. doi: 10.1111/j.1748-7692.2010.00371.x

Miller, L.J., A.D. Mackey, M. Solangi, and S.A. Kuczaj. 2013. Population abundance and habitat utilization of bottlenose dolphins in the Mississippi Sound. Aquatic Conservation: Marine and Freshwater Ecosystems 23(1):145–151. doi: 10.1002/aqc.2278

Odell, D.K. 1979. A preliminary study of the ecology and population biology of the bottlenose dolphin in Southeast Florida; Final report to the Marine Mammal Commission in fulfillment of contract MM4AC003. University of Miami: Miami, FL. 26 p.

Odell, D.K. and E.D. Asper. 1990. Distribution and movements of freeze-branded bottlenose dolphins in the Indian and Banana Rivers, Florida. In: Leatherwood, S. and R. Reeves (Eds.), The Bottlenose Dolphin. Academic Press, San Diego, CA. pp. 515–540.

Page-Karjian, A., C.F. Lo, B. Ritchie, C.A. Harms, D.S. Rotstein, S. Han, S.M. Hassan, A.F. Lehner, J.P. Buchweitz, V.G. Thayer, J.M. Sullivan, E.F. Christiansen, and J.R. Perrault. 2020. Anthropogenic contaminants and histopathological findings in stranded cetaceans in the southeastern United States, 2012–2018. Frontiers in Marine Science 7:630. doi: 10.3389/fmars.2020.00630

Pate, S.M. and W.E. McFee. 2012. Prey species of bottlenose dolphins (*Tursiops truncatus*) from South Carolina Waters. Southeastern Naturalist 11(1):1–22. doi: 10.1656/058.011.0101

Perrin, W.F. and S.B. Reilly. 1984. Reproductive parameters of dolphins and small whales of the family Delphinidae. In: Perrin, W.F., R.L. Brownell, Jr., and D.P. DeMaster (Eds.), Reproduction in Whales, Dolphins, and Porpoises. pp. 97–134.

Pulster, E.L. and K.A. Maruya. 2008. Geographic specificity of Aroclor 1268 in bottlenose dolphins (*Tursiops truncatus*) frequenting the Turtle/Brunswick River Estuary, Georgia (USA). Science of the Total Environment 393(2-3):367–375. doi: 10.1016/j.scitotenv.2007.12.031

Pulster, E.L., K.L. Smalling, E. Zolman, L. Schwacke, and K.A. Maruya. 2009. Persistent organochlorine pollutants and toxaphene congener profiles in bottlenose dolphins (*Tursiops truncatus*) frequenting the Turtle/Brunswick River Estuary, Georgia, USA. Environmental Toxicology and Chemistry 28(7):1390–1399. doi: 10.1897/08-240.1

Reif, J.S., A.M. Schaefer, G.D. Bossart, and P.A. Fair. 2017. Health and Environmental Risk Assessment Project for bottlenose dolphins *Tursiops truncatus* from the southeastern USA. II. Environmental aspects. Diseases of Aquatic Organisms 125(2):155–166. doi: 10.3354/dao03143

Rossman, S., P.H. Ostrom, M. Stolen, N.B. Barros, H. Gandhi, C.A. Stricker, and R.S. Wells. 2015. Individual specialization in the foraging habits of female bottlenose dolphins living in a trophically diverse and habitat rich estuary. Oecologia 178(2):415–425. doi: 10.1007/s00442-015-3241-6

Schwacke, L.H., E.O. Voit, L.J. Hansen, R.S. Wells, G.B. Mitchum, A.A. Hohn, and P.A. Fair. 2002. Probabilistic risk assessment of reproductive effects of polychlorinated biphenyls on bottlenose dolphins (*Tursiops truncatus*) from the southeast United States coast. Environmental Toxicology and Chemistry 21(12):2752–2764. doi: 10.1002/etc.5620211232

Schwacke, L.H., E.S. Zolman, B.C. Balmer, S. De Guise, R.C. George, J. Hoguet, A.A. Hohn, J.R. Kucklick, S. Lamb, M. Levin, J.A. Litz, W.E. McFee, N.J. Place, F.I. Townsend, R.S. Wells, and T.K. Rowles. 2012. Anaemia, hypothyroidism and immune suppression associated with polychlorinated biphenyl exposure in bottlenose dolphins (*Tursiops truncatus*). Proceedings of the Royal Society B: Biological Sciences 279(1726):48–57. doi: 10.1098/rspb.2011.0665

Sergeant, D.E., D.K. Caldwell, and M.C. Caldwell. 1973. Age, growth, and maturity of bottlenosed dolphin (*Tursiops-truncatus*) from northeast Florida. Journal of the Fisheries Research Board of Canada 30(7):1009–1011. doi: 10.1139/f73-165

Shane, S.H., R.S. Wells, and B. Würsig. 1986. Ecology, behavior and social organization of the bottlenose dolphin: A review. Marine Mammal Science 2:34–63. doi: 10.1111/j.1748-7692.1986.tb00026.x

Sloan, P.E. 2006. Residency patterns, seasonality and habitat use among bottlenose dolphins, *Tursiops truncatus*, in the Cape Romain National Wildlife Refuge, S.C. University of North Carolina Wilmington. Thesis. 75 p.

Stavros, H.-C.W., G.D. Bossart, T.C. Hulsey, and P.A. Fair. 2007. Trace element concentrations in skin of free-ranging bottlenose dolphins (*Tursiops truncatus*) from the southeast Atlantic coast. Science of the Total Environment 388(1-3):300–315. doi: 10.1016/j.scitotenv.2007.07.030

Stavros, H.-C.W., G.D. Bossart, T.C. Hulsey, and P.A. Fair. 2008. Trace element concentrations in blood of free-ranging bottlenose dolphins (*Tursiops truncatus*): Influence of age, sex and location. Marine Pollution Bulletin 56(2):371–379. doi: 10.1016/j.marpolbul.2007.10.030

Stavros, H.-C.W., M. Stolen, W.N. Durden, W. McFee, G.D. Bossart, and P.A. Fair. 2011. Correlation and toxicological inference of trace elements in tissues from stranded and free-ranging bottlenose dolphins (*Tursiops truncatus*). Chemosphere 82(11):1649–1661. doi: 10.1016/j.chemosphere.2010.11.019

Taylor, B.L., S.J. Chivers, J. Larese, and W.F. Perrin. 2007. Generation length and percent mature estimates for IUCN assessments of cetaceans. National Oceanic and Atmospheric Administration, National Marine Fisheries Service, Southwest Fisheries Science Center: La Jolla, CA. Administrative Report LJ-07-01. 24 p.

Thayer, V.G. 2008. Life history parameters and social associations of female bottlenose dolphins (*Tursiops truncatus*) off North Carolina, USA. Duke University. Dissertation. 164 p.

Thayer, V.G., A.J. Read, A.S. Friedlaender, D.R. Colby, A.A. Hohn, W.A. McLellan, D.A. Pabst, J.L. Dearolf, N.I. Bowles, J.R. Russell, and K.A. Rittmaster. 2003. Reproductive seasonality of western Atlantic bottlenose dolphins off North Carolina, USA. Marine Mammal Science 19(4):617–629. doi: 10.1111/j.1748-7692.2003.tb01120.x

Titcomb, E.M., J.S. Reif, P.A. Fair, H.-C.W. Stavros, M. Mazzoil, G.D. Bossart, and A.M. Schaefer. 2017. Blood mercury concentrations in common bottlenose dolphins from the Indian River Lagoon, Florida: Patterns of social distribution. Marine Mammal Science 33(3):771–784. doi: 10.1111/mms.12390

Torres, L.G. 2007. Top predator distribution and foraging ecology in Florida Bay, Florida. Duke University. Dissertation. 225 p.

Torres, L.G. and A.J. Read. 2009. Where to catch a fish? The influence of foraging tactics on the ecology of bottlenose dolphins (*Tursiops truncatus*) in Florida Bay, Florida. Marine Mammal Science 25(4):797–815. doi: 10.1111/j.1748-7692.2009.00297.x

Vollmer, N.L. and P.E. Rosel. 2013. A review of common bottlenose dolphins (*Tursiops truncatus truncatus*) in the Northern Gulf of Mexico: Population biology, potential threats, and management. Southeastern Naturalist 12(m6):1–43. doi: 10.1656/058.012.m601

Waring, G., E. Josephson, K. Maze-Foley, and P. Rosel. 2014. U.S. Atlantic and Gulf of Mexico Marine Mammal Stock Assessments - 2013. National Oceanic and Atmospheric Administration, National Marine Fisheries Service, Northeast Fisheries Science Center: Woods Hole, MA. NOAA Technical Memorandum NMFS-NE-228. 464 p. doi: 10.7289/V51G0J70

Waring, G.T., E. Josephson, K. Maze-Foley, and P.E. Rosel. 2016. U.S. Atlantic and Gulf of Mexico marine mammal stock assessments - 2015. National Oceanic and Atmospheric Administration, National Marine Fisheries Service, Northeast Fisheries Science Center: Woods Hole, MA. NOAA Technical Memorandum NMFS-NE-238. 501 p. doi: 10.7289/V57S7KTN

Wells, R.S. 2000. Reproduction in wild bottlenose dolphins: Overview of patterns observed during a long-term study. In: Duffield, D. and T. Robeck (Eds.), Bottlenose Dolphin Reproduction Workshop. AZA Marine Mammal Taxon Advisory Group, Silver Spring, MD. pp. 57–74.

Wells, R.S. 2003. Dolphin social complexity: Lessons from long-term study and life history. In: de Waal, F.B.M. and P.L. Tyack (Eds.), Animal Social Complexity: Intelligence, Culture, and Individualized Societies Harvard University Press, Cambridge, MA. pp. 32–56.

Wells, R.S. 2014. Social structure and life history of bottlenose dolphins near Sarasota Bay, Florida: Insights from four decades and five generations. Primates and cetaceans. pp. 149–172. doi: 10.1007/978-4-431-54523-1_8

Wells, R.S., A. Natoli, and G. Braulik. 2019. *Tursiops truncatus* (errata version published in 2019). *The IUCN Red List of Threatened Species* 2019: e.T22563A156932432. https://dx.doi.org/10.2305/IUCN.UK.2019-1.RLTS.T22563A156932432.en

Wells, R.S. and M.D. Scott. 1999. Bottlenose dolphin *Tursiops truncatus* (Montagu, 1821). In: Ridgway, S.H. and R. Harrison (Eds.), Handbook of marine mammals. Volume 6: The second book of dolphins and the porpoises. Academic Press, San Diego, CA. pp. 137–182.

Wells, R.S. and M.D. Scott. 2018. Bottlenose dolphin, *Tursiops truncatus*, common bottlenose dolphin. In: Würsig, B., J.G.M. Thewissen, and K.M. Kovacs (Eds.), Encyclopedia of Marine Mammals (Third Edition). Academic Press, London, UK. pp. 118–125. doi: 10.1016/B978-0-12-804327-1.00072-8

Wells, R.S., M.D. Scott, and A.B. Irvine. 1987. The social structure of free-ranging bottlenose dolphins. In: Genoways, H.H. (Ed.), Current Mammalogy. Plenum Press, New York, NY. pp. 247–305.

Wilson, R.M., R.B. Tyson, J.A. Nelson, B.C. Balmer, J.P. Chanton, and D.P. Nowacek. 2017. Niche differentiation and prey selectivity among common bottlenose dolphins (*Tursiops truncatus*) sighted in St. George Sound, Gulf of Mexico. Frontiers in Marine Science 4:235. doi: 10.3389/fmars.2017.00235

Yordy, J.E., M.A. Mollenhauer, R.M. Wilson, R.S. Wells, A. Hohn, J. Sweeney, L.H. Schwacke, T.K. Rowles, J.R. Kucklick, and M.M. Peden-Adams. 2010. Complex contaminant exposure in cetaceans: a comparative E-Screen analysis of bottlenose dolphin blubber and mixtures of four persistent organic pollutants. Environmental Toxicology and Chemistry 29(10):2143–2153. doi: 10.1002/etc.279

Common bottlenose dolphin − *Tursiops truncatus*

Central Georgia Estuarine System Stock

Overall Vulnerability Rank = Very High

Biological Sensitivity = Very High

Climate Exposure = Very High

Data Quality = 93% of scores ≥ 2

	Tursiops truncatus	Expert Scores	Data Quality	Expert Scores Plots (Portion by Category)
Sensitivity attributes	Prey/Diet Specificity	1.8	2.0	
	Habitat Specificity	3.8	2.0	
	Site Fidelity	3.9	2.8	
	Lifetime Reproductive Potential	1.0	2.2	
	Generation Length	2.8	2.2	
	Reproductive Plasticity	1.4	2.2	
	Migration	3.9	2.8	
	Home Range	3.1	2.8	
	Stock Abundance	3.0	2.8	
	Stock Abundance Trend	2.1	1.8	
	Cumulative Stressors	2.8	2.5	
	Sensitivity Score	Very High		
Exposure variables	Sea Surface Temperature (Change in mean)	4.0	3.0	
	Sea Surface Temperature (Change in variability)	1.3	3.0	
	Air Temperature (Change in mean)	4.0	3.0	
	Air Temperature (Change in variability)	1.0	3.0	
	Precipitation (Change in mean)	1.1	3.0	
	Precipitation (Change in variability)	1.9	3.0	
	Sea Surface Salinity (Change in mean)	2.3	3.0	
	Sea Surface Salinity (Change in variability)	1.0	3.0	
	Ocean pH (Change in mean)	4.0	2.7	
	Ocean pH (Change in variability)	1.0	2.7	
	Sea ice coverage (Change in mean)	1.0	3.0	
	Sea ice coverage (Change in variability)	1.0	3.0	
	Dissolved oxygen (Change in mean)	4.0	2.7	
	Dissolved oxygen (Change in variability)	1.0	2.7	
	Circulation	4.0	1.7	
	Sea level rise	4.0	3.0	
	Exposure Score	Very High		
Overall Vulnerability Rank		Very High		

Low
Moderate
High
Very High

Common bottlenose dolphin (Central Georgia Estuarine System Stock)

Tursiops truncatus

CVA Results Summary

Overall Climate Vulnerability Rank: Very High (100% certainty from bootstrap analysis).

Climate Exposure: Very High. Six exposure factors scored greater than 3.5: Air Temperature (Standard anomaly) (4.00), Circulation (4.00), Dissolved oxygen (Standard anomaly) (4.00), Ocean pH (Standard anomaly) (4.00), Sea level rise (4.00), and Sea Surface Temperature (Standard anomaly) (4.00).

Biological Sensitivity: Very High. Three sensitivity attributes scored greater than 3.5: Migration (3.90), Site Fidelity (3.90), and Habitat Specificity (3.75).

Distributional Response: High

Abundance Response: High

Phenology Response: High

Data Quality: 93% of the data quality scores were 2 or greater, 91% of sensitivity attribute data quality scores were 2 or higher.

Background Information

Stock Description

The Central Georgia Estuarine System (CGES) Stock includes common bottlenose dolphins found in the estuarine and nearshore (≤ 1km from shore) coastal waters of the western North Atlantic along the Georgia coast from the northern extent of Ossabaw Sound in the north to the Altamaha River in the south (Waring et al. 2016).

Prey/Diet Specificity

Generally, common bottlenose dolphins consume a large variety of fish and/or squid (Barros and Odell 1990; Mead and Potter 1990; Wells and Scott 2018). Common bottlenose dolphins seem to show a consistent preference for sound-producing fishes (e.g., sciaenids, toadfish; Berens McCabe et al. 2010; Bowen-Stevens et al. 2021). Estuarine stocks along the southeast U.S. Atlantic coast have been reported to consume a variety of prey, with preference for sciaenids (Gannon and Waples 2004; Pate and McFee 2012). In Sarasota Bay, Florida, Rossman et al. (2015) found some individual common bottlenose dolphins to show diet specialization despite the broader study group showing a generalist diet. Specialization can be influenced by locally abundant prey species (Shane et al. 1986; Vollmer and Rosel 2013) or can result from resource partitioning amongst sympatric populations (e.g., Torres and Read 2009; Wilson et al. 2017).

Habitat Specificity

Common bottlenose dolphins are found in a variety of marine and estuarine habitats (Wells and Scott 2018). The area inhabited by the CGES stock is characterized by extensive estuarine tidal marsh systems that include the Altamaha River (Waring et al. 2016).

Site Fidelity

Common bottlenose dolphins in central and southern Georgia show strong site fidelity (Balmer et al. 2011; Kucklick et al. 2011; Balmer et al. 2013). Other estuarine stocks in the western North Atlantic have shown site fidelity (Waring et al. 2016), such as in South Carolina (Sloan 2006; Waring et al. 2016), northern Florida (Caldwell 2001), central Florida (Odell and Asper 1990; Mazzoil et al. 2005, 2008a, 2008b), and southern Florida (Litz 2007; Torres 2007; McClellan et al. 2000).

Lifetime Reproductive Potential

Taylor et al. (2007) reported the interbirth interval of common bottlenose dolphins as 3.80 years, though other studies have shown it to be closer to three years (Leatherwood 1977; Wells and Scott 1999; Connor et al. 2000; Wells 2003). Thayer (2008) estimated mean interbirth interval of 2.9 years in North Carolina.

Generally, females reach sexual maturity before males, with females reaching sexual maturity at 5–13 years (Sergeant et al. 1973; Perrin and Reilly 1984; Wells et al. 1987; Mead and Potter 1990; Mann et al. 2000; Wells 2000; Wells and Scott 2018).

Females reproduce through most of their adult lives (Leatherwood 1977; Hohn et al. 1989; Wells and Scott 1999; Wells 2000; Wells 2003), but may produce fewer calves after they reach 30 years of age (Wells 2000). Sarasota Bay common bottlenose dolphins can give birth when they are as young as 6 years of age, and can continue until they are 48 years old (Wells and Scott 2018). Reproductive lifespan would therefore be up to 42 years based on long-term observation studies (Wells 2014; Wells and Scott 2018).

Longitudinal studies from Sarasota Bay, Florida have found that female bottlenose dolphins can live up to 67 years (Wells and Scott 2018). Studies based on growth layer groups in teeth suggest female bottlenose dolphins can live to 41 years along the Texas coast (Fernandez and Hohn 1998) and to 30 years along the Mississippi coast (Mattson et al. 2006).

Generation Length

Taylor et al. (2007) reported common bottlenose dolphin generation length of 20.6–21.1 years.

Reproductive Plasticity

Common bottlenose dolphin calving is generally diffusely seasonal, with one or more peaks, although births have been reported from all seasons (Vollmer and Rosel 2013; Wells and Scott 2018). Calf sightings in South Carolina peak in spring and early summer and again in early autumn (McFee et al. 2014). In North Carolina, Thayer et al. (2003) observed a strong birth peak in spring based on stranding data and a diffuse birth peak from late spring to early fall based on sightings. Calf sightings peak in the

spring and summer months in Florida (Wells et al. 1987), Mississippi/Louisiana (Miller et al. 2010; Miller et al. 2013), and Texas (McHugh 1989; Henderson and Würsig 2007).

Migration

The estuarine stocks in the western North Atlantic are thought to primarily comprise stable resident communities. However, transient or seasonal movements of some individuals into and out of stock areas are recorded in some areas (LaBrecque et al. 2015; Waring et al. 2016).

Other estuarine stocks in the western North Atlantic are known to spend warm months in estuarine waters then move to nearshore waters during cool months (Gubbins et al. 2003; Durden et al. 2011; Waring et al. 2014; Waring et al. 2016), though some remain entirely in estuarine waters (Mazzoil et al. 2008a).

Home Range

The home ranges of individuals within the western North Atlantic estuarine stocks generally include the areas within the stock boundaries and brief excursions beyond the stock boundaries (LaBrecque et al. 2015; Waring et al. 2016). Studies have shown dolphin populations with constrained ranges in Georgia (Brunswick and Sapelo; Balmer et al. 2013); South Carolina (Silva et al. 2019), and North Carolina (Urian et al. 2013). Balmer et al. (2018), using satellite telemetry data, identified that dolphins considered to be members of the SGES Stock had different movement patterns with some individuals remaining entirely within the estuarine waters, while other animals use the larger estuarine waters, sounds, and to some degree coastal waters.

Stock Abundance

Balmer et al. (2013) estimated an average abundance of 192 individuals (CV=0.04), based on winter surveys from 2008 and 2009 from approximately half of the entire range of the CGES.

Stock Abundance Trend

Data are insufficient to complete an abundance trend analysis for this stock (Waring et al. 2016).

Cumulative Stressors

Common bottlenose dolphins face a wide range of individual and cumulative natural and anthropogenic threats (see reviews by Vollmer and Rosel 2013; Wells and Scott 2018; and Wells et al. 2019). These include such threats as shark predation, harmful algal blooms, recreational and commercial fishing gear, vessel traffic, pollution, habitat alteration, and provisioning.

The Central Georgia Estuarine Stock common bottlenose dolphin stock interacts with the blue crab trap/pot fishery, however levels of serious injury and mortality are unknown due to a lack of observer coverage (Waring et al. 2016). Individuals of this stock have been reported stranded, with some strandings showing evidence of human interactions, and other strandings occurring during Unusual Mortality Events (UMEs; Waring et al. 2014; also see NOAA National Marine Mammal Health and Stranding Response Database). During the period 2009–2013, 24 common bottlenose dolphins stranded within the CGES, with two individuals presenting interactions with crab trap/pot gear (Waring et al.

2014; Seguel et al. 2020). The cause of death for six of the other stranded individuals was likely high concentrations of persistent organic pollutants (POPs; Seguel et al. 2020).

The nearshore and estuarine habitats along the Atlantic seaboard are adjacent to areas of high human population and some are highly industrialized. Studies of bottlenose dolphin tissues from estuaries in the southeast U.S. have shown persistent organic pollutant (POP; Schwacke et al. 2002, 2012; Hansen et al. 2004; Litz et al. 2007; Pulster et al. 2009; Fair et al. 2010; Yordy et al. 2010; Balmer et al. 2011; Kucklick et al. 2011; Reif et al. 2017) and heavy metal (Durden et al. 2007; Stavros et al. 2007, 2008, 2011; Titcomb et al. 2017; Reif et al. 2017; Page-Karjian et al. 2020) concentrations that may result in adverse effects on health or reproductive rates, particularly in urbanized, developed, and agricultural areas. In Biscayne Bay, Florida, elevated POP concentrations were observed in individuals associated with more polluted areas compared to individuals associated with the less polluted areas of Biscayne Bay, suggesting that exposure to pollutants may be highly variable based on geography (Litz et al. 2007; Kucklick et al. 2011). Polychlorinated biphenyls (PCBs) have been found in Sapelo Island waters within the boundaries of this stock, though at lower concentrations than nearby Brunswick, GA (Wirth et al. 2014).

A UME was declared in the summer of 2013 for the mid-Atlantic coast from New York to Brevard County, Florida, with more than 1500 common bottlenose dolphins stranding as a result of morbillivirus (Waring et al. 2016). Strandings were more prevalent along oceanic shores than in estuaries, suggesting that coastal stocks were more impacted by the UME than estuarine stocks (Waring et al. 2016).

Balmer et al. (2018) found coastal dolphins in southern Georgia had a significantly higher prevalence of positive dolphin morbillivirus (DMV) antibody titers (0.67; N = 2/3) than sound and estuary dolphins (0.13; N = 2/16), suggesting the coastal stock may have experienced greater exposure to DMV as compared to the sound and estuary stocks, which may be vulnerable to DMV infection in the future.

Further Reading

Waring, G.T., E. Josephson, K. Maze-Foley, and P.E. Rosel. 2016. U.S. Atlantic and Gulf of Mexico Marine Mammal Stock Assessments - 2015. National Oceanic and Atmospheric Administration, National Marine Fisheries Service, Northeast Fisheries Science Center: Woods Hole, MA. NOAA Technical Memorandum NMFS-NE-238. 501 p. doi: 10.7289/V57S7KTN

Wells, R.S. and M.D. Scott. 2018. Bottlenose Dolphin, *Tursiops truncatus*, Common Bottlenose Dolphin. In: Würsig, B., J.G.M. Thewissen, and K.M. Kovacs (Eds.), Encyclopedia of Marine Mammals (Third Edition). Academic Press, London, UK. pp. 118–125. doi: 10.1016/B978-0-12-804327-1.00072-8

Wells, R.S., A. Natoli, and G. Braulik. 2019. *Tursiops truncatus* (errata version published in 2019). The IUCN Red List of Threatened Species 2019: e.T22563A156932432. https://dx.doi.org/10.2305/IUCN.UK.2019-1.RLTS.T22563A156932432.en

References

Balmer, B., E. Zolman, T. Rowles, C. Smith, F. Townsend, D. Fauquier, C. George, T. Goldstein, L. Hansen, B. Quigley, W. McFee, J. Morey, P. Rosel, J. Saliki, T. Speakman, and L. Schwacke. 2018. Ranging patterns, spatial overlap, and association with dolphin morbillivirus exposure in common bottlenose dolphins (*Tursiops truncatus*) along the Georgia, USA coast. Ecology and Evolution 8(24):12890–12904. doi: 10.1002/ece3.4727

Balmer, B.C., L.H. Schwacke, R.S. Wells, J.D. Adams, R. Clay George, S.M. Lane, W.A. McLellan, P.E. Rosel, K. Sparks, T. Speakman, E.S. Zolman, and D. Ann Pabst. 2013. Comparison of abundance and habitat usage for common bottlenose dolphins between sites exposed to differential anthropogenic stressors within the estuaries of southern Georgia, U.S.A. Marine Mammal Science 29(2):E114–E135. doi: 10.1111/j.1748-7692.2012.00598.x

Balmer, B.C., L.H. Schwacke, R.S. Wells, R.C. George, J. Hoguet, J.R. Kucklick, S.M. Lane, A. Martinez, W.A. McLellan, P.E. Rosel, T.K. Rowles, K. Sparks, T. Speakman, E.S. Zolman, and D.A. Pabst. 2011. Relationship between persistent organic pollutants (POPs) and ranging patterns in common bottlenose dolphins (*Tursiops truncatus*) from coastal Georgia, USA. Science of the Total Environment 409(11):2094–2101. doi: 10.1016/j.scitotenv.2011.01.052

Barros, N.B. and D.K. Odell. 1990. Food habits of bottlenose dolphins in the southeastern United States In: Leatherwood, S. and R.R. Reeves (Eds.), The Bottlenose Dolphin. Academic Press, San Diego, CA. pp. 309–328. doi: 10.1016/B978-0-12-440280-5.50020-2

Berens McCabe, E.J., D.P. Gannon, N.B. Barros, and R.S. Wells. 2010. Prey selection by resident common bottlenose dolphins (*Tursiops truncatus*) in Sarasota Bay, Florida. Marine Biology 157(5):931–942. doi: 10.1007/s00227-009-1371-2

Bowen-Stevens, S.R., D.P. Gannon, R.A. Hazelkorn, G. Lovewell, K.M. Volker, S. Smith, M.C. Tumlin, and J. Litz. 2021. Diet of common bottlenose dolphins, *Tursiops truncatus*, that stranded in and near Barataria Bay, Louisiana, 2010–2012. Southeastern Naturalist 20(1). doi: 10.1656/058.020.0113

Caldwell, M. 2001. Social and genetic structure of bottlenose dolphin (*Tursiops truncatus*) in Jacksonville, Florida. University of Miami. 143 p.

Connor, R.C., R.S. Wells, J. Mann, and A.J. Read. 2000. The bottlenose dolphin: Social relationships in a fission-fusion society. In: Mann, J., R. Connor, P. Tyack, and H. Whitehead (Eds.), Cetacean Societies: Field Studies of Dolphins and Whales. University of Chicago Press, Chicago, IL. pp. 91–126.

Durden, W.N., E.D. Stolen, and M.K. Stolen. 2011. Abundance, distribution, and group composition of Indian River Lagoon bottlenose dolphins (*Tursiops truncatus*). Aquatic Mammals 37(2):175–186. doi: 10.1578/Am.37.2.2011.175

Durden, W.N., M.K. Stolen, D.H. Adams, and E.D. Stolen. 2007. Mercury and selenium concentrations in stranded bottlenose dolphins from the Indian River Lagoon system, Florida. Bulletin of Marine Science 81(1):37–54.

Fair, P.A., J. Adams, G. Mitchum, T.C. Hulsey, J.S. Reif, M. Houde, D. Muir, E. Wirth, D. Wetzel, E. Zolman, W. McFee, and G.D. Bossart. 2010. Contaminant blubber burdens in Atlantic bottlenose dolphins (*Tursiops truncatus*) from two southeastern US estuarine areas: Concentrations and patterns of PCBs, pesticides, PBDEs, PFCs, and PAHs. Science of the Total Environment 408(7):1577–1597. doi: 10.1016/j.scitotenv.2009.12.021

Fernandez, S. and A.A. Hohn. 1998. Age, growth, and calving season of bottlenose dolphins, *Tursiops truncatus*, off coastal Texas. Fishery Bulletin 96(2):357–365.

Gannon, D.P. and D.M. Waples. 2004. Diets of coastal bottlenose dolphins from the U. S. Mid-Atlantic coast differ by habitat. Marine Mammal Science 20(3):527–545. doi: 10.1111/j.1748-7692.2004.tb01177.x

Gubbins, C.M., M. Caldwell, S.G. Barco, K.A. Rittmaster, N. Bowles, and V.G. Thayer. 2003. Abundance and sighting patterns of bottlenose dolphins (*Tursiops truncatus*) at four northwest Atlantic coastal sites. Journal of Cetacean Research and Management 5(2):141–148.

Hansen, L.J., L.H. Schwacke, G.B. Mitchum, A.A. Hohn, R.S. Wells, E.S. Zolman, and P.A. Fair. 2004. Geographic variation in polychorinated biphenyl and organochlorine pesticide concentrations in the blubber of bottlenose dolphins from the US Atlantic coast. Science of the Total Environment 319(1-3):147–172. doi: 10.1016/S0048-9697(03)00371-1

Henderson, E.E. and B. Würsig. 2007. Behavior patterns of bottlenose dolphins in San Luis Pass, Texas. Gulf of Mexico Science 25(2):153–161. doi: 10.18785/goms.2502.06

Hohn, A.A., M.D. Scott, R.S. Wells, J.C. Sweeney, and A.B. Irvine. 1989. Growth layers in teeth from known-age, free-ranging bottlenose dolphins. Marine Mammal Science 5(4):315–342. doi: 10.1111/j.1748-7692.1989.tb00346.x

Kucklick, J., L. Schwacke, R. Wells, A. Hohn, A. Guichard, J. Yordy, L. Hansen, E. Zolman, R. Wilson, J. Litz, D. Nowacek, T. Rowles, R. Pugh, B. Balmer, C. Sinclair, and P. Rosel. 2011. Bottlenose dolphins as indicators of persistent organic pollutants in the western North Atlantic Ocean and Northern Gulf of Mexico. Environmental Science & Technology 45(10):4270–4277. doi: 10.1021/es1042244

LaBrecque, E., C. Curtice, J. Harrison, S.M. Van Parijs, and P.N. Halpin. 2015. 2. Biologically Important Areas for Cetaceans Within U.S. Waters – East Coast Region. Aquatic Mammals 41(1):17–29. doi: 10.1578/am.41.1.2015.17

Leatherwood, S. 1977. Some preliminary impressions on the numbers and social behavior of free-swimming bottlenosed dolphin calves (*Tursiops truncatus*) in the northern Gulf of Mexico. In: Ridgway, S.H. and K. Bernirschke (Eds.), Breeding Dolphins; Present Status, Suggestions for the Future. Final report to the US Marine Mammal Commission, Contract MM6AC009, MMC-76/07, Washington, DC. pp. 143–167.

Litz, J.A. 2007. Social structure, genetic structure, and persistent organohalogen pollutants in bottlenose dolphins (*Tursiops truncatus*) in Biscayne Bay, Florida. University of Miami. 140 p.

Litz, J.A., L.P. Garrison, L.A. Fieber, A. Martinez, J.P. Contillo, and J.R. Kucklick. 2007. Fine-scale spatial variation of persistent organic pollutants in bottlenose dolphins (*Tursiops truncatus*) in Biscayne Bay, Florida. Environmental Science & Technology 41(21):7222–7228. doi: 10.1021/es070440r

Mann, J., R.C. Connor, L.M. Barre, and M.R. Heithaus. 2000. Female reproductive success in bottlenose dolphins (*Tursiops* sp.): life history, habitat, provisioning, and group-size effects. Behavioral Ecology 11(2):210–219. doi: 10.1093/beheco/11.2.210

Mattson, M.C., K.D. Mullin, G.W. Ingram, and W. Hoggard. 2006. Age structure and growth of the bottlenose dolphin (*Tursiops truncatus*) from strandings in the Mississippi sound region of the north-central Gulf of Mexico from 1986 to 2003. Marine Mammal Science 22(3):654–666. doi: 10.1111/j.1748-7692.2006.00057.x

Mazzoil, M., S.D. McCulloch, and R.H. Defran. 2005. Observations on the site fidelity of bottlenose dolphins (*Tursiops truncatus*) in the Indian River Lagoon, Florida. Florida Scientist 68(4):217–226.

Mazzoil, M., J.S. Reif, M. Youngbluth, M.E. Murdoch, S.E. Bechdel, E. Howells, S.D. McCulloch, L.J. Hansen, and G.D. Bossart. 2008a. Home ranges of bottlenose dolphins (*Tursiops truncatus*) in the Indian River Lagoon, Florida: Environmental correlates and implications for management strategies. Ecohealth 5(3):278–288. doi: 10.1007/s10393-008-0194-9

Mazzoil, M.S., S.D. McCulloch, M.J. Youngbluth, D.S. Kilpatrick, M.E. Murdoch, B. Mase-Guthrie, D.K. Odell, and G.D. Bossart. 2008b. Radio-tracking and survivorship of two rehabilitated bottlenose dolphins (*Tursiops truncatus*) in the Indian River Lagoon, Florida. Aquatic Mammals 34:54–64. doi: 10.1578/AM.34.1.2008.54

McClellan, D.B., J. Browder, J.L. Tobias, G.J. Konoval, M.D. Heron, O.L. Bass, and J. Osborne. 2000. Opportunistic sightings of bottlenose dolphin, *Tursiops truncatus*, along the southeast Florida coast and Florida Bay, 1992-1997. National Oceanic and Atmospheric Administration, National Marine Fisheries Service, Southeast Fisheries Science Center: Miami, FL. NOAA Technical Memorandum NMFS-SEFSC-435. 18 p.

McFee, W.E., T.R. Speakman, L. Balthis, J.D. Adams, and E.S. Zolman. 2014. Reproductive seasonality of a recently designated bottlenose dolphin stock near Charleston, South Carolina, U.S.A. Marine Mammal Science 30(2):528–543. doi: 10.1111/mms.12055

McHugh, M.B. 1989. Population numbers and feeding behavior of the Atlantic bottlenose dolphin (*Tursiops truncatus*) near Aransas Pass, Texas. University of Texas at Austin. Thesis. 96 p.

Mead, J.G. and C.W. Potter. 1990. Natural history of bottlenose dolphins along the central Atlantic coast of the United States. In: Leatherwood, S. and R.R. Reeves (Eds.), The Bottlenose Dolphin. Academic Press, San Diego, CA. pp. 165–195.

Miller, L.J., A.D. Mackey, T. Hoffland, M. Solangi, and S.A. Kuczaj. 2010. Potential effects of a major hurricane on Atlantic bottlenose dolphin (*Tursiops truncatus*) reproduction in the Mississippi Sound. Marine Mammal Science 26(3):707–715. doi: 10.1111/j.1748-7692.2010.00371.x

Miller, L.J., A.D. Mackey, M. Solangi, and S.A. Kuczaj. 2013. Population abundance and habitat utilization of bottlenose dolphins in the Mississippi Sound. Aquatic Conservation: Marine and Freshwater Ecosystems 23(1):145–151. doi: 10.1002/aqc.2278

Odell, D.K. and E.D. Asper. 1990. Distribution and movements of freeze-branded bottlenose dolphins in the Indian and Banana Rivers, Florida. In: Leatherwood, S. and R. Reeves (Eds.), The Bottlenose Dolphin. Academic Press, San Diego, CA. pp. 515–540.

Page-Karjian, A., C.F. Lo, B. Ritchie, C.A. Harms, D.S. Rotstein, S. Han, S.M. Hassan, A.F. Lehner, J.P. Buchweitz, V.G. Thayer, J.M. Sullivan, E.F. Christiansen, and J.R. Perrault. 2020. Anthropogenic contaminants and histopathological findings in stranded cetaceans in the southeastern United States, 2012–2018. Frontiers in Marine Science 7:630. doi: 10.3389/fmars.2020.00630

Pate, S.M. and W.E. McFee. 2012. Prey species of bottlenose dolphins (*Tursiops truncatus*) from South Carolina Waters. Southeastern Naturalist 11(1):1–22. doi: 10.1656/058.011.0101

Perrin, W.F. and S.B. Reilly. 1984. Reproductive parameters of dolphins and small whales of the family Delphinidae. In: Perrin, W.F., R.L. Brownell, Jr., and D.P. DeMaster (Eds.), Reproduction in Whales, Dolphins, and Porpoises. pp. 97–134.

Pulster, E.L., K.L. Smalling, E. Zolman, L. Schwacke, and K.A. Maruya. 2009. Persistent organochlorine pollutants and toxaphene congener profiles in bottlenose dolphins (*Tursiops truncatus*) frequenting the Turtle/Brunswick River Estuary, Georgia, USA. Environmental Toxicology and Chemistry 28(7):1390–1399. doi: 10.1897/08-240.1

Reif, J.S., A.M. Schaefer, G.D. Bossart, and P.A. Fair. 2017. Health and Environmental Risk Assessment Project for bottlenose dolphins *Tursiops truncatus* from the southeastern USA. II. Environmental aspects. Diseases of Aquatic Organisms 125(2):155–166. doi: 10.3354/dao03143

Rossman, S., P.H. Ostrom, M. Stolen, N.B. Barros, H. Gandhi, C.A. Stricker, and R.S. Wells. 2015. Individual specialization in the foraging habits of female bottlenose dolphins living in a trophically diverse and habitat rich estuary. Oecologia 178(2):415–425. doi: 10.1007/s00442-015-3241-6

Schwacke, L.H., E.O. Voit, L.J. Hansen, R.S. Wells, G.B. Mitchum, A.A. Hohn, and P.A. Fair. 2002. Probabilistic risk assessment of reproductive effects of polychlorinated biphenyls on bottlenose dolphins (*Tursiops truncatus*) from the southeast United States coast. Environmental Toxicology and Chemistry 21(12):2752–2764. doi: 10.1002/etc.5620211232

Schwacke, L.H., E.S. Zolman, B.C. Balmer, S. De Guise, R.C. George, J. Hoguet, A.A. Hohn, J.R. Kucklick, S. Lamb, M. Levin, J.A. Litz, W.E. McFee, N.J. Place, F.I. Townsend, R.S. Wells, and T.K. Rowles. 2012. Anaemia, hypothyroidism and immune suppression associated with polychlorinated biphenyl exposure in bottlenose dolphins (*Tursiops truncatus*). Proceedings of the Royal Society B: Biological Sciences 279(1726):48–57. doi: 10.1098/rspb.2011.0665

Seguel, M., R.C. George, G. Maboni, S. Sanchez, A. Page-Karjian, E. Wirth, W. McFee, and N.L. Gottdenker. 2020. Pathologic findings and causes of death in bottlenose dolphins *Tursiops truncatus* stranded along the Georgia coast, USA (2007-2013). Diseases of Aquatic Organisms 141:25–38. doi: 10.3354/dao03509

Sergeant, D.E., D.K. Caldwell, and M.C. Caldwell. 1973. Age, growth, and maturity of bottlenosed dolphin (*Tursiops-truncatus*) from northeast Florida. Journal of the Fisheries Research Board of Canada 30(7):1009–1011. doi: 10.1139/f73-165

Shane, S.H., R.S. Wells, and B. Würsig. 1986. Ecology, behavior and social organization of the bottlenose dolphin: A review. Marine Mammal Science 2:34–63. doi: 10.1111/j.1748-7692.1986.tb00026.x

Silva, D., B. Tramutolo, E. DeSalvio, T. Speakman, and R. Young. 2019. Abundance and movements of the Northern South Carolina Estuarine System Stock of bottlenose dolphins (*Tursiops truncatus*) (USA). Journal of Marine Animals and Their Ecology 11(1):8–18.

Sloan, P.E. 2006. Residency patterns, seasonality and habitat use among bottlenose dolphins, *Tursiops truncatus*, in the Cape Romain National Wildlife Refuge, S.C. University of North Carolina Wilmington. Thesis. 75 p.

Stavros, H.-C.W., G.D. Bossart, T.C. Hulsey, and P.A. Fair. 2007. Trace element concentrations in skin of free-ranging bottlenose dolphins (*Tursiops truncatus*) from the southeast Atlantic coast. Science of the Total Environment 388(1-3):300–315. doi: 10.1016/j.scitotenv.2007.07.030

Stavros, H.-C.W., G.D. Bossart, T.C. Hulsey, and P.A. Fair. 2008. Trace element concentrations in blood of free-ranging bottlenose dolphins (*Tursiops truncatus*): Influence of age, sex and location. Marine Pollution Bulletin 56(2):371–379. doi: 10.1016/j.marpolbul.2007.10.030

Stavros, H.-C.W., M. Stolen, W.N. Durden, W. McFee, G.D. Bossart, and P.A. Fair. 2011. Correlation and toxicological inference of trace elements in tissues from stranded and free-ranging bottlenose dolphins (*Tursiops truncatus*). Chemosphere 82(11):1649–1661. doi: 10.1016/j.chemosphere.2010.11.019

Taylor, B.L., S.J. Chivers, J. Larese, and W.F. Perrin. 2007. Generation length and percent mature estimates for IUCN assessments of cetaceans. National Oceanic and Atmospheric Administration, National Marine Fisheries Service, Southwest Fisheries Science Center: La Jolla, CA. Administrative Report LJ-07-01. 24 p.

Thayer, V.G. 2008. Life history parameters and social associations of female bottlenose dolphins (*Tursiops truncatus*) off North Carolina, USA. Duke University. Dissertation. 164 p.

Thayer, V.G., A.J. Read, A.S. Friedlaender, D.R. Colby, A.A. Hohn, W.A. McLellan, D.A. Pabst, J.L. Dearolf, N.I. Bowles, J.R. Russell, and K.A. Rittmaster. 2003. Reproductive seasonality of western Atlantic bottlenose dolphins off North Carolina, USA. Marine Mammal Science 19(4):617–629. doi: 10.1111/j.1748-7692.2003.tb01120.x

Titcomb, E.M., J.S. Reif, P.A. Fair, H.-C.W. Stavros, M. Mazzoil, G.D. Bossart, and A.M. Schaefer. 2017. Blood mercury concentrations in common bottlenose dolphins from the Indian River Lagoon, Florida: Patterns of social distribution. Marine Mammal Science 33(3):771–784. doi: 10.1111/mms.12390

Torres, L.G. 2007. Top predator distribution and foraging ecology in Florida Bay, Florida. Duke University. Dissertation. 225 p.

Torres, L.G. and A.J. Read. 2009. Where to catch a fish? The influence of foraging tactics on the ecology of bottlenose dolphins (*Tursiops truncatus*) in Florida Bay, Florida. Marine Mammal Science 25(4):797–815. doi: 10.1111/j.1748-7692.2009.00297.x

Urian, K.W., D.M. Waples, R.B. Tyson, L.E. Hodge, and A.J. Read. 2013. Abundance of bottlenose dolphins (*Tursiops truncatus*) in estuarine and near-shore waters of North Carolina, USA. Journal of the North Carolina Academy of Science 129(4):165–171. doi: 10.7572/2167-5880-129.4.165

Vollmer, N.L. and P.E. Rosel. 2013. A review of common bottlenose dolphins (*Tursiops truncatus truncatus*) in the Northern Gulf of Mexico: Population biology, potential threats, and management. Southeastern Naturalist 12(m6):1–43. doi: 10.1656/058.012.m601

Waring, G., E. Josephson, K. Maze-Foley, and P. Rosel. 2014. U.S. Atlantic and Gulf of Mexico Marine Mammal Stock Assessments - 2013. National Oceanic and Atmospheric Administration, National

Marine Fisheries Service, Northeast Fisheries Science Center: Woods Hole, MA. NOAA Technical Memorandum NMFS-NE-228. 464 p. doi: 10.7289/V51G0J70
Waring, G.T., E. Josephson, K. Maze-Foley, and P.E. Rosel. 2016. U.S. Atlantic and Gulf of Mexico marine mammal stock assessments - 2015. National Oceanic and Atmospheric Administration, National Marine Fisheries Service, Northeast Fisheries Science Center: Woods Hole, MA. NOAA Technical Memorandum NMFS-NE-238. 501 p. doi: 10.7289/V57S7KTN
Wells, R.S. 2000. Reproduction in wild bottlenose dolphins: Overview of patterns observed during a long-term study. In: Duffield, D. and T. Robeck (Eds.), Bottlenose Dolphin Reproduction Workshop. AZA Marine Mammal Taxon Advisory Group, Silver Spring, MD. pp. 57–74.
Wells, R.S. 2003. Dolphin social complexity: Lessons from long-term study and life history. In: de Waal, F.B.M. and P.L. Tyack (Eds.), Animal Social Complexity: Intelligence, Culture, and Individualized Societies Harvard University Press, Cambridge, MA. pp. 32–56.
Wells, R.S. 2014. Social structure and life history of bottlenose dolphins near Sarasota Bay, Florida: Insights from four decades and five generations. Primates and cetaceans. pp. 149–172. doi: 10.1007/978-4-431-54523-1_8
Wells, R.S., A. Natoli, and G. Braulik. 2019. *Tursiops truncatus* (errata version published in 2019). *The IUCN Red List of Threatened Species* 2019: e.T22563A156932432. https://dx.doi.org/10.2305/IUCN.UK.2019-1.RLTS.T22563A156932432.en
Wells, R.S. and M.D. Scott. 1999. Bottlenose dolphin *Tursiops truncatus* (Montagu, 1821). In: Ridgway, S.H. and R. Harrison (Eds.), Handbook of marine mammals. Volume 6: The second book of dolphins and the porpoises. Academic Press, San Diego, CA. pp. 137–182.
Wells, R.S. and M.D. Scott. 2018. Bottlenose dolphin, *Tursiops truncatus*, common bottlenose dolphin. In: Würsig, B., J.G.M. Thewissen, and K.M. Kovacs (Eds.), Encyclopedia of Marine Mammals (Third Edition). Academic Press, London, UK. pp. 118–125. doi: 10.1016/B978-0-12-804327-1.00072-8
Wells, R.S., M.D. Scott, and A.B. Irvine. 1987. The social structure of free-ranging bottlenose dolphins. In: Genoways, H.H. (Ed.), Current Mammalogy. Plenum Press, New York, NY. pp. 247–305.
Wilson, R.M., R.B. Tyson, J.A. Nelson, B.C. Balmer, J.P. Chanton, and D.P. Nowacek. 2017. Niche differentiation and prey selectivity among common bottlenose dolphins (*Tursiops truncatus*) sighted in St. George Sound, Gulf of Mexico. Frontiers in Marine Science 4:235. doi: 10.3389/fmars.2017.00235
Wirth, E.F., P.L. Pennington, C. Cooksey, L. Schwacke, L. Balthis, J. Hyland, and M.H. Fulton. 2014. Distribution and sources of PCBs (Aroclor 1268) in the Sapelo Island National Estuarine Research Reserve. Environmental Monitoring and Assessment 186(12):8717–8726. doi: 10.1007/s10661-014-4039-4
Yordy, J.E., M.A. Mollenhauer, R.M. Wilson, R.S. Wells, A. Hohn, J. Sweeney, L.H. Schwacke, T.K. Rowles, J.R. Kucklick, and M.M. Peden-Adams. 2010. Complex contaminant exposure in cetaceans: a comparative E-Screen analysis of bottlenose dolphin blubber and mixtures of four persistent organic pollutants. Environmental Toxicology and Chemistry 29(10):2143–2153. doi: 10.1002/etc.279

Common bottlenose dolphin − *Tursiops truncatus*
Charleston Estuarine System Stock

Overall Vulnerability Rank = Very High

Biological Sensitivity = Very High

Climate Exposure = Very High

Data Quality = 96% of scores ≥ 2

	Tursiops truncatus	Expert Scores	Data Quality	Expert Scores Plots (Portion by Category)
Sensitivity attributes	Prey/Diet Specificity	2.3	3.0	
	Habitat Specificity	3.7	2.3	
	Site Fidelity	4.0	3.0	
	Lifetime Reproductive Potential	1.0	2.3	
	Generation Length	2.7	2.3	
	Reproductive Plasticity	1.2	2.3	
	Migration	3.9	3.0	
	Home Range	3.1	3.0	
	Stock Abundance	3.0	3.0	
	Stock Abundance Trend	2.3	2.3	
	Cumulative Stressors	3.1	2.7	
	**Sensitivity Score**	Very High		
Exposure variables	Sea Surface Temperature (Change in mean)	4.0	3.0	
	Sea Surface Temperature (Change in variability)	1.3	3.0	
	Air Temperature (Change in mean)	4.0	3.0	
	Air Temperature (Change in variability)	1.0	3.0	
	Precipitation (Change in mean)	1.1	3.0	
	Precipitation (Change in variability)	2.0	3.0	
	Sea Surface Salinity (Change in mean)	3.1	3.0	
	Sea Surface Salinity (Change in variability)	1.0	3.0	
	Ocean pH (Change in mean)	4.0	3.0	
	Ocean pH (Change in variability)	1.0	3.0	
	Sea ice coverage (Change in mean)	1.0	3.0	
	Sea ice coverage (Change in variability)	1.0	3.0	
	Dissolved oxygen (Change in mean)	4.0	3.0	
	Dissolved oxygen (Change in variability)	1.0	3.0	
	Circulation	4.0	1.7	
	Sea level rise	4.0	3.0	
	**Exposure Score**	Very High		
**Overall Vulnerability Rank**		Very High		

Low | Moderate | High | Very High

Common bottlenose dolphin (Charleston Estuarine System Stock)

Tursiops truncatus

CVA Results Summary

Overall Climate Vulnerability Rank: Very High (100% certainty from bootstrap analysis).

Climate Exposure: Very High. Six exposure factors scored greater than 3.5: Air Temperature (Standard anomaly) (4.00), Circulation (4.00), Dissolved oxygen (Standard anomaly) (4.00), Ocean pH (Standard anomaly) (4.00), Sea level rise (4.00), and Sea Surface Temperature (Standard anomaly) (4.00).

Biological Sensitivity: Very High. Three sensitivity attributes scored greater than 3.5: Site Fidelity (4.00), Migration (3.93), and Habitat Specificity (3.67).

Distributional Response: High

Abundance Response: High

Phenology Response: High

Data Quality: 96% of the data quality scores were 2 or greater, 100% of sensitivity attribute data quality scores were 2 or higher.

Background Information

Stock Description

The Charleston Estuarine System (CES) Stock includes common bottlenose dolphins in the estuarine and nearshore (≤ 1km from shore) coastal waters of the western North Atlantic along the South Carolina coast from Price Inlet in the north to the North Edisto River in the south. It includes waters in the Intracoastal Waterway (ICW), Charleston Harbor, the main channels and creeks of the Ashley, Cooper, Wando, Stono and North Edisto Rivers (Waring et al. 2016).

Prey/Diet Specificity

Generally, common bottlenose dolphins consume a large variety of fish and/or squid (Barros and Odell 1990; Mead and Potter 1990; Wells and Scott 2018). Common bottlenose dolphins seem to show a consistent preference for sound-producing fishes (e.g., sciaenids, toadfish; Berens McCabe et al. 2010; Bowen-Stevens et al. 2021). Estuarine stocks along the southeast U.S. Atlantic coast have been reported to consume a variety of prey, with preference for sciaenids (Gannon and Waples 2004; Pate and McFee 2012). Pate and McFee (2012) identified 42 prey species from 24 families in a study of stranded dolphins along the South Carolina coast. In Sarasota Bay, Florida, Rossman et al. (2015) found some individual common bottlenose dolphins to show diet specialization despite the broader study group showing a generalist diet. Specialization can be influenced by locally abundant prey species (Shane et al. 1986; Vollmer and Rosel 2013) or can result from resource partitioning amongst sympatric populations (e.g., Torres and Read 2009; Wilson et al. 2017). Stable isotope data suggests differences in common

bottlenose dolphin forage composition among estuaries along the South Carolina coast and between estuaries and the nearshore coastal populations (Olin et al. 2012)

Habitat Specificity

Common bottlenose dolphins are found in a variety of marine and estuarine habitats (Wells and Scott 2018). The area inhabited by the CES stock is characterized by estuarine tidal marsh systems, river channels, tidal creeks, and open water habitat (Waring et al. 2016).

Site Fidelity

Common bottlenose dolphins in the CES Stock show evidence of site fidelity (Speakman et al. 2006). Other estuarine stocks in the western North Atlantic have shown site fidelity (Waring et al. 2016), such as in South Carolina (Sloan 2006; Waring et al. 2016), central and southern Georgia (Balmer et al. 2011; Kucklick et al. 2011; Balmer et al. 2013), northern Florida (Caldwell 2001), central Florida (Odell and Asper 1990; Mazzoil et al. 2005, 2008a, 2008b), and southern Florida (Litz 2007; Torres 2007; McClellan et al. 2000).

Lifetime Reproductive Potential

Taylor et al. (2007) reported the interbirth interval of common bottlenose dolphins as 3.80 years, though other studies have shown it to be closer to three years (Leatherwood 1977; Wells and Scott 1999; Connor et al. 2000; Wells 2003). Thayer (2008) estimated mean interbirth interval of 2.9 years in North Carolina.

Generally, females reach sexual maturity before males, with females reaching sexual maturity at 5–13 years (Sergeant et al. 1973; Perrin and Reilly 1984; Wells et al. 1987; Mead and Potter 1990; Mann et al. 2000; Wells 2000; Wells and Scott 2018).

Females reproduce through most of their adult lives (Leatherwood 1977; Hohn et al. 1989; Wells and Scott 1999; Wells 2000; Wells 2003), but may produce fewer calves after they reach 30 years of age (Wells 2000). Sarasota Bay common bottlenose dolphins can give birth when they are as young as 6 years of age, and can continue until they are 48 years old (Wells and Scott 2018). Reproductive lifespan would therefore be up to 42 years based on long-term observation studies (Wells 2014; Wells and Scott 2018).

Longitudinal studies from Sarasota Bay, Florida have found that female common bottlenose dolphins can live up to 67 years (Wells and Scott 2018). Studies based on growth layer groups in teeth suggest female common bottlenose dolphins can live to 41 years along the Texas coast (Fernandez and Hohn 1998) and to 30 years along the Mississippi coast (Mattson et al. 2006).

Generation Length

Taylor et al. (2007) reported common bottlenose dolphin generation length of 20.6–21.1 years.

Reproductive Plasticity

Common bottlenose dolphin calving is generally diffusely seasonal, with one or more peaks, although births have been reported from all seasons (Vollmer and Rosel 2013; Wells and Scott 2018). Calf sightings in South Carolina peak in spring and early summer and again in early autumn (McFee et al. 2014). In North Carolina, Thayer et al. (2003) observed a strong birth peak in spring based on stranding data and a diffuse birth peak from late spring to early fall based on sightings.

Migration

The estuarine stocks in the western North Atlantic are thought to primarily comprise stable resident communities. However, transient or seasonal movements of some individuals into and out of stock areas are recorded in some areas (Waring et al. 2016; LaBrecque et al. 2015). Taylor et al. (2016) found seasonal abundance and temporary emigration patterns in the CES, with low abundance in winter, and peak abundance in summer.

Other estuarine stocks in the western North Atlantic are known to spend warm months in estuarine waters then move to nearshore waters during cool months (Gubbins et al. 2003; Durden et al. 2011; Waring et al. 2014; Waring et al. 2016), though some remain entirely in estuarine waters (Mazzoil et al. 2008a).

Home Range

The home ranges of individual common bottlenose dolphins within the CES generally include the areas within the stock boundaries and brief excursions beyond the stock boundaries (Gubbins 2002a, 2002b, 2002c; Zolman 2002; Gubbins et al. 2003; Speakman et al. 2006; Laska et al. 2011).

Stock Abundance

Speakman et al. (2010) estimated this stock's abundance at 289 individuals (CV=0.03) based on seasonal (January, April, July, October) surveys during 2004–2006. However, this estimate did not cover the entire range of the stock, and may represent an undercount (Waring et al. 2016).

Stock Abundance Trend

Data are insufficient to complete an abundance trend analysis for this stock (Waring et al. 2016).

Cumulative Stressors

Common bottlenose dolphins face a wide range of individual and cumulative natural and anthropogenic threats (see reviews by Vollmer and Rosel 2013; Wells and Scott 2018; and Wells et al. 2019). These include such threats as shark predation, harmful algal blooms, recreational and commercial fishing gear, vessel traffic, pollution, habitat alteration, and provisioning.

Common bottlenose dolphins in the CES stock interact with the blue crab trap/pot fishery, however levels of serious injury and mortality are unknown due to a lack of observer coverage (Waring et al. 2016). Individuals of this stock have been reported stranded, with some strandings showing evidence of

human interactions, and other strandings occurring during Unusual Mortality Events (UMEs; Waring et al. 2016; also see NOAA National Marine Mammal Health and Stranding Response Database).

A UME was declared in the summer of 2013 for the mid-Atlantic coast from New York to Brevard County, Florida, with more than 1500 common bottlenose dolphins stranding as a result of morbillivirus (Waring et al. 2016). Strandings were more prevalent along oceanic shores than in estuaries, suggesting that coastal stocks were more impacted by the UME than estuarine stocks (Waring et al. 2016). A UME for bottlenose dolphins was declared in winter and spring of 2011 in South Carolina, during which common bottlenose dolphins stranded at a rate nearly three times the historical average, with evidence suggesting an unusually cold winter and decreased prey abundance contributed to the UME (Waring et al. 2016).

The nearshore and estuarine habitats along the Atlantic seaboard are adjacent to areas of high human population and some are highly industrialized. Studies of bottlenose dolphin tissues from estuaries in the southeast U.S. have shown persistent organic pollutant (POP; Schwacke et al. 2002, 2012; Hansen et al. 2004; Litz et al. 2007; Pulster et al. 2009; Fair et al. 2010; Yordy et al. 2010; Balmer et al. 2011; Kucklick et al. 2011; ; Reif et al. 2017) and heavy metal (Durden et al. 2007; Stavros et al. 2007, 2008, 2011; Titcomb et al. 2017; Reif et al. 2017; Page-Karjian et al. 2020) concentrations that may result in adverse effects on health or reproductive rates, particularly in urbanized, developed, and agricultural areas. Common bottlenose dolphins in the CES have been found with elevated levels of POPs (Hansen et al. 2004), polybrominated diphenyl ethers (PBDE; Fair et al. 2007; Kucklick et al. 2011; Adams et al. 2014), perfluoroalkyl compounds (PFCs; Adams et al. 2008), and per- and polyfluoroalkyl substances (PFAS; Fair et al. 2012; Fair et al. 2013; De Silva et al. 2016; Lynch et al. 2019). In South Carolina, urban areas showed greater numbers of strandings than areas with primarily agricultural input (McFee and Burdett 2007). Adams et al. (2008) found dolphins in the CES that spent most of their time in urbanized and industrial areas had significantly higher plasma PFC concentrations than dolphins that spent most of their time in less developed areas. These findings are consistent with a study from Biscayne Bay, Florida, that showed elevated POP concentrations in individuals associated with more polluted areas compared to individuals associated with less polluted areas, suggesting that exposure to pollutants may be highly variable based on geography (Litz et al. 2007). Common bottlenose dolphins stranded along the South Carolina coast have been found with ingested microplastics (Battaglia et al. 2020). Individuals of this stock have been found with mercury in blood and skin samples, though at lower concentrations relative to individuals in the Indian River Lagoon in Florida (Schaefer et al. 2011).

Common bottlenose dolphins in the CES have tested negative for titers to both dolphin morbillivirus and porpoise morbillivirus, indicating that these dolphins have not been exposed to morbillivirus in recent years and may therefore be susceptible to future infection (Rowles et al. 2011; Bossart et al. 2010; Bossart et al. 2017).

Common bottlenose dolphins stranded in the CES and other regions of South Carolina show a high prevalence of the gram-negative facultative intracellular bacterium *Brucella ceti* found in the tissues of brain, lung, and other tissues (McFee et al. 2020). Nearly 32% of dolphins tested positive for *B. ceti* over the six year study period, coinciding with the calving period.

Further Reading

Waring, G.T., E. Josephson, K. Maze-Foley, and P.E. Rosel. 2016. U.S. Atlantic and Gulf of Mexico Marine Mammal Stock Assessments - 2015. National Oceanic and Atmospheric Administration, National Marine Fisheries Service, Northeast Fisheries Science Center: Woods Hole, MA. NOAA Technical Memorandum NMFS-NE-238. 501 p. doi: 10.7289/V57S7KTN

Wells, R.S. and M.D. Scott. 2018. Bottlenose Dolphin, *Tursiops truncatus*, Common Bottlenose Dolphin. In: Würsig, B., J.G.M. Thewissen, and K.M. Kovacs (Eds.), Encyclopedia of Marine Mammals (Third Edition). Academic Press, London, UK. pp. 118–125. doi: 10.1016/B978-0-12-804327-1.00072-8

Wells, R.S., A. Natoli, and G. Braulik. 2019. *Tursiops truncatus* (errata version published in 2019). The IUCN Red List of Threatened Species 2019: e.T22563A156932432. https://dx.doi.org/10.2305/IUCN.UK.2019-1.RLTS.T22563A156932432.en

References

Adams, J., M. Houde, D. Muir, T. Speakman, G. Bossart, and P. Fair. 2008. Land use and the spatial distribution of perfluoroalkyl compounds as measured in the plasma of bottlenose dolphins (*Tursiops truncatus*). Marine Environmental Research 66(4):430–437. doi: 10.1016/j.marenvres.2008.07.004

Adams, J., T. Speakman, E. Zolman, G. Mitchum, E. Wirth, G.D. Bossart, and P.A. Fair. 2014. The relationship between land use and emerging and legacy contaminants in an Apex predator, the bottlenose dolphin (*Tursiops truncatus*), from two adjacent estuarine watersheds. Environmental Research 135:346–353. doi: 10.1016/j.envres.2014.08.037

Balmer, B.C., L.H. Schwacke, R.S. Wells, J.D. Adams, R. Clay George, S.M. Lane, W.A. McLellan, P.E. Rosel, K. Sparks, T. Speakman, E.S. Zolman, and D. Ann Pabst. 2013. Comparison of abundance and habitat usage for common bottlenose dolphins between sites exposed to differential anthropogenic stressors within the estuaries of southern Georgia, U.S.A. Marine Mammal Science 29(2):E114–E135. doi: 10.1111/j.1748-7692.2012.00598.x

Balmer, B.C., L.H. Schwacke, R.S. Wells, R.C. George, J. Hoguet, J.R. Kucklick, S.M. Lane, A. Martinez, W.A. McLellan, P.E. Rosel, T.K. Rowles, K. Sparks, T. Speakman, E.S. Zolman, and D.A. Pabst. 2011. Relationship between persistent organic pollutants (POPs) and ranging patterns in common bottlenose dolphins (*Tursiops truncatus*) from coastal Georgia, USA. Science of the Total Environment 409(11):2094–2101. doi: 10.1016/j.scitotenv.2011.01.052

Barros, N.B. and D.K. Odell. 1990. Food habits of bottlenose dolphins in the southeastern United States In: Leatherwood, S. and R.R. Reeves (Eds.), The Bottlenose Dolphin. Academic Press, San Diego, CA. pp. 309–328. doi: 10.1016/B978-0-12-440280-5.50020-2

Battaglia, F.M., B.A. Beckingham, and W.E. McFee. 2020. First report from North America of microplastics in the gastrointestinal tract of stranded bottlenose dolphins (*Tursiops truncatus*). Marine Pollution Bulletin 160:111677. doi: 10.1016/j.marpolbul.2020.111677

Berens McCabe, E.J., D.P. Gannon, N.B. Barros, and R.S. Wells. 2010. Prey selection by resident common bottlenose dolphins (*Tursiops truncatus*) in Sarasota Bay, Florida. Marine Biology 157(5):931–942. doi: 10.1007/s00227-009-1371-2

Bossart, G.D., P. Fair, A.M. Schaefer, and J.S. Reif. 2017. Health and Environmental Risk Assessment Project for bottlenose dolphins *Tursiops truncatus* from the southeastern USA. I. Infectious diseases. Diseases of Aquatic Organisms 125(2):141–153. doi: 10.3354/dao03142

Bossart, G.D., J.S. Reif, A.M. Schaefer, J. Goldstein, P.A. Fair, and J.T. Saliki. 2010. Morbillivirus infection in free-ranging Atlantic bottlenose dolphins (*Tursiops truncatus*) from the Southeastern United

States: Seroepidemiologic and pathologic evidence of subclinical infection. Veterinary Microbiology 143(2-4):160–166. doi: 10.1016/j.vetmic.2009.11.024

Bowen-Stevens, S.R., D.P. Gannon, R.A. Hazelkorn, G. Lovewell, K.M. Volker, S. Smith, M.C. Tumlin, and J. Litz. 2021. Diet of common bottlenose dolphins, *Tursiops truncatus*, that stranded in and near Barataria Bay, Louisiana, 2010–2012. Southeastern Naturalist 20(1). doi: 10.1656/058.020.0113

Caldwell, M. 2001. Social and genetic structure of bottlenose dolphin (*Tursiops truncatus*) in Jacksonville, Florida. University of Miami. 143 p.

Connor, R.C., R.S. Wells, J. Mann, and A.J. Read. 2000. The bottlenose dolphin: Social relationships in a fission-fusion society. In: Mann, J., R. Connor, P. Tyack, and H. Whitehead (Eds.), Cetacean Societies: Field Studies of Dolphins and Whales. University of Chicago Press, Chicago, IL. pp. 91–126.

De Silva, A.O., C. Spencer, K.C. Ho, M. Al Tarhuni, C. Go, M. Houde, S.R. de Solla, R.A. Lavoie, L.E. King, D.C. Muir, P.A. Fair, R.S. Wells, and G.D. Bossart. 2016. Perfluoroalkylphosphinic acids in northern pike (*Esox lucius*), double-crested cormorants (*Phalacrocorax auritus*), and bottlenose dolphins (*Tursiops truncatus*) in relation to other perfluoroalkyl acids. Environmental Science & Technology 50(20):10903–10913. doi: 10.1021/acs.est.6b03515

Durden, W.N., E.D. Stolen, and M.K. Stolen. 2011. Abundance, distribution, and group composition of Indian River Lagoon bottlenose dolphins (*Tursiops truncatus*). Aquatic Mammals 37(2):175–186. doi: 10.1578/Am.37.2.2011.175

Durden, W.N., M.K. Stolen, D.H. Adams, and E.D. Stolen. 2007. Mercury and selenium concentrations in stranded bottlenose dolphins from the Indian River Lagoon system, Florida. Bulletin of Marine Science 81(1):37–54.

Fair, P.A., J. Adams, G. Mitchum, T.C. Hulsey, J.S. Reif, M. Houde, D. Muir, E. Wirth, D. Wetzel, E. Zolman, W. McFee, and G.D. Bossart. 2010. Contaminant blubber burdens in Atlantic bottlenose dolphins (*Tursiops truncatus*) from two southeastern US estuarine areas: Concentrations and patterns of PCBs, pesticides, PBDEs, PFCs, and PAHs. Science of the Total Environment 408(7):1577–1597. doi: 10.1016/j.scitotenv.2009.12.021

Fair, P.A., M. Houde, T.C. Hulsey, G.D. Bossart, J. Adams, L. Balthis, and D.C.G. Muir. 2012. Assessment of perfluorinated compounds (PFCs) in plasma of bottlenose dolphins from two southeast US estuarine areas: relationship with age, sex and geographic locations. Marine Pollution Bulletin 64(1):66–74. doi: 10.1016/j.marpolbul.2011.10.022

Fair, P.A., T. Romano, A.M. Schaefer, J.S. Reif, G.D. Bossart, M. Houde, D. Muir, J. Adams, C. Rice, T.C. Hulsey, and M. Peden-Adams. 2013. Associations between perfluoroalkyl compounds and immune and clinical chemistry parameters in highly exposed bottlenose dolphins (*Tursiops truncatus*). Environmental Toxicology and Chemistry 32(4):736–746. doi: 10.1002/etc.2122

Fairfield-Walsh, C. and L.P. Garrison. 2007. Estimated bycatch of marine mammals and turtles in the U.S. Atlantic pelagic longline fleet during 2006. National Oceanic and Atmospheric Administration, National Marine Fisheries Service, Southeast Fisheries Science Center: Miami, FL. NOAA Technical Memorandum NMFS-SEFSC-560. 54 p.

Fernandez, S. and A.A. Hohn. 1998. Age, growth, and calving season of bottlenose dolphins, *Tursiops truncatus*, off coastal Texas. Fishery Bulletin 96(2):357–365.

Gannon, D.P. and D.M. Waples. 2004. Diets of coastal bottlenose dolphins from the U. S. Mid-Atlantic coast differ by habitat. Marine Mammal Science 20(3):527–545. doi: 10.1111/j.1748-7692.2004.tb01177.x

Gubbins, C. 2002a. Use of home ranges by resident bottlenose dolphins (*Tursiops truncatus*) in a South Carolina estuary. Journal of Mammalogy 83(1):178–187. doi: 10.1644/1545-1542(2002)083<0178:Uohrbr>2.0.Co;2

Gubbins, C.M. 2002b. Association patterns of resident bottlenose dolphins (*Tursiops truncatus*) in a South Carolina estuary. Aquatic Mammals 28:24–31.

Gubbins, C.M. 2002c. The dolphins of Hilton Head: Their natural history. University of South Carolina Press, Columbia, SC. 96 p. doi: 10.2307/j.ctv1htpdsv

Gubbins, C.M., M. Caldwell, S.G. Barco, K.A. Rittmaster, N. Bowles, and V.G. Thayer. 2003. Abundance and sighting patterns of bottlenose dolphins (*Tursiops truncatus*) at four northwest Atlantic coastal sites. Journal of Cetacean Research and Management 5(2):141–148.

Hansen, L.J., L.H. Schwacke, G.B. Mitchum, A.A. Hohn, R.S. Wells, E.S. Zolman, and P.A. Fair. 2004. Geographic variation in polychorinated biphenyl and organochlorine pesticide concentrations in the blubber of bottlenose dolphins from the US Atlantic coast. Science of the Total Environment 319(1-3):147–172. doi: 10.1016/S0048-9697(03)00371-1

Hohn, A.A., M.D. Scott, R.S. Wells, J.C. Sweeney, and A.B. Irvine. 1989. Growth layers in teeth from known-age, free-ranging bottlenose dolphins. Marine Mammal Science 5(4):315–342. doi: 10.1111/j.1748-7692.1989.tb00346.x

Kucklick, J., L. Schwacke, R. Wells, A. Hohn, A. Guichard, J. Yordy, L. Hansen, E. Zolman, R. Wilson, J. Litz, D. Nowacek, T. Rowles, R. Pugh, B. Balmer, C. Sinclair, and P. Rosel. 2011. Bottlenose dolphins as indicators of persistent organic pollutants in the western North Atlantic Ocean and Northern Gulf of Mexico. Environmental Science & Technology 45(10):4270–4277. doi: 10.1021/es1042244

LaBrecque, E., C. Curtice, J. Harrison, S.M. Van Parijs, and P.N. Halpin. 2015. 2. Biologically Important Areas for Cetaceans Within U.S. Waters – East Coast Region. Aquatic Mammals 41(1):17–29. doi: 10.1578/am.41.1.2015.17

Laska, D., T. Speakman, and P.A. Fair. 2011. Community overlap of bottlenose dolphins (*Tursiops truncatus*) found in coastal waters near Charleston, South Carolina. Journal of Marine Animals and Their Ecology 4(2):10–18.

Leatherwood, S. 1977. Some preliminary impressions on the numbers and social behavior of free-swimming bottlenosed dolphin calves (*Tursiops truncatus*) in the northern Gulf of Mexico. In: Ridgway, S.H. and K. Bernirschke (Eds.), Breeding Dolphins; Present Status, Suggestions for the Future. Final report to the US Marine Mammal Commission, Contract MM6AC009, MMC-76/07, Washington, DC. pp. 143–167.

Litz, J.A. 2007. Social structure, genetic structure, and persistent organohalogen pollutants in bottlenose dolphins (*Tursiops truncatus*) in Biscayne Bay, Florida. University of Miami. 140 p.

Litz, J.A., L.P. Garrison, L.A. Fieber, A. Martinez, J.P. Contillo, and J.R. Kucklick. 2007. Fine-scale spatial variation of persistent organic pollutants in bottlenose dolphins (*Tursiops truncatus*) in Biscayne Bay, Florida. Environmental Science & Technology 41(21):7222–7228. doi: 10.1021/es070440r

Lynch, K.M., P.A. Fair, M. Houde, D.C.G. Muir, K. Kannan, G.D. Bossart, S.M. Bartell, and M.O. Gribble. 2019. Temporal trends in per- and polyfluoroalkyl substances in bottlenose dolphins (*Tursiops truncatus*) of Indian River Lagoon, Florida and Charleston, South Carolina. Environmental Science & Technology 53(24):14194–14203. doi: 10.1021/acs.est.9b04585

Mann, J., R.C. Connor, L.M. Barre, and M.R. Heithaus. 2000. Female reproductive success in bottlenose dolphins (*Tursiops* sp.): life history, habitat, provisioning, and group-size effects. Behavioral Ecology 11(2):210–219. doi: 10.1093/beheco/11.2.210

Mattson, M.C., K.D. Mullin, G.W. Ingram, and W. Hoggard. 2006. Age structure and growth of the bottlenose dolphin (*Tursiops truncatus*) from strandings in the Mississippi sound region of the north-central Gulf of Mexico from 1986 to 2003. Marine Mammal Science 22(3):654–666. doi: 10.1111/j.1748-7692.2006.00057.x

Mazzoil, M., S.D. McCulloch, and R.H. Defran. 2005. Observations on the site fidelity of bottlenose dolphins (*Tursiops truncatus*) in the Indian River Lagoon, Florida. Florida Scientist 68(4):217–226.

Mazzoil, M., J.S. Reif, M. Youngbluth, M.E. Murdoch, S.E. Bechdel, E. Howells, S.D. McCulloch, L.J. Hansen, and G.D. Bossart. 2008a. Home ranges of bottlenose dolphins (*Tursiops truncatus*) in the Indian River Lagoon, Florida: Environmental correlates and implications for management strategies. Ecohealth 5(3):278–288. doi: 10.1007/s10393-008-0194-9

Mazzoil, M.S., S.D. McCulloch, M.J. Youngbluth, D.S. Kilpatrick, M.E. Murdoch, B. Mase-Guthrie, D.K. Odell, and G.D. Bossart. 2008b. Radio-tracking and survivorship of two rehabilitated bottlenose dolphins (*Tursiops truncatus*) in the Indian River Lagoon, Florida. Aquatic Mammals 34:54–64. doi: 10.1578/AM.34.1.2008.54

McClellan, D.B., J. Browder, J.L. Tobias, G.J. Konoval, M.D. Heron, O.L. Bass, and J. Osborne. 2000. Opportunistic sightings of bottlenose dolphin, *Tursiops truncatus*, along the southeast Florida coast and Florida Bay, 1992-1997. National Oceanic and Atmospheric Administration, National Marine Fisheries Service, Southeast Fisheries Science Center: Miami, FL. NOAA Technical Memorandum NMFS-SEFSC-435. 18 p.

McFee, W.E. and L.G. Burdett. 2007. Spatial and temporal analysis of bottlenose dolphin strandings in South Carolina, 1992-2005. National Oceanic and Atmospheric Administration, National Ocean Service, National Centers for Coastal Ocean Science: Charleston, SC. NOAA Technical Memorandum NOS-NCCOS-46. 12 p.

McFee, W.E., T.R. Speakman, L. Balthis, J.D. Adams, and E.S. Zolman. 2014. Reproductive seasonality of a recently designated bottlenose dolphin stock near Charleston, South Carolina, U.S.A. Marine Mammal Science 30(2):528–543. doi: 10.1111/mms.12055

McFee, W.E., D. Wu, K. Colegrove, K. Terio, L. Balthis, and R. Young. 2020. Occurrence of *Brucella ceti* in stranded bottlenose dolphins *Tursiops truncatus* coincides with calving season. Diseases of Aquatic Organisms 141:185–193. doi: 10.3354/dao03526

Mead, J.G. and C.W. Potter. 1990. Natural history of bottlenose dolphins along the central Atlantic coast of the United States. In: Leatherwood, S. and R.R. Reeves (Eds.), The Bottlenose Dolphin. Academic Press, San Diego, CA. pp. 165–195.

Odell, D.K. and E.D. Asper. 1990. Distribution and movements of freeze-branded bottlenose dolphins in the Indian and Banana Rivers, Florida. In: Leatherwood, S. and R. Reeves (Eds.), The Bottlenose Dolphin. Academic Press, San Diego, CA. pp. 515–540.

Olin, J.A., P.A. Fair, M.A. Recks, E. Zolman, J. Adams, and A.T. Fisk. 2012. Unique seasonal forage bases within a local population of bottlenose dolphin (*Tursiops truncatus*). Marine Mammal Science 28(1):E28–E40. doi: 10.1111/j.1748-7692.2011.00470.x

Page-Karjian, A., C.F. Lo, B. Ritchie, C.A. Harms, D.S. Rotstein, S. Han, S.M. Hassan, A.F. Lehner, J.P. Buchweitz, V.G. Thayer, J.M. Sullivan, E.F. Christiansen, and J.R. Perrault. 2020. Anthropogenic contaminants and histopathological findings in stranded cetaceans in the southeastern United States, 2012–2018. Frontiers in Marine Science 7:630. doi: 10.3389/fmars.2020.00630

Pate, S.M. and W.E. McFee. 2012. Prey species of bottlenose dolphins (*Tursiops truncatus*) from South Carolina Waters. Southeastern Naturalist 11(1):1–22. doi: 10.1656/058.011.0101

Perrin, W.F. and S.B. Reilly. 1984. Reproductive parameters of dolphins and small whales of the family Delphinidae. In: Perrin, W.F., R.L. Brownell, Jr., and D.P. DeMaster (Eds.), Reproduction in Whales, Dolphins, and Porpoises. pp. 97–134.

Pulster, E.L., K.L. Smalling, E. Zolman, L. Schwacke, and K.A. Maruya. 2009. Persistent organochlorine pollutants and toxaphene congener profiles in bottlenose dolphins (*Tursiops truncatus*) frequenting the Turtle/Brunswick River Estuary, Georgia, USA. Environmental Toxicology and Chemistry 28(7):1390–1399. doi: 10.1897/08-240.1

Reif, J.S., A.M. Schaefer, G.D. Bossart, and P.A. Fair. 2017. Health and Environmental Risk Assessment Project for bottlenose dolphins *Tursiops truncatus* from the southeastern USA. II. Environmental aspects. Diseases of Aquatic Organisms 125(2):155–166. doi: 10.3354/dao03143

Rossman, S., P.H. Ostrom, M. Stolen, N.B. Barros, H. Gandhi, C.A. Stricker, and R.S. Wells. 2015. Individual specialization in the foraging habits of female bottlenose dolphins living in a trophically diverse and habitat rich estuary. Oecologia 178(2):415–425. doi: 10.1007/s00442-015-3241-6

Rowles, T.K., L.S. Schwacke, R.S. Wells, J.T. Saliki, L. Hansen, A. Hohn, F. Townsend, R.A. Sayre, and A.J. Hall. 2011. Evidence of susceptibility to morbillivirus infection in cetaceans from the United States. Marine Mammal Science 27(1):1–19. doi: 10.1111/j.1748-7692.2010.00393.x

Schaefer, A.M., H.C. Stavros, G.D. Bossart, P.A. Fair, J.D. Goldstein, and J.S. Reif. 2011. Associations between mercury and hepatic, renal, endocrine, and hematological parameters in Atlantic bottlenose dolphins (*Tursiops truncatus*) along the eastern coast of Florida and South Carolina. Arch Environ Contam Toxicol 61(4):688–695. doi: 10.1007/s00244-011-9651-5

Schwacke, L.H., E.O. Voit, L.J. Hansen, R.S. Wells, G.B. Mitchum, A.A. Hohn, and P.A. Fair. 2002. Probabilistic risk assessment of reproductive effects of polychlorinated biphenyls on bottlenose dolphins (*Tursiops truncatus*) from the southeast United States coast. Environmental Toxicology and Chemistry 21(12):2752–2764. doi: 10.1002/etc.5620211232

Schwacke, L.H., E.S. Zolman, B.C. Balmer, S. De Guise, R.C. George, J. Hoguet, A.A. Hohn, J.R. Kucklick, S. Lamb, M. Levin, J.A. Litz, W.E. McFee, N.J. Place, F.I. Townsend, R.S. Wells, and T.K. Rowles. 2012. Anaemia, hypothyroidism and immune suppression associated with polychlorinated biphenyl exposure in bottlenose dolphins (*Tursiops truncatus*). Proceedings of the Royal Society B: Biological Sciences 279(1726):48–57. doi: 10.1098/rspb.2011.0665

Sergeant, D.E., D.K. Caldwell, and M.C. Caldwell. 1973. Age, growth, and maturity of bottlenosed dolphin (*Tursiops-truncatus*) from northeast Florida. Journal of the Fisheries Research Board of Canada 30(7):1009–1011. doi: 10.1139/f73-165

Shane, S.H., R.S. Wells, and B. Würsig. 1986. Ecology, behavior and social organization of the bottlenose dolphin: A review. Marine Mammal Science 2:34–63. doi: 10.1111/j.1748-7692.1986.tb00026.x

Sloan, P.E. 2006. Residency patterns, seasonality and habitat use among bottlenose dolphins, *Tursiops truncatus*, in the Cape Romain National Wildlife Refuge, S.C. University of North Carolina Wilmington. Thesis. 75 p.

Speakman, T., E. Zolman, J. Adams, R.H. Defran, D. Laska, L. Schwacke, J. Craigie, and P. Fair. 2006. Temporal and spatial aspects of bottlenose dolphin occurrence in coastal and estuarine waters near Charleston, South Carolina. National Oceanic and Atmospheric Administration, National Ocean Service, National Centers for Coastal Ocean Science: Charleston, SC. NOAA Technical Memorandum NOS-NCCOS-37. 50 p.

Speakman, T.R., S.M. Lane, L.H. Schwacke, P.A. Fair, and E.S. Zolman. 2010. Mark-recapture estimates of seasonal abundance and survivorship for bottlenose dolphins (*Tursiops truncatus*) near Charleston, South Carolina, USA. Journal of Cetacean Research and Management 11(2):153–162.

Stavros, H.-C.W., G.D. Bossart, T.C. Hulsey, and P.A. Fair. 2007. Trace element concentrations in skin of free-ranging bottlenose dolphins (*Tursiops truncatus*) from the southeast Atlantic coast. Science of the Total Environment 388(1-3):300–315. doi: 10.1016/j.scitotenv.2007.07.030

Stavros, H.-C.W., G.D. Bossart, T.C. Hulsey, and P.A. Fair. 2008. Trace element concentrations in blood of free-ranging bottlenose dolphins (*Tursiops truncatus*): Influence of age, sex and location. Marine Pollution Bulletin 56(2):371–379. doi: 10.1016/j.marpolbul.2007.10.030

Stavros, H.-C.W., M. Stolen, W.N. Durden, W. McFee, G.D. Bossart, and P.A. Fair. 2011. Correlation and toxicological inference of trace elements in tissues from stranded and free-ranging bottlenose dolphins (*Tursiops truncatus*). Chemosphere 82(11):1649–1661. doi: 10.1016/j.chemosphere.2010.11.019

Taylor, A.R., J.H. Schacke, T.R. Speakman, S.B. Castleberry, and R.B. Chandler. 2016. Factors related to common bottlenose dolphin (*Tursiops truncatus*) seasonal migration along South Carolina and Georgia coasts, USA. Animal Migration 3(1):14–26. doi: 10.1515/ami-2016-0002

Taylor, B.L., S.J. Chivers, J. Larese, and W.F. Perrin. 2007. Generation length and percent mature estimates for IUCN assessments of cetaceans. National Oceanic and Atmospheric Administration, National Marine Fisheries Service, Southwest Fisheries Science Center: La Jolla, CA. Administrative Report LJ-07-01. 24 p.

Thayer, V.G. 2008. Life history parameters and social associations of female bottlenose dolphins (*Tursiops truncatus*) off North Carolina, USA. Duke University. Dissertation. 164 p.

Thayer, V.G., A.J. Read, A.S. Friedlaender, D.R. Colby, A.A. Hohn, W.A. McLellan, D.A. Pabst, J.L. Dearolf, N.I. Bowles, J.R. Russell, and K.A. Rittmaster. 2003. Reproductive seasonality of western Atlantic bottlenose dolphins off North Carolina, USA. Marine Mammal Science 19(4):617–629. doi: 10.1111/j.1748-7692.2003.tb01120.x

Titcomb, E.M., J.S. Reif, P.A. Fair, H.-C.W. Stavros, M. Mazzoil, G.D. Bossart, and A.M. Schaefer. 2017. Blood mercury concentrations in common bottlenose dolphins from the Indian River Lagoon, Florida: Patterns of social distribution. Marine Mammal Science 33(3):771–784. doi: 10.1111/mms.12390

Torres, L.G. 2007. Top predator distribution and foraging ecology in Florida Bay, Florida. Duke University. Dissertation. 225 p.

Torres, L.G. and A.J. Read. 2009. Where to catch a fish? The influence of foraging tactics on the ecology of bottlenose dolphins (*Tursiops truncatus*) in Florida Bay, Florida. Marine Mammal Science 25(4):797–815. doi: 10.1111/j.1748-7692.2009.00297.x

Vollmer, N.L. and P.E. Rosel. 2013. A review of common bottlenose dolphins (*Tursiops truncatus truncatus*) in the Northern Gulf of Mexico: Population biology, potential threats, and management. Southeastern Naturalist 12(m6):1–43. doi: 10.1656/058.012.m601

Waring, G., E. Josephson, K. Maze-Foley, and P. Rosel. 2014. U.S. Atlantic and Gulf of Mexico Marine Mammal Stock Assessments - 2013. National Oceanic and Atmospheric Administration, National Marine Fisheries Service, Northeast Fisheries Science Center: Woods Hole, MA. NOAA Technical Memorandum NMFS-NE-228. 464 p. doi: 10.7289/V51G0J70

Waring, G.T., E. Josephson, K. Maze-Foley, and P.E. Rosel. 2016. U.S. Atlantic and Gulf of Mexico marine mammal stock assessments - 2015. National Oceanic and Atmospheric Administration, National Marine Fisheries Service, Northeast Fisheries Science Center: Woods Hole, MA. NOAA Technical Memorandum NMFS-NE-238. 501 p. doi: 10.7289/V57S7KTN

Wells, R.S. 2000. Reproduction in wild bottlenose dolphins: Overview of patterns observed during a long-term study. In: Duffield, D. and T. Robeck (Eds.), Bottlenose Dolphin Reproduction Workshop. AZA Marine Mammal Taxon Advisory Group, Silver Spring, MD. pp. 57–74.

Wells, R.S. 2003. Dolphin social complexity: Lessons from long-term study and life history. In: de Waal, F.B.M. and P.L. Tyack (Eds.), Animal Social Complexity: Intelligence, Culture, and Individualized Societies Harvard University Press, Cambridge, MA. pp. 32–56.

Wells, R.S. 2014. Social structure and life history of bottlenose dolphins near Sarasota Bay, Florida: Insights from four decades and five generations. Primates and cetaceans. pp. 149–172. doi: 10.1007/978-4-431-54523-1_8

Wells, R.S., A. Natoli, and G. Braulik. 2019. *Tursiops truncatus* (errata version published in 2019). *The IUCN Red List of Threatened Species* 2019: e.T22563A156932432. https://dx.doi.org/10.2305/IUCN.UK.2019-1.RLTS.T22563A156932432.en

Wells, R.S. and M.D. Scott. 1999. Bottlenose dolphin *Tursiops truncatus* (Montagu, 1821). In: Ridgway, S.H. and R. Harrison (Eds.), Handbook of marine mammals. Volume 6: The second book of dolphins and the porpoises. Academic Press, San Diego, CA. pp. 137–182.

Wells, R.S. and M.D. Scott. 2018. Bottlenose dolphin, *Tursiops truncatus*, common bottlenose dolphin. In: Würsig, B., J.G.M. Thewissen, and K.M. Kovacs (Eds.), Encyclopedia of Marine Mammals (Third Edition). Academic Press, London, UK. pp. 118–125. doi: 10.1016/B978-0-12-804327-1.00072-8

Wells, R.S., M.D. Scott, and A.B. Irvine. 1987. The social structure of free-ranging bottlenose dolphins. In: Genoways, H.H. (Ed.), Current Mammalogy. Plenum Press, New York, NY. pp. 247–305.

Wilson, R.M., R.B. Tyson, J.A. Nelson, B.C. Balmer, J.P. Chanton, and D.P. Nowacek. 2017. Niche differentiation and prey selectivity among common bottlenose dolphins (*Tursiops truncatus*) sighted in St. George Sound, Gulf of Mexico. Frontiers in Marine Science 4:235. doi: 10.3389/fmars.2017.00235

Yordy, J.E., M.A. Mollenhauer, R.M. Wilson, R.S. Wells, A. Hohn, J. Sweeney, L.H. Schwacke, T.K. Rowles, J.R. Kucklick, and M.M. Peden-Adams. 2010. Complex contaminant exposure in cetaceans: a comparative E-Screen analysis of bottlenose dolphin blubber and mixtures of four persistent organic pollutants. Environmental Toxicology and Chemistry 29(10):2143–2153. doi: 10.1002/etc.279

Zolman, E.S. 2002. Residence patterns of bottlenose dolphins (*Tursiops truncatus*) in the Stono River estuary, Charleston County, South Carolina, USA. Marine Mammal Science 18(4):879–892. doi: 10.1111/j.1748-7692.2002.tb01079.x

Common bottlenose dolphin − *Tursiops truncatus*

Florida Bay Stock

Overall Vulnerability Rank = Very High

Biological Sensitivity = Very High

Climate Exposure = Very High

Data Quality = 93% of scores ≥ 2

	Tursiops truncatus	Expert Scores	Data Quality	Expert Scores Plots (Portion by Category)
Sensitivity attributes	Prey/Diet Specificity	2.6	2.7	
	Habitat Specificity	3.5	2.3	
	Site Fidelity	3.8	2.7	
	Lifetime Reproductive Potential	2.0	2.3	
	Generation Length	2.7	2.3	
	Reproductive Plasticity	1.1	2.3	
	Migration	3.8	2.3	
	Home Range	3.7	2.3	
	Stock Abundance	3.0	2.3	
	Stock Abundance Trend	2.2	1.3	
	Cumulative Stressors	2.9	1.7	
	Sensitivity Score	Very High		
Exposure variables	Sea Surface Temperature (Change in mean)	3.9	3.0	
	Sea Surface Temperature (Change in variability)	1.1	3.0	
	Air Temperature (Change in mean)	2.9	3.0	
	Air Temperature (Change in variability)	1.1	3.0	
	Precipitation (Change in mean)	1.1	3.0	
	Precipitation (Change in variability)	1.1	3.0	
	Sea Surface Salinity (Change in mean)	3.3	2.7	
	Sea Surface Salinity (Change in variability)	1.1	3.0	
	Ocean pH (Change in mean)	3.9	3.0	
	Ocean pH (Change in variability)	1.1	3.0	
	Sea ice coverage (Change in mean)	1.0	3.0	
	Sea ice coverage (Change in variability)	1.0	3.0	
	Dissolved oxygen (Change in mean)	3.9	3.0	
	Dissolved oxygen (Change in variability)	1.1	3.0	
	Circulation	3.4	2.0	
	Sea level rise	3.9	3.0	
	Exposure Score	Very High		
Overall Vulnerability Rank		Very High		

Low Moderate High Very High

Common bottlenose dolphin (Florida Bay Stock)

Tursiops truncatus

CVA Results Summary

Overall Climate Vulnerability Rank: Very High (100% certainty from bootstrap analysis).

Climate Exposure: Very High. Four exposure factors scored greater than 3.5: Ocean pH (Standard anomaly) (3.93), Dissolved oxygen (Standard anomaly) (3.87), Sea level rise (3.87), and Sea Surface Temperature (Standard anomaly) (3.87).

Biological Sensitivity: Very High. Three sensitivity attributes scored greater than 3.5: Migration (3.80), Site Fidelity (3.80), and Home Range (3.73).

Distributional Response: High

Abundance Response: High

Phenology Response: High

Data Quality: 93 % of the data quality scores were 2 or greater, 82 % of sensitivity attribute data quality scores were 2 or higher.

Background Information

Stock Description

The Florida Bay Stock includes common bottlenose dolphins found in Florida Bay and within the Gulf of Mexico-side portion of the Florida Keys National Marine Sanctuary (FKNMS) southwest to Marathon, Florida (Waring et al. 2014).

Prey/Diet Specificity

Generally, common bottlenose dolphins consume a large variety of fish and/or squid (Barros and Odell 1990; Mead and Potter 1990; Wells and Scott 2018). Common bottlenose dolphins seem to show a consistent preference for sound-producing fishes (e.g., sciaenids, toadfish; Berens McCabe et al. 2010; Bowen-Stevens et al. 2021). Estuarine stocks along the southeast U.S. Atlantic coast have been reported to consume a variety of prey, with preference for sciaenids (Gannon and Waples 2004; Pate and McFee 2012). Pate and McFee (2012) identified 42 prey species from 24 families in a study of stranded dolphins along the South Carolina coast. In Sarasota Bay, Florida, Rossman et al. (2015) found some individual common bottlenose dolphins to show diet specialization despite the broader study group showing a generalist diet. Specialization can be influenced by locally abundant prey species (Shane et al. 1986; Vollmer and Rosel 2013) or can result from resource partitioning amongst sympatric populations (e.g., Torres and Read 2009; Wilson et al. 2017). Bottlenose dolphins in Florida Bay use three main foraging tactics, which vary across multiple habitats. However, individuals tend to specialize and only use one foraging tactic (Torres and Read 2009; Engleby and Powell 2019).

Habitat Specificity

Common bottlenose dolphins are found in a variety of marine and estuarine habitats (Wells and Scott 2018). The area inhabited by the Florida Bay Stock includes interconnected basins, grassy mud banks, mangrove islands, and shallow open water habitat, with runoff from the Everglades a primary input of freshwater (Fourqurean and Robblee 1999).

Site Fidelity

Torres (2007) found high individual site fidelity for common bottlenose dolphins in Florida Bay during summers (June-August) from 2002 to 2005. From August 2010 to June 2012, dedicated surveys were also carried out in the Florida coastal Everglades, including the northern portion of Florida Bay to investigate the occurrence, relative abundance, and behavior of bottlenose dolphins and suggested the need for additional research on site fidelity in Florida Bay due to low levels or resighting (Sarabia et al. 2018). Other estuarine stocks in the western North Atlantic have shown site fidelity (Waring et al. 2014), such as in South Carolina (Sloan 2006; Waring et al. 2016), central and southern Georgia (Balmer et al. 2011; Kucklick et al. 2011; Balmer et al. 2013), northern Florida (Caldwell 2001), central Florida (Odell and Asper 1990; Mazzoil et al. 2005, 2008a, 2008b), and southern Florida (Litz 2007; Torres 2007; McClellan et al. 2000).

Lifetime Reproductive Potential

Taylor et al. (2007) reported the interbirth interval of common bottlenose dolphins as 3.80 years, though other studies have shown it to be closer to three years (Leatherwood 1977; Wells and Scott 1999; Connor et al. 2000; Wells 2003). Thayer (2008) estimated mean interbirth interval of 2.9 years in North Carolina.

Generally, females reach sexual maturity before males, with females reaching sexual maturity at 5–13 years (Sergeant et al. 1973; Perrin and Reilly 1984; Wells et al. 1987; Mead and Potter 1990; Mann et al. 2000; Wells 2000; Wells and Scott 2018).

Females reproduce through most of their adult lives (Leatherwood 1977; Hohn et al. 1989; Wells and Scott 1999; Wells 2000; Wells 2003), but may produce fewer calves after they reach 30 years of age (Wells 2000). Sarasota Bay common bottlenose dolphins can give birth when they are as young as 6 years of age, and can continue until they are 48 years old (Wells and Scott 2018). Reproductive lifespan would therefore be up to 42 years based on long-term observation studies (Wells 2014; Wells and Scott 2018).

Longitudinal studies from Sarasota Bay, Florida have found that female bottlenose dolphins can live up to 67 years (Wells and Scott 2018). Studies based on growth layer groups in teeth suggest female bottlenose dolphins can live to 41 years along the Texas coast (Fernandez and Hohn 1998) and to 30 years along the Mississippi coast (Mattson et al. 2006).

Generation Length

Taylor et al. (2007) reported common bottlenose dolphin generation length of 20.6–21.1 years.

Reproductive Plasticity

Common bottlenose dolphin calving is generally diffusely seasonal, with one or more peaks, although births have been reported from all seasons (Vollmer and Rosel 2013; Wells and Scott 2018). A mid-summer calving peak (July through August) has been reported for bottlenose dolphins off Puerto Rico (Rodríguez-Ferrer 2001). Calf sightings in South Carolina peak in spring and early summer and again in early autumn (McFee et al. 2014). In North Carolina, Thayer et al. (2003) observed a strong birth peak in spring based on stranding data and a diffuse birth peak from late spring to early fall based on sightings. Calf sightings peak in the spring and summer months in Florida (Wells et al. 1987), Mississippi/Louisiana (Miller et al. 2010; Miller et al. 2013), and Texas (McHugh 1989; Henderson and Würsig 2007). Calf sightings in South Carolina peak in spring and early summer and again in early autumn (McFee et al. 2014).

Migration

The estuarine stocks in the western North Atlantic are thought to primarily comprise stable resident communities. However, transient or seasonal movements of some individuals into and out of stock areas are recorded in some areas (Waring et al. 2014; LaBrecque et al. 2015).

Other estuarine stocks in the western North Atlantic are known to spend warm months in estuarine waters then move to nearshore waters during cool months (Gubbins et al. 2003; Durden et al. 2011; Waring et al. 2014; Waring et al. 2016), though some remain entirely in estuarine waters (Mazzoil et al. 2008a).

Home Range

The home ranges of individuals within the western North Atlantic estuarine stocks are considered to generally include the areas within the stock boundaries and brief excursions beyond the stock boundaries (Waring et al. 2014; LaBrecque et al. 2015). Surveys conducted by the Dolphin Ecology Project since 1999 suggest the presence of common bottlenose dolphins throughout Florida Bay (Engleby et al. 2002). Studies have shown dolphin populations with constrained ranges in Georgia (Brunswick and Sapelo; Balmer et al. 2013); South Carolina (Silva et al. 2019), and North Carolina (Urian et al. 2013). Balmer et al. (2018), using satellite telemetry data, identified that dolphins considered to be members of the SGES Stock had different movement patterns with some individuals remaining entirely within the estuarine waters, while other animals use the larger estuarine waters, sounds, and to some degree coastal waters.

Stock Abundance

Torres (2007) identified 437 individuals from a summer-months photo-id catalog. Both of these studies may not differentiate between resident and non-resident individuals (Waring et al. 2014).

Stock Abundance Trend

Data are insufficient to complete an abundance trend analysis for this stock (Waring et al. 2014).

Cumulative Stressors

Common bottlenose dolphins face a wide range of individual and cumulative natural and anthropogenic threats (see reviews by Vollmer and Rosel 2013; Wells and Scott 2018; and Wells et al. 2019). These include such threats as shark predation, harmful algal blooms, recreational and commercial fishing gear, vessel traffic, pollution, habitat alteration, and provisioning.

Common bottlenose dolphins in Florida Bay interact with the hook and line fishery (Waring et al. 2014). Individuals of this stock have been reported stranded, with some strandings showing evidence of human interactions (Waring et al. 2014; also see NOAA National Marine Mammal Health and Stranding Response Database). For example, during the period 2007–2011, five common bottlenose dolphins stranded within the boundaries of Florida Bay, with one individual presenting evidence of a propeller scar (Waring et al. 2014).

The nearshore and estuarine habitats along the Atlantic seaboard are adjacent to areas of high human population and some are highly industrialized. Studies of bottlenose dolphin tissues from estuaries in the southeast U.S. have shown persistent organic pollutant (POP; Schwacke et al. 2002, 2012; Hansen et al. 2004; Litz et al. 2007; Pulster et al. 2009; Fair et al. 2010; Yordy et al. 2010; Balmer et al. 2011; Kucklick et al. 2011; Reif et al. 2017) and heavy metal (Durden et al. 2007; Stavros et al. 2007, 2008, 2011; Titcomb et al. 2017; Reif et al. 2017; Page-Karjian et al. 2020) concentrations that may result in adverse effects on health or reproductive rates, particularly in urbanized, developed, and agricultural areas. In nearby Biscayne Bay, Florida, elevated POP concentrations were observed in individuals associated with more polluted areas compared to individuals associated with the less polluted areas of Biscayne Bay, suggesting that exposure to pollutants may be highly variable based on geography (Litz et al. 2007; Kucklick et al. 2011). However, studies have shown POP levels in Florida Bay dolphins to be among the lowest of all sampled in the southeast U.S. (Fair et al. 2003; Schwacke et al. 2004; Damseaux et al. 2017). In contrast, mercury has been detected in this stock at high concentrations relative to other stocks in the southeast United States (Damseaux et al. 2017).

The Florida Everglades that bound the northern edge of Florida Bay have been subjected to engineered flood control and water diversion that affect freshwater flow into Florida Bay and resulting changes in salinity and cascading effects on biota and habitat (Rudnick et al. 2005).

Further Reading

Waring, G.T., E. Josephson, K. Maze-Foley, P.E. Rosel, T.V.N. Cole, L. Engleby, L.P. Garrison, A. Henry, K. Mullin, C. Orphanides, R.M. Pace, D.L. Palka, M. Lyssikatos, and F.W. Wenzel. 2014. U.S. Atlantic and Gulf of Mexico Marine Mammal Stock Assessments - 2013. National Oceanic and Atmospheric Administration, National Marine Fisheries Service, Northeast Fisheries Science Center: Woods Hole, MA. NOAA Technical Memorandum NMFS-NE-228. 464 p. doi: 10.7289/V51G0J70

Wells, R.S. and M.D. Scott. 2018. Bottlenose Dolphin, *Tursiops truncatus*, Common Bottlenose Dolphin. In: Würsig, B., J.G.M. Thewissen, and K.M. Kovacs (Eds.), Encyclopedia of Marine Mammals (Third Edition). Academic Press, London, UK. pp. 118–125. doi: 10.1016/B978-0-12-804327-1.00072-8

Wells, R.S., A. Natoli, and G. Braulik. 2019. *Tursiops truncatus* (errata version published in 2019). The IUCN Red List of Threatened Species 2019: e.T22563A156932432. https://dx.doi.org/10.2305/IUCN.UK.2019-1.RLTS.T22563A156932432.en

References

Balmer, B., E. Zolman, T. Rowles, C. Smith, F. Townsend, D. Fauquier, C. George, T. Goldstein, L. Hansen, B. Quigley, W. McFee, J. Morey, P. Rosel, J. Saliki, T. Speakman, and L. Schwacke. 2018. Ranging patterns, spatial overlap, and association with dolphin morbillivirus exposure in common bottlenose dolphins (*Tursiops truncatus*) along the Georgia, USA coast. Ecology and Evolution 8(24):12890–12904. doi: 10.1002/ece3.4727

Balmer, B.C., L.H. Schwacke, R.S. Wells, J.D. Adams, R. Clay George, S.M. Lane, W.A. McLellan, P.E. Rosel, K. Sparks, T. Speakman, E.S. Zolman, and D. Ann Pabst. 2013. Comparison of abundance and habitat usage for common bottlenose dolphins between sites exposed to differential anthropogenic stressors within the estuaries of southern Georgia, U.S.A. Marine Mammal Science 29(2):E114–E135. doi: 10.1111/j.1748-7692.2012.00598.x

Balmer, B.C., L.H. Schwacke, R.S. Wells, R.C. George, J. Hoguet, J.R. Kucklick, S.M. Lane, A. Martinez, W.A. McLellan, P.E. Rosel, T.K. Rowles, K. Sparks, T. Speakman, E.S. Zolman, and D.A. Pabst. 2011. Relationship between persistent organic pollutants (POPs) and ranging patterns in common bottlenose dolphins (*Tursiops truncatus*) from coastal Georgia, USA. Science of the Total Environment 409(11):2094–2101. doi: 10.1016/j.scitotenv.2011.01.052

Barros, N.B. and D.K. Odell. 1990. Food habits of bottlenose dolphins in the southeastern United States In: Leatherwood, S. and R.R. Reeves (Eds.), The Bottlenose Dolphin. Academic Press, San Diego, CA. pp. 309–328. doi: 10.1016/B978-0-12-440280-5.50020-2

Berens McCabe, E.J., D.P. Gannon, N.B. Barros, and R.S. Wells. 2010. Prey selection by resident common bottlenose dolphins (*Tursiops truncatus*) in Sarasota Bay, Florida. Marine Biology 157(5):931–942. doi: 10.1007/s00227-009-1371-2

Bowen-Stevens, S.R., D.P. Gannon, R.A. Hazelkorn, G. Lovewell, K.M. Volker, S. Smith, M.C. Tumlin, and J. Litz. 2021. Diet of common bottlenose dolphins, *Tursiops truncatus*, that stranded in and near Barataria Bay, Louisiana, 2010–2012. Southeastern Naturalist 20(1). doi: 10.1656/058.020.0113

Caldwell, M. 2001. Social and genetic structure of bottlenose dolphin (*Tursiops truncatus*) in Jacksonville, Florida. University of Miami. 143 p.

Connor, R.C., R.S. Wells, J. Mann, and A.J. Read. 2000. The bottlenose dolphin: Social relationships in a fission-fusion society. In: Mann, J., R. Connor, P. Tyack, and H. Whitehead (Eds.), Cetacean Societies: Field Studies of Dolphins and Whales. University of Chicago Press, Chicago, IL. pp. 91–126.

Damseaux, F., J.J. Kiszka, M.R. Heithaus, G. Scholl, G. Eppe, J.P. Thome, J. Lewis, W. Hao, M.C. Fontaine, and K. Das. 2017. Spatial variation in the accumulation of POPs and mercury in bottlenose dolphins of the Lower Florida Keys and the coastal Everglades (South Florida). Environmental Pollution 220(Pt A):577–587. doi: 10.1016/j.envpol.2016.10.005

Durden, W.N., E.D. Stolen, and M.K. Stolen. 2011. Abundance, distribution, and group composition of Indian River Lagoon bottlenose dolphins (*Tursiops truncatus*). Aquatic Mammals 37(2):175–186. doi: 10.1578/Am.37.2.2011.175

Durden, W.N., M.K. Stolen, D.H. Adams, and E.D. Stolen. 2007. Mercury and selenium concentrations in stranded bottlenose dolphins from the Indian River Lagoon system, Florida. Bulletin of Marine Science 81(1):37–54.

Engleby, L.K. and J.R. Powell. 2019. Detailed observations and mechanisms of mud ring feeding by common bottlenose dolphins (*Tursiops truncatus truncatus*) in Florida Bay, Florida, U.S.A. Marine Mammal Science 35(3):1162–1172. doi: 10.1111/mms.12583

Engleby, L.K., A.J. Read, D. Waples, and L. Torres. 2002. Habitat use of bottlenose dolphins (*Tursiops truncatus*) in Florida Bay. Final Report to the Southeast Fisheries Science Center, National Marine Fisheries Service. 12 p.

Fair, P., L. Schwacke, E. Zolman, W. McFee, and L. Engleby. 2003. Assessment of contaminant concentrations in tissues of bottlenose dolphins (*Tursiops truncatus*) in Florida Bay. Harbor Branch Oceanographic Institute Protect Wild Dolphins Grant. RFP-PWD-2001. 18 p.

Fair, P.A., J. Adams, G. Mitchum, T.C. Hulsey, J.S. Reif, M. Houde, D. Muir, E. Wirth, D. Wetzel, E. Zolman, W. McFee, and G.D. Bossart. 2010. Contaminant blubber burdens in Atlantic bottlenose dolphins (*Tursiops truncatus*) from two southeastern US estuarine areas: Concentrations and patterns of PCBs, pesticides, PBDEs, PFCs, and PAHs. Science of the Total Environment 408(7):1577–1597. doi: 10.1016/j.scitotenv.2009.12.021

Fernandez, S. and A.A. Hohn. 1998. Age, growth, and calving season of bottlenose dolphins, *Tursiops truncatus*, off coastal Texas. Fishery Bulletin 96(2):357–365.

Fourqurean, J.W. and M.B. Robblee. 1999. Florida Bay: A history of recent ecological changes. Estuaries 22(2B):345–357. doi: 10.2307/1353203

Gannon, D.P. and D.M. Waples. 2004. Diets of coastal bottlenose dolphins from the U. S. Mid-Atlantic coast differ by habitat. Marine Mammal Science 20(3):527–545. doi: 10.1111/j.1748-7692.2004.tb01177.x

Gubbins, C.M., M. Caldwell, S.G. Barco, K.A. Rittmaster, N. Bowles, and V.G. Thayer. 2003. Abundance and sighting patterns of bottlenose dolphins (*Tursiops truncatus*) at four northwest Atlantic coastal sites. Journal of Cetacean Research and Management 5(2):141–148.

Hansen, L.J., L.H. Schwacke, G.B. Mitchum, A.A. Hohn, R.S. Wells, E.S. Zolman, and P.A. Fair. 2004. Geographic variation in polychorinated biphenyl and organochlorine pesticide concentrations in the blubber of bottlenose dolphins from the US Atlantic coast. Science of the Total Environment 319(1-3):147–172. doi: 10.1016/S0048-9697(03)00371-1

Henderson, E.E. and B. Würsig. 2007. Behavior patterns of bottlenose dolphins in San Luis Pass, Texas. Gulf of Mexico Science 25(2):153–161. doi: 10.18785/goms.2502.06

Hohn, A.A., M.D. Scott, R.S. Wells, J.C. Sweeney, and A.B. Irvine. 1989. Growth layers in teeth from known-age, free-ranging bottlenose dolphins. Marine Mammal Science 5(4):315–342. doi: 10.1111/j.1748-7692.1989.tb00346.x

Kucklick, J., L. Schwacke, R. Wells, A. Hohn, A. Guichard, J. Yordy, L. Hansen, E. Zolman, R. Wilson, J. Litz, D. Nowacek, T. Rowles, R. Pugh, B. Balmer, C. Sinclair, and P. Rosel. 2011. Bottlenose dolphins as indicators of persistent organic pollutants in the western North Atlantic Ocean and Northern Gulf of Mexico. Environmental Science & Technology 45(10):4270–4277. doi: 10.1021/es1042244

LaBrecque, E., C. Curtice, J. Harrison, S.M. Van Parijs, and P.N. Halpin. 2015. 2. Biologically Important Areas for Cetaceans Within U.S. Waters – East Coast Region. Aquatic Mammals 41(1):17–29. doi: 10.1578/am.41.1.2015.17

Leatherwood, S. 1977. Some preliminary impressions on the numbers and social behavior of free-swimming bottlenosed dolphin calves (*Tursiops truncatus*) in the northern Gulf of Mexico. In: Ridgway, S.H. and K. Bernirschke (Eds.), Breeding Dolphins; Present Status, Suggestions for the Future. Final report to the US Marine Mammal Commission, Contract MM6AC009, MMC-76/07, Washington, DC. pp. 143–167.

Litz, J.A. 2007. Social structure, genetic structure, and persistent organohalogen pollutants in bottlenose dolphins (*Tursiops truncatus*) in Biscayne Bay, Florida. University of Miami. 140 p.

Litz, J.A., L.P. Garrison, L.A. Fieber, A. Martinez, J.P. Contillo, and J.R. Kucklick. 2007. Fine-scale spatial variation of persistent organic pollutants in bottlenose dolphins (*Tursiops truncatus*) in Biscayne Bay, Florida. Environmental Science & Technology 41(21):7222–7228. doi: 10.1021/es070440r

Mann, J., R.C. Connor, L.M. Barre, and M.R. Heithaus. 2000. Female reproductive success in bottlenose dolphins (*Tursiops* sp.): life history, habitat, provisioning, and group-size effects. Behavioral Ecology 11(2):210–219. doi: 10.1093/beheco/11.2.210

Mattson, M.C., K.D. Mullin, G.W. Ingram, and W. Hoggard. 2006. Age structure and growth of the bottlenose dolphin (*Tursiops truncatus*) from strandings in the Mississippi sound region of the north-central Gulf of Mexico from 1986 to 2003. Marine Mammal Science 22(3):654–666. doi: 10.1111/j.1748-7692.2006.00057.x

Mazzoil, M., S.D. McCulloch, and R.H. Defran. 2005. Observations on the site fidelity of bottlenose dolphins (*Tursiops truncatus*) in the Indian River Lagoon, Florida. Florida Scientist 68(4):217–226.

Mazzoil, M., J.S. Reif, M. Youngbluth, M.E. Murdoch, S.E. Bechdel, E. Howells, S.D. McCulloch, L.J. Hansen, and G.D. Bossart. 2008a. Home ranges of bottlenose dolphins (*Tursiops truncatus*) in the Indian River Lagoon, Florida: Environmental correlates and implications for management strategies. Ecohealth 5(3):278–288. doi: 10.1007/s10393-008-0194-9

Mazzoil, M.S., S.D. McCulloch, M.J. Youngbluth, D.S. Kilpatrick, M.E. Murdoch, B. Mase-Guthrie, D.K. Odell, and G.D. Bossart. 2008b. Radio-tracking and survivorship of two rehabilitated bottlenose dolphins (*Tursiops truncatus*) in the Indian River Lagoon, Florida. Aquatic Mammals 34:54–64. doi: 10.1578/AM.34.1.2008.54

McClellan, D.B., J. Browder, J.L. Tobias, G.J. Konoval, M.D. Heron, O.L. Bass, and J. Osborne. 2000. Opportunistic sightings of bottlenose dolphin, *Tursiops truncatus*, along the southeast Florida coast and Florida Bay, 1992-1997. National Oceanic and Atmospheric Administration, National Marine Fisheries Service, Southeast Fisheries Science Center: Miami, FL. NOAA Technical Memorandum NMFS-SEFSC-435. 18 p.

McFee, W.E., T.R. Speakman, L. Balthis, J.D. Adams, and E.S. Zolman. 2014. Reproductive seasonality of a recently designated bottlenose dolphin stock near Charleston, South Carolina, U.S.A. Marine Mammal Science 30(2):528–543. doi: 10.1111/mms.12055

McHugh, M.B. 1989. Population numbers and feeding behavior of the Atlantic bottlenose dolphin (*Tursiops truncatus*) near Aransas Pass, Texas. University of Texas at Austin. Thesis. 96 p.

Mead, J.G. and C.W. Potter. 1990. Natural history of bottlenose dolphins along the central Atlantic coast of the United States. In: Leatherwood, S. and R.R. Reeves (Eds.), The Bottlenose Dolphin. Academic Press, San Diego, CA. pp. 165–195.

Miller, L.J., A.D. Mackey, T. Hoffland, M. Solangi, and S.A. Kuczaj. 2010. Potential effects of a major hurricane on Atlantic bottlenose dolphin (*Tursiops truncatus*) reproduction in the Mississippi Sound. Marine Mammal Science 26(3):707–715. doi: 10.1111/j.1748-7692.2010.00371.x

Miller, L.J., A.D. Mackey, M. Solangi, and S.A. Kuczaj. 2013. Population abundance and habitat utilization of bottlenose dolphins in the Mississippi Sound. Aquatic Conservation: Marine and Freshwater Ecosystems 23(1):145–151. doi: 10.1002/aqc.2278

Odell, D.K. and E.D. Asper. 1990. Distribution and movements of freeze-branded bottlenose dolphins in the Indian and Banana Rivers, Florida. In: Leatherwood, S. and R. Reeves (Eds.), The Bottlenose Dolphin. Academic Press, San Diego, CA. pp. 515–540.

Page-Karjian, A., C.F. Lo, B. Ritchie, C.A. Harms, D.S. Rotstein, S. Han, S.M. Hassan, A.F. Lehner, J.P. Buchweitz, V.G. Thayer, J.M. Sullivan, E.F. Christiansen, and J.R. Perrault. 2020. Anthropogenic contaminants and histopathological findings in stranded cetaceans in the southeastern United States, 2012–2018. Frontiers in Marine Science 7:630. doi: 10.3389/fmars.2020.00630

Pate, S.M. and W.E. McFee. 2012. Prey species of bottlenose dolphins (*Tursiops truncatus*) from South Carolina Waters. Southeastern Naturalist 11(1):1–22. doi: 10.1656/058.011.0101

Perrin, W.F. and S.B. Reilly. 1984. Reproductive parameters of dolphins and small whales of the family Delphinidae. In: Perrin, W.F., R.L. Brownell, Jr., and D.P. DeMaster (Eds.), Reproduction in Whales, Dolphins, and Porpoises. pp. 97–134.

Pulster, E.L., K.L. Smalling, E. Zolman, L. Schwacke, and K.A. Maruya. 2009. Persistent organochlorine pollutants and toxaphene congener profiles in bottlenose dolphins (*Tursiops truncatus*) frequenting the Turtle/Brunswick River Estuary, Georgia, USA. Environmental Toxicology and Chemistry 28(7):1390–1399. doi: 10.1897/08-240.1

Reif, J.S., A.M. Schaefer, G.D. Bossart, and P.A. Fair. 2017. Health and Environmental Risk Assessment Project for bottlenose dolphins *Tursiops truncatus* from the southeastern USA. II. Environmental aspects. Diseases of Aquatic Organisms 125(2):155–166. doi: 10.3354/dao03143

Rodríguez-Ferrer, G. 2001. The bottlenose dolphin (*Tursiops truncatus* Montague 1821) of the southwest coast of Puerto Rico: An assessment of its status and habitat use. University of Puerto Rico. Thesis.

Rossman, S., P.H. Ostrom, M. Stolen, N.B. Barros, H. Gandhi, C.A. Stricker, and R.S. Wells. 2015. Individual specialization in the foraging habits of female bottlenose dolphins living in a trophically diverse and habitat rich estuary. Oecologia 178(2):415–425. doi: 10.1007/s00442-015-3241-6

Rudnick, D.T., P.B. Ortner, J.A. Browder, and S.M. Davis. 2005. A conceptual ecological model of Florida Bay. Wetlands 25(4):870–883. doi: 10.1672/0277-5212(2005)025[0870:acemof]2.0.co;2

Sarabia, R.E., M.R. Heithaus, and J.J. Kiszka. 2018. Spatial and temporal variation in abundance, group size and behaviour of bottlenose dolphins in the Florida coastal Everglades. Journal of the Marine Biological Association of the United Kingdom 98(5):1097–1107. doi: 10.1017/s002531541700090x

Schwacke, L., A.J. Hall, R.S. Wells, G.D. Bossart, P. Fair, A.A. Hohn, P.R. Becker, J. Kucklick, G.B. Mitchum, and P.E. Rosel. 2004. Health and risk assessment for bottlenose dolphin (*Tursiops truncatus*) populations along the southeast United States coast: Current status and future plans. Paper presented to the IWC Scientific Committee: Sorrento, Italy. SC/56/E20 15 p.

Schwacke, L.H., E.O. Voit, L.J. Hansen, R.S. Wells, G.B. Mitchum, A.A. Hohn, and P.A. Fair. 2002. Probabilistic risk assessment of reproductive effects of polychlorinated biphenyls on bottlenose dolphins (*Tursiops truncatus*) from the southeast United States coast. Environmental Toxicology and Chemistry 21(12):2752–2764. doi: 10.1002/etc.5620211232

Schwacke, L.H., E.S. Zolman, B.C. Balmer, S. De Guise, R.C. George, J. Hoguet, A.A. Hohn, J.R. Kucklick, S. Lamb, M. Levin, J.A. Litz, W.E. McFee, N.J. Place, F.I. Townsend, R.S. Wells, and T.K. Rowles. 2012. Anaemia, hypothyroidism and immune suppression associated with polychlorinated biphenyl exposure in bottlenose dolphins (*Tursiops truncatus*). Proceedings of the Royal Society B: Biological Sciences 279(1726):48–57. doi: 10.1098/rspb.2011.0665

Sergeant, D.E., D.K. Caldwell, and M.C. Caldwell. 1973. Age, growth, and maturity of bottlenosed dolphin (*Tursiops-truncatus*) from northeast Florida. Journal of the Fisheries Research Board of Canada 30(7):1009–1011. doi: 10.1139/f73-165

Shane, S.H., R.S. Wells, and B. Würsig. 1986. Ecology, behavior and social organization of the bottlenose dolphin: A review. Marine Mammal Science 2:34–63. doi: 10.1111/j.1748-7692.1986.tb00026.x

Silva, D., B. Tramutolo, E. DeSalvio, T. Speakman, and R. Young. 2019. Abundance and movements of the Northern South Carolina Estuarine System Stock of bottlenose dolphins (*Tursiops truncatus*) (USA). Journal of Marine Animals and Their Ecology 11(1):8–18.

Sloan, P.E. 2006. Residency patterns, seasonality and habitat use among bottlenose dolphins, *Tursiops truncatus*, in the Cape Romain National Wildlife Refuge, S.C. University of North Carolina Wilmington. Thesis. 75 p.

Stavros, H.-C.W., G.D. Bossart, T.C. Hulsey, and P.A. Fair. 2007. Trace element concentrations in skin of free-ranging bottlenose dolphins (*Tursiops truncatus*) from the southeast Atlantic coast. Science of the Total Environment 388(1-3):300–315. doi: 10.1016/j.scitotenv.2007.07.030

Stavros, H.-C.W., G.D. Bossart, T.C. Hulsey, and P.A. Fair. 2008. Trace element concentrations in blood of free-ranging bottlenose dolphins (*Tursiops truncatus*): Influence of age, sex and location. Marine Pollution Bulletin 56(2):371–379. doi: 10.1016/j.marpolbul.2007.10.030

Stavros, H.-C.W., M. Stolen, W.N. Durden, W. McFee, G.D. Bossart, and P.A. Fair. 2011. Correlation and toxicological inference of trace elements in tissues from stranded and free-ranging bottlenose dolphins (*Tursiops truncatus*). Chemosphere 82(11):1649–1661. doi: 10.1016/j.chemosphere.2010.11.019

Taylor, B.L., S.J. Chivers, J. Larese, and W.F. Perrin. 2007. Generation length and percent mature estimates for IUCN assessments of cetaceans. National Oceanic and Atmospheric Administration, National Marine Fisheries Service, Southwest Fisheries Science Center: La Jolla, CA. Administrative Report LJ-07-01. 24 p.

Thayer, V.G. 2008. Life history parameters and social associations of female bottlenose dolphins (*Tursiops truncatus*) off North Carolina, USA. Duke University. Dissertation. 164 p.

Thayer, V.G., A.J. Read, A.S. Friedlaender, D.R. Colby, A.A. Hohn, W.A. McLellan, D.A. Pabst, J.L. Dearolf, N.I. Bowles, J.R. Russell, and K.A. Rittmaster. 2003. Reproductive seasonality of western Atlantic bottlenose dolphins off North Carolina, USA. Marine Mammal Science 19(4):617–629. doi: 10.1111/j.1748-7692.2003.tb01120.x

Titcomb, E.M., J.S. Reif, P.A. Fair, H.-C.W. Stavros, M. Mazzoil, G.D. Bossart, and A.M. Schaefer. 2017. Blood mercury concentrations in common bottlenose dolphins from the Indian River Lagoon, Florida: Patterns of social distribution. Marine Mammal Science 33(3):771–784. doi: 10.1111/mms.12390

Torres, L.G. 2007. Top predator distribution and foraging ecology in Florida Bay, Florida. Duke University. Dissertation. 225 p.

Torres, L.G. and A.J. Read. 2009. Where to catch a fish? The influence of foraging tactics on the ecology of bottlenose dolphins (*Tursiops truncatus*) in Florida Bay, Florida. Marine Mammal Science 25(4):797–815. doi: 10.1111/j.1748-7692.2009.00297.x

Urian, K.W., D.M. Waples, R.B. Tyson, L.E. Hodge, and A.J. Read. 2013. Abundance of bottlenose dolphins (*Tursiops truncatus*) in estuarine and near-shore waters of North Carolina, USA. Journal of the North Carolina Academy of Science 129(4):165–171. doi: 10.7572/2167-5880-129.4.165

Vollmer, N.L. and P.E. Rosel. 2013. A review of common bottlenose dolphins (*Tursiops truncatus truncatus*) in the Northern Gulf of Mexico: Population biology, potential threats, and management. Southeastern Naturalist 12(m6):1–43. doi: 10.1656/058.012.m601

Waring, G., E. Josephson, K. Maze-Foley, and P. Rosel. 2014. U.S. Atlantic and Gulf of Mexico Marine Mammal Stock Assessments - 2013. National Oceanic and Atmospheric Administration, National Marine Fisheries Service, Northeast Fisheries Science Center: Woods Hole, MA. NOAA Technical Memorandum NMFS-NE-228. 464 p. doi: 10.7289/V51G0J70

Waring, G.T., E. Josephson, K. Maze-Foley, and P.E. Rosel. 2016. U.S. Atlantic and Gulf of Mexico marine mammal stock assessments - 2015. National Oceanic and Atmospheric Administration, National Marine Fisheries Service, Northeast Fisheries Science Center: Woods Hole, MA. NOAA Technical Memorandum NMFS-NE-238. 501 p. doi: 10.7289/V57S7KTN

Wells, R.S. 2000. Reproduction in wild bottlenose dolphins: Overview of patterns observed during a long-term study. In: Duffield, D. and T. Robeck (Eds.), Bottlenose Dolphin Reproduction Workshop. AZA Marine Mammal Taxon Advisory Group, Silver Spring, MD. pp. 57–74.

Wells, R.S. 2003. Dolphin social complexity: Lessons from long-term study and life history. In: de Waal, F.B.M. and P.L. Tyack (Eds.), Animal Social Complexity: Intelligence, Culture, and Individualized Societies Harvard University Press, Cambridge, MA. pp. 32–56.

Wells, R.S. 2014. Social structure and life history of bottlenose dolphins near Sarasota Bay, Florida: Insights from four decades and five generations. Primates and cetaceans. pp. 149–172. doi: 10.1007/978-4-431-54523-1_8

Wells, R.S., A. Natoli, and G. Braulik. 2019. *Tursiops truncatus* (errata version published in 2019). *The IUCN Red List of Threatened Species* 2019: e.T22563A156932432. https://dx.doi.org/10.2305/IUCN.UK.2019-1.RLTS.T22563A156932432.en

Wells, R.S. and M.D. Scott. 1999. Bottlenose dolphin *Tursiops truncatus* (Montagu, 1821). In: Ridgway, S.H. and R. Harrison (Eds.), Handbook of marine mammals. Volume 6: The second book of dolphins and the porpoises. Academic Press, San Diego, CA. pp. 137–182.

Wells, R.S. and M.D. Scott. 2018. Bottlenose dolphin, *Tursiops truncatus*, common bottlenose dolphin. In: Würsig, B., J.G.M. Thewissen, and K.M. Kovacs (Eds.), Encyclopedia of Marine Mammals (Third Edition). Academic Press, London, UK. pp. 118–125. doi: 10.1016/B978-0-12-804327-1.00072-8

Wells, R.S., M.D. Scott, and A.B. Irvine. 1987. The social structure of free-ranging bottlenose dolphins. In: Genoways, H.H. (Ed.), Current Mammalogy. Plenum Press, New York, NY. pp. 247–305.

Wilson, R.M., R.B. Tyson, J.A. Nelson, B.C. Balmer, J.P. Chanton, and D.P. Nowacek. 2017. Niche differentiation and prey selectivity among common bottlenose dolphins (*Tursiops truncatus*) sighted in St. George Sound, Gulf of Mexico. Frontiers in Marine Science 4:235. doi: 10.3389/fmars.2017.00235

Yordy, J.E., M.A. Mollenhauer, R.M. Wilson, R.S. Wells, A. Hohn, J. Sweeney, L.H. Schwacke, T.K. Rowles, J.R. Kucklick, and M.M. Peden-Adams. 2010. Complex contaminant exposure in cetaceans: a comparative E-Screen analysis of bottlenose dolphin blubber and mixtures of four persistent organic pollutants. Environmental Toxicology and Chemistry 29(10):2143–2153. doi: 10.1002/etc.279

Common bottlenose dolphin − *Tursiops truncatus*

Indian River Lagoon Estuarine System Stock

Overall Vulnerability Rank = Very High

Biological Sensitivity = Very High

Climate Exposure = Very High

Data Quality = 100% of scores≥ 2

	Tursiops truncatus	Expert Scores	Data Quality	Expert Scores Plots (Portion by Category)
Sensitivity attributes	Prey/Diet Specificity	2.4	2.2	
	Habitat Specificity	3.4	2.0	
	Site Fidelity	3.7	2.8	
	Lifetime Reproductive Potential	1.9	2.2	
	Generation Length	2.7	2.2	
	Reproductive Plasticity	1.2	2.0	
	Migration	3.9	3.0	
	Home Range	3.8	3.0	
	Stock Abundance	2.9	3.0	
	Stock Abundance Trend	2.7	2.2	
	Cumulative Stressors	3.5	2.5	
	Sensitivity Score	Very High		
Exposure variables	Sea Surface Temperature (Change in mean)	4.0	3.0	
	Sea Surface Temperature (Change in variability)	1.8	3.0	
	Air Temperature (Change in mean)	3.2	3.0	
	Air Temperature (Change in variability)	1.0	3.0	
	Precipitation (Change in mean)	1.1	3.0	
	Precipitation (Change in variability)	1.1	3.0	
	Sea Surface Salinity (Change in mean)	3.4	2.8	
	Sea Surface Salinity (Change in variability)	1.1	2.8	
	Ocean pH (Change in mean)	3.7	3.0	
	Ocean pH (Change in variability)	1.1	3.0	
	Sea ice coverage (Change in mean)	1.0	3.0	
	Sea ice coverage (Change in variability)	1.0	3.0	
	Dissolved oxygen (Change in mean)	3.7	2.8	
	Dissolved oxygen (Change in variability)	1.0	2.8	
	Circulation	3.9	2.5	
	Sea level rise	4.0	3.0	
	Exposure Score	Very High		
Overall Vulnerability Rank		Very High		

Low
Moderate
High
Very High

Common bottlenose dolphin (Indian River Lagoon Estuarine System Stock)

Tursiops truncatus

CVA Results Summary

Overall Climate Vulnerability Rank: Very High (100% certainty from bootstrap analysis).

Climate Exposure: Very High. Five exposure factors scored greater than 3.5: Sea level rise (3.95), Sea Surface Temperature (Standard anomaly) (3.95), Circulation (3.90), Dissolved oxygen (Standard anomaly) (3.70), and Ocean pH (Standard anomaly) (3.70).

Biological Sensitivity: Very High. Four sensitivity attributes scored greater than or equal to 3.5: Migration (3.90), Home Range (3.80), Site Fidelity (3.70), and Cumulative Stressors (3.50).

Distributional Response: High

Abundance Response: Very High

Phenology Response: High

Data Quality: 100% of the data quality scores were 2 or greater, 100% of sensitivity attribute data quality scores were 2 or higher.

Background Information

Stock Description

The Indian River Lagoon Estuarine System (IRLES) Stock includes common bottlenose dolphins in estuarine waters of Florida from Ponce de Leon Inlet in the north to Jupiter Inlet in the south, including the Intracoastal Waterway, Mosquito Lagoon, Indian River, Banana River and the St. Lucie Estuary (Waring et al. 2016).

Prey/Diet Specificity

Generally, common bottlenose dolphins consume a large variety of fish and/or squid (Barros and Odell 1990; Mead and Potter 1990; Wells and Scott 2018). Common bottlenose dolphins seem to show a consistent preference for sound-producing fishes (e.g., sciaenids, toadfish; Berens McCabe et al. 2010; Bowen-Stevens et al. 2021). Estuarine stocks along the southeast U.S. Atlantic coast have been reported to consume a variety of prey, with preference for sciaenids (Gannon and Waples 2004; Pate and McFee 2012). In Sarasota Bay, Florida, Rossman et al. (2015) found some individual common bottlenose dolphins to show diet specialization despite the broader study group showing a generalist diet. Specialization can be influenced by locally abundant prey species (Shane et al. 1986; Vollmer and Rosel 2013) or can result from resource partitioning amongst sympatric populations (e.g., Torres and Read 2009; Wilson et al. 2017).

Habitat Specificity

Common bottlenose dolphins are found in a variety of marine and estuarine habitats (Wells and Scott 2018). The area inhabited by the IRLES stock is characterized by estuarine tidal marsh systems (Waring et al. 2016). Greller et al. (2020) found salinity and dissolved oxygen to be key factors in common bottlenose dolphin habitat use in the IRLES.

Site Fidelity

Common bottlenose dolphins in the IRLES show strong site fidelity (Odell and Asper 1990; Mazzoil et al. 2005, 2008a, 2008b, 2020). Other estuarine stocks in the western North Atlantic have shown site fidelity (Waring et al. 2016), such as in southern Florida (Litz 2007; Torres 2007; McClellan et al. 2000; Torres 2007; Sarabia et al. 2018), northern Florida (Caldwell 2001), central and southern Georgia (Balmer et al. 2011; Kucklick et al. 2011; Balmer et al. 2013), and South Carolina (Sloan 2006; Speakman et al. 2006; Waring et al. 2016).

Lifetime Reproductive Potential

Taylor et al. (2007) reported the interbirth interval of common bottlenose dolphins as 3.80 years, though other studies have shown it to be closer to three years (Leatherwood 1977; Wells and Scott 1999; Connor et al. 2000; Wells 2003). Thayer (2008) estimated mean interbirth interval of 2.9 years in North Carolina.

Generally, females reach sexual maturity before males, with females reaching sexual maturity at 5–13 years (Sergeant et al. 1973; Perrin and Reilly 1984; Wells et al. 1987; Mead and Potter 1990; Mann et al. 2000; Wells 2000; Wells and Scott 2018).

Females reproduce through most of their adult lives (Leatherwood 1977; Hohn et al. 1989; Wells and Scott 1999; Wells 2000; Wells 2003), but may produce fewer calves after they reach 30 years of age (Wells 2000). Sarasota Bay common bottlenose dolphins can give birth when they are as young as 6 years of age, and can continue until they are 48 years old (Wells and Scott 2018). Reproductive lifespan would therefore be up to 42 years based on long-term observation studies (Wells 2014; Wells and Scott 2018).

Longitudinal studies from Sarasota Bay, Florida have found that female bottlenose dolphins can live up to 67 years (Wells and Scott 2018). Studies based on growth layer groups in teeth suggest female bottlenose dolphins can live to 41 years along the Texas coast (Fernandez and Hohn 1998) and to 30 years along the Mississippi coast (Mattson et al. 2006).

Generation Length

Taylor et al. (2007) reported common bottlenose dolphin generation length of 20.6–21.1 years.

Reproductive Plasticity

Common bottlenose dolphin calving is generally diffusely seasonal, with one or more peaks, although births have been reported from all seasons (Vollmer and Rosel 2013; Wells and Scott 2018). A mid-summer calving peak (July through August) has been reported for bottlenose dolphins off Puerto Rico

(Rodríguez-Ferrer 2001). Calf sightings in South Carolina peak in spring and early summer and again in early autumn (McFee et al. 2014). In North Carolina, Thayer et al. (2003) observed a strong birth peak in spring based on stranding data and a diffuse birth peak from late spring to early fall based on sightings. Calf sightings peak in the spring and summer months in Florida (Wells et al. 1987), Mississippi/Louisiana (Miller et al. 2010; Miller et al. 2013), and Texas (McHugh 1989; Henderson and Würsig 2007). Calf sightings in South Carolina peak in spring and early summer and again in early autumn (McFee et al. 2014).

Migration

The estuarine stocks in the western North Atlantic are thought to primarily comprise stable resident communities. However, transient or seasonal movements of some individuals into and out of stock areas are recorded in some areas (LaBrecque et al. 2015; Waring et al. 2016). In the IRLES, Durden et al. (2011) suggested movement of common bottlenose dolphins between the estuary and nearshore coastal waters, which was later supported by satellite tag data (Hartel et al. 2020). However, other studies suggest minimal movement between the estuary and nearshore coastal waters (Mazzoil et al. 2008a, 2011; Durden et al. 2019).

Other estuarine stocks in the western North Atlantic are known to spend warm months in estuarine waters then move to nearshore waters during cool months (Gubbins et al. 2003; Waring et al. 2014; Waring et al. 2016).

Home Range

The home ranges of individuals within the western North Atlantic estuarine stocks are considered to generally include the areas within the stock boundaries and brief excursions beyond the stock boundaries (LaBrecque et al. 2015; Waring et al. 2016), though studies may underestimate the size of the home range if the survey area is not sufficiently large (Nekolny et al. 2017). Two radio-tracked rehabilitated dolphins in the IRLES remained within the IRLES until their deaths (100 days and 7 days post-tagging; Mazzoil et al. 2008b). Stable isotope analysis has suggested subpopulations within the IRLES based on home range (Browning et al. 2014).

Studies have shown dolphin populations with constrained ranges in Georgia (Brunswick and Sapelo; Balmer et al. 2013); South Carolina (Silva et al. 2019), and North Carolina (Urian et al. 2013). Balmer et al. (2018), using satellite telemetry data, identified that dolphins considered to be members of the South Georgia Estuarine System Stock had different movement patterns with some individuals remaining entirely within the estuarine waters, while other animals use the larger estuarine waters, sounds, and to some degree coastal waters.

Stock Abundance

Mazzoil et al. (2008a) identified 615 individuals during 2002-2005 photo-ID studies. Aerial surveys conducted from summer 2002 through spring 2004 provided estimates ranging from 362 (CV=0.29) for summer 2003 to 1316 (CV=0.24; Durden et al. 2011). Overall mean abundance during the summer 2002 through spring 2004 period was estimated at 662 individuals (CV=0.09; Durden et al. 2011). Durden et al. (2017) estimated the population at 1,032 individuals (95% CI: 809–1,255) using 2005–2011 aerial

survey data. Durden et al. (2021) estimated the population at 1,032 individuals (95% CI: 969–1,098) using 2016 and 2017 boat-based survey data.

Stock Abundance Trend

Data are insufficient to complete an abundance trend analysis for this stock (Waring et al. 2016).

Cumulative Stressors

Common bottlenose dolphins face a wide range of individual and cumulative natural and anthropogenic threats (see reviews by Vollmer and Rosel 2013; Wells and Scott 2018; and Wells et al. 2019). These include such threats as shark predation, harmful algal blooms (Fire et al. 2015, 2020), recreational and commercial fishing gear (Stolen et al. 2013; Marks et al. 2020), vessel traffic, pollution, habitat alteration, and provisioning.

Common bottlenose dolphins in the IRLES stock interact with the blue crab trap/pot, stone crab trap/pot, and hook and line fisheries (Waring et al. 2016). Individuals of this stock have been reported stranded, with some strandings showing evidence of human interactions, and other strandings occurring during Unusual Mortality Events (UMEs; Waring et al. 2016; also see NOAA National Marine Mammal Health and Stranding Response Database). For example, Stolen et al. (2007) reported that 834 total dolphins stranded in the IRLES during the period 1977–2005, with more strandings in spring and summer. During the period 2009–2013, 227 common bottlenose dolphins stranded within the IRLES, with 30 individuals presenting interactions with fishing gear (Waring et al. 2016).

A UME was declared in the summer of 2013 for the mid-Atlantic coast from New York to Brevard County, Florida, with more than 1500 common bottlenose dolphins stranding as a result of morbillivirus (Waring et al. 2016). Strandings were more prevalent along oceanic shores than in estuaries, suggesting that coastal stocks were more impacted by the UME than estuarine stocks (Waring et al. 2016). During 2013, the IRLES Stock experienced a separate UME attributed to ecological factors (NMFS 2022). The IRLES Stock also experienced UMEs in 2001 and 2008, with toxins and infections contributing as possible causes (Fire et al. 2015; Waring et al. 2016). Dolphin morbillivirus has been detected in individuals from this stock during and outside of UMEs (Bossart et al. 2010, 2011, 2017, 2019).

The nearshore and estuarine habitats along the Atlantic seaboard are adjacent to areas of high human population and some are highly industrialized. Studies of bottlenose dolphin tissues from estuaries in the southeast U.S. have shown persistent organic pollutant (POP; Schwacke et al. 2002, 2012; Hansen et al. 2004; Litz et al. 2007; Pulster et al. 2009; Fair et al. 2010; Yordy et al. 2010; Balmer et al. 2011; Kucklick et al. 2011; ; Reif et al. 2017) and heavy metal (Durden et al. 2007; Stavros et al. 2007, 2008, 2011; Titcomb et al. 2017; Reif et al. 2017; Page-Karjian et al. 2020) concentrations that may result in adverse effects on health or reproductive rates, particularly in urbanized, developed, and agricultural areas. In Biscayne Bay, Florida, elevated POP concentrations were observed in individuals associated with more polluted areas compared to individuals associated with the less polluted areas of Biscayne Bay, suggesting that exposure to pollutants may be highly variable based on geography (Litz et al. 2007; Kucklick et al. 2011). In the IRLES, bottlenose dolphin tissues have shown high persistent POP concentrations (Hansen et al. 2004; Durden et al. 2007; Stavros et al. 2007, 2008; Fair et al. 2010; Stavros et al. 2011; Reif et al. 2017; Lynch et al. 2019), as well as high levels of lead, mercury, and selenium (Schaefer et al. 2011, 2015; Stavros et al. 2011; Titcomb et al. 2017; Reif et al. 2017; Page-

Karjian et al. 2020) that may result in adverse effects on health or reproductive rates. The IRLES stock has also been found to have high concentrations of per- and poly-fluoroalkyl substances (PFAS; Fair et al. 2012).

Human feeding of dolphins has been observed in the IRLES, with possible implications for increased interactions with fishing gear (Waring et al. 2016). Vessel strikes and altered behavior due to anthropogenic sound are additional threats to dolphins in IRLES (Bechdel et al. 2009).

Further Reading

Waring, G.T., E. Josephson, K. Maze-Foley, and P.E. Rosel. 2016. U.S. Atlantic and Gulf of Mexico Marine Mammal Stock Assessments - 2015. National Oceanic and Atmospheric Administration, National Marine Fisheries Service, Northeast Fisheries Science Center: Woods Hole, MA. NOAA Technical Memorandum NMFS-NE-238. 501 p. doi: 10.7289/V57S7KTN

Wells, R.S. and M.D. Scott. 2018. Bottlenose Dolphin, *Tursiops truncatus*, Common Bottlenose Dolphin. In: Würsig, B., J.G.M. Thewissen, and K.M. Kovacs (Eds.), Encyclopedia of Marine Mammals (Third Edition). Academic Press, London, UK. pp. 118–125. doi: 10.1016/B978-0-12-804327-1.00072-8

Wells, R.S., A. Natoli, and G. Braulik. 2019. *Tursiops truncatus* (errata version published in 2019). The IUCN Red List of Threatened Species 2019: e.T22563A156932432. https://dx.doi.org/10.2305/IUCN.UK.2019-1.RLTS.T22563A156932432.en

References

Balmer, B., E. Zolman, T. Rowles, C. Smith, F. Townsend, D. Fauquier, C. George, T. Goldstein, L. Hansen, B. Quigley, W. McFee, J. Morey, P. Rosel, J. Saliki, T. Speakman, and L. Schwacke. 2018. Ranging patterns, spatial overlap, and association with dolphin morbillivirus exposure in common bottlenose dolphins (*Tursiops truncatus*) along the Georgia, USA coast. Ecology and Evolution 8(24):12890–12904. doi: 10.1002/ece3.4727

Balmer, B.C., L.H. Schwacke, R.S. Wells, J.D. Adams, R. Clay George, S.M. Lane, W.A. McLellan, P.E. Rosel, K. Sparks, T. Speakman, E.S. Zolman, and D. Ann Pabst. 2013. Comparison of abundance and habitat usage for common bottlenose dolphins between sites exposed to differential anthropogenic stressors within the estuaries of southern Georgia, U.S.A. Marine Mammal Science 29(2):E114–E135. doi: 10.1111/j.1748-7692.2012.00598.x

Balmer, B.C., L.H. Schwacke, R.S. Wells, R.C. George, J. Hoguet, J.R. Kucklick, S.M. Lane, A. Martinez, W.A. McLellan, P.E. Rosel, T.K. Rowles, K. Sparks, T. Speakman, E.S. Zolman, and D.A. Pabst. 2011. Relationship between persistent organic pollutants (POPs) and ranging patterns in common bottlenose dolphins (*Tursiops truncatus*) from coastal Georgia, USA. Science of the Total Environment 409(11):2094–2101. doi: 10.1016/j.scitotenv.2011.01.052

Barros, N.B. and D.K. Odell. 1990. Food habits of bottlenose dolphins in the southeastern United States In: Leatherwood, S. and R.R. Reeves (Eds.), The Bottlenose Dolphin. Academic Press, San Diego, CA. pp. 309–328. doi: 10.1016/B978-0-12-440280-5.50020-2

Bechdel, S.E., M.S. Mazzoil, M.E. Murdoch, E.M. Howells, J.S. Reif, S.D. McCulloch, A.M. Schaefer, and G.D. Bossart. 2009. Prevalence and impacts of motorized vessels on bottlenose dolphins (*Tursiops truncatus*) in the Indian River Lagoon, Florida. Aquatic Mammals 35(3):367–377. doi: 10.1578/am.35.3.2009.367

Berens McCabe, E.J., D.P. Gannon, N.B. Barros, and R.S. Wells. 2010. Prey selection by resident common bottlenose dolphins (*Tursiops truncatus*) in Sarasota Bay, Florida. Marine Biology 157(5):931–942. doi: 10.1007/s00227-009-1371-2

Bossart, G.D., P. Fair, A.M. Schaefer, and J.S. Reif. 2017. Health and Environmental Risk Assessment Project for bottlenose dolphins *Tursiops truncatus* from the southeastern USA. I. Infectious diseases. Diseases of Aquatic Organisms 125(2):141–153. doi: 10.3354/dao03142

Bossart, G.D., J.S. Reif, A.M. Schaefer, J. Goldstein, P.A. Fair, and J.T. Saliki. 2010. Morbillivirus infection in free-ranging Atlantic bottlenose dolphins (*Tursiops truncatus*) from the Southeastern United States: Seroepidemiologic and pathologic evidence of subclinical infection. Veterinary Microbiology 143(2-4):160–166. doi: 10.1016/j.vetmic.2009.11.024

Bossart, G.D., T.A. Romano, M.M. Peden-Adams, A. Schaefer, S. McCulloch, J.D. Goldstein, C.D. Rice, J.T. Saliki, P.A. Fair, and J.S. Reif. 2011. Clinicoimmunopathologic findings in Atlantic bottlenose dolphins *Tursiops truncatus* with positive cetacean morbillivirus antibody titers. Diseases of Aquatic Organisms 97(2):103–112. doi: 10.3354/dao02410

Bossart, G.D., T.A. Romano, M.M. Peden-Adams, A.M. Schaefer, C.D. Rice, P.A. Fair, and J.S. Reif. 2019. Comparative innate and adaptive immune responses in Atlantic bottlenose dolphins (*Tursiops truncatus*) with viral, bacterial, and fungal infections. Frontiers in Immunology 10:1125. doi: 10.3389/fimmu.2019.01125

Bowen-Stevens, S.R., D.P. Gannon, R.A. Hazelkorn, G. Lovewell, K.M. Volker, S. Smith, M.C. Tumlin, and J. Litz. 2021. Diet of common bottlenose dolphins, *Tursiops truncatus*, that stranded in and near Barataria Bay, Louisiana, 2010–2012. Southeastern Naturalist 20(1). doi: 10.1656/058.020.0113

Browning, N.E., S.D. McCulloch, G.D. Bossart, and G.A.J. Worthy. 2014. Fine-scale population structure of estuarine bottlenose dolphins (*Tursiops truncatus*) assessed using stable isotope ratios and fatty acid signature analyses. Marine Biology 161(6):1307–1317. doi: 10.1007/s00227-014-2420-z

Caldwell, M. 2001. Social and genetic structure of bottlenose dolphin (*Tursiops truncatus*) in Jacksonville, Florida. University of Miami. 143 p.

Connor, R.C., R.S. Wells, J. Mann, and A.J. Read. 2000. The bottlenose dolphin: Social relationships in a fission-fusion society. In: Mann, J., R. Connor, P. Tyack, and H. Whitehead (Eds.), Cetacean Societies: Field Studies of Dolphins and Whales. University of Chicago Press, Chicago, IL. pp. 91–126.

Durden, W.N., G. O'Corry-Crowe, S. Shippee, T. Jablonski, S. Rodgers, M. Mazzoil, E. Howells, E. Hartel, B. Potgieter, C. Londono, L. Moreland, F. Townsend, S. McCulloch, and G. Bossart. 2019. Small-scale movement patterns, activity budgets, and association patterns of radio-tagged Indian River Lagoon bottlenose dolphins (*Tursiops truncatus*). Aquatic Mammals 45(1):66–87. doi: 10.1578/am.45.1.2019.66

Durden, W.N., E.D. Stolen, T. Jablonski, L. Moreland, E. Howells, A. Sleeman, M. Denny, G. Biedenbach, and M. Mazzoil. 2021. Abundance and demography of common bottlenose dolphins (*Tursiops truncatus truncatus*) in the Indian River Lagoon, Florida: A robust design capture-recapture analysis. PLoS ONE 16(4):e0250657. doi: 10.1371/journal.pone.0250657

Durden, W.N., E.D. Stolen, T.A. Jablonski, S.A. Puckett, and M.K. Stolen. 2017. Monitoring seasonal abundance of indian river lagoon bottlenose dolphins (*Tursiops truncatus*) using aerial surveys. Aquatic Mammals 43(1):90–112. doi: 10.1578/am.43.1.2017.90

Durden, W.N., E.D. Stolen, and M.K. Stolen. 2011. Abundance, distribution, and group composition of Indian River Lagoon bottlenose dolphins (*Tursiops truncatus*). Aquatic Mammals 37(2):175–186. doi: 10.1578/Am.37.2.2011.175

Durden, W.N., M.K. Stolen, D.H. Adams, and E.D. Stolen. 2007. Mercury and selenium concentrations in stranded bottlenose dolphins from the Indian River Lagoon system, Florida. Bulletin of Marine Science 81(1):37–54.

Fair, P.A., J. Adams, G. Mitchum, T.C. Hulsey, J.S. Reif, M. Houde, D. Muir, E. Wirth, D. Wetzel, E. Zolman, W. McFee, and G.D. Bossart. 2010. Contaminant blubber burdens in Atlantic bottlenose dolphins (*Tursiops truncatus*) from two southeastern US estuarine areas: Concentrations and patterns of PCBs, pesticides, PBDEs, PFCs, and PAHs. Science of the Total Environment 408(7):1577–1597. doi: 10.1016/j.scitotenv.2009.12.021

Fair, P.A., M. Houde, T.C. Hulsey, G.D. Bossart, J. Adams, L. Balthis, and D.C.G. Muir. 2012. Assessment of perfluorinated compounds (PFCs) in plasma of bottlenose dolphins from two southeast US estuarine areas: relationship with age, sex and geographic locations. Marine Pollution Bulletin 64(1):66–74. doi: 10.1016/j.marpolbul.2011.10.022

Fernandez, S. and A.A. Hohn. 1998. Age, growth, and calving season of bottlenose dolphins, *Tursiops truncatus*, off coastal Texas. Fishery Bulletin 96(2):357–365.

Fire, S.E., J.A. Browning, W.N. Durden, and M.K. Stolen. 2020. Comparison of during-bloom and inter-bloom brevetoxin and saxitoxin concentrations in Indian River Lagoon bottlenose dolphins, 2002-2011. Aquatic Toxicolology 218:105371. doi: 10.1016/j.aquatox.2019.105371

Fire, S.E., L.J. Flewelling, M. Stolen, W. Noke Durden, M. de Wit, A.C. Spellman, and Z. Wang. 2015. Brevetoxin-associated mass mortality event of bottlenose dolphins and manatees along the east coast of Florida, USA. Marine Ecology Progress Series 526:241–251. doi: 10.3354/meps11225

Gannon, D.P. and D.M. Waples. 2004. Diets of coastal bottlenose dolphins from the U. S. Mid-Atlantic coast differ by habitat. Marine Mammal Science 20(3):527–545. doi: 10.1111/j.1748-7692.2004.tb01177.x

Greller, R., M. Mazzoli, E. Titcomb, B. Nelson, R. Paperno, and S.H. Markwith. 2020. Environmental drivers of habitat use by common bottlenose dolphins (*Tursiops truncatus*) in the Indian River Lagoon, Florida, USA. Marine Mammal Science 37(2):512–532. doi: 10.1111/mms.12767

Gubbins, C.M., M. Caldwell, S.G. Barco, K.A. Rittmaster, N. Bowles, and V.G. Thayer. 2003. Abundance and sighting patterns of bottlenose dolphins (*Tursiops truncatus*) at four northwest Atlantic coastal sites. Journal of Cetacean Research and Management 5(2):141–148.

Hansen, L.J., L.H. Schwacke, G.B. Mitchum, A.A. Hohn, R.S. Wells, E.S. Zolman, and P.A. Fair. 2004. Geographic variation in polychorinated biphenyl and organochlorine pesticide concentrations in the blubber of bottlenose dolphins from the US Atlantic coast. Science of the Total Environment 319(1-3):147–172. doi: 10.1016/S0048-9697(03)00371-1

Hartel, E.F., W. Noke Durden, and G. O'Corry-Crowe. 2020. Testing satellite telemetry within narrow ecosystems: nocturnal movements and habitat use of bottlenose dolphins within a convoluted estuarine system. Animal Biotelemetry 8(1):13. doi: 10.1186/s40317-020-00200-4

Henderson, E.E. and B. Würsig. 2007. Behavior patterns of bottlenose dolphins in San Luis Pass, Texas. Gulf of Mexico Science 25(2):153–161. doi: 10.18785/goms.2502.06

Hohn, A.A., M.D. Scott, R.S. Wells, J.C. Sweeney, and A.B. Irvine. 1989. Growth layers in teeth from known-age, free-ranging bottlenose dolphins. Marine Mammal Science 5(4):315–342. doi: 10.1111/j.1748-7692.1989.tb00346.x

Kucklick, J., L. Schwacke, R. Wells, A. Hohn, A. Guichard, J. Yordy, L. Hansen, E. Zolman, R. Wilson, J. Litz, D. Nowacek, T. Rowles, R. Pugh, B. Balmer, C. Sinclair, and P. Rosel. 2011. Bottlenose dolphins as indicators of persistent organic pollutants in the western North Atlantic Ocean and Northern Gulf of Mexico. Environmental Science & Technology 45(10):4270–4277. doi: 10.1021/es1042244

LaBrecque, E., C. Curtice, J. Harrison, S.M. Van Parijs, and P.N. Halpin. 2015. 2. Biologically Important Areas for Cetaceans Within U.S. Waters – East Coast Region. Aquatic Mammals 41(1):17–29. doi: 10.1578/am.41.1.2015.17

Leatherwood, S. 1977. Some preliminary impressions on the numbers and social behavior of free-swimming bottlenosed dolphin calves (*Tursiops truncatus*) in the northern Gulf of Mexico. In:

Ridgway, S.H. and K. Bernirschke (Eds.), Breeding Dolphins; Present Status, Suggestions for the Future. Final report to the US Marine Mammal Commission, Contract MM6AC009, MMC-76/07, Washington, DC. pp. 143–167.

Litz, J.A. 2007. Social structure, genetic structure, and persistent organohalogen pollutants in bottlenose dolphins (*Tursiops truncatus*) in Biscayne Bay, Florida. University of Miami. 140 p.

Litz, J.A., L.P. Garrison, L.A. Fieber, A. Martinez, J.P. Contillo, and J.R. Kucklick. 2007. Fine-scale spatial variation of persistent organic pollutants in bottlenose dolphins (*Tursiops truncatus*) in Biscayne Bay, Florida. Environmental Science & Technology 41(21):7222–7228. doi: 10.1021/es070440r

Lynch, K.M., P.A. Fair, M. Houde, D.C.G. Muir, K. Kannan, G.D. Bossart, S.M. Bartell, and M.O. Gribble. 2019. Temporal trends in per- and polyfluoroalkyl substances in bottlenose dolphins (*Tursiops truncatus*) of Indian River Lagoon, Florida and Charleston, South Carolina. Environmental Science & Technology 53(24):14194–14203. doi: 10.1021/acs.est.9b04585

Mann, J., R.C. Connor, L.M. Barre, and M.R. Heithaus. 2000. Female reproductive success in bottlenose dolphins (*Tursiops* sp.): life history, habitat, provisioning, and group-size effects. Behavioral Ecology 11(2):210–219. doi: 10.1093/beheco/11.2.210

Marks, W., S. Burton, E. Stratton, E. Zolman, G. Biedenbach, and A. Page-Karjian. 2020. A case study of monofilament line entanglement in a common bottlenose dolphin (*Tursiops truncatus*): entanglement, disentanglement, and subsequent death. BMC Vet Res 16(1):223. doi: 10.1186/s12917-020-02436-x

Mattson, M.C., K.D. Mullin, G.W. Ingram, and W. Hoggard. 2006. Age structure and growth of the bottlenose dolphin (*Tursiops truncatus*) from strandings in the Mississippi sound region of the north-central Gulf of Mexico from 1986 to 2003. Marine Mammal Science 22(3):654–666. doi: 10.1111/j.1748-7692.2006.00057.x

Mazzoil, M., Q. Gibson, W.N. Durden, R. Borkowski, G. Biedenbach, Z. McKenna, N. Gordon, K. Brightwell, M. Denny, E. Howells, J. Jakush, L. Moreland, A. Perna, G. Pinto, and M. Caldwell. 2020. Spatiotemporal movements of common bottlenose dolphins (*Tursiops truncatus truncatus*) in northeast Florida, USA. Aquatic Mammals 46(3):285–300. doi: 10.1578/am.46.3.2020.285

Mazzoil, M., S.D. McCulloch, and R.H. Defran. 2005. Observations on the site fidelity of bottlenose dolphins (*Tursiops truncatus*) in the Indian River Lagoon, Florida. Florida Scientist 68(4):217–226.

Mazzoil, M., M.E. Murdoch, E. Howells, S.E. Bechdel, M. deSieyes, J.S. Reif, G.D. Bossart, and S.D. McCulloch. 2011. Occurrence, site fidelity, and group size of bottlenose dolphins (*Tursiops truncatus*) along the Atlantic Ocean in Florida, and evaluation of movements into the Indian River Lagoon, Florida. Florida Scientist 74:25–37.

Mazzoil, M., J.S. Reif, M. Youngbluth, M.E. Murdoch, S.E. Bechdel, E. Howells, S.D. McCulloch, L.J. Hansen, and G.D. Bossart. 2008a. Home ranges of bottlenose dolphins (*Tursiops truncatus*) in the Indian River Lagoon, Florida: Environmental correlates and implications for management strategies. Ecohealth 5(3):278–288. doi: 10.1007/s10393-008-0194-9

Mazzoil, M.S., S.D. McCulloch, M.J. Youngbluth, D.S. Kilpatrick, M.E. Murdoch, B. Mase-Guthrie, D.K. Odell, and G.D. Bossart. 2008b. Radio-tracking and survivorship of two rehabilitated bottlenose dolphins (*Tursiops truncatus*) in the Indian River Lagoon, Florida. Aquatic Mammals 34:54–64. doi: 10.1578/AM.34.1.2008.54

McClellan, D.B., J. Browder, J.L. Tobias, G.J. Konoval, M.D. Heron, O.L. Bass, and J. Osborne. 2000. Opportunistic sightings of bottlenose dolphin, *Tursiops truncatus*, along the southeast Florida coast and Florida Bay, 1992-1997. National Oceanic and Atmospheric Administration, National Marine Fisheries Service, Southeast Fisheries Science Center: Miami, FL. NOAA Technical Memorandum NMFS-SEFSC-435. 18 p.

McFee, W.E., T.R. Speakman, L. Balthis, J.D. Adams, and E.S. Zolman. 2014. Reproductive seasonality of a recently designated bottlenose dolphin stock near Charleston, South Carolina, U.S.A. Marine Mammal Science 30(2):528–543. doi: 10.1111/mms.12055

McHugh, M.B. 1989. Population numbers and feeding behavior of the Atlantic bottlenose dolphin (*Tursiops truncatus*) near Aransas Pass, Texas. University of Texas at Austin. Thesis. 96 p.

Mead, J.G. and C.W. Potter. 1990. Natural history of bottlenose dolphins along the central Atlantic coast of the United States. In: Leatherwood, S. and R.R. Reeves (Eds.), The Bottlenose Dolphin. Academic Press, San Diego, CA. pp. 165–195.

Miller, L.J., A.D. Mackey, T. Hoffland, M. Solangi, and S.A. Kuczaj. 2010. Potential effects of a major hurricane on Atlantic bottlenose dolphin (*Tursiops truncatus*) reproduction in the Mississippi Sound. Marine Mammal Science 26(3):707–715. doi: 10.1111/j.1748-7692.2010.00371.x

Miller, L.J., A.D. Mackey, M. Solangi, and S.A. Kuczaj. 2013. Population abundance and habitat utilization of bottlenose dolphins in the Mississippi Sound. Aquatic Conservation: Marine and Freshwater Ecosystems 23(1):145–151. doi: 10.1002/aqc.2278

National Marine Fisheries Service (NMFS). 2022. Active and closed Unusual Mortality Events. https://www.fisheries.noaa.gov/national/marine-life-distress/active-and-closed-unusual-mortality-events

Nekolny, S.R., M. Denny, G. Biedenbach, E.M. Howells, M. Mazzoil, W.N. Durden, L. Moreland, J. David Lambert, and Q.A. Gibson. 2017. Effects of study area size on home range estimates of common bottlenose dolphins *Tursiops truncatus*. Curr Zool 63(6):693–701. doi: 10.1093/cz/zox049

Odell, D.K. and E.D. Asper. 1990. Distribution and movements of freeze-branded bottlenose dolphins in the Indian and Banana Rivers, Florida. In: Leatherwood, S. and R. Reeves (Eds.), The Bottlenose Dolphin. Academic Press, San Diego, CA. pp. 515–540.

Page-Karjian, A., C.F. Lo, B. Ritchie, C.A. Harms, D.S. Rotstein, S. Han, S.M. Hassan, A.F. Lehner, J.P. Buchweitz, V.G. Thayer, J.M. Sullivan, E.F. Christiansen, and J.R. Perrault. 2020. Anthropogenic contaminants and histopathological findings in stranded cetaceans in the southeastern United States, 2012–2018. Frontiers in Marine Science 7:630. doi: 10.3389/fmars.2020.00630

Pate, S.M. and W.E. McFee. 2012. Prey species of bottlenose dolphins (*Tursiops truncatus*) from South Carolina Waters. Southeastern Naturalist 11(1):1–22. doi: 10.1656/058.011.0101

Perrin, W.F. and S.B. Reilly. 1984. Reproductive parameters of dolphins and small whales of the family Delphinidae. In: Perrin, W.F., R.L. Brownell, Jr., and D.P. DeMaster (Eds.), Reproduction in Whales, Dolphins, and Porpoises. pp. 97–134.

Pulster, E.L., K.L. Smalling, E. Zolman, L. Schwacke, and K.A. Maruya. 2009. Persistent organochlorine pollutants and toxaphene congener profiles in bottlenose dolphins (*Tursiops truncatus*) frequenting the Turtle/Brunswick River Estuary, Georgia, USA. Environmental Toxicology and Chemistry 28(7):1390–1399. doi: 10.1897/08-240.1

Reif, J.S., A.M. Schaefer, G.D. Bossart, and P.A. Fair. 2017. Health and Environmental Risk Assessment Project for bottlenose dolphins *Tursiops truncatus* from the southeastern USA. II. Environmental aspects. Diseases of Aquatic Organisms 125(2):155–166. doi: 10.3354/dao03143

Rodríguez-Ferrer, G. 2001. The bottlenose dolphin (*Tursiops truncatus* Montague 1821) of the southwest coast of Puerto Rico: An assessment of its status and habitat use. University of Puerto Rico. Thesis.

Rossman, S., P.H. Ostrom, M. Stolen, N.B. Barros, H. Gandhi, C.A. Stricker, and R.S. Wells. 2015. Individual specialization in the foraging habits of female bottlenose dolphins living in a trophically diverse and habitat rich estuary. Oecologia 178(2):415–425. doi: 10.1007/s00442-015-3241-6

Sarabia, R.E., M.R. Heithaus, and J.J. Kiszka. 2018. Spatial and temporal variation in abundance, group size and behaviour of bottlenose dolphins in the Florida coastal Everglades. Journal of the

Marine Biological Association of the United Kingdom 98(5):1097–1107. doi: 10.1017/s002531541700090x

Schaefer, A.M., H.C. Stavros, G.D. Bossart, P.A. Fair, J.D. Goldstein, and J.S. Reif. 2011. Associations between mercury and hepatic, renal, endocrine, and hematological parameters in Atlantic bottlenose dolphins (*Tursiops truncatus*) along the eastern coast of Florida and South Carolina. Arch Environ Contam Toxicol 61(4):688–695. doi: 10.1007/s00244-011-9651-5

Schaefer, A.M., E.M. Titcomb, P.A. Fair, H.W. Stavros, M. Mazzoil, G.D. Bossart, and J.S. Reif. 2015. Mercury concentrations in Atlantic bottlenose dolphins (*Tursiops truncatus*) inhabiting the Indian River Lagoon, Florida: Patterns of spatial and temporal distribution. Marine Pollution Bulletin 97(1-2):544–547. doi: 10.1016/j.marpolbul.2015.05.007

Schwacke, L.H., E.O. Voit, L.J. Hansen, R.S. Wells, G.B. Mitchum, A.A. Hohn, and P.A. Fair. 2002. Probabilistic risk assessment of reproductive effects of polychlorinated biphenyls on bottlenose dolphins (*Tursiops truncatus*) from the southeast United States coast. Environmental Toxicology and Chemistry 21(12):2752–2764. doi: 10.1002/etc.5620211232

Schwacke, L.H., E.S. Zolman, B.C. Balmer, S. De Guise, R.C. George, J. Hoguet, A.A. Hohn, J.R. Kucklick, S. Lamb, M. Levin, J.A. Litz, W.E. McFee, N.J. Place, F.I. Townsend, R.S. Wells, and T.K. Rowles. 2012. Anaemia, hypothyroidism and immune suppression associated with polychlorinated biphenyl exposure in bottlenose dolphins (*Tursiops truncatus*). Proceedings of the Royal Society B: Biological Sciences 279(1726):48–57. doi: 10.1098/rspb.2011.0665

Sergeant, D.E., D.K. Caldwell, and M.C. Caldwell. 1973. Age, growth, and maturity of bottlenosed dolphin (*Tursiops-truncatus*) from northeast Florida. Journal of the Fisheries Research Board of Canada 30(7):1009–1011. doi: 10.1139/f73-165

Shane, S.H., R.S. Wells, and B. Würsig. 1986. Ecology, behavior and social organization of the bottlenose dolphin: A review. Marine Mammal Science 2:34–63. doi: 10.1111/j.1748-7692.1986.tb00026.x

Silva, D., B. Tramutolo, E. DeSalvio, T. Speakman, and R. Young. 2019. Abundance and movements of the Northern South Carolina Estuarine System Stock of bottlenose dolphins (*Tursiops truncatus*) (USA). Journal of Marine Animals and Their Ecology 11(1):8–18.

Sloan, P.E. 2006. Residency patterns, seasonality and habitat use among bottlenose dolphins, *Tursiops truncatus*, in the Cape Romain National Wildlife Refuge, S.C. University of North Carolina Wilmington. Thesis. 75 p.

Speakman, T., E. Zolman, J. Adams, R.H. Defran, D. Laska, L. Schwacke, J. Craigie, and P. Fair. 2006. Temporal and spatial aspects of bottlenose dolphin occurrence in coastal and estuarine waters near Charleston, South Carolina. National Oceanic and Atmospheric Administration, National Ocean Service, National Centers for Coastal Ocean Science: Charleston, SC. NOAA Technical Memorandum NOS-NCCOS-37. 50 p.

Stavros, H.-C.W., G.D. Bossart, T.C. Hulsey, and P.A. Fair. 2007. Trace element concentrations in skin of free-ranging bottlenose dolphins (*Tursiops truncatus*) from the southeast Atlantic coast. Science of the Total Environment 388(1-3):300–315. doi: 10.1016/j.scitotenv.2007.07.030

Stavros, H.-C.W., G.D. Bossart, T.C. Hulsey, and P.A. Fair. 2008. Trace element concentrations in blood of free-ranging bottlenose dolphins (*Tursiops truncatus*): Influence of age, sex and location. Marine Pollution Bulletin 56(2):371–379. doi: 10.1016/j.marpolbul.2007.10.030

Stavros, H.-C.W., M. Stolen, W.N. Durden, W. McFee, G.D. Bossart, and P.A. Fair. 2011. Correlation and toxicological inference of trace elements in tissues from stranded and free-ranging bottlenose dolphins (*Tursiops truncatus*). Chemosphere 82(11):1649–1661. doi: 10.1016/j.chemosphere.2010.11.019

Stolen, M.K., W.N. Durden, and D.K. Odell. 2007. Historical synthesis of bottlenose dolphin (*Tursiops truncatus*) stranding data in the Indian River Lagoon system, Florida, from 1977–2005. Florida Scientist 70(1):45–54.

Taylor, B.L., S.J. Chivers, J. Larese, and W.F. Perrin. 2007. Generation length and percent mature estimates for IUCN assessments of cetaceans. National Oceanic and Atmospheric Administration, National Marine Fisheries Service, Southwest Fisheries Science Center: La Jolla, CA. Administrative Report LJ-07-01. 24 p.

Thayer, V.G. 2008. Life history parameters and social associations of female bottlenose dolphins (*Tursiops truncatus*) off North Carolina, USA. Duke University. Dissertation. 164 p.

Thayer, V.G., A.J. Read, A.S. Friedlaender, D.R. Colby, A.A. Hohn, W.A. McLellan, D.A. Pabst, J.L. Dearolf, N.I. Bowles, J.R. Russell, and K.A. Rittmaster. 2003. Reproductive seasonality of western Atlantic bottlenose dolphins off North Carolina, USA. Marine Mammal Science 19(4):617–629. doi: 10.1111/j.1748-7692.2003.tb01120.x

Titcomb, E.M., J.S. Reif, P.A. Fair, H.-C.W. Stavros, M. Mazzoil, G.D. Bossart, and A.M. Schaefer. 2017. Blood mercury concentrations in common bottlenose dolphins from the Indian River Lagoon, Florida: Patterns of social distribution. Marine Mammal Science 33(3):771–784. doi: 10.1111/mms.12390

Torres, L.G. 2007. Top predator distribution and foraging ecology in Florida Bay, Florida. Duke University. Dissertation. 225 p.

Torres, L.G. and A.J. Read. 2009. Where to catch a fish? The influence of foraging tactics on the ecology of bottlenose dolphins (*Tursiops truncatus*) in Florida Bay, Florida. Marine Mammal Science 25(4):797–815. doi: 10.1111/j.1748-7692.2009.00297.x

Urian, K.W., D.M. Waples, R.B. Tyson, L.E. Hodge, and A.J. Read. 2013. Abundance of bottlenose dolphins (*Tursiops truncatus*) in estuarine and near-shore waters of North Carolina, USA. Journal of the North Carolina Academy of Science 129(4):165–171. doi: 10.7572/2167-5880-129.4.165

Vollmer, N.L. and P.E. Rosel. 2013. A review of common bottlenose dolphins (*Tursiops truncatus truncatus*) in the Northern Gulf of Mexico: Population biology, potential threats, and management. Southeastern Naturalist 12(m6):1–43. doi: 10.1656/058.012.m601

Waring, G., E. Josephson, K. Maze-Foley, and P. Rosel. 2014. U.S. Atlantic and Gulf of Mexico Marine Mammal Stock Assessments - 2013. National Oceanic and Atmospheric Administration, National Marine Fisheries Service, Northeast Fisheries Science Center: Woods Hole, MA. NOAA Technical Memorandum NMFS-NE-228. 464 p. doi: 10.7289/V51G0J70

Waring, G.T., E. Josephson, K. Maze-Foley, and P.E. Rosel. 2016. U.S. Atlantic and Gulf of Mexico marine mammal stock assessments - 2015. National Oceanic and Atmospheric Administration, National Marine Fisheries Service, Northeast Fisheries Science Center: Woods Hole, MA. NOAA Technical Memorandum NMFS-NE-238. 501 p. doi: 10.7289/V57S7KTN

Wells, R.S. 2000. Reproduction in wild bottlenose dolphins: Overview of patterns observed during a long-term study. In: Duffield, D. and T. Robeck (Eds.), Bottlenose Dolphin Reproduction Workshop. AZA Marine Mammal Taxon Advisory Group, Silver Spring, MD. pp. 57–74.

Wells, R.S. 2003. Dolphin social complexity: Lessons from long-term study and life history. In: de Waal, F.B.M. and P.L. Tyack (Eds.), Animal Social Complexity: Intelligence, Culture, and Individualized Societies Harvard University Press, Cambridge, MA. pp. 32–56.

Wells, R.S. 2014. Social structure and life history of bottlenose dolphins near Sarasota Bay, Florida: Insights from four decades and five generations. Primates and cetaceans. pp. 149–172. doi: 10.1007/978-4-431-54523-1_8

Wells, R.S., A. Natoli, and G. Braulik. 2019. *Tursiops truncatus* (errata version published in 2019). *The IUCN Red List of Threatened Species* 2019: e.T22563A156932432. https://dx.doi.org/10.2305/IUCN.UK.2019-1.RLTS.T22563A156932432.en

Wells, R.S. and M.D. Scott. 1999. Bottlenose dolphin *Tursiops truncatus* (Montagu, 1821). In: Ridgway, S.H. and R. Harrison (Eds.), Handbook of marine mammals. Volume 6: The second book of dolphins and the porpoises. Academic Press, San Diego, CA. pp. 137–182.

Wells, R.S. and M.D. Scott. 2018. Bottlenose dolphin, *Tursiops truncatus*, common bottlenose dolphin. In: Würsig, B., J.G.M. Thewissen, and K.M. Kovacs (Eds.), Encyclopedia of Marine Mammals (Third Edition). Academic Press, London, UK. pp. 118–125. doi: 10.1016/B978-0-12-804327-1.00072-8

Wells, R.S., M.D. Scott, and A.B. Irvine. 1987. The social structure of free-ranging bottlenose dolphins. In: Genoways, H.H. (Ed.), Current Mammalogy. Plenum Press, New York, NY. pp. 247–305.

Wilson, R.M., R.B. Tyson, J.A. Nelson, B.C. Balmer, J.P. Chanton, and D.P. Nowacek. 2017. Niche differentiation and prey selectivity among common bottlenose dolphins (*Tursiops truncatus*) sighted in St. George Sound, Gulf of Mexico. Frontiers in Marine Science 4:235. doi: 10.3389/fmars.2017.00235

Yordy, J.E., M.A. Mollenhauer, R.M. Wilson, R.S. Wells, A. Hohn, J. Sweeney, L.H. Schwacke, T.K. Rowles, J.R. Kucklick, and M.M. Peden-Adams. 2010. Complex contaminant exposure in cetaceans: a comparative E-Screen analysis of bottlenose dolphin blubber and mixtures of four persistent organic pollutants. Environmental Toxicology and Chemistry 29(10):2143–2153. doi: 10.1002/etc.279

Common bottlenose dolphin − *Tursiops truncatus*

Jacksonville Estuarine System Stock

Overall Vulnerability Rank = Very High

Biological Sensitivity = Very High

Climate Exposure = Very High

Data Quality = 93% of scores ≥ 2

	Tursiops truncatus	Expert Scores	Data Quality	Expert Scores Plots (Portion by Category)
Sensitivity attributes	Prey/Diet Specificity	2.0	2.0	
	Habitat Specificity	3.5	1.8	
	Site Fidelity	3.5	2.5	
	Lifetime Reproductive Potential	1.6	2.2	
	Generation Length	2.8	2.2	
	Reproductive Plasticity	1.6	2.0	
	Migration	3.8	2.2	
	Home Range	3.3	2.2	
	Stock Abundance	2.9	2.2	
	Stock Abundance Trend	2.0	1.0	
	Cumulative Stressors	3.2	2.0	
	Sensitivity Score	Very High		
Exposure variables	Sea Surface Temperature (Change in mean)	3.9	2.7	
	Sea Surface Temperature (Change in variability)	1.1	2.7	
	Air Temperature (Change in mean)	3.9	2.7	
	Air Temperature (Change in variability)	1.1	2.7	
	Precipitation (Change in mean)	1.1	2.7	
	Precipitation (Change in variability)	1.1	2.7	
	Sea Surface Salinity (Change in mean)	2.7	2.7	
	Sea Surface Salinity (Change in variability)	1.1	2.7	
	Ocean pH (Change in mean)	3.9	2.7	
	Ocean pH (Change in variability)	1.7	2.7	
	Sea ice coverage (Change in mean)	1.0	3.0	
	Sea ice coverage (Change in variability)	1.0	3.0	
	Dissolved oxygen (Change in mean)	3.9	2.7	
	Dissolved oxygen (Change in variability)	1.6	2.7	
	Circulation	3.1	2.3	
	Sea level rise	3.9	2.7	
	Exposure Score	Very High		
	Overall Vulnerability Rank	Very High		

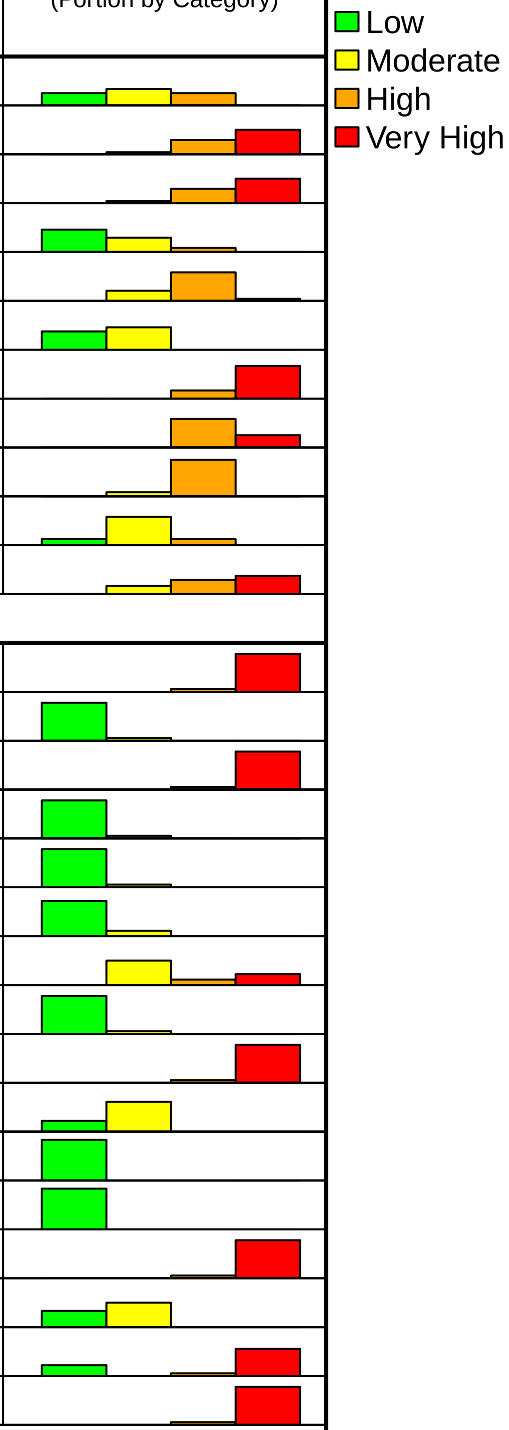

Common bottlenose dolphin (Jacksonville Estuarine System Stock)

Tursiops truncatus

CVA Results Summary

Overall Climate Vulnerability Rank: Very High (100% certainty from bootstrap analysis).

Climate Exposure: Very High. Five exposure factors scored greater than 3.5: Air Temperature (Standard anomaly) (3.93), Dissolved oxygen (Standard anomaly) (3.93), Ocean pH (Standard anomaly) (3.93), Sea level rise (3.93), and Sea Surface Temperature (Standard anomaly) (3.93).

Biological Sensitivity: Very High. Three sensitivity attributes scored greater than 3.5: Migration (3.80), Habitat Specificity (3.55), and Site Fidelity (3.55).

Distributional Response: High

Abundance Response: High

Phenology Response: High

Data Quality: 93% of the data quality scores were 2 or greater, 82% of sensitivity attribute data quality scores were 2 or higher.

Background Information

Stock Description

The Jacksonville Estuarine System (JES) Stock includes common bottlenose dolphins found in estuarine waters of Florida from the Florida/Georgia border at Cumberland Sound in the north to Jacksonville Beach in the south, including St. Mary's, Amelia, Nassau, Fort George, and St. Johns Rivers (Waring et al. 2016).

Prey/Diet Specificity

Generally, common bottlenose dolphins consume a large variety of fish and/or squid (Barros and Odell 1990; Mead and Potter 1990; Wells and Scott 2018). Common bottlenose dolphins seem to show a consistent preference for sound-producing fishes (e.g., sciaenids, toadfish; Berens McCabe et al. 2010; Bowen-Stevens et al. 2021). Estuarine stocks along the southeast U.S. Atlantic coast have been reported to consume a variety of prey, with preference for sciaenids (Gannon and Waples 2004; Pate and McFee 2012). In Sarasota Bay, Florida, Rossman et al. (2015) found some individual common bottlenose dolphins to show diet specialization despite the broader study group showing a generalist diet. Specialization can be influenced by locally abundant prey species (Shane et al. 1986; Vollmer and Rosel 2013) or can result from resource partitioning amongst sympatric populations (e.g., Torres and Read 2009; Wilson et al. 2017).

Habitat Specificity

Common bottlenose dolphins are found in a variety of marine and estuarine habitats (Wells and Scott 2018). The area inhabited by the JES stock is characterized by estuarine tidal marshes and shallow riverine systems with sand, mud or oyster bed substrate (Waring et al. 2016). One river, the St. Johns River, is deep with swift currents (Caldwell 2001). Individuals within this stock have shown variable patterns of preference for different types of habitat, with individuals in the northern areas of the range showing greater preference and individuals in the southern areas showing less preference (Caldwell 2016a, 2016b).

Site Fidelity

Common bottlenose dolphins in the JES comprise several groups with differing site fidelity: one group showed year-round site fidelity, one showed summer site fidelity, and one group showed no long-term site fidelity (Caldwell 2001, 2016a). A separate study also found subgroups with high levels of fidelity to distinct areas of the estuary (Mazzoil et al. 2020). Other estuarine stocks in the western North Atlantic have shown site fidelity (Waring et al. 2016), such as in central Florida (Odell and Asper 1990; Mazzoil et al. 2005, 2008a, 2008b), southern Florida (Litz 2007; Torres 2007; McClellan et al. 2000; Torres 2007; Sarabia et al. 2018), central and southern Georgia (Balmer et al. 2011; Kucklick et al. 2011; Balmer et al. 2013), and South Carolina (Sloan 2006; Speakman et al. 2006; Waring et al. 2016).

Lifetime Reproductive Potential

Taylor et al. (2007) reported the interbirth interval of common bottlenose dolphins as 3.80 years, though other studies have shown it to be closer to three years (Leatherwood 1977; Wells and Scott 1999; Connor et al. 2000; Wells 2003). Thayer (2008) estimated mean interbirth interval of 2.9 years in North Carolina.

Generally, females reach sexual maturity before males, with females reaching sexual maturity at 5–13 years (Sergeant et al. 1973; Perrin and Reilly 1984; Wells et al. 1987; Mead and Potter 1990; Mann et al. 2000; Wells 2000; Wells and Scott 2018).

Females reproduce through most of their adult lives (Leatherwood 1977; Hohn et al. 1989; Wells and Scott 1999; Wells 2000; Wells 2003), but may produce fewer calves after they reach 30 years of age (Wells 2000). Sarasota Bay common bottlenose dolphins can give birth when they are as young as 6 years of age, and can continue until they are 48 years old (Wells and Scott 2018). Reproductive lifespan would therefore be up to 42 years based on long-term observation studies (Wells 2014; Wells and Scott 2018).

Longitudinal studies from Sarasota Bay, Florida have found that female bottlenose dolphins can live up to 67 years (Wells and Scott 2018). Studies based on growth layer groups in teeth suggest female bottlenose dolphins can live to 41 years along the Texas coast (Fernandez and Hohn 1998) and to 30 years along the Mississippi coast (Mattson et al. 2006).

Generation Length

Taylor et al. (2007) reported common bottlenose dolphin generation length of 20.6–21.1 years.

Reproductive Plasticity

Common bottlenose dolphin calving is generally diffusely seasonal, with one or more peaks, although births have been reported from all seasons (Vollmer and Rosel 2013; Wells and Scott 2018). A mid-summer calving peak (July through August) has been reported for bottlenose dolphins off Puerto Rico (Rodríguez-Ferrer 2001). Calf sightings in South Carolina peak in spring and early summer and again in early autumn (McFee et al. 2014). In North Carolina, Thayer et al. (2003) observed a strong birth peak in spring based on stranding data and a diffuse birth peak from late spring to early fall based on sightings. Calf sightings peak in the spring and summer months in Florida (Wells et al. 1987), Mississippi/Louisiana (Miller et al. 2010; Miller et al. 2013), and Texas (McHugh 1989; Henderson and Würsig 2007). Calf sightings in South Carolina peak in spring and early summer and again in early autumn (McFee et al. 2014).

Migration

The estuarine stocks in the western North Atlantic are thought to primarily comprise stable resident communities. However, transient or seasonal movements of some individuals into and out of stock areas are recorded in some areas (LaBrecque et al. 2015; Waring et al. 2016). In the JES, Caldwell (2001) identified one group of dolphins with year-round residency, and two groups that showed movement into and out of the JES.

Other estuarine stocks in the western North Atlantic are known to spend warm months in estuarine waters then move to nearshore waters during cool months (Gubbins et al. 2003; Waring et al. 2014; Waring et al. 2016).

Home Range

The home ranges of individuals within the western North Atlantic estuarine stocks are considered to generally include the areas within the stock boundaries and brief excursions beyond the stock boundaries (LaBrecque et al. 2015; Waring et al. 2016), though studies may underestimate the size of the home range if the survey area is not sufficiently large (Nekolny et al. 2017). In the nearby Indian River Lagoon Estuarine System (IRLES), two radio-tracked rehabilitated dolphins remained within the IRLES until their deaths (100 days and 7 days post-tagging; Mazzoil et al. 2008b). Studies have shown dolphin populations with constrained ranges in Georgia (Brunswick and Sapelo; Balmer et al. 2013); South Carolina (Silva et al. 2019), and North Carolina (Urian et al. 2013). Balmer et al. (2018), using satellite telemetry data, identified that dolphins considered to be members of the South Georgia Estuarine System Stock had different movement patterns with some individuals remaining entirely within the estuarine waters, while other animals use the larger estuarine waters, sounds, and to some degree coastal waters.

Stock Abundance

Caldwell (2001) identified 122 dolphins that were resighted at least 10 times in the JES from 1994 to 1997. Gubbins et al. (2003) estimated the abundance in the JES at 412 individuals (CV=0.06), however did not distinguish between resident and non-resident (Waring et al. 2016).

Stock Abundance Trend

Data are insufficient to complete an abundance trend analysis for this stock (Waring et al. 2016).

Cumulative Stressors

Common bottlenose dolphins face a wide range of individual and cumulative natural and anthropogenic threats (see reviews by Vollmer and Rosel 2013; Wells and Scott 2018; and Wells et al. 2019). These include such threats as shark predation, harmful algal blooms (Brown et al. 2018, recreational and commercial fishing gear, vessel traffic, pollution, habitat alteration, and provisioning.

Common bottlenose dolphins in the JES stock interact with the blue crab trap/pot, stone crab trap/pot, and hook and line fisheries (Waring et al. 2016). Individuals of this stock have been reported stranded, with some strandings showing evidence of human interactions, and other strandings occurring during Unusual Mortality Events (UMEs; Waring et al. 2016; also see NOAA National Marine Mammal Health and Stranding Response Database). For example, during the period 2009–2013, 71 common bottlenose dolphins stranded within the JES, with 18 individuals presenting human interactions such as fishing gear and vessel interactions (Waring et al. 2016).

A UME was declared in the summer of 2013 for the mid-Atlantic coast from New York to Brevard County, Florida, with more than 1500 common bottlenose dolphins stranding as a result of morbillivirus (Waring et al. 2016). Strandings were more prevalent along oceanic shores than in estuaries, suggesting that coastal stocks were more impacted by the UME than estuarine stocks (Waring et al. 2016). In 2010, 23 individuals in the St. Johns River were involved in a UME of undetermined cause (Waring et al. 2016; NMFS 2022).

The nearshore and estuarine habitats along the Atlantic seaboard are adjacent to areas of high human population and some are highly industrialized. Studies of bottlenose dolphin tissues from estuaries in the southeast U.S. have shown persistent organic pollutant (POP; Schwacke et al. 2002, 2012; Hansen et al. 2004; Litz et al. 2007; Pulster et al. 2009; Fair et al. 2010; Yordy et al. 2010; Balmer et al. 2011; Kucklick et al. 2011; Reif et al. 2017) and heavy metal (Durden et al. 2007; Stavros et al. 2007, 2008, 2011; Titcomb et al. 2017; Reif et al. 2017; Page-Karjian et al. 2020) concentrations that may result in adverse effects on health or reproductive rates, particularly in urbanized, developed, and agricultural areas. In Biscayne Bay, Florida, elevated POP concentrations were observed in individuals associated with more polluted areas compared to individuals associated with the less polluted areas of Biscayne Bay, suggesting that exposure to pollutants may be highly variable based on geography (Litz et al. 2007; Kucklick et al. 2011). The JES Stock inhabits a highly urbanized and industrialized area. No published contaminant studies were found for the JES Stock.

Further Reading

Waring, G.T., E. Josephson, K. Maze-Foley, and P.E. Rosel. 2016. U.S. Atlantic and Gulf of Mexico Marine Mammal Stock Assessments - 2015. National Oceanic and Atmospheric Administration, National Marine Fisheries Service, Northeast Fisheries Science Center: Woods Hole, MA. NOAA Technical Memorandum NMFS-NE-238. 501 p. doi: 10.7289/V57S7KTN

Wells, R.S. and M.D. Scott. 2018. Bottlenose Dolphin, *Tursiops truncatus*, Common Bottlenose Dolphin. In: Würsig, B., J.G.M. Thewissen, and K.M. Kovacs (Eds.), Encyclopedia of Marine Mammals

(Third Edition). Academic Press, London, UK. pp. 118–125. doi: 10.1016/B978-0-12-804327-1.00072-8
Wells, R.S., A. Natoli, and G. Braulik. 2019. *Tursiops truncatus* (errata version published in 2019). The IUCN Red List of Threatened Species 2019: e.T22563A156932432. https://dx.doi.org/10.2305/IUCN.UK.2019-1.RLTS.T22563A156932432.en

References

Balmer, B., E. Zolman, T. Rowles, C. Smith, F. Townsend, D. Fauquier, C. George, T. Goldstein, L. Hansen, B. Quigley, W. McFee, J. Morey, P. Rosel, J. Saliki, T. Speakman, and L. Schwacke. 2018. Ranging patterns, spatial overlap, and association with dolphin morbillivirus exposure in common bottlenose dolphins (*Tursiops truncatus*) along the Georgia, USA coast. Ecology and Evolution 8(24):12890–12904. doi: 10.1002/ece3.4727

Balmer, B.C., L.H. Schwacke, R.S. Wells, J.D. Adams, R. Clay George, S.M. Lane, W.A. McLellan, P.E. Rosel, K. Sparks, T. Speakman, E.S. Zolman, and D. Ann Pabst. 2013. Comparison of abundance and habitat usage for common bottlenose dolphins between sites exposed to differential anthropogenic stressors within the estuaries of southern Georgia, U.S.A. Marine Mammal Science 29(2):E114–E135. doi: 10.1111/j.1748-7692.2012.00598.x

Balmer, B.C., L.H. Schwacke, R.S. Wells, R.C. George, J. Hoguet, J.R. Kucklick, S.M. Lane, A. Martinez, W.A. McLellan, P.E. Rosel, T.K. Rowles, K. Sparks, T. Speakman, E.S. Zolman, and D.A. Pabst. 2011. Relationship between persistent organic pollutants (POPs) and ranging patterns in common bottlenose dolphins (*Tursiops truncatus*) from coastal Georgia, USA. Science of the Total Environment 409(11):2094–2101. doi: 10.1016/j.scitotenv.2011.01.052

Barros, N.B. and D.K. Odell. 1990. Food habits of bottlenose dolphins in the southeastern United States In: Leatherwood, S. and R.R. Reeves (Eds.), The Bottlenose Dolphin. Academic Press, San Diego, CA. pp. 309–328. doi: 10.1016/B978-0-12-440280-5.50020-2

Berens McCabe, E.J., D.P. Gannon, N.B. Barros, and R.S. Wells. 2010. Prey selection by resident common bottlenose dolphins (*Tursiops truncatus*) in Sarasota Bay, Florida. Marine Biology 157(5):931–942. doi: 10.1007/s00227-009-1371-2

Bowen-Stevens, S.R., D.P. Gannon, R.A. Hazelkorn, G. Lovewell, K.M. Volker, S. Smith, M.C. Tumlin, and J. Litz. 2021. Diet of common bottlenose dolphins, *Tursiops truncatus*, that stranded in and near Barataria Bay, Louisiana, 2010–2012. Southeastern Naturalist 20(1). doi: 10.1656/058.020.0113

Brown, A., A. Foss, M.A. Miller, and Q. Gibson. 2018. Detection of cyanotoxins (microcystins/nodularins) in livers from estuarine and coastal bottlenose dolphins (*Tursiops truncatus*) from Northeast Florida. Harmful Algae 76:22–34. doi: 10.1016/j.hal.2018.04.011

Caldwell, M. 2001. Social and genetic structure of bottlenose dolphin (*Tursiops truncatus*) in Jacksonville, Florida. University of Miami. 143 p.

Caldwell, M. 2016a. Historical evidence of *Tursiops truncatus* exhibiting habitat preference and seasonal fidelity in Northeast Florida. Aquatic Mammals 42(1):89–103. doi: 10.1578/am.42.1.2016.89

Caldwell, M. 2016b. Historical seasonal density and distribution patterns of *Tursiops truncatus* in Northeast Florida. Aquatic Mammals 42(1):74–88. doi: 10.1578/am.42.1.2016.74

Connor, R.C., R.S. Wells, J. Mann, and A.J. Read. 2000. The bottlenose dolphin: Social relationships in a fission-fusion society. In: Mann, J., R. Connor, P. Tyack, and H. Whitehead (Eds.), Cetacean Societies: Field Studies of Dolphins and Whales. University of Chicago Press, Chicago, IL. pp. 91–126.

Durden, W.N., M.K. Stolen, D.H. Adams, and E.D. Stolen. 2007. Mercury and selenium concentrations in stranded bottlenose dolphins from the Indian River Lagoon system, Florida. Bulletin of Marine Science 81(1):37–54.

Fair, P.A., J. Adams, G. Mitchum, T.C. Hulsey, J.S. Reif, M. Houde, D. Muir, E. Wirth, D. Wetzel, E. Zolman, W. McFee, and G.D. Bossart. 2010. Contaminant blubber burdens in Atlantic bottlenose dolphins (*Tursiops truncatus*) from two southeastern US estuarine areas: Concentrations and patterns of PCBs, pesticides, PBDEs, PFCs, and PAHs. Science of the Total Environment 408(7):1577–1597. doi: 10.1016/j.scitotenv.2009.12.021

Fernandez, S. and A.A. Hohn. 1998. Age, growth, and calving season of bottlenose dolphins, *Tursiops truncatus*, off coastal Texas. Fishery Bulletin 96(2):357–365.

Gannon, D.P. and D.M. Waples. 2004. Diets of coastal bottlenose dolphins from the U. S. Mid-Atlantic coast differ by habitat. Marine Mammal Science 20(3):527–545. doi: 10.1111/j.1748-7692.2004.tb01177.x

Gubbins, C.M., M. Caldwell, S.G. Barco, K.A. Rittmaster, N. Bowles, and V.G. Thayer. 2003. Abundance and sighting patterns of bottlenose dolphins (*Tursiops truncatus*) at four northwest Atlantic coastal sites. Journal of Cetacean Research and Management 5(2):141–148.

Hansen, L.J., L.H. Schwacke, G.B. Mitchum, A.A. Hohn, R.S. Wells, E.S. Zolman, and P.A. Fair. 2004. Geographic variation in polychorinated biphenyl and organochlorine pesticide concentrations in the blubber of bottlenose dolphins from the US Atlantic coast. Science of the Total Environment 319(1-3):147–172. doi: 10.1016/S0048-9697(03)00371-1

Henderson, E.E. and B. Würsig. 2007. Behavior patterns of bottlenose dolphins in San Luis Pass, Texas. Gulf of Mexico Science 25(2):153–161. doi: 10.18785/goms.2502.06

Hohn, A.A., M.D. Scott, R.S. Wells, J.C. Sweeney, and A.B. Irvine. 1989. Growth layers in teeth from known-age, free-ranging bottlenose dolphins. Marine Mammal Science 5(4):315–342. doi: 10.1111/j.1748-7692.1989.tb00346.x

Kucklick, J., L. Schwacke, R. Wells, A. Hohn, A. Guichard, J. Yordy, L. Hansen, E. Zolman, R. Wilson, J. Litz, D. Nowacek, T. Rowles, R. Pugh, B. Balmer, C. Sinclair, and P. Rosel. 2011. Bottlenose dolphins as indicators of persistent organic pollutants in the western North Atlantic Ocean and Northern Gulf of Mexico. Environmental Science & Technology 45(10):4270–4277. doi: 10.1021/es1042244

LaBrecque, E., C. Curtice, J. Harrison, S.M. Van Parijs, and P.N. Halpin. 2015. 2. Biologically Important Areas for Cetaceans Within U.S. Waters – East Coast Region. Aquatic Mammals 41(1):17–29. doi: 10.1578/am.41.1.2015.17

Leatherwood, S. 1977. Some preliminary impressions on the numbers and social behavior of free-swimming bottlenosed dolphin calves (*Tursiops truncatus*) in the northern Gulf of Mexico. In: Ridgway, S.H. and K. Bernirschke (Eds.), Breeding Dolphins; Present Status, Suggestions for the Future. Final report to the US Marine Mammal Commission, Contract MM6AC009, MMC-76/07, Washington, DC. pp. 143–167.

Litz, J.A. 2007. Social structure, genetic structure, and persistent organohalogen pollutants in bottlenose dolphins (*Tursiops truncatus*) in Biscayne Bay, Florida. University of Miami. 140 p.

Litz, J.A., L.P. Garrison, L.A. Fieber, A. Martinez, J.P. Contillo, and J.R. Kucklick. 2007. Fine-scale spatial variation of persistent organic pollutants in bottlenose dolphins (*Tursiops truncatus*) in Biscayne Bay, Florida. Environmental Science & Technology 41(21):7222–7228. doi: 10.1021/es070440r

Mann, J., R.C. Connor, L.M. Barre, and M.R. Heithaus. 2000. Female reproductive success in bottlenose dolphins (*Tursiops* sp.): life history, habitat, provisioning, and group-size effects. Behavioral Ecology 11(2):210–219. doi: 10.1093/beheco/11.2.210

Mattson, M.C., K.D. Mullin, G.W. Ingram, and W. Hoggard. 2006. Age structure and growth of the bottlenose dolphin (*Tursiops truncatus*) from strandings in the Mississippi sound region of the north-central Gulf of Mexico from 1986 to 2003. Marine Mammal Science 22(3):654–666. doi: 10.1111/j.1748-7692.2006.00057.x

Mazzoil, M., Q. Gibson, W.N. Durden, R. Borkowski, G. Biedenbach, Z. McKenna, N. Gordon, K. Brightwell, M. Denny, E. Howells, J. Jakush, L. Moreland, A. Perna, G. Pinto, and M. Caldwell. 2020. Spatiotemporal movements of common bottlenose dolphins (*Tursiops truncatus truncatus*) in northeast Florida, USA. Aquatic Mammals 46(3):285–300. doi: 10.1578/am.46.3.2020.285

Mazzoil, M., S.D. McCulloch, and R.H. Defran. 2005. Observations on the site fidelity of bottlenose dolphins (*Tursiops truncatus*) in the Indian River Lagoon, Florida. Florida Scientist 68(4):217–226.

Mazzoil, M., J.S. Reif, M. Youngbluth, M.E. Murdoch, S.E. Bechdel, E. Howells, S.D. McCulloch, L.J. Hansen, and G.D. Bossart. 2008a. Home ranges of bottlenose dolphins (*Tursiops truncatus*) in the Indian River Lagoon, Florida: Environmental correlates and implications for management strategies. Ecohealth 5(3):278–288. doi: 10.1007/s10393-008-0194-9

Mazzoil, M.S., S.D. McCulloch, M.J. Youngbluth, D.S. Kilpatrick, M.E. Murdoch, B. Mase-Guthrie, D.K. Odell, and G.D. Bossart. 2008b. Radio-tracking and survivorship of two rehabilitated bottlenose dolphins (*Tursiops truncatus*) in the Indian River Lagoon, Florida. Aquatic Mammals 34:54–64. doi: 10.1578/AM.34.1.2008.54

McClellan, D.B., J. Browder, J.L. Tobias, G.J. Konoval, M.D. Heron, O.L. Bass, and J. Osborne. 2000. Opportunistic sightings of bottlenose dolphin, *Tursiops truncatus*, along the southeast Florida coast and Florida Bay, 1992-1997. National Oceanic and Atmospheric Administration, National Marine Fisheries Service, Southeast Fisheries Science Center: Miami, FL. NOAA Technical Memorandum NMFS-SEFSC-435. 18 p.

McFee, W.E., T.R. Speakman, L. Balthis, J.D. Adams, and E.S. Zolman. 2014. Reproductive seasonality of a recently designated bottlenose dolphin stock near Charleston, South Carolina, U.S.A. Marine Mammal Science 30(2):528–543. doi: 10.1111/mms.12055

McHugh, M.B. 1989. Population numbers and feeding behavior of the Atlantic bottlenose dolphin (*Tursiops truncatus*) near Aransas Pass, Texas. University of Texas at Austin. Thesis. 96 p.

Mead, J.G. and C.W. Potter. 1990. Natural history of bottlenose dolphins along the central Atlantic coast of the United States. In: Leatherwood, S. and R.R. Reeves (Eds.), The Bottlenose Dolphin. Academic Press, San Diego, CA. pp. 165–195.

Miller, L.J., A.D. Mackey, T. Hoffland, M. Solangi, and S.A. Kuczaj. 2010. Potential effects of a major hurricane on Atlantic bottlenose dolphin (*Tursiops truncatus*) reproduction in the Mississippi Sound. Marine Mammal Science 26(3):707–715. doi: 10.1111/j.1748-7692.2010.00371.x

Miller, L.J., A.D. Mackey, M. Solangi, and S.A. Kuczaj. 2013. Population abundance and habitat utilization of bottlenose dolphins in the Mississippi Sound. Aquatic Conservation: Marine and Freshwater Ecosystems 23(1):145–151. doi: 10.1002/aqc.2278

National Marine Fisheries Service (NMFS). 2022. Active and closed Unusual Mortality Events. https://www.fisheries.noaa.gov/national/marine-life-distress/active-and-closed-unusual-mortality-events

Nekolny, S.R., M. Denny, G. Biedenbach, E.M. Howells, M. Mazzoil, W.N. Durden, L. Moreland, J. David Lambert, and Q.A. Gibson. 2017. Effects of study area size on home range estimates of common bottlenose dolphins *Tursiops truncatus*. Curr Zool 63(6):693–701. doi: 10.1093/cz/zox049

Odell, D.K. and E.D. Asper. 1990. Distribution and movements of freeze-branded bottlenose dolphins in the Indian and Banana Rivers, Florida. In: Leatherwood, S. and R. Reeves (Eds.), The Bottlenose Dolphin. Academic Press, San Diego, CA. pp. 515–540.

Page-Karjian, A., C.F. Lo, B. Ritchie, C.A. Harms, D.S. Rotstein, S. Han, S.M. Hassan, A.F. Lehner, J.P. Buchweitz, V.G. Thayer, J.M. Sullivan, E.F. Christiansen, and J.R. Perrault. 2020. Anthropogenic contaminants and histopathological findings in stranded cetaceans in the southeastern United States, 2012–2018. Frontiers in Marine Science 7:630. doi: 10.3389/fmars.2020.00630

Pate, S.M. and W.E. McFee. 2012. Prey species of bottlenose dolphins (*Tursiops truncatus*) from South Carolina Waters. Southeastern Naturalist 11(1):1–22. doi: 10.1656/058.011.0101

Perrin, W.F. and S.B. Reilly. 1984. Reproductive parameters of dolphins and small whales of the family Delphinidae. In: Perrin, W.F., R.L. Brownell, Jr., and D.P. DeMaster (Eds.), Reproduction in Whales, Dolphins, and Porpoises. pp. 97–134.

Pulster, E.L., K.L. Smalling, E. Zolman, L. Schwacke, and K.A. Maruya. 2009. Persistent organochlorine pollutants and toxaphene congener profiles in bottlenose dolphins (*Tursiops truncatus*) frequenting the Turtle/Brunswick River Estuary, Georgia, USA. Environmental Toxicology and Chemistry 28(7):1390–1399. doi: 10.1897/08-240.1

Reif, J.S., A.M. Schaefer, G.D. Bossart, and P.A. Fair. 2017. Health and Environmental Risk Assessment Project for bottlenose dolphins *Tursiops truncatus* from the southeastern USA. II. Environmental aspects. Diseases of Aquatic Organisms 125(2):155–166. doi: 10.3354/dao03143

Rodríguez-Ferrer, G. 2001. The bottlenose dolphin (*Tursiops truncatus* Montague 1821) of the southwest coast of Puerto Rico: An assessment of its status and habitat use. University of Puerto Rico. Thesis.

Rossman, S., P.H. Ostrom, M. Stolen, N.B. Barros, H. Gandhi, C.A. Stricker, and R.S. Wells. 2015. Individual specialization in the foraging habits of female bottlenose dolphins living in a trophically diverse and habitat rich estuary. Oecologia 178(2):415–425. doi: 10.1007/s00442-015-3241-6

Sarabia, R.E., M.R. Heithaus, and J.J. Kiszka. 2018. Spatial and temporal variation in abundance, group size and behaviour of bottlenose dolphins in the Florida coastal Everglades. Journal of the Marine Biological Association of the United Kingdom 98(5):1097–1107. doi: 10.1017/s002531541700090x

Schwacke, L.H., E.O. Voit, L.J. Hansen, R.S. Wells, G.B. Mitchum, A.A. Hohn, and P.A. Fair. 2002. Probabilistic risk assessment of reproductive effects of polychlorinated biphenyls on bottlenose dolphins (*Tursiops truncatus*) from the southeast United States coast. Environmental Toxicology and Chemistry 21(12):2752–2764. doi: 10.1002/etc.5620211232

Schwacke, L.H., E.S. Zolman, B.C. Balmer, S. De Guise, R.C. George, J. Hoguet, A.A. Hohn, J.R. Kucklick, S. Lamb, M. Levin, J.A. Litz, W.E. McFee, N.J. Place, F.I. Townsend, R.S. Wells, and T.K. Rowles. 2012. Anaemia, hypothyroidism and immune suppression associated with polychlorinated biphenyl exposure in bottlenose dolphins (*Tursiops truncatus*). Proceedings of the Royal Society B: Biological Sciences 279(1726):48–57. doi: 10.1098/rspb.2011.0665

Sergeant, D.E., D.K. Caldwell, and M.C. Caldwell. 1973. Age, growth, and maturity of bottlenosed dolphin (*Tursiops-truncatus*) from northeast Florida. Journal of the Fisheries Research Board of Canada 30(7):1009–1011. doi: 10.1139/f73-165

Shane, S.H., R.S. Wells, and B. Würsig. 1986. Ecology, behavior and social organization of the bottlenose dolphin: A review. Marine Mammal Science 2:34–63. doi: 10.1111/j.1748-7692.1986.tb00026.x

Silva, D., B. Tramutolo, E. DeSalvio, T. Speakman, and R. Young. 2019. Abundance and movements of the Northern South Carolina Estuarine System Stock of bottlenose dolphins (*Tursiops truncatus*) (USA). Journal of Marine Animals and Their Ecology 11(1):8–18.

Sloan, P.E. 2006. Residency patterns, seasonality and habitat use among bottlenose dolphins, *Tursiops truncatus*, in the Cape Romain National Wildlife Refuge, S.C. University of North Carolina Wilmington. Thesis. 75 p.

Speakman, T., E. Zolman, J. Adams, R.H. Defran, D. Laska, L. Schwacke, J. Craigie, and P. Fair. 2006. Temporal and spatial aspects of bottlenose dolphin occurrence in coastal and estuarine waters near Charleston, South Carolina. National Oceanic and Atmospheric Administration, National Ocean Service, National Centers for Coastal Ocean Science: Charleston, SC. NOAA Technical Memorandum NOS-NCCOS-37. 50 p.

Stavros, H.-C.W., G.D. Bossart, T.C. Hulsey, and P.A. Fair. 2007. Trace element concentrations in skin of free-ranging bottlenose dolphins (*Tursiops truncatus*) from the southeast Atlantic coast. Science of the Total Environment 388(1-3):300–315. doi: 10.1016/j.scitotenv.2007.07.030

Stavros, H.-C.W., G.D. Bossart, T.C. Hulsey, and P.A. Fair. 2008. Trace element concentrations in blood of free-ranging bottlenose dolphins (*Tursiops truncatus*): Influence of age, sex and location. Marine Pollution Bulletin 56(2):371–379. doi: 10.1016/j.marpolbul.2007.10.030

Stavros, H.-C.W., M. Stolen, W.N. Durden, W. McFee, G.D. Bossart, and P.A. Fair. 2011. Correlation and toxicological inference of trace elements in tissues from stranded and free-ranging bottlenose dolphins (*Tursiops truncatus*). Chemosphere 82(11):1649–1661. doi: 10.1016/j.chemosphere.2010.11.019

Taylor, B.L., S.J. Chivers, J. Larese, and W.F. Perrin. 2007. Generation length and percent mature estimates for IUCN assessments of cetaceans. National Oceanic and Atmospheric Administration, National Marine Fisheries Service, Southwest Fisheries Science Center: La Jolla, CA. Administrative Report LJ-07-01. 24 p.

Thayer, V.G. 2008. Life history parameters and social associations of female bottlenose dolphins (*Tursiops truncatus*) off North Carolina, USA. Duke University. Dissertation. 164 p.

Thayer, V.G., A.J. Read, A.S. Friedlaender, D.R. Colby, A.A. Hohn, W.A. McLellan, D.A. Pabst, J.L. Dearolf, N.I. Bowles, J.R. Russell, and K.A. Rittmaster. 2003. Reproductive seasonality of western Atlantic bottlenose dolphins off North Carolina, USA. Marine Mammal Science 19(4):617–629. doi: 10.1111/j.1748-7692.2003.tb01120.x

Titcomb, E.M., J.S. Reif, P.A. Fair, H.-C.W. Stavros, M. Mazzoil, G.D. Bossart, and A.M. Schaefer. 2017. Blood mercury concentrations in common bottlenose dolphins from the Indian River Lagoon, Florida: Patterns of social distribution. Marine Mammal Science 33(3):771–784. doi: 10.1111/mms.12390

Torres, L.G. 2007. Top predator distribution and foraging ecology in Florida Bay, Florida. Duke University. Dissertation. 225 p.

Torres, L.G. and A.J. Read. 2009. Where to catch a fish? The influence of foraging tactics on the ecology of bottlenose dolphins (*Tursiops truncatus*) in Florida Bay, Florida. Marine Mammal Science 25(4):797–815. doi: 10.1111/j.1748-7692.2009.00297.x

Urian, K.W., D.M. Waples, R.B. Tyson, L.E. Hodge, and A.J. Read. 2013. Abundance of bottlenose dolphins (*Tursiops truncatus*) in estuarine and near-shore waters of North Carolina, USA. Journal of the North Carolina Academy of Science 129(4):165–171. doi: 10.7572/2167-5880-129.4.165

Vollmer, N.L. and P.E. Rosel. 2013. A review of common bottlenose dolphins (*Tursiops truncatus truncatus*) in the Northern Gulf of Mexico: Population biology, potential threats, and management. Southeastern Naturalist 12(m6):1–43. doi: 10.1656/058.012.m601

Waring, G., E. Josephson, K. Maze-Foley, and P. Rosel. 2014. U.S. Atlantic and Gulf of Mexico Marine Mammal Stock Assessments - 2013. National Oceanic and Atmospheric Administration, National Marine Fisheries Service, Northeast Fisheries Science Center: Woods Hole, MA. NOAA Technical Memorandum NMFS-NE-228. 464 p. doi: 10.7289/V51G0J70

Waring, G.T., E. Josephson, K. Maze-Foley, and P.E. Rosel. 2016. U.S. Atlantic and Gulf of Mexico marine mammal stock assessments - 2015. National Oceanic and Atmospheric Administration, National Marine Fisheries Service, Northeast Fisheries Science Center: Woods Hole, MA. NOAA Technical Memorandum NMFS-NE-238. 501 p. doi: 10.7289/V57S7KTN

Wells, R.S. 2000. Reproduction in wild bottlenose dolphins: Overview of patterns observed during a long-term study. In: Duffield, D. and T. Robeck (Eds.), Bottlenose Dolphin Reproduction Workshop. AZA Marine Mammal Taxon Advisory Group, Silver Spring, MD. pp. 57–74.

Wells, R.S. 2003. Dolphin social complexity: Lessons from long-term study and life history. In: de Waal, F.B.M. and P.L. Tyack (Eds.), Animal Social Complexity: Intelligence, Culture, and Individualized Societies Harvard University Press, Cambridge, MA. pp. 32–56.

Wells, R.S. 2014. Social structure and life history of bottlenose dolphins near Sarasota Bay, Florida: Insights from four decades and five generations. Primates and cetaceans. pp. 149–172. doi: 10.1007/978-4-431-54523-1_8

Wells, R.S., A. Natoli, and G. Braulik. 2019. *Tursiops truncatus* (errata version published in 2019). *The IUCN Red List of Threatened Species* 2019: e.T22563A156932432. https://dx.doi.org/10.2305/IUCN.UK.2019-1.RLTS.T22563A156932432.en

Wells, R.S. and M.D. Scott. 1999. Bottlenose dolphin *Tursiops truncatus* (Montagu, 1821). In: Ridgway, S.H. and R. Harrison (Eds.), Handbook of marine mammals. Volume 6: The second book of dolphins and the porpoises. Academic Press, San Diego, CA. pp. 137–182.

Wells, R.S. and M.D. Scott. 2018. Bottlenose dolphin, *Tursiops truncatus*, common bottlenose dolphin. In: Würsig, B., J.G.M. Thewissen, and K.M. Kovacs (Eds.), Encyclopedia of Marine Mammals (Third Edition). Academic Press, London, UK. pp. 118–125. doi: 10.1016/B978-0-12-804327-1.00072-8

Wells, R.S., M.D. Scott, and A.B. Irvine. 1987. The social structure of free-ranging bottlenose dolphins. In: Genoways, H.H. (Ed.), Current Mammalogy. Plenum Press, New York, NY. pp. 247–305.

Wilson, R.M., R.B. Tyson, J.A. Nelson, B.C. Balmer, J.P. Chanton, and D.P. Nowacek. 2017. Niche differentiation and prey selectivity among common bottlenose dolphins (*Tursiops truncatus*) sighted in St. George Sound, Gulf of Mexico. Frontiers in Marine Science 4:235. doi: 10.3389/fmars.2017.00235

Yordy, J.E., M.A. Mollenhauer, R.M. Wilson, R.S. Wells, A. Hohn, J. Sweeney, L.H. Schwacke, T.K. Rowles, J.R. Kucklick, and M.M. Peden-Adams. 2010. Complex contaminant exposure in cetaceans: a comparative E-Screen analysis of bottlenose dolphin blubber and mixtures of four persistent organic pollutants. Environmental Toxicology and Chemistry 29(10):2143–2153. doi: 10.1002/etc.279

Common bottlenose dolphin − *Tursiops truncatus*

Northern Georgia/ Southern South Carolina Estuarine System Stock

Overall Vulnerability Rank = Very High

Biological Sensitivity = Very High

Climate Exposure = Very High

Data Quality = 93% of scores ≥ 2

	Tursiops truncatus	Expert Scores	Data Quality	Expert Scores Plots (Portion by Category)
Sensitivity attributes	Prey/Diet Specificity	1.8	2.0	
	Habitat Specificity	3.7	2.0	
	Site Fidelity	4.0	2.7	
	Lifetime Reproductive Potential	1.0	2.3	
	Generation Length	2.7	2.3	
	Reproductive Plasticity	1.2	2.3	
	Migration	3.9	2.7	
	Home Range	3.1	2.7	
	Stock Abundance	3.0	2.3	
	Stock Abundance Trend	2.1	1.7	
	Cumulative Stressors	3.0	2.7	
	Sensitivity Score	Very High		
Exposure variables	Sea Surface Temperature (Change in mean)	4.0	3.0	
	Sea Surface Temperature (Change in variability)	1.0	3.0	
	Air Temperature (Change in mean)	4.0	3.0	
	Air Temperature (Change in variability)	1.0	3.0	
	Precipitation (Change in mean)	1.0	3.0	
	Precipitation (Change in variability)	2.0	3.0	
	Sea Surface Salinity (Change in mean)	2.4	3.0	
	Sea Surface Salinity (Change in variability)	1.0	3.0	
	Ocean pH (Change in mean)	4.0	3.0	
	Ocean pH (Change in variability)	1.0	3.0	
	Sea ice coverage (Change in mean)	1.0	3.0	
	Sea ice coverage (Change in variability)	1.0	3.0	
	Dissolved oxygen (Change in mean)	4.0	3.0	
	Dissolved oxygen (Change in variability)	1.0	3.0	
	Circulation	4.0	1.7	
	Sea level rise	4.0	3.0	
	Exposure Score	Very High		
Overall Vulnerability Rank		Very High		

- Low
- Moderate
- High
- Very High

Common bottlenose dolphin (Northern Georgia/Southern South Carolina Estuarine System Stock)

Tursiops truncatus

CVA Results Summary

Overall Climate Vulnerability Rank: Very High (100% certainty from bootstrap analysis).

Climate Exposure: Very High. Six exposure factors scored greater than 3.5: Air Temperature (Standard anomaly) (4.0), Circulation (4.0), Dissolved oxygen (Standard anomaly) (4.0), Ocean pH (Standard anomaly) (4.0), Sea level rise (4.0), and Sea Surface Temperature (Standard anomaly) (4.0).

Biological Sensitivity: Very High. Three sensitivity attributes scored greater than 3.5: Site Fidelity (4.00), Migration (3.93), and Habitat Specificity (3.67).

Distributional Response: High

Abundance Response: High

Phenology Response: High

Data Quality: 93% of the data quality scores were 2 or greater, 91% of sensitivity attribute data quality scores were 2 or higher.

Background Information

Stock Description

The Northern Georgia/Southern South Carolina Estuarine System (NGSSCES) Stock includes common bottlenose dolphins found in estuarine waters and nearshore waters (out to 1km) of Georgia and South Carolina from the southern extent of the North Edisto River in the north to the northern extent of Ossabaw Sound in the south, including St. Helena, Port Royal, Calibogue and Wassaw Sounds (Waring et al. 2016).

Prey/Diet Specificity

Generally, common bottlenose dolphins consume a large variety of fish and/or squid (Barros and Odell 1990; Mead and Potter 1990; Wells and Scott 2018). Common bottlenose dolphins seem to show a consistent preference for sound-producing fishes (e.g., sciaenids, toadfish; Berens McCabe et al. 2010; Bowen-Stevens et al. 2021). Estuarine stocks along the southeast U.S. Atlantic coast have been reported to consume a variety of prey, with preference for sciaenids (Gannon and Waples 2004; Pate and McFee 2012). Pate and McFee (2012) identified 42 prey species from 24 families in a study of stranded dolphins along the South Carolina coast. In Sarasota Bay, Florida, Rossman et al. (2015) found some individual common bottlenose dolphins to show diet specialization despite the broader study group showing a generalist diet. Specialization can be influenced by locally abundant prey species (Shane et al. 1986; Vollmer and Rosel 2013) or can result from resource partitioning amongst sympatric populations (e.g., Torres and Read 2009; Wilson et al. 2017). Individuals of this stock have been observed strand-feeding

for mullet (*Mugil* sp.) and menhaden (*Brevoortia tyrannus*; Gisburne and Connor 2015). Stable isotope data suggests differences in common bottlenose dolphin forage composition among estuaries along the South Carolina coast and between estuaries and the nearshore coastal populations (Olin et al. 2012).

Habitat Specificity

Common bottlenose dolphins are found in a variety of marine and estuarine habitats (Wells and Scott 2018). The area inhabited by the NGSSCES Stock is characterized by extensive tidal marshes, shallow estuaries, and riverine input (Savannah, Coosawhatchie, Combahee Rivers; Waring et al. 2016).

Site Fidelity

No specific site fidelity information was found for this stock. Other estuarine stocks in the western North Atlantic have shown site fidelity (Waring et al. 2016), such as in South Carolina (Sloan 2006; Speakman et al. 2006; Waring et al. 2016), central and southern Georgia (Balmer et al. 2011; Kucklick et al. 2011; Balmer et al. 2013), northern Florida (Caldwell 2001), central Florida (Odell and Asper 1990; Mazzoil et al. 2005, 2008a, 2008b), and southern Florida (Litz 2007; Torres 2007; McClellan et al. 2000).

Lifetime Reproductive Potential

Taylor et al. (2007) reported the interbirth interval of common bottlenose dolphins as 3.80 years, though other studies have shown it to be closer to three years (Leatherwood 1977; Wells and Scott 1999; Connor et al. 2000; Wells 2003). Thayer (2008) estimated mean interbirth interval of 2.9 years in North Carolina.

Generally, females reach sexual maturity before males, with females reaching sexual maturity at 5–13 years (Sergeant et al. 1973; Perrin and Reilly 1984; Wells et al. 1987; Mead and Potter 1990; Mann et al. 2000; Wells 2000; Wells and Scott 2018).

Females reproduce through most of their adult lives (Leatherwood 1977; Hohn et al. 1989; Wells and Scott 1999; Wells 2000; Wells 2003), but may produce fewer calves after they reach 30 years of age (Wells 2000). Sarasota Bay common bottlenose dolphins can give birth when they are as young as 6 years of age, and can continue until they are 48 years old (Wells and Scott 2018). Reproductive lifespan would therefore be up to 42 years based on long-term observation studies (Wells 2014; Wells and Scott 2018).

Longitudinal studies from Sarasota Bay, Florida have found that female bottlenose dolphins can live up to 67 years (Wells and Scott 2018). Studies based on growth layer groups in teeth suggest female bottlenose dolphins can live to 41 years along the Texas coast (Fernandez and Hohn 1998) and to 30 years along the Mississippi coast (Mattson et al. 2006).

Generation Length

Taylor et al. (2007) reported common bottlenose dolphin generation length of 20.6–21.1 years.

Reproductive Plasticity

Common bottlenose dolphin calving is generally diffusely seasonal, with one or more peaks, although births have been reported from all seasons (Vollmer and Rosel 2013; Wells and Scott 2018). Calf sightings in South Carolina peak in spring and early summer and again in early autumn (McFee et al. 2014). In North Carolina, Thayer et al. (2003) observed a strong birth peak in spring based on stranding data and a diffuse birth peak from late spring to early fall based on sightings.

Migration

The estuarine stocks in the western North Atlantic are thought to primarily comprise stable resident communities. However, transient or seasonal movements of some individuals into and out of stock areas are recorded in some areas (LaBrecque et al. 2015; Waring et al. 2016).

Gubbins et al. (2003) reported seasonal variation in dolphin abundance near Hilton Head, with peaks in abundance during May and July. Taylor et al. (2016) found stronger interannual variation in abundance compared to seasonal variation in abundance in the area around St. Catherine's. Other estuarine stocks in the western North Atlantic are known to spend warm months in estuarine waters then move to nearshore waters during cool months (Gubbins et al. 2003; Durden et al. 2011; Waring et al. 2014; Waring et al. 2016), though some remain entirely in estuarine waters (Mazzoil et al. 2008a).

Home Range

The home ranges of individuals within the western North Atlantic estuarine stocks are considered to generally include the areas within the stock boundaries and brief excursions beyond the stock boundaries (LaBrecque et al. 2015; Waring et al. 2016). Gubbins (2002a, 2002b, 2002c) identified both resident and transient individuals in the area around Hilton Head. Studies have shown dolphin populations with constrained ranges in Georgia (Brunswick and Sapelo; Balmer et al. 2013); South Carolina (Silva et al. 2019), and North Carolina (Urian et al. 2013). Balmer et al. (2018), using satellite telemetry data, identified that dolphins considered to be members of the South Georgia Estuarine System Stock had different movement patterns with some individuals remaining entirely within the estuarine waters, while other animals use the larger estuarine waters, sounds, and to some degree coastal waters.

Stock Abundance

Based on photo-ID data from 1994 to 1998, 234 individually identified dolphins were observed (Gubbins et al. 2003), which included 52 year-round residents and an unspecified number of seasonal residents and transients.

Gubbins et al. (2003) estimated to abundance of common bottlenose dolphins in the area around Hilton head be 525 individuals (CV=0.16) based on mark-recapture analyses; however, this estimate did not differentiate between resident and transient dolphins and did not include the entire range occupied by the NGSSCES Stock.

Stock Abundance Trend

The data are insufficient to evaluate population trends for this stock (Waring et al. 2016).

Cumulative Stressors

Common bottlenose dolphins face a wide range of individual and cumulative natural and anthropogenic threats (see reviews by Vollmer and Rosel 2013; Wells and Scott 2018; and Wells et al. 2019). These include such threats as shark predation, harmful algal blooms, recreational and commercial fishing gear, vessel traffic, pollution, habitat alteration, and provisioning.

Common bottlenose dolphins in the NGSSCES stock interact with the blue crab trap/pot and hook and line fisheries (Waring et al. 2016). Individuals of this stock have been reported stranded, with some strandings showing evidence of human interactions, and other strandings occurring during Unusual Mortality Events (UMEs; Waring et al. 2016; also see NOAA National Marine Mammal Health and Stranding Response Database). During the period 2009–2013, 105 common bottlenose dolphins stranded within the NGSSCES, with 18 individuals presenting human interactions such as fishing gear and vessel interactions (Waring et al. 2016; Seguel et al. 2020). The cause of death for six of the other stranded individuals was likely high concentrations of persistent organic pollutants (POPs; Seguel et al. 2020).

A UME was declared in the summer of 2013 for the mid-Atlantic coast from New York to Brevard County, Florida, with more than 1500 common bottlenose dolphins stranding as a result of morbillivirus (Waring et al. 2016). Strandings were more prevalent along oceanic shores than in estuaries, suggesting that coastal stocks were more impacted by the UME than estuarine stocks (Waring et al. 2016). A UME for bottlenose dolphins was declared in winter and spring of 2011 in South Carolina, during which common bottlenose dolphins stranded at a rate nearly three times the historical average, with evidence suggesting an unusually cold winter and decreased prey abundance contributed to the UME (Waring et al. 2016).

Common bottlenose dolphins stranded in South Carolina show a high prevalence of the gram-negative facultative intracellular bacterium *Brucella ceti* found in the tissues of brain, lung, and other tissues (McFee et al. 2020). Nearly 32% of dolphins tested positive for *B. ceti* over the six year study period, coinciding with the calving period.

The nearshore and estuarine habitats along the Atlantic seaboard are adjacent to areas of high human population and some are highly industrialized. Studies of bottlenose dolphin tissues from estuaries in the southeast U.S. have shown persistent organic pollutant (POP; Schwacke et al. 2002, 2012; Hansen et al. 2004; Litz et al. 2007; Pulster et al. 2009; Fair et al. 2010; Yordy et al. 2010; Balmer et al. 2011; Kucklick et al. 2011; ; Reif et al. 2017) and heavy metal (Durden et al. 2007; Stavros et al. 2007, 2008, 2011; Titcomb et al. 2017; Reif et al. 2017; Page-Karjian et al. 2020) concentrations that may result in adverse effects on health or reproductive rates, particularly in urbanized, developed, and agricultural areas. In Biscayne Bay, Florida, elevated POP concentrations were observed in individuals associated with more polluted areas compared to individuals associated with the less polluted areas of Biscayne Bay, suggesting that exposure to pollutants may be highly variable based on geography (Litz et al. 2007; Kucklick et al. 2011). The NGSSCES Stock inhabits a highly urbanized and industrialized area that includes the large population centers of Savannah, Georgia, and Hilton Head, South Carolina. Analyses of contaminants have found elevated PCB concentrations in dolphins in the Turtle/Brunswick River Estuary relative to dolphins in the Savannah area (Pulster and Maryua 2008; Pulster et al. 2009). Common bottlenose dolphins stranded along the South Carolina coast have been found with ingested microplastics (Battaglia et al. 2020).

Human feeding of dolphins has been observed in the NGSSCES, with possible implications for increased interactions with fishing gear and behavioral adaptation (Powell and Wells 2011; Wu 2013; Kovacs and Cox 2014; Perrtree et al. 2014; Hazelkorn et al. 2016). Vessel strikes, habitat degradation, and altered behavior due to boating activity are additional threats to dolphins in NGSSCES (Richardson et al. 1995; Ketten 1998; Gubbins 2002b, 2003; Mattson et al. 2005).

Further Reading

Waring, G.T., E. Josephson, K. Maze-Foley, and P.E. Rosel. 2016. U.S. Atlantic and Gulf of Mexico Marine Mammal Stock Assessments - 2015. National Oceanic and Atmospheric Administration, National Marine Fisheries Service, Northeast Fisheries Science Center: Woods Hole, MA. NOAA Technical Memorandum NMFS-NE-238. 501 p. doi: 10.7289/V57S7KTN

Wells, R.S. and M.D. Scott. 2018. Bottlenose Dolphin, *Tursiops truncatus*, Common Bottlenose Dolphin. In: Würsig, B., J.G.M. Thewissen, and K.M. Kovacs (Eds.), Encyclopedia of Marine Mammals (Third Edition). Academic Press, London, UK. pp. 118–125. doi: 10.1016/B978-0-12-804327-1.00072-8

Wells, R.S., A. Natoli, and G. Braulik. 2019. *Tursiops truncatus* (errata version published in 2019). The IUCN Red List of Threatened Species 2019: e.T22563A156932432. https://dx.doi.org/10.2305/IUCN.UK.2019-1.RLTS.T22563A156932432.en

References

Balmer, B., E. Zolman, T. Rowles, C. Smith, F. Townsend, D. Fauquier, C. George, T. Goldstein, L. Hansen, B. Quigley, W. McFee, J. Morey, P. Rosel, J. Saliki, T. Speakman, and L. Schwacke. 2018. Ranging patterns, spatial overlap, and association with dolphin morbillivirus exposure in common bottlenose dolphins (*Tursiops truncatus*) along the Georgia, USA coast. Ecology and Evolution 8(24):12890–12904. doi: 10.1002/ece3.4727

Balmer, B.C., L.H. Schwacke, R.S. Wells, J.D. Adams, R. Clay George, S.M. Lane, W.A. McLellan, P.E. Rosel, K. Sparks, T. Speakman, E.S. Zolman, and D. Ann Pabst. 2013. Comparison of abundance and habitat usage for common bottlenose dolphins between sites exposed to differential anthropogenic stressors within the estuaries of southern Georgia, U.S.A. Marine Mammal Science 29(2):E114–E135. doi: 10.1111/j.1748-7692.2012.00598.x

Balmer, B.C., L.H. Schwacke, R.S. Wells, R.C. George, J. Hoguet, J.R. Kucklick, S.M. Lane, A. Martinez, W.A. McLellan, P.E. Rosel, T.K. Rowles, K. Sparks, T. Speakman, E.S. Zolman, and D.A. Pabst. 2011. Relationship between persistent organic pollutants (POPs) and ranging patterns in common bottlenose dolphins (*Tursiops truncatus*) from coastal Georgia, USA. Science of the Total Environment 409(11):2094–2101. doi: 10.1016/j.scitotenv.2011.01.052

Barros, N.B. and D.K. Odell. 1990. Food habits of bottlenose dolphins in the southeastern United States In: Leatherwood, S. and R.R. Reeves (Eds.), The Bottlenose Dolphin. Academic Press, San Diego, CA. pp. 309–328. doi: 10.1016/B978-0-12-440280-5.50020-2

Battaglia, F.M., B.A. Beckingham, and W.E. McFee. 2020. First report from North America of microplastics in the gastrointestinal tract of stranded bottlenose dolphins (*Tursiops truncatus*). Marine Pollution Bulletin 160:111677. doi: 10.1016/j.marpolbul.2020.111677

Berens McCabe, E.J., D.P. Gannon, N.B. Barros, and R.S. Wells. 2010. Prey selection by resident common bottlenose dolphins (*Tursiops truncatus*) in Sarasota Bay, Florida. Marine Biology 157(5):931–942. doi: 10.1007/s00227-009-1371-2

Bowen-Stevens, S.R., D.P. Gannon, R.A. Hazelkorn, G. Lovewell, K.M. Volker, S. Smith, M.C. Tumlin, and J. Litz. 2021. Diet of common bottlenose dolphins, *Tursiops truncatus*, that stranded in and near Barataria Bay, Louisiana, 2010–2012. Southeastern Naturalist 20(1). doi: 10.1656/058.020.0113

Caldwell, M. 2001. Social and genetic structure of bottlenose dolphin (*Tursiops truncatus*) in Jacksonville, Florida. University of Miami. 143 p.

Connor, R.C., R.S. Wells, J. Mann, and A.J. Read. 2000. The bottlenose dolphin: Social relationships in a fission-fusion society. In: Mann, J., R. Connor, P. Tyack, and H. Whitehead (Eds.), Cetacean Societies: Field Studies of Dolphins and Whales. University of Chicago Press, Chicago, IL. pp. 91–126.

Durden, W.N., E.D. Stolen, and M.K. Stolen. 2011. Abundance, distribution, and group composition of Indian River Lagoon bottlenose dolphins (*Tursiops truncatus*). Aquatic Mammals 37(2):175–186. doi: 10.1578/Am.37.2.2011.175

Durden, W.N., M.K. Stolen, D.H. Adams, and E.D. Stolen. 2007. Mercury and selenium concentrations in stranded bottlenose dolphins from the Indian River Lagoon system, Florida. Bulletin of Marine Science 81(1):37–54.

Fair, P.A., J. Adams, G. Mitchum, T.C. Hulsey, J.S. Reif, M. Houde, D. Muir, E. Wirth, D. Wetzel, E. Zolman, W. McFee, and G.D. Bossart. 2010. Contaminant blubber burdens in Atlantic bottlenose dolphins (*Tursiops truncatus*) from two southeastern US estuarine areas: Concentrations and patterns of PCBs, pesticides, PBDEs, PFCs, and PAHs. Science of the Total Environment 408(7):1577–1597. doi: 10.1016/j.scitotenv.2009.12.021

Fernandez, S. and A.A. Hohn. 1998. Age, growth, and calving season of bottlenose dolphins, *Tursiops truncatus*, off coastal Texas. Fishery Bulletin 96(2):357–365.

Gannon, D.P. and D.M. Waples. 2004. Diets of coastal bottlenose dolphins from the U. S. Mid-Atlantic coast differ by habitat. Marine Mammal Science 20(3):527–545. doi: 10.1111/j.1748-7692.2004.tb01177.x

Gisburne, T.J. and R.C. Connor. 2015. Group size and feeding success in strand-feeding bottlenose dolphins (*Tursiops truncatus*) in Bull Creek, South Carolina. Marine Mammal Science 31(3):1252–1257. doi: 10.1111/mms.12207

Gubbins, C. 2002a. Use of home ranges by resident bottlenose dolphins (*Tursiops truncatus*) in a South Carolina estuary. Journal of Mammalogy 83(1):178–187. doi: 10.1644/1545-1542(2002)083<0178:Uohrbr>2.0.Co;2

Gubbins, C.M. 2002b. Association patterns of resident bottlenose dolphins (*Tursiops truncatus*) in a South Carolina estuary. Aquatic Mammals 28:24–31.

Gubbins, C.M. 2002c. The dolphins of Hilton Head: Their natural history. University of South Carolina Press, Columbia, SC. 96 p. doi: 10.2307/j.ctv1htpdsv

Gubbins, C.M., M. Caldwell, S.G. Barco, K.A. Rittmaster, N. Bowles, and V.G. Thayer. 2003. Abundance and sighting patterns of bottlenose dolphins (*Tursiops truncatus*) at four northwest Atlantic coastal sites. Journal of Cetacean Research and Management 5(2):141–148.

Hansen, L.J., L.H. Schwacke, G.B. Mitchum, A.A. Hohn, R.S. Wells, E.S. Zolman, and P.A. Fair. 2004. Geographic variation in polychorinated biphenyl and organochlorine pesticide concentrations in the blubber of bottlenose dolphins from the US Atlantic coast. Science of the Total Environment 319(1-3):147–172. doi: 10.1016/S0048-9697(03)00371-1

Hazelkorn, R.A., B.A. Schulte, and T.M. Cox. 2016. Persistent effects of begging on common bottlenose dolphin (*Tursiops truncatus*) behavior in an estuarine population. Aquatic Mammals 42(4):531–541. doi: 10.1578/am.42.4.2016.531

Hohn, A.A., M.D. Scott, R.S. Wells, J.C. Sweeney, and A.B. Irvine. 1989. Growth layers in teeth from known-age, free-ranging bottlenose dolphins. Marine Mammal Science 5(4):315–342. doi: 10.1111/j.1748-7692.1989.tb00346.x

Ketten, D.R. 1998. Marine mammal auditory systems: A summary of audiometric and anatomical data and its implications for underwater acoustic impacts. National Oceanic and Atmospheric Administration, National Marine Fisheries Service, Southwest Fisheries Science Center: La Jolla, CA. NOAA Technical Memorandum NMFS-SWFSC-256. 74 p.

Kovacs, C. and T. Cox. 2014. Quantification of interactions between common bottlenose dolphins (*Tursiops truncatus*) and a commercial shrimp trawler near Savannah, Georgia. Aquatic Mammals 40(1):81–94. doi: 10.1578/Am.40.1.2014.81

Kucklick, J., L. Schwacke, R. Wells, A. Hohn, A. Guichard, J. Yordy, L. Hansen, E. Zolman, R. Wilson, J. Litz, D. Nowacek, T. Rowles, R. Pugh, B. Balmer, C. Sinclair, and P. Rosel. 2011. Bottlenose dolphins as indicators of persistent organic pollutants in the western North Atlantic Ocean and Northern Gulf of Mexico. Environmental Science & Technology 45(10):4270–4277. doi: 10.1021/es1042244

LaBrecque, E., C. Curtice, J. Harrison, S.M. Van Parijs, and P.N. Halpin. 2015. 2. Biologically Important Areas for Cetaceans Within U.S. Waters – East Coast Region. Aquatic Mammals 41(1):17–29. doi: 10.1578/am.41.1.2015.17

Leatherwood, S. 1977. Some preliminary impressions on the numbers and social behavior of free-swimming bottlenosed dolphin calves (*Tursiops truncatus*) in the northern Gulf of Mexico. In: Ridgway, S.H. and K. Bernirschke (Eds.), Breeding Dolphins; Present Status, Suggestions for the Future. Final report to the US Marine Mammal Commission, Contract MM6AC009, MMC-76/07, Washington, DC. pp. 143–167.

Litz, J.A. 2007. Social structure, genetic structure, and persistent organohalogen pollutants in bottlenose dolphins (*Tursiops truncatus*) in Biscayne Bay, Florida. University of Miami. 140 p.

Litz, J.A., L.P. Garrison, L.A. Fieber, A. Martinez, J.P. Contillo, and J.R. Kucklick. 2007. Fine-scale spatial variation of persistent organic pollutants in bottlenose dolphins (*Tursiops truncatus*) in Biscayne Bay, Florida. Environmental Science & Technology 41(21):7222–7228. doi: 10.1021/es070440r

Mann, J., R.C. Connor, L.M. Barre, and M.R. Heithaus. 2000. Female reproductive success in bottlenose dolphins (*Tursiops* sp.): life history, habitat, provisioning, and group-size effects. Behavioral Ecology 11(2):210–219. doi: 10.1093/beheco/11.2.210

Mattson, M.C., K.D. Mullin, G.W. Ingram, and W. Hoggard. 2006. Age structure and growth of the bottlenose dolphin (*Tursiops truncatus*) from strandings in the Mississippi sound region of the north-central Gulf of Mexico from 1986 to 2003. Marine Mammal Science 22(3):654–666. doi: 10.1111/j.1748-7692.2006.00057.x

Mattson, M.C., J.A. Thomas, and D. St. Aubin. 2005. Effects of boat activity on the behavior of bottlenose dolphins (*Tursiops truncatus*) in waters surrounding Hilton Head Island, South Carolina. Aquatic Mammals 31(1):133–140. doi: 10.1578/am.31.1.2005.133

Mazzoil, M., S.D. McCulloch, and R.H. Defran. 2005. Observations on the site fidelity of bottlenose dolphins (*Tursiops truncatus*) in the Indian River Lagoon, Florida. Florida Scientist 68(4):217–226.

Mazzoil, M., J.S. Reif, M. Youngbluth, M.E. Murdoch, S.E. Bechdel, E. Howells, S.D. McCulloch, L.J. Hansen, and G.D. Bossart. 2008a. Home ranges of bottlenose dolphins (*Tursiops truncatus*) in the Indian River Lagoon, Florida: Environmental correlates and implications for management strategies. Ecohealth 5(3):278–288. doi: 10.1007/s10393-008-0194-9

Mazzoil, M.S., S.D. McCulloch, M.J. Youngbluth, D.S. Kilpatrick, M.E. Murdoch, B. Mase-Guthrie, D.K. Odell, and G.D. Bossart. 2008b. Radio-tracking and survivorship of two rehabilitated bottlenose dolphins (*Tursiops truncatus*) in the Indian River Lagoon, Florida. Aquatic Mammals 34:54–64. doi: 10.1578/AM.34.1.2008.54

McClellan, D.B., J. Browder, J.L. Tobias, G.J. Konoval, M.D. Heron, O.L. Bass, and J. Osborne. 2000. Opportunistic sightings of bottlenose dolphin, *Tursiops truncatus*, along the southeast Florida coast and Florida Bay, 1992-1997. National Oceanic and Atmospheric Administration, National

Marine Fisheries Service, Southeast Fisheries Science Center: Miami, FL. NOAA Technical Memorandum NMFS-SEFSC-435. 18 p.

McFee, W.E., T.R. Speakman, L. Balthis, J.D. Adams, and E.S. Zolman. 2014. Reproductive seasonality of a recently designated bottlenose dolphin stock near Charleston, South Carolina, U.S.A. Marine Mammal Science 30(2):528–543. doi: 10.1111/mms.12055

McFee, W.E., D. Wu, K. Colegrove, K. Terio, L. Balthis, and R. Young. 2020. Occurrence of *Brucella ceti* in stranded bottlenose dolphins *Tursiops truncatus* coincides with calving season. Diseases of Aquatic Organisms 141:185–193. doi: 10.3354/dao03526

Mead, J.G. and C.W. Potter. 1990. Natural history of bottlenose dolphins along the central Atlantic coast of the United States. In: Leatherwood, S. and R.R. Reeves (Eds.), The Bottlenose Dolphin. Academic Press, San Diego, CA. pp. 165–195.

Odell, D.K. and E.D. Asper. 1990. Distribution and movements of freeze-branded bottlenose dolphins in the Indian and Banana Rivers, Florida. In: Leatherwood, S. and R. Reeves (Eds.), The Bottlenose Dolphin. Academic Press, San Diego, CA. pp. 515–540.

Olin, J.A., P.A. Fair, M.A. Recks, E. Zolman, J. Adams, and A.T. Fisk. 2012. Unique seasonal forage bases within a local population of bottlenose dolphin (*Tursiops truncatus*). Marine Mammal Science 28(1):E28–E40. doi: 10.1111/j.1748-7692.2011.00470.x

Page-Karjian, A., C.F. Lo, B. Ritchie, C.A. Harms, D.S. Rotstein, S. Han, S.M. Hassan, A.F. Lehner, J.P. Buchweitz, V.G. Thayer, J.M. Sullivan, E.F. Christiansen, and J.R. Perrault. 2020. Anthropogenic contaminants and histopathological findings in stranded cetaceans in the southeastern United States, 2012–2018. Frontiers in Marine Science 7:630. doi: 10.3389/fmars.2020.00630

Pate, S.M. and W.E. McFee. 2012. Prey species of bottlenose dolphins (*Tursiops truncatus*) from South Carolina Waters. Southeastern Naturalist 11(1):1–22. doi: 10.1656/058.011.0101

Perrin, W.F. and S.B. Reilly. 1984. Reproductive parameters of dolphins and small whales of the family Delphinidae. In: Perrin, W.F., R.L. Brownell, Jr., and D.P. DeMaster (Eds.), Reproduction in Whales, Dolphins, and Porpoises. pp. 97–134.

Perrtree, R.M., C.J. Kovacs, and T.M. Cox. 2014. Standardization and application of metrics to quantify human-interaction behaviors by the bottlenose dolphin (*Tursiops* spp.). Marine Mammal Science 30(4):1320–1334. doi: 10.1111/mms.12114

Powell, J.R. and R.S. Wells. 2011. Recreational fishing depredation and associated behaviors involving common bottlenose dolphins (*Tursiops truncatus*) in Sarasota Bay, Florida. Marine Mammal Science 27(1):111–129. doi: 10.1111/j.1748-7692.2010.00401.x

Pulster, E.L. and K.A. Maruya. 2008. Geographic specificity of Aroclor 1268 in bottlenose dolphins (*Tursiops truncatus*) frequenting the Turtle/Brunswick River Estuary, Georgia (USA). Science of the Total Environment 393(2-3):367–375. doi: 10.1016/j.scitotenv.2007.12.031

Pulster, E.L., K.L. Smalling, E. Zolman, L. Schwacke, and K.A. Maruya. 2009. Persistent organochlorine pollutants and toxaphene congener profiles in bottlenose dolphins (*Tursiops truncatus*) frequenting the Turtle/Brunswick River Estuary, Georgia, USA. Environmental Toxicology and Chemistry 28(7):1390–1399. doi: 10.1897/08-240.1

Reif, J.S., A.M. Schaefer, G.D. Bossart, and P.A. Fair. 2017. Health and Environmental Risk Assessment Project for bottlenose dolphins *Tursiops truncatus* from the southeastern USA. II. Environmental aspects. Diseases of Aquatic Organisms 125(2):155–166. doi: 10.3354/dao03143

Richardson, W.J., C.R. Greene, Jr., C.I. Malme, and D. Thomas, II. 1995. Marine mammals and noise. Academic Press, San Diego, CA. 576 p.

Rossman, S., P.H. Ostrom, M. Stolen, N.B. Barros, H. Gandhi, C.A. Stricker, and R.S. Wells. 2015. Individual specialization in the foraging habits of female bottlenose dolphins living in a trophically diverse and habitat rich estuary. Oecologia 178(2):415–425. doi: 10.1007/s00442-015-3241-6

Schwacke, L.H., E.O. Voit, L.J. Hansen, R.S. Wells, G.B. Mitchum, A.A. Hohn, and P.A. Fair. 2002. Probabilistic risk assessment of reproductive effects of polychlorinated biphenyls on bottlenose dolphins (*Tursiops truncatus*) from the southeast United States coast. Environmental Toxicology and Chemistry 21(12):2752–2764. doi: 10.1002/etc.5620211232

Schwacke, L.H., E.S. Zolman, B.C. Balmer, S. De Guise, R.C. George, J. Hoguet, A.A. Hohn, J.R. Kucklick, S. Lamb, M. Levin, J.A. Litz, W.E. McFee, N.J. Place, F.I. Townsend, R.S. Wells, and T.K. Rowles. 2012. Anaemia, hypothyroidism and immune suppression associated with polychlorinated biphenyl exposure in bottlenose dolphins (*Tursiops truncatus*). Proceedings of the Royal Society B: Biological Sciences 279(1726):48–57. doi: 10.1098/rspb.2011.0665

Seguel, M., R.C. George, G. Maboni, S. Sanchez, A. Page-Karjian, E. Wirth, W. McFee, and N.L. Gottdenker. 2020. Pathologic findings and causes of death in bottlenose dolphins *Tursiops truncatus* stranded along the Georgia coast, USA (2007-2013). Diseases of Aquatic Organisms 141:25–38. doi: 10.3354/dao03509

Sergeant, D.E., D.K. Caldwell, and M.C. Caldwell. 1973. Age, growth, and maturity of bottlenosed dolphin (*Tursiops-truncatus*) from northeast Florida. Journal of the Fisheries Research Board of Canada 30(7):1009–1011. doi: 10.1139/f73-165

Shane, S.H., R.S. Wells, and B. Würsig. 1986. Ecology, behavior and social organization of the bottlenose dolphin: A review. Marine Mammal Science 2:34–63. doi: 10.1111/j.1748-7692.1986.tb00026.x

Silva, D., B. Tramutolo, E. DeSalvio, T. Speakman, and R. Young. 2019. Abundance and movements of the Northern South Carolina Estuarine System Stock of bottlenose dolphins (*Tursiops truncatus*) (USA). Journal of Marine Animals and Their Ecology 11(1):8–18.

Sloan, P.E. 2006. Residency patterns, seasonality and habitat use among bottlenose dolphins, *Tursiops truncatus*, in the Cape Romain National Wildlife Refuge, S.C. University of North Carolina Wilmington. Thesis. 75 p.

Speakman, T., E. Zolman, J. Adams, R.H. Defran, D. Laska, L. Schwacke, J. Craigie, and P. Fair. 2006. Temporal and spatial aspects of bottlenose dolphin occurrence in coastal and estuarine waters near Charleston, South Carolina. National Oceanic and Atmospheric Administration, National Ocean Service, National Centers for Coastal Ocean Science: Charleston, SC. NOAA Technical Memorandum NOS-NCCOS-37. 50 p.

Stavros, H.-C.W., G.D. Bossart, T.C. Hulsey, and P.A. Fair. 2007. Trace element concentrations in skin of free-ranging bottlenose dolphins (*Tursiops truncatus*) from the southeast Atlantic coast. Science of the Total Environment 388(1-3):300–315. doi: 10.1016/j.scitotenv.2007.07.030

Stavros, H.-C.W., G.D. Bossart, T.C. Hulsey, and P.A. Fair. 2008. Trace element concentrations in blood of free-ranging bottlenose dolphins (*Tursiops truncatus*): Influence of age, sex and location. Marine Pollution Bulletin 56(2):371–379. doi: 10.1016/j.marpolbul.2007.10.030

Stavros, H.-C.W., M. Stolen, W.N. Durden, W. McFee, G.D. Bossart, and P.A. Fair. 2011. Correlation and toxicological inference of trace elements in tissues from stranded and free-ranging bottlenose dolphins (*Tursiops truncatus*). Chemosphere 82(11):1649–1661. doi: 10.1016/j.chemosphere.2010.11.019

Taylor, A.R., J.H. Schacke, T.R. Speakman, S.B. Castleberry, and R.B. Chandler. 2016. Factors related to common bottlenose dolphin (*Tursiops truncatus*) seasonal migration along South Carolina and Georgia coasts, USA. Animal Migration 3(1):14–26. doi: 10.1515/ami-2016-0002

Taylor, B.L., S.J. Chivers, J. Larese, and W.F. Perrin. 2007. Generation length and percent mature estimates for IUCN assessments of cetaceans. National Oceanic and Atmospheric Administration, National Marine Fisheries Service, Southwest Fisheries Science Center: La Jolla, CA. Administrative Report LJ-07-01. 24 p.

Thayer, V.G. 2008. Life history parameters and social associations of female bottlenose dolphins (*Tursiops truncatus*) off North Carolina, USA. Duke University. Dissertation. 164 p.

Thayer, V.G., A.J. Read, A.S. Friedlaender, D.R. Colby, A.A. Hohn, W.A. McLellan, D.A. Pabst, J.L. Dearolf, N.I. Bowles, J.R. Russell, and K.A. Rittmaster. 2003. Reproductive seasonality of western Atlantic bottlenose dolphins off North Carolina, USA. Marine Mammal Science 19(4):617–629. doi: 10.1111/j.1748-7692.2003.tb01120.x

Titcomb, E.M., J.S. Reif, P.A. Fair, H.-C.W. Stavros, M. Mazzoil, G.D. Bossart, and A.M. Schaefer. 2017. Blood mercury concentrations in common bottlenose dolphins from the Indian River Lagoon, Florida: Patterns of social distribution. Marine Mammal Science 33(3):771–784. doi: 10.1111/mms.12390

Torres, L.G. 2007. Top predator distribution and foraging ecology in Florida Bay, Florida. Duke University. Dissertation. 225 p.

Torres, L.G. and A.J. Read. 2009. Where to catch a fish? The influence of foraging tactics on the ecology of bottlenose dolphins (*Tursiops truncatus*) in Florida Bay, Florida. Marine Mammal Science 25(4):797–815. doi: 10.1111/j.1748-7692.2009.00297.x

Urian, K.W., D.M. Waples, R.B. Tyson, L.E. Hodge, and A.J. Read. 2013. Abundance of bottlenose dolphins (*Tursiops truncatus*) in estuarine and near-shore waters of North Carolina, USA. Journal of the North Carolina Academy of Science 129(4):165–171. doi: 10.7572/2167-5880-129.4.165

Vollmer, N.L. and P.E. Rosel. 2013. A review of common bottlenose dolphins (*Tursiops truncatus truncatus*) in the Northern Gulf of Mexico: Population biology, potential threats, and management. Southeastern Naturalist 12(m6):1–43. doi: 10.1656/058.012.m601

Waring, G., E. Josephson, K. Maze-Foley, and P. Rosel. 2014. U.S. Atlantic and Gulf of Mexico Marine Mammal Stock Assessments - 2013. National Oceanic and Atmospheric Administration, National Marine Fisheries Service, Northeast Fisheries Science Center: Woods Hole, MA. NOAA Technical Memorandum NMFS-NE-228. 464 p. doi: 10.7289/V51G0J70

Waring, G.T., E. Josephson, K. Maze-Foley, and P.E. Rosel. 2016. U.S. Atlantic and Gulf of Mexico marine mammal stock assessments - 2015. National Oceanic and Atmospheric Administration, National Marine Fisheries Service, Northeast Fisheries Science Center: Woods Hole, MA. NOAA Technical Memorandum NMFS-NE-238. 501 p. doi: 10.7289/V57S7KTN

Wells, R.S. 2000. Reproduction in wild bottlenose dolphins: Overview of patterns observed during a long-term study. In: Duffield, D. and T. Robeck (Eds.), Bottlenose Dolphin Reproduction Workshop. AZA Marine Mammal Taxon Advisory Group, Silver Spring, MD. pp. 57–74.

Wells, R.S. 2003. Dolphin social complexity: Lessons from long-term study and life history. In: de Waal, F.B.M. and P.L. Tyack (Eds.), Animal Social Complexity: Intelligence, Culture, and Individualized Societies Harvard University Press, Cambridge, MA. pp. 32–56.

Wells, R.S. 2014. Social structure and life history of bottlenose dolphins near Sarasota Bay, Florida: Insights from four decades and five generations. Primates and cetaceans. pp. 149–172. doi: 10.1007/978-4-431-54523-1_8

Wells, R.S., A. Natoli, and G. Braulik. 2019. *Tursiops truncatus* (errata version published in 2019). *The IUCN Red List of Threatened Species* 2019: e.T22563A156932432. https://dx.doi.org/10.2305/IUCN.UK.2019-1.RLTS.T22563A156932432.en

Wells, R.S. and M.D. Scott. 1999. Bottlenose dolphin *Tursiops truncatus* (Montagu, 1821). In: Ridgway, S.H. and R. Harrison (Eds.), Handbook of marine mammals. Volume 6: The second book of dolphins and the porpoises. Academic Press, San Diego, CA. pp. 137–182.

Wells, R.S. and M.D. Scott. 2018. Bottlenose dolphin, *Tursiops truncatus*, common bottlenose dolphin. In: Würsig, B., J.G.M. Thewissen, and K.M. Kovacs (Eds.), Encyclopedia of Marine Mammals (Third Edition). Academic Press, London, UK. pp. 118–125. doi: 10.1016/B978-0-12-804327-1.00072-8

Wells, R.S., M.D. Scott, and A.B. Irvine. 1987. The social structure of free-ranging bottlenose dolphins. In: Genoways, H.H. (Ed.), Current Mammalogy. Plenum Press, New York, NY. pp. 247–305.

Wilson, R.M., R.B. Tyson, J.A. Nelson, B.C. Balmer, J.P. Chanton, and D.P. Nowacek. 2017. Niche differentiation and prey selectivity among common bottlenose dolphins (*Tursiops truncatus*) sighted in St. George Sound, Gulf of Mexico. Frontiers in Marine Science 4:235. doi: 10.3389/fmars.2017.00235

Wu, C. 2013. Human and boat interactions with common bottlenose dolphins (*Tursiops truncatus*) in the waterways around Savannah, Georgia. Savannah State University. Thesis. 101 p.

Yordy, J.E., M.A. Mollenhauer, R.M. Wilson, R.S. Wells, A. Hohn, J. Sweeney, L.H. Schwacke, T.K. Rowles, J.R. Kucklick, and M.M. Peden-Adams. 2010. Complex contaminant exposure in cetaceans: a comparative E-Screen analysis of bottlenose dolphin blubber and mixtures of four persistent organic pollutants. Environmental Toxicology and Chemistry 29(10):2143–2153. doi: 10.1002/etc.279

Common bottlenose dolphin − *Tursiops truncatus*

Northern North Carolina Estuarine System Stock

Overall Vulnerability Rank = Very High

Biological Sensitivity = Very High

Climate Exposure = Very High

Data Quality = 100% of scores≥ 2

	Tursiops truncatus	Expert Scores	Data Quality	Expert Scores Plots (Portion by Category)
Sensitivity attributes	Prey/Diet Specificity	2.4	2.5	
	Habitat Specificity	3.4	2.0	
	Site Fidelity	3.9	2.0	
	Lifetime Reproductive Potential	1.3	2.5	
	Generation Length	3.0	2.5	
	Reproductive Plasticity	1.5	2.5	
	Migration	3.9	2.5	
	Home Range	3.7	2.0	
	Stock Abundance	3.0	3.0	
	Stock Abundance Trend	2.0	3.0	
	Cumulative Stressors	3.1	2.0	
	Sensitivity Score	Very High		
Exposure variables	Sea Surface Temperature (Change in mean)	4.0	2.5	
	Sea Surface Temperature (Change in variability)	1.0	2.5	
	Air Temperature (Change in mean)	4.0	2.5	
	Air Temperature (Change in variability)	1.0	2.5	
	Precipitation (Change in mean)	1.0	2.5	
	Precipitation (Change in variability)	1.5	2.5	
	Sea Surface Salinity (Change in mean)	3.3	2.5	
	Sea Surface Salinity (Change in variability)	1.4	2.5	
	Ocean pH (Change in mean)	4.0	2.5	
	Ocean pH (Change in variability)	1.0	2.5	
	Sea ice coverage (Change in mean)	1.0	2.5	
	Sea ice coverage (Change in variability)	1.0	2.5	
	Dissolved oxygen (Change in mean)	4.0	2.5	
	Dissolved oxygen (Change in variability)	1.0	2.5	
	Circulation	3.8	2.5	
	Sea level rise	4.0	2.5	
	Exposure Score	Very High		
	Overall Vulnerability Rank	Very High		

Low
Moderate
High
Very High

Common bottlenose dolphin (Northern North Carolina Estuarine System Stock)

Tursiops truncatus

CVA Results Summary

Overall Climate Vulnerability Rank: Very High (100% certainty from bootstrap analysis).

Climate Exposure: Very High. Six exposure factors scored greater than 3.5: Air Temperature (Standard anomaly) (4.0), Dissolved oxygen (Standard anomaly) (4.0), Ocean pH (Standard anomaly) (4.0), Sea level rise (4.0), Sea Surface Temperature (Standard anomaly) (4.0), and Circulation (3.8).

Biological Sensitivity: Very High. Three sensitivity attributes score greater than 3.5: Migration (3.9), Site Fidelity (3.9), and Home Range (3.7).

Distributional Response: High

Abundance Response: High

Phenology Response: High

Data Quality: 100 % of the data quality scores were 2 or greater, 100 % of sensitivity attribute data quality scores were 2 or higher.

Background Information

Stock Description

The Northern North Carolina Estuarine System (NNCES) Stock includes common bottlenose dolphins found in waters of the Pamlico Sound estuarine system and nearshore coastal waters (out to 1km) of North Carolina from Beaufort north to southern Virginia and the lower Chesapeake Bay (Hayes et al. 2021). During colder water months, many animals move out of the estuaries and occupy coastal waters (≤3 km from shore) between the New River and Oregon Inlet, North Carolina.

Prey/Diet Specificity

Generally, common bottlenose dolphins consume a large variety of fish and/or squid (Barros and Odell 1990; Mead and Potter 1990; Wells and Scott 2018). Common bottlenose dolphins seem to show a consistent preference for sound-producing fishes (e.g., sciaenids, toadfish; Berens McCabe et al. 2010; Bowen-Stevens et al. 2021). Estuarine stocks along the southeast U.S. Atlantic coast have been reported to consume a variety of prey, with preference for sciaenids (Gannon and Waples 2004; Pate and McFee 2012). Pate and McFee (2012) identified 42 prey species from 24 families in a study of stranded dolphins along the South Carolina coast. In Sarasota Bay, Florida, Rossman et al. (2015) found some individual common bottlenose dolphins to show diet specialization despite the broader study group showing a generalist diet. Specialization can be influenced by locally abundant prey species (Shane et al. 1986; Vollmer and Rosel 2013) or can result from resource partitioning amongst sympatric populations (e.g., Torres and Read 2009; Wilson et al. 2017).

Habitat Specificity

Common bottlenose dolphins are found in a variety of marine and estuarine habitats (Wells and Scott 2018). The area inhabited by the NNCES Stock is characterized by tidal marshes, estuarine waters, and shallow open water (Hayes et al. 2021).

Site Fidelity

No specific site fidelity information was found for this stock. Other estuarine stocks in the western North Atlantic have shown site fidelity (Hayes et al. 2021), such as in South Carolina (Sloan 2006; Speakman et al. 2006; Hayes et al. 2021), central and southern Georgia (Balmer et al. 2011; Kucklick et al. 2011; Balmer et al. 2013), northern Florida (Caldwell 2001), central Florida coast (Odell and Asper 1990; Mazzoil et al. 2005, 2008a, 2008b), and southern Florida (Litz 2007; Torres 2007; McClellan et al. 2000). The site fidelity shown in other estuarine stocks suggests this stock may also show site fidelity.

Lifetime Reproductive Potential

Taylor et al. (2007) reported the interbirth interval of common bottlenose dolphins as 3.80 years, though other studies have shown it to be closer to three years (Leatherwood 1977; Wells and Scott 1999; Connor et al. 2000; Wells 2003). Thayer (2008) estimated mean interbirth interval of 2.9 years in North Carolina.

Generally, females reach sexual maturity before males, with females reaching sexual maturity at 5–13 years (Sergeant et al. 1973; Perrin and Reilly 1984; Wells et al. 1987; Mead and Potter 1990; Mann et al. 2000; Wells 2000; Wells and Scott 2018).

Females reproduce through most of their adult lives (Leatherwood 1977; Hohn et al. 1989; Wells and Scott 1999; Wells 2000; Wells 2003), but may produce fewer calves after they reach 30 years of age (Wells 2000). Sarasota Bay common bottlenose dolphins can give birth when they are as young as 6 years of age, and can continue until they are 48 years old (Wells and Scott 2018). Reproductive lifespan would therefore be up to 42 years based on long-term observation studies (Wells 2014; Wells and Scott 2018).

Longitudinal studies from Sarasota Bay, Florida have found that female bottlenose dolphins can live up to 67 years (Wells and Scott 2018). Studies based on growth layer groups in teeth suggest female bottlenose dolphins can live to 41 years along the Texas coast (Fernandez and Hohn 1998) and to 30 years along the Mississippi coast (Mattson et al. 2006).

Generation Length

Taylor et al. (2007) reported common bottlenose dolphin generation length of 20.6–21.1 years.

Reproductive Plasticity

Common bottlenose dolphin calving is generally diffusely seasonal, with one or more peaks, although births have been reported from all seasons (Vollmer and Rosel 2013; Wells and Scott 2018). In North Carolina, Thayer et al. (2003) observed a strong birth peak in spring based on stranding data and a

diffuse birth peak from late spring to early fall based on sightings. Calf sightings in the Charleston Estuarine System Stock peak in spring and early summer and again in early autumn (McFee et al. 2014).

Migration

The estuarine stocks in the western North Atlantic are thought to primarily comprise stable resident communities. However, transient or seasonal movements of some individuals into and out of stock areas are recorded in some areas (LaBrecque et al. 2015; Hayes et al. 2021).

Some of the NNCES Stock moves out of Pamlico Sound during colder water months to occupy coastal waters (< 3km from shore) between the New River and Cape Hatteras (Goodman Hall et al. 2013; Hayes et al. 2021). Individuals of the NNCES Stock that are found in Chesapeake Bay show a similar seasonal pattern with a peak in sightings during the summer (Rodriguez et al. 2021).

Home Range

The home ranges of individuals within the western North Atlantic estuarine stocks are considered to generally include the areas within the stock boundaries and brief excursions beyond the stock boundaries (LaBrecque et al. 2015; Hayes et al. 2021). Individuals of this stock are known to move as far north as Virginia Beach and Chesapeake Bay, as far south as New River, North Carolina and into the coastal waters off of Cape Hatteras (Hayes et al. 2021; also see Hansen and Wells 1996; Cortese 2000; NMFS 2001; Read et al. 2003; Goodman Hall et al. 2013). Studies have shown dolphin populations with constrained ranges in Georgia (Brunswick and Sapelo; Balmer et al. 2013); South Carolina (Silva et al. 2019), and North Carolina (Urian et al. 2013). Balmer et al. (2018), using satellite telemetry data, identified that dolphins considered to be members of the South Georgia Estuarine System Stock had different movement patterns with some individuals remaining entirely within the estuarine waters, while other animals use the larger estuarine waters, sounds, and to some degree coastal waters.

Stock Abundance

The best available abundance estimate for the NNCES stock is 823 individuals (CV=0.06) based on photo-ID mark-recapture surveys in summer 2013 (Gorgone et al. 2014). The survey did not cover the entire stock’s range (i.e., coastal waters), thus, the estimate may be negatively biased.

Stock Abundance Trend

A trend analysis has not been conducted for this stock (Hayes et al. 2021), however Gorgone et al. (2014) noted that the 2013 abundance estimate was similar to estimates using different methods from 2006 (Urian et al. 2013) and 2000 (Read et al. 2003).

Cumulative Stressors

Common bottlenose dolphins face a wide range of individual and cumulative natural and anthropogenic threats (see reviews by Vollmer and Rosel 2013; Wells and Scott 2018; and Wells et al. 2019). These include such threats as shark predation, harmful algal blooms, recreational and commercial fishing gear (Goodman Hall et al. 2013; Byrd et al. 2014; Byrd and Hohn 2017), vessel traffic, pollution, habitat alteration, and provisioning.

Common bottlenose dolphins in the NNCES Stock interact with several commercial fisheries. These include gillnet, long haul seine, haul/beach seine, pound net, roe mullet stop net, blue crab trap/pot, and hook and line fisheries. The minimum total mean annual human-caused mortality and serious injury for fisheries and other human causes ranged between 7.2 and 30.0 dolphins annually during 2014–2018 (Hayes et al. 2021). During 2014–2018, 480 common bottlenose dolphins stranded that could be considered part of the NNCES, with 66 individuals presenting evidence of human interactions such as fishing gear and vessel interactions (Hayes et al. 2021; also see NOAA National Marine Mammal Health and Stranding Response Database).

An Unusual Mortality Event (UME) was declared in the summer of 2013 for the mid-Atlantic coast from New York to Brevard County, Florida, with more than 1600 common bottlenose dolphins stranding as a result of morbillivirus (Hayes et al. 2021). Strandings were more prevalent along oceanic shores than in estuaries, suggesting that coastal stocks were more impacted by the UME than estuarine stocks (Hayes et al. 2021). In 2009, 40 individuals in Virginia were involved in a UME of undetermined cause (NMFS 2022).

The nearshore and estuarine habitats along the Atlantic seaboard are adjacent to areas of high human population and some are highly industrialized. Studies of bottlenose dolphin tissues from estuaries in the southeast U.S. have shown persistent organic pollutant (POP; Schwacke et al. 2002, 2012; Hansen et al. 2004; Litz et al. 2007; Pulster et al. 2009; Fair et al. 2010; Yordy et al. 2010; Balmer et al. 2011; Kucklick et al. 2011; ; Reif et al. 2017) and heavy metal (Durden et al. 2007; Stavros et al. 2007, 2008, 2011; Titcomb et al. 2017; Reif et al. 2017; Page-Karjian et al. 2020) concentrations that may result in adverse effects on health or reproductive rates, particularly in urbanized, developed, and agricultural areas. In Biscayne Bay, Florida, elevated POP concentrations were observed in individuals associated with more polluted areas compared to individuals associated with the less polluted areas of Biscayne Bay, suggesting that exposure to pollutants may be highly variable based on geography (Litz et al. 2007; Kucklick et al. 2011). The NNCES Stock inhabits an area that receives runoff from agricultural, industrial, and urban sources. Analyses of common bottlenose dolphins around the Beaufort, NC area have found elevated levels of POPs(Schwacke et al. 2002; Hansen et al. 2004). Individuals from this stock have been found to have detectable concentrations of lead, mercury, and selenium, though at lower levels than the Indian River Lagoon Estuarine System in Florida (Page-Karjian et al. 2020).

Further Reading

Hayes, S.A., E. Josephson, K. Maze-Foley, P.E. Rosel, and J. Turek. 2021. US Atlantic and Gulf of Mexico Marine Mammal Stock Assessments - 2020. National Oceanic and Atmospheric Administration, National Marine Fisheries Service, Northeast Fisheries Science Center: Woods Hole, MA. NOAA Technical Memorandum NMFS-NE-271. 394 p.

Wells, R.S. and M.D. Scott. 2018. Bottlenose Dolphin, *Tursiops truncatus*, Common Bottlenose Dolphin. In: Würsig, B., J.G.M. Thewissen, and K.M. Kovacs (Eds.), Encyclopedia of Marine Mammals (Third Edition). Academic Press, London, UK. pp. 118–125. doi: 10.1016/B978-0-12-804327-1.00072-8

Wells, R.S., A. Natoli, and G. Braulik. 2019. *Tursiops truncatus* (errata version published in 2019). The IUCN Red List of Threatened Species 2019: e.T22563A156932432. https://dx.doi.org/10.2305/IUCN.UK.2019-1.RLTS.T22563A156932432.en

References

Balmer, B., E. Zolman, T. Rowles, C. Smith, F. Townsend, D. Fauquier, C. George, T. Goldstein, L. Hansen, B. Quigley, W. McFee, J. Morey, P. Rosel, J. Saliki, T. Speakman, and L. Schwacke. 2018. Ranging patterns, spatial overlap, and association with dolphin morbillivirus exposure in common bottlenose dolphins (*Tursiops truncatus*) along the Georgia, USA coast. Ecology and Evolution 8(24):12890–12904. doi: 10.1002/ece3.4727

Balmer, B.C., L.H. Schwacke, R.S. Wells, J.D. Adams, R. Clay George, S.M. Lane, W.A. McLellan, P.E. Rosel, K. Sparks, T. Speakman, E.S. Zolman, and D. Ann Pabst. 2013. Comparison of abundance and habitat usage for common bottlenose dolphins between sites exposed to differential anthropogenic stressors within the estuaries of southern Georgia, U.S.A. Marine Mammal Science 29(2):E114–E135. doi: 10.1111/j.1748-7692.2012.00598.x

Balmer, B.C., L.H. Schwacke, R.S. Wells, R.C. George, J. Hoguet, J.R. Kucklick, S.M. Lane, A. Martinez, W.A. McLellan, P.E. Rosel, T.K. Rowles, K. Sparks, T. Speakman, E.S. Zolman, and D.A. Pabst. 2011. Relationship between persistent organic pollutants (POPs) and ranging patterns in common bottlenose dolphins (*Tursiops truncatus*) from coastal Georgia, USA. Science of the Total Environment 409(11):2094–2101. doi: 10.1016/j.scitotenv.2011.01.052

Barros, N.B. and D.K. Odell. 1990. Food habits of bottlenose dolphins in the southeastern United States In: Leatherwood, S. and R.R. Reeves (Eds.), The Bottlenose Dolphin. Academic Press, San Diego, CA. pp. 309–328. doi: 10.1016/B978-0-12-440280-5.50020-2

Berens McCabe, E.J., D.P. Gannon, N.B. Barros, and R.S. Wells. 2010. Prey selection by resident common bottlenose dolphins (*Tursiops truncatus*) in Sarasota Bay, Florida. Marine Biology 157(5):931–942. doi: 10.1007/s00227-009-1371-2

Bowen-Stevens, S.R., D.P. Gannon, R.A. Hazelkorn, G. Lovewell, K.M. Volker, S. Smith, M.C. Tumlin, and J. Litz. 2021. Diet of common bottlenose dolphins, *Tursiops truncatus*, that stranded in and near Barataria Bay, Louisiana, 2010–2012. Southeastern Naturalist 20(1). doi: 10.1656/058.020.0113

Byrd, B.L. and A.A. Hohn. 2017. Differential risk of bottlenose dolphin (*Tursiops truncatus*) bycatch in North Carolina, USA. Aquatic Mammals 43(5):558–569. doi: 10.1578/Am.43.5.2017.558

Byrd, B.L., A.A. Hohn, G.N. Lovewell, K.M. Altman, S.G. Barco, A. Friedlaender, C.A. Harms, W.A. McLellan, K.T. Moore, P.E. Rosel, and V.G. Thayer. 2014. Strandings as indicators of marine mammal biodiversity and human interactions off the coast of North Carolina. Fishery Bulletin 112(1):1–23. doi: 10.7755/fb.112.1.1

Caldwell, M. 2001. Social and genetic structure of bottlenose dolphin (*Tursiops truncatus*) in Jacksonville, Florida. University of Miami. 143 p.

Connor, R.C., R.S. Wells, J. Mann, and A.J. Read. 2000. The bottlenose dolphin: Social relationships in a fission-fusion society. In: Mann, J., R. Connor, P. Tyack, and H. Whitehead (Eds.), Cetacean Societies: Field Studies of Dolphins and Whales. University of Chicago Press, Chicago, IL. pp. 91–126.

Cortese, N.A. 2000. Delineation of bottlenose dolphin populations in the western Atlantic Ocean using stable isotopes. University of Virginia. Thesis. 118 p.

Durden, W.N., M.K. Stolen, D.H. Adams, and E.D. Stolen. 2007. Mercury and selenium concentrations in stranded bottlenose dolphins from the Indian River Lagoon system, Florida. Bulletin of Marine Science 81(1):37–54.

Fair, P.A., J. Adams, G. Mitchum, T.C. Hulsey, J.S. Reif, M. Houde, D. Muir, E. Wirth, D. Wetzel, E. Zolman, W. McFee, and G.D. Bossart. 2010. Contaminant blubber burdens in Atlantic bottlenose dolphins (*Tursiops truncatus*) from two southeastern US estuarine areas: Concentrations and patterns of PCBs, pesticides, PBDEs, PFCs, and PAHs. Science of the Total Environment 408(7):1577–1597. doi: 10.1016/j.scitotenv.2009.12.021

Fernandez, S. and A.A. Hohn. 1998. Age, growth, and calving season of bottlenose dolphins, *Tursiops truncatus*, off coastal Texas. Fishery Bulletin 96(2):357–365.

Gannon, D.P. and D.M. Waples. 2004. Diets of coastal bottlenose dolphins from the U. S. Mid-Atlantic coast differ by habitat. Marine Mammal Science 20(3):527–545. doi: 10.1111/j.1748-7692.2004.tb01177.x

Goodman Hall, A., J. Braun McNeill, P.B. Conn, E. Davenport, and A.A. Hohn. 2013. Seasonal co-occurrence of sea turtles, bottlenose dolphins, and commercial gill nets in southern Pamlico and northern Core Sounds, and adjacent coastal waters of North Carolina, USA. Endangered Species Research 22(3):235–249. doi: 10.3354/esr00539

Gorgone, A.M., T. Eguchi, B.L. Byrd, K.M. Altman, and A.A. Hohn. 2014. Estimating the abundance of the Northern North Carolina Estuarine System Stock of common bottlenose dolphins (*Tursiops truncatus*). National Oceanic and Atmospheric Administration, National Marine Fisheries Service, Southeast Fisheries Science Center, NOAA Beaufort Laboratory: Beaufort, NC. NOAA Technical Memorandum NMFS-SEFSC-664. 22 p. doi: 10.7289/V5VH5KS8

Hansen, L.J., L.H. Schwacke, G.B. Mitchum, A.A. Hohn, R.S. Wells, E.S. Zolman, and P.A. Fair. 2004. Geographic variation in polychorinated biphenyl and organochlorine pesticide concentrations in the blubber of bottlenose dolphins from the US Atlantic coast. Science of the Total Environment 319(1-3):147–172. doi: 10.1016/S0048-9697(03)00371-1

Hansen, L.J. and R.S. Wells. 1996. Bottlenose dolphin health assessment: Field report on sampling near Beaufort, North Carolina, during July, 1995. National Oceanic and Atmospheric Administration, National Marine Fisheries Service, Southeast Fisheries Science Center, Charleston Laboratory: Charleston, SC. NOAA Technical Memorandum NMFS-SEFSC-382. 24 p.

Hayes, S.A., E. Josephson, K. Maze-Foley, P.E. Rosel, and J. Turek. 2021. US Atlantic and Gulf of Mexico marine mammal stock assessments - 2020. National Oceanic and Atmospheric Administration, National Marine Fisheries Service, Northeast Fisheries Science Center: Woods Hole, MA. NOAA Technical Memorandum NMFS-NE-271. 394 p.

Hohn, A.A., M.D. Scott, R.S. Wells, J.C. Sweeney, and A.B. Irvine. 1989. Growth layers in teeth from known-age, free-ranging bottlenose dolphins. Marine Mammal Science 5(4):315–342. doi: 10.1111/j.1748-7692.1989.tb00346.x

Kucklick, J., L. Schwacke, R. Wells, A. Hohn, A. Guichard, J. Yordy, L. Hansen, E. Zolman, R. Wilson, J. Litz, D. Nowacek, T. Rowles, R. Pugh, B. Balmer, C. Sinclair, and P. Rosel. 2011. Bottlenose dolphins as indicators of persistent organic pollutants in the western North Atlantic Ocean and Northern Gulf of Mexico. Environmental Science & Technology 45(10):4270–4277. doi: 10.1021/es1042244

LaBrecque, E., C. Curtice, J. Harrison, S.M. Van Parijs, and P.N. Halpin. 2015. 2. Biologically Important Areas for Cetaceans Within U.S. Waters – East Coast Region. Aquatic Mammals 41(1):17–29. doi: 10.1578/am.41.1.2015.17

Leatherwood, S. 1977. Some preliminary impressions on the numbers and social behavior of free-swimming bottlenosed dolphin calves (*Tursiops truncatus*) in the northern Gulf of Mexico. In: Ridgway, S.H. and K. Bernirschke (Eds.), Breeding Dolphins; Present Status, Suggestions for the Future. Final report to the US Marine Mammal Commission, Contract MM6AC009, MMC-76/07, Washington, DC. pp. 143–167.

Litz, J.A. 2007. Social structure, genetic structure, and persistent organohalogen pollutants in bottlenose dolphins (*Tursiops truncatus*) in Biscayne Bay, Florida. University of Miami. 140 p.

Litz, J.A., L.P. Garrison, L.A. Fieber, A. Martinez, J.P. Contillo, and J.R. Kucklick. 2007. Fine-scale spatial variation of persistent organic pollutants in bottlenose dolphins (*Tursiops truncatus*) in Biscayne Bay, Florida. Environmental Science & Technology 41(21):7222–7228. doi: 10.1021/es070440r

Mann, J., R.C. Connor, L.M. Barre, and M.R. Heithaus. 2000. Female reproductive success in bottlenose dolphins (*Tursiops* sp.): life history, habitat, provisioning, and group-size effects. Behavioral Ecology 11(2):210–219. doi: 10.1093/beheco/11.2.210

Mattson, M.C., K.D. Mullin, G.W. Ingram, and W. Hoggard. 2006. Age structure and growth of the bottlenose dolphin (*Tursiops truncatus*) from strandings in the Mississippi sound region of the north-central Gulf of Mexico from 1986 to 2003. Marine Mammal Science 22(3):654–666. doi: 10.1111/j.1748-7692.2006.00057.x

Mazzoil, M., S.D. McCulloch, and R.H. Defran. 2005. Observations on the site fidelity of bottlenose dolphins (*Tursiops truncatus*) in the Indian River Lagoon, Florida. Florida Scientist 68(4):217–226.

Mazzoil, M., J.S. Reif, M. Youngbluth, M.E. Murdoch, S.E. Bechdel, E. Howells, S.D. McCulloch, L.J. Hansen, and G.D. Bossart. 2008a. Home ranges of bottlenose dolphins (*Tursiops truncatus*) in the Indian River Lagoon, Florida: Environmental correlates and implications for management strategies. Ecohealth 5(3):278–288. doi: 10.1007/s10393-008-0194-9

Mazzoil, M.S., S.D. McCulloch, M.J. Youngbluth, D.S. Kilpatrick, M.E. Murdoch, B. Mase-Guthrie, D.K. Odell, and G.D. Bossart. 2008b. Radio-tracking and survivorship of two rehabilitated bottlenose dolphins (*Tursiops truncatus*) in the Indian River Lagoon, Florida. Aquatic Mammals 34:54–64. doi: 10.1578/AM.34.1.2008.54

McClellan, D.B., J. Browder, J.L. Tobias, G.J. Konoval, M.D. Heron, O.L. Bass, and J. Osborne. 2000. Opportunistic sightings of bottlenose dolphin, *Tursiops truncatus*, along the southeast Florida coast and Florida Bay, 1992-1997. National Oceanic and Atmospheric Administration, National Marine Fisheries Service, Southeast Fisheries Science Center: Miami, FL. NOAA Technical Memorandum NMFS-SEFSC-435. 18 p.

McFee, W.E., T.R. Speakman, L. Balthis, J.D. Adams, and E.S. Zolman. 2014. Reproductive seasonality of a recently designated bottlenose dolphin stock near Charleston, South Carolina, U.S.A. Marine Mammal Science 30(2):528–543. doi: 10.1111/mms.12055

Mead, J.G. and C.W. Potter. 1990. Natural history of bottlenose dolphins along the central Atlantic coast of the United States. In: Leatherwood, S. and R.R. Reeves (Eds.), The Bottlenose Dolphin. Academic Press, San Diego, CA. pp. 165–195.

National Marine Fisheries Service (NMFS). 2001. Preliminary stock structure of coastal bottlenose dolphins along the Atlantic coast of the US. NMFS/SEFSC Report prepared for the Bottlenose Dolphin Take Reduction Team. National Oceanic and Atmospheric Administration, National Marine Fisheries Service, Southeast Fisheries Science Center. 41 p.

National Marine Fisheries Service (NMFS). 2022. Active and closed Unusual Mortality Events. https://www.fisheries.noaa.gov/national/marine-life-distress/active-and-closed-unusual-mortality-events

Odell, D.K. and E.D. Asper. 1990. Distribution and movements of freeze-branded bottlenose dolphins in the Indian and Banana Rivers, Florida. In: Leatherwood, S. and R. Reeves (Eds.), The Bottlenose Dolphin. Academic Press, San Diego, CA. pp. 515–540.

Page-Karjian, A., C.F. Lo, B. Ritchie, C.A. Harms, D.S. Rotstein, S. Han, S.M. Hassan, A.F. Lehner, J.P. Buchweitz, V.G. Thayer, J.M. Sullivan, E.F. Christiansen, and J.R. Perrault. 2020. Anthropogenic contaminants and histopathological findings in stranded cetaceans in the southeastern United States, 2012–2018. Frontiers in Marine Science 7:630. doi: 10.3389/fmars.2020.00630

Pate, S.M. and W.E. McFee. 2012. Prey species of bottlenose dolphins (*Tursiops truncatus*) from South Carolina Waters. Southeastern Naturalist 11(1):1–22. doi: 10.1656/058.011.0101

Perrin, W.F. and S.B. Reilly. 1984. Reproductive parameters of dolphins and small whales of the family Delphinidae. In: Perrin, W.F., R.L. Brownell, Jr., and D.P. DeMaster (Eds.), Reproduction in Whales, Dolphins, and Porpoises. pp. 97–134.

Pulster, E.L., K.L. Smalling, E. Zolman, L. Schwacke, and K.A. Maruya. 2009. Persistent organochlorine pollutants and toxaphene congener profiles in bottlenose dolphins (*Tursiops truncatus*) frequenting the Turtle/Brunswick River Estuary, Georgia, USA. Environmental Toxicology and Chemistry 28(7):1390–1399. doi: 10.1897/08-240.1

Read, A.J., K.W. Urian, B. Wilson, and D.M. Waples. 2003. Abundance of bottlenose dolphins in the bays, sounds, and estuaries of North Carolina. Marine Mammal Science 19(1):59–73. doi: 10.1111/j.1748-7692.2003.tb01092.x

Reif, J.S., A.M. Schaefer, G.D. Bossart, and P.A. Fair. 2017. Health and Environmental Risk Assessment Project for bottlenose dolphins *Tursiops truncatus* from the southeastern USA. II. Environmental aspects. Diseases of Aquatic Organisms 125(2):155–166. doi: 10.3354/dao03143

Rodriguez, L.K., A.D. Fandel, B.R. Colbert, J.C. Testa, and H. Bailey. 2021. Spatial and temporal variation in the occurrence of bottlenose dolphins in the Chesapeake Bay, USA, using citizen science sighting data. PLoS ONE 16(5):e0251637. doi: 10.1371/journal.pone.0251637

Rossman, S., P.H. Ostrom, M. Stolen, N.B. Barros, H. Gandhi, C.A. Stricker, and R.S. Wells. 2015. Individual specialization in the foraging habits of female bottlenose dolphins living in a trophically diverse and habitat rich estuary. Oecologia 178(2):415–425. doi: 10.1007/s00442-015-3241-6

Schwacke, L.H., E.O. Voit, L.J. Hansen, R.S. Wells, G.B. Mitchum, A.A. Hohn, and P.A. Fair. 2002. Probabilistic risk assessment of reproductive effects of polychlorinated biphenyls on bottlenose dolphins (*Tursiops truncatus*) from the southeast United States coast. Environmental Toxicology and Chemistry 21(12):2752–2764. doi: 10.1002/etc.5620211232

Schwacke, L.H., E.S. Zolman, B.C. Balmer, S. De Guise, R.C. George, J. Hoguet, A.A. Hohn, J.R. Kucklick, S. Lamb, M. Levin, J.A. Litz, W.E. McFee, N.J. Place, F.I. Townsend, R.S. Wells, and T.K. Rowles. 2012. Anaemia, hypothyroidism and immune suppression associated with polychlorinated biphenyl exposure in bottlenose dolphins (*Tursiops truncatus*). Proceedings of the Royal Society B: Biological Sciences 279(1726):48–57. doi: 10.1098/rspb.2011.0665

Sergeant, D.E., D.K. Caldwell, and M.C. Caldwell. 1973. Age, growth, and maturity of bottlenosed dolphin (*Tursiops-truncatus*) from northeast Florida. Journal of the Fisheries Research Board of Canada 30(7):1009–1011. doi: 10.1139/f73-165

Shane, S.H., R.S. Wells, and B. Würsig. 1986. Ecology, behavior and social organization of the bottlenose dolphin: A review. Marine Mammal Science 2:34–63. doi: 10.1111/j.1748-7692.1986.tb00026.x

Silva, D., B. Tramutolo, E. DeSalvio, T. Speakman, and R. Young. 2019. Abundance and movements of the Northern South Carolina Estuarine System Stock of bottlenose dolphins (*Tursiops truncatus*) (USA). Journal of Marine Animals and Their Ecology 11(1):8–18.

Sloan, P.E. 2006. Residency patterns, seasonality and habitat use among bottlenose dolphins, *Tursiops truncatus*, in the Cape Romain National Wildlife Refuge, S.C. University of North Carolina Wilmington. Thesis. 75 p.

Speakman, T., E. Zolman, J. Adams, R.H. Defran, D. Laska, L. Schwacke, J. Craigie, and P. Fair. 2006. Temporal and spatial aspects of bottlenose dolphin occurrence in coastal and estuarine waters near Charleston, South Carolina. National Oceanic and Atmospheric Administration, National Ocean Service, National Centers for Coastal Ocean Science: Charleston, SC. NOAA Technical Memorandum NOS-NCCOS-37. 50 p.

Stavros, H.-C.W., G.D. Bossart, T.C. Hulsey, and P.A. Fair. 2007. Trace element concentrations in skin of free-ranging bottlenose dolphins (*Tursiops truncatus*) from the southeast Atlantic coast. Science of the Total Environment 388(1-3):300–315. doi: 10.1016/j.scitotenv.2007.07.030

Stavros, H.-C.W., G.D. Bossart, T.C. Hulsey, and P.A. Fair. 2008. Trace element concentrations in blood of free-ranging bottlenose dolphins (*Tursiops truncatus*): Influence of age, sex and location. Marine Pollution Bulletin 56(2):371–379. doi: 10.1016/j.marpolbul.2007.10.030

Stavros, H.-C.W., M. Stolen, W.N. Durden, W. McFee, G.D. Bossart, and P.A. Fair. 2011. Correlation and toxicological inference of trace elements in tissues from stranded and free-ranging bottlenose dolphins (*Tursiops truncatus*). Chemosphere 82(11):1649–1661. doi: 10.1016/j.chemosphere.2010.11.019

Taylor, B.L., S.J. Chivers, J. Larese, and W.F. Perrin. 2007. Generation length and percent mature estimates for IUCN assessments of cetaceans. National Oceanic and Atmospheric Administration, National Marine Fisheries Service, Southwest Fisheries Science Center: La Jolla, CA. Administrative Report LJ-07-01. 24 p.

Thayer, V.G. 2008. Life history parameters and social associations of female bottlenose dolphins (*Tursiops truncatus*) off North Carolina, USA. Duke University. Dissertation. 164 p.

Thayer, V.G., A.J. Read, A.S. Friedlaender, D.R. Colby, A.A. Hohn, W.A. McLellan, D.A. Pabst, J.L. Dearolf, N.I. Bowles, J.R. Russell, and K.A. Rittmaster. 2003. Reproductive seasonality of western Atlantic bottlenose dolphins off North Carolina, USA. Marine Mammal Science 19(4):617–629. doi: 10.1111/j.1748-7692.2003.tb01120.x

Titcomb, E.M., J.S. Reif, P.A. Fair, H.-C.W. Stavros, M. Mazzoil, G.D. Bossart, and A.M. Schaefer. 2017. Blood mercury concentrations in common bottlenose dolphins from the Indian River Lagoon, Florida: Patterns of social distribution. Marine Mammal Science 33(3):771–784. doi: 10.1111/mms.12390

Torres, L.G. 2007. Top predator distribution and foraging ecology in Florida Bay, Florida. Duke University. Dissertation. 225 p.

Torres, L.G. and A.J. Read. 2009. Where to catch a fish? The influence of foraging tactics on the ecology of bottlenose dolphins (*Tursiops truncatus*) in Florida Bay, Florida. Marine Mammal Science 25(4):797–815. doi: 10.1111/j.1748-7692.2009.00297.x

Urian, K.W., D.M. Waples, R.B. Tyson, L.E. Hodge, and A.J. Read. 2013. Abundance of bottlenose dolphins (*Tursiops truncatus*) in estuarine and near-shore waters of North Carolina, USA. Journal of the North Carolina Academy of Science 129(4):165–171. doi: 10.7572/2167-5880-129.4.165

Vollmer, N.L. and P.E. Rosel. 2013. A review of common bottlenose dolphins (*Tursiops truncatus truncatus*) in the Northern Gulf of Mexico: Population biology, potential threats, and management. Southeastern Naturalist 12(m6):1–43. doi: 10.1656/058.012.m601

Wells, R.S. 2000. Reproduction in wild bottlenose dolphins: Overview of patterns observed during a long-term study. In: Duffield, D. and T. Robeck (Eds.), Bottlenose Dolphin Reproduction Workshop. AZA Marine Mammal Taxon Advisory Group, Silver Spring, MD. pp. 57–74.

Wells, R.S. 2003. Dolphin social complexity: Lessons from long-term study and life history. In: de Waal, F.B.M. and P.L. Tyack (Eds.), Animal Social Complexity: Intelligence, Culture, and Individualized Societies Harvard University Press, Cambridge, MA. pp. 32–56.

Wells, R.S. 2014. Social structure and life history of bottlenose dolphins near Sarasota Bay, Florida: Insights from four decades and five generations. Primates and cetaceans. pp. 149–172. doi: 10.1007/978-4-431-54523-1_8

Wells, R.S., A. Natoli, and G. Braulik. 2019. *Tursiops truncatus* (errata version published in 2019). *The IUCN Red List of Threatened Species* 2019: e.T22563A156932432. https://dx.doi.org/10.2305/IUCN.UK.2019-1.RLTS.T22563A156932432.en

Wells, R.S. and M.D. Scott. 1999. Bottlenose dolphin *Tursiops truncatus* (Montagu, 1821). In: Ridgway, S.H. and R. Harrison (Eds.), Handbook of marine mammals. Volume 6: The second book of dolphins and the porpoises. Academic Press, San Diego, CA. pp. 137–182.

Wells, R.S. and M.D. Scott. 2018. Bottlenose dolphin, *Tursiops truncatus*, common bottlenose dolphin. In: Würsig, B., J.G.M. Thewissen, and K.M. Kovacs (Eds.), Encyclopedia of Marine Mammals (Third Edition). Academic Press, London, UK. pp. 118–125. doi: 10.1016/B978-0-12-804327-1.00072-8

Wells, R.S., M.D. Scott, and A.B. Irvine. 1987. The social structure of free-ranging bottlenose dolphins. In: Genoways, H.H. (Ed.), Current Mammalogy. Plenum Press, New York, NY. pp. 247–305.

Wilson, R.M., R.B. Tyson, J.A. Nelson, B.C. Balmer, J.P. Chanton, and D.P. Nowacek. 2017. Niche differentiation and prey selectivity among common bottlenose dolphins (*Tursiops truncatus*) sighted in St. George Sound, Gulf of Mexico. Frontiers in Marine Science 4:235. doi: 10.3389/fmars.2017.00235

Yordy, J.E., M.A. Mollenhauer, R.M. Wilson, R.S. Wells, A. Hohn, J. Sweeney, L.H. Schwacke, T.K. Rowles, J.R. Kucklick, and M.M. Peden-Adams. 2010. Complex contaminant exposure in cetaceans: a comparative E-Screen analysis of bottlenose dolphin blubber and mixtures of four persistent organic pollutants. Environmental Toxicology and Chemistry 29(10):2143–2153. doi: 10.1002/etc.279

Common bottlenose dolphin − *Tursiops truncatus*

Northern South Carolina Estuarine System Stock

Overall Vulnerability Rank = Very High

Biological Sensitivity = Very High

Climate Exposure = Very High

Data Quality = 96% of scores ≥ 2

	Tursiops truncatus	Expert Scores	Data Quality	Expert Scores Plots (Portion by Category)
Sensitivity attributes	Prey/Diet Specificity	2.1	2.0	
	Habitat Specificity	3.7	2.0	
	Site Fidelity	4.0	2.7	
	Lifetime Reproductive Potential	1.5	2.0	
	Generation Length	3.0	2.0	
	Reproductive Plasticity	1.7	2.0	
	Migration	3.9	2.7	
	Home Range	3.3	2.7	
	Stock Abundance	3.0	2.7	
	Stock Abundance Trend	2.2	1.3	
	Cumulative Stressors	2.2	2.3	
	Sensitivity Score	Very High		
Exposure variables	Sea Surface Temperature (Change in mean)	4.0	3.0	
	Sea Surface Temperature (Change in variability)	1.0	3.0	
	Air Temperature (Change in mean)	4.0	3.0	
	Air Temperature (Change in variability)	1.0	3.0	
	Precipitation (Change in mean)	1.0	3.0	
	Precipitation (Change in variability)	2.0	3.0	
	Sea Surface Salinity (Change in mean)	3.7	3.0	
	Sea Surface Salinity (Change in variability)	1.0	3.0	
	Ocean pH (Change in mean)	4.0	2.7	
	Ocean pH (Change in variability)	1.0	2.7	
	Sea ice coverage (Change in mean)	1.0	3.0	
	Sea ice coverage (Change in variability)	1.0	3.0	
	Dissolved oxygen (Change in mean)	4.0	3.0	
	Dissolved oxygen (Change in variability)	1.0	3.0	
	Circulation	4.0	2.0	
	Sea level rise	4.0	3.0	
	Exposure Score	Very High		
Overall Vulnerability Rank		Very High		

Low
Moderate
High
Very High

Common bottlenose dolphin (Northern South Carolina Estuarine System Stock)

Tursiops truncatus

CVA Results Summary

Overall Climate Vulnerability Rank: Very High (100% certainty from bootstrap analysis).

Climate Exposure: Very High. Seven exposure factors scored greater than 3.5: Air Temperature (Standard anomaly) (4.00), Circulation (4.00), Dissolved oxygen (Standard anomaly) (4.00), Ocean pH (Standard anomaly) (4.00), Sea level rise (4.00), Sea Surface Temperature (Standard anomaly) (4.00), and Sea Surface Salinity (Standard anomaly) (3.73).

Biological Sensitivity: Very High. Three sensitivity attributes scored greater than 3.5: Site Fidelity (4.00), Migration (3.93), and Habitat Specificity (3.67).

Distributional Response: High

Abundance Response: High

Phenology Response: High

Data Quality: 96 % of the data quality scores were 2 or greater 91 % of sensitivity attribute data quality scores were 2 or higher.

Background Information

Stock Description

The Northern South Carolina Estuarine System (NSCES) Stock includes common bottlenose dolphins found in estuarine waters and nearshore waters (out to 1km) of South Carolina from the southern extent of the Murrells Inlet in the north to Price Inlet in the south (Waring et al. 2016).

Prey/Diet Specificity

Generally, common bottlenose dolphins consume a large variety of fish and/or squid (Barros and Odell 1990; Mead and Potter 1990; Wells and Scott 2018). Common bottlenose dolphins seem to show a consistent preference for sound-producing fishes (e.g., sciaenids, toadfish; Berens McCabe et al. 2010; Bowen-Stevens et al. 2021). Estuarine stocks along the southeast U.S. Atlantic coast have been reported to consume a variety of prey, with preference for sciaenids (Gannon and Waples 2004; Pate and McFee 2012). Pate and McFee (2012) identified 42 prey species from 24 families in a study of stranded dolphins along the South Carolina coast. In Sarasota Bay, Florida, Rossman et al. (2015) found some individual common bottlenose dolphins to show diet specialization despite the broader study group showing a generalist diet. Specialization can be influenced by locally abundant prey species (Shane et al. 1986; Vollmer and Rosel 2013) or can result from resource partitioning amongst sympatric populations (e.g., Torres and Read 2009; Wilson et al. 2017).

Habitat Specificity

Common bottlenose dolphins are found in a variety of marine and estuarine habitats (Wells and Scott 2018). The area inhabited by the NSCES Stock is characterized by tidal salt marshes, estuarine waters, and shallow open water with mudflats and oyster reefs (Waring et al. 2016).

Site Fidelity

No specific site fidelity information was found for this stock. Sloan (2006) observed twelve year-round residents of the NSCES Stock in Cape Romain National Wildlife Refuge that showed long-term site fidelity. Brusa (2012) identified 84 individuals displaying short-term site fidelity and 11 individuals displaying long-term site fidelity in North Inlet and Winyah Bay. Other estuarine stocks in the western North Atlantic have shown site fidelity (Waring et al. 2016), such as in South Carolina (Speakman et al. 2006; Waring et al. 2016), central and southern Georgia (Balmer et al. 2011; Kucklick et al. 2011; Balmer et al. 2013), northern Florida (Caldwell 2001), central Florida (Odell and Asper 1990; Mazzoil et al. 2005, 2008a, 2008b), and southern Florida (Litz 2007; Torres 2007; McClellan et al. 2000).

Lifetime Reproductive Potential

Taylor et al. (2007) reported the interbirth interval of common bottlenose dolphins as 3.80 years, though other studies have shown it to be closer to three years (Leatherwood 1977; Wells and Scott 1999; Connor et al. 2000; Wells 2003). Thayer (2008) estimated mean interbirth interval of 2.9 years in North Carolina.

Generally, females reach sexual maturity before males, with females reaching sexual maturity at 5–13 years (Sergeant et al. 1973; Perrin and Reilly 1984; Wells et al. 1987; Mead and Potter 1990; Mann et al. 2000; Wells 2000; Wells and Scott 2018).

Females reproduce through most of their adult lives (Leatherwood 1977; Hohn et al. 1989; Wells and Scott 1999; Wells 2000; Wells 2003), but may produce fewer calves after they reach 30 years of age (Wells 2000). Sarasota Bay common bottlenose dolphins can give birth when they are as young as 6 years of age, and can continue until they are 48 years old (Wells and Scott 2018). Reproductive lifespan would therefore be up to 42 years based on long-term observation studies (Wells 2014; Wells and Scott 2018).

Longitudinal studies from Sarasota Bay, Florida have found that female bottlenose dolphins can live up to 67 years (Wells and Scott 2018). Studies based on growth layer groups in teeth suggest female bottlenose dolphins can live to 41 years along the Texas coast (Fernandez and Hohn 1998) and to 30 years along the Mississippi coast (Mattson et al. 2006).

Generation Length

Taylor et al. (2007) reported common bottlenose dolphin generation length of 20.6–21.1 years.

Reproductive Plasticity

Common bottlenose dolphin calving is generally diffusely seasonal, with one or more peaks, although births have been reported from all seasons (Vollmer and Rosel 2013; Wells and Scott 2018). Calf

sightings in South Carolina peak in spring and early summer and again in early autumn (McFee et al. 2014). In North Carolina, Thayer et al. (2003) observed a strong birth peak in spring based on stranding data and a diffuse birth peak from late spring to early fall based on sightings.

Migration

The estuarine stocks in the western North Atlantic are thought to primarily comprise stable resident communities. However, transient or seasonal movements of some individuals into and out of stock areas are recorded in some areas (LaBrecque et al. 2015; Waring et al. 2016).

Dunn et al. (2014) suggested seasonal migrations of common bottlenose dolphins in the NSCES Stock, with different assemblages in the summer and winter.

Home Range

The home ranges of individuals within the western North Atlantic estuarine stocks are considered to generally include the areas within the stock boundaries and brief excursions beyond the stock boundaries (LaBrecque et al. 2015; Waring et al. 2016). In a photo ID study in North Inlet and Winyah Bay, Brusa (2012) identified 3 dolphins that were sighted only in North Inlet, 38 dolphins sighted only in Winyah Bay only, and 41 dolphins sighted in both, suggesting that part of the population maintains a constrained range while another segment of the population ventures farther. Brusa (2012) estimated mean home range of 32.79±13.02 km2 during the warm season and 4.73±3.51 km2 during the cold season.

Studies have shown dolphin populations with constrained ranges in Georgia (Brunswick and Sapelo; Balmer et al. 2013); South Carolina (Silva et al. 2019), and North Carolina (Urian et al. 2013). Balmer et al. (2018), using satellite telemetry data, identified that dolphins considered to be members of the South Georgia Estuarine System Stock had different movement patterns with some individuals remaining entirely within the estuarine waters, while other animals use the larger estuarine waters, sounds, and to some degree coastal waters.

Stock Abundance

Sloan (2006) identified 121 unique individuals in Cape Romain National Wildlife Refuge. Brusa et al. (2016) estimated 117 individuals (95% CI 92 to 142) during the warm season and 74 individuals (95% CI 48 to 100) during the cold season in North Inlet and Winyah Bay. Previously, Brusa (2012) identified 103 unique individuals during the warm season and 37 unique individuals during the cold season in North Inlet and Winyah Bay. However, all three of these estimates did not differentiate between resident and transient dolphins and did not include the entire range occupied by the NSCES Stock.

Stock Abundance Trend

The data are insufficient to evaluate population trends for this stock (Waring et al. 2016).

Cumulative Stressors

Common bottlenose dolphins face a wide range of individual and cumulative natural and anthropogenic threats (see reviews by Vollmer and Rosel 2013; Wells and Scott 2018; and Wells et al. 2019). These

include such threats as shark predation, harmful algal blooms, recreational and commercial fishing gear, vessel traffic, pollution, habitat alteration, and provisioning.

Common bottlenose dolphins in the NSCES stock interact with the inshore gillnet and blue crab trap/pot fisheries (Waring et al. 2016). Individuals of this stock have been reported stranded, with some strandings showing evidence of human interactions, and other strandings occurring during Unusual Mortality Events (UMEs; Waring et al. 2016; also see NOAA National Marine Mammal Health and Stranding Response Database). During the period 2009–2013, 11 common bottlenose dolphins stranded within the NSCES, with one individual presenting interactions with fishing gear (Waring et al. 2016).

A UME was declared in the summer of 2013 for the mid-Atlantic coast from New York to Brevard County, Florida, with more than 1500 common bottlenose dolphins stranding as a result of morbillivirus (Waring et al. 2016; NMFS 2022). Strandings were more prevalent along oceanic shores than in estuaries, suggesting that coastal stocks were more impacted by the UME than estuarine stocks (Waring et al. 2016). An UME for bottlenose dolphins was declared in winter and spring of 2011 in South Carolina, during which common bottlenose dolphins stranded at a rate nearly three times the historical average, with evidence suggesting an unusually cold winter and decreased prey abundance contributed to the UME (Waring et al. 2016; NMFS 2022).

The nearshore and estuarine habitats along the Atlantic seaboard are adjacent to areas of high human population and some are highly industrialized. Studies of bottlenose dolphin tissues from estuaries in the southeast U.S. have shown persistent organic pollutant (POP; Schwacke et al. 2002, 2012; Hansen et al. 2004; Litz et al. 2007; Pulster et al. 2009; Fair et al. 2010; Yordy et al. 2010; Balmer et al. 2011; Kucklick et al. 2011; Reif et al. 2017) and heavy metal (Durden et al. 2007; Stavros et al. 2007, 2008, 2011; Titcomb et al. 2017; Reif et al. 2017; Page-Karjian et al. 2020) concentrations that may result in adverse effects on health or reproductive rates, particularly in urbanized, developed, and agricultural areas. In Biscayne Bay, Florida, elevated POP concentrations were observed in individuals associated with more polluted areas compared to individuals associated with the less polluted areas of Biscayne Bay, suggesting that exposure to pollutants may be highly variable based on geography (Litz et al. 2007; Kucklick et al. 2011). In South Carolina, urban areas showed greater numbers of strandings than areas with primarily agricultural input (McFee and Burdett 2007). Common bottlenose dolphins in the nearby Charleston Estuarine System have been found with elevated levels of POPs (Hansen et al. 2004), polybrominated diphenyl ethers (PBDE; Fair et al. 2007; Kucklick et al. 2011; Adams et al. 2014), perfluoroalkyl compounds (PFCs; Adams et al. 2008). Adams et al. (2008) found dolphins in the Charleston Estuarine System that spent most of their time in urbanized and industrial areas had significantly higher plasma PFC concentrations than dolphins that spent most of their time in less developed areas. Common bottlenose dolphins stranded along the South Carolina coast have been found with ingested microplastics (Battaglia et al. 2020).

Common bottlenose dolphins stranded in South Carolina show a high prevalence of the gram-negative facultative intracellular bacterium *Brucella ceti* found in the tissues of brain, lung, and other tissues (McFee et al. 2020). Nearly 32% of dolphins tested positive for *B. ceti* over the six year study period, coinciding with the calving period.

Further Reading

Waring, G.T., E. Josephson, K. Maze-Foley, and P.E. Rosel. 2016. U.S. Atlantic and Gulf of Mexico Marine Mammal Stock Assessments - 2015. National Oceanic and Atmospheric Administration, National Marine Fisheries Service, Northeast Fisheries Science Center: Woods Hole, MA. NOAA Technical Memorandum NMFS-NE-238. 501 p. doi: 10.7289/V57S7KTN

Wells, R.S. and M.D. Scott. 2018. Bottlenose Dolphin, *Tursiops truncatus*, Common Bottlenose Dolphin. In: Würsig, B., J.G.M. Thewissen, and K.M. Kovacs (Eds.), Encyclopedia of Marine Mammals (Third Edition). Academic Press, London, UK. pp. 118–125. doi: 10.1016/B978-0-12-804327-1.00072-8

Wells, R.S., A. Natoli, and G. Braulik. 2019. *Tursiops truncatus* (errata version published in 2019). The IUCN Red List of Threatened Species 2019: e.T22563A156932432. https://dx.doi.org/10.2305/IUCN.UK.2019-1.RLTS.T22563A156932432.en

References

Adams, J., M. Houde, D. Muir, T. Speakman, G. Bossart, and P. Fair. 2008. Land use and the spatial distribution of perfluoroalkyl compounds as measured in the plasma of bottlenose dolphins (*Tursiops truncatus*). Marine Environmental Research 66(4):430–437. doi: 10.1016/j.marenvres.2008.07.004

Adams, J., T. Speakman, E. Zolman, G. Mitchum, E. Wirth, G.D. Bossart, and P.A. Fair. 2014. The relationship between land use and emerging and legacy contaminants in an Apex predator, the bottlenose dolphin (*Tursiops truncatus*), from two adjacent estuarine watersheds. Environmental Research 135:346–353. doi: 10.1016/j.envres.2014.08.037

Balmer, B., E. Zolman, T. Rowles, C. Smith, F. Townsend, D. Fauquier, C. George, T. Goldstein, L. Hansen, B. Quigley, W. McFee, J. Morey, P. Rosel, J. Saliki, T. Speakman, and L. Schwacke. 2018. Ranging patterns, spatial overlap, and association with dolphin morbillivirus exposure in common bottlenose dolphins (*Tursiops truncatus*) along the Georgia, USA coast. Ecology and Evolution 8(24):12890–12904. doi: 10.1002/ece3.4727

Balmer, B.C., L.H. Schwacke, R.S. Wells, J.D. Adams, R. Clay George, S.M. Lane, W.A. McLellan, P.E. Rosel, K. Sparks, T. Speakman, E.S. Zolman, and D. Ann Pabst. 2013. Comparison of abundance and habitat usage for common bottlenose dolphins between sites exposed to differential anthropogenic stressors within the estuaries of southern Georgia, U.S.A. Marine Mammal Science 29(2):E114–E135. doi: 10.1111/j.1748-7692.2012.00598.x

Balmer, B.C., L.H. Schwacke, R.S. Wells, R.C. George, J. Hoguet, J.R. Kucklick, S.M. Lane, A. Martinez, W.A. McLellan, P.E. Rosel, T.K. Rowles, K. Sparks, T. Speakman, E.S. Zolman, and D.A. Pabst. 2011. Relationship between persistent organic pollutants (POPs) and ranging patterns in common bottlenose dolphins (*Tursiops truncatus*) from coastal Georgia, USA. Science of the Total Environment 409(11):2094–2101. doi: 10.1016/j.scitotenv.2011.01.052

Barros, N.B. and D.K. Odell. 1990. Food habits of bottlenose dolphins in the southeastern United States In: Leatherwood, S. and R.R. Reeves (Eds.), The Bottlenose Dolphin. Academic Press, San Diego, CA. pp. 309–328. doi: 10.1016/B978-0-12-440280-5.50020-2

Battaglia, F.M., B.A. Beckingham, and W.E. McFee. 2020. First report from North America of microplastics in the gastrointestinal tract of stranded bottlenose dolphins (*Tursiops truncatus*). Marine Pollution Bulletin 160:111677. doi: 10.1016/j.marpolbul.2020.111677

Berens McCabe, E.J., D.P. Gannon, N.B. Barros, and R.S. Wells. 2010. Prey selection by resident common bottlenose dolphins (*Tursiops truncatus*) in Sarasota Bay, Florida. Marine Biology 157(5):931–942. doi: 10.1007/s00227-009-1371-2

Bowen-Stevens, S.R., D.P. Gannon, R.A. Hazelkorn, G. Lovewell, K.M. Volker, S. Smith, M.C. Tumlin, and J. Litz. 2021. Diet of common bottlenose dolphins, *Tursiops truncatus*, that stranded in and near Barataria Bay, Louisiana, 2010–2012. Southeastern Naturalist 20(1). doi: 10.1656/058.020.0113

Brusa, J.L. 2012. Distribution and social structure of an estuarine bottlenose dolphin (*Tursiops truncatus*) population in northern South Carolina. Coastal Carolina University. Thesis. 84 p.

Brusa, J.L., R.F. Young, and T. Swanson. 2016. Abundance, ranging patterns, and social behavior of bottlenose dolphins (*Tursiops truncatus*) in an estuarine terminus. Aquatic Mammals 42(1):109–121. doi: 10.1578/am.42.1.2016.109

Caldwell, M. 2001. Social and genetic structure of bottlenose dolphin (*Tursiops truncatus*) in Jacksonville, Florida. University of Miami. 143 p.

Connor, R.C., R.S. Wells, J. Mann, and A.J. Read. 2000. The bottlenose dolphin: Social relationships in a fission-fusion society. In: Mann, J., R. Connor, P. Tyack, and H. Whitehead (Eds.), Cetacean Societies: Field Studies of Dolphins and Whales. University of Chicago Press, Chicago, IL. pp. 91–126.

Dunn, C.N., D.C. Silva, E.P. Deborde, and R.F. Young. 2014. Distribution and movements of bottlenose dolphins (*Tursiops truncatus*) along the northern South Carolina coast: A research partnership with ecotourism. The Coastal Business Journal 14(1):63–76.

Durden, W.N., M.K. Stolen, D.H. Adams, and E.D. Stolen. 2007. Mercury and selenium concentrations in stranded bottlenose dolphins from the Indian River Lagoon system, Florida. Bulletin of Marine Science 81(1):37–54.

Fair, P.A., J. Adams, G. Mitchum, T.C. Hulsey, J.S. Reif, M. Houde, D. Muir, E. Wirth, D. Wetzel, E. Zolman, W. McFee, and G.D. Bossart. 2010. Contaminant blubber burdens in Atlantic bottlenose dolphins (*Tursiops truncatus*) from two southeastern US estuarine areas: Concentrations and patterns of PCBs, pesticides, PBDEs, PFCs, and PAHs. Science of the Total Environment 408(7):1577–1597. doi: 10.1016/j.scitotenv.2009.12.021

Fair, P.A., G. Mitchum, T.C. Hulsey, J. Adams, E. Zolman, W. McFee, E. Wirth, and G.D. Bossart. 2007. Polybrominated diphenyl ethers (PBDEs) in blubber of free-ranging bottlenose dolphins (*Tursiops truncatus*) from two southeast Atlantic estuarine areas. Archives of Environmental Contamination and Toxicology 53(3):483–494. doi: 10.1007/s00244-006-0244-7

Fernandez, S. and A.A. Hohn. 1998. Age, growth, and calving season of bottlenose dolphins, *Tursiops truncatus*, off coastal Texas. Fishery Bulletin 96(2):357–365.

Gannon, D.P. and D.M. Waples. 2004. Diets of coastal bottlenose dolphins from the U. S. Mid-Atlantic coast differ by habitat. Marine Mammal Science 20(3):527–545. doi: 10.1111/j.1748-7692.2004.tb01177.x

Hansen, L.J., L.H. Schwacke, G.B. Mitchum, A.A. Hohn, R.S. Wells, E.S. Zolman, and P.A. Fair. 2004. Geographic variation in polychorinated biphenyl and organochlorine pesticide concentrations in the blubber of bottlenose dolphins from the US Atlantic coast. Science of the Total Environment 319(1-3):147–172. doi: 10.1016/S0048-9697(03)00371-1

Hohn, A.A., M.D. Scott, R.S. Wells, J.C. Sweeney, and A.B. Irvine. 1989. Growth layers in teeth from known-age, free-ranging bottlenose dolphins. Marine Mammal Science 5(4):315–342. doi: 10.1111/j.1748-7692.1989.tb00346.x

Kucklick, J., L. Schwacke, R. Wells, A. Hohn, A. Guichard, J. Yordy, L. Hansen, E. Zolman, R. Wilson, J. Litz, D. Nowacek, T. Rowles, R. Pugh, B. Balmer, C. Sinclair, and P. Rosel. 2011. Bottlenose dolphins as indicators of persistent organic pollutants in the western North Atlantic Ocean and Northern Gulf of Mexico. Environmental Science & Technology 45(10):4270–4277. doi: 10.1021/es1042244

LaBrecque, E., C. Curtice, J. Harrison, S.M. Van Parijs, and P.N. Halpin. 2015. 2. Biologically Important Areas for Cetaceans Within U.S. Waters – East Coast Region. Aquatic Mammals 41(1):17–29. doi: 10.1578/am.41.1.2015.17

Leatherwood, S. 1977. Some preliminary impressions on the numbers and social behavior of free-swimming bottlenosed dolphin calves (*Tursiops truncatus*) in the northern Gulf of Mexico. In: Ridgway, S.H. and K. Bernirschke (Eds.), Breeding Dolphins; Present Status, Suggestions for the Future. Final report to the US Marine Mammal Commission, Contract MM6AC009, MMC-76/07, Washington, DC. pp. 143–167.

Litz, J.A. 2007. Social structure, genetic structure, and persistent organohalogen pollutants in bottlenose dolphins (*Tursiops truncatus*) in Biscayne Bay, Florida. University of Miami. 140 p.

Litz, J.A., L.P. Garrison, L.A. Fieber, A. Martinez, J.P. Contillo, and J.R. Kucklick. 2007. Fine-scale spatial variation of persistent organic pollutants in bottlenose dolphins (*Tursiops truncatus*) in Biscayne Bay, Florida. Environmental Science & Technology 41(21):7222–7228. doi: 10.1021/es070440r

Mann, J., R.C. Connor, L.M. Barre, and M.R. Heithaus. 2000. Female reproductive success in bottlenose dolphins (*Tursiops* sp.): life history, habitat, provisioning, and group-size effects. Behavioral Ecology 11(2):210–219. doi: 10.1093/beheco/11.2.210

Mattson, M.C., K.D. Mullin, G.W. Ingram, and W. Hoggard. 2006. Age structure and growth of the bottlenose dolphin (*Tursiops truncatus*) from strandings in the Mississippi sound region of the north-central Gulf of Mexico from 1986 to 2003. Marine Mammal Science 22(3):654–666. doi: 10.1111/j.1748-7692.2006.00057.x

Mazzoil, M., S.D. McCulloch, and R.H. Defran. 2005. Observations on the site fidelity of bottlenose dolphins (*Tursiops truncatus*) in the Indian River Lagoon, Florida. Florida Scientist 68(4):217–226.

Mazzoil, M., J.S. Reif, M. Youngbluth, M.E. Murdoch, S.E. Bechdel, E. Howells, S.D. McCulloch, L.J. Hansen, and G.D. Bossart. 2008. Home ranges of bottlenose dolphins (*Tursiops truncatus*) in the Indian River Lagoon, Florida: Environmental correlates and implications for management strategies. Ecohealth 5(3):278–288. doi: 10.1007/s10393-008-0194-9

Mazzoil, M.S., S.D. McCulloch, M.J. Youngbluth, D.S. Kilpatrick, M.E. Murdoch, B. Mase-Guthrie, D.K. Odell, and G.D. Bossart. 2008. Radio-tracking and survivorship of two rehabilitated bottlenose dolphins (*Tursiops truncatus*) in the Indian River Lagoon, Florida. Aquatic Mammals 34:54–64. doi: 10.1578/AM.34.1.2008.54

McClellan, D.B., J. Browder, J.L. Tobias, G.J. Konoval, M.D. Heron, O.L. Bass, and J. Osborne. 2000. Opportunistic sightings of bottlenose dolphin, *Tursiops truncatus*, along the southeast Florida coast and Florida Bay, 1992-1997. National Oceanic and Atmospheric Administration, National Marine Fisheries Service, Southeast Fisheries Science Center: Miami, FL. NOAA Technical Memorandum NMFS-SEFSC-435. 18 p.

McFee, W.E. and L.G. Burdett. 2007. Spatial and temporal analysis of bottlenose dolphin strandings in South Carolina, 1992-2005. National Oceanic and Atmospheric Administration, National Ocean Service, National Centers for Coastal Ocean Science: Charleston, SC. NOAA Technical Memorandum NOS-NCCOS-46. 12 p.

McFee, W.E., T.R. Speakman, L. Balthis, J.D. Adams, and E.S. Zolman. 2014. Reproductive seasonality of a recently designated bottlenose dolphin stock near Charleston, South Carolina, U.S.A. Marine Mammal Science 30(2):528–543. doi: 10.1111/mms.12055

McFee, W.E., D. Wu, K. Colegrove, K. Terio, L. Balthis, and R. Young. 2020. Occurrence of *Brucella ceti* in stranded bottlenose dolphins *Tursiops truncatus* coincides with calving season. Diseases of Aquatic Organisms 141:185–193. doi: 10.3354/dao03526

Mead, J.G. and C.W. Potter. 1990. Natural history of bottlenose dolphins along the central Atlantic coast of the United States. In: Leatherwood, S. and R.R. Reeves (Eds.), The Bottlenose Dolphin. Academic Press, San Diego, CA. pp. 165–195.

Odell, D.K. and E.D. Asper. 1990. Distribution and movements of freeze-branded bottlenose dolphins in the Indian and Banana Rivers, Florida. In: Leatherwood, S. and R. Reeves (Eds.), The Bottlenose Dolphin. Academic Press, San Diego, CA. pp. 515–540.

Page-Karjian, A., C.F. Lo, B. Ritchie, C.A. Harms, D.S. Rotstein, S. Han, S.M. Hassan, A.F. Lehner, J.P. Buchweitz, V.G. Thayer, J.M. Sullivan, E.F. Christiansen, and J.R. Perrault. 2020. Anthropogenic contaminants and histopathological findings in stranded cetaceans in the southeastern United States, 2012–2018. Frontiers in Marine Science 7:630. doi: 10.3389/fmars.2020.00630

Pate, S.M. and W.E. McFee. 2012. Prey species of bottlenose dolphins (*Tursiops truncatus*) from South Carolina Waters. Southeastern Naturalist 11(1):1–22. doi: 10.1656/058.011.0101

Perrin, W.F. and S.B. Reilly. 1984. Reproductive parameters of dolphins and small whales of the family Delphinidae. In: Perrin, W.F., R.L. Brownell, Jr., and D.P. DeMaster (Eds.), Reproduction in Whales, Dolphins, and Porpoises. pp. 97–134.

Pulster, E.L., K.L. Smalling, E. Zolman, L. Schwacke, and K.A. Maruya. 2009. Persistent organochlorine pollutants and toxaphene congener profiles in bottlenose dolphins (*Tursiops truncatus*) frequenting the Turtle/Brunswick River Estuary, Georgia, USA. Environmental Toxicology and Chemistry 28(7):1390–1399. doi: 10.1897/08-240.1

Reif, J.S., A.M. Schaefer, G.D. Bossart, and P.A. Fair. 2017. Health and Environmental Risk Assessment Project for bottlenose dolphins *Tursiops truncatus* from the southeastern USA. II. Environmental aspects. Diseases of Aquatic Organisms 125(2):155–166. doi: 10.3354/dao03143

Rossman, S., P.H. Ostrom, M. Stolen, N.B. Barros, H. Gandhi, C.A. Stricker, and R.S. Wells. 2015. Individual specialization in the foraging habits of female bottlenose dolphins living in a trophically diverse and habitat rich estuary. Oecologia 178(2):415–425. doi: 10.1007/s00442-015-3241-6

Schwacke, L.H., E.O. Voit, L.J. Hansen, R.S. Wells, G.B. Mitchum, A.A. Hohn, and P.A. Fair. 2002. Probabilistic risk assessment of reproductive effects of polychlorinated biphenyls on bottlenose dolphins (*Tursiops truncatus*) from the southeast United States coast. Environmental Toxicology and Chemistry 21(12):2752–2764. doi: 10.1002/etc.5620211232

Schwacke, L.H., E.S. Zolman, B.C. Balmer, S. De Guise, R.C. George, J. Hoguet, A.A. Hohn, J.R. Kucklick, S. Lamb, M. Levin, J.A. Litz, W.E. McFee, N.J. Place, F.I. Townsend, R.S. Wells, and T.K. Rowles. 2012. Anaemia, hypothyroidism and immune suppression associated with polychlorinated biphenyl exposure in bottlenose dolphins (*Tursiops truncatus*). Proceedings of the Royal Society B: Biological Sciences 279(1726):48–57. doi: 10.1098/rspb.2011.0665

Sergeant, D.E., D.K. Caldwell, and M.C. Caldwell. 1973. Age, growth, and maturity of bottlenosed dolphin (*Tursiops-truncatus*) from northeast Florida. Journal of the Fisheries Research Board of Canada 30(7):1009–1011. doi: 10.1139/f73-165

Shane, S.H., R.S. Wells, and B. Würsig. 1986. Ecology, behavior and social organization of the bottlenose dolphin: A review. Marine Mammal Science 2:34–63. doi: 10.1111/j.1748-7692.1986.tb00026.x

Silva, D., B. Tramutolo, E. DeSalvio, T. Speakman, and R. Young. 2019. Abundance and movements of the Northern South Carolina Estuarine System Stock of bottlenose dolphins (*Tursiops truncatus*) (USA). Journal of Marine Animals and Their Ecology 11(1):8–18.

Sloan, P.E. 2006. Residency patterns, seasonality and habitat use among bottlenose dolphins, *Tursiops truncatus*, in the Cape Romain National Wildlife Refuge, S.C. University of North Carolina Wilmington. Thesis. 75 p.

Speakman, T., E. Zolman, J. Adams, R.H. Defran, D. Laska, L. Schwacke, J. Craigie, and P. Fair. 2006. Temporal and spatial aspects of bottlenose dolphin occurrence in coastal and estuarine waters near Charleston, South Carolina. National Oceanic and Atmospheric Administration, National Ocean Service, National Centers for Coastal Ocean Science: Charleston, SC. NOAA Technical Memorandum NOS-NCCOS-37. 50 p.

Stavros, H.-C.W., G.D. Bossart, T.C. Hulsey, and P.A. Fair. 2007. Trace element concentrations in skin of free-ranging bottlenose dolphins (*Tursiops truncatus*) from the southeast Atlantic coast. Science of the Total Environment 388(1-3):300–315. doi: 10.1016/j.scitotenv.2007.07.030

Stavros, H.-C.W., G.D. Bossart, T.C. Hulsey, and P.A. Fair. 2008. Trace element concentrations in blood of free-ranging bottlenose dolphins (*Tursiops truncatus*): Influence of age, sex and location. Marine Pollution Bulletin 56(2):371–379. doi: 10.1016/j.marpolbul.2007.10.030

Stavros, H.-C.W., M. Stolen, W.N. Durden, W. McFee, G.D. Bossart, and P.A. Fair. 2011. Correlation and toxicological inference of trace elements in tissues from stranded and free-ranging bottlenose dolphins (*Tursiops truncatus*). Chemosphere 82(11):1649–1661. doi: 10.1016/j.chemosphere.2010.11.019

Taylor, B.L., S.J. Chivers, J. Larese, and W.F. Perrin. 2007. Generation length and percent mature estimates for IUCN assessments of cetaceans. National Oceanic and Atmospheric Administration, National Marine Fisheries Service, Southwest Fisheries Science Center: La Jolla, CA. Administrative Report LJ-07-01. 24 p.

Thayer, V.G. 2008. Life history parameters and social associations of female bottlenose dolphins (*Tursiops truncatus*) off North Carolina, USA. Duke University. Dissertation. 164 p.

Thayer, V.G., A.J. Read, A.S. Friedlaender, D.R. Colby, A.A. Hohn, W.A. McLellan, D.A. Pabst, J.L. Dearolf, N.I. Bowles, J.R. Russell, and K.A. Rittmaster. 2003. Reproductive seasonality of western Atlantic bottlenose dolphins off North Carolina, USA. Marine Mammal Science 19(4):617–629. doi: 10.1111/j.1748-7692.2003.tb01120.x

Titcomb, E.M., J.S. Reif, P.A. Fair, H.-C.W. Stavros, M. Mazzoil, G.D. Bossart, and A.M. Schaefer. 2017. Blood mercury concentrations in common bottlenose dolphins from the Indian River Lagoon, Florida: Patterns of social distribution. Marine Mammal Science 33(3):771–784. doi: 10.1111/mms.12390

Torres, L.G. 2007. Top predator distribution and foraging ecology in Florida Bay, Florida. Duke University. Dissertation. 225 p.

Torres, L.G. and A.J. Read. 2009. Where to catch a fish? The influence of foraging tactics on the ecology of bottlenose dolphins (*Tursiops truncatus*) in Florida Bay, Florida. Marine Mammal Science 25(4):797–815. doi: 10.1111/j.1748-7692.2009.00297.x

Urian, K.W., D.M. Waples, R.B. Tyson, L.E. Hodge, and A.J. Read. 2013. Abundance of bottlenose dolphins (*Tursiops truncatus*) in estuarine and near-shore waters of North Carolina, USA. Journal of the North Carolina Academy of Science 129(4):165–171. doi: 10.7572/2167-5880-129.4.165

Vollmer, N.L. and P.E. Rosel. 2013. A review of common bottlenose dolphins (*Tursiops truncatus truncatus*) in the Northern Gulf of Mexico: Population biology, potential threats, and management. Southeastern Naturalist 12(m6):1–43. doi: 10.1656/058.012.m601

Waring, G.T., E. Josephson, K. Maze-Foley, and P.E. Rosel. 2016. U.S. Atlantic and Gulf of Mexico marine mammal stock assessments - 2015. National Oceanic and Atmospheric Administration, National Marine Fisheries Service, Northeast Fisheries Science Center: Woods Hole, MA. NOAA Technical Memorandum NMFS-NE-238. 501 p. doi: 10.7289/V57S7KTN

Wells, R.S. 2000. Reproduction in wild bottlenose dolphins: Overview of patterns observed during a long-term study. In: Duffield, D. and T. Robeck (Eds.), Bottlenose Dolphin Reproduction Workshop. AZA Marine Mammal Taxon Advisory Group, Silver Spring, MD. pp. 57–74.

Wells, R.S. 2003. Dolphin social complexity: Lessons from long-term study and life history. In: de Waal, F.B.M. and P.L. Tyack (Eds.), Animal Social Complexity: Intelligence, Culture, and Individualized Societies Harvard University Press, Cambridge, MA. pp. 32–56.

Wells, R.S. 2014. Social structure and life history of bottlenose dolphins near Sarasota Bay, Florida: Insights from four decades and five generations. Primates and cetaceans. pp. 149–172. doi: 10.1007/978-4-431-54523-1_8

Wells, R.S., A. Natoli, and G. Braulik. 2019. *Tursiops truncatus* (errata version published in 2019). *The IUCN Red List of Threatened Species* 2019: e.T22563A156932432. https://dx.doi.org/10.2305/IUCN.UK.2019-1.RLTS.T22563A156932432.en

Wells, R.S. and M.D. Scott. 1999. Bottlenose dolphin *Tursiops truncatus* (Montagu, 1821). In: Ridgway, S.H. and R. Harrison (Eds.), Handbook of marine mammals. Volume 6: The second book of dolphins and the porpoises. Academic Press, San Diego, CA. pp. 137–182.

Wells, R.S. and M.D. Scott. 2018. Bottlenose dolphin, *Tursiops truncatus*, common bottlenose dolphin. In: Würsig, B., J.G.M. Thewissen, and K.M. Kovacs (Eds.), Encyclopedia of Marine Mammals (Third Edition). Academic Press, London, UK. pp. 118–125. doi: 10.1016/B978-0-12-804327-1.00072-8

Wells, R.S., M.D. Scott, and A.B. Irvine. 1987. The social structure of free-ranging bottlenose dolphins. In: Genoways, H.H. (Ed.), Current Mammalogy. Plenum Press, New York, NY. pp. 247–305.

Wilson, R.M., R.B. Tyson, J.A. Nelson, B.C. Balmer, J.P. Chanton, and D.P. Nowacek. 2017. Niche differentiation and prey selectivity among common bottlenose dolphins (*Tursiops truncatus*) sighted in St. George Sound, Gulf of Mexico. Frontiers in Marine Science 4:235. doi: 10.3389/fmars.2017.00235

Yordy, J.E., M.A. Mollenhauer, R.M. Wilson, R.S. Wells, A. Hohn, J. Sweeney, L.H. Schwacke, T.K. Rowles, J.R. Kucklick, and M.M. Peden-Adams. 2010. Complex contaminant exposure in cetaceans: a comparative E-Screen analysis of bottlenose dolphin blubber and mixtures of four persistent organic pollutants. Environmental Toxicology and Chemistry 29(10):2143–2153. doi: 10.1002/etc.279

Common bottlenose dolphin − *Tursiops truncatus*

Puerto Rico and US Virgin Islands Stock

Overall Vulnerability Rank = High

Biological Sensitivity = Moderate

Climate Exposure = Very High

Data Quality = 59% of scores ≥ 2

	Tursiops truncatus	Expert Scores	Data Quality	Expert Scores Plots (Portion by Category)
Sensitivity attributes	Prey/Diet Specificity	2.3	2.0	
	Habitat Specificity	2.4	1.8	
	Site Fidelity	2.5	1.5	
	Lifetime Reproductive Potential	1.6	2.0	
	Generation Length	2.9	2.0	
	Reproductive Plasticity	1.8	1.5	
	Migration	3.5	1.2	
	Home Range	2.7	1.8	
	Stock Abundance	2.6	1.0	
	Stock Abundance Trend	2.3	0.8	
	Cumulative Stressors	2.3	2.0	
	**Sensitivity Score**	Moderate		
Exposure variables	Sea Surface Temperature (Change in mean)	3.8	2.2	
	Sea Surface Temperature (Change in variability)	1.5	2.2	
	Air Temperature (Change in mean)	3.6	2.2	
	Air Temperature (Change in variability)	1.4	2.2	
	Precipitation (Change in mean)	1.6	2.2	
	Precipitation (Change in variability)	1.6	2.2	
	Sea Surface Salinity (Change in mean)	2.6	2.2	
	Sea Surface Salinity (Change in variability)	1.4	2.2	
	Ocean pH (Change in mean)	3.8	2.2	
	Ocean pH (Change in variability)	1.6	2.2	
	Sea ice coverage (Change in mean)	1.0	1.8	
	Sea ice coverage (Change in variability)	1.0	1.8	
	Dissolved oxygen (Change in mean)	3.6	2.2	
	Dissolved oxygen (Change in variability)	1.4	2.2	
	Circulation	2.4	1.5	
	Sea level rise	2.2	1.8	
	**Exposure Score**	Very High		
**Overall Vulnerability Rank**		High		

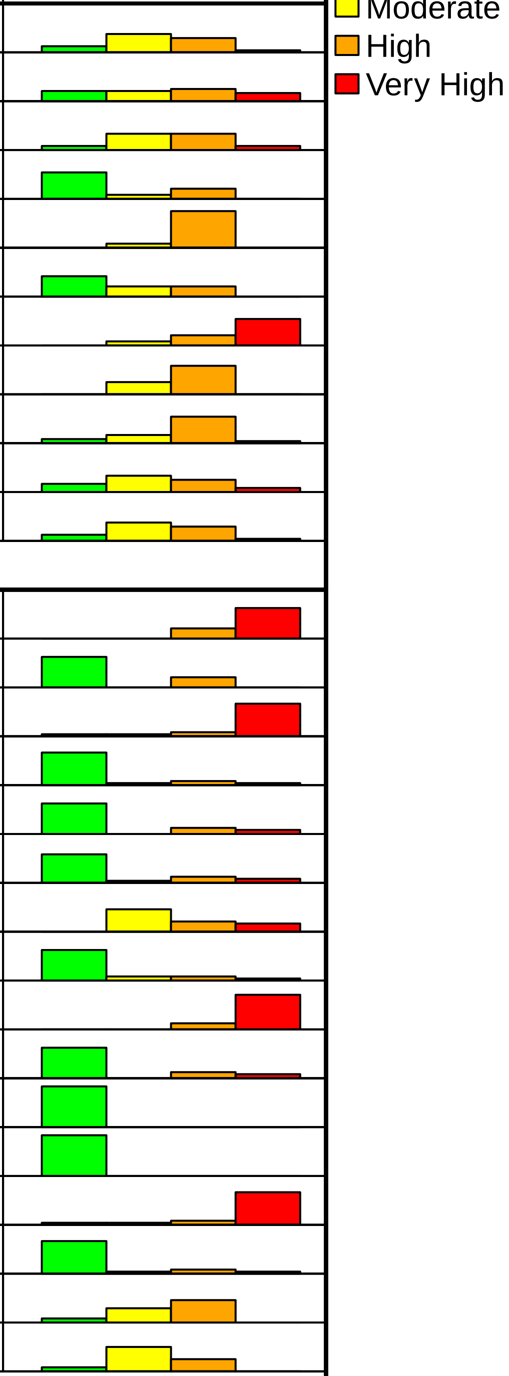

Common bottlenose dolphin (Puerto Rico and US Virgin Islands Stock)

Tursiops truncatus

CVA Results Summary

Overall Climate Vulnerability Rank: High (83% certainty from bootstrap analysis).

Climate Exposure: Very High. Four exposure factors scored greater than 3.5: Ocean pH (Standard anomaly) (3.85), Sea Surface Temperature (Standard anomaly) (3.75), Air Temperature (Standard anomaly) (3.65), and Dissolved oxygen (Standard anomaly) (3.65).

Biological Sensitivity: Moderate. Five sensitivity attributes scored greater than or equal to 2.5: Migration (3.55), Generation Time (2.90), Home Range (2.70), Species Abundance (2.65), and Site Fidelity (2.50).

Distributional Response: Moderate

Abundance Response: Moderate

Phenology Response: Moderate

Data Quality: 59% of the data quality scores were 2 or greater, 36% of sensitivity attribute data quality scores were 2 or higher.

Background Information

Stock Description

This stock includes common bottlenose dolphins found in the waters of Puerto Rico and U.S. Virgin Islands and adjacent offshore waters. This stock is considered separate from the Atlantic Ocean and Gulf of Mexico stocks for management purposes (Waring et al. 2012).

Prey/Diet Specificity

Generally, common bottlenose dolphins consume a large variety of fish and/or squid (Barros and Odell 1990; Mead and Potter 1990; Wells and Scott 2018). Common bottlenose dolphins seem to show a consistent preference for sound-producing fishes (e.g., sciaenids, toadfish; Berens McCabe et al. 2010; Bowen-Stevens et al. 2021). In Sarasota Bay, Florida, Rossman et al. (2015) found some individual common bottlenose dolphins to show diet specialization despite the broader study group showing a generalist diet. Specialization can be influenced by locally abundant prey species (Shane et al. 1986; Vollmer and Rosel 2013) or can result from resource partitioning amongst sympatric populations (e.g., Torres and Read 2009; Wilson et al. 2017). Few records of common bottlenose diet exist for Puerto Rico and the U.S. Virgin Islands, though two records describe mortality due to ingestion of prey: an agujon (*Tylosurus acus*) that punctured the pleural cavity (Carrasquillo-Casado et al. 2002) and a black margate (*Anisotremus surinamensis*) that become lodged in the esophagus (Mignucci-Giannoni et al. 2009).

Habitat Specificity

Common bottlenose dolphins are found in a variety of marine and estuarine habitats (Wells and Scott 2018). The common bottlenose dolphin is found in nearshore shelf waters of Puerto Rico and the U.S. Virgin Islands (Erdman 1970; Erdman et al. 1973; Taruski and Winn 1976; Mignucci-Giannoni 1998; Rodriguez-Ferrer et al. 2020). Common bottlenose dolphins are known to use inshore waters such as mangrove channels (Erdman 1970) and also have been sighted in offshore waters and near the self-edge (Mignucci-Giannoni 1998; Rodriguez-Ferrer et al. 2020). Mignucci-Giannoni (1998) noted a high occurrence of common bottlenose dolphins found associated with areas of low sea floor relief. Off the southwest coast of Puerto Rico, Rodriguez-Ferrer et al. (2020) found common bottlenose dolphins most frequently with sea grass beds, sandy bottom, and reefs, while few common bottlenose dolphins were found associated with silty-clay substrate.

Site Fidelity

No specific site fidelity information was found for this stock.

Estuarine stocks in the western North Atlantic have shown site fidelity (Waring et al. 2012) in South Carolina (Sloan 2006; Waring et al. 2016), Georgia (Pulster and Maruya 2008; Balmer et al. 2011; Kucklick et al. 2011; Balmer et al. 2013), northern Florida (Caldwell 2001), central Florida (Odell and Asper 1990; Mazzoil et al. 2005; Mazzoil et al. 2008a; Mazzoil et al. 2008b), and southern Florida (Litz 2007; Torres 2007; McClellan et al. 2000). Common bottlenose dolphins in Gulf of Mexico bay, sound, and estuary (BSE) stocks show site fidelity (LaBrecque et al. 2015; Hayes et al. 2022), such as in Matagorda Bay, Espiritu Santo Bay, and San Luis Pass in Texas (Maze and Würsig 1999; Lynn and Würsig 2002; Irwin and Würsig 2004); Barataria Bay in Louisiana (Miller 2003; McDonald et al. 2017; Wells et al. 2017); Mississippi Sound in Mississippi (Hubard et al. 2004; Mackey 2010; Mullin et al. 2017); Charlotte Harbor and Pine Island Sound (Bassos-Hull et al. 2013), Sarasota Bay (Wells 1986; Wells 1991; Wells 2014), St. Andrew Bay (Balmer et al. 2019), St. Joseph Bay (Balmer et al. 2008; Balmer et al. 2018), St. Vincent Sound and Apalachicola Bay (Tyson et al. 2011), and Tampa Bay in Florida (Wells et al. 1996; Urian et al. 2009).

Lifetime Reproductive Potential

Taylor et al. (2007) reported the interbirth interval of common bottlenose dolphins as 3.80 years, though other studies have shown it to be closer to three years (Leatherwood 1977; Wells and Scott 1999; Connor et al. 2000; Wells 2003).

Generally, females reach sexual maturity before males, with females reaching sexual maturity at 5–13 years (Sergeant et al. 1973; Perrin and Reilly 1984; Wells et al. 1987; Mead and Potter 1990; Mann et al. 2000; Wells 2000; Wells and Scott 2018).

Females reproduce through most of their adult lives (Leatherwood 1977; Hohn et al. 1989; Wells and Scott 1999; Wells 2000; Wells 2003), but may produce fewer calves after they reach 30 years of age (Wells 2000). Sarasota Bay common bottlenose dolphins can give birth when they are as young as 6 years of age, and can continue until they are 48 years old (Wells and Scott 2018). Reproductive lifespan would therefore be up to 42 years based on long-term observation studies (Wells 2014; Wells and Scott 2018).

Longitudinal studies from Sarasota Bay, Florida have found that female bottlenose dolphins can live up to 67 years (Wells and Scott 2018). Studies based on growth layer groups in teeth suggest female bottlenose dolphins can live to 41 years along the Texas coast (Fernandez and Hohn 1998) and to 30 years along the Mississippi coast (Mattson et al. 2006).

Generation Length

Taylor et al. (2007) reported common bottlenose dolphin generation length of 20.6–21.1 years.

Reproductive Plasticity

Common bottlenose dolphin calving is generally diffusely seasonal, with one or more peaks, although births have been reported from all seasons (Vollmer and Rosel 2013; Wells and Scott 2018). A mid-summer calving peak (July through August) has been reported for bottlenose dolphins off Puerto Rico (Rodríguez-Ferrer 2001). Calf sightings peak in the spring and summer months in Florida (Wells et al. 1987), Mississippi/Louisiana (Miller et al. 2010; Miller et al. 2013), and Texas (McHugh 1989; Henderson and Würsig 2007).

Migration

Off Puerto Rico, bottlenose dolphins are common throughout the year (Mignucci-Giannoni 1989, 1998) with a peak occurrence during the fall (Rodríguez-Ferrer 2001). Seasonal inshore-offshore movements are suspected (Mignucci-Giannoni 1998).

Home Range

Bottlenose dolphins are common throughout the Caribbean (Waring et al. 2012). This species has been reported off Cuba (van Waerebeek et al. 2006; Whitt et al. 2011), Dominican Republic (Whaley et al. 2006; Parsons et al. 2010), Puerto Rico and the Virgin Islands (Erdman et al. 1973; Mignucci-Giannoni 1998; Rodríguez-Ferrer 2001), Guadeloupe, St. Lucia and Barbados (Yoshida et al. 2010), Martinique (Jérémie et al. 2006), the eastern Caribbean (Guadeloupe to St. Vincent and the Grenadines; Caldwell et al. 1971; Caldwell and Caldwell 1975; Yoshida et al. 2010), Trinidad (van Bree 1975), Venezuela (Romero et al. 2001; Romero et al. 2002; Oviedo et al. 2005), Leeward Netherlands Antilles (Debrot et al. 1998), Colombia (Romero et al. 2001; Pardo and Palacios 2006; Fraija et al. 2009; Pardo et al. 2009), Panama (Pardo et al. 2009), and Belize (Jefferson and Lynn 1994; Grigg and Markowitz 1997; Campbell et al. 2002; Kerr et al. 2005).

Because bottlenose dolphins are widely distributed throughout the Caribbean Sea, bottlenose dolphins of the Puerto Rico and U.S. Virgin Islands stock are likely trans-boundary with waters near adjacent Caribbean islands (Waring et al. 2012).

Stock Abundance

The most recent abundance estimate of 314 individuals was based on surveys conducted off southwest Puerto Rico between April 1999 and January 2002 (Rodríguez-Ferrer 2001).

Stock Abundance Trend

The data are insufficient to evaluate population trends for this stock (Waring et al. 2012).

Cumulative Stressors

Common bottlenose dolphins face a wide range of individual and cumulative natural and anthropogenic threats (see reviews by Vollmer and Rosel 2013; Wells and Scott 2018; and Wells et al. 2019). These include such threats as shark predation, harmful algal blooms, recreational and commercial fishing gear, vessel traffic, pollution, habitat alteration, and provisioning.

Although no longer present in U.S. waters, common bottlenose dolphin fisheries continue to exist elsewhere in the Caribbean (e.g., Caldwell et al. 1971; Caldwell and Caldwell 1975; Price 1985; Reeves 1988; Romero et al. 2001; Hoyt and Hvenegaard 2002; Mohammed et al. 2003; Vail 2005; World Council of Whalers 2008) and also in Venezuela (Romero et al. 2001). Live-capture fisheries for use in captivity exist or have existed recently in the Dominican Republic, Haiti, Cuba, and Honduras (van Waerebeek et al. 2006; Espinosa and Orta 2007; Parsons et al. 2010; Waring et al. 2012).

Common bottlenose dolphins in the Puerto Rico and U.S. Virgin Islands stock interact with pelagic longline fisheries (e.g., pelagic swordfish, tunas, billfish) and trap/pot fisheries (spiny lobster and mixed-species; Waring et al. 2012). Although no mortality or serious injury was reported during the period 2001-2015, this may represent an underestimation due to a lack of observer coverage in some years and some fisheries (Garrison 2003; Garrison and Richards 2004; Garrison 2005; Fairfield Walsh and Garrison 2006; Fairfield-Walsh and Garrison 2007; Fairfield and Garrison 2008; Garrison et al. 2009; Garrison and Stokes 2010; 2012a; 2012b; 2013; 2014; 2016; 2017)

Legacy impacts on common bottlenose dolphins from naval operations at Roosevelt Roads in Puerto Rico that ceased in 2004 are unknown (Waring et al. 2012).

Coastal pollution may be an issue for the common bottlenose dolphin Puerto Rico and U.S. Virgin Islands stock. Parts of Vieques Island, Puerto Rico are listed on the U.S. Environmental Protection Agency's (EPA) Superfund National Priorities List due to unexploded ordnance and associated hazardous materials (Whitall et al. 2016; EPA 2018).

Distribution

Modeled distribution of the common bottlenose dolphin in southwestern Puerto Rico is presented by Rodriguez-Ferrer et al. (2020).

Further Reading

Jefferson, T.A., M.A. Webber, and R.L. Pitman. 2015. Marine Mammals of the World (Second Edition). Academic Press, San Diego, CA. 616 p. doi: 10.1016/c2012-0-06919-0

Waring, G.T., E. Josephson, K. Maze-Foley, and P.E. Rosel. 2012. U.S. Atlantic and Gulf of Mexico Marine Mammal Stock Assessments - 2011. National Oceanic and Atmospheric Administration, National Marine Fisheries Service, Northeast Fisheries Science Center: Woods Hole, MA. NOAA Technical Memorandum NMFS-NE-221. 319 p.

Wells, R.S. and M.D. Scott. 2018. Bottlenose Dolphin, *Tursiops truncatus*, Common Bottlenose Dolphin. In: Würsig, B., J.G.M. Thewissen, and K.M. Kovacs (Eds.), Encyclopedia of Marine Mammals

(Third Edition). Academic Press, London, UK. pp. 118–125. doi: 10.1016/B978-0-12-804327-1.00072-8
Wells, R.S., A. Natoli, and G. Braulik. 2019. *Tursiops truncatus* (errata version published in 2019). The IUCN Red List of Threatened Species 2019: e.T22563A156932432. https://dx.doi.org/10.2305/IUCN.UK.2019-1.RLTS.T22563A156932432.en

References

Balmer, B., S. Watwood, B. Quigley, T. Speakman, K. Barry, K. Mullin, P. Rosel, C. Sinclair, E. Zolman, and L. Schwacke. 2019. Common bottlenose dolphin (*Tursiops truncatus*) abundance and distribution patterns in St. Andrew Bay, Florida, USA. Aquatic Conservation: Marine and Freshwater Ecosystems 29(3):486–498. doi: 10.1002/aqc.3001
Balmer, B., E. Zolman, T. Rowles, C. Smith, F. Townsend, D. Fauquier, C. George, T. Goldstein, L. Hansen, B. Quigley, W. McFee, J. Morey, P. Rosel, J. Saliki, T. Speakman, and L. Schwacke. 2018. Ranging patterns, spatial overlap, and association with dolphin morbillivirus exposure in common bottlenose dolphins (*Tursiops truncatus*) along the Georgia, USA coast. Ecology and Evolution 8(24):12890–12904. doi: 10.1002/ece3.4727
Balmer, B.C. 2011. Bottlenose dolphin (*Tursiops truncatus*) stock structure within the estuaries of southern Georgia. University of North Carolina Wilmington. 134 p.
Balmer, B.C., L.H. Schwacke, R.S. Wells, J.D. Adams, R. Clay George, S.M. Lane, W.A. McLellan, P.E. Rosel, K. Sparks, T. Speakman, E.S. Zolman, and D. Ann Pabst. 2013. Comparison of abundance and habitat usage for common bottlenose dolphins between sites exposed to differential anthropogenic stressors within the estuaries of southern Georgia, U.S.A. Marine Mammal Science 29(2):E114–E135. doi: 10.1111/j.1748-7692.2012.00598.x
Balmer, B.C., R.S. Wells, S.M. Nowacek, D.P. Nowacek, L.H. Schwacke, W.A. McLellan, F.S. Scharf, T.K. Rowles, L.J. Hansen, T.R. Spradlin, and D.A. Pabst. 2008. Seasonal abundance and distribution patterns of common bottlenose dolphins (*Tursiops truncatus*) near St. Joseph Bay, Florida, USA. Journal of Cetacean Research and Management 10(2):157–167.
Barros, N.B. and D.K. Odell. 1990. Food habits of bottlenose dolphins in the southeastern United States In: Leatherwood, S. and R.R. Reeves (Eds.), The Bottlenose Dolphin. Academic Press, San Diego, CA. pp. 309–328. doi: 10.1016/B978-0-12-440280-5.50020-2
Bassos-Hull, K., R.M. Perrtree, C.C. Shepard, S. Schilling, A.A. Barleycorn, J.B. Allen, B.C. Balmer, W.E. Pine, and R.S. Wells. 2013. Long-term site fidelity and seasonal abundance estimates of common bottlenose dolphins (*Tursiops truncatus*) along the southwest coast of Florida and responses to natural perturbations. Journal of Cetacean Research and Management 13(1):19–30. doi: 10.47536/jcrm.v13i1.551
Berens McCabe, E.J., D.P. Gannon, N.B. Barros, and R.S. Wells. 2010. Prey selection by resident common bottlenose dolphins (*Tursiops truncatus*) in Sarasota Bay, Florida. Marine Biology 157(5):931–942. doi: 10.1007/s00227-009-1371-2
Bowen-Stevens, S.R., D.P. Gannon, R.A. Hazelkorn, G. Lovewell, K.M. Volker, S. Smith, M.C. Tumlin, and J. Litz. 2021. Diet of common bottlenose dolphins, *Tursiops truncatus*, that stranded in and near Barataria Bay, Louisiana, 2010–2012. Southeastern Naturalist 20(1). doi: 10.1656/058.020.0113
Caldwell, D.K. and M.C. Caldwell. 1975. Dolphin and small whale fisheries of the Caribbean and West Indies: Occurrence, history, and catch statistics — with special reference to the Lesser Antillean island of St. Vincent. Journal of the Fisheries Research Board of Canada 32(7):1105–1110. doi: 10.1139/f75-132
Caldwell, D.K., M.C. Caldwell, W.F. Rathjen, and J.R. Sullivan. 1971. Cetaceans from the Lesser Antillean island of St. Vincent. Fishery Bulletin 69:303–312.

Caldwell, M. 2001. Social and genetic structure of bottlenose dolphin (*Tursiops truncatus*) in Jacksonville, Florida. University of Miami. 143 p.

Campbell, G.S., B.A. Bilgre, and R.H. Defran. 2002. Bottlenose dolphins (*Tursiops truncatus*) in Turneffe Atoll, Belize: Occurrence, site fidelity, group size, and abundance. Aquatic Mammals 28:170–180.

Carrasquillo-Casado, B., M. Alsina-Guerrero, M.A. Cardona-Maldonado, E.H. Williams, and A.A. Mignucci-Giannoni. 2002. Fatal pneumonia and pleuritis caused by an agujon beak penetration in a bottlenose dolphin from Puerto Rico. Journal of Aquatic Animal Health 14(1):65–67. doi: 10.1577/1548-8667(2002)014<0065:fpapcb>2.0.co;2

Connor, R.C., R.S. Wells, J. Mann, and A.J. Read. 2000. The bottlenose dolphin: Social relationships in a fission-fusion society. In: Mann, J., R. Connor, P. Tyack, and H. Whitehead (Eds.), Cetacean Societies: Field Studies of Dolphins and Whales. University of Chicago Press, Chicago, IL. pp. 91–126.

Debrot, A.O., J.A. De Meyer, and P.J.E. Dezentjé. 1998. Additional records and a review of the cetacean fauna of the Leeward Dutch Antilles. Caribbean Journal of Science 34(3-4):204–210.

Environmental Protection Agency (EPA). 2021. Atlantic Fleet Weapons Training Area, Vieques, PR. https://www.epa.gov/superfund/national-priorities-list-npl-sites-state#PR

Erdman, D.S. 1970. Marine mammals from Puerto Rico to Antigua. Journal of Mammalogy 51(3):636. doi: 10.2307/1378416

Erdman, D.S., J. Harms, and M. Marcial-Flores. 1973. Cetacean records from the northeastern Caribbean region. Cetology 17:1–14.

Espinosa, J. and J. Orta. 2007. Biota marina. In: González, H.A. (Ed.), Biodiversidad de Cuba. Ediciones Polymita, S.A., Ciudad de Guatemala. pp. 72–141.

Fairfield, C.P. and L.P. Garrison. 2008. Estimated bycatch of marine mammals and turtles in the U.S. Atlantic pelagic longline fleet during 2007. National Oceanic and Atmospheric Administration, National Marine Fisheries Service, Southeast Fisheries Science Center: Miami, FL. NOAA Technical Memorandum NMFS-SEFSC-572. 62 p.

Fairfield Walsh, C. and L.P. Garrison. 2006. Estimated bycatch of marine mammals and turtles in the U.S. Atlantic pelagic longline fleet during 2005. National Oceanic and Atmospheric Administration, National Marine Fisheries Service, Southeast Fisheries Science Center: Miami, FL. NOAA Technical Memorandum NMFS-SEFSC-539. 52 p.

Fairfield-Walsh, C. and L.P. Garrison. 2007. Estimated bycatch of marine mammals and turtles in the U.S. Atlantic pelagic longline fleet during 2006. National Oceanic and Atmospheric Administration, National Marine Fisheries Service, Southeast Fisheries Science Center: Miami, FL. NOAA Technical Memorandum NMFS-SEFSC-560. 54 p.

Fernandez, S. and A.A. Hohn. 1998. Age, growth, and calving season of bottlenose dolphins, *Tursiops truncatus*, off coastal Texas. Fishery Bulletin 96(2):357–365.

Fraija, N., L. Flórez-González, and A. Jáuregui. 2009. Cetacean occurrence in the Santa Marta Region, Colombian Caribbean, February-May 2007. Latin American Journal of Aquatic Mammals 7(1-2):69–73. doi: 10.5597/lajam00137

Garrison, L.P. 2003. Estimated bycatch of marine mammals and turtles in the U.S. Atlantic pelagic longline fleet during 2001-2002. National Oceanic and Atmospheric Administration, National Marine Fisheries Service, Southeast Fisheries Science Center: Miami, FL. NOAA Technical Memorandum NMFS-SEFSC-515. 52 p.

Garrison, L.P. 2005. Estimated bycatch of marine mammals and turtles in the U.S. Atlantic pelagic longline fleet during 2004. National Oceanic and Atmospheric Administration, National Marine Fisheries Service, Southeast Fisheries Science Center: Miami, FL. NOAA Technical Memorandum NMFS-SEFSC-531. 57 p.

Garrison, L.P. and P.M. Richards. 2004. Estimated bycatch of marine mammals and turtles in the U.S. Atlantic pelagic longline fleet during 2003. National Oceanic and Atmospheric Administration, National Marine Fisheries Service, Southeast Fisheries Science Center: Miami, FL. NOAA Technical Memorandum NMFS-SEFSC-527. 57 p.

Garrison, L.P. and L. Stokes. 2010. Estimated bycatch of marine mammals and turtles in the U.S. Atlantic pelagic longline fleet during 2009. National Oceanic and Atmospheric Administration, National Marine Fisheries Service, Southeast Fisheries Science Center: Miami, FL. NOAA Technical Memorandum NMFS-SEFSC-607. 64 p.

Garrison, L.P. and L. Stokes. 2012a. Estimated bycatch of marine mammals and sea turtles in the U.S. Atlantic pelagic longline fleet during 2010. National Oceanic and Atmospheric Administration, National Marine Fisheries Service, Southeast Fisheries Science Center: Miami, FL. NOAA Technical Memorandum NMFS-SEFSC-624. 59 p.

Garrison, L.P. and L. Stokes. 2012b. Estimated bycatch of marine mammals and sea turtles in the U.S. Atlantic pelagic longline fleet during 2011. National Oceanic and Atmospheric Administration, National Marine Fisheries Service, Southeast Fisheries Science Center: Miami, FL. NOAA Technical Memorandum NMFS-SEFSC-632. 61 p.

Garrison, L.P. and L. Stokes. 2013. Estimated bycatch of marine mammals and sea turtles in the U.S. Atlantic pelagic longline fleet during 2012. National Oceanic and Atmospheric Administration, National Marine Fisheries Service, Southeast Fisheries Science Center: Miami, FL. NOAA Technical Memorandum NMFS-SEFSC-655. 62 p.

Garrison, L.P. and L. Stokes. 2014. Estimated bycatch of marine mammals and sea turtles in the U.S. Atlantic pelagic longline fleet during 2013. National Oceanic and Atmospheric Administration, National Marine Fisheries Service, Southeast Fisheries Science Center: Miami, FL. NOAA Technical Memorandum NMFS-SEFSC-667. 61 p.

Garrison, L.P. and L. Stokes. 2016. Estimated bycatch of marine mammals and sea turtles in the U.S. Atlantic pelagic longline fleet during 2014. National Oceanic and Atmospheric Administration, National Marine Fisheries Service, Southeast Fisheries Science Center: Miami, FL. NOAA Technical Memorandum NMFS-SEFSC-696. 62 p. doi: 10.7289/V5/TM-SEFSC-696

Garrison, L.P. and L. Stokes. 2017. Estimated bycatch of marine mammals and sea turtles in the U.S. Atlantic pelagic longline fleet during 2015. National Oceanic and Atmospheric Administration, National Marine Fisheries Service, Southeast Fisheries Science Center: Miami, FL. NOAA Technical Memorandum NMFS-SEFSC-709. 61 p. doi: 10.25923/kaf6-5c33

Garrison, L.P., L. Stokes, and C. Fairfield. 2009. Estimated bycatch of marine mammals and sea turtles in the U.S. Atlantic pelagic longline fleet during 2008. National Oceanic and Atmospheric Administration, National Marine Fisheries Service, Southeast Fisheries Science Center: Miami, FL. NOAA Technical Memorandum NMFS-SEFSC-591. 63 p.

Grigg, E. and H. Markowitz. 1997. Habitat use by bottlenose dolphins (*Tursiops truncatus*) at Turneffe Atoll, Belize. Aquatic Mammals 23:163–170.

Hayes, S.A., E. Josephson, K. Maze-Foley, P.E. Rosel, and J. Wallace. 2022. U.S. Atlantic and Gulf of Mexico marine mammal stock assessments 2021. National Oceanic and Atmospheric Administration, National Marine Fisheries Service, Northeast Fisheries Science Center: Woods Hole, MA. NOAA Technical Memorandum NMFS-NE-288. 380 p. doi: 10.25923/6tt7-kc16

Henderson, E.E. and B. Würsig. 2007. Behavior patterns of bottlenose dolphins in San Luis Pass, Texas. Gulf of Mexico Science 25(2):153–161. doi: 10.18785/goms.2502.06

Hohn, A.A., M.D. Scott, R.S. Wells, J.C. Sweeney, and A.B. Irvine. 1989. Growth layers in teeth from known-age, free-ranging bottlenose dolphins. Marine Mammal Science 5(4):315–342. doi: 10.1111/j.1748-7692.1989.tb00346.x

Hoyt, E. and G.T. Hvenegaard. 2002. A review of whale-watching and whaling with applications for the Caribbean. Coastal Management 30(4):381–399. doi: 10.1080/089207502900273

Hubard, C.W., K. Maze-Foley, K.D. Mullin, and W.W. Schroeder. 2004. Seasonal abundance and site fidelity of bottlenose dolphins (*Tursiops truncatus*) in Mississippi Sound. Aquatic Mammals 30(2):299–310. doi: 10.1578/am.30.2.2004.299

Irwin, L.-J. and B. Würsig. 2004. A small resident community of bottlenose dolphins, *Tursiops truncatus*, in Texas: Monitoring recommendations. Gulf of Mexico Science 22(1):13–21. doi: 10.18785/goms.2201.02

Jefferson, T.A. and S.K. Lynn. 1994. Marine mammal sightings in the Caribbean Sea and Gulf of Mexico, summer 1991. Caribbean Journal of Science 30(1-2):83–89.

Jérémie, S., A. Gannier, S. Bourreau, and J.-C. Nicolas. 2006. Cetaceans of Martinique Island (Lesser Antilles): Occurrence and distribution obtained from a small boat dedicated survey. Paper SC/58/SM23. 58th Annual Meeting of the International Whaling Commission, June 2006, St. Kitts and Nevis, West Indies.

Kerr, K.A., R.H. Defran, and G.S. Campbell. 2005. Bottlenose dolphins (*Tursiops truncatus*) in the Drowned Cayes, Belize: Group size, site fidelity and abundance. Caribbean Journal of Science 41(1):172–177.

Kucklick, J., L. Schwacke, R. Wells, A. Hohn, A. Guichard, J. Yordy, L. Hansen, E. Zolman, R. Wilson, J. Litz, D. Nowacek, T. Rowles, R. Pugh, B. Balmer, C. Sinclair, and P. Rosel. 2011. Bottlenose dolphins as indicators of persistent organic pollutants in the western North Atlantic Ocean and Northern Gulf of Mexico. Environmental Science & Technology 45(10):4270–4277. doi: 10.1021/es1042244

LaBrecque, E., C. Curtice, J. Harrison, S.M. Van Parijs, and P.N. Halpin. 2015. 3. Biologically Important Areas for cetaceans within U.S. Waters – Gulf of Mexico Region. Aquatic Mammals 41(1):30–38. doi: 10.1578/am.41.1.2015.30

Leatherwood, S. 1977. Some preliminary impressions on the numbers and social behavior of free-swimming bottlenosed dolphin calves (*Tursiops truncatus*) in the northern Gulf of Mexico. In: Ridgway, S.H. and K. Bernirschke (Eds.), Breeding Dolphins; Present Status, Suggestions for the Future. Final report to the US Marine Mammal Commission, Contract MM6AC009, MMC-76/07, Washington, DC. pp. 143–167.

Litz, J.A. 2007. Social structure, genetic structure, and persistent organohalogen pollutants in bottlenose dolphins (*Tursiops truncatus*) in Biscayne Bay, Florida. University of Miami. 140 p.

Lynn, S.K. and B. Würsig. 2002. Summer movement patterns of bottlenose dolphins in a Texas bay. Gulf of Mexico Science 20(1):25–37. doi: 10.18785/goms.2001.03

Mackey, A.D. 2010. Site fidelity and association patterns of bottlenose dolphins (*Tursiops truncatus*) in the Mississippi Sound. University of Southern Mississippi. Thesis. 133 p.

Mann, J., R.C. Connor, L.M. Barre, and M.R. Heithaus. 2000. Female reproductive success in bottlenose dolphins (*Tursiops* sp.): life history, habitat, provisioning, and group-size effects. Behavioral Ecology 11(2):210–219. doi: 10.1093/beheco/11.2.210

Mattson, M.C., K.D. Mullin, G.W. Ingram, and W. Hoggard. 2006. Age structure and growth of the bottlenose dolphin (*Tursiops truncatus*) from strandings in the Mississippi sound region of the north-central Gulf of Mexico from 1986 to 2003. Marine Mammal Science 22(3):654–666. doi: 10.1111/j.1748-7692.2006.00057.x

Maze, K.S. and B. Würsig. 1999. Bottlenosed dolphins of San Luis Pass, Texas: Occurrence patterns, site-fidelity, and habitat use. Aquatic Mammals 25:91–104.

Mazzoil, M., S.D. McCulloch, and R.H. Defran. 2005. Observations on the site fidelity of bottlenose dolphins (*Tursiops truncatus*) in the Indian River Lagoon, Florida. Florida Scientist 68(4):217–226.

Mazzoil, M., J.S. Reif, M. Youngbluth, M.E. Murdoch, S.E. Bechdel, E. Howells, S.D. McCulloch, L.J. Hansen, and G.D. Bossart. 2008. Home ranges of bottlenose dolphins (*Tursiops truncatus*) in the Indian River Lagoon, Florida: Environmental correlates and implications for management strategies. Ecohealth 5(3):278–288. doi: 10.1007/s10393-008-0194-9

Mazzoil, M.S., S.D. McCulloch, M.J. Youngbluth, D.S. Kilpatrick, M.E. Murdoch, B. Mase-Guthrie, D.K. Odell, and G.D. Bossart. 2008. Radio-tracking and survivorship of two rehabilitated bottlenose dolphins (*Tursiops truncatus*) in the Indian River Lagoon, Florida. Aquatic Mammals 34:54–64. doi: 10.1578/AM.34.1.2008.54

McClellan, D.B., J. Browder, J.L. Tobias, G.J. Konoval, M.D. Heron, O.L. Bass, and J. Osborne. 2000. Opportunistic sightings of bottlenose dolphin, *Tursiops truncatus*, along the southeast Florida coast and Florida Bay, 1992-1997. National Oceanic and Atmospheric Administration, National Marine Fisheries Service, Southeast Fisheries Science Center: Miami, FL. NOAA Technical Memorandum NMFS-SEFSC-435. 18 p.

McDonald, T.L., F.E. Hornsby, T.R. Speakman, E.S. Zolman, K.D. Mullin, C. Sinclair, P.E. Rosel, L. Thomas, and L.H. Schwacke. 2017. Survival, density, and abundance of common bottlenose dolphins in Barataria Bay (USA) following the Deepwater Horizon oil spill. Endangered Species Research 33:193–209. doi: 10.3354/esr00806

McHugh, M.B. 1989. Population numbers and feeding behavior of the Atlantic bottlenose dolphin (*Tursiops truncatus*) near Aransas Pass, Texas. University of Texas at Austin. Thesis. 96 p.

Mead, J.G. and C.W. Potter. 1990. Natural history of bottlenose dolphins along the central Atlantic coast of the United States. In: Leatherwood, S. and R.R. Reeves (Eds.), The Bottlenose Dolphin. Academic Press, San Diego, CA. pp. 165–195.

Mignucci-Giannoni, A.A. 1989. Zoogeography of marine mammals in Puerto Rico and the Virgin Islands. University of Rhode Island. Thesis. 448 p.

Mignucci-Giannoni, A.A. 1998. Zoogeography of cetaceans off Puerto Rico and the Virgin Islands. Caribbean Journal of Science 34(3-4):173–190.

Mignucci-Giannoni, A.A., R.J. Rosario-Delestre, M.M. Alsina-Guerrero, L. Falcon-Matos, L. Guzman-Ramirez, E.H. Williams, Jr., G.D. Bossart, and J.S. Reidenberg. 2009. Asphyxiation in a bottlenose dolphin (*Tursiops truncatus*) from Puerto Rico due to choking on a black margate (*Anisotremus surinamensis*). Aquatic Mammals 35(1):48–54. doi: 10.1578/AM.35.1.2009.48

Miller, C.E. 2003. Abundance trends and environmental habitat usage patterns of bottlenose dolphins (*Tursiops truncatus*) in lower Barataria and Caminada bays, Louisiana. Louisiana State University and Agricultural & Mechanical College. Dissertation. 125 p.

Miller, L.J., A.D. Mackey, T. Hoffland, M. Solangi, and S.A. Kuczaj. 2010. Potential effects of a major hurricane on Atlantic bottlenose dolphin (*Tursiops truncatus*) reproduction in the Mississippi Sound. Marine Mammal Science 26(3):707–715. doi: 10.1111/j.1748-7692.2010.00371.x

Miller, L.J., A.D. Mackey, M. Solangi, and S.A. Kuczaj. 2013. Population abundance and habitat utilization of bottlenose dolphins in the Mississippi Sound. Aquatic Conservation: Marine and Freshwater Ecosystems 23(1):145–151. doi: 10.1002/aqc.2278

Mohammed, E., L.E. Straker, and C. Jardine. 2003. St. Vincent and the Grenadines: Reconstructed fisheries catches and fishing effort, 1942-2001. Fisheries Centre Research Reports 11(6):95–116.

Mullin, K.D., T. McDonald, R.S. Wells, B.C. Balmer, T. Speakman, C. Sinclair, E.S. Zolman, F. Hornsby, S.M. McBride, K.A. Wilkinson, and L.H. Schwacke. 2017. Density, abundance, survival, and ranging patterns of common bottlenose dolphins (*Tursiops truncatus*) in Mississippi Sound following the Deepwater Horizon oil spill. PLoS ONE 12(10):e0186265. doi: 10.1371/journal.pone.0186265

Odell, D.K. and E.D. Asper. 1990. Distribution and movements of freeze-branded bottlenose dolphins in the Indian and Banana Rivers, Florida. In: Leatherwood, S. and R. Reeves (Eds.), The Bottlenose Dolphin. Academic Press, San Diego, CA. pp. 515–540.

Oviedo, L., N. Silva, L. Bermudez, and D.K. Odell. 2005. Distribution of bottlenose dolphins (*Tursiops truncatus*) on the east coast of Isla Margarita and the Los Frailes Archipelago, Venezuela. Aquatic Mammals 31(4):442–446. doi: 10.1578/AM.31.4.2005.442

Pardo, M.A., A. Mejía-Fajardo, S. Beltrán-Pedreros, F. Trujillo, I. Kerr, and D.M. Palacios. 2009. Odontocete sightings collected during offshore cruises in the western and southwestern Caribbean Sea. Latin American Journal of Aquatic Mammals 7(1-2):57–62. doi: 10.5597/lajam00135

Pardo, M.A. and D.M. Palacios. 2006. Cetacean occurrence in the Santa Marta region, Colombian Caribbean, 2004-2005. Latin American Journal of Aquatic Mammals 5(2):129–134. doi: 10.5597/lajam00105

Parsons, E.C.M., I. Bonnelly de Calventi, A. Whaley, N.A. Rose, and S. Sherwin. 2010. A note on illegal captures of wild bottlenose dolphins (*Tursiops truncatus*) from the coastal waters of the Dominican Republic. Journal of International Wildlife Law and Policy 13(3):240–244. doi: 10.1080/13880292.2010.503123

Perrin, W.F. and S.B. Reilly. 1984. Reproductive parameters of dolphins and small whales of the family Delphinidae. In: Perrin, W.F., R.L. Brownell, Jr., and D.P. DeMaster (Eds.), Reproduction in Whales, Dolphins, and Porpoises. pp. 97–134.

Price, W.S. 1985. Whaling in the Caribbean: Historical perspective and update. Reports of the International Whaling Commission 35:413–420.

Pulster, E.L. and K.A. Maruya. 2008. Geographic specificity of Aroclor 1268 in bottlenose dolphins (*Tursiops truncatus*) frequenting the Turtle/Brunswick River Estuary, Georgia (USA). Science of the Total Environment 393(2-3):367–375. doi: 10.1016/j.scitotenv.2007.12.031

Reeves, R. 1988. Exploitation of cetaceans in St. Lucia, Lesser Antilles, January 1987. Reports of the International Whaling Commission 38(445-447).

Rodríguez-Ferrer, G. 2001. The bottlenose dolphin (*Tursiops truncatus* Montague 1821) of the southwest coast of Puerto Rico: An assessment of its status and habitat use. University of Puerto Rico. Thesis.

Rodriguez-Ferrer, G., J.J. Cruz-Motta, N.V. Schizas, and R.S. Appeldoorn. 2020. Modelling distribution of the common bottlenose dolphin, *Tursiops truncatus* off the southwest coast of Puerto Rico. Journal of Marine Systems 210:103371. doi: 10.1016/j.jmarsys.2020.103371

Romero, A., A.I. Agudo, S.M. Green, and G. Notarbartolo di Sciara. 2001. Cetaceans of Venezuela: Their distribution and conservation status. National Oceanic and Atmospheric Administration, National Marine Fisheries Service: Seattle, WA. NOAA Technical Report NMFS 151. 60 p.

Romero, A., R. Baker, and J.E. Creswell. 2002. Environmental history of marine mammal exploitation in Trinidad and Tobago, W.I., and its ecological impact. Environment and History 8:255–274.

Rossman, S., P.H. Ostrom, M. Stolen, N.B. Barros, H. Gandhi, C.A. Stricker, and R.S. Wells. 2015. Individual specialization in the foraging habits of female bottlenose dolphins living in a trophically diverse and habitat rich estuary. Oecologia 178(2):415–425. doi: 10.1007/s00442-015-3241-6

Sergeant, D.E., D.K. Caldwell, and M.C. Caldwell. 1973. Age, growth, and maturity of bottlenosed dolphin (*Tursiops-truncatus*) from northeast Florida. Journal of the Fisheries Research Board of Canada 30(7):1009–1011. doi: 10.1139/f73-165

Shane, S.H., R.S. Wells, and B. Würsig. 1986. Ecology, behavior and social organization of the bottlenose dolphin: A review. Marine Mammal Science 2:34–63. doi: 10.1111/j.1748-7692.1986.tb00026.x

Sloan, P.E. 2006. Residency patterns, seasonality and habitat use among bottlenose dolphins, *Tursiops truncatus*, in the Cape Romain National Wildlife Refuge, S.C. University of North Carolina Wilmington. Thesis. 75 p.

Taruski, A.G. and H.E. Winn. 1976. Winter sightings of odontocetes in the West Indies. Cetology 22:1–12.

Taylor, B.L., S.J. Chivers, J. Larese, and W.F. Perrin. 2007. Generation length and percent mature estimates for IUCN assessments of cetaceans. National Oceanic and Atmospheric Administration, National Marine Fisheries Service, Southwest Fisheries Science Center: La Jolla, CA. Administrative Report LJ-07-01. 24 p.

Torres, L.G. 2007. Top predator distribution and foraging ecology in Florida Bay, Florida. Duke University. Dissertation. 225 p.

Torres, L.G. and A.J. Read. 2009. Where to catch a fish? The influence of foraging tactics on the ecology of bottlenose dolphins (*Tursiops truncatus*) in Florida Bay, Florida. Marine Mammal Science 25(4):797–815. doi: 10.1111/j.1748-7692.2009.00297.x

Tyson, R.B., S.M. Nowacek, and D.P. Nowacek. 2011. Community structure and abundance of bottlenose dolphins *Tursiops truncatus* in coastal waters of the northeast Gulf of Mexico. Marine Ecology Progress Series 438:253–265. doi: 10.3354/meps09292

Urian, K.W., S. Hofmann, R.S. Wells, and A.J. Read. 2009. Fine-scale population structure of bottlenose dolphins (*Tursiops truncatus*) in Tampa Bay, Florida. Marine Mammal Science 25(3):619–638. doi: 10.1111/j.1748-7692.2009.00284.x

Vail, C. 2005. Socio-economic assessment of marine mammal utilization in the wider Caribbean region: Captivity, viewing and hunting. Information paper presented at the United Nations Environment Programme Regional Workshop of Experts on the Development of the Marine Mammal Action Plan for the Wider Caribbean Region, Barbados, 18-21 July 2005.

van Bree, P.J.H. 1975. Preliminary list of the cetaceans of the southern Caribbean. Stud. Fauna Curacao Carib. Isl 48:79–87.

van Waerebeek, K., M. Sequeira, C. Williamson, G.P. Sanino, P. Gallego, and P. Carmo. 2006. Live-captures of common bottlenose dolphins *Tursiops truncatus* and unassessed bycatch in Cuban waters: Evidence of sustainability found wanting. Latin American Journal of Aquatic Mammals 5(1):39–48. doi: 10.5597/lajam00090

Vollmer, N.L. and P.E. Rosel. 2013. A review of common bottlenose dolphins (*Tursiops truncatus truncatus*) in the Northern Gulf of Mexico: Population biology, potential threats, and management. Southeastern Naturalist 12(m6):1–43. doi: 10.1656/058.012.m601

Waring, G.T., E. Josephson, K. Maze-Foley, and P.E. Rosel. 2012. U.S. Atlantic and Gulf of Mexico marine mammal stock assessments - 2011. National Oceanic and Atmospheric Administration, National Marine Fisheries Service, Northeast Fisheries Science Center: Woods Hole, MA. NOAA Technical Memorandum NMFS-NE-221. 319 p.

Waring, G.T., E. Josephson, K. Maze-Foley, and P.E. Rosel. 2016. U.S. Atlantic and Gulf of Mexico marine mammal stock assessments - 2015. National Oceanic and Atmospheric Administration, National Marine Fisheries Service, Northeast Fisheries Science Center: Woods Hole, MA. NOAA Technical Memorandum NMFS-NE-238. 501 p. doi: 10.7289/V57S7KTN

Wells, R.S. 1986. Population structure of bottlenose dolphins: Behavioral studies along the central west coast of Florida. Contract Report to National Marine Fisheries Service, Southeast Fisheries Center. Contract No. 45-WCNF-5-003BB. 58 p.

Wells, R.S. 1991. The role of long-term study in understanding the social structure of a bottlenose dolphin community. In: Pryor, K. and K. Norris (Eds.), Dolphin Societies: Discoveries and Puzzles. University of California Press, Berkeley, CA. pp. 199–226.

Wells, R.S. 2000. Reproduction in wild bottlenose dolphins: Overview of patterns observed during a long-term study. In: Duffield, D. and T. Robeck (Eds.), Bottlenose Dolphin Reproduction Workshop. AZA Marine Mammal Taxon Advisory Group, Silver Spring, MD. pp. 57–74.

Wells, R.S. 2003. Dolphin social complexity: Lessons from long-term study and life history. In: de Waal, F.B.M. and P.L. Tyack (Eds.), Animal Social Complexity: Intelligence, Culture, and Individualized Societies Harvard University Press, Cambridge, MA. pp. 32–56.

Wells, R.S. 2014. Social structure and life history of bottlenose dolphins near Sarasota Bay, Florida: Insights from four decades and five generations. Primates and cetaceans. pp. 149–172. doi: 10.1007/978-4-431-54523-1_8

Wells, R.S., M.K. Bassos, K.W. Urian, W.J. Carr, and M.D. Scott. 1996. Low-level monitoring of bottlenose dolphins, *Tursiops truncatus*, in Charlotte Harbor, Florida: 1990-1994. National Oceanic and Atmospheric Administration, National Marine Fisheries Service, Southeast Fisheries Science Center: Miami, FL. NOAA Technical Memorandum NMFS-SEFSC-384. 86 p.

Wells, R.S., A. Natoli, and G. Braulik. 2019. *Tursiops truncatus* (errata version published in 2019). *The IUCN Red List of Threatened Species* 2019: e.T22563A156932432. https://dx.doi.org/10.2305/IUCN.UK.2019-1.RLTS.T22563A156932432.en

Wells, R.S., L.H. Schwacke, T.K. Rowles, B.C. Balmer, E. Zolman, T. Speakman, F.I. Townsend, M.C. Tumlin, A. Barleycorn, and K.A. Wilkinson. 2017. Ranging patterns of common bottlenose dolphins *Tursiops truncatus* in Barataria Bay, Louisiana, following the Deepwater Horizon oil spill. Endangered Species Research 33:159–180. doi: 10.3354/esr00732

Wells, R.S. and M.D. Scott. 1999. Bottlenose dolphin *Tursiops truncatus* (Montagu, 1821). In: Ridgway, S.H. and R. Harrison (Eds.), Handbook of marine mammals. Volume 6: The second book of dolphins and the porpoises. Academic Press, San Diego, CA. pp. 137–182.

Wells, R.S. and M.D. Scott. 2018. Bottlenose dolphin, *Tursiops truncatus*, common bottlenose dolphin. In: Würsig, B., J.G.M. Thewissen, and K.M. Kovacs (Eds.), Encyclopedia of Marine Mammals (Third Edition). Academic Press, London, UK. pp. 118–125. doi: 10.1016/B978-0-12-804327-1.00072-8

Wells, R.S., M.D. Scott, and A.B. Irvine. 1987. The social structure of free-ranging bottlenose dolphins. In: Genoways, H.H. (Ed.), Current Mammalogy. Plenum Press, New York, NY. pp. 247–305.

Whaley, A.R., E.C.M. Parsons, R. Sellares, and I. de C. Bonnelly. 2006. Dolphin ecology and behavior in the southeastern waters of the Dominican Republic: Preliminary observations. 58th Annual Meeting of the International Whaling Commission: St. Kitts and Nevis. SC/58/SM12. 8 p.

Whitall, D.R., A.L. Mason, M. Fulton, E. Wirth, D. Wehner, A. Ramos-Alvarez, A.S. Pait, B. West, E. Pisarski, B. Shaddrix, and L. Reed. 2016. Contaminants in marine resources of Vieques, Puerto Rico. NOAA's National Centers for Coastal Ocean Science Office of Response and Restoration and Coral Reef Conservation Program and Coral Reef Conservation Program: Silver Spring, MD. NOAA Technical Memorandum NOS-NCCOS-223. 70 p. doi: 10.7289/V5/TM-NOS-NCCOS-223

Whitt, A.D., T.A. Jefferson, M. Blanco, D. Fertl, and D. Rees. 2011. A review of marine mammal records of Cuba. Latin American Journal of Aquatic Mammals 9(2):65–122. doi: 10.5597/lajam00175

Wilson, R.M., R.B. Tyson, J.A. Nelson, B.C. Balmer, J.P. Chanton, and D.P. Nowacek. 2017. Niche differentiation and prey selectivity among common bottlenose dolphins (*Tursiops truncatus*) sighted in St. George Sound, Gulf of Mexico. Frontiers in Marine Science 4:235. doi: 10.3389/fmars.2017.00235

World Council of Whalers. 2008. World whaling – Caribbean. http://www.worldwhalers.com/whaling_around_the_world/caribbean.htm

Yoshida, H., J. Compton, S. Punnett, T. Lovell, K. Draper, G. Franklin, N. Norris, P. Phillip, R. Wilkins, and H. Kato. 2010. Cetacean sightings in the eastern Caribbean and adjacent waters, spring 2004. Aquatic Mammals 36(2):154–161. doi: 10.1578/Am.36.2.2010.154

Common bottlenose dolphin − *Tursiops truncatus*

Southern Georgia Estuarine System Stock

Overall Vulnerability Rank = Very High

Biological Sensitivity = High

Climate Exposure = Very High

Data Quality = 96% of scores ≥ 2

	Tursiops truncatus	Expert Scores	Data Quality	Expert Scores Plots (Portion by Category)
Sensitivity attributes	Prey/Diet Specificity	1.9	2.0	
	Habitat Specificity	3.4	2.0	
	Site Fidelity	4.0	2.7	
	Lifetime Reproductive Potential	1.3	2.0	
	Generation Length	2.9	2.0	
	Reproductive Plasticity	1.1	2.3	
	Migration	4.0	2.3	
	Home Range	3.5	2.3	
	Stock Abundance	3.0	2.7	
	Stock Abundance Trend	2.3	1.3	
	Cumulative Stressors	2.7	2.3	
	Sensitivity Score	High		
Exposure variables	Sea Surface Temperature (Change in mean)	4.0	3.0	
	Sea Surface Temperature (Change in variability)	1.0	3.0	
	Air Temperature (Change in mean)	4.0	3.0	
	Air Temperature (Change in variability)	1.0	3.0	
	Precipitation (Change in mean)	1.0	3.0	
	Precipitation (Change in variability)	1.7	3.0	
	Sea Surface Salinity (Change in mean)	1.8	3.0	
	Sea Surface Salinity (Change in variability)	1.0	3.0	
	Ocean pH (Change in mean)	4.0	3.0	
	Ocean pH (Change in variability)	1.1	3.0	
	Sea ice coverage (Change in mean)	1.0	3.0	
	Sea ice coverage (Change in variability)	1.0	3.0	
	Dissolved oxygen (Change in mean)	4.0	3.0	
	Dissolved oxygen (Change in variability)	1.1	3.0	
	Circulation	3.9	2.0	
	Sea level rise	4.0	3.0	
	Exposure Score	Very High		
Overall Vulnerability Rank		Very High		

Low
Moderate
High
Very High

Common bottlenose dolphin (Southern Georgia Estuarine System Stock)

Tursiops truncatus

CVA Results Summary

Overall Climate Vulnerability Rank: Very High (100% certainty from bootstrap analysis).

Climate Exposure: Very High. Six exposure factors scored greater than 3.5: Air Temperature (Standard anomaly) (4.00), Dissolved oxygen (Standard anomaly) (4.00), Ocean pH (Standard anomaly) (4.00), Sea level rise (4.00), Sea Surface Temperature (Standard anomaly) (4.00), and Circulation (3.93).

Biological Sensitivity: High. Five sensitivity attributes scored greater than or equal to 3.0: Migration (4.00), Site Fidelity (4.00), Home Range (3.47), Habitat Specificity (3.40), and Species Abundance (3.00).

Distributional Response: High

Abundance Response: High

Phenology Response: High

Data Quality: 96% of the data quality scores were 2 or greater, 91% of sensitivity attribute data quality scores were 2 or higher.

Background Information

Stock Description

The Southern Georgia Estuarine System Stock (SGES) Stock includes common bottlenose dolphins found in estuarine waters of Georgia from the Altamaha River in the north to the Florida/Georgia border at Cumberland Sound in the south (Waring et al. 2016).

Prey/Diet Specificity

Generally, common bottlenose dolphins consume a large variety of fish and/or squid (Barros and Odell 1990; Mead and Potter 1990; Wells and Scott 2018). Common bottlenose dolphins seem to show a consistent preference for sound-producing fishes (e.g., sciaenids, toadfish; Berens McCabe et al. 2010; Bowen-Stevens et al. 2021). Estuarine stocks along the southeast U.S. Atlantic coast have been reported to consume a variety of prey, with preference for sciaenids (Gannon and Waples 2004; Pate and McFee 2012). In Sarasota Bay, Florida, Rossman et al. (2015) found some individual common bottlenose dolphins to show diet specialization despite the broader study group showing a generalist diet. Specialization can be influenced by locally abundant prey species (Shane et al. 1986; Vollmer and Rosel 2013) or can result from resource partitioning amongst sympatric populations (e.g., Torres and Read 2009; Wilson et al. 2017).

Habitat Specificity

Common bottlenose dolphins are found in a variety of marine and estuarine habitats (Wells and Scott 2018). The area inhabited by the SGES Stock is characterized by extensive tidal marshes and shallow riverine systems (Waring et al. 2016).

Site Fidelity

Common bottlenose dolphins in the SGES Stock show evidence of site fidelity (Balmer et al. 2011; Balmer et al. 2013). Other estuarine stocks in the western North Atlantic have shown site fidelity (Waring et al. 2016), such as in South Carolina (Sloan 2006; Speakman et al. 2006; Waring et al. 2016), central Georgia (Kucklick et al. 2011), northern Florida (Caldwell 2001), central Florida (Odell and Asper 1990; Mazzoil et al. 2005, 2008a, 2008b), and southern Florida (Litz 2007; Torres 2007; McClellan et al. 2000).

Lifetime Reproductive Potential

Taylor et al. (2007) reported the interbirth interval of common bottlenose dolphins as 3.80 years, though other studies have shown it to be closer to three years (Leatherwood 1977; Wells and Scott 1999; Connor et al. 2000; Wells 2003). Thayer (2008) estimated mean interbirth interval of 2.9 years in North Carolina.

Generally, females reach sexual maturity before males, with females reaching sexual maturity at 5–13 years (Sergeant et al. 1973; Perrin and Reilly 1984; Wells et al. 1987; Mead and Potter 1990; Mann et al. 2000; Wells 2000; Wells and Scott 2018).

Females reproduce through most of their adult lives (Leatherwood 1977; Hohn et al. 1989; Wells and Scott 1999; Wells 2000; Wells 2003), but may produce fewer calves after they reach 30 years of age (Wells 2000). Sarasota Bay common bottlenose dolphins can give birth when they are as young as 6 years of age, and can continue until they are 48 years old (Wells and Scott 2018). Reproductive lifespan would therefore be up to 42 years based on long-term observation studies (Wells 2014; Wells and Scott 2018).

Longitudinal studies from Sarasota Bay, Florida have found that female bottlenose dolphins can live up to 67 years (Wells and Scott 2018). Studies based on growth layer groups in teeth suggest female bottlenose dolphins can live to 41 years along the Texas coast (Fernandez and Hohn 1998) and to 30 years along the Mississippi coast (Mattson et al. 2006).

Generation Length

Taylor et al. (2007) reported common bottlenose dolphin generation length of 20.6–21.1 years.

Reproductive Plasticity

Common bottlenose dolphin calving is generally diffusely seasonal, with one or more peaks, although births have been reported from all seasons (Vollmer and Rosel 2013; Wells and Scott 2018). Calf sightings in South Carolina peak in spring and early summer and again in early autumn (McFee et al. 2014). In North Carolina, Thayer et al. (2003) observed a strong birth peak in spring based on stranding

data and a diffuse birth peak from late spring to early fall based on sightings. Calf sightings peak in the spring and summer months in Florida (Wells et al. 1987), Mississippi/Louisiana (Miller et al. 2010; Miller et al. 2013), and Texas (McHugh 1989; Henderson and Würsig 2007).

Migration

The estuarine stocks in the western North Atlantic are thought to primarily comprise stable resident communities. However, transient or seasonal movements of some individuals into and out of stock areas are recorded in some areas (LaBrecque et al. 2015; Waring et al. 2016). Other estuarine stocks in the western North Atlantic are known to spend warm months in estuarine waters then move to nearshore waters during cool months (Gubbins et al. 2003; Waring et al. 2014; Waring et al. 2016).

Home Range

The home ranges of individuals within the western North Atlantic estuarine stocks are considered to generally include the areas within the stock boundaries and brief excursions beyond the stock boundaries (reviewed in LaBrecque et al. 2015). Studies have shown dolphin populations with constrained ranges in Georgia (Brunswick and Sapelo; Balmer et al. 2013); South Carolina (Silva et al. 2019), and North Carolina (Urian et al. 2013). Balmer et al. (2018), using satellite telemetry data, identified that dolphins considered to be members of the SGES Stock had different movement patterns with some individuals remaining entirely within the estuarine waters, while other animals use the larger estuarine waters, sounds, and to some degree coastal waters.

Stock Abundance

Balmer et al. (2013) estimated the abundance of a portion of the SGES stock at 194 individuals (CV=0.05) based on winter 2008 and winter 2009 surveys. However, this estimate did not cover the entire range of the stock, and may represent an undercount (Waring et al. 2016).

Stock Abundance Trend

Data are insufficient to complete an abundance trend analysis for this stock (Waring et al. 2016).

Cumulative Stressors

Common bottlenose dolphins face a wide range of individual and cumulative natural and anthropogenic threats (see reviews by Vollmer and Rosel 2013; Wells and Scott 2018; and Wells et al. 2019). These include such threats as shark predation, harmful algal blooms, recreational and commercial fishing gear, vessel traffic, pollution, habitat alteration, and provisioning.

Common bottlenose dolphins in the SGES Stock interact with the blue crab trap/pot fishery (Waring et al. 2016). Individuals of this stock have been reported stranded, with some strandings showing evidence of human interactions, and other strandings occurring during Unusual Mortality Events (UMEs; also see NOAA National Marine Mammal Health and Stranding Response Database). For example, During the period 2009–2013, 31 common bottlenose dolphins stranded within the SGES, with no individuals presenting clear indications of human interactions such as fishing gear and vessel interactions (NOAA National Marine Mammal Health and Stranding Response Database unpublished data, as cited by

Waring et al. 2016; Seguel et al. 2020). The cause of death for six other stranded individuals was likely high concentrations of persistent organic pollutants (POPs; Seguel et al. 2020).

An Unusual Mortality Event (UME) was declared in the summer of 2013 for the mid-Atlantic coast from New York to Brevard County, Florida, with more than 1500 common bottlenose dolphins stranding as a result of morbillivirus (Waring et al. 2016). Strandings were more prevalent along oceanic shores than in estuaries, suggesting that coastal stocks were more impacted by the UME than estuarine stocks (Waring et al. 2016).

The nearshore and estuarine habitats along the Atlantic seaboard are adjacent to areas of high human population and some are highly industrialized. Studies of bottlenose dolphin tissues from estuaries in the southeast U.S. have shown persistent organic pollutant (POP; Schwacke et al. 2002, 2012; Hansen et al. 2004; Litz et al. 2007; Pulster et al. 2009; Fair et al. 2010; Yordy et al. 2010; Balmer et al. 2011; Kucklick et al. 2011; Reif et al. 2017) and heavy metal (Durden et al. 2007; Stavros et al. 2007, 2008, 2011; Titcomb et al. 2017; Reif et al. 2017; Page-Karjian et al. 2020) concentrations that may result in adverse effects on health or reproductive rates, particularly in urbanized, developed, and agricultural areas. In Biscayne Bay, Florida, elevated POP concentrations were observed in individuals associated with more polluted areas compared to individuals associated with the less polluted areas of Biscayne Bay, suggesting that exposure to pollutants may be highly variable based on geography (Litz et al. 2007; Kucklick et al. 2011). The SGES Stock inhabits a highly industrialized area that includes four Environmental Protection Agency declared Superfund sites (EPA 2021). Analyses of contaminants have found elevated polychlorinated biphenyl (PCB) concentrations in dolphins in the Turtle/Brunswick River Estuary relative to dolphins in other areas (Pulster and Maryua 2008; Sanger et al. 2008; Pulster et al. 2009; Kucklick et al. 2011; Schwacke et al. 2012). Human feeding of dolphins has been observed in the SGES, with possible implications for increased interactions with fishing gear and behavioral adaptation (Kovacs and Cox 2014; Perrtree et al. 2014; Wu 2013).

Further Reading

Waring, G.T., E. Josephson, K. Maze-Foley, and P.E. Rosel. 2016. U.S. Atlantic and Gulf of Mexico Marine Mammal Stock Assessments - 2015. National Oceanic and Atmospheric Administration, National Marine Fisheries Service, Northeast Fisheries Science Center: Woods Hole, MA. NOAA Technical Memorandum NMFS-NE-238. 501 p. doi: 10.7289/V57S7KTN

Wells, R.S. and M.D. Scott. 2018. Bottlenose Dolphin, *Tursiops truncatus*, Common Bottlenose Dolphin. In: Würsig, B., J.G.M. Thewissen, and K.M. Kovacs (Eds.), Encyclopedia of Marine Mammals (Third Edition). Academic Press, London, UK. pp. 118–125. doi: 10.1016/B978-0-12-804327-1.00072-8

Wells, R.S., A. Natoli, and G. Braulik. 2019. *Tursiops truncatus* (errata version published in 2019). The IUCN Red List of Threatened Species 2019: e.T22563A156932432. https://dx.doi.org/10.2305/IUCN.UK.2019-1.RLTS.T22563A156932432.en

References

Balmer, B., E. Zolman, T. Rowles, C. Smith, F. Townsend, D. Fauquier, C. George, T. Goldstein, L. Hansen, B. Quigley, W. McFee, J. Morey, P. Rosel, J. Saliki, T. Speakman, and L. Schwacke. 2018. Ranging patterns, spatial overlap, and association with dolphin morbillivirus exposure in common

bottlenose dolphins (*Tursiops truncatus*) along the Georgia, USA coast. Ecology and Evolution 8(24):12890–12904. doi: 10.1002/ece3.4727

Balmer, B.C., L.H. Schwacke, R.S. Wells, J.D. Adams, R. Clay George, S.M. Lane, W.A. McLellan, P.E. Rosel, K. Sparks, T. Speakman, E.S. Zolman, and D. Ann Pabst. 2013. Comparison of abundance and habitat usage for common bottlenose dolphins between sites exposed to differential anthropogenic stressors within the estuaries of southern Georgia, U.S.A. Marine Mammal Science 29(2):E114–E135. doi: 10.1111/j.1748-7692.2012.00598.x

Balmer, B.C., L.H. Schwacke, R.S. Wells, R.C. George, J. Hoguet, J.R. Kucklick, S.M. Lane, A. Martinez, W.A. McLellan, P.E. Rosel, T.K. Rowles, K. Sparks, T. Speakman, E.S. Zolman, and D.A. Pabst. 2011. Relationship between persistent organic pollutants (POPs) and ranging patterns in common bottlenose dolphins (*Tursiops truncatus*) from coastal Georgia, USA. Science of the Total Environment 409(11):2094–2101. doi: 10.1016/j.scitotenv.2011.01.052

Barros, N.B. and D.K. Odell. 1990. Food habits of bottlenose dolphins in the southeastern United States In: Leatherwood, S. and R.R. Reeves (Eds.), The Bottlenose Dolphin. Academic Press, San Diego, CA. pp. 309–328. doi: 10.1016/B978-0-12-440280-5.50020-2

Berens McCabe, E.J., D.P. Gannon, N.B. Barros, and R.S. Wells. 2010. Prey selection by resident common bottlenose dolphins (*Tursiops truncatus*) in Sarasota Bay, Florida. Marine Biology 157(5):931–942. doi: 10.1007/s00227-009-1371-2

Bowen-Stevens, S.R., D.P. Gannon, R.A. Hazelkorn, G. Lovewell, K.M. Volker, S. Smith, M.C. Tumlin, and J. Litz. 2021. Diet of common bottlenose dolphins, *Tursiops truncatus*, that stranded in and near Barataria Bay, Louisiana, 2010–2012. Southeastern Naturalist 20(1). doi: 10.1656/058.020.0113

Caldwell, M. 2001. Social and genetic structure of bottlenose dolphin (*Tursiops truncatus*) in Jacksonville, Florida. University of Miami. 143 p.

Connor, R.C., R.S. Wells, J. Mann, and A.J. Read. 2000. The bottlenose dolphin: Social relationships in a fission-fusion society. In: Mann, J., R. Connor, P. Tyack, and H. Whitehead (Eds.), Cetacean Societies: Field Studies of Dolphins and Whales. University of Chicago Press, Chicago, IL. pp. 91–126.

Durden, W.N., M.K. Stolen, D.H. Adams, and E.D. Stolen. 2007. Mercury and selenium concentrations in stranded bottlenose dolphins from the Indian River Lagoon system, Florida. Bulletin of Marine Science 81(1):37–54.

Environmental Protection Agency (EPA). 2021. Superfund. https://www.epa.gov/superfund

Fair, P.A., J. Adams, G. Mitchum, T.C. Hulsey, J.S. Reif, M. Houde, D. Muir, E. Wirth, D. Wetzel, E. Zolman, W. McFee, and G.D. Bossart. 2010. Contaminant blubber burdens in Atlantic bottlenose dolphins (*Tursiops truncatus*) from two southeastern US estuarine areas: Concentrations and patterns of PCBs, pesticides, PBDEs, PFCs, and PAHs. Science of the Total Environment 408(7):1577–1597. doi: 10.1016/j.scitotenv.2009.12.021

Fernandez, S. and A.A. Hohn. 1998. Age, growth, and calving season of bottlenose dolphins, *Tursiops truncatus*, off coastal Texas. Fishery Bulletin 96(2):357–365.

Gannon, D.P. and D.M. Waples. 2004. Diets of coastal bottlenose dolphins from the U. S. Mid-Atlantic coast differ by habitat. Marine Mammal Science 20(3):527–545. doi: 10.1111/j.1748-7692.2004.tb01177.x

Gubbins, C.M., M. Caldwell, S.G. Barco, K.A. Rittmaster, N. Bowles, and V.G. Thayer. 2003. Abundance and sighting patterns of bottlenose dolphins (*Tursiops truncatus*) at four northwest Atlantic coastal sites. Journal of Cetacean Research and Management 5(2):141–148.

Hansen, L.J., L.H. Schwacke, G.B. Mitchum, A.A. Hohn, R.S. Wells, E.S. Zolman, and P.A. Fair. 2004. Geographic variation in polychorinated biphenyl and organochlorine pesticide concentrations in the blubber of bottlenose dolphins from the US Atlantic coast. Science of the Total Environment 319(1-3):147–172. doi: 10.1016/S0048-9697(03)00371-1

Henderson, E.E. and B. Würsig. 2007. Behavior patterns of bottlenose dolphins in San Luis Pass, Texas. Gulf of Mexico Science 25(2):153–161. doi: 10.18785/goms.2502.06

Hohn, A.A., M.D. Scott, R.S. Wells, J.C. Sweeney, and A.B. Irvine. 1989. Growth layers in teeth from known-age, free-ranging bottlenose dolphins. Marine Mammal Science 5(4):315–342. doi: 10.1111/j.1748-7692.1989.tb00346.x

Kovacs, C. and T. Cox. 2014. Quantification of interactions between common bottlenose dolphins (*Tursiops truncatus*) and a commercial shrimp trawler near Savannah, Georgia. Aquatic Mammals 40(1):81–94. doi: 10.1578/Am.40.1.2014.81

Kucklick, J., L. Schwacke, R. Wells, A. Hohn, A. Guichard, J. Yordy, L. Hansen, E. Zolman, R. Wilson, J. Litz, D. Nowacek, T. Rowles, R. Pugh, B. Balmer, C. Sinclair, and P. Rosel. 2011. Bottlenose dolphins as indicators of persistent organic pollutants in the western North Atlantic Ocean and Northern Gulf of Mexico. Environmental Science & Technology 45(10):4270–4277. doi: 10.1021/es1042244

LaBrecque, E., C. Curtice, J. Harrison, S.M. Van Parijs, and P.N. Halpin. 2015. 2. Biologically Important Areas for Cetaceans Within U.S. Waters – East Coast Region. Aquatic Mammals 41(1):17–29. doi: 10.1578/am.41.1.2015.17

Leatherwood, S. 1977. Some preliminary impressions on the numbers and social behavior of free-swimming bottlenosed dolphin calves (*Tursiops truncatus*) in the northern Gulf of Mexico. In: Ridgway, S.H. and K. Bernirschke (Eds.), Breeding Dolphins; Present Status, Suggestions for the Future. Final report to the US Marine Mammal Commission, Contract MM6AC009, MMC-76/07, Washington, DC. pp. 143–167.

Litz, J.A. 2007. Social structure, genetic structure, and persistent organohalogen pollutants in bottlenose dolphins (*Tursiops truncatus*) in Biscayne Bay, Florida. University of Miami. 140 p.

Litz, J.A., L.P. Garrison, L.A. Fieber, A. Martinez, J.P. Contillo, and J.R. Kucklick. 2007. Fine-scale spatial variation of persistent organic pollutants in bottlenose dolphins (*Tursiops truncatus*) in Biscayne Bay, Florida. Environmental Science & Technology 41(21):7222–7228. doi: 10.1021/es070440r

Mann, J., R.C. Connor, L.M. Barre, and M.R. Heithaus. 2000. Female reproductive success in bottlenose dolphins (*Tursiops* sp.): life history, habitat, provisioning, and group-size effects. Behavioral Ecology 11(2):210–219. doi: 10.1093/beheco/11.2.210

Mattson, M.C., K.D. Mullin, G.W. Ingram, and W. Hoggard. 2006. Age structure and growth of the bottlenose dolphin (*Tursiops truncatus*) from strandings in the Mississippi sound region of the north-central Gulf of Mexico from 1986 to 2003. Marine Mammal Science 22(3):654–666. doi: 10.1111/j.1748-7692.2006.00057.x

Maze-Foley, K. and L.P. Garrison. 2018. Serious injury determinations for small cetaceans off the southeast U.S. coast, 2012–2016. National Oceanic and Atmospheric Administration, National Marine Fisheries Service, Southeast Fisheries Science Center: Miami, FL. Southeast Fisheries Science Center Reference Document PRBD 2018-04. 31 p. doi: 10.25923/xhfz-5g76

Mazzoil, M., S.D. McCulloch, and R.H. Defran. 2005. Observations on the site fidelity of bottlenose dolphins (*Tursiops truncatus*) in the Indian River Lagoon, Florida. Florida Scientist 68(4):217–226.

Mazzoil, M., J.S. Reif, M. Youngbluth, M.E. Murdoch, S.E. Bechdel, E. Howells, S.D. McCulloch, L.J. Hansen, and G.D. Bossart. 2008. Home ranges of bottlenose dolphins (*Tursiops truncatus*) in the Indian River Lagoon, Florida: Environmental correlates and implications for management strategies. Ecohealth 5(3):278–288. doi: 10.1007/s10393-008-0194-9

Mazzoil, M.S., S.D. McCulloch, M.J. Youngbluth, D.S. Kilpatrick, M.E. Murdoch, B. Mase-Guthrie, D.K. Odell, and G.D. Bossart. 2008. Radio-tracking and survivorship of two rehabilitated bottlenose dolphins (*Tursiops truncatus*) in the Indian River Lagoon, Florida. Aquatic Mammals 34:54–64. doi: 10.1578/AM.34.1.2008.54

McClellan, D.B., J. Browder, J.L. Tobias, G.J. Konoval, M.D. Heron, O.L. Bass, and J. Osborne. 2000. Opportunistic sightings of bottlenose dolphin, *Tursiops truncatus*, along the southeast Florida coast and Florida Bay, 1992-1997. National Oceanic and Atmospheric Administration, National Marine Fisheries Service, Southeast Fisheries Science Center: Miami, FL. NOAA Technical Memorandum NMFS-SEFSC-435. 18 p.

McFee, W.E., T.R. Speakman, L. Balthis, J.D. Adams, and E.S. Zolman. 2014. Reproductive seasonality of a recently designated bottlenose dolphin stock near Charleston, South Carolina, U.S.A. Marine Mammal Science 30(2):528–543. doi: 10.1111/mms.12055

McHugh, M.B. 1989. Population numbers and feeding behavior of the Atlantic bottlenose dolphin (*Tursiops truncatus*) near Aransas Pass, Texas. University of Texas at Austin. Thesis. 96 p.

Mead, J.G. and C.W. Potter. 1990. Natural history of bottlenose dolphins along the central Atlantic coast of the United States. In: Leatherwood, S. and R.R. Reeves (Eds.), The Bottlenose Dolphin. Academic Press, San Diego, CA. pp. 165–195.

Miller, L.J., A.D. Mackey, T. Hoffland, M. Solangi, and S.A. Kuczaj. 2010. Potential effects of a major hurricane on Atlantic bottlenose dolphin (*Tursiops truncatus*) reproduction in the Mississippi Sound. Marine Mammal Science 26(3):707–715. doi: 10.1111/j.1748-7692.2010.00371.x

Miller, L.J., A.D. Mackey, M. Solangi, and S.A. Kuczaj. 2013. Population abundance and habitat utilization of bottlenose dolphins in the Mississippi Sound. Aquatic Conservation: Marine and Freshwater Ecosystems 23(1):145–151. doi: 10.1002/aqc.2278

Odell, D.K. and E.D. Asper. 1990. Distribution and movements of freeze-branded bottlenose dolphins in the Indian and Banana Rivers, Florida. In: Leatherwood, S. and R. Reeves (Eds.), The Bottlenose Dolphin. Academic Press, San Diego, CA. pp. 515–540.

Page-Karjian, A., C.F. Lo, B. Ritchie, C.A. Harms, D.S. Rotstein, S. Han, S.M. Hassan, A.F. Lehner, J.P. Buchweitz, V.G. Thayer, J.M. Sullivan, E.F. Christiansen, and J.R. Perrault. 2020. Anthropogenic contaminants and histopathological findings in stranded cetaceans in the southeastern United States, 2012–2018. Frontiers in Marine Science 7:630. doi: 10.3389/fmars.2020.00630

Pate, S.M. and W.E. McFee. 2012. Prey species of bottlenose dolphins (*Tursiops truncatus*) from South Carolina Waters. Southeastern Naturalist 11(1):1–22. doi: 10.1656/058.011.0101

Perrin, W.F. and S.B. Reilly. 1984. Reproductive parameters of dolphins and small whales of the family Delphinidae. In: Perrin, W.F., R.L. Brownell, Jr., and D.P. DeMaster (Eds.), Reproduction in Whales, Dolphins, and Porpoises. pp. 97–134.

Perrtree, R.M., C.J. Kovacs, and T.M. Cox. 2014. Standardization and application of metrics to quantify human-interaction behaviors by the bottlenose dolphin (*Tursiops* spp.). Marine Mammal Science 30(4):1320–1334. doi: 10.1111/mms.12114

Pulster, E.L. and K.A. Maruya. 2008. Geographic specificity of Aroclor 1268 in bottlenose dolphins (*Tursiops truncatus*) frequenting the Turtle/Brunswick River Estuary, Georgia (USA). Science of the Total Environment 393(2-3):367–375. doi: 10.1016/j.scitotenv.2007.12.031

Pulster, E.L., K.L. Smalling, E. Zolman, L. Schwacke, and K.A. Maruya. 2009. Persistent organochlorine pollutants and toxaphene congener profiles in bottlenose dolphins (*Tursiops truncatus*) frequenting the Turtle/Brunswick River Estuary, Georgia, USA. Environmental Toxicology and Chemistry 28(7):1390–1399. doi: 10.1897/08-240.1

Reif, J.S., A.M. Schaefer, G.D. Bossart, and P.A. Fair. 2017. Health and Environmental Risk Assessment Project for bottlenose dolphins *Tursiops truncatus* from the southeastern USA. II. Environmental aspects. Diseases of Aquatic Organisms 125(2):155–166. doi: 10.3354/dao03143

Rossman, S., P.H. Ostrom, M. Stolen, N.B. Barros, H. Gandhi, C.A. Stricker, and R.S. Wells. 2015. Individual specialization in the foraging habits of female bottlenose dolphins living in a trophically diverse and habitat rich estuary. Oecologia 178(2):415–425. doi: 10.1007/s00442-015-3241-6

Sanger, D., A. Blair, G. DiDonato, T. Washburn, S. Jones, R. Chapman, D. Bergquist, G. Riekerk, E. Wirth, J. Stewart, D. White, L. Vandiver, S. White, and D. Whitall. 2008. Support for integrated ecosystem assessments of NOAA's National Estuarine Research Reserves System (NERRS), Volume I: The impacts of coastal development on the ecology and human well-being of tidal creek ecosystems of the US Southeast. National Oceanic and Atmospheric Administration, National Ocean Service, National Centers for Coastal Ocean Science: Charleston, SC. NOAA Technical Memorandum NOS-NCCOS-82. 76 p.

Schwacke, L.H., E.O. Voit, L.J. Hansen, R.S. Wells, G.B. Mitchum, A.A. Hohn, and P.A. Fair. 2002. Probabilistic risk assessment of reproductive effects of polychlorinated biphenyls on bottlenose dolphins (*Tursiops truncatus*) from the southeast United States coast. Environmental Toxicology and Chemistry 21(12):2752–2764. doi: 10.1002/etc.5620211232

Schwacke, L.H., E.S. Zolman, B.C. Balmer, S. De Guise, R.C. George, J. Hoguet, A.A. Hohn, J.R. Kucklick, S. Lamb, M. Levin, J.A. Litz, W.E. McFee, N.J. Place, F.I. Townsend, R.S. Wells, and T.K. Rowles. 2012. Anaemia, hypothyroidism and immune suppression associated with polychlorinated biphenyl exposure in bottlenose dolphins (*Tursiops truncatus*). Proceedings of the Royal Society B: Biological Sciences 279(1726):48–57. doi: 10.1098/rspb.2011.0665

Seguel, M., R.C. George, G. Maboni, S. Sanchez, A. Page-Karjian, E. Wirth, W. McFee, and N.L. Gottdenker. 2020. Pathologic findings and causes of death in bottlenose dolphins *Tursiops truncatus* stranded along the Georgia coast, USA (2007-2013). Diseases of Aquatic Organisms 141:25–38. doi: 10.3354/dao03509

Sergeant, D.E., D.K. Caldwell, and M.C. Caldwell. 1973. Age, growth, and maturity of bottlenosed dolphin (*Tursiops-truncatus*) from northeast Florida. Journal of the Fisheries Research Board of Canada 30(7):1009–1011. doi: 10.1139/f73-165

Shane, S.H., R.S. Wells, and B. Würsig. 1986. Ecology, behavior and social organization of the bottlenose dolphin: A review. Marine Mammal Science 2:34–63. doi: 10.1111/j.1748-7692.1986.tb00026.x

Silva, D., B. Tramutolo, E. DeSalvio, T. Speakman, and R. Young. 2019. Abundance and movements of the Northern South Carolina Estuarine System Stock of bottlenose dolphins (*Tursiops truncatus*) (USA). Journal of Marine Animals and Their Ecology 11(1):8–18.

Sloan, P.E. 2006. Residency patterns, seasonality and habitat use among bottlenose dolphins, *Tursiops truncatus*, in the Cape Romain National Wildlife Refuge, S.C. University of North Carolina Wilmington. Thesis. 75 p.

Speakman, T., E. Zolman, J. Adams, R.H. Defran, D. Laska, L. Schwacke, J. Craigie, and P. Fair. 2006. Temporal and spatial aspects of bottlenose dolphin occurrence in coastal and estuarine waters near Charleston, South Carolina. National Oceanic and Atmospheric Administration, National Ocean Service, National Centers for Coastal Ocean Science: Charleston, SC. NOAA Technical Memorandum NOS-NCCOS-37. 50 p.

Stavros, H.-C.W., G.D. Bossart, T.C. Hulsey, and P.A. Fair. 2007. Trace element concentrations in skin of free-ranging bottlenose dolphins (*Tursiops truncatus*) from the southeast Atlantic coast. Science of the Total Environment 388(1-3):300–315. doi: 10.1016/j.scitotenv.2007.07.030

Stavros, H.-C.W., G.D. Bossart, T.C. Hulsey, and P.A. Fair. 2008. Trace element concentrations in blood of free-ranging bottlenose dolphins (*Tursiops truncatus*): Influence of age, sex and location. Marine Pollution Bulletin 56(2):371–379. doi: 10.1016/j.marpolbul.2007.10.030

Stavros, H.-C.W., M. Stolen, W.N. Durden, W. McFee, G.D. Bossart, and P.A. Fair. 2011. Correlation and toxicological inference of trace elements in tissues from stranded and free-ranging bottlenose dolphins (*Tursiops truncatus*). Chemosphere 82(11):1649–1661. doi: 10.1016/j.chemosphere.2010.11.019

Taylor, B.L., S.J. Chivers, J. Larese, and W.F. Perrin. 2007. Generation length and percent mature estimates for IUCN assessments of cetaceans. National Oceanic and Atmospheric

Administration, National Marine Fisheries Service, Southwest Fisheries Science Center: La Jolla, CA. Administrative Report LJ-07-01. 24 p.

Thayer, V.G. 2008. Life history parameters and social associations of female bottlenose dolphins (*Tursiops truncatus*) off North Carolina, USA. Duke University. Dissertation. 164 p.

Thayer, V.G., A.J. Read, A.S. Friedlaender, D.R. Colby, A.A. Hohn, W.A. McLellan, D.A. Pabst, J.L. Dearolf, N.I. Bowles, J.R. Russell, and K.A. Rittmaster. 2003. Reproductive seasonality of western Atlantic bottlenose dolphins off North Carolina, USA. Marine Mammal Science 19(4):617–629. doi: 10.1111/j.1748-7692.2003.tb01120.x

Titcomb, E.M., J.S. Reif, P.A. Fair, H.-C.W. Stavros, M. Mazzoil, G.D. Bossart, and A.M. Schaefer. 2017. Blood mercury concentrations in common bottlenose dolphins from the Indian River Lagoon, Florida: Patterns of social distribution. Marine Mammal Science 33(3):771–784. doi: 10.1111/mms.12390

Torres, L.G. 2007. Top predator distribution and foraging ecology in Florida Bay, Florida. Duke University. Dissertation. 225 p.

Torres, L.G. and A.J. Read. 2009. Where to catch a fish? The influence of foraging tactics on the ecology of bottlenose dolphins (*Tursiops truncatus*) in Florida Bay, Florida. Marine Mammal Science 25(4):797–815. doi: 10.1111/j.1748-7692.2009.00297.x

Urian, K.W., D.M. Waples, R.B. Tyson, L.E. Hodge, and A.J. Read. 2013. Abundance of bottlenose dolphins (*Tursiops truncatus*) in estuarine and near-shore waters of North Carolina, USA. Journal of the North Carolina Academy of Science 129(4):165–171. doi: 10.7572/2167-5880-129.4.165

Vollmer, N.L. and P.E. Rosel. 2013. A review of common bottlenose dolphins (*Tursiops truncatus truncatus*) in the Northern Gulf of Mexico: Population biology, potential threats, and management. Southeastern Naturalist 12(m6):1–43. doi: 10.1656/058.012.m601

Waring, G., E. Josephson, K. Maze-Foley, and P. Rosel. 2014. U.S. Atlantic and Gulf of Mexico Marine Mammal Stock Assessments - 2013. National Oceanic and Atmospheric Administration, National Marine Fisheries Service, Northeast Fisheries Science Center: Woods Hole, MA. NOAA Technical Memorandum NMFS-NE-228. 464 p. doi: 10.7289/V51G0J70

Waring, G.T., E. Josephson, K. Maze-Foley, and P.E. Rosel. 2016. U.S. Atlantic and Gulf of Mexico marine mammal stock assessments - 2015. National Oceanic and Atmospheric Administration, National Marine Fisheries Service, Northeast Fisheries Science Center: Woods Hole, MA. NOAA Technical Memorandum NMFS-NE-238. 501 p. doi: 10.7289/V57S7KTN

Wells, R.S. 2000. Reproduction in wild bottlenose dolphins: Overview of patterns observed during a long-term study. In: Duffield, D. and T. Robeck (Eds.), Bottlenose Dolphin Reproduction Workshop. AZA Marine Mammal Taxon Advisory Group, Silver Spring, MD. pp. 57–74.

Wells, R.S. 2003. Dolphin social complexity: Lessons from long-term study and life history. In: de Waal, F.B.M. and P.L. Tyack (Eds.), Animal Social Complexity: Intelligence, Culture, and Individualized Societies Harvard University Press, Cambridge, MA. pp. 32–56.

Wells, R.S. 2014. Social structure and life history of bottlenose dolphins near Sarasota Bay, Florida: Insights from four decades and five generations. Primates and cetaceans. pp. 149–172. doi: 10.1007/978-4-431-54523-1_8

Wells, R.S., A. Natoli, and G. Braulik. 2019. *Tursiops truncatus* (errata version published in 2019). *The IUCN Red List of Threatened Species* 2019: e.T22563A156932432. https://dx.doi.org/10.2305/IUCN.UK.2019-1.RLTS.T22563A156932432.en

Wells, R.S. and M.D. Scott. 1999. Bottlenose dolphin *Tursiops truncatus* (Montagu, 1821). In: Ridgway, S.H. and R. Harrison (Eds.), Handbook of marine mammals. Volume 6: The second book of dolphins and the porpoises. Academic Press, San Diego, CA. pp. 137–182.

Wells, R.S. and M.D. Scott. 2018. Bottlenose dolphin, *Tursiops truncatus*, common bottlenose dolphin. In: Würsig, B., J.G.M. Thewissen, and K.M. Kovacs (Eds.), Encyclopedia of Marine Mammals

(Third Edition). Academic Press, London, UK. pp. 118–125. doi: 10.1016/B978-0-12-804327-1.00072-8
Wells, R.S., M.D. Scott, and A.B. Irvine. 1987. The social structure of free-ranging bottlenose dolphins. In: Genoways, H.H. (Ed.), Current Mammalogy. Plenum Press, New York, NY. pp. 247–305.
Wilson, R.M., R.B. Tyson, J.A. Nelson, B.C. Balmer, J.P. Chanton, and D.P. Nowacek. 2017. Niche differentiation and prey selectivity among common bottlenose dolphins (*Tursiops truncatus*) sighted in St. George Sound, Gulf of Mexico. Frontiers in Marine Science 4:235. doi: 10.3389/fmars.2017.00235
Wu, C. 2013. Human and boat interactions with common bottlenose dolphins (*Tursiops truncatus*) in the waterways around Savannah, Georgia. Savannah State University. Thesis. 101 p.
Yordy, J.E., M.A. Mollenhauer, R.M. Wilson, R.S. Wells, A. Hohn, J. Sweeney, L.H. Schwacke, T.K. Rowles, J.R. Kucklick, and M.M. Peden-Adams. 2010. Complex contaminant exposure in cetaceans: a comparative E-Screen analysis of bottlenose dolphin blubber and mixtures of four persistent organic pollutants. Environmental Toxicology and Chemistry 29(10):2143–2153. doi: 10.1002/etc.279

Common bottlenose dolphin − *Tursiops truncatus*
Southern North Carolina Estuarine System Stock

Overall Vulnerability Rank = Very High

Biological Sensitivity = Very High

Climate Exposure = Very High

Data Quality = 100% of scores≥ 2

	Tursiops truncatus	Expert Scores	Data Quality	Expert Scores Plots (Portion by Category)
Sensitivity attributes	Prey/Diet Specificity	2.1	2.3	
	Habitat Specificity	3.6	2.0	
	Site Fidelity	4.0	2.3	
	Lifetime Reproductive Potential	1.3	2.3	
	Generation Length	2.6	2.3	
	Reproductive Plasticity	1.5	2.3	
	Migration	3.9	2.7	
	Home Range	3.4	2.7	
	Stock Abundance	3.0	2.7	
	Stock Abundance Trend	2.2	2.3	
	Cumulative Stressors	2.9	3.0	
	Sensitivity Score	Very High		
Exposure variables	Sea Surface Temperature (Change in mean)	4.0	2.7	
	Sea Surface Temperature (Change in variability)	1.0	2.7	
	Air Temperature (Change in mean)	4.0	2.7	
	Air Temperature (Change in variability)	1.0	2.7	
	Precipitation (Change in mean)	1.0	2.7	
	Precipitation (Change in variability)	2.0	2.7	
	Sea Surface Salinity (Change in mean)	3.9	2.7	
	Sea Surface Salinity (Change in variability)	1.5	2.7	
	Ocean pH (Change in mean)	4.0	2.7	
	Ocean pH (Change in variability)	1.0	2.7	
	Sea ice coverage (Change in mean)	1.0	2.7	
	Sea ice coverage (Change in variability)	1.0	2.7	
	Dissolved oxygen (Change in mean)	4.0	2.7	
	Dissolved oxygen (Change in variability)	1.0	2.7	
	Circulation	3.9	2.3	
	Sea level rise	4.0	2.7	
	Exposure Score	Very High		
Overall Vulnerability Rank		Very High		

Low
Moderate
High
Very High

Common bottlenose dolphin (Southern North Carolina Estuarine System Stock)

Tursiops truncatus

CVA Results Summary

Overall Climate Vulnerability Rank: Very High (100% certainty from bootstrap analysis).

Climate Exposure: Very High. Seven exposure factors scored greater than 3.5: Air Temperature (Standard anomaly) (4.00), Dissolved oxygen (Standard anomaly) (4.00), Ocean pH (Standard anomaly) (4.00), Sea level rise (4.00), Sea Surface Temperature (Standard anomaly) (4.00), Sea Surface Salinity (Standard anomaly) (3.93), and Circulation (3.87).

Biological Sensitivity: Very High. Three sensitivity attributes scored greater than 3.5: Site Fidelity (4.00), Migration (3.93), and Habitat Specificity (3.60).

Distributional Response: High

Abundance Response: High

Phenology Response: High

Data Quality: 100 % of the data quality scores were 2 or greater. 100 % of sensitivity attribute data quality scores were 2 or higher.

Background Information

Stock Description

The Southern North Carolina Estuarine System (SNCES) Stock includes common bottlenose dolphins found in estuarine and nearshore coastal waters (≤ 3 km from shore) between the Little River Inlet estuary and the New River, North Carolina (Hayes et al. 2021).

Prey/Diet Specificity

Generally, common bottlenose dolphins consume a large variety of fish and/or squid (Barros and Odell 1990; Mead and Potter 1990; Wells and Scott 2018). Common bottlenose dolphins seem to show a consistent preference for sound-producing fishes (e.g., sciaenids, toadfish; Berens McCabe et al. 2010; Bowen-Stevens et al. 2021). Estuarine stocks along the southeast U.S. Atlantic coast have been reported to consume a variety of prey, with preference for sciaenids (Gannon and Waples 2004; Pate and McFee 2012). Pate and McFee (2012) identified 42 prey species from 24 families in a study of stranded dolphins along the South Carolina coast. In Sarasota Bay, Florida, Rossman et al. (2015) found some individual common bottlenose dolphins to show diet specialization despite the broader study group showing a generalist diet. Specialization can be influenced by locally abundant prey species (Shane et al. 1986; Vollmer and Rosel 2013) or can result from resource partitioning amongst sympatric populations (e.g., Torres and Read 2009; Wilson et al. 2017).

Habitat Specificity

Common bottlenose dolphins are found in a variety of marine and estuarine habitats (Wells and Scott 2018). The area inhabited by the SNCES Stock is characterized by tidal marshes, estuarine waters, and shallow open water (Hayes et al. 2021).

Site Fidelity

No specific site fidelity information was found for this stock. Other estuarine stocks in the western North Atlantic have shown site fidelity (Hayes et al. 2021), such as in South Carolina (Sloan 2006; Speakman et al. 2006; Hayes et al. 2021), central and southern Georgia (Balmer et al. 2011; Kucklick et al. 2011; Balmer et al. 2013), northern Florida (Caldwell 2001), central Florida (Odell and Asper 1990; Mazzoil et al. 2005, 2008a, 2008b), and southern Florida (Litz 2007; Torres 2007; McClellan et al. 2000).

Lifetime Reproductive Potential

Taylor et al. (2007) reported the interbirth interval of common bottlenose dolphins as 3.80 years, though other studies have shown it to be closer to three years (Leatherwood 1977; Wells and Scott 1999; Connor et al. 2000; Wells 2003). Thayer (2008) estimated mean interbirth interval of 2.9 years in North Carolina.

Generally, females reach sexual maturity before males, with females reaching sexual maturity at 5–13 years (Sergeant et al. 1973; Perrin and Reilly 1984; Wells et al. 1987; Mead and Potter 1990; Mann et al. 2000; Wells 2000; Wells and Scott 2018).

Females reproduce through most of their adult lives (Leatherwood 1977; Hohn et al. 1989; Wells and Scott 1999; Wells 2000; Wells 2003), but may produce fewer calves after they reach 30 years of age (Wells 2000). Sarasota Bay common bottlenose dolphins can give birth when they are as young as 6 years of age, and can continue until they are 48 years old (Wells and Scott 2018). Reproductive lifespan would therefore be up to 42 years based on long-term observation studies (Wells 2014; Wells and Scott 2018).

Longitudinal studies from Sarasota Bay, Florida have found that female bottlenose dolphins can live up to 67 years (Wells and Scott 2018). Studies based on growth layer groups in teeth suggest female bottlenose dolphins can live to 41 years along the Texas coast (Fernandez and Hohn 1998) and to 30 years along the Mississippi coast (Mattson et al. 2006).

Generation Length

Taylor et al. (2007) reported common bottlenose dolphin generation length of 20.6–21.1 years.

Reproductive Plasticity

Common bottlenose dolphin calving is generally diffusely seasonal, with one or more peaks, although births have been reported from all seasons (Vollmer and Rosel 2013; Wells and Scott 2018). In North Carolina, Thayer et al. (2003) observed a strong birth peak in spring based on stranding data and a diffuse birth peak from late spring to early fall based on sightings. Calf sightings in the Charleston Estuarine System Stock peak in spring and early summer and again in early autumn (McFee et al. 2014).

Migration

The estuarine stocks in the western North Atlantic are thought to primarily comprise stable resident communities. However, transient or seasonal movements of some individuals into and out of stock areas are recorded in some areas (LaBrecque et al. 2015; Hayes et al. 2021).

Members of the SNCES Stock do not undertake large-scale migratory movements but do display seasonal movements, expanding their range slightly north to include parts of Core Sound and southern Pamlico Sound during warm months (Hayes et al. 2021).

Home Range

The home ranges of individuals within the western North Atlantic estuarine stocks are considered to generally include the areas within the stock boundaries and brief excursions beyond the stock boundaries (LaBrecque et al. 2015; Hayes et al. 2021). Members of the SNCES Stock show relatively constrained ranges based on photo-ID, tag telemetry and genetic data (Hansen and Wells 1996; Hayes et al. 2018). Studies have shown dolphin populations with constrained ranges in Georgia (Brunswick and Sapelo; Balmer et al. 2013); South Carolina (Silva et al. 2019), and North Carolina (Urian et al. 2014). Balmer et al. (2018), using satellite telemetry data, identified that dolphins considered to be members of the South Georgia Estuarine System Stock had different movement patterns with some individuals remaining entirely within the estuarine waters, while other animals use the larger estuarine waters, sounds, and to some degree coastal waters.

Stock Abundance

Urian et al. (2014) estimated the abundance for the SNCES Stock at 188 individuals (CV=0.19, 95% confidence interval=118-257) based on 2006 photo-ID mark-recapture surveys. However, the current population size of the SNCES Stock is considered unknown because the surveys are more than eight years old.

Stock Abundance Trend

A trend analysis has not been conducted for this stock (Hayes et al. 2021). Previously, Read et al. (2003) estimated the abundance of the SNCES Stock at 141 individuals (CV=0.15, 95% confidence interval=112-200) based on a 2000 photo-ID mark-recapture survey of inshore waters. However, this survey did not include coastal waters and should be considered negatively biased.

Cumulative Stressors

Common bottlenose dolphins face a wide range of individual and cumulative natural and anthropogenic threats (see reviews by Vollmer and Rosel 2013; Wells and Scott 2018; and Wells et al. 2019). These include such threats as shark predation, harmful algal blooms, recreational and commercial fishing gear (Byrd and Hohn 2010, 2017; Byrd et al. 2014), vessel traffic, pollution, habitat alteration, and provisioning.

Common bottlenose dolphins in the SNCES Stock interact with several commercial fisheries, including gillnet, long haul seine, roe mullet stop net, blue crab trap/pot, and hook and line fisheries. The minimum total mean annual human-caused mortality and serious injury for fisheries and other human

causes was 0.4 individuals per year during 2014–2018 (Hayes et al. 2021). During 2014–2018, 53 common bottlenose dolphins stranded within the SNCES Stock. No evidence of human interaction, such as fishing gear and vessel interactions, could be detected for 30 of these animals and it could not be determined if there was evidence of human interaction for 18 animals (Maze-Foley et al. 2019).

An Unusual Mortality Event (UME) was declared in the summer of 2013 for the mid-Atlantic coast from New York to Brevard County, Florida, with more than 1600 common bottlenose dolphins stranding as a result of morbillivirus (Hayes et al. 2021; NMFS 2022). Strandings were more prevalent along oceanic shores than in estuaries, suggesting that coastal stocks were more impacted by the UME than estuarine stocks (Hayes et al. 2021).

The nearshore and estuarine habitats along the Atlantic seaboard are adjacent to areas of high human population and some are highly industrialized. Studies of bottlenose dolphin tissues from estuaries in the southeast U.S. have shown persistent organic pollutant (POP; Schwacke et al. 2002, 2012; Hansen et al. 2004; Litz et al. 2007; Pulster et al. 2009; Fair et al. 2010; Yordy et al. 2010a; Balmer et al. 2011; Kucklick et al. 2011; Reif et al. 2017) and heavy metal (Durden et al. 2007; Stavros et al. 2007, 2008, 2011; Titcomb et al. 2017; Reif et al. 2017; Page-Karjian et al. 2020) concentrations that may result in adverse effects on health or reproductive rates, particularly in urbanized, developed, and agricultural areas. In Biscayne Bay, Florida, elevated POP concentrations were observed in individuals associated with more polluted areas compared to individuals associated with the less polluted areas of Biscayne Bay, suggesting that exposure to pollutants may be highly variable based on geography (Litz et al. 2007; Kucklick et al. 2011). The SNCES Stock inhabits an area that receives runoff from agricultural, industrial, and urban sources. Analyses of common bottlenose dolphins along the North Carolina coast have found elevated levels of POPs, including polychlorinated biphenyls (PCBs) and polybrominated diphenyl ethers (PBDEs; Schwacke et al. 2002; Hansen et al. 2004; Yordy et al. 2010a, 2010b). Individuals from this stock have been found to have detectable concentrations of lead, mercury, and selenium, though at lower levels than the Indian River Lagoon Estuarine System in Florida (Page-Karjian et al. 2020).

Further Reading

Hayes, S.A., E. Josephson, K. Maze-Foley, P.E. Rosel, and J. Turek. 2021. US Atlantic and Gulf of Mexico Marine Mammal Stock Assessments - 2020. National Oceanic and Atmospheric Administration, National Marine Fisheries Service, Northeast Fisheries Science Center: Woods Hole, MA. NOAA Technical Memorandum NMFS-NE-271. 394 p.

Wells, R.S. and M.D. Scott. 2018. Bottlenose Dolphin, *Tursiops truncatus*, Common Bottlenose Dolphin. In: Würsig, B., J.G.M. Thewissen, and K.M. Kovacs (Eds.), Encyclopedia of Marine Mammals (Third Edition). Academic Press, London, UK. pp. 118–125. doi: 10.1016/B978-0-12-804327-1.00072-8

Wells, R.S., A. Natoli, and G. Braulik. 2019. *Tursiops truncatus* (errata version published in 2019). The IUCN Red List of Threatened Species 2019: e.T22563A156932432. https://dx.doi.org/10.2305/IUCN.UK.2019-1.RLTS.T22563A156932432.en

References

Balmer, B., E. Zolman, T. Rowles, C. Smith, F. Townsend, D. Fauquier, C. George, T. Goldstein, L. Hansen, B. Quigley, W. McFee, J. Morey, P. Rosel, J. Saliki, T. Speakman, and L. Schwacke. 2018. Ranging patterns, spatial overlap, and association with dolphin morbillivirus exposure in common

bottlenose dolphins (*Tursiops truncatus*) along the Georgia, USA coast. Ecology and Evolution 8(24):12890–12904. doi: 10.1002/ece3.4727

Balmer, B.C., L.H. Schwacke, R.S. Wells, J.D. Adams, R. Clay George, S.M. Lane, W.A. McLellan, P.E. Rosel, K. Sparks, T. Speakman, E.S. Zolman, and D. Ann Pabst. 2013. Comparison of abundance and habitat usage for common bottlenose dolphins between sites exposed to differential anthropogenic stressors within the estuaries of southern Georgia, U.S.A. Marine Mammal Science 29(2):E114–E135. doi: 10.1111/j.1748-7692.2012.00598.x

Balmer, B.C., L.H. Schwacke, R.S. Wells, R.C. George, J. Hoguet, J.R. Kucklick, S.M. Lane, A. Martinez, W.A. McLellan, P.E. Rosel, T.K. Rowles, K. Sparks, T. Speakman, E.S. Zolman, and D.A. Pabst. 2011. Relationship between persistent organic pollutants (POPs) and ranging patterns in common bottlenose dolphins (*Tursiops truncatus*) from coastal Georgia, USA. Science of the Total Environment 409(11):2094–2101. doi: 10.1016/j.scitotenv.2011.01.052

Barros, N.B. and D.K. Odell. 1990. Food habits of bottlenose dolphins in the southeastern United States In: Leatherwood, S. and R.R. Reeves (Eds.), The Bottlenose Dolphin. Academic Press, San Diego, CA. pp. 309–328. doi: 10.1016/B978-0-12-440280-5.50020-2

Berens McCabe, E.J., D.P. Gannon, N.B. Barros, and R.S. Wells. 2010. Prey selection by resident common bottlenose dolphins (*Tursiops truncatus*) in Sarasota Bay, Florida. Marine Biology 157(5):931–942. doi: 10.1007/s00227-009-1371-2

Bowen-Stevens, S.R., D.P. Gannon, R.A. Hazelkorn, G. Lovewell, K.M. Volker, S. Smith, M.C. Tumlin, and J. Litz. 2021. Diet of common bottlenose dolphins, *Tursiops truncatus*, that stranded in and near Barataria Bay, Louisiana, 2010–2012. Southeastern Naturalist 20(1). doi: 10.1656/058.020.0113

Byrd, B.L. and A.A. Hohn. 2017. Differential risk of bottlenose dolphin (*Tursiops truncatus*) bycatch in North Carolina, USA. Aquatic Mammals 43(5):558–569. doi: 10.1578/Am.43.5.2017.558

Byrd, B.L., A.A. Hohn, G.N. Lovewell, K.M. Altman, S.G. Barco, A. Friedlaender, C.A. Harms, W.A. McLellan, K.T. Moore, P.E. Rosel, and V.G. Thayer. 2014. Strandings as indicators of marine mammal biodiversity and human interactions off the coast of North Carolina. Fishery Bulletin 112(1):1–23. doi: 10.7755/fb.112.1.1

Caldwell, M. 2001. Social and genetic structure of bottlenose dolphin (*Tursiops truncatus*) in Jacksonville, Florida. University of Miami. 143 p.

Connor, R.C., R.S. Wells, J. Mann, and A.J. Read. 2000. The bottlenose dolphin: Social relationships in a fission-fusion society. In: Mann, J., R. Connor, P. Tyack, and H. Whitehead (Eds.), Cetacean Societies: Field Studies of Dolphins and Whales. University of Chicago Press, Chicago, IL. pp. 91–126.

Durden, W.N., M.K. Stolen, D.H. Adams, and E.D. Stolen. 2007. Mercury and selenium concentrations in stranded bottlenose dolphins from the Indian River Lagoon system, Florida. Bulletin of Marine Science 81(1):37–54.

Fair, P.A., J. Adams, G. Mitchum, T.C. Hulsey, J.S. Reif, M. Houde, D. Muir, E. Wirth, D. Wetzel, E. Zolman, W. McFee, and G.D. Bossart. 2010. Contaminant blubber burdens in Atlantic bottlenose dolphins (*Tursiops truncatus*) from two southeastern US estuarine areas: Concentrations and patterns of PCBs, pesticides, PBDEs, PFCs, and PAHs. Science of the Total Environment 408(7):1577–1597. doi: 10.1016/j.scitotenv.2009.12.021

Fernandez, S. and A.A. Hohn. 1998. Age, growth, and calving season of bottlenose dolphins, *Tursiops truncatus*, off coastal Texas. Fishery Bulletin 96(2):357–365.

Gannon, D.P. and D.M. Waples. 2004. Diets of coastal bottlenose dolphins from the U. S. Mid-Atlantic coast differ by habitat. Marine Mammal Science 20(3):527–545. doi: 10.1111/j.1748-7692.2004.tb01177.x

Hansen, L.J., L.H. Schwacke, G.B. Mitchum, A.A. Hohn, R.S. Wells, E.S. Zolman, and P.A. Fair. 2004. Geographic variation in polychorinated biphenyl and organochlorine pesticide concentrations in

the blubber of bottlenose dolphins from the US Atlantic coast. Science of the Total Environment 319(1-3):147–172. doi: 10.1016/S0048-9697(03)00371-1
Hansen, L.J. and R.S. Wells. 1996. Bottlenose dolphin health assessment: Field report on sampling near Beaufort, North Carolina, during July, 1995. National Oceanic and Atmospheric Administration, National Marine Fisheries Service, Southeast Fisheries Science Center, Charleston Laboratory: Charleston, SC. NOAA Technical Memorandum NMFS-SEFSC-382. 24 p.
Hayes, S.A., E. Josephson, K. Maze-Foley, and P.E. Rosel. 2018. US Atlantic and Gulf of Mexico marine mammal stock assessments - 2017: (Second Edition). National Oceanic and Atmospheric Administration, National Marine Fisheries Service, Northeast Fisheries Science Center: Woods Hole, MA. NOAA Technical Memorandum NMFS-NE-245. 371 p. doi: 10.25923/e764-9g81
Hayes, S.A., E. Josephson, K. Maze-Foley, P.E. Rosel, and J. Turek. 2021. US Atlantic and Gulf of Mexico marine mammal stock assessments - 2020. National Oceanic and Atmospheric Administration, National Marine Fisheries Service, Northeast Fisheries Science Center: Woods Hole, MA. NOAA Technical Memorandum NMFS-NE-271. 394 p.
Hohn, A.A., M.D. Scott, R.S. Wells, J.C. Sweeney, and A.B. Irvine. 1989. Growth layers in teeth from known-age, free-ranging bottlenose dolphins. Marine Mammal Science 5(4):315–342. doi: 10.1111/j.1748-7692.1989.tb00346.x
Kucklick, J., L. Schwacke, R. Wells, A. Hohn, A. Guichard, J. Yordy, L. Hansen, E. Zolman, R. Wilson, J. Litz, D. Nowacek, T. Rowles, R. Pugh, B. Balmer, C. Sinclair, and P. Rosel. 2011. Bottlenose dolphins as indicators of persistent organic pollutants in the western North Atlantic Ocean and Northern Gulf of Mexico. Environmental Science & Technology 45(10):4270–4277. doi: 10.1021/es1042244
LaBrecque, E., C. Curtice, J. Harrison, S.M. Van Parijs, and P.N. Halpin. 2015. 2. Biologically Important Areas for Cetaceans Within U.S. Waters – East Coast Region. Aquatic Mammals 41(1):17–29. doi: 10.1578/am.41.1.2015.17
Leatherwood, S. 1977. Some preliminary impressions on the numbers and social behavior of free-swimming bottlenosed dolphin calves (*Tursiops truncatus*) in the northern Gulf of Mexico. In: Ridgway, S.H. and K. Bernirschke (Eds.), Breeding Dolphins; Present Status, Suggestions for the Future. Final report to the US Marine Mammal Commission, Contract MM6AC009, MMC-76/07, Washington, DC. pp. 143–167.
Litz, J.A. 2007. Social structure, genetic structure, and persistent organohalogen pollutants in bottlenose dolphins (*Tursiops truncatus*) in Biscayne Bay, Florida. University of Miami. 140 p.
Litz, J.A., L.P. Garrison, L.A. Fieber, A. Martinez, J.P. Contillo, and J.R. Kucklick. 2007. Fine-scale spatial variation of persistent organic pollutants in bottlenose dolphins (*Tursiops truncatus*) in Biscayne Bay, Florida. Environmental Science & Technology 41(21):7222–7228. doi: 10.1021/es070440r
Mann, J., R.C. Connor, L.M. Barre, and M.R. Heithaus. 2000. Female reproductive success in bottlenose dolphins (*Tursiops* sp.): life history, habitat, provisioning, and group-size effects. Behavioral Ecology 11(2):210–219. doi: 10.1093/beheco/11.2.210
Mattson, M.C., K.D. Mullin, G.W. Ingram, and W. Hoggard. 2006. Age structure and growth of the bottlenose dolphin (*Tursiops truncatus*) from strandings in the Mississippi sound region of the north-central Gulf of Mexico from 1986 to 2003. Marine Mammal Science 22(3):654–666. doi: 10.1111/j.1748-7692.2006.00057.x
Maze-Foley, K., B.L. Byrd, S.C. Horstman, and J.R. Powell. 2019. Analysis of stranding data to support estimates of mortality and serious injury in common bottlenose dolphin (*Tursiops truncatus truncatus*) stock assessments for the Atlantic Ocean and Gulf of Mexico. National Oceanic and Atmospheric Administration, National Marine Fisheries Service, Southeast Fisheries Science Center: Miami, FL. NOAA Technical Memorandum NMFS-SEFSC-742. 31 p. doi: 10.25923/jt1x-jv75

Mazzoil, M., S.D. McCulloch, and R.H. Defran. 2005. Observations on the site fidelity of bottlenose dolphins (*Tursiops truncatus*) in the Indian River Lagoon, Florida. Florida Scientist 68(4):217–226.

Mazzoil, M., J.S. Reif, M. Youngbluth, M.E. Murdoch, S.E. Bechdel, E. Howells, S.D. McCulloch, L.J. Hansen, and G.D. Bossart. 2008a. Home ranges of bottlenose dolphins (*Tursiops truncatus*) in the Indian River Lagoon, Florida: Environmental correlates and implications for management strategies. Ecohealth 5(3):278–288. doi: 10.1007/s10393-008-0194-9

Mazzoil, M.S., S.D. McCulloch, M.J. Youngbluth, D.S. Kilpatrick, M.E. Murdoch, B. Mase-Guthrie, D.K. Odell, and G.D. Bossart. 2008b. Radio-tracking and survivorship of two rehabilitated bottlenose dolphins (*Tursiops truncatus*) in the Indian River Lagoon, Florida. Aquatic Mammals 34:54–64. doi: 10.1578/AM.34.1.2008.54

McClellan, D.B., J. Browder, J.L. Tobias, G.J. Konoval, M.D. Heron, O.L. Bass, and J. Osborne. 2000. Opportunistic sightings of bottlenose dolphin, *Tursiops truncatus*, along the southeast Florida coast and Florida Bay, 1992-1997. National Oceanic and Atmospheric Administration, National Marine Fisheries Service, Southeast Fisheries Science Center: Miami, FL. NOAA Technical Memorandum NMFS-SEFSC-435. 18 p.

McFee, W.E., T.R. Speakman, L. Balthis, J.D. Adams, and E.S. Zolman. 2014. Reproductive seasonality of a recently designated bottlenose dolphin stock near Charleston, South Carolina, U.S.A. Marine Mammal Science 30(2):528–543. doi: 10.1111/mms.12055

Mead, J.G. and C.W. Potter. 1990. Natural history of bottlenose dolphins along the central Atlantic coast of the United States. In: Leatherwood, S. and R.R. Reeves (Eds.), The Bottlenose Dolphin. Academic Press, San Diego, CA. pp. 165–195.

National Marine Fisheries Service (NMFS). 2022. Active and closed Unusual Mortality Events. https://www.fisheries.noaa.gov/national/marine-life-distress/active-and-closed-unusual-mortality-events

Odell, D.K. and E.D. Asper. 1990. Distribution and movements of freeze-branded bottlenose dolphins in the Indian and Banana Rivers, Florida. In: Leatherwood, S. and R. Reeves (Eds.), The Bottlenose Dolphin. Academic Press, San Diego, CA. pp. 515–540.

Page-Karjian, A., C.F. Lo, B. Ritchie, C.A. Harms, D.S. Rotstein, S. Han, S.M. Hassan, A.F. Lehner, J.P. Buchweitz, V.G. Thayer, J.M. Sullivan, E.F. Christiansen, and J.R. Perrault. 2020. Anthropogenic contaminants and histopathological findings in stranded cetaceans in the southeastern United States, 2012–2018. Frontiers in Marine Science 7:630. doi: 10.3389/fmars.2020.00630

Pate, S.M. and W.E. McFee. 2012. Prey species of bottlenose dolphins (*Tursiops truncatus*) from South Carolina Waters. Southeastern Naturalist 11(1):1–22. doi: 10.1656/058.011.0101

Perrin, W.F. and S.B. Reilly. 1984. Reproductive parameters of dolphins and small whales of the family Delphinidae. In: Perrin, W.F., R.L. Brownell, Jr., and D.P. DeMaster (Eds.), Reproduction in Whales, Dolphins, and Porpoises. pp. 97–134.

Pulster, E.L., K.L. Smalling, E. Zolman, L. Schwacke, and K.A. Maruya. 2009. Persistent organochlorine pollutants and toxaphene congener profiles in bottlenose dolphins (*Tursiops truncatus*) frequenting the Turtle/Brunswick River Estuary, Georgia, USA. Environmental Toxicology and Chemistry 28(7):1390–1399. doi: 10.1897/08-240.1

Read, A.J., K.W. Urian, B. Wilson, and D.M. Waples. 2003. Abundance of bottlenose dolphins in the bays, sounds, and estuaries of North Carolina. Marine Mammal Science 19(1):59–73. doi: 10.1111/j.1748-7692.2003.tb01092.x

Reif, J.S., A.M. Schaefer, G.D. Bossart, and P.A. Fair. 2017. Health and Environmental Risk Assessment Project for bottlenose dolphins *Tursiops truncatus* from the southeastern USA. II. Environmental aspects. Diseases of Aquatic Organisms 125(2):155–166. doi: 10.3354/dao03143

Rossman, S., P.H. Ostrom, M. Stolen, N.B. Barros, H. Gandhi, C.A. Stricker, and R.S. Wells. 2015. Individual specialization in the foraging habits of female bottlenose dolphins living in a

trophically diverse and habitat rich estuary. Oecologia 178(2):415–425. doi: 10.1007/s00442-015-3241-6

Schwacke, L.H., E.O. Voit, L.J. Hansen, R.S. Wells, G.B. Mitchum, A.A. Hohn, and P.A. Fair. 2002. Probabilistic risk assessment of reproductive effects of polychlorinated biphenyls on bottlenose dolphins (*Tursiops truncatus*) from the southeast United States coast. Environmental Toxicology and Chemistry 21(12):2752–2764. doi: 10.1002/etc.5620211232

Schwacke, L.H., E.S. Zolman, B.C. Balmer, S. De Guise, R.C. George, J. Hoguet, A.A. Hohn, J.R. Kucklick, S. Lamb, M. Levin, J.A. Litz, W.E. McFee, N.J. Place, F.I. Townsend, R.S. Wells, and T.K. Rowles. 2012. Anaemia, hypothyroidism and immune suppression associated with polychlorinated biphenyl exposure in bottlenose dolphins (*Tursiops truncatus*). Proceedings of the Royal Society B: Biological Sciences 279(1726):48–57. doi: 10.1098/rspb.2011.0665

Sergeant, D.E., D.K. Caldwell, and M.C. Caldwell. 1973. Age, growth, and maturity of bottlenosed dolphin (*Tursiops-truncatus*) from northeast Florida. Journal of the Fisheries Research Board of Canada 30(7):1009–1011. doi: 10.1139/f73-165

Shane, S.H., R.S. Wells, and B. Würsig. 1986. Ecology, behavior and social organization of the bottlenose dolphin: A review. Marine Mammal Science 2:34–63. doi: 10.1111/j.1748-7692.1986.tb00026.x

Silva, D., B. Tramutolo, E. DeSalvio, T. Speakman, and R. Young. 2019. Abundance and movements of the Northern South Carolina Estuarine System Stock of bottlenose dolphins (*Tursiops truncatus*) (USA). Journal of Marine Animals and Their Ecology 11(1):8–18.

Sloan, P.E. 2006. Residency patterns, seasonality and habitat use among bottlenose dolphins, *Tursiops truncatus*, in the Cape Romain National Wildlife Refuge, S.C. University of North Carolina Wilmington. Thesis. 75 p.

Speakman, T., E. Zolman, J. Adams, R.H. Defran, D. Laska, L. Schwacke, J. Craigie, and P. Fair. 2006. Temporal and spatial aspects of bottlenose dolphin occurrence in coastal and estuarine waters near Charleston, South Carolina. National Oceanic and Atmospheric Administration, National Ocean Service, National Centers for Coastal Ocean Science: Charleston, SC. NOAA Technical Memorandum NOS-NCCOS-37. 50 p.

Stavros, H.-C.W., G.D. Bossart, T.C. Hulsey, and P.A. Fair. 2007. Trace element concentrations in skin of free-ranging bottlenose dolphins (*Tursiops truncatus*) from the southeast Atlantic coast. Science of the Total Environment 388(1-3):300–315. doi: 10.1016/j.scitotenv.2007.07.030

Stavros, H.-C.W., G.D. Bossart, T.C. Hulsey, and P.A. Fair. 2008. Trace element concentrations in blood of free-ranging bottlenose dolphins (*Tursiops truncatus*): Influence of age, sex and location. Marine Pollution Bulletin 56(2):371–379. doi: 10.1016/j.marpolbul.2007.10.030

Stavros, H.-C.W., M. Stolen, W.N. Durden, W. McFee, G.D. Bossart, and P.A. Fair. 2011. Correlation and toxicological inference of trace elements in tissues from stranded and free-ranging bottlenose dolphins (*Tursiops truncatus*). Chemosphere 82(11):1649–1661. doi: 10.1016/j.chemosphere.2010.11.019

Taylor, B.L., S.J. Chivers, J. Larese, and W.F. Perrin. 2007. Generation length and percent mature estimates for IUCN assessments of cetaceans. National Oceanic and Atmospheric Administration, National Marine Fisheries Service, Southwest Fisheries Science Center: La Jolla, CA. Administrative Report LJ-07-01. 24 p.

Thayer, V.G. 2008. Life history parameters and social associations of female bottlenose dolphins (*Tursiops truncatus*) off North Carolina, USA. Duke University. Dissertation. 164 p.

Thayer, V.G., A.J. Read, A.S. Friedlaender, D.R. Colby, A.A. Hohn, W.A. McLellan, D.A. Pabst, J.L. Dearolf, N.I. Bowles, J.R. Russell, and K.A. Rittmaster. 2003. Reproductive seasonality of western Atlantic bottlenose dolphins off North Carolina, USA. Marine Mammal Science 19(4):617–629. doi: 10.1111/j.1748-7692.2003.tb01120.x

Titcomb, E.M., J.S. Reif, P.A. Fair, H.-C.W. Stavros, M. Mazzoil, G.D. Bossart, and A.M. Schaefer. 2017. Blood mercury concentrations in common bottlenose dolphins from the Indian River Lagoon, Florida: Patterns of social distribution. Marine Mammal Science 33(3):771–784. doi: 10.1111/mms.12390

Torres, L.G. 2007. Top predator distribution and foraging ecology in Florida Bay, Florida. Duke University. Dissertation. 225 p.

Torres, L.G. and A.J. Read. 2009. Where to catch a fish? The influence of foraging tactics on the ecology of bottlenose dolphins (*Tursiops truncatus*) in Florida Bay, Florida. Marine Mammal Science 25(4):797–815. doi: 10.1111/j.1748-7692.2009.00297.x

Urian, K.W., D.M. Waples, R.B. Tyson, L.E. Hodge, and A.J. Read. 2013. Abundance of bottlenose dolphins (*Tursiops truncatus*) in estuarine and near-shore waters of North Carolina, USA. Journal of the North Carolina Academy of Science 129(4):165–171. doi: 10.7572/2167-5880-129.4.165

Vollmer, N.L. and P.E. Rosel. 2013. A review of common bottlenose dolphins (*Tursiops truncatus truncatus*) in the Northern Gulf of Mexico: Population biology, potential threats, and management. Southeastern Naturalist 12(m6):1–43. doi: 10.1656/058.012.m601

Wells, R.S. 2000. Reproduction in wild bottlenose dolphins: Overview of patterns observed during a long-term study. In: Duffield, D. and T. Robeck (Eds.), Bottlenose Dolphin Reproduction Workshop. AZA Marine Mammal Taxon Advisory Group, Silver Spring, MD. pp. 57–74.

Wells, R.S. 2003. Dolphin social complexity: Lessons from long-term study and life history. In: de Waal, F.B.M. and P.L. Tyack (Eds.), Animal Social Complexity: Intelligence, Culture, and Individualized Societies Harvard University Press, Cambridge, MA. pp. 32–56.

Wells, R.S. 2014. Social structure and life history of bottlenose dolphins near Sarasota Bay, Florida: Insights from four decades and five generations. Primates and cetaceans. pp. 149–172. doi: 10.1007/978-4-431-54523-1_8

Wells, R.S., A. Natoli, and G. Braulik. 2019. *Tursiops truncatus* (errata version published in 2019). *The IUCN Red List of Threatened Species* 2019: e.T22563A156932432. https://dx.doi.org/10.2305/IUCN.UK.2019-1.RLTS.T22563A156932432.en

Wells, R.S. and M.D. Scott. 1999. Bottlenose dolphin *Tursiops truncatus* (Montagu, 1821). In: Ridgway, S.H. and R. Harrison (Eds.), Handbook of marine mammals. Volume 6: The second book of dolphins and the porpoises. Academic Press, San Diego, CA. pp. 137–182.

Wells, R.S. and M.D. Scott. 2018. Bottlenose dolphin, *Tursiops truncatus*, common bottlenose dolphin. In: Würsig, B., J.G.M. Thewissen, and K.M. Kovacs (Eds.), Encyclopedia of Marine Mammals (Third Edition). Academic Press, London, UK. pp. 118–125. doi: 10.1016/B978-0-12-804327-1.00072-8

Wells, R.S., M.D. Scott, and A.B. Irvine. 1987. The social structure of free-ranging bottlenose dolphins. In: Genoways, H.H. (Ed.), Current Mammalogy. Plenum Press, New York, NY. pp. 247–305.

Wilson, R.M., R.B. Tyson, J.A. Nelson, B.C. Balmer, J.P. Chanton, and D.P. Nowacek. 2017. Niche differentiation and prey selectivity among common bottlenose dolphins (*Tursiops truncatus*) sighted in St. George Sound, Gulf of Mexico. Frontiers in Marine Science 4:235. doi: 10.3389/fmars.2017.00235

Yordy, J.E., M.A. Mollenhauer, R.M. Wilson, R.S. Wells, A. Hohn, J. Sweeney, L.H. Schwacke, T.K. Rowles, J.R. Kucklick, and M.M. Peden-Adams. 2010a. Complex contaminant exposure in cetaceans: a comparative E-Screen analysis of bottlenose dolphin blubber and mixtures of four persistent organic pollutants. Environmental Toxicology and Chemistry 29(10):2143–2153. doi: 10.1002/etc.279

Yordy, J.E., D.A. Pabst, W.A. McLellan, R.S. Wells, T.K. Rowles, and J.R. Kucklick. 2010b. Tissue-specific distribution and whole-body burden estimates of persistent organic pollutants in the bottlenose

dolphin (*Tursiops truncatus*). Environmental Toxicology and Chemistry 29(6):1263–1273. doi: 10.1002/etc.152

Common bottlenose dolphin − *Tursiops truncatus*

Western North Atlantic migratory stocks

Overall Vulnerability Rank = High

Biological Sensitivity = Moderate

Climate Exposure = Very High

Data Quality = 96% of scores ≥ 2

	Tursiops truncatus	Expert Scores	Data Quality	Expert Scores Plots (Portion by Category)
Sensitivity attributes	Prey/Diet Specificity	1.9	2.5	
	Habitat Specificity	2.0	2.5	
	Site Fidelity	2.0	2.0	
	Lifetime Reproductive Potential	1.2	2.2	
	Generation Length	2.8	2.2	
	Reproductive Plasticity	1.4	1.5	
	Migration	2.4	2.5	
	Home Range	2.0	2.8	
	Stock Abundance	1.6	2.0	
	Stock Abundance Trend	2.8	2.0	
	Cumulative Stressors	2.5	2.5	
	Sensitivity Score	Moderate		
Exposure variables	Sea Surface Temperature (Change in mean)	4.0	2.5	
	Sea Surface Temperature (Change in variability)	1.0	2.5	
	Air Temperature (Change in mean)	4.0	2.5	
	Air Temperature (Change in variability)	1.0	2.5	
	Precipitation (Change in mean)	1.0	2.5	
	Precipitation (Change in variability)	1.4	2.5	
	Sea Surface Salinity (Change in mean)	2.4	2.5	
	Sea Surface Salinity (Change in variability)	2.6	2.5	
	Ocean pH (Change in mean)	4.0	2.5	
	Ocean pH (Change in variability)	1.1	2.5	
	Sea ice coverage (Change in mean)	1.0	2.5	
	Sea ice coverage (Change in variability)	1.0	2.5	
	Dissolved oxygen (Change in mean)	4.0	2.5	
	Dissolved oxygen (Change in variability)	1.2	2.5	
	Circulation	2.1	2.2	
	Sea level rise	3.8	2.5	
	Exposure Score	Very High		
	Overall Vulnerability Rank	High		

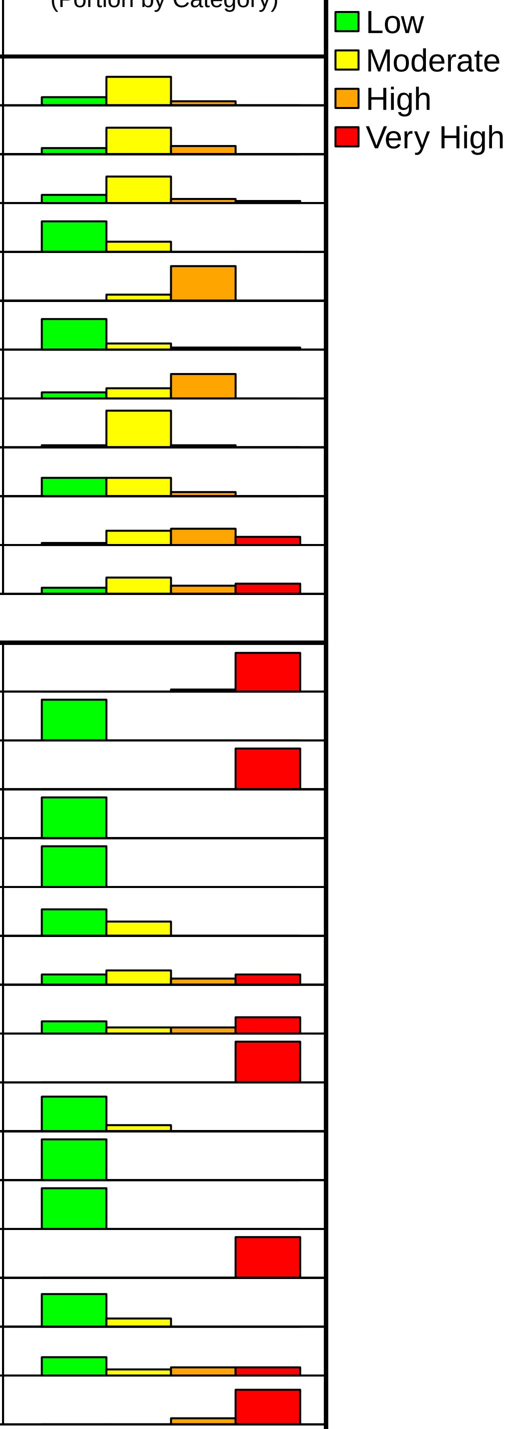

Common bottlenose dolphin (Western North Atlantic, Northern and Southern Migratory Coastal Stocks)

Tursiops truncatus

CVA Results Summary

Overall Climate Vulnerability Rank: High (98% certainty from bootstrap analysis).

Climate Exposure: Very High. Five exposure factors scored greater than 3.5: Air Temperature (Standard anomaly) (4.00), Dissolved oxygen (Standard anomaly) (4.00), Ocean pH (Standard anomaly) (4.00), Sea Surface Temperature (Standard anomaly) (3.95), and Sea level rise (3.85).

Biological Sensitivity: Moderate. Three sensitivity attributes scored greater than 2.5: Generation Time (2.85), Species Abundance Trend (2.75), Cumulative Stressors (2.55).

Distributional Response: High

Abundance Response: Moderate

Phenology Response: Moderate

Data Quality: 96% of the data quality scores were 2 or greater, 91% of sensitivity attribute data quality scores were 2 or higher.

Background Information

Stock Description

This stock group includes the western North Atlantic common bottlenose dolphin Northern Migratory Coastal Stock and Southern Migratory Coastal Stock.

The Northern Migratory Coastal Stock overlaps the least with other stocks during the warm water months (July and August) and is best defined during this time period when the animals occupy waters from the shoreline to approximately the 20-m isobath between Assateague, Virginia and Long Island, New York. The offshore boundary of the Northern Migratory Stock is the 20-m isobath in the summer north of Cape Hatteras, North Carolina and the 200-m isobath in the winter between Cape Hatteras and Cape Lookout, North Carolina (Hayes et al. 2021).

The Southern Migratory Coastal Stock are poorly understood and thought to migrate seasonally along the Atlantic coast between North Carolina and northern Florida. The offshore boundary of the Southern Migratory Stock is the 20-m isobath north of Cape Hatteras and the 200-m isobath south of Cape Hatteras (Hayes et al. 2021).

Prey/Diet Specificity

Generally, common bottlenose dolphins consume a large variety of fish and/or squid (Barros and Odell 1990; Mead and Potter 1990; Wells and Scott 2018). Common bottlenose dolphins seem to show a consistent preference for sound-producing fishes (e.g., sciaenids, toadfish; Berens McCabe et al. 2010;

Bowen-Stevens et al. 2021). Estuarine stocks along the southeast U.S. Atlantic coast have been reported to consume a variety of prey, with preference for sciaenids (Gannon and Waples 2004; Pate and McFee 2012). Pate and McFee (2012) identified 42 prey species from 24 families in a study of stranded dolphins along the South Carolina coast. In Sarasota Bay, Florida, Rossman et al. (2015) found some individual common bottlenose dolphins to show diet specialization despite the broader study group showing a generalist diet. Specialization can be influenced by locally abundant prey species (Shane et al. 1986; Vollmer and Rosel 2013) or can result from resource partitioning amongst sympatric populations (e.g., Torres and Read 2009; Wilson et al. 2017).

Habitat Specificity

Common bottlenose dolphins are found in a variety of marine and estuarine habitats (Wells and Scott 2018). Sea surface temperature and chlorophyll a have been correlated with common bottlenose dolphin passive acoustic monitoring detections in the coastal waters of Maryland (Bailey et al. 2021). In coastal waters off of New Jersey, two clusters of common bottlenose dolphin have been identified that differ in depth and distance from shore (Toth et al. 2012).

Site Fidelity

No specific site fidelity information was found for this stock.

Lifetime Reproductive Potential

Taylor et al. (2007) reported the interbirth interval of common bottlenose dolphins as 3.80 years, though other studies have shown it to be closer to three years (Leatherwood 1977; Wells and Scott 1999; Connor et al. 2000; Wells 2003). Thayer (2008) estimated mean interbirth interval of 2.9 years in North Carolina.

Generally, females reach sexual maturity before males, with females reaching sexual maturity at 5–13 years (Sergeant et al. 1973; Perrin and Reilly 1984; Wells et al. 1987; Mead and Potter 1990; Mann et al. 2000; Wells 2000; Wells and Scott 2018).

Females reproduce through most of their adult lives (Leatherwood 1977; Hohn et al. 1989; Wells and Scott 1999; Wells 2000; Wells 2003), but may produce fewer calves after they reach 30 years of age (Wells 2000). Sarasota Bay common bottlenose dolphins can give birth when they are as young as 6 years of age, and can continue until they are 48 years old (Wells and Scott 2018). Reproductive lifespan would therefore be up to 42 years based on long-term observation studies (Wells 2014; Wells and Scott 2018).

Longitudinal studies from Sarasota Bay, Florida have found that female bottlenose dolphins can live up to 67 years (Wells and Scott 2018). Studies based on growth layer groups in teeth suggest female bottlenose dolphins can live to 41 years along the Texas coast (Fernandez and Hohn 1998) and to 30 years along the Mississippi coast (Mattson et al. 2006).

Generation Length

Taylor et al. (2007) reported common bottlenose dolphin generation length of 20.6–21.1 years.

Reproductive Plasticity

Common bottlenose dolphin calving is generally diffusely seasonal, with one or more peaks, although births have been reported from all seasons (Vollmer and Rosel 2013; Wells and Scott 2018). Calf sightings in South Carolina peak in spring and early summer and again in early autumn (McFee et al. 2014). In North Carolina, Thayer et al. (2003) observed a strong birth peak in spring based on stranding data and a diffuse birth peak from late spring to early fall based on sightings. Calf sightings peak in the spring and summer months in Florida (Wells et al. 1987), Mississippi/Louisiana (Miller et al. 2010; Miller et al. 2013), and Texas (McHugh 1989; Henderson and Würsig 2007).

Migration

During winter months, the Northern Migratory Coastal Stock moves from waters as far north as New Jersey south to waters near Cape Lookout, North Carolina (Kenney 1990; Read et al. 2003; Torres et al. 2005). During January and February, the Southern Migratory Coastal Stock migrates south from waters of southern Virginia and north central North Carolina to waters south of Cape Fear, North Carolina, and as far south as northern Florida (Barco et al. 1999).

Home Range

Data are insufficient to characterize home range patterns (or the lack thereof) for these migratory stocks. Tracking studies have shown common bottlenose dolphins can travel distances greater than 2000 km (Wells et al. 1999). Estrada and Hohn (2003) observed migrating bottlenose dolphins along the East Coast of the United States travel a mean distance of 32.7 km/day while non-migrating dolphins traveled a mean distance of 24.5 km/day. Klatsky et al. (2007) found dolphins off Bermuda traveled a mean distance of 28.3 km/day.

Stock Abundance

The best available abundance estimate for the Northern Migratory Coastal Stock of common bottlenose dolphins in the western North Atlantic is 6,639 (CV=0.41; Garrison et al. 2017). This estimate was derived from aerial surveys conducted during the summer of 2016 covering coastal and shelf waters from Assateague, Virginia, to Sandy Hook, New Jersey.

The best available abundance estimate for the Southern Migratory Coastal Stock of common bottlenose dolphins in the western North Atlantic is 3,751 (CV=0.60; Garrison et al. 2017). This estimate was derived from aerial surveys conducted during the summer of 2016 covering coastal and shelf waters from Florida to New Jersey.

Stock Abundance Trend

Although there was no statistically significant difference in abundance for this stock between the 2010–2011 and 2016 surveys, a statistically significant decline in population size of all common bottlenose dolphins in coastal waters from New Jersey to Florida between 2010–2011 and 2016 was detected (Garrison et al. 2017), concurrent with a large UME in the area; however, there is limited power to evaluate trends given uncertainty in stock distribution, lack of precision in abundance estimates, and a limited number of surveys (Hayes et al. 2021).

Cumulative Stressors

Common bottlenose dolphins face a wide range of individual and cumulative natural and anthropogenic threats (see reviews by Vollmer and Rosel 2013; Wells and Scott 2018; and Wells et al. 2019). These include such threats as shark predation, harmful algal blooms, recreational and commercial fishing gear (Barco et al. 2010; Hall et al. 2013; Byrd et al. 2014; Wirth and Warren 2019; Epple et al. 2020), vessel traffic, pollution, habitat alteration, and provisioning.

Common bottlenose dolphins in the Northern Migratory Coastal Stock interact with several commercial fisheries. These include gillnet, pound net, menhaden purse seine, blue crab trap/pot, haul/beach seine, and hook and line fisheries. The minimum total mean annual human-caused mortality and serious injury for fisheries and other human causes ranged between 12.2 and 21.5 individuals per year during 2014–2018 (Hayes et al. 2021). During 2014–2018, 692 common bottlenose dolphins stranded that were assigned to the Northern Migratory Coastal Stock, with 80 of these strandings showing evidence of human interaction such as fishing gear and vessel interactions (Hayes et al. 2021; also see NOAA National Marine Mammal Health and Stranding Response Database).

Common bottlenose dolphins in the Southern Migratory Coastal Stock interact with several commercial fisheries, including gillnet, pound net, blue crab trap/pot, roe mullet stop net, menhaden purse seine, haul/beach seine, shrimp trawl, and hook and line fisheries. The minimum total mean annual human-caused mortality and serious injury for fisheries and other human causes ranged between 0 and 18.3 individuals per year during 2014–2018 (Hayes et al. 2021). During 2014–2018, 565 common bottlenose dolphins stranded that were assigned to the Southern Migratory Coastal Stock, with 59 of these strandings showing evidence of human interaction such as fishing gear and vessel interactions (Hayes et al. 2021; also see NOAA National Marine Mammal Health and Stranding Response Database).

An Unusual Mortality Event (UME) was declared in the summer of 2013 for the mid-Atlantic coast from New York to Brevard County, Florida, with more than 1600 common bottlenose dolphins stranding as a result of morbillivirus (Hayes et al. 2021). Strandings were more prevalent along oceanic shores than in estuaries, suggesting that coastal stocks were more impacted by the UME than estuarine stocks (Hayes et al. 2021). Almost 350 strandings were recovered from New York, New Jersey, Delaware, and Maryland. The majority of these likely came from the Northern Migratory Coastal Stock. It is unknown how many dolphins from the Southern Migratory Coastal Stocks died during the event (Hayes et al. 2021).

The nearshore and estuarine habitats along the Atlantic seaboard are adjacent to areas of high human population and some are highly industrialized. Studies of bottlenose dolphin tissues from estuaries in the southeast U.S. have shown persistent organic pollutant (POP; Schwacke et al. 2002, 2012; Hansen et al. 2004; Litz et al. 2007; Pulster et al. 2009; Fair et al. 2010; Yordy et al. 2010; Balmer et al. 2011; Kucklick et al. 2011; ; Reif et al. 2017) and heavy metal (Durden et al. 2007; Stavros et al. 2007, 2008, 2011; Titcomb et al. 2017; Reif et al. 2017; Page-Karjian et al. 2020) concentrations that may result in adverse effects on health or reproductive rates, particularly in urbanized, developed, and agricultural areas.

Storm events have been shown to affect common bottlenose dolphin distribution and foraging (Fandel et al. 2020).

Distribution and Sightings

Density model results for common bottlenose dolphins in the western North Atlantic are presented by Roberts et al. (2016a, 2016b, 2017, 2018), Mannocci et al. (2017), and Palka et al. (2021).

Further Reading

Hayes, S.A., E. Josephson, K. Maze-Foley, P.E. Rosel, and J. Turek. 2021. US Atlantic and Gulf of Mexico Marine Mammal Stock Assessments - 2020. National Oceanic and Atmospheric Administration, National Marine Fisheries Service, Northeast Fisheries Science Center: Woods Hole, MA. NOAA Technical Memorandum NMFS-NE-271. 394 p.

Wells, R.S. and M.D. Scott. 2018. Bottlenose Dolphin, *Tursiops truncatus*, Common Bottlenose Dolphin. In: Würsig, B., J.G.M. Thewissen, and K.M. Kovacs (Eds.), Encyclopedia of Marine Mammals (Third Edition). Academic Press, London, UK. pp. 118–125. doi: 10.1016/B978-0-12-804327-1.00072-8

Wells, R.S., A. Natoli, and G. Braulik. 2019. *Tursiops truncatus* (errata version published in 2019). The IUCN Red List of Threatened Species 2019: e.T22563A156932432. https://dx.doi.org/10.2305/IUCN.UK.2019-1.RLTS.T22563A156932432.en

References

Bailey, H., A.D. Fandel, K. Silva, E. Gryzb, E. McDonald, A.L. Hoover, M.B. Ogburn, and A.N. Rice. 2021. Identifying and predicting occurrence and abundance of a vocal animal species based on individually specific calls. Ecosphere 12(8):e03685. doi: 10.1002/ecs2.3685

Balmer, B.C., L.H. Schwacke, R.S. Wells, R.C. George, J. Hoguet, J.R. Kucklick, S.M. Lane, A. Martinez, W.A. McLellan, P.E. Rosel, T.K. Rowles, K. Sparks, T. Speakman, E.S. Zolman, and D.A. Pabst. 2011. Relationship between persistent organic pollutants (POPs) and ranging patterns in common bottlenose dolphins (*Tursiops truncatus*) from coastal Georgia, USA. Science of the Total Environment 409(11):2094–2101. doi: 10.1016/j.scitotenv.2011.01.052

Barco, S.G., L.R. D'Eri, B.L. Woodward, J.P. Winn, and D.S. Rotstein. 2010. Spectra (R) fishing twine entanglement of a bottlenose dolphin: A case study and experimental modeling. Marine Pollution Bulletin 60(9):1477–1481. doi: 10.1016/j.marpolbul.2010.05.005

Barco, S.G., W.M. Swingle, W.A. Mclellan, R.N. Harris, and D.A. Pabst. 1999. Local abundance and distribution of bottlenose dolphins (*Tursiops truncatus*) in the nearshore waters of Virginia Beach, Virginia. Marine Mammal Science 15(2):394–408. doi: 10.1111/j.1748-7692.1999.tb00809.x

Barros, N.B. and D.K. Odell. 1990. Food habits of bottlenose dolphins in the southeastern United States In: Leatherwood, S. and R.R. Reeves (Eds.), The Bottlenose Dolphin. Academic Press, San Diego, CA. pp. 309–328. doi: 10.1016/B978-0-12-440280-5.50020-2

Berens McCabe, E.J., D.P. Gannon, N.B. Barros, and R.S. Wells. 2010. Prey selection by resident common bottlenose dolphins (*Tursiops truncatus*) in Sarasota Bay, Florida. Marine Biology 157(5):931–942. doi: 10.1007/s00227-009-1371-2

Bowen-Stevens, S.R., D.P. Gannon, R.A. Hazelkorn, G. Lovewell, K.M. Volker, S. Smith, M.C. Tumlin, and J. Litz. 2021. Diet of common bottlenose dolphins, *Tursiops truncatus*, that stranded in and near Barataria Bay, Louisiana, 2010–2012. Southeastern Naturalist 20(1). doi: 10.1656/058.020.0113

Byrd, B.L., A.A. Hohn, G.N. Lovewell, K.M. Altman, S.G. Barco, A. Friedlaender, C.A. Harms, W.A. McLellan, K.T. Moore, P.E. Rosel, and V.G. Thayer. 2014. Strandings as indicators of marine mammal biodiversity and human interactions off the coast of North Carolina. Fishery Bulletin 112(1):1–23. doi: 10.7755/fb.112.1.1

Connor, R.C., R.S. Wells, J. Mann, and A.J. Read. 2000. The bottlenose dolphin: Social relationships in a fission-fusion society. In: Mann, J., R. Connor, P. Tyack, and H. Whitehead (Eds.), Cetacean Societies: Field Studies of Dolphins and Whales. University of Chicago Press, Chicago, IL. pp. 91–126.

Durden, W.N., M.K. Stolen, D.H. Adams, and E.D. Stolen. 2007. Mercury and selenium concentrations in stranded bottlenose dolphins from the Indian River Lagoon system, Florida. Bulletin of Marine Science 81(1):37–54.

Epple, A.L., J.T. Daniel, S.G. Barco, D.S. Rotstein, and A.M. Costidis. 2020. Novel necropsy findings linked to peracute underwater entrapment in bottlenose dolphins (*Tursiops truncatus*). Frontiers in Marine Science 7:503. doi: 10.3389/fmars.2020.00503

Estrada, E.L. and A.A. Hohn. 2003. Satellite monitored movements of bottlenose dolphins along the Atlantic coast of the US. 15th Biennial Conference on the Biology of Marine Mammals, Greensboro, NC, 14-19 December 2003

Fair, P.A., J. Adams, G. Mitchum, T.C. Hulsey, J.S. Reif, M. Houde, D. Muir, E. Wirth, D. Wetzel, E. Zolman, W. McFee, and G.D. Bossart. 2010. Contaminant blubber burdens in Atlantic bottlenose dolphins (*Tursiops truncatus*) from two southeastern US estuarine areas: Concentrations and patterns of PCBs, pesticides, PBDEs, PFCs, and PAHs. Science of the Total Environment 408(7):1577–1597. doi: 10.1016/j.scitotenv.2009.12.021

Fandel, A.D., A. Garrod, A.L. Hoover, J.E. Wingfield, V. Lyubchich, D.H. Secor, K.B. Hodge, A.N. Rice, and H. Bailey. 2020. Effects of intense storm events on dolphin occurrence and foraging behavior. Scientific Reports 10(1):19247. doi: 10.1038/s41598-020-76077-3

Fernandez, S. and A.A. Hohn. 1998. Age, growth, and calving season of bottlenose dolphins, *Tursiops truncatus*, off coastal Texas. Fishery Bulletin 96(2):357–365.

Gannon, D.P. and D.M. Waples. 2004. Diets of coastal bottlenose dolphins from the U. S. Mid-Atlantic coast differ by habitat. Marine Mammal Science 20(3):527–545. doi: 10.1111/j.1748-7692.2004.tb01177.x

Garrison, L.P., A.A. Hohn, and L.J. Hansen. 2017. Seasonal movements of Atlantic common bottlenose dolphin stocks based on tag telemetry data. National Oceanic and Atmospheric Administration, National Marine Fisheries Service, Southeast Fisheries Science Center, Protected Resources and Biodiversity Division: Miami, FL. PRBD-2017-02. 75 p.

Hall, A.G., J.B. McNeill, P.B. Conn, E. Davenport, and A.A. Hohn. 2013. Seasonal co-occurrence of sea turtles, bottlenose dolphins, and commercial gill nets in southern Pamlico and northern Core Sounds, and adjacent coastal waters of North Carolina, USA. Endangered Species Research 22(3):235–249. doi: 10.3354/esr00539

Hansen, L.J., L.H. Schwacke, G.B. Mitchum, A.A. Hohn, R.S. Wells, E.S. Zolman, and P.A. Fair. 2004. Geographic variation in polychorinated biphenyl and organochlorine pesticide concentrations in the blubber of bottlenose dolphins from the US Atlantic coast. Science of the Total Environment 319(1-3):147–172. doi: 10.1016/S0048-9697(03)00371-1

Hayes, S.A., E. Josephson, K. Maze-Foley, P.E. Rosel, and J. Turek. 2021. US Atlantic and Gulf of Mexico marine mammal stock assessments - 2020. National Oceanic and Atmospheric Administration, National Marine Fisheries Service, Northeast Fisheries Science Center: Woods Hole, MA. NOAA Technical Memorandum NMFS-NE-271. 394 p.

Henderson, E.E. and B. Würsig. 2007. Behavior patterns of bottlenose dolphins in San Luis Pass, Texas. Gulf of Mexico Science 25(2):153–161. doi: 10.18785/goms.2502.06

Hohn, A.A., M.D. Scott, R.S. Wells, J.C. Sweeney, and A.B. Irvine. 1989. Growth layers in teeth from known-age, free-ranging bottlenose dolphins. Marine Mammal Science 5(4):315–342. doi: 10.1111/j.1748-7692.1989.tb00346.x

Kenney, R.D. 1990. Bottlenose dolphins off the northeastern United States. In: Leatherwood, S. and R.R. Reeves (Eds.), The Bottlenose Dolphin. Academic Press, San Diego, CA. pp. 369–386.

Klatsky, L.J., R.S. Wells, and J.C. Sweeney. 2007. Offshore bottlenose dolphins (*Tursiops truncatus*): movement and dive behavior near the Bermuda Pedestal. Journal of Mammalogy 88(1):59–66. doi: 10.1644/05-mamm-a-365r1.1

Kucklick, J., L. Schwacke, R. Wells, A. Hohn, A. Guichard, J. Yordy, L. Hansen, E. Zolman, R. Wilson, J. Litz, D. Nowacek, T. Rowles, R. Pugh, B. Balmer, C. Sinclair, and P. Rosel. 2011. Bottlenose dolphins as indicators of persistent organic pollutants in the western North Atlantic Ocean and Northern Gulf of Mexico. Environmental Science & Technology 45(10):4270–4277. doi: 10.1021/es1042244

Leatherwood, S. 1977. Some preliminary impressions on the numbers and social behavior of free-swimming bottlenosed dolphin calves (*Tursiops truncatus*) in the northern Gulf of Mexico. In: Ridgway, S.H. and K. Bernirschke (Eds.), Breeding Dolphins; Present Status, Suggestions for the Future. Final report to the US Marine Mammal Commission, Contract MM6AC009, MMC-76/07, Washington, DC. pp. 143–167.

Litz, J.A. 2007. Social structure, genetic structure, and persistent organohalogen pollutants in bottlenose dolphins (*Tursiops truncatus*) in Biscayne Bay, Florida. University of Miami. 140 p.

Mann, J., R.C. Connor, L.M. Barre, and M.R. Heithaus. 2000. Female reproductive success in bottlenose dolphins (*Tursiops* sp.): life history, habitat, provisioning, and group-size effects. Behavioral Ecology 11(2):210–219. doi: 10.1093/beheco/11.2.210

Mannocci, L., J.J. Roberts, D.L. Miller, and P.N. Halpin. 2017. Extrapolating cetacean densities to quantitatively assess human impacts on populations in the high seas. Conservation Biology 31(3):601–614. doi: 10.1111/cobi.12856

Mattson, M.C., K.D. Mullin, G.W. Ingram, and W. Hoggard. 2006. Age structure and growth of the bottlenose dolphin (*Tursiops truncatus*) from strandings in the Mississippi sound region of the north-central Gulf of Mexico from 1986 to 2003. Marine Mammal Science 22(3):654–666. doi: 10.1111/j.1748-7692.2006.00057.x

McFee, W.E., T.R. Speakman, L. Balthis, J.D. Adams, and E.S. Zolman. 2014. Reproductive seasonality of a recently designated bottlenose dolphin stock near Charleston, South Carolina, U.S.A. Marine Mammal Science 30(2):528–543. doi: 10.1111/mms.12055

McHugh, M.B. 1989. Population numbers and feeding behavior of the Atlantic bottlenose dolphin (*Tursiops truncatus*) near Aransas Pass, Texas. University of Texas at Austin. Thesis. 96 p.

Mead, J.G. and C.W. Potter. 1990. Natural history of bottlenose dolphins along the central Atlantic coast of the United States. In: Leatherwood, S. and R.R. Reeves (Eds.), The Bottlenose Dolphin. Academic Press, San Diego, CA. pp. 165–195.

Miller, L.J., A.D. Mackey, T. Hoffland, M. Solangi, and S.A. Kuczaj. 2010. Potential effects of a major hurricane on Atlantic bottlenose dolphin (*Tursiops truncatus*) reproduction in the Mississippi Sound. Marine Mammal Science 26(3):707–715. doi: 10.1111/j.1748-7692.2010.00371.x

Miller, L.J., A.D. Mackey, M. Solangi, and S.A. Kuczaj. 2013. Population abundance and habitat utilization of bottlenose dolphins in the Mississippi Sound. Aquatic Conservation: Marine and Freshwater Ecosystems 23(1):145–151. doi: 10.1002/aqc.2278

Page-Karjian, A., C.F. Lo, B. Ritchie, C.A. Harms, D.S. Rotstein, S. Han, S.M. Hassan, A.F. Lehner, J.P. Buchweitz, V.G. Thayer, J.M. Sullivan, E.F. Christiansen, and J.R. Perrault. 2020. Anthropogenic contaminants and histopathological findings in stranded cetaceans in the southeastern United States, 2012–2018. Frontiers in Marine Science 7:630. doi: 10.3389/fmars.2020.00630

Palka, D., L. Aichinger Dias, E. Broughton, S. Chavez-Rosales, D. Cholewiak, G. Davis, A. DeAngelis, L. Garrison, H. Haas, J. Hatch, K. Hyde, M. Jech, E. Josephson, L. Mueller-Brennan, C. Orphanides, N. Pegg, C. Sasso, D. Sigourney, M. Soldevilla, and H. Walsh. 2021. Atlantic Marine Assessment

Program for Protected Species: FY15 – FY19. US Department of the Interior, Bureau of Ocean Energy Management: Washington, DC. OCS Study BOEM 2021-051. 330 p.
Pate, S.M. and W.E. McFee. 2012. Prey species of bottlenose dolphins (*Tursiops truncatus*) from South Carolina Waters. Southeastern Naturalist 11(1):1–22. doi: 10.1656/058.011.0101
Perrin, W.F. and S.B. Reilly. 1984. Reproductive parameters of dolphins and small whales of the family Delphinidae. In: Perrin, W.F., R.L. Brownell, Jr., and D.P. DeMaster (Eds.), Reproduction in Whales, Dolphins, and Porpoises. pp. 97–134.
Pulster, E.L., K.L. Smalling, E. Zolman, L. Schwacke, and K.A. Maruya. 2009. Persistent organochlorine pollutants and toxaphene congener profiles in bottlenose dolphins (*Tursiops truncatus*) frequenting the Turtle/Brunswick River Estuary, Georgia, USA. Environmental Toxicology and Chemistry 28(7):1390–1399. doi: 10.1897/08-240.1
Read, A.J., K.W. Urian, B. Wilson, and D.M. Waples. 2003. Abundance of bottlenose dolphins in the bays, sounds, and estuaries of North Carolina. Marine Mammal Science 19(1):59–73. doi: 10.1111/j.1748-7692.2003.tb01092.x
Reif, J.S., A.M. Schaefer, G.D. Bossart, and P.A. Fair. 2017. Health and Environmental Risk Assessment Project for bottlenose dolphins *Tursiops truncatus* from the southeastern USA. II. Environmental aspects. Diseases of Aquatic Organisms 125(2):155–166. doi: 10.3354/dao03143
Roberts, J.J., B.D. Best, L. Mannocci, E. Fujioka, P.N. Halpin, D.L. Palka, L.P. Garrison, K.D. Mullin, T.V.N. Cole, C.B. Khan, W.A. McLellan, D.A. Pabst, and G.G. Lockhart. 2016a. Habitat-based cetacean density models for the US Atlantic and Gulf of Mexico. Scientific Reports 6:22615. doi: 10.1038/Srep22615
Roberts, J.J., L. Mannocci, and P.N. Halpin. 2016b. Final project report: Marine species density data gap assessments and update for the AFTT Study Area, 2015-2016 (Base Year). Document version 1.0. Report prepared for Naval Facilities Engineering Command, Atlantic by the Duke University Marine Geospatial Ecology Lab: Durham, NC. 21 p.
Roberts, J.J., L. Mannocci, and P.N. Halpin. 2017. Final Project Report: Marine Species Density Data Gap Assessments and Update for the AFTT Study Area, 2016-2017 (Opt. Year 1). Document version 1.4. Report prepared for Naval Facilities Engineering Command, Atlantic by the Duke University Marine Geospatial Ecology Lab: Durham, NC. 76 p.
Roberts, J.J., L. Mannocci, R.S. Schick, and P.N. Halpin. 2018. Final Project Report: Marine Species Density Data Gap Assessments and Update for the AFTT Study Area, 2017-2018 (Opt. Year 2). Document version 1.2. Report prepared for Naval Facilities Engineering Command, Atlantic by the Duke University Marine Geospatial Ecology Lab: Durham, NC. 113 p.
Rossman, S., P.H. Ostrom, M. Stolen, N.B. Barros, H. Gandhi, C.A. Stricker, and R.S. Wells. 2015. Individual specialization in the foraging habits of female bottlenose dolphins living in a trophically diverse and habitat rich estuary. Oecologia 178(2):415–425. doi: 10.1007/s00442-015-3241-6
Schwacke, L.H., E.O. Voit, L.J. Hansen, R.S. Wells, G.B. Mitchum, A.A. Hohn, and P.A. Fair. 2002. Probabilistic risk assessment of reproductive effects of polychlorinated biphenyls on bottlenose dolphins (*Tursiops truncatus*) from the southeast United States coast. Environmental Toxicology and Chemistry 21(12):2752–2764. doi: 10.1002/etc.5620211232
Schwacke, L.H., E.S. Zolman, B.C. Balmer, S. De Guise, R.C. George, J. Hoguet, A.A. Hohn, J.R. Kucklick, S. Lamb, M. Levin, J.A. Litz, W.E. McFee, N.J. Place, F.I. Townsend, R.S. Wells, and T.K. Rowles. 2012. Anaemia, hypothyroidism and immune suppression associated with polychlorinated biphenyl exposure in bottlenose dolphins (*Tursiops truncatus*). Proceedings of the Royal Society B: Biological Sciences 279(1726):48–57. doi: 10.1098/rspb.2011.0665

Sergeant, D.E., D.K. Caldwell, and M.C. Caldwell. 1973. Age, growth, and maturity of bottlenosed dolphin (*Tursiops-truncatus*) from northeast Florida. Journal of the Fisheries Research Board of Canada 30(7):1009–1011. doi: 10.1139/f73-165

Shane, S.H., R.S. Wells, and B. Würsig. 1986. Ecology, behavior and social organization of the bottlenose dolphin: A review. Marine Mammal Science 2:34–63. doi: 10.1111/j.1748-7692.1986.tb00026.x

Stavros, H.-C.W., G.D. Bossart, T.C. Hulsey, and P.A. Fair. 2007. Trace element concentrations in skin of free-ranging bottlenose dolphins (*Tursiops truncatus*) from the southeast Atlantic coast. Science of the Total Environment 388(1-3):300–315. doi: 10.1016/j.scitotenv.2007.07.030

Stavros, H.-C.W., G.D. Bossart, T.C. Hulsey, and P.A. Fair. 2008. Trace element concentrations in blood of free-ranging bottlenose dolphins (*Tursiops truncatus*): Influence of age, sex and location. Marine Pollution Bulletin 56(2):371–379. doi: 10.1016/j.marpolbul.2007.10.030

Stavros, H.-C.W., M. Stolen, W.N. Durden, W. McFee, G.D. Bossart, and P.A. Fair. 2011. Correlation and toxicological inference of trace elements in tissues from stranded and free-ranging bottlenose dolphins (*Tursiops truncatus*). Chemosphere 82(11):1649–1661. doi: 10.1016/j.chemosphere.2010.11.019

Taylor, B.L., S.J. Chivers, J. Larese, and W.F. Perrin. 2007. Generation length and percent mature estimates for IUCN assessments of cetaceans. National Oceanic and Atmospheric Administration, National Marine Fisheries Service, Southwest Fisheries Science Center: La Jolla, CA. Administrative Report LJ-07-01. 24 p.

Thayer, V.G. 2008. Life history parameters and social associations of female bottlenose dolphins (*Tursiops truncatus*) off North Carolina, USA. Duke University. Dissertation. 164 p.

Thayer, V.G., A.J. Read, A.S. Friedlaender, D.R. Colby, A.A. Hohn, W.A. McLellan, D.A. Pabst, J.L. Dearolf, N.I. Bowles, J.R. Russell, and K.A. Rittmaster. 2003. Reproductive seasonality of western Atlantic bottlenose dolphins off North Carolina, USA. Marine Mammal Science 19(4):617–629. doi: 10.1111/j.1748-7692.2003.tb01120.x

Titcomb, E.M., J.S. Reif, P.A. Fair, H.-C.W. Stavros, M. Mazzoil, G.D. Bossart, and A.M. Schaefer. 2017. Blood mercury concentrations in common bottlenose dolphins from the Indian River Lagoon, Florida: Patterns of social distribution. Marine Mammal Science 33(3):771–784. doi: 10.1111/mms.12390

Torres, L.G., W.A. Mclellan, E. Meagher, and D.A. Pabst. 2005. Seasonal distribution and relative abundance of bottlenose dolphins, *Tursiops truncatus*, along the US mid-Atlantic coast. Journal of Cetacean Research and Management 7(2):153–161.

Torres, L.G. and A.J. Read. 2009. Where to catch a fish? The influence of foraging tactics on the ecology of bottlenose dolphins (*Tursiops truncatus*) in Florida Bay, Florida. Marine Mammal Science 25(4):797–815. doi: 10.1111/j.1748-7692.2009.00297.x

Toth, J.L., A.A. Hohn, K.W. Able, and A.M. Gorgone. 2011. Patterns of seasonal occurrence, distribution, and site fidelity of coastal bottlenose dolphins (*Tursiops truncatus*) in southern New Jersey, U.S.A. Marine Mammal Science 27(1):94–110. doi: 10.1111/j.1748-7692.2010.00396.x

Vollmer, N.L. and P.E. Rosel. 2013. A review of common bottlenose dolphins (*Tursiops truncatus truncatus*) in the Northern Gulf of Mexico: Population biology, potential threats, and management. Southeastern Naturalist 12(m6):1–43. doi: 10.1656/058.012.m601

Wells, R.S. 2000. Reproduction in wild bottlenose dolphins: Overview of patterns observed during a long-term study. In: Duffield, D. and T. Robeck (Eds.), Bottlenose Dolphin Reproduction Workshop. AZA Marine Mammal Taxon Advisory Group, Silver Spring, MD. pp. 57–74.

Wells, R.S. 2003. Dolphin social complexity: Lessons from long-term study and life history. In: de Waal, F.B.M. and P.L. Tyack (Eds.), Animal Social Complexity: Intelligence, Culture, and Individualized Societies Harvard University Press, Cambridge, MA. pp. 32–56.

Wells, R.S. 2014. Social structure and life history of bottlenose dolphins near Sarasota Bay, Florida: Insights from four decades and five generations. Primates and cetaceans. pp. 149–172. doi: 10.1007/978-4-431-54523-1_8

Wells, R.S., A. Natoli, and G. Braulik. 2019. *Tursiops truncatus* (errata version published in 2019). *The IUCN Red List of Threatened Species* 2019: e.T22563A156932432. https://dx.doi.org/10.2305/IUCN.UK.2019-1.RLTS.T22563A156932432.en

Wells, R.S., H.L. Rhinehart, P. Cunningham, J. Whaley, M. Baran, C. Koberna, and D.P. Costa. 1999. Long distance offshore movements of bottlenose dolphins. Marine Mammal Science 15(4):1098–1114. doi: 10.1111/j.1748-7692.1999.tb00879.x

Wells, R.S. and M.D. Scott. 1999. Bottlenose dolphin *Tursiops truncatus* (Montagu, 1821). In: Ridgway, S.H. and R. Harrison (Eds.), Handbook of marine mammals. Volume 6: The second book of dolphins and the porpoises. Academic Press, San Diego, CA. pp. 137–182.

Wells, R.S. and M.D. Scott. 2018. Bottlenose dolphin, *Tursiops truncatus*, common bottlenose dolphin. In: Würsig, B., J.G.M. Thewissen, and K.M. Kovacs (Eds.), Encyclopedia of Marine Mammals (Third Edition). Academic Press, London, UK. pp. 118–125. doi: 10.1016/B978-0-12-804327-1.00072-8

Wells, R.S., M.D. Scott, and A.B. Irvine. 1987. The social structure of free-ranging bottlenose dolphins. In: Genoways, H.H. (Ed.), Current Mammalogy. Plenum Press, New York, NY. pp. 247–305.

Wilson, R.M., R.B. Tyson, J.A. Nelson, B.C. Balmer, J.P. Chanton, and D.P. Nowacek. 2017. Niche differentiation and prey selectivity among common bottlenose dolphins (*Tursiops truncatus*) sighted in St. George Sound, Gulf of Mexico. Frontiers in Marine Science 4:235. doi: 10.3389/fmars.2017.00235

Wirth, C. and J.D. Warren. 2019. Overlapping use of an artificial reef by humans and an apex predator (*Tursiops truncatus*) in the New York Bight. Marine Mammal Science 35(1):271–283. doi: 10.1111/mms.12515

Yordy, J.E., M.A. Mollenhauer, R.M. Wilson, R.S. Wells, A. Hohn, J. Sweeney, L.H. Schwacke, T.K. Rowles, J.R. Kucklick, and M.M. Peden-Adams. 2010. Complex contaminant exposure in cetaceans: a comparative E-Screen analysis of bottlenose dolphin blubber and mixtures of four persistent organic pollutants. Environmental Toxicology and Chemistry 29(10):2143–2153. doi: 10.1002/etc.279

Common bottlenose dolphin − *Tursiops truncatus*

Western North Atlantic non-migratory coastal stocks

Overall Vulnerability Rank = High

Biological Sensitivity = Moderate

Climate Exposure = Very High

Data Quality = 96% of scores ≥ 2

	Tursiops truncatus	Expert Scores	Data Quality	Expert Scores Plots (Portion by Category)
Sensitivity attributes	Prey/Diet Specificity	1.8	2.5	
	Habitat Specificity	2.6	2.8	
	Site Fidelity	2.4	2.8	
	Lifetime Reproductive Potential	1.1	2.0	
	Generation Length	2.7	2.0	
	Reproductive Plasticity	1.4	2.0	
	Migration	3.3	2.2	
	Home Range	2.3	2.8	
	Stock Abundance	1.8	2.5	
	Stock Abundance Trend	2.5	1.8	
	Cumulative Stressors	2.3	2.8	
	Sensitivity Score	Moderate		
Exposure variables	Sea Surface Temperature (Change in mean)	4.0	3.0	
	Sea Surface Temperature (Change in variability)	1.0	3.0	
	Air Temperature (Change in mean)	4.0	3.0	
	Air Temperature (Change in variability)	1.0	3.0	
	Precipitation (Change in mean)	1.0	3.0	
	Precipitation (Change in variability)	1.2	3.0	
	Sea Surface Salinity (Change in mean)	3.7	3.0	
	Sea Surface Salinity (Change in variability)	1.0	3.0	
	Ocean pH (Change in mean)	4.0	3.0	
	Ocean pH (Change in variability)	1.1	3.0	
	Sea ice coverage (Change in mean)	1.0	3.0	
	Sea ice coverage (Change in variability)	1.0	3.0	
	Dissolved oxygen (Change in mean)	4.0	3.0	
	Dissolved oxygen (Change in variability)	1.1	3.0	
	Circulation	2.3	2.7	
	Sea level rise	2.0	3.0	
	Exposure Score	Very High		
Overall Vulnerability Rank		High		

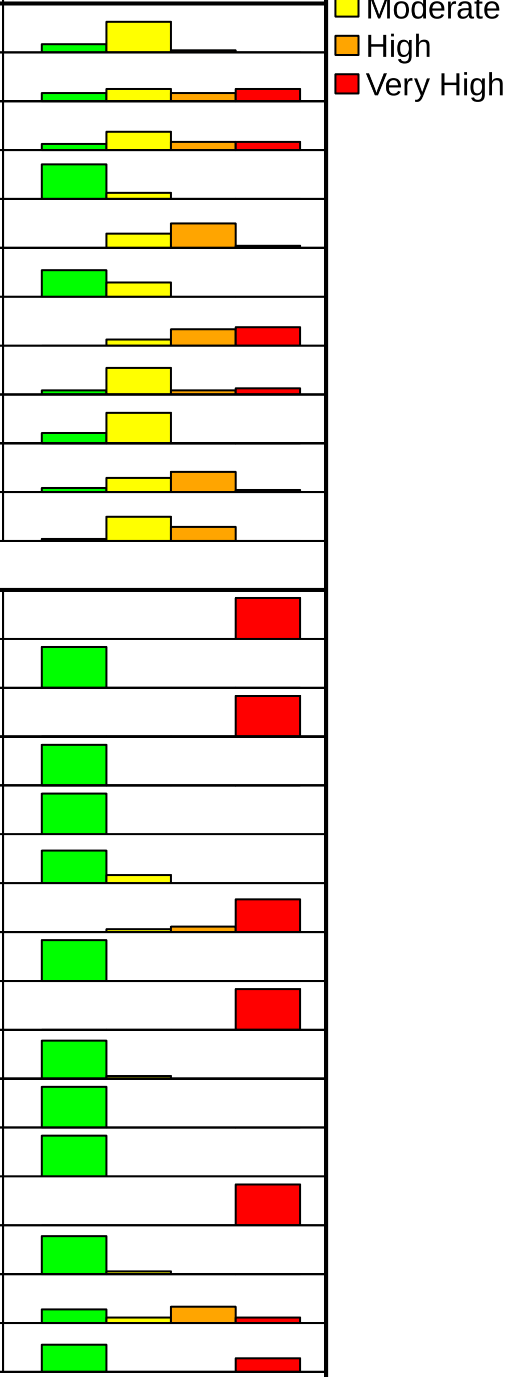

Common bottlenose dolphin (Western North Atlantic: South Carolina-Georgia, Northern Florida, and Central Florida Coastal Stocks)

Tursiops truncatus

CVA Results Summary

Overall Climate Vulnerability Rank: High (91% certainty from bootstrap analysis).

Climate Exposure: Very High. Five exposure factors scored greater than 3.5: Air Temperature (Standard anomaly) (4.00), Dissolved oxygen (Standard anomaly) (4.00), Ocean pH (Standard anomaly) (4.00), Sea Surface Temperature (Standard anomaly) (4.00), and Sea Surface Salinity (Standard anomaly) (3.73).

Biological Sensitivity: Moderate. Four sensitivity attributes scored greater than or equal to 2.5: Migration (3.30), Generation Time (2.70), Habitat Specificity (2.60), and Species Abundance Trend (2.50).

Distributional Response: Moderate

Abundance Response: Moderate

Phenology Response: Moderate

Data Quality: 96% of the data quality scores were 2 or greater, 91% of sensitivity attribute data quality scores were 2 or higher.

Background Information

Stock Description

This stock group includes the western North Atlantic common bottlenose dolphin South Carolina/Georgia Coastal Stock, Northern Florida Coastal Stock, and Central Florida Coastal Stock. This group includes dolphins of the coastal morphotype inhabiting coastal waters from the shoreline to approximately the 200m isobath from the Little River Inlet, South Carolina (33.8°N), south to the western end of Vaca Key (24.7°N, 81.1°W; Hayes et al. 2018).

Prey/Diet Specificity

Generally, common bottlenose dolphins consume a large variety of fish and/or squid (Barros and Odell 1990; Mead and Potter 1990; Wells and Scott 2018). Common bottlenose dolphins seem to show a consistent preference for sound-producing fishes (e.g., sciaenids, toadfish; Berens McCabe et al. 2010; Bowen-Stevens et al. 2021). Estuarine stocks along the southeast U.S. Atlantic coast have been reported to consume a variety of prey, with preference for sciaenids (Gannon and Waples 2004; Pate and McFee 2012). Pate and McFee (2012) identified 42 prey species from 24 families in a study of stranded dolphins along the South Carolina coast. In Sarasota Bay, Florida, Rossman et al. (2015) found some individual common bottlenose dolphins to show diet specialization despite the broader study group showing a generalist diet. Specialization can be influenced by locally abundant prey species (Shane et al. 1986; Vollmer and Rosel 2013) or can result from resource partitioning amongst sympatric populations (e.g., Torres and Read 2009; Wilson et al. 2017).

Habitat Specificity

Common bottlenose dolphins are found in a variety of marine and estuarine habitats (Wells and Scott 2018).

Site Fidelity

No specific site fidelity information was found for this stock.

Lifetime Reproductive Potential

Taylor et al. (2007) reported the interbirth interval of common bottlenose dolphins as 3.80 years, though other studies have shown it to be closer to three years (Leatherwood 1977; Wells and Scott 1999; Connor et al. 2000; Wells 2003). Thayer (2008) estimated mean interbirth interval of 2.9 years in North Carolina.

Generally, females reach sexual maturity before males, with females reaching sexual maturity at 5–13 years (Sergeant et al. 1973; Perrin and Reilly 1984; Wells et al. 1987; Mead and Potter 1990; Mann et al. 2000; Wells 2000; Wells and Scott 2018).

Females reproduce through most of their adult lives (Leatherwood 1977; Hohn et al. 1989; Wells and Scott 1999; Wells 2000; Wells 2003), but may produce fewer calves after they reach 30 years of age (Wells 2000). Sarasota Bay common bottlenose dolphins can give birth when they are as young as 6 years of age, and can continue until they are 48 years old (Wells and Scott 2018). Reproductive lifespan would therefore be up to 42 years based on long-term observation studies (Wells 2014; Wells and Scott 2018).

Longitudinal studies from Sarasota Bay, Florida have found that female bottlenose dolphins can live up to 67 years (Wells and Scott 2018). Studies based on growth layer groups in teeth suggest female bottlenose dolphins can live to 41 years along the Texas coast (Fernandez and Hohn 1998) and to 30 years along the Mississippi coast (Mattson et al. 2006).

Generation Length

Taylor et al. (2007) reported common bottlenose dolphin generation length of 20.6–21.1 years.

Reproductive Plasticity

Common bottlenose dolphin calving is generally diffusely seasonal, with one or more peaks, although births have been reported from all seasons (Vollmer and Rosel 2013; Wells and Scott 2018). Calf sightings in South Carolina peak in spring and early summer and again in early autumn (McFee et al. 2014). In North Carolina, Thayer et al. (2003) observed a strong birth peak in spring based on stranding data and a diffuse birth peak from late spring to early fall based on sightings. Calf sightings peak in the spring and summer months in Florida (Wells et al. 1987), Mississippi/Louisiana (Miller et al. 2010; Miller et al. 2013), and Texas (McHugh 1989; Henderson and Würsig 2007).

Migration

This stock group is considered non-migratory.

Home Range

Data are insufficient to characterize home range patterns (or the lack thereof) for this stock.

Stock Abundance

The abundance of the South Carolina/Georgia Coastal Stock of common bottlenose dolphins in the western North Atlantic was estimated at 6,027 individuals (CV=0.34); for the Northern Florida Coastal Stock is 877 individuals (CV=0.49); and for the Central Florida Coastal Stock is 1,218 individuals (CV=0.35) based on summer 2016 aerial surveys over coastal and shelf waters from Florida to New Jersey (Hayes et al. 2018) .

Stock Abundance Trend

Analysis of trends in abundance suggest a possible decline in the South Carolina/Georgia Coastal Stock abundance between 2002–2004 and 2016; a probable decline in Northern Florida Coastal Stock abundance between 2010–2011, and 2016; and a possible decline in Central Florida Coastal Stock abundance between 2010–2011, and 2016; however, the ability to detect trends is limited by uncertainty in stock distribution, lack of precision in abundance estimates, and gaps in surveys (Hayes et al. 2018).

Cumulative Stressors

Common bottlenose dolphins face a wide range of individual and cumulative natural and anthropogenic threats (see reviews by Vollmer and Rosel 2013; Wells and Scott 2018; and Wells et al. 2019). These include such threats as shark predation, harmful algal blooms (Fire et al. 2015; Davis et al. 2019), recreational and commercial fishing gear (Kroetz et al. 2020), vessel traffic, pollution, habitat alteration, and provisioning.

Common bottlenose dolphins in the South Carolina/Georgia Coastal Stock, Northern Florida Coastal Stock, and Central Florida Coastal Stock interact with several commercial fisheries, including the Southeastern U.S. Atlantic Shark Gillnet, Southeast Atlantic Gillnet, blue crab trap/pot, shrimp trawl, cannonball jellyfish trawl, Florida spiny lobster trap/pot, and hook and line fisheries (Hayes et al. 2018). Individuals of this stock have been reported stranded, with some strandings showing evidence of human interactions, and other strandings occurring during Unusual Mortality Events (UMEs; Hayes et al. 2018; also see NOAA National Marine Mammal Health and Stranding Response Database).

A UME was declared in the summer of 2013 for the mid-Atlantic coast from New York to Brevard County, Florida, with more than 1600 common bottlenose dolphins stranding as a result of morbillivirus (Morris et al. 2015; Balmer et al. 2018; Hayes et al. 2018; NMFS 2022). Strandings were more prevalent along oceanic shores than in estuaries, suggesting that coastal stocks were more impacted by the UME than estuarine stocks (Hayes et al. 2018).

The nearshore and estuarine habitats along the Atlantic seaboard are adjacent to areas of high human population and some are highly industrialized. Studies of bottlenose dolphin tissues from estuaries in the southeast U.S. have shown persistent organic pollutant (POP; Schwacke et al. 2002, 2012; Hansen et al. 2004; Litz et al. 2007; Pulster et al. 2009; Fair et al. 2010; Yordy et al. 2010; Balmer et al. 2011; Kucklick et al. 2011; Reif et al. 2017) and heavy metal (Durden et al. 2007; Stavros et al. 2007, 2008,

2011; Titcomb et al. 2017; Reif et al. 2017; Page-Karjian et al. 2020) concentrations that may result in adverse effects on health or reproductive rates, particularly in urbanized, developed, and agricultural areas.

Distribution and Sightings

Density model results for common bottlenose dolphins in the western North Atlantic are presented by Roberts et al. (2016a, 2016b, 2017, 2018), Mannocci et al. (2017), and Palka et al. (2021).

Further Reading

Hayes, S.A., E. Josephson, and K. Maze-Foley, P.E. Rosel. 2018. US Atlantic and Gulf of Mexico Marine Mammal Stock Assessments - 2017: (Second Edition). National Oceanic and Atmospheric Administration, National Marine Fisheries Service, Northeast Fisheries Science Center: Woods Hole, MA. NOAA Technical Memorandum NMFS-NE-245. 371 p. doi: 10.25923/e764-9g81

Wells, R.S. and M.D. Scott. 2018. Bottlenose Dolphin, *Tursiops truncatus*, Common Bottlenose Dolphin. In: Würsig, B., J.G.M. Thewissen, and K.M. Kovacs (Eds.), Encyclopedia of Marine Mammals (Third Edition). Academic Press, London, UK. pp. 118–125. doi: 10.1016/B978-0-12-804327-1.00072-8

Wells, R.S., A. Natoli, and G. Braulik. 2019. *Tursiops truncatus* (errata version published in 2019). The IUCN Red List of Threatened Species 2019: e.T22563A156932432. https://dx.doi.org/10.2305/IUCN.UK.2019-1.RLTS.T22563A156932432.en

References

Balmer, B., E. Zolman, T. Rowles, C. Smith, F. Townsend, D. Fauquier, C. George, T. Goldstein, L. Hansen, B. Quigley, W. McFee, J. Morey, P. Rosel, J. Saliki, T. Speakman, and L. Schwacke. 2018. Ranging patterns, spatial overlap, and association with dolphin morbillivirus exposure in common bottlenose dolphins (*Tursiops truncatus*) along the Georgia, USA coast. Ecology and Evolution 8(24):12890–12904. doi: 10.1002/ece3.4727

Balmer, B.C., L.H. Schwacke, R.S. Wells, R.C. George, J. Hoguet, J.R. Kucklick, S.M. Lane, A. Martinez, W.A. McLellan, P.E. Rosel, T.K. Rowles, K. Sparks, T. Speakman, E.S. Zolman, and D.A. Pabst. 2011. Relationship between persistent organic pollutants (POPs) and ranging patterns in common bottlenose dolphins (*Tursiops truncatus*) from coastal Georgia, USA. Science of the Total Environment 409(11):2094–2101. doi: 10.1016/j.scitotenv.2011.01.052

Barros, N.B. and D.K. Odell. 1990. Food habits of bottlenose dolphins in the southeastern United States In: Leatherwood, S. and R.R. Reeves (Eds.), The Bottlenose Dolphin. Academic Press, San Diego, CA. pp. 309–328. doi: 10.1016/B978-0-12-440280-5.50020-2

Berens McCabe, E.J., D.P. Gannon, N.B. Barros, and R.S. Wells. 2010. Prey selection by resident common bottlenose dolphins (*Tursiops truncatus*) in Sarasota Bay, Florida. Marine Biology 157(5):931–942. doi: 10.1007/s00227-009-1371-2

Bowen-Stevens, S.R., D.P. Gannon, R.A. Hazelkorn, G. Lovewell, K.M. Volker, S. Smith, M.C. Tumlin, and J. Litz. 2021. Diet of common bottlenose dolphins, *Tursiops truncatus*, that stranded in and near Barataria Bay, Louisiana, 2010–2012. Southeastern Naturalist 20(1). doi: 10.1656/058.020.0113

Connor, R.C., R.S. Wells, J. Mann, and A.J. Read. 2000. The bottlenose dolphin: Social relationships in a fission-fusion society. In: Mann, J., R. Connor, P. Tyack, and H. Whitehead (Eds.), Cetacean Societies: Field Studies of Dolphins and Whales. University of Chicago Press, Chicago, IL. pp. 91–126.

Davis, D.A., K. Mondo, E. Stern, A.K. Annor, S.J. Murch, T.M. Coyne, L.E. Brand, M.E. Niemeyer, S. Sharp, W.G. Bradley, P.A. Cox, and D.C. Mash. 2019. Cyanobacterial neurotoxin BMAA and brain pathology in stranded dolphins. PLoS ONE 14(3):e0213346. doi: 10.1371/journal.pone.0213346

Durden, W.N., M.K. Stolen, D.H. Adams, and E.D. Stolen. 2007. Mercury and selenium concentrations in stranded bottlenose dolphins from the Indian River Lagoon system, Florida. Bulletin of Marine Science 81(1):37–54.

Fair, P.A., J. Adams, G. Mitchum, T.C. Hulsey, J.S. Reif, M. Houde, D. Muir, E. Wirth, D. Wetzel, E. Zolman, W. McFee, and G.D. Bossart. 2010. Contaminant blubber burdens in Atlantic bottlenose dolphins (*Tursiops truncatus*) from two southeastern US estuarine areas: Concentrations and patterns of PCBs, pesticides, PBDEs, PFCs, and PAHs. Science of the Total Environment 408(7):1577–1597. doi: 10.1016/j.scitotenv.2009.12.021

Fernandez, S. and A.A. Hohn. 1998. Age, growth, and calving season of bottlenose dolphins, *Tursiops truncatus*, off coastal Texas. Fishery Bulletin 96(2):357–365.

Fire, S.E., L.J. Flewelling, M. Stolen, W. Noke Durden, M. de Wit, A.C. Spellman, and Z. Wang. 2015. Brevetoxin-associated mass mortality event of bottlenose dolphins and manatees along the east coast of Florida, USA. Marine Ecology Progress Series 526:241–251. doi: 10.3354/meps11225

Gannon, D.P. and D.M. Waples. 2004. Diets of coastal bottlenose dolphins from the U. S. Mid-Atlantic coast differ by habitat. Marine Mammal Science 20(3):527–545. doi: 10.1111/j.1748-7692.2004.tb01177.x

Hansen, L.J., L.H. Schwacke, G.B. Mitchum, A.A. Hohn, R.S. Wells, E.S. Zolman, and P.A. Fair. 2004. Geographic variation in polychorinated biphenyl and organochlorine pesticide concentrations in the blubber of bottlenose dolphins from the US Atlantic coast. Science of the Total Environment 319(1-3):147–172. doi: 10.1016/S0048-9697(03)00371-1

Hayes, S.A., E. Josephson, K. Maze-Foley, and P.E. Rosel. 2018. US Atlantic and Gulf of Mexico marine mammal stock assessments - 2017: (Second Edition). National Oceanic and Atmospheric Administration, National Marine Fisheries Service, Northeast Fisheries Science Center: Woods Hole, MA. NOAA Technical Memorandum NMFS-NE-245. 371 p. doi: 10.25923/e764-9g81

Henderson, E.E. and B. Würsig. 2007. Behavior patterns of bottlenose dolphins in San Luis Pass, Texas. Gulf of Mexico Science 25(2):153–161. doi: 10.18785/goms.2502.06

Hohn, A.A., M.D. Scott, R.S. Wells, J.C. Sweeney, and A.B. Irvine. 1989. Growth layers in teeth from known-age, free-ranging bottlenose dolphins. Marine Mammal Science 5(4):315–342. doi: 10.1111/j.1748-7692.1989.tb00346.x

Kroetz, A.M., A.N. Mathers, and J.K. Carlson. 2020. Evaluating protected species bycatch in the U.S. Southeast Gillnet Fishery. Fisheries Research 228:105573. doi: 10.1016/j.fishres.2020.105573

Kucklick, J., L. Schwacke, R. Wells, A. Hohn, A. Guichard, J. Yordy, L. Hansen, E. Zolman, R. Wilson, J. Litz, D. Nowacek, T. Rowles, R. Pugh, B. Balmer, C. Sinclair, and P. Rosel. 2011. Bottlenose dolphins as indicators of persistent organic pollutants in the western North Atlantic Ocean and Northern Gulf of Mexico. Environmental Science & Technology 45(10):4270–4277. doi: 10.1021/es1042244

Leatherwood, S. 1977. Some preliminary impressions on the numbers and social behavior of free-swimming bottlenosed dolphin calves (*Tursiops truncatus*) in the northern Gulf of Mexico. In: Ridgway, S.H. and K. Bernirschke (Eds.), Breeding Dolphins; Present Status, Suggestions for the Future. Final report to the US Marine Mammal Commission, Contract MM6AC009, MMC-76/07, Washington, DC. pp. 143–167.

Litz, J.A. 2007. Social structure, genetic structure, and persistent organohalogen pollutants in bottlenose dolphins (*Tursiops truncatus*) in Biscayne Bay, Florida. University of Miami. 140 p.

Mann, J., R.C. Connor, L.M. Barre, and M.R. Heithaus. 2000. Female reproductive success in bottlenose dolphins (*Tursiops* sp.): life history, habitat, provisioning, and group-size effects. Behavioral Ecology 11(2):210–219. doi: 10.1093/beheco/11.2.210

Mannocci, L., J.J. Roberts, D.L. Miller, and P.N. Halpin. 2017. Extrapolating cetacean densities to quantitatively assess human impacts on populations in the high seas. Conservation Biology 31(3):601–614. doi: 10.1111/cobi.12856

Mattson, M.C., K.D. Mullin, G.W. Ingram, and W. Hoggard. 2006. Age structure and growth of the bottlenose dolphin (*Tursiops truncatus*) from strandings in the Mississippi sound region of the north-central Gulf of Mexico from 1986 to 2003. Marine Mammal Science 22(3):654–666. doi: 10.1111/j.1748-7692.2006.00057.x

McFee, W.E., T.R. Speakman, L. Balthis, J.D. Adams, and E.S. Zolman. 2014. Reproductive seasonality of a recently designated bottlenose dolphin stock near Charleston, South Carolina, U.S.A. Marine Mammal Science 30(2):528–543. doi: 10.1111/mms.12055

McHugh, M.B. 1989. Population numbers and feeding behavior of the Atlantic bottlenose dolphin (*Tursiops truncatus*) near Aransas Pass, Texas. University of Texas at Austin. Thesis. 96 p.

Mead, J.G. and C.W. Potter. 1990. Natural history of bottlenose dolphins along the central Atlantic coast of the United States. In: Leatherwood, S. and R.R. Reeves (Eds.), The Bottlenose Dolphin. Academic Press, San Diego, CA. pp. 165–195.

Miller, L.J., A.D. Mackey, T. Hoffland, M. Solangi, and S.A. Kuczaj. 2010. Potential effects of a major hurricane on Atlantic bottlenose dolphin (*Tursiops truncatus*) reproduction in the Mississippi Sound. Marine Mammal Science 26(3):707–715. doi: 10.1111/j.1748-7692.2010.00371.x

Miller, L.J., A.D. Mackey, M. Solangi, and S.A. Kuczaj. 2013. Population abundance and habitat utilization of bottlenose dolphins in the Mississippi Sound. Aquatic Conservation: Marine and Freshwater Ecosystems 23(1):145–151. doi: 10.1002/aqc.2278

Morris, S.E., J.L. Zelner, D.A. Fauquier, T.K. Rowles, P.E. Rosel, F. Gulland, and B.T. Grenfell. 2015. Partially observed epidemics in wildlife hosts: modelling an outbreak of dolphin morbillivirus in the northwestern Atlantic, June 2013-2014. Journal of the Royal Society Interface 12(112):20150676. doi: 10.1098/rsif.2015.0676

National Marine Fisheries Service (NMFS). 2022. Active and closed Unusual Mortality Events. https://www.fisheries.noaa.gov/national/marine-life-distress/active-and-closed-unusual-mortality-events

Palka, D., L. Aichinger Dias, E. Broughton, S. Chavez-Rosales, D. Cholewiak, G. Davis, A. DeAngelis, L. Garrison, H. Haas, J. Hatch, K. Hyde, M. Jech, E. Josephson, L. Mueller-Brennan, C. Orphanides, N. Pegg, C. Sasso, D. Sigourney, M. Soldevilla, and H. Walsh. 2021. Atlantic Marine Assessment Program for Protected Species: FY15 – FY19. US Department of the Interior, Bureau of Ocean Energy Management: Washington, DC. OCS Study BOEM 2021-051. 330 p.

Pate, S.M. and W.E. McFee. 2012. Prey species of bottlenose dolphins (*Tursiops truncatus*) from South Carolina Waters. Southeastern Naturalist 11(1):1–22. doi: 10.1656/058.011.0101

Perrin, W.F. and S.B. Reilly. 1984. Reproductive parameters of dolphins and small whales of the family Delphinidae. In: Perrin, W.F., R.L. Brownell, Jr., and D.P. DeMaster (Eds.), Reproduction in Whales, Dolphins, and Porpoises. pp. 97–134.

Pulster, E.L., K.L. Smalling, E. Zolman, L. Schwacke, and K.A. Maruya. 2009. Persistent organochlorine pollutants and toxaphene congener profiles in bottlenose dolphins (*Tursiops truncatus*) frequenting the Turtle/Brunswick River Estuary, Georgia, USA. Environmental Toxicology and Chemistry 28(7):1390–1399. doi: 10.1897/08-240.1

Reif, J.S., A.M. Schaefer, G.D. Bossart, and P.A. Fair. 2017. Health and Environmental Risk Assessment Project for bottlenose dolphins *Tursiops truncatus* from the southeastern USA. II. Environmental aspects. Diseases of Aquatic Organisms 125(2):155–166. doi: 10.3354/dao03143

Roberts, J.J., B.D. Best, L. Mannocci, E. Fujioka, P.N. Halpin, D.L. Palka, L.P. Garrison, K.D. Mullin, T.V.N. Cole, C.B. Khan, W.A. McLellan, D.A. Pabst, and G.G. Lockhart. 2016a. Habitat-based cetacean density models for the US Atlantic and Gulf of Mexico. Scientific Reports 6:22615. doi: 10.1038/Srep22615

Roberts, J.J., L. Mannocci, and P.N. Halpin. 2016b. Final project report: Marine species density data gap assessments and update for the AFTT Study Area, 2015-2016 (Base Year). Document version 1.0. Report prepared for Naval Facilities Engineering Command, Atlantic by the Duke University Marine Geospatial Ecology Lab: Durham, NC. 21 p.

Roberts, J.J., L. Mannocci, and P.N. Halpin. 2017. Final Project Report: Marine Species Density Data Gap Assessments and Update for the AFTT Study Area, 2016-2017 (Opt. Year 1). Document version 1.4. Report prepared for Naval Facilities Engineering Command, Atlantic by the Duke University Marine Geospatial Ecology Lab: Durham, NC. 76 p.

Roberts, J.J., L. Mannocci, R.S. Schick, and P.N. Halpin. 2018. Final Project Report: Marine Species Density Data Gap Assessments and Update for the AFTT Study Area, 2017-2018 (Opt. Year 2). Document version 1.2. Report prepared for Naval Facilities Engineering Command, Atlantic by the Duke University Marine Geospatial Ecology Lab: Durham, NC. 113 p.

Rossman, S., P.H. Ostrom, M. Stolen, N.B. Barros, H. Gandhi, C.A. Stricker, and R.S. Wells. 2015. Individual specialization in the foraging habits of female bottlenose dolphins living in a trophically diverse and habitat rich estuary. Oecologia 178(2):415–425. doi: 10.1007/s00442-015-3241-6

Schwacke, L.H., E.O. Voit, L.J. Hansen, R.S. Wells, G.B. Mitchum, A.A. Hohn, and P.A. Fair. 2002. Probabilistic risk assessment of reproductive effects of polychlorinated biphenyls on bottlenose dolphins (*Tursiops truncatus*) from the southeast United States coast. Environmental Toxicology and Chemistry 21(12):2752–2764. doi: 10.1002/etc.5620211232

Schwacke, L.H., E.S. Zolman, B.C. Balmer, S. De Guise, R.C. George, J. Hoguet, A.A. Hohn, J.R. Kucklick, S. Lamb, M. Levin, J.A. Litz, W.E. McFee, N.J. Place, F.I. Townsend, R.S. Wells, and T.K. Rowles. 2012. Anaemia, hypothyroidism and immune suppression associated with polychlorinated biphenyl exposure in bottlenose dolphins (*Tursiops truncatus*). Proceedings of the Royal Society B: Biological Sciences 279(1726):48–57. doi: 10.1098/rspb.2011.0665

Sergeant, D.E., D.K. Caldwell, and M.C. Caldwell. 1973. Age, growth, and maturity of bottlenosed dolphin (*Tursiops-truncatus*) from northeast Florida. Journal of the Fisheries Research Board of Canada 30(7):1009–1011. doi: 10.1139/f73-165

Shane, S.H., R.S. Wells, and B. Würsig. 1986. Ecology, behavior and social organization of the bottlenose dolphin: A review. Marine Mammal Science 2:34–63. doi: 10.1111/j.1748-7692.1986.tb00026.x

Stavros, H.-C.W., G.D. Bossart, T.C. Hulsey, and P.A. Fair. 2007. Trace element concentrations in skin of free-ranging bottlenose dolphins (*Tursiops truncatus*) from the southeast Atlantic coast. Science of the Total Environment 388(1-3):300–315. doi: 10.1016/j.scitotenv.2007.07.030

Stavros, H.-C.W., G.D. Bossart, T.C. Hulsey, and P.A. Fair. 2008. Trace element concentrations in blood of free-ranging bottlenose dolphins (*Tursiops truncatus*): Influence of age, sex and location. Marine Pollution Bulletin 56(2):371–379. doi: 10.1016/j.marpolbul.2007.10.030

Stavros, H.-C.W., M. Stolen, W.N. Durden, W. McFee, G.D. Bossart, and P.A. Fair. 2011. Correlation and toxicological inference of trace elements in tissues from stranded and free-ranging bottlenose dolphins (*Tursiops truncatus*). Chemosphere 82(11):1649–1661. doi: 10.1016/j.chemosphere.2010.11.019

Taylor, B.L., S.J. Chivers, J. Larese, and W.F. Perrin. 2007. Generation length and percent mature estimates for IUCN assessments of cetaceans. National Oceanic and Atmospheric Administration, National Marine Fisheries Service, Southwest Fisheries Science Center: La Jolla, CA. Administrative Report LJ-07-01. 24 p.

Thayer, V.G. 2008. Life history parameters and social associations of female bottlenose dolphins (*Tursiops truncatus*) off North Carolina, USA. Duke University. Dissertation. 164 p.

Thayer, V.G., A.J. Read, A.S. Friedlaender, D.R. Colby, A.A. Hohn, W.A. McLellan, D.A. Pabst, J.L. Dearolf, N.I. Bowles, J.R. Russell, and K.A. Rittmaster. 2003. Reproductive seasonality of western Atlantic bottlenose dolphins off North Carolina, USA. Marine Mammal Science 19(4):617–629. doi: 10.1111/j.1748-7692.2003.tb01120.x

Titcomb, E.M., J.S. Reif, P.A. Fair, H.-C.W. Stavros, M. Mazzoil, G.D. Bossart, and A.M. Schaefer. 2017. Blood mercury concentrations in common bottlenose dolphins from the Indian River Lagoon, Florida: Patterns of social distribution. Marine Mammal Science 33(3):771–784. doi: 10.1111/mms.12390

Torres, L.G. and A.J. Read. 2009. Where to catch a fish? The influence of foraging tactics on the ecology of bottlenose dolphins (*Tursiops truncatus*) in Florida Bay, Florida. Marine Mammal Science 25(4):797–815. doi: 10.1111/j.1748-7692.2009.00297.x

Vollmer, N.L. and P.E. Rosel. 2013. A review of common bottlenose dolphins (*Tursiops truncatus truncatus*) in the Northern Gulf of Mexico: Population biology, potential threats, and management. Southeastern Naturalist 12(m6):1–43. doi: 10.1656/058.012.m601

Wells, R.S. 2000. Reproduction in wild bottlenose dolphins: Overview of patterns observed during a long-term study. In: Duffield, D. and T. Robeck (Eds.), Bottlenose Dolphin Reproduction Workshop. AZA Marine Mammal Taxon Advisory Group, Silver Spring, MD. pp. 57–74.

Wells, R.S. 2003. Dolphin social complexity: Lessons from long-term study and life history. In: de Waal, F.B.M. and P.L. Tyack (Eds.), Animal Social Complexity: Intelligence, Culture, and Individualized Societies Harvard University Press, Cambridge, MA. pp. 32–56.

Wells, R.S. 2014. Social structure and life history of bottlenose dolphins near Sarasota Bay, Florida: Insights from four decades and five generations. Primates and cetaceans. pp. 149–172. doi: 10.1007/978-4-431-54523-1_8

Wells, R.S., A. Natoli, and G. Braulik. 2019. *Tursiops truncatus* (errata version published in 2019). *The IUCN Red List of Threatened Species* 2019: e.T22563A156932432. https://dx.doi.org/10.2305/IUCN.UK.2019-1.RLTS.T22563A156932432.en

Wells, R.S. and M.D. Scott. 1999. Bottlenose dolphin *Tursiops truncatus* (Montagu, 1821). In: Ridgway, S.H. and R. Harrison (Eds.), Handbook of marine mammals. Volume 6: The second book of dolphins and the porpoises. Academic Press, San Diego, CA. pp. 137–182.

Wells, R.S. and M.D. Scott. 2018. Bottlenose dolphin, *Tursiops truncatus*, common bottlenose dolphin. In: Würsig, B., J.G.M. Thewissen, and K.M. Kovacs (Eds.), Encyclopedia of Marine Mammals (Third Edition). Academic Press, London, UK. pp. 118–125. doi: 10.1016/B978-0-12-804327-1.00072-8

Wells, R.S., M.D. Scott, and A.B. Irvine. 1987. The social structure of free-ranging bottlenose dolphins. In: Genoways, H.H. (Ed.), Current Mammalogy. Plenum Press, New York, NY. pp. 247–305.

Wilson, R.M., R.B. Tyson, J.A. Nelson, B.C. Balmer, J.P. Chanton, and D.P. Nowacek. 2017. Niche differentiation and prey selectivity among common bottlenose dolphins (*Tursiops truncatus*) sighted in St. George Sound, Gulf of Mexico. Frontiers in Marine Science 4:235. doi: 10.3389/fmars.2017.00235

Yordy, J.E., M.A. Mollenhauer, R.M. Wilson, R.S. Wells, A. Hohn, J. Sweeney, L.H. Schwacke, T.K. Rowles, J.R. Kucklick, and M.M. Peden-Adams. 2010. Complex contaminant exposure in cetaceans: a comparative E-Screen analysis of bottlenose dolphin blubber and mixtures of four persistent organic pollutants. Environmental Toxicology and Chemistry 29(10):2143–2153. doi: 10.1002/etc.279

Common bottlenose dolphin − *Tursiops truncatus*

Western North Atlantic, offshore Stock

Overall Vulnerability Rank = Low

Biological Sensitivity = Low

Climate Exposure = High

Data Quality = 93% of scores ≥ 2

	Tursiops truncatus	Expert Scores	Data Quality	Expert Scores Plots (Portion by Category)
Sensitivity attributes	Prey/Diet Specificity	1.9	2.7	
	Habitat Specificity	1.8	3.0	
	Site Fidelity	1.0	2.7	
	Lifetime Reproductive Potential	1.4	2.3	
	Generation Length	2.5	1.7	
	Reproductive Plasticity	1.4	2.3	
	Migration	1.7	2.7	
	Home Range	2.1	2.7	
	Stock Abundance	1.3	2.7	
	Stock Abundance Trend	1.9	1.3	
	Cumulative Stressors	1.6	2.7	
	Sensitivity Score	Low		
Exposure variables	Sea Surface Temperature (Change in mean)	3.8	3.0	
	Sea Surface Temperature (Change in variability)	1.3	3.0	
	Air Temperature (Change in mean)	3.0	2.3	
	Air Temperature (Change in variability)	1.0	2.3	
	Precipitation (Change in mean)	1.0	3.0	
	Precipitation (Change in variability)	1.2	3.0	
	Sea Surface Salinity (Change in mean)	2.3	3.0	
	Sea Surface Salinity (Change in variability)	1.7	3.0	
	Ocean pH (Change in mean)	3.3	3.0	
	Ocean pH (Change in variability)	1.1	3.0	
	Sea ice coverage (Change in mean)	1.1	3.0	
	Sea ice coverage (Change in variability)	1.0	3.0	
	Dissolved oxygen (Change in mean)	3.0	3.0	
	Dissolved oxygen (Change in variability)	1.1	3.0	
	Circulation	1.3	2.7	
	Sea level rise	1.1	3.0	
	Exposure Score	High		
Overall Vulnerability Rank		Low		

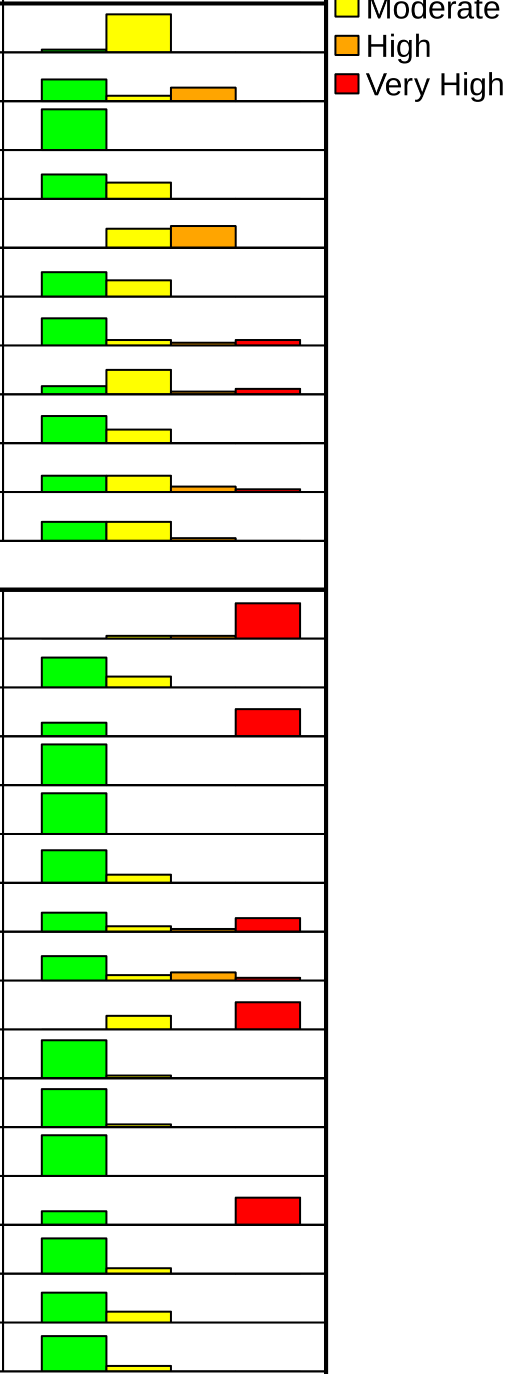

Common bottlenose dolphin (Western North Atlantic, Offshore Stock)

Tursiops truncatus

CVA Results Summary

Overall Climate Vulnerability Rank: Low (92% certainty from bootstrap analysis).

Climate Exposure: High. Four exposure factors scored greater than or equal to 3.0: Sea Surface Temperature (Standard anomaly) (3.80), Ocean pH (Standard anomaly) (3.33), Air Temperature (Standard anomaly) (3.00), and Dissolved oxygen (Standard anomaly) (3.00).

Biological Sensitivity: Low. Only one sensitivity attribute scored greater than 2.50: Generation Time (2.53).

Distributional Response: High

Abundance Response: Low

Phenology Response: High

Data Quality: 93% of the data quality scores were 2 or greater, 82% of sensitivity attribute data quality scores were 2 or higher.

Background Information

Stock Description

This stock includes common bottlenose dolphins of the oceanic morphotype, generally found seaward from the 25 m isobath in the U.S. Exclusive Economic Zone and adjacent offshore and Canadian waters of the western North Atlantic (Hayes et al. 2020).

Prey/Diet Specificity

Generally, common bottlenose dolphins consume a large variety of fish and/or squid (Barros and Odell 1990; Mead and Potter 1990; Wells and Scott 2018). Common bottlenose dolphins seem to show a consistent preference for sound-producing fishes (e.g., sciaenids, toadfish; Berens McCabe et al. 2010; Bowen-Stevens et al. 2021). Estuarine stocks along the southeast U.S. Atlantic coast have been reported to consume a variety of prey, with preference for sciaenids (Gannon and Waples 2004; Pate and McFee 2012). Pate and McFee (2012) identified 42 prey species from 24 families in a study of stranded dolphins along the South Carolina coast. In Sarasota Bay, Florida, Rossman et al. (2015) found some individual common bottlenose dolphins to show diet specialization despite the broader study group showing a generalist diet. Specialization can be influenced by locally abundant prey species (Shane et al. 1986; Vollmer and Rosel 2013) or can result from resource partitioning amongst sympatric populations (e.g., Torres and Read 2009; Wilson et al. 2017).

Habitat Specificity

Common bottlenose dolphins are found in a variety of marine and estuarine habitats (Wells and Scott 2018).

Site Fidelity

No specific site fidelity information was found for this stock.

Lifetime Reproductive Potential

Taylor et al. (2007) reported the interbirth interval of common bottlenose dolphins as 3.80 years, though other studies have shown it to be closer to three years (Leatherwood 1977; Wells and Scott 1999; Connor et al. 2000; Wells 2003). Thayer (2008) estimated mean interbirth interval of 2.9 years in North Carolina.

Generally, females reach sexual maturity before males, with females reaching sexual maturity at 5–13 years (Sergeant et al. 1973; Perrin and Reilly 1984; Wells et al. 1987; Mead and Potter 1990; Mann et al. 2000; Wells 2000; Wells and Scott 2018).

Females reproduce through most of their adult lives (Leatherwood 1977; Hohn et al. 1989; Wells and Scott 1999; Wells 2000; Wells 2003), but may produce fewer calves after they reach 30 years of age (Wells 2000). Sarasota Bay common bottlenose dolphins can give birth when they are as young as 6 years of age, and can continue until they are 48 years old (Wells and Scott 2018). Reproductive lifespan would therefore be up to 42 years based on long-term observation studies (Wells 2014; Wells and Scott 2018).

Longitudinal studies from Sarasota Bay, Florida have found that female bottlenose dolphins can live up to 67 years (Wells and Scott 2018). Studies based on growth layer groups in teeth suggest female bottlenose dolphins can live to 41 years along the Texas coast (Fernandez and Hohn 1998) and to 30 years along the Mississippi coast (Mattson et al. 2006).

Generation Length

Taylor et al. (2007) reported common bottlenose dolphin generation length of 20.6–21.1 years.

Reproductive Plasticity

Common bottlenose dolphin calving is generally diffusely seasonal, with one or more peaks, although births have been reported from all seasons (Vollmer and Rosel 2013; Wells and Scott 2018). Calf sightings in South Carolina peak in spring and early summer and again in early autumn (McFee et al. 2014). In North Carolina, Thayer et al. (2003) observed a strong birth peak in spring based on stranding data and a diffuse birth peak from late spring to early fall based on sightings. Calf sightings peak in the spring and summer months in Florida (Wells et al. 1987), Mississippi/Louisiana (Miller et al. 2010; Miller et al. 2013), and Texas (McHugh 1989; Henderson and Würsig 2007).

Migration

Studies relating to the migratory patterns (or the lack thereof) for this stock were not found in the literature. The nearby coastal migratory stocks display seasonal north-south movements. During winter months, the Northern Migratory Coastal Stock moves from waters as far north as New Jersey to waters near Cape Hatteras (Kenney 1990; Read et al. 2003; Torres et al. 2005). During January and February, the Southern Migratory Coastal Stock migrates south from waters of southern Virginia and north central North Carolina to waters south of Cape Fear, North Carolina, and as far south as coastal Florida (Barco et al. 1999).

Home Range

Data are insufficient to characterize home range patterns (or the lack thereof) for this stock. Tracking studies have shown common bottlenose dolphins can travel distances greater than 2000km (Wells et al. 1999). Estrada and Hohn (2003) observed migrating bottlenose dolphins along the East Coast of the United States travel a mean distance of 32.7 km/day while non-migrating dolphins traveled a mean distance of 24.5 km/day. Klatsky et al. (2007) found dolphins off Bermuda traveled a mean distance of 28.3 km/day.

Stock Abundance

The best abundance estimate for this stock is 62,851 individuals (CV=0.23) based on summer 2016 surveys (Garrison 2020; Palka 2020).

Stock Abundance Trend

An analysis of population estimates from 2004 (54,739; CV=0.24), 2011 (77,532; CV=0.40), and 2016 (62,851; CV=0.23) did not indicate a statistically significant trend in abundance (Hayes et al. 2020).

Cumulative Stressors

Common bottlenose dolphins face a wide range of individual and cumulative natural and anthropogenic threats (see reviews by Vollmer and Rosel 2013; Wells and Scott 2018; and Wells et al. 2019). These include such threats as shark predation, harmful algal blooms, recreational and commercial fishing gear, vessel traffic, pollution, habitat alteration, and provisioning.

Common bottlenose dolphins in the Western North Atlantic Offshore Stock interact with the bottom trawl fishery, with mean annual human-caused mortality and serious injury of 10.42 (CV=0.62) dolphins in the Northeast and 12.08 (CV=0.39) dolphins in the Mid-Atlantic during the period 2014–2018 (Lyssikatos et al. 2020).

An Unusual Mortality Event (UME) was declared in the summer of 2013 for the mid-Atlantic coast from New York to Brevard County, Florida, with more than 1500 common bottlenose dolphins stranding as a result of morbillivirus (Waring et al. 2016; NMFS 2022). Strandings were more prevalent along oceanic shores than in estuaries, suggesting that coastal stocks were more impacted by the UME than estuarine stocks (Waring et al. 2016).

Storm events have been shown to affect common bottlenose dolphin distribution and foraging (Fandel et al. 2020).

Distribution and Sightings

Density model results for common bottlenose dolphins in the western North Atlantic are presented by Roberts et al. (2016a, 2016b, 2017, 2018), Mannocci et al. (2017), and Palka et al. (2021).

Further Reading

Hayes, S.A., E. Josephson, K. Maze-Foley, and P.E. Rosel. 2020. US Atlantic and Gulf of Mexico Marine Mammal Stock Assessments - 2019. National Oceanic and Atmospheric Administration, National Marine Fisheries Service, Northeast Fisheries Science Center: Woods Hole, MA. NOAA Technical Memorandum NMFS-NE-264. 479 p.

Wells, R.S. and M.D. Scott. 2018. Bottlenose Dolphin, *Tursiops truncatus*, Common Bottlenose Dolphin. In: Würsig, B., J.G.M. Thewissen, and K.M. Kovacs (Eds.), Encyclopedia of Marine Mammals (Third Edition). Academic Press, London, UK. pp. 118–125. doi: 10.1016/B978-0-12-804327-1.00072-8

Wells, R.S., A. Natoli, and G. Braulik. 2019. *Tursiops truncatus* (errata version published in 2019). The IUCN Red List of Threatened Species 2019: e.T22563A156932432. https://dx.doi.org/10.2305/IUCN.UK.2019-1.RLTS.T22563A156932432.en

References

Barco, S.G., W.M. Swingle, W.A. Mclellan, R.N. Harris, and D.A. Pabst. 1999. Local abundance and distribution of bottlenose dolphins (*Tursiops truncatus*) in the nearshore waters of Virginia Beach, Virginia. Marine Mammal Science 15(2):394–408. doi: 10.1111/j.1748-7692.1999.tb00809.x

Barros, N.B. and D.K. Odell. 1990. Food habits of bottlenose dolphins in the southeastern United States In: Leatherwood, S. and R.R. Reeves (Eds.), The Bottlenose Dolphin. Academic Press, San Diego, CA. pp. 309–328. doi: 10.1016/B978-0-12-440280-5.50020-2

Berens McCabe, E.J., D.P. Gannon, N.B. Barros, and R.S. Wells. 2010. Prey selection by resident common bottlenose dolphins (*Tursiops truncatus*) in Sarasota Bay, Florida. Marine Biology 157(5):931–942. doi: 10.1007/s00227-009-1371-2

Bowen-Stevens, S.R., D.P. Gannon, R.A. Hazelkorn, G. Lovewell, K.M. Volker, S. Smith, M.C. Tumlin, and J. Litz. 2021. Diet of common bottlenose dolphins, *Tursiops truncatus*, that stranded in and near Barataria Bay, Louisiana, 2010–2012. Southeastern Naturalist 20(1). doi: 10.1656/058.020.0113

Connor, R.C., R.S. Wells, J. Mann, and A.J. Read. 2000. The bottlenose dolphin: Social relationships in a fission-fusion society. In: Mann, J., R. Connor, P. Tyack, and H. Whitehead (Eds.), Cetacean Societies: Field Studies of Dolphins and Whales. University of Chicago Press, Chicago, IL. pp. 91–126.

Durden, W.N., E.D. Stolen, and M.K. Stolen. 2011. Abundance, distribution, and group composition of Indian River Lagoon bottlenose dolphins (*Tursiops truncatus*). Aquatic Mammals 37(2):175–186. doi: 10.1578/Am.37.2.2011.175

Estrada, E.L. and A.A. Hohn. 2003. Satellite monitored movements of bottlenose dolphins along the Atlantic coast of the US. 15th Biennial Conference on the Biology of Marine Mammals, Greensboro, NC, 14-19 December 2003

Fair, P.A., J. Adams, G. Mitchum, T.C. Hulsey, J.S. Reif, M. Houde, D. Muir, E. Wirth, D. Wetzel, E. Zolman, W. McFee, and G.D. Bossart. 2010. Contaminant blubber burdens in Atlantic bottlenose dolphins (*Tursiops truncatus*) from two southeastern US estuarine areas: Concentrations and patterns of PCBs, pesticides, PBDEs, PFCs, and PAHs. Science of the Total Environment 408(7):1577–1597. doi: 10.1016/j.scitotenv.2009.12.021

Fandel, A.D., A. Garrod, A.L. Hoover, J.E. Wingfield, V. Lyubchich, D.H. Secor, K.B. Hodge, A.N. Rice, and H. Bailey. 2020. Effects of intense storm events on dolphin occurrence and foraging behavior. Scientific Reports 10(1):19247. doi: 10.1038/s41598-020-76077-3

Fernandez, S. and A.A. Hohn. 1998. Age, growth, and calving season of bottlenose dolphins, *Tursiops truncatus*, off coastal Texas. Fishery Bulletin 96(2):357–365.

Gannon, D.P. and D.M. Waples. 2004. Diets of coastal bottlenose dolphins from the U. S. Mid-Atlantic coast differ by habitat. Marine Mammal Science 20(3):527–545. doi: 10.1111/j.1748-7692.2004.tb01177.x

Garrison, L.P. 2020. Abundance of marine mammals in waters of the U.S. southeastern Atlantic during summer 2016. National Oceanic and Atmospheric Administration, National Marine Fisheries Service, Southeast Fisheries Science Center, Protected Resources and Biodiversity Division: Miami, FL. PRBD-2020-04. 17 p.

Hansen, L.J., L.H. Schwacke, G.B. Mitchum, A.A. Hohn, R.S. Wells, E.S. Zolman, and P.A. Fair. 2004. Geographic variation in polychorinated biphenyl and organochlorine pesticide concentrations in the blubber of bottlenose dolphins from the US Atlantic coast. Science of the Total Environment 319(1-3):147–172. doi: 10.1016/S0048-9697(03)00371-1

Hayes, S.A., E. Josephson, K. Maze-Foley, and P.E. Rosel. 2020. US Atlantic and Gulf of Mexico marine mammal stock assessments - 2019. National Oceanic and Atmospheric Administration, National Marine Fisheries Service, Northeast Fisheries Science Center: Woods Hole, MA. NOAA Technical Memorandum NMFS-NE-264. 479 p.

Henderson, E.E. and B. Würsig. 2007. Behavior patterns of bottlenose dolphins in San Luis Pass, Texas. Gulf of Mexico Science 25(2):153–161. doi: 10.18785/goms.2502.06

Hohn, A.A., M.D. Scott, R.S. Wells, J.C. Sweeney, and A.B. Irvine. 1989. Growth layers in teeth from known-age, free-ranging bottlenose dolphins. Marine Mammal Science 5(4):315–342. doi: 10.1111/j.1748-7692.1989.tb00346.x

Kenney, R.D. 1990. Bottlenose dolphins off the northeastern United States. In: Leatherwood, S. and R.R. Reeves (Eds.), The Bottlenose Dolphin. Academic Press, San Diego, CA. pp. 369–386.

Klatsky, L.J., R.S. Wells, and J.C. Sweeney. 2007. Offshore bottlenose dolphins (*Tursiops truncatus*): movement and dive behavior near the Bermuda Pedestal. Journal of Mammalogy 88(1):59–66. doi: 10.1644/05-mamm-a-365r1.1

Leatherwood, S. 1977. Some preliminary impressions on the numbers and social behavior of free-swimming bottlenosed dolphin calves (*Tursiops truncatus*) in the northern Gulf of Mexico. In: Ridgway, S.H. and K. Bernirschke (Eds.), Breeding Dolphins; Present Status, Suggestions for the Future. Final report to the US Marine Mammal Commission, Contract MM6AC009, MMC-76/07, Washington, DC. pp. 143–167.

Lyssikatos, M.C., S. Chavez-Rosales, and J. Hatch. 2021. Estimates of cetacean and pinniped bycatch in Northeast and Mid-Atlantic bottom trawl fisheries, 2014-2018. National Oceanic and Atmospheric Administration, National Marine Fisheries Service, Northeast Fisheries Science Center: Woods Hole, MA. Northeast Fisheries Science Center Reference Document 21-02. 12 p.

Mann, J., R.C. Connor, L.M. Barre, and M.R. Heithaus. 2000. Female reproductive success in bottlenose dolphins (*Tursiops* sp.): life history, habitat, provisioning, and group-size effects. Behavioral Ecology 11(2):210–219. doi: 10.1093/beheco/11.2.210

Mannocci, L., J.J. Roberts, D.L. Miller, and P.N. Halpin. 2017. Extrapolating cetacean densities to quantitatively assess human impacts on populations in the high seas. Conservation Biology 31(3):601–614. doi: 10.1111/cobi.12856

Mattson, M.C., K.D. Mullin, G.W. Ingram, and W. Hoggard. 2006. Age structure and growth of the bottlenose dolphin (*Tursiops truncatus*) from strandings in the Mississippi sound region of the north-central Gulf of Mexico from 1986 to 2003. Marine Mammal Science 22(3):654–666. doi: 10.1111/j.1748-7692.2006.00057.x

McFee, W.E., T.R. Speakman, L. Balthis, J.D. Adams, and E.S. Zolman. 2014. Reproductive seasonality of a recently designated bottlenose dolphin stock near Charleston, South Carolina, U.S.A. Marine Mammal Science 30(2):528–543. doi: 10.1111/mms.12055

McHugh, M.B. 1989. Population numbers and feeding behavior of the Atlantic bottlenose dolphin (*Tursiops truncatus*) near Aransas Pass, Texas. University of Texas at Austin. Thesis. 96 p.

Mead, J.G. and C.W. Potter. 1990. Natural history of bottlenose dolphins along the central Atlantic coast of the United States. In: Leatherwood, S. and R.R. Reeves (Eds.), The Bottlenose Dolphin. Academic Press, San Diego, CA. pp. 165–195.

Miller, L.J., A.D. Mackey, T. Hoffland, M. Solangi, and S.A. Kuczaj. 2010. Potential effects of a major hurricane on Atlantic bottlenose dolphin (*Tursiops truncatus*) reproduction in the Mississippi Sound. Marine Mammal Science 26(3):707–715. doi: 10.1111/j.1748-7692.2010.00371.x

Miller, L.J., A.D. Mackey, M. Solangi, and S.A. Kuczaj. 2013. Population abundance and habitat utilization of bottlenose dolphins in the Mississippi Sound. Aquatic Conservation: Marine and Freshwater Ecosystems 23(1):145–151. doi: 10.1002/aqc.2278

Palka, D. 2020. Cetacean abundance estimates in US northwestern Atlantic Ocean waters from summer 2016 line transect surveys conducted by the Northeast Fisheries Science Center. National Oceanic and Atmospheric Administration, National Marine Fisheries Service, Northeast Fisheries Science Center: Woods Hole, MA. Northeast Fisheries Science Center Reference Document 20-05. 60 p.

Palka, D., L. Aichinger Dias, E. Broughton, S. Chavez-Rosales, D. Cholewiak, G. Davis, A. DeAngelis, L. Garrison, H. Haas, J. Hatch, K. Hyde, M. Jech, E. Josephson, L. Mueller-Brennan, C. Orphanides, N. Pegg, C. Sasso, D. Sigourney, M. Soldevilla, and H. Walsh. 2021. Atlantic Marine Assessment Program for Protected Species: FY15 – FY19. US Department of the Interior, Bureau of Ocean Energy Management: Washington, DC. OCS Study BOEM 2021-051. 330 p.

Pate, S.M. and W.E. McFee. 2012. Prey species of bottlenose dolphins (*Tursiops truncatus*) from South Carolina Waters. Southeastern Naturalist 11(1):1–22. doi: 10.1656/058.011.0101

Perrin, W.F. and S.B. Reilly. 1984. Reproductive parameters of dolphins and small whales of the family Delphinidae. In: Perrin, W.F., R.L. Brownell, Jr., and D.P. DeMaster (Eds.), Reproduction in Whales, Dolphins, and Porpoises. pp. 97–134.

Read, A.J., K.W. Urian, B. Wilson, and D.M. Waples. 2003. Abundance of bottlenose dolphins in the bays, sounds, and estuaries of North Carolina. Marine Mammal Science 19(1):59–73. doi: 10.1111/j.1748-7692.2003.tb01092.x

Roberts, J.J., B.D. Best, L. Mannocci, E. Fujioka, P.N. Halpin, D.L. Palka, L.P. Garrison, K.D. Mullin, T.V.N. Cole, C.B. Khan, W.A. McLellan, D.A. Pabst, and G.G. Lockhart. 2016a. Habitat-based cetacean density models for the US Atlantic and Gulf of Mexico. Scientific Reports 6:22615. doi: 10.1038/Srep22615

Roberts, J.J., L. Mannocci, and P.N. Halpin. 2016b. Final project report: Marine species density data gap assessments and update for the AFTT Study Area, 2015-2016 (Base Year). Document version 1.0. Report prepared for Naval Facilities Engineering Command, Atlantic by the Duke University Marine Geospatial Ecology Lab: Durham, NC. 21 p.

Roberts, J.J., L. Mannocci, and P.N. Halpin. 2017. Final Project Report: Marine Species Density Data Gap Assessments and Update for the AFTT Study Area, 2016-2017 (Opt. Year 1). Document version 1.4. Report prepared for Naval Facilities Engineering Command, Atlantic by the Duke University Marine Geospatial Ecology Lab: Durham, NC. 76 p.

Roberts, J.J., L. Mannocci, R.S. Schick, and P.N. Halpin. 2018. Final Project Report: Marine Species Density Data Gap Assessments and Update for the AFTT Study Area, 2017-2018 (Opt. Year 2). Document version 1.2. Report prepared for Naval Facilities Engineering Command, Atlantic by the Duke University Marine Geospatial Ecology Lab: Durham, NC. 113 p.

Rossman, S., P.H. Ostrom, M. Stolen, N.B. Barros, H. Gandhi, C.A. Stricker, and R.S. Wells. 2015. Individual specialization in the foraging habits of female bottlenose dolphins living in a trophically diverse and habitat rich estuary. Oecologia 178(2):415–425. doi: 10.1007/s00442-015-3241-6

Sergeant, D.E., D.K. Caldwell, and M.C. Caldwell. 1973. Age, growth, and maturity of bottlenosed dolphin (*Tursiops-truncatus*) from northeast Florida. Journal of the Fisheries Research Board of Canada 30(7):1009–1011. doi: 10.1139/f73-165

Shane, S.H., R.S. Wells, and B. Würsig. 1986. Ecology, behavior and social organization of the bottlenose dolphin: A review. Marine Mammal Science 2:34–63. doi: 10.1111/j.1748-7692.1986.tb00026.x

Taylor, B.L., S.J. Chivers, J. Larese, and W.F. Perrin. 2007. Generation length and percent mature estimates for IUCN assessments of cetaceans. National Oceanic and Atmospheric Administration, National Marine Fisheries Service, Southwest Fisheries Science Center: La Jolla, CA. Administrative Report LJ-07-01. 24 p.

Thayer, V.G. 2008. Life history parameters and social associations of female bottlenose dolphins (*Tursiops truncatus*) off North Carolina, USA. Duke University. Dissertation. 164 p.

Thayer, V.G., A.J. Read, A.S. Friedlaender, D.R. Colby, A.A. Hohn, W.A. McLellan, D.A. Pabst, J.L. Dearolf, N.I. Bowles, J.R. Russell, and K.A. Rittmaster. 2003. Reproductive seasonality of western Atlantic bottlenose dolphins off North Carolina, USA. Marine Mammal Science 19(4):617–629. doi: 10.1111/j.1748-7692.2003.tb01120.x

Torres, L.G. and A.J. Read. 2009. Where to catch a fish? The influence of foraging tactics on the ecology of bottlenose dolphins (*Tursiops truncatus*) in Florida Bay, Florida. Marine Mammal Science 25(4):797–815. doi: 10.1111/j.1748-7692.2009.00297.x

Torres, L.G. and D.L. Urban. 2005. Using spatial analysis to assess bottlenose dolphins as an indicator of healthy fish habitat. In: Bortone, S.A. (Ed.), Estuarine indicators. CRC Press, Boca Raton, FL. pp. 423–436.

Vollmer, N.L. and P.E. Rosel. 2013. A review of common bottlenose dolphins (*Tursiops truncatus truncatus*) in the Northern Gulf of Mexico: Population biology, potential threats, and management. Southeastern Naturalist 12(m6):1–43. doi: 10.1656/058.012.m601

Waring, G.T., E. Josephson, K. Maze-Foley, and P.E. Rosel. 2016. U.S. Atlantic and Gulf of Mexico marine mammal stock assessments - 2015. National Oceanic and Atmospheric Administration, National Marine Fisheries Service, Northeast Fisheries Science Center: Woods Hole, MA. NOAA Technical Memorandum NMFS-NE-238. 501 p. doi: 10.7289/V57S7KTN

Wells, R.S. 2000. Reproduction in wild bottlenose dolphins: Overview of patterns observed during a long-term study. In: Duffield, D. and T. Robeck (Eds.), Bottlenose Dolphin Reproduction Workshop. AZA Marine Mammal Taxon Advisory Group, Silver Spring, MD. pp. 57–74.

Wells, R.S. 2003. Dolphin social complexity: Lessons from long-term study and life history. In: de Waal, F.B.M. and P.L. Tyack (Eds.), Animal Social Complexity: Intelligence, Culture, and Individualized Societies Harvard University Press, Cambridge, MA. pp. 32–56.

Wells, R.S. 2014. Social structure and life history of bottlenose dolphins near Sarasota Bay, Florida: Insights from four decades and five generations. Primates and cetaceans. pp. 149–172. doi: 10.1007/978-4-431-54523-1_8

Wells, R.S., A. Natoli, and G. Braulik. 2019. *Tursiops truncatus* (errata version published in 2019). *The IUCN Red List of Threatened Species* 2019: e.T22563A156932432. https://dx.doi.org/10.2305/IUCN.UK.2019-1.RLTS.T22563A156932432.en

Wells, R.S., H.L. Rhinehart, P. Cunningham, J. Whaley, M. Baran, C. Koberna, and D.P. Costa. 1999. Long distance offshore movements of bottlenose dolphins. Marine Mammal Science 15(4):1098–1114. doi: 10.1111/j.1748-7692.1999.tb00879.x

Wells, R.S. and M.D. Scott. 1999. Bottlenose dolphin *Tursiops truncatus* (Montagu, 1821). In: Ridgway, S.H. and R. Harrison (Eds.), Handbook of marine mammals. Volume 6: The second book of dolphins and the porpoises. Academic Press, San Diego, CA. pp. 137–182.

Wells, R.S. and M.D. Scott. 2018. Bottlenose dolphin, *Tursiops truncatus*, common bottlenose dolphin. In: Würsig, B., J.G.M. Thewissen, and K.M. Kovacs (Eds.), Encyclopedia of Marine Mammals (Third Edition). Academic Press, London, UK. pp. 118–125. doi: 10.1016/B978-0-12-804327-1.00072-8

Wells, R.S., M.D. Scott, and A.B. Irvine. 1987. The social structure of free-ranging bottlenose dolphins. In: Genoways, H.H. (Ed.), Current Mammalogy. Plenum Press, New York, NY. pp. 247–305.

Wilson, R.M., R.B. Tyson, J.A. Nelson, B.C. Balmer, J.P. Chanton, and D.P. Nowacek. 2017. Niche differentiation and prey selectivity among common bottlenose dolphins (*Tursiops truncatus*) sighted in St. George Sound, Gulf of Mexico. Frontiers in Marine Science 4:235. doi: 10.3389/fmars.2017.00235

Common dolphin − *Delphinus delphis delphis*

Western North Atlantic Stock

Overall Vulnerability Rank = Low

Biological Sensitivity = Low

Climate Exposure = High

Data Quality = 85% of scores ≥ 2

	Delphinus delphis delphis	Expert Scores	Data Quality	Expert Scores Plots (Portion by Category)
Sensitivity attributes	Prey/Diet Specificity	1.5	2.8	
	Habitat Specificity	1.8	3.0	
	Site Fidelity	1.8	1.8	
	Lifetime Reproductive Potential	1.6	2.5	
	Generation Length	1.8	1.8	
	Reproductive Plasticity	1.6	1.8	
	Migration	2.4	2.8	
	Home Range	1.8	3.0	
	Stock Abundance	1.1	3.0	
	Stock Abundance Trend	2.0	0.8	
	Cumulative Stressors	1.8	2.5	
	Sensitivity Score	Low		
Exposure variables	Sea Surface Temperature (Change in mean)	3.5	3.0	
	Sea Surface Temperature (Change in variability)	1.2	3.0	
	Air Temperature (Change in mean)	3.1	2.5	
	Air Temperature (Change in variability)	1.1	2.5	
	Precipitation (Change in mean)	1.1	3.0	
	Precipitation (Change in variability)	1.3	3.0	
	Sea Surface Salinity (Change in mean)	2.4	2.5	
	Sea Surface Salinity (Change in variability)	2.1	2.5	
	Ocean pH (Change in mean)	3.4	3.0	
	Ocean pH (Change in variability)	1.4	3.0	
	Sea ice coverage (Change in mean)	1.1	2.5	
	Sea ice coverage (Change in variability)	1.0	2.5	
	Dissolved oxygen (Change in mean)	2.5	3.0	
	Dissolved oxygen (Change in variability)	1.2	3.0	
	Circulation	2.0	2.2	
	Sea level rise	1.4	2.2	
	Exposure Score	High		
Overall Vulnerability Rank		Low		

Low
Moderate
High
Very High

Common dolphin, short-beaked (Western North Atlantic Stock)

Delphinus delphis delphis

CVA Results Summary

Overall Climate Vulnerability Rank: Low (98% certainty from bootstrap analysis).

Climate Exposure: High. Three exposure factors scored greater than 3.0: Sea Surface Temperature (Standard anomaly) (3.50), Ocean pH (Standard anomaly) (3.35), and Air Temperature (Standard anomaly) (3.15).

Biological Sensitivity: Low. No sensitivity attributes scored greater than 2.5.

Distributional Response: High

Abundance Response: Low

Phenology Response: Moderate

Data Quality: 85 % of the data quality scores were 2 or greater. 64% of sensitivity attribute data quality scores were 2 or higher.

Background Information

Stock Description

The short-beaked common dolphin western North Atlantic stock includes individuals that reside in or visit waters of the U.S. Exclusive Economic Zone in the western North Atlantic and adjacent offshore and Canadian waters (Hayes et al. 2022).

Prey/Diet Specificity

Common dolphins consume a variety of epipelagic and mesopelagic fishes and squids associated with the deep scattering layer and are known to adapt their diet based on prey abundance (Young and Cockcroft 1994; Evans 1994; Ohizumi et al. 1998; Goold 2000; De Pierrepont et al. 2005; Pusineri et al. 2007). In the western North Atlantic, long-finned squid (*Loligo pealei*) and Atlantic mackerel (*Scomber scombrus*) are frequent prey species while herring, whiting (*Micromesistius poutassou*), pilchard, and anchovy are also part of the diet (Waring et al. 1990; Overholtz and Waring 1991; Lahaye et al. 2005).

Habitat Specificity

In the western North Atlantic, short-beaked common dolphins are found in continental shelf waters, over prominent underwater topography, and in association with Gulf Stream features (Hui 1979; CETAP 1982; Selzer and Payne 1988; Waring et al. 1992; Evans 1994; Hamazaki 2002; Doksaeter et al. 2008; Waring et al. 2008).

Site Fidelity

Short-beaked common dolphins are generally considered to have low site fidelity; however a study from the coast of Portugal suggested some degree of site fidelity (Ball et al. 2017).

Lifetime Reproductive Potential

Calving intervals of 1-3 years have been reported for this species (Jefferson et al. 2015). Taylor et al. (2007) reported an interbirth interval of 2.10 years based on values reported by Danil and Chivers (2007).

Reported sexual maturity ages vary greatly across populations (Jefferson et al. 2015). For short-beaked common dolphins in the western North Atlantic, Westgate and Read (2007) reported 8.3 and 9.5 years as the age of female and male sexual maturity, respectively.

Generation Length

Taylor et al. (2007) reported a generation length of 14.1 years at r = 0.02 and 14.8 years at r = 0.0 based on values reported by Perrin and Reilly (1984), Ferrero and Walker (1995), and Danil and Chivers (2007).

Reproductive Plasticity

In the western North Atlantic, mating occurs primarily during July and August and gestation is estimated at 11-12 months (Westgate and Read 2007). Calving peaks during mid-summer (Westgate and Read 2007).

Migration

Although short-beaked common dolphins can be found from Cape Hatteras to Nova Scotia year-round (CETAP 1982), they do exhibit seasonal movements. From mid-January to May, they are most common Cape Hatteras to Georges Bank. They are known to move onto Georges Bank, Gulf of Maine, and the Scotian Shelf from mid-summer to autumn (Sergeant et al. 1970; Hain et al. 1981; CETAP 1982; Payne et al. 1984; Selzer and Payne 1988; Gowans and Whitehead 1995).

Home Range

In the western North Atlantic, short-beaked common dolphins are known to range from Newfoundland to Florida. They are more common in temperate, cooler waters of the northwestern Atlantic (Gaskin 1992; Waring and Palka 2002; Jefferson et al. 2009; Perrin 2018) and are not currently thought to occur south of South Carolina with any regularity (Jefferson et al. 2009).

Stock Abundance

Recent abundance estimates have been generated for portions of this stock based on 2016 surveys: 48,723 (CV=0.48) for the Newfoundland/Labrador portion, 43,124 (CV=0.28) for the Bay of Fundy/Scotian Shelf/Gulf of St. Lawrence portion, and 80,227 (CV=0.31) and 900 (CV=0.57) for U.S. waters of the western North Atlantic (Lawson and Gosselin 2018; Garrison 2020; Palka 2020). The entire

stock estimate is 172,974 individuals (CV=0.21) and the minimum population estimate for this stock is 145,216 (Hayes et al. 2022).

Stock Abundance Trend

A trend analysis has not been conducted for this stock.

Cumulative Stressors

Short-beaked common dolphins interact with the Northeast sink gillnet, Mid-Atlantic gillnet, Northeast bottom trawl, Mid-Atlantic bottom trawl, and pelagic longline fisheries. During 2014–2018, total annual estimated average fishery-related mortality or serious injury to this stock was 399 individuals (CV=0.05), and 499 strandings were reported along the U.S. Atlantic coast (Garrison and Stokes 2016, 2017, 2019, 2020a, 2020b; Lyssikatos et al. 2020; Hayes et al. 2022.

Distribution and Sightings

Density model results for sho dolphin in the western North Atlantic are presented by Roberts et al. (2016a, 2016b, 2017, 2018), Mannocci et al. (2017), Chavez-Rosales et al. (2019), and Palka et al. (2021a, 2021b).

Further Reading

Department of the Navy. 2008. Marine Resources Assessment Update for the Virginia Capes Operating Area. Department of the Navy, U.S. Fleet Forces Command, Norfolk, VA. Contract #N62470-D-9997, CTO 0056. Prepared by Geo-Marine, Inc., Hampton, VA. 711 p.

Hammond, P.S., G. Bearzi, A. Bjørge, K. Forney, L. Karczmarski, T. Kasuya, W.F. Perrin, M.D. Scott, J.Y. Wang, R.S. Wells, and B. Wilson. 2008. *Delphinus delphis*. The IUCN Red List of Threatened Species 2008: e.T6336A12649851. https://dx.doi.org/10.2305/IUCN.UK.2008.RLTS.T6336A12649851.en

Hayes, S.A., E. Josephson, K. Maze-Foley, P.E. Rosel, and J. Wallace. 2022. U.S. Atlantic and Gulf of Mexico marine mammal stock assessments 2021. National Oceanic and Atmospheric Administration, National Marine Fisheries Service, Northeast Fisheries Science Center: Woods Hole, MA. NOAA Technical Memorandum NMFS-NE-288. 380 p. doi: 10.25923/6tt7-kc16

Jefferson, T.A., M.A. Webber, and R.L. Pitman. 2015. Marine Mammals of the World (Second Edition). 616 p. doi: 10.1016/c2012-0-06919-0

Perrin, W.F. 2018. Common Dolphin: *Delphinus delphis*. In: Würsig, B., J.G.M. Thewissen, and K.M. Kovacs (Eds.), Encyclopedia of Marine Mammals (Third Edition). Academic Press, London, UK. pp. 205–209. doi: 10.1016/B978-0-12-804327-1.00095-9

References

Ball, L., K. Shreves, M. Pilot, and A.E. Moura. 2017. Temporal and geographic patterns of kinship structure in common dolphins (*Delphinus delphis*) suggest site fidelity and female-biased long-distance dispersal. Behavioral Ecology and Sociobiology 71(8):123. doi: 10.1007/s00265-017-2351-z

Cetacean and Turtle Assessment Program (CETAP). 1982. A characterization of marine mammals and turtles in the Mid- and North Atlantic areas of the U.S. outer continental shelf, final report. Contract AA551-CT8-48. Bureau of Land Management: Washington, DC. 538 p.

Chavez-Rosales, S., D.L. Palka, L.P. Garrison, and E.A. Josephson. 2019. Environmental predictors of habitat suitability and occurrence of cetaceans in the western North Atlantic Ocean. Scientific Reports 9(1):5833. doi: 10.1038/s41598-019-42288-6

Danil, K. and S.J. Chivers. 2007. Growth and reproduction of female short-beaked common dolphins, *Delphinus delphis*, in the eastern tropical Pacific. Canadian Journal of Zoology 85:108–121. doi: 10.1139/z06-188

De Pierrepont, J.F., B. Dubois, S. Desormonts, M.B. Santos, and J.P. Robin. 2005. Stomach contents of English Channel cetaceans stranded on the coast of Normandy. Journal of the Marine Biological Association of the United Kingdom 85(6):1539–1546. doi: 10.1017/s0025315405012762

Doksaeter, L., E. Olsen, L. Nottestad, and A. Ferno. 2008. Distribution and feeding ecology of dolphins along the Mid-Atlantic Ridge between Iceland and the Azores. Deep-Sea Research Part Ii-Topical Studies in Oceanography 55(1-2):243–253. doi: 10.1016/j.dsr2.2007.09.009

Evans, W.E. 1994. Common dolphin, white-bellied porpoise *Delphinus delphis* Linnaeus, 1758. In: Ridgway, S.H. and R. Harrison (Eds.), Handbook of marine mammals. Volume 5: The first book of dolphins. Academic Press, San Diego, CA. pp. 191–224.

Ferrero, R.C. and W.A. Walker. 1995. Growth and reproduction of the common dolphin, Delphinus delphis Linnaeus, in the offshore waters of the north Pacific Ocean. Fishery Bulletin 93:483–494.

Garrison, L.P. 2020. Abundance of marine mammals in waters of the U.S. southeastern Atlantic during summer 2016. National Oceanic and Atmospheric Administration, National Marine Fisheries Service, Southeast Fisheries Science Center, Protected Resources and Biodiversity Division: Miami, FL. PRBD-2020-04. 17 p.

Garrison, L.P. and L. Stokes. 2016. Estimated bycatch of marine mammals and sea turtles in the U.S. Atlantic pelagic longline fleet during 2014. National Oceanic and Atmospheric Administration, National Marine Fisheries Service, Southeast Fisheries Science Center: Miami, FL. NOAA Technical Memorandum NMFS-SEFSC-696. 62 p. doi: 10.7289/V5/TM-SEFSC-696

Garrison, L.P. and L. Stokes. 2017. Estimated bycatch of marine mammals and sea turtles in the U.S. Atlantic pelagic longline fleet during 2015. National Oceanic and Atmospheric Administration, National Marine Fisheries Service, Southeast Fisheries Science Center: Miami, FL. NOAA Technical Memorandum NMFS-SEFSC-709. 61 p. doi: 10.25923/kaf6-5c33

Garrison, L.P. and L. Stokes. 2019. Estimated bycatch of marine mammals and sea turtles in the U.S. Atlantic pelagic longline fleet during 2016. National Oceanic and Atmospheric Administration, National Marine Fisheries Service, Southeast Fisheries Science Center: Miami, FL. Southeast Fisheries Science Center Reference Document PRBD-2019-01. 43 p. doi: 10.25923/d893-d142

Garrison, L.P. and L. Stokes. 2020a. Estimated bycatch of marine mammals and sea turtles in the U.S. Atlantic pelagic longline fleet during 2017. National Oceanic and Atmospheric Administration, National Marine Fisheries Service, Southeast Fisheries Science Center: Miami, FL. Southeast Fisheries Science Center Reference Document PRBD 2020-05. 58 p. doi: 10.25923/a3hs-ea60

Garrison, L.P. and L. Stokes. 2020b. Estimated bycatch of marine mammals and sea turtles in the U.S. Atlantic pelagic longline fleet during 2018. National Oceanic and Atmospheric Administration, National Marine Fisheries Service, Southeast Fisheries Science Center: Miami, FL. Southeast Fisheries Science Center Reference Document PRBD 2020-08. 44 p. doi: 10.25923/777n-w316

Gaskin, D.E. 1992. Status of the common dolphin, *Delphinus delphis*, in Canada. Canadian Field-Naturalist 106(1):55–63.

Goold, J.C. 2000. A diel pattern in vocal activity of short-beaked common dolphins, *Delphinus delphis*. Marine Mammal Science 16(1):240–244. doi: 10.1111/j.1748-7692.2000.tb00915.x

Gowans, S. and H. Whitehead. 1995. Distribution and habitat partitioning by small odontocetes in the Gully, a submarine-canyon on the Scotian-Shelf. Canadian Journal of Zoology 73(9):1599–1608. doi: 10.1139/z95-190

Hain, J.H.W., R.K. Edel, H.E. Hays, S.K. Katona, and J.D. Roanowicz. 1981. General distribution of cetaceans in the continental shelf waters of the northeastern United States. A characterization of marine mammals and turtles in the mid- and north Atlantic areas of the US outer continental shelf. Annual report for 1979. Bureau of Land Management, Washington, DC. pp. II.1–II.345.

Hamazaki, T. 2002. Spatiotemporal prediction models of cetacean habitats in the mid-western North Atlantic Ocean (from Cape Hatteras, North Carolina, USA to Nova Scotia, Canada). Marine Mammal Science 18(4):920–939. doi: 10.1111/j.1748-7692.2002.tb01082.x

Hayes, S.A., E. Josephson, K. Maze-Foley, P.E. Rosel, and J. Wallace. 2022. U.S. Atlantic and Gulf of Mexico marine mammal stock assessments 2021. National Oceanic and Atmospheric Administration, National Marine Fisheries Service, Northeast Fisheries Science Center: Woods Hole, MA. NOAA Technical Memorandum NMFS-NE-288. 380 p. doi: 10.25923/6tt7-kc16

Hui, C.A. 1979. Undersea topography and distribution of dolphins of the genus *Delphinus* in the Southern-California Bight. Journal of Mammalogy 60(3):521–527. doi: 10.2307/1380092

Jefferson, T.A., D. Fertl, J. Bolaños-Jiménez, and A.N. Zerbini. 2009. Distribution of common dolphins (*Delphinus* spp.) in the western Atlantic Ocean: a critical re-examination. Marine Biology 156(6):1109–1124. doi: 10.1007/s00227-009-1152-y

Jefferson, T.A., M.A. Webber, and R.L. Pitman. 2015. Marine mammals of the world (second edition). Academic Press, San Diego, CA. 616 p. doi: 10.1016/c2012-0-06919-0

Lahaye, V., P. Bustamante, J. Spitz, W. Dabin, K. Das, G.J. Pierce, and F. Caurant. 2005. Long-term dietary segregation of common dolphins *Delphinus delphis* in the Bay of Biscay, determined using cadmium as an ecological tracer. Marine Ecology Progress Series 305:275–285. doi: 10.3354/meps305275

Lawson, J. and J.-F. Gosselin. 2018. Estimates of cetacean abundance from the 2016 NAISS aerial surveys of eastern Canadian waters, with a comparison to estimates from the 2007 TNASS. NAMMCO SC/25/AE/09. 40 p.

Lyssikatos, M.C., S. Chavez-Rosales, and J.M. Hatch. 2020. Estimates of cetacean and pinniped bycatch in Northeast and Mid-Atlantic bottom trawl fisheries, 2013-2017. National Oceanic and Atmospheric Administration, National Marine Fisheries Service, Northeast Fisheries Science Center: Woods Hole, MA. Northeast Fisheries Science Center Reference Document 20-04. 11 p. doi: 10.25923/5we2-g460

Mannocci, L., J.J. Roberts, D.L. Miller, and P.N. Halpin. 2017. Extrapolating cetacean densities to quantitatively assess human impacts on populations in the high seas. Conservation Biology 31(3):601–614. doi: 10.1111/cobi.12856

Ohizumi, H., M. Yoshioka, K. Mori, and N. Miyazaki. 1998. Stomach contents of common dolphins (*Delphinus delphis*) in the pelagic western North Pacific. Marine Mammal Science 14(4):835–844. doi: 10.1111/j.1748-7692.1998.tb00767.x

Overholtz, W.J. and G.T. Waring. 1991. Diet composition of pilot whales *Globicephala sp* and common dolphins *Delphinus-delphis* in the Mid-Atlantic Bight during Spring 1989. Fishery Bulletin 89(4):723–728.

Palka, D. 2020. Cetacean abundance estimates in US northwestern Atlantic Ocean waters from summer 2016 line transect surveys conducted by the Northeast Fisheries Science Center. National Oceanic and Atmospheric Administration, National Marine Fisheries Service, Northeast Fisheries Science Center: Woods Hole, MA. Northeast Fisheries Science Center Reference Document 20-05. 60 p.

Palka, D., L. Aichinger Dias, E. Broughton, S. Chavez-Rosales, D. Cholewiak, G. Davis, A. DeAngelis, L. Garrison, H. Haas, J. Hatch, K. Hyde, M. Jech, E. Josephson, L. Mueller-Brennan, C. Orphanides, N. Pegg, C. Sasso, D. Sigourney, M. Soldevilla, and H. Walsh. 2021a. Atlantic Marine Assessment Program for Protected Species: FY15 – FY19. US Department of the Interior, Bureau of Ocean Energy Management: Washington, DC. OCS Study BOEM 2021-051. 330 p.

Palka, D., L. Aichinger Dias, E. Broughton, S. Chavez-Rosales, D. Cholewiak, G. Davis, A. DeAngelis, L. Garrison, H. Haas, J. Hatch, K. Hyde, M. Jech, E. Josephson, L. Mueller-Brennan, C. Orphanides, N. Pegg, C. Sasso, D. Sigourney, M. Soldevilla, and H. Walsh. 2021b. Supplement to final report BOEM 2021-051 Atlantic Marine Assessment Program for Protected Species: FY15 – FY19 Appendix I generalize additive density-habitat models and maps, by species. US Department of the Interior, Bureau of Ocean Energy Management: Washington, DC. OCS Study BOEM 2021-051. 330 p.

Payne, P.M., L.A. Selzer, and A.R. Knowlton. 1984. Distribution and density of cetaceans, marine turtles, and seabirds in the shelf waters of the northeastern United States, June 1980 - December 1983, based on shipboard observations. National Oceanic and Atmospheric Administration, National Marine Fisheries Service, Northeast Fisheries Science Center: Woods Hole, MA. Contract number NA-81-FA-C-00023. 246 p.

Perrin, W.F. 2018. Common dolphin: *Delphinus delphis*. In: Würsig, B., J.G.M. Thewissen, and K.M. Kovacs (Eds.), Encyclopedia of Marine Mammals (Third Edition). Academic Press, London, UK. pp. 205–209. doi: 10.1016/B978-0-12-804327-1.00095-9

Perrin, W.F. and S.B. Reilly. 1984. Reproductive parameters of dolphins and small whales of the family Delphinidae. In: Perrin, W.F., R.L. Brownell, Jr., and D.P. DeMaster (Eds.), Reproduction in Whales, Dolphins, and Porpoises. pp. 97–134.

Pusineri, C., V. Magnin, L. Meynier, J. Spitz, S. Hassani, and V. Ridoux. 2007. Food and feeding ecology of the common dolphin (*Delphinus delphis*) in the oceanic Northeast Atlantic and comparison with its diet in neritic areas. Marine Mammal Science 23(1):30–47. doi: 10.1111/j.1748-7692.2006.00088.x

Roberts, J.J., B.D. Best, L. Mannocci, E. Fujioka, P.N. Halpin, D.L. Palka, L.P. Garrison, K.D. Mullin, T.V.N. Cole, C.B. Khan, W.A. McLellan, D.A. Pabst, and G.G. Lockhart. 2016a. Habitat-based cetacean density models for the US Atlantic and Gulf of Mexico. Scientific Reports 6:22615. doi: 10.1038/Srep22615

Roberts, J.J., L. Mannocci, and P.N. Halpin. 2016b. Final project report: Marine species density data gap assessments and update for the AFTT Study Area, 2015-2016 (Base Year). Document version 1.0. Report prepared for Naval Facilities Engineering Command, Atlantic by the Duke University Marine Geospatial Ecology Lab: Durham, NC. 21 p.

Roberts, J.J., L. Mannocci, and P.N. Halpin. 2017. Final Project Report: Marine Species Density Data Gap Assessments and Update for the AFTT Study Area, 2016-2017 (Opt. Year 1). Document version 1.4. Report prepared for Naval Facilities Engineering Command, Atlantic by the Duke University Marine Geospatial Ecology Lab: Durham, NC. 76 p.

Roberts, J.J., L. Mannocci, R.S. Schick, and P.N. Halpin. 2018. Final Project Report: Marine Species Density Data Gap Assessments and Update for the AFTT Study Area, 2017-2018 (Opt. Year 2). Document version 1.2. Report prepared for Naval Facilities Engineering Command, Atlantic by the Duke University Marine Geospatial Ecology Lab: Durham, NC. 113 p.

Selzer, L.A. and P.M. Payne. 1988. The distribution of white-sided (*Lagenorhynchus-acutus*) and common dolphins (*Delphinus-delphis*) vs environmental features of the continental-shelf of the northeastern United-States. Marine Mammal Science 4(2):141–153. doi: 10.1111/j.1748-7692.1988.tb00194.x

Sergeant, D.E., A.W. Mansfield, and B. Beck. 1970. Inshore records of cetacea for eastern Canada, 1949-68. Journal of the Fisheries Research Board of Canada 27(11):1903–&. doi: 10.1139/f70-216

Taylor, B.L., S.J. Chivers, J. Larese, and W.F. Perrin. 2007. Generation length and percent mature estimates for IUCN assessments of cetaceans. National Oceanic and Atmospheric Administration, National Marine Fisheries Service, Southwest Fisheries Science Center: La Jolla, CA. Administrative Report LJ-07-01. 24 p.

Waring, G.T., C.P. Fairfield, C.M. Ruhsam, and M. Sano. 1992. Cetaceans associated with Gulf Stream Features off the Northeastern USA Shelf. International Council for the Exploration of the Sea: Copenhagen, Denmark. CM 1992/N:12. 28 p.

Waring, G.T., P. Gerrior, P.M. Payne, B.L. Parry, and J.R. Nicolas. 1990. Incidental take of marine mammals in foreign fishery activities off the Northeast United-States, 1977-88. Fishery Bulletin 88(2):347–360.

Waring, G.T., L. Nøttestad, E. Olsen, H. Skov, and G. Vikingsson. 2008. Distribution and density estimates of cetaceans along the mid-Atlantic Ridge during summer 2004. Journal of Cetacean Research and Management 10(2):137–146.

Waring, G.T. and D.L. Palka. 2002. North Atlantic marine mammals. In: Perrin, W.F., B. Würsig, and J.G.M. Thewissen (Eds.), Encyclopedia of Marine Mammals. Academic Press., San Diego, CA. pp. 802–806.

Westgate, A.J. and A.J. Read. 2007. Reproduction in short-beaked common dolphins (*Delphinus delphis*) from the western North Atlantic. Marine Biology 150(5):1011–1024. doi: 10.1007/s00227-006-0394-1

Young, D.D. and V.G. Cockcroft. 1994. Diet of common dolphins (*Delphinus delphis*) off the south-east coast of southern Africa: opportunism or specialization? Journal of Zoology 234(1):41–53. doi: 10.1111/j.1469-7998.1994.tb06055.x

Cuvier's beaked whale − *Ziphius cavirostris*

Gulf of Mexico Stock

Overall Vulnerability Rank = Very High

Biological Sensitivity = High

Climate Exposure = Very High

Data Quality = 70% of scores ≥ 2

	Ziphius cavirostris	Expert Scores	Data Quality	Expert Scores Plots (Portion by Category)
Sensitivity attributes	Prey/Diet Specificity	2.6	2.0	
	Habitat Specificity	2.4	2.3	
	Site Fidelity	2.7	1.3	
	Lifetime Reproductive Potential	3.2	1.3	
	Generation Length	2.7	1.3	
	Reproductive Plasticity	2.0	1.0	
	Migration	3.1	1.7	
	Home Range	2.7	1.7	
	Stock Abundance	2.9	1.7	
	Stock Abundance Trend	2.7	0.7	
	Cumulative Stressors	2.9	2.0	
	Sensitivity Score	High		
Exposure variables	Sea Surface Temperature (Change in mean)	3.5	2.7	
	Sea Surface Temperature (Change in variability)	1.5	2.7	
	Air Temperature (Change in mean)	4.0	2.7	
	Air Temperature (Change in variability)	2.0	2.7	
	Precipitation (Change in mean)	2.0	2.7	
	Precipitation (Change in variability)	1.0	2.3	
	Sea Surface Salinity (Change in mean)	2.9	2.3	
	Sea Surface Salinity (Change in variability)	1.7	2.3	
	Ocean pH (Change in mean)	3.6	2.7	
	Ocean pH (Change in variability)	1.6	2.7	
	Sea ice coverage (Change in mean)	1.0	3.0	
	Sea ice coverage (Change in variability)	1.0	3.0	
	Dissolved oxygen (Change in mean)	3.6	2.7	
	Dissolved oxygen (Change in variability)	1.6	2.7	
	Circulation	2.7	2.0	
	Sea level rise	1.0	3.0	
	Exposure Score	Very High		
Overall Vulnerability Rank		Very High		

Low
Moderate
High
Very High

Cuvier's beaked whale (Gulf of Mexico Stock)

Ziphius cavirostris

CVA Results Summary

Overall Climate Vulnerability Rank: Very High (63% certainty from bootstrap analysis).

Climate Exposure: Very High. Three exposure factors scored greater than 3.5: Air Temperature (Standard anomaly) (4.00), Dissolved oxygen (Standard anomaly) (3.60), and Ocean pH (Standard anomaly) (3.60).

Biological Sensitivity: High. Two sensitivity attributes scored greater than 3.0: Lifetime Reproductive Potential (3.20) and Migration (3.13).

Distributional Response: Moderate

Abundance Response: Moderate

Phenology Response: Moderate

Data Quality: 70% of the data quality scores were 2 or greater, 27% of sensitivity attribute data quality scores were 2 or higher.

Background Information

Stock Description

The Cuvier's beaked whale Gulf of Mexico stock includes individuals that reside in or visit waters of the U.S. Exclusive Economic Zone of Gulf of Mexico and adjacent offshore and Mexican waters (Hayes et al. 2021). This stock is considered separately from the Puerto Rico/U.S. Virgin Islands and western North Atlantic stocks.

Prey/Diet Specificity

West et al. (2017) compiled Cuvier's beaked whale diet information from 18 studies, which included 45 stomach analyses. World-wide, the most important cephalopod families in the diet are Cranchiidae, Gonatidae, Histioteuthidae, Octopoteuthidae, Ommastrephidae, Onychoteuthidae, Pholidoteuthidae, and Mastigoteuthidae, all within the order Oegopsida (West et al. 2017). Cranchiidae is common prey in all locations (West et al. 2017). Histioteuthidae and Cranchiidae are the most common prey in the North Atlantic (West et al. 2017).

Stomach content data for Cuvier's beaked whales in the Gulf of Mexico is limited to a single stranding, which contained remains of the squid *Loligo peali* and unidentified cephalopods (Fertl et al. 1997).

Habitat Specificity

Generally, little is known about beaked whale habitat preferences due to their elusive nature (Barlow et al. 2006). Beaked whales are typically found in continental slope and oceanic waters deeper than 200 m

(Ritter and Brederlau 1999; Gannier 2000; Waring et al. 2001; Cañadas et al. 2002; Pitman 2002; MacLeod et al. 2004; Ferguson 2005; MacLeod and Zuur 2005; Ferguson et al. 2006; MacLeod and Mitchell 2006). Beaked whales are only occasionally reported in waters over the continental shelf (Pitman 2002). Cuvier's beaked whales have been found associated with physical features such as the continental slope, canyons, escarpments, and oceanic islands (Waring et al. 2001; Baird et al. 2004; MacLeod et al. 2004; MacLeod and D'Amico 2006).

In the Gulf of Mexico, beaked whales are found in waters with a bottom depth ranging from 420m to 3,487m (Ward et al. 2005) while few beaked whales are sighted on the continental shelf (e.g., Fritts et al. 1983; Esher et al. 1992). Habitat characterization modeling predicted areas greater than 1000m in bottom depth as potential beaked whale habitat (Ward et al. 2005). The probability of beaked whale presence is greatest along the continental slope (Ward et al. 2005). Habitat-based density models predicted concentrations of beaked whales (and sperm whales) near off-shelf submarine canyons at the mouth of the Mississippi River and the central northern Gulf, and along the continental slope (Roberts et al. 2016a). Off the U.S. East Coast, the models predicted highest densities along the continental slope, in and around submarine canyons, and near seamounts, particularly in cold, productive waters, consistent with their reported habitats (Roberts et al. 2016a).

Based on data collected during GulfCet I, Davis et al. (1998) reported that beaked whales were found in areas of steep SST gradients, and suggested that beaked whales (and other deep-diving cetaceans in the Gulf) may be foraging along thermal fronts associated with eddy systems.

Würsig (2017) [adapted from Maze-Foley and Mullin 2006] reported that Cuvier's beaked whales were encountered during on-effort surveys by NMFS in the Northern Oceanic Gulf during the period from 1991–2001 at a mean depth of 1884m (range 1179m-3221m depth) and mean water temperature of 26.01 degrees C (range 24.3-28.3 degrees C).

Martinez et al. (2010) reported sighting Cuvier's beaked whales within or near the Deepwater Horizon site between 28 April – 31 July, 2010. Martinez et al. (2010) also reported sighting beaked whales in the southeastern Gulf of Mexico near the Dry Tortugas.

Site Fidelity

Site fidelity information for the Gulf of Mexico is not available, though there are known hotspots such as Mississippi Canyon (e.g., Hildebrand et al. 2015). Site fidelity has been reported for Cuvier's beaked whales in the Ligurian Sea (Ballardini et al. 2005), Hawaii (McSweeney et al. 2007), and Canary Islands (Aguilar de Soto and Hammond 2014).

From passive acoustic monitoring data, beaked whales (Cuvier's and Gervais') showed a significant higher detection rate in the Dry Tortugas site (13.4 animals/1000km2) when compared to the Mississippi and Green canyons sites (2.6 animals/1,000 km2 and 1.8 animals/1,000 km2, respectively; Table 9 in Hildebrand et al. 2012).

Lifetime Reproductive Potential

Taylor et al. (2007) did not include interbirth intervals for Ziphiids. Interbirth intervals for other odontocetes (not including phocoenids or highly matrilineal species) range between 2-4.7 years (Taylor et al. 2007). In Hawaii, one photo-identified female had a calving interval greater than 6 years (Baird

2018). In the Canary Islands, Aguilar de Soto and Hammond (2014) estimated calving interval to be approximately 4 years using photo-ID of two females associated with calves.

Taylor et al. (2007) reported age of last reproduction for Cuvier's beaked whale as 46 and for Mesoplodont species as ranging between 21-44, with most around 40. While Ziphiid age of first reproduction is not reported, odontocetes (not including phocoenids or highly matrilineal species) range between 7-14 years (Taylor et al. 2007). Other studies suggest a maximum age of females at 30 years and males at 36 years, based on growth layer groups that may be annual layers (Mitchell 1975; Mead 1984; Houston 1990; Baird 2018).

Research on related Blainville's beaked whales in the Bahamas suggests males and females reach sexual maturity at age nine and maximum age is greater than 23 years (Claridge 2013).

Generation Length

Taylor et al. (2007) did not report Ziphiid generation length, but other odontocetes (not including phocoenids or highly matrilineal species) range between 11-27 years.

Reproductive Plasticity

Information regarding Cuvier's beaked whale reproductive seasonality, location, and habitat in the northern Gulf of Mexico was not found in the literature.

Migration

During a 3-year monitoring period following the Deepwater Horizon oil spill, Cuvier's beaked whales were present only seasonally in the western Gulf of Mexico (Green Canyon and Mississippi Canyon), with periods of low density during the summer and higher density in the winter, while a high density was maintained at an eastern Gulf of Mexico site (Dry Tortugas; Hildebrand et al. 2015).

Home Range

Information regarding Cuvier's beaked whale site fidelity in the northern Gulf of Mexico was not found in the literature.

Stock Abundance

Garrison et al. (2020) estimated Cuvier's beaked whales in the northern Gulf of Mexico at 18 (CV=0.75) and unidentified Ziiphidae in the northern Gulf of Mexico at 181 individuals (CV=0.31) based on 2018 surveys. Previously, Waring et al. (2013) estimated Cuvier's beaked whales in the northern Gulf of Mexico at 74 (CV=1.04) and unidentified Ziiphidae in the northern Gulf of Mexico at 74 (CV=1.04), based on a summer 2009 oceanic survey covering waters from the 200m isobath to the seaward extent of the U.S. EEZ, and not including the southern Gulf of Mexico.

Earlier estimates include a 1991-1994 survey, a 1996-2001 survey, and a 2003-2004 survey (Waring et al. 2009). From the 2003-2004 survey, the best estimate of Cuvier's beaked whales was 65 (CV=0.67), however Cuvier's beaked whales may also be in the estimate of unidentified Ziphiidae, which was 337 (CV=0.40; Mullin 2007). From the 1996-2001 survey 1996 to 2001, the best estimate of Cuvier's beaked

whales was 95 (CV=0.47), while the estimate for unidentified Ziphiidae was 146 (CV=0.46; Mullin and Fulling 2004).

Survey Period	Area	Cuvier's beaked whale		Unidentified Ziphiidae		Reference
		NBest	CV	NBest	CV	
1991-1994	GOM Oceanic waters	30	0.50	117	0.38	Hansen et al. 1995
1996-2001	GOM Oceanic waters	95	0.47	146	0.46	Mullin and Fulling 2004
2003-2004	GOM Oceanic waters	65	0.67	337	0.40	Mullin 2007
2009	GOM Oceanic waters	74	1.04	74	1.04	Waring et al. 2013
2018	GOM Oceanic waters	18	0.75	181	0.31	Garrison et al. 2020

Stock Abundance Trend

Despite four point estimates of Cuvier's beaked whale abundance from 1991 through 2009, temporal trends remain difficult to discern without an analysis of all the survey data to understand the potential effects of covariates on the estimates (Waring et al. 2013).

Cumulative Stressors

Cuvier's beaked whale in the northern Gulf of Mexico may interact with longline fisheries. Hayes et al. (2021) reported no fishing-related mortality of a Cuvier's beaked whale during 2014–2018 (Garrison and Stokes 2016, 2017, 2019, 2020a, 2020b). An interaction with the pelagic longline fishery resulted in 1 unidentified beaked whale released alive with no serious injury after an entanglement in 2007 (Fairfield and Garrison 2008). The stock has the potential to interact with commercial and recreational fisheries in the Gulf of Mexico through entanglement in or ingestion of fishing gear, declining prey stocks, and vessel strikes (MMC 2015).

One Cuvier's beaked whale stranded with no indication of human interaction in Texas in October 2004 and two unidentified beaked whales mass stranded in Florida in December 1999 (Waring et al. 2009).

Impacts on beaked whales associated with naval activities (e.g., sonar) include skin lesions (Jepson et al. 2003; Fernández et al. 2005; Jepson et al. 2016) and mass strandings (Simmonds and Lopez-Jurado 1991; Frantzis 1998).

The oil, dispersant and burn residue compounds from the Deepwater Horizon (DWH) incident present persistent ecological concerns along coastal areas from western Louisiana to the Florida Panhandle (NOAA 2011). Aichinger Dias et al. (2017) documented occurrences of Cuvier's beaked whales swimming in or near petroleum products, or with oil adhered to their bodies, which may result in a number of adverse effects (Geraci 1990). Seismic exploration, explosive platform removal, vessel and air traffic, and oil spills are threats to marine mammals associated with oil and gas exploration (MMC 2015). Vessel

strikes, oil spills and other hazardous material discharges, marine debris, and noise are threats to marine mammals associated with shipping (MMC 2015). The deep-diving nature of beaked whales that makes sighting them difficult during surveys may limit their exposure to vessel strikes.

Cuvier's beaked whale predators include tiger sharks, white sharks, and cookiecutter sharks (Pérez-Zayas et al. 2002; Cárdenas Hinojosa et al. 2015; Baird 2018).

Other relevant information

In his review of global climate change, range changes and potential implications for the conservation of marine cetaceans, MacLeod (2009) categorized the Cuvier's beaked whale as a warmer water-limited (WWL) species and speculated that as a WWL species, the range of Cuvier's beaked whale is likely to expand polewards in all oceans.

Learmonth et al. (2006) in their table for potential effects of climate change on species range had a question mark for Cuvier's bw and how they would be affected.

Studies of deep-water currents and their potential role in increasing deep-water productivity as well as other proximal factors need to be considered for deepwater species in the Gulf, as well as the dynamic nature of the warm- and cold-core rings.

Distribution and Sightings

Density model results for Cuvier's beaked whale in the Gulf of Mexico are presented by Roberts et al. (2016a, 2016b, 2017) and Mannocci et al. (2017).

Further Reading

Allen, B.M., Brownell, R.L. and Mead, J.G., 2012. Species review of Cuvier's beaked whale, *Ziphius cavirostris*. Reports of International Whaling Commission. https://swfsc.noaa.gov/publications/CR/2011/2011Allen2.pdf

Baird, R.W. 2018. Cuvier's beaked whale *Ziphius cavirostris*. Encyclopedia of marine mammals. Third edition. B. Würsig, J. G. M. Thewissen and K. M. Kovacs. San Diego, California, Elsevier/Academic Press: 254-257.

Taylor, B.L., Baird, R., Barlow, J., Dawson, S.M., Ford, J., Mead, J.G., Notarbartolo di Sciara, G., Wade, P. & Pitman, R.L. 2008. *Ziphius cavirostris*. The IUCN Red List of Threatened Species 2008: e.T23211A9429826. https://dx.doi.org/10.2305/IUCN.UK.2008.RLTS.T23211A9429826.en

Valdivia, A (2017) Petition to list the Gulf of Mexico Cuvier's beaked whale (*Ziphius cavirostris*) as endangered under the Endangered Species Act. Center for Biological Diversity, 57 pp. http://sero.nmfs.noaa.gov/protected_resources/listing_petitions/documents/cbd_petition_to_list_the_gulf_of_mexico_cuvier_s_beaked_whale_under_the_esa_final.pdf

References

Aguilar de Soto, N. and P. Hammond. 2014. Population parameters of Blainville´s and Cuvier´s beaked whales. Report to Office of Naval Research for Award No. N00014-12-1-0626. 8 p.

Aichinger Dias, L., J. Litz, L. Garrison, A. Martinez, K. Barry, and T. Speakman. 2017. Exposure of cetaceans to petroleum products following the Deepwater Horizon oil spill in the Gulf of Mexico. Endangered Species Research 33:119–125. doi: 10.3354/esr00770

Baird, R.W. 2018. Cuvier’s beaked whale: *Ziphius cavirostris*. In: Würsig, B., J.G.M. Thewissen, and K.M. Kovacs (Eds.), Encyclopedia of Marine Mammals (Third Edition). Academic Press, London, UK. pp. 234–237. doi: 10.1016/B978-0-12-804327-1.00100-X

Baird, R.W., D.J. McSweeney, A.D. Ligon, and D.L. Webster. 2004. Tagging feasibility and diving of Cuvier's beaked whales (*Ziphius cavirostris*) and Blainville's beaked whales (*Mesoplodon densirostris*) in Hawai'i. National Oceanic and Atmospheric Administration, National Marine Fisheries Service, Southwest Fisheries Science Center: La Jolla, CA. Order No. AB133F-03-SE-0986. 15 p.

Ballardini, M., T. Pusser, and B. Nani. 2005. Photo-identification of Cuvier’s beaked whales (*Ziphius cavirostris*) in the northern Ligurian Sea. Proceedings of the 14th annual conference of the European Cetacean Society, La Rochelle. pp. 4–7.

Barlow, J., M.C. Ferguson, W.F. Perrin, L. Ballance, T. Gerrodette, G. Joyce, C.D. MacLeod, K. Mullin, D.L. Palka, and G. Waring. 2006. Abundance and densities of beaked and bottlenose whales (family Ziphiidae). Journal of Cetacean Research and Management 7:263–270.

Cañadas, A., R. Sagarminaga, and S. García-Tiscar. 2002. Cetacean distribution related with depth and slope in the Mediterranean waters off southern Spain. Deep Sea Research Part I: Oceanographic Research Papers 49(11):2053–2073. doi: 10.1016/s0967-0637(02)00123-1

Cárdenas-Hinojosa, G., M. Hoyos-Padilla, and L. Rojas-Bracho. 2015. Occurrence of Cuvier’s beaked whales (*Ziphius cavirostris*) at Guadalupe Island, Mexico, from 2006 to 2009. Latin American Journal of Aquatic Mammals 10(1):38–47. doi: 10.5597/lajam00192

Claridge, D.E. 2013. Population ecology of Blainville's beaked whales (*Mesoplodon densirostris*). University of St Andrews. 296 p.

Davis, R.W., G.S. Fargion, N. May, T.D. Leming, M. Baumgartner, W.E. Evans, L.J. Hansen, and K. Mullin. 1998. Physical habitat of cetaceans along the continental slope in the northcentral and western Gulf of Mexico. Marine Mammal Science 14(3):490–507. doi: 10.1111/j.1748-7692.1998.tb00738.x

Esher, R.J., C. Levenson, and T.D. Drummer. 1992. Aerial surveys of endangered and protected species in the Empress II ship trial operating area in the Gulf of Mexico. Final report. Naval Research Laboratory: Stennis Space Center, MS. NRL/MR/7174-92-7002. 55 p.

Fairfield, C.P. and L.P. Garrison. 2008. Estimated bycatch of marine mammals and turtles in the U.S. Atlantic pelagic longline fleet during 2007. National Oceanic and Atmospheric Administration, National Marine Fisheries Service, Southeast Fisheries Science Center: Miami, FL. NOAA Technical Memorandum NMFS-SEFSC-572. 62 p.

Ferguson, M.C. 2005. Cetacean population density in the eastern Pacific Ocean: Analyzing patterns with predictive spatial models. University of California, San Diego. 203 p.

Ferguson, M.C., J. Barlow, S.B. Reilly, and T. Gerrodette. 2006. Predicting Cuvier's (*Ziphius cavirostris*) and *Mesoplodon* beaked whale population density from habitat characteristics in the eastern tropical Pacific Ocean. Journal of Cetacean Research and Management 7(3):287–299.

Fernandez, A., J.F. Edwards, F. Rodriguez, A. Espinosa de los Monteros, P. Herraez, P. Castro, J.R. Jaber, V. Martin, and M. Arbelo. 2005. "Gas and fat embolic syndrome" involving a mass stranding of beaked whales (Family Ziphiidae) exposed to anthropogenic sonar signals. Veterinary Pathology 42(4):446–457. doi: 10.1354/vp.42-4-446

Fertl, D., A.J. Schiro, S. Collier, and G.A.J. Worthy. 1997. Stranding of a Cuvier's beaked whale (*Ziphius cavirostris*) in southern Texas, with comments on stomach contents. Gulf of Mexico Science 15(2):92–93. doi: 10.18785/goms.1502.05

Frantzis, A. 1998. Does acoustic testing strand whales? Nature 392(6671):29. doi: 10.1038/32068
Fritts, T.H., U.S. Fish and Wildlife Service. Division of Biological Services., United States. Minerals Management Service., and Denver Wildlife Research Center. Museum Section. 1983. Turtles, birds, and mammals in the northern Gulf of Mexico and nearby Atlantic waters : an overview based on aerial surveys of OCS areas, with emphasis on oil and gas effects. The Division : Minerals Management Service, Washington, DC. p.
Gannier, A. 2000. Distribution of cetaceans off the Society Islands (French Polynesia) as obtained from dedicated surveys. Aquatic Mammals 26(2):111–126.
Garrison, L.P., J. Ortega-Ortiz, and G. Rappucci. 2020. Abundance of marine mammals in waters of the U.S. Gulf of Mexico during the summers of 2017 and 2018. National Oceanic and Atmospheric Administration, National Marine Fisheries Service, Southeast Fisheries Science Center: Miami, FL. Southeast Fisheries Science Center Reference Document PRBD-2020-07. 56 p. doi: 10.25923/3px6-9v48
Garrison, L.P. and L. Stokes. 2016. Estimated bycatch of marine mammals and sea turtles in the U.S. Atlantic pelagic longline fleet during 2014. National Oceanic and Atmospheric Administration, National Marine Fisheries Service, Southeast Fisheries Science Center: Miami, FL. NOAA Technical Memorandum NMFS-SEFSC-696. 62 p. doi: 10.7289/V5/TM-SEFSC-696
Garrison, L.P. and L. Stokes. 2017. Estimated bycatch of marine mammals and sea turtles in the U.S. Atlantic pelagic longline fleet during 2015. National Oceanic and Atmospheric Administration, National Marine Fisheries Service, Southeast Fisheries Science Center: Miami, FL. NOAA Technical Memorandum NMFS-SEFSC-709. 61 p. doi: 10.25923/kaf6-5c33
Garrison, L.P. and L. Stokes. 2019. Estimated bycatch of marine mammals and sea turtles in the U.S. Atlantic pelagic longline fleet during 2016. National Oceanic and Atmospheric Administration, National Marine Fisheries Service, Southeast Fisheries Science Center: Miami, FL. Southeast Fisheries Science Center Reference Document PRBD-2019-01. 43 p. doi: 10.25923/d893-d142
Garrison, L.P. and L. Stokes. 2020a. Estimated bycatch of marine mammals and sea turtles in the U.S. Atlantic pelagic longline fleet during 2017. National Oceanic and Atmospheric Administration, National Marine Fisheries Service, Southeast Fisheries Science Center: Miami, FL. Southeast Fisheries Science Center Reference Document PRBD 2020-05. 58 p. doi: 10.25923/a3hs-ea60
[truncated: 399,382 more chars]
